# A genome scan for milk production traits in dairy goats reveals two new mutations in *Dgat1* reducing milk fat content

**DOI:** 10.1038/s41598-017-02052-0

**Published:** 2017-05-12

**Authors:** Pauline Martin, Isabelle Palhière, Cyrielle Maroteau, Philippe Bardou, Kamila Canale-Tabet, Julien Sarry, Florent Woloszyn, Justine Bertrand-Michel, Ines Racke, Hüseyin Besir, Rachel Rupp, Gwenola Tosser-Klopp

**Affiliations:** 1 GenPhySE, Université de Toulouse, INRA, INPT, ENVT, Castanet Tolosan France; 2 Division of Molecular and Clinical Medecine, School of Medecine, University of Dundee, Ninewells Hospital and Medical School, Dundee, UK; 3 INRA, Sigenae, Castanet-Tolosan France; 4 MetaToul-Lipidomic Core Facility, MetaboHUB, INSERM U 1048, Toulouse, France; 5 Protein Expression and Purification Core Facility, EMBL Heidelberg, Heidelberg, Germany

## Abstract

The quantity of milk and milk fat and proteins are particularly important traits in dairy livestock. However, little is known about the regions of the genome that influence these traits in goats. We conducted a genome wide association study in French goats and identified 109 regions associated with dairy traits. For a major region on chromosome 14 closely associated with fat content, the Diacylglycerol O-Acyltransferase 1 (*DGAT1*) gene turned out to be a functional and positional candidate gene. The caprine reference sequence of this gene was completed and 29 polymorphisms were found in the gene sequence, including two novel exonic mutations: R251L and R396W, leading to substitutions in the protein sequence. The R251L mutation was found in the Saanen breed at a frequency of 3.5% and the R396W mutation both in the Saanen and Alpine breeds at a frequencies of 13% and 7% respectively. The R396W mutation explained 46% of the genetic variance of the trait, and the R251L mutation 6%. Both mutations were associated with a notable decrease in milk fat content. Their causality was then demonstrated by a functional test. These results provide new knowledge on the genetic basis of milk synthesis and will help improve the management of the French dairy goat breeding program.

## Introduction

In Europe, goat farming mainly targets cheese production. The average milk production level of the animals differs between regions of the world, partly due to different farm management systems but also to different genetics (breeds, selection). Goat breeding programs are still rare. Some countries have created collective structures to control performance and to estimate breeding values. The French breeding scheme is unique in the number of animals it includes and the high AI rate (40%). Like in the case of dairy sheep and dairy cows, the objectives of selection are generally the quantity and composition of the milk. Dairy traits are fundamental in livestock production. The efficiency of the French breeding program has been responsible for an annual genetic gain of +13 kg per year for milk yield and of +0.1 g/kg per year for fat and protein contents for the past ten years. Today France is the first producer of goat milk in the European Union, it produces 27% of the total volume of milk from only 10% of the animals, and is the fifth largest producer in the world^1^.

The composition of goat milk differs from that of cattle. It contains more minerals and more calcium, particularly due to its specific casein composition, which results in larger micelles^2,3^. The fatty acid composition of goat milk also differs, with a higher proportion of short and medium fatty acid chains which are also grouped in smaller fat globules^3,4^. The proportion of caproic (C6 :0), caprylic (C8 :0), capric (C10 :0) and lauric acids (C12 :0) is considerably higher in goat milk^3,5^, and these are the fatty acids that give a typical flavor to goat milk and cheese^6^. The composition of goat milk makes it easier to digest than that of cows, it has a higher nutritional value and is healthier and less allergenic^3,7,8^.

The availability of genome sequencing data has opened up new fields of investigation in domestic ruminant species with sequenced genomes^9–11^. The development of high-density single nucleotide polymorphism (SNP) arrays and their application in genome-wide association studies has facilitated the identification of regions that control quantitative traits in dairy cattle (for example^12–20^). However, very little is known about the loci controlling milk traits in goats. Using a candidate gene approach, the effect of caseins, especially that of the α_s1_ casein (*CSN1S1*), on the composition of milk (mainly protein content) is well documented in goats^21–23^. Many variants (>15) have been found for the different caseins^24–28^. Among the *CSN1S1* variants, the effect varies from +3.6 g/L to almost no casein synthesized by homozygous animals carrying the null allele.

However, no genome wide association study of milk production has been conducted in goats. The GoatSNP50 BeadChip was released in 2011^29^ and made this type of analysis possible. A large family design was therefore implemented in France to provide more information about the genetic control of milk traits.

The aim of the present work was to perform linkage analyses (LA) and linkage disequilibrium (LD) analyses, based on GoatSNP50 BeadChip data, to identify the genomic regions responsible for the quantity and composition of goat milk.

## Results

### Discovery of QTLs for milk production traits

QTLs associated with milk yield (MY), protein and fat yield (PY and FY, respectively) as well as protein content (PC) and fat content (FC), were mapped by means of a genome scan (29 autosomes) using both haplotype-based linkage and association analyses in 1,941 dairy goats distributed in 20 half-sib families. All the goats and their 20 sires were genotyped with the 50 K GoatSNP50 Beadchip (Illumina, San Diego, CA). Analyses were first conducted independently in each breed and then combined in a joint analysis.

**Figure 1:**
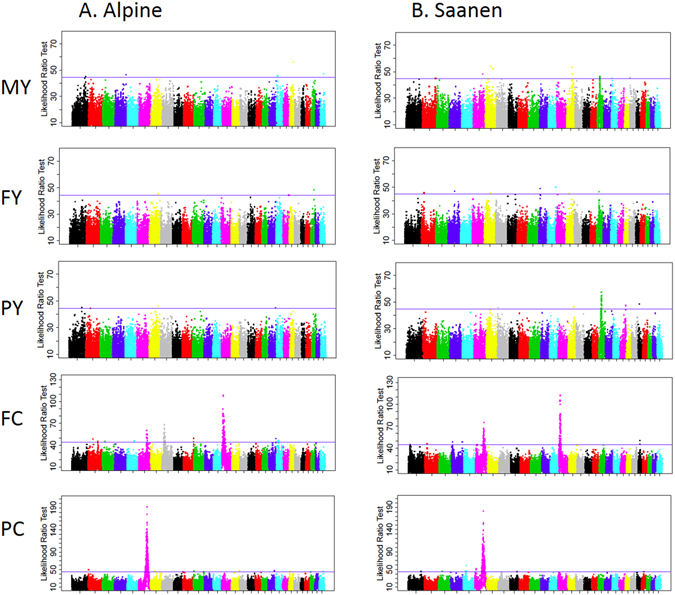
Manhattan plot of likelihood ratio test profiles for five milk production traits: milk yield (MY), fat yield (FY), protein yield (PY), fat content (FC) and protein content (PC) in Alpine (**A**) and Saanen goat breeds (**B**). The solid horizontal lines represent the 5% genome-wide thresholds (averaged over the 29 autosomes).

Haplotype-based linkage was used to detect 24 QTLs at a 1% chromosome-wide threshold of significance (Table 1). Among them, 11 hits in four regions of chromosomes CHI 1, 6, 14, and 21 exceeded the 5% genome-wide threshold of significance. Many more QTLs were detected using association mapping, giving a total of 85 hits, which exceeded the 5% genome-wide threshold of significance (Fig. 1A,B, Table S1, Fig. S1). Genome scans are shown in Table S2.

**Table 1:** Genome scan for milk production traits in a daughter design of 1,941 dairy goats, based on haplotype-based linkage analyses.

CH I	Trait	Breed	Significance level	LRT	Position (Mb)	95% –CI (min max)	Substitution effect	Candidate genes
1	PC	Alpine	***	41.6	1.382	1.362–1.449	0.36	PDE9A
1	PC	All	**	51.8	1.418	1.404–1.434	0.30	
2	FY	All	**	51.8	0.239	0.237–0.241	0.33	
5	PC	Saanen	**	31.5	0.205	0.196–0.224	0.32	
6	FC	All	***	66.2	0.764	0.743–0.812	0.32	
6	FC	Saanen	***	44.3	0.805	0.746–0.872	0.44	Caseins cluster
6	PC	Alpine	***	62.5	0.814	0.786–0.848	0.37	Caseins cluster
6	PC	All	***	161.3	0.824	0.792–0.842	0.50	Caseins cluster
6	PC	Saanen	***	99.6	0.824	0.791–0.845	0.66	Caseins cluster
7	FC	Alpine	**	36.8	0.037	0.013–0.042	0.30	SLC27A1
11	PC	Alpine	**	39.9	0.900	0.887–0.906	0.35	PAEP
14	FY	Alpine	***	39.9	0.034	0.025–0.046	0.30	
14	FY	All	**	53.8	0.037	0.025–0.048	0.28	
14	FC	All	***	126.2	0.111	0.092–0.124	0.48	DGAT1
14	FC	Saanen	***	60.8	0.123	0.092–0.143	0.50	
14	FC	Alpine	***	70.8	0.155	0.146–0.160	0.42	
19	PY	All	**	52.3	0.285	0.248–0.290	0.27	PLD2 GGT6 ALOX12, ALOX 12B, ALOX 15
21	FC	All	**	52.0	0.554	0.526–0.576	0.27	
21	FC	Alpine	**	37.2	0.571	0.538–0.588	0.27	
21	PC	All	**	49.9	0.578	0.570–0.585	0.27	
21	PY	All	***	58.0	0.634	0.627–0.640	0.31	
21	MY	All	**	53.2	0.635	0.627–0.642	0.29	
25	FC	Saanen	**	32.4	0.101	0.099–0.103	0.35	
28	PC	Alpine	**	33.7	0.322	0.295–0.361	0.31	

Significance level: ***: 5% genome-wide; ** 1% chromosome-wide. The 95% confidence intervals of the QTL locations were estimated by logarithm of odds drop-off.

**Figure 2:**
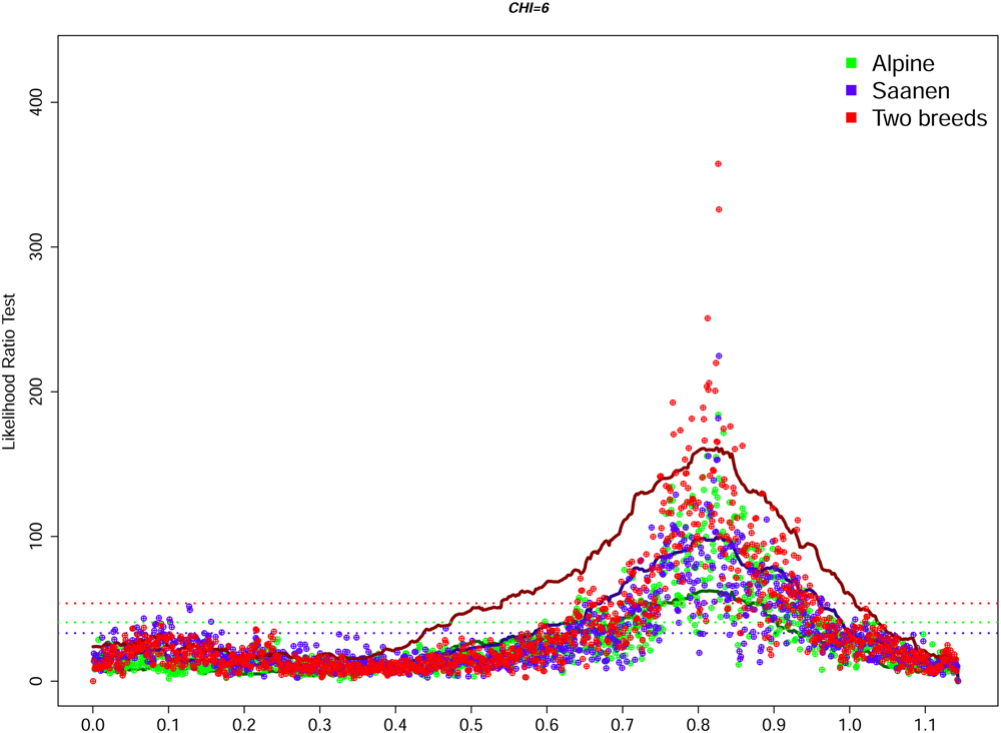
Global likelihood ratio test profiles for protein content on CHI 6 based on both linkage and haplotype-based linkage (solid lines) and association (dotted line) analyses. The dotted horizontal lines represent 5% genome-wide thresholds.

**Figure 3:**
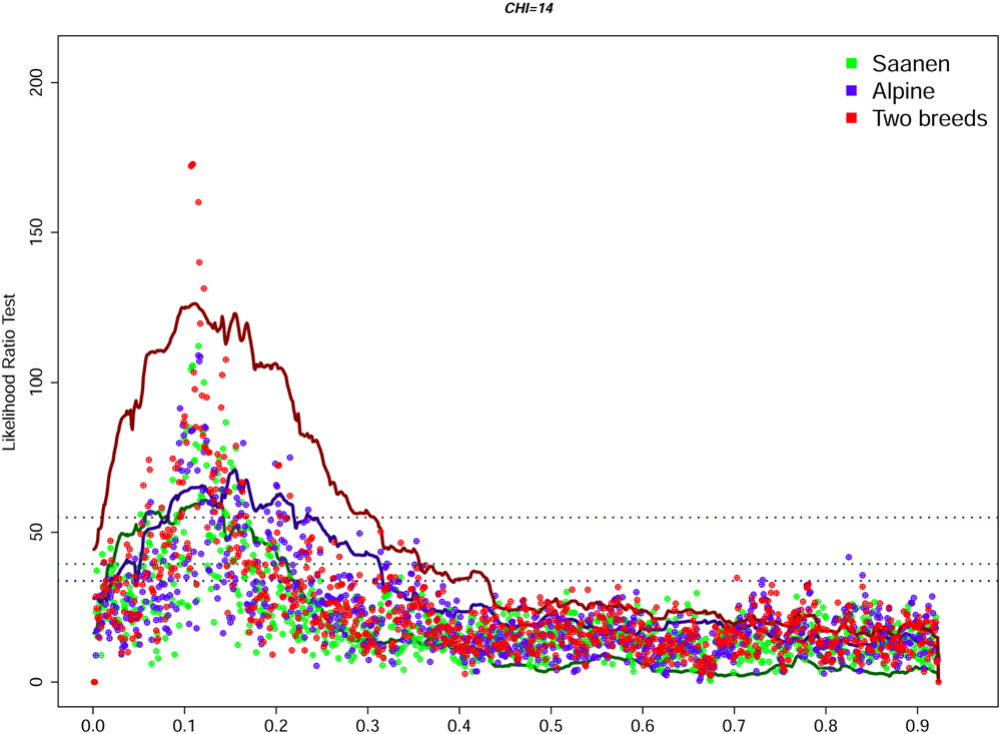
Global likelihood ratio test profiles for fat content on CHI 14 based on both linkage and haplotype-based linkage (solid lines) and association (dotted line) analyses. The dotted horizontal lines represent 5% genome-wide thresholds.

Among the many QTLs detected, two highly significant (5% genome-wide threshold) regions were mapped to similar locations in the Saanen and Alpine breeds by both the association and linkage analyses (Table 1, Fig. 1A,B, Table S1, Fig. S1). The first QTL was on CHI 6, and was associated with PC in all analyses in the region of the casein genes, i.e. 82.5–82.8 Mb (Table S1, Fig. 2). This region was also associated with FC in all association analyses and in two of the three linkage analyses (in the Saanen breed and in the two breeds combined analyses). The second QTL was on CHI 14 associated with FC (Table 1, Fig. 3, Table S1) in the region of the *DGAT1* gene, which codes for a key enzyme involved in the synthesis of milk triglycerides. Both regions exhibited the highest average substitution effects, from 0.37 to 0.66 standard deviation (Table 1). In addition, CHI 6 and CHI 14 haplotypes (association analysis) explained 39.1% and 6.8% of variance of the PC and FC traits in the analyses of the two breeds, respectively. A region of CHI 21, spanning 11.6 Mb (52.6–64.2), was associated with PC (Table 1, Table S1), as well as with FC, PY and MY (Table 1). The most significant breed specific QTLs were found for PC on CHI 1, 136.2–14.9 Mb (linkage analysis, Table 1) and for FC on CHI 8, 22.8–23.1 Mb (association analysis, Fig. 1A, Table S1) in Alpine goats. In the Saanen breed, a region of chromosome 19 was highly significantly associated with yield, including PY, FY, and MY (Fig. 1B, Table S1). In this breed, this region spanned a confidence interval of 4 Mb, from 22.0 to 26.0 Mb (Table S1).

### Fine-mapping identification of non-synonymous mutations in the *DGAT1* gene

Discovery of candidate non-synonymous mutations in the *DGAT1* gene for the CHI14 QTL.

Using the cattle *DGAT1* mRNA sequence, we identified part of exon 1, exons 3–5 and 13–17. The combination of classic and long range PCR amplification followed by Sanger sequencing enabled us to construct an improved sequence by filling 7,725 out of the 8,728 undetermined nucleotides belonging to N blocks in the reference genome. This 37,251 bp sequence is available under accession number LT221856.

**Figure 4:**
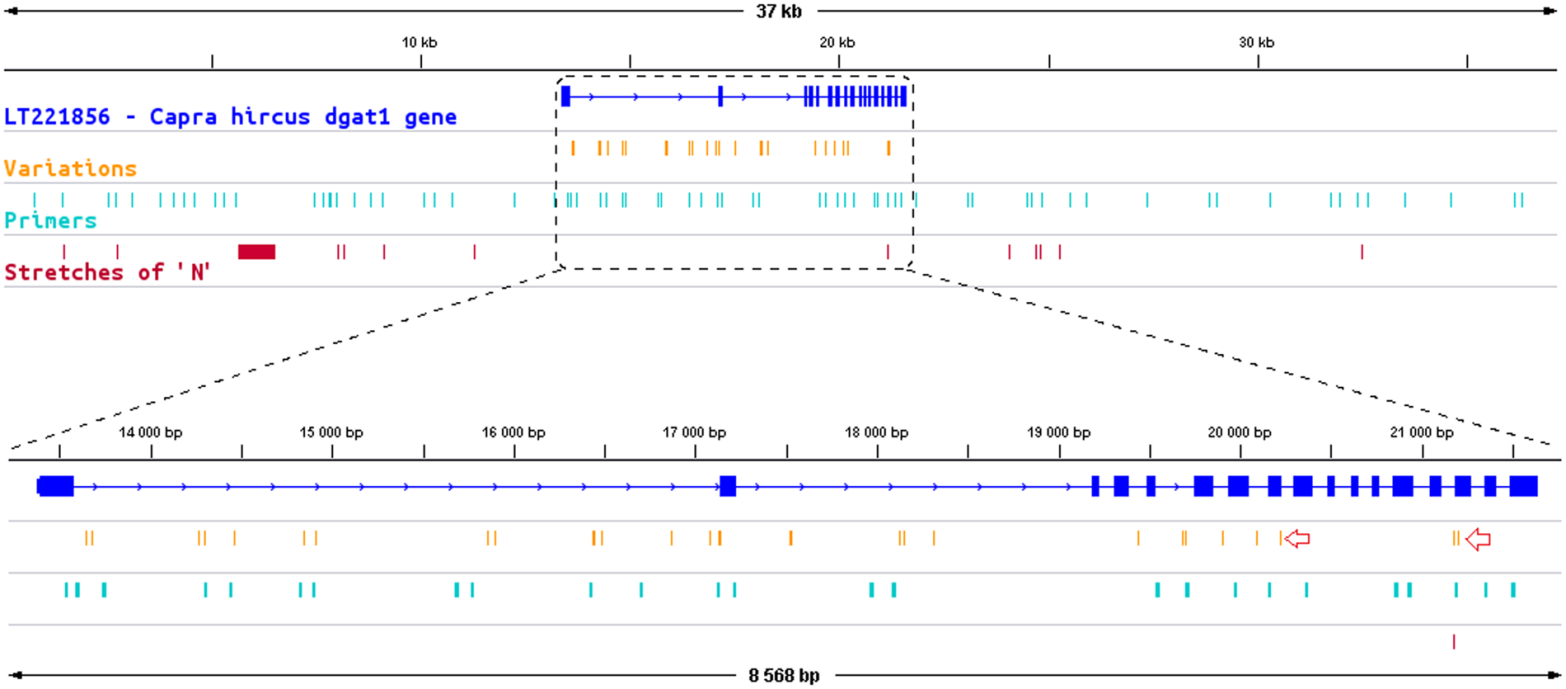
Determination of DGAT1 gene structure and polymorphism. The intron/exon structure of the LT221856 sequence is shown together with the SNPs detected and the primers used for sequencing. The location of the remaining N stretches is shown, together with their length (bp). A zoom on the coding region is also shown. The red arrows indicate the position of the R251L and R396W mutations.

The SNP discovery in 2 Alpine and 2 Saanen animals with extreme phenotypes led to the identification of 29 polymorphisms (27 SNPs, a 1 bp and a 8-bp insertion) on the *DGAT1* gene. The genotypes of the 20 bucks of the QTL design were determined for those 29 SNPs. SNPs were submitted to NCBI under ss numbers 1971466334–1971466359 and 1971466361–1971466363. Among these polymorphisms, 27 were intronic and two were located in exons 8 (NCBI_ss# 1971466363) and 15 (NCBI_ss# 1971466359), being responsible for the R251L and R396W substitutions in the DGAT1 protein sequence, respectively (Fig. 4).

### Conservation of the DGAT1 protein sequence between ruminants

The goat DGAT1 protein sequence encompassed 489 amino acids, like in sheep and cattle. This sequence is extremely well conserved between these three species, as can be seen in Fig. S2. There was no difference between the goat and the sheep protein sequence, whereas four divergent amino acid sites were found in the cattle sequence.

The DGAT1 protein is still highly conserved at the scale of all ruminant species, with at least 88% of homology, as shown in Table S3. No variation across species was found at positions 251 and 396, where goat mutations were detected in the present study.

### *DGAT1* mutation frequency

The frequency of the genomic T mutation responsible for the R396W polymorphism was 12.0% in Saanen females and 8.1% in Alpine females,. The frequencies observed in AI males differed: 14.4% in the Saanen breed and 5.6% in the Alpine breed. No change in T frequency was observed in AI males between the year of birth 1998 and 2011 in either breed (Fig. S3, annual regression coefficient = 0.1).

At the R251L locus, the frequency of the T-allele was 4.4% in females and 2.6% in AI males of the Saanen breed. In the Alpine breed, no AI males were carriers of the T mutation and the frequency in females was 0.7%.

### Association of the *DGAT1* genotype with milk production traits and fatty acid composition of milk

**Figure 5:**
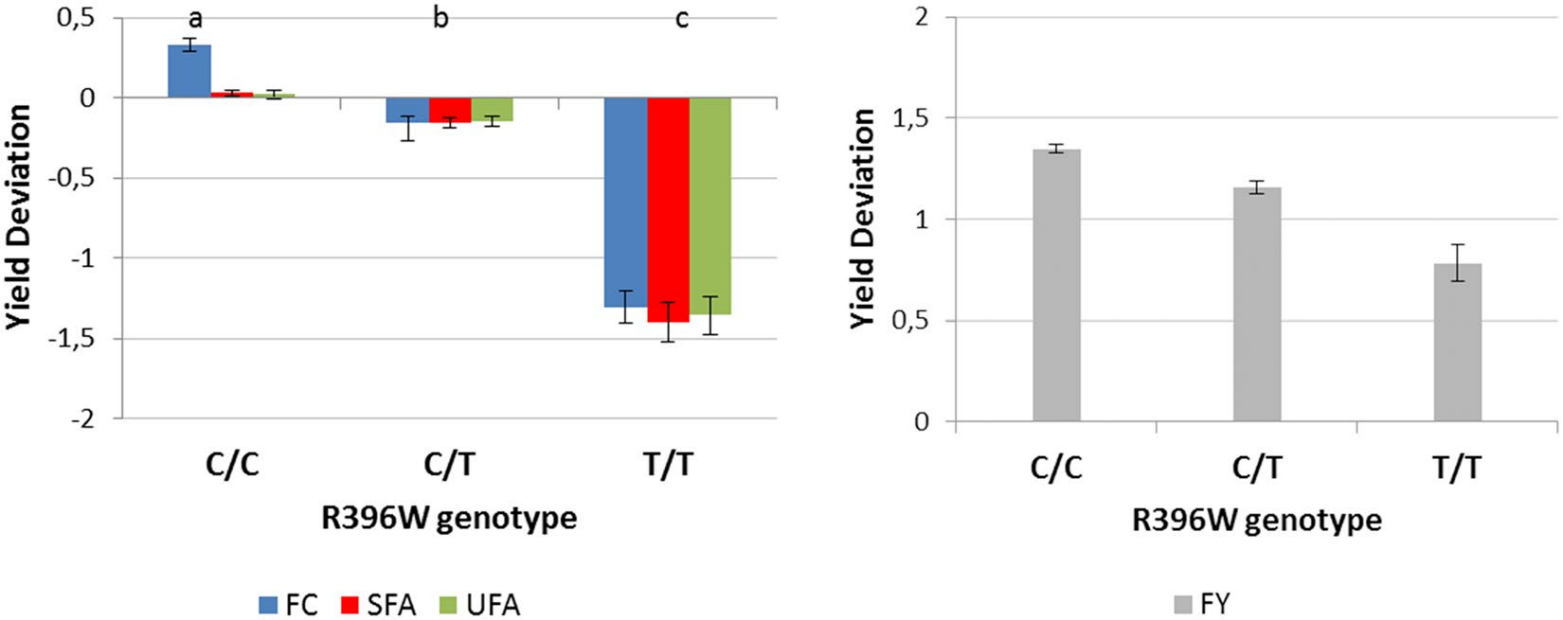
Effect of R396W genotype of DGAT1 gene on fat content (FC), quantity of saturated fatty acids (SFA) in milk, quantity of unsaturated fatty acids (UFA) in milk and fat yield (FY). The LS means have been estimated by using a mixed model including the genotype and the sire effect. Error bars indicate standard errors. Traits are expressed as the standard deviation of yield deviations. Lower case letters show significant differences in the trait between genotypes, as determined by a t-test at p < 0.05 for FY, and p < 0.005 for FC, SFA and UFA.

Analysis of variance confirmed that the T-allele at codons 251 and 396 led to a dramatic decrease in milk fat content. The average FC (LS means of yield deviations) of females of each genotype at the R396W locus is shown in Fig. 5A. Concerning the effect of the R251L and R396W, significant differences between genotypes were also observed in FY with a difference of 3.05 kg (*i.e* 0.57 SD) between the two homozygous types (Fig. 5B). No significant effect of the R396W mutation was found on MY, PY or PC.

Concerning fatty acids, the R396W mutation consistently caused a significant decrease in the quantity of saturated and unsaturated fatty acids in milk (Fig. 5A). But the respective proportions of saturated and unsaturated fatty acids in the fat were not significantly affected. The R251L locus only affected fat content. Heterozygous carriers (G/T) had a significantly lower average fat content (−1.11 g/kg, *i.e*. −0.36 SD) than the homozygous wild type (G/G). The LS means of homozygous individuals (T/T)) were not estimated because too few females carried this genotype. The variance in fat content explained by the *DGAT1* mutation differed significantly depending on the mutation: 46% for the R396W mutation and 6% for the R251L mutation, respectively.

### Effect of the R251L and R396W mutations on the enzymatic activity of recombinant DGAT1

These two exonic mutations led to modifications in the amino acid sequence of the DGAT1 protein and were associated with changes in milk composition, e.g. a decrease in fat content. But even though these were the most likely causal mutations, an indirect effect due to linkage with the true genetic variant could not be excluded.

**Figure 6:**
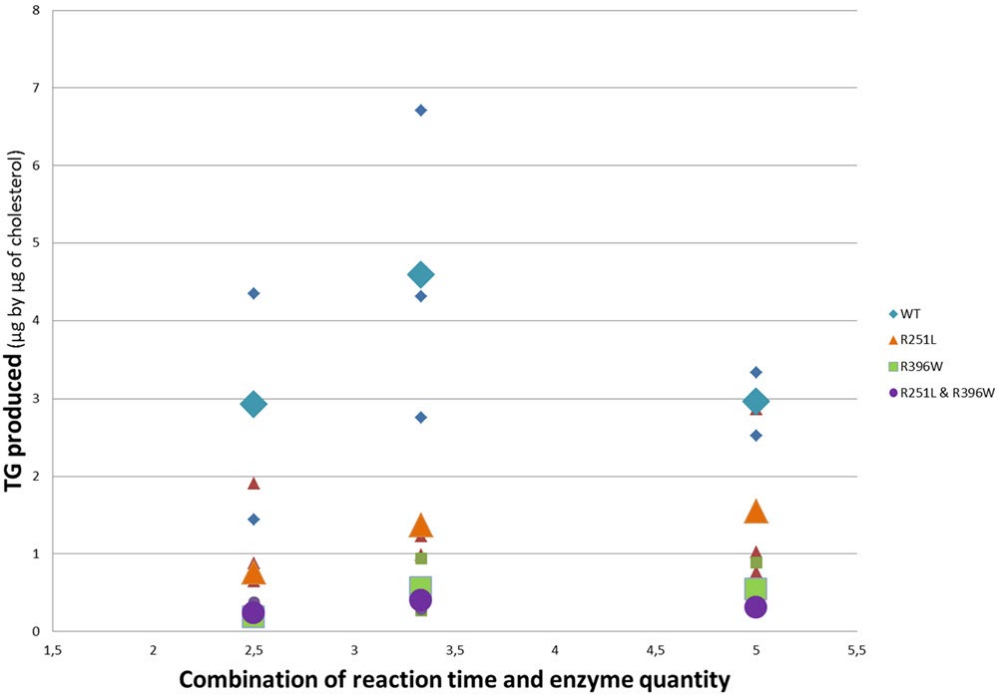
Quantity of triglyceride produced in each combination of reaction time and enzyme quantity for the four DGAT1 constructs: wild type, with the R251L mutation, with the R396W mutation, and with both mutations. This quantity of triglyceride has been corrected by an internal standard (TG19) and by the amount of measured cholesterol. The small symbols correspond to individual measurements and the large symbols are the mean of the corresponding group.

A functional test was consequently performed to prove their causality. First, using a baculovirus expression system in Sf21 insect cells (details in the Materials and Method section), one wild type and three different types of recombinant DGAT1 protein were produced: one with the R251L mutation only, one with the R396W mutation only and one carrying both mutations. Membrane fractions containing the proteins were prepared and the amount of neo-synthesized triglyceride from diglyceride produced by each membrane preparation under three different conditions was measured by gas chromatography – flame ionization detector (GCFID). The three conditions consisted in different amounts of incubated microsomes (10; 7.5 and 5 µL of protein solution) associated with an inversely proportional incubation time (2.5; 3.3 and 5 min), expected to yield identical amounts of product for a given protein, assuming enzymatic stability and lack of inhibition. The results are presented in Fig. 6. No activity was observed for the negative control membrane fraction. Analysis of these results with a generalized linear model showed that the condition effect was not significant (P = 0.10) thereby confirming that we were working in steady-state conditions, as required. In these conditions, the amount of synthesized triglycerides is indeed directly correlated with protein activity. In contrast, the construct effect was highly significant (P < 0.0001), indicating that the amount of triglyceride produced differed between the type of DGAT1 proteins included in the membrane fraction. DGAT1 constructions harboring the R251L and R396W mutation synthesized only 35% and 12% of the triglycerides, respectively, compared to the wild-type DGAT1 construction. The DGAT1 construction harboring both mutations showed a relative triglyceride synthesis of 10%.

## Discussion

In genome-wide association studies, we identified a large number of QTLs in 13 chromosomal regions, supporting the polygenic nature of dairy traits. Interestingly, a number of QTL regions included strong functional candidate genes or corresponded to orthologous regions associated with milk traits in cattle, as reported in the QTL database QTLdb (http://www.animalgenome.org/QTLdb).

### A large number of QTLs with significant overlap across the cattle, sheep, and goat genome

Only some of the loci associated with production traits were common to the two breeds. Indeed, 13% to 19% of the QTLs for LA were detected in one breed only and 27% to 30% of the QTLs for LD were breed specific. These results suggest that the main genes responsible for milk composition - or allele frequencies - differ between the two goat breeds and that there is some level of genetic heterogeneity in the determinism of dairy traits in the Saanen and Alpine goat breeds.

The different QTLs found on CHI 2^20,30^, CHI 5^31–33^, CHI 18^19,34,35^, CHI 19^32,34,36–38^, CHI 21^39,40^, CHI 24^41^, CHI 25^42^ and even on CHI 8 detected only in LD (Table S1)^43^, all point to orthologous bovine QTL regions. However, the number of QTLs detected for milk traits in cattle is very high: QTLdb indeed accounts for more than 6,000 cattle data points, most of which concern milk traits^44^. Moreover, some of these bovine QTLs detected using low density marker panels (microsatellites), span large confidence intervals, together covering almost the whole genome, so it is not surprising that our QTLs overlap some of them. The QTL on CHI 28 was the only one of our significant signals with no corresponding bovine QTL. Under the conservative assumption that underlying causal genetic variants are the same (or are at least located on the same genes), the present study could help refine the confidence intervals of these bovine QTLs. We also found an orthologous ovine QTL for CHI 5^45^, for CHI 19 but with a large interval (up to 30 Mb)^46,47^ and for CHI 21^48^.

### Four strong candidate genes for protein and fat QTLs

A QTL for percentage protein was found at the orthologous bovine position of our QTL on the CHI 1 in Chinese Holstein cows^49^. Later, 11 more SNPs were identified on the phosphodiesterase 9A (*PDE9A*) gene, located in the QTL confidence interval, associated with the different milk production traits^50^. This gene is involved in the activation of the cGMP dependent pathway and is therefore a strong candidate for our QTL for PC on CHI1. In return, our data could help find the causal SNP - or exclude false positive SNPs - for the cattle QTL.

The QTL found for PC on the CHI 5 in the association analyses is located at a distance of one Mb from the lactalbumin alpha (*LALBA*) gene, which is a strong functional candidate gene. This gene codes for a major whey protein of the milk that is involved in the lactose synthase binary complex^51^. Polymorphisms in this gene influence milk traits in mice^52^, cattle^42,53^ and sheep^51^.

Concerning the QTL for fat content on CHI 7, the long chain fatty acid transport protein 1 gene (*SLC27A1*) has been proposed as a possible candidate gene for a QTL in the orthologous cattle genomic region^54^. Indeed, in an association study of 48 Chinese Holstein, Lv *et al*.^54^ identified a synonymous SNP in exon 3 of the gene associated with milk yield. This gene, which is involved in lipid metabolism, is a strong functional candidate gene. However, its localization on the goat chromosome 7 at 5.2 Mb is outside the confidence interval of our QTL on CHI 7 [1.3–4.2 Mb].

Finally, for the QTL for PC on CHI 11, the confidence interval includes the progestagen-associated endometrial protein gene (*PAEP*) which codes for the β lactoglobulin precursor and is known for its effect on milk protein content in cattle^55^. The β-lactoglobulin, which is absent from human milk, is one of the major whey proteins in ruminants and, moreover is considered to be a dominant milk allergen^56^. Implementing selection on this locus could be one way to reduce β lactoglobulin, thereby reducing the allergenic properties of goat milk.

In the Alpine breed, the QTL for FC on CHI 8 [22.8–23.1] spans the region of a single gene, the myeloid/lymphoid or mixed-lineage leukemia; translocated to, 3 gene (*MLLT3*) with no straightforward functional role in lipid metabolism. In contrast, the confidence interval for the QTL on CHI 19 (22–27.6 Mb), included 192 annotated genes. Among them, a few are related to fatty acid and lipid metabolism pathways: phospholipase D2 (*PLD2*), gamma-glutamyltransferase 6 (*GGT6*) and arachidonate lipoxygenase (*ALOX*) 12, −12B, −15.

### The major region detected on CHI 6 corresponds to the casein cluster

The effect of caseins, especially *CSN1S1*, on the PC of milk and on other milk traits is well known in goats. A large number of variants has been found for the different caseins^21–28^. Nine of the existing variants (A, B1, B2, B3, B4, C, H, L and M are described as having a marked effect on the quantity of casein produced, two (E and I) have intermediate effects, three (D, F and G) have a weak effect and three others (O1, O2, N) are depicted as null alleles^57,58^. Alleles A, B, C, E and F were found in the French dairy goat population, with a preponderance of alleles E and F in the Saanen and Alpine breeds before 2000^59^. Some alleles, including the E allele, contain insertions that cannot be typed with the 50 K chip^60^.

The frequencies of the *CSN1S1* genotype in French goats were recently estimated^28^. The most frequent *CSN1S1* genotypes in the French dairy goat population are genotypes AA and AE carried by more than 50% of all goats. More than 25% of the progeny-tested males carried the AA genotype. After estimating genetic parameters, Carillier-Jacquin *et al*.^28^ found that the polymorphism of *CSN1S1* explained 24.4% of PC in the Saanen breed and 38.2% of PC in the Alpine breed.

QTL mapping to the goat casein cluster in the present study explained 39.1% of the variance in PC, suggesting that the GoatSNP50 BeadChip-based haplotypes mainly capture genetic variability due to the *CSN1S1*gene. Other polymorphisms probably play a role within the casein cluster (*CSN1S2*, *CSN2*, *CSN3*genes) and will be investigated in further comprehensive analyses of this region. QTLs for traits related to milk composition have been found in orthologous regions in other ruminant species^14,19,20,61–64^. Only a few polymorphisms in casein genes have been reported in cattle^65–68^. The large number of polymorphisms in *CSN1S1* gene and their marked effect on milk composition is specific to the goat.

### The DGAT1 protein is conserved between species but with a large number of existing variants

The *DGAT1* gene is known to influence milk composition. This gene codes for a microsomal enzyme that catalyzes the last and limiting step of triglyceride synthesis, i.e. the transformation from a diacylglycerol to a triacylglycerol^69,70^. This enzyme, which was first known for its action in the formation of adipose tissue, has been shown to play a key role in lactation i.e DGAT1 knock-out mice were indeed unable to synthesize milk^71^.

Ours is the first study to report on the effect of the *DGAT1* gene polymorphisms in the goat species and its influence on milk fat content. However, a bovine QTL for milk composition has been already associated with the *DGAT1* gene in a genome scan^15,72–75^. The authors identified a non-synonymous mutation, K232A, which had an effect on milk fat content^76–78^ The results obtained by these authors strongly suggested that DGAT1 alone accounts for the QTL effect in the genomic region they had identified. and they showed that the enzymatic activity of the K232A recombinant DGAT1 protein is characterized by a lower Vmax. In sheep, a corresponding QTL has also been detected for dairy traits at the proximal end of ovine chromosome 9 (OAR9), the sheep homolog of BTA14^61^. Scata *et al*.^79^ completed the sheep DGAT1 sequence and found five polymorphisms, including two associated with an effect on milk composition.

Further, DGAT1 catalyzes the last step of triglyceride synthesis and its high protein sequence conservation between ruminant species supports the hypothesis that this protein plays a major role in biological functions. However, some variations have been reported within species. As DGAT1 was suspected to act in cattle and sheep, several authors already searched for polymorphisms in the goat *DGAT1* gene by partially sequencing it, but most of the polymorphisms mapped to intronic and promoter regions^80–84^. The bovine mutation K232A was also the subject of unsuccessful searches^85^.

The DGAT1 protein is located on the membrane of the endoplasmic reticulum. Little is known about its three-dimensional structure, as no crystal structure of it or any closely homologous protein has yet been determined, but different hypotheses or predictions of the 2D or 3D structure have been proposed. Topology models predict eight^86^ or six^87^ transmembrane domains, whereas *in vitro* constructs predict only three and a N terminus oriented toward the cytosol^86^. If the active site was originally assumed to be on the cytosolic side of the membrane^88,89^, a more recent study suggests that a putative catalytic histidine is involved in the luminal side of the membrane. The histidine located at position 416, is not far from our major mutation R396W which, according to the present study, located in a very active part of the protein^86^. Based on their model, the R251L mutation would map near a transmembrane domain.

### Importance of the DGAT1 mutations in goat

Like other *DGAT1* mutations known to be associated with milk traits in ruminants, the major effect of the R251L and R396W mutations is on fat content^76–79,82^. The R396W mutation explains as much as 46% of the variance of the trait, which is similar to the variant with the strongest effect found in cattle^76^. We found no significant effect of the mutations on milk yield or protein content traits in contrast to what was found for the bovine K232A mutation^76,78^.

The frequency of the two mutations described here is lower than the frequency usually found for the alanine residue of the K232A mutation in different bovine populations^78^. The frequency of the bovine mutation is highly variable and differs among breeds and geographical areas. The positive effect of the mutation on MYand PC may have been selected in some breeding strategies, depending on the relative weight given to the different milk traits. Caprine mutations, although associated with lower fat contents, have not been eliminated, and their frequencies have remained stable in the male population in recent years.

The functional test revealed significant effects of the mutations on triglyceride production and strongly supports the hypothesis that these are causal mutations. We used a test that is quite similar to the functional test carried out in cattle: we measured the production of triglycerides using partially purified recombinant proteins. However, to enable more accurate estimations, we used the amount of cholesterol as a normalization factor of the membrane quantities used in the enzymatic reaction.

Another interesting point is that the R396W mutation has a stronger effect than the R251L, as shown both by the results of the *in vitro* functional test and of the *in vivo* milk analysis. However, the effect of the mutation estimated from these two experiments is not comparable.

The main possible future application of this work is the use of DGAT1 in the breeding scheme. We propose a molecular tool to test two novel mutations in the *DGAT1* gene that can be genotyped on a routine and large scale basis. One short term application in goat breeding schemes would be to include information on the DGAT1 genotype in the estimation of breeding values for fat content. Recently, Carillier-Jacquin *et al*.^28^ investigated the benefits of including *CSN1S1* major gene effect in the genetic evaluation of French dairy goats. Their results showed an improvement in predictive ability (from 6% to 27%) for the estimated breeding value, both in a genetic and genomic evaluation model (even if only males are genotyped). The potential gain of including the *DGAT1* gene for the accuracy of the prediction could even be higher than the gain estimated for the multi-allelic *CSN1S1* as they explain the same range of phenotypic variance in their respective traits and the *DGAT1* mutations are only bi-allelic. The difficulty of predicting genotypes of ungenotyped animals is indeed the main limitation to improving the accuracy of the estimated breeding values and the prediction is easier for bi-allelic mutations. On the other hand, the *DGAT1* genotyping result may help breeders chose among half-sib candidate bucks which one to bring to the breeding center for further selection and progeny testing.

## Conclusions

In this study, we identified a large number of QTLs for dairy traits in goats. Further, we identified two mutations in the *DGAT1* gene associated with a reduction in fat content and proved their causality with a functional test. These results advance our understanding of the genetic architecture of caprine milk composition and will be useful for breeding programs. Our results could also help develop a naturally low fat milk market, and are possibly transferable to other breeds by introgression or new genetic engeneering such as CRISPR-cas9^96^, if and when this technique receives social acceoptance for the genetic improvement of animals.

## Materials and Methods

### Resource population

The data for QTL detection came from a total of 2,209 commercial French dairy goats sampled in 2010 as part of the national “Genomcap” and the EU “3SR” (www.3srbreeding.eu) projects. The 2,209 animals were distributed in 20 half-sib families sired by 9 Saanen and 11 Alpine artificial insemination (AI) bucks. Family size averaged 109 (±16) daughters per buck and ranged from to 73 to 126. AI bucks were chosen to be both representative of the genetic diversity of the whole population and to maximize the genetic diversity between families. They were chosen among the widely used AI bucks with large numbers of daughters in commercial farms in 2009 and 2010.

### Ethics Statement

DNA samples for this study are stored at the Laboratoire d’Analyses Génétiques pour les Espèces Animales (LABOGENA, Jouy en Josas, France; www.labogena.fr). Neither sperm collection nor blood sampling was performed specifically for this study. Sperm was collected from bucks by the goat breeding organization Capgenes (Mignaloux Beauvoir, France; http://www.capgenes.com/), with the authorization of the DGAL (*Direction Générale de l’ALimentation*) FR CC 860. Sperm was collected at artificial insemination stations, and we used extra doses from this collection. Blood samples were taken at commercial farms. The animals were not part of any experimental design. They were sampled by veterinarians and/or under veterinarian supervision as part of routine management practices. Extra samples were requested when blood sampling took place.

### Genome-wide SNP genotyping

All 2,209 animals were genotyped using the Illumina GoatSNP50 BeadChip (53,347 SNPs). DNA was extracted from blood samples and genotyping was performed at LABOGENA, following the manufacturer’s instructions.

Data were filtered using an in-house pipeline. Briefly, any individual with a call rate <95% (N = 16) or showing pedigree inconsistency (N = 228, i.e. 10%) was discarded. SNP quality control included the following inclusion criteria: call rate >99%, minor allele frequency >1% and Hardy-Weinberg P-value > 10^–6^. After filtering, a total of 44,612 SNPs distributed on goat autosomes were retained for further analyses. Marker order and positions were based on the caprine Assembly CHIR_1.0 downloaded (December 9, 2014) from the following link: http://bioinformatics.tecnoparco.org/SNPchimp/index.php/download/download-goat-data.

### Phenotypic measurements

The traits considered were MY, PY, FY, PC and FC. Traits were standardized to 250 days of lactation. For QTL detection, yield deviations (YD) for all five traits were provided by the French national genetic evaluation computing centre^97^. For all traits, YD were raw data corrected for the fixed effects included in the genetic evaluation model: flock, age at kidding, month of kidding, dry period length, year by region combination and the random permanent environment. Next, YD were averaged over all the lactations of one animal (maximum 3 lactations per animal). A total of 1,941 phenotyped and genotyped animals were used for QTL detection. Family size averaged 97 (±16) daughters per buck (range: 60 to 115).

### Association mapping

For QTL detection, both linkage analyses (LA) and linkage disequilibrium (LD) using interval mapping were applied to the data using the QTLMap software (ref.^98^; http://dga7.jouy.inra.fr/qtlmap/). For LA, interval mapping^99^ was performed with the likelihood ratio test (LRT) using within-sire linear regression^100^. The QTL effect (average substitution effect) was expressed in deviation units (SD). Linkage disequilibrium was based on a regression analysis of the phenotypes onto founder haplotypes^101^. Analyses were performed for each haplotype of four consecutive SNPs along the chromosome. The computations of phase and transmission probabilities were optimized to be rapid and as exact as possible.

Chromosome-wide significance levels were calculated with QTLMap, using the current family structure and the MY phenotypes. For LA, the empirical 5% and 1% chromosome-wide significance levels of the test statistics were estimated from 1,000 within-family permutations^102^ for each chromosome. For LD, the empirical chromosome-wide significance level of the test statistics was estimated from 1,000 simulations for each chromosome, assuming a trait of heritability equal to 0.35. The 5% genome-wise thresholds were obtained by applying the Bonferroni correction P_genome-wise_ = 1 - (1 - P_chromosome-wise_)^n^, where n is the number of autosomes analyzed, i.e. 29.

The 95% confidence intervals of the QTL locations were estimated with the logarithm of odds drop-off^99^ implemented in QTLMap software. In practice, the bounds of the interval were the two locations where the likelihood was equal to the maximum likelihood minus 3.84 [$={x}_{(\mathrm{1.0.05})}^{2}$].

### Sequencing and SNP calling for the 20 AI buck

Because evidence already existed for high conservation between cattle and goat DGAT1 sequences^80^, the 1785 bp Bos taurus mRNA sequence (NM_174693.2) was used to identify the orthologous *DGAT1* region in the Capra hircus CHIR_1.0 genome and the exonic regions, through **sim4** program^103^. Using this information, N blocks (>9 consecutive undetermined nucleotides) were identified in the reference sequence in the *DGAT1* orthologous region extended to 15 kb upward and forward. PCR amplifications were performed to produce DNA fragments containing one or several N blocks, using the Long PCR Enzyme Mix provided by Fermentas (http://www.fermentas.de). Either PCR or internal primers were used for the Sanger sequencing reaction with the BigDye Terminator v3.1 Cycle Sequencing Kit (http://www.appliedbiosystems.com), after ExoSAP treatment^104^, and amplicons were analyzed on a ABI3730 DNA analyzer (ThermoFisher Scientific).

Sequences were aligned using **DNAbaser** software (http://www.dnabaser.com/) to generate a consensus sequence from two Alpine and two Saanen animals with extreme phenotypes for fat content.

SNP discovery was carried out by Sanger sequencing on the same animals. The genotypes of the discovered SNPs were determined using the Sanger sequencing method described above for the relevant PCR products of the remaining bucks of the QTL design.

The primers used in this study are listed in Table S4.

### Comparison of protein sequences among ruminants

The sequence of the goat *DGAT1* gene was then translated and the corresponding protein sequence was compared to those of other ruminants, The sheep and bovine sequences were extracted from Uniprot (http://www.uniprot.org/ entries A8VJM4 and Q8MK44 respectively). The weblogo software (http://weblogo.threeplusone.com/) was used to obtain a graphical representation of the conservation of the protein between three domestic ruminant (cattle, sheep and goats). Predicted protein sequences of other ruminant species (Ceratotherium simum; Bulbalus bubalis; Camelus dromedaries; Camelus bactrianus and Vicugna_pacos) were extracted from NCBI (http://www.ncbi.nlm.nih.gov/gene/?term=dgat1). A percent identity matrix was then created between all the species retained using the Clustal omega software (http://www.ebi.ac.uk/Tools/msa/clustalo/).

### DGAT1 genotyping

SNP ss# 1971466363 (corresponding to R251L mutation) and SNP ss# 1971466359 (corresponding to R396W mutation) were genotyped using a PCR-RFLP test, using *BstUI* and *MspI* (New England Biolabs) enzymes respectively. Briefly, 50 ng of the appropriate PCR product were digested with 10 U of enzyme according to the manufacturer’s instructions. After migration on a 3% TBE agarose gel, the genotypes were assessed independently by two different people. All the females from the resource population and 752 AI males were genotyped in this way.

### Association of DGAT1 genotype with milk production traits and milk fatty acid composition

A single SNP test of association was performed for each of the two mutations by performing an analysis of variance (ANOVA), using the mixed procedure in the statistical analysis system (SAS 9.1). The dependent variables were yield deviations (YD). For milk production traits (MY, PY, FY, PC, FC), YD were calculated in the context of the national genetic evaluation, similar to those used for the QTL detection. For milk fatty acid composition, YD were estimated for two traits, saturated and unsaturated fatty acids, predicted from mid-infrared spectrometry^105^. Test-day fatty acid compositions from females during their first and second lactations were corrected for six fixed effects: herd-test-day, day in milk, parity, month of kidding, season of measurement, time of day (morning or evening) of milking, intra lactation stage, and spectrometers (n = 6 machines in three laboratories, MilkoScan FT6000 and MilkoScan FT+; Foss Electric, Hillerod, Denmark).

Fatty acid composition was expressed in two different measurement units: g per 100 g of milk and g per 100 g of fat. For each of the two mutations, the three possible genotypes were fitted as a fixed explanatory variable and a significance threshold of p < 0.01 was selected. The varcomp procedure of SAS was used to estimate the proportion of variance explained by the genotype. The sire was included as a random effect in both mixed and varcomp models. For the R396W polymorphism, analyses were performed on the two breeds pooled. For the R251L polymorphism, only the Saanen breed was analyzed because too few females were carriers of the mutation in the Alpine breed.

Traits are expressed as the standard deviation of YD. The values of standard deviations were 5.39 kg for FY, 3.12 g/kg for FC, 0.50 g per 100 g of milk for SFA, 0.17 g per 100 g of milk for UFA.

### Expression of recombinant DGAT1 in Sf21 cells

DGAT1 cDNAs were obtained by RT-PCR from total RNA extracted from blood samples of two R396W heterozygous Alpine goats. The DGAT1 coding sequence was then amplified using primers 1 & 2 and cloned into TOPO TA vector (Invitrogen). The coding sequence was then reamplified from the TOPO plasmid for cloning into the pFastBac1 vector using primers 3 and 4. PCR amplifications were conducted on an ABI 9700 thermocycler (Applied Biosystems) with the following program: 1 min initial denaturation at 98 °C, 65 cycles of 30 s at 98 °C, 30 s extension at 68 °C and 75 s at 72 °C, followed by 10 min final extension at 72 °C. The 25 µL amplification mixture contained 100 ng of DNA, 1 unit Q5 high fidelity DNA polymerase (New England Biolabs), 5 µL Q5 PCR buffer, 5 µL Q5 enhancer, dNTP 200 µM and 12.5 pmol of each primer.

The PCR products were purified using QIAquick PCR purification kit (QIAgen) and double digested, as were the vectors, with BamHI-HF and XhoI (New England Biolabs). The digested inserts were then ligated into the pFastBac1 linearized vector. The different alleles (wild type, mutated R251L, mutated both R251L and R396W) were obtained from the mutated R396W clones using the QuickChange II Site-Directed Mutagenesis Kit (Agilent) to obtain the R251L mutation and to generate the wild-type codon at position 396. At each step, inserts of the clones were completely sequenced to check the integrity of the insert sequence. The primers used are listed in Table S4.

Transposition of the coding sequence into DH10Bac and multiplication and titration of recombinant virus using Sf21 cells were performed using a Bac-To-Bac Baculovirus Expression System, (Invitrogen) according to the manufacturer’s recommendations.

The harvested Sf21 cells obtained after infection were resuspended in 5 mL of a buffer containing 50 mM Tris HCl and 250 mM NaCl at pH 8. 200 µL of 25X Protease inhibitor was added and the solution was disrupted by sonication, 4 times x 10 pulses at 40%. Nuclei and large cellular debris were pelleted by centrifuging at 10,000 × g for 10 min. The supernatant was then ultracentrifuged for 1 h at 100,000 × g at 4 °C and the resulting pellet was resuspended in 250 µL of the same buffer as above.

### DGAT1 Activity Assay

DGAT1 activity was tested using the wild type DGAT1, the three recombinant DGAT1 (mutated R251L, mutated R396W and mutated both R251L and R396W) and a membrane fraction of Sf21 transfected with DDX3X used as negative control. DDX3X was a 662 amino-acid protein with no known implication in lipid metabolism.

The activity of each sample was tested in a final volume of 160 µL containing 250 mM sucrose, 1 mM EDTA, 20 mM MgCl2, 100 mM Tris HCl (pH 7.5), 20 µg free fatty acid BSA, 20 µg of diacylglycerol 10:10 and 10 µg of 14-acylCoA. The reaction was triggered by adding to this assay mixture 10, 7.5 or 5 µL of protein solution at 37 °C. The time of incubation was inversely proportional to the amount of protein added: 2.5, 3.3 or 5 min. The reaction was stopped on ice. Lipids were then extracted using the Bligh and Dyer method^106^ in presence of an internal standard (glyceryl trinonadecanoate (Tg19) 8 mg) and solid phase extraction (SPE) was carried out. Briefly a SiOH glass cartridge (200 mg, Macherey Nagel) was equilibrated with 2 ml of dichloromethane, and the extract was then put down in 20 µl of 10% methanol in dichloromethane, and neutral lipids were eluted with 2 ml of the same mixture. The final extract was concentrated, dissolved in 20 µl of ethyl acetate and analyzed by gas chromatography with flame ionization detection using the FOCUS Thermo Electron system equiped with an Zebron-1 Phenomenex fused silica capillary columns (5 m × 0.32 mm i.d, 0.50 µm film thickness). Oven temperature was programmed from 200 °C to 350 °C at a rate of 5 °C per min and the carrier gas was hydrogen (0.5 bar). The injector and the detector were programmed at 315 °C and 345 °C respectively^107^.

The amount of measured triglyceride 10:10:14 was then corrected by an internal standard (TG19) and by the amount of measured cholesterol. As the cholesterol reflects the membrane concentration, it was used to normalize the amount of membrane between the samples. Each condition was repeated three times.

### Avaliability of data and materials

SNPs were submitted to NCBI (dbSNP) under ss numbers 1971466334-1971466359 and 1971466361-1971466363. All other relevant data are within the article and its supplementary information files.

## Electronic supplementary material

Supplementary information
