# Author Correction: A genome scan for milk production traits in dairy goats reveals two new mutations in *Dgat1* reducing milk fat content

**DOI:** 10.1038/s41598-018-22118-x

**Published:** 2018-03-01

**Authors:** Pauline Martin, Isabelle Palhière, Cyrielle Maroteau, Philippe Bardou, Kamila Canale-Tabet, Julien Sarry, Florent Woloszyn, Justine Bertrand-Michel, Ines Racke, Hüseyin Besir, Rachel Rupp, Gwenola Tosser-Klopp

**Affiliations:** 1 GenPhySE, Université de Toulouse, INRA, INPT, ENVT, Castanet Tolosan, France; 2 Division of Molecular and Clinical Medecine, School of Medecine, University of Dundee, Ninewells Hospital and Medical School, Dundee, UK; 3 INRA, Sigenae, Castanet-Tolosan, France; 4 MetaToul-Lipidomic Core Facility, MetaboHUB, INSERM U 1048 Toulouse, France; 5 Protein Expression and Purification Core Facility, EMBL Heidelberg, Heidelberg, Germany

Correction to: *Scientific Reports* 10.1038/s41598-017-02052-0, published online 12 May 2017

The Acknowledgements section in this Article is incomplete.

“The authors thank Pierre Martin and all the Capgenes breeding organizations for providing the data and their helpful contributions. This work was supported by grants from French organizations through “PhénoFinlait” (a research programm including INRA, APIS-GENE, ALLICE (formerly UNCEIA), CAPGENES and FCEL) and EC (FP7/2007–2013), grant no. 245140, “3SR”, Sustainable Solutions for Small Ruminants (http://www.3srbreeding.eu/). The authors thank all the partners involved. The authors also thank Cécile Albenne for her precious help in enzymology, Stéphane Fabre, Marc Teisser and Julie Demars for their involvement and help with softwares and molecular part, and Aurélie Tircazes for her help with genotype calling. Lipidomic analyses were performed on the Toulouse MetaToul-Lipidomique Core Facility (I2MC, Inserm 1048, Toulouse, France), MetaboHUB-ANR-11-INBS-0010.”.

should read:

“The authors thank Pierre Martin and all the Capgenes breeding organizations for providing the data and their helpful contributions. This work was supported by grants from French organizations through “PhénoFinlait” (a research programm including INRA, APIS-GENE, ALLICE (formerly UNCEIA), CAPGENES and FCEL) and EC (FP7/2007–2013), grant no. 245140, “3SR”, Sustainable Solutions for Small Ruminants (http://www.3srbreeding.eu/). The authors thank all the partners involved. The authors also thank Cécile Albenne for her precious help in enzymology, Stéphane Fabre, Marc Teisser and Julie Demars for their involvement and help with softwares and molecular part, and Aurélie Tircazes for her help with genotype calling. Lipidomic analyses were performed on the Toulouse MetaToul-Lipidomique Core Facility (I2MC, Inserm 1048, Toulouse, France), MetaboHUB-ANR-11-INBS-0010. Pauline Martin received a doctoral research grant from “Région Midi-Pyrénées”, Animal Genetics Department from INRA and GENOMCAP research programm including INRA, APIS-GENE, ALLICE (formerly UNCEIA), CAPGENES and FCEL). Cyrielle Maroteau received a CIFRE grant from ALLICE”.

