# Supplementary material for: A genome scan for milk production traits in dairy goats reveals two new mutations in Dgat1 reducing milk fat content
Source: Sci Rep. 2017 May 12;7:1872. doi: 10.1038/s41598-017-02052-0 (PMC5431851; doi:10.1038/s41598-017-02052-0)
Supplement: Supplementary file 1 — Supplementary information [file 41598_2017_2052_MOESM1_ESM.pdf]

A genome scan for milk production traits in dairy goats reveals two new mutations in *Dgat1* reducing milk fat content

Pauline Martin[1],

Isabelle Palhière[1],

Cyrielle Maroteau[1,2],

Philippe Bardou[1,3],

Kamila Canale-Tabet[1],

Julien Sarry[1],

Florent Woloszyn[1],

Justine Bertrand-Michel[4],

Ines Racke[5],

Hüseyin Besir[5],

Rachel Rupp [1¶]

Gwenola Tosser-Klopp[1* ¶]

Supporting information

S1 Table: Genome scan for milk production traits in a daughter design of 1,961 dairy goats, based on association analyses (***: 5% genome-wide significance).

S1 Fig: Manhattan plot of likelihood ratio test profiles for five milk production traits: milk yield (MY), fat yield (FY), protein yield (PY), fat content (FC) and proteincontent (PC) in the joint Alpine and Saanen population. The solid horizontal lines represent the 5% genome-wide thresholds (average over the 29 autosomes).

S2 Table: Whole results from the linkage and association analyses for five milk production traits: milk yield (MY), fat yield (FY), protein yield (PY), fat content (FC) and protein content (PC) in the tree populations, i.e. Saanene, Alpine and joint Alpine and Saanen populations. The Likelihood ratio tests (LRT) are given for each position tested over the 29 autosomes.

S2 Fig: Conservation of the DGAT1 protein sequence between cattle, sheep and goat species, using the weblogo software (**http://weblogo.threeplusone.com/**). Amino-acids are colored according to their chemical properties: hydrophilic amino acids (RKDENQ) in blue, neutral amino acids (SGHTAP) in green and hydrophobic amino acids (YVMCLFIW) in black. The statistical model for letter height and error bars is detailed in the weblogo manual (http://weblogo.threeplusone.com/manual.html).

S3 Fig: Frequency of the T allele for the R396W in the artificial insemination males depending on their year of birth.

S3 Table: Percent identity matrix of the DGAT1 protein sequence between different species of ruminants.

S4 Table: List of primers used in the study. Each primer is defined by a name, its sequence, its strand, its location on LT221856 (bp), its location on chromosome 14 of the reference genome, and indications of the protocol used and the parts of the gene concerned are given.

S1 Table

CHI	TRAIT	Breed	LRTmax	Position (*100 Mb)	**95%CI** (*100 Mb)		
					min	max	
2	PC	Alpine	49,98	0,057	0,056	0,058	1
2	FC	Two breeds	52,17	0,059	0,058	0,060	2
2	FC	Alpine	48,31	0,464	0,463	0,465	3
3	PC	Saanen	46,23	0,359	0,358	0,360	4
4	FC	Two breeds	52,69	0,080	0,079	0,081	5
4	MY	Alpine	46,45	1,034	1,033	1,035	6
5	PC	Two breeds	49,93	0,316	0,315	0,317	7
5	PC	Saanen	59,10	0,317	0,316	0,318	8
**6**	**FC**	**Alpine**	**60,06**	**0,764**	**0,763**	**0,765**	9
**6**	**FC**	**Two breeds**	**83,34**	**0,826**	**0,825**	**0,828**	10
**6**	**PC**	**Two breeds**	**357,51**	**0,826**	**0,825**	**0,827**	11
**6**	**PC**	**Alpine**	**192,29**	**0,826**	**0,825**	**0,827**	12
**6**	**FC**	**Saanen**	**75,31**	**0,827**	**0,826**	**0,828**	13
**6**	**PC**	**Saanen**	**224,74**	**0,827**	**0,826**	**0,828**	14
6	MY	Saanen	48,15	0,898	0,897	0,899	15
7	FY	Two breeds	58,54	0,287	0,286	0,288	16
7	PY	Two breeds	52,18	0,298	0,297	0,299	17
7	PC	Alpine	46,82	0,403	0,402	0,404	18
7	MY	Saanen	54,02	0,554	0,553	0,555	19
7	MY	Two breeds	47,67	0,679	0,678	0,680	20
7	FY	Alpine	45,41	0,822	0,821	0,823	21
7	PY	Alpine	46,25	0,822	0,821	0,823	22
8	PY	Saanen	45,77	0,067	0,066	0,068	23
**8**	**FC**	**Two breeds**	**60,97**	**0,189**	**0,188**	**0,190**	24
**8**	**FC**	**Alpine**	**68,05**	**0,230**	**0,228**	**0,231**	25
8	MY	Saanen	44,37	0,491	0,490	0,492	26
8	MY	Two breeds	45,27	0,707	0,706	0,708	27
8	PY	Two breeds	45,86	0,968	0,967	0,969	28
10	FC	Alpine	49,46	0,983	0,981	0,984	29
11	PC	Alpine	46,99	0,059	0,058	0,060	30
11	FY	Two breeds	53,51	0,440	0,439	0,441	31
11	PY	Two breeds	51,85	0,460	0,459	0,461	32
11	PC	Two breeds	49,23	1,001	1,000	1,003	33
12	FC	Two breeds	46,48	0,087	0,086	0,088	34
12	FY	Saanen	48,98	0,150	0,149	0,151	35
13	FY	Saanen	50,10	0,784	0,783	0,785	36
14	PC	Saanen	43,04	0,099	0,098	0,101	37
**14**	**FC**	**Two breeds**	**172,77**	**0,109**	**0,106**	**0,110**	38
**14**	**FC**	**Saanen**	**112,05**	**0,115**	**0,114**	**0,116**	39
**14**	**FC**	**Alpine**	**108,35**	**0,116**	**0,114**	**0,118**	40
14	FY	Saanen	44,72	0,147	0,146	0,148	41
15	FY	Saanen	45,08	0,298	0,297	0,299	42
15	MY	Saanen	53,19	0,544	0,543	0,545	43
15	PY	Saanen	46,49	0,544	0,543	0,545	44
18	FC	Two breeds	43,50	0,003	0,002	0,004	45

18	MY	Saanen	43,67	0,217	0,216	0,218	46
18	PC	Alpine	43,74	0,564	0,563	0,565	47
19	FY	Saanen	46,68	0,221	0,220	0,222	48
19	MY	Saanen	46,42	0,246	0,245	0,248	49
**19**	**PY**	**Saanen**	**57,34**	**0,258**	**0,256**	**0,260**	50
19	PY	Two breeds	54,38	0,275	0,274	0,276	51
20	PC	Alpine	47,46	0,582	0,581	0,583	52
20	FC	Two breeds	50,99	0,592	0,591	0,593	53
20	PY	Alpine	44,88	0,629	0,628	0,630	54
20	FC	Alpine	49,03	0,674	0,673	0,675	55
21	MY	Saanen	45,18	0,080	0,079	0,081	56
21	MY	Two breeds	48,91	0,115	0,114	0,116	57
21	MY	Alpine	45,59	0,115	0,113	0,116	58
21	FC	Alpine	46,37	0,196	0,193	0,197	59
21	PC	Two breeds	44,97	0,597	0,596	0,598	60
22	PY	Two breeds	45,07	0,464	0,462	0,466	61
22	PY	Saanen	47,29	0,527	0,526	0,528	62
23	PC	Alpine	46,04	0,099	0,098	0,100	63
23	FY	Two breeds	45,34	0,217	0,216	0,218	64
23	MY	Alpine	56,00	0,331	0,330	0,332	65
23	PY	Alpine	41,99	0,331	0,330	0,332	66
23	FC	Two breeds	47,10	0,401	0,400	0,403	67
23	PC	Two breeds	43,66	0,405	0,404	0,406	68
24	MY	Saanen	45,26	0,036	0,035	0,037	69
24	PC	Saanen	42,22	0,395	0,394	0,396	70
25	MY	Two breeds	43,11	0,103	0,102	0,104	71
25	PY	Two breeds	43,76	0,103	0,102	0,104	72
25	PY	Saanen	48,48	0,116	0,115	0,117	73
25	FC	Saanen	50,35	0,154	0,153	0,155	74
26	PC	Alpine	41,31	0,062	0,061	0,064	75
26	FC	Alpine	41,35	0,164	0,163	0,165	76
26	PC	Saanen	45,11	0,243	0,242	0,244	77
27	PC	Alpine	45,06	0,058	0,057	0,059	78
27	FY	Alpine	48,31	0,317	0,316	0,318	79
27	FC	Two breeds	52,17	0,368	0,367	0,369	80
27	MY	Alpine	41,93	0,404	0,402	0,405	81
28	FC	Two breeds	44,09	0,042	0,041	0,043	82
29	MY	Alpine	47,20	0,348	0,346	0,348	83
29	PC	Alpine	46,08	0,348	0,346	0,348	84
29	PC	Two breeds	44,67	0,388	0,385	0,388	85

S1 Fig

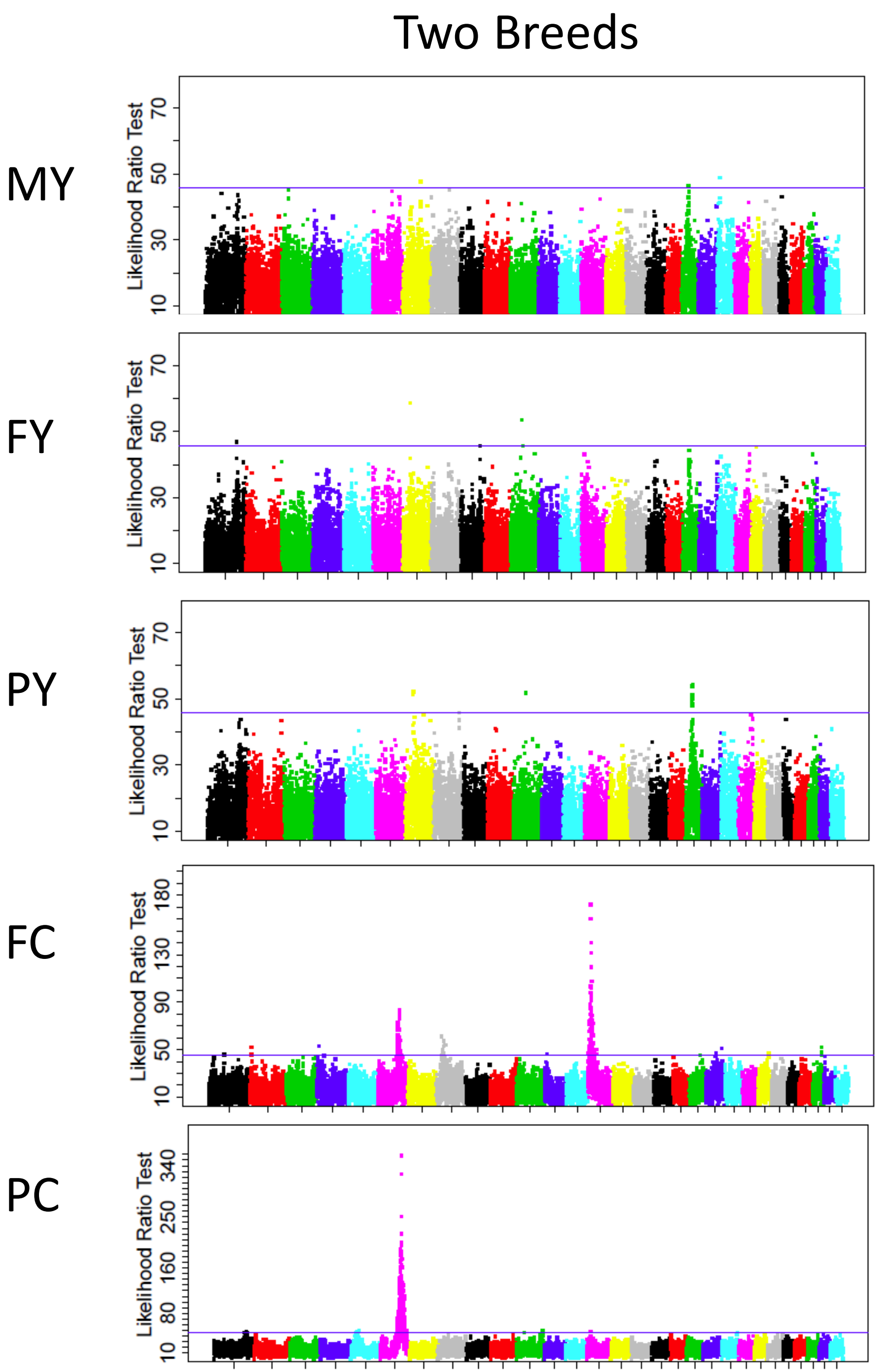

S2 Table

CHI	position (x100Mb)	LRT_MY	LRT_FY	LRT_PY	LRT_FC	LRT_PC
1	0.0005	0.000	0.000	0.000	0.000	0.000
1	0.0015	12.794	15.465	7.730	18.188	18.923
1	0.0025	12.258	17.044	12.745	27.020	22.763
1	0.0035	19.902	22.441	17.938	28.432	10.927
1	0.0045	10.166	12.248	8.271	16.609	16.692
1	0.0055	7.404	16.194	11.877	15.139	20.401
1	0.0065	5.168	9.583	2.631	23.268	16.272
1	0.0075	10.250	21.016	14.009	19.131	14.826
1	0.0085	11.803	10.569	8.082	16.840	21.786
1	0.0095	7.035	6.915	5.193	14.178	12.378
1	0.0105	6.978	9.155	4.138	18.545	15.241
1	0.0115	12.354	18.481	14.506	25.044	22.841
1	0.0125	14.033	8.227	7.862	12.707	15.408
1	0.0135	8.255	7.537	3.934	5.729	7.093
1	0.0145	8.390	10.090	6.744	13.562	11.057
1	0.0155	9.290	9.099	6.949	9.916	5.067
1	0.0165	11.239	12.278	9.567	12.154	18.435
1	0.0175	8.727	13.025	7.064	13.244	13.709
1	0.0185	11.784	14.226	11.801	14.756	12.664
1	0.0195	8.257	10.835	5.559	26.380	11.843
1	0.0205	6.849	7.455	5.971	23.449	15.554
1	0.0215	6.679	8.811	3.413	9.011	5.147
1	0.0225	4.650	7.348	6.479	12.263	13.171
1	0.0235	4.532	3.823	4.777	16.686	18.183
1	0.0245	8.053	8.634	7.546	9.821	6.060
1	0.0255	5.145	9.463	6.687	8.293	10.988
1	0.0265	5.940	7.967	4.483	16.464	7.705
1	0.0275	8.477	4.651	3.121	9.506	9.302
1	0.0285	7.729	4.158	6.100	8.769	8.379
1	0.0295	10.796	9.353	9.164	8.515	11.223
1	0.0305	19.069	15.824	13.244	9.668	12.204
1	0.0315	9.361	9.407	7.931	6.756	11.210
1	0.0325	14.653	12.363	11.697	12.723	16.813
1	0.0335	12.385	12.283	9.570	13.414	12.381
1	0.0345	8.319	10.696	8.364	9.879	7.475
1	0.0355	10.383	9.230	6.801	16.604	19.443
1	0.0365	13.683	12.894	8.339	7.383	6.353
1	0.0375	12.836	7.858	7.793	5.622	9.506
1	0.0385	23.228	13.504	15.312	13.681	8.591
1	0.0395	10.128	8.976	4.606	16.745	6.857
1	0.0405	21.391	15.374	10.661	10.433	14.143
1	0.0415	14.944	14.107	11.571	16.798	15.026
1	0.0425	14.833	13.964	11.433	16.506	14.962
1	0.0435	8.180	12.965	4.742	19.384	13.667
1	0.0445	15.336	11.731	8.652	23.305	18.140
1	0.0455	15.261	11.598	8.478	23.817	18.298
1	0.0465	16.584	23.407	11.377	24.166	11.702

1	0.0475	7.228	9.176	5.325	24.131	18.886
1	0.0485	13.468	11.000	7.627	21.781	19.346
1	0.0495	8.095	8.715	6.929	5.102	11.554
1	0.0505	9.134	12.189	3.891	5.127	11.717
1	0.0515	10.531	6.597	2.712	16.576	22.649
1	0.0525	8.045	5.847	2.778	10.598	19.448
1	0.0535	11.308	11.797	8.074	9.870	9.563
1	0.0545	9.063	5.897	4.848	7.721	19.160
1	0.0555	8.488	9.636	10.520	10.449	23.593
1	0.0565	11.491	8.516	7.135	14.274	22.949
1	0.0575	7.224	6.624	4.120	13.097	15.220
1	0.0585	9.085	8.991	6.895	12.731	11.977
1	0.0595	9.659	15.190	11.321	15.853	14.306
1	0.0605	12.200	18.439	9.964	16.871	10.261
1	0.0615	15.242	19.614	15.079	15.172	8.860
1	0.0625	17.334	17.186	11.396	14.045	13.619
1	0.0635	13.855	8.417	14.620	5.013	13.428
1	0.0645	11.124	8.472	11.892	9.682	9.720
1	0.0655	11.710	15.607	10.808	20.770	14.299
1	0.0665	8.021	9.759	8.106	20.837	19.935
1	0.0675	10.260	4.367	6.723	15.286	13.894
1	0.0685	12.600	7.565	8.730	15.804	13.686
1	0.0695	6.362	6.581	4.620	27.271	29.443
1	0.0705	7.139	2.398	6.967	16.754	22.818
1	0.0715	13.149	9.426	13.633	18.726	31.791
1	0.0725	10.095	10.679	13.107	18.239	22.206
1	0.0735	5.836	7.652	6.280	26.757	21.815
1	0.0745	4.750	8.008	2.463	28.312	21.605
1	0.0755	6.364	9.782	6.354	16.840	13.502
1	0.0765	14.759	8.764	14.686	18.235	25.616
1	0.0775	11.333	14.417	12.362	19.821	22.420
1	0.0785	12.440	16.920	19.238	14.619	28.985
1	0.0795	17.838	20.993	18.044	17.866	34.350
1	0.0805	7.860	5.639	5.242	18.235	28.810
1	0.0815	14.506	9.451	8.170	21.624	15.779
1	0.0825	7.054	14.159	9.547	21.324	11.354
1	0.0835	12.538	17.167	13.693	29.059	20.261
1	0.0845	6.463	1.880	4.658	7.143	18.743
1	0.0855	8.844	3.754	6.129	9.695	13.787
1	0.0865	11.071	11.554	12.111	12.451	15.244
1	0.0875	8.070	9.319	8.145	10.195	8.693
1	0.0885	3.935	6.416	4.746	15.723	10.493
1	0.0895	11.108	9.938	13.077	19.356	24.563
1	0.0905	15.003	7.807	12.222	26.853	34.215
1	0.0915	7.592	9.093	6.054	24.543	18.868
1	0.0925	9.865	10.917	8.298	18.944	11.955
1	0.0935	14.919	20.637	16.691	18.574	10.052
1	0.0945	9.300	16.545	12.201	19.790	9.936
1	0.0955	9.994	11.758	7.173	15.680	4.847
1	0.0965	8.928	11.497	6.656	18.669	13.340

1	0.0975	12.073	9.896	12.430	16.902	19.724
1	0.0985	12.308	13.790	11.032	15.197	12.937
1	0.0995	15.064	15.016	12.524	19.457	27.459
1	0.1005	13.793	11.318	11.051	8.212	15.622
1	0.1015	10.795	9.416	8.399	10.455	8.664
1	0.1025	5.653	11.867	5.506	18.706	12.343
1	0.1035	8.565	8.324	6.705	24.106	10.842
1	0.1045	11.999	13.242	13.792	11.835	9.447
1	0.1055	15.219	12.585	12.880	9.664	12.416
1	0.1065	10.936	8.954	9.683	13.111	11.877
1	0.1075	7.665	3.404	6.860	14.756	10.977
1	0.1085	3.852	3.218	4.897	8.034	15.379
1	0.1095	6.714	6.064	7.898	8.789	19.866
1	0.1105	18.471	17.110	15.394	18.730	19.427
1	0.1115	16.709	12.632	9.395	8.665	15.758
1	0.1125	16.263	10.452	8.724	26.516	25.508
1	0.1135	20.186	13.589	12.358	26.340	15.916
1	0.1145	15.441	9.189	11.582	15.652	17.650
1	0.1155	10.382	18.477	12.475	28.791	15.109
1	0.1165	9.487	14.960	6.872	24.675	13.320
1	0.1175	12.470	10.019	11.941	14.402	11.184
1	0.1185	16.134	16.970	12.201	20.943	6.479
1	0.1195	16.164	17.074	12.191	20.859	6.560
1	0.1205	18.709	13.048	12.560	20.171	15.755
1	0.1215	12.710	12.787	12.146	21.164	20.909
1	0.1225	21.796	11.142	13.066	24.227	8.533
1	0.1235	14.684	12.922	14.974	11.935	10.534
1	0.1245	14.618	11.680	14.098	17.481	11.257
1	0.1255	5.409	3.212	4.689	22.435	14.091
1	0.1265	19.066	17.391	13.239	22.871	19.678
1	0.1275	13.672	14.461	11.895	19.726	11.638
1	0.1285	15.995	14.542	10.247	24.917	14.557
1	0.1295	14.121	8.820	6.257	9.669	19.343
1	0.1305	7.304	9.879	5.054	9.912	9.676
1	0.1315	11.441	26.976	11.592	40.627	9.363
1	0.1325	16.122	16.288	16.042	21.133	8.857
1	0.1335	11.621	7.873	5.169	15.140	4.239
1	0.1345	8.728	7.590	5.272	27.822	7.641
1	0.1355	13.309	9.512	8.935	34.719	13.870
1	0.1365	13.971	12.688	13.411	12.898	34.199
1	0.1375	12.367	13.328	10.676	20.292	14.698
1	0.1385	15.181	16.494	13.411	17.149	14.062
1	0.1395	22.448	19.543	13.482	18.997	11.698
1	0.1405	18.262	18.725	17.459	22.714	17.929
1	0.1415	9.559	20.815	8.231	33.727	16.379
1	0.1425	13.394	16.651	6.882	16.500	8.724
1	0.1435	7.209	13.505	10.171	12.509	14.438
1	0.1445	9.554	19.613	12.804	20.928	9.382
1	0.1455	23.598	22.795	16.272	31.364	19.063
1	0.1465	19.456	13.709	11.653	32.049	23.850

1	0.1475	9.622	11.442	11.031	13.191	13.288
1	0.1485	7.936	15.153	8.611	19.089	13.383
1	0.1495	23.880	19.927	14.714	17.562	22.424
1	0.1505	30.817	31.743	21.239	37.518	28.553
1	0.1515	9.433	19.720	10.035	32.536	20.232
1	0.1525	12.822	24.436	13.202	43.004	22.287
1	0.1535	14.604	20.891	15.178	38.842	23.909
1	0.1545	19.960	16.007	12.310	7.316	6.416
1	0.1555	18.680	22.183	15.098	22.267	8.727
1	0.1565	17.287	14.205	7.649	14.742	11.708
1	0.1575	24.436	15.640	24.272	7.991	21.252
1	0.1585	36.213	28.949	26.918	24.090	29.611
1	0.1595	9.733	8.475	5.059	16.488	20.683
1	0.1605	6.068	12.583	5.378	14.110	11.926
1	0.1615	7.373	9.199	6.034	19.636	10.175
1	0.1625	21.616	17.286	13.220	29.019	19.782
1	0.1635	18.786	11.899	12.958	25.510	18.459
1	0.1645	13.732	11.349	12.173	15.767	20.548
1	0.1655	23.830	19.683	15.922	24.787	18.738
1	0.1665	22.779	20.374	12.565	30.380	23.473
1	0.1675	22.690	20.221	12.607	30.383	23.480
1	0.1685	16.010	11.886	7.990	24.483	15.400
1	0.1695	12.380	7.823	8.402	24.395	10.074
1	0.1705	15.241	15.968	7.429	44.342	16.598
1	0.1715	12.719	12.828	8.592	21.229	17.591
1	0.1725	18.516	13.611	14.997	20.248	22.308
1	0.1735	20.943	7.698	13.288	10.847	8.330
1	0.1745	13.558	12.366	9.101	21.827	13.392
1	0.1755	17.378	18.847	12.027	29.833	22.544
1	0.1765	10.767	13.995	10.245	21.296	14.050
1	0.1775	10.810	13.929	10.278	21.263	14.058
1	0.1785	9.148	13.245	11.458	13.864	5.684
1	0.1795	5.030	5.698	5.022	8.652	6.206
1	0.1805	8.732	13.469	6.927	17.641	7.207
1	0.1815	11.140	22.354	8.335	32.506	9.163
1	0.1825	19.697	13.277	12.981	15.947	6.301
1	0.1835	26.646	18.685	19.136	21.174	17.185
1	0.1845	36.863	23.822	24.776	23.258	16.076
1	0.1855	20.116	7.649	9.799	17.978	20.500
1	0.1865	19.403	18.096	17.064	35.786	21.621
1	0.1875	20.371	20.156	21.482	17.382	12.103
1	0.1885	18.956	14.497	18.221	13.937	12.087
1	0.1895	14.541	7.731	6.602	15.017	11.635
1	0.1905	11.563	7.643	6.870	21.717	17.251
1	0.1915	9.233	19.738	8.748	38.257	22.479
1	0.1925	8.820	11.009	7.015	17.949	6.489
1	0.1935	15.256	18.848	12.837	31.929	14.576
1	0.1945	14.531	20.194	11.640	41.921	20.911
1	0.1955	7.619	15.477	7.858	20.626	11.804
1	0.1965	11.181	14.986	6.878	22.182	15.832

1	0.1975	13.029	16.278	10.883	24.898	21.595
1	0.1985	11.986	15.370	12.901	21.164	17.971
1	0.1995	14.938	19.815	15.972	20.740	11.681
1	0.2005	17.047	21.290	17.724	29.953	12.508
1	0.2015	30.889	21.133	27.962	36.489	19.959
1	0.2025	6.256	13.363	9.186	18.104	11.190
1	0.2035	16.799	14.283	15.128	11.856	17.940
1	0.2045	5.912	6.666	8.988	17.530	14.406
1	0.2055	7.061	4.453	5.449	23.606	22.950
1	0.2065	22.437	11.341	18.375	28.035	16.161
1	0.2075	17.411	10.852	11.345	28.481	17.148
1	0.2085	17.308	10.809	11.325	28.471	17.115
1	0.2095	10.002	6.413	6.141	14.273	28.584
1	0.2105	7.868	9.618	11.950	19.885	14.393
1	0.2115	13.490	18.188	11.519	23.829	10.909
1	0.2125	14.394	9.177	8.956	17.477	13.813
1	0.2135	14.317	9.117	8.956	17.423	13.899
1	0.2145	10.258	7.076	8.485	21.161	13.052
1	0.2155	13.779	14.109	11.143	19.824	18.252
1	0.2165	15.192	15.599	18.047	22.868	14.012
1	0.2175	11.997	12.024	9.026	16.386	15.875
1	0.2185	14.803	9.036	6.017	26.083	12.650
1	0.2195	18.737	8.869	7.799	27.472	15.685
1	0.2205	22.963	22.728	26.134	17.105	23.637
1	0.2215	22.994	22.734	26.137	17.081	23.663
1	0.2225	5.881	12.407	6.524	11.650	5.090
1	0.2235	9.902	12.146	9.783	11.893	6.698
1	0.2245	16.849	7.961	12.319	19.362	6.503
1	0.2255	18.051	12.401	10.892	14.392	21.268
1	0.2265	19.753	12.424	23.370	12.556	22.650
1	0.2275	17.098	13.480	17.469	33.873	23.119
1	0.2285	10.799	11.095	8.841	23.833	21.769
1	0.2295	14.771	11.665	13.243	36.491	27.615
1	0.2305	10.452	8.084	8.238	30.071	19.955
1	0.2315	14.522	9.801	10.562	35.262	25.786
1	0.2325	11.988	12.684	11.443	29.192	22.304
1	0.2335	20.124	14.569	9.969	41.605	37.121
1	0.2345	18.690	15.237	10.534	33.992	26.696
1	0.2355	19.047	12.085	12.268	19.531	21.318
1	0.2365	14.465	8.182	13.516	18.923	12.469
1	0.2375	12.745	12.170	10.390	25.908	16.938
1	0.2385	4.971	3.945	3.711	19.671	10.773
1	0.2395	14.467	6.006	7.448	24.708	18.821
1	0.2405	16.922	15.474	11.224	26.214	14.961
1	0.2415	10.421	7.822	11.221	28.774	21.486
1	0.2425	21.441	18.254	15.467	35.188	13.949
1	0.2435	10.448	11.449	9.477	35.146	10.488
1	0.2445	19.582	14.268	14.484	19.297	9.274
1	0.2455	19.693	14.107	8.918	11.497	17.132
1	0.2465	20.177	9.852	11.414	12.205	14.179

1	0.2475	19.642	17.673	17.284	18.326	13.834
1	0.2485	24.780	15.535	13.092	25.114	15.608
1	0.2495	28.593	18.029	18.208	35.365	19.945
1	0.2505	12.914	5.852	9.746	11.713	16.528
1	0.2515	9.086	9.291	8.443	18.820	19.000
1	0.2525	14.995	13.640	14.162	16.643	12.590
1	0.2535	6.805	8.778	8.509	18.442	7.477
1	0.2545	11.240	12.317	12.055	17.144	18.307
1	0.2555	14.623	12.427	9.194	21.231	17.574
1	0.2565	10.986	4.186	6.378	17.904	8.051
1	0.2575	23.070	12.438	14.720	10.826	17.532
1	0.2585	26.269	11.741	10.269	24.757	20.830
1	0.2595	16.717	8.619	13.083	21.163	32.835
1	0.2605	17.986	12.522	14.556	12.752	18.653
1	0.2615	23.081	12.134	13.161	24.884	13.658
1	0.2625	19.599	2.833	5.728	19.860	26.895
1	0.2635	5.714	2.430	1.437	11.541	12.784
1	0.2645	14.233	10.102	11.391	19.609	13.872
1	0.2655	16.900	10.585	9.072	22.127	11.996
1	0.2665	9.043	6.630	9.128	17.931	18.218
1	0.2675	10.186	6.563	12.746	21.092	18.054
1	0.2685	15.171	6.872	10.572	21.301	21.471
1	0.2695	10.462	9.814	8.958	20.622	13.442
1	0.2705	7.793	5.695	9.810	17.716	9.479
1	0.2715	23.796	14.189	20.894	8.895	11.148
1	0.2725	19.987	19.193	24.055	17.185	16.924
1	0.2735	12.983	8.059	9.789	19.830	21.940
1	0.2745	12.973	5.502	9.066	7.875	14.985
1	0.2755	20.652	12.052	18.953	22.724	14.960
1	0.2765	17.977	9.283	8.855	18.388	10.924
1	0.2775	10.670	10.110	11.471	16.284	10.139
1	0.2785	6.555	7.251	9.362	9.973	8.686
1	0.2795	17.807	12.854	18.605	14.378	9.256
1	0.2805	19.736	7.962	14.106	6.390	15.155
1	0.2815	17.313	8.158	8.466	7.995	14.794
1	0.2825	11.142	3.217	9.821	13.288	13.238
1	0.2835	13.017	5.861	10.173	13.476	16.887
1	0.2845	18.038	15.449	16.953	15.409	11.707
1	0.2855	9.673	7.486	10.842	17.670	13.489
1	0.2865	30.278	14.669	30.728	20.536	13.599
1	0.2875	30.737	13.529	27.471	17.237	13.245
1	0.2885	19.420	19.589	20.289	16.270	21.461
1	0.2895	5.991	9.509	10.670	24.582	16.403
1	0.2905	7.327	11.366	6.375	19.040	14.529
1	0.2915	6.375	8.224	6.859	21.559	14.170
1	0.2925	16.405	16.650	21.257	19.911	11.637
1	0.2935	12.117	13.768	15.960	25.617	19.591
1	0.2945	16.999	9.635	15.978	18.985	18.997
1	0.2955	11.521	4.741	9.858	23.247	17.586
1	0.2965	4.989	6.821	11.727	19.165	12.813

1	0.2975	22.209	15.992	18.052	21.032	14.511
1	0.2985	13.892	19.221	15.366	33.081	12.648
1	0.2995	19.993	22.745	21.801	29.607	21.035
1	0.3005	7.809	9.082	11.075	7.879	6.199
1	0.3015	30.837	17.463	28.195	10.259	9.097
1	0.3025	14.376	8.459	10.645	6.326	19.501
1	0.3035	6.563	10.139	9.695	17.292	16.334
1	0.3045	12.789	9.916	9.619	23.037	9.845
1	0.3055	9.897	8.850	6.546	25.104	18.905
1	0.3065	15.220	11.956	18.938	8.878	12.228
1	0.3075	12.911	8.831	12.501	19.623	17.026
1	0.3085	13.375	9.612	10.659	12.492	15.286
1	0.3095	13.363	9.607	10.649	12.499	15.278
1	0.3105	17.229	18.785	20.530	12.146	17.269
1	0.3115	26.059	23.839	27.210	26.325	15.882
1	0.3125	13.546	12.818	15.761	22.233	14.655
1	0.3135	12.993	18.224	13.774	26.181	25.519
1	0.3145	12.762	5.637	9.776	18.917	9.045
1	0.3155	14.542	9.693	15.897	7.193	6.979
1	0.3165	24.253	16.390	25.390	18.145	12.825
1	0.3175	13.782	8.652	15.944	12.644	8.439
1	0.3185	15.777	9.341	19.525	7.832	10.799
1	0.3195	6.743	5.226	9.623	7.458	15.005
1	0.3205	14.967	14.190	19.141	14.759	17.592
1	0.3215	15.163	14.008	15.912	14.440	13.862
1	0.3225	8.444	11.565	12.304	4.499	10.714
1	0.3235	10.237	14.468	13.599	24.818	14.562
1	0.3245	19.244	12.220	15.641	27.634	23.387
1	0.3255	22.458	24.650	24.005	31.153	18.127
1	0.3265	27.122	12.411	21.149	24.478	12.703
1	0.3275	30.170	12.969	18.029	22.238	12.567
1	0.3285	26.833	6.335	16.831	16.650	17.877
1	0.3295	20.935	8.579	16.028	15.881	16.775
1	0.3305	18.341	16.331	14.506	26.326	16.361
1	0.3315	17.694	13.730	15.672	24.443	18.502
1	0.3325	37.628	14.207	29.414	29.687	15.573
1	0.3335	13.181	5.329	10.211	16.062	13.548
1	0.3345	18.426	8.420	16.997	26.018	21.731
1	0.3355	18.137	11.357	18.625	25.699	22.214
1	0.3365	17.555	10.869	17.312	29.348	20.943
1	0.3375	31.102	21.206	25.636	14.456	15.954
1	0.3385	18.233	13.201	15.395	13.866	22.849
1	0.3395	21.457	9.518	20.994	19.176	16.992
1	0.3405	20.634	9.683	18.868	33.127	19.867
1	0.3415	5.463	1.657	3.243	15.724	7.454
1	0.3425	8.929	8.577	9.129	19.653	18.680
1	0.3435	17.467	12.901	13.737	36.187	25.308
1	0.3445	14.053	7.996	7.780	30.444	14.392
1	0.3455	14.187	9.843	14.192	26.783	13.357
1	0.3465	18.249	9.125	17.088	23.112	20.904

1	0.3475	28.542	24.402	21.929	16.953	15.310
1	0.3485	36.197	25.807	24.851	16.592	15.049
1	0.3495	19.828	14.512	21.822	14.567	6.613
1	0.3505	24.140	14.868	21.047	29.985	10.900
1	0.3515	17.931	17.602	20.297	11.609	9.377
1	0.3525	19.627	21.916	22.903	23.570	16.828
1	0.3535	23.192	12.697	17.682	26.874	16.978
1	0.3545	21.145	14.522	18.026	19.446	7.639
1	0.3555	33.519	17.363	26.031	19.284	11.834
1	0.3565	23.696	19.560	22.203	16.603	6.162
1	0.3575	11.171	14.990	16.444	19.144	8.589
1	0.3585	14.782	12.144	13.053	17.355	16.086
1	0.3595	14.317	7.814	11.638	12.387	12.509
1	0.3605	12.471	9.454	18.302	10.646	11.152
1	0.3615	19.810	13.577	18.935	15.919	12.171
1	0.3625	30.172	18.630	23.936	22.990	20.177
1	0.3635	16.707	13.474	11.782	10.097	25.024
1	0.3645	13.125	10.606	12.396	13.622	25.780
1	0.3655	29.031	16.303	21.768	11.331	15.021
1	0.3665	12.726	7.529	12.579	11.696	13.733
1	0.3675	12.028	11.634	13.684	5.212	10.330
1	0.3685	9.578	8.434	9.235	1.822	4.498
1	0.3695	23.942	6.851	14.079	23.240	13.876
1	0.3705	10.180	5.313	8.778	19.819	18.146
1	0.3715	16.736	4.578	6.016	15.792	21.509
1	0.3725	31.604	9.771	18.335	19.453	14.560
1	0.3735	24.523	8.894	18.265	20.336	13.666
1	0.3745	13.726	8.636	9.007	24.294	7.835
1	0.3755	19.627	10.125	11.993	23.199	25.123
1	0.3765	17.934	15.417	15.876	21.309	27.452
1	0.3775	16.866	9.008	11.267	17.993	14.193
1	0.3785	7.429	9.310	2.896	12.430	8.517
1	0.3795	11.862	17.942	9.584	17.860	8.736
1	0.3805	8.272	7.828	5.544	22.653	21.275
1	0.3815	15.405	13.710	18.662	26.377	26.814
1	0.3825	11.294	20.781	11.998	23.022	19.655
1	0.3835	8.913	8.795	6.145	19.636	15.383
1	0.3845	8.066	8.487	10.779	25.405	7.359
1	0.3855	7.026	8.226	9.147	22.143	16.143
1	0.3865	4.285	3.878	2.828	22.540	16.243
1	0.3875	9.507	8.706	9.587	16.055	23.306
1	0.3885	18.265	10.018	13.560	29.707	25.830
1	0.3895	16.033	8.963	12.098	23.561	27.446
1	0.3905	13.576	6.412	8.054	20.462	13.935
1	0.3915	12.213	6.032	6.562	17.325	15.906
1	0.3925	11.978	13.236	8.346	19.971	12.564
1	0.3935	10.542	7.377	9.878	10.336	13.002
1	0.3945	13.042	9.847	10.345	11.390	18.514
1	0.3955	21.735	16.330	20.737	23.265	18.296
1	0.3965	10.551	13.566	11.452	13.227	14.829

1	0.3975	6.771	7.301	9.132	11.878	10.544
1	0.3985	3.871	2.918	3.238	12.424	5.813
1	0.3995	13.461	18.941	13.351	29.594	13.520
1	0.4005	9.700	14.560	11.014	26.892	8.659
1	0.4015	12.580	10.387	12.798	31.144	14.925
1	0.4025	22.689	15.557	15.377	14.571	27.659
1	0.4035	16.566	13.702	15.313	12.897	14.326
1	0.4045	9.680	7.467	8.558	12.910	16.137
1	0.4055	18.550	12.043	8.953	16.939	19.850
1	0.4065	15.298	18.790	18.220	18.474	14.375
1	0.4075	17.269	9.227	13.766	15.161	21.088
1	0.4085	16.865	10.030	9.648	17.386	12.833
1	0.4095	5.762	15.349	8.752	20.112	6.922
1	0.4105	17.276	15.133	17.120	14.653	6.365
1	0.4115	8.047	5.505	6.048	18.603	5.757
1	0.4125	6.687	2.310	6.316	21.886	16.487
1	0.4135	15.082	10.231	12.938	19.771	9.311
1	0.4145	5.195	13.124	8.150	10.064	7.765
1	0.4155	19.622	22.832	27.173	7.999	8.042
1	0.4165	23.534	16.791	23.937	18.371	18.985
1	0.4175	15.248	9.516	16.922	15.939	15.823
1	0.4185	18.684	6.657	13.079	25.413	11.223
1	0.4195	14.275	8.357	10.861	18.943	17.030
1	0.4205	12.774	12.367	10.225	7.160	13.940
1	0.4215	12.016	7.444	9.971	11.691	18.051
1	0.4225	14.013	8.598	12.431	6.650	10.041
1	0.4235	17.457	9.916	14.685	21.604	11.725
1	0.4245	17.461	9.931	14.691	21.625	11.727
1	0.4255	17.494	7.035	13.043	20.892	11.333
1	0.4265	17.474	7.048	13.023	20.906	11.338
1	0.4275	14.805	7.257	8.878	26.818	16.615
1	0.4285	14.976	11.271	12.139	22.139	13.262
1	0.4295	11.663	8.238	12.138	10.155	17.887
1	0.4305	9.028	13.991	11.217	14.880	10.099
1	0.4315	10.639	12.937	11.937	18.223	11.113
1	0.4325	7.649	10.842	10.940	19.747	11.074
1	0.4335	7.694	14.705	9.621	20.334	11.188
1	0.4345	10.387	15.575	15.649	21.849	25.599
1	0.4355	13.006	10.922	20.614	20.610	21.601
1	0.4365	19.584	10.261	18.806	13.571	13.730
1	0.4375	14.789	14.327	14.943	17.980	4.735
1	0.4385	17.543	12.330	13.697	17.991	16.001
1	0.4395	13.774	13.864	12.239	9.330	9.294
1	0.4405	16.186	13.110	14.689	20.683	6.695
1	0.4415	24.530	18.482	21.894	8.563	16.178
1	0.4425	25.336	19.965	27.844	13.074	9.572
1	0.4435	7.801	12.625	11.097	23.292	13.925
1	0.4445	8.247	14.845	14.375	18.858	21.500
1	0.4455	25.409	19.089	19.303	25.720	15.241
1	0.4465	14.030	14.439	6.671	10.358	10.471

1	0.4475	11.228	7.998	7.167	7.125	12.665
1	0.4485	18.025	11.578	9.286	19.691	18.176
1	0.4495	18.056	11.388	9.271	19.626	18.135
1	0.4505	10.377	13.195	9.148	18.704	8.163
1	0.4515	11.960	9.569	10.550	19.642	13.898
1	0.4525	9.169	5.733	5.118	16.765	6.322
1	0.4535	10.818	7.298	8.715	14.292	5.619
1	0.4545	13.935	22.832	13.990	16.627	20.308
1	0.4555	8.798	8.873	6.044	22.889	13.238
1	0.4565	11.945	8.456	8.246	16.204	19.244
1	0.4575	5.990	11.509	8.036	13.120	18.541
1	0.4585	13.058	6.157	9.279	8.480	8.188
1	0.4595	12.792	4.203	6.921	10.792	13.645
1	0.4605	15.150	11.591	19.056	19.065	8.269
1	0.4615	8.566	18.755	14.420	25.370	17.594
1	0.4625	19.351	8.198	13.138	23.506	20.077
1	0.4635	17.281	12.449	13.230	22.816	19.960
1	0.4645	18.709	12.698	9.591	25.849	13.928
1	0.4655	7.443	8.978	7.397	18.512	7.003
1	0.4665	13.210	8.686	9.554	18.693	13.254
1	0.4675	19.812	9.241	15.340	17.163	18.517
1	0.4685	15.803	20.749	17.510	21.185	20.802
1	0.4695	19.807	16.966	18.316	14.766	22.344
1	0.4705	10.574	8.368	7.202	15.185	19.011
1	0.4715	10.196	12.671	13.150	29.143	12.392
1	0.4725	8.544	8.634	9.494	28.342	15.739
1	0.4735	8.890	14.646	7.430	28.255	24.782
1	0.4745	12.577	9.025	13.209	18.257	19.708
1	0.4755	9.140	9.199	9.861	15.492	21.113
1	0.4765	7.129	3.002	7.335	5.971	6.819
1	0.4775	7.428	8.144	6.466	8.864	5.341
1	0.4785	7.663	9.774	9.560	12.794	20.824
1	0.4795	16.778	15.486	15.498	25.150	26.882
1	0.4805	11.969	12.919	16.835	20.615	14.387
1	0.4815	13.412	7.116	13.275	14.042	16.546
1	0.4825	9.016	10.239	11.650	15.929	3.658
1	0.4835	13.522	15.962	21.322	18.848	10.062
1	0.4845	13.153	11.627	17.580	10.650	5.343
1	0.4855	11.603	14.828	12.302	23.538	9.862
1	0.4865	12.211	13.333	15.883	21.247	9.186
1	0.4875	20.329	17.750	21.325	19.016	7.040
1	0.4885	6.790	7.819	5.136	13.325	5.766
1	0.4895	9.537	10.714	16.577	9.577	11.569
1	0.4905	18.225	17.574	32.008	9.476	9.816
1	0.4915	18.192	17.572	31.969	9.423	9.796
1	0.4925	3.437	11.466	9.743	8.080	13.087
1	0.4935	9.731	12.791	12.276	8.118	9.445
1	0.4945	13.851	10.249	9.163	14.631	9.264
1	0.4955	9.622	11.644	10.474	10.620	10.004
1	0.4965	10.333	6.014	8.989	7.350	12.727

1	0.4975	17.209	20.542	22.897	15.548	17.333
1	0.4985	9.599	13.132	16.061	15.518	24.654
1	0.4995	9.180	17.458	9.790	13.569	11.064
1	0.5005	7.770	11.923	8.607	18.263	18.366
1	0.5015	4.844	12.431	8.325	22.892	17.393
1	0.5025	7.177	11.580	10.536	20.393	21.524
1	0.5035	13.209	20.337	16.414	20.137	26.891
1	0.5045	14.376	13.534	11.781	21.241	20.804
1	0.5055	15.909	18.205	13.522	29.570	19.601
1	0.5065	12.727	10.863	12.896	28.151	16.314
1	0.5075	15.731	7.949	14.231	11.032	10.716
1	0.5085	13.202	9.464	14.584	14.601	15.490
1	0.5095	7.954	9.511	11.575	20.391	15.273
1	0.5105	15.272	14.804	18.108	26.730	21.918
1	0.5115	20.329	15.099	18.657	28.785	17.270
1	0.5125	23.627	15.893	20.269	22.181	7.860
1	0.5135	24.467	8.585	24.415	20.115	6.529
1	0.5145	17.454	16.319	15.139	25.543	15.805
1	0.5155	9.388	11.725	10.616	17.827	19.131
1	0.5165	10.914	17.679	11.973	28.944	20.690
1	0.5175	14.041	10.812	9.502	15.952	9.324
1	0.5185	13.692	11.452	13.599	8.454	33.211
1	0.5195	32.219	20.231	30.146	11.536	22.498
1	0.5205	23.767	18.691	23.913	10.729	14.845
1	0.5215	14.681	8.018	15.366	15.370	13.795
1	0.5225	5.540	3.998	7.258	12.838	24.619
1	0.5235	14.431	15.483	24.770	28.027	25.773
1	0.5245	11.826	15.291	11.742	28.386	18.952
1	0.5255	15.151	13.642	16.358	27.282	14.476
1	0.5265	12.275	15.824	12.014	23.105	13.745
1	0.5275	14.152	8.986	9.901	20.403	13.399
1	0.5285	3.840	10.664	6.772	15.248	12.436
1	0.5295	5.907	7.705	7.158	16.595	15.283
1	0.5305	5.327	6.014	4.392	20.822	9.253
1	0.5315	20.010	16.420	24.133	15.777	9.561
1	0.5325	21.495	19.648	20.675	19.577	9.063
1	0.5335	18.119	17.661	20.667	14.123	21.576
1	0.5345	21.041	16.165	17.157	17.073	22.239
1	0.5355	22.500	10.272	20.286	25.687	20.332
1	0.5365	14.258	15.170	17.957	30.827	12.735
1	0.5375	12.877	6.907	11.535	26.842	20.015
1	0.5385	12.471	9.285	15.938	19.109	13.207
1	0.5395	8.479	6.012	9.945	14.108	18.244
1	0.5405	8.575	7.654	5.100	12.328	8.753
1	0.5415	23.145	9.802	15.864	16.112	18.280
1	0.5425	15.355	10.787	12.653	15.287	32.970
1	0.5435	19.348	13.862	18.217	11.704	18.595
1	0.5445	13.098	9.055	12.939	12.717	11.489
1	0.5455	6.900	4.055	4.113	11.624	10.926
1	0.5465	11.306	5.627	10.227	13.368	17.000

1	0.5475	5.383	5.483	1.478	17.791	18.120
1	0.5485	7.944	13.486	8.596	15.976	15.469
1	0.5495	8.146	8.873	4.249	14.558	14.422
1	0.5505	9.813	8.113	7.027	15.367	24.575
1	0.5515	14.555	8.939	11.538	12.033	22.534
1	0.5525	16.092	9.762	10.723	13.383	28.496
1	0.5535	7.907	7.449	9.856	20.047	21.754
1	0.5545	28.040	17.828	29.871	20.378	11.883
1	0.5555	12.455	5.523	11.947	15.370	24.957
1	0.5565	14.994	7.064	12.521	19.444	27.827
1	0.5575	20.705	7.653	11.617	19.313	36.384
1	0.5585	23.668	12.317	21.146	15.895	15.058
1	0.5595	21.964	15.548	18.027	12.434	13.833
1	0.5605	20.946	19.013	15.898	11.914	31.060
1	0.5615	15.734	7.950	7.378	16.000	30.733
1	0.5625	14.938	9.766	11.240	10.260	18.825
1	0.5635	29.048	14.408	21.022	23.837	15.070
1	0.5645	23.191	15.642	21.176	15.013	30.056
1	0.5655	27.938	19.164	23.521	9.733	30.738
1	0.5665	11.654	6.673	6.916	17.128	15.245
1	0.5675	12.980	8.564	11.790	19.455	15.674
1	0.5685	17.828	10.743	13.439	18.971	31.133
1	0.5695	24.199	14.524	15.367	20.156	17.347
1	0.5705	20.926	9.535	13.537	22.648	16.038
1	0.5715	20.932	9.565	13.575	22.651	16.047
1	0.5725	22.497	15.960	17.079	35.319	32.205
1	0.5735	10.686	8.342	7.080	11.885	28.966
1	0.5745	10.071	7.089	5.248	14.617	31.556
1	0.5755	11.830	13.476	12.492	20.551	17.522
1	0.5765	8.644	12.389	7.752	14.614	26.675
1	0.5775	14.633	17.610	14.328	15.669	26.511
1	0.5785	14.926	11.757	10.572	8.371	27.142
1	0.5795	23.059	19.569	20.861	17.556	22.278
1	0.5805	15.050	10.017	15.864	20.427	21.566
1	0.5815	14.937	6.808	12.618	21.696	21.486
1	0.5825	6.758	4.547	6.994	8.497	4.325
1	0.5835	6.656	2.970	6.075	14.941	16.299
1	0.5845	4.914	10.251	6.487	27.746	24.555
1	0.5855	15.488	11.947	12.516	19.604	27.732
1	0.5865	11.383	3.325	6.399	16.818	10.585
1	0.5875	15.969	9.030	11.471	14.793	15.325
1	0.5885	6.931	7.123	7.804	14.347	14.860
1	0.5895	11.718	10.181	11.159	11.645	23.258
1	0.5905	18.561	13.781	16.019	14.271	27.224
1	0.5915	12.344	10.120	12.896	10.132	16.352
1	0.5925	6.353	9.333	6.815	7.838	14.869
1	0.5935	8.868	8.933	7.165	10.619	21.532
1	0.5945	17.078	15.820	17.118	17.341	24.397
1	0.5955	11.703	7.001	6.369	14.396	15.061
1	0.5965	25.695	14.074	25.124	9.493	10.542

1	0.5975	19.535	12.479	12.789	9.354	18.752
1	0.5985	23.671	25.415	24.898	11.709	17.721
1	0.5995	16.891	19.460	19.656	11.305	17.952
1	0.6005	25.878	18.750	16.624	15.783	23.720
1	0.6015	9.452	8.198	7.299	12.877	16.660
1	0.6025	16.943	16.158	14.132	9.776	14.564
1	0.6035	19.977	19.082	20.231	14.820	17.722
1	0.6045	20.789	22.762	19.935	17.699	12.715
1	0.6055	19.112	22.491	21.492	14.638	14.116
1	0.6065	23.583	28.786	23.228	9.906	9.699
1	0.6075	14.936	19.278	16.998	8.764	10.545
1	0.6085	23.255	21.236	20.552	6.276	27.121
1	0.6095	14.725	16.363	14.585	5.944	21.585
1	0.6105	21.242	16.269	16.383	7.675	16.745
1	0.6115	20.176	14.850	18.978	6.664	13.675
1	0.6125	22.775	22.213	15.995	13.445	25.061
1	0.6135	19.306	22.206	19.220	15.496	29.400
1	0.6145	20.142	25.625	18.892	8.685	30.591
1	0.6155	23.398	27.195	21.009	7.596	29.675
1	0.6165	17.344	18.938	14.269	11.923	17.899
1	0.6175	12.662	14.677	13.012	13.953	20.908
1	0.6185	25.271	24.934	20.527	18.555	14.388
1	0.6195	26.122	17.257	19.679	11.663	19.765
1	0.6205	30.162	25.324	29.094	11.180	18.806
1	0.6215	29.522	17.144	23.233	9.573	20.436
1	0.6225	16.002	11.832	13.535	9.862	21.960
1	0.6235	26.645	16.401	23.747	17.951	22.191
1	0.6245	23.009	23.888	22.422	13.559	19.213
1	0.6255	23.133	18.613	17.493	14.250	18.552
1	0.6265	17.982	8.878	10.204	8.391	17.242
1	0.6275	21.286	13.939	17.530	11.301	16.237
1	0.6285	14.409	13.081	17.108	9.663	21.856
1	0.6295	20.727	17.565	21.122	11.541	18.528
1	0.6305	25.428	16.176	21.243	7.121	18.097
1	0.6315	25.445	16.168	21.235	7.119	18.091
1	0.6325	8.733	6.947	5.761	8.824	15.795
1	0.6335	9.484	5.195	4.220	4.158	13.330
1	0.6345	8.661	6.533	2.859	4.369	11.035
1	0.6355	3.070	3.726	1.371	4.996	6.693
1	0.6365	23.771	17.273	23.484	7.455	20.244
1	0.6375	18.827	20.420	20.079	12.740	22.763
1	0.6385	18.844	20.451	20.103	12.759	22.823
1	0.6395	24.594	18.278	17.191	13.267	20.334
1	0.6405	16.040	12.984	15.513	11.777	24.888
1	0.6415	23.220	13.814	21.384	14.337	21.306
1	0.6425	12.691	8.225	7.649	5.822	13.782
1	0.6435	15.156	17.214	13.358	16.297	15.243
1	0.6445	2.745	2.882	2.503	4.152	14.779
1	0.6455	15.011	8.911	13.001	16.319	14.511
1	0.6465	9.397	8.164	9.095	6.963	18.888

1	0.6475	10.278	7.626	9.386	7.471	17.801
1	0.6485	9.311	3.562	7.120	3.001	13.626
1	0.6495	6.512	4.835	8.434	3.727	20.022
1	0.6505	21.586	6.113	14.249	10.349	28.529
1	0.6515	16.488	8.593	10.562	7.134	20.130
1	0.6525	11.744	12.772	14.198	15.624	19.510
1	0.6535	16.100	6.751	17.427	6.046	18.166
1	0.6545	21.661	8.345	15.327	7.929	17.386
1	0.6555	20.339	17.710	23.475	19.648	31.239
1	0.6565	26.075	16.340	14.823	14.619	25.138
1	0.6575	25.995	16.454	14.576	14.606	25.237
1	0.6585	19.378	14.208	12.018	19.010	30.113
1	0.6595	18.980	8.814	15.881	13.683	29.409
1	0.6605	12.049	13.557	10.144	15.825	25.634
1	0.6615	20.136	11.214	16.123	14.266	22.668
1	0.6625	21.243	12.575	9.302	11.226	27.395
1	0.6635	15.142	10.324	7.380	16.412	22.311
1	0.6645	18.000	14.487	20.077	12.629	19.249
1	0.6655	11.074	12.441	9.650	9.344	19.922
1	0.6665	13.540	16.330	16.291	9.194	26.823
1	0.6675	20.652	8.887	22.681	14.179	16.668
1	0.6685	26.363	9.432	19.720	10.725	21.059
1	0.6695	25.262	14.847	24.967	7.203	11.721
1	0.6705	17.331	11.553	20.168	14.666	22.285
1	0.6715	13.780	10.536	10.126	18.320	29.611
1	0.6725	9.841	12.347	13.760	15.623	25.796
1	0.6735	16.992	13.051	17.693	9.809	31.045
1	0.6745	16.255	14.836	18.885	14.782	26.598
1	0.6755	22.229	14.026	19.994	10.712	22.594
1	0.6765	6.230	4.805	11.983	5.228	14.832
1	0.6775	14.906	17.982	22.433	21.190	18.453
1	0.6785	20.238	19.866	19.522	13.208	18.771
1	0.6795	25.449	13.736	22.232	12.865	21.053
1	0.6805	17.765	11.361	22.291	12.613	12.276
1	0.6815	13.238	13.244	14.013	11.415	17.433
1	0.6825	6.309	6.078	4.693	8.457	18.144
1	0.6835	16.644	9.593	12.656	13.089	18.018
1	0.6845	17.582	8.356	20.167	7.791	10.565
1	0.6855	11.430	10.693	13.830	7.908	15.486
1	0.6865	11.220	4.412	11.539	10.432	17.999
1	0.6875	9.805	6.641	11.333	12.493	6.809
1	0.6885	15.353	9.118	16.752	2.403	9.629
1	0.6895	12.027	7.941	12.523	10.310	10.788
1	0.6905	11.917	9.412	11.568	5.540	10.625
1	0.6915	20.809	8.896	20.928	10.036	17.127
1	0.6925	21.052	10.238	17.336	8.631	9.988
1	0.6935	7.037	0.470	2.514	3.248	1.009
1	0.6945	7.380	3.102	7.032	8.052	10.675
1	0.6955	16.373	6.341	16.721	18.083	15.081
1	0.6965	11.034	10.451	18.038	8.268	15.559

1	0.6975	7.903	8.211	11.296	7.030	3.024
1	0.6985	13.890	6.693	6.020	7.097	6.812
1	0.6995	19.206	13.790	17.650	5.876	11.308
1	0.7005	14.681	7.914	11.670	10.352	19.411
1	0.7015	13.651	5.975	10.488	9.001	10.258
1	0.7025	6.875	1.282	2.932	4.767	4.655
1	0.7035	14.831	6.679	11.942	9.549	16.992
1	0.7045	14.890	11.463	17.419	13.122	19.214
1	0.7055	23.971	18.819	28.777	10.377	24.601
1	0.7065	3.136	7.178	10.564	7.199	7.022
1	0.7075	7.877	5.453	13.169	12.947	8.149
1	0.7085	8.819	5.286	14.485	10.160	7.874
1	0.7095	21.264	20.740	22.759	23.319	16.900
1	0.7105	12.147	13.243	13.525	16.155	23.236
1	0.7115	14.181	9.437	14.200	11.261	14.907
1	0.7125	12.576	15.125	14.452	9.936	19.242
1	0.7135	26.572	25.132	26.777	22.615	24.292
1	0.7145	21.650	19.869	25.246	17.699	17.441
1	0.7155	17.269	14.690	19.694	14.269	14.804
1	0.7165	23.630	17.127	19.325	12.591	14.882
1	0.7175	13.178	8.706	15.389	15.390	13.292
1	0.7185	13.301	7.957	12.834	14.540	15.927
1	0.7195	9.114	11.730	9.660	14.134	13.634
1	0.7205	15.818	9.992	13.859	16.882	9.191
1	0.7215	12.483	7.921	10.312	16.336	13.622
1	0.7225	24.189	11.644	20.304	18.256	14.238
1	0.7235	16.251	11.484	17.317	18.296	8.083
1	0.7245	9.322	7.748	10.092	13.556	14.427
1	0.7255	12.955	10.886	18.968	11.648	11.872
1	0.7265	20.872	22.522	16.759	20.542	11.173
1	0.7275	17.541	15.285	14.909	22.793	10.940
1	0.7285	18.503	13.473	14.871	6.333	16.769
1	0.7295	18.551	13.461	14.907	6.287	16.702
1	0.7305	18.930	13.594	9.493	12.517	13.183
1	0.7315	16.341	12.296	12.612	9.546	12.694
1	0.7325	17.743	12.336	16.476	10.572	11.126
1	0.7335	17.499	19.645	23.904	17.952	10.889
1	0.7345	13.278	15.626	15.209	22.188	18.102
1	0.7355	18.912	14.606	18.691	10.053	14.341
1	0.7365	42.173	20.783	28.970	9.390	10.720
1	0.7375	17.278	14.421	18.214	13.339	5.913
1	0.7385	13.006	11.766	12.533	13.107	7.266
1	0.7395	12.414	10.240	10.066	13.477	3.725
1	0.7405	15.997	11.115	12.014	12.635	5.321
1	0.7415	21.814	16.757	20.824	25.480	18.644
1	0.7425	22.796	13.057	16.656	22.646	12.756
1	0.7435	16.494	12.672	14.004	21.492	6.389
1	0.7445	12.706	11.891	12.307	24.258	11.835
1	0.7455	12.263	12.349	9.040	15.325	5.180
1	0.7465	13.557	10.427	14.637	11.514	11.598

1	0.7475	18.225	16.538	14.869	26.121	23.240
1	0.7485	16.432	17.229	12.819	25.397	17.685
1	0.7495	10.135	7.582	7.907	18.672	8.494
1	0.7505	15.044	10.043	14.526	4.727	4.891
1	0.7515	15.460	7.785	9.714	10.278	12.169
1	0.7525	18.445	10.261	14.513	21.015	17.209
1	0.7535	12.732	5.455	5.985	18.272	20.067
1	0.7545	11.378	10.855	8.446	25.212	20.820
1	0.7555	11.368	10.271	9.942	17.858	18.660
1	0.7565	10.809	11.528	9.553	23.249	10.617
1	0.7575	13.807	7.477	8.608	21.980	16.254
1	0.7585	9.884	8.489	4.349	20.072	27.048
1	0.7595	7.748	9.186	9.363	20.165	9.407
1	0.7605	9.746	8.124	7.062	17.917	13.699
1	0.7615	6.296	7.927	7.084	16.609	16.005
1	0.7625	8.408	10.020	12.628	16.585	11.459
1	0.7635	5.245	8.468	6.107	26.081	12.862
1	0.7645	6.744	9.740	7.161	19.975	23.856
1	0.7655	10.347	5.059	6.867	17.239	13.175
1	0.7665	15.582	10.346	8.112	10.219	17.252
1	0.7675	9.805	12.382	14.267	13.718	21.882
1	0.7685	25.860	19.711	23.685	23.224	16.695
1	0.7695	17.270	12.575	18.512	23.778	16.555
1	0.7705	22.488	18.407	22.412	23.101	16.780
1	0.7715	11.266	12.488	10.114	12.861	13.521
1	0.7725	12.214	7.457	7.772	21.889	20.277
1	0.7735	9.598	5.358	8.513	12.706	14.621
1	0.7745	14.337	7.227	12.346	13.348	11.204
1	0.7755	20.372	15.292	13.070	18.797	15.162
1	0.7765	24.469	14.263	21.829	18.070	15.119
1	0.7775	12.813	6.862	7.809	12.777	16.611
1	0.7785	21.268	15.603	21.438	15.741	21.915
1	0.7795	27.970	15.128	18.243	18.808	18.576
1	0.7805	18.697	5.386	17.564	13.828	7.175
1	0.7815	16.992	5.936	13.372	14.189	13.199
1	0.7825	22.057	6.055	17.183	17.580	17.079
1	0.7835	11.933	7.011	11.401	5.896	14.061
1	0.7845	15.551	6.712	11.491	7.283	20.450
1	0.7855	11.056	7.831	7.306	10.374	25.654
1	0.7865	7.390	7.808	8.006	17.189	16.683
1	0.7875	12.908	9.029	10.268	10.850	20.943
1	0.7885	19.192	8.892	16.255	17.471	11.671
1	0.7895	10.642	9.822	13.862	16.031	11.210
1	0.7905	13.558	11.048	12.982	14.347	13.792
1	0.7915	28.577	10.891	17.103	17.241	15.815
1	0.7925	20.509	12.002	17.512	17.040	19.549
1	0.7935	13.557	8.885	9.486	15.042	25.376
1	0.7945	9.388	3.144	5.491	14.193	23.036
1	0.7955	12.998	14.326	14.216	16.478	14.199
1	0.7965	18.543	14.878	14.932	25.691	15.657

1	0.7975	20.937	14.442	10.056	24.811	14.617
1	0.7985	18.903	8.268	12.579	19.731	22.409
1	0.7995	21.477	7.790	9.883	7.257	12.336
1	0.8005	14.449	6.499	11.176	11.615	15.636
1	0.8015	18.213	12.220	13.258	12.848	12.480
1	0.8025	9.406	4.124	11.402	8.445	10.469
1	0.8035	2.093	3.169	4.253	5.413	5.493
1	0.8045	6.882	3.116	4.620	18.001	20.401
1	0.8055	11.599	8.205	7.249	13.730	8.594
1	0.8065	9.415	2.003	5.576	10.301	10.201
1	0.8075	13.033	6.305	6.502	7.529	8.920
1	0.8085	7.663	4.675	3.655	10.876	9.138
1	0.8095	13.760	9.055	10.023	14.743	10.783
1	0.8105	19.297	14.561	12.964	21.643	18.368
1	0.8115	14.414	7.619	7.715	14.577	19.381
1	0.8125	16.333	9.147	14.050	9.985	13.667
1	0.8135	15.638	10.520	15.078	9.336	10.223
1	0.8145	16.408	5.989	7.651	18.558	18.822
1	0.8155	12.193	10.824	10.786	15.400	11.648
1	0.8165	1.870	3.475	1.288	3.207	5.229
1	0.8175	13.703	9.191	10.892	6.645	6.039
1	0.8185	16.520	13.310	16.561	10.364	9.376
1	0.8195	18.927	18.090	20.361	14.253	5.899
1	0.8205	14.593	10.091	10.523	15.906	13.635
1	0.8215	13.294	8.676	8.069	18.192	12.314
1	0.8225	12.918	10.351	6.935	16.440	22.585
1	0.8235	15.989	8.763	9.023	16.408	21.592
1	0.8245	7.167	5.967	2.921	10.595	4.583
1	0.8255	15.552	6.149	9.623	12.639	7.739
1	0.8265	17.490	9.743	11.268	7.241	8.761
1	0.8275	11.285	9.659	8.424	13.618	12.089
1	0.8285	14.971	7.757	11.183	19.084	18.660
1	0.8295	21.404	10.241	18.448	15.955	11.195
1	0.8305	25.619	10.933	16.844	13.276	17.293
1	0.8315	13.984	6.193	9.272	13.203	17.603
1	0.8325	19.505	10.367	15.674	18.699	14.162
1	0.8335	16.634	6.840	12.797	8.032	3.560
1	0.8345	23.003	6.346	11.984	11.193	11.822
1	0.8355	10.457	8.345	9.945	9.083	10.545
1	0.8365	18.840	9.650	10.864	11.573	12.674
1	0.8375	13.371	7.168	8.432	6.634	11.017
1	0.8385	7.607	8.166	5.696	17.466	11.663
1	0.8395	12.746	12.340	9.813	19.496	12.970
1	0.8405	11.817	13.553	6.253	8.557	16.744
1	0.8415	24.544	17.887	13.319	18.233	22.002
1	0.8425	21.267	17.829	16.746	16.867	21.481
1	0.8435	21.139	15.624	13.629	15.528	23.590
1	0.8445	4.630	3.982	1.701	17.085	8.412
1	0.8455	11.059	4.792	4.810	17.844	19.014
1	0.8465	18.174	8.909	11.620	27.138	24.423

1	0.8475	12.916	6.022	10.042	1.444	12.679
1	0.8485	6.904	2.931	6.029	0.193	1.966
1	0.8495	9.796	4.779	6.370	7.502	6.742
1	0.8505	9.571	3.867	6.200	5.596	6.492
1	0.8515	10.352	4.464	6.286	10.465	11.671
1	0.8525	10.289	4.401	6.600	13.257	15.412
1	0.8535	17.228	5.257	14.728	19.242	15.717
1	0.8545	21.873	6.448	15.942	30.852	13.960
1	0.8555	8.716	6.510	4.368	23.313	14.265
1	0.8565	12.974	7.356	7.472	7.878	15.849
1	0.8575	13.880	9.329	8.660	9.838	7.661
1	0.8585	14.176	8.703	13.610	11.129	11.342
1	0.8595	13.012	8.983	11.337	15.270	27.433
1	0.8605	11.425	11.265	15.464	15.853	19.025
1	0.8615	16.433	10.141	10.528	12.157	14.754
1	0.8625	8.917	8.127	11.002	6.463	2.905
1	0.8635	22.657	11.925	16.809	22.278	25.041
1	0.8645	18.948	7.800	12.979	14.795	16.070
1	0.8655	23.658	7.484	12.411	12.112	13.196
1	0.8665	23.032	8.796	10.816	16.287	12.226
1	0.8675	25.955	10.250	15.629	13.329	17.965
1	0.8685	21.530	10.729	12.840	17.487	13.516
1	0.8695	22.468	13.961	12.436	20.038	14.585
1	0.8705	30.099	7.774	19.775	17.778	20.135
1	0.8715	21.497	13.175	17.333	10.804	16.127
1	0.8725	11.875	6.048	10.199	9.514	8.418
1	0.8735	13.445	7.073	11.284	15.896	18.646
1	0.8745	23.441	13.029	15.078	10.889	14.989
1	0.8755	11.447	3.434	6.678	17.871	13.165
1	0.8765	10.957	10.590	10.368	19.718	9.369
1	0.8775	27.539	15.100	18.211	12.582	9.514
1	0.8785	18.898	8.484	12.571	20.387	11.044
1	0.8795	11.402	7.321	11.905	21.184	14.306
1	0.8805	11.268	6.408	11.505	17.539	14.673
1	0.8815	12.419	6.246	9.145	22.492	29.368
1	0.8825	12.135	5.352	11.845	19.971	23.820
1	0.8835	12.802	8.481	12.950	16.845	12.666
1	0.8845	12.828	15.557	9.691	23.797	19.506
1	0.8855	8.643	9.879	6.076	23.973	15.197
1	0.8865	14.309	14.559	8.228	20.536	12.824
1	0.8875	18.259	16.337	14.233	31.990	16.580
1	0.8885	10.404	10.399	6.834	26.060	23.121
1	0.8895	15.983	8.073	7.968	10.693	4.614
1	0.8905	7.743	7.147	6.636	15.913	8.446
1	0.8915	8.360	5.352	6.659	13.712	11.391
1	0.8925	14.062	3.613	9.048	22.210	24.659
1	0.8935	16.627	7.570	6.815	16.662	25.007
1	0.8945	22.294	13.057	16.073	27.917	13.411
1	0.8955	12.482	8.694	10.428	16.599	15.254
1	0.8965	17.440	9.088	12.660	24.257	19.896

1	0.8975	5.814	9.151	5.122	20.334	18.175
1	0.8985	16.049	10.777	9.628	11.638	23.129
1	0.8995	22.881	18.928	21.439	23.483	16.634
1	0.9005	15.284	18.820	11.578	28.375	13.700
1	0.9015	28.813	28.100	28.326	21.239	11.085
1	0.9025	16.850	8.056	12.332	17.557	19.639
1	0.9035	18.375	17.827	16.213	25.438	15.838
1	0.9045	15.303	11.988	14.271	14.137	16.312
1	0.9055	20.005	11.903	12.113	15.113	15.016
1	0.9065	26.482	13.805	23.458	14.794	14.996
1	0.9075	21.139	5.591	20.492	15.812	8.416
1	0.9085	17.123	5.660	20.816	10.625	11.571
1	0.9095	13.709	6.208	10.890	18.070	16.524
1	0.9105	24.383	10.281	15.831	23.919	15.334
1	0.9115	12.908	7.364	10.089	11.936	19.996
1	0.9125	30.724	18.184	25.533	19.113	13.468
1	0.9135	15.292	13.296	17.078	15.795	19.860
1	0.9145	18.630	11.409	20.291	9.911	12.307
1	0.9155	15.908	9.954	10.502	14.340	19.527
1	0.9165	23.624	14.627	19.510	25.286	20.219
1	0.9175	22.369	8.506	13.735	12.434	17.428
1	0.9185	10.457	4.896	6.330	16.193	19.960
1	0.9195	13.059	5.804	9.616	15.662	20.625
1	0.9205	13.476	9.712	10.222	10.903	14.458
1	0.9215	13.111	7.570	11.518	13.556	18.206
1	0.9225	22.194	18.490	17.627	13.024	17.297
1	0.9235	15.697	7.451	13.003	13.642	12.813
1	0.9245	16.744	11.104	14.386	14.994	16.425
1	0.9255	31.397	25.233	25.717	15.877	17.642
1	0.9265	13.853	10.154	7.837	13.642	21.364
1	0.9275	16.184	10.942	11.326	12.448	16.311
1	0.9285	14.607	9.633	9.499	11.963	17.576
1	0.9295	20.027	7.873	15.654	13.385	15.643
1	0.9305	22.907	9.137	21.746	15.348	23.535
1	0.9315	22.069	8.070	25.322	12.987	13.054
1	0.9325	12.078	11.083	10.806	15.912	7.044
1	0.9335	19.795	17.178	12.545	15.848	16.714
1	0.9345	6.060	4.299	5.021	12.023	9.093
1	0.9355	13.526	8.865	10.953	10.631	7.897
1	0.9365	15.461	7.392	11.088	8.003	6.831
1	0.9375	20.039	13.517	16.519	8.350	9.694
1	0.9385	28.851	15.614	19.581	6.377	14.381
1	0.9395	22.214	15.052	17.459	9.906	19.533
1	0.9405	31.524	14.988	20.305	12.198	23.255
1	0.9415	6.955	3.975	7.043	10.391	19.566
1	0.9425	12.218	8.689	9.248	10.850	17.343
1	0.9435	16.860	12.161	15.587	10.936	11.036
1	0.9445	11.888	6.248	5.547	13.021	6.899
1	0.9455	21.894	9.330	23.848	14.719	6.288
1	0.9465	36.359	12.510	25.180	16.987	13.608

1	0.9475	16.560	8.869	9.446	13.910	15.778
1	0.9485	14.978	8.800	11.050	9.772	14.384
1	0.9495	19.643	8.338	13.224	9.756	13.285
1	0.9505	13.003	9.705	10.159	8.837	8.929
1	0.9515	11.816	20.158	17.279	17.380	10.074
1	0.9525	11.740	20.187	17.265	17.501	10.095
1	0.9535	9.226	4.430	8.925	6.893	4.637
1	0.9545	18.625	10.240	14.592	16.503	9.996
1	0.9555	18.688	11.924	18.395	11.631	11.382
1	0.9565	19.182	16.500	22.668	15.302	16.890
1	0.9575	7.327	14.850	9.074	17.417	17.032
1	0.9585	12.160	8.499	9.095	14.497	20.819
1	0.9595	24.073	22.133	25.030	21.058	20.288
1	0.9605	13.880	19.226	12.285	29.245	21.436
1	0.9615	19.107	25.233	21.933	26.055	18.499
1	0.9625	10.451	14.411	10.452	21.993	11.420
1	0.9635	15.163	13.727	13.454	15.212	21.016
1	0.9645	14.245	9.552	10.115	16.826	10.779
1	0.9655	18.999	11.433	14.496	14.227	17.219
1	0.9665	14.051	7.039	10.374	24.491	14.125
1	0.9675	9.124	5.780	5.577	14.527	9.710
1	0.9685	5.715	10.244	5.559	23.011	18.945
1	0.9695	9.945	4.471	5.632	14.429	21.559
1	0.9705	8.124	5.044	7.725	16.849	23.560
1	0.9715	7.343	10.689	9.284	16.609	26.204
1	0.9725	19.439	20.260	19.843	18.487	9.019
1	0.9735	19.828	21.516	14.516	16.419	28.815
1	0.9745	15.874	13.406	20.810	19.297	26.379
1	0.9755	15.788	13.389	20.735	19.227	26.354
1	0.9765	13.695	15.095	24.553	19.263	16.021
1	0.9775	19.244	19.321	17.989	18.076	12.964
1	0.9785	20.093	12.178	13.454	9.042	10.739
1	0.9795	5.635	9.068	6.470	20.633	9.945
1	0.9805	17.763	18.936	23.158	20.182	19.212
1	0.9815	15.414	9.693	12.440	9.539	10.968
1	0.9825	14.707	8.340	8.835	10.193	12.041
1	0.9835	15.079	11.256	7.244	8.750	12.304
1	0.9845	19.589	11.016	12.737	7.954	10.244
1	0.9855	12.368	6.560	7.761	8.696	10.919
1	0.9865	16.487	12.635	12.569	9.672	14.429
1	0.9875	21.843	14.570	14.688	17.858	19.734
1	0.9885	19.715	16.775	18.874	15.629	14.117
1	0.9895	17.322	15.040	15.378	22.027	12.140
1	0.9905	16.446	9.592	12.919	12.843	16.243
1	0.9915	13.555	10.745	11.777	8.425	10.510
1	0.9925	13.835	13.003	11.671	17.695	10.062
1	0.9935	8.990	7.089	6.347	14.059	14.674
1	0.9945	10.885	9.058	8.878	21.601	20.230
1	0.9955	12.796	12.883	8.964	13.059	18.448
1	0.9965	11.619	9.471	7.992	15.660	21.740

1	0.9975	11.612	9.468	8.047	15.928	21.789
1	0.9985	16.420	12.576	10.684	14.062	20.538
1	0.9995	19.311	10.787	14.626	17.515	19.407
1	1.0005	19.967	13.797	15.282	13.407	22.869
1	1.0015	8.297	11.854	11.064	19.425	23.652
1	1.0025	18.925	15.820	19.349	11.269	11.541
1	1.0035	13.915	11.510	9.112	12.110	23.731
1	1.0045	11.995	7.709	8.989	16.828	22.356
1	1.0055	8.266	9.016	9.247	10.665	10.731
1	1.0065	15.162	15.354	24.041	14.801	14.304
1	1.0075	21.652	18.162	20.699	8.778	24.158
1	1.0085	3.999	4.131	3.601	8.580	5.993
1	1.0095	7.086	5.071	4.151	12.306	12.200
1	1.0105	17.112	12.935	11.837	10.816	17.340
1	1.0115	24.304	15.779	20.348	21.876	19.674
1	1.0125	18.043	16.342	12.508	17.335	14.676
1	1.0135	22.163	13.573	17.726	19.735	11.453
1	1.0145	15.663	5.336	11.460	22.504	16.034
1	1.0155	22.044	14.748	16.548	31.089	17.566
1	1.0165	17.385	17.473	15.804	23.890	13.173
1	1.0175	14.860	7.945	10.296	13.729	11.270
1	1.0185	11.629	7.566	9.714	17.864	6.006
1	1.0195	6.215	4.347	4.184	10.878	9.712
1	1.0205	8.989	9.592	7.140	12.293	3.905
1	1.0215	11.810	7.450	9.794	28.036	12.952
1	1.0225	11.809	7.450	9.794	28.022	12.948
1	1.0235	7.147	7.466	7.387	30.731	9.102
1	1.0245	10.201	7.774	7.118	11.864	13.368
1	1.0255	23.639	22.542	21.728	23.725	14.195
1	1.0265	17.625	5.920	16.920	23.875	9.172
1	1.0275	11.344	4.385	11.212	19.752	9.036
1	1.0285	10.350	9.473	12.051	22.607	9.498
1	1.0295	9.601	2.579	8.476	12.711	11.359
1	1.0305	12.704	7.733	10.452	6.890	18.813
1	1.0315	13.084	12.659	10.949	17.677	10.507
1	1.0325	17.099	7.325	9.642	11.968	11.948
1	1.0335	21.930	10.938	14.908	24.447	12.620
1	1.0345	8.183	6.012	5.411	12.091	6.449
1	1.0355	17.662	9.435	17.226	26.156	10.639
1	1.0365	17.211	8.282	15.070	26.317	17.344
1	1.0375	7.571	6.365	8.249	16.336	16.069
1	1.0385	15.220	16.609	10.455	16.864	11.617
1	1.0395	18.445	14.961	17.346	12.444	15.334
1	1.0405	13.651	9.723	10.851	9.984	16.757
1	1.0415	13.078	5.454	10.664	14.262	18.913
1	1.0425	13.604	9.975	15.618	25.722	10.122
1	1.0435	11.472	4.909	10.564	24.231	13.110
1	1.0445	19.133	7.372	12.620	24.640	10.956
1	1.0455	19.827	11.541	10.199	13.193	18.607
1	1.0465	14.522	10.758	9.665	11.611	11.916

1	1.0475	23.636	11.082	12.959	18.973	21.294
1	1.0485	7.032	3.084	4.824	16.531	6.055
1	1.0495	9.757	5.000	6.927	16.166	9.310
1	1.0505	3.515	1.956	6.058	6.496	4.026
1	1.0515	11.327	6.089	15.076	14.541	9.475
1	1.0525	9.358	9.799	11.052	14.426	11.873
1	1.0535	17.987	10.291	13.111	14.884	6.860
1	1.0545	14.241	9.082	11.455	25.457	11.372
1	1.0555	18.858	8.472	12.713	15.771	22.712
1	1.0565	9.079	10.371	9.076	7.414	6.413
1	1.0575	8.086	6.831	7.925	11.263	10.501
1	1.0585	13.445	17.252	10.911	24.263	12.210
1	1.0595	14.498	9.679	12.593	21.206	16.879
1	1.0605	21.364	15.259	16.842	29.846	19.482
1	1.0615	20.399	18.077	18.395	16.085	11.394
1	1.0625	9.016	10.020	8.554	11.457	8.868
1	1.0635	16.841	12.447	10.272	25.603	19.983
1	1.0645	22.556	8.941	14.031	18.221	16.807
1	1.0655	13.889	6.907	9.880	32.049	9.487
1	1.0665	11.056	9.928	8.057	25.612	11.677
1	1.0675	16.189	13.679	17.819	22.524	14.414
1	1.0685	20.606	11.101	12.549	34.180	14.118
1	1.0695	20.816	9.366	16.106	12.833	8.907
1	1.0705	16.559	10.408	11.951	5.374	14.692
1	1.0715	10.034	7.235	6.413	15.502	10.365
1	1.0725	2.196	1.150	2.415	6.702	3.884
1	1.0735	14.856	6.827	10.503	19.035	10.861
1	1.0745	16.593	8.555	11.258	22.111	16.864
1	1.0755	18.742	4.440	8.963	29.666	16.045
1	1.0765	17.878	10.336	15.439	28.187	16.945
1	1.0775	20.503	12.985	16.977	15.560	11.732
1	1.0785	12.567	12.785	14.750	22.374	16.640
1	1.0795	5.659	10.263	8.395	16.422	17.839
1	1.0805	10.129	13.217	14.837	12.523	14.689
1	1.0815	9.703	9.829	11.107	18.158	24.142
1	1.0825	15.018	12.274	14.298	26.693	20.730
1	1.0835	26.903	14.455	19.307	13.721	18.564
1	1.0845	12.336	9.404	9.239	19.133	13.657
1	1.0855	23.309	18.610	27.376	23.501	16.952
1	1.0865	12.037	7.131	9.731	12.545	6.893
1	1.0875	10.867	6.713	5.798	21.141	5.880
1	1.0885	12.290	4.232	8.395	35.545	3.741
1	1.0895	14.340	8.268	10.473	15.656	12.446
1	1.0905	14.129	7.609	12.455	15.899	10.793
1	1.0915	15.446	12.420	14.286	30.035	15.855
1	1.0925	18.454	16.592	16.482	41.615	19.949
1	1.0935	9.740	10.888	11.588	13.112	22.801
1	1.0945	9.547	11.487	13.676	16.491	12.471
1	1.0955	19.371	12.630	21.043	16.275	12.686
1	1.0965	20.641	17.926	23.352	16.627	17.444

1	1.0975	13.120	16.702	12.997	27.952	21.776
1	1.0985	13.944	16.016	16.374	25.798	19.546
1	1.0995	19.425	17.269	23.124	23.329	22.039
1	1.1005	24.502	14.040	25.677	16.526	15.838
1	1.1015	22.267	14.036	20.485	14.193	9.396
1	1.1025	15.903	13.192	15.727	3.538	8.218
1	1.1035	16.471	11.181	16.097	23.904	11.681
1	1.1045	14.437	13.379	14.971	14.216	4.628
1	1.1055	14.182	15.668	15.909	20.250	22.804
1	1.1065	8.158	12.221	12.340	19.188	22.137
1	1.1075	9.618	8.838	14.102	8.719	15.681
1	1.1085	16.035	7.962	17.013	17.611	20.913
1	1.1095	15.256	10.917	16.777	10.815	16.450
1	1.1105	12.196	11.035	13.058	14.859	10.496
1	1.1115	13.164	9.598	16.124	12.361	9.468
1	1.1125	29.597	11.744	24.377	17.689	7.834
1	1.1135	22.195	18.729	22.117	21.116	16.769
1	1.1145	12.201	15.351	15.964	14.117	20.419
1	1.1155	14.485	16.406	16.539	9.888	18.339
1	1.1165	22.073	15.553	20.517	17.066	13.597
1	1.1175	7.435	6.938	6.448	17.699	11.559
1	1.1185	2.749	6.494	6.200	20.684	15.355
1	1.1195	4.325	11.464	6.260	27.110	12.730
1	1.1205	6.835	10.744	10.603	21.218	11.932
1	1.1215	11.634	9.103	11.268	25.446	15.328
1	1.1225	20.334	16.827	11.777	7.985	18.623
1	1.1235	4.126	4.965	4.105	6.134	17.942
1	1.1245	7.793	0.878	4.380	11.819	20.419
1	1.1255	5.360	1.086	5.930	7.216	7.912
1	1.1265	10.434	5.547	13.655	4.118	10.324
1	1.1275	10.993	8.796	9.207	5.596	18.698
1	1.1285	6.148	5.644	6.616	5.075	11.506
1	1.1295	6.099	5.614	6.536	5.129	11.635
1	1.1305	19.183	15.771	26.381	6.610	20.780
1	1.1315	11.666	18.558	18.168	15.736	20.698
1	1.1325	17.928	11.003	11.641	14.004	14.842
1	1.1335	16.122	13.187	13.480	16.727	19.158
1	1.1345	16.241	8.576	17.547	15.891	14.173
1	1.1355	14.182	13.228	15.445	6.367	5.924
1	1.1365	25.404	17.755	23.418	15.874	12.611
1	1.1375	15.655	12.557	15.620	12.467	14.563
1	1.1385	9.198	8.522	11.779	8.029	15.572
1	1.1395	10.253	18.481	17.983	12.778	18.005
1	1.1405	25.424	32.820	34.203	12.653	25.394
1	1.1415	20.562	8.531	20.015	18.429	7.341
1	1.1425	20.702	12.737	21.665	22.171	11.793
1	1.1435	14.777	7.168	14.021	12.990	17.119
1	1.1445	15.912	16.756	17.394	10.656	14.253
1	1.1455	13.270	10.592	10.819	10.330	12.486
1	1.1465	8.186	8.595	6.857	16.145	8.403

1	1.1475	11.764	17.817	16.792	21.220	10.092
1	1.1485	14.327	10.807	18.009	11.124	11.953
1	1.1495	7.430	9.474	9.035	22.699	12.302
1	1.1505	8.706	9.325	9.178	13.699	15.193
1	1.1515	7.500	14.087	8.950	19.370	21.896
1	1.1525	17.618	12.274	14.565	19.153	15.324
1	1.1535	15.027	13.788	15.923	9.929	18.438
1	1.1545	14.616	12.506	11.353	7.660	16.104
1	1.1555	19.769	16.886	20.245	15.416	18.970
1	1.1565	13.562	14.172	13.633	9.193	14.138
1	1.1575	13.920	14.754	20.630	9.143	7.139
1	1.1585	17.794	10.791	18.814	6.311	20.503
1	1.1595	12.841	14.938	17.248	8.798	11.537
1	1.1605	13.291	16.027	16.359	8.486	5.994
1	1.1615	10.952	9.073	11.630	13.310	19.766
1	1.1625	6.175	3.837	7.094	14.689	21.411
1	1.1635	12.112	8.024	14.392	12.430	29.480
1	1.1645	6.733	12.582	10.182	17.958	37.159
1	1.1655	3.376	6.472	9.390	7.240	26.207
1	1.1665	4.254	5.666	5.921	12.562	7.917
1	1.1675	10.947	11.354	18.078	6.328	8.741
1	1.1685	15.340	11.817	21.960	8.650	16.796
1	1.1695	16.124	9.597	18.164	8.958	10.031
1	1.1705	9.896	6.781	8.711	8.760	10.374
1	1.1715	12.771	10.741	12.765	6.723	15.405
1	1.1725	13.235	15.347	17.941	12.597	16.241
1	1.1735	16.664	10.742	18.518	17.262	24.712
1	1.1745	5.579	11.086	10.240	17.186	15.432
1	1.1755	5.025	8.462	7.263	22.162	10.603
1	1.1765	13.988	13.533	12.010	14.024	12.745
1	1.1775	21.637	16.290	20.794	6.907	16.235
1	1.1785	21.044	14.867	18.742	15.210	13.778
1	1.1795	24.799	19.659	22.204	17.556	22.833
1	1.1805	21.606	12.747	19.249	12.772	13.831
1	1.1815	15.481	16.836	15.961	13.889	16.995
1	1.1825	19.545	11.559	16.331	10.819	10.900
1	1.1835	14.659	15.578	16.203	14.580	12.138
1	1.1845	16.379	11.798	14.184	16.825	11.256
1	1.1855	9.022	5.545	10.494	3.545	8.718
1	1.1865	15.596	11.817	15.925	13.557	16.695
1	1.1875	18.944	15.366	23.723	16.135	27.091
1	1.1885	19.568	18.511	16.261	15.982	17.359
1	1.1895	13.910	13.026	12.392	11.092	19.735
1	1.1905	20.906	18.964	15.867	10.533	19.238
1	1.1915	21.831	19.290	21.949	14.356	24.231
1	1.1925	16.317	11.981	18.246	17.161	26.315
1	1.1935	29.607	17.177	22.547	17.480	24.647
1	1.1945	31.329	17.859	20.122	16.163	15.396
1	1.1955	21.815	17.321	22.599	13.348	14.511
1	1.1965	12.943	14.682	17.650	12.889	9.466

1	1.1975	15.154	10.236	14.061	12.252	11.241
1	1.1985	17.912	9.932	15.962	13.857	19.314
1	1.1995	14.960	6.584	13.627	10.897	15.317
1	1.2005	23.494	10.231	17.719	10.266	19.507
1	1.2015	15.335	14.994	20.401	25.164	23.709
1	1.2025	9.756	11.853	11.473	22.382	11.298
1	1.2035	19.238	20.994	29.097	23.644	19.941
1	1.2045	19.316	19.606	31.234	16.756	18.212
1	1.2055	20.075	18.714	22.996	23.442	25.123
1	1.2065	14.907	12.657	20.105	20.880	26.708
1	1.2075	18.697	17.991	20.801	24.461	16.580
1	1.2085	24.122	21.499	29.545	15.119	9.278
1	1.2095	27.133	21.248	27.117	11.746	21.439
1	1.2105	10.467	10.957	18.346	20.365	16.789
1	1.2115	25.222	13.185	25.162	10.955	23.640
1	1.2125	20.710	16.871	17.764	8.018	16.815
1	1.2135	23.168	22.705	16.776	16.525	29.290
1	1.2145	11.423	13.509	15.203	11.899	19.812
1	1.2155	19.137	18.166	17.925	10.736	25.108
1	1.2165	11.034	13.688	13.119	17.885	9.284
1	1.2175	17.857	17.874	18.092	16.140	13.977
1	1.2185	17.740	17.775	18.090	16.402	14.105
1	1.2195	17.774	21.422	24.324	17.349	23.810
1	1.2205	19.540	24.034	23.297	16.909	19.024
1	1.2215	21.388	18.185	18.811	21.133	21.283
1	1.2225	18.732	24.392	14.563	19.332	14.160
1	1.2235	31.987	24.636	30.210	18.496	20.608
1	1.2245	22.892	20.834	23.158	17.206	19.865
1	1.2255	10.540	17.695	11.842	25.035	19.472
1	1.2265	20.763	19.881	22.380	16.286	23.983
1	1.2275	37.680	31.351	28.764	13.436	24.014
1	1.2285	21.816	15.364	18.581	15.186	20.320
1	1.2295	32.807	15.239	24.231	14.058	24.457
1	1.2305	12.233	16.072	14.803	18.284	27.245
1	1.2315	12.072	20.631	9.982	17.470	8.039
1	1.2325	20.695	25.824	24.183	11.949	26.658
1	1.2335	22.758	31.338	27.367	15.685	16.610
1	1.2345	44.504	41.457	37.673	29.553	23.164
1	1.2355	25.230	21.219	17.520	13.614	16.359
1	1.2365	20.337	18.267	13.393	17.034	20.418
1	1.2375	6.712	13.476	6.996	17.224	13.817
1	1.2385	17.957	10.349	13.154	10.621	10.781
1	1.2395	16.299	12.837	12.172	11.050	13.264
1	1.2405	22.493	12.653	14.565	3.491	12.123
1	1.2415	8.648	4.295	8.547	12.617	20.634
1	1.2425	16.505	17.120	11.056	24.525	10.331
1	1.2435	20.664	17.100	12.288	21.761	19.236
1	1.2445	33.047	29.602	19.006	21.330	45.477
1	1.2455	22.655	25.440	17.130	25.285	30.810
1	1.2465	28.136	14.845	20.992	31.292	27.022

1	1.2475	18.171	14.193	13.793	10.170	25.375
1	1.2485	12.954	11.075	15.416	17.078	16.969
1	1.2495	13.199	6.829	12.334	19.133	16.824
1	1.2505	6.499	6.802	7.020	25.122	25.222
1	1.2515	14.976	16.831	11.311	16.692	26.732
1	1.2525	21.562	28.428	17.759	23.744	16.144
1	1.2535	22.649	27.154	17.268	22.613	17.965
1	1.2545	40.219	38.423	26.407	12.406	21.364
1	1.2555	26.749	29.284	19.621	11.851	23.738
1	1.2565	21.414	23.476	20.508	29.469	25.911
1	1.2575	19.705	19.643	18.290	18.571	30.186
1	1.2585	23.020	23.563	15.700	17.083	20.534
1	1.2595	18.440	16.053	15.932	19.743	23.290
1	1.2605	24.876	23.764	18.579	18.679	18.879
1	1.2615	24.393	20.431	17.679	11.037	20.252
1	1.2625	16.969	12.842	14.509	14.900	17.370
1	1.2635	33.269	23.885	24.003	17.284	22.817
1	1.2645	27.104	24.564	27.404	20.859	20.855
1	1.2655	12.254	11.145	15.945	15.521	16.466
1	1.2665	7.777	10.031	4.917	31.602	8.287
1	1.2675	17.519	19.950	11.025	33.849	17.074
1	1.2685	11.444	24.349	14.726	29.767	16.374
1	1.2695	10.372	12.620	11.921	20.197	20.573
1	1.2705	25.225	21.011	19.619	27.908	28.674
1	1.2715	23.807	24.308	12.167	23.359	31.753
1	1.2725	22.032	23.310	12.293	18.188	24.125
1	1.2735	12.934	16.733	10.402	22.172	21.361
1	1.2745	14.335	17.867	13.527	15.145	13.606
1	1.2755	12.981	17.108	13.283	11.587	21.462
1	1.2765	34.688	25.825	17.685	18.068	31.594
1	1.2775	34.650	25.824	17.704	18.048	31.595
1	1.2785	11.571	12.823	9.098	18.983	38.722
1	1.2795	11.510	12.882	9.120	19.031	38.912
1	1.2805	15.953	10.233	10.128	5.838	21.935
1	1.2815	14.801	17.054	11.598	11.281	11.656
1	1.2825	9.822	11.405	9.626	15.832	9.904
1	1.2835	4.133	9.241	4.131	14.402	6.988
1	1.2845	6.152	8.578	3.214	16.708	11.492
1	1.2855	8.700	7.326	8.133	9.946	10.379
1	1.2865	15.006	19.002	16.387	13.942	5.156
1	1.2875	18.766	23.616	15.584	14.088	27.172
1	1.2885	7.292	11.064	5.383	10.966	9.215
1	1.2895	6.540	16.365	11.750	15.399	24.773
1	1.2905	15.107	16.274	19.476	22.741	13.945
1	1.2915	15.105	15.730	14.882	18.232	20.093
1	1.2925	8.247	19.047	16.908	21.427	20.737
1	1.2935	3.255	12.134	6.860	20.110	16.273
1	1.2945	6.347	14.063	11.927	21.102	18.310
1	1.2955	26.951	24.286	18.623	28.841	24.978
1	1.2965	14.126	20.551	13.852	31.638	35.456

1	1.2975	11.398	19.298	16.668	18.764	21.610
1	1.2985	21.353	18.572	21.749	7.430	18.173
1	1.2995	9.683	14.269	13.094	9.284	22.291
1	1.3005	1.520	5.402	5.234	2.577	8.043
1	1.3015	1.718	6.075	4.245	2.941	3.089
1	1.3025	2.031	3.631	2.657	2.970	2.709
1	1.3035	1.032	5.621	2.204	6.178	2.802
1	1.3045	1.069	7.077	3.295	7.268	2.941
1	1.3055	19.192	22.790	19.664	18.999	14.953
1	1.3065	15.225	23.855	19.792	23.175	16.393
1	1.3075	15.054	17.159	10.870	9.497	10.903
1	1.3085	15.998	19.824	15.069	19.154	11.724
1	1.3095	16.018	19.861	15.080	19.169	11.739
1	1.3105	14.520	18.837	17.287	26.322	22.618
1	1.3115	8.468	15.143	9.480	21.217	25.496
1	1.3125	12.470	20.825	12.065	23.744	25.925
1	1.3135	18.913	19.171	14.995	21.936	12.161
1	1.3145	19.108	21.141	16.724	9.025	12.418
1	1.3155	9.658	8.902	9.678	11.254	12.535
1	1.3165	9.257	12.072	12.339	19.332	14.838
1	1.3175	26.761	15.294	28.076	19.062	20.557
1	1.3185	11.299	17.387	14.562	10.952	22.386
1	1.3195	22.204	23.716	22.539	13.055	27.367
1	1.3205	21.416	20.658	20.331	21.867	19.092
1	1.3215	18.851	23.319	19.919	20.698	11.609
1	1.3225	14.551	12.644	14.282	10.574	15.533
1	1.3235	7.770	9.991	6.978	13.106	14.523
1	1.3245	9.275	13.090	9.371	5.237	9.292
1	1.3255	19.132	26.830	20.805	14.963	24.886
1	1.3265	11.351	7.937	10.889	4.372	21.112
1	1.3275	7.469	10.877	11.166	5.258	8.412
1	1.3285	22.260	29.196	26.824	11.170	9.651
1	1.3295	27.079	32.113	25.845	16.811	15.927
1	1.3305	14.712	15.931	14.209	9.502	5.903
1	1.3315	18.147	14.065	16.196	10.550	16.446
1	1.3325	10.248	13.095	10.299	5.757	3.742
1	1.3335	14.555	14.486	12.948	4.316	11.987
1	1.3345	5.556	10.316	7.298	10.137	13.938
1	1.3355	14.955	15.899	12.441	15.403	10.379
1	1.3365	13.249	19.307	11.089	16.170	15.432
1	1.3375	26.231	30.937	28.268	24.254	23.278
1	1.3385	13.295	10.391	16.196	14.107	12.871
1	1.3395	21.750	14.082	21.625	15.818	7.221
1	1.3405	14.521	16.162	20.070	16.074	7.875
1	1.3415	25.307	17.316	22.208	9.113	23.467
1	1.3425	23.082	27.315	30.065	12.077	12.890
1	1.3435	8.946	12.516	8.657	9.701	9.969
1	1.3445	10.921	10.051	10.447	8.782	10.741
1	1.3455	19.530	12.772	18.130	7.527	15.224
1	1.3465	20.072	14.899	18.840	10.954	20.980

1	1.3475	17.275	16.849	18.236	8.305	15.712
1	1.3485	17.871	21.336	16.878	18.728	18.581
1	1.3495	17.758	21.328	16.800	18.782	18.706
1	1.3505	17.422	13.063	19.307	4.657	13.450
1	1.3515	18.696	20.794	20.385	10.055	18.178
1	1.3525	14.249	14.880	19.524	6.910	12.205
1	1.3535	12.306	14.120	11.749	20.283	20.572
1	1.3545	27.974	27.187	30.013	5.888	14.516
1	1.3555	32.124	23.942	33.531	16.662	18.655
1	1.3565	16.666	13.704	16.934	7.896	19.649
1	1.3575	8.113	8.167	12.060	9.259	17.250
1	1.3585	13.560	14.176	11.437	16.577	13.924
1	1.3595	12.891	16.276	17.669	11.752	24.263
1	1.3605	13.316	17.345	22.166	15.706	19.734
1	1.3615	21.411	19.378	33.908	14.056	16.241
1	1.3625	13.754	6.152	8.408	11.002	23.680
1	1.3635	19.131	22.389	13.389	24.974	25.698
1	1.3645	19.114	22.327	13.351	24.991	25.737
1	1.3655	22.925	11.964	23.404	8.607	6.018
1	1.3665	21.963	16.928	26.902	9.622	9.843
1	1.3675	28.802	26.515	34.105	17.441	21.641
1	1.3685	15.573	8.698	10.936	16.822	17.228
1	1.3695	15.562	8.665	10.927	16.782	17.229
1	1.3705	21.930	16.966	20.978	11.884	16.045
1	1.3715	18.055	17.917	19.029	9.287	14.078
1	1.3725	27.296	19.321	24.450	10.353	8.007
1	1.3735	8.243	6.329	11.248	5.046	3.334
1	1.3745	26.400	14.834	27.605	13.303	11.817
1	1.3755	26.355	20.095	28.801	27.798	16.771
1	1.3765	23.547	14.913	23.738	21.631	22.048
1	1.3775	18.342	21.354	23.803	34.154	27.162
1	1.3785	23.085	17.935	27.637	19.343	18.170
1	1.3795	15.300	18.028	20.683	21.822	21.970
1	1.3805	19.986	16.212	23.485	18.284	16.265
1	1.3815	13.564	25.171	18.446	25.282	14.797
1	1.3825	17.232	14.678	20.478	8.651	16.623
1	1.3835	21.281	12.783	23.084	13.749	8.952
1	1.3845	9.774	8.028	12.282	16.145	12.678
1	1.3855	23.405	15.028	25.513	16.757	14.788
1	1.3865	26.466	25.077	34.912	15.152	10.763
1	1.3875	25.251	24.024	26.553	24.153	15.199
1	1.3885	21.484	18.741	24.916	28.675	13.738
1	1.3895	19.960	23.320	22.370	27.080	18.927
1	1.3905	22.999	15.545	24.038	11.487	24.393
1	1.3915	18.297	15.004	22.814	21.453	12.822
1	1.3925	18.212	14.910	17.837	16.454	28.852
1	1.3935	20.916	15.696	10.659	16.120	26.744
1	1.3945	19.309	16.926	14.765	24.601	8.981
1	1.3955	17.408	14.923	8.239	27.472	27.423
1	1.3965	24.109	13.117	15.274	25.921	26.304

1	1.3975	18.612	13.180	19.944	19.931	14.298
1	1.3985	26.189	15.427	21.028	28.064	17.583
1	1.3995	21.219	13.487	20.640	18.932	27.592
1	1.4005	9.211	12.778	9.878	23.014	20.053
1	1.4015	11.568	9.748	9.115	13.048	22.005
1	1.4025	11.691	16.696	11.369	21.546	22.402
1	1.4035	7.355	13.912	11.157	16.842	20.893
1	1.4045	18.885	27.107	26.131	28.983	22.602
1	1.4055	14.534	18.554	19.454	11.123	16.172
1	1.4065	11.486	10.413	13.145	13.083	16.445
1	1.4075	8.376	9.683	10.534	5.591	4.414
1	1.4085	11.258	13.255	17.649	21.091	20.604
1	1.4095	22.058	19.533	25.429	17.200	20.131
1	1.4105	15.300	18.739	19.330	16.129	22.804
1	1.4115	14.465	14.048	13.379	14.824	20.582
1	1.4125	13.793	11.318	13.101	13.782	13.539
1	1.4135	19.923	20.344	17.735	21.633	21.709
1	1.4145	12.219	11.256	14.875	8.337	18.961
1	1.4155	3.672	5.611	5.300	3.920	14.045
1	1.4165	20.393	20.790	24.489	15.961	23.077
1	1.4175	12.776	8.432	14.926	24.394	20.593
1	1.4185	16.259	16.198	17.946	22.194	19.385
1	1.4195	15.170	16.963	17.805	15.508	27.345
1	1.4205	9.532	11.993	13.534	15.070	4.107
1	1.4215	11.293	7.840	5.819	22.015	15.333
1	1.4225	6.745	8.125	6.166	30.937	15.993
1	1.4235	5.757	10.545	3.599	18.568	17.412
1	1.4245	9.006	14.098	13.046	13.954	13.209
1	1.4255	12.933	15.133	14.081	16.401	22.814
1	1.4265	7.782	11.487	5.467	3.615	14.001
1	1.4275	15.536	11.337	15.449	16.164	17.133
1	1.4285	8.950	10.756	14.266	7.210	13.317
1	1.4295	3.381	9.745	11.067	10.979	17.669
1	1.4305	13.154	13.641	18.933	10.646	21.570
1	1.4315	15.128	13.592	23.652	6.167	18.802
1	1.4325	9.456	12.470	12.603	6.584	10.288
1	1.4335	14.671	15.464	11.273	7.685	21.049
1	1.4345	16.918	18.494	16.989	12.674	32.257
1	1.4355	16.453	16.776	12.236	11.055	13.613
1	1.4365	18.832	18.789	17.135	19.022	22.679
1	1.4375	13.317	9.229	11.674	13.401	26.084
1	1.4385	13.287	9.198	11.625	13.411	26.133
1	1.4395	10.718	9.526	13.095	11.530	16.194
1	1.4405	8.732	6.536	5.278	10.285	29.294
1	1.4415	5.429	4.725	7.135	5.755	22.362
1	1.4425	7.171	4.847	3.481	9.574	13.305
1	1.4435	13.565	16.556	13.182	15.635	24.222
1	1.4445	14.261	20.274	18.053	17.885	14.680
1	1.4455	8.738	11.479	9.756	13.490	14.819
1	1.4465	16.566	13.142	15.013	14.093	19.623

1	1.4475	24.397	25.917	31.224	14.359	15.314
1	1.4485	17.139	26.414	22.559	17.956	11.336
1	1.4495	16.204	21.771	18.241	15.768	11.657
1	1.4505	13.048	8.054	10.677	8.533	5.668
1	1.4515	18.345	16.005	18.396	15.682	12.530
1	1.4525	11.443	13.627	14.837	17.811	27.100
1	1.4535	22.836	17.745	24.826	13.682	14.883
1	1.4545	15.594	18.251	13.092	25.074	22.498
1	1.4555	23.620	26.961	28.024	16.116	12.488
1	1.4565	14.399	17.635	14.626	15.406	16.044
1	1.4575	6.834	15.836	12.854	16.576	19.960
1	1.4585	11.752	22.137	11.448	21.887	22.313
1	1.4595	16.670	17.055	16.119	24.218	21.089
1	1.4605	9.234	4.954	9.074	3.278	5.303
1	1.4615	9.459	8.739	8.906	4.092	6.862
1	1.4625	17.743	18.359	16.684	21.461	16.772
1	1.4635	14.950	16.074	15.092	12.141	16.280
1	1.4645	24.311	19.567	22.863	15.728	16.267
1	1.4655	15.688	17.300	17.273	14.596	17.551
1	1.4665	8.464	15.681	13.206	9.220	6.259
1	1.4675	10.570	8.664	7.938	9.902	16.173
1	1.4685	12.974	15.841	12.919	4.018	7.008
1	1.4695	12.175	6.603	4.651	3.516	7.020
1	1.4705	14.444	7.807	9.542	18.364	14.076
1	1.4715	17.662	12.105	10.298	9.959	10.301
1	1.4725	20.640	16.966	15.707	10.436	13.725
1	1.4735	14.269	10.144	13.037	15.980	29.205
1	1.4745	24.493	19.101	14.475	20.943	24.529
1	1.4755	18.246	17.451	11.976	24.397	16.077
1	1.4765	17.604	19.341	15.181	19.898	20.026
1	1.4775	12.224	15.512	12.599	21.169	19.727
1	1.4785	9.187	7.007	3.754	16.255	9.182
1	1.4795	11.947	14.204	11.151	20.506	9.258
1	1.4805	19.202	18.905	16.084	23.338	23.919
1	1.4815	6.944	10.941	6.590	17.315	12.413
1	1.4825	6.888	11.202	6.691	17.412	12.293
1	1.4835	7.540	13.108	8.461	16.368	28.071
1	1.4845	20.379	23.714	18.659	11.569	22.404
1	1.4855	7.664	12.486	7.720	15.994	10.253
1	1.4865	23.753	15.937	20.126	29.450	19.493
1	1.4875	16.076	17.241	19.664	16.246	18.581
1	1.4885	7.109	17.267	14.815	18.938	10.211
1	1.4895	13.177	21.926	15.093	23.334	5.561
1	1.4905	15.305	16.797	13.906	22.482	13.448
1	1.4915	13.579	18.183	9.137	17.480	19.352
1	1.4925	16.625	15.101	11.696	23.604	12.388
1	1.4935	16.119	15.108	11.728	23.733	12.708
1	1.4945	10.307	13.088	12.404	18.290	24.259
1	1.4955	15.420	20.081	15.278	22.177	23.222
1	1.4965	15.010	18.322	20.233	13.563	20.290

1	1.4975	24.512	22.165	19.026	23.599	17.842
1	1.4985	10.774	25.028	14.801	28.141	19.633
1	1.4995	21.004	20.709	13.036	18.847	15.065
1	1.5005	26.655	30.281	30.291	16.501	20.753
1	1.5015	22.569	22.296	18.962	14.377	25.465
1	1.5025	8.322	7.537	5.865	8.665	13.842
1	1.5035	15.620	10.242	8.121	10.269	10.303
1	1.5045	3.533	6.909	5.617	14.275	16.389
1	1.5055	14.593	10.529	10.955	19.051	20.529
1	1.5065	9.473	7.700	11.023	7.516	15.487
1	1.5075	16.031	17.182	18.621	14.204	12.187
1	1.5085	15.220	19.178	20.230	20.783	7.807
1	1.5095	11.433	16.062	11.422	15.738	8.303
1	1.5105	14.935	12.038	10.588	17.606	9.559
1	1.5115	13.952	8.193	12.262	19.336	13.048
1	1.5125	18.961	10.550	18.066	28.217	16.065
1	1.5135	16.143	9.283	15.595	17.998	19.101
1	1.5145	19.240	17.425	13.625	19.267	20.081
1	1.5155	17.970	9.676	10.430	15.396	10.772
1	1.5165	21.797	14.272	16.948	17.472	14.614
1	1.5175	14.327	20.745	16.857	14.819	15.740
1	1.5185	10.961	12.835	12.294	15.923	14.395
1	1.5195	12.025	13.313	13.182	22.690	14.895
1	1.5205	8.674	5.943	9.492	4.146	7.007
1	1.5215	9.880	10.246	13.881	7.205	12.949
1	1.5225	14.122	22.094	16.846	23.715	12.756
1	1.5235	18.994	25.647	17.386	19.109	16.547
1	1.5245	8.992	11.141	12.226	17.642	20.538
1	1.5255	22.400	16.309	18.150	21.411	21.557
1	1.5265	23.000	13.709	20.811	24.396	14.567
1	1.5275	22.970	10.423	18.242	15.913	17.856
1	1.5285	8.000	4.286	9.644	7.910	10.158
1	1.5295	9.622	11.018	10.922	15.299	17.262
1	1.5305	23.480	27.325	26.958	16.913	18.405
1	1.5315	18.413	21.769	21.891	15.517	17.512
1	1.5325	18.837	25.669	24.766	23.706	16.155
1	1.5335	28.006	30.953	35.077	14.792	21.180
1	1.5345	25.115	24.017	30.711	15.571	9.966
1	1.5355	16.009	17.693	15.379	12.861	7.714
1	1.5365	15.949	13.677	19.289	11.495	11.738
1	1.5375	12.779	16.953	17.661	31.036	28.655
1	1.5385	16.836	21.906	20.288	27.699	21.285
1	1.5395	17.201	15.117	17.233	10.286	18.587
1	1.5405	21.336	16.633	20.516	13.006	14.767
1	1.5415	12.367	11.877	14.607	15.000	11.573
1	1.5425	10.602	13.419	15.278	20.802	17.282
1	1.5435	9.307	8.230	8.520	15.939	16.349
1	1.5445	9.224	9.461	8.327	6.761	12.452
1	1.5455	15.969	21.245	16.491	19.011	5.087
1	1.5465	15.903	22.041	19.427	11.471	6.638

1	1.5475	7.137	9.734	9.892	14.617	15.918
1	1.5485	8.622	9.086	10.066	12.562	16.052
1	1.5495	0.000	0.000	0.000	0.000	0.000
2	0.0006	0.000	0.000	0.000	0.000	0.000
2	0.0016	0.000	0.000	0.000	0.000	0.000
2	0.0026	5.672	1.856	2.068	14.728	17.130
2	0.0036	3.768	3.313	6.221	11.694	9.675
2	0.0046	8.197	4.948	6.461	17.160	25.277
2	0.0056	6.664	4.581	6.789	16.204	15.164
2	0.0066	7.859	7.753	14.414	21.221	13.881
2	0.0076	11.132	8.478	18.365	20.767	14.965
2	0.0086	8.544	7.457	9.287	28.552	13.509
2	0.0096	8.020	8.607	14.504	29.111	16.074
2	0.0106	9.777	12.672	16.361	25.578	35.049
2	0.0116	19.874	16.850	24.164	26.475	32.241
2	0.0126	19.163	12.137	23.140	17.297	16.854
2	0.0136	9.883	15.260	16.354	22.487	16.171
2	0.0146	15.298	18.149	22.587	16.472	23.533
2	0.0156	22.825	16.034	22.535	25.283	22.401
2	0.0166	17.827	15.414	15.706	17.603	17.050
2	0.0176	22.977	15.421	22.939	14.435	17.017
2	0.0186	13.074	12.912	17.398	11.180	10.925
2	0.0196	18.352	16.760	21.791	11.316	19.364
2	0.0206	17.943	16.430	21.369	11.530	19.617
2	0.0216	17.428	21.923	20.660	14.183	9.416
2	0.0226	17.797	20.335	18.424	7.872	5.766
2	0.0236	14.963	15.466	16.673	13.633	4.724
2	0.0246	27.781	23.350	28.139	14.975	13.045
2	0.0256	15.742	17.539	15.181	8.703	8.139
2	0.0266	16.889	14.482	14.382	9.036	7.408
2	0.0276	10.939	10.964	14.622	6.049	7.442
2	0.0286	12.547	10.288	14.350	4.576	8.085
2	0.0296	24.701	21.291	27.893	7.048	14.146
2	0.0306	16.077	17.633	17.714	12.651	13.881
2	0.0316	29.578	22.110	25.197	19.476	20.789
2	0.0326	15.288	21.607	14.272	17.976	14.935
2	0.0336	15.432	17.806	18.237	14.781	10.527
2	0.0346	14.294	19.255	19.544	21.620	9.585
2	0.0356	20.627	24.797	22.659	12.551	8.831
2	0.0366	15.588	20.339	25.329	16.627	15.537
2	0.0376	14.414	14.936	18.681	18.778	17.197
2	0.0386	28.517	23.466	28.445	18.262	20.441
2	0.0396	25.903	24.662	23.238	20.943	15.954
2	0.0406	14.477	15.319	21.767	22.957	13.817
2	0.0416	23.986	26.302	30.994	21.540	18.594
2	0.0426	22.467	14.967	25.660	18.740	19.433
2	0.0436	21.470	36.686	36.547	33.662	21.050
2	0.0446	15.734	15.243	23.861	21.544	21.710
2	0.0456	17.414	19.964	21.732	25.726	24.057
2	0.0466	15.379	19.387	18.881	19.960	32.248

2	0.0476	15.850	17.332	20.288	16.165	26.260
2	0.0486	23.004	21.663	24.302	9.305	24.330
2	0.0496	9.465	9.116	9.932	16.012	17.575
2	0.0506	21.361	21.670	32.394	11.434	19.577
2	0.0516	15.164	16.837	25.018	14.641	20.895
2	0.0526	14.378	11.913	13.362	21.261	18.704
2	0.0536	18.875	22.835	30.294	18.756	17.209
2	0.0546	12.188	15.211	18.509	23.613	28.016
2	0.0556	13.408	21.487	17.048	20.419	14.057
2	0.0566	10.015	8.954	9.994	16.049	7.384
2	0.0576	12.771	13.073	14.789	18.652	23.232
2	0.0586	11.326	15.721	18.467	27.166	19.621
2	0.0596	24.300	32.829	32.068	26.969	27.687
2	0.0606	11.656	17.665	13.872	23.935	35.104
2	0.0616	14.664	19.538	22.500	13.453	11.793
2	0.0626	14.388	19.989	16.900	19.496	12.073
2	0.0636	5.440	6.235	7.512	15.942	14.915
2	0.0646	8.253	3.671	5.994	12.392	22.045
2	0.0656	16.794	20.612	19.241	20.898	12.088
2	0.0666	16.553	25.438	19.929	18.487	13.347
2	0.0676	6.468	7.746	10.056	11.334	21.492
2	0.0686	10.279	13.466	13.520	7.472	7.280
2	0.0696	14.012	17.468	18.754	9.606	20.815
2	0.0706	21.335	29.563	29.746	16.981	14.527
2	0.0716	13.271	17.484	21.427	15.922	10.701
2	0.0726	17.423	16.161	20.301	10.506	20.313
2	0.0736	23.962	18.729	21.255	19.011	28.076
2	0.0746	14.777	18.648	15.117	24.638	19.838
2	0.0756	14.407	16.259	15.076	9.362	15.707
2	0.0766	12.132	16.744	14.622	14.588	26.100
2	0.0776	9.909	9.076	13.951	13.298	16.128
2	0.0786	12.836	12.608	16.624	13.231	22.525
2	0.0796	9.290	9.883	12.749	5.905	19.346
2	0.0806	14.700	19.014	21.465	11.131	18.348
2	0.0816	12.094	11.214	17.283	6.248	12.330
2	0.0826	7.282	4.918	7.980	7.211	10.882
2	0.0836	17.661	20.594	31.440	7.621	20.235
2	0.0846	16.257	25.990	32.126	14.152	19.122
2	0.0856	26.795	35.047	31.756	19.291	25.991
2	0.0866	15.463	16.429	19.325	13.553	26.674
2	0.0876	4.667	9.740	9.753	13.310	26.148
2	0.0886	9.231	15.397	15.833	12.090	19.027
2	0.0896	9.200	13.560	14.566	13.398	12.733
2	0.0906	11.391	15.281	14.691	12.561	18.525
2	0.0916	10.492	7.459	13.213	18.948	30.547
2	0.0926	13.777	16.537	20.003	11.174	16.220
2	0.0936	13.077	14.616	16.442	5.165	4.844
2	0.0946	20.702	25.646	25.285	14.751	19.361
2	0.0956	19.390	24.403	26.624	13.208	21.373
2	0.0966	13.256	13.600	23.549	16.661	18.199

2	0.0976	20.471	6.705	17.080	13.057	23.137
2	0.0986	12.736	18.698	24.718	25.629	27.102
2	0.0996	13.362	19.387	25.943	12.165	16.143
2	0.1006	14.062	22.895	30.865	22.568	21.476
2	0.1016	10.404	23.834	17.046	20.696	13.195
2	0.1026	11.389	16.921	10.021	18.618	12.443
2	0.1036	8.836	11.421	10.754	9.350	18.027
2	0.1046	11.177	14.220	13.430	22.167	14.250
2	0.1056	7.129	13.051	12.205	25.849	24.589
2	0.1066	8.971	9.533	9.721	18.788	28.544
2	0.1076	17.802	15.402	19.634	9.423	13.437
2	0.1086	17.493	13.989	18.712	13.317	14.545
2	0.1096	12.660	11.918	14.062	16.073	18.443
2	0.1106	13.695	13.502	14.594	14.258	14.753
2	0.1116	8.689	11.750	7.816	14.377	14.488
2	0.1126	4.933	8.125	4.112	23.327	16.071
2	0.1136	6.372	8.604	10.204	9.934	12.011
2	0.1146	9.047	7.274	10.044	9.448	12.593
2	0.1156	8.578	7.128	10.160	15.792	17.161
2	0.1166	3.617	6.394	4.840	7.603	6.649
2	0.1176	10.900	9.966	10.657	12.173	13.256
2	0.1186	13.480	10.328	17.477	11.496	12.519
2	0.1196	4.274	5.193	7.806	5.535	7.107
2	0.1206	5.122	14.359	11.475	23.629	23.911
2	0.1216	9.353	11.888	11.573	11.916	23.512
2	0.1226	10.544	14.711	12.679	13.718	18.033
2	0.1236	13.388	16.069	10.740	20.187	14.681
2	0.1246	11.196	16.960	12.888	23.164	26.537
2	0.1256	12.146	14.625	14.552	14.253	14.877
2	0.1266	22.709	21.951	15.641	19.523	20.093
2	0.1276	21.739	26.270	23.697	23.418	13.533
2	0.1286	9.803	10.332	16.720	11.563	11.479
2	0.1296	10.307	9.976	15.874	9.483	8.089
2	0.1306	16.061	23.080	21.302	16.756	13.109
2	0.1316	14.254	19.393	14.057	16.708	10.801
2	0.1326	8.744	8.749	9.681	9.857	11.280
2	0.1336	11.902	17.842	14.554	20.849	9.808
2	0.1346	16.273	17.706	21.689	18.605	13.900
2	0.1356	16.023	22.215	19.717	23.780	7.020
2	0.1366	17.540	18.719	23.348	20.916	20.465
2	0.1376	23.407	20.006	25.578	10.822	18.433
2	0.1386	20.522	23.571	27.760	16.132	21.120
2	0.1396	21.407	24.139	26.676	8.299	18.050
2	0.1406	19.191	20.411	26.976	4.394	12.598
2	0.1416	6.107	10.367	10.428	6.381	12.550
2	0.1426	3.408	19.122	12.904	14.473	8.118
2	0.1436	3.810	11.270	13.120	10.974	15.629
2	0.1446	18.543	15.510	19.932	5.759	14.863
2	0.1456	18.139	26.902	23.538	25.420	23.611
2	0.1466	18.322	26.861	23.649	25.762	23.880

2	0.1476	25.985	22.245	21.783	13.451	16.586
2	0.1486	23.769	22.247	24.098	20.862	23.998
2	0.1496	22.664	30.560	35.862	13.512	14.642
2	0.1506	22.115	29.974	28.338	15.110	10.532
2	0.1516	24.839	35.758	33.608	14.518	10.155
2	0.1526	11.525	17.704	14.513	15.340	15.928
2	0.1536	13.568	17.539	19.819	15.581	20.167
2	0.1546	18.916	22.426	24.966	11.938	9.829
2	0.1556	8.946	14.840	13.005	17.244	11.463
2	0.1566	16.045	18.903	17.207	24.124	14.721
2	0.1576	16.001	18.906	17.096	24.282	14.636
2	0.1586	20.681	21.757	18.334	21.081	11.673
2	0.1596	12.230	16.905	10.704	21.369	8.560
2	0.1606	18.444	20.415	12.766	13.237	12.066
2	0.1616	20.022	15.040	24.445	7.451	10.864
2	0.1626	15.984	20.040	21.334	9.666	9.141
2	0.1636	15.886	18.196	21.475	16.014	10.562
2	0.1646	6.050	6.762	11.025	11.702	17.231
2	0.1656	6.823	10.010	15.224	8.174	16.223
2	0.1666	6.811	10.006	11.549	19.377	11.948
2	0.1676	8.470	8.592	12.450	9.332	15.243
2	0.1686	9.071	9.410	10.276	11.506	7.935
2	0.1696	20.711	20.122	21.501	16.373	10.103
2	0.1706	11.389	5.918	6.996	12.771	23.032
2	0.1716	12.378	8.781	9.094	12.909	14.289
2	0.1726	13.338	11.505	12.449	16.214	12.764
2	0.1736	10.834	17.385	19.400	12.747	26.098
2	0.1746	19.234	20.860	22.298	16.169	15.457
2	0.1756	20.417	20.564	23.970	16.177	11.470
2	0.1766	14.146	22.569	19.191	12.511	8.714
2	0.1776	25.525	32.722	30.154	26.080	14.748
2	0.1786	15.215	23.451	22.227	21.281	23.218
2	0.1796	15.002	28.626	25.817	24.736	13.530
2	0.1806	20.711	21.310	24.330	28.015	18.385
2	0.1816	25.289	19.719	27.735	16.767	10.696
2	0.1826	15.116	15.600	17.782	10.339	3.588
2	0.1836	17.510	15.414	17.115	6.346	11.662
2	0.1846	8.355	4.849	5.729	16.189	9.162
2	0.1856	12.629	9.628	11.900	14.278	10.915
2	0.1866	15.689	17.229	20.974	12.539	24.265
2	0.1876	13.650	21.148	20.650	12.343	9.251
2	0.1886	7.636	16.537	16.522	9.055	9.613
2	0.1896	12.177	25.297	18.503	15.734	13.576
2	0.1906	12.141	18.573	13.864	13.617	8.291
2	0.1916	8.123	23.545	20.326	21.064	19.939
2	0.1926	11.981	20.996	18.628	20.267	11.760
2	0.1936	20.962	14.859	21.843	7.012	7.102
2	0.1946	12.424	13.367	15.440	9.802	5.280
2	0.1956	13.578	19.059	21.969	13.198	21.021
2	0.1966	14.549	21.167	23.861	14.957	20.250

2	0.1976	12.650	23.196	18.555	24.753	17.812
2	0.1986	8.385	12.481	17.917	14.558	15.627
2	0.1996	8.949	11.124	8.034	12.879	7.223
2	0.2006	13.808	19.300	19.818	18.087	22.289
2	0.2016	9.329	13.321	11.580	18.949	20.678
2	0.2026	6.167	7.085	9.730	11.211	16.906
2	0.2036	11.932	11.012	9.350	13.342	16.839
2	0.2046	17.163	7.484	9.061	12.065	13.439
2	0.2056	6.056	9.002	11.556	14.589	10.099
2	0.2066	16.541	18.407	19.964	25.548	10.416
2	0.2076	7.227	8.114	7.799	16.106	6.209
2	0.2086	9.682	4.740	9.748	9.615	7.412
2	0.2096	12.670	8.452	14.523	6.700	6.851
2	0.2106	11.562	8.132	8.847	18.465	16.795
2	0.2116	7.453	8.279	8.407	19.356	11.569
2	0.2126	9.997	11.522	9.745	13.830	5.634
2	0.2136	23.963	12.819	18.013	25.135	7.481
2	0.2146	7.369	9.695	9.995	21.803	10.050
2	0.2156	10.508	10.003	11.206	23.121	10.347
2	0.2166	10.557	5.816	5.809	14.627	13.699
2	0.2176	13.647	13.764	8.495	16.800	15.572
2	0.2186	21.483	22.383	16.121	17.844	13.124
2	0.2196	10.305	7.378	10.321	8.082	10.630
2	0.2206	14.686	17.224	17.611	13.712	14.688
2	0.2216	12.261	16.292	13.252	15.324	11.098
2	0.2226	12.598	8.410	8.634	27.554	25.051
2	0.2236	10.561	20.068	11.833	32.387	12.544
2	0.2246	10.947	14.432	18.810	12.549	6.280
2	0.2256	12.963	15.891	13.391	40.479	25.679
2	0.2266	13.440	16.781	14.893	39.292	17.985
2	0.2276	14.944	22.873	18.388	45.802	8.296
2	0.2286	6.268	15.082	15.426	19.863	7.918
2	0.2296	14.131	16.031	15.413	13.572	6.320
2	0.2306	14.221	19.454	16.061	33.390	10.580
2	0.2316	24.223	33.185	28.119	32.379	9.685
2	0.2326	16.711	21.174	16.110	27.458	5.194
2	0.2336	14.051	19.277	16.757	11.528	4.510
2	0.2346	14.078	19.365	16.865	11.577	4.509
2	0.2356	13.578	15.360	13.177	9.520	6.756
2	0.2366	15.766	13.547	17.268	7.562	10.432
2	0.2376	13.701	15.456	13.705	7.963	12.420
2	0.2386	6.367	13.095	9.084	14.374	8.227
2	0.2396	6.380	13.080	9.057	14.449	8.235
2	0.2406	6.395	13.063	9.033	14.521	8.242
2	0.2416	11.950	21.595	17.165	21.708	5.022
2	0.2426	16.141	26.851	14.114	28.814	13.944
2	0.2436	7.967	24.003	13.302	31.770	13.469
2	0.2446	9.368	17.892	11.713	17.626	12.088
2	0.2456	18.223	27.739	22.415	23.639	12.931
2	0.2466	7.512	21.229	13.616	34.974	15.172

2	0.2476	8.896	21.344	10.759	29.275	5.835
2	0.2486	8.876	21.308	10.733	29.157	5.827
2	0.2496	5.319	13.712	5.687	14.488	4.941
2	0.2506	5.823	10.754	10.630	16.982	5.717
2	0.2516	13.342	18.169	15.951	18.320	4.398
2	0.2526	12.074	15.860	17.060	12.060	4.390
2	0.2536	16.333	18.161	18.544	19.487	8.785
2	0.2546	17.618	20.591	18.970	23.993	13.524
2	0.2556	20.762	34.735	27.934	24.416	9.458
2	0.2566	20.349	25.054	23.307	8.775	4.092
2	0.2576	12.433	17.052	13.512	22.080	8.593
2	0.2586	16.771	22.049	17.142	27.109	5.740
2	0.2596	20.474	30.983	28.881	15.862	10.000
2	0.2606	22.298	33.864	35.095	18.452	15.118
2	0.2616	25.046	45.864	37.737	33.762	12.471
2	0.2626	11.379	12.979	13.843	19.980	6.736
2	0.2636	30.932	39.013	42.441	26.347	16.414
2	0.2646	16.722	21.833	17.588	23.121	7.033
2	0.2656	14.558	21.009	19.428	22.565	17.070
2	0.2666	14.524	20.959	19.338	22.548	17.146
2	0.2676	13.476	14.638	16.192	16.578	9.519
2	0.2686	9.991	11.537	8.421	14.183	7.028
2	0.2696	17.571	16.170	15.772	23.095	10.902
2	0.2706	20.475	18.881	20.404	12.915	11.237
2	0.2716	12.115	19.790	14.165	13.101	4.757
2	0.2726	15.698	26.754	17.327	24.260	10.012
2	0.2736	20.619	16.798	23.758	7.744	6.476
2	0.2746	16.888	11.156	20.289	4.247	6.937
2	0.2756	13.396	15.615	17.383	16.099	7.837
2	0.2766	19.944	15.917	22.464	12.572	7.408
2	0.2776	12.683	13.427	11.272	17.128	14.119
2	0.2786	16.095	34.084	22.233	26.274	5.799
2	0.2796	7.585	6.900	7.393	7.738	3.549
2	0.2806	25.195	13.057	16.470	20.225	8.645
2	0.2816	19.050	16.492	15.740	18.077	6.968
2	0.2826	13.600	14.727	19.386	9.187	8.590
2	0.2836	8.803	13.055	11.361	16.674	11.184
2	0.2846	7.921	12.448	11.796	12.592	5.647
2	0.2856	17.824	17.885	23.449	14.453	12.710
2	0.2866	28.840	28.831	30.244	11.034	15.884
2	0.2876	18.332	18.740	19.224	7.796	11.074
2	0.2886	19.047	16.401	20.238	8.754	13.690
2	0.2896	6.899	11.561	10.061	19.833	6.380
2	0.2906	8.863	13.059	12.156	17.279	7.509
2	0.2916	11.820	11.999	16.264	12.255	13.714
2	0.2926	27.470	24.605	28.139	12.050	14.519
2	0.2936	13.557	16.083	15.475	15.691	11.053
2	0.2946	18.352	23.019	23.231	8.818	13.864
2	0.2956	19.133	18.507	19.116	8.142	19.551
2	0.2966	18.954	12.655	12.682	5.596	10.099

2	0.2976	8.957	8.648	10.358	11.274	8.854
2	0.2986	12.760	20.973	11.494	19.810	12.114
2	0.2996	15.878	17.256	15.611	13.629	12.885
2	0.3006	21.816	14.981	24.368	15.863	8.877
2	0.3016	25.970	17.708	23.665	16.293	18.861
2	0.3026	17.217	17.529	23.145	16.198	12.240
2	0.3036	29.010	17.730	25.760	10.345	11.486
2	0.3046	9.853	10.950	8.217	12.262	12.566
2	0.3056	17.681	13.388	13.406	12.253	6.644
2	0.3066	12.153	7.535	18.388	11.101	13.160
2	0.3076	13.697	13.649	20.705	15.126	15.875
2	0.3086	19.171	19.741	23.011	9.447	5.583
2	0.3096	18.849	17.454	20.074	13.607	12.695
2	0.3106	15.356	14.416	17.224	14.505	13.177
2	0.3116	8.395	10.267	10.603	6.498	8.972
2	0.3126	11.391	10.803	15.185	6.987	6.742
2	0.3136	14.926	13.484	17.933	7.533	12.364
2	0.3146	13.285	6.629	11.053	5.376	10.946
2	0.3156	5.586	6.854	6.272	5.240	2.111
2	0.3166	11.641	8.445	11.056	7.561	7.009
2	0.3176	13.682	15.916	18.776	7.973	7.834
2	0.3186	16.568	14.866	18.744	7.328	7.162
2	0.3196	26.088	19.239	25.238	9.811	11.139
2	0.3206	9.523	10.022	12.915	9.955	12.973
2	0.3216	15.397	7.684	14.663	18.314	10.252
2	0.3226	15.547	8.917	15.735	13.509	7.000
2	0.3236	11.826	7.512	12.058	7.174	5.248
2	0.3246	12.797	6.058	10.203	10.671	8.706
2	0.3256	9.476	10.890	10.856	17.116	8.263
2	0.3266	3.066	5.903	4.542	10.559	4.917
2	0.3276	8.841	9.713	14.353	14.158	10.277
2	0.3286	10.705	13.470	15.752	7.513	9.474
2	0.3296	3.518	9.545	6.814	8.391	10.142
2	0.3306	5.642	4.391	4.050	8.554	5.497
2	0.3316	9.448	7.582	11.027	10.446	17.257
2	0.3326	6.920	9.464	8.037	13.462	11.694
2	0.3336	12.413	12.389	13.072	17.064	11.761
2	0.3346	11.124	13.351	13.784	6.843	8.902
2	0.3356	7.500	10.992	12.983	16.064	22.745
2	0.3366	10.311	11.868	11.134	10.008	21.299
2	0.3376	14.289	11.852	13.846	7.555	14.397
2	0.3386	9.085	6.853	12.584	8.339	12.208
2	0.3396	12.244	8.467	13.088	17.107	8.907
2	0.3406	3.760	4.290	1.987	7.797	4.114
2	0.3416	4.859	5.696	3.719	15.023	9.385
2	0.3426	6.252	1.264	4.668	4.984	5.673
2	0.3436	3.386	5.486	5.898	6.125	1.842
2	0.3446	4.013	4.035	4.136	3.307	4.919
2	0.3456	4.879	8.344	8.600	8.242	9.718
2	0.3466	9.652	15.687	8.504	7.054	9.450

2	0.3476	8.096	10.167	7.347	9.671	2.464
2	0.3486	9.546	11.794	8.216	16.646	19.237
2	0.3496	10.729	14.642	14.695	15.672	11.209
2	0.3506	16.581	13.530	13.365	21.857	12.868
2	0.3516	6.692	5.428	8.550	14.652	11.259
2	0.3526	4.063	4.035	5.284	7.042	14.886
2	0.3536	6.076	5.587	6.016	12.317	10.407
2	0.3546	7.889	7.106	5.113	17.067	7.297
2	0.3556	9.239	4.107	9.723	19.480	14.154
2	0.3566	10.376	6.906	9.909	12.447	12.064
2	0.3576	9.206	7.069	6.457	11.661	11.843
2	0.3586	13.213	6.931	11.927	10.783	10.218
2	0.3596	10.004	8.755	8.872	11.993	12.361
2	0.3606	7.854	5.150	7.100	8.037	11.191
2	0.3616	7.463	4.856	6.473	8.820	11.519
2	0.3626	6.816	3.903	5.569	8.020	15.509
2	0.3636	6.690	3.644	4.220	13.646	8.684
2	0.3646	1.916	0.566	1.316	7.284	2.607
2	0.3656	1.009	3.019	3.207	10.256	8.686
2	0.3666	9.784	7.311	14.339	10.328	14.963
2	0.3676	10.109	9.542	10.075	12.000	7.561
2	0.3686	12.852	8.702	12.855	8.835	6.265
2	0.3696	17.543	20.857	14.164	14.638	16.637
2	0.3706	11.106	13.943	9.309	5.324	7.418
2	0.3716	23.028	9.880	13.484	12.931	14.496
2	0.3726	13.576	9.423	9.207	17.996	20.401
2	0.3736	6.938	5.802	3.419	11.301	12.211
2	0.3746	3.626	4.617	3.109	8.200	7.552
2	0.3756	21.477	9.786	17.806	12.184	9.707
2	0.3766	8.613	9.544	9.184	15.979	11.639
2	0.3776	5.796	7.526	7.143	12.949	11.412
2	0.3786	6.285	9.771	2.765	5.818	11.901
2	0.3796	13.367	14.449	10.695	14.871	17.333
2	0.3806	15.680	17.726	14.553	11.900	15.855
2	0.3816	17.746	17.840	15.790	13.113	15.702
2	0.3826	12.113	14.211	10.461	7.033	9.242
2	0.3836	2.428	4.141	2.719	11.459	3.653
2	0.3846	5.059	5.855	7.643	9.239	12.217
2	0.3856	6.449	6.608	6.661	12.980	7.859
2	0.3866	12.437	9.092	14.633	13.124	7.614
2	0.3876	7.456	8.004	11.348	11.860	6.552
2	0.3886	8.577	8.765	11.596	10.921	8.904
2	0.3896	8.569	6.289	5.447	11.759	6.978
2	0.3906	4.923	8.998	5.581	9.572	9.962
2	0.3916	12.306	12.062	10.708	12.003	16.610
2	0.3926	4.574	6.150	7.445	16.426	16.298
2	0.3936	11.791	13.230	16.576	7.437	7.976
2	0.3946	13.602	13.121	17.559	8.489	11.408
2	0.3956	17.013	9.813	16.181	16.513	24.072
2	0.3966	23.556	16.219	16.806	7.299	30.480

2	0.3976	11.406	13.420	7.633	10.187	24.500
2	0.3986	13.562	20.879	18.361	14.732	11.971
2	0.3996	4.102	5.968	6.839	14.098	11.348
2	0.4006	5.936	12.377	8.623	9.586	11.908
2	0.4016	17.601	17.950	13.834	9.606	16.332
2	0.4026	15.308	13.212	13.200	5.903	10.443
2	0.4036	13.051	13.525	11.162	6.345	14.457
2	0.4046	17.398	17.492	20.048	18.885	14.050
2	0.4056	21.387	17.572	20.117	12.257	16.478
2	0.4066	14.798	15.283	9.302	13.962	12.233
2	0.4076	12.828	15.013	8.760	12.352	12.524
2	0.4086	16.498	13.604	13.197	9.634	10.546
2	0.4096	4.518	3.539	2.583	10.939	7.046
2	0.4106	5.398	3.213	4.817	10.043	7.517
2	0.4116	13.130	8.963	14.960	4.054	8.381
2	0.4126	8.485	8.480	9.328	7.289	10.894
2	0.4136	12.242	9.441	11.726	14.737	14.362
2	0.4146	9.090	8.909	10.374	5.026	15.564
2	0.4156	8.235	8.364	9.446	7.237	8.137
2	0.4166	3.399	4.132	5.627	7.858	4.560
2	0.4176	6.870	2.994	8.606	5.392	5.215
2	0.4186	4.254	3.535	8.770	10.894	11.365
2	0.4196	4.506	7.032	9.126	11.189	12.973
2	0.4206	3.791	7.801	5.181	8.521	15.823
2	0.4216	11.050	15.116	13.108	23.093	21.979
2	0.4226	11.675	6.941	13.071	15.958	16.248
2	0.4236	16.758	13.012	18.978	10.097	14.537
2	0.4246	16.398	13.525	17.729	10.127	20.250
2	0.4256	13.293	11.402	11.979	8.620	20.392
2	0.4266	11.435	9.996	13.266	2.188	13.200
2	0.4276	9.582	8.450	11.471	6.485	10.019
2	0.4286	13.540	8.789	12.356	13.516	9.265
2	0.4296	15.012	7.597	13.913	10.157	8.693
2	0.4306	19.894	15.242	23.483	12.024	16.479
2	0.4316	16.052	10.232	14.550	17.103	13.539
2	0.4326	6.978	8.974	7.063	9.484	12.263
2	0.4336	9.933	11.827	7.083	4.236	16.599
2	0.4346	11.375	9.931	13.080	7.118	23.768
2	0.4356	4.014	10.027	6.425	20.964	11.518
2	0.4366	8.852	13.414	13.315	15.979	7.594
2	0.4376	23.090	18.646	24.038	12.302	14.119
2	0.4386	23.166	15.955	17.376	6.427	21.495
2	0.4396	16.616	10.761	11.726	4.685	17.196
2	0.4406	16.611	10.764	11.726	4.689	17.182
2	0.4416	20.070	18.809	19.516	18.544	22.859
2	0.4426	26.612	19.244	23.280	17.356	17.798
2	0.4436	13.183	10.686	13.639	12.544	7.980
2	0.4446	8.298	7.130	13.425	6.230	7.344
2	0.4456	10.896	5.648	13.381	8.109	8.519
2	0.4466	13.106	7.689	11.787	17.989	9.445

2	0.4476	9.314	3.940	9.408	13.171	11.500
2	0.4486	10.227	7.084	12.194	15.766	12.039
2	0.4496	16.359	13.139	17.389	32.028	13.714
2	0.4506	5.961	6.763	7.575	8.786	5.183
2	0.4516	10.669	7.685	12.159	24.390	20.308
2	0.4526	11.123	11.301	9.415	23.164	10.099
2	0.4536	12.724	14.167	13.528	9.510	8.275
2	0.4546	11.248	16.570	13.662	14.072	25.068
2	0.4556	9.633	16.119	11.755	17.164	11.051
2	0.4566	7.327	10.965	14.110	16.825	9.239
2	0.4576	8.993	8.205	13.167	11.590	8.198
2	0.4586	4.705	5.928	5.600	14.654	11.142
2	0.4596	9.381	8.640	5.141	10.691	10.782
2	0.4606	9.597	15.469	15.000	20.941	32.322
2	0.4616	18.083	24.533	15.621	21.462	19.897
2	0.4626	12.143	15.655	12.457	15.046	11.069
2	0.4636	6.540	23.187	16.034	28.812	15.804
2	0.4646	13.414	12.955	14.384	18.381	15.193
2	0.4656	12.807	17.706	18.193	12.189	10.087
2	0.4666	13.445	18.193	11.316	14.010	11.240
2	0.4676	8.401	4.926	12.578	4.389	8.012
2	0.4686	17.450	10.501	14.999	10.043	18.517
2	0.4696	14.658	12.358	17.638	10.215	18.070
2	0.4706	9.529	6.682	7.379	12.883	11.987
2	0.4716	11.580	12.296	16.797	13.619	18.675
2	0.4726	11.566	12.247	16.778	13.588	18.751
2	0.4736	6.241	5.309	4.793	15.267	5.550
2	0.4746	6.186	5.108	4.366	14.350	2.091
2	0.4756	1.682	0.983	0.923	4.926	2.822
2	0.4766	3.421	1.723	7.328	11.707	8.718
2	0.4776	9.885	8.705	18.119	16.576	17.717
2	0.4786	21.850	14.507	17.867	10.287	6.407
2	0.4796	11.807	11.134	12.869	17.129	10.187
2	0.4806	18.063	12.198	16.082	26.830	19.075
2	0.4816	16.297	15.647	13.917	21.320	19.209
2	0.4826	15.449	12.750	13.916	12.364	19.832
2	0.4836	12.254	11.569	14.323	13.084	16.341
2	0.4846	13.979	13.435	18.801	11.869	10.869
2	0.4856	13.093	9.359	12.965	4.707	7.434
2	0.4866	15.768	5.291	18.277	11.899	8.078
2	0.4876	17.391	13.332	20.283	8.811	6.283
2	0.4886	19.054	11.148	23.043	9.985	5.726
2	0.4896	16.516	10.944	14.985	6.114	17.328
2	0.4906	21.175	19.654	23.271	12.258	25.094
2	0.4916	18.806	17.748	20.162	15.464	9.575
2	0.4926	23.875	17.647	28.969	13.580	9.584
2	0.4936	19.807	17.678	23.843	17.136	14.530
2	0.4946	14.960	12.537	18.517	10.924	18.629
2	0.4956	13.786	12.121	14.181	17.032	18.434
2	0.4966	10.448	10.809	10.671	11.948	16.185

2	0.4976	8.574	12.784	12.509	21.090	17.003
2	0.4986	21.764	17.477	18.851	18.943	27.367
2	0.4996	9.979	15.004	10.375	10.119	12.139
2	0.5006	20.729	13.275	18.034	16.090	11.123
2	0.5016	24.948	8.735	21.928	13.194	11.744
2	0.5026	24.737	12.447	22.653	11.278	6.924
2	0.5036	16.453	11.326	16.293	9.934	7.891
2	0.5046	19.367	9.626	18.711	10.495	9.715
2	0.5056	9.800	6.840	15.410	6.997	5.727
2	0.5066	13.688	14.601	20.654	4.418	4.041
2	0.5076	17.107	10.314	14.308	6.539	4.434
2	0.5086	25.838	18.024	21.978	9.554	7.455
2	0.5096	20.684	13.532	16.796	13.469	15.652
2	0.5106	12.201	7.951	11.929	11.431	9.455
2	0.5116	17.783	10.128	16.654	15.394	16.483
2	0.5126	17.516	8.461	15.887	8.898	7.949
2	0.5136	10.243	6.702	10.204	9.255	13.201
2	0.5146	13.424	8.378	8.800	7.565	15.571
2	0.5156	16.179	16.122	15.694	21.412	12.432
2	0.5166	7.485	11.313	15.177	11.411	10.963
2	0.5176	11.186	7.992	12.582	3.912	6.415
2	0.5186	9.813	7.940	7.995	6.466	13.113
2	0.5196	11.948	9.407	10.959	8.079	18.389
2	0.5206	14.170	10.782	11.572	8.386	9.902
2	0.5216	14.200	15.944	13.656	10.899	7.231
2	0.5226	13.607	12.989	12.137	16.182	10.810
2	0.5236	11.879	10.894	14.792	20.966	10.755
2	0.5246	12.567	13.112	11.835	18.297	8.231
2	0.5256	12.949	14.193	13.867	13.351	11.042
2	0.5266	19.968	20.431	15.059	27.437	17.831
2	0.5276	19.505	19.040	19.097	15.191	9.252
2	0.5286	6.123	9.471	8.824	14.232	9.033
2	0.5296	9.536	6.979	7.908	10.501	15.105
2	0.5306	12.286	12.397	12.044	18.569	9.093
2	0.5316	9.380	15.734	8.404	15.559	5.131
2	0.5326	7.404	13.648	8.040	15.376	4.287
2	0.5336	14.970	21.967	12.961	32.357	17.829
2	0.5346	16.248	20.201	13.880	19.770	11.477
2	0.5356	15.979	8.902	11.785	8.935	7.463
2	0.5366	18.652	11.451	15.209	2.877	5.996
2	0.5376	8.839	7.879	7.271	4.070	7.389
2	0.5386	18.553	11.515	13.464	7.620	14.079
2	0.5396	9.301	9.313	13.622	11.248	9.814
2	0.5406	22.832	12.667	14.194	9.155	17.074
2	0.5416	18.969	12.823	12.594	8.825	10.950
2	0.5426	12.817	13.558	11.850	5.240	22.152
2	0.5436	11.385	8.614	6.365	17.739	9.426
2	0.5446	12.220	10.557	11.331	11.076	9.861
2	0.5456	12.198	10.533	11.301	11.006	9.862
2	0.5466	13.483	10.769	11.251	14.602	9.908

2	0.5476	10.611	9.522	12.053	10.168	11.986
2	0.5486	8.325	6.679	9.045	9.594	5.522
2	0.5496	7.945	3.908	2.589	7.735	6.623
2	0.5506	6.973	6.990	4.229	5.721	5.924
2	0.5516	11.402	7.107	5.704	5.762	4.183
2	0.5526	14.654	13.785	12.091	9.161	13.017
2	0.5536	11.418	13.084	8.036	20.396	10.466
2	0.5546	12.022	10.189	6.684	11.276	7.880
2	0.5556	13.140	15.107	15.951	10.581	10.041
2	0.5566	12.369	10.509	14.010	10.307	10.061
2	0.5576	7.731	7.109	9.534	22.227	22.071
2	0.5586	6.389	8.507	4.467	12.327	13.391
2	0.5596	11.149	12.249	6.127	19.716	14.630
2	0.5606	5.281	8.660	3.723	21.117	10.543
2	0.5616	10.707	11.787	10.053	13.603	8.814
2	0.5626	15.217	6.669	8.763	17.900	20.338
2	0.5636	8.897	9.028	8.187	12.789	16.399
2	0.5646	8.973	8.945	8.169	12.823	16.331
2	0.5656	18.870	16.261	10.117	21.440	18.325
2	0.5666	11.250	9.323	8.457	13.640	6.819
2	0.5676	11.297	9.353	8.431	13.638	6.887
2	0.5686	11.345	9.384	8.403	13.636	6.954
2	0.5696	15.557	13.242	9.488	18.991	10.465
2	0.5706	17.358	11.159	12.371	20.576	13.128
2	0.5716	17.383	11.138	12.461	20.515	13.130
2	0.5726	8.866	8.992	5.102	15.639	6.589
2	0.5736	9.902	7.721	7.851	9.753	10.719
2	0.5746	8.289	6.919	5.255	11.568	5.660
2	0.5756	8.773	8.509	6.329	7.398	6.890
2	0.5766	10.703	5.935	7.287	5.588	7.800
2	0.5776	5.307	4.431	4.413	23.583	13.478
2	0.5786	4.081	3.854	1.644	14.442	11.390
2	0.5796	13.688	11.705	7.779	15.621	11.931
2	0.5806	6.886	2.342	3.652	3.001	5.715
2	0.5816	17.014	7.508	9.796	10.581	13.268
2	0.5826	21.588	12.033	15.017	11.872	8.581
2	0.5836	15.231	5.479	10.822	4.684	10.229
2	0.5846	17.445	9.246	9.720	9.990	22.195
2	0.5856	14.682	6.286	6.518	8.162	12.105
2	0.5866	11.416	5.612	6.238	16.528	10.458
2	0.5876	15.856	6.272	5.825	11.514	6.428
2	0.5886	16.050	12.820	6.927	8.883	4.561
2	0.5896	13.713	6.138	5.299	10.512	6.061
2	0.5906	9.397	7.480	5.939	17.310	3.598
2	0.5916	15.334	8.125	6.501	20.889	16.591
2	0.5926	15.922	4.636	5.758	10.061	9.198
2	0.5936	11.053	4.808	6.127	4.078	4.549
2	0.5946	19.786	17.390	12.686	24.873	10.128
2	0.5956	19.737	17.382	12.641	24.867	10.193
2	0.5966	19.478	19.205	15.088	16.359	19.083

2	0.5976	16.955	13.963	13.606	7.636	22.152
2	0.5986	14.600	7.446	8.278	14.852	14.841
2	0.5996	13.625	8.794	7.054	17.472	12.685
2	0.6006	22.817	8.825	11.519	16.444	13.842
2	0.6016	22.790	8.887	11.492	16.277	13.848
2	0.6026	15.112	4.958	7.718	5.408	10.011
2	0.6036	6.480	5.128	5.173	5.769	8.674
2	0.6046	6.463	5.118	5.176	5.771	8.649
2	0.6056	18.432	14.058	10.225	9.372	12.368
2	0.6066	8.712	4.585	5.818	5.820	10.885
2	0.6076	19.970	7.516	13.042	9.205	11.853
2	0.6086	15.488	3.916	10.482	4.031	6.781
2	0.6096	8.558	4.547	5.542	7.383	13.042
2	0.6106	15.793	6.480	12.134	9.052	6.516
2	0.6116	7.490	6.248	9.653	8.098	7.232
2	0.6126	14.065	7.611	13.365	11.303	4.207
2	0.6136	12.624	12.230	7.234	16.691	11.706
2	0.6146	11.189	11.269	7.700	12.419	10.014
2	0.6156	12.090	10.397	8.887	11.446	7.961
2	0.6166	13.765	7.040	10.132	8.023	7.416
2	0.6176	11.643	12.360	12.792	19.815	6.845
2	0.6186	9.867	6.685	9.269	12.277	8.747
2	0.6196	16.683	8.261	7.859	24.416	11.573
2	0.6206	14.472	8.675	9.151	21.842	14.659
2	0.6216	9.173	4.907	4.922	12.740	14.047
2	0.6226	17.858	12.855	10.528	13.634	9.915
2	0.6236	14.856	7.928	9.561	13.645	10.965
2	0.6246	8.668	7.022	7.624	16.261	13.923
2	0.6256	14.474	3.910	11.646	10.578	6.095
2	0.6266	13.151	5.191	8.970	9.645	15.442
2	0.6276	14.612	11.800	6.718	8.626	16.571
2	0.6286	14.611	11.800	6.717	8.626	16.573
2	0.6296	7.262	7.587	4.000	7.896	11.049
2	0.6306	12.333	5.475	11.776	7.967	11.260
2	0.6316	9.334	6.944	4.377	11.094	13.492
2	0.6326	17.457	21.264	11.902	10.237	19.989
2	0.6336	19.020	21.392	17.013	15.189	16.951
2	0.6346	9.793	6.804	8.330	7.591	19.776
2	0.6356	12.025	4.680	6.969	4.983	16.393
2	0.6366	14.397	14.971	11.978	7.789	17.419
2	0.6376	19.964	13.617	15.283	7.779	25.269
2	0.6386	28.692	15.173	17.653	23.524	21.955
2	0.6396	23.169	8.761	11.981	18.603	22.910
2	0.6406	11.210	5.642	4.227	10.197	18.099
2	0.6416	20.105	7.014	12.944	7.208	18.970
2	0.6426	14.602	8.985	7.589	11.720	9.445
2	0.6436	12.851	6.029	5.415	13.381	20.511
2	0.6446	16.922	13.400	12.704	28.368	11.695
2	0.6456	20.494	10.969	8.269	8.071	14.459
2	0.6466	34.283	6.036	13.048	11.705	14.695

2	0.6476	38.785	8.267	15.838	16.905	12.167
2	0.6486	38.782	8.266	15.837	16.906	12.169
2	0.6496	12.867	6.364	10.816	10.233	18.889
2	0.6506	11.410	9.256	9.116	11.527	15.910
2	0.6516	11.426	5.060	10.919	14.105	11.321
2	0.6526	21.172	12.128	11.230	9.498	5.002
2	0.6536	17.773	9.976	9.380	10.471	7.714
2	0.6546	10.957	6.320	7.873	7.274	6.796
2	0.6556	17.700	7.944	6.261	7.066	10.143
2	0.6566	14.807	7.454	7.958	4.369	10.316
2	0.6576	20.317	13.011	11.698	12.189	14.126
2	0.6586	11.980	6.520	8.632	6.203	9.909
2	0.6596	21.148	6.627	14.788	6.615	1.255
2	0.6606	33.257	16.876	25.467	20.129	10.861
2	0.6616	16.628	13.835	13.326	13.774	14.312
2	0.6626	24.809	8.828	14.200	16.296	10.042
2	0.6636	25.507	11.620	13.420	13.238	10.535
2	0.6646	22.599	10.717	14.711	7.941	6.646
2	0.6656	9.946	13.193	12.034	10.561	12.816
2	0.6666	12.224	9.685	10.953	6.346	8.318
2	0.6676	17.482	9.986	14.532	11.741	9.481
2	0.6686	9.204	7.053	14.797	7.933	4.543
2	0.6696	12.530	10.011	18.545	8.297	14.460
2	0.6706	16.556	14.855	18.325	10.557	7.062
2	0.6716	8.925	10.764	10.683	15.846	7.619
2	0.6726	9.299	5.286	8.307	9.281	3.709
2	0.6736	14.558	8.041	8.456	4.671	3.381
2	0.6746	10.243	1.923	7.302	4.014	5.964
2	0.6756	11.596	7.065	12.127	5.259	9.602
2	0.6766	14.691	12.997	14.692	12.317	8.959
2	0.6776	13.219	13.265	14.948	3.222	8.523
2	0.6786	8.506	8.184	8.814	8.995	5.371
2	0.6796	16.408	19.528	18.555	14.458	6.674
2	0.6806	16.464	10.776	13.586	11.222	3.616
2	0.6816	19.272	14.559	16.334	21.978	7.125
2	0.6826	11.268	12.511	13.083	16.521	7.238
2	0.6836	8.341	8.640	6.451	10.904	6.270
2	0.6846	10.530	11.298	6.814	9.204	15.415
2	0.6856	9.391	8.759	11.357	15.157	10.772
2	0.6866	13.651	17.280	17.875	12.165	11.179
2	0.6876	18.832	12.143	14.086	10.856	9.399
2	0.6886	18.442	11.636	12.176	17.218	16.749
2	0.6896	6.428	10.947	8.817	9.160	11.027
2	0.6906	11.977	10.609	9.498	7.307	12.490
2	0.6916	8.516	3.600	7.820	5.518	10.629
2	0.6926	8.976	6.910	7.338	2.801	4.686
2	0.6936	10.886	9.621	16.195	18.336	14.858
2	0.6946	11.011	6.231	9.444	7.477	2.889
2	0.6956	11.344	8.223	9.884	15.554	4.933
2	0.6966	17.922	16.006	23.866	10.156	8.742

2	0.6976	24.949	15.032	21.686	11.270	11.904
2	0.6986	21.462	11.487	16.705	9.912	23.934
2	0.6996	13.296	16.936	17.696	9.576	19.460
2	0.7006	7.257	12.510	12.697	13.418	12.976
2	0.7016	9.023	5.286	9.764	5.933	10.605
2	0.7026	9.018	5.285	9.759	5.930	10.604
2	0.7036	23.028	10.024	18.322	12.419	9.499
2	0.7046	5.051	5.574	6.919	12.262	14.141
2	0.7056	4.502	1.514	5.887	14.099	8.243
2	0.7066	9.235	6.745	9.714	13.577	11.452
2	0.7076	2.165	1.839	4.100	11.126	6.018
2	0.7086	2.165	1.839	4.097	11.125	5.971
2	0.7096	2.038	2.087	3.264	7.642	10.487
2	0.7106	3.047	2.599	3.118	7.923	10.500
2	0.7116	3.037	2.599	3.107	7.912	10.466
2	0.7126	5.556	5.355	9.267	8.377	8.701
2	0.7136	13.459	9.557	11.394	16.726	13.532
2	0.7146	11.899	9.528	14.304	8.732	10.685
2	0.7156	7.581	8.253	8.957	7.511	8.905
2	0.7166	3.224	4.786	3.965	10.233	5.728
2	0.7176	6.078	5.974	6.851	11.805	5.555
2	0.7186	13.906	10.138	12.834	12.500	7.074
2	0.7196	16.255	11.497	21.726	11.188	21.676
2	0.7206	11.444	6.483	11.817	7.671	13.522
2	0.7216	11.782	11.333	11.799	5.000	16.364
2	0.7226	13.413	10.909	11.061	4.594	19.516
2	0.7236	3.854	4.392	4.670	5.883	13.544
2	0.7246	8.906	4.348	7.431	3.755	10.839
2	0.7256	21.906	12.165	17.841	14.239	18.260
2	0.7266	6.596	9.052	8.694	12.422	17.782
2	0.7276	4.524	7.040	6.678	14.602	9.276
2	0.7286	11.431	11.584	13.337	20.413	11.477
2	0.7296	8.924	8.773	12.052	15.992	10.386
2	0.7306	13.835	6.438	11.075	13.317	13.831
2	0.7316	10.601	6.739	7.768	5.965	3.123
2	0.7326	21.051	8.236	17.549	17.462	14.496
2	0.7336	22.687	9.122	17.057	16.199	11.207
2	0.7346	11.192	6.554	13.649	11.924	10.330
2	0.7356	12.755	12.388	15.571	7.137	9.511
2	0.7366	8.602	7.796	9.814	8.312	4.548
2	0.7376	15.213	10.665	16.091	12.552	7.402
2	0.7386	10.594	5.607	9.504	13.889	15.108
2	0.7396	9.281	10.355	9.122	15.749	7.435
2	0.7406	20.738	10.923	15.985	12.665	11.647
2	0.7416	21.629	8.106	19.264	13.661	19.389
2	0.7426	9.217	11.878	13.992	16.595	13.597
2	0.7436	7.514	4.451	5.641	9.194	5.100
2	0.7446	13.831	7.048	13.357	7.807	5.618
2	0.7456	25.966	8.163	22.503	15.369	6.774
2	0.7466	10.728	6.263	8.925	9.870	12.790

2	0.7476	7.831	7.920	9.826	3.310	9.422
2	0.7486	7.838	7.923	9.831	3.381	9.435
2	0.7496	5.371	4.364	7.835	8.143	10.357
2	0.7506	11.128	10.171	14.735	17.569	11.570
2	0.7516	15.516	10.197	15.485	13.975	9.226
2	0.7526	12.071	8.442	10.495	13.548	11.347
2	0.7536	20.060	10.966	17.441	13.507	9.476
2	0.7546	13.661	11.934	12.217	12.961	10.638
2	0.7556	17.454	10.682	12.945	13.169	12.843
2	0.7566	14.545	9.386	13.822	10.857	10.931
2	0.7576	16.293	8.928	12.248	13.541	9.672
2	0.7586	9.786	6.412	7.442	10.628	7.905
2	0.7596	12.406	7.550	10.473	14.597	10.211
2	0.7606	11.813	10.974	12.231	15.692	14.722
2	0.7616	12.112	11.359	12.991	12.446	12.756
2	0.7626	9.035	5.855	10.177	13.477	9.733
2	0.7636	5.082	2.763	4.639	6.072	4.607
2	0.7646	7.447	6.762	6.630	8.115	10.293
2	0.7656	2.317	1.309	1.622	5.706	7.724
2	0.7666	6.483	6.541	4.723	12.646	5.449
2	0.7676	10.669	9.145	8.017	8.403	9.178
2	0.7686	10.821	5.208	10.523	10.015	7.591
2	0.7696	13.642	9.381	12.800	11.710	6.474
2	0.7706	9.936	6.858	7.016	5.651	8.245
2	0.7716	12.172	3.215	8.475	13.619	12.090
2	0.7726	10.755	2.892	7.420	12.278	10.807
2	0.7736	16.707	8.694	13.177	15.185	12.412
2	0.7746	2.021	2.829	3.172	2.491	3.167
2	0.7756	10.180	6.745	10.239	5.341	6.846
2	0.7766	10.179	6.750	10.242	5.344	6.852
2	0.7776	13.192	8.091	13.731	12.762	4.340
2	0.7786	8.957	6.251	9.585	5.363	7.370
2	0.7796	12.651	6.841	10.211	5.287	7.537
2	0.7806	8.148	7.206	7.643	7.911	4.607
2	0.7816	6.864	3.609	5.422	3.789	2.350
2	0.7826	4.863	5.491	4.228	23.967	10.986
2	0.7836	13.021	11.563	10.862	28.469	16.696
2	0.7846	15.710	10.779	12.776	15.759	9.829
2	0.7856	10.447	5.823	6.129	11.280	6.906
2	0.7866	6.479	6.525	4.296	14.816	3.225
2	0.7876	3.892	8.002	4.295	17.136	4.648
2	0.7886	8.340	11.386	7.313	13.732	6.830
2	0.7896	10.371	6.349	9.508	7.317	4.822
2	0.7906	11.312	9.962	10.305	9.558	6.401
2	0.7916	12.948	8.835	8.428	8.970	5.274
2	0.7926	20.663	13.976	12.805	19.931	8.530
2	0.7936	20.713	14.109	12.842	20.071	8.481
2	0.7946	22.787	16.105	23.539	15.812	16.307
2	0.7956	11.181	8.502	12.611	5.915	11.143
2	0.7966	9.816	7.716	9.908	15.149	14.137

2	0.7976	18.879	14.847	19.717	14.068	12.404
2	0.7986	11.645	5.115	10.687	10.884	10.270
2	0.7996	15.512	13.869	14.095	20.590	12.203
2	0.8006	13.052	10.981	10.526	10.646	20.196
2	0.8016	29.259	15.389	24.996	14.129	21.105
2	0.8026	21.541	11.522	18.905	11.071	22.580
2	0.8036	14.792	11.267	14.249	14.500	22.730
2	0.8046	19.257	8.767	18.440	10.140	13.680
2	0.8056	20.219	9.899	18.006	12.469	9.831
2	0.8066	8.549	6.575	8.504	11.181	9.646
2	0.8076	8.550	6.576	8.506	11.181	9.634
2	0.8086	8.550	6.578	8.507	11.181	9.622
2	0.8096	17.871	9.431	11.535	10.712	12.779
2	0.8106	24.425	12.424	20.606	13.306	14.962
2	0.8116	14.386	9.529	11.567	10.046	21.192
2	0.8126	21.081	9.428	14.111	10.641	17.202
2	0.8136	14.015	6.642	11.373	15.831	11.046
2	0.8146	14.048	5.697	8.770	10.685	6.765
2	0.8156	12.870	7.958	10.234	4.670	19.194
2	0.8166	15.249	9.646	11.901	5.851	12.774
2	0.8176	17.981	10.468	12.025	5.896	13.909
2	0.8186	20.375	11.724	13.834	4.553	13.223
2	0.8196	7.737	5.095	5.100	2.374	6.441
2	0.8206	7.062	7.404	9.567	6.372	13.732
2	0.8216	4.905	7.099	7.738	5.955	9.066
2	0.8226	12.948	5.560	15.575	12.446	6.621
2	0.8236	20.364	12.227	20.745	21.161	6.682
2	0.8246	21.841	12.967	18.355	13.499	7.914
2	0.8256	16.396	9.504	12.135	5.456	11.351
2	0.8266	15.316	10.763	12.706	5.240	9.825
2	0.8276	7.953	7.252	7.028	11.498	7.388
2	0.8286	13.879	6.074	9.621	23.688	7.729
2	0.8296	8.557	6.937	9.011	13.707	12.030
2	0.8306	9.802	5.397	9.935	11.907	13.068
2	0.8316	11.680	4.575	9.015	12.899	15.731
2	0.8326	11.680	4.574	9.015	12.900	15.730
2	0.8336	13.330	5.592	12.214	11.295	19.542
2	0.8346	7.176	1.879	6.587	7.951	6.429
2	0.8356	14.547	6.720	13.968	9.520	7.093
2	0.8366	14.460	6.382	12.919	5.268	9.541
2	0.8376	10.730	12.581	14.024	11.879	20.586
2	0.8386	18.821	14.420	19.688	17.692	14.174
2	0.8396	18.701	14.441	19.615	17.689	14.012
2	0.8406	11.115	10.305	10.005	18.837	11.956
2	0.8416	9.579	9.158	9.376	15.976	7.417
2	0.8426	10.247	8.031	10.056	16.955	13.406
2	0.8436	13.114	8.138	12.848	19.722	11.182
2	0.8446	17.789	4.593	14.331	14.503	8.857
2	0.8456	12.395	2.686	8.695	9.403	13.507
2	0.8466	14.712	8.305	13.282	14.367	15.107

2	0.8476	3.285	5.285	4.387	8.667	10.551
2	0.8486	5.722	5.238	4.563	11.994	7.197
2	0.8496	7.260	7.371	4.192	17.725	9.592
2	0.8506	14.404	15.092	12.544	31.697	18.545
2	0.8516	6.882	11.338	5.652	32.369	16.684
2	0.8526	5.942	6.482	3.992	13.740	13.470
2	0.8536	5.942	6.481	3.993	13.741	13.469
2	0.8546	7.869	3.182	4.219	15.565	5.149
2	0.8556	3.882	6.296	5.235	2.381	2.701
2	0.8566	9.726	18.671	10.972	11.889	9.921
2	0.8576	11.117	12.055	8.247	14.316	14.049
2	0.8586	7.230	4.951	4.721	6.459	16.056
2	0.8596	9.580	12.769	9.383	5.090	8.043
2	0.8606	14.239	9.956	13.103	14.502	8.506
2	0.8616	10.842	8.649	7.486	19.745	18.035
2	0.8626	2.929	6.480	4.617	26.227	12.355
2	0.8636	8.838	7.714	8.933	7.972	8.400
2	0.8646	18.393	10.952	16.659	14.741	5.083
2	0.8656	16.939	11.433	16.008	16.737	9.470
2	0.8666	15.805	11.752	17.259	15.109	16.429
2	0.8676	6.668	11.073	8.651	13.534	8.543
2	0.8686	3.791	11.556	5.625	14.637	8.005
2	0.8696	6.446	5.798	10.032	10.052	8.426
2	0.8706	14.764	12.096	12.238	11.902	19.669
2	0.8716	24.051	20.515	22.785	9.175	9.503
2	0.8726	9.348	8.268	11.806	8.292	14.818
2	0.8736	14.095	11.391	16.216	11.238	10.573
2	0.8746	23.052	21.646	24.276	7.966	3.950
2	0.8756	16.371	14.330	17.738	17.659	18.830
2	0.8766	16.345	14.117	17.609	17.545	18.671
2	0.8776	13.297	8.608	13.146	12.825	7.026
2	0.8786	11.336	5.576	13.473	11.699	8.947
2	0.8796	16.298	10.845	12.506	12.144	19.414
2	0.8806	18.695	14.940	18.841	10.205	12.104
2	0.8816	19.816	18.001	24.928	19.570	4.064
2	0.8826	16.757	10.799	16.772	11.534	11.376
2	0.8836	13.390	7.041	10.823	6.240	6.394
2	0.8846	19.090	13.850	14.504	8.649	5.625
2	0.8856	12.185	8.573	8.809	18.916	12.721
2	0.8866	17.080	8.310	10.115	10.929	14.911
2	0.8876	15.130	13.504	11.788	5.279	11.049
2	0.8886	12.558	12.664	10.705	4.125	19.026
2	0.8896	7.660	8.643	7.854	3.027	3.009
2	0.8906	22.124	26.097	22.385	11.786	5.785
2	0.8916	22.015	26.025	22.303	11.703	5.860
2	0.8926	14.544	14.180	12.716	17.698	9.719
2	0.8936	16.044	14.994	14.417	7.142	3.334
2	0.8946	11.922	10.428	11.285	12.295	2.640
2	0.8956	18.365	17.184	15.745	10.711	4.927
2	0.8966	13.783	15.897	12.380	14.367	5.514

2	0.8976	15.614	17.123	13.583	12.190	13.511
2	0.8986	24.255	31.333	19.132	20.641	9.804
2	0.8996	27.875	28.903	20.917	11.211	9.462
2	0.9006	5.848	9.953	4.630	9.050	14.045
2	0.9016	9.376	6.161	8.442	10.349	7.629
2	0.9026	10.867	6.352	11.229	9.655	6.689
2	0.9036	9.061	8.600	9.398	11.482	11.165
2	0.9046	11.579	13.810	10.254	8.746	8.314
2	0.9056	10.836	10.180	10.159	7.816	9.983
2	0.9066	7.254	10.146	10.716	6.167	5.376
2	0.9076	9.029	10.668	11.558	6.589	5.694
2	0.9086	10.402	9.415	6.895	8.590	8.207
2	0.9096	11.572	11.902	9.610	7.454	5.787
2	0.9106	12.196	17.097	13.241	5.819	4.668
2	0.9116	15.451	23.206	19.768	9.981	9.466
2	0.9126	15.262	15.936	16.630	5.648	7.436
2	0.9136	14.014	20.659	16.869	12.264	8.495
2	0.9146	23.958	19.489	20.653	16.052	7.043
2	0.9156	22.957	21.859	18.575	15.969	12.490
2	0.9166	31.723	22.472	27.274	15.229	11.131
2	0.9176	10.279	22.742	14.482	17.447	8.765
2	0.9186	17.540	17.017	13.614	22.317	16.612
2	0.9196	13.959	7.830	7.470	12.423	14.867
2	0.9206	20.489	26.878	19.964	20.106	12.904
2	0.9216	17.941	20.277	16.372	17.577	10.069
2	0.9226	6.803	11.763	5.657	9.183	8.667
2	0.9236	11.783	15.382	8.844	5.626	13.408
2	0.9246	16.127	18.605	16.296	13.431	12.667
2	0.9256	22.510	15.148	15.173	11.949	8.829
2	0.9266	11.829	9.286	7.842	13.102	10.917
2	0.9276	14.477	7.183	9.410	13.316	8.580
2	0.9286	22.089	13.257	16.567	7.941	10.573
2	0.9296	13.536	11.365	11.106	11.443	8.799
2	0.9306	5.576	7.663	7.472	15.494	14.933
2	0.9316	2.334	3.993	5.313	7.121	7.048
2	0.9326	3.720	9.289	10.120	7.466	7.738
2	0.9336	8.667	8.554	4.948	12.201	9.675
2	0.9346	23.408	19.919	15.763	14.581	13.775
2	0.9356	19.587	16.330	14.237	16.179	8.382
2	0.9366	17.094	7.954	14.346	3.267	6.360
2	0.9376	22.569	17.896	21.700	27.627	8.520
2	0.9386	25.592	13.121	21.802	24.467	13.806
2	0.9396	26.666	14.071	20.056	14.837	10.525
2	0.9406	14.594	15.065	10.363	18.096	14.733
2	0.9416	14.000	12.646	11.500	18.469	17.178
2	0.9426	21.965	16.145	19.915	15.259	10.758
2	0.9436	27.718	20.871	17.181	10.046	13.323
2	0.9446	26.723	18.344	19.605	12.555	13.909
2	0.9456	24.435	22.867	22.491	8.676	10.529
2	0.9466	18.720	17.063	15.423	10.078	9.457

2	0.9476	10.776	15.151	9.020	8.729	4.954
2	0.9486	9.242	11.472	6.758	14.773	6.279
2	0.9496	10.927	8.827	10.120	14.484	11.844
2	0.9506	12.617	6.922	9.294	9.179	3.405
2	0.9516	11.678	5.094	6.992	7.934	2.839
2	0.9526	20.992	10.656	14.564	13.953	8.214
2	0.9536	9.831	7.278	7.988	7.113	12.257
2	0.9546	8.553	10.132	7.943	12.793	8.294
2	0.9556	15.449	10.891	8.402	22.924	16.684
2	0.9566	10.661	9.132	8.794	8.822	17.379
2	0.9576	13.199	6.742	12.527	18.840	18.232
2	0.9586	17.769	5.084	14.711	10.912	12.794
2	0.9596	15.824	14.418	15.553	17.332	21.807
2	0.9606	15.803	8.882	13.381	20.064	11.818
2	0.9616	21.185	10.233	16.193	9.501	12.826
2	0.9626	14.740	8.475	11.554	12.235	7.327
2	0.9636	13.361	6.708	11.589	6.681	7.366
2	0.9646	5.810	6.332	7.649	13.529	17.176
2	0.9656	9.353	5.569	8.131	9.481	21.255
2	0.9666	3.988	3.690	3.913	10.693	12.665
2	0.9676	10.080	1.881	3.665	17.925	12.217
2	0.9686	9.194	6.751	4.982	12.069	16.615
2	0.9696	10.001	9.206	7.910	27.219	13.329
2	0.9706	7.419	7.363	7.530	15.950	8.820
2	0.9716	4.248	7.807	8.268	6.000	4.118
2	0.9726	12.251	8.975	13.827	18.585	5.328
2	0.9736	14.693	10.605	12.676	17.468	25.615
2	0.9746	12.694	9.928	8.779	22.933	14.745
2	0.9756	12.699	7.697	9.192	13.469	14.734
2	0.9766	15.974	9.823	12.763	13.395	14.145
2	0.9776	17.298	10.058	17.588	15.991	15.070
2	0.9786	11.814	8.490	8.107	13.433	28.564
2	0.9796	9.522	9.222	8.675	14.475	8.803
2	0.9806	12.761	9.596	9.289	11.684	10.088
2	0.9816	24.319	24.719	24.470	20.668	5.915
2	0.9826	24.258	24.751	24.486	20.660	5.960
2	0.9836	15.372	15.463	17.500	17.505	10.088
2	0.9846	15.915	8.327	13.049	20.731	13.648
2	0.9856	12.193	11.856	14.004	20.080	8.688
2	0.9866	15.869	9.105	18.575	23.815	14.168
2	0.9876	19.590	8.845	17.206	24.979	12.623
2	0.9886	12.284	13.492	9.684	29.439	18.486
2	0.9896	13.986	11.295	13.167	16.049	14.185
2	0.9906	17.632	16.010	16.411	7.952	17.862
2	0.9916	18.409	14.992	15.855	25.242	27.629
2	0.9926	15.820	14.062	14.772	23.312	20.877
2	0.9936	12.771	12.369	11.210	22.519	15.757
2	0.9946	20.465	14.296	22.746	16.804	25.587
2	0.9956	6.819	5.656	6.737	13.663	10.762
2	0.9966	10.054	7.100	11.740	18.153	5.545

2	0.9976	12.747	11.598	13.523	16.183	13.192
2	0.9986	10.458	11.022	8.617	18.059	21.910
2	0.9996	16.917	13.042	14.914	19.438	17.887
2	1.0006	15.813	8.515	11.585	10.655	17.674
2	1.0016	14.930	13.432	9.325	13.356	19.835
2	1.0026	2.294	3.360	3.605	14.724	12.064
2	1.0036	15.129	11.915	12.153	8.740	11.965
2	1.0046	15.973	13.288	14.279	13.023	18.264
2	1.0056	7.855	10.422	13.309	11.782	9.410
2	1.0066	4.277	5.946	5.634	9.644	11.119
2	1.0076	5.716	4.572	6.293	10.398	13.935
2	1.0086	8.249	6.777	6.378	23.456	14.152
2	1.0096	6.889	2.741	6.979	13.116	6.268
2	1.0106	10.014	3.733	5.845	15.187	15.740
2	1.0116	4.546	2.405	5.595	7.014	7.048
2	1.0126	13.871	17.795	16.329	16.242	22.676
2	1.0136	14.139	14.947	17.239	12.086	13.725
2	1.0146	14.656	19.612	14.970	11.974	9.987
2	1.0156	10.622	13.217	12.127	21.909	14.847
2	1.0166	8.896	9.403	17.135	19.792	16.122
2	1.0176	10.190	10.725	7.514	13.008	23.210
2	1.0186	15.765	19.782	16.043	28.312	14.850
2	1.0196	14.075	7.263	10.986	17.707	17.934
2	1.0206	6.492	7.206	8.697	10.660	13.397
2	1.0216	7.809	12.626	12.738	23.587	11.702
2	1.0226	17.215	13.779	18.900	12.357	12.520
2	1.0236	8.269	7.162	8.028	11.914	11.419
2	1.0246	10.184	4.629	8.137	25.684	12.722
2	1.0256	10.585	7.230	8.466	17.116	14.444
2	1.0266	14.898	4.791	13.576	14.215	11.801
2	1.0276	25.810	11.133	20.684	15.636	16.961
2	1.0286	25.709	10.131	20.952	21.183	16.694
2	1.0296	20.621	15.889	20.774	15.344	10.260
2	1.0306	8.153	8.777	9.601	16.332	5.795
2	1.0316	6.209	9.880	10.887	22.767	5.731
2	1.0326	11.207	10.313	9.449	22.340	8.218
2	1.0336	17.308	12.692	17.374	23.648	21.427
2	1.0346	9.980	12.090	10.109	24.267	12.607
2	1.0356	21.948	16.232	17.670	17.120	11.461
2	1.0366	16.083	13.284	10.737	16.448	10.162
2	1.0376	16.196	9.633	12.163	15.956	17.251
2	1.0386	11.576	4.123	9.910	8.255	10.097
2	1.0396	20.261	15.350	15.345	21.249	8.891
2	1.0406	12.335	14.307	10.530	37.399	16.938
2	1.0416	8.912	9.431	6.454	24.023	16.374
2	1.0426	16.686	7.849	12.463	24.143	14.329
2	1.0436	15.627	9.427	16.682	17.618	12.072
2	1.0446	21.260	16.581	16.206	7.109	8.381
2	1.0456	13.382	12.865	11.394	27.341	9.121
2	1.0466	16.786	12.659	19.242	27.395	8.013

2	1.0476	11.452	10.287	9.700	17.142	17.091
2	1.0486	11.217	15.293	9.669	16.232	23.059
2	1.0496	11.740	7.559	9.089	27.817	14.183
2	1.0506	14.400	16.367	16.492	13.488	8.199
2	1.0516	15.845	10.489	14.627	18.193	23.407
2	1.0526	16.220	18.300	17.121	20.305	6.759
2	1.0536	18.224	17.771	15.511	17.448	12.541
2	1.0546	10.845	12.126	9.435	16.531	7.759
2	1.0556	8.216	11.550	5.662	29.303	12.560
2	1.0566	8.636	10.543	6.355	9.781	7.962
2	1.0576	14.189	8.530	8.816	30.907	13.774
2	1.0586	9.780	11.113	6.557	18.462	4.712
2	1.0596	9.033	10.526	5.582	21.160	5.048
2	1.0606	8.402	11.822	7.242	28.288	12.049
2	1.0616	15.065	13.094	11.136	16.166	11.980
2	1.0626	11.822	14.066	10.763	20.041	5.877
2	1.0636	9.152	17.216	5.994	21.296	4.835
2	1.0646	7.078	6.520	6.760	5.063	3.904
2	1.0656	12.699	14.308	10.682	11.852	9.119
2	1.0666	16.737	11.173	11.203	24.241	12.901
2	1.0676	13.079	13.669	15.315	18.978	7.230
2	1.0686	8.433	13.424	8.574	19.034	12.875
2	1.0696	11.744	10.734	7.721	24.797	10.383
2	1.0706	11.612	13.353	7.389	25.281	25.701
2	1.0716	10.234	7.724	10.836	28.473	20.008
2	1.0726	18.396	17.905	17.233	21.953	17.897
2	1.0736	10.690	11.496	12.686	21.572	8.939
2	1.0746	17.886	18.808	22.039	25.604	9.950
2	1.0756	12.754	12.321	13.205	9.555	14.392
2	1.0766	8.473	9.994	6.574	11.262	4.880
2	1.0776	9.548	11.243	9.782	2.997	3.629
2	1.0786	11.973	8.608	11.712	12.492	8.796
2	1.0796	19.215	15.463	15.428	8.849	18.947
2	1.0806	19.953	14.881	17.817	12.699	4.559
2	1.0816	14.703	10.523	13.415	13.578	18.889
2	1.0826	19.299	12.663	16.526	11.419	23.803
2	1.0836	10.492	9.654	11.817	7.673	4.920
2	1.0846	8.216	7.323	9.969	3.911	3.376
2	1.0856	5.025	5.500	5.571	2.623	0.315
2	1.0866	19.929	12.165	19.802	2.628	9.665
2	1.0876	12.589	10.494	11.046	6.599	25.774
2	1.0886	18.320	18.135	15.293	23.258	24.535
2	1.0896	18.225	15.601	19.512	22.163	11.537
2	1.0906	16.776	12.169	15.100	21.019	7.099
2	1.0916	20.470	14.620	19.278	15.072	10.069
2	1.0926	16.539	11.706	15.991	30.239	27.003
2	1.0936	27.172	13.156	24.483	22.923	11.090
2	1.0946	13.625	8.361	15.699	20.570	6.889
2	1.0956	12.266	8.614	16.723	15.293	8.192
2	1.0966	14.971	7.845	15.582	16.894	6.303

2	1.0976	17.893	12.892	20.336	6.290	7.610
2	1.0986	16.419	16.804	16.738	20.597	9.383
2	1.0996	8.856	10.409	10.367	10.416	4.244
2	1.1006	17.888	14.022	18.004	13.704	5.931
2	1.1016	22.809	12.021	20.127	24.179	14.553
2	1.1026	20.812	15.059	20.034	21.955	26.170
2	1.1036	11.583	12.709	12.961	11.703	19.854
2	1.1046	16.806	16.337	16.871	11.991	14.549
2	1.1056	11.250	11.563	13.377	20.157	21.500
2	1.1066	12.779	11.771	13.711	27.037	22.667
2	1.1076	18.799	14.502	18.420	13.886	17.093
2	1.1086	21.942	19.202	18.768	13.935	27.342
2	1.1096	20.603	20.257	19.396	22.568	9.880
2	1.1106	28.065	26.569	29.343	18.164	7.887
2	1.1116	24.696	25.237	20.301	22.184	14.779
2	1.1126	24.856	22.055	23.345	14.210	12.111
2	1.1136	22.246	18.418	18.311	17.902	10.861
2	1.1146	21.902	15.865	14.725	12.480	11.382
2	1.1156	18.937	11.747	14.001	9.448	12.915
2	1.1166	13.310	13.118	11.385	10.530	8.571
2	1.1176	11.473	14.157	12.558	9.926	6.841
2	1.1186	15.232	20.536	15.261	14.322	13.940
2	1.1196	5.487	6.725	7.429	6.176	7.165
2	1.1206	5.348	5.268	6.218	7.675	4.444
2	1.1216	7.294	10.240	9.339	10.234	7.249
2	1.1226	12.626	14.142	13.530	11.151	16.806
2	1.1236	16.307	19.559	16.194	26.723	12.633
2	1.1246	14.781	17.755	17.174	6.570	13.001
2	1.1256	11.988	18.788	14.752	26.215	13.188
2	1.1266	14.149	12.415	10.927	17.932	17.974
2	1.1276	15.010	14.985	9.745	21.004	16.222
2	1.1286	17.330	12.861	15.532	18.278	9.749
2	1.1296	12.522	11.894	8.922	10.998	19.507
2	1.1306	10.439	10.419	8.213	9.278	8.147
2	1.1316	5.718	9.584	6.456	7.895	8.297
2	1.1326	12.956	22.395	17.165	18.783	20.004
2	1.1336	23.440	18.529	27.411	14.303	18.210
2	1.1346	16.038	10.962	11.927	11.024	13.373
2	1.1356	10.420	12.020	9.896	9.854	7.549
2	1.1366	21.875	21.299	18.961	19.087	13.852
2	1.1376	16.564	17.124	12.954	9.435	8.464
2	1.1386	16.484	15.664	10.094	14.433	13.816
2	1.1396	23.874	18.334	14.863	7.416	12.654
2	1.1406	15.854	12.247	9.947	10.475	24.489
2	1.1416	28.379	29.219	22.711	10.638	16.034
2	1.1426	23.769	16.330	16.702	10.917	7.221
2	1.1436	21.785	14.770	14.501	15.916	12.261
2	1.1446	27.577	32.086	24.645	13.392	10.291
2	1.1456	25.378	14.865	20.225	15.232	10.881
2	1.1466	25.390	14.864	20.251	15.243	10.873

2	1.1476	18.381	11.374	14.172	16.663	11.116
2	1.1486	28.124	18.446	21.535	15.710	12.138
2	1.1496	17.646	15.601	14.551	24.361	7.276
2	1.1506	21.296	18.760	16.735	6.742	3.810
2	1.1516	21.839	20.059	17.145	4.848	2.506
2	1.1526	25.181	24.051	19.736	9.975	16.951
2	1.1536	25.129	24.265	19.711	9.751	17.003
2	1.1546	12.329	8.573	6.763	11.684	8.391
2	1.1556	18.817	13.670	16.792	5.396	6.374
2	1.1566	20.573	11.024	15.947	20.600	10.875
2	1.1576	27.086	14.583	22.111	19.110	10.015
2	1.1586	24.029	20.096	22.441	12.518	8.319
2	1.1596	17.679	17.438	13.442	11.027	13.484
2	1.1606	27.823	22.366	22.578	16.602	8.841
2	1.1616	23.538	25.240	21.994	17.297	20.378
2	1.1626	22.150	18.937	23.112	10.906	11.095
2	1.1636	15.900	14.097	15.777	7.671	9.720
2	1.1646	20.784	16.698	16.879	9.008	12.821
2	1.1656	24.177	21.052	19.505	28.849	16.958
2	1.1666	24.353	17.782	22.440	22.125	12.476
2	1.1676	19.790	7.836	16.006	20.657	12.877
2	1.1686	22.706	9.868	14.908	11.272	13.027
2	1.1696	23.220	18.242	18.356	20.267	9.982
2	1.1706	17.099	13.865	9.831	13.824	8.322
2	1.1716	10.941	12.092	9.031	14.219	7.002
2	1.1726	6.532	3.074	6.784	11.158	11.256
2	1.1736	11.976	17.487	10.131	9.571	3.957
2	1.1746	15.003	21.290	12.775	12.958	7.950
2	1.1756	17.430	17.493	12.538	18.001	14.098
2	1.1766	32.688	32.548	27.281	25.967	22.164
2	1.1776	36.438	32.984	26.474	23.315	13.383
2	1.1786	28.722	13.652	24.120	12.249	12.180
2	1.1796	9.885	8.152	9.193	10.232	6.939
2	1.1806	17.494	16.662	13.039	6.883	9.838
2	1.1816	16.384	10.270	13.376	9.617	7.881
2	1.1826	15.641	16.414	14.178	4.051	3.399
2	1.1836	2.841	5.922	3.251	5.641	2.934
2	1.1846	13.242	14.418	10.745	10.094	13.035
2	1.1856	22.068	16.957	12.805	7.438	25.925
2	1.1866	31.007	15.208	19.110	17.928	18.126
2	1.1876	34.897	19.010	25.950	31.054	21.067
2	1.1886	20.518	15.711	17.014	15.864	16.155
2	1.1896	25.088	7.959	15.938	21.756	18.369
2	1.1906	34.959	29.796	31.504	14.698	21.726
2	1.1916	34.958	29.796	31.504	14.698	21.726
2	1.1926	11.528	10.190	8.587	7.054	7.781
2	1.1936	16.337	7.375	11.145	13.709	15.572
2	1.1946	19.115	15.421	15.715	5.924	16.615
2	1.1956	20.737	17.006	18.792	8.041	9.876
2	1.1966	30.485	15.701	20.848	7.415	17.597

2	1.1976	38.439	29.511	23.967	15.655	21.623
2	1.1986	13.306	8.785	4.855	7.245	17.373
2	1.1996	24.611	22.980	17.283	13.367	20.834
2	1.2006	29.540	23.643	21.112	11.311	20.725
2	1.2016	14.023	14.525	9.511	6.260	5.448
2	1.2026	12.664	9.630	7.406	12.099	10.459
2	1.2036	9.031	13.249	8.570	16.850	19.958
2	1.2046	9.142	9.966	6.388	4.977	14.483
2	1.2056	12.732	13.054	8.708	13.195	13.822
2	1.2066	17.469	15.870	11.353	18.496	19.084
2	1.2076	17.461	15.904	11.353	18.512	19.209
2	1.2086	23.550	16.099	12.786	18.831	26.575
2	1.2096	15.475	11.344	8.771	20.812	23.191
2	1.2106	13.001	7.798	8.003	20.761	17.087
2	1.2116	17.934	18.648	16.392	13.263	16.436
2	1.2126	12.884	11.597	12.718	12.699	26.235
2	1.2136	15.374	16.083	14.652	12.132	22.118
2	1.2146	22.656	14.500	13.749	14.116	19.921
2	1.2156	18.886	16.097	14.884	13.878	14.956
2	1.2166	22.582	14.500	19.293	9.005	18.703
2	1.2176	11.388	9.846	11.458	5.172	1.689
2	1.2186	32.693	19.880	22.710	14.683	15.003
2	1.2196	29.163	22.880	27.440	24.580	12.420
2	1.2206	21.211	16.721	18.350	18.071	21.749
2	1.2216	18.075	16.931	19.962	12.027	18.595
2	1.2226	17.836	16.695	19.729	12.131	18.582
2	1.2236	23.957	15.205	16.704	22.708	23.830
2	1.2246	35.671	21.125	26.771	13.276	16.017
2	1.2256	22.463	19.823	15.287	15.457	22.220
2	1.2266	21.181	13.730	18.844	5.587	12.138
2	1.2276	23.322	17.685	20.657	4.304	24.433
2	1.2286	13.730	11.109	7.322	6.455	14.146
2	1.2296	15.514	16.346	12.573	13.092	15.506
2	1.2306	31.655	13.494	15.339	3.741	17.957
2	1.2316	29.785	20.653	16.825	15.724	20.755
2	1.2326	19.321	22.419	22.137	12.408	14.922
2	1.2336	17.738	14.157	18.154	4.571	16.137
2	1.2346	19.481	15.281	18.312	8.774	10.569
2	1.2356	17.983	9.958	15.074	13.866	16.060
2	1.2366	5.955	5.574	7.995	12.866	9.042
2	1.2376	23.879	15.945	15.789	18.800	23.235
2	1.2386	28.491	15.702	20.869	12.015	15.126
2	1.2396	35.791	23.749	30.430	23.579	30.004
2	1.2406	27.294	22.103	25.001	17.824	26.036
2	1.2416	44.954	22.278	33.366	12.681	20.776
2	1.2426	32.450	26.483	27.860	18.471	22.444
2	1.2436	34.748	20.899	28.176	18.678	17.089
2	1.2446	17.848	13.164	15.303	11.926	11.270
2	1.2456	31.806	15.603	22.781	15.255	13.360
2	1.2466	35.123	21.568	23.042	15.105	18.334

2	1.2476	12.587	11.795	14.015	13.585	21.787
2	1.2486	15.646	11.969	10.985	21.200	20.215
2	1.2496	8.274	13.231	11.581	17.446	13.059
2	1.2506	10.675	15.436	13.971	8.222	16.119
2	1.2516	12.148	17.286	20.570	12.285	19.922
2	1.2526	16.941	15.591	21.353	11.824	21.701
2	1.2536	14.428	12.587	17.024	8.898	24.350
2	1.2546	17.573	21.216	21.144	19.676	13.018
2	1.2556	20.581	23.968	20.636	22.120	18.490
2	1.2566	12.801	11.680	17.887	6.589	9.784
2	1.2576	12.393	12.140	18.348	20.915	18.720
2	1.2586	16.794	12.462	20.465	11.746	10.512
2	1.2596	14.412	13.025	14.711	9.880	19.191
2	1.2606	37.309	23.291	32.370	20.452	28.096
2	1.2616	25.013	17.152	20.277	16.268	30.861
2	1.2626	27.435	14.278	23.352	9.192	19.620
2	1.2636	26.883	24.998	21.413	13.072	17.318
2	1.2646	18.009	21.773	22.001	15.526	14.243
2	1.2656	13.113	15.999	22.884	19.624	14.858
2	1.2666	18.822	22.132	26.627	20.142	19.644
2	1.2676	17.944	17.467	23.362	23.812	28.345
2	1.2686	18.327	13.696	19.305	16.059	20.680
2	1.2696	10.672	9.010	10.701	20.461	27.923
2	1.2706	15.486	14.095	11.762	12.077	21.235
2	1.2716	11.645	8.821	13.819	16.642	21.172
2	1.2726	14.609	14.418	18.047	5.447	11.532
2	1.2736	15.689	13.175	15.995	10.149	19.635
2	1.2746	22.032	11.971	17.957	10.230	11.085
2	1.2756	7.947	13.492	12.570	16.393	17.087
2	1.2766	9.253	13.079	13.213	23.317	23.853
2	1.2776	5.999	9.000	13.009	15.560	26.368
2	1.2786	12.502	12.273	11.932	17.096	18.636
2	1.2796	14.190	13.139	18.435	31.449	15.259
2	1.2806	14.204	11.126	19.053	5.350	8.696
2	1.2816	10.188	7.636	16.462	9.044	9.370
2	1.2826	27.164	20.630	23.428	10.345	23.340
2	1.2836	24.352	17.811	21.784	19.165	23.364
2	1.2846	20.244	23.862	21.109	14.978	27.780
2	1.2856	15.403	12.731	17.836	10.839	20.448
2	1.2866	9.479	8.758	11.646	8.535	26.655
2	1.2876	9.200	6.817	10.878	15.173	31.349
2	1.2886	9.505	8.874	14.617	4.281	14.458
2	1.2896	12.216	7.409	12.526	9.995	18.496
2	1.2906	11.437	3.846	8.348	7.957	16.566
2	1.2916	16.562	10.213	20.338	7.190	17.174
2	1.2926	14.198	11.010	16.217	13.001	16.780
2	1.2936	6.998	7.783	13.373	7.040	11.046
2	1.2946	15.878	13.044	15.480	12.290	25.275
2	1.2956	10.522	11.660	16.487	16.133	22.362
2	1.2966	13.255	12.931	16.175	10.440	7.699

2	1.2976	13.718	14.211	21.295	14.922	15.132
2	1.2986	26.608	12.334	27.186	15.175	21.047
2	1.2996	22.769	12.806	24.463	27.166	34.498
2	1.3006	16.541	6.721	13.783	14.538	15.659
2	1.3016	18.530	14.970	24.872	17.577	10.094
2	1.3026	22.008	22.668	30.353	9.374	17.495
2	1.3036	17.210	16.875	23.594	14.737	15.034
2	1.3046	29.692	18.031	23.106	12.077	16.712
2	1.3056	11.155	9.583	18.354	6.881	21.809
2	1.3066	24.851	19.245	19.717	11.986	26.585
2	1.3076	24.800	26.372	24.684	22.026	32.923
2	1.3086	17.358	17.869	17.523	15.778	14.579
2	1.3096	9.367	6.941	9.113	14.930	20.555
2	1.3106	17.776	11.007	16.036	22.359	22.932
2	1.3116	11.278	12.018	9.184	11.415	23.706
2	1.3126	32.965	14.545	15.275	7.330	22.121
2	1.3136	14.399	20.501	13.996	18.583	27.750
2	1.3146	16.169	24.693	21.273	31.208	33.488
2	1.3156	11.915	5.976	13.202	28.540	21.463
2	1.3166	6.159	4.281	5.998	10.088	16.232
2	1.3176	14.552	11.390	16.984	13.056	17.541
2	1.3186	26.557	12.095	19.956	18.226	30.161
2	1.3196	23.212	7.863	14.451	24.579	24.377
2	1.3206	22.994	7.818	14.065	24.274	24.488
2	1.3216	16.952	10.099	13.198	15.698	24.340
2	1.3226	12.994	9.042	11.023	12.610	18.833
2	1.3236	15.605	11.630	18.066	12.611	15.410
2	1.3246	18.360	5.338	12.147	15.197	12.908
2	1.3256	9.615	5.687	8.324	12.519	26.347
2	1.3266	6.315	2.944	4.129	2.743	9.197
2	1.3276	9.353	11.292	12.880	2.914	6.170
2	1.3286	17.462	9.451	17.130	7.957	24.806
2	1.3296	14.451	13.116	19.813	7.746	25.734
2	1.3306	26.741	19.607	29.417	11.768	16.491
2	1.3316	20.098	13.088	24.249	15.232	22.522
2	1.3326	9.922	11.567	13.719	9.966	13.597
2	1.3336	16.458	11.642	16.408	14.910	23.493
2	1.3346	20.323	18.821	14.717	14.967	21.665
2	1.3356	24.930	20.740	18.968	9.092	16.152
2	1.3366	19.839	11.768	16.446	17.702	17.139
2	1.3376	19.466	21.206	19.632	18.701	18.769
2	1.3386	23.787	20.874	21.388	13.531	22.759
2	1.3396	12.491	16.338	17.630	8.506	13.775
2	1.3406	17.717	20.902	23.031	6.578	11.248
2	1.3416	11.322	11.685	18.324	7.018	15.954
2	1.3426	8.742	16.761	13.660	10.374	13.665
2	1.3436	8.736	16.667	13.675	10.166	13.644
2	1.3446	5.579	11.233	9.330	13.136	14.914
2	1.3456	9.696	11.083	11.642	7.427	16.341
2	1.3466	9.008	12.437	10.194	7.968	14.468

2	1.3476	10.136	11.335	11.084	7.881	17.112
2	1.3486	13.050	10.397	15.177	14.186	19.741
2	1.3496	9.668	4.831	6.672	16.334	21.099
2	1.3506	9.648	4.829	6.649	16.333	21.102
2	1.3516	11.829	12.504	14.048	15.868	26.221
2	1.3526	10.217	9.085	14.655	12.689	23.062
2	1.3536	17.325	14.619	23.719	15.847	32.671
3	0.0007	0.000	0.000	0.000	0.000	0.000
3	0.0017	13.373	7.361	9.464	11.913	22.983
3	0.0027	17.074	13.047	13.569	14.453	19.961
3	0.0037	10.468	13.685	12.540	8.290	20.502
3	0.0047	14.976	11.958	10.368	21.040	23.669
3	0.0057	15.091	18.655	15.773	23.629	22.690
3	0.0067	8.780	14.163	14.765	17.536	16.009
3	0.0077	16.795	17.298	19.023	10.671	23.792
3	0.0087	11.177	14.183	16.684	12.181	19.217
3	0.0097	21.501	23.144	18.506	14.874	22.061
3	0.0107	14.396	13.089	17.994	15.706	11.734
3	0.0117	11.789	6.144	10.744	7.087	14.010
3	0.0127	8.193	12.859	12.308	9.663	12.941
3	0.0137	8.790	10.341	13.413	12.943	19.537
3	0.0147	7.351	14.303	10.349	20.909	12.292
3	0.0157	14.045	17.121	10.952	19.368	13.425
3	0.0167	12.797	11.598	14.707	9.088	13.636
3	0.0177	10.695	9.374	12.508	12.896	16.874
3	0.0187	13.119	16.328	20.148	10.714	17.714
3	0.0197	11.267	6.902	12.780	5.675	17.764
3	0.0207	11.430	11.568	11.847	10.496	7.152
3	0.0217	9.589	20.625	14.470	16.095	12.924
3	0.0227	12.937	17.643	12.935	16.607	16.582
3	0.0237	12.135	18.329	13.084	12.024	20.701
3	0.0247	16.200	19.156	18.645	14.485	18.502
3	0.0257	15.700	18.750	19.816	15.812	11.378
3	0.0267	13.176	16.766	15.707	13.133	24.694
3	0.0277	14.253	10.941	18.588	7.982	15.880
3	0.0287	12.319	6.287	11.514	4.374	19.024
3	0.0297	10.761	13.920	11.385	18.920	13.304
3	0.0307	13.604	16.094	17.465	16.159	21.776
3	0.0317	15.400	12.597	16.380	11.889	18.074
3	0.0327	15.515	12.369	16.328	11.688	17.745
3	0.0337	11.343	7.645	5.483	13.321	15.280
3	0.0347	12.035	16.707	15.625	25.216	13.581
3	0.0357	13.374	12.656	11.269	16.843	13.100
3	0.0367	14.191	25.467	17.303	20.687	11.865
3	0.0377	20.199	26.496	26.032	14.239	11.059
3	0.0387	16.753	23.566	23.433	16.665	13.727
3	0.0397	6.777	10.268	10.243	17.062	7.275
3	0.0407	11.279	15.520	12.359	16.797	8.892
3	0.0417	13.153	18.993	16.316	13.522	6.771
3	0.0427	20.071	26.169	15.959	11.690	15.202

3	0.0437	13.454	18.049	16.448	11.748	9.092
3	0.0447	20.201	27.352	25.970	15.528	16.742
3	0.0457	12.507	11.399	10.894	10.783	14.002
3	0.0467	17.544	16.941	17.616	14.914	19.202
3	0.0477	14.990	15.887	19.518	25.272	18.650
3	0.0487	17.292	14.896	19.069	17.942	8.745
3	0.0497	9.895	7.818	7.767	20.627	5.279
3	0.0507	9.695	5.833	7.477	10.570	8.970
3	0.0517	10.991	6.810	8.486	11.740	11.952
3	0.0527	15.972	15.391	10.057	15.519	15.106
3	0.0537	18.205	26.379	15.205	23.311	12.996
3	0.0547	13.580	15.943	11.953	14.761	7.814
3	0.0557	33.424	16.353	20.981	12.463	10.385
3	0.0567	33.497	16.439	21.036	12.466	10.479
3	0.0577	25.124	22.459	22.636	17.344	12.924
3	0.0587	15.854	10.382	12.477	11.802	15.183
3	0.0597	15.609	18.059	12.888	30.117	15.026
3	0.0607	23.658	19.266	19.243	23.798	7.098
3	0.0617	31.142	21.822	21.499	32.604	19.221
3	0.0627	12.607	12.931	14.416	14.945	14.134
3	0.0637	10.587	14.880	13.383	14.277	13.491
3	0.0647	13.560	21.031	17.797	17.862	9.917
3	0.0657	30.385	15.721	25.258	15.142	15.609
3	0.0667	17.544	13.484	19.133	14.390	14.850
3	0.0677	11.684	12.091	15.122	16.469	24.716
3	0.0687	20.574	21.886	25.516	13.137	10.492
3	0.0697	17.299	24.104	25.773	23.744	18.037
3	0.0707	22.051	18.435	18.872	27.870	10.253
3	0.0717	27.138	19.025	28.219	18.911	8.129
3	0.0727	19.623	16.034	19.336	21.690	8.929
3	0.0737	24.883	19.669	14.180	12.539	17.069
3	0.0747	16.873	10.579	5.745	22.153	22.183
3	0.0757	39.179	22.915	23.331	27.191	25.908
3	0.0767	12.516	10.677	11.514	8.857	8.818
3	0.0777	10.392	24.007	11.713	18.979	8.947
3	0.0787	17.580	23.434	20.462	8.883	8.827
3	0.0797	23.415	25.788	31.361	21.064	18.113
3	0.0807	17.830	19.584	13.844	12.706	20.848
3	0.0817	31.737	31.562	25.936	5.610	16.323
3	0.0827	28.570	25.033	22.181	17.849	12.493
3	0.0837	24.847	28.962	28.552	18.293	11.864
3	0.0847	24.966	28.974	28.780	18.100	12.182
3	0.0857	17.799	24.610	19.788	21.342	21.321
3	0.0867	17.917	24.616	19.868	21.257	21.392
3	0.0877	13.684	14.678	14.328	10.621	18.631
3	0.0887	17.270	19.671	19.119	9.523	16.641
3	0.0897	24.847	21.880	25.810	12.503	10.205
3	0.0907	24.859	30.286	26.710	11.682	9.309
3	0.0917	23.087	31.462	22.922	12.314	15.236
3	0.0927	18.831	26.047	21.266	16.056	12.069

3	0.0937	18.199	24.896	24.013	15.032	17.240
3	0.0947	20.956	19.444	16.017	15.336	12.871
3	0.0957	13.498	29.169	16.323	25.950	21.986
3	0.0967	9.774	13.186	8.345	24.734	31.286
3	0.0977	9.773	13.205	8.333	24.716	31.295
3	0.0987	35.598	13.702	14.181	13.632	21.603
3	0.0997	37.506	16.154	17.132	17.304	22.364
3	0.1007	32.195	15.209	15.446	8.003	21.900
3	0.1017	27.724	12.526	10.815	18.119	13.366
3	0.1027	9.571	10.009	9.913	12.470	11.610
3	0.1037	29.804	10.969	18.605	15.802	17.529
3	0.1047	12.417	19.098	14.886	9.779	4.547
3	0.1057	12.531	10.950	6.947	16.299	18.498
3	0.1067	28.346	23.258	22.300	10.209	18.105
3	0.1077	20.632	18.426	17.043	10.136	25.475
3	0.1087	20.644	18.390	16.978	10.083	25.415
3	0.1097	7.858	10.333	10.345	8.522	18.459
3	0.1107	24.540	14.089	13.130	22.094	11.455
3	0.1117	20.781	25.446	18.866	19.768	9.118
3	0.1127	19.718	24.052	16.539	20.693	11.565
3	0.1137	29.204	18.312	22.242	11.572	15.919
3	0.1147	15.681	14.051	15.669	13.596	18.600
3	0.1157	18.641	17.433	17.179	7.719	21.259
3	0.1167	23.381	22.030	16.651	9.216	23.091
3	0.1177	23.348	22.001	16.695	8.977	23.173
3	0.1187	16.497	14.150	13.020	17.090	28.864
3	0.1197	31.292	18.489	13.306	25.392	21.824
3	0.1207	19.555	9.189	10.582	14.611	20.525
3	0.1217	26.741	23.411	19.294	25.229	19.883
3	0.1227	26.236	13.848	16.442	18.897	18.529
3	0.1237	26.149	13.846	16.429	18.940	18.409
3	0.1247	31.465	26.126	26.969	21.828	15.361
3	0.1257	28.310	11.473	19.061	12.687	16.013
3	0.1267	14.227	16.993	8.899	27.253	13.276
3	0.1277	12.290	13.534	15.492	6.057	14.694
3	0.1287	8.517	14.868	12.072	14.348	17.125
3	0.1297	8.567	15.002	12.152	14.460	16.977
3	0.1307	15.722	14.537	21.125	14.421	19.452
3	0.1317	9.444	10.723	13.984	16.657	18.316
3	0.1327	26.860	17.767	18.932	7.534	16.858
3	0.1337	8.748	9.522	7.589	8.087	24.979
3	0.1347	6.448	6.651	8.434	6.601	29.384
3	0.1357	29.216	12.963	19.034	16.280	14.462
3	0.1367	29.223	12.949	19.030	16.254	14.446
3	0.1377	8.514	8.948	13.016	7.723	16.268
3	0.1387	18.409	22.197	28.212	12.575	18.877
3	0.1397	11.054	13.188	15.753	7.887	13.421
3	0.1407	13.190	11.260	15.952	7.862	14.891
3	0.1417	3.858	8.123	8.780	6.816	6.048
3	0.1427	3.399	7.060	7.643	4.789	4.941

3	0.1437	3.445	7.118	7.715	4.919	4.852
3	0.1447	35.311	23.801	24.442	6.303	13.729
3	0.1457	28.176	18.373	16.280	8.635	11.432
3	0.1467	21.199	12.621	15.530	9.146	15.146
3	0.1477	12.813	11.643	10.057	17.372	20.426
3	0.1487	11.767	14.615	13.116	12.073	16.311
3	0.1497	23.639	9.037	18.632	10.032	16.379
3	0.1507	12.778	11.595	12.456	24.549	14.357
3	0.1517	8.132	12.955	11.061	14.331	17.187
3	0.1527	1.393	2.758	2.474	5.177	7.352
3	0.1537	4.740	3.195	5.296	7.441	8.179
3	0.1547	17.655	8.844	17.807	13.283	12.205
3	0.1557	19.211	13.896	17.791	7.052	7.140
3	0.1567	13.207	13.404	14.592	12.119	10.474
3	0.1577	10.186	8.439	12.054	11.331	31.364
3	0.1587	10.186	8.470	12.064	11.248	31.315
3	0.1597	19.715	7.831	18.303	18.057	29.900
3	0.1607	18.278	13.352	12.598	31.709	26.837
3	0.1617	12.765	12.440	17.913	12.429	20.451
3	0.1627	25.718	16.313	22.609	24.004	36.872
3	0.1637	21.166	18.726	15.625	15.177	24.830
3	0.1647	10.243	13.812	10.927	15.721	19.503
3	0.1657	15.046	7.865	10.517	13.222	21.523
3	0.1667	17.918	13.368	13.013	7.594	20.763
3	0.1677	8.843	4.171	7.768	5.713	13.492
3	0.1687	16.986	10.023	12.661	9.951	23.290
3	0.1697	20.319	10.869	13.843	4.885	17.023
3	0.1707	8.932	3.983	3.837	16.760	28.530
3	0.1717	13.245	7.947	13.156	10.671	23.145
3	0.1727	11.415	9.585	13.061	13.193	26.311
3	0.1737	3.751	6.357	7.530	6.376	23.300
3	0.1747	13.194	10.000	10.241	17.265	30.023
3	0.1757	3.489	5.951	6.576	7.473	12.098
3	0.1767	5.640	8.547	6.495	10.499	11.779
3	0.1777	13.277	8.574	10.922	11.173	13.890
3	0.1787	6.788	3.370	6.530	16.406	16.057
3	0.1797	15.221	10.108	11.452	11.913	18.731
3	0.1807	7.226	7.449	8.668	10.859	11.047
3	0.1817	7.736	10.146	14.304	7.205	10.212
3	0.1827	7.736	10.146	14.309	7.205	10.152
3	0.1837	10.406	6.342	13.187	9.889	13.445
3	0.1847	15.237	18.540	17.477	9.566	19.450
3	0.1857	7.267	7.555	10.193	13.563	14.207
3	0.1867	9.902	8.704	10.261	8.160	8.104
3	0.1877	8.675	11.882	10.332	11.115	24.061
3	0.1887	24.914	20.595	23.574	6.496	20.733
3	0.1897	18.246	14.661	20.948	15.825	15.399
3	0.1907	10.330	12.066	13.364	29.109	25.657
3	0.1917	8.617	6.388	7.902	16.541	15.227
3	0.1927	19.235	19.871	19.440	12.566	17.838

3	0.1937	6.659	6.191	7.639	17.137	13.348
3	0.1947	12.473	2.325	6.741	16.722	11.640
3	0.1957	7.697	3.369	8.080	9.483	8.512
3	0.1967	12.127	5.894	14.404	15.663	16.892
3	0.1977	9.945	9.359	10.513	14.831	21.254
3	0.1987	11.185	11.504	12.496	12.394	9.263
3	0.1997	11.510	9.455	13.893	10.410	10.465
3	0.2007	14.685	18.994	22.293	22.387	19.943
3	0.2017	22.236	23.023	20.108	18.384	21.376
3	0.2027	12.394	14.288	15.696	11.029	18.522
3	0.2037	17.013	7.198	7.098	7.929	20.106
3	0.2047	18.282	18.448	17.421	11.463	23.218
3	0.2057	7.790	11.439	9.196	12.723	16.553
3	0.2067	8.017	10.385	8.766	6.355	17.904
3	0.2077	15.077	18.553	13.539	21.802	19.545
3	0.2087	11.991	21.037	13.477	15.597	9.888
3	0.2097	14.697	13.327	15.599	10.019	16.090
3	0.2107	10.491	12.595	11.504	8.835	3.793
3	0.2117	12.819	17.611	12.638	12.754	20.809
3	0.2127	8.007	12.021	7.784	7.132	15.904
3	0.2137	10.164	15.370	10.805	16.156	13.153
3	0.2147	7.405	14.682	11.780	20.823	15.624
3	0.2157	13.917	20.166	12.177	24.826	21.978
3	0.2167	16.921	20.918	21.532	14.766	12.996
3	0.2177	12.694	18.900	16.409	9.373	14.431
3	0.2187	10.755	13.202	8.554	12.664	10.683
3	0.2197	18.799	14.153	14.158	15.914	26.955
3	0.2207	13.582	19.825	19.936	13.006	20.543
3	0.2217	12.973	9.071	10.492	10.107	13.690
3	0.2227	16.536	12.565	16.050	10.782	18.044
3	0.2237	9.631	11.257	6.812	26.045	20.366
3	0.2247	16.034	11.098	11.326	21.885	18.011
3	0.2257	17.098	19.767	19.667	12.916	11.681
3	0.2267	2.338	2.479	4.032	6.241	8.856
3	0.2277	7.845	5.049	8.368	12.250	24.458
3	0.2287	22.747	7.203	8.299	17.792	16.095
3	0.2297	11.884	11.720	7.516	12.053	23.269
3	0.2307	11.887	11.717	7.505	12.052	23.271
3	0.2317	11.431	9.762	7.252	6.638	18.513
3	0.2327	13.014	10.351	12.289	19.113	20.315
3	0.2337	19.552	11.056	11.989	9.064	17.330
3	0.2347	17.374	11.486	11.381	8.880	18.471
3	0.2357	10.317	11.336	11.278	17.202	10.666
3	0.2367	2.409	2.651	2.081	14.350	12.348
3	0.2377	5.880	5.291	5.305	4.699	8.897
3	0.2387	12.656	15.168	11.894	11.026	18.473
3	0.2397	8.668	12.156	9.054	7.664	11.116
3	0.2407	10.176	9.486	8.018	13.998	12.665
3	0.2417	5.447	2.713	5.165	2.910	7.886
3	0.2427	6.421	4.483	5.256	8.487	12.196

3	0.2437	17.317	8.797	10.253	12.453	10.175
3	0.2447	13.490	8.797	11.061	9.689	19.261
3	0.2457	14.836	7.528	12.788	10.088	14.818
3	0.2467	9.262	4.518	6.308	8.047	13.541
3	0.2477	8.366	7.600	5.207	22.376	11.255
3	0.2487	21.168	18.500	10.348	27.024	29.587
3	0.2497	17.147	8.744	8.813	17.560	18.811
3	0.2507	17.499	7.538	12.640	17.782	22.508
3	0.2517	14.796	10.642	15.057	23.670	15.730
3	0.2527	14.243	8.826	14.698	16.434	11.683
3	0.2537	18.078	17.749	18.674	15.416	13.512
3	0.2547	20.119	11.994	15.018	22.197	33.735
3	0.2557	16.538	19.488	17.890	13.328	24.718
3	0.2567	16.914	18.959	14.473	21.294	29.479
3	0.2577	11.445	9.755	10.356	16.576	14.860
3	0.2587	18.783	15.649	18.348	17.005	22.616
3	0.2597	43.522	14.053	15.444	19.677	28.285
3	0.2607	17.807	11.623	12.609	13.870	8.682
3	0.2617	5.219	8.390	5.273	20.554	19.951
3	0.2627	5.531	1.560	4.619	3.437	0.898
3	0.2637	9.103	7.266	14.775	2.231	10.168
3	0.2647	8.647	7.741	15.203	3.035	10.853
3	0.2657	8.643	7.744	15.204	3.083	10.861
3	0.2667	5.767	2.606	3.649	4.317	7.567
3	0.2677	3.507	1.296	2.705	4.049	5.556
3	0.2687	5.202	6.183	8.459	6.612	12.273
3	0.2697	13.951	13.719	15.994	7.022	14.933
3	0.2707	13.988	13.551	15.821	7.214	14.976
3	0.2717	20.546	28.745	26.272	11.641	20.852
3	0.2727	9.188	11.296	16.350	22.530	28.821
3	0.2737	7.089	7.143	10.593	7.173	20.044
3	0.2747	8.716	5.065	7.489	9.772	13.336
3	0.2757	16.947	17.393	11.372	17.725	15.957
3	0.2767	16.094	17.287	11.239	19.864	8.752
3	0.2777	9.779	9.132	7.767	16.123	13.669
3	0.2787	18.579	12.763	12.460	24.381	22.364
3	0.2797	14.517	9.482	11.387	16.078	23.328
3	0.2807	16.331	9.430	11.201	15.737	22.098
3	0.2817	12.766	13.795	10.152	28.687	15.023
3	0.2827	13.001	9.322	9.353	16.420	21.222
3	0.2837	14.979	8.011	10.781	18.396	12.984
3	0.2847	22.543	15.029	21.382	18.217	18.737
3	0.2857	11.264	8.602	11.021	16.765	14.229
3	0.2867	5.873	4.932	4.820	21.419	13.617
3	0.2877	7.069	5.194	8.534	22.922	21.950
3	0.2887	10.808	12.034	12.113	25.050	18.533
3	0.2897	17.838	11.854	17.226	13.958	14.920
3	0.2907	11.487	6.325	2.605	19.143	24.816
3	0.2917	11.286	13.300	8.536	26.856	22.929
3	0.2927	6.867	9.582	8.195	27.758	11.418

3	0.2937	8.785	8.635	5.096	15.050	12.050
3	0.2947	9.164	8.622	8.392	16.471	17.149
3	0.2957	11.981	3.608	6.163	18.259	18.287
3	0.2967	8.381	8.413	8.788	17.720	25.836
3	0.2977	9.660	9.851	7.751	8.758	24.328
3	0.2987	7.787	5.459	4.854	11.121	21.099
3	0.2997	16.340	7.625	12.274	20.160	23.228
3	0.3007	9.314	6.934	8.195	21.283	20.778
3	0.3017	8.390	7.147	5.155	16.218	13.966
3	0.3027	8.126	5.282	6.388	18.262	19.125
3	0.3037	8.940	6.622	9.738	14.026	8.518
3	0.3047	10.261	7.177	7.842	13.417	26.346
3	0.3057	10.455	8.862	11.541	23.424	25.140
3	0.3067	8.371	4.931	6.655	24.881	29.283
3	0.3077	11.716	10.261	10.020	22.836	31.748
3	0.3087	6.923	10.391	12.312	11.740	16.340
3	0.3097	15.701	9.352	11.787	26.693	24.695
3	0.3107	11.071	7.337	6.500	16.313	8.810
3	0.3117	21.934	12.188	14.414	11.124	25.711
3	0.3127	19.495	9.522	8.776	15.165	31.721
3	0.3137	10.427	14.283	12.555	7.586	21.256
3	0.3147	12.972	17.147	15.380	17.716	28.212
3	0.3157	10.061	10.450	7.176	17.286	20.037
3	0.3167	10.111	13.865	7.730	17.308	17.421
3	0.3177	13.151	7.788	7.458	13.338	17.537
3	0.3187	12.397	6.930	7.467	13.747	14.884
3	0.3197	10.316	8.005	8.132	5.972	15.811
3	0.3207	10.051	13.452	11.102	12.231	22.866
3	0.3217	7.853	6.627	4.983	18.776	22.314
3	0.3227	10.164	7.387	6.161	19.026	16.786
3	0.3237	6.991	7.669	6.060	21.313	20.584
3	0.3247	12.106	11.996	9.297	24.125	18.297
3	0.3257	9.326	13.985	9.782	27.665	14.729
3	0.3267	11.334	10.619	11.177	12.150	17.967
3	0.3277	7.533	6.772	6.218	6.904	9.393
3	0.3287	10.074	10.615	10.076	6.134	13.494
3	0.3297	11.756	9.313	11.653	4.336	13.871
3	0.3307	7.788	7.894	9.369	8.764	11.086
3	0.3317	16.359	14.862	12.916	18.136	23.781
3	0.3327	7.172	13.264	10.312	16.616	20.439
3	0.3337	16.146	14.022	9.660	16.942	20.611
3	0.3347	9.386	15.103	10.891	16.090	14.443
3	0.3357	7.612	7.108	6.278	9.019	12.427
3	0.3367	13.431	10.441	10.501	16.562	12.879
3	0.3377	19.529	11.016	14.667	24.165	18.739
3	0.3387	13.610	13.570	15.021	14.202	10.206
3	0.3397	19.180	11.844	16.084	17.860	13.011
3	0.3407	13.880	7.291	9.302	19.618	16.394
3	0.3417	19.585	17.784	17.750	22.852	19.745
3	0.3427	13.916	9.733	7.293	25.453	25.669

3	0.3437	11.206	9.031	8.914	18.416	15.960
3	0.3447	8.440	7.590	11.675	11.070	11.947
3	0.3457	8.918	12.933	10.978	17.176	13.774
3	0.3467	11.166	7.497	7.815	15.651	14.861
3	0.3477	12.710	13.359	10.129	17.754	10.276
3	0.3487	10.589	8.241	7.734	15.265	17.272
3	0.3497	12.492	12.309	9.623	17.193	13.246
3	0.3507	15.426	11.835	14.409	19.048	23.768
3	0.3517	13.492	18.625	13.767	23.114	27.785
3	0.3527	22.112	19.136	17.277	15.802	30.856
3	0.3537	31.686	21.641	17.656	17.824	21.177
3	0.3547	15.999	9.687	9.712	14.544	24.290
3	0.3557	12.549	9.872	4.798	18.786	27.865
3	0.3567	8.527	4.249	5.040	16.468	25.418
3	0.3577	9.637	4.336	4.776	17.402	36.551
3	0.3587	13.086	15.217	9.906	26.455	46.229
3	0.3597	7.486	15.320	8.961	21.421	23.229
3	0.3607	8.745	10.644	12.998	17.343	13.157
3	0.3617	13.226	10.186	12.511	18.808	10.326
3	0.3627	9.849	12.884	7.490	14.505	4.837
3	0.3637	7.346	6.203	5.277	12.634	14.868
3	0.3647	18.419	19.392	16.619	25.179	29.890
3	0.3657	10.985	4.173	5.331	16.118	19.599
3	0.3667	8.957	1.626	4.984	8.795	5.382
3	0.3677	15.724	8.042	9.610	21.447	14.602
3	0.3687	20.428	14.846	15.525	10.344	7.871
3	0.3697	19.492	12.526	14.996	13.145	18.476
3	0.3707	29.677	26.074	17.799	22.056	25.173
3	0.3717	29.667	26.066	17.797	22.056	25.168
3	0.3727	32.717	36.115	33.398	23.992	23.156
3	0.3737	15.342	14.496	9.460	14.651	14.395
3	0.3747	15.253	12.367	6.398	11.222	15.328
3	0.3757	17.332	11.133	11.554	24.957	18.835
3	0.3767	12.914	8.420	8.581	29.027	19.803
3	0.3777	18.376	15.986	17.501	18.202	19.577
3	0.3787	9.711	10.064	13.787	13.533	15.774
3	0.3797	14.612	13.736	11.173	19.893	21.223
3	0.3807	19.316	13.452	17.445	22.488	32.042
3	0.3817	12.786	16.820	14.152	13.784	12.429
3	0.3827	17.544	12.866	12.139	14.425	5.757
3	0.3837	15.375	7.158	9.448	15.279	7.309
3	0.3847	17.181	11.172	12.964	19.164	20.283
3	0.3857	9.620	4.570	5.816	15.546	26.786
3	0.3867	9.197	4.309	5.395	18.295	19.790
3	0.3877	8.053	3.801	4.990	16.420	11.464
3	0.3887	3.113	3.001	3.707	10.436	26.505
3	0.3897	7.318	5.403	3.138	15.813	20.611
3	0.3907	21.724	11.976	10.542	25.733	30.368
3	0.3917	15.599	9.204	8.376	31.076	25.453
3	0.3927	19.802	11.640	12.030	23.025	16.730

3	0.3937	5.513	4.370	2.986	12.930	9.501
3	0.3947	13.327	10.326	9.404	10.392	7.321
3	0.3957	10.922	13.028	9.191	19.777	15.937
3	0.3967	11.428	6.711	8.350	14.766	13.788
3	0.3977	24.480	16.947	13.492	17.651	23.261
3	0.3987	17.496	10.905	8.366	15.167	25.399
3	0.3997	6.905	7.949	7.558	11.306	10.362
3	0.4007	13.767	15.709	11.538	26.651	19.788
3	0.4017	12.980	17.284	12.067	20.050	23.348
3	0.4027	6.256	5.378	3.865	21.807	23.235
3	0.4037	8.172	6.987	7.714	33.330	27.193
3	0.4047	7.809	6.783	9.998	20.732	15.423
3	0.4057	13.796	7.511	13.600	25.677	14.457
3	0.4067	28.240	18.815	27.869	15.673	16.237
3	0.4077	16.602	11.236	11.455	22.558	17.550
3	0.4087	5.388	5.891	5.184	12.017	15.218
3	0.4097	6.966	10.225	11.426	13.220	20.363
3	0.4107	3.016	5.874	6.506	13.012	13.568
3	0.4117	17.281	7.556	15.591	29.428	20.015
3	0.4127	17.281	7.548	15.578	29.423	20.005
3	0.4137	6.855	9.984	15.966	11.366	12.356
3	0.4147	17.232	17.083	17.205	20.687	14.626
3	0.4157	20.102	15.182	15.609	21.955	17.335
3	0.4167	6.833	6.814	9.862	16.168	11.522
3	0.4177	14.359	12.055	12.951	14.793	14.195
3	0.4187	14.507	12.298	10.039	16.745	15.709
3	0.4197	13.284	11.057	9.898	10.748	19.620
3	0.4207	7.057	7.571	8.701	14.955	21.684
3	0.4217	16.506	10.190	14.862	18.341	25.101
3	0.4227	18.277	12.531	11.672	29.673	19.555
3	0.4237	16.293	11.075	9.566	20.005	10.074
3	0.4247	17.217	15.078	15.225	13.853	15.480
3	0.4257	19.346	21.283	22.787	30.021	17.375
3	0.4267	28.148	23.623	28.751	16.524	18.063
3	0.4277	13.974	7.182	8.380	14.576	18.867
3	0.4287	4.666	4.540	4.524	12.829	10.698
3	0.4297	6.807	3.408	8.908	20.026	18.113
3	0.4307	11.646	13.060	15.375	25.935	23.275
3	0.4317	13.979	15.312	13.851	15.021	15.968
3	0.4327	11.217	9.090	6.086	18.836	15.511
3	0.4337	13.230	10.308	16.481	14.206	20.544
3	0.4347	15.586	12.215	18.972	18.719	10.553
3	0.4357	11.403	17.202	9.304	27.665	17.308
3	0.4367	13.899	18.544	12.839	27.538	17.744
3	0.4377	16.485	17.324	14.226	24.296	14.421
3	0.4387	14.621	15.330	19.231	22.768	11.244
3	0.4397	9.481	16.834	12.326	20.068	7.382
3	0.4407	3.908	3.900	7.406	18.481	20.833
3	0.4417	5.968	8.690	10.166	22.184	22.602
3	0.4427	11.870	12.293	12.676	30.496	23.439

3	0.4437	11.799	14.430	14.465	17.736	17.451
3	0.4447	11.638	14.443	14.368	17.515	17.072
3	0.4457	10.675	12.101	11.414	17.294	17.421
3	0.4467	14.989	14.645	15.880	16.084	18.685
3	0.4477	14.116	10.651	17.695	23.574	15.936
3	0.4487	18.845	7.476	16.846	20.819	13.419
3	0.4497	12.641	13.235	20.166	14.177	11.673
3	0.4507	10.661	9.599	14.766	15.673	14.155
3	0.4517	10.123	10.427	12.980	19.759	13.967
3	0.4527	10.515	13.877	13.953	29.859	22.376
3	0.4537	10.813	15.868	11.086	28.549	15.646
3	0.4547	11.180	12.469	11.148	13.598	15.727
3	0.4557	9.990	11.955	14.490	13.233	11.227
3	0.4567	13.362	7.520	13.523	15.865	17.743
3	0.4577	17.443	14.726	17.596	18.873	18.542
3	0.4587	9.267	7.850	8.588	11.984	15.488
3	0.4597	15.424	20.002	16.515	11.416	14.538
3	0.4607	9.931	15.271	14.206	12.936	22.908
3	0.4617	21.708	14.263	22.470	18.056	19.435
3	0.4627	14.481	11.446	14.161	11.683	20.920
3	0.4637	14.841	14.386	18.633	14.758	22.467
3	0.4647	16.465	18.147	23.694	18.116	23.714
3	0.4657	13.705	13.970	21.609	16.154	35.487
3	0.4667	10.398	5.809	8.720	23.135	21.777
3	0.4677	13.723	9.835	12.113	14.472	16.423
3	0.4687	14.295	13.944	11.635	14.699	9.342
3	0.4697	5.677	4.368	7.303	19.176	25.134
3	0.4707	6.523	5.156	4.504	19.325	21.091
3	0.4717	16.449	11.877	13.603	13.336	15.039
3	0.4727	18.490	15.067	21.772	20.265	14.680
3	0.4737	11.248	8.287	14.879	9.686	10.195
3	0.4747	17.239	13.837	18.126	13.867	10.774
3	0.4757	20.805	16.292	20.712	17.854	10.225
3	0.4767	7.395	7.230	8.917	16.216	5.044
3	0.4777	11.730	5.537	10.666	13.752	6.633
3	0.4787	8.552	2.884	5.843	7.862	5.500
3	0.4797	9.722	11.856	12.819	12.940	11.869
3	0.4807	26.366	23.640	31.970	20.694	24.190
3	0.4817	30.339	26.775	35.369	8.477	15.293
3	0.4827	24.992	16.565	27.812	6.866	18.098
3	0.4837	11.070	6.588	13.166	7.503	17.112
3	0.4847	8.072	18.346	11.867	21.687	9.321
3	0.4857	4.998	9.480	6.496	20.435	12.177
3	0.4867	5.740	8.058	6.077	14.931	6.578
3	0.4877	3.264	8.326	5.501	7.214	7.206
3	0.4887	5.522	8.641	3.830	12.224	14.363
3	0.4897	15.569	13.726	12.626	17.505	10.689
3	0.4907	23.713	16.548	21.220	18.971	15.822
3	0.4917	24.655	16.453	22.653	15.268	14.030
3	0.4927	19.273	10.950	18.144	13.532	9.526

3	0.4937	19.258	11.575	17.588	21.351	24.673
3	0.4947	14.343	6.906	12.730	20.260	17.199
3	0.4957	4.487	7.029	5.945	9.581	18.820
3	0.4967	18.213	14.985	21.689	25.846	13.269
3	0.4977	16.244	7.647	12.573	10.763	10.563
3	0.4987	12.858	8.499	12.202	10.900	10.365
3	0.4997	9.648	5.459	8.812	11.758	4.186
3	0.5007	13.233	4.876	7.051	11.659	24.480
3	0.5017	15.410	10.435	15.526	11.538	20.795
3	0.5027	17.895	13.359	23.881	11.919	19.362
3	0.5037	9.581	3.610	6.909	11.243	14.559
3	0.5047	13.087	9.942	13.190	9.341	15.712
3	0.5057	19.319	15.890	23.685	13.945	21.718
3	0.5067	14.604	17.112	19.368	15.833	13.771
3	0.5077	9.837	10.209	13.714	11.239	9.189
3	0.5087	24.794	13.100	23.380	19.791	23.457
3	0.5097	18.178	10.726	11.845	12.036	18.191
3	0.5107	30.049	16.792	21.567	21.544	19.904
3	0.5117	14.394	8.843	11.049	5.299	13.033
3	0.5127	17.117	7.842	10.741	10.883	7.791
3	0.5137	15.030	12.646	14.321	9.308	5.329
3	0.5147	14.958	12.570	14.262	9.261	5.469
3	0.5157	14.890	12.497	14.200	9.216	5.619
3	0.5167	18.334	13.133	18.379	18.220	9.577
3	0.5177	15.347	10.746	14.079	16.113	10.831
3	0.5187	12.908	12.394	11.591	16.979	9.101
3	0.5197	25.301	19.066	25.211	2.380	9.332
3	0.5207	11.546	11.498	13.227	11.888	9.736
3	0.5217	14.198	9.073	11.484	16.266	13.562
3	0.5227	15.371	7.895	11.438	12.893	9.645
3	0.5237	9.214	6.898	11.261	9.411	8.387
3	0.5247	13.043	14.813	13.519	19.278	10.670
3	0.5257	17.289	22.413	28.095	9.367	22.776
3	0.5267	18.583	16.582	16.340	17.394	14.269
3	0.5277	27.813	24.205	31.336	18.525	15.287
3	0.5287	12.429	6.841	13.227	18.007	6.788
3	0.5297	10.397	7.720	8.128	15.323	12.492
3	0.5307	20.021	14.337	17.783	26.237	21.117
3	0.5317	10.671	19.904	13.828	17.824	10.058
3	0.5327	7.499	13.264	6.142	11.378	8.926
3	0.5337	9.465	19.552	8.922	17.682	6.381
3	0.5347	10.513	19.488	16.621	12.257	7.918
3	0.5357	25.752	20.709	26.184	18.993	10.480
3	0.5367	11.872	9.041	13.318	9.680	7.728
3	0.5377	12.738	8.027	14.124	6.069	9.117
3	0.5387	11.916	10.506	7.470	12.394	10.545
3	0.5397	8.650	5.279	4.928	9.349	11.393
3	0.5407	17.683	11.672	16.435	9.828	8.850
3	0.5417	24.086	13.930	17.199	10.753	13.537
3	0.5427	5.992	3.068	1.789	10.237	14.077

3	0.5437	33.129	16.359	22.204	9.452	12.044
3	0.5447	12.312	9.355	9.618	7.587	12.842
3	0.5457	10.175	6.687	6.998	8.275	8.166
3	0.5467	13.374	6.743	5.946	14.399	13.208
3	0.5477	18.893	5.548	10.238	8.619	13.371
3	0.5487	8.232	5.786	5.187	8.107	9.893
3	0.5497	10.046	8.726	7.050	6.038	5.874
3	0.5507	18.789	19.662	18.647	15.914	13.765
3	0.5517	16.245	12.218	15.644	8.873	16.624
3	0.5527	11.667	12.768	13.367	13.066	13.813
3	0.5537	14.175	7.134	8.760	6.863	13.806
3	0.5547	11.937	7.328	13.783	7.725	19.032
3	0.5557	17.296	8.609	11.178	10.017	19.490
3	0.5567	29.481	21.170	25.469	13.473	19.459
3	0.5577	27.329	20.010	24.437	12.625	13.847
3	0.5587	10.941	7.116	9.009	9.583	9.905
3	0.5597	13.017	8.283	9.751	7.618	19.675
3	0.5607	26.218	14.577	22.344	5.897	9.466
3	0.5617	24.003	13.423	18.945	3.294	8.483
3	0.5627	5.017	4.551	4.355	3.858	5.644
3	0.5637	10.921	9.930	12.568	5.727	10.361
3	0.5647	13.282	7.436	9.333	6.217	9.980
3	0.5657	10.512	8.428	5.518	2.688	12.289
3	0.5667	4.315	5.494	3.345	7.559	12.460
3	0.5677	8.199	6.887	7.417	7.603	4.379
3	0.5687	30.004	17.789	26.506	14.985	21.526
3	0.5697	22.879	19.356	18.199	11.468	13.479
3	0.5707	25.189	16.486	15.850	11.605	19.247
3	0.5717	18.876	13.834	18.147	3.477	19.194
3	0.5727	10.604	12.660	9.325	3.241	17.698
3	0.5737	22.877	15.079	20.859	5.304	11.505
3	0.5747	16.638	14.806	15.326	4.824	12.489
3	0.5757	9.639	10.125	7.868	12.452	9.277
3	0.5767	12.199	8.465	5.615	5.703	18.716
3	0.5777	15.068	13.731	16.658	3.566	13.810
3	0.5787	16.396	11.382	15.657	10.499	11.702
3	0.5797	17.490	12.415	14.321	7.364	8.520
3	0.5807	22.184	13.746	11.941	4.077	7.494
3	0.5817	17.753	12.251	13.791	10.262	10.353
3	0.5827	13.770	15.074	8.231	5.120	9.242
3	0.5837	7.563	13.951	9.347	8.242	9.116
3	0.5847	15.933	14.322	12.719	7.343	11.920
3	0.5857	17.217	12.659	13.543	5.874	15.052
3	0.5867	19.515	15.419	16.249	7.035	15.484
3	0.5877	7.823	9.713	10.655	7.686	8.118
3	0.5887	12.063	10.493	8.136	6.685	4.504
3	0.5897	10.293	9.947	7.822	4.106	4.570
3	0.5907	9.579	15.329	15.847	6.546	4.261
3	0.5917	16.711	15.639	9.949	4.596	12.876
3	0.5927	14.394	7.422	7.466	8.010	16.808

3	0.5937	27.762	17.670	21.377	6.977	14.521
3	0.5947	15.367	11.214	12.481	6.840	8.713
3	0.5957	6.280	7.108	8.027	11.429	8.623
3	0.5967	22.560	15.375	12.529	9.108	18.707
3	0.5977	21.239	8.802	10.430	8.879	12.630
3	0.5987	32.139	19.313	21.478	12.995	4.618
3	0.5997	7.415	14.063	8.201	6.282	9.132
3	0.6007	31.302	24.001	35.936	7.161	6.808
3	0.6017	22.954	10.591	21.866	10.714	5.972
3	0.6027	14.217	9.446	8.183	9.033	24.829
3	0.6037	19.452	21.613	16.921	10.968	14.336
3	0.6047	12.418	8.049	6.731	9.335	18.734
3	0.6057	13.367	8.648	9.366	7.006	12.639
3	0.6067	3.796	3.219	5.214	4.982	6.928
3	0.6077	13.169	9.984	11.955	16.594	13.594
3	0.6087	8.475	7.402	8.700	8.829	10.052
3	0.6097	9.799	8.990	7.427	7.284	12.266
3	0.6107	13.919	10.879	7.425	11.041	13.467
3	0.6117	19.239	13.750	14.848	10.912	17.012
3	0.6127	19.129	21.531	17.091	12.040	19.101
3	0.6137	19.183	21.662	17.199	12.083	19.040
3	0.6147	17.889	9.615	12.103	6.340	14.897
3	0.6157	21.556	11.576	21.762	22.702	14.648
3	0.6167	12.817	11.256	13.274	8.404	12.638
3	0.6177	13.204	8.517	11.391	11.354	24.814
3	0.6187	11.677	12.477	9.248	17.811	16.116
3	0.6197	23.904	14.340	18.917	14.255	11.779
3	0.6207	21.041	12.852	16.973	13.236	8.824
3	0.6217	12.476	9.949	10.350	11.367	10.601
3	0.6227	14.686	15.128	13.742	17.356	7.231
3	0.6237	17.506	7.791	15.263	19.577	13.056
3	0.6247	17.700	21.588	13.401	25.646	14.961
3	0.6257	8.273	10.233	5.739	21.264	11.208
3	0.6267	6.329	4.264	3.664	18.677	17.382
3	0.6277	14.589	15.171	7.696	14.609	6.288
3	0.6287	10.688	12.544	5.730	14.034	11.557
3	0.6297	13.028	8.071	13.038	21.788	9.217
3	0.6307	17.176	12.819	17.045	20.131	11.629
3	0.6317	10.591	11.799	6.337	19.138	11.603
3	0.6327	15.471	10.912	8.408	27.439	22.138
3	0.6337	10.742	4.738	9.458	13.387	23.807
3	0.6347	6.825	13.867	6.835	26.579	16.822
3	0.6357	9.541	12.451	8.464	22.324	7.722
3	0.6367	10.590	14.198	9.109	12.716	11.556
3	0.6377	13.450	14.925	9.234	16.323	20.911
3	0.6387	16.446	24.249	11.893	18.298	13.522
3	0.6397	13.358	5.259	10.551	29.574	16.587
3	0.6407	10.950	3.236	4.262	19.274	19.123
3	0.6417	20.590	10.728	16.508	28.731	12.922
3	0.6427	22.081	14.109	15.120	22.921	15.674

3	0.6437	13.287	9.117	7.938	16.742	21.265
3	0.6447	13.244	13.747	8.017	8.833	8.755
3	0.6457	9.652	11.883	9.957	9.789	13.862
3	0.6467	17.284	14.258	12.173	14.563	14.240
3	0.6477	22.073	25.899	20.257	24.359	14.305
3	0.6487	16.174	26.866	17.401	18.157	15.139
3	0.6497	18.174	27.796	18.058	16.663	13.669
3	0.6507	22.743	23.765	20.801	22.173	17.338
3	0.6517	16.749	20.006	15.282	15.340	15.452
3	0.6527	13.130	18.370	11.256	15.863	13.943
3	0.6537	10.365	10.297	10.579	6.342	10.919
3	0.6547	17.848	13.933	13.355	15.733	16.411
3	0.6557	23.379	13.525	19.255	14.547	22.482
3	0.6567	14.670	8.794	13.795	9.528	13.201
3	0.6577	11.532	9.842	10.536	7.510	20.156
3	0.6587	9.407	11.235	9.738	19.578	22.324
3	0.6597	18.316	18.310	18.654	20.408	14.074
3	0.6607	9.254	9.552	13.832	9.596	8.540
3	0.6617	12.556	10.865	11.812	7.108	12.019
3	0.6627	17.705	23.244	14.701	16.458	9.986
3	0.6637	16.116	19.762	14.412	14.924	17.015
3	0.6647	14.845	10.340	12.048	13.306	12.158
3	0.6657	11.956	21.937	12.794	16.945	11.537
3	0.6667	10.034	8.066	15.554	21.913	21.576
3	0.6677	15.751	12.062	16.179	13.589	10.632
3	0.6687	15.848	12.142	13.942	14.941	21.746
3	0.6697	30.445	29.052	30.258	11.596	14.907
3	0.6707	11.543	18.253	12.331	11.474	13.303
3	0.6717	5.406	8.555	6.137	9.897	11.297
3	0.6727	6.641	8.149	9.844	13.573	9.637
3	0.6737	7.513	11.666	9.826	8.786	12.109
3	0.6747	10.986	15.228	13.732	8.227	12.194
3	0.6757	13.906	20.933	17.040	10.049	11.837
3	0.6767	8.375	14.938	13.455	4.583	13.232
3	0.6777	23.437	15.573	23.249	13.441	7.843
3	0.6787	15.439	17.715	20.126	8.425	3.812
3	0.6797	4.657	10.522	7.054	7.291	6.080
3	0.6807	8.006	13.582	9.626	9.033	4.852
3	0.6817	9.331	13.401	11.501	8.660	16.149
3	0.6827	18.026	10.376	14.227	12.138	8.929
3	0.6837	25.577	20.202	23.398	16.857	12.250
3	0.6847	14.916	13.751	15.625	14.647	7.538
3	0.6857	12.410	11.384	14.423	13.465	10.850
3	0.6867	16.052	13.593	15.697	15.968	10.875
3	0.6877	11.129	13.483	10.902	9.749	7.442
3	0.6887	9.404	7.935	8.074	5.461	11.985
3	0.6897	8.318	4.136	5.917	5.790	12.392
3	0.6907	9.940	4.541	9.431	10.488	17.918
3	0.6917	5.774	3.725	6.049	8.861	9.973
3	0.6927	8.827	7.647	7.815	11.426	9.978

3	0.6937	17.100	12.973	16.128	8.236	16.912
3	0.6947	21.240	15.485	18.110	13.341	12.589
3	0.6957	10.812	6.035	9.877	9.334	19.584
3	0.6967	5.294	5.829	6.054	7.696	14.263
3	0.6977	7.685	6.676	7.965	8.853	15.957
3	0.6987	6.070	11.034	6.507	6.116	5.847
3	0.6997	15.378	12.485	15.917	4.223	8.249
3	0.7007	8.606	7.206	9.405	13.223	13.631
3	0.7017	9.020	6.216	8.599	6.330	11.060
3	0.7027	5.546	3.336	4.381	4.776	8.108
3	0.7037	6.680	4.919	7.275	7.574	13.837
3	0.7047	4.517	6.863	8.311	8.680	12.256
3	0.7057	9.752	9.682	9.657	7.134	10.040
3	0.7067	9.327	4.313	7.347	8.775	13.709
3	0.7077	9.347	5.737	8.467	10.206	9.875
3	0.7087	12.763	10.293	9.637	10.158	6.665
3	0.7097	7.426	15.222	10.892	9.617	9.231
3	0.7107	8.619	12.493	13.870	8.022	12.498
3	0.7117	15.349	11.458	12.195	9.591	16.927
3	0.7127	8.189	8.231	5.961	8.360	9.960
3	0.7137	5.955	3.711	6.314	5.810	8.404
3	0.7147	4.166	6.309	8.538	7.588	9.081
3	0.7157	10.678	7.809	11.578	15.058	13.197
3	0.7167	5.494	9.631	11.748	5.219	4.581
3	0.7177	3.961	6.868	7.027	2.454	9.616
3	0.7187	6.540	3.306	5.760	4.181	14.922
3	0.7197	10.860	10.520	15.239	11.787	11.125
3	0.7207	9.178	6.820	10.082	8.117	15.847
3	0.7217	9.164	7.556	8.339	8.433	16.404
3	0.7227	6.837	8.634	8.637	9.466	15.130
3	0.7237	5.562	13.242	10.414	11.417	6.178
3	0.7247	6.991	7.431	6.177	10.578	13.509
3	0.7257	9.207	9.013	8.207	7.407	13.672
3	0.7267	7.279	8.591	8.229	6.842	11.287
3	0.7277	25.241	12.698	25.813	16.468	20.625
3	0.7287	15.170	11.772	17.689	13.902	14.618
3	0.7297	6.294	12.059	11.922	6.744	12.481
3	0.7307	5.916	11.901	8.403	5.647	12.460
3	0.7317	4.766	9.010	7.077	3.772	21.516
3	0.7327	4.895	6.694	6.779	3.150	14.975
3	0.7337	4.770	3.915	3.860	7.640	16.145
3	0.7347	7.306	9.436	9.117	11.342	15.382
3	0.7357	5.088	5.515	7.128	9.243	19.464
3	0.7367	2.711	5.989	4.144	7.012	25.489
3	0.7377	4.737	4.468	6.160	7.143	22.304
3	0.7387	8.854	7.212	10.866	7.961	13.422
3	0.7397	7.580	9.006	10.743	3.405	15.736
3	0.7407	15.537	19.905	20.513	9.419	13.189
3	0.7417	6.837	8.168	8.388	10.724	12.797
3	0.7427	11.443	10.450	10.080	13.688	24.038

3	0.7437	2.380	2.013	1.119	13.392	9.254
3	0.7447	8.647	8.142	5.577	16.070	12.528
3	0.7457	20.762	11.507	17.233	19.610	19.364
3	0.7467	13.376	11.128	11.904	9.920	17.173
3	0.7477	23.569	24.034	20.137	12.109	17.727
3	0.7487	14.150	11.200	14.050	11.088	15.383
3	0.7497	10.112	11.743	11.579	10.582	9.562
3	0.7507	13.877	16.575	18.639	13.306	12.460
3	0.7517	9.073	7.688	12.184	12.719	9.131
3	0.7527	17.888	11.249	15.020	10.352	9.643
3	0.7537	6.889	5.642	8.056	3.856	9.328
3	0.7547	5.954	8.017	8.003	10.597	13.550
3	0.7557	9.990	10.861	8.240	10.264	16.630
3	0.7567	7.521	9.550	8.855	8.735	12.458
3	0.7577	8.510	8.147	10.213	8.102	13.057
3	0.7587	11.994	13.781	14.393	15.672	14.038
3	0.7597	11.840	14.842	13.994	14.972	12.485
3	0.7607	29.191	18.917	21.387	12.056	12.589
3	0.7617	25.831	11.394	12.621	7.375	16.142
3	0.7627	32.110	13.887	23.490	17.108	15.008
3	0.7637	32.715	22.384	27.864	22.702	13.305
3	0.7647	6.838	5.554	6.793	16.443	22.756
3	0.7657	16.074	14.135	14.643	16.065	23.309
3	0.7667	12.544	9.394	10.947	8.311	34.291
3	0.7677	7.573	11.215	8.183	10.010	10.774
3	0.7687	9.751	9.495	7.330	17.239	17.174
3	0.7697	12.153	8.519	8.297	11.646	14.486
3	0.7707	10.837	17.738	10.163	12.433	14.196
3	0.7717	8.286	9.418	14.736	9.923	13.778
3	0.7727	8.557	8.293	10.203	11.188	11.439
3	0.7737	5.015	8.025	4.824	8.296	4.927
3	0.7747	10.074	10.009	8.251	3.479	12.613
3	0.7757	14.679	18.475	9.059	19.914	3.505
3	0.7767	5.974	5.658	5.991	8.923	15.763
3	0.7777	2.535	9.812	2.735	13.982	10.741
3	0.7787	4.325	11.445	5.754	12.320	8.891
3	0.7797	6.766	15.963	8.534	17.009	17.461
3	0.7807	9.938	10.986	9.419	17.619	15.179
3	0.7817	11.023	9.685	10.116	16.583	28.654
3	0.7827	13.357	8.846	9.901	14.588	27.320
3	0.7837	12.154	9.599	11.907	9.052	18.320
3	0.7847	14.193	9.829	10.590	15.303	24.037
3	0.7857	6.870	5.334	7.281	14.828	15.152
3	0.7867	11.806	4.467	6.247	10.314	15.425
3	0.7877	7.596	6.255	4.086	10.985	15.517
3	0.7887	15.518	12.577	8.597	13.504	22.831
3	0.7897	9.128	6.799	10.364	4.482	11.717
3	0.7907	8.328	8.552	8.564	7.308	14.137
3	0.7917	6.224	7.216	8.539	11.436	16.262
3	0.7927	11.783	9.871	7.311	19.092	27.776

3	0.7937	14.193	8.845	9.463	13.427	7.167
3	0.7947	5.369	11.156	4.416	13.785	23.011
3	0.7957	7.134	11.116	5.669	15.126	19.053
3	0.7967	11.312	16.189	11.950	10.905	13.920
3	0.7977	7.508	10.087	7.777	14.184	9.132
3	0.7987	7.533	10.139	7.777	14.209	8.968
3	0.7997	14.740	15.500	14.037	12.504	12.437
3	0.8007	12.524	14.106	12.467	21.599	14.868
3	0.8017	17.064	15.383	13.184	21.988	34.727
3	0.8027	7.638	8.549	6.425	25.767	33.548
3	0.8037	11.258	17.803	12.312	13.146	11.546
3	0.8047	13.884	17.667	16.196	13.954	16.154
3	0.8057	9.411	15.111	12.746	15.168	14.961
3	0.8067	13.441	16.594	11.739	7.248	9.502
3	0.8077	9.432	13.194	8.202	9.057	6.556
3	0.8087	8.179	10.028	10.149	17.297	34.663
3	0.8097	7.592	8.644	9.692	13.673	17.367
3	0.8107	6.652	7.599	10.158	14.730	18.713
3	0.8117	10.061	7.118	10.657	9.741	12.692
3	0.8127	9.668	12.340	11.388	8.701	16.989
3	0.8137	18.241	12.710	16.443	9.065	21.115
3	0.8147	11.417	11.889	8.478	12.201	23.897
3	0.8157	14.480	16.781	9.193	13.792	19.333
3	0.8167	15.040	16.785	11.924	13.222	17.927
3	0.8177	16.481	20.150	15.968	17.377	20.329
3	0.8187	25.340	19.642	23.382	10.889	21.472
3	0.8197	17.097	12.840	13.882	13.036	11.947
3	0.8207	12.008	14.500	11.746	16.563	13.750
3	0.8217	18.211	12.333	16.849	11.130	16.964
3	0.8227	13.979	11.276	16.795	10.738	15.681
3	0.8237	20.474	11.909	14.178	10.787	30.643
3	0.8247	20.470	11.900	14.192	10.789	30.669
3	0.8257	18.957	11.068	9.338	14.407	29.671
3	0.8267	13.709	16.005	7.896	9.900	22.326
3	0.8277	20.056	17.536	11.097	11.788	11.040
3	0.8287	14.134	15.953	11.847	18.652	11.238
3	0.8297	12.493	14.267	14.062	15.516	6.122
3	0.8307	16.998	13.921	13.458	12.577	15.254
3	0.8317	17.022	13.899	13.489	12.577	15.258
3	0.8327	16.017	16.396	12.537	9.396	9.730
3	0.8337	11.844	15.713	15.549	17.583	15.492
3	0.8347	15.233	10.988	15.100	10.850	12.867
3	0.8357	11.457	14.584	13.096	12.976	15.781
3	0.8367	12.316	12.474	17.947	12.427	20.951
3	0.8377	14.551	13.594	17.914	16.205	24.039
3	0.8387	18.788	14.708	17.827	12.907	31.640
3	0.8397	18.722	14.719	17.801	12.914	31.676
3	0.8407	10.294	16.566	11.005	16.782	6.148
3	0.8417	17.297	16.793	17.439	13.669	19.518
3	0.8427	12.004	8.098	12.210	8.948	7.024

3	0.8437	8.685	3.446	7.626	6.534	8.759
3	0.8447	10.636	6.038	8.309	9.915	18.226
3	0.8457	14.211	12.919	11.040	18.755	17.501
3	0.8467	9.042	8.242	5.672	9.467	11.905
3	0.8477	9.060	8.224	5.702	9.457	11.931
3	0.8487	9.079	8.206	5.732	9.448	11.955
3	0.8497	8.956	9.523	7.328	12.630	9.048
3	0.8507	6.767	6.069	6.034	4.746	14.476
3	0.8517	10.449	7.151	12.549	4.157	16.524
3	0.8527	14.002	9.366	15.568	12.987	15.520
3	0.8537	26.308	21.977	23.690	12.389	18.496
3	0.8547	10.440	7.238	7.641	2.024	13.393
3	0.8557	8.763	11.182	9.224	9.132	12.074
3	0.8567	3.997	7.670	5.670	10.071	12.671
3	0.8577	16.324	13.542	10.370	12.711	14.487
3	0.8587	15.574	14.841	18.217	8.656	13.614
3	0.8597	14.822	11.742	13.255	11.152	24.547
3	0.8607	15.116	13.500	22.400	5.305	24.588
3	0.8617	9.422	10.820	15.915	14.523	23.294
3	0.8627	7.068	5.115	4.749	3.983	12.751
3	0.8637	17.488	15.605	14.524	9.136	30.599
3	0.8647	20.107	18.536	13.453	6.967	24.452
3	0.8657	16.577	11.453	13.000	7.316	19.274
3	0.8667	18.598	16.933	18.366	5.023	12.118
3	0.8677	27.241	20.823	21.044	9.500	16.554
3	0.8687	17.184	17.115	14.847	9.996	16.376
3	0.8697	9.240	7.642	10.323	9.076	16.732
3	0.8707	15.672	18.985	18.770	6.483	12.798
3	0.8717	15.716	18.965	18.914	6.429	12.912
3	0.8727	9.906	10.005	12.404	6.718	12.876
3	0.8737	9.307	6.361	4.440	6.318	7.831
3	0.8747	7.783	10.488	8.512	9.861	9.266
3	0.8757	14.863	7.302	12.061	19.051	16.215
3	0.8767	8.456	7.531	8.476	22.637	12.393
3	0.8777	17.613	18.244	16.785	10.548	14.281
3	0.8787	13.927	12.511	16.192	14.017	8.784
3	0.8797	16.221	12.201	16.263	10.069	13.905
3	0.8807	22.515	13.010	26.881	11.016	27.958
3	0.8817	18.743	14.142	12.736	8.538	11.255
3	0.8827	5.908	5.589	7.506	3.103	3.876
3	0.8837	12.179	19.168	18.844	8.138	18.778
3	0.8847	12.181	19.161	18.864	8.099	18.491
3	0.8857	26.479	19.945	24.626	9.608	27.020
3	0.8867	14.938	10.105	14.097	8.357	20.652
3	0.8877	15.681	10.534	13.240	3.207	15.305
3	0.8887	15.205	18.312	18.932	10.228	19.984
3	0.8897	11.162	11.258	14.086	7.739	14.201
3	0.8907	7.346	10.254	10.509	11.101	14.162
3	0.8917	6.845	10.934	13.178	7.248	6.546
3	0.8927	5.982	5.759	7.989	11.385	18.715

3	0.8937	5.111	6.269	6.471	10.364	9.570
3	0.8947	16.460	22.956	22.490	15.517	19.037
3	0.8957	16.445	22.932	22.447	15.538	18.976
3	0.8967	24.647	18.721	27.332	17.135	15.469
3	0.8977	10.586	8.371	15.601	16.207	8.612
3	0.8987	10.604	8.399	15.605	16.255	8.649
3	0.8997	10.622	8.425	15.609	16.303	8.686
3	0.9007	8.626	5.964	6.992	9.369	16.798
3	0.9017	13.265	15.676	16.215	10.296	25.776
3	0.9027	11.505	7.644	11.272	5.342	3.115
3	0.9037	12.208	6.528	10.669	3.375	2.352
3	0.9047	13.309	5.381	12.880	11.543	11.939
3	0.9057	4.847	0.799	4.174	11.710	14.898
3	0.9067	13.875	9.302	10.792	10.356	16.102
3	0.9077	10.884	11.148	12.326	10.484	16.691
3	0.9087	12.962	10.849	15.459	4.756	13.381
3	0.9097	12.482	10.561	12.974	9.085	7.813
3	0.9107	19.808	17.821	16.227	6.271	16.163
3	0.9117	12.494	14.918	10.128	9.567	17.816
3	0.9127	15.748	17.248	15.208	14.742	20.034
3	0.9137	11.771	16.371	15.572	16.919	19.544
3	0.9147	4.525	2.437	4.363	1.468	10.366
3	0.9157	7.795	7.254	5.355	4.445	12.119
3	0.9167	2.894	4.760	3.933	7.040	10.766
3	0.9177	7.839	9.236	7.940	9.458	4.515
3	0.9187	2.608	2.981	2.777	1.415	1.703
3	0.9197	0.582	0.005	0.019	0.068	0.782
3	0.9207	10.410	8.705	4.421	4.073	6.736
3	0.9217	12.251	10.672	7.036	4.103	7.912
3	0.9227	13.381	6.317	5.874	11.605	5.635
3	0.9237	9.198	10.127	5.173	7.806	12.089
3	0.9247	15.446	13.225	15.648	15.907	17.115
3	0.9257	9.946	7.351	14.921	13.754	19.630
3	0.9267	7.999	8.735	8.475	10.902	9.513
3	0.9277	13.362	3.508	10.783	9.962	9.691
3	0.9287	2.540	1.091	1.519	6.681	7.121
3	0.9297	2.123	1.026	1.432	5.182	3.394
3	0.9307	3.489	3.763	2.952	3.533	2.807
3	0.9317	6.036	4.298	3.912	7.432	12.733
3	0.9327	5.697	6.224	4.536	6.267	20.382
3	0.9337	14.409	9.659	9.415	8.149	23.536
3	0.9347	11.102	6.343	6.938	5.871	22.592
3	0.9357	21.921	13.095	19.554	14.670	14.327
3	0.9367	10.664	8.387	7.623	6.125	15.768
3	0.9377	8.472	6.320	9.388	8.407	10.865
3	0.9387	15.224	12.407	14.215	9.530	17.019
3	0.9397	7.767	6.114	8.998	5.402	14.602
3	0.9407	12.011	9.380	10.866	20.542	23.875
3	0.9417	8.440	6.173	7.451	7.128	11.979
3	0.9427	7.904	4.024	5.398	8.980	14.943

3	0.9437	7.558	4.731	7.381	6.662	23.709
3	0.9447	7.940	5.579	6.113	5.446	14.603
3	0.9457	8.969	10.677	4.140	7.136	18.631
3	0.9467	15.290	13.718	12.314	13.610	14.567
3	0.9477	17.498	8.163	8.813	13.811	25.625
3	0.9487	19.613	14.067	11.119	16.848	34.253
3	0.9497	10.187	10.254	9.444	14.351	27.164
3	0.9507	15.015	11.695	8.765	12.706	34.837
3	0.9517	15.256	12.991	10.464	11.830	16.931
3	0.9527	12.637	12.285	9.636	9.299	23.926
3	0.9537	6.521	6.026	8.533	6.441	8.241
3	0.9547	12.886	6.593	9.361	9.518	13.686
3	0.9557	12.789	9.364	9.275	9.320	19.559
3	0.9567	13.145	7.579	8.749	6.390	18.437
3	0.9577	13.189	7.651	8.791	6.237	18.374
3	0.9587	5.163	7.903	8.592	6.208	13.882
3	0.9597	5.775	10.471	10.353	7.271	17.474
3	0.9607	5.766	7.584	9.427	4.903	29.214
3	0.9617	8.404	9.240	11.501	6.672	22.780
3	0.9627	6.219	10.649	10.441	13.698	13.326
3	0.9637	4.713	10.363	10.276	18.025	11.677
3	0.9647	11.043	8.640	9.834	6.934	10.180
3	0.9657	7.728	8.793	11.780	7.625	8.698
3	0.9667	8.441	6.760	7.469	10.777	13.436
3	0.9677	21.724	11.973	13.861	11.562	19.604
3	0.9687	7.765	11.381	11.156	13.171	11.960
3	0.9697	5.098	7.188	8.670	10.232	6.452
3	0.9707	7.683	9.356	12.394	8.112	12.012
3	0.9717	7.792	10.252	13.812	9.695	14.178
3	0.9727	7.580	7.123	7.851	9.801	15.828
3	0.9737	12.697	13.255	13.184	21.563	22.930
3	0.9747	10.377	11.773	14.954	6.996	14.483
3	0.9757	8.688	6.494	8.963	4.533	6.880
3	0.9767	5.453	6.319	7.730	8.249	8.356
3	0.9777	9.900	12.802	9.958	12.289	20.631
3	0.9787	9.603	11.550	9.638	8.937	11.798
3	0.9797	7.798	9.649	5.220	4.908	8.711
3	0.9807	9.783	11.837	12.185	16.064	32.718
3	0.9817	15.657	13.634	10.807	9.699	17.814
3	0.9827	6.187	9.109	5.144	8.267	10.329
3	0.9837	5.257	10.886	9.849	6.482	5.973
3	0.9847	12.200	11.438	11.429	11.569	10.993
3	0.9857	19.688	9.749	12.426	10.517	15.634
3	0.9867	13.190	9.013	10.243	15.913	22.628
3	0.9877	19.107	12.925	15.639	14.761	19.470
3	0.9887	16.647	12.456	16.117	18.640	18.262
3	0.9897	7.876	7.884	10.898	9.708	10.602
3	0.9907	12.686	15.182	16.890	11.466	12.244
3	0.9917	6.351	11.716	7.729	17.563	9.392
3	0.9927	10.366	12.184	10.798	22.273	12.956

3	0.9937	15.009	9.805	11.107	14.527	13.406
3	0.9947	7.956	11.412	14.878	11.902	19.103
3	0.9957	13.042	13.544	16.906	17.364	10.438
3	0.9967	6.016	8.186	8.254	15.916	12.544
3	0.9977	7.471	6.598	10.324	10.049	9.048
3	0.9987	15.540	13.169	16.165	9.925	20.357
3	0.9997	11.877	6.453	10.819	13.244	13.143
3	1.0007	6.730	6.314	8.229	14.851	17.514
3	1.0017	5.017	4.986	3.501	3.569	14.062
3	1.0027	12.185	10.709	13.076	13.049	18.716
3	1.0037	13.610	11.926	19.979	23.143	11.533
3	1.0047	11.335	12.771	18.567	15.248	15.753
3	1.0057	15.762	17.348	19.963	11.479	19.888
3	1.0067	13.634	12.647	19.570	9.439	8.400
3	1.0077	13.099	15.584	12.503	9.947	17.062
3	1.0087	12.538	13.615	14.020	13.116	6.952
3	1.0097	11.221	8.555	12.288	6.120	11.848
3	1.0107	8.713	13.166	14.498	22.222	19.421
3	1.0117	11.163	14.095	14.246	18.893	12.608
3	1.0127	10.939	11.591	13.931	7.796	1.726
3	1.0137	5.619	2.343	3.239	7.851	8.748
3	1.0147	9.621	6.428	11.124	12.574	11.256
3	1.0157	9.174	7.647	8.426	19.457	20.310
3	1.0167	12.299	12.879	9.893	8.233	14.362
3	1.0177	13.041	7.568	10.284	16.232	14.308
3	1.0187	18.226	14.552	11.474	18.157	19.611
3	1.0197	15.848	13.865	7.263	9.727	15.949
3	1.0207	12.896	10.128	10.241	11.501	9.596
3	1.0217	15.412	14.363	11.233	9.484	11.073
3	1.0227	15.276	12.336	10.932	20.233	16.613
3	1.0237	15.427	17.541	16.916	14.592	12.932
3	1.0247	14.739	20.577	16.552	25.879	20.908
3	1.0257	6.073	5.516	6.306	12.404	7.992
3	1.0267	9.785	10.057	8.942	10.569	6.669
3	1.0277	9.467	7.169	8.727	16.103	13.513
3	1.0287	12.315	13.103	16.149	19.167	19.842
3	1.0297	11.252	12.098	11.128	15.804	16.767
3	1.0307	12.424	16.906	15.199	20.189	17.141
3	1.0317	12.079	11.511	12.481	20.774	24.558
3	1.0327	13.950	9.457	11.673	19.309	12.503
3	1.0337	19.471	23.168	18.625	22.080	19.056
3	1.0347	15.025	18.926	11.688	20.479	23.679
3	1.0357	13.375	16.378	12.658	11.921	15.111
3	1.0367	16.284	15.922	13.005	15.119	21.006
3	1.0377	12.586	6.605	11.023	10.706	9.692
3	1.0387	15.454	7.612	5.568	28.291	24.335
3	1.0397	17.662	9.263	7.852	18.418	15.624
3	1.0407	16.051	12.206	10.866	17.462	22.045
3	1.0417	19.080	11.299	11.085	19.772	18.294
3	1.0427	17.090	16.618	15.133	13.805	8.826

3	1.0437	13.978	7.727	7.296	6.051	14.183
3	1.0447	11.147	7.902	5.992	11.036	8.359
3	1.0457	13.350	13.445	11.096	19.955	24.056
3	1.0467	16.340	13.649	16.023	25.202	34.201
3	1.0477	14.129	8.593	3.575	13.824	26.692
3	1.0487	15.730	5.647	5.465	16.398	18.311
3	1.0497	14.787	6.201	7.132	4.517	13.683
3	1.0507	19.828	20.411	21.450	14.470	19.206
3	1.0517	13.667	12.132	12.424	12.291	9.056
3	1.0527	26.289	23.380	22.704	17.198	20.691
3	1.0537	30.347	25.010	27.262	19.621	15.476
3	1.0547	29.560	21.889	21.468	24.408	21.132
3	1.0557	29.611	21.886	21.486	24.230	21.141
3	1.0567	14.251	10.550	12.209	16.443	17.137
3	1.0577	11.955	13.031	12.548	14.306	4.900
3	1.0587	21.987	14.625	15.981	10.806	15.699
3	1.0597	12.444	13.718	9.121	12.268	16.674
3	1.0607	28.396	10.896	12.729	16.189	20.635
3	1.0617	27.969	16.831	21.568	13.015	17.833
3	1.0627	26.648	15.525	15.837	15.231	22.312
3	1.0637	16.126	14.648	12.099	13.707	25.549
3	1.0647	12.924	15.263	9.886	9.032	14.494
3	1.0657	9.511	14.555	5.833	7.153	8.796
3	1.0667	18.880	20.356	13.087	10.700	18.433
3	1.0677	16.371	18.239	16.226	15.743	12.026
3	1.0687	30.670	19.196	16.997	22.687	10.304
3	1.0697	29.408	24.371	29.700	30.533	18.159
3	1.0707	22.849	18.316	22.080	20.138	19.590
3	1.0717	22.253	19.945	19.215	23.435	35.399
3	1.0727	29.804	26.698	28.183	16.898	22.529
3	1.0737	21.346	13.719	15.494	12.098	10.900
3	1.0747	16.040	18.905	16.883	17.482	23.544
3	1.0757	21.057	23.251	28.612	18.849	13.390
3	1.0767	13.521	13.616	10.394	25.762	14.453
3	1.0777	32.205	23.262	18.859	26.054	22.555
3	1.0787	29.930	30.214	23.847	19.319	17.480
3	1.0797	29.436	19.841	24.474	24.196	17.206
3	1.0807	29.655	17.516	24.922	15.917	11.922
3	1.0817	17.001	15.180	20.508	11.279	8.211
3	1.0827	18.526	16.776	16.184	9.284	13.395
3	1.0837	17.325	13.582	16.732	16.022	14.886
3	1.0847	13.026	10.195	13.696	8.893	6.446
3	1.0857	19.375	19.985	16.693	26.368	15.037
3	1.0867	21.496	11.300	12.880	16.031	8.339
3	1.0877	14.319	12.586	8.946	8.330	4.849
3	1.0887	12.965	13.060	14.668	11.867	14.886
3	1.0897	13.042	7.662	11.760	11.661	8.897
3	1.0907	27.144	20.894	17.830	28.122	27.935
3	1.0917	18.490	12.267	11.782	21.530	23.336
3	1.0927	12.968	12.623	8.474	8.042	10.715

3	1.0937	10.531	5.658	11.358	9.460	6.424
3	1.0947	21.032	12.315	16.741	16.243	10.078
3	1.0957	14.709	9.464	10.442	11.150	10.899
3	1.0967	24.550	18.883	18.296	19.613	18.719
3	1.0977	13.771	12.271	9.927	22.004	21.738
3	1.0987	15.953	12.409	14.244	18.106	17.688
3	1.0997	12.962	8.575	12.712	14.355	12.820
3	1.1007	9.801	6.901	6.893	10.617	8.849
3	1.1017	18.709	15.646	19.293	15.760	11.872
3	1.1027	14.278	19.627	16.847	21.796	12.880
3	1.1037	12.718	10.503	15.929	15.638	7.642
3	1.1047	17.571	11.910	14.212	26.975	13.844
3	1.1057	17.398	17.680	20.857	21.632	15.283
3	1.1067	20.808	23.575	30.533	13.471	9.462
3	1.1077	16.130	20.745	26.238	8.203	9.051
3	1.1087	12.350	11.895	8.846	8.792	13.919
3	1.1097	21.414	16.991	21.665	12.465	15.203
3	1.1107	15.749	20.607	22.714	11.583	11.490
3	1.1117	20.499	19.306	19.740	13.135	15.383
3	1.1127	14.798	14.155	15.284	14.767	12.071
3	1.1137	9.249	14.285	11.960	19.884	11.654
3	1.1147	7.775	12.334	10.216	14.891	7.370
3	1.1157	8.556	15.130	14.343	25.822	11.762
3	1.1167	18.896	12.851	20.471	15.204	12.265
3	1.1177	15.166	17.064	23.785	21.381	13.991
3	1.1187	17.656	11.479	11.983	17.593	18.711
3	1.1197	28.021	17.834	27.841	21.468	20.804
3	1.1207	17.272	12.076	12.560	16.280	19.367
3	1.1217	28.100	23.443	31.246	21.826	19.641
3	1.1227	17.950	10.546	23.073	24.117	12.780
3	1.1237	14.735	9.511	16.104	20.568	12.210
3	1.1247	18.525	11.821	16.943	23.366	27.787
3	1.1257	19.037	10.403	21.235	11.362	22.299
3	1.1267	13.970	13.222	19.438	17.154	21.643
3	1.1277	25.550	16.039	25.497	16.213	12.051
3	1.1287	10.751	6.373	10.549	13.313	7.375
3	1.1297	16.534	13.424	17.017	17.774	12.790
3	1.1307	9.786	8.275	9.859	21.303	11.083
3	1.1317	12.737	9.770	15.339	25.054	6.700
3	1.1327	19.430	12.607	18.117	21.162	17.848
3	1.1337	21.311	23.397	26.608	24.782	17.447
3	1.1347	10.355	8.754	13.758	17.373	5.277
3	1.1357	14.505	13.884	19.463	25.502	9.017
3	1.1367	18.353	8.819	15.837	25.314	15.202
3	1.1377	15.481	16.033	19.621	29.310	9.779
3	1.1387	16.521	18.359	24.399	26.784	18.519
3	1.1397	14.210	9.677	16.374	19.644	19.446
3	1.1407	17.298	18.257	15.328	11.763	18.644
3	1.1417	28.476	13.733	23.201	17.009	17.544
3	1.1427	25.551	16.511	25.562	25.713	12.554

3	1.1437	25.457	16.670	25.376	26.424	12.593
3	1.1447	28.550	22.593	27.364	20.722	11.198
3	1.1457	23.038	16.284	17.260	18.655	16.689
3	1.1467	28.247	15.995	20.342	24.868	21.849
3	1.1477	15.613	13.751	16.971	27.352	15.258
3	1.1487	11.310	7.268	7.491	15.702	8.611
3	1.1497	10.214	6.682	10.160	18.152	13.035
3	1.1507	16.440	13.467	11.989	18.244	4.620
3	1.1517	9.462	13.665	13.064	23.731	20.760
3	1.1527	10.133	13.933	12.495	23.252	16.509
3	1.1537	8.745	10.973	7.568	18.767	14.850
3	1.1547	8.746	10.937	7.601	18.562	14.699
3	1.1557	17.126	16.360	17.750	14.340	15.711
3	1.1567	15.095	12.227	14.328	19.432	16.381
3	1.1577	15.253	8.105	12.356	21.639	15.575
3	1.1587	15.167	8.015	12.298	21.376	15.471
3	1.1597	20.434	11.803	14.638	22.265	17.159
3	1.1607	20.909	12.052	18.396	23.712	20.148
3	1.1617	18.064	16.253	16.098	22.304	7.868
3	1.1627	12.564	11.189	11.675	20.246	16.719
3	1.1637	13.662	9.072	12.734	14.450	10.764
3	1.1647	10.678	3.775	7.650	20.307	12.110
3	1.1657	10.952	6.194	11.470	15.645	11.079
3	1.1667	13.126	9.380	17.020	20.304	13.764
3	1.1677	1.669	1.207	2.259	0.001	0.021
4	0.0002	0.000	0.000	0.000	0.000	0.000
4	0.0012	10.970	14.138	12.645	21.448	27.846
4	0.0022	7.484	11.196	9.800	22.008	24.932
4	0.0032	10.342	12.309	12.930	15.545	14.545
4	0.0042	3.724	5.801	3.324	15.703	8.211
4	0.0052	7.268	5.213	6.597	14.759	14.103
4	0.0062	6.891	5.684	5.433	14.883	22.741
4	0.0072	15.426	3.642	14.105	33.310	43.966
4	0.0082	13.257	8.745	13.686	22.156	28.456
4	0.0092	11.352	13.750	8.213	17.944	14.236
4	0.0102	5.979	7.064	7.947	20.985	21.776
4	0.0112	11.546	9.247	11.679	20.446	21.811
4	0.0122	11.515	7.362	11.421	23.077	32.227
4	0.0132	17.952	12.029	13.388	24.285	28.752
4	0.0142	14.704	11.939	12.544	16.875	26.258
4	0.0152	6.736	6.973	11.805	16.952	26.000
4	0.0162	7.401	8.727	5.584	17.076	15.904
4	0.0172	11.708	12.910	9.584	26.355	22.128
4	0.0182	8.923	8.233	6.652	18.503	28.292
4	0.0192	10.940	10.046	12.048	8.532	10.735
4	0.0202	11.276	14.279	8.320	21.280	19.970
4	0.0212	3.900	3.547	4.662	14.884	13.294
4	0.0222	20.142	15.851	17.900	11.787	14.694
4	0.0232	15.281	18.592	19.146	23.063	19.404
4	0.0242	7.034	15.274	9.488	28.947	17.071

4	0.0252	4.769	13.947	8.431	31.848	21.254
4	0.0262	18.717	12.892	11.626	41.071	29.696
4	0.0272	17.119	15.074	12.235	34.221	23.832
4	0.0282	8.186	11.630	8.407	30.811	16.888
4	0.0292	10.169	15.590	6.621	31.301	19.134
4	0.0302	7.712	11.508	5.494	31.176	12.938
4	0.0312	14.905	13.368	12.353	42.409	19.930
4	0.0322	11.323	14.085	12.987	20.709	21.302
4	0.0332	17.646	22.628	16.768	22.944	18.758
4	0.0342	23.424	25.150	16.341	32.711	22.047
4	0.0352	18.234	14.922	12.815	30.568	17.159
4	0.0362	17.248	18.552	13.364	27.730	26.850
4	0.0372	11.385	11.879	10.102	23.643	23.920
4	0.0382	8.241	9.350	7.070	20.825	20.750
4	0.0392	10.602	5.120	8.406	7.570	11.707
4	0.0402	9.520	7.551	4.452	16.083	22.225
4	0.0412	15.598	11.322	17.475	19.585	18.393
4	0.0422	17.327	19.165	12.720	23.968	25.787
4	0.0432	16.953	19.072	15.923	19.817	22.898
4	0.0442	16.272	20.155	20.888	21.977	24.432
4	0.0452	8.635	10.954	11.779	15.834	13.648
4	0.0462	13.091	7.007	13.241	15.595	8.304
4	0.0472	12.346	18.236	12.443	24.726	24.452
4	0.0482	16.574	21.366	16.119	18.722	16.293
4	0.0492	10.001	14.745	12.425	21.200	21.741
4	0.0502	15.547	13.446	14.988	6.948	20.298
4	0.0512	10.438	19.899	12.189	23.851	20.657
4	0.0522	12.210	14.625	10.551	15.881	23.675
4	0.0532	7.138	12.347	9.909	13.948	14.681
4	0.0542	4.761	9.445	7.260	13.919	16.963
4	0.0552	12.401	15.339	15.982	16.494	11.123
4	0.0562	11.617	20.487	16.190	18.948	13.613
4	0.0572	5.899	6.884	7.061	17.334	28.636
4	0.0582	5.850	10.236	7.831	24.314	25.201
4	0.0592	8.050	14.956	8.951	17.978	13.665
4	0.0602	10.793	9.968	8.740	19.904	14.544
4	0.0612	6.256	13.825	7.126	24.150	24.529
4	0.0622	11.746	14.289	10.927	14.774	15.293
4	0.0632	16.609	20.994	22.526	12.474	15.189
4	0.0642	10.451	14.154	12.450	10.113	12.340
4	0.0652	9.452	13.041	10.953	26.258	15.385
4	0.0662	8.952	7.773	9.314	26.839	12.113
4	0.0672	26.085	17.289	17.812	16.307	10.209
4	0.0682	12.648	7.568	9.460	4.811	12.302
4	0.0692	3.042	2.688	3.684	8.116	4.179
4	0.0702	14.816	11.749	20.042	18.549	17.425
4	0.0712	24.621	32.361	25.881	28.181	18.070
4	0.0722	21.273	15.779	13.947	21.460	14.027
4	0.0732	10.205	14.844	10.632	23.250	19.165
4	0.0742	5.293	18.637	11.564	18.346	13.806

4	0.0752	1.759	3.143	2.739	3.041	3.110
4	0.0762	5.430	6.293	5.838	16.558	14.500
4	0.0772	5.519	6.591	8.438	14.130	12.652
4	0.0782	11.304	9.203	7.340	12.236	18.582
4	0.0792	13.997	8.029	9.156	28.885	17.585
4	0.0802	11.231	18.613	4.978	36.762	18.517
4	0.0812	14.485	21.172	11.273	35.829	17.882
4	0.0822	5.478	12.773	8.449	42.291	17.253
4	0.0832	9.909	9.888	7.205	23.133	27.394
4	0.0842	4.840	17.922	7.378	31.456	11.579
4	0.0852	4.111	14.626	7.887	25.908	16.175
4	0.0862	11.797	10.656	10.354	33.666	23.142
4	0.0872	20.487	10.637	11.714	30.498	25.164
4	0.0882	13.019	16.213	12.535	22.949	20.821
4	0.0892	16.549	18.292	16.349	24.375	14.879
4	0.0902	12.053	8.943	15.624	27.558	26.573
4	0.0912	21.765	15.918	15.143	25.875	13.299
4	0.0922	21.791	15.745	15.274	25.524	13.103
4	0.0932	15.934	19.486	18.024	8.467	10.505
4	0.0942	16.708	7.882	10.800	27.238	29.022
4	0.0952	10.184	15.615	9.707	21.607	18.449
4	0.0962	9.327	15.046	8.469	26.067	14.456
4	0.0972	13.137	15.384	12.399	35.143	19.758
4	0.0982	21.104	27.591	26.088	36.384	16.665
4	0.0992	9.330	16.126	9.466	32.672	12.298
4	0.1002	7.938	8.950	6.726	12.136	15.448
4	0.1012	12.009	15.117	11.687	14.606	14.529
4	0.1022	10.241	20.546	12.713	31.116	10.904
4	0.1032	10.307	20.609	15.536	26.404	10.110
4	0.1042	21.059	23.042	22.943	41.191	17.976
4	0.1052	13.529	13.619	14.537	43.760	20.716
4	0.1062	13.766	15.238	11.538	25.879	27.396
4	0.1072	8.093	13.201	8.019	40.241	20.868
4	0.1082	6.624	7.037	4.042	25.966	16.910
4	0.1092	7.600	12.609	7.194	32.152	19.141
4	0.1102	3.343	14.410	7.193	37.455	20.830
4	0.1112	3.370	14.417	7.235	37.318	20.844
4	0.1122	7.263	13.780	11.083	17.819	15.418
4	0.1132	11.025	24.880	13.508	26.075	12.622
4	0.1142	15.371	20.884	19.417	24.192	11.232
4	0.1152	17.124	17.248	17.264	12.288	12.245
4	0.1162	11.031	18.069	15.572	27.846	17.503
4	0.1172	18.689	22.377	21.911	24.495	15.562
4	0.1182	14.567	23.099	21.110	26.871	28.682
4	0.1192	10.695	21.554	16.040	48.377	23.200
4	0.1202	3.860	15.385	9.331	40.486	20.237
4	0.1212	2.859	8.912	6.622	25.737	25.765
4	0.1222	6.294	8.313	8.487	12.809	13.436
4	0.1232	3.762	5.105	6.493	8.476	15.300
4	0.1242	11.813	17.148	14.448	14.819	26.652

4	0.1252	7.192	18.990	9.196	30.644	20.415
4	0.1262	19.041	20.595	26.061	28.425	18.560
4	0.1272	14.565	22.678	20.775	14.744	12.400
4	0.1282	13.811	17.184	18.749	18.922	23.002
4	0.1292	13.768	17.114	18.533	18.772	22.800
4	0.1302	5.638	9.902	15.907	8.714	7.462
4	0.1312	14.346	18.036	21.217	18.703	18.176
4	0.1322	12.351	10.222	13.192	24.807	14.488
4	0.1332	11.874	12.840	8.799	36.283	14.397
4	0.1342	20.398	17.046	13.930	38.796	19.660
4	0.1352	8.869	10.477	6.829	19.111	29.961
4	0.1362	18.740	24.390	26.575	24.123	18.856
4	0.1372	13.501	19.098	18.212	17.606	4.482
4	0.1382	5.407	7.701	6.413	9.995	14.903
4	0.1392	5.698	6.689	7.743	6.919	15.127
4	0.1402	9.923	8.627	7.416	11.252	12.654
4	0.1412	10.171	19.497	8.031	21.548	15.688
4	0.1422	15.700	19.739	16.278	15.203	8.011
4	0.1432	15.454	26.024	18.814	25.334	16.631
4	0.1442	17.642	10.584	15.373	31.167	18.979
4	0.1452	11.660	5.781	14.545	17.768	18.904
4	0.1462	16.186	16.538	19.679	13.060	11.536
4	0.1472	22.053	24.881	19.813	16.437	12.543
4	0.1482	17.479	22.296	19.630	28.213	13.119
4	0.1492	14.517	7.907	12.193	13.120	15.034
4	0.1502	13.934	12.046	9.300	16.091	8.902
4	0.1512	25.471	12.888	21.679	25.291	11.243
4	0.1522	19.806	6.816	10.137	21.003	19.392
4	0.1532	9.452	10.445	14.924	8.457	13.750
4	0.1542	9.246	6.978	8.349	15.756	6.655
4	0.1552	9.643	10.480	9.781	19.291	9.078
4	0.1562	7.381	10.799	8.253	26.668	12.875
4	0.1572	26.134	19.363	25.007	25.713	17.736
4	0.1582	11.780	7.373	9.681	25.972	16.761
4	0.1592	5.335	7.158	5.045	22.445	10.202
4	0.1602	11.410	12.855	13.691	27.243	10.766
4	0.1612	10.152	12.149	8.063	20.810	11.002
4	0.1622	13.693	14.982	11.749	15.838	11.840
4	0.1632	12.025	8.846	10.685	31.412	20.753
4	0.1642	14.251	10.956	9.806	18.413	10.334
4	0.1652	10.500	8.812	8.186	22.244	12.700
4	0.1662	9.500	5.236	6.435	22.850	11.362
4	0.1672	6.088	8.748	7.186	27.109	19.465
4	0.1682	7.869	8.605	7.206	18.847	19.809
4	0.1692	9.483	6.644	8.652	25.861	11.808
4	0.1702	13.607	11.508	11.920	32.008	11.745
4	0.1712	13.534	11.439	11.883	32.044	11.764
4	0.1722	7.759	10.413	7.876	34.959	12.174
4	0.1732	8.628	16.548	8.187	31.657	14.406
4	0.1742	5.878	11.849	7.969	35.181	12.436

4	0.1752	6.007	15.041	8.335	31.284	11.833
4	0.1762	10.594	9.654	7.086	24.833	15.589
4	0.1772	10.305	18.971	18.134	25.943	19.748
4	0.1782	8.633	18.547	17.689	23.203	19.065
4	0.1792	14.417	23.026	17.039	32.984	26.043
4	0.1802	13.946	13.204	19.094	15.753	23.146
4	0.1812	6.614	12.766	16.321	23.697	16.917
4	0.1822	9.181	14.119	17.173	21.094	12.722
4	0.1832	11.490	18.066	10.549	12.121	6.747
4	0.1842	11.428	16.488	18.150	12.413	9.964
4	0.1852	14.293	10.917	14.511	12.048	27.795
4	0.1862	19.320	11.855	17.763	24.422	28.081
4	0.1872	11.392	9.881	8.272	11.755	7.752
4	0.1882	11.401	16.017	10.896	14.103	7.113
4	0.1892	20.371	15.748	21.341	21.255	7.509
4	0.1902	8.898	12.233	9.150	21.802	18.062
4	0.1912	6.317	10.601	12.844	13.726	15.580
4	0.1922	14.697	6.217	13.460	17.529	15.736
4	0.1932	9.861	11.744	10.200	17.061	17.763
4	0.1942	5.456	10.728	9.786	13.582	14.352
4	0.1952	10.497	10.581	11.080	31.366	24.402
4	0.1962	13.545	14.854	13.625	33.316	26.607
4	0.1972	10.022	10.068	12.805	18.529	15.265
4	0.1982	10.564	15.889	10.657	9.235	14.832
4	0.1992	17.566	12.444	20.345	25.893	6.472
4	0.2002	11.920	13.138	13.288	22.601	18.838
4	0.2012	17.053	10.611	13.695	7.352	7.825
4	0.2022	8.666	7.088	8.861	7.612	7.137
4	0.2032	13.566	9.706	11.386	16.700	11.549
4	0.2042	21.504	17.979	18.988	16.454	19.478
4	0.2052	16.965	12.762	16.645	28.260	13.860
4	0.2062	12.432	16.719	16.520	21.218	9.592
4	0.2072	4.577	5.656	5.115	13.733	8.292
4	0.2082	9.264	18.054	9.589	18.885	19.100
4	0.2092	10.766	9.992	13.805	10.432	9.742
4	0.2102	15.152	18.946	20.486	10.556	15.075
4	0.2112	11.703	10.094	10.069	14.544	18.553
4	0.2122	16.385	18.313	14.321	21.171	20.412
4	0.2132	7.597	13.365	9.321	17.816	8.144
4	0.2142	14.737	15.321	11.808	18.980	10.951
4	0.2152	14.994	14.582	18.047	5.625	6.824
4	0.2162	10.318	3.941	11.323	13.620	8.159
4	0.2172	4.563	12.030	6.979	13.916	7.783
4	0.2182	17.741	21.671	12.986	23.534	8.398
4	0.2192	17.802	21.723	13.023	23.515	8.364
4	0.2202	17.864	21.774	13.060	23.495	8.336
4	0.2212	12.103	16.225	9.903	16.362	12.740
4	0.2222	11.725	8.901	8.897	12.971	11.222
4	0.2232	8.064	10.508	3.417	18.295	13.032
4	0.2242	7.998	10.455	3.379	18.301	13.033

4	0.2252	7.931	10.401	3.341	18.306	13.034
4	0.2262	7.863	10.346	3.303	18.310	13.035
4	0.2272	20.102	15.566	15.056	10.100	14.226
4	0.2282	10.042	11.885	10.126	27.837	12.853
4	0.2292	3.517	8.350	3.470	14.720	4.748
4	0.2302	6.645	13.509	4.601	16.266	11.164
4	0.2312	14.186	13.229	14.860	32.136	10.187
4	0.2322	14.473	9.393	11.836	30.751	18.648
4	0.2332	6.274	7.422	6.571	37.677	13.682
4	0.2342	2.884	13.093	5.613	34.044	15.851
4	0.2352	8.561	17.138	12.264	19.350	16.689
4	0.2362	6.820	11.787	5.738	20.394	13.799
4	0.2372	14.237	15.899	9.749	31.035	11.705
4	0.2382	14.229	15.896	9.763	31.044	11.707
4	0.2392	8.464	7.947	8.213	19.065	9.625
4	0.2402	16.308	15.997	18.234	16.308	14.315
4	0.2412	17.032	18.755	21.898	18.065	19.055
4	0.2422	17.450	10.357	8.050	24.121	12.518
4	0.2432	12.624	15.334	15.590	24.702	18.607
4	0.2442	10.109	7.523	7.034	19.840	8.503
4	0.2452	7.234	7.679	5.522	3.035	3.797
4	0.2462	7.492	6.255	6.733	4.550	4.262
4	0.2472	8.793	7.221	8.806	4.604	6.215
4	0.2482	10.141	13.261	12.794	12.990	6.308
4	0.2492	8.837	14.324	9.058	19.855	6.087
4	0.2502	7.272	6.262	11.072	13.757	7.165
4	0.2512	8.014	10.487	7.645	13.969	6.119
4	0.2522	9.213	13.300	5.827	23.390	8.728
4	0.2532	13.640	17.658	10.550	22.712	13.348
4	0.2542	12.316	20.637	12.581	28.593	11.876
4	0.2552	8.328	11.043	10.946	3.938	4.342
4	0.2562	17.860	18.485	20.640	12.070	1.872
4	0.2572	13.084	17.326	16.550	14.630	4.080
4	0.2582	17.194	24.307	19.718	23.802	19.165
4	0.2592	10.635	15.504	9.118	27.106	17.117
4	0.2602	10.445	15.249	9.011	26.957	17.114
4	0.2612	27.451	20.422	17.636	31.234	11.407
4	0.2622	14.697	16.244	12.430	24.985	11.322
4	0.2632	9.723	11.749	6.111	24.688	14.385
4	0.2642	14.192	15.487	17.228	16.651	9.262
4	0.2652	11.671	20.132	18.038	17.698	9.815
4	0.2662	7.380	13.448	11.197	18.075	14.755
4	0.2672	9.686	11.993	13.840	7.371	6.510
4	0.2682	3.493	4.536	3.346	16.543	7.838
4	0.2692	5.027	10.787	8.056	20.287	7.765
4	0.2702	5.532	8.304	6.207	9.215	7.486
4	0.2712	6.045	9.000	5.895	14.287	6.641
4	0.2722	10.672	11.917	9.841	16.207	6.631
4	0.2732	7.344	14.610	7.002	28.352	11.526
4	0.2742	13.415	11.736	11.992	13.243	12.146

4	0.2752	9.514	16.233	13.485	17.187	14.962
4	0.2762	14.382	18.226	16.675	23.966	18.957
4	0.2772	15.225	15.188	14.268	19.276	13.073
4	0.2782	7.098	6.040	5.972	5.543	6.838
4	0.2792	7.538	9.241	7.460	15.319	8.221
4	0.2802	18.980	16.466	13.589	24.835	14.873
4	0.2812	18.973	16.477	13.583	24.852	14.842
4	0.2822	10.331	14.990	11.690	20.122	8.224
4	0.2832	7.392	13.516	8.738	13.384	7.884
4	0.2842	18.860	15.852	20.848	10.574	8.466
4	0.2852	26.966	22.488	26.503	16.573	7.561
4	0.2862	19.633	12.035	17.829	24.372	11.404
4	0.2872	14.743	14.435	15.082	19.642	11.983
4	0.2882	16.835	17.787	15.051	17.066	15.488
4	0.2892	13.879	19.847	14.879	17.218	15.877
4	0.2902	8.816	6.467	6.384	9.433	8.743
4	0.2912	12.520	16.420	14.873	11.961	12.298
4	0.2922	8.534	12.376	9.217	12.494	9.394
4	0.2932	13.437	14.319	15.839	6.970	10.850
4	0.2942	6.392	5.118	4.569	5.041	11.333
4	0.2952	14.250	15.632	12.163	22.693	10.225
4	0.2962	10.693	18.458	10.137	19.189	14.025
4	0.2972	20.039	21.582	13.485	26.087	16.631
4	0.2982	18.275	11.683	16.409	10.780	18.119
4	0.2992	28.437	23.391	30.956	17.050	15.792
4	0.3002	13.967	11.338	15.854	17.236	9.482
4	0.3012	15.786	15.867	14.006	10.129	9.573
4	0.3022	9.323	12.683	15.412	7.708	5.755
4	0.3032	9.228	12.555	15.277	7.680	5.712
4	0.3042	9.135	12.427	15.143	7.655	5.678
4	0.3052	9.045	12.301	15.010	7.633	5.652
4	0.3062	5.314	11.243	7.176	13.368	13.598
4	0.3072	12.191	14.717	12.228	21.854	18.686
4	0.3082	18.606	11.139	13.756	21.251	9.286
4	0.3092	17.160	10.553	13.030	11.488	5.819
4	0.3102	8.553	3.987	9.058	10.054	6.587
4	0.3112	18.519	11.306	18.227	9.280	10.339
4	0.3122	26.547	10.880	19.523	11.081	8.594
4	0.3132	19.545	14.142	17.344	4.767	9.987
4	0.3142	12.014	9.432	12.475	1.537	4.572
4	0.3152	13.086	13.490	13.775	17.527	10.373
4	0.3162	18.774	18.854	16.522	14.952	9.716
4	0.3172	16.731	12.234	14.482	10.781	11.300
4	0.3182	7.114	8.681	11.340	6.946	2.893
4	0.3192	14.237	11.908	12.607	8.045	14.879
4	0.3202	11.825	9.707	9.834	12.437	10.585
4	0.3212	11.125	12.121	12.860	12.019	7.334
4	0.3222	6.181	2.933	3.878	10.033	5.053
4	0.3232	9.234	4.178	9.334	9.296	6.254
4	0.3242	11.643	7.589	12.022	14.120	13.730

4	0.3252	9.123	12.557	14.824	14.325	10.088
4	0.3262	6.237	3.453	6.920	6.338	9.977
4	0.3272	16.345	5.059	13.315	17.682	13.713
4	0.3282	22.330	16.088	16.053	18.152	15.048
4	0.3292	13.424	13.888	13.267	11.352	15.495
4	0.3302	8.762	7.007	10.133	12.113	8.659
4	0.3312	9.176	6.861	9.942	8.511	3.425
4	0.3322	13.511	11.279	12.642	10.787	5.441
4	0.3332	13.468	11.610	13.142	24.010	19.875
4	0.3342	14.783	10.294	17.131	17.877	8.792
4	0.3352	8.708	8.418	9.395	11.684	9.872
4	0.3362	8.391	11.558	9.060	17.636	10.143
4	0.3372	18.075	16.020	18.869	11.557	16.304
4	0.3382	11.349	10.607	14.356	14.493	16.926
4	0.3392	21.563	20.807	25.380	9.684	15.395
4	0.3402	20.384	14.250	22.130	10.693	8.537
4	0.3412	21.666	18.496	23.211	10.248	12.763
4	0.3422	15.019	6.737	18.171	11.224	11.785
4	0.3432	15.405	10.335	19.082	14.560	16.607
4	0.3442	7.936	10.698	14.336	12.442	12.516
4	0.3452	7.858	9.945	10.738	14.971	10.611
4	0.3462	6.282	8.522	7.945	7.679	6.686
4	0.3472	5.806	7.624	7.603	9.059	15.912
4	0.3482	10.827	14.943	15.568	5.938	8.572
4	0.3492	17.019	13.175	16.306	13.019	10.614
4	0.3502	12.739	9.747	14.198	7.786	14.005
4	0.3512	23.159	19.805	24.437	11.461	19.149
4	0.3522	9.538	9.584	12.219	9.034	8.739
4	0.3532	2.408	9.299	6.457	12.010	6.670
4	0.3542	9.182	14.171	10.742	14.496	10.025
4	0.3552	9.061	9.397	8.165	3.597	9.406
4	0.3562	13.210	13.028	10.905	15.043	16.749
4	0.3572	9.275	8.926	10.202	11.373	10.850
4	0.3582	21.544	9.945	20.543	10.771	14.841
4	0.3592	22.458	10.150	20.794	10.305	14.240
4	0.3602	5.754	8.427	5.360	8.188	14.027
4	0.3612	9.252	12.799	12.713	11.361	18.637
4	0.3622	7.502	5.683	9.585	9.914	11.768
4	0.3632	4.226	5.947	10.301	8.089	10.876
4	0.3642	1.469	2.821	2.432	6.989	5.010
4	0.3652	5.393	4.553	4.352	7.734	7.406
4	0.3662	10.635	17.865	14.065	18.994	13.905
4	0.3672	16.430	12.373	12.576	15.370	17.262
4	0.3682	9.639	11.485	9.311	18.831	9.790
4	0.3692	9.186	10.672	8.785	14.440	7.904
4	0.3702	13.264	13.691	16.992	20.989	16.131
4	0.3712	10.551	11.838	14.585	16.883	12.972
4	0.3722	7.333	7.137	11.812	14.444	8.861
4	0.3732	6.785	6.354	10.188	3.053	6.891
4	0.3742	6.844	8.497	8.878	8.622	8.082

4	0.3752	10.473	10.923	11.806	13.734	18.424
4	0.3762	11.628	8.580	16.083	7.399	12.644
4	0.3772	10.158	10.285	10.860	21.286	7.090
4	0.3782	13.166	25.039	21.411	21.360	10.388
4	0.3792	12.322	18.521	15.571	21.978	18.725
4	0.3802	5.431	11.479	6.536	17.682	11.569
4	0.3812	3.608	11.321	6.518	18.702	12.669
4	0.3822	12.917	12.136	11.613	13.514	11.651
4	0.3832	13.004	11.898	10.078	7.423	9.514
4	0.3842	15.586	20.539	17.701	18.177	12.241
4	0.3852	10.492	13.887	8.408	13.254	8.635
4	0.3862	12.007	16.836	10.961	19.641	7.698
4	0.3872	11.154	11.951	12.427	20.638	12.629
4	0.3882	13.292	11.891	12.603	17.536	11.051
4	0.3892	9.168	10.981	12.349	12.194	14.903
4	0.3902	17.599	5.199	8.869	8.326	17.396
4	0.3912	8.234	8.956	7.846	14.927	8.639
4	0.3922	9.554	15.194	5.766	15.693	7.468
4	0.3932	12.440	21.737	15.193	21.878	11.438
4	0.3942	3.038	3.863	4.604	6.262	6.830
4	0.3952	2.831	2.620	5.205	5.471	4.978
4	0.3962	7.837	2.439	10.029	7.300	3.509
4	0.3972	6.746	2.388	5.002	7.284	7.572
4	0.3982	7.043	6.362	7.194	7.411	8.400
4	0.3992	7.043	6.361	7.192	7.413	8.401
4	0.4002	19.014	18.638	21.526	20.933	9.955
4	0.4012	17.202	22.995	14.624	11.000	13.500
4	0.4022	4.398	3.483	7.493	6.350	7.721
4	0.4032	14.887	8.931	12.718	18.319	14.944
4	0.4042	9.946	7.273	8.595	14.213	15.535
4	0.4052	11.451	13.928	13.309	6.696	14.496
4	0.4062	15.802	13.997	17.936	13.452	16.235
4	0.4072	25.453	18.041	20.062	13.950	14.269
4	0.4082	18.397	18.658	14.187	19.426	19.266
4	0.4092	13.728	18.136	15.953	8.440	8.446
4	0.4102	3.582	4.344	4.078	2.176	1.478
4	0.4112	8.416	8.770	6.297	2.511	1.751
4	0.4122	13.529	10.586	13.334	10.203	10.589
4	0.4132	15.737	15.997	17.851	14.451	11.458
4	0.4142	15.521	17.484	18.134	6.286	11.115
4	0.4152	11.749	17.525	14.670	9.897	11.937
4	0.4162	16.120	11.145	11.140	11.895	9.314
4	0.4172	10.274	4.870	3.844	16.002	9.514
4	0.4182	16.716	20.537	15.206	21.488	17.376
4	0.4192	17.737	8.710	13.017	7.990	11.798
4	0.4202	10.611	8.147	10.842	10.999	13.757
4	0.4212	10.510	12.095	14.719	14.758	9.662
4	0.4222	10.376	18.036	14.763	17.699	13.871
4	0.4232	10.360	17.992	14.723	17.652	13.882
4	0.4242	15.407	9.805	12.967	19.738	14.091

4	0.4252	13.587	8.619	14.076	12.869	12.864
4	0.4262	5.401	7.738	6.885	9.214	6.756
4	0.4272	9.023	8.952	7.265	7.877	14.494
4	0.4282	21.364	14.977	10.652	14.345	13.619
4	0.4292	11.580	7.337	12.623	9.058	6.002
4	0.4302	4.365	4.111	3.688	11.701	8.608
4	0.4312	20.119	13.383	17.070	5.779	8.785
4	0.4322	23.928	21.358	23.366	16.792	10.695
4	0.4332	15.912	10.432	11.161	22.908	14.267
4	0.4342	11.410	7.640	11.328	13.593	12.962
4	0.4352	17.333	13.282	16.157	11.369	12.979
4	0.4362	17.691	12.564	15.815	11.871	10.077
4	0.4372	10.030	6.710	8.695	10.825	11.619
4	0.4382	6.789	5.221	6.271	11.862	5.904
4	0.4392	8.194	2.541	4.820	3.933	12.445
4	0.4402	17.643	11.884	11.904	1.857	12.377
4	0.4412	7.938	8.758	9.742	15.449	16.926
4	0.4422	9.138	7.138	9.109	11.112	19.399
4	0.4432	10.239	8.642	9.484	5.843	9.902
4	0.4442	12.724	6.025	9.091	8.229	14.713
4	0.4452	18.855	13.691	17.435	16.941	11.353
4	0.4462	6.929	13.036	6.134	23.199	16.665
4	0.4472	4.569	8.981	4.052	12.863	16.484
4	0.4482	10.837	11.638	11.023	10.962	13.870
4	0.4492	7.883	10.559	11.276	5.398	4.735
4	0.4502	10.703	7.951	9.643	10.477	16.685
4	0.4512	17.566	11.767	15.311	10.767	12.826
4	0.4522	14.864	15.404	12.608	8.394	7.949
4	0.4532	7.142	8.148	3.294	10.849	8.601
4	0.4542	7.504	5.604	8.242	11.295	9.234
4	0.4552	8.583	5.902	7.138	5.088	10.416
4	0.4562	8.779	9.226	10.269	9.509	9.808
4	0.4572	11.879	5.856	7.054	9.160	16.617
4	0.4582	23.770	17.738	16.695	12.571	18.752
4	0.4592	23.388	20.824	19.798	4.772	9.177
4	0.4602	23.743	22.395	24.862	6.324	11.773
4	0.4612	17.872	13.939	18.089	6.113	5.824
4	0.4622	17.054	9.326	13.893	8.974	18.607
4	0.4632	15.760	16.572	18.823	11.297	27.371
4	0.4642	28.768	20.355	25.650	9.845	8.943
4	0.4652	19.049	14.290	17.100	12.743	8.131
4	0.4662	8.224	11.198	8.490	15.552	11.069
4	0.4672	12.986	10.361	12.590	18.466	30.611
4	0.4682	20.503	6.649	13.508	17.914	28.184
4	0.4692	17.033	14.618	17.066	17.242	14.494
4	0.4702	8.963	13.807	9.335	9.713	13.933
4	0.4712	10.929	12.177	9.889	10.354	8.876
4	0.4722	10.689	8.148	11.619	15.751	12.461
4	0.4732	22.804	18.653	21.440	21.543	26.358
4	0.4742	7.563	2.870	4.549	6.203	0.969

4	0.4752	11.234	5.158	8.658	6.254	4.682
4	0.4762	8.727	8.161	7.882	6.119	7.763
4	0.4772	15.302	14.105	12.854	4.835	14.451
4	0.4782	13.155	8.892	10.773	14.766	16.909
4	0.4792	17.137	7.693	12.249	9.258	15.932
4	0.4802	15.301	11.405	13.748	14.001	13.371
4	0.4812	19.952	17.442	19.176	20.390	21.154
4	0.4822	21.967	14.706	16.611	18.375	17.190
4	0.4832	25.425	17.473	19.894	23.151	9.978
4	0.4842	25.443	22.706	16.182	21.200	21.313
4	0.4852	15.057	20.222	10.873	8.065	11.721
4	0.4862	8.743	6.137	9.137	9.787	15.059
4	0.4872	10.027	12.290	11.379	8.032	9.610
4	0.4882	10.663	10.824	12.848	9.221	8.672
4	0.4892	18.613	17.076	22.576	14.855	13.928
4	0.4902	22.291	17.728	21.870	13.952	15.074
4	0.4912	26.351	19.593	22.767	25.133	32.733
4	0.4922	17.373	15.598	13.274	15.318	17.439
4	0.4932	12.240	13.459	10.437	15.428	19.151
4	0.4942	18.409	19.463	21.297	9.729	14.654
4	0.4952	14.737	13.190	17.762	8.269	7.149
4	0.4962	22.647	20.214	23.614	24.400	19.004
4	0.4972	17.294	20.370	20.846	25.008	19.555
4	0.4982	15.160	25.649	21.416	21.936	18.646
4	0.4992	22.796	22.930	19.737	15.084	17.865
4	0.5002	15.513	15.806	13.746	18.695	18.093
4	0.5012	12.669	10.994	10.535	12.108	15.999
4	0.5022	13.890	11.566	9.004	26.273	20.286
4	0.5032	14.288	12.398	12.869	22.549	22.394
4	0.5042	22.164	24.478	27.685	10.650	26.149
4	0.5052	17.395	11.465	13.255	17.625	14.698
4	0.5062	15.971	16.786	16.442	17.729	16.855
4	0.5072	18.517	15.051	18.564	7.969	12.382
4	0.5082	12.657	15.024	15.741	23.502	31.748
4	0.5092	18.935	20.947	18.516	27.441	41.978
4	0.5102	18.942	20.941	18.536	27.448	41.981
4	0.5112	24.619	28.165	25.377	19.630	11.874
4	0.5122	14.460	27.249	20.166	20.847	5.777
4	0.5132	15.596	27.986	19.347	18.477	9.017
4	0.5142	23.346	24.635	18.428	16.639	16.329
4	0.5152	9.793	12.834	7.019	11.017	10.182
4	0.5162	14.174	22.465	16.455	11.189	11.420
4	0.5172	12.935	16.080	13.148	8.699	4.502
4	0.5182	15.255	13.882	16.989	22.373	11.910
4	0.5192	17.457	7.045	15.040	12.918	9.959
4	0.5202	4.890	7.228	5.408	8.735	7.970
4	0.5212	11.869	16.971	11.993	18.790	5.912
4	0.5222	6.915	5.595	12.310	6.733	5.232
4	0.5232	12.437	8.660	9.070	5.004	9.252
4	0.5242	9.170	11.613	9.825	5.713	6.279

4	0.5252	11.183	12.148	10.814	16.311	8.441
4	0.5262	11.179	12.150	10.795	16.314	8.441
4	0.5272	7.477	4.221	4.540	12.395	6.374
4	0.5282	6.521	7.036	3.667	8.176	5.515
4	0.5292	7.924	9.263	5.959	10.852	8.935
4	0.5302	8.600	7.873	8.854	8.328	12.885
4	0.5312	10.325	9.837	10.483	11.047	8.631
4	0.5322	17.097	14.274	11.776	18.033	15.942
4	0.5332	23.146	18.994	12.603	7.006	23.336
4	0.5342	14.874	28.067	13.303	12.491	16.851
4	0.5352	18.106	19.786	16.304	15.675	11.369
4	0.5362	12.668	14.641	11.854	14.703	11.661
4	0.5372	12.670	14.646	11.854	14.706	11.660
4	0.5382	10.926	20.883	12.267	20.292	8.678
4	0.5392	11.974	21.042	10.142	13.977	13.638
4	0.5402	12.206	19.060	10.630	22.726	6.652
4	0.5412	19.677	17.951	14.768	22.254	17.841
4	0.5422	18.766	20.515	14.523	18.738	12.174
4	0.5432	33.449	22.699	27.719	9.425	12.419
4	0.5442	19.930	20.444	23.277	9.919	11.039
4	0.5452	20.451	19.452	17.654	9.359	9.392
4	0.5462	16.229	19.099	13.753	19.321	16.583
4	0.5472	12.728	14.887	10.457	21.012	16.675
4	0.5482	15.411	24.652	12.339	20.047	10.893
4	0.5492	22.392	24.392	16.336	10.296	20.251
4	0.5502	15.597	11.920	6.839	11.762	15.457
4	0.5512	21.515	20.363	14.600	15.201	10.399
4	0.5522	12.064	10.754	9.078	8.787	7.449
4	0.5532	14.794	15.700	14.840	15.270	8.638
4	0.5542	12.159	18.486	13.972	16.583	17.019
4	0.5552	9.628	7.339	7.668	17.272	24.844
4	0.5562	22.017	14.270	15.411	20.378	18.951
4	0.5572	19.582	13.607	14.991	11.527	17.021
4	0.5582	9.754	7.364	7.801	9.610	11.390
4	0.5592	7.957	5.405	3.950	7.496	6.021
4	0.5602	13.469	13.391	15.731	13.843	8.608
4	0.5612	13.560	13.392	15.739	13.973	8.662
4	0.5622	11.024	10.995	8.091	13.779	14.664
4	0.5632	11.048	11.020	8.091	13.782	14.707
4	0.5642	17.346	17.425	14.395	8.330	11.320
4	0.5652	16.863	17.726	11.865	9.202	18.222
4	0.5662	21.530	28.967	22.377	10.187	13.002
4	0.5672	14.619	21.235	11.922	11.003	9.763
4	0.5682	12.496	14.972	14.445	9.874	15.414
4	0.5692	10.318	13.191	7.050	11.450	8.062
4	0.5702	18.934	18.573	18.418	14.630	7.809
4	0.5712	12.332	11.791	7.437	3.447	11.221
4	0.5722	9.437	11.316	5.943	6.379	11.915
4	0.5732	14.913	13.706	10.053	17.974	12.984
4	0.5742	32.413	35.832	27.534	9.810	20.414

4	0.5752	7.537	11.907	6.579	9.240	8.387
4	0.5762	14.098	11.468	14.026	10.225	8.276
4	0.5772	11.715	9.130	9.382	11.955	9.045
4	0.5782	13.139	10.529	9.413	11.390	13.742
4	0.5792	9.814	10.784	10.089	18.644	19.241
4	0.5802	24.227	21.813	24.403	13.906	17.593
4	0.5812	16.804	17.824	16.868	7.850	10.156
4	0.5822	13.363	14.465	13.664	16.177	10.251
4	0.5832	21.570	22.322	19.713	14.805	16.188
4	0.5842	26.965	21.210	21.151	19.896	25.386
4	0.5852	15.555	17.569	12.128	13.878	17.924
4	0.5862	24.496	19.195	18.069	12.672	5.668
4	0.5872	21.477	20.055	17.133	11.691	12.476
4	0.5882	15.167	18.552	18.116	4.799	1.912
4	0.5892	16.142	22.438	19.645	11.301	22.797
4	0.5902	24.636	18.640	18.618	19.798	10.479
4	0.5912	18.032	11.819	10.589	18.299	16.468
4	0.5922	15.707	9.935	11.174	15.904	25.257
4	0.5932	22.455	20.677	15.943	16.438	24.402
4	0.5942	17.494	16.639	11.267	14.458	20.314
4	0.5952	11.702	13.225	6.430	21.100	22.695
4	0.5962	21.918	21.112	18.498	15.076	17.292
4	0.5972	15.791	20.266	19.576	10.775	12.208
4	0.5982	19.303	24.704	17.062	10.944	20.841
4	0.5992	27.164	31.736	19.751	12.479	11.974
4	0.6002	10.879	12.089	12.213	13.828	11.692
4	0.6012	22.642	17.478	19.670	9.931	10.221
4	0.6022	20.790	16.799	17.384	13.621	26.111
4	0.6032	19.426	23.597	20.383	11.976	17.409
4	0.6042	17.963	20.303	19.486	9.022	20.815
4	0.6052	9.161	13.951	19.802	6.764	19.078
4	0.6062	10.834	17.948	14.458	11.364	10.808
4	0.6072	15.294	28.264	17.068	24.130	19.242
4	0.6082	21.235	26.858	15.594	11.442	12.189
4	0.6092	12.161	24.325	13.862	20.965	15.303
4	0.6102	10.828	23.166	17.696	15.040	22.171
4	0.6112	6.890	11.364	10.582	16.208	14.066
4	0.6122	19.628	15.802	17.618	20.084	9.988
4	0.6132	21.778	21.997	17.359	24.601	12.644
4	0.6142	15.952	19.317	16.344	16.892	14.706
4	0.6152	13.815	12.487	11.668	15.244	18.688
4	0.6162	38.742	46.914	32.656	23.292	17.649
4	0.6172	19.239	36.879	18.705	17.989	12.516
4	0.6182	18.819	17.770	17.604	8.159	19.000
4	0.6192	18.839	19.437	24.638	21.252	17.305
4	0.6202	13.922	20.446	13.979	8.380	11.213
4	0.6212	18.957	26.551	20.406	14.755	16.582
4	0.6222	24.663	32.397	26.890	25.311	12.529
4	0.6232	13.286	17.809	17.222	20.497	16.785
4	0.6242	10.043	12.908	8.218	16.511	17.028

4	0.6252	20.752	21.870	19.742	14.264	14.978
4	0.6262	21.879	27.489	16.490	31.319	24.504
4	0.6272	11.392	12.061	12.610	17.466	9.381
4	0.6282	10.062	19.212	11.101	22.621	4.594
4	0.6292	11.567	30.431	16.650	25.892	19.488
4	0.6302	12.531	17.072	14.831	16.495	17.777
4	0.6312	12.020	17.760	13.884	7.011	19.554
4	0.6322	9.619	15.759	9.295	24.546	10.507
4	0.6332	17.452	19.545	13.118	24.817	10.212
4	0.6342	15.538	17.788	12.124	22.443	7.681
4	0.6352	20.926	14.779	12.623	22.475	18.371
4	0.6362	13.665	16.216	11.731	19.232	8.931
4	0.6372	14.735	24.062	17.657	24.325	9.057
4	0.6382	14.532	17.112	9.063	20.718	5.954
4	0.6392	12.618	18.706	12.684	13.078	12.759
4	0.6402	16.483	21.784	19.231	25.640	11.526
4	0.6412	14.125	26.823	22.841	23.350	17.011
4	0.6422	13.602	12.379	13.560	12.288	15.533
4	0.6432	19.584	16.264	12.834	14.609	9.317
4	0.6442	13.758	25.874	15.657	29.805	15.643
4	0.6452	15.687	21.098	16.511	16.944	13.700
4	0.6462	5.620	11.418	6.955	9.961	13.263
4	0.6472	18.964	17.076	13.750	14.624	20.644
4	0.6482	23.870	23.875	18.822	14.943	20.340
4	0.6492	23.449	26.004	18.637	17.142	8.887
4	0.6502	17.922	16.575	14.232	14.934	7.213
4	0.6512	21.633	24.537	17.876	17.051	13.990
4	0.6522	13.101	18.852	13.467	17.687	11.008
4	0.6532	12.451	20.775	19.872	15.482	14.463
4	0.6542	14.990	19.892	15.043	19.116	8.781
4	0.6552	28.124	16.439	15.065	17.210	25.551
4	0.6562	19.739	21.270	17.992	19.494	22.300
4	0.6572	17.945	20.220	17.533	22.056	17.477
4	0.6582	7.217	10.834	6.494	10.724	6.749
4	0.6592	13.368	10.677	7.054	13.176	8.158
4	0.6602	14.473	15.890	12.842	13.817	12.325
4	0.6612	23.274	27.986	24.919	33.424	14.464
4	0.6622	25.851	21.470	19.660	31.774	14.362
4	0.6632	13.111	15.008	13.386	26.202	9.033
4	0.6642	16.489	19.393	10.278	32.421	11.625
4	0.6652	16.598	19.944	14.750	25.592	9.240
4	0.6662	19.033	18.191	15.274	16.099	9.648
4	0.6672	22.391	19.946	19.568	17.734	13.775
4	0.6682	14.676	16.377	12.091	26.522	13.699
4	0.6692	18.090	18.635	14.096	27.559	9.507
4	0.6702	19.352	20.704	16.538	22.661	15.903
4	0.6712	22.718	18.208	19.929	25.052	19.640
4	0.6722	7.792	6.287	6.529	12.806	16.991
4	0.6732	11.477	9.826	10.105	23.786	20.084
4	0.6742	9.148	17.568	12.071	18.930	22.709

4	0.6752	12.758	26.950	19.020	22.015	24.698
4	0.6762	15.467	21.888	21.558	19.538	16.625
4	0.6772	19.900	18.977	17.978	14.309	22.463
4	0.6782	17.397	18.831	14.280	28.786	34.988
4	0.6792	8.140	22.119	12.109	18.786	30.582
4	0.6802	12.921	24.188	12.562	11.920	31.626
4	0.6812	17.786	24.177	15.130	17.857	12.276
4	0.6822	12.870	17.766	8.793	15.142	9.394
4	0.6832	8.752	15.984	10.908	20.942	11.422
4	0.6842	9.454	14.938	9.911	24.938	7.990
4	0.6852	12.252	17.458	11.263	27.278	24.726
4	0.6862	13.399	20.881	11.476	34.313	13.571
4	0.6872	13.431	20.873	11.482	34.269	13.610
4	0.6882	12.344	22.069	12.578	25.090	11.603
4	0.6892	17.648	20.809	17.903	26.249	15.105
4	0.6902	26.305	27.572	25.093	28.001	25.213
4	0.6912	3.960	13.636	2.230	22.394	24.323
4	0.6922	8.903	14.393	5.779	10.869	25.122
4	0.6932	13.147	15.487	13.395	12.299	13.737
4	0.6942	6.798	13.179	5.856	16.854	12.456
4	0.6952	9.105	7.864	8.377	15.730	11.815
4	0.6962	15.540	19.607	13.322	9.684	11.098
4	0.6972	12.912	13.174	10.927	17.808	14.983
4	0.6982	8.839	13.170	11.693	17.339	11.665
4	0.6992	16.350	14.530	15.138	23.333	14.590
4	0.7002	18.139	14.500	16.630	18.372	18.914
4	0.7012	10.916	12.601	15.652	20.137	11.526
4	0.7022	6.181	9.431	8.164	13.965	13.614
4	0.7032	12.998	17.876	15.927	22.383	16.323
4	0.7042	12.698	18.594	11.500	29.786	19.160
4	0.7052	18.907	27.457	19.181	22.476	20.867
4	0.7062	12.766	14.578	11.192	15.813	16.117
4	0.7072	12.213	16.264	12.941	13.682	8.670
4	0.7082	11.264	11.757	10.752	10.583	12.550
4	0.7092	22.478	27.141	23.513	23.497	25.140
4	0.7102	5.076	9.501	7.906	17.168	10.176
4	0.7112	9.686	4.780	6.872	13.530	16.335
4	0.7122	10.730	13.021	12.913	13.223	14.527
4	0.7132	16.079	18.228	19.016	22.703	10.079
4	0.7142	16.251	16.500	14.579	22.424	14.766
4	0.7152	6.046	10.600	7.490	10.875	3.830
4	0.7162	5.816	10.101	10.474	16.342	13.444
4	0.7172	9.452	16.435	11.099	21.110	15.649
4	0.7182	6.744	10.698	11.923	9.132	9.710
4	0.7192	10.862	9.213	13.135	7.793	19.531
4	0.7202	11.985	20.221	13.030	15.729	8.834
4	0.7212	11.198	8.872	6.689	13.173	12.627
4	0.7222	10.231	7.485	10.619	8.128	8.163
4	0.7232	13.266	9.257	13.920	9.213	7.146
4	0.7242	20.080	16.377	19.126	13.700	14.773

4	0.7252	10.676	11.708	7.096	6.790	12.186
4	0.7262	11.708	9.500	6.951	12.030	12.851
4	0.7272	11.649	7.604	7.019	10.589	9.954
4	0.7282	7.263	20.322	5.719	26.185	19.445
4	0.7292	15.424	15.906	11.256	24.554	16.628
4	0.7302	7.287	7.639	4.795	11.555	6.951
4	0.7312	7.690	12.607	12.181	11.124	4.203
4	0.7322	7.962	8.903	7.329	7.519	10.070
4	0.7332	5.969	5.575	5.020	9.438	7.177
4	0.7342	10.330	11.790	10.361	7.259	6.644
4	0.7352	23.176	20.014	22.920	19.639	9.243
4	0.7362	8.552	16.481	14.394	14.848	5.323
4	0.7372	10.708	17.092	17.166	13.113	7.340
4	0.7382	12.406	16.413	16.545	13.136	14.370
4	0.7392	7.140	12.088	11.700	8.080	11.545
4	0.7402	4.908	11.704	9.881	7.271	7.439
4	0.7412	6.982	7.014	3.281	6.043	9.797
4	0.7422	8.950	8.175	5.411	8.689	18.888
4	0.7432	6.301	8.509	6.715	9.261	7.456
4	0.7442	10.489	3.695	9.486	11.957	28.259
4	0.7452	19.740	12.771	20.024	11.023	8.972
4	0.7462	15.025	15.515	14.462	9.821	15.713
4	0.7472	12.341	11.585	6.868	5.909	13.441
4	0.7482	26.288	24.080	22.047	8.065	17.645
4	0.7492	12.307	14.100	10.095	12.079	14.324
4	0.7502	20.566	15.623	15.523	6.414	15.905
4	0.7512	12.942	17.010	16.054	12.311	18.264
4	0.7522	8.073	7.218	7.305	10.908	23.150
4	0.7532	13.400	18.288	15.982	8.870	11.694
4	0.7542	13.272	13.885	13.860	9.185	11.570
4	0.7552	15.523	11.840	12.637	19.414	12.826
4	0.7562	12.394	14.246	12.990	13.950	25.254
4	0.7572	9.167	13.587	11.476	12.179	15.225
4	0.7582	13.069	13.602	10.267	12.415	17.074
4	0.7592	12.473	5.626	12.821	3.184	11.621
4	0.7602	13.534	17.359	15.823	19.837	16.836
4	0.7612	12.339	13.595	11.792	13.669	10.600
4	0.7622	10.831	12.769	14.874	14.455	14.208
4	0.7632	14.569	19.035	19.239	16.095	9.179
4	0.7642	15.931	15.784	16.553	15.001	9.605
4	0.7652	10.371	8.076	11.614	15.816	8.082
4	0.7662	15.350	15.948	17.939	14.668	5.634
4	0.7672	8.088	18.217	16.757	21.046	11.041
4	0.7682	13.376	14.834	13.807	7.973	11.455
4	0.7692	14.919	19.500	14.499	17.014	9.529
4	0.7702	14.991	19.540	14.549	17.014	9.522
4	0.7712	13.241	16.576	20.276	3.685	4.114
4	0.7722	14.613	12.149	19.478	3.390	13.981
4	0.7732	19.093	13.476	14.384	10.488	12.267
4	0.7742	7.767	6.750	6.673	9.002	12.321

4	0.7752	7.730	7.474	10.882	7.591	8.961
4	0.7762	9.317	10.858	11.355	15.736	18.702
4	0.7772	16.419	9.985	13.698	10.340	9.247
4	0.7782	13.295	12.540	12.457	10.094	5.443
4	0.7792	6.262	5.595	7.528	4.755	6.109
4	0.7802	1.979	5.889	2.467	6.334	1.259
4	0.7812	5.146	6.621	2.481	3.808	5.735
4	0.7822	11.777	10.396	9.762	9.821	12.436
4	0.7832	8.162	8.692	8.127	6.221	10.975
4	0.7842	6.940	8.371	12.346	5.880	8.197
4	0.7852	10.696	10.823	13.904	0.740	1.978
4	0.7862	9.169	8.554	10.635	6.023	4.264
4	0.7872	6.100	5.802	2.630	5.711	10.833
4	0.7882	13.215	10.487	17.453	10.177	8.587
4	0.7892	12.123	11.576	12.285	18.355	15.581
4	0.7902	9.043	8.098	7.451	19.206	9.869
4	0.7912	25.727	21.191	20.679	8.728	8.927
4	0.7922	9.484	9.487	12.254	4.002	7.836
4	0.7932	17.420	13.627	18.410	12.900	10.311
4	0.7942	9.290	3.967	13.704	6.963	10.853
4	0.7952	10.676	6.981	15.551	5.570	10.417
4	0.7962	15.227	15.250	15.383	8.407	19.945
4	0.7972	15.666	13.879	16.901	13.994	21.981
4	0.7982	6.921	9.025	10.659	14.532	13.672
4	0.7992	6.176	11.043	14.285	11.332	16.654
4	0.8002	10.421	10.476	10.546	12.102	11.688
4	0.8012	7.481	6.985	6.590	15.526	22.149
4	0.8022	6.750	6.441	5.845	13.338	17.750
4	0.8032	15.550	12.523	14.100	14.077	11.934
4	0.8042	13.666	9.438	11.608	11.931	9.429
4	0.8052	13.374	13.282	13.003	19.502	14.166
4	0.8062	14.769	9.931	15.093	8.080	10.870
4	0.8072	11.047	5.871	9.601	5.724	7.011
4	0.8082	5.723	8.050	5.177	8.961	8.952
4	0.8092	11.625	5.174	6.835	7.594	14.582
4	0.8102	12.072	11.378	9.349	11.118	14.976
4	0.8112	7.461	13.469	11.439	9.013	7.626
4	0.8122	18.560	17.343	19.247	10.779	12.835
4	0.8132	16.697	20.349	14.680	13.090	15.098
4	0.8142	15.272	16.560	11.810	8.627	16.191
4	0.8152	10.498	12.927	8.280	8.471	9.391
4	0.8162	15.971	10.727	14.810	26.989	21.510
4	0.8172	14.589	14.909	13.564	13.697	12.768
4	0.8182	7.595	7.789	7.829	13.840	10.681
4	0.8192	14.103	11.097	16.442	22.801	13.989
4	0.8202	13.987	10.873	13.850	11.501	20.928
4	0.8212	8.699	11.213	10.549	15.781	11.785
4	0.8222	7.800	9.774	8.002	11.526	7.176
4	0.8232	7.475	9.698	8.392	22.105	11.618
4	0.8242	4.505	7.940	4.032	13.792	11.524

4	0.8252	3.882	8.191	8.649	9.609	11.492
4	0.8262	14.045	14.382	15.462	11.491	10.690
4	0.8272	14.908	14.878	9.651	18.643	19.103
4	0.8282	5.477	13.536	5.225	18.302	11.861
4	0.8292	6.770	15.299	8.315	24.174	7.073
4	0.8302	3.825	10.518	6.630	11.782	4.817
4	0.8312	9.670	15.269	9.578	12.203	3.078
4	0.8322	11.107	14.184	8.243	12.820	3.926
4	0.8332	12.636	14.340	12.664	20.226	7.306
4	0.8342	19.860	20.160	16.066	24.653	20.449
4	0.8352	20.460	25.177	19.549	15.473	12.507
4	0.8362	13.240	8.527	10.380	14.624	11.155
4	0.8372	16.861	19.825	13.072	10.660	15.970
4	0.8382	15.485	8.593	11.517	11.784	15.266
4	0.8392	15.400	8.577	11.450	11.722	15.264
4	0.8402	11.265	9.423	8.749	14.473	17.327
4	0.8412	6.012	16.743	11.662	9.883	13.998
4	0.8422	6.009	16.749	11.631	9.930	14.112
4	0.8432	19.669	12.288	18.526	10.926	14.771
4	0.8442	19.445	19.481	13.450	14.278	24.757
4	0.8452	15.594	11.133	12.426	22.675	29.753
4	0.8462	25.809	24.766	18.353	22.407	22.032
4	0.8472	6.368	12.333	8.186	28.883	19.059
4	0.8482	20.956	18.357	14.378	17.818	26.138
4	0.8492	15.674	16.613	10.526	34.518	17.926
4	0.8502	17.301	16.953	16.092	23.759	23.724
4	0.8512	15.697	18.534	12.185	22.394	25.064
4	0.8522	8.590	11.382	11.965	18.961	17.319
4	0.8532	7.850	9.746	9.531	10.600	10.441
4	0.8542	22.465	22.354	13.859	25.032	19.918
4	0.8552	11.318	9.116	11.453	13.308	11.614
4	0.8562	13.962	16.487	16.663	17.400	20.058
4	0.8572	11.080	11.816	11.496	10.650	16.599
4	0.8582	21.546	18.885	15.981	20.541	21.569
4	0.8592	24.627	31.013	25.246	16.133	19.325
4	0.8602	14.638	19.558	12.879	12.624	21.091
4	0.8612	8.440	5.328	5.480	11.374	17.686
4	0.8622	9.383	10.977	10.209	11.682	12.906
4	0.8632	12.602	13.412	12.314	16.707	10.074
4	0.8642	4.010	3.692	6.669	6.799	5.824
4	0.8652	12.669	10.303	10.320	14.860	16.132
4	0.8662	18.161	15.435	11.627	9.274	18.205
4	0.8672	12.975	12.098	7.660	7.393	14.829
4	0.8682	15.564	17.373	16.728	12.584	16.299
4	0.8692	17.071	8.702	14.638	20.296	24.754
4	0.8702	16.256	15.555	13.731	12.669	16.350
4	0.8712	19.562	19.295	22.157	10.418	11.632
4	0.8722	6.396	10.549	11.894	16.347	18.795
4	0.8732	10.077	8.298	12.092	13.616	14.116
4	0.8742	8.436	10.854	11.698	11.046	9.511

4	0.8752	29.488	30.835	28.992	14.053	12.923
4	0.8762	27.341	32.734	30.495	13.368	17.634
4	0.8772	20.703	21.768	21.476	11.922	18.025
4	0.8782	18.811	16.907	22.523	6.185	12.706
4	0.8792	15.546	16.898	20.150	5.635	11.591
4	0.8802	22.944	20.127	23.327	16.462	17.276
4	0.8812	23.612	29.926	29.338	8.387	17.331
4	0.8822	17.032	16.449	19.240	7.616	10.136
4	0.8832	16.407	13.141	14.125	13.363	10.311
4	0.8842	16.846	20.520	16.812	8.457	12.880
4	0.8852	17.826	21.938	21.332	6.831	14.434
4	0.8862	19.573	18.387	22.787	13.015	20.863
4	0.8872	12.927	15.878	21.340	13.579	9.683
4	0.8882	20.202	24.404	20.233	8.234	8.952
4	0.8892	14.888	13.090	16.785	14.771	10.718
4	0.8902	31.811	29.749	28.124	11.523	7.361
4	0.8912	27.215	27.241	23.607	13.616	11.676
4	0.8922	14.597	16.174	20.653	13.501	16.415
4	0.8932	8.601	14.014	12.770	13.441	17.214
4	0.8942	14.095	18.646	14.904	17.744	7.467
4	0.8952	16.791	18.887	18.178	6.461	5.850
4	0.8962	17.920	20.674	22.065	10.200	7.646
4	0.8972	16.200	16.656	17.661	5.951	11.709
4	0.8982	14.607	13.994	15.526	9.128	11.529
4	0.8992	12.701	14.599	15.997	7.909	10.578
4	0.9002	20.868	25.147	23.869	10.378	17.937
4	0.9012	8.321	13.195	17.868	13.569	27.184
4	0.9022	9.563	13.922	19.684	16.020	22.305
4	0.9032	9.611	14.035	19.736	16.081	22.407
4	0.9042	6.313	9.656	10.180	11.880	16.570
4	0.9052	6.954	9.784	7.175	13.429	12.215
4	0.9062	11.406	11.786	16.475	6.136	13.296
4	0.9072	12.386	12.162	13.674	8.715	8.425
4	0.9082	13.941	12.686	12.759	4.963	5.771
4	0.9092	14.496	17.279	21.061	8.460	19.042
4	0.9102	19.332	17.684	23.923	18.053	17.923
4	0.9112	13.575	8.008	13.809	11.075	13.079
4	0.9122	13.254	11.536	16.459	7.091	6.431
4	0.9132	18.104	17.715	24.161	8.691	10.539
4	0.9142	3.767	2.535	5.990	3.723	11.214
4	0.9152	6.806	9.349	11.749	3.046	14.288
4	0.9162	7.963	11.804	8.445	3.918	13.151
4	0.9172	7.062	9.189	9.707	6.801	4.529
4	0.9182	5.930	6.572	8.977	6.313	4.618
4	0.9192	1.579	4.735	4.019	5.319	4.439
4	0.9202	4.084	10.526	8.797	8.259	22.660
4	0.9212	22.725	19.650	26.550	10.309	31.834
4	0.9222	14.411	18.113	18.648	14.798	19.303
4	0.9232	8.882	15.366	12.863	8.692	11.706
4	0.9242	10.964	20.282	16.942	17.257	14.797

4	0.9252	4.838	7.186	5.005	10.100	6.884
4	0.9262	4.525	4.927	5.787	2.066	2.850
4	0.9272	7.060	6.977	10.252	5.150	5.706
4	0.9282	9.809	12.008	13.692	9.192	12.442
4	0.9292	9.157	10.035	10.478	11.472	6.763
4	0.9302	9.677	8.490	12.818	4.915	5.919
4	0.9312	30.142	25.661	31.407	18.376	19.875
4	0.9322	16.888	18.808	19.254	8.958	9.681
4	0.9332	11.190	18.026	22.009	10.121	8.107
4	0.9342	8.263	11.969	15.636	9.918	4.320
4	0.9352	23.598	18.621	22.299	14.357	9.984
4	0.9362	18.626	20.538	28.120	9.617	21.664
4	0.9372	15.847	15.310	19.569	7.425	10.689
4	0.9382	19.446	16.904	20.046	14.819	8.174
4	0.9392	9.583	13.487	11.763	12.800	5.971
4	0.9402	10.822	16.914	16.021	15.163	6.312
4	0.9412	16.841	24.359	28.234	14.699	11.092
4	0.9422	14.498	22.538	16.644	26.262	6.378
4	0.9432	6.530	12.256	7.407	14.585	4.669
4	0.9442	5.462	7.319	8.860	10.252	9.783
4	0.9452	6.636	8.222	6.769	15.169	25.163
4	0.9462	16.900	19.510	15.311	16.985	12.833
4	0.9472	10.787	12.808	11.308	14.683	10.540
4	0.9482	9.124	12.430	7.129	18.338	14.948
4	0.9492	13.613	20.353	15.562	23.759	22.855
4	0.9502	12.097	13.374	9.884	11.983	5.933
4	0.9512	16.729	22.661	11.417	9.933	9.008
4	0.9522	11.711	15.698	14.769	13.963	8.132
4	0.9532	6.624	17.475	12.782	17.673	7.046
4	0.9542	12.745	21.560	17.426	14.285	9.982
4	0.9552	24.393	28.064	31.205	18.713	18.147
4	0.9562	25.790	34.568	30.787	14.461	13.909
4	0.9572	15.311	18.006	18.264	21.590	15.516
4	0.9582	20.430	19.134	27.340	11.727	13.512
4	0.9592	27.860	31.011	27.561	20.004	10.089
4	0.9602	27.147	39.099	32.294	19.902	7.949
4	0.9612	16.065	25.552	16.862	15.890	7.139
4	0.9622	21.333	30.898	19.634	22.784	19.304
4	0.9632	12.811	20.218	9.233	14.842	16.388
4	0.9642	7.679	6.947	4.655	8.998	6.053
4	0.9652	4.254	4.122	3.520	12.911	15.056
4	0.9662	11.731	19.193	12.299	12.434	15.787
4	0.9672	14.399	17.958	15.562	22.703	14.261
4	0.9682	21.084	18.087	18.396	21.656	15.665
4	0.9692	28.094	29.544	23.239	20.768	15.877
4	0.9702	23.976	19.231	22.616	13.632	8.968
4	0.9712	21.770	19.634	21.897	9.011	8.936
4	0.9722	4.196	6.405	6.117	1.959	7.589
4	0.9732	4.743	7.458	6.567	1.312	8.596
4	0.9742	7.644	10.925	8.417	3.573	13.706

4	0.9752	9.881	11.343	8.109	7.443	10.786
4	0.9762	5.573	4.303	3.994	2.915	5.910
4	0.9772	34.066	35.773	33.294	14.895	12.166
4	0.9782	17.909	23.583	17.226	13.347	9.879
4	0.9792	11.268	18.196	6.988	17.536	10.203
4	0.9802	23.222	24.030	22.352	18.259	15.715
4	0.9812	21.910	21.371	24.232	11.858	11.153
4	0.9822	8.841	14.429	9.111	21.855	10.601
4	0.9832	11.369	14.003	13.677	16.352	6.235
4	0.9842	16.122	26.647	18.814	23.713	9.634
4	0.9852	14.495	23.698	14.297	21.375	8.205
4	0.9862	12.662	27.825	14.623	26.710	6.456
4	0.9872	23.623	28.350	22.049	25.806	23.263
4	0.9882	26.481	27.572	27.230	12.681	12.637
4	0.9892	12.281	11.228	10.442	1.273	4.447
4	0.9902	8.794	8.187	7.964	14.251	10.052
4	0.9912	13.544	19.964	14.382	16.427	9.888
4	0.9922	3.166	12.407	5.450	12.813	3.217
4	0.9932	3.301	9.018	5.029	5.735	7.610
4	0.9942	8.452	11.273	14.337	21.357	20.329
4	0.9952	9.580	19.942	11.724	29.793	23.443
4	0.9962	15.820	20.343	13.470	19.389	15.761
4	0.9972	15.349	18.007	11.352	12.688	9.181
4	0.9982	6.394	9.657	4.950	7.186	10.179
4	0.9992	16.315	15.325	13.221	9.156	3.563
4	1.0002	5.230	6.399	7.574	6.994	2.168
4	1.0012	10.292	12.460	11.001	11.752	6.426
4	1.0022	19.954	24.973	21.043	19.788	21.256
4	1.0032	16.124	21.105	15.949	21.393	21.190
4	1.0042	14.715	20.368	17.140	19.749	11.855
4	1.0052	11.322	15.947	12.390	15.827	7.630
4	1.0062	11.235	10.748	13.044	28.546	8.623
4	1.0072	21.872	36.312	30.790	22.704	21.650
4	1.0082	21.635	28.022	25.626	21.315	16.680
4	1.0092	21.772	24.724	20.622	18.413	11.270
4	1.0102	15.581	21.024	18.513	14.218	7.968
4	1.0112	13.930	18.480	20.128	14.994	6.271
4	1.0122	10.333	11.962	12.776	10.555	1.379
4	1.0132	11.626	17.229	16.455	11.605	5.757
4	1.0142	20.811	37.350	26.660	19.519	16.816
4	1.0152	18.130	24.143	21.552	11.926	7.688
4	1.0162	5.912	9.686	9.331	11.482	5.360
4	1.0172	12.053	18.585	15.837	22.630	7.745
4	1.0182	24.503	27.279	24.709	21.943	6.476
4	1.0192	9.187	7.551	10.037	11.323	23.791
4	1.0202	11.235	12.392	12.841	7.070	10.560
4	1.0212	8.675	10.260	13.645	10.980	18.558
4	1.0222	21.176	31.863	24.858	30.261	19.281
4	1.0232	27.238	31.326	23.006	32.576	24.417
4	1.0242	15.128	17.848	15.717	36.653	11.524

4	1.0252	19.532	18.457	23.356	39.882	27.273
4	1.0262	19.303	18.305	23.106	39.718	27.390
4	1.0272	22.961	27.200	25.400	48.346	17.347
4	1.0282	23.127	27.253	25.583	48.275	17.532
4	1.0292	21.082	13.780	19.216	24.416	28.475
4	1.0302	10.966	7.250	10.056	20.507	21.848
4	1.0312	9.132	8.327	10.098	14.655	17.931
4	1.0322	20.468	16.046	18.832	15.685	20.380
4	1.0332	24.595	9.941	27.023	20.336	7.645
4	1.0342	16.486	9.031	20.804	25.745	10.838
4	1.0352	12.092	18.294	11.592	22.909	11.411
4	1.0362	14.629	12.494	13.674	26.723	18.322
4	1.0372	15.419	13.608	11.830	25.724	21.918
4	1.0382	20.345	16.652	22.401	16.533	18.965
4	1.0392	20.393	16.794	22.423	16.440	18.988
4	1.0402	20.451	16.936	22.451	16.356	19.012
4	1.0412	10.658	13.090	13.151	23.643	8.689
4	1.0422	13.718	12.373	11.437	13.918	4.093
4	1.0432	14.714	16.275	14.884	16.670	11.112
4	1.0442	19.656	21.152	26.235	12.657	15.662
4	1.0452	23.076	26.013	24.031	12.399	22.929
4	1.0462	12.600	15.934	8.916	22.896	11.800
4	1.0472	10.574	18.755	10.524	20.987	13.337
4	1.0482	5.764	18.566	10.427	14.086	10.828
4	1.0492	11.976	19.365	9.806	17.852	9.916
4	1.0502	10.144	13.270	11.740	12.978	16.702
4	1.0512	9.157	14.752	10.354	12.081	17.436
4	1.0522	11.937	15.793	12.793	10.639	16.988
4	1.0532	16.823	14.662	13.966	12.421	21.598
4	1.0542	17.074	14.856	14.078	12.229	21.818
4	1.0552	16.219	17.875	12.874	9.927	22.491
4	1.0562	8.909	13.363	5.668	12.296	11.492
4	1.0572	31.291	21.151	20.929	13.820	11.659
4	1.0582	18.603	23.423	13.398	27.153	18.099
4	1.0592	21.610	21.967	15.283	12.917	24.218
4	1.0602	14.270	18.686	14.328	16.191	7.639
4	1.0612	15.558	17.423	10.923	23.076	21.455
4	1.0622	15.913	22.328	13.687	17.454	10.661
4	1.0632	8.608	12.454	6.583	10.660	15.800
4	1.0642	19.012	23.236	16.289	11.465	13.116
4	1.0652	18.982	23.265	16.092	11.325	13.165
4	1.0662	17.782	25.779	19.844	15.325	15.419
4	1.0672	15.356	31.726	19.135	16.837	9.791
4	1.0682	11.092	13.776	10.247	15.463	13.522
4	1.0692	21.063	21.700	19.890	11.362	14.401
4	1.0702	12.120	14.229	16.004	27.986	9.974
4	1.0712	14.010	25.559	18.613	19.241	7.775
4	1.0722	12.973	11.121	13.316	23.955	22.485
4	1.0732	14.837	17.161	13.499	13.452	14.039
4	1.0742	19.852	15.216	11.293	12.628	12.198

4	1.0752	23.941	21.680	24.791	23.427	12.965
4	1.0762	19.472	23.812	21.202	13.456	14.956
4	1.0772	15.518	15.119	11.548	24.095	19.846
4	1.0782	16.018	20.798	16.639	25.515	10.580
4	1.0792	11.373	16.808	12.846	16.017	8.799
4	1.0802	11.231	16.237	14.530	20.987	19.660
4	1.0812	18.732	21.659	18.517	11.138	20.344
4	1.0822	18.888	18.480	18.020	17.752	5.543
4	1.0832	15.509	16.453	15.539	23.725	17.295
4	1.0842	8.737	12.348	10.474	20.990	15.091
4	1.0852	10.737	12.322	12.155	19.044	11.416
4	1.0862	7.106	9.067	7.982	18.898	6.236
4	1.0872	13.595	13.151	14.040	18.545	16.521
4	1.0882	11.620	10.542	11.770	13.616	19.806
4	1.0892	10.920	8.923	11.571	6.265	5.512
4	1.0902	11.757	10.512	8.792	10.731	15.506
4	1.0912	5.126	7.803	4.855	23.563	24.922
4	1.0922	11.429	17.246	10.719	24.310	18.574
4	1.0932	15.232	14.789	15.116	14.803	17.230
4	1.0942	9.217	11.307	10.869	12.123	7.472
4	1.0952	13.143	18.346	14.003	14.145	10.033
4	1.0962	20.896	21.554	16.697	24.505	17.277
4	1.0972	21.008	21.598	16.755	24.712	17.622
4	1.0982	20.251	18.370	14.316	17.293	11.702
4	1.0992	20.340	18.380	14.366	17.315	11.873
4	1.1002	11.362	6.074	6.483	8.935	10.306
4	1.1012	7.705	14.546	9.382	17.269	13.397
4	1.1022	7.592	14.418	9.280	17.277	13.514
4	1.1032	11.099	14.787	10.653	35.785	16.833
4	1.1042	8.924	14.318	9.403	18.668	6.491
4	1.1052	6.580	20.186	11.485	24.191	23.392
4	1.1062	4.538	10.198	5.559	21.098	24.085
4	1.1072	11.559	10.357	10.204	15.751	32.877
4	1.1082	8.776	12.817	6.115	12.656	20.779
4	1.1092	11.497	6.406	5.844	7.431	9.999
4	1.1102	14.448	6.314	4.437	10.410	22.771
4	1.1112	13.509	14.038	8.979	22.750	38.005
4	1.1122	9.347	13.187	9.610	11.398	21.905
4	1.1132	11.635	7.736	7.015	12.434	16.700
4	1.1142	15.151	21.437	20.999	11.594	16.099
4	1.1152	5.558	13.551	3.925	20.907	16.367
4	1.1162	14.164	15.280	9.629	21.712	19.754
4	1.1172	11.762	12.936	7.151	12.943	19.513
4	1.1182	7.747	15.533	7.822	13.063	13.032
4	1.1192	10.310	13.903	10.840	18.259	17.352
4	1.1202	3.507	7.622	4.940	22.642	12.354
4	1.1212	9.088	11.623	7.303	23.382	12.377
4	1.1222	6.458	6.295	6.032	12.382	10.989
4	1.1232	5.609	9.981	7.212	15.674	18.697
4	1.1242	3.172	7.959	5.083	12.902	12.945

4	1.1252	5.355	9.299	6.291	13.686	9.083
4	1.1262	10.461	12.748	10.795	11.353	11.231
4	1.1272	9.973	13.118	10.616	11.829	6.024
4	1.1282	6.202	7.026	7.525	8.331	18.201
4	1.1292	5.068	9.644	9.404	13.658	16.442
4	1.1302	6.712	12.054	6.482	13.048	11.040
4	1.1312	4.058	12.488	7.138	19.371	13.231
4	1.1322	8.592	15.726	9.445	20.360	6.297
4	1.1332	6.974	11.644	10.364	9.837	12.690
4	1.1342	4.564	4.830	4.935	11.970	14.648
4	1.1352	10.945	7.794	8.691	12.031	14.892
4	1.1362	7.864	8.070	7.418	9.691	17.109
4	1.1372	7.955	8.285	7.508	9.449	16.149
4	1.1382	6.957	4.023	4.942	7.623	9.530
4	1.1392	5.360	13.459	8.900	17.738	4.669
4	1.1402	4.120	6.226	9.536	8.444	16.682
4	1.1412	5.101	12.211	9.594	15.798	14.448
4	1.1422	4.423	9.921	7.599	19.137	19.862
4	1.1432	4.978	10.200	6.338	14.195	13.576
4	1.1442	11.052	12.563	11.345	5.678	8.526
4	1.1452	11.042	14.861	11.762	14.632	18.038
4	1.1462	7.807	11.535	10.371	14.756	18.475
4	1.1472	14.092	13.775	14.905	8.754	3.827
4	1.1482	15.964	17.536	14.789	15.765	10.665
4	1.1492	15.937	17.462	14.781	15.716	10.768
4	1.1502	8.494	12.196	14.105	6.489	4.691
4	1.1512	10.855	16.085	16.511	18.823	21.814
4	1.1522	7.363	12.241	14.308	19.995	26.097
4	1.1532	8.974	13.672	9.909	25.336	14.963
4	1.1542	10.807	8.250	8.413	18.638	14.920
4	1.1552	7.555	9.097	7.028	9.927	6.432
4	1.1562	5.370	3.951	7.589	5.000	12.971
4	1.1572	10.624	9.834	9.135	7.957	14.179
4	1.1582	9.313	8.611	6.448	10.885	21.362
4	1.1592	0.000	0.000	0.000	0.000	0.000
5	0.0010	0.000	0.000	0.000	0.000	0.000
5	0.0020	0.000	0.000	0.000	0.000	0.000
5	0.0030	19.825	15.471	19.573	18.098	18.898
5	0.0040	12.762	5.631	10.963	15.497	17.199
5	0.0050	10.431	16.002	12.263	11.873	7.420
5	0.0060	9.330	16.062	15.176	20.489	12.235
5	0.0070	12.028	13.644	12.635	20.423	10.062
5	0.0080	7.767	8.113	8.582	23.662	16.075
5	0.0090	7.398	8.949	6.792	25.275	19.520
5	0.0100	5.915	10.626	8.570	17.714	5.679
5	0.0110	14.048	14.042	18.790	20.554	20.930
5	0.0120	20.729	16.145	23.057	13.559	20.923
5	0.0130	10.009	5.897	9.717	22.132	16.617
5	0.0140	11.631	5.826	7.799	18.977	15.698
5	0.0150	4.713	6.941	8.948	17.373	21.303

5	0.0160	6.932	9.114	4.434	16.718	11.947
5	0.0170	6.702	8.646	5.204	10.640	17.556
5	0.0180	13.376	11.812	13.124	22.596	13.148
5	0.0190	11.815	12.170	14.260	20.406	11.673
5	0.0200	13.071	12.136	17.504	24.959	10.870
5	0.0210	12.112	13.495	12.931	6.693	16.699
5	0.0220	13.741	9.757	14.156	28.167	26.636
5	0.0230	12.594	14.022	11.283	25.776	20.022
5	0.0240	13.210	14.398	11.352	17.099	15.471
5	0.0250	5.776	4.970	7.797	15.859	11.513
5	0.0260	7.553	10.403	9.735	12.273	24.667
5	0.0270	9.562	6.449	6.715	13.884	34.479
5	0.0280	8.267	6.236	7.433	11.709	32.256
5	0.0290	12.324	6.471	9.477	5.126	9.116
5	0.0300	10.788	5.849	5.171	4.324	15.752
5	0.0310	14.750	11.433	11.083	10.100	20.718
5	0.0320	11.757	11.769	10.552	9.287	10.468
5	0.0330	9.038	5.149	8.961	4.633	11.313
5	0.0340	14.221	8.659	10.393	6.157	10.371
5	0.0350	7.962	7.614	8.473	10.974	16.760
5	0.0360	23.536	18.446	16.033	12.968	18.231
5	0.0370	13.087	13.310	11.466	9.318	14.290
5	0.0380	13.105	13.136	10.997	9.306	13.971
5	0.0390	14.931	7.888	11.063	4.130	19.516
5	0.0400	3.574	7.518	4.175	10.650	12.117
5	0.0410	7.806	7.748	6.159	18.014	14.919
5	0.0420	7.760	13.008	10.192	27.400	9.740
5	0.0430	6.763	6.211	4.852	21.780	18.031
5	0.0440	7.736	5.472	5.603	10.638	16.004
5	0.0450	8.370	6.519	5.909	4.395	23.183
5	0.0460	12.872	10.074	10.031	14.995	18.681
5	0.0470	20.128	20.421	16.498	14.763	11.490
5	0.0480	12.321	13.027	9.425	9.770	10.142
5	0.0490	10.942	17.049	14.806	20.819	9.688
5	0.0500	13.451	8.694	10.380	16.261	17.427
5	0.0510	15.242	6.225	10.232	14.701	20.645
5	0.0520	12.697	14.261	12.682	10.465	10.956
5	0.0530	16.589	11.436	11.884	18.786	24.992
5	0.0540	16.713	11.568	11.928	18.748	24.937
5	0.0550	18.777	9.559	15.635	13.772	15.069
5	0.0560	14.224	7.779	10.332	30.136	31.621
5	0.0570	18.499	12.140	13.953	25.568	27.814
5	0.0580	22.056	17.992	15.592	17.111	18.543
5	0.0590	17.958	11.037	17.563	19.292	14.814
5	0.0600	17.963	11.041	17.552	19.271	14.875
5	0.0610	18.097	6.639	14.340	12.643	29.974
5	0.0620	25.400	18.747	19.616	13.763	19.334
5	0.0630	16.336	13.061	14.175	12.383	19.225
5	0.0640	15.875	9.241	16.388	11.911	23.426
5	0.0650	20.241	14.815	14.006	27.692	19.800

5	0.0660	21.906	11.820	13.139	9.325	25.343
5	0.0670	18.767	14.244	14.351	15.776	20.480
5	0.0680	28.664	21.578	23.150	10.951	25.330
5	0.0690	21.120	11.602	9.259	13.005	20.677
5	0.0700	17.896	4.370	8.264	22.517	20.613
5	0.0710	18.457	8.834	11.142	16.129	21.693
5	0.0720	12.844	11.072	14.762	19.447	18.774
5	0.0730	20.608	9.211	13.313	10.945	14.067
5	0.0740	23.440	8.713	12.626	14.718	8.564
5	0.0750	13.757	9.126	12.767	13.452	7.543
5	0.0760	15.757	13.852	16.572	18.716	8.591
5	0.0770	15.055	11.518	11.974	25.250	20.216
5	0.0780	22.861	15.917	26.055	21.345	14.068
5	0.0790	23.272	17.865	22.644	14.674	9.733
5	0.0800	23.724	12.240	20.665	19.784	25.229
5	0.0810	14.450	15.603	14.100	25.579	10.545
5	0.0820	18.177	13.280	14.503	25.472	16.830
5	0.0830	20.341	11.802	10.313	9.807	11.821
5	0.0840	13.045	10.818	12.097	14.731	20.740
5	0.0850	18.030	13.045	14.493	33.138	13.711
5	0.0860	16.012	10.349	13.931	28.664	22.700
5	0.0870	11.334	6.396	10.328	22.045	10.985
5	0.0880	17.699	6.610	16.236	16.887	13.547
5	0.0890	17.959	6.777	15.090	13.277	10.557
5	0.0900	10.029	5.056	8.227	18.186	8.987
5	0.0910	24.066	10.355	12.682	19.983	13.568
5	0.0920	17.792	8.043	7.788	36.161	13.697
5	0.0930	11.578	7.498	12.763	19.720	12.283
5	0.0940	16.619	13.294	15.754	15.255	18.636
5	0.0950	14.810	13.129	18.448	6.304	13.393
5	0.0960	18.287	17.927	18.120	20.857	15.179
5	0.0970	14.602	17.519	22.261	13.825	15.326
5	0.0980	11.045	14.278	20.538	6.430	6.912
5	0.0990	10.385	13.504	11.219	15.442	15.935
5	0.1000	10.774	16.413	10.999	20.362	12.913
5	0.1010	7.440	9.886	8.595	11.899	11.661
5	0.1020	11.438	2.992	3.888	10.809	15.738
5	0.1030	20.738	25.210	25.072	23.195	22.852
5	0.1040	9.604	13.794	8.996	27.338	17.508
5	0.1050	9.558	16.094	11.883	17.017	8.067
5	0.1060	13.710	10.723	14.367	7.213	13.702
5	0.1070	18.121	17.477	21.495	12.055	11.522
5	0.1080	7.541	4.289	4.003	10.222	9.908
5	0.1090	18.130	16.644	18.602	15.157	10.800
5	0.1100	17.202	15.680	17.109	29.148	20.789
5	0.1110	13.059	11.097	16.312	17.444	18.271
5	0.1120	13.224	12.538	14.667	25.211	13.898
5	0.1130	17.237	15.216	22.543	15.899	11.211
5	0.1140	10.969	8.249	13.289	8.649	7.899
5	0.1150	19.008	16.732	21.959	17.823	17.100

5	0.1160	16.342	14.951	19.382	19.823	14.918
5	0.1170	13.259	13.261	20.210	9.967	13.782
5	0.1180	18.741	13.687	22.017	15.893	17.565
5	0.1190	13.158	8.879	11.583	19.939	18.063
5	0.1200	15.160	12.575	18.800	14.972	8.408
5	0.1210	10.974	14.601	20.522	9.729	19.537
5	0.1220	9.261	20.139	19.548	18.292	15.856
5	0.1230	14.281	16.591	16.334	19.018	12.378
5	0.1240	17.234	15.337	17.480	17.354	17.050
5	0.1250	13.443	16.128	14.317	18.454	18.463
5	0.1260	7.941	15.673	12.638	19.212	9.895
5	0.1270	22.094	16.535	28.610	11.617	11.116
5	0.1280	19.996	19.932	29.655	15.061	12.732
5	0.1290	17.654	14.869	18.378	10.487	8.176
5	0.1300	24.485	20.152	23.666	12.113	6.952
5	0.1310	18.643	7.787	13.560	14.571	10.652
5	0.1320	18.203	9.412	13.145	17.236	10.300
5	0.1330	21.467	8.668	14.710	14.669	9.182
5	0.1340	12.087	13.345	16.340	21.827	10.531
5	0.1350	7.174	13.040	9.317	23.955	11.711
5	0.1360	7.146	13.005	9.340	23.857	11.733
5	0.1370	13.231	9.627	11.298	16.136	10.441
5	0.1380	14.456	9.574	11.575	14.049	9.589
5	0.1390	10.881	10.651	10.541	26.424	13.709
5	0.1400	11.433	15.633	13.271	38.142	15.460
5	0.1410	12.472	12.108	15.674	13.921	12.263
5	0.1420	11.257	18.340	20.216	23.332	17.267
5	0.1430	7.390	9.310	6.992	21.995	7.603
5	0.1440	12.496	10.291	15.671	10.905	21.788
5	0.1450	5.389	9.115	11.995	14.342	15.686
5	0.1460	18.993	13.438	17.172	10.360	20.756
5	0.1470	12.180	11.619	13.177	14.389	9.412
5	0.1480	11.439	14.365	15.252	18.062	8.600
5	0.1490	10.801	10.565	10.401	12.729	9.237
5	0.1500	2.507	7.661	7.286	19.491	21.481
5	0.1510	6.871	9.648	7.412	15.937	10.818
5	0.1520	8.297	12.503	10.134	19.135	15.349
5	0.1530	16.982	21.995	21.172	17.584	24.212
5	0.1540	14.932	16.745	15.718	20.067	19.231
5	0.1550	8.258	14.353	11.805	10.344	24.679
5	0.1560	3.233	11.618	9.696	10.488	13.490
5	0.1570	9.869	7.629	9.284	14.807	14.486
5	0.1580	7.639	5.952	8.223	9.889	10.315
5	0.1590	7.667	5.977	8.177	9.876	10.375
5	0.1600	12.512	10.961	12.803	26.757	18.885
5	0.1610	7.823	8.288	8.106	14.222	16.174
5	0.1620	11.533	9.681	10.372	24.256	16.926
5	0.1630	13.563	8.671	13.512	28.643	23.586
5	0.1640	16.099	14.784	19.900	10.694	19.079
5	0.1650	8.750	10.495	9.746	14.136	18.994

5	0.1660	23.421	20.341	24.192	20.891	15.331
5	0.1670	12.547	15.366	14.699	18.619	12.464
5	0.1680	15.306	13.890	15.814	17.434	11.948
5	0.1690	11.550	19.034	13.827	26.564	19.578
5	0.1700	12.992	10.685	13.458	26.445	19.596
5	0.1710	9.760	18.510	13.812	21.013	17.676
5	0.1720	7.589	17.587	11.164	15.119	4.638
5	0.1730	13.289	20.004	18.622	8.692	8.335
5	0.1740	15.082	23.165	18.576	23.168	15.688
5	0.1750	17.676	10.224	12.701	20.187	20.010
5	0.1760	17.335	17.310	19.974	23.642	24.214
5	0.1770	14.685	14.995	15.061	17.112	16.440
5	0.1780	9.001	4.625	8.078	15.211	15.117
5	0.1790	8.063	12.038	14.814	19.599	26.699
5	0.1800	11.263	9.408	13.677	8.381	16.514
5	0.1810	15.403	12.911	12.300	18.257	19.967
5	0.1820	9.958	10.124	9.677	15.095	9.000
5	0.1830	9.944	10.124	9.676	15.074	9.000
5	0.1840	18.375	11.328	16.772	10.367	22.941
5	0.1850	11.239	6.639	14.575	13.485	7.211
5	0.1860	11.062	6.512	13.834	17.917	9.400
5	0.1870	8.657	12.978	17.420	16.939	17.389
5	0.1880	13.535	18.878	18.551	15.355	18.614
5	0.1890	4.367	9.666	12.385	15.180	25.569
5	0.1900	13.757	19.708	20.237	10.209	17.747
5	0.1910	15.135	11.115	15.694	12.743	20.423
5	0.1920	11.725	11.313	14.889	10.758	31.191
5	0.1930	9.091	10.606	10.101	9.652	25.147
5	0.1940	9.036	10.539	10.020	9.645	25.169
5	0.1950	10.021	10.584	12.583	12.913	19.102
5	0.1960	9.388	11.733	14.024	12.934	20.131
5	0.1970	16.085	9.775	11.168	15.273	23.084
5	0.1980	23.052	20.552	17.504	17.938	34.597
5	0.1990	25.522	21.902	16.559	14.925	29.765
5	0.2000	11.073	13.290	14.973	10.333	14.277
5	0.2010	15.752	19.035	19.466	19.386	25.585
5	0.2020	9.078	13.027	13.086	13.781	16.575
5	0.2030	10.221	13.183	10.852	16.421	31.749
5	0.2040	10.098	13.230	10.828	16.451	31.774
5	0.2050	10.016	13.040	10.890	19.282	24.152
5	0.2060	18.285	18.180	18.540	13.195	24.892
5	0.2070	4.105	5.810	6.156	13.076	22.538
5	0.2080	4.326	7.253	9.130	13.672	17.943
5	0.2090	5.516	6.461	9.139	9.863	15.704
5	0.2100	15.225	13.145	13.996	18.096	25.128
5	0.2110	9.473	10.242	10.942	14.365	27.439
5	0.2120	13.915	6.970	10.451	14.193	31.333
5	0.2130	5.438	10.300	9.930	19.203	25.846
5	0.2140	2.993	5.434	6.889	25.296	37.105
5	0.2150	8.580	13.723	17.287	26.227	37.161

5	0.2160	24.678	19.679	21.078	23.136	39.468
5	0.2170	6.908	9.288	12.646	28.602	42.011
5	0.2180	17.074	15.982	14.649	19.770	35.431
5	0.2190	21.370	20.084	15.694	17.284	26.822
5	0.2200	13.725	10.732	13.085	21.554	16.461
5	0.2210	12.320	10.421	17.981	16.070	28.607
5	0.2220	19.381	16.467	20.386	24.944	30.889
5	0.2230	18.919	15.401	20.469	14.311	25.590
5	0.2240	13.995	8.611	8.529	29.376	40.644
5	0.2250	6.230	8.689	9.697	22.581	26.471
5	0.2260	12.271	9.844	8.217	34.585	37.274
5	0.2270	23.522	15.424	11.045	25.879	33.995
5	0.2280	17.841	23.564	23.211	23.348	24.265
5	0.2290	8.470	9.766	13.222	5.100	16.586
5	0.2300	12.228	16.424	17.890	18.268	27.844
5	0.2310	14.346	16.576	20.594	24.347	41.367
5	0.2320	7.382	15.543	15.671	27.466	22.546
5	0.2330	10.676	16.069	12.095	31.682	27.561
5	0.2340	12.597	12.167	11.602	22.874	23.079
5	0.2350	16.944	18.594	18.862	26.521	23.293
5	0.2360	10.509	12.446	10.533	24.202	25.826
5	0.2370	9.356	15.609	8.232	23.129	36.390
5	0.2380	15.901	15.021	13.445	21.759	25.818
5	0.2390	17.244	20.910	15.643	21.340	25.151
5	0.2400	7.584	16.343	7.557	22.714	17.737
5	0.2410	5.526	18.088	8.914	23.360	27.941
5	0.2420	6.926	11.096	7.300	22.668	28.956
5	0.2430	10.268	13.127	13.813	22.297	33.856
5	0.2440	13.301	18.564	17.591	25.487	30.194
5	0.2450	10.510	16.595	14.996	32.918	29.721
5	0.2460	10.653	16.745	15.248	32.589	29.735
5	0.2470	10.449	12.221	8.640	28.121	33.089
5	0.2480	12.461	10.962	6.755	22.426	46.916
5	0.2490	12.288	12.598	8.324	10.802	25.023
5	0.2500	5.900	5.399	8.606	13.456	34.252
5	0.2510	6.478	7.824	11.326	27.686	28.784
5	0.2520	8.571	10.224	7.091	27.566	26.335
5	0.2530	7.187	8.064	6.054	22.570	31.697
5	0.2540	19.629	17.595	14.699	22.476	41.664
5	0.2550	9.586	13.530	14.009	16.664	34.910
5	0.2560	10.448	10.435	12.462	20.027	31.726
5	0.2570	11.106	13.220	17.058	11.334	19.950
5	0.2580	14.199	15.231	21.438	15.833	9.453
5	0.2590	5.310	8.893	10.534	15.338	19.239
5	0.2600	5.312	8.878	10.536	15.358	19.266
5	0.2610	11.420	22.281	18.212	33.361	37.022
5	0.2620	4.266	16.491	8.450	30.125	30.312
5	0.2630	9.073	14.719	15.065	27.452	26.343
5	0.2640	13.062	22.717	21.086	37.814	31.702
5	0.2650	8.107	14.568	7.451	15.007	23.009

5	0.2660	10.544	13.470	12.659	15.109	11.787
5	0.2670	5.332	10.149	8.876	26.789	28.922
5	0.2680	6.520	8.332	6.771	17.653	25.831
5	0.2690	12.442	22.892	13.544	24.740	20.189
5	0.2700	7.189	8.337	6.752	25.217	22.063
5	0.2710	5.801	12.519	6.315	15.072	11.703
5	0.2720	18.107	23.420	20.977	19.273	21.318
5	0.2730	16.884	17.523	14.392	19.243	23.201
5	0.2740	13.685	11.221	9.492	11.581	15.254
5	0.2750	4.045	8.425	7.712	6.640	15.720
5	0.2760	8.273	10.554	6.215	26.308	21.170
5	0.2770	8.504	14.367	13.177	29.591	14.889
5	0.2780	8.276	19.664	13.777	24.664	22.734
5	0.2790	4.124	20.796	10.079	32.889	24.521
5	0.2800	4.765	8.864	6.771	20.432	28.993
5	0.2810	5.996	9.170	8.731	9.019	10.916
5	0.2820	9.725	14.735	11.773	22.596	13.590
5	0.2830	10.049	16.120	19.034	19.681	14.354
5	0.2840	7.780	11.559	12.340	9.232	11.071
5	0.2850	6.143	15.006	9.992	22.265	13.201
5	0.2860	13.323	20.790	16.010	13.383	13.815
5	0.2870	10.796	17.371	12.091	19.736	17.161
5	0.2880	10.801	17.368	12.085	19.743	17.162
5	0.2890	15.075	18.330	13.058	16.963	4.563
5	0.2900	7.987	16.270	10.040	22.864	12.692
5	0.2910	11.332	8.815	9.957	14.854	13.136
5	0.2920	9.544	13.270	9.288	11.958	12.515
5	0.2930	19.265	16.509	15.317	8.277	11.083
5	0.2940	8.262	12.955	10.435	30.722	25.960
5	0.2950	11.450	21.740	16.532	26.309	25.377
5	0.2960	6.847	18.555	11.608	30.972	22.047
5	0.2970	9.730	20.300	13.569	27.210	21.465
5	0.2980	4.648	9.193	5.338	23.028	22.322
5	0.2990	8.928	14.364	10.214	13.008	13.603
5	0.3000	6.374	16.321	7.037	23.514	16.544
5	0.3010	9.041	21.822	16.737	20.778	21.586
5	0.3020	10.909	24.911	21.044	15.204	11.111
5	0.3030	12.850	18.399	17.563	17.071	17.781
5	0.3040	13.446	13.433	15.103	16.858	13.538
5	0.3050	15.309	21.881	17.367	28.676	28.213
5	0.3060	15.887	25.201	19.396	30.949	27.554
5	0.3070	3.684	17.751	12.750	23.819	21.827
5	0.3080	8.403	19.077	18.101	22.714	20.446
5	0.3090	5.817	4.076	5.499	13.663	18.493
5	0.3100	9.148	10.889	5.557	14.892	23.334
5	0.3110	10.923	20.153	19.464	15.236	22.961
5	0.3120	12.127	22.690	16.902	22.597	34.154
5	0.3130	11.259	22.081	16.453	27.424	38.324
5	0.3140	10.330	17.906	11.394	29.694	29.986
5	0.3150	10.327	17.859	11.370	29.586	29.991

5	0.3160	5.245	14.279	14.351	29.722	45.530
5	0.3170	17.855	20.407	19.952	33.034	59.097
5	0.3180	17.604	13.517	16.270	16.394	36.540
5	0.3190	15.858	18.567	17.592	37.659	25.690
5	0.3200	15.870	18.574	17.598	37.672	25.683
5	0.3210	17.425	13.911	15.277	18.491	32.941
5	0.3220	12.740	15.284	13.865	17.353	40.629
5	0.3230	11.424	10.298	10.344	20.888	37.365
5	0.3240	11.712	17.806	11.827	18.546	19.736
5	0.3250	15.435	18.037	17.680	28.120	24.373
5	0.3260	11.980	21.558	14.833	18.851	32.484
5	0.3270	10.729	17.352	15.671	19.832	22.883
5	0.3280	9.974	10.371	12.521	19.065	33.835
5	0.3290	7.096	11.005	9.998	22.523	26.129
5	0.3300	1.093	10.591	9.559	16.123	10.738
5	0.3310	4.464	12.696	8.877	16.846	10.147
5	0.3320	10.303	13.686	9.782	17.291	16.209
5	0.3330	17.086	13.702	7.107	22.846	18.053
5	0.3340	20.995	16.201	12.454	14.413	14.698
5	0.3350	14.533	14.021	12.922	9.334	15.445
5	0.3360	8.401	15.183	14.276	20.661	14.752
5	0.3370	20.056	18.790	13.753	23.025	25.399
5	0.3380	8.014	13.327	10.547	32.620	22.786
5	0.3390	18.067	27.455	16.433	30.729	21.114
5	0.3400	19.079	20.559	17.923	34.090	28.028
5	0.3410	15.960	16.119	16.725	19.413	13.178
5	0.3420	17.688	18.663	20.227	17.643	11.455
5	0.3430	13.620	18.356	19.166	20.444	9.920
5	0.3440	5.791	14.098	14.326	13.844	8.679
5	0.3450	6.077	9.457	12.138	2.205	4.353
5	0.3460	13.398	15.235	11.688	17.846	23.171
5	0.3470	13.797	11.244	15.630	17.973	27.874
5	0.3480	11.886	16.912	10.848	19.695	10.580
5	0.3490	8.309	5.517	4.168	24.675	18.909
5	0.3500	5.691	9.914	5.522	25.522	14.173
5	0.3510	12.323	13.280	9.398	19.103	9.409
5	0.3520	5.498	12.056	7.311	25.356	8.661
5	0.3530	14.222	24.190	15.267	22.190	9.474
5	0.3540	17.197	22.253	15.936	13.559	13.234
5	0.3550	14.436	11.674	8.339	19.587	15.057
5	0.3560	15.755	13.147	6.402	16.870	16.851
5	0.3570	15.786	12.962	6.292	17.166	16.482
5	0.3580	10.120	15.336	9.322	16.352	13.972
5	0.3590	6.662	10.198	6.478	15.454	9.554
5	0.3600	12.108	10.948	7.217	14.345	13.953
5	0.3610	7.588	9.697	8.331	10.750	11.564
5	0.3620	5.569	6.401	3.938	11.845	10.423
5	0.3630	12.693	10.497	6.704	19.116	13.524
5	0.3640	7.641	5.425	8.127	12.482	4.872
5	0.3650	7.249	8.365	8.848	16.089	7.951

5	0.3660	9.242	16.385	10.059	17.858	8.545
5	0.3670	12.548	14.935	9.674	19.749	17.743
5	0.3680	9.674	16.048	11.972	20.889	18.726
5	0.3690	10.238	10.684	11.750	16.008	11.515
5	0.3700	11.819	16.521	16.724	15.013	7.186
5	0.3710	6.177	5.140	10.799	7.365	5.455
5	0.3720	3.160	9.990	8.507	26.243	14.558
5	0.3730	13.734	22.807	16.557	25.925	15.861
5	0.3740	10.672	10.052	10.184	14.464	17.007
5	0.3750	11.267	15.901	10.916	17.061	21.371
5	0.3760	11.731	10.113	7.722	6.232	7.575
5	0.3770	10.184	22.815	15.630	22.108	26.799
5	0.3780	11.954	15.762	14.179	20.736	25.248
5	0.3790	13.298	13.110	10.598	12.754	15.789
5	0.3800	13.179	10.273	12.030	11.424	19.232
5	0.3810	17.187	14.552	11.175	19.069	11.547
5	0.3820	14.467	21.364	23.660	15.242	19.844
5	0.3830	17.912	15.351	19.120	20.538	25.873
5	0.3840	14.480	14.328	11.024	26.857	34.081
5	0.3850	12.428	15.898	13.877	20.047	14.790
5	0.3860	16.056	20.565	19.193	19.906	8.611
5	0.3870	14.870	19.790	23.015	21.365	12.771
5	0.3880	14.801	19.689	22.934	21.524	12.663
5	0.3890	17.372	12.808	10.105	15.244	15.417
5	0.3900	16.239	13.255	13.193	10.314	11.104
5	0.3910	7.242	9.393	7.125	12.944	10.831
5	0.3920	7.256	9.394	7.107	12.805	10.900
5	0.3930	7.273	9.396	7.103	12.664	10.969
5	0.3940	10.061	12.530	11.882	10.101	8.706
5	0.3950	11.153	17.382	17.837	15.308	11.420
5	0.3960	12.523	10.386	17.207	12.473	10.969
5	0.3970	17.702	14.614	24.326	11.437	11.812
5	0.3980	17.841	14.725	24.575	11.272	11.842
5	0.3990	17.985	14.832	24.816	11.128	11.861
5	0.4000	18.133	14.935	25.042	11.013	11.861
5	0.4010	18.492	17.723	12.506	20.645	13.140
5	0.4020	18.517	17.691	12.478	20.643	13.139
5	0.4030	18.543	17.658	12.452	20.639	13.137
5	0.4040	12.457	12.218	7.238	16.783	16.345
5	0.4050	12.470	12.118	7.129	16.793	16.354
5	0.4060	12.482	12.016	7.026	16.796	16.369
5	0.4070	16.511	12.462	12.630	11.065	16.284
5	0.4080	12.275	13.356	19.116	10.317	21.295
5	0.4090	17.767	17.132	20.158	18.812	21.613
5	0.4100	17.833	17.147	20.260	18.810	21.541
5	0.4110	14.464	12.124	13.847	11.893	14.987
5	0.4120	27.965	20.175	23.300	15.332	7.780
5	0.4130	13.561	13.173	8.477	13.283	12.695
5	0.4140	7.784	9.345	7.160	7.182	11.674
5	0.4150	13.616	10.551	15.732	8.926	15.410

5	0.4160	19.725	11.461	20.472	12.044	18.433
5	0.4170	10.705	10.288	10.041	15.304	10.020
5	0.4180	21.430	24.809	21.042	18.916	14.877
5	0.4190	7.478	12.745	5.516	14.207	10.573
5	0.4200	4.355	3.923	3.740	11.350	5.941
5	0.4210	9.457	15.319	11.230	18.578	10.605
5	0.4220	10.697	8.020	8.042	18.056	10.253
5	0.4230	2.179	7.716	2.552	11.784	2.220
5	0.4240	5.907	8.516	1.453	23.167	19.536
5	0.4250	4.627	5.307	2.968	11.204	9.891
5	0.4260	9.132	11.714	7.742	4.120	14.111
5	0.4270	11.316	16.038	11.445	15.655	19.840
5	0.4280	10.828	13.322	11.879	17.506	9.544
5	0.4290	4.758	12.656	9.782	17.418	15.088
5	0.4300	8.959	17.867	13.425	14.455	10.397
5	0.4310	14.553	22.524	12.099	19.702	8.064
5	0.4320	13.709	12.732	15.810	12.555	9.950
5	0.4330	10.102	17.816	9.809	25.697	13.693
5	0.4340	4.425	9.064	7.411	13.530	19.092
5	0.4350	12.308	12.224	13.871	7.605	16.360
5	0.4360	12.914	15.850	15.138	12.878	18.230
5	0.4370	6.385	7.644	9.138	17.995	15.533
5	0.4380	7.391	12.391	10.794	22.316	13.441
5	0.4390	19.685	24.270	17.936	19.621	17.068
5	0.4400	6.765	16.353	13.258	22.669	15.469
5	0.4410	14.530	16.584	19.893	13.906	16.280
5	0.4420	10.134	14.489	12.094	12.772	8.487
5	0.4430	16.252	14.253	12.930	17.685	8.497
5	0.4440	7.252	13.419	11.680	18.732	19.750
5	0.4450	11.875	11.276	13.269	20.833	15.129
5	0.4460	16.272	15.556	15.211	19.039	15.412
5	0.4470	6.040	8.959	4.925	20.796	15.669
5	0.4480	8.238	10.505	9.067	15.708	9.559
5	0.4490	7.885	7.671	11.600	12.444	15.725
5	0.4500	5.900	13.097	9.895	19.322	13.861
5	0.4510	16.705	19.809	18.705	15.963	13.174
5	0.4520	9.071	15.009	6.179	23.811	13.904
5	0.4530	25.163	22.452	22.403	16.978	15.716
5	0.4540	23.831	22.615	17.511	14.764	19.278
5	0.4550	18.307	18.642	16.489	15.571	16.393
5	0.4560	18.220	17.580	20.022	20.156	17.294
5	0.4570	13.040	11.879	12.091	19.002	14.637
5	0.4580	18.908	17.498	18.756	21.869	14.675
5	0.4590	9.350	13.618	9.759	14.377	12.034
5	0.4600	9.438	7.287	6.875	13.008	9.466
5	0.4610	10.060	4.889	8.265	12.619	11.348
5	0.4620	6.359	5.567	7.479	20.010	7.585
5	0.4630	11.961	10.453	10.322	16.168	10.087
5	0.4640	6.493	5.594	8.164	15.774	10.051
5	0.4650	8.954	3.493	8.838	20.239	11.945

5	0.4660	10.244	9.937	7.048	17.111	11.749
5	0.4670	13.189	10.319	8.779	15.783	12.007
5	0.4680	8.028	5.559	9.914	18.583	4.660
5	0.4690	12.083	10.891	14.219	13.111	5.274
5	0.4700	17.183	11.222	13.247	8.457	8.275
5	0.4710	10.727	11.435	11.459	12.318	16.960
5	0.4720	11.582	5.879	8.710	10.106	7.571
5	0.4730	10.279	8.482	9.961	11.871	5.797
5	0.4740	20.202	16.967	18.779	26.962	16.789
5	0.4750	15.833	14.278	17.730	24.814	6.021
5	0.4760	5.535	8.235	6.451	16.506	10.390
5	0.4770	5.993	13.077	12.334	17.589	12.824
5	0.4780	5.995	13.066	12.316	17.438	12.846
5	0.4790	4.429	5.832	5.684	4.149	8.173
5	0.4800	2.595	4.799	7.268	5.325	6.120
5	0.4810	9.072	9.519	9.299	10.070	12.372
5	0.4820	20.394	15.160	15.658	16.131	9.968
5	0.4830	27.483	14.954	15.266	12.320	18.532
5	0.4840	16.465	13.327	14.713	18.972	13.108
5	0.4850	13.934	15.745	12.899	12.685	17.528
5	0.4860	16.247	22.752	14.619	14.286	8.900
5	0.4870	17.415	11.444	15.834	18.310	11.889
5	0.4880	17.582	15.206	16.829	14.374	12.481
5	0.4890	11.048	9.351	8.555	18.157	12.922
5	0.4900	7.818	14.959	9.760	12.739	8.500
5	0.4910	17.603	13.578	14.893	9.284	9.458
5	0.4920	6.382	4.757	3.950	15.438	13.321
5	0.4930	13.484	13.146	12.581	13.489	10.561
5	0.4940	13.442	18.988	15.943	15.185	8.950
5	0.4950	20.080	11.255	13.704	7.103	10.026
5	0.4960	3.090	1.360	2.625	7.215	6.816
5	0.4970	21.831	21.783	24.483	10.558	17.693
5	0.4980	11.916	14.217	12.103	9.966	4.391
5	0.4990	6.538	7.246	4.750	4.696	5.102
5	0.5000	9.810	9.712	6.904	17.981	9.283
5	0.5010	6.020	5.218	4.663	11.120	4.939
5	0.5020	8.774	9.096	6.011	10.009	7.115
5	0.5030	11.654	4.368	8.772	13.049	6.188
5	0.5040	8.831	10.553	12.811	5.979	6.566
5	0.5050	10.444	8.676	5.507	2.972	1.926
5	0.5060	15.273	9.340	11.142	12.188	6.888
5	0.5070	11.365	12.318	7.858	14.504	5.553
5	0.5080	12.459	11.127	8.536	13.952	8.060
5	0.5090	12.394	11.282	12.957	12.222	8.179
5	0.5100	16.934	9.141	10.658	18.017	11.085
5	0.5110	21.977	11.845	11.419	10.082	14.043
5	0.5120	16.409	9.569	10.111	10.630	22.720
5	0.5130	13.976	6.914	3.975	8.679	11.335
5	0.5140	17.235	7.043	5.484	13.542	4.873
5	0.5150	9.396	12.404	7.125	17.550	7.091

5	0.5160	13.048	7.719	8.905	15.301	11.997
5	0.5170	15.562	15.212	12.403	20.824	19.478
5	0.5180	8.603	8.304	7.563	8.702	7.891
5	0.5190	3.581	5.923	5.826	10.586	4.114
5	0.5200	5.375	6.773	4.494	8.808	8.385
5	0.5210	18.998	11.041	13.655	18.248	6.598
5	0.5220	7.720	8.117	4.892	12.920	9.646
5	0.5230	15.395	8.204	7.932	14.914	11.109
5	0.5240	9.767	4.149	7.090	3.728	7.774
5	0.5250	12.130	4.332	6.957	3.182	6.181
5	0.5260	6.687	4.231	7.428	3.378	3.560
5	0.5270	7.064	7.700	5.966	2.073	3.738
5	0.5280	4.672	3.372	1.895	7.368	7.978
5	0.5290	4.876	7.177	3.338	14.615	8.131
5	0.5300	4.927	7.231	3.366	14.606	8.165
5	0.5310	14.545	12.999	11.567	9.861	10.161
5	0.5320	9.531	8.333	9.247	7.125	10.291
5	0.5330	8.426	8.260	9.798	14.373	11.527
5	0.5340	16.085	11.361	17.321	22.902	13.824
5	0.5350	16.156	9.829	12.924	14.768	6.589
5	0.5360	12.788	8.600	13.983	6.892	3.957
5	0.5370	20.351	15.787	19.078	7.096	5.548
5	0.5380	14.267	9.980	14.759	3.524	9.318
5	0.5390	10.862	9.684	15.272	2.893	5.843
5	0.5400	9.608	9.407	11.900	5.126	4.560
5	0.5410	21.298	16.860	20.067	8.042	9.256
5	0.5420	17.440	8.574	12.216	3.492	11.418
5	0.5430	14.050	8.427	8.980	6.625	11.413
5	0.5440	11.563	5.956	8.025	3.419	5.300
5	0.5450	18.740	10.491	10.443	3.358	5.490
5	0.5460	18.708	10.824	11.398	2.959	7.374
5	0.5470	16.915	8.513	15.761	4.281	7.453
5	0.5480	23.867	16.086	20.134	7.959	10.537
5	0.5490	13.206	14.906	16.497	9.296	12.755
5	0.5500	10.690	9.043	10.509	4.457	10.425
5	0.5510	13.710	9.452	12.735	5.526	10.054
5	0.5520	4.832	5.757	6.909	6.489	9.616
5	0.5530	4.849	5.742	6.914	6.487	9.608
5	0.5540	7.412	8.609	10.951	10.763	16.117
5	0.5550	13.057	14.249	15.038	5.192	8.007
5	0.5560	12.596	11.221	12.314	4.003	6.185
5	0.5570	16.630	11.941	12.304	5.254	5.364
5	0.5580	9.096	6.569	5.293	10.307	16.206
5	0.5590	8.343	7.405	9.712	6.027	20.708
5	0.5600	17.925	16.215	16.481	3.193	7.426
5	0.5610	13.420	8.761	7.346	16.811	17.657
5	0.5620	12.747	13.361	12.884	18.884	16.290
5	0.5630	10.325	12.783	10.121	15.090	20.971
5	0.5640	12.083	12.143	9.913	10.186	14.624
5	0.5650	18.046	9.442	11.076	8.983	8.426

5	0.5660	15.356	11.792	10.787	9.369	6.859
5	0.5670	11.179	7.429	5.737	3.836	6.349
5	0.5680	23.401	15.200	19.355	5.424	4.797
5	0.5690	7.187	5.763	7.325	8.313	4.035
5	0.5700	12.621	13.347	13.666	12.676	11.379
5	0.5710	12.617	13.350	13.670	12.675	11.377
5	0.5720	12.614	13.353	13.673	12.674	11.375
5	0.5730	23.837	21.427	24.523	12.330	10.083
5	0.5740	32.089	24.724	31.311	11.256	7.580
5	0.5750	21.774	16.453	21.423	10.090	5.829
5	0.5760	21.783	16.459	21.420	10.126	5.827
5	0.5770	24.414	20.165	21.479	9.301	8.372
5	0.5780	24.114	11.140	17.727	18.233	20.955
5	0.5790	24.581	8.608	17.662	5.858	9.280
5	0.5800	18.447	7.102	10.897	6.317	14.390
5	0.5810	9.018	6.522	8.937	2.831	8.049
5	0.5820	14.470	9.778	14.330	5.012	12.042
5	0.5830	10.369	18.674	12.019	10.290	12.184
5	0.5840	14.617	8.139	10.341	5.414	10.421
5	0.5850	18.929	10.470	15.519	3.833	8.507
5	0.5860	15.174	8.920	17.178	9.380	15.156
5	0.5870	21.302	15.141	11.521	9.770	9.300
5	0.5880	17.983	11.037	8.645	9.160	8.663
5	0.5890	16.801	10.277	9.123	7.783	5.658
5	0.5900	13.264	6.765	4.937	3.364	5.787
5	0.5910	15.640	11.553	7.899	3.635	2.689
5	0.5920	9.437	11.036	8.645	17.692	16.953
5	0.5930	12.524	15.989	10.781	11.698	12.208
5	0.5940	14.309	14.409	15.925	8.890	9.906
5	0.5950	14.876	13.332	12.984	5.498	8.252
5	0.5960	28.846	16.210	22.769	14.800	9.966
5	0.5970	14.634	15.760	15.559	10.807	13.453
5	0.5980	8.685	4.667	7.894	5.969	8.509
5	0.5990	8.972	6.847	9.533	5.698	7.671
5	0.6000	14.328	16.264	15.990	11.713	7.179
5	0.6010	14.401	16.401	16.059	11.692	7.187
5	0.6020	9.240	9.875	8.659	16.034	14.476
5	0.6030	11.661	10.395	7.571	13.276	5.889
5	0.6040	12.210	12.367	12.415	3.871	2.062
5	0.6050	9.289	9.808	8.347	10.063	3.326
5	0.6060	10.219	10.447	6.711	8.177	13.744
5	0.6070	16.829	18.491	15.151	14.785	13.518
5	0.6080	11.184	15.834	13.503	24.712	14.217
5	0.6090	9.999	9.028	7.359	11.162	8.690
5	0.6100	12.238	11.200	10.573	4.805	4.903
5	0.6110	19.871	18.669	17.630	14.946	11.187
5	0.6120	16.751	17.619	15.650	14.000	13.667
5	0.6130	12.979	21.835	12.414	20.804	13.080
5	0.6140	9.965	19.296	16.736	18.364	10.676
5	0.6150	14.107	10.367	13.992	9.811	7.232

5	0.6160	14.104	10.503	14.094	9.772	7.223
5	0.6170	24.138	18.420	17.055	14.087	17.229
5	0.6180	18.536	14.892	14.907	12.058	3.799
5	0.6190	9.710	11.090	7.985	6.403	7.907
5	0.6200	8.727	13.616	8.209	11.925	11.540
5	0.6210	18.452	13.152	16.065	10.272	13.512
5	0.6220	15.129	9.476	16.156	14.676	19.473
5	0.6230	12.337	7.066	10.694	11.079	10.957
5	0.6240	10.938	15.166	13.294	12.480	11.724
5	0.6250	15.080	16.736	14.987	11.107	11.931
5	0.6260	11.527	16.862	10.734	18.188	10.199
5	0.6270	18.071	16.865	18.886	7.673	9.199
5	0.6280	23.431	22.729	20.719	5.711	15.578
5	0.6290	18.517	17.795	16.448	13.198	10.007
5	0.6300	13.571	15.565	15.915	13.556	14.748
5	0.6310	7.808	7.579	5.768	10.827	2.980
5	0.6320	10.991	11.078	11.888	19.393	10.415
5	0.6330	29.284	16.549	24.888	10.421	16.750
5	0.6340	22.993	16.124	15.679	13.934	3.610
5	0.6350	24.217	13.638	14.295	11.306	8.407
5	0.6360	11.420	14.888	10.567	7.079	5.681
5	0.6370	6.968	8.276	7.596	5.904	9.038
5	0.6380	15.418	14.757	15.627	6.535	3.828
5	0.6390	8.676	14.838	9.400	10.625	5.725
5	0.6400	8.531	13.807	7.482	10.832	6.160
5	0.6410	17.595	14.787	13.139	10.384	9.179
5	0.6420	15.290	10.646	15.254	6.516	14.896
5	0.6430	7.893	10.613	12.922	10.493	18.369
5	0.6440	9.334	12.281	12.078	12.878	11.856
5	0.6450	18.185	9.075	13.821	7.404	10.127
5	0.6460	18.945	12.768	11.376	4.596	8.727
5	0.6470	8.464	3.425	5.994	5.667	6.433
5	0.6480	14.144	20.053	23.270	18.528	11.419
5	0.6490	17.233	18.940	16.286	23.681	11.333
5	0.6500	6.866	11.340	8.352	21.909	14.425
5	0.6510	8.095	11.514	9.987	12.323	7.215
5	0.6520	7.682	11.793	9.208	19.188	12.892
5	0.6530	6.755	15.239	9.075	15.934	13.927
5	0.6540	5.262	9.561	8.038	13.043	22.791
5	0.6550	4.510	12.332	7.593	19.639	16.226
5	0.6560	4.116	7.985	5.252	7.561	7.563
5	0.6570	5.988	11.647	6.882	7.759	3.714
5	0.6580	6.388	8.911	5.806	8.851	6.629
5	0.6590	7.700	13.089	7.526	16.995	14.690
5	0.6600	27.519	25.072	19.441	21.159	18.821
5	0.6610	11.025	12.362	9.612	24.074	14.291
5	0.6620	19.875	13.695	17.891	9.397	7.372
5	0.6630	15.578	12.330	9.564	12.388	10.073
5	0.6640	18.123	11.443	11.453	12.572	10.956
5	0.6650	16.436	11.119	11.838	18.866	5.251

5	0.6660	14.313	19.619	17.482	9.077	6.426
5	0.6670	12.632	10.087	9.282	7.588	4.269
5	0.6680	10.011	11.593	8.126	5.836	5.470
5	0.6690	12.636	16.883	12.398	15.690	22.331
5	0.6700	14.181	15.406	11.593	13.145	10.896
5	0.6710	10.506	7.388	6.977	11.364	9.503
5	0.6720	7.766	7.794	4.520	4.579	7.898
5	0.6730	10.508	9.683	7.075	13.661	12.459
5	0.6740	11.480	12.578	10.577	20.461	11.174
5	0.6750	11.520	17.608	8.067	19.165	11.936
5	0.6760	7.186	17.859	7.023	19.660	14.000
5	0.6770	9.214	16.182	8.986	17.909	6.175
5	0.6780	10.738	14.006	8.050	20.311	16.375
5	0.6790	9.025	8.787	3.663	12.494	6.690
5	0.6800	5.737	9.843	3.659	11.071	4.442
5	0.6810	5.647	9.789	3.588	11.078	4.403
5	0.6820	3.079	5.063	3.940	4.691	4.131
5	0.6830	2.999	5.030	3.899	4.734	4.150
5	0.6840	10.146	5.786	9.654	20.577	14.729
5	0.6850	4.759	7.530	2.048	22.376	13.145
5	0.6860	9.840	8.706	9.256	7.624	7.410
5	0.6870	9.212	13.008	11.978	11.057	10.769
5	0.6880	9.253	12.242	7.093	10.420	6.637
5	0.6890	22.027	9.926	12.277	13.952	8.314
5	0.6900	13.402	6.341	7.915	6.833	8.605
5	0.6910	13.382	6.327	7.900	6.813	8.626
5	0.6920	10.137	7.071	8.497	4.387	3.930
5	0.6930	5.543	2.926	5.192	1.056	2.182
5	0.6940	6.545	9.791	5.653	15.343	4.078
5	0.6950	19.906	17.441	14.831	8.901	4.899
5	0.6960	19.897	17.438	14.829	8.905	4.897
5	0.6970	17.301	4.952	6.608	19.840	10.985
5	0.6980	13.137	9.426	10.097	16.789	14.021
5	0.6990	15.938	9.298	9.026	10.488	7.676
5	0.7000	13.286	8.302	5.732	7.230	12.294
5	0.7010	4.297	6.949	5.612	15.390	17.052
5	0.7020	11.504	12.102	10.325	17.476	24.982
5	0.7030	11.065	11.342	8.549	17.572	15.548
5	0.7040	10.746	10.902	9.773	15.095	9.011
5	0.7050	9.196	10.868	12.714	15.527	11.803
5	0.7060	11.336	13.145	13.586	14.218	12.330
5	0.7070	10.756	6.733	8.558	5.157	15.850
5	0.7080	19.208	15.545	19.284	10.373	14.497
5	0.7090	13.934	10.864	9.406	12.785	12.614
5	0.7100	7.753	9.327	7.580	9.748	12.865
5	0.7110	24.914	12.268	20.991	12.084	10.281
5	0.7120	27.786	13.448	20.884	12.397	7.211
5	0.7130	22.963	9.795	17.398	14.802	8.667
5	0.7140	7.049	6.104	6.857	8.706	9.198
5	0.7150	7.597	6.529	7.050	4.377	7.250

5	0.7160	4.264	2.653	2.331	4.503	6.413
5	0.7170	6.147	12.094	7.349	9.190	10.968
5	0.7180	2.578	3.640	4.520	2.266	5.324
5	0.7190	2.484	4.039	2.971	2.180	9.235
5	0.7200	2.537	5.902	4.998	5.035	12.009
5	0.7210	8.038	15.962	11.645	11.256	8.402
5	0.7220	11.621	18.030	17.103	6.383	17.454
5	0.7230	7.702	12.335	6.268	5.559	14.340
5	0.7240	16.139	12.825	11.677	7.174	9.629
5	0.7250	9.641	14.012	8.136	7.968	10.197
5	0.7260	10.634	11.883	11.823	10.975	15.860
5	0.7270	3.853	7.861	5.177	12.270	6.814
5	0.7280	16.678	10.004	13.995	5.790	8.026
5	0.7290	14.478	17.062	17.607	11.855	14.472
5	0.7300	14.093	19.519	17.140	15.096	17.244
5	0.7310	19.275	20.234	18.530	14.446	9.393
5	0.7320	13.152	15.680	16.027	14.677	8.283
5	0.7330	6.420	14.216	12.288	9.582	8.452
5	0.7340	11.153	11.138	11.113	9.352	9.004
5	0.7350	13.405	9.567	14.316	12.660	6.889
5	0.7360	4.857	7.202	4.224	15.962	9.162
5	0.7370	5.201	2.910	3.186	1.319	5.019
5	0.7380	6.946	14.253	8.219	22.700	13.912
5	0.7390	4.238	7.482	3.177	5.881	7.243
5	0.7400	4.237	7.475	3.176	5.885	7.269
5	0.7410	13.731	13.186	14.900	8.548	10.826
5	0.7420	7.400	1.829	7.736	16.401	18.528
5	0.7430	6.567	2.874	6.192	15.808	19.125
5	0.7440	7.853	11.944	7.521	19.352	6.253
5	0.7450	7.855	5.820	7.371	12.768	8.403
5	0.7460	3.326	7.133	3.831	14.675	9.828
5	0.7470	4.199	6.061	3.875	6.932	9.428
5	0.7480	10.634	8.890	9.533	5.771	8.925
5	0.7490	6.953	13.725	10.782	8.414	6.164
5	0.7500	6.702	15.804	9.460	14.226	5.164
5	0.7510	2.873	8.087	3.521	19.017	4.402
5	0.7520	1.622	4.053	1.809	10.687	3.519
5	0.7530	6.991	9.969	4.609	10.581	6.886
5	0.7540	5.201	4.539	2.368	15.378	9.807
5	0.7550	6.088	7.451	8.978	5.506	5.080
5	0.7560	7.685	8.660	10.375	8.646	2.911
5	0.7570	11.324	7.258	5.875	8.614	11.632
5	0.7580	13.803	18.868	11.233	9.951	11.029
5	0.7590	25.701	21.481	17.813	5.535	15.961
5	0.7600	12.638	8.512	4.611	8.725	17.076
5	0.7610	8.047	8.823	7.439	6.087	6.713
5	0.7620	5.359	7.253	5.879	9.604	9.057
5	0.7630	8.881	8.444	8.678	15.474	12.981
5	0.7640	9.161	8.552	8.149	13.436	9.134
5	0.7650	6.184	6.116	4.604	17.111	11.855

5	0.7660	8.150	12.147	7.169	12.720	8.361
5	0.7670	10.305	16.751	12.717	18.430	20.480
5	0.7680	9.948	13.173	15.015	7.261	20.020
5	0.7690	9.445	9.709	12.393	9.715	12.893
5	0.7700	13.066	17.398	20.734	6.186	9.910
5	0.7710	2.093	11.125	6.838	10.862	11.513
5	0.7720	2.732	6.119	4.874	9.271	15.158
5	0.7730	10.966	10.415	11.075	9.395	7.363
5	0.7740	11.498	8.107	10.298	14.219	6.750
5	0.7750	19.287	21.545	16.907	10.249	8.132
5	0.7760	9.048	11.374	10.749	11.379	6.061
5	0.7770	10.738	8.555	7.169	3.411	2.866
5	0.7780	21.210	17.255	16.322	12.152	11.291
5	0.7790	20.835	16.251	16.540	3.491	4.953
5	0.7800	8.562	12.427	9.129	9.457	2.565
5	0.7810	11.052	16.454	14.165	15.177	6.360
5	0.7820	13.300	19.229	13.787	12.668	14.787
5	0.7830	14.376	18.272	12.303	14.252	16.506
5	0.7840	20.231	17.866	19.840	9.466	9.294
5	0.7850	16.059	10.229	10.607	9.277	5.037
5	0.7860	11.169	12.345	10.567	5.331	6.524
5	0.7870	22.092	19.673	17.893	7.873	7.782
5	0.7880	11.386	8.329	8.415	7.400	6.054
5	0.7890	4.012	8.017	9.084	4.950	3.338
5	0.7900	11.815	15.569	12.531	8.589	10.620
5	0.7910	9.811	15.667	9.618	9.810	9.629
5	0.7920	17.356	19.933	11.020	15.012	18.494
5	0.7930	22.472	18.280	15.507	15.763	16.320
5	0.7940	18.596	14.728	11.243	26.035	15.596
5	0.7950	12.089	12.979	8.769	18.454	17.249
5	0.7960	19.176	18.754	24.240	12.950	16.353
5	0.7970	26.270	24.398	28.641	16.546	7.729
5	0.7980	21.527	18.052	19.139	8.842	10.383
5	0.7990	21.454	18.025	19.169	8.762	10.278
5	0.8000	21.589	19.682	27.429	14.567	23.075
5	0.8010	20.174	18.185	21.749	9.739	16.711
5	0.8020	29.706	29.324	42.257	11.201	18.827
5	0.8030	9.866	6.299	9.536	3.414	12.005
5	0.8040	7.712	6.584	5.937	3.886	11.194
5	0.8050	18.796	19.765	20.100	7.878	24.279
5	0.8060	23.366	21.465	21.198	16.327	22.563
5	0.8070	10.613	10.474	7.633	5.551	14.091
5	0.8080	6.707	5.650	8.386	2.965	1.506
5	0.8090	11.336	10.259	11.843	2.682	7.614
5	0.8100	12.655	12.659	9.329	13.875	10.822
5	0.8110	11.557	12.507	9.612	10.906	30.134
5	0.8120	24.628	18.861	20.859	10.736	25.700
5	0.8130	18.573	18.728	17.474	7.766	26.174
5	0.8140	35.830	26.599	33.321	23.280	18.596
5	0.8150	19.676	11.336	10.773	18.947	26.016

5	0.8160	22.527	16.815	23.371	16.815	11.866
5	0.8170	16.138	15.128	10.098	19.237	19.360
5	0.8180	11.188	12.673	15.812	10.297	15.081
5	0.8190	11.607	18.486	17.692	13.156	13.570
5	0.8200	23.344	27.746	29.401	14.743	22.765
5	0.8210	16.315	23.608	16.450	12.188	21.082
5	0.8220	24.231	33.334	25.201	11.076	24.323
5	0.8230	11.188	14.786	8.488	8.910	28.137
5	0.8240	11.783	13.025	11.229	18.766	9.312
5	0.8250	23.365	23.149	26.369	12.175	12.407
5	0.8260	10.844	14.374	14.280	13.323	22.249
5	0.8270	23.561	24.254	19.440	11.070	22.089
5	0.8280	12.861	16.310	14.360	10.390	22.132
5	0.8290	22.126	16.343	21.653	15.674	23.414
5	0.8300	12.982	13.813	14.122	9.523	16.876
5	0.8310	17.607	14.048	18.104	13.768	11.893
5	0.8320	23.575	18.970	21.889	13.088	19.667
5	0.8330	8.184	10.650	5.571	12.949	16.673
5	0.8340	8.283	10.686	5.571	13.059	16.978
5	0.8350	20.878	13.788	18.886	5.351	18.661
5	0.8360	16.208	11.894	16.066	4.418	14.692
5	0.8370	13.917	12.937	9.036	10.710	21.268
5	0.8380	10.552	10.046	6.873	6.028	19.002
5	0.8390	25.690	22.341	25.636	9.544	13.673
5	0.8400	25.707	22.347	25.626	9.581	13.722
5	0.8410	18.382	17.480	17.906	12.201	16.820
5	0.8420	15.853	16.648	10.106	10.530	14.942
5	0.8430	13.047	14.848	10.524	16.298	11.232
5	0.8440	14.488	16.634	13.705	15.894	23.106
5	0.8450	10.609	13.091	10.825	10.646	9.788
5	0.8460	8.519	13.330	10.709	15.877	18.787
5	0.8470	8.537	13.352	10.761	15.878	18.824
5	0.8480	15.990	11.880	8.813	10.382	11.132
5	0.8490	14.260	13.391	7.868	9.808	9.482
5	0.8500	11.363	9.903	11.374	7.291	15.806
5	0.8510	11.470	7.126	6.929	7.712	9.141
5	0.8520	21.515	20.165	15.454	12.330	26.140
5	0.8530	8.899	12.465	9.616	9.321	18.440
5	0.8540	9.472	7.840	8.887	7.261	8.492
5	0.8550	17.425	16.424	14.797	14.982	23.614
5	0.8560	7.664	13.651	5.434	10.577	14.074
5	0.8570	14.742	15.805	9.318	10.449	18.972
5	0.8580	13.682	19.375	12.217	14.352	16.181
5	0.8590	7.311	10.258	6.108	9.067	8.305
5	0.8600	2.658	5.376	4.219	13.896	5.853
5	0.8610	8.635	11.900	16.839	13.617	21.084
5	0.8620	17.192	9.302	11.934	14.107	23.803
5	0.8630	11.104	9.425	8.742	19.349	14.764
5	0.8640	14.710	12.714	10.600	14.508	14.140
5	0.8650	13.750	13.183	10.412	12.368	9.354

5	0.8660	22.857	20.831	21.098	12.595	14.153
5	0.8670	21.573	15.581	17.104	13.988	23.018
5	0.8680	8.654	10.867	8.177	15.038	8.404
5	0.8690	4.990	8.543	6.343	24.043	13.954
5	0.8700	10.927	13.493	12.158	12.237	10.036
5	0.8710	12.837	17.738	18.639	13.453	15.137
5	0.8720	9.333	8.367	8.357	22.382	27.394
5	0.8730	4.133	4.885	4.828	14.116	14.958
5	0.8740	13.820	17.632	18.621	15.558	20.381
5	0.8750	11.088	11.666	14.920	13.620	8.096
5	0.8760	13.110	13.716	11.977	16.688	19.036
5	0.8770	9.947	14.512	12.335	15.690	9.030
5	0.8780	8.328	8.194	9.495	16.598	18.434
5	0.8790	8.325	8.862	6.647	17.416	12.892
5	0.8800	8.817	11.422	13.667	14.470	16.147
5	0.8810	19.054	21.114	21.268	18.504	16.908
5	0.8820	12.348	10.322	9.161	21.948	11.379
5	0.8830	10.339	10.705	9.688	9.231	10.017
5	0.8840	9.130	11.841	10.870	17.229	14.394
5	0.8850	8.601	9.579	11.870	21.579	21.847
5	0.8860	20.199	9.750	12.147	18.878	19.044
5	0.8870	16.016	12.176	12.013	11.451	12.417
5	0.8880	11.760	12.747	10.839	11.136	13.601
5	0.8890	20.292	11.867	13.321	6.660	17.718
5	0.8900	13.100	10.756	12.309	8.322	18.989
5	0.8910	20.138	21.087	22.975	14.132	30.149
5	0.8920	22.442	24.249	20.687	6.998	23.599
5	0.8930	10.851	16.662	9.941	13.460	21.004
5	0.8940	9.405	9.991	13.443	10.243	12.160
5	0.8950	21.714	16.381	16.186	9.237	12.506
5	0.8960	25.140	17.948	21.673	21.911	7.656
5	0.8970	7.155	12.728	8.971	14.558	10.295
5	0.8980	2.883	6.996	3.899	11.514	8.034
5	0.8990	6.589	8.196	3.215	7.302	5.043
5	0.9000	14.719	22.558	18.809	22.365	22.899
5	0.9010	11.562	16.700	12.952	13.979	17.743
5	0.9020	9.792	21.531	15.044	27.049	22.058
5	0.9030	13.676	14.342	11.399	14.531	14.641
5	0.9040	13.875	11.209	18.779	9.207	14.470
5	0.9050	10.784	14.762	16.012	13.017	22.400
5	0.9060	15.461	18.882	15.278	20.802	19.299
5	0.9070	21.535	25.437	16.242	20.058	27.144
5	0.9080	13.292	16.002	17.482	14.527	26.247
5	0.9090	17.496	18.156	13.205	18.499	19.397
5	0.9100	9.781	12.697	8.885	13.990	19.142
5	0.9110	11.080	10.258	9.164	9.048	11.342
5	0.9120	13.114	13.372	10.802	16.407	14.940
5	0.9130	9.904	20.935	14.691	18.529	18.487
5	0.9140	13.869	27.544	17.510	27.225	19.704
5	0.9150	14.265	25.994	18.218	15.554	26.675

5	0.9160	15.010	16.930	13.608	11.621	15.944
5	0.9170	8.856	17.245	15.737	8.707	22.095
5	0.9180	12.361	13.852	14.029	9.346	20.676
5	0.9190	12.595	16.711	15.046	13.380	21.453
5	0.9200	10.184	18.123	16.006	24.183	13.645
5	0.9210	12.227	13.949	17.532	9.506	14.130
5	0.9220	9.711	7.828	11.195	9.452	15.868
5	0.9230	12.462	16.121	16.391	11.569	16.885
5	0.9240	13.112	19.875	18.214	22.384	19.023
5	0.9250	17.125	20.986	13.958	17.537	20.649
5	0.9260	19.387	17.803	19.186	16.621	14.457
5	0.9270	27.978	17.249	16.430	15.657	24.751
5	0.9280	14.577	20.572	22.532	15.350	20.989
5	0.9290	4.219	8.121	9.503	13.790	16.778
5	0.9300	16.811	10.077	14.829	11.734	17.543
5	0.9310	17.909	17.100	21.017	18.203	18.820
5	0.9320	16.444	13.583	14.488	12.197	8.093
5	0.9330	23.926	14.641	16.982	10.136	7.505
5	0.9340	9.456	14.413	11.846	14.310	10.556
5	0.9350	11.440	10.477	11.728	11.591	25.195
5	0.9360	15.151	18.481	16.496	25.009	21.270
5	0.9370	10.007	18.203	10.486	20.513	10.318
5	0.9380	10.012	18.222	10.483	20.514	10.299
5	0.9390	10.017	18.240	10.481	20.515	10.279
5	0.9400	10.022	18.258	10.478	20.514	10.258
5	0.9410	15.767	21.556	16.111	16.244	13.143
5	0.9420	16.498	18.761	17.228	10.616	14.909
5	0.9430	16.470	18.493	18.924	23.398	15.119
5	0.9440	12.723	19.228	17.115	23.689	22.884
5	0.9450	9.726	15.834	12.779	20.435	15.220
5	0.9460	16.400	22.908	15.314	20.639	19.850
5	0.9470	11.140	6.581	12.605	9.278	10.631
5	0.9480	10.737	6.707	3.072	4.799	16.227
5	0.9490	13.007	9.482	3.233	11.095	19.535
5	0.9500	5.702	12.571	4.967	14.428	16.024
5	0.9510	8.202	20.071	12.599	22.429	8.459
5	0.9520	13.165	14.067	17.972	21.288	15.710
5	0.9530	20.686	27.623	26.662	11.023	20.937
5	0.9540	20.616	24.433	24.606	25.290	16.886
5	0.9550	11.844	18.120	14.638	21.157	10.213
5	0.9560	15.380	21.241	14.170	26.382	15.195
5	0.9570	7.327	9.983	6.091	25.120	10.781
5	0.9580	18.718	18.328	22.803	20.760	12.286
5	0.9590	11.373	15.335	10.957	29.695	13.748
5	0.9600	12.793	19.606	10.345	26.734	16.132
5	0.9610	9.677	28.355	13.750	37.608	17.782
5	0.9620	4.496	11.542	8.536	24.061	11.189
5	0.9630	5.614	5.172	6.478	11.227	11.712
5	0.9640	7.285	19.895	15.960	18.948	12.378
5	0.9650	10.867	16.957	19.469	11.840	18.339

5	0.9660	9.053	10.241	11.896	4.667	18.702
5	0.9670	9.149	10.328	12.030	4.790	18.830
5	0.9680	8.930	17.332	16.132	19.045	17.924
5	0.9690	7.204	14.562	13.225	15.371	13.496
5	0.9700	5.380	5.619	8.633	7.530	10.015
5	0.9710	11.148	12.292	11.501	9.082	18.940
5	0.9720	15.781	18.735	14.572	10.456	5.440
5	0.9730	7.308	10.017	10.658	14.259	7.594
5	0.9740	14.386	24.835	21.082	19.057	16.494
5	0.9750	7.435	8.369	6.152	19.323	8.224
5	0.9760	8.467	11.911	8.763	21.823	8.614
5	0.9770	10.848	15.076	13.855	20.661	12.938
5	0.9780	7.694	18.725	10.773	25.933	14.665
5	0.9790	3.093	9.700	4.926	12.444	2.799
5	0.9800	3.124	11.401	3.862	14.890	4.747
5	0.9810	15.300	22.823	16.138	20.681	12.269
5	0.9820	11.389	18.512	18.387	16.679	11.785
5	0.9830	11.930	7.655	14.549	11.402	14.574
5	0.9840	7.530	6.644	10.734	12.111	10.296
5	0.9850	7.708	11.688	7.987	15.022	8.987
5	0.9860	6.096	11.714	6.277	20.216	13.567
5	0.9870	4.610	3.472	5.385	9.174	5.982
5	0.9880	4.321	2.098	4.264	8.255	10.468
5	0.9890	7.597	4.306	6.220	7.791	8.529
5	0.9900	8.776	8.119	8.123	11.667	12.073
5	0.9910	12.720	11.982	8.579	14.884	12.784
5	0.9920	8.623	12.381	7.994	13.460	6.185
5	0.9930	11.711	5.459	11.606	12.598	11.601
5	0.9940	7.862	14.351	11.459	23.436	11.190
5	0.9950	9.138	16.241	11.052	24.256	10.918
5	0.9960	15.878	19.033	15.771	22.118	12.757
5	0.9970	6.739	11.197	7.073	12.246	11.933
5	0.9980	13.234	18.378	14.024	17.003	9.542
5	0.9990	10.639	11.603	15.144	17.935	9.867
5	1.0000	8.681	8.876	11.084	14.230	13.830
5	1.0010	7.100	11.832	8.248	23.149	9.258
5	1.0020	5.149	7.871	6.322	10.400	11.069
5	1.0030	5.171	12.730	5.615	19.864	12.219
5	1.0040	8.244	5.921	10.772	16.999	7.815
5	1.0050	12.196	14.266	10.576	32.982	17.963
5	1.0060	14.047	23.868	17.785	17.395	7.660
5	1.0070	8.609	11.896	11.805	12.804	7.781
5	1.0080	2.986	4.306	9.146	10.625	12.103
5	1.0090	7.052	7.862	9.442	10.358	17.648
5	1.0100	13.805	18.266	15.638	14.037	19.971
5	1.0110	21.855	21.797	20.147	15.742	16.058
5	1.0120	24.768	20.992	18.480	9.711	18.892
5	1.0130	16.334	21.188	15.569	14.948	11.001
5	1.0140	9.877	8.802	11.197	12.409	18.438
5	1.0150	6.101	7.670	5.726	12.901	15.334

5	1.0160	6.419	4.822	7.330	9.440	13.478
5	1.0170	15.353	16.995	17.033	20.560	16.714
5	1.0180	15.623	13.679	11.940	18.614	13.730
5	1.0190	10.765	13.423	9.694	12.313	17.555
5	1.0200	4.411	5.033	3.301	14.632	6.387
5	1.0210	8.660	12.826	11.353	15.122	17.408
5	1.0220	11.232	13.401	13.317	9.099	29.772
5	1.0230	11.609	11.310	12.187	12.635	18.812
5	1.0240	16.014	15.559	19.151	10.578	12.519
5	1.0250	7.991	4.243	6.910	7.204	18.520
5	1.0260	5.895	4.366	6.440	3.872	19.046
5	1.0270	1.682	2.651	3.019	3.945	14.656
5	1.0280	6.071	6.443	5.384	4.426	13.204
5	1.0290	13.192	9.297	12.743	8.673	7.829
5	1.0300	9.774	8.627	12.241	14.774	9.227
5	1.0310	8.563	9.490	12.071	15.708	8.238
5	1.0320	6.018	7.295	6.802	9.769	8.750
5	1.0330	2.380	6.481	7.578	10.463	16.093
5	1.0340	8.259	4.550	7.110	6.624	22.279
5	1.0350	9.136	9.901	11.137	11.369	11.745
5	1.0360	16.525	14.618	17.513	8.941	19.304
5	1.0370	16.492	14.498	17.750	9.017	19.026
5	1.0380	8.303	9.938	9.629	12.622	16.259
5	1.0390	10.803	11.094	10.397	11.756	17.939
5	1.0400	8.720	9.921	9.677	18.178	12.068
5	1.0410	9.535	12.048	9.375	9.652	9.837
5	1.0420	7.114	4.701	5.016	6.010	5.256
5	1.0430	9.656	8.913	10.461	10.044	4.738
5	1.0440	8.122	6.537	8.014	15.296	12.768
5	1.0450	17.039	16.349	17.965	10.407	10.818
5	1.0460	9.388	15.326	13.155	16.332	9.194
5	1.0470	8.385	13.444	13.972	14.831	10.947
5	1.0480	11.333	11.230	8.726	6.022	19.373
5	1.0490	5.697	7.409	4.793	3.313	3.648
5	1.0500	5.636	9.129	5.703	10.975	6.913
5	1.0510	12.028	14.327	15.681	7.669	15.580
5	1.0520	23.449	17.911	19.005	18.764	18.963
5	1.0530	17.367	13.554	13.800	7.049	15.319
5	1.0540	11.665	9.578	14.066	9.110	22.477
5	1.0550	11.645	9.438	13.978	9.527	22.574
5	1.0560	14.191	11.263	15.782	11.172	23.578
5	1.0570	7.355	8.060	6.483	5.461	7.629
5	1.0580	12.644	14.147	18.671	18.830	22.688
5	1.0590	14.100	9.342	13.155	19.185	17.546
5	1.0600	23.731	14.736	20.709	15.773	14.455
5	1.0610	20.251	18.672	22.045	13.111	23.153
5	1.0620	13.051	9.289	12.368	7.836	17.553
5	1.0630	15.817	18.293	15.881	10.777	10.713
5	1.0640	16.174	17.560	14.026	14.016	11.254
5	1.0650	18.145	16.919	15.800	10.352	14.954

5	1.0660	17.349	16.562	18.040	13.492	17.482
5	1.0670	14.001	16.405	18.743	11.113	17.523
5	1.0680	17.854	19.328	23.510	11.160	10.421
5	1.0690	14.118	6.576	6.937	15.733	14.850
5	1.0700	13.062	16.290	14.311	17.271	8.610
5	1.0710	9.884	9.092	8.181	9.554	22.458
5	1.0720	6.117	11.861	9.762	12.816	9.118
5	1.0730	6.267	6.250	8.617	6.747	13.524
5	1.0740	4.902	7.968	7.237	1.804	2.693
5	1.0750	8.466	8.912	9.104	4.430	7.710
5	1.0760	14.617	12.078	14.266	4.710	7.700
5	1.0770	11.127	8.941	9.882	6.512	7.877
5	1.0780	26.146	13.743	24.859	11.882	13.643
5	1.0790	25.959	13.417	24.635	11.877	13.533
5	1.0800	22.947	15.798	19.242	18.932	30.659
5	1.0810	22.193	13.145	21.333	20.669	10.387
5	1.0820	32.645	35.488	27.410	17.131	7.520
5	1.0830	15.466	19.976	14.477	10.557	9.760
5	1.0840	17.577	26.802	16.594	11.281	14.270
5	1.0850	21.084	24.786	17.386	12.504	17.715
5	1.0860	26.271	20.207	22.511	14.008	15.154
5	1.0870	19.668	24.021	17.770	9.603	11.638
5	1.0880	17.326	21.291	15.413	10.287	12.645
5	1.0890	10.944	20.683	12.603	18.040	10.664
5	1.0900	10.072	10.883	10.254	4.290	9.671
5	1.0910	20.334	16.001	18.537	21.268	11.440
5	1.0920	16.138	16.413	12.264	10.089	5.655
5	1.0930	16.887	31.476	20.446	21.332	5.922
5	1.0940	13.113	21.419	12.179	18.620	7.035
5	1.0950	12.669	10.725	10.201	7.852	12.333
5	1.0960	14.678	13.728	13.169	13.750	5.491
5	1.0970	11.189	8.992	9.862	14.173	21.583
5	1.0980	10.252	9.342	8.566	16.736	13.701
5	1.0990	11.757	11.243	11.258	9.800	6.835
5	1.1000	7.991	8.277	4.630	8.892	11.563
5	1.1010	8.612	16.239	9.100	21.046	7.158
5	1.1020	15.668	13.718	10.281	6.110	8.023
5	1.1030	8.127	12.975	7.736	16.246	6.731
5	1.1040	7.242	8.698	6.158	12.359	5.459
5	1.1050	10.889	15.272	12.434	8.126	4.628
5	1.1060	9.156	8.979	8.579	10.132	11.979
5	1.1070	6.094	6.229	6.147	11.533	11.539
5	1.1080	11.259	10.583	10.268	6.779	10.711
5	1.1090	11.303	5.745	9.061	7.949	5.905
5	1.1100	0.000	0.000	0.000	0.000	0.000
6	0.0003	0.000	0.000	0.000	0.000	0.000
6	0.0013	11.226	9.496	12.940	10.276	19.061
6	0.0023	7.374	12.766	7.941	8.559	13.572
6	0.0033	8.011	11.396	10.938	2.722	17.576
6	0.0043	8.204	15.068	14.149	11.464	18.094

6	0.0053	5.338	6.638	7.980	6.517	11.793
6	0.0063	13.261	12.852	16.245	13.909	12.681
6	0.0073	27.679	17.370	13.940	24.265	9.985
6	0.0083	18.562	14.540	13.213	17.714	15.019
6	0.0093	11.813	13.414	8.342	11.557	8.642
6	0.0103	12.275	21.179	16.044	10.768	7.229
6	0.0113	24.633	15.522	19.314	18.040	19.458
6	0.0123	27.853	17.297	22.291	14.229	30.134
6	0.0133	8.385	9.257	11.530	14.644	14.332
6	0.0143	15.807	7.998	9.671	14.422	22.022
6	0.0153	15.697	9.637	11.096	14.897	13.776
6	0.0163	18.356	14.102	12.644	22.257	15.949
6	0.0173	23.003	11.486	9.954	18.969	17.876
6	0.0183	2.541	5.371	2.058	15.247	16.786
6	0.0193	13.701	14.770	11.017	16.071	25.776
6	0.0203	15.219	18.410	10.616	17.867	22.721
6	0.0213	11.801	16.049	10.423	19.333	12.200
6	0.0223	7.912	4.395	5.095	6.061	9.119
6	0.0233	20.728	8.257	8.060	15.858	15.021
6	0.0243	6.329	7.619	4.570	11.043	13.979
6	0.0253	21.009	23.346	16.667	12.588	19.715
6	0.0263	21.982	22.098	17.913	19.111	22.315
6	0.0273	5.896	10.126	6.704	23.152	34.168
6	0.0283	7.362	15.748	7.076	25.234	28.781
6	0.0293	17.673	24.896	15.861	22.184	35.539
6	0.0303	29.275	33.198	21.697	24.480	20.557
6	0.0313	15.227	12.590	13.360	19.767	11.067
6	0.0323	13.445	9.786	12.625	19.169	20.222
6	0.0333	6.740	11.637	10.299	17.392	9.422
6	0.0343	12.291	10.842	10.824	25.882	15.252
6	0.0353	10.279	10.836	11.119	14.063	16.135
6	0.0363	12.202	20.349	11.252	23.972	18.099
6	0.0373	20.963	16.294	16.602	11.801	8.037
6	0.0383	10.946	11.682	14.522	18.138	10.647
6	0.0393	14.474	14.687	13.556	21.181	18.260
6	0.0403	17.486	14.681	12.381	16.358	16.660
6	0.0413	18.235	19.498	13.010	39.508	18.798
6	0.0423	12.235	11.614	7.101	31.445	25.639
6	0.0433	14.061	19.225	11.112	37.425	25.901
6	0.0443	14.252	19.391	12.224	38.206	24.583
6	0.0453	12.113	17.828	19.668	32.364	22.922
6	0.0463	8.350	19.116	15.373	22.594	15.798
6	0.0473	14.115	16.421	20.089	19.646	23.067
6	0.0483	10.066	15.579	14.756	16.904	26.751
6	0.0493	10.784	11.771	13.292	12.299	24.140
6	0.0503	10.849	11.824	13.364	12.308	24.447
6	0.0513	8.733	11.369	12.109	18.235	24.176
6	0.0523	8.125	11.689	13.724	15.610	28.342
6	0.0533	14.462	13.563	19.190	14.159	36.486
6	0.0543	11.838	12.969	16.052	9.305	26.292

6	0.0553	15.965	17.109	14.856	11.420	15.855
6	0.0563	12.945	17.546	14.204	16.507	21.822
6	0.0573	20.505	20.762	23.372	26.283	27.524
6	0.0583	20.676	18.634	16.527	22.727	37.158
6	0.0593	20.919	18.328	18.292	16.416	32.538
6	0.0603	9.664	12.023	8.584	13.290	25.016
6	0.0613	12.323	10.706	6.326	18.078	25.244
6	0.0623	16.017	21.592	13.094	26.157	27.647
6	0.0633	22.450	27.043	20.376	21.445	34.173
6	0.0643	11.808	16.067	14.184	25.756	33.071
6	0.0653	9.395	13.334	10.972	21.068	22.426
6	0.0663	19.026	15.698	25.349	17.702	25.720
6	0.0673	29.561	22.907	24.261	27.509	43.342
6	0.0683	37.345	30.621	32.828	28.493	28.725
6	0.0693	16.725	16.436	11.766	20.785	32.027
6	0.0703	19.307	30.023	24.944	27.539	18.519
6	0.0713	20.109	21.324	20.853	27.730	22.882
6	0.0723	16.222	18.775	21.190	25.181	13.608
6	0.0733	24.672	30.321	22.423	25.931	23.158
6	0.0743	25.455	30.342	22.508	25.892	24.713
6	0.0753	25.356	28.097	19.879	17.836	17.938
6	0.0763	21.951	25.577	19.050	11.853	17.701
6	0.0773	14.756	13.986	13.725	9.970	12.901
6	0.0783	14.259	17.679	11.199	19.212	30.371
6	0.0793	13.961	19.839	16.003	15.057	15.934
6	0.0803	14.362	13.856	12.459	18.228	13.999
6	0.0813	31.779	41.012	28.742	30.504	35.027
6	0.0823	15.346	28.791	18.181	36.089	39.190
6	0.0833	15.446	28.835	18.307	36.256	39.676
6	0.0843	18.758	19.608	17.199	23.387	30.946
6	0.0853	12.033	17.781	18.845	28.897	27.287
6	0.0863	19.325	36.364	24.270	32.594	25.527
6	0.0873	18.139	32.277	20.008	26.330	43.695
6	0.0883	9.121	18.943	14.478	27.731	30.514
6	0.0893	21.336	17.567	19.457	19.427	30.337
6	0.0903	23.860	22.898	23.097	20.033	34.917
6	0.0913	21.069	26.087	23.686	21.037	32.417
6	0.0923	16.533	14.187	17.375	22.686	27.727
6	0.0933	17.651	20.067	17.137	17.701	37.534
6	0.0943	9.033	9.980	9.272	22.148	32.262
6	0.0953	23.426	15.445	14.954	14.840	32.175
6	0.0963	20.697	17.170	19.022	15.783	26.168
6	0.0973	15.162	19.149	15.724	25.159	22.625
6	0.0983	23.517	22.168	20.152	18.539	10.511
6	0.0993	8.706	4.208	9.006	2.004	16.726
6	0.1003	24.767	14.520	16.908	22.405	15.584
6	0.1013	22.976	22.080	22.344	14.793	42.681
6	0.1023	19.219	20.646	19.029	24.180	40.608
6	0.1033	21.827	20.419	18.447	23.660	30.065
6	0.1043	18.271	17.239	18.699	34.057	37.378

6	0.1053	23.032	19.471	14.636	26.871	25.873
6	0.1063	26.955	13.621	16.942	25.084	19.300
6	0.1073	16.074	12.589	13.481	24.802	26.080
6	0.1083	22.725	17.323	21.917	24.346	27.707
6	0.1093	20.723	18.039	17.221	28.647	26.503
6	0.1103	17.023	11.511	16.263	25.312	19.941
6	0.1113	13.183	7.750	11.004	24.688	18.878
6	0.1123	16.304	9.043	7.446	16.616	16.697
6	0.1133	17.460	11.777	13.599	26.074	21.034
6	0.1143	7.317	8.456	7.744	17.596	16.681
6	0.1153	19.549	12.120	11.475	18.507	29.760
6	0.1163	18.734	11.565	13.610	34.647	29.079
6	0.1173	14.404	11.260	11.827	30.180	17.177
6	0.1183	15.820	9.819	13.844	28.673	14.817
6	0.1193	18.574	8.860	19.651	7.687	26.388
6	0.1203	13.417	12.700	11.508	16.048	28.606
6	0.1213	24.998	15.017	16.738	22.211	18.010
6	0.1223	11.503	10.665	11.090	21.966	13.708
6	0.1233	14.949	16.340	17.909	32.013	24.395
6	0.1243	14.203	16.285	16.214	43.589	28.695
6	0.1253	16.638	9.669	20.607	23.058	23.490
6	0.1263	22.849	15.154	23.222	19.268	28.185
6	0.1273	15.143	7.584	7.098	26.313	51.663
6	0.1283	17.131	7.379	16.432	32.923	49.198
6	0.1293	8.837	9.468	9.245	26.828	31.490
6	0.1303	10.015	11.472	9.851	22.550	26.026
6	0.1313	20.971	17.749	20.211	32.182	29.570
6	0.1323	16.211	9.563	12.946	24.228	12.021
6	0.1333	20.107	13.947	21.331	16.415	9.069
6	0.1343	26.259	16.701	21.249	21.477	18.414
6	0.1353	8.866	11.402	12.249	32.614	20.107
6	0.1363	20.160	14.280	18.523	19.042	29.926
6	0.1373	18.761	18.223	11.316	38.926	28.746
6	0.1383	11.611	11.322	8.358	21.000	28.278
6	0.1393	11.280	13.381	14.080	16.595	12.849
6	0.1403	17.247	10.545	18.101	36.859	22.569
6	0.1413	6.956	8.616	9.614	24.956	24.303
6	0.1423	23.686	23.131	21.767	21.444	20.446
6	0.1433	12.558	11.077	8.993	26.514	19.849
6	0.1443	16.299	17.965	17.359	25.493	26.262
6	0.1453	14.428	16.363	18.176	18.840	22.092
6	0.1463	24.869	14.581	12.231	15.591	27.105
6	0.1473	32.889	23.068	19.797	20.604	33.156
6	0.1483	19.443	18.075	19.649	21.131	25.658
6	0.1493	16.994	15.768	18.181	12.171	11.844
6	0.1503	11.470	8.475	12.733	28.253	18.559
6	0.1513	23.259	17.159	19.551	34.399	28.745
6	0.1523	24.082	17.542	16.673	22.492	31.598
6	0.1533	17.721	23.402	17.876	24.746	31.146
6	0.1543	12.778	16.113	9.635	28.270	26.455

6	0.1553	34.434	35.562	29.205	24.317	24.230
6	0.1563	29.941	31.016	27.877	44.462	41.090
6	0.1573	13.137	12.204	15.583	25.927	25.581
6	0.1583	21.170	22.822	21.782	22.983	21.133
6	0.1593	15.194	16.992	13.333	29.084	18.843
6	0.1603	9.980	9.835	7.401	16.694	10.550
6	0.1613	7.959	6.439	6.581	15.224	18.721
6	0.1623	26.437	21.168	21.003	21.821	30.622
6	0.1633	29.656	22.757	25.060	25.615	12.435
6	0.1643	23.763	22.300	19.407	9.221	9.654
6	0.1653	22.367	16.564	19.396	14.229	13.653
6	0.1663	22.773	16.020	19.390	13.313	13.072
6	0.1673	28.677	22.273	23.353	17.700	19.837
6	0.1683	21.943	17.336	14.621	12.065	20.606
6	0.1693	24.387	22.386	15.543	15.665	23.730
6	0.1703	29.166	30.428	39.664	21.597	33.689
6	0.1713	31.268	34.425	33.728	17.002	17.290
6	0.1723	27.140	23.374	21.821	34.425	15.316
6	0.1733	22.520	22.448	23.467	15.327	16.256
6	0.1743	20.103	14.393	15.861	5.410	11.624
6	0.1753	17.137	10.377	11.832	7.553	13.150
6	0.1763	6.778	6.823	10.060	10.200	12.952
6	0.1773	9.247	15.242	14.088	16.455	17.039
6	0.1783	21.148	28.284	18.568	26.649	17.180
6	0.1793	16.532	13.317	13.099	22.152	23.560
6	0.1803	19.505	24.831	12.867	27.740	13.075
6	0.1813	18.592	10.011	14.226	20.805	13.903
6	0.1823	9.365	10.000	8.651	5.464	4.455
6	0.1833	17.983	16.547	13.428	6.723	7.745
6	0.1843	18.631	18.313	19.356	19.118	17.727
6	0.1853	28.941	24.891	21.645	11.911	21.897
6	0.1863	11.272	14.824	9.211	27.440	10.272
6	0.1873	12.100	6.414	7.234	21.296	6.105
6	0.1883	11.724	7.068	7.003	22.579	5.864
6	0.1893	13.602	10.124	9.810	12.722	14.003
6	0.1903	23.629	28.808	16.897	31.190	18.426
6	0.1913	16.782	18.132	14.538	19.729	12.569
6	0.1923	12.714	10.141	12.391	25.864	9.283
6	0.1933	8.000	12.957	9.315	12.806	5.779
6	0.1943	22.228	21.892	16.976	12.953	9.978
6	0.1953	8.105	7.517	4.811	18.217	12.204
6	0.1963	9.328	10.498	6.693	21.439	20.074
6	0.1973	17.821	16.323	16.275	13.237	17.220
6	0.1983	13.692	15.056	11.734	17.090	16.264
6	0.1993	19.132	20.826	21.009	14.585	21.264
6	0.2003	26.415	34.777	25.507	17.659	14.264
6	0.2013	17.689	27.189	21.194	18.837	10.929
6	0.2023	19.139	21.711	14.165	19.407	18.608
6	0.2033	11.777	23.655	15.852	20.407	7.920
6	0.2043	11.684	23.129	15.799	19.861	8.291

6	0.2053	8.172	20.244	11.801	19.883	20.916
6	0.2063	12.840	12.298	14.548	14.742	15.106
6	0.2073	20.895	26.344	24.697	17.666	17.826
6	0.2083	14.796	14.902	14.635	19.072	8.473
6	0.2093	19.186	20.899	21.643	31.685	14.836
6	0.2103	12.416	18.214	14.098	33.538	24.607
6	0.2113	14.886	13.178	18.235	29.381	11.691
6	0.2123	15.567	25.596	18.144	23.247	17.578
6	0.2133	14.348	23.772	18.281	24.540	22.386
6	0.2143	16.798	23.174	22.759	26.722	19.725
6	0.2153	15.872	17.334	14.795	28.496	29.855
6	0.2163	13.473	19.428	18.467	24.815	28.903
6	0.2173	11.692	18.378	15.718	33.227	24.974
6	0.2183	10.401	14.194	14.596	29.034	24.938
6	0.2193	8.309	10.955	13.493	19.298	16.029
6	0.2203	6.220	9.093	12.786	19.499	18.102
6	0.2213	8.000	7.962	7.678	24.816	18.594
6	0.2223	10.031	11.035	13.376	8.901	18.578
6	0.2233	17.381	16.034	17.860	19.402	19.566
6	0.2243	15.460	15.676	13.675	17.815	21.621
6	0.2253	18.473	14.559	20.051	11.721	18.158
6	0.2263	11.397	9.791	15.754	5.144	3.258
6	0.2273	7.145	10.350	10.241	12.926	11.726
6	0.2283	29.626	19.975	29.499	14.541	9.801
6	0.2293	7.127	8.872	13.689	10.617	15.857
6	0.2303	3.226	10.143	5.180	12.446	12.780
6	0.2313	8.940	8.161	5.227	13.927	13.875
6	0.2323	5.643	9.186	4.830	12.036	12.389
6	0.2333	6.505	17.122	8.668	22.997	18.129
6	0.2343	5.249	7.120	3.382	20.971	14.441
6	0.2353	10.498	10.829	12.833	10.930	12.948
6	0.2363	11.077	14.225	17.634	16.193	13.105
6	0.2373	8.490	10.967	11.414	12.596	15.215
6	0.2383	13.675	15.722	20.605	10.974	21.060
6	0.2393	6.412	7.779	10.709	5.113	19.905
6	0.2403	17.143	9.341	9.156	20.335	15.027
6	0.2413	10.631	10.685	10.703	24.513	14.984
6	0.2423	3.456	3.883	4.966	10.721	12.129
6	0.2433	11.430	8.075	10.083	12.819	16.080
6	0.2443	11.808	11.069	12.144	13.684	16.313
6	0.2453	8.817	11.990	14.156	14.819	20.003
6	0.2463	16.374	22.654	24.354	19.697	15.935
6	0.2473	16.303	22.664	24.324	20.090	15.922
6	0.2483	12.734	14.232	14.626	16.073	18.516
6	0.2493	9.185	7.681	7.693	18.759	10.244
6	0.2503	9.556	7.996	5.957	14.877	15.665
6	0.2513	8.775	12.693	3.357	17.445	20.239
6	0.2523	10.757	13.024	7.141	11.070	11.686
6	0.2533	13.529	6.965	5.474	13.744	16.830
6	0.2543	16.136	9.101	8.838	15.529	18.516

6	0.2553	17.968	21.624	11.788	25.736	8.899
6	0.2563	19.380	24.124	13.495	24.757	9.825
6	0.2573	7.903	3.121	5.041	15.027	12.293
6	0.2583	18.614	10.278	8.608	19.404	14.546
6	0.2593	10.026	7.204	7.384	9.479	12.722
6	0.2603	5.732	7.240	7.240	15.028	9.841
6	0.2613	13.062	6.508	12.503	18.941	8.015
6	0.2623	10.099	7.158	6.978	10.138	8.691
6	0.2633	12.546	6.813	12.133	15.223	7.568
6	0.2643	15.548	10.486	12.594	19.994	9.071
6	0.2653	25.545	9.607	16.895	23.348	24.233
6	0.2663	17.227	10.200	12.132	12.262	18.916
6	0.2673	12.249	8.382	9.910	15.949	19.183
6	0.2683	10.468	5.253	5.714	8.077	8.206
6	0.2693	8.625	8.619	8.578	8.965	2.486
6	0.2703	6.784	4.279	7.151	4.316	2.316
6	0.2713	5.014	5.007	7.707	13.429	11.839
6	0.2723	6.188	4.769	9.700	10.091	20.899
6	0.2733	6.174	4.763	9.654	10.088	20.916
6	0.2743	15.867	7.934	7.561	19.176	25.360
6	0.2753	18.247	17.700	11.335	18.167	7.848
6	0.2763	14.937	9.467	12.991	8.989	6.993
6	0.2773	12.589	10.911	9.828	16.711	8.962
6	0.2783	9.721	13.624	6.893	26.177	7.448
6	0.2793	10.182	13.619	7.608	20.783	10.364
6	0.2803	14.431	18.523	9.543	20.699	25.253
6	0.2813	12.192	11.779	11.612	17.193	16.403
6	0.2823	18.802	11.861	12.181	21.023	22.333
6	0.2833	15.067	5.624	9.315	8.684	9.972
6	0.2843	19.353	15.514	15.619	18.250	7.554
6	0.2853	25.562	14.077	13.523	20.240	9.359
6	0.2863	11.392	6.803	7.201	9.371	8.805
6	0.2873	18.440	13.726	14.410	27.135	9.989
6	0.2883	12.562	10.564	8.369	16.971	6.660
6	0.2893	17.661	13.350	12.825	17.457	10.494
6	0.2903	12.667	15.223	10.349	20.555	13.702
6	0.2913	8.306	10.425	7.019	16.401	10.680
6	0.2923	14.913	12.468	9.965	21.865	15.616
6	0.2933	24.155	13.936	16.376	17.271	8.106
6	0.2943	24.115	16.048	16.740	16.332	10.453
6	0.2953	19.080	8.392	17.390	15.788	24.792
6	0.2963	9.712	4.253	9.005	15.102	6.507
6	0.2973	9.385	4.754	9.733	17.803	7.954
6	0.2983	15.785	11.299	13.608	19.694	12.482
6	0.2993	18.687	9.879	14.664	23.029	16.484
6	0.3003	12.686	10.151	10.480	20.640	19.218
6	0.3013	13.331	11.483	9.172	11.573	6.166
6	0.3023	12.557	11.784	7.921	16.191	6.575
6	0.3033	8.459	17.018	7.883	18.081	8.021
6	0.3043	12.420	16.410	9.178	21.664	5.823

6	0.3053	15.517	5.510	8.890	18.126	13.604
6	0.3063	17.836	10.620	13.828	17.988	12.423
6	0.3073	15.598	12.545	10.427	26.098	17.985
6	0.3083	14.578	19.708	12.568	37.095	15.079
6	0.3093	9.877	7.982	7.411	27.036	13.817
6	0.3103	10.119	6.961	5.006	29.226	12.325
6	0.3113	12.241	10.497	9.463	20.297	8.573
6	0.3123	28.399	25.168	21.792	24.053	17.935
6	0.3133	19.522	20.626	12.500	15.423	15.342
6	0.3143	22.556	21.149	15.968	16.729	6.846
6	0.3153	16.984	13.095	11.009	14.259	11.278
6	0.3163	8.370	13.710	7.470	28.655	10.774
6	0.3173	20.776	15.565	17.274	19.727	9.682
6	0.3183	22.062	21.467	19.254	16.104	11.715
6	0.3193	7.291	11.277	6.988	18.276	5.495
6	0.3203	12.523	12.989	11.189	14.411	4.982
6	0.3213	17.990	14.652	14.839	10.379	6.945
6	0.3223	10.103	10.947	16.452	4.644	9.043
6	0.3233	14.889	11.421	11.770	13.823	13.431
6	0.3243	21.988	18.773	13.062	18.098	20.404
6	0.3253	13.998	9.294	6.550	18.847	32.373
6	0.3263	4.111	2.400	1.510	11.767	8.142
6	0.3273	17.140	17.928	17.833	12.423	7.830
6	0.3283	20.408	25.887	17.815	21.532	11.790
6	0.3293	13.359	13.723	9.295	24.956	9.155
6	0.3303	31.122	20.355	22.046	25.974	7.169
6	0.3313	21.754	15.421	17.168	9.961	5.073
6	0.3323	25.859	16.888	17.890	26.868	16.023
6	0.3333	20.284	19.520	13.451	6.650	20.326
6	0.3343	19.822	10.604	13.787	9.873	11.458
6	0.3353	24.286	14.422	9.801	27.565	30.094
6	0.3363	18.173	12.939	10.169	23.704	20.062
6	0.3373	22.866	16.767	16.270	14.549	13.317
6	0.3383	22.495	15.832	16.137	13.576	13.600
6	0.3393	18.014	13.329	15.143	12.267	10.208
6	0.3403	18.415	14.278	15.072	13.259	10.001
6	0.3413	19.692	16.151	16.965	11.627	10.394
6	0.3423	21.944	14.004	16.362	22.096	10.102
6	0.3433	10.630	5.911	9.174	16.841	17.041
6	0.3443	8.282	6.965	5.283	6.030	6.676
6	0.3453	10.355	6.078	6.134	14.157	12.353
6	0.3463	13.103	11.506	11.198	12.263	8.726
6	0.3473	19.123	20.213	18.426	25.770	12.057
6	0.3483	18.752	13.965	16.487	23.944	30.178
6	0.3493	10.663	9.216	6.957	16.088	12.330
6	0.3503	12.525	11.281	7.661	16.658	12.473
6	0.3513	7.824	5.623	4.743	7.625	7.671
6	0.3523	19.638	19.545	15.994	15.328	6.512
6	0.3533	13.090	14.339	11.422	18.639	11.230
6	0.3543	12.236	16.343	11.797	26.254	15.626

6	0.3553	9.272	5.962	13.549	16.894	11.454
6	0.3563	10.417	9.150	9.485	10.507	15.807
6	0.3573	11.456	6.496	7.449	15.039	12.472
6	0.3583	18.553	20.608	13.925	28.399	15.918
6	0.3593	13.042	20.711	15.886	17.138	13.530
6	0.3603	14.796	12.064	11.124	13.835	14.429
6	0.3613	18.956	14.710	13.093	17.001	23.980
6	0.3623	15.779	11.040	11.102	8.154	12.765
6	0.3633	12.958	13.877	11.183	14.081	8.088
6	0.3643	12.107	6.950	11.248	10.593	20.238
6	0.3653	24.354	21.831	20.010	12.837	9.864
6	0.3663	17.176	16.432	17.740	8.244	4.636
6	0.3673	14.410	23.143	17.268	26.418	6.565
6	0.3683	24.085	15.939	21.144	14.331	8.487
6	0.3693	15.225	17.511	15.638	24.653	15.359
6	0.3703	11.404	25.891	14.073	25.930	13.514
6	0.3713	8.676	15.721	10.868	14.467	7.759
6	0.3723	13.843	12.840	9.318	6.264	12.948
6	0.3733	8.745	9.234	5.591	14.484	13.250
6	0.3743	12.300	10.839	7.704	12.288	14.738
6	0.3753	11.571	12.610	9.806	12.124	9.521
6	0.3763	11.603	21.032	16.141	16.266	8.716
6	0.3773	8.427	11.125	9.427	13.035	7.508
6	0.3783	10.800	13.130	10.788	6.652	9.131
6	0.3793	9.865	14.333	13.662	12.384	11.951
6	0.3803	6.083	16.514	13.351	18.967	11.321
6	0.3813	11.236	21.041	18.523	8.954	16.678
6	0.3823	9.991	16.956	11.880	16.625	11.881
6	0.3833	17.463	16.947	19.717	12.132	23.603
6	0.3843	19.213	21.999	14.774	19.970	16.781
6	0.3853	8.216	16.229	9.385	23.850	9.249
6	0.3863	11.890	21.841	12.687	25.933	13.449
6	0.3873	6.155	6.778	8.441	18.846	7.401
6	0.3883	5.053	7.352	9.278	12.578	6.936
6	0.3893	8.600	14.145	6.138	16.589	11.329
6	0.3903	8.465	15.646	7.238	18.525	10.517
6	0.3913	10.323	17.624	9.212	20.632	10.500
6	0.3923	9.053	10.283	10.122	28.221	12.401
6	0.3933	10.381	13.995	13.553	18.774	8.654
6	0.3943	10.891	9.578	7.274	8.172	5.348
6	0.3953	15.630	14.281	12.009	13.271	5.626
6	0.3963	14.310	13.899	10.862	11.240	8.022
6	0.3973	14.817	17.122	10.381	15.492	6.631
6	0.3983	11.014	11.802	10.291	6.588	6.584
6	0.3993	10.139	7.654	4.869	9.747	8.275
6	0.4003	11.527	7.971	6.950	8.647	8.944
6	0.4013	13.239	6.789	6.306	10.315	8.661
6	0.4023	10.854	6.680	6.019	16.274	19.821
6	0.4033	12.730	6.188	5.936	7.473	9.586
6	0.4043	12.794	6.483	6.120	6.580	9.461

6	0.4053	12.861	6.304	7.058	7.936	5.412
6	0.4063	13.563	7.126	5.565	13.776	8.430
6	0.4073	20.819	17.873	11.997	17.414	13.006
6	0.4083	7.248	13.689	6.220	19.150	7.429
6	0.4093	9.227	9.712	8.905	12.965	8.557
6	0.4103	20.162	18.573	16.938	18.570	16.622
6	0.4113	20.120	18.623	16.950	19.603	16.931
6	0.4123	20.066	18.698	16.961	20.484	17.175
6	0.4133	24.908	19.922	23.880	8.297	13.616
6	0.4143	24.979	20.025	24.029	8.063	13.686
6	0.4153	11.775	4.751	8.680	7.412	7.474
6	0.4163	7.830	4.401	9.146	13.294	5.724
6	0.4173	13.630	7.374	14.272	5.163	4.594
6	0.4183	13.807	7.828	14.475	5.193	4.565
6	0.4193	17.763	10.236	15.262	14.537	16.155
6	0.4203	15.336	7.160	9.624	14.775	10.196
6	0.4213	11.357	9.383	7.437	9.898	8.798
6	0.4223	11.374	9.006	7.412	9.351	8.938
6	0.4233	11.431	8.694	7.409	8.940	9.120
6	0.4243	14.440	7.549	13.089	8.321	9.534
6	0.4253	17.378	20.584	22.978	11.862	20.219
6	0.4263	17.386	20.575	22.978	11.879	20.175
6	0.4273	17.821	17.354	19.225	13.187	12.501
6	0.4283	14.927	12.033	17.604	9.149	8.001
6	0.4293	17.618	11.961	18.345	5.831	10.601
6	0.4303	17.394	8.314	13.331	12.963	15.386
6	0.4313	5.593	5.141	5.991	15.892	11.632
6	0.4323	14.003	5.393	5.372	8.004	11.272
6	0.4333	14.553	14.976	15.279	9.409	7.181
6	0.4343	15.178	17.332	11.738	11.923	4.885
6	0.4353	10.205	11.935	12.755	14.895	17.075
6	0.4363	8.776	13.668	8.701	20.600	13.902
6	0.4373	10.861	9.958	13.357	14.091	7.855
6	0.4383	13.039	17.682	15.827	12.067	5.588
6	0.4393	6.092	6.148	6.089	9.858	21.845
6	0.4403	15.282	15.933	19.774	12.505	15.862
6	0.4413	14.074	18.433	20.697	10.052	16.943
6	0.4423	6.863	5.845	7.156	5.081	2.503
6	0.4433	7.977	8.590	9.260	8.972	7.322
6	0.4443	9.574	14.203	12.080	9.947	9.647
6	0.4453	10.114	13.880	7.455	15.743	10.455
6	0.4463	8.741	15.689	13.371	14.920	6.478
6	0.4473	13.502	15.143	14.390	12.963	13.329
6	0.4483	17.610	8.157	12.870	13.629	16.830
6	0.4493	12.031	10.112	8.186	20.284	15.166
6	0.4503	8.305	9.643	5.002	17.952	12.820
6	0.4513	3.232	8.671	5.590	12.896	8.973
6	0.4523	13.679	18.797	13.192	11.029	14.071
6	0.4533	8.391	11.909	8.811	14.000	9.986
6	0.4543	7.057	9.591	6.456	15.969	18.692

6	0.4553	13.010	12.314	10.238	11.346	15.887
6	0.4563	7.297	13.294	10.465	12.405	17.752
6	0.4573	13.232	16.859	17.413	8.873	18.966
6	0.4583	18.119	24.467	19.672	23.618	10.285
6	0.4593	8.145	9.493	11.114	15.109	10.455
6	0.4603	12.621	23.954	16.163	24.143	12.131
6	0.4613	5.824	13.730	8.475	15.464	17.443
6	0.4623	9.672	8.174	9.812	14.647	15.453
6	0.4633	7.565	11.230	6.798	12.745	11.770
6	0.4643	11.272	14.634	10.257	11.499	16.349
6	0.4653	17.320	16.807	19.743	5.770	13.985
6	0.4663	12.985	17.218	19.040	14.493	36.422
6	0.4673	13.675	24.134	13.385	19.256	18.035
6	0.4683	9.002	14.397	13.317	17.182	14.688
6	0.4693	12.557	15.910	13.633	22.510	21.128
6	0.4703	11.356	9.127	10.018	13.023	3.924
6	0.4713	14.379	13.821	14.884	12.522	6.102
6	0.4723	16.234	15.512	15.929	11.383	6.990
6	0.4733	12.901	14.926	17.053	9.403	7.330
6	0.4743	12.280	9.013	9.691	8.164	7.103
6	0.4753	9.048	11.434	8.556	11.261	9.318
6	0.4763	18.763	16.903	10.044	26.805	14.519
6	0.4773	19.306	30.297	23.144	19.396	14.331
6	0.4783	19.946	32.695	23.635	21.660	14.374
6	0.4793	18.854	16.856	18.564	20.337	17.921
6	0.4803	14.987	12.868	14.832	8.214	11.559
6	0.4813	12.564	13.800	12.001	7.674	13.199
6	0.4823	17.400	16.649	15.329	14.994	3.679
6	0.4833	20.395	17.826	17.528	12.769	11.517
6	0.4843	15.108	17.540	13.247	23.579	15.430
6	0.4853	25.191	21.738	19.116	20.003	15.751
6	0.4863	17.693	17.904	14.540	16.958	15.600
6	0.4873	15.355	16.105	11.247	15.720	13.320
6	0.4883	12.384	18.255	12.978	15.629	13.358
6	0.4893	15.998	12.579	13.290	12.945	25.509
6	0.4903	19.686	15.677	12.275	29.601	24.032
6	0.4913	18.143	18.984	15.319	22.657	15.861
6	0.4923	10.099	7.819	4.939	15.794	13.612
6	0.4933	15.523	5.771	11.221	9.197	12.191
6	0.4943	12.347	8.133	10.125	10.841	16.081
6	0.4953	13.514	18.295	11.497	20.990	15.103
6	0.4963	8.017	10.543	9.152	10.541	3.250
6	0.4973	9.642	13.368	11.586	14.351	7.505
6	0.4983	12.404	18.031	13.872	15.572	10.313
6	0.4993	9.483	18.097	12.475	24.237	15.712
6	0.5003	13.250	17.980	10.509	31.338	16.621
6	0.5013	14.642	26.232	16.766	28.554	27.929
6	0.5023	8.692	11.624	12.767	20.013	21.223
6	0.5033	10.373	9.053	14.239	9.817	15.490
6	0.5043	16.285	18.980	17.421	12.573	17.287

6	0.5053	15.211	14.563	12.273	8.332	13.957
6	0.5063	10.360	18.311	13.139	22.916	17.391
6	0.5073	12.714	11.288	16.830	10.093	15.864
6	0.5083	9.287	11.598	13.574	5.293	8.784
6	0.5093	10.362	9.507	8.079	19.029	9.796
6	0.5103	9.302	6.663	8.781	8.703	10.679
6	0.5113	17.199	15.537	14.448	29.990	12.829
6	0.5123	10.773	13.067	13.126	14.430	7.599
6	0.5133	10.113	14.110	14.356	9.534	13.167
6	0.5143	8.782	12.090	12.004	10.084	15.647
6	0.5153	9.941	9.707	10.951	6.715	10.306
6	0.5163	5.239	6.212	7.027	13.321	3.559
6	0.5173	11.816	7.756	11.017	9.675	12.419
6	0.5183	18.390	18.434	18.527	15.526	13.590
6	0.5193	12.734	12.830	10.025	5.802	12.255
6	0.5203	18.687	19.721	17.110	9.525	11.578
6	0.5213	15.421	18.823	21.001	17.657	22.223
6	0.5223	9.922	15.093	12.354	13.039	8.015
6	0.5233	22.019	27.918	26.886	25.926	18.254
6	0.5243	14.526	18.852	18.479	16.671	14.307
6	0.5253	11.928	13.517	12.773	8.687	15.545
6	0.5263	14.622	20.935	15.502	7.669	17.009
6	0.5273	13.208	15.938	10.348	14.272	15.171
6	0.5283	11.597	14.946	9.616	12.626	9.137
6	0.5293	15.452	22.499	13.827	14.311	10.274
6	0.5303	13.850	20.722	16.051	28.110	12.942
6	0.5313	20.484	26.239	22.034	15.468	19.236
6	0.5323	5.819	6.707	8.956	14.761	15.053
6	0.5333	26.053	21.738	29.139	12.976	10.310
6	0.5343	12.320	12.730	14.603	11.197	16.152
6	0.5353	10.940	12.575	13.985	8.590	8.708
6	0.5363	7.420	9.708	9.226	20.855	10.802
6	0.5373	8.464	12.984	11.680	11.001	9.605
6	0.5383	10.304	15.451	10.586	15.896	12.314
6	0.5393	14.042	18.141	18.821	18.260	16.015
6	0.5403	12.966	18.564	19.544	10.476	13.297
6	0.5413	13.577	16.670	8.912	14.832	21.656
6	0.5423	15.835	14.448	9.966	10.464	19.632
6	0.5433	18.452	21.488	21.124	23.308	18.889
6	0.5443	15.610	22.965	22.552	24.401	10.563
6	0.5453	7.131	18.695	13.365	17.958	11.313
6	0.5463	12.579	15.685	16.685	21.357	15.430
6	0.5473	8.621	13.061	13.643	10.496	13.298
6	0.5483	9.132	8.850	6.513	10.980	8.831
6	0.5493	14.203	13.188	14.488	5.724	8.864
6	0.5503	10.390	14.088	12.904	7.986	7.662
6	0.5513	9.792	13.067	12.464	9.850	14.777
6	0.5523	15.381	24.985	16.600	13.376	15.071
6	0.5533	11.254	18.545	16.897	15.938	15.872
6	0.5543	7.222	7.928	6.972	12.001	12.984

6	0.5553	16.085	18.677	14.153	21.977	17.109
6	0.5563	14.013	23.230	17.889	16.778	16.923
6	0.5573	15.878	14.351	13.899	21.733	22.034
6	0.5583	14.137	11.469	8.748	8.981	16.173
6	0.5593	13.178	16.578	14.880	12.284	8.546
6	0.5603	19.625	17.615	16.618	18.847	17.675
6	0.5613	18.629	18.056	12.167	15.264	24.416
6	0.5623	21.460	14.014	16.345	14.880	22.460
6	0.5633	9.330	13.499	10.494	13.606	16.498
6	0.5643	20.589	28.786	20.305	11.109	14.876
6	0.5653	29.288	28.532	25.262	23.231	28.611
6	0.5663	27.787	20.302	20.832	29.385	21.143
6	0.5673	17.085	12.679	17.227	15.072	18.498
6	0.5683	22.563	14.842	12.574	20.219	19.800
6	0.5693	17.446	14.608	13.271	18.151	19.897
6	0.5703	20.687	14.066	18.719	17.915	10.127
6	0.5713	8.614	5.631	5.132	21.403	12.569
6	0.5723	9.223	11.648	7.383	25.010	23.334
6	0.5733	10.440	13.078	7.812	20.339	19.964
6	0.5743	20.471	21.373	14.591	17.931	27.572
6	0.5753	16.584	15.566	15.181	16.707	24.768
6	0.5763	26.032	29.941	19.770	23.074	10.949
6	0.5773	21.068	25.554	17.487	21.776	14.251
6	0.5783	26.313	26.319	22.699	18.997	21.503
6	0.5793	24.242	26.605	24.331	27.996	12.948
6	0.5803	24.392	17.387	18.535	43.477	15.969
6	0.5813	14.458	11.274	10.737	21.849	21.224
6	0.5823	29.332	31.140	27.370	25.113	26.307
6	0.5833	26.626	31.254	26.937	18.984	23.984
6	0.5843	21.934	24.560	21.133	22.396	12.272
6	0.5853	16.514	14.641	19.605	17.908	10.015
6	0.5863	14.741	17.942	13.328	23.442	15.021
6	0.5873	17.227	21.613	16.717	24.493	15.428
6	0.5883	21.484	21.282	14.308	14.698	14.209
6	0.5893	35.014	34.902	28.883	28.503	26.446
6	0.5903	18.127	17.818	15.844	15.078	17.428
6	0.5913	22.632	17.302	11.177	7.719	22.065
6	0.5923	16.125	9.003	8.784	14.909	25.441
6	0.5933	14.375	8.766	9.582	18.118	23.593
6	0.5943	22.236	10.081	15.116	13.917	16.409
6	0.5953	15.219	15.350	11.933	11.296	11.194
6	0.5963	16.280	17.937	15.856	18.781	22.347
6	0.5973	25.234	20.065	16.226	14.815	21.718
6	0.5983	27.211	30.800	25.838	21.989	11.840
6	0.5993	12.606	11.622	11.855	15.648	17.152
6	0.6003	20.703	23.919	28.672	16.761	19.882
6	0.6013	32.443	27.838	35.098	20.951	14.244
6	0.6023	16.913	11.376	16.218	16.772	22.637
6	0.6033	10.481	10.674	12.253	25.893	33.548
6	0.6043	31.000	31.456	33.092	24.548	33.945

6	0.6053	13.902	12.776	11.153	16.484	28.238
6	0.6063	33.950	32.060	31.838	18.003	22.960
6	0.6073	33.662	33.561	30.186	31.612	25.647
6	0.6083	26.969	18.163	22.225	13.563	14.776
6	0.6093	28.725	22.824	19.862	16.032	9.894
6	0.6103	21.026	19.690	18.472	16.738	17.103
6	0.6113	24.231	20.169	19.484	12.347	18.223
6	0.6123	23.648	24.134	23.479	23.719	30.352
6	0.6133	29.517	28.810	28.661	20.548	30.918
6	0.6143	24.289	27.097	26.493	17.172	9.718
6	0.6153	22.021	12.602	14.243	20.776	22.451
6	0.6163	25.293	26.392	27.610	13.155	24.533
6	0.6173	27.357	24.260	31.712	16.411	13.312
6	0.6183	28.004	28.141	38.118	16.904	24.408
6	0.6193	13.866	17.109	17.710	13.727	7.680
6	0.6203	11.935	21.018	17.970	18.543	21.669
6	0.6213	25.682	20.177	24.120	21.744	28.344
6	0.6223	20.611	18.856	18.603	12.156	20.793
6	0.6233	20.065	12.893	19.332	11.793	27.646
6	0.6243	30.812	22.619	31.785	18.584	22.385
6	0.6253	26.873	26.688	31.070	21.927	16.207
6	0.6263	26.084	22.501	29.722	20.493	13.870
6	0.6273	18.821	12.445	15.820	20.410	24.690
6	0.6283	12.239	12.617	14.839	25.759	36.507
6	0.6293	12.398	13.192	14.720	28.057	37.225
6	0.6303	25.443	15.664	25.855	26.453	13.876
6	0.6313	28.357	19.480	25.049	25.523	21.274
6	0.6323	26.227	14.210	24.346	18.081	27.050
6	0.6333	30.387	18.911	23.373	15.720	17.650
6	0.6343	16.879	14.613	18.424	14.159	37.656
6	0.6353	21.142	10.280	18.812	7.757	30.384
6	0.6363	28.106	13.950	24.077	20.495	28.631
6	0.6373	19.104	13.560	14.538	22.727	25.355
6	0.6383	8.564	11.270	5.965	31.305	22.158
6	0.6393	27.036	26.046	24.991	27.249	35.600
6	0.6403	29.431	32.846	40.632	18.683	39.571
6	0.6413	18.683	25.479	29.112	19.748	12.826
6	0.6423	15.350	8.467	11.081	10.799	21.230
6	0.6433	15.505	8.308	11.002	10.767	21.460
6	0.6443	13.135	18.031	21.815	22.601	37.281
6	0.6453	13.614	15.716	25.905	20.649	39.889
6	0.6463	13.029	15.875	25.195	23.010	40.491
6	0.6473	10.953	14.773	20.092	10.620	26.553
6	0.6483	20.822	20.467	28.142	29.682	32.244
6	0.6493	24.702	22.400	27.309	31.665	33.835
6	0.6503	32.101	23.844	27.589	24.217	52.703
6	0.6513	14.483	10.842	7.565	16.374	41.909
6	0.6523	26.351	37.462	32.268	18.876	20.307
6	0.6533	14.919	25.472	18.444	25.030	30.245
6	0.6543	18.189	18.050	23.878	31.232	44.055

6	0.6553	14.479	18.618	24.998	27.364	34.619
6	0.6563	35.399	27.964	34.935	19.251	54.159
6	0.6573	21.412	21.429	25.251	26.798	40.274
6	0.6583	14.385	9.804	20.218	19.751	23.225
6	0.6593	16.437	6.412	17.469	22.744	14.966
6	0.6603	13.517	12.411	14.616	23.384	19.868
6	0.6613	20.030	11.086	18.690	14.634	35.374
6	0.6623	17.171	10.301	14.407	22.334	46.375
6	0.6633	14.090	12.831	14.712	9.818	34.247
6	0.6643	14.616	15.447	16.562	9.005	40.601
6	0.6653	9.916	6.906	11.260	9.796	26.621
6	0.6663	9.353	12.061	8.852	21.270	31.622
6	0.6673	16.143	11.792	13.107	11.638	15.752
6	0.6683	13.364	11.205	14.901	20.923	25.826
6	0.6693	10.473	14.694	14.547	25.382	43.035
6	0.6703	10.530	16.615	14.520	26.416	33.531
6	0.6713	16.706	26.109	30.115	17.706	19.715
6	0.6723	6.968	10.959	12.775	17.648	36.658
6	0.6733	16.224	13.823	23.431	12.756	36.194
6	0.6743	9.082	11.762	11.993	11.024	34.150
6	0.6753	8.077	9.200	10.909	12.905	45.100
6	0.6763	21.228	13.779	22.860	19.615	13.805
6	0.6773	21.004	13.863	22.964	19.352	13.791
6	0.6783	4.860	9.252	8.804	14.924	8.933
6	0.6793	10.310	12.695	13.627	12.862	14.584
6	0.6803	14.888	14.139	19.560	5.717	29.384
6	0.6813	9.818	13.541	12.452	12.888	20.833
6	0.6823	12.394	8.106	7.724	16.645	30.465
6	0.6833	7.636	8.730	11.777	14.019	30.383
6	0.6843	12.314	13.632	18.656	22.787	34.725
6	0.6853	10.021	13.711	17.328	18.176	33.281
6	0.6863	5.101	6.326	6.665	10.117	36.434
6	0.6873	16.675	19.424	26.195	17.799	29.268
6	0.6883	16.690	22.516	25.484	11.503	35.584
6	0.6893	19.361	13.547	12.407	11.882	28.775
6	0.6903	14.523	9.489	8.787	15.029	24.356
6	0.6913	23.890	27.004	26.559	14.137	26.086
6	0.6923	25.039	23.682	29.046	16.926	22.020
6	0.6933	27.583	29.849	30.224	18.232	27.039
6	0.6943	18.601	28.397	26.368	21.477	25.866
6	0.6953	11.222	9.917	10.363	12.346	12.845
6	0.6963	11.805	8.360	10.428	19.217	37.390
6	0.6973	14.582	8.029	11.789	24.995	42.754
6	0.6983	17.573	16.655	12.924	27.315	52.283
6	0.6993	22.036	15.083	15.350	12.666	9.171
6	0.7003	29.351	15.675	20.957	7.615	17.425
6	0.7013	13.410	9.729	11.154	16.763	23.763
6	0.7023	12.017	13.387	12.967	20.740	49.869
6	0.7033	13.852	19.568	18.160	20.693	38.478
6	0.7043	15.827	9.441	11.516	24.007	25.495

6	0.7053	10.842	8.826	12.427	10.327	13.554
6	0.7063	24.358	17.127	20.966	19.899	55.256
6	0.7073	21.394	12.342	15.880	23.125	39.358
6	0.7083	24.731	24.919	23.755	38.158	70.673
6	0.7093	25.728	16.178	27.686	25.240	58.741
6	0.7103	15.572	15.348	9.647	29.830	48.967
6	0.7113	11.113	10.502	13.438	17.455	46.917
6	0.7123	19.961	16.917	16.890	30.811	53.370
6	0.7133	16.921	21.118	17.567	26.949	50.320
6	0.7143	28.552	22.803	21.281	19.921	65.078
6	0.7153	34.205	19.467	25.754	22.716	50.773
6	0.7163	22.604	16.207	20.831	15.070	14.827
6	0.7173	17.251	13.295	15.034	26.004	40.250
6	0.7183	19.466	11.497	12.093	30.067	51.030
6	0.7193	20.602	11.155	11.798	30.659	51.844
6	0.7203	18.923	13.027	10.778	33.808	49.910
6	0.7213	18.973	13.318	10.955	33.936	49.447
6	0.7223	13.892	8.547	6.262	31.273	62.372
6	0.7233	15.942	21.534	15.154	44.898	82.866
6	0.7243	18.142	25.530	18.659	48.658	83.536
6	0.7253	9.018	11.460	7.630	22.658	20.734
6	0.7263	6.244	8.173	8.274	15.620	19.103
6	0.7273	13.294	13.453	10.625	24.845	41.855
6	0.7283	10.304	9.169	8.006	17.189	36.027
6	0.7293	8.605	20.528	17.963	26.710	46.598
6	0.7303	20.522	21.855	25.379	26.340	53.861
6	0.7313	21.138	16.780	18.130	30.042	62.679
6	0.7323	39.524	25.481	25.516	26.979	71.554
6	0.7333	28.696	21.735	25.554	26.564	69.199
6	0.7343	25.646	17.829	19.401	18.165	38.254
6	0.7353	20.165	21.808	26.301	14.208	38.537
6	0.7363	16.577	17.673	24.243	17.211	49.930
6	0.7373	11.775	12.223	15.139	19.715	58.515
6	0.7383	14.210	17.221	18.328	43.021	106.204
6	0.7393	16.103	18.075	13.829	49.273	79.968
6	0.7403	18.980	15.523	20.776	42.855	78.273
6	0.7413	16.066	19.929	18.648	21.543	47.196
6	0.7423	7.873	16.999	18.070	15.721	33.405
6	0.7433	14.964	12.094	14.378	31.468	37.522
6	0.7443	22.797	17.721	24.458	30.400	60.962
6	0.7453	17.141	11.785	12.150	32.591	51.596
6	0.7463	10.231	8.448	6.799	41.267	46.039
6	0.7473	24.016	11.213	16.464	23.577	36.661
6	0.7483	18.682	10.131	13.547	27.606	28.220
6	0.7493	19.233	10.018	4.588	46.705	62.265
6	0.7503	16.407	10.519	13.532	53.785	100.590
6	0.7513	18.296	9.523	11.111	46.452	79.314
6	0.7523	22.802	7.847	10.538	42.954	74.372
6	0.7533	30.813	15.544	20.943	35.358	73.673
6	0.7543	26.795	16.838	22.496	39.535	83.828

6	0.7553	27.696	18.957	22.120	42.606	80.014
6	0.7563	24.294	23.840	22.230	49.094	85.770
6	0.7573	15.429	14.241	10.152	41.536	95.814
6	0.7583	21.949	14.124	10.223	44.900	94.703
6	0.7593	11.169	10.330	9.106	33.206	58.055
6	0.7603	15.640	14.130	12.631	56.756	96.491
6	0.7613	21.514	19.946	10.594	44.009	78.193
6	0.7623	14.849	15.127	8.744	36.561	86.386
6	0.7633	10.419	13.184	8.283	43.213	80.488
6	0.7643	11.936	13.409	10.217	42.548	79.543
6	0.7653	15.872	9.177	12.671	46.569	79.552
6	0.7663	21.913	8.191	11.471	55.022	107.800
6	0.7673	18.528	9.861	12.983	52.885	104.747
6	0.7683	25.096	23.231	22.573	59.010	106.674
6	0.7693	26.218	30.377	26.595	59.256	104.635
6	0.7703	15.136	16.937	18.862	66.843	128.872
6	0.7713	20.473	16.745	17.714	45.219	104.355
6	0.7723	20.239	28.615	24.890	33.947	49.328
6	0.7733	20.111	28.560	23.908	34.381	48.566
6	0.7743	15.381	15.516	17.686	25.782	40.329
6	0.7753	15.192	11.689	13.371	25.092	68.995
6	0.7763	15.480	12.780	9.636	29.575	101.617
6	0.7773	17.234	7.701	5.411	39.447	95.141
6	0.7783	14.580	8.707	6.071	37.715	73.203
6	0.7793	11.885	7.058	7.804	29.274	71.643
6	0.7803	12.947	7.622	12.079	38.552	70.136
6	0.7813	27.960	16.938	15.787	66.118	97.577
6	0.7823	12.562	10.822	8.380	59.763	90.972
6	0.7833	22.309	22.736	17.214	60.987	106.154
6	0.7843	13.816	17.164	12.551	46.860	82.993
6	0.7853	16.468	7.390	7.362	37.136	92.532
6	0.7863	26.012	25.643	20.652	47.523	80.634
6	0.7873	21.998	17.147	10.919	50.951	73.539
6	0.7883	16.002	17.945	10.296	53.859	69.124
6	0.7893	12.386	10.944	7.614	24.264	57.444
6	0.7903	15.477	8.491	4.097	41.371	84.479
6	0.7913	10.745	4.520	3.097	51.242	99.305
6	0.7923	13.873	6.337	12.237	37.014	63.294
6	0.7933	12.001	13.417	15.713	41.019	45.111
6	0.7943	23.457	17.617	25.676	36.479	67.901
6	0.7953	12.381	16.769	13.021	34.804	78.602
6	0.7963	16.300	10.171	8.820	62.493	121.153
6	0.7973	13.064	9.469	8.076	47.888	80.877
6	0.7983	16.795	21.781	13.711	31.328	54.743
6	0.7993	13.012	13.445	8.590	21.639	69.407
6	0.8003	6.150	13.804	11.274	24.176	51.864
6	0.8013	2.387	3.989	3.890	10.206	19.563
6	0.8023	14.891	7.651	11.705	5.919	32.815
6	0.8033	31.911	20.773	28.526	36.552	92.361
6	0.8043	20.639	12.534	19.817	41.546	88.402

6	0.8053	18.389	7.709	13.772	48.319	118.122
6	0.8063	8.729	11.100	12.355	14.546	32.653
6	0.8073	22.236	21.640	26.851	32.177	54.041
6	0.8083	9.609	21.244	21.552	64.638	66.159
6	0.8093	5.809	6.246	5.256	21.369	45.071
6	0.8103	10.397	9.405	11.239	16.195	41.523
6	0.8113	15.739	13.928	19.776	45.534	122.294
6	0.8123	5.786	12.310	13.015	27.815	85.654
6	0.8133	12.166	13.883	15.486	46.081	155.554
6	0.8143	14.748	14.974	10.227	57.909	121.865
6	0.8153	18.372	20.949	13.598	45.026	119.233
6	0.8163	28.431	14.278	22.868	34.919	114.617
6	0.8173	27.142	6.039	10.288	37.979	116.383
6	0.8183	20.427	11.151	14.235	29.057	79.863
6	0.8193	15.592	12.704	10.389	32.410	78.328
6	0.8203	11.391	15.019	13.391	58.587	138.793
6	0.8213	8.815	13.160	16.961	24.080	54.918
6	0.8223	12.914	10.925	13.665	36.880	103.048
6	0.8233	17.683	11.831	12.917	27.801	103.296
6	0.8243	29.438	16.429	20.568	35.044	152.850
6	0.8253	29.635	16.527	20.630	34.973	153.107
6	0.8263	17.265	15.558	16.495	62.899	181.669
6	0.8273	6.996	9.499	12.541	75.311	224.735
6	0.8283	4.613	3.004	3.901	20.652	66.276
6	0.8293	18.636	12.683	20.365	25.785	86.417
6	0.8303	16.579	15.935	17.335	27.194	60.277
6	0.8313	14.426	7.504	10.787	26.798	72.541
6	0.8323	19.150	8.410	14.731	31.784	88.330
6	0.8333	14.948	19.475	10.917	37.262	90.507
6	0.8343	17.113	18.594	11.441	41.539	78.626
6	0.8353	17.878	10.567	5.824	37.016	91.754
6	0.8363	10.251	22.563	17.806	24.919	60.237
6	0.8373	8.833	17.722	22.304	36.114	45.271
6	0.8383	11.717	14.717	12.268	24.470	55.976
6	0.8393	11.728	14.718	11.785	24.295	56.306
6	0.8403	23.072	13.056	11.595	24.550	66.367
6	0.8413	17.609	17.184	13.804	23.875	32.013
6	0.8423	15.205	19.276	27.731	39.084	93.139
6	0.8433	27.153	29.595	28.421	23.045	57.377
6	0.8443	10.896	17.960	11.741	50.911	93.675
6	0.8453	3.977	13.793	9.832	37.909	65.705
6	0.8463	6.595	11.237	9.469	37.903	82.710
6	0.8473	17.019	25.017	21.372	29.906	39.759
6	0.8483	18.665	20.904	16.694	45.120	93.465
6	0.8493	20.842	19.371	17.096	24.731	61.036
6	0.8503	21.809	12.246	11.791	38.858	66.115
6	0.8513	20.875	3.881	15.321	39.980	102.953
6	0.8523	17.513	5.943	8.090	34.237	96.324
6	0.8533	19.382	5.110	12.838	42.309	90.001
6	0.8543	18.685	12.966	10.332	13.606	20.628

6	0.8553	10.060	14.319	12.406	10.716	15.351
6	0.8563	5.958	12.834	10.898	15.087	16.883
6	0.8573	34.112	18.480	25.984	36.942	100.631
6	0.8583	14.800	11.500	7.176	38.341	99.431
6	0.8593	14.947	12.373	9.543	31.839	66.970
6	0.8603	13.417	12.269	9.218	19.072	25.912
6	0.8613	15.853	12.059	12.919	21.445	35.673
6	0.8623	8.781	8.017	7.732	28.707	45.740
6	0.8633	15.206	14.208	10.417	12.309	45.304
6	0.8643	13.769	6.090	5.335	20.406	62.987
6	0.8653	20.291	11.695	8.386	29.524	50.273
6	0.8663	17.983	22.321	17.875	31.326	60.562
6	0.8673	15.437	18.508	11.448	38.870	67.087
6	0.8683	18.101	14.070	11.683	38.617	75.318
6	0.8693	17.465	9.280	11.186	33.488	80.069
6	0.8703	26.114	12.959	15.741	49.926	76.860
6	0.8713	21.970	13.843	16.389	30.749	49.897
6	0.8723	15.575	11.434	14.160	20.469	34.063
6	0.8733	19.486	23.096	14.712	20.342	30.656
6	0.8743	9.498	6.620	4.661	15.971	49.422
6	0.8753	26.006	10.249	8.793	35.491	73.996
6	0.8763	26.535	10.466	9.354	37.919	73.467
6	0.8773	34.680	22.318	13.695	32.253	74.453
6	0.8783	20.154	7.527	6.563	32.634	66.570
6	0.8793	9.592	7.997	7.860	31.218	60.674
6	0.8803	26.946	18.551	12.618	36.372	64.910
6	0.8813	11.079	15.149	6.857	11.828	13.123
6	0.8823	10.621	14.570	7.774	26.237	20.871
6	0.8833	17.125	15.701	16.795	24.583	23.567
6	0.8843	18.830	9.153	6.944	49.733	80.694
6	0.8853	10.845	11.619	8.777	40.211	73.098
6	0.8863	18.162	8.897	7.579	28.690	46.652
6	0.8873	24.629	21.710	17.618	28.681	32.883
6	0.8883	15.513	9.928	11.304	21.299	51.296
6	0.8893	24.035	17.641	15.765	32.529	69.154
6	0.8903	32.851	17.813	11.415	52.495	111.687
6	0.8913	26.516	12.206	10.890	30.058	72.169
6	0.8923	9.764	7.083	6.683	18.386	35.322
6	0.8933	16.596	9.373	6.881	31.538	51.637
6	0.8943	20.566	9.728	6.649	19.941	53.461
6	0.8953	22.855	9.682	6.855	28.144	72.009
6	0.8963	11.078	3.613	3.712	15.185	29.215
6	0.8973	19.268	12.780	12.095	21.046	42.551
6	0.8983	48.149	34.920	29.779	38.085	96.548
6	0.8993	17.898	17.890	15.762	33.989	44.040
6	0.9003	17.940	17.728	15.719	33.677	44.022
6	0.9013	20.126	6.754	8.587	23.701	52.278
6	0.9023	29.335	14.500	11.494	43.715	77.793
6	0.9033	20.078	14.317	11.268	32.807	67.512
6	0.9043	23.062	15.587	12.364	31.318	64.293

6	0.9053	31.559	16.325	13.374	19.591	72.044
6	0.9063	20.452	14.423	10.895	25.979	54.885
6	0.9073	20.472	11.631	7.415	18.236	53.400
6	0.9083	22.020	10.468	10.458	28.900	56.024
6	0.9093	22.709	4.709	8.225	40.866	62.838
6	0.9103	22.753	10.201	9.089	38.960	72.855
6	0.9113	20.203	9.624	9.768	20.454	49.518
6	0.9123	16.791	10.641	8.447	32.148	50.694
6	0.9133	21.499	11.603	11.608	25.453	66.043
6	0.9143	14.606	13.341	10.256	23.299	53.743
6	0.9153	23.189	11.996	10.505	27.505	73.155
6	0.9163	30.771	23.933	23.956	14.393	26.141
6	0.9173	13.506	10.669	7.512	23.982	44.369
6	0.9183	12.887	12.615	10.138	25.836	48.959
6	0.9193	22.897	11.877	9.318	30.775	61.972
6	0.9203	18.417	6.743	9.400	23.078	40.299
6	0.9213	22.299	14.109	12.476	36.616	60.750
6	0.9223	30.375	22.801	20.327	28.191	65.756
6	0.9233	20.011	13.235	6.433	22.239	63.159
6	0.9243	18.320	9.447	6.762	26.659	60.236
6	0.9253	17.375	11.712	7.639	25.195	52.597
6	0.9263	25.865	7.659	15.129	36.387	70.511
6	0.9273	14.489	8.406	6.840	17.355	45.681
6	0.9283	17.561	7.986	4.280	19.070	60.611
6	0.9293	15.484	12.267	6.207	22.474	52.274
6	0.9303	28.968	17.400	14.367	26.947	68.286
6	0.9313	18.086	11.873	6.245	29.496	71.402
6	0.9323	20.996	8.006	7.482	27.775	58.493
6	0.9333	15.130	12.394	6.677	25.204	50.318
6	0.9343	16.674	12.647	5.487	20.908	60.730
6	0.9353	12.403	13.528	6.669	24.886	54.496
6	0.9363	6.542	9.419	4.913	28.162	28.001
6	0.9373	21.372	16.626	15.850	29.207	36.573
6	0.9383	17.500	16.462	7.810	25.326	46.776
6	0.9393	24.556	14.739	12.985	41.443	47.480
6	0.9403	23.634	10.405	14.804	32.483	33.363
6	0.9413	12.991	13.155	8.255	25.309	40.479
6	0.9423	10.435	7.240	8.256	40.945	35.293
6	0.9433	17.785	6.567	8.154	40.060	57.646
6	0.9443	10.621	8.764	7.154	27.057	44.142
6	0.9453	7.396	7.227	4.833	12.390	32.851
6	0.9463	4.218	2.525	3.273	7.764	22.440
6	0.9473	14.775	10.883	8.494	19.860	33.985
6	0.9483	25.705	9.822	13.868	14.263	38.772
6	0.9493	25.758	9.758	13.956	14.073	38.766
6	0.9503	13.105	8.469	10.831	24.615	38.108
6	0.9513	20.658	15.182	15.054	29.486	41.901
6	0.9523	14.767	15.360	11.346	22.925	44.562
6	0.9533	12.956	14.151	11.431	17.513	25.094
6	0.9543	19.328	13.608	8.530	14.422	26.199

6	0.9553	15.794	11.566	10.258	13.435	34.217
6	0.9563	8.690	9.545	8.755	11.896	31.106
6	0.9573	18.627	15.181	16.449	8.543	32.818
6	0.9583	23.245	19.795	17.767	29.546	47.386
6	0.9593	12.385	10.290	11.131	14.626	28.719
6	0.9603	13.736	12.070	10.820	11.855	31.497
6	0.9613	16.155	12.674	8.440	19.549	52.794
6	0.9623	22.447	20.196	13.489	24.896	57.188
6	0.9633	26.284	19.091	14.788	27.252	56.094
6	0.9643	4.671	7.599	5.952	19.540	19.661
6	0.9653	13.343	15.495	8.532	18.222	23.663
6	0.9663	26.998	26.447	22.153	13.717	30.595
6	0.9673	22.596	22.526	19.690	11.956	23.804
6	0.9683	22.452	22.447	19.444	12.021	23.195
6	0.9693	12.683	6.424	6.493	9.583	25.100
6	0.9703	19.567	21.493	17.865	25.274	52.672
6	0.9713	34.518	18.793	22.424	29.588	37.905
6	0.9723	25.847	14.622	17.226	19.790	34.733
6	0.9733	32.922	11.010	21.575	25.662	37.169
6	0.9743	15.234	15.868	15.756	9.814	25.312
6	0.9753	21.812	19.772	10.336	15.297	17.170
6	0.9763	7.754	11.142	6.505	15.199	32.559
6	0.9773	25.835	19.283	14.866	19.090	38.485
6	0.9783	25.157	22.944	17.062	24.456	32.073
6	0.9793	10.480	13.986	7.681	20.475	22.269
6	0.9803	14.543	11.099	7.012	24.043	30.723
6	0.9813	37.326	27.276	24.861	22.685	28.444
6	0.9823	34.647	17.139	21.924	18.308	38.211
6	0.9833	32.834	28.175	24.106	23.761	36.758
6	0.9843	8.333	5.272	8.347	17.113	23.546
6	0.9853	22.227	14.171	17.885	10.232	23.342
6	0.9863	16.360	9.787	10.897	21.204	37.044
6	0.9873	19.994	10.890	17.333	21.200	27.538
6	0.9883	31.414	29.427	26.729	13.736	25.869
6	0.9893	15.404	17.712	17.622	17.352	28.234
6	0.9903	13.719	16.440	15.923	17.263	32.033
6	0.9913	17.409	19.464	17.835	18.576	26.194
6	0.9923	16.523	13.800	9.617	10.036	26.764
6	0.9933	12.755	16.241	10.383	8.072	20.992
6	0.9943	2.094	6.759	3.408	5.719	12.530
6	0.9953	19.879	13.527	8.772	13.602	28.113
6	0.9963	14.287	11.347	9.090	14.464	16.509
6	0.9973	13.590	18.306	10.602	23.045	29.982
6	0.9983	19.646	14.133	14.779	15.446	33.636
6	0.9993	19.887	26.776	23.708	17.316	32.899
6	1.0003	10.294	16.613	14.371	19.784	33.882
6	1.0013	12.822	18.375	18.529	13.388	28.864
6	1.0023	12.810	18.415	18.604	13.789	28.547
6	1.0033	27.771	31.195	22.106	18.049	32.945
6	1.0043	22.980	25.874	19.838	30.016	38.158

6	1.0053	18.927	17.410	14.684	30.451	33.283
6	1.0063	21.561	21.072	13.691	24.952	37.507
6	1.0073	35.438	22.657	20.303	22.786	42.664
6	1.0083	27.711	20.297	20.828	18.365	35.837
6	1.0093	15.465	24.536	11.833	19.568	18.034
6	1.0103	6.365	5.112	8.286	20.595	18.845
6	1.0113	10.448	15.793	6.770	23.916	25.425
6	1.0123	32.036	21.144	19.407	8.983	31.228
6	1.0133	17.547	17.773	14.922	27.671	27.687
6	1.0143	18.814	16.690	13.808	17.621	22.253
6	1.0153	16.757	22.711	11.988	24.095	32.134
6	1.0163	20.168	18.971	20.966	14.563	24.765
6	1.0173	24.678	22.558	19.156	17.117	25.479
6	1.0183	19.514	13.891	16.278	19.373	25.261
6	1.0193	12.532	11.699	9.189	22.684	28.960
6	1.0203	18.461	18.342	11.364	26.364	36.150
6	1.0213	22.348	21.938	13.137	29.984	35.826
6	1.0223	28.782	19.589	18.964	31.721	39.658
6	1.0233	22.848	14.361	16.083	18.701	29.231
6	1.0243	18.443	13.775	18.723	16.190	15.455
6	1.0253	21.578	18.566	16.515	22.102	14.455
6	1.0263	20.858	18.246	15.864	22.197	15.874
6	1.0273	23.809	14.546	16.582	15.606	25.804
6	1.0283	23.577	14.352	16.541	15.570	25.773
6	1.0293	18.566	12.871	19.530	11.904	14.531
6	1.0303	17.586	12.417	13.446	15.118	14.721
6	1.0313	17.844	19.023	12.604	8.814	15.165
6	1.0323	19.476	15.533	13.215	11.591	19.449
6	1.0333	13.260	18.326	11.640	7.833	21.213
6	1.0343	20.171	21.431	15.893	17.511	27.181
6	1.0353	11.345	10.124	9.251	12.396	13.093
6	1.0363	9.399	16.545	10.952	26.158	15.607
6	1.0373	16.610	21.583	20.276	15.806	15.455
6	1.0383	17.686	19.974	18.828	18.224	19.431
6	1.0393	23.178	25.527	26.138	18.915	29.894
6	1.0403	15.347	19.993	17.817	11.672	22.826
6	1.0413	17.698	28.262	15.768	10.382	29.823
6	1.0423	21.686	23.074	23.014	18.489	32.519
6	1.0433	9.003	5.105	8.506	14.214	21.333
6	1.0443	14.208	21.156	20.861	29.798	17.627
6	1.0453	17.396	19.522	17.790	24.253	27.489
6	1.0463	10.346	7.783	12.816	5.962	28.765
6	1.0473	13.513	15.806	18.609	13.462	31.669
6	1.0483	10.942	13.237	16.541	15.876	18.582
6	1.0493	8.875	10.293	12.126	24.351	22.614
6	1.0503	22.667	17.105	24.515	21.109	29.573
6	1.0513	11.012	14.195	15.170	18.181	18.228
6	1.0523	9.233	12.944	14.434	15.620	26.362
6	1.0533	17.882	16.207	19.858	8.464	26.936
6	1.0543	17.282	17.659	21.309	30.082	29.189

6	1.0553	9.771	9.860	18.589	14.284	21.777
6	1.0563	18.289	9.648	21.542	10.641	17.387
6	1.0573	19.288	13.353	27.527	8.076	19.509
6	1.0583	22.902	21.559	22.364	12.610	16.381
6	1.0593	13.693	11.281	12.837	11.396	27.383
6	1.0603	13.270	8.518	14.124	11.426	31.170
6	1.0613	21.767	15.206	18.693	6.914	21.326
6	1.0623	7.685	8.108	13.302	8.591	9.028
6	1.0633	17.271	11.816	17.449	12.223	28.855
6	1.0643	12.385	8.417	14.820	12.033	22.222
6	1.0653	7.404	4.617	9.093	9.601	14.871
6	1.0663	4.790	3.151	4.532	13.766	14.464
6	1.0673	3.884	7.449	9.222	18.743	13.240
6	1.0683	3.438	12.869	6.966	12.047	11.367
6	1.0693	12.930	12.139	21.348	12.327	12.902
6	1.0703	10.935	10.348	13.999	8.960	9.978
6	1.0713	12.300	13.949	14.732	15.536	17.013
6	1.0723	8.858	11.517	12.912	20.989	18.574
6	1.0733	16.325	16.085	18.081	12.245	23.472
6	1.0743	12.419	9.642	9.539	13.521	16.839
6	1.0753	12.023	5.404	7.848	24.953	24.410
6	1.0763	13.177	11.660	14.129	14.165	15.217
6	1.0773	17.344	17.552	21.242	5.898	11.391
6	1.0783	13.809	9.375	8.481	30.233	20.940
6	1.0793	20.215	22.635	15.373	32.270	23.278
6	1.0803	9.525	5.978	10.110	23.642	14.728
6	1.0813	13.933	12.331	13.819	34.478	15.613
6	1.0823	11.354	14.801	14.218	18.884	20.707
6	1.0833	4.648	9.776	10.326	16.612	18.064
6	1.0843	6.510	5.822	6.421	24.908	32.014
6	1.0853	28.903	14.627	24.683	13.067	20.682
6	1.0863	16.350	14.316	16.475	14.056	15.051
6	1.0873	13.397	10.909	14.160	24.184	15.691
6	1.0883	8.012	9.056	11.525	15.336	13.060
6	1.0893	11.431	8.856	14.072	14.287	23.006
6	1.0903	15.026	17.727	17.273	23.319	18.575
6	1.0913	15.007	16.086	19.236	15.893	14.394
6	1.0923	17.595	15.742	22.424	6.168	11.641
6	1.0933	20.900	19.325	20.463	14.656	15.173
6	1.0943	20.920	19.281	20.504	14.887	15.460
6	1.0953	11.256	14.841	15.976	12.311	17.932
6	1.0963	8.213	12.535	11.873	13.622	16.171
6	1.0973	6.333	7.239	11.060	13.010	27.613
6	1.0983	6.411	8.512	10.547	10.484	10.285
6	1.0993	9.585	8.203	11.233	6.134	14.087
6	1.1003	10.268	8.752	11.902	5.900	13.857
6	1.1013	8.499	12.835	14.902	15.807	15.355
6	1.1023	7.414	13.761	14.389	13.342	14.429
6	1.1033	12.771	22.163	18.420	15.224	14.951
6	1.1043	15.505	17.565	15.169	23.024	9.847

6	1.1053	7.673	8.657	9.401	12.052	10.920
6	1.1063	8.705	12.308	10.004	6.092	5.512
6	1.1073	17.436	19.682	19.529	16.442	13.325
6	1.1083	10.802	19.717	15.840	11.025	10.757
6	1.1093	14.956	22.094	22.109	13.707	10.809
6	1.1103	15.654	21.051	21.299	13.117	7.977
6	1.1113	7.253	5.499	11.081	3.626	4.993
6	1.1123	7.445	9.740	12.257	12.073	5.220
6	1.1133	5.184	4.913	7.885	8.274	7.366
6	1.1143	10.265	13.356	18.269	11.167	8.027
6	1.1153	9.713	14.903	20.640	13.920	12.231
6	1.1163	7.427	15.043	20.402	18.936	11.783
6	1.1173	6.866	13.251	16.542	10.905	16.165
6	1.1183	13.620	16.083	22.882	4.177	14.885
6	1.1193	16.640	22.321	21.737	7.665	12.138
6	1.1203	8.798	10.608	11.515	12.269	7.720
6	1.1213	11.477	11.548	13.084	14.834	9.542
6	1.1223	10.999	10.128	13.489	14.105	9.501
6	1.1233	8.980	12.389	8.043	17.850	4.513
6	1.1243	7.401	7.386	11.112	10.452	11.154
6	1.1253	20.134	18.543	16.823	12.553	14.146
6	1.1263	15.303	20.483	21.733	23.408	17.440
6	1.1273	13.782	13.536	15.544	10.803	12.468
6	1.1283	13.091	11.863	13.624	10.696	12.488
6	1.1293	13.197	16.811	24.181	8.283	15.888
6	1.1303	11.693	18.575	17.726	15.661	11.307
6	1.1313	14.054	22.766	14.325	20.033	11.107
6	1.1323	16.128	22.375	24.257	22.769	16.687
6	1.1333	18.602	19.923	23.398	16.330	16.087
6	1.1343	12.413	11.261	13.580	9.933	14.059
6	1.1353	21.564	17.179	28.837	4.667	15.003
6	1.1363	22.109	18.849	25.514	10.312	14.089
6	1.1373	16.160	17.572	21.232	11.497	6.632
6	1.1383	21.969	26.433	22.991	15.568	14.206
6	1.1393	11.823	14.948	13.746	20.495	11.870
6	1.1403	22.242	22.267	23.477	21.281	12.379
6	1.1413	11.621	11.559	8.667	14.887	9.669
6	1.1423	7.440	9.692	7.001	15.743	10.313
6	1.1433	0.000	0.000	0.000	0.000	0.000
7	0.0002	0.000	0.000	0.000	0.000	0.000
7	0.0012	13.170	8.764	12.471	8.709	13.473
7	0.0022	12.478	12.725	11.756	8.979	14.982
7	0.0032	15.653	14.187	16.758	20.756	18.007
7	0.0042	16.940	17.235	14.431	15.308	35.299
7	0.0052	26.378	17.622	18.134	23.528	21.818
7	0.0062	15.166	12.399	8.579	9.876	25.507
7	0.0072	11.992	7.565	6.553	7.983	24.946
7	0.0082	13.272	18.740	15.948	7.591	20.081
7	0.0092	16.776	19.087	15.471	9.250	20.464
7	0.0102	14.580	16.763	17.558	7.675	16.608

7	0.0112	21.981	18.561	21.483	9.358	16.897
7	0.0122	13.380	17.684	15.742	13.826	19.102
7	0.0132	5.490	14.310	6.349	14.918	19.531
7	0.0142	11.635	19.869	9.688	12.903	12.626
7	0.0152	9.695	22.348	18.264	8.585	11.951
7	0.0162	14.614	21.468	14.749	9.895	15.540
7	0.0172	15.696	20.376	14.543	11.081	13.644
7	0.0182	10.775	19.516	13.525	13.292	11.534
7	0.0192	21.840	20.972	17.922	13.451	15.189
7	0.0202	11.081	16.616	14.120	12.267	9.893
7	0.0212	8.533	14.384	12.205	14.482	8.620
7	0.0222	13.054	17.627	19.148	17.351	25.936
7	0.0232	12.961	17.611	19.090	17.319	25.815
7	0.0242	15.185	17.639	15.477	17.487	20.583
7	0.0252	12.053	17.131	15.336	11.646	10.140
7	0.0262	32.838	21.056	22.061	17.980	16.022
7	0.0272	21.059	14.180	20.188	18.031	19.331
7	0.0282	17.892	16.268	16.640	11.047	18.481
7	0.0292	11.801	11.667	11.990	11.550	14.768
7	0.0302	11.584	15.587	14.681	17.403	15.339
7	0.0312	12.502	9.170	12.420	6.717	19.075
7	0.0322	26.359	26.136	36.941	11.569	15.061
7	0.0332	11.784	17.284	15.212	16.941	15.411
7	0.0342	17.543	13.952	17.110	8.629	12.239
7	0.0352	15.762	15.073	17.173	7.220	13.621
7	0.0362	15.928	20.208	17.260	12.265	20.695
7	0.0372	21.208	20.389	24.093	5.236	9.145
7	0.0382	18.391	19.193	21.177	9.373	6.431
7	0.0392	22.680	22.792	26.866	11.362	21.393
7	0.0402	11.203	12.750	16.403	8.798	18.806
7	0.0412	14.888	17.457	19.800	10.728	16.661
7	0.0422	7.476	11.435	11.383	6.532	7.297
7	0.0432	6.671	10.257	11.568	10.118	10.756
7	0.0442	7.527	15.391	12.873	10.974	7.824
7	0.0452	6.450	6.291	9.858	7.352	24.528
7	0.0462	16.674	12.020	22.941	14.548	18.281
7	0.0472	16.233	11.385	15.983	11.437	9.164
7	0.0482	13.868	11.734	15.978	3.671	4.283
7	0.0492	19.785	11.671	15.253	7.204	8.973
7	0.0502	12.514	10.436	11.969	6.791	8.126
7	0.0512	15.716	10.260	11.515	14.310	15.367
7	0.0522	16.045	19.402	19.359	22.330	11.644
7	0.0532	20.959	20.513	22.886	12.765	16.340
7	0.0542	12.912	13.381	15.335	14.144	9.760
7	0.0552	7.238	9.931	8.721	14.555	17.237
7	0.0562	6.518	7.499	6.865	22.153	14.044
7	0.0572	14.047	11.069	18.458	20.070	16.334
7	0.0582	17.741	11.744	15.957	18.162	13.773
7	0.0592	14.189	7.828	11.024	20.895	13.270
7	0.0602	4.167	4.103	2.880	10.881	6.120

7	0.0612	5.827	6.875	5.541	16.912	14.157
7	0.0622	12.315	9.077	8.540	25.126	14.190
7	0.0632	17.957	11.507	13.703	23.711	25.372
7	0.0642	11.359	13.657	13.404	17.839	13.793
7	0.0652	16.070	19.102	17.017	22.771	19.457
7	0.0662	12.919	15.539	15.110	27.176	26.561
7	0.0672	18.104	11.225	12.834	29.917	29.544
7	0.0682	37.084	25.597	24.660	30.757	18.962
7	0.0692	6.568	8.346	8.253	8.013	5.448
7	0.0702	29.863	17.951	24.607	28.949	12.901
7	0.0712	16.361	11.147	19.550	24.307	10.180
7	0.0722	19.294	12.491	24.699	13.753	7.755
7	0.0732	19.309	10.813	19.835	15.868	12.939
7	0.0742	18.486	17.588	22.758	21.688	5.407
7	0.0752	18.275	17.346	22.677	20.986	5.328
7	0.0762	18.828	10.750	14.737	29.036	5.330
7	0.0772	25.016	16.220	18.274	22.076	10.614
7	0.0782	28.337	18.273	18.940	35.452	24.834
7	0.0792	28.065	12.055	21.954	41.173	20.796
7	0.0802	17.637	17.416	18.539	19.944	16.794
7	0.0812	17.531	17.668	18.282	19.228	15.877
7	0.0822	11.449	10.665	13.725	11.916	6.270
7	0.0832	29.060	21.707	24.335	13.869	7.796
7	0.0842	7.443	9.839	9.306	5.449	4.931
7	0.0852	8.322	5.859	8.271	10.553	4.506
7	0.0862	24.286	14.147	33.272	20.741	15.179
7	0.0872	19.591	15.845	25.414	20.210	18.577
7	0.0882	25.233	15.018	23.067	21.699	14.185
7	0.0892	16.071	12.293	23.095	18.261	7.264
7	0.0902	17.041	27.924	28.847	30.681	7.264
7	0.0912	25.546	14.391	33.463	21.650	12.239
7	0.0922	15.983	13.157	23.039	9.477	8.901
7	0.0932	18.489	15.543	17.923	16.472	6.637
7	0.0942	6.313	8.089	3.326	13.091	12.758
7	0.0952	18.461	18.947	21.252	15.522	12.561
7	0.0962	18.355	18.675	21.107	15.269	12.556
7	0.0972	12.070	7.882	12.688	13.557	7.110
7	0.0982	14.764	12.196	12.762	22.375	10.884
7	0.0992	10.324	15.583	11.991	16.812	12.550
7	0.1002	20.928	15.954	25.862	20.135	8.081
7	0.1012	30.586	29.075	34.492	5.856	7.391
7	0.1022	18.281	17.052	19.762	13.585	14.236
7	0.1032	6.807	10.417	6.365	15.125	10.054
7	0.1042	6.789	8.428	6.202	8.883	12.807
7	0.1052	10.472	8.860	8.604	7.060	9.581
7	0.1062	8.337	9.342	8.011	10.886	9.896
7	0.1072	6.008	7.995	9.595	16.301	20.760
7	0.1082	12.095	8.789	17.679	14.029	9.396
7	0.1092	12.093	8.776	17.635	14.060	9.518
7	0.1102	17.575	14.837	17.635	10.245	17.926

7	0.1112	21.493	23.052	29.808	16.591	14.118
7	0.1122	9.808	10.519	10.424	16.732	17.462
7	0.1132	8.408	7.768	6.257	9.404	6.150
7	0.1142	20.962	12.177	23.143	11.316	16.053
7	0.1152	24.307	17.813	21.990	17.866	16.302
7	0.1162	14.019	17.314	12.368	16.482	16.988
7	0.1172	34.858	28.140	30.662	26.768	17.627
7	0.1182	13.775	17.703	16.387	11.799	9.240
7	0.1192	13.741	17.641	16.340	11.764	9.188
7	0.1202	7.294	11.918	7.297	15.011	11.190
7	0.1212	7.284	11.917	7.275	14.977	11.135
7	0.1222	9.533	9.005	10.448	14.335	14.275
7	0.1232	14.717	7.943	9.357	12.818	11.961
7	0.1242	20.098	12.060	15.793	14.845	13.342
7	0.1252	16.455	12.171	19.359	14.662	9.840
7	0.1262	28.705	18.352	26.663	14.199	14.321
7	0.1272	22.197	20.063	21.297	13.676	10.489
7	0.1282	41.693	33.464	34.075	16.424	12.102
7	0.1292	38.051	33.402	34.276	20.832	12.986
7	0.1302	34.575	35.226	33.500	24.771	17.009
7	0.1312	9.134	10.925	10.740	12.718	12.135
7	0.1322	20.192	19.482	28.026	11.810	19.515
7	0.1332	30.312	24.184	35.935	14.642	15.089
7	0.1342	25.869	20.573	17.808	15.530	11.151
7	0.1352	13.000	16.319	10.909	9.251	17.592
7	0.1362	22.786	16.687	15.785	14.584	28.095
7	0.1372	23.037	17.226	16.854	11.430	21.593
7	0.1382	28.462	22.706	23.382	23.013	26.402
7	0.1392	27.558	19.800	24.471	15.624	15.367
7	0.1402	28.260	20.776	20.797	13.068	27.862
7	0.1412	23.395	21.783	19.730	9.633	18.836
7	0.1422	19.576	26.766	23.691	10.408	8.340
7	0.1432	19.596	26.840	23.750	10.436	8.157
7	0.1442	20.576	19.508	14.746	9.614	12.562
7	0.1452	12.912	20.939	15.834	11.813	13.796
7	0.1462	23.577	26.747	22.245	12.172	8.036
7	0.1472	16.988	9.737	9.874	4.649	10.989
7	0.1482	20.680	16.716	13.607	11.270	19.896
7	0.1492	23.135	22.867	22.955	10.935	19.631
7	0.1502	20.793	22.673	18.365	5.413	12.294
7	0.1512	15.050	18.503	16.586	3.946	9.066
7	0.1522	28.729	30.692	26.646	10.458	26.307
7	0.1532	23.837	29.509	29.028	20.028	19.921
7	0.1542	13.413	18.535	17.619	23.657	20.962
7	0.1552	10.196	8.302	9.416	8.771	4.889
7	0.1562	9.452	11.090	8.952	7.077	13.902
7	0.1572	16.446	15.621	15.380	13.921	15.819
7	0.1582	11.936	13.886	16.055	11.161	18.554
7	0.1592	13.221	23.239	14.421	13.099	15.062
7	0.1602	11.600	15.968	13.363	8.918	12.239

7	0.1612	16.780	12.327	10.066	10.943	17.708
7	0.1622	28.202	22.351	17.541	15.618	16.086
7	0.1632	15.756	8.687	7.410	12.171	16.901
7	0.1642	15.724	8.676	7.436	12.179	16.903
7	0.1652	4.373	3.279	1.962	5.347	4.834
7	0.1662	10.433	10.594	7.927	8.067	20.492
7	0.1672	20.541	18.562	15.365	14.594	12.018
7	0.1682	21.814	14.215	18.038	15.125	20.963
7	0.1692	18.561	14.563	17.824	12.962	20.152
7	0.1702	39.250	30.361	29.836	9.851	9.972
7	0.1712	22.314	18.436	13.329	14.778	25.652
7	0.1722	15.502	19.065	13.866	12.451	19.111
7	0.1732	17.598	16.895	13.606	3.386	4.090
7	0.1742	23.885	20.468	16.647	6.027	6.599
7	0.1752	20.814	24.263	22.395	8.952	6.820
7	0.1762	20.826	24.254	22.391	8.928	6.816
7	0.1772	12.843	10.854	11.816	9.186	10.749
7	0.1782	13.498	5.887	10.518	5.015	6.421
7	0.1792	13.463	5.879	10.491	5.004	6.439
7	0.1802	7.573	8.072	9.763	2.110	2.834
7	0.1812	10.141	10.191	11.268	9.444	10.158
7	0.1822	10.975	10.010	10.544	6.940	5.744
7	0.1832	28.606	28.068	21.472	7.572	12.887
7	0.1842	21.665	27.066	18.684	11.754	12.455
7	0.1852	31.647	28.927	27.630	16.343	8.142
7	0.1862	37.006	22.398	24.141	21.488	16.153
7	0.1872	12.227	9.128	8.599	10.700	13.928
7	0.1882	29.194	36.254	32.181	11.342	5.572
7	0.1892	32.589	38.280	33.942	12.364	8.646
7	0.1902	17.313	15.496	14.250	1.951	7.299
7	0.1912	2.008	2.446	2.536	3.107	11.683
7	0.1922	4.078	3.525	2.640	6.026	17.253
7	0.1932	10.089	7.072	9.912	6.438	19.071
7	0.1942	10.308	9.683	10.985	5.718	10.573
7	0.1952	33.003	33.976	31.056	14.720	10.373
7	0.1962	16.610	18.902	17.398	12.679	11.870
7	0.1972	21.137	19.527	17.376	11.074	17.535
7	0.1982	13.461	9.523	10.957	9.892	18.398
7	0.1992	17.819	13.320	17.510	7.236	9.968
7	0.2002	24.985	18.831	27.326	11.979	5.413
7	0.2012	20.565	14.668	22.424	5.511	7.256
7	0.2022	17.229	15.864	19.566	7.793	9.999
7	0.2032	21.353	16.506	23.826	9.456	4.501
7	0.2042	12.427	14.329	14.640	8.170	8.635
7	0.2052	5.497	12.426	9.392	11.305	8.846
7	0.2062	16.058	12.507	26.813	6.872	19.475
7	0.2072	24.049	21.722	22.590	12.419	16.653
7	0.2082	38.602	22.081	34.387	15.439	7.680
7	0.2092	25.816	26.173	28.328	14.067	10.106
7	0.2102	23.304	15.846	19.205	13.428	12.044

7	0.2112	12.834	10.716	12.160	15.785	15.118
7	0.2122	18.963	21.227	19.327	10.919	16.893
7	0.2132	20.541	19.483	18.495	5.403	9.716
7	0.2142	14.007	7.774	14.254	12.893	12.410
7	0.2152	11.784	9.664	14.321	6.932	18.719
7	0.2162	25.388	16.287	26.165	17.451	8.433
7	0.2172	32.888	32.043	32.987	10.972	12.983
7	0.2182	22.511	9.894	15.334	11.403	11.001
7	0.2192	20.303	13.266	20.188	11.525	7.208
7	0.2202	19.940	19.193	20.504	7.386	26.507
7	0.2212	18.660	17.068	21.422	12.096	11.387
7	0.2222	8.797	12.194	13.491	13.792	10.824
7	0.2232	26.221	13.727	16.925	15.381	12.440
7	0.2242	21.212	16.176	15.689	10.558	12.062
7	0.2252	29.482	27.840	37.667	24.973	11.764
7	0.2262	30.686	24.316	34.535	12.511	9.277
7	0.2272	25.648	25.792	27.399	8.450	13.275
7	0.2282	23.979	20.911	18.733	9.859	13.373
7	0.2292	30.172	27.794	27.615	15.142	8.995
7	0.2302	23.616	29.180	20.617	17.686	7.768
7	0.2312	36.298	40.088	36.101	12.574	9.381
7	0.2322	26.852	29.642	31.205	12.509	13.501
7	0.2332	22.991	36.308	26.690	14.125	18.447
7	0.2342	11.198	16.506	12.296	8.338	16.520
7	0.2352	9.648	8.705	12.465	12.040	16.076
7	0.2362	25.506	20.913	24.422	6.044	11.722
7	0.2372	13.809	11.960	8.866	7.016	26.727
7	0.2382	14.892	6.404	9.575	9.009	16.746
7	0.2392	21.788	11.480	13.644	9.663	18.256
7	0.2402	12.295	12.613	12.502	6.899	3.814
7	0.2412	9.767	14.321	13.817	8.789	10.721
7	0.2422	6.393	4.770	4.456	11.898	16.644
7	0.2432	7.687	8.584	8.546	22.108	23.692
7	0.2442	7.168	9.502	5.620	13.912	26.238
7	0.2452	14.944	10.542	8.193	17.743	22.701
7	0.2462	12.508	7.260	10.822	12.702	12.870
7	0.2472	26.433	30.161	18.626	9.792	15.205
7	0.2482	16.612	29.041	14.162	10.174	16.048
7	0.2492	17.967	26.152	18.173	8.556	13.213
7	0.2502	27.421	27.963	22.257	6.621	31.598
7	0.2512	15.140	13.206	13.468	13.303	13.805
7	0.2522	23.437	20.581	21.498	19.463	21.152
7	0.2532	13.704	18.828	12.711	26.325	18.005
7	0.2542	9.054	12.456	7.836	19.043	18.077
7	0.2552	24.359	37.381	26.151	27.776	23.291
7	0.2562	14.317	14.976	13.918	18.665	10.179
7	0.2572	21.802	19.611	25.899	21.047	6.893
7	0.2582	23.721	30.568	28.289	21.804	21.671
7	0.2592	10.238	7.998	7.303	17.237	9.972
7	0.2602	25.194	19.609	17.062	16.511	20.473

7	0.2612	18.451	15.174	12.204	15.511	9.196
7	0.2622	30.299	36.225	30.802	23.154	9.260
7	0.2632	30.946	31.574	25.470	17.761	10.036
7	0.2642	25.697	15.378	18.266	14.992	16.118
7	0.2652	25.734	15.411	18.312	14.895	16.085
7	0.2662	15.939	12.889	11.693	18.963	16.791
7	0.2672	22.105	14.857	16.179	14.768	23.942
7	0.2682	23.843	15.536	15.003	20.171	22.228
7	0.2692	29.571	26.605	24.402	13.928	23.657
7	0.2702	25.874	20.097	23.501	9.237	14.723
7	0.2712	25.679	16.396	19.419	13.263	12.020
7	0.2722	16.481	17.480	17.672	21.260	10.382
7	0.2732	26.841	29.069	29.026	14.038	10.059
7	0.2742	24.194	28.520	32.699	13.497	10.763
7	0.2752	14.536	14.133	17.671	16.165	10.633
7	0.2762	22.756	14.129	20.499	13.517	9.351
7	0.2772	19.503	13.058	16.722	12.036	9.539
7	0.2782	16.744	10.124	7.703	10.723	13.337
7	0.2792	16.752	10.155	7.839	10.767	13.286
7	0.2802	19.933	19.228	10.736	13.780	9.039
7	0.2812	10.154	15.132	10.209	18.831	10.901
7	0.2822	19.280	24.732	17.656	24.580	8.708
7	0.2832	23.585	21.396	24.563	18.124	13.048
7	0.2842	44.297	33.372	38.967	11.918	12.097
7	0.2852	29.641	34.548	29.498	14.677	7.831
7	0.2862	30.294	38.653	33.649	17.321	6.907
7	0.2872	17.021	17.547	17.738	19.040	8.360
7	0.2882	22.353	17.416	19.362	12.019	5.762
7	0.2892	22.039	27.656	20.934	16.177	5.311
7	0.2902	17.067	15.603	13.551	15.680	13.787
7	0.2912	12.857	15.708	16.488	19.255	12.302
7	0.2922	9.915	15.458	12.180	19.021	5.682
7	0.2932	27.924	16.934	26.438	20.668	7.881
7	0.2942	20.939	15.575	19.608	26.199	8.399
7	0.2952	6.201	5.309	6.705	13.181	5.421
7	0.2962	18.452	12.462	17.563	15.793	15.346
7	0.2972	26.573	26.797	32.097	13.009	16.945
7	0.2982	29.014	30.094	37.581	13.936	10.370
7	0.2992	30.611	21.936	30.342	17.968	16.228
7	0.3002	32.403	22.147	30.942	17.976	11.771
7	0.3012	29.728	19.046	29.683	19.569	10.869
7	0.3022	22.589	15.885	22.569	20.154	15.087
7	0.3032	30.150	16.096	25.792	9.648	4.906
7	0.3042	25.306	25.272	31.575	12.757	6.430
7	0.3052	25.303	25.265	31.558	12.756	6.427
7	0.3062	21.718	14.813	23.283	12.711	4.449
7	0.3072	14.220	9.414	13.881	8.936	4.538
7	0.3082	20.996	13.414	14.797	8.954	7.925
7	0.3092	20.017	17.423	20.032	8.473	9.682
7	0.3102	15.560	13.166	14.957	12.999	8.430

7	0.3112	14.727	15.814	15.200	13.091	9.734
7	0.3122	28.421	28.886	31.964	10.888	9.861
7	0.3132	29.601	31.440	28.600	16.749	13.229
7	0.3142	21.728	25.354	30.345	16.005	10.320
7	0.3152	22.494	29.810	27.664	15.141	2.145
7	0.3162	24.866	20.746	28.351	9.696	3.839
7	0.3172	20.464	22.174	19.816	11.029	9.669
7	0.3182	24.477	24.967	33.196	12.896	10.099
7	0.3192	22.721	22.491	20.652	18.908	13.423
7	0.3202	22.514	22.654	24.641	13.713	7.199
7	0.3212	23.437	16.905	20.291	19.242	8.324
7	0.3222	25.099	13.006	19.846	14.506	7.579
7	0.3232	31.800	15.912	24.026	11.959	7.248
7	0.3242	14.739	11.843	16.843	9.919	14.503
7	0.3252	15.418	18.162	22.587	13.125	8.200
7	0.3262	10.864	6.089	13.212	16.380	18.191
7	0.3272	14.604	7.940	14.315	19.585	26.948
7	0.3282	14.946	12.946	14.399	17.793	8.193
7	0.3292	11.342	5.999	11.427	11.259	10.503
7	0.3302	6.776	5.951	10.987	3.212	9.507
7	0.3312	27.945	32.874	31.230	29.070	16.252
7	0.3322	21.477	19.576	19.184	8.675	8.596
7	0.3332	17.581	13.807	13.196	11.204	17.999
7	0.3342	16.049	15.606	18.742	10.172	9.548
7	0.3352	19.938	17.542	19.475	18.639	10.725
7	0.3362	18.696	15.379	19.315	21.093	12.868
7	0.3372	11.820	15.791	13.740	29.940	14.094
7	0.3382	18.549	23.450	25.642	13.662	24.244
7	0.3392	18.326	15.912	20.291	11.259	12.622
7	0.3402	20.019	23.977	20.299	24.577	21.359
7	0.3412	23.244	19.812	22.341	25.575	16.247
7	0.3422	19.926	14.652	23.066	21.813	14.654
7	0.3432	14.632	16.896	20.085	17.675	8.660
7	0.3442	20.337	16.085	26.886	27.434	22.437
7	0.3452	29.059	17.166	32.123	25.150	25.281
7	0.3462	16.265	11.699	18.740	14.642	19.303
7	0.3472	7.185	10.584	11.894	3.230	12.287
7	0.3482	22.158	16.628	12.746	7.941	9.169
7	0.3492	9.643	10.729	6.429	4.803	2.082
7	0.3502	16.451	30.349	20.345	7.642	8.819
7	0.3512	13.594	16.880	14.673	12.887	16.530
7	0.3522	14.618	21.575	16.381	12.210	10.997
7	0.3532	14.074	9.074	9.095	12.253	14.470
7	0.3542	11.724	8.241	11.129	14.229	12.065
7	0.3552	15.378	15.750	17.148	16.296	15.417
7	0.3562	27.833	21.178	26.315	18.316	9.728
7	0.3572	35.468	34.667	38.624	20.319	17.369
7	0.3582	29.258	19.545	30.313	12.956	14.291
7	0.3592	29.169	19.516	30.351	12.905	14.246
7	0.3602	13.549	20.911	19.677	16.263	11.103

7	0.3612	14.572	22.733	21.868	25.707	20.935
7	0.3622	16.184	15.997	16.953	9.589	12.351
7	0.3632	9.656	11.443	13.771	9.167	7.975
7	0.3642	16.604	11.770	17.037	7.995	15.454
7	0.3652	10.115	9.284	13.374	6.768	7.496
7	0.3662	13.602	21.420	21.866	10.417	11.612
7	0.3672	13.302	14.120	18.624	12.505	11.239
7	0.3682	16.145	8.920	13.179	10.908	10.272
7	0.3692	36.233	21.393	27.381	15.330	13.307
7	0.3702	17.622	11.911	15.189	14.480	19.800
7	0.3712	42.367	27.712	35.354	9.543	22.359
7	0.3722	25.171	25.145	23.880	10.115	8.719
7	0.3732	38.195	39.311	43.735	10.220	18.026
7	0.3742	19.179	17.267	18.516	17.191	12.646
7	0.3752	16.879	11.251	17.499	10.957	8.104
7	0.3762	27.706	32.764	33.982	18.002	5.519
7	0.3772	18.617	26.011	22.256	13.810	7.552
7	0.3782	14.361	10.085	15.501	7.612	12.575
7	0.3792	24.572	23.991	36.200	6.360	16.190
7	0.3802	24.614	24.012	36.257	6.360	16.209
7	0.3812	27.006	33.155	37.318	11.465	20.073
7	0.3822	26.992	33.102	37.303	11.445	20.075
7	0.3832	31.928	29.276	34.041	13.676	16.988
7	0.3842	34.585	39.925	41.618	16.762	12.968
7	0.3852	14.838	18.994	19.767	18.736	8.730
7	0.3862	11.264	15.635	9.138	20.829	14.684
7	0.3872	11.823	16.229	14.695	13.479	11.560
7	0.3882	26.244	34.241	30.952	32.919	15.410
7	0.3892	26.299	36.506	30.743	16.719	15.098
7	0.3902	29.595	32.621	28.190	12.208	16.191
7	0.3912	27.453	35.853	30.029	13.977	16.108
7	0.3922	29.332	33.030	33.955	14.480	16.980
7	0.3932	24.975	24.092	26.980	22.427	11.902
7	0.3942	24.999	24.032	26.937	22.373	11.900
7	0.3952	19.308	19.911	18.901	12.664	21.511
7	0.3962	13.519	16.248	11.415	9.725	14.360
7	0.3972	11.640	12.478	11.171	10.646	9.607
7	0.3982	16.072	11.636	12.455	9.530	8.533
7	0.3992	16.171	11.662	12.537	9.577	8.563
7	0.4002	12.611	11.130	10.656	4.152	3.747
7	0.4012	7.873	11.276	16.787	10.210	16.728
7	0.4022	9.526	13.203	16.446	12.159	8.189
7	0.4032	22.336	7.739	18.307	17.786	6.297
7	0.4042	21.066	14.012	20.863	11.523	14.949
7	0.4052	19.387	26.716	24.468	15.489	13.329
7	0.4062	15.955	20.505	19.499	10.439	11.859
7	0.4072	25.425	25.097	29.454	6.044	12.672
7	0.4082	19.117	25.699	26.184	12.809	18.175
7	0.4092	7.811	13.413	9.808	11.044	13.632
7	0.4102	5.953	12.616	6.201	10.331	19.263

7	0.4112	22.808	20.473	25.784	4.848	20.310
7	0.4122	26.099	17.785	26.924	11.438	16.688
7	0.4132	9.454	23.514	17.575	13.412	19.965
7	0.4142	22.245	25.273	28.573	17.698	32.568
7	0.4152	29.421	35.392	34.415	16.968	19.871
7	0.4162	31.256	25.021	33.115	10.218	19.363
7	0.4172	35.900	34.160	37.839	21.377	20.163
7	0.4182	27.481	28.675	34.267	16.119	22.091
7	0.4192	28.848	25.682	32.658	12.850	15.684
7	0.4202	20.171	20.443	26.540	8.009	3.955
7	0.4212	20.916	24.229	28.652	8.802	1.626
7	0.4222	27.212	29.570	31.904	13.198	14.561
7	0.4232	12.018	8.761	14.133	11.952	18.469
7	0.4242	6.803	12.573	10.597	8.274	6.684
7	0.4252	13.236	15.835	18.426	11.983	15.898
7	0.4262	11.314	15.433	16.868	8.627	11.329
7	0.4272	28.702	32.067	31.320	15.292	12.327
7	0.4282	19.405	22.816	20.079	12.507	13.638
7	0.4292	10.685	10.949	15.384	10.749	16.797
7	0.4302	16.342	8.825	13.505	8.866	4.910
7	0.4312	19.253	11.557	15.520	10.023	9.919
7	0.4322	11.118	5.434	8.800	9.526	3.910
7	0.4332	15.354	12.295	15.232	16.685	10.979
7	0.4342	18.682	23.808	22.171	7.526	5.133
7	0.4352	25.954	25.641	28.975	17.242	10.448
7	0.4362	28.374	25.084	31.775	12.426	8.880
7	0.4372	15.920	14.167	19.183	9.332	5.816
7	0.4382	9.782	10.167	8.966	7.003	6.526
7	0.4392	14.939	9.207	15.079	17.802	7.996
7	0.4402	14.342	12.349	15.836	7.997	15.280
7	0.4412	13.454	9.523	10.700	13.882	14.132
7	0.4422	15.770	11.678	15.150	18.092	12.290
7	0.4432	19.016	16.610	20.214	12.646	15.178
7	0.4442	12.348	13.949	20.265	11.690	16.480
7	0.4452	18.597	16.299	26.458	21.359	25.539
7	0.4462	16.366	15.769	18.530	15.108	18.827
7	0.4472	21.174	24.027	25.933	14.320	14.219
7	0.4482	12.921	10.739	16.319	11.196	7.492
7	0.4492	17.487	14.659	23.489	6.438	10.806
7	0.4502	14.985	18.949	22.329	24.087	19.636
7	0.4512	15.146	10.602	14.823	16.529	12.737
7	0.4522	15.717	17.570	20.419	10.597	14.820
7	0.4532	16.509	14.501	20.435	6.883	19.423
7	0.4542	9.206	5.654	6.505	9.282	12.912
7	0.4552	8.668	4.462	7.846	8.295	7.331
7	0.4562	2.668	0.910	1.379	3.880	4.132
7	0.4572	8.096	3.268	10.757	7.437	13.138
7	0.4582	4.072	2.109	9.184	4.988	7.806
7	0.4592	1.228	2.006	5.201	1.015	8.200
7	0.4602	8.351	1.715	8.681	3.030	5.331

7	0.4612	7.882	1.443	7.712	3.001	5.365
7	0.4622	9.104	9.954	9.860	8.354	3.878
7	0.4632	13.093	12.232	9.822	5.878	2.797
7	0.4642	8.481	4.663	10.800	2.424	3.821
7	0.4652	6.700	3.614	8.789	2.606	1.965
7	0.4662	2.004	1.923	0.603	1.963	1.476
7	0.4672	12.540	9.753	17.359	4.245	4.063
7	0.4682	10.594	7.961	13.746	5.214	1.111
7	0.4692	5.008	5.097	6.302	1.682	0.619
7	0.4702	7.787	6.649	13.064	4.441	1.816
7	0.4712	13.651	9.252	18.425	8.145	9.449
7	0.4722	24.040	21.040	25.946	12.019	11.410
7	0.4732	21.869	19.041	23.955	11.900	8.242
7	0.4742	20.594	22.748	26.600	21.586	4.964
7	0.4752	23.073	25.143	24.429	14.602	8.245
7	0.4762	1.867	5.039	4.402	3.493	4.804
7	0.4772	9.837	6.571	13.201	5.036	5.491
7	0.4782	11.921	5.143	9.859	8.033	7.656
7	0.4792	11.273	3.273	9.117	6.950	8.786
7	0.4802	7.020	1.619	6.278	6.665	8.249
7	0.4812	4.230	1.531	9.583	6.838	5.602
7	0.4822	13.685	13.361	13.422	2.239	9.512
7	0.4832	12.948	15.576	11.294	7.483	6.326
7	0.4842	12.338	8.004	6.023	1.140	11.973
7	0.4852	13.239	9.786	7.198	10.144	19.018
7	0.4862	7.478	4.124	4.010	7.491	14.731
7	0.4872	7.479	4.120	4.007	7.499	14.725
7	0.4882	9.814	6.284	4.869	7.408	24.780
7	0.4892	16.283	8.972	18.769	6.614	11.121
7	0.4902	16.864	23.930	22.371	19.377	8.601
7	0.4912	4.542	6.358	7.379	6.893	8.279
7	0.4922	5.992	3.005	6.032	7.984	7.335
7	0.4932	6.413	3.278	7.732	3.763	4.826
7	0.4942	12.459	3.642	11.606	10.083	6.882
7	0.4952	17.043	5.634	15.118	10.561	7.079
7	0.4962	29.259	30.147	32.650	17.044	11.369
7	0.4972	21.503	15.562	20.254	9.104	11.992
7	0.4982	11.006	15.673	15.671	7.930	8.876
7	0.4992	20.670	22.889	21.476	14.891	9.494
7	0.5002	20.775	22.975	21.520	14.843	9.575
7	0.5012	19.119	21.619	28.074	16.826	8.327
7	0.5022	2.806	3.700	4.026	7.443	7.648
7	0.5032	12.063	14.674	16.639	4.506	7.549
7	0.5042	24.504	17.432	22.744	23.453	13.183
7	0.5052	12.166	6.502	10.875	8.437	17.102
7	0.5062	13.986	4.067	13.841	20.552	10.651
7	0.5072	8.782	8.779	9.366	16.447	7.810
7	0.5082	13.722	10.938	13.356	15.684	11.893
7	0.5092	26.876	33.191	25.748	14.656	20.684
7	0.5102	22.418	19.989	24.046	8.176	19.837

7	0.5112	20.905	14.236	20.774	10.690	21.310
7	0.5122	13.910	12.341	13.049	11.760	14.849
7	0.5132	19.863	15.152	20.058	9.065	16.633
7	0.5142	29.020	31.713	27.489	17.483	14.241
7	0.5152	15.386	13.681	16.499	12.014	18.439
7	0.5162	17.272	17.280	16.493	13.221	11.827
7	0.5172	9.418	8.323	12.983	7.788	12.409
7	0.5182	14.320	10.331	17.961	13.120	14.694
7	0.5192	13.922	7.591	16.253	13.006	24.531
7	0.5202	7.598	6.573	10.232	9.581	22.723
7	0.5212	11.249	6.272	14.344	12.901	15.588
7	0.5222	11.234	9.041	15.224	17.115	7.570
7	0.5232	17.915	20.465	9.852	17.195	17.165
7	0.5242	20.758	16.578	13.009	25.844	23.398
7	0.5252	12.825	12.942	9.837	9.722	12.002
7	0.5262	9.808	12.948	7.225	8.993	29.317
7	0.5272	17.556	20.880	12.430	23.835	13.516
7	0.5282	31.095	25.094	21.201	17.016	17.327
7	0.5292	31.106	25.104	21.218	17.069	17.350
7	0.5302	15.355	18.513	18.053	11.531	9.808
7	0.5312	15.084	13.507	17.727	17.862	16.970
7	0.5322	26.660	19.176	25.122	9.163	10.812
7	0.5332	17.987	16.385	18.565	17.289	17.622
7	0.5342	19.189	12.105	14.546	10.847	24.288
7	0.5352	23.833	17.958	18.562	16.445	17.313
7	0.5362	16.214	17.653	18.746	9.866	7.706
7	0.5372	11.168	12.591	15.050	15.919	34.486
7	0.5382	10.916	14.169	13.615	12.257	12.982
7	0.5392	23.213	22.213	24.172	13.891	9.293
7	0.5402	13.497	17.803	13.962	24.894	13.560
7	0.5412	8.780	9.497	13.478	28.728	24.330
7	0.5422	11.405	12.762	14.399	15.382	19.529
7	0.5432	13.343	12.723	11.027	13.432	19.345
7	0.5442	8.540	12.284	11.650	14.432	10.236
7	0.5452	24.896	21.734	19.014	14.131	8.137
7	0.5462	32.058	26.699	22.668	8.320	17.869
7	0.5472	7.051	6.927	6.024	17.405	10.631
7	0.5482	17.190	7.926	7.156	19.031	16.178
7	0.5492	16.029	8.215	13.618	12.532	20.601
7	0.5502	20.955	18.761	13.092	11.470	7.142
7	0.5512	18.964	13.996	18.919	11.848	16.258
7	0.5522	20.099	13.761	11.419	9.087	11.418
7	0.5532	16.002	7.516	8.964	7.695	25.026
7	0.5542	54.015	45.602	40.294	12.728	21.619
7	0.5552	14.575	13.736	9.073	11.941	11.627
7	0.5562	9.709	12.083	10.815	10.195	13.829
7	0.5572	23.862	25.556	22.629	9.415	10.546
7	0.5582	24.762	16.438	20.245	19.435	13.663
7	0.5592	17.668	9.097	16.659	18.942	21.287
7	0.5602	9.482	6.751	10.181	12.035	13.423

7	0.5612	6.334	5.702	7.120	15.443	5.745
7	0.5622	17.388	11.409	14.854	15.775	12.126
7	0.5632	17.018	11.266	14.045	11.434	11.347
7	0.5642	16.165	16.955	12.727	10.812	9.772
7	0.5652	21.370	23.432	14.847	17.121	7.113
7	0.5662	7.228	8.161	3.226	13.074	14.213
7	0.5672	13.550	9.671	12.892	12.758	11.910
7	0.5682	13.534	9.670	12.902	12.726	11.986
7	0.5692	12.423	11.495	12.044	5.859	12.661
7	0.5702	14.132	12.066	14.095	6.465	9.306
7	0.5712	8.538	3.727	6.259	9.910	11.547
7	0.5722	23.013	14.787	12.200	7.124	6.474
7	0.5732	13.071	7.811	10.838	16.424	10.205
7	0.5742	14.934	13.188	17.154	9.158	15.031
7	0.5752	10.640	8.697	12.964	2.666	5.312
7	0.5762	10.653	12.569	15.994	7.390	1.290
7	0.5772	15.126	12.837	11.419	12.699	8.586
7	0.5782	18.385	17.490	14.674	11.742	10.595
7	0.5792	23.276	18.571	20.268	18.518	22.454
7	0.5802	7.848	11.352	8.414	13.371	13.770
7	0.5812	24.556	14.597	20.180	8.544	12.120
7	0.5822	11.312	6.135	11.539	8.306	11.122
7	0.5832	14.431	9.389	17.588	10.094	9.588
7	0.5842	19.235	18.235	25.319	14.254	18.431
7	0.5852	12.244	13.849	20.382	12.774	17.523
7	0.5862	19.425	19.853	16.817	6.401	8.820
7	0.5872	20.876	13.963	17.443	13.594	9.744
7	0.5882	8.688	9.114	7.377	8.504	4.699
7	0.5892	35.952	21.061	19.000	6.368	9.415
7	0.5902	27.738	13.510	20.522	2.166	8.160
7	0.5912	16.975	8.713	13.541	7.654	9.294
7	0.5922	9.259	6.227	8.313	7.097	7.812
7	0.5932	14.086	15.118	16.478	14.786	9.769
7	0.5942	10.909	8.900	13.625	12.513	20.471
7	0.5952	15.502	10.785	13.717	11.835	6.734
7	0.5962	18.107	16.591	17.907	11.006	14.371
7	0.5972	11.299	7.149	9.931	4.621	8.954
7	0.5982	18.837	20.322	20.886	10.584	7.958
7	0.5992	19.275	18.440	20.225	7.158	13.210
7	0.6002	24.244	18.802	26.898	11.987	11.817
7	0.6012	26.993	23.787	29.760	11.062	14.596
7	0.6022	24.158	27.260	25.209	13.692	14.106
7	0.6032	18.128	16.170	20.935	17.460	12.958
7	0.6042	13.652	5.484	13.895	11.589	12.487
7	0.6052	7.096	4.747	11.060	7.321	6.023
7	0.6062	6.729	8.149	8.886	9.746	5.413
7	0.6072	10.943	11.477	12.048	10.459	12.034
7	0.6082	28.633	17.256	19.405	15.445	10.816
7	0.6092	29.395	24.800	22.464	15.076	8.172
7	0.6102	18.650	19.028	21.491	8.751	4.998

7	0.6112	16.448	12.107	17.306	10.846	11.307
7	0.6122	16.273	12.761	16.313	5.629	5.131
7	0.6132	15.281	12.195	16.217	5.740	4.597
7	0.6142	24.231	23.112	28.175	16.555	7.660
7	0.6152	10.042	16.092	17.774	11.433	10.732
7	0.6162	13.654	14.123	22.913	11.490	17.562
7	0.6172	23.694	26.311	27.647	10.940	11.749
7	0.6182	6.930	11.080	10.518	12.669	11.181
7	0.6192	13.912	10.159	12.233	6.058	8.727
7	0.6202	8.285	5.723	10.403	6.999	9.170
7	0.6212	10.904	11.591	18.225	3.914	8.075
7	0.6222	16.305	16.988	15.065	6.057	5.300
7	0.6232	9.700	14.040	12.479	3.983	4.129
7	0.6242	15.540	24.426	19.626	9.482	3.964
7	0.6252	8.544	7.771	8.970	9.069	4.113
7	0.6262	6.661	2.142	2.185	7.776	9.031
7	0.6272	13.468	16.110	16.081	7.627	6.753
7	0.6282	13.208	11.640	15.032	6.486	12.970
7	0.6292	20.217	13.057	17.024	10.255	18.098
7	0.6302	9.105	4.769	8.857	8.840	13.012
7	0.6312	13.228	11.507	12.987	6.263	15.290
7	0.6322	9.746	4.562	11.629	15.779	22.242
7	0.6332	16.817	9.058	12.400	16.505	23.804
7	0.6342	11.612	8.171	9.053	8.510	16.767
7	0.6352	16.234	14.214	12.668	14.609	14.673
7	0.6362	11.736	6.063	9.675	6.138	17.678
7	0.6372	17.070	7.339	14.295	15.825	12.328
7	0.6382	14.587	6.010	10.604	16.979	11.923
7	0.6392	18.228	7.560	12.132	8.683	12.078
7	0.6402	18.344	15.372	15.029	4.587	15.208
7	0.6412	22.437	22.543	20.501	10.820	14.578
7	0.6422	20.192	17.607	16.132	12.256	12.917
7	0.6432	17.849	11.453	13.802	12.685	22.257
7	0.6442	17.497	11.307	13.447	12.679	22.318
7	0.6452	15.041	14.835	14.934	10.139	17.745
7	0.6462	16.802	18.410	16.227	9.640	26.224
7	0.6472	14.831	13.051	11.419	5.673	21.439
7	0.6482	14.305	12.919	9.965	10.681	19.609
7	0.6492	14.825	9.675	12.070	13.552	13.490
7	0.6502	22.377	13.731	15.372	9.739	17.681
7	0.6512	25.984	18.869	15.252	12.071	17.067
7	0.6522	4.554	5.119	6.972	17.703	20.338
7	0.6532	4.915	8.745	6.842	21.593	22.600
7	0.6542	13.842	12.041	14.209	6.162	19.827
7	0.6552	11.529	11.809	11.396	5.054	9.066
7	0.6562	14.534	14.379	14.480	3.902	13.078
7	0.6572	6.749	7.126	7.273	1.979	8.714
7	0.6582	15.091	14.356	14.967	4.469	18.117
7	0.6592	20.531	18.945	14.582	9.228	22.175
7	0.6602	18.428	18.832	14.928	6.870	23.567

7	0.6612	17.307	19.280	12.559	13.927	25.278
7	0.6622	29.059	34.671	23.476	14.252	18.881
7	0.6632	21.114	21.202	18.398	11.183	16.231
7	0.6642	35.948	23.405	26.706	11.286	13.213
7	0.6652	9.472	9.010	12.545	6.283	4.436
7	0.6662	12.850	13.628	10.681	11.954	8.905
7	0.6672	14.703	11.573	14.005	10.417	14.484
7	0.6682	24.326	19.906	21.530	11.495	12.889
7	0.6692	21.877	16.851	17.284	11.760	13.501
7	0.6702	24.657	19.542	20.701	13.360	18.425
7	0.6712	11.172	10.744	16.056	7.199	16.743
7	0.6722	9.737	12.107	8.137	8.040	9.528
7	0.6732	9.759	12.151	8.207	8.058	9.476
7	0.6742	22.571	21.594	13.006	15.641	21.764
7	0.6752	24.608	19.303	16.078	11.068	21.440
7	0.6762	21.733	21.300	15.002	14.512	21.322
7	0.6772	33.135	19.901	27.555	12.118	17.487
7	0.6782	33.131	19.730	27.504	12.159	17.427
7	0.6792	30.751	21.316	23.765	20.047	21.653
7	0.6802	22.345	13.814	10.267	11.368	21.125
7	0.6812	24.252	15.798	16.129	6.161	18.147
7	0.6822	25.598	16.478	18.253	7.650	13.391
7	0.6832	7.237	8.934	7.203	6.535	13.403
7	0.6842	14.746	10.156	10.816	20.711	14.560
7	0.6852	18.318	8.949	9.272	15.353	16.167
7	0.6862	12.835	9.580	10.829	17.426	15.092
7	0.6872	32.144	13.361	15.025	12.060	17.208
7	0.6882	16.873	12.485	14.244	10.838	13.793
7	0.6892	5.773	4.791	5.951	1.338	11.485
7	0.6902	12.752	9.106	7.099	10.586	9.201
7	0.6912	13.091	5.290	9.935	11.284	20.511
7	0.6922	16.039	9.819	13.759	16.906	20.760
7	0.6932	21.610	22.794	15.819	13.090	24.540
7	0.6942	20.148	9.426	14.855	9.439	18.941
7	0.6952	18.848	10.985	10.438	9.362	20.337
7	0.6962	18.793	14.618	10.055	13.250	17.614
7	0.6972	32.497	21.167	24.368	12.398	15.253
7	0.6982	14.332	13.855	11.291	5.105	15.206
7	0.6992	6.239	8.399	6.251	8.993	17.658
7	0.7002	20.264	15.129	12.070	13.316	21.134
7	0.7012	19.436	8.697	10.703	13.867	28.293
7	0.7022	7.555	10.945	9.730	10.717	16.235
7	0.7032	21.132	23.014	14.112	9.792	12.384
7	0.7042	28.653	13.452	21.996	13.898	14.010
7	0.7052	28.574	13.405	21.925	13.894	13.997
7	0.7062	25.264	15.148	23.765	19.744	16.864
7	0.7072	21.437	19.106	14.139	11.825	17.157
7	0.7082	14.790	14.551	9.688	8.434	21.657
7	0.7092	14.691	14.514	9.636	8.296	21.500
7	0.7102	23.400	21.695	16.654	7.318	15.466

7	0.7112	17.774	15.340	15.181	15.692	11.391
7	0.7122	19.258	16.034	16.655	6.152	13.791
7	0.7132	22.946	23.506	21.148	13.406	18.482
7	0.7142	16.959	10.550	10.315	8.942	10.904
7	0.7152	18.425	14.562	17.344	9.541	10.066
7	0.7162	25.683	15.940	20.271	9.517	8.106
7	0.7172	19.203	12.107	15.889	10.452	4.112
7	0.7182	20.877	14.954	18.803	20.117	7.470
7	0.7192	38.416	15.066	19.306	10.585	8.438
7	0.7202	17.390	12.213	14.548	13.203	13.930
7	0.7212	52.223	15.779	32.117	16.067	9.288
7	0.7222	24.329	15.002	18.189	26.420	22.476
7	0.7232	21.233	11.621	16.781	10.420	6.778
7	0.7242	15.764	14.925	12.149	17.374	7.877
7	0.7252	13.461	10.284	12.552	10.613	8.580
7	0.7262	6.266	6.873	5.164	3.953	0.949
7	0.7272	7.881	5.806	3.385	6.808	2.422
7	0.7282	17.136	15.673	13.517	12.510	13.030
7	0.7292	16.548	16.931	17.299	11.239	7.305
7	0.7302	16.744	17.354	15.786	14.889	7.140
7	0.7312	20.480	17.930	16.273	13.846	12.454
7	0.7322	17.024	17.544	14.528	11.536	9.470
7	0.7332	7.357	10.280	8.902	21.101	10.472
7	0.7342	17.678	13.844	19.345	7.951	4.485
7	0.7352	16.891	12.800	18.501	8.948	6.679
7	0.7362	8.326	7.569	9.245	12.065	9.765
7	0.7372	8.245	10.400	7.599	12.916	9.920
7	0.7382	8.753	9.449	12.293	7.389	7.968
7	0.7392	10.412	17.313	12.928	14.137	12.187
7	0.7402	8.005	9.563	8.578	11.352	5.190
7	0.7412	17.567	15.630	21.086	6.872	10.310
7	0.7422	7.120	8.296	8.603	8.697	8.828
7	0.7432	6.264	5.178	7.179	13.923	13.469
7	0.7442	13.622	15.446	13.323	13.853	8.341
7	0.7452	9.043	7.272	6.159	9.484	4.203
7	0.7462	10.610	5.899	6.618	16.043	7.953
7	0.7472	4.741	3.141	4.750	4.683	8.509
7	0.7482	12.377	13.463	16.653	9.609	14.456
7	0.7492	10.949	13.445	15.193	6.781	13.491
7	0.7502	12.077	12.381	17.693	5.942	12.059
7	0.7512	6.791	10.668	8.246	9.674	10.763
7	0.7522	13.985	12.880	12.526	10.971	14.346
7	0.7532	18.298	14.695	18.526	15.105	6.912
7	0.7542	10.600	6.843	7.898	9.270	9.870
7	0.7552	20.010	21.402	22.938	8.844	11.375
7	0.7562	15.966	14.707	22.177	9.759	14.941
7	0.7572	16.694	11.323	18.406	11.717	13.853
7	0.7582	9.170	15.843	8.736	22.114	9.320
7	0.7592	14.711	13.002	14.107	21.267	10.799
7	0.7602	13.969	12.070	14.198	12.508	10.677

7	0.7612	15.348	8.932	12.340	8.879	13.514
7	0.7622	18.131	17.310	16.425	16.468	11.720
7	0.7632	9.228	9.189	8.442	19.556	10.480
7	0.7642	13.470	11.583	13.675	11.580	12.360
7	0.7652	14.162	9.184	18.924	10.550	7.994
7	0.7662	14.353	9.063	14.462	5.658	10.240
7	0.7672	15.127	12.152	12.486	6.447	8.844
7	0.7682	21.344	19.825	19.019	6.703	13.528
7	0.7692	21.183	22.191	22.585	6.759	6.791
7	0.7702	10.794	14.690	17.316	7.081	6.355
7	0.7712	16.607	14.751	16.762	16.958	10.293
7	0.7722	22.858	17.675	21.274	15.636	12.296
7	0.7732	12.003	11.898	14.981	13.536	8.360
7	0.7742	13.207	18.490	14.240	22.090	5.070
7	0.7752	11.472	14.360	11.123	14.028	10.271
7	0.7762	7.489	6.636	9.527	7.902	7.678
7	0.7772	8.549	7.441	8.357	15.368	7.392
7	0.7782	12.574	18.753	16.560	20.904	12.323
7	0.7792	15.399	21.322	19.623	19.517	14.258
7	0.7802	8.755	12.581	12.198	14.129	9.881
7	0.7812	9.008	9.462	7.533	7.436	12.123
7	0.7822	17.500	18.210	19.295	8.572	9.012
7	0.7832	11.895	16.116	11.205	12.570	7.468
7	0.7842	19.472	23.960	18.642	17.674	16.227
7	0.7852	11.139	16.961	18.308	13.633	14.305
7	0.7862	17.840	14.336	22.980	13.531	14.022
7	0.7872	10.620	11.869	17.053	12.241	15.413
7	0.7882	8.396	13.401	13.922	9.002	13.101
7	0.7892	16.014	16.535	21.179	8.163	20.523
7	0.7902	16.358	17.444	21.120	12.041	11.232
7	0.7912	15.813	22.878	20.515	17.820	9.075
7	0.7922	11.323	20.350	12.381	18.978	9.814
7	0.7932	22.069	25.432	18.356	16.645	14.466
7	0.7942	8.759	10.613	10.481	6.509	8.196
7	0.7952	11.574	8.664	10.939	6.899	14.176
7	0.7962	17.630	18.658	17.541	13.721	15.304
7	0.7972	9.498	13.323	8.529	9.668	13.398
7	0.7982	11.294	14.515	16.235	12.402	5.949
7	0.7992	18.229	17.073	21.451	19.431	12.211
7	0.8002	15.251	19.829	16.938	22.093	14.165
7	0.8012	17.431	19.657	21.894	18.211	13.303
7	0.8022	9.721	13.273	10.085	10.428	6.891
7	0.8032	13.428	11.857	12.456	12.843	10.174
7	0.8042	14.557	13.679	14.098	1.192	0.418
7	0.8052	19.638	20.582	19.059	7.466	8.659
7	0.8062	9.324	18.193	14.645	22.271	8.819
7	0.8072	10.866	12.796	10.809	9.619	6.091
7	0.8082	9.820	17.543	15.353	11.802	10.144
7	0.8092	13.251	16.287	13.467	10.693	3.975
7	0.8102	9.971	13.918	8.559	13.109	7.190

7	0.8112	2.029	3.517	2.252	4.588	5.261
7	0.8122	9.869	13.381	13.563	8.206	4.697
7	0.8132	18.681	16.166	12.964	12.592	7.745
7	0.8142	7.275	11.188	7.369	15.081	15.371
7	0.8152	12.762	15.169	10.544	8.456	11.947
7	0.8162	13.070	12.368	14.112	7.124	5.793
7	0.8172	12.353	16.275	14.945	13.451	10.547
7	0.8182	17.602	15.621	24.115	8.845	5.291
7	0.8192	17.755	18.613	21.589	26.431	9.784
7	0.8202	5.275	10.927	8.028	7.339	3.482
7	0.8212	7.813	10.330	7.587	10.078	6.540
7	0.8222	15.563	18.001	16.102	7.051	5.307
7	0.8232	16.667	19.583	17.596	12.214	6.739
7	0.8242	17.268	23.188	24.293	15.254	9.583
7	0.8252	15.022	17.381	15.967	12.823	7.069
7	0.8262	14.533	20.691	17.539	11.122	8.143
7	0.8272	17.541	18.368	17.377	10.413	9.242
7	0.8282	24.679	16.951	21.662	18.856	7.500
7	0.8292	24.678	16.949	21.662	18.861	7.498
7	0.8302	19.288	21.923	17.607	15.541	13.106
7	0.8312	16.790	15.858	12.980	13.673	9.824
7	0.8322	17.251	17.018	18.196	12.187	9.203
7	0.8332	7.993	5.512	5.335	5.507	1.630
7	0.8342	6.882	9.981	6.736	5.793	3.176
7	0.8352	6.872	9.928	6.703	5.793	3.179
7	0.8362	14.979	13.948	12.028	11.570	6.604
7	0.8372	2.840	6.506	5.303	7.461	3.605
7	0.8382	11.412	16.108	15.043	13.016	7.224
7	0.8392	14.272	13.738	13.921	9.744	8.582
7	0.8402	17.227	21.579	19.127	12.856	11.981
7	0.8412	21.823	29.027	32.951	13.406	10.920
7	0.8422	12.609	12.762	18.211	7.061	7.176
7	0.8432	7.485	7.729	7.545	3.604	9.656
7	0.8442	6.293	8.668	5.120	3.956	18.691
7	0.8452	14.108	22.216	16.948	11.329	15.320
7	0.8462	14.042	22.151	16.947	11.334	15.293
7	0.8472	15.759	19.211	15.713	7.877	17.699
7	0.8482	10.703	21.606	16.042	12.304	11.052
7	0.8492	10.742	17.002	18.820	13.352	14.237
7	0.8502	11.172	18.688	20.221	18.764	17.587
7	0.8512	9.953	11.623	12.271	21.360	21.275
7	0.8522	9.942	11.700	12.332	21.492	21.439
7	0.8532	9.935	11.780	12.400	21.627	21.605
7	0.8542	9.932	11.863	12.475	21.764	21.769
7	0.8552	18.882	22.222	17.505	20.655	20.009
7	0.8562	17.518	19.044	18.403	17.753	20.426
7	0.8572	27.364	28.587	35.870	12.248	11.500
7	0.8582	22.617	19.439	24.918	5.906	7.259
7	0.8592	9.458	8.546	7.276	4.757	3.318
7	0.8602	11.616	10.544	14.461	5.351	8.089

7	0.8612	12.019	16.511	18.165	20.074	9.777
7	0.8622	11.935	16.554	18.162	20.068	9.761
7	0.8632	17.322	14.635	17.650	7.466	14.331
7	0.8642	16.002	15.634	14.059	9.934	12.168
7	0.8652	13.970	15.183	16.091	8.573	8.719
7	0.8662	17.440	16.153	20.829	13.436	8.726
7	0.8672	18.781	17.132	21.198	13.453	6.857
7	0.8682	21.944	23.994	26.668	8.941	10.730
7	0.8692	23.296	25.185	26.823	9.361	12.367
7	0.8702	22.299	25.433	28.023	16.209	6.670
7	0.8712	16.575	22.584	24.059	9.178	4.244
7	0.8722	17.014	19.744	24.366	10.817	6.011
7	0.8732	17.249	23.350	23.905	9.607	9.273
7	0.8742	12.119	13.538	19.128	16.259	15.687
7	0.8752	6.897	6.463	11.177	5.806	8.121
7	0.8762	11.770	21.526	14.440	19.063	8.994
7	0.8772	19.832	13.558	20.215	12.653	10.623
7	0.8782	29.399	19.908	30.061	12.380	17.806
7	0.8792	20.882	18.146	24.702	8.475	9.682
7	0.8802	17.761	16.086	15.312	21.658	16.459
7	0.8812	8.727	6.874	5.804	16.229	14.694
7	0.8822	11.344	10.466	8.785	14.104	11.944
7	0.8832	19.780	23.249	17.868	17.252	12.080
7	0.8842	20.978	22.368	20.146	19.740	15.768
7	0.8852	20.660	22.148	19.354	19.493	15.641
7	0.8862	14.883	16.061	12.638	7.304	5.525
7	0.8872	15.088	22.110	19.803	8.877	14.164
7	0.8882	14.971	22.235	19.641	9.039	14.071
7	0.8892	18.554	25.656	21.451	18.821	15.207
7	0.8902	4.859	6.741	7.092	5.956	2.668
7	0.8912	13.916	13.717	10.593	11.401	8.777
7	0.8922	20.314	14.461	15.354	7.604	13.612
7	0.8932	11.888	12.269	12.420	10.150	25.621
7	0.8942	6.000	6.986	6.102	10.701	26.760
7	0.8952	8.944	10.734	10.237	5.461	10.056
7	0.8962	16.789	20.232	22.949	16.154	18.991
7	0.8972	16.260	24.041	23.025	9.356	8.678
7	0.8982	6.674	6.505	8.234	7.355	5.719
7	0.8992	5.352	5.867	5.532	14.072	8.767
7	0.9002	17.568	14.646	16.415	11.075	7.715
7	0.9012	18.417	11.228	14.261	12.153	5.316
7	0.9022	16.046	15.841	16.673	14.792	15.973
7	0.9032	11.932	16.412	17.543	17.392	13.255
7	0.9042	20.575	17.841	18.607	8.657	11.492
7	0.9052	34.561	26.221	38.703	10.191	12.319
7	0.9062	23.497	18.585	23.960	11.708	12.302
7	0.9072	12.624	14.281	11.763	13.560	12.358
7	0.9082	12.807	9.450	10.026	9.545	10.590
7	0.9092	13.743	9.343	10.886	14.163	11.997
7	0.9102	24.187	14.274	20.019	15.840	12.798

7	0.9112	16.305	15.300	20.173	9.725	12.029
7	0.9122	21.066	14.783	21.611	19.552	11.583
7	0.9132	16.752	23.592	20.228	12.774	17.710
7	0.9142	10.818	14.500	13.960	8.505	11.463
7	0.9152	14.191	15.936	18.035	12.550	14.197
7	0.9162	17.879	11.619	13.315	9.027	9.223
7	0.9172	8.257	12.317	11.871	7.620	10.702
7	0.9182	13.781	9.378	8.353	6.556	10.844
7	0.9192	13.949	9.547	8.482	6.568	10.770
7	0.9202	22.374	18.927	16.555	22.964	16.001
7	0.9212	11.134	10.388	8.842	14.164	15.173
7	0.9222	11.089	7.286	12.384	11.573	12.407
7	0.9232	14.597	11.276	12.584	20.209	28.037
7	0.9242	5.938	6.676	7.720	18.621	21.920
7	0.9252	13.289	8.211	10.647	21.544	27.817
7	0.9262	13.627	5.979	15.814	20.013	9.411
7	0.9272	14.041	12.206	15.872	12.741	7.640
7	0.9282	9.863	10.025	10.737	12.824	11.825
7	0.9292	10.591	5.924	7.981	7.552	7.733
7	0.9302	9.196	5.526	9.769	8.287	10.007
7	0.9312	5.823	6.319	9.117	4.382	12.291
7	0.9322	27.391	20.787	22.645	12.829	14.086
7	0.9332	28.476	19.292	23.522	13.141	15.939
7	0.9342	18.073	15.119	17.821	11.916	10.184
7	0.9352	24.393	17.081	20.109	9.633	13.305
7	0.9362	11.645	11.647	12.734	7.969	9.768
7	0.9372	15.031	13.090	16.029	6.742	6.669
7	0.9382	17.212	7.908	11.626	9.664	16.239
7	0.9392	15.616	8.395	9.773	6.626	5.237
7	0.9402	16.415	10.446	16.656	5.728	9.408
7	0.9412	10.276	7.838	11.381	2.789	3.117
7	0.9422	12.566	15.932	12.528	14.541	9.269
7	0.9432	17.344	21.856	22.074	10.920	9.532
7	0.9442	19.764	23.457	23.093	8.804	7.735
7	0.9452	33.646	29.980	33.294	13.952	12.007
7	0.9462	25.601	28.959	28.100	19.900	8.187
7	0.9472	21.466	24.039	25.502	9.886	15.948
7	0.9482	28.858	16.265	17.014	6.605	12.272
7	0.9492	9.520	11.329	14.960	8.617	8.952
7	0.9502	16.090	11.565	17.336	9.807	12.452
7	0.9512	5.548	7.373	13.208	6.648	10.907
7	0.9522	20.719	19.614	15.145	10.519	9.229
7	0.9532	16.066	14.989	12.643	5.780	5.956
7	0.9542	8.420	7.826	11.267	8.013	21.108
7	0.9552	17.850	14.552	15.730	15.639	12.573
7	0.9562	23.653	14.473	17.909	11.846	12.482
7	0.9572	16.930	16.251	16.502	7.628	8.286
7	0.9582	5.963	9.849	10.408	7.551	6.697
7	0.9592	10.833	11.509	10.552	15.432	10.140
7	0.9602	18.389	19.890	18.081	13.332	13.086

7	0.9612	22.736	18.694	18.655	10.096	7.459
7	0.9622	19.576	15.588	15.799	15.530	10.154
7	0.9632	16.969	22.365	17.161	17.350	12.610
7	0.9642	21.110	24.445	22.066	16.237	13.199
7	0.9652	19.707	20.524	20.457	11.743	10.032
7	0.9662	14.626	12.977	12.653	8.292	12.059
7	0.9672	15.566	13.271	15.411	15.971	11.860
7	0.9682	18.168	20.966	20.835	16.393	13.902
7	0.9692	24.238	19.392	18.878	14.124	6.239
7	0.9702	13.241	10.658	10.406	11.021	6.824
7	0.9712	16.362	9.949	15.913	5.789	7.277
7	0.9722	15.286	14.245	12.754	9.673	8.171
7	0.9732	15.309	14.274	12.821	9.634	8.191
7	0.9742	18.163	14.623	19.940	7.858	14.714
7	0.9752	12.824	13.048	14.426	9.375	17.642
7	0.9762	11.248	12.321	16.675	8.332	14.156
7	0.9772	23.251	21.271	21.714	14.123	10.868
7	0.9782	19.475	19.572	19.536	8.268	11.047
7	0.9792	10.323	11.007	12.949	10.709	10.978
7	0.9802	11.254	15.283	15.263	7.297	12.750
7	0.9812	11.127	11.256	14.230	4.807	6.414
7	0.9822	7.994	8.658	10.809	5.138	11.700
7	0.9832	12.612	12.458	14.859	14.212	17.367
7	0.9842	18.450	18.686	17.322	25.622	16.166
7	0.9852	16.627	15.278	16.382	13.404	10.059
7	0.9862	28.190	20.565	24.502	16.914	23.523
7	0.9872	22.081	20.517	23.489	16.259	19.579
7	0.9882	13.234	14.498	12.188	14.116	15.956
7	0.9892	24.545	20.215	23.404	13.000	7.781
7	0.9902	20.032	19.247	19.596	16.003	16.800
7	0.9912	18.919	23.586	23.849	12.775	17.973
7	0.9922	8.253	8.998	7.986	13.124	18.700
7	0.9932	12.902	13.785	9.387	14.336	8.836
7	0.9942	15.571	9.442	8.460	10.001	8.204
7	0.9952	13.900	13.046	10.516	15.253	6.746
7	0.9962	12.312	12.931	17.242	14.318	11.279
7	0.9972	16.642	20.501	23.460	20.152	12.076
7	0.9982	13.853	16.115	22.168	7.977	6.856
7	0.9992	27.103	30.736	26.524	16.575	5.513
7	1.0002	16.123	14.552	9.218	10.124	11.927
7	1.0012	18.316	24.055	18.771	19.784	16.614
7	1.0022	20.431	12.010	14.275	12.752	10.669
7	1.0032	25.196	15.693	16.642	25.556	22.950
7	1.0042	17.820	18.484	17.219	15.278	24.169
7	1.0052	23.426	26.621	20.812	16.842	20.095
7	1.0062	19.052	18.929	16.180	13.710	8.913
7	1.0072	20.423	28.441	24.053	12.949	13.003
7	1.0082	16.241	23.282	18.919	13.448	10.976
7	1.0092	21.515	22.889	18.690	12.128	19.556
7	1.0102	19.678	20.889	16.521	7.308	14.055

7	1.0112	10.832	13.688	10.671	14.188	15.841
7	1.0122	30.358	20.845	23.296	9.082	21.665
7	1.0132	7.261	9.519	15.280	5.620	2.006
7	1.0142	15.201	18.984	16.865	14.161	14.307
7	1.0152	20.202	27.345	20.206	15.931	16.160
7	1.0162	27.188	30.869	25.177	15.324	13.989
7	1.0172	15.118	20.761	14.140	19.781	15.917
7	1.0182	31.455	28.616	24.390	16.540	22.217
7	1.0192	29.888	21.057	20.362	10.079	16.417
7	1.0202	22.846	27.697	23.337	14.908	14.318
7	1.0212	22.528	24.866	19.651	17.812	18.123
7	1.0222	18.777	15.113	15.519	7.915	14.069
7	1.0232	29.025	30.815	25.604	21.343	19.761
7	1.0242	16.575	16.893	14.313	15.427	17.682
7	1.0252	10.764	16.297	12.702	24.933	6.784
7	1.0262	13.304	15.296	13.602	22.061	15.681
7	1.0272	17.266	20.953	13.190	22.449	8.400
7	1.0282	18.617	19.865	16.597	18.656	11.582
7	1.0292	33.361	29.234	31.065	16.265	13.117
7	1.0302	23.175	17.675	21.707	8.124	10.724
7	1.0312	13.034	15.740	15.375	6.505	11.260
7	1.0322	15.465	21.059	19.510	10.957	13.327
7	1.0332	15.726	24.070	22.721	21.367	9.617
7	1.0342	15.405	19.276	14.932	15.368	11.477
7	1.0352	15.790	20.642	16.275	15.536	18.921
7	1.0362	12.443	16.976	15.459	13.421	12.951
7	1.0372	10.046	13.199	11.690	11.456	9.575
7	1.0382	22.264	28.394	21.641	12.299	15.928
7	1.0392	13.339	14.972	9.665	12.174	8.891
7	1.0402	18.085	19.652	13.834	6.894	12.974
7	1.0412	13.041	16.419	17.043	5.630	11.991
7	1.0422	18.009	18.988	19.794	8.425	24.553
7	1.0432	12.630	18.957	13.009	10.107	12.614
7	1.0442	15.286	23.980	15.918	10.117	14.880
7	1.0452	16.007	23.680	15.323	22.543	15.521
7	1.0462	19.031	19.873	15.079	9.044	18.926
7	1.0472	16.941	15.355	16.887	6.263	4.489
7	1.0482	10.995	7.480	9.961	15.377	11.902
7	1.0492	25.004	16.374	21.214	14.295	9.192
7	1.0502	11.823	21.573	16.478	17.781	8.789
7	1.0512	12.246	17.353	14.302	10.697	7.656
7	1.0522	17.052	22.565	15.065	21.632	17.678
7	1.0532	14.229	16.895	11.382	20.744	12.668
7	1.0542	15.461	14.053	14.409	21.175	27.708
7	1.0552	20.121	18.237	22.736	10.172	19.011
7	1.0562	18.564	17.741	21.137	20.584	26.270
7	1.0572	18.794	18.347	23.193	9.611	6.588
7	1.0582	14.123	12.705	11.948	17.388	18.644
7	1.0592	15.802	11.511	12.050	16.163	11.577
7	1.0602	10.809	8.004	8.297	13.931	20.940

7	1.0612	11.608	8.977	8.983	10.189	15.145
7	1.0622	10.987	11.068	8.353	7.403	8.489
7	1.0632	4.521	4.771	7.579	6.329	6.829
7	1.0642	15.255	9.668	10.285	7.558	15.560
7	1.0652	0.000	0.000	0.000	0.000	0.000
8	0.0004	0.000	0.000	0.000	0.000	0.000
8	0.0014	10.299	10.058	13.511	7.035	9.281
8	0.0024	10.300	10.057	13.508	7.036	9.274
8	0.0034	13.205	12.160	9.940	18.566	11.607
8	0.0044	16.520	22.625	21.159	18.136	14.801
8	0.0054	31.971	36.657	40.957	14.891	9.510
8	0.0064	23.147	19.729	23.840	6.288	11.098
8	0.0074	23.449	23.542	24.625	17.110	15.511
8	0.0084	13.455	10.988	10.196	11.217	7.136
8	0.0094	17.682	14.590	22.959	16.505	8.418
8	0.0104	9.968	8.981	15.276	10.747	8.157
8	0.0114	15.461	20.532	25.317	8.431	7.371
8	0.0124	9.397	13.909	12.539	21.893	11.535
8	0.0134	4.657	11.348	8.432	20.188	12.749
8	0.0144	12.000	13.176	16.867	11.106	10.887
8	0.0154	14.762	8.734	16.817	16.865	13.362
8	0.0164	23.302	28.402	35.624	15.988	10.439
8	0.0174	27.373	21.939	35.421	11.438	20.919
8	0.0184	19.912	18.826	23.261	9.946	21.698
8	0.0194	21.111	20.351	24.809	9.019	8.779
8	0.0204	21.040	27.384	31.222	8.678	6.755
8	0.0214	16.975	26.149	28.098	12.239	9.057
8	0.0224	18.831	30.165	31.366	13.207	15.247
8	0.0234	12.166	12.554	9.821	9.619	14.031
8	0.0244	10.785	6.923	11.793	7.119	6.549
8	0.0254	14.666	11.899	10.624	14.902	8.757
8	0.0264	12.591	12.901	19.864	13.725	7.928
8	0.0274	19.535	28.119	31.573	16.981	14.361
8	0.0284	19.498	28.239	31.621	17.164	14.369
8	0.0294	21.756	23.377	25.147	25.347	15.897
8	0.0304	16.082	19.774	15.980	21.049	12.518
8	0.0314	5.907	11.292	11.826	7.158	8.358
8	0.0324	16.504	25.994	20.253	14.977	5.229
8	0.0334	11.509	18.378	13.021	14.400	7.670
8	0.0344	25.654	31.850	26.278	3.772	8.352
8	0.0354	20.488	31.347	26.389	18.259	20.339
8	0.0364	21.251	36.528	25.115	10.244	21.140
8	0.0374	17.140	20.082	23.679	9.418	7.160
8	0.0384	12.825	13.565	10.218	9.689	9.368
8	0.0394	15.312	9.526	9.742	10.364	8.722
8	0.0404	17.301	15.276	14.734	10.396	9.784
8	0.0414	16.630	16.241	13.869	16.455	10.800
8	0.0424	20.437	23.421	24.721	12.176	9.287
8	0.0434	17.825	17.193	24.037	12.486	10.376
8	0.0444	14.149	22.162	25.722	12.033	8.249

8	0.0454	7.916	15.473	15.461	12.300	5.552
8	0.0464	19.200	21.981	26.999	8.203	7.444
8	0.0474	26.753	37.476	32.033	12.851	3.354
8	0.0484	11.854	19.259	19.409	13.890	17.640
8	0.0494	18.069	24.385	24.247	15.140	12.979
8	0.0504	6.517	6.603	9.017	9.924	6.356
8	0.0514	11.514	15.343	20.038	10.169	6.014
8	0.0524	21.264	29.153	30.556	20.225	4.853
8	0.0534	16.414	21.321	20.578	13.222	7.842
8	0.0544	18.432	27.750	27.372	10.276	3.329
8	0.0554	8.766	10.990	6.816	19.217	5.228
8	0.0564	13.590	25.082	13.670	26.758	12.597
8	0.0574	23.852	26.615	18.588	27.137	14.600
8	0.0584	28.431	23.297	24.145	6.971	8.451
8	0.0594	20.715	23.757	30.582	7.696	14.641
8	0.0604	26.483	31.917	39.492	8.437	11.198
8	0.0614	7.644	11.674	11.716	5.427	4.015
8	0.0624	29.162	27.756	24.557	10.999	7.881
8	0.0634	16.897	18.364	21.029	6.105	13.893
8	0.0644	9.743	14.465	10.744	24.392	19.290
8	0.0654	11.041	16.949	19.275	7.160	5.647
8	0.0664	18.670	23.436	23.467	7.108	12.318
8	0.0674	38.572	39.965	45.773	17.990	16.673
8	0.0684	18.621	19.921	19.147	9.759	14.199
8	0.0694	15.563	14.348	12.036	5.722	12.284
8	0.0704	10.867	13.984	15.554	8.965	9.956
8	0.0714	12.132	19.038	17.011	15.665	10.227
8	0.0724	16.655	26.811	22.462	14.125	8.055
8	0.0734	28.719	32.298	30.975	19.307	16.784
8	0.0744	16.305	17.008	15.758	8.202	9.329
8	0.0754	27.354	19.268	25.433	15.069	20.153
8	0.0764	11.479	30.590	19.569	14.465	8.882
8	0.0774	9.767	23.200	16.535	6.101	13.372
8	0.0784	15.225	23.796	17.090	17.173	13.065
8	0.0794	33.090	38.550	37.764	11.743	14.076
8	0.0804	21.854	21.509	18.872	9.958	10.547
8	0.0814	9.099	13.364	11.855	15.101	20.227
8	0.0824	17.426	17.610	20.855	10.469	17.163
8	0.0834	25.056	28.541	27.875	13.011	13.145
8	0.0844	23.551	25.949	29.150	11.053	13.229
8	0.0854	20.862	27.281	25.418	10.157	12.991
8	0.0864	14.261	25.551	22.129	21.590	22.740
8	0.0874	9.056	11.082	19.361	6.165	15.262
8	0.0884	27.247	32.930	33.592	6.379	12.557
8	0.0894	17.816	24.163	24.319	9.481	10.034
8	0.0904	16.654	16.712	23.885	17.028	13.093
8	0.0914	25.868	22.474	32.400	16.696	17.592
8	0.0924	9.532	10.591	10.878	8.455	17.488
8	0.0934	15.006	15.895	14.289	14.369	13.973
8	0.0944	8.998	13.838	12.354	7.649	8.660

8	0.0954	7.988	8.890	8.843	2.874	8.018
8	0.0964	14.102	13.508	12.293	5.537	12.386
8	0.0974	15.181	16.610	13.542	5.722	16.754
8	0.0984	18.547	24.753	17.312	9.389	12.967
8	0.0994	26.276	25.071	19.729	7.411	7.666
8	0.1004	14.715	17.583	18.534	9.454	14.332
8	0.1014	31.651	39.230	32.934	11.836	15.978
8	0.1024	15.459	20.150	18.986	13.212	17.621
8	0.1034	16.017	15.814	17.896	10.273	6.486
8	0.1044	8.245	12.906	12.441	9.130	6.941
8	0.1054	16.832	28.566	23.824	14.859	11.402
8	0.1064	23.002	36.103	28.309	20.009	7.497
8	0.1074	28.607	26.598	26.945	7.041	6.977
8	0.1084	22.985	22.092	22.925	20.797	9.445
8	0.1094	20.896	21.746	20.630	15.801	7.696
8	0.1104	18.659	23.951	22.027	18.481	11.951
8	0.1114	19.499	20.872	26.860	19.673	18.781
8	0.1124	15.280	20.339	17.841	23.027	13.263
8	0.1134	19.267	22.564	22.288	13.913	9.338
8	0.1144	11.286	11.823	14.010	10.917	18.657
8	0.1154	12.144	12.665	17.012	14.750	21.047
8	0.1164	7.756	7.665	11.711	12.726	19.883
8	0.1174	12.387	7.539	15.188	11.188	13.146
8	0.1184	14.393	7.443	13.624	8.972	11.013
8	0.1194	10.475	9.665	10.829	7.887	13.928
8	0.1204	23.597	12.717	18.720	17.159	11.413
8	0.1214	21.399	11.363	16.494	15.415	9.961
8	0.1224	8.773	9.698	9.892	14.290	6.606
8	0.1234	8.796	9.617	9.952	14.409	6.768
8	0.1244	12.478	7.154	14.426	10.784	6.099
8	0.1254	12.442	7.088	14.485	10.750	5.997
8	0.1264	18.504	10.953	23.587	6.285	8.249
8	0.1274	15.957	13.825	15.863	7.283	7.072
8	0.1284	10.812	7.733	12.371	7.028	4.366
8	0.1294	9.992	8.363	12.919	10.149	3.632
8	0.1304	3.711	4.678	7.498	14.138	10.198
8	0.1314	14.954	18.379	23.886	14.891	13.728
8	0.1324	11.286	16.366	15.323	15.725	13.952
8	0.1334	11.726	10.081	13.799	12.565	9.815
8	0.1344	18.509	11.885	20.165	15.502	11.407
8	0.1354	14.685	18.704	13.141	9.658	8.579
8	0.1364	10.814	15.744	12.350	12.529	7.858
8	0.1374	12.492	11.928	15.078	23.099	26.484
8	0.1384	10.192	11.874	9.303	23.023	14.773
8	0.1394	13.242	13.448	14.479	21.751	21.880
8	0.1404	16.875	16.661	17.606	27.938	15.250
8	0.1414	11.035	9.693	13.717	11.393	11.013
8	0.1424	19.728	21.625	23.059	20.055	10.941
8	0.1434	16.249	9.393	13.397	20.646	15.362
8	0.1444	11.955	13.212	9.307	14.620	21.764

8	0.1454	21.124	29.157	22.682	22.771	26.070
8	0.1464	8.828	12.505	12.151	11.257	5.699
8	0.1474	10.705	6.699	6.035	35.934	20.531
8	0.1484	12.434	12.593	14.210	6.439	12.902
8	0.1494	19.116	21.331	28.120	14.797	10.111
8	0.1504	14.678	12.465	13.806	15.369	12.312
8	0.1514	14.561	13.234	18.967	18.873	26.835
8	0.1524	15.279	17.829	16.488	25.098	25.534
8	0.1534	10.583	8.585	8.132	22.182	20.261
8	0.1544	12.081	15.062	14.590	29.460	32.825
8	0.1554	12.519	15.159	11.475	15.552	5.830
8	0.1564	17.658	16.075	15.785	25.694	14.081
8	0.1574	18.448	14.479	15.992	24.961	12.658
8	0.1584	16.732	12.305	9.317	30.248	32.396
8	0.1594	16.041	14.480	14.843	12.829	25.434
8	0.1604	12.415	6.319	10.689	10.898	7.726
8	0.1614	11.259	11.707	12.576	23.182	18.079
8	0.1624	11.604	11.002	10.695	16.764	11.398
8	0.1634	9.981	22.090	15.822	19.869	13.949
8	0.1644	8.979	11.869	9.430	14.579	15.406
8	0.1654	16.473	14.266	12.332	12.844	17.020
8	0.1664	11.677	14.930	12.004	4.627	17.308
8	0.1674	4.880	6.080	5.318	9.236	10.690
8	0.1684	12.204	11.318	10.740	9.109	13.240
8	0.1694	11.659	12.827	12.420	13.943	23.906
8	0.1704	14.468	23.711	22.195	11.469	22.289
8	0.1714	21.597	23.533	27.300	15.439	20.069
8	0.1724	8.650	13.899	17.406	17.148	11.849
8	0.1734	15.797	4.919	17.066	6.389	9.218
8	0.1744	8.874	9.383	10.001	5.638	8.357
8	0.1754	30.604	22.086	22.363	15.688	17.682
8	0.1764	10.654	6.256	8.528	10.623	17.500
8	0.1774	12.534	11.427	11.625	8.137	15.207
8	0.1784	15.732	13.300	15.922	12.055	19.257
8	0.1794	11.901	5.119	7.049	12.747	14.770
8	0.1804	10.617	4.806	7.602	11.713	11.300
8	0.1814	10.041	14.320	10.255	12.466	15.496
8	0.1824	9.100	4.597	6.432	16.387	10.358
8	0.1834	13.422	12.638	19.327	21.841	17.457
8	0.1844	5.751	11.974	15.236	27.059	13.200
8	0.1854	17.046	11.624	23.848	20.463	13.650
8	0.1864	22.501	26.829	27.523	8.690	13.249
8	0.1874	20.174	25.248	23.895	13.786	20.812
8	0.1884	14.000	13.763	15.112	13.650	10.804
8	0.1894	18.252	16.059	22.966	14.928	9.753
8	0.1904	9.368	9.760	12.815	19.387	18.944
8	0.1914	16.648	11.755	12.169	8.191	10.784
8	0.1924	17.002	15.963	18.628	18.736	20.105
8	0.1934	14.870	8.856	9.962	24.781	20.089
8	0.1944	22.228	9.702	15.724	23.477	20.246

8	0.1954	13.248	10.640	13.361	15.600	15.832
8	0.1964	14.799	11.347	18.306	14.381	28.360
8	0.1974	27.376	13.598	24.702	10.473	8.417
8	0.1984	10.138	5.965	9.795	15.152	15.922
8	0.1994	10.380	13.937	14.350	17.748	20.313
8	0.2004	8.134	11.359	11.009	15.796	19.621
8	0.2014	31.170	20.764	21.298	7.964	23.364
8	0.2024	10.612	4.215	12.033	5.890	9.585
8	0.2034	9.771	8.573	13.622	7.867	15.244
8	0.2044	25.460	18.077	21.664	19.406	17.479
8	0.2054	12.318	14.666	13.155	12.506	10.791
8	0.2064	15.590	15.028	15.193	13.367	15.770
8	0.2074	12.210	10.427	14.936	10.663	17.209
8	0.2084	6.309	7.735	9.694	12.506	14.421
8	0.2094	12.327	9.035	12.937	10.657	5.486
8	0.2104	10.619	11.797	8.602	18.810	14.517
8	0.2114	14.453	14.608	13.118	16.150	10.627
8	0.2124	14.356	14.503	13.037	16.245	10.864
8	0.2134	14.261	14.390	12.945	16.341	11.101
8	0.2144	17.619	21.700	20.193	14.469	13.679
8	0.2154	5.052	6.459	9.613	9.442	12.235
8	0.2164	11.729	15.373	18.289	10.911	16.926
8	0.2174	12.414	15.770	16.003	14.313	5.101
8	0.2184	19.763	27.262	26.019	16.508	12.405
8	0.2194	16.138	18.502	14.154	24.650	17.508
8	0.2204	11.407	13.331	13.954	16.195	8.642
8	0.2214	16.844	12.071	19.091	20.386	16.108
8	0.2224	16.841	12.098	19.112	20.368	16.159
8	0.2234	8.433	9.885	12.405	21.939	8.136
8	0.2244	26.927	20.262	28.743	23.107	10.560
8	0.2254	11.566	19.293	12.615	21.776	23.798
8	0.2264	10.942	17.883	13.413	13.077	14.060
8	0.2274	12.971	22.377	28.345	12.807	14.452
8	0.2284	38.706	28.433	36.989	13.735	26.885
8	0.2294	11.541	10.048	9.094	17.596	26.585
8	0.2304	17.023	16.786	22.831	31.783	17.464
8	0.2314	19.267	30.738	31.161	27.508	24.041
8	0.2324	26.039	23.057	34.149	22.508	24.420
8	0.2334	24.408	19.292	26.896	18.897	23.608
8	0.2344	11.842	13.078	21.508	9.487	17.980
8	0.2354	10.058	20.079	15.469	23.190	21.575
8	0.2364	13.203	16.697	16.849	21.849	20.085
8	0.2374	19.281	15.968	23.585	19.570	18.943
8	0.2384	19.473	17.631	23.653	9.017	20.608
8	0.2394	27.912	28.343	31.249	13.745	13.840
8	0.2404	14.503	10.088	12.300	12.420	22.380
8	0.2414	34.093	21.162	27.025	34.543	34.805
8	0.2424	10.519	21.603	16.557	27.205	19.184
8	0.2434	11.172	12.003	16.751	25.956	15.658
8	0.2444	16.631	21.660	17.293	19.913	16.263

8	0.2454	13.935	16.683	18.962	11.897	18.350
8	0.2464	7.010	6.228	9.122	14.308	13.679
8	0.2474	10.192	7.541	11.823	16.204	16.991
8	0.2484	10.102	10.448	11.902	8.660	14.494
8	0.2494	19.655	21.110	31.831	16.934	24.705
8	0.2504	20.916	14.143	16.693	19.434	14.161
8	0.2514	17.274	20.268	20.539	12.413	10.609
8	0.2524	15.580	12.638	13.443	24.756	24.168
8	0.2534	27.332	29.823	28.938	34.962	15.605
8	0.2544	17.201	23.672	26.536	17.627	15.071
8	0.2554	12.092	17.663	17.834	19.037	13.976
8	0.2564	7.917	11.286	11.976	15.049	7.516
8	0.2574	11.620	17.268	18.780	15.445	8.136
8	0.2584	16.711	24.176	23.914	12.608	11.647
8	0.2594	16.835	17.806	23.939	20.901	28.426
8	0.2604	21.394	21.656	22.524	26.793	27.104
8	0.2614	13.829	13.343	18.393	18.975	25.821
8	0.2624	20.839	21.061	23.775	12.805	9.702
8	0.2634	36.543	34.339	37.510	14.753	20.993
8	0.2644	2.935	7.398	2.458	16.578	7.110
8	0.2654	24.766	26.657	26.762	23.349	14.068
8	0.2664	10.056	12.369	9.535	16.384	11.207
8	0.2674	26.892	16.933	21.340	13.992	12.454
8	0.2684	12.807	15.140	13.084	18.102	14.299
8	0.2694	25.094	20.977	21.145	25.293	16.282
8	0.2704	18.044	16.543	16.155	12.146	17.769
8	0.2714	21.111	19.937	19.618	15.194	14.648
8	0.2724	16.956	10.351	15.140	17.952	14.811
8	0.2734	17.324	11.741	17.608	15.451	18.569
8	0.2744	15.063	15.280	16.658	16.461	26.473
8	0.2754	12.081	7.844	10.772	11.268	6.932
8	0.2764	12.408	10.180	11.749	13.425	16.616
8	0.2774	17.849	7.495	14.754	13.356	20.856
8	0.2784	15.869	14.329	20.093	19.497	13.375
8	0.2794	6.123	5.639	6.052	5.103	4.945
8	0.2804	15.012	7.652	11.358	14.610	6.501
8	0.2814	19.510	10.874	16.876	8.464	16.522
8	0.2824	19.330	10.076	16.351	15.024	18.964
8	0.2834	8.657	6.994	8.633	12.889	16.646
8	0.2844	12.261	13.425	9.270	15.368	19.554
8	0.2854	16.999	13.790	15.049	16.037	12.835
8	0.2864	7.902	7.205	10.534	21.878	14.281
8	0.2874	20.040	14.062	13.798	21.469	17.793
8	0.2884	33.281	25.166	32.036	20.878	26.050
8	0.2894	28.491	17.759	26.048	13.435	22.376
8	0.2904	12.578	10.709	15.854	13.012	11.117
8	0.2914	12.629	21.557	22.056	20.833	13.193
8	0.2924	10.062	11.445	12.498	23.623	16.848
8	0.2934	17.808	17.787	18.397	24.043	16.987
8	0.2944	10.465	11.344	12.096	18.120	9.597

8	0.2954	32.542	22.694	30.701	31.301	19.024
8	0.2964	20.178	18.760	18.162	16.623	8.074
8	0.2974	15.949	17.214	17.963	18.601	9.583
8	0.2984	11.295	12.607	13.728	13.468	9.457
8	0.2994	15.915	17.819	15.477	25.274	30.326
8	0.3004	14.234	15.914	9.530	24.930	20.683
8	0.3014	23.696	20.859	14.255	21.499	21.403
8	0.3024	6.647	10.907	5.434	12.404	14.742
8	0.3034	6.715	9.866	9.314	12.852	9.330
8	0.3044	19.570	15.658	15.891	16.938	14.070
8	0.3054	19.478	10.632	18.730	20.154	18.225
8	0.3064	22.485	14.454	19.310	26.400	22.025
8	0.3074	9.321	13.689	9.748	25.310	11.733
8	0.3084	7.198	14.123	7.782	21.134	14.987
8	0.3094	9.138	8.715	9.821	8.545	10.496
8	0.3104	13.934	8.623	14.974	10.869	8.370
8	0.3114	15.850	13.611	22.716	15.807	13.087
8	0.3124	10.118	9.586	17.398	16.534	6.394
8	0.3134	12.512	8.935	12.264	9.293	2.893
8	0.3144	12.711	9.095	8.442	4.381	6.153
8	0.3154	21.741	16.392	11.299	19.769	22.731
8	0.3164	25.554	19.858	19.822	22.025	18.725
8	0.3174	26.429	25.988	23.627	26.852	21.043
8	0.3184	26.426	25.985	23.624	26.852	21.041
8	0.3194	24.201	13.903	20.680	21.194	16.648
8	0.3204	14.043	11.687	15.859	16.239	20.635
8	0.3214	10.547	9.977	13.305	22.145	20.467
8	0.3224	6.571	13.002	14.708	18.813	12.156
8	0.3234	10.869	6.004	5.408	20.514	20.707
8	0.3244	12.805	4.823	8.371	20.068	23.321
8	0.3254	17.040	8.766	13.684	19.892	20.613
8	0.3264	18.476	14.979	21.485	14.860	21.486
8	0.3274	16.871	10.044	12.761	17.538	25.572
8	0.3284	9.777	5.396	12.622	13.766	10.428
8	0.3294	17.670	23.772	15.406	24.991	21.879
8	0.3304	14.244	8.986	10.597	18.772	19.069
8	0.3314	17.840	12.955	13.456	14.881	17.926
8	0.3324	16.851	11.504	9.051	21.183	16.013
8	0.3334	41.758	15.458	28.902	34.103	31.371
8	0.3344	10.586	17.163	15.395	29.326	17.406
8	0.3354	11.281	8.722	12.103	9.179	14.811
8	0.3364	9.865	9.596	13.462	24.811	24.510
8	0.3374	10.147	11.811	11.372	22.521	16.962
8	0.3384	17.536	21.288	18.232	27.637	36.549
8	0.3394	11.351	10.844	11.464	14.412	39.411
8	0.3404	18.442	17.033	18.734	24.094	42.988
8	0.3414	18.414	17.083	18.712	24.188	42.992
8	0.3424	21.115	26.293	20.829	14.637	24.335
8	0.3434	12.315	17.490	14.710	18.652	15.407
8	0.3444	21.826	14.803	15.715	18.686	15.883

8	0.3454	13.397	7.896	14.955	9.843	8.669
8	0.3464	13.812	13.771	18.243	19.666	14.209
8	0.3474	16.780	15.230	17.492	15.299	17.816
8	0.3484	16.774	15.218	17.488	15.274	17.819
8	0.3494	17.911	11.026	17.015	17.002	19.050
8	0.3504	12.774	7.600	13.468	14.769	21.025
8	0.3514	22.982	19.265	19.463	19.684	9.587
8	0.3524	18.101	16.773	17.345	16.087	21.791
8	0.3534	25.785	23.689	22.605	14.204	18.383
8	0.3544	5.110	7.499	7.491	18.316	11.343
8	0.3554	25.188	19.436	21.273	22.147	21.228
8	0.3564	23.799	18.468	19.562	22.815	29.674
8	0.3574	14.911	18.901	19.372	16.039	29.850
8	0.3584	16.264	8.188	9.924	18.505	24.957
8	0.3594	31.663	29.452	27.630	26.878	30.566
8	0.3604	18.093	13.992	17.333	13.528	26.242
8	0.3614	9.158	8.432	11.085	20.557	13.415
8	0.3624	5.894	11.954	6.634	17.551	13.339
8	0.3634	8.083	12.663	10.668	15.277	13.318
8	0.3644	10.741	10.144	7.222	15.903	15.659
8	0.3654	11.411	19.103	14.296	13.514	5.434
8	0.3664	8.654	14.115	9.670	16.527	3.700
8	0.3674	14.519	17.870	16.942	15.806	6.725
8	0.3684	7.438	12.839	10.232	20.938	8.535
8	0.3694	20.778	21.717	23.262	15.470	9.236
8	0.3704	21.698	22.794	21.443	17.486	18.722
8	0.3714	8.460	9.913	10.555	15.760	10.580
8	0.3724	7.701	10.583	5.726	21.062	10.394
8	0.3734	7.527	10.365	7.542	12.738	4.519
8	0.3744	9.936	9.001	10.230	5.237	5.547
8	0.3754	11.500	12.902	9.186	2.978	10.675
8	0.3764	13.124	17.566	10.292	18.023	17.235
8	0.3774	5.456	8.780	7.275	19.815	10.993
8	0.3784	14.006	9.712	8.858	32.695	22.927
8	0.3794	7.126	11.069	6.260	9.634	10.566
8	0.3804	7.039	10.305	8.994	10.799	8.190
8	0.3814	7.587	13.555	14.793	10.294	14.885
8	0.3824	10.003	7.642	16.207	21.032	14.702
8	0.3834	18.845	12.931	13.811	16.755	13.825
8	0.3844	20.855	15.852	20.314	19.785	15.528
8	0.3854	21.425	9.483	18.631	17.972	19.671
8	0.3864	12.621	7.492	13.826	14.707	21.761
8	0.3874	13.988	3.673	10.187	8.735	10.941
8	0.3884	20.525	17.309	19.907	16.817	35.325
8	0.3894	15.011	14.878	12.936	27.861	31.677
8	0.3904	11.966	8.755	9.638	22.715	23.270
8	0.3914	11.996	12.810	15.387	17.698	13.568
8	0.3924	19.108	17.818	14.407	16.066	15.833
8	0.3934	22.594	18.866	20.928	13.608	14.211
8	0.3944	12.692	11.785	14.481	40.659	31.213

8	0.3954	22.362	12.507	15.546	24.203	28.749
8	0.3964	13.357	9.084	9.171	26.609	25.366
8	0.3974	23.050	16.409	16.245	33.394	24.590
8	0.3984	13.704	7.180	7.961	20.525	18.950
8	0.3994	13.526	13.689	11.112	17.286	9.209
8	0.4004	13.864	15.942	8.969	32.208	24.085
8	0.4014	9.621	6.343	3.462	31.155	22.746
8	0.4024	6.987	4.481	1.800	30.915	13.487
8	0.4034	25.055	13.369	18.100	24.078	22.246
8	0.4044	18.009	10.899	16.223	16.174	17.375
8	0.4054	11.351	9.475	12.914	14.556	18.302
8	0.4064	15.193	9.223	16.927	16.955	13.822
8	0.4074	26.492	20.823	19.188	30.566	29.931
8	0.4084	17.351	13.362	11.999	26.995	23.719
8	0.4094	16.368	9.118	11.240	21.528	16.618
8	0.4104	19.260	10.440	12.373	14.595	11.576
8	0.4114	11.873	10.955	9.629	20.008	14.151
8	0.4124	13.291	9.385	10.364	23.033	29.435
8	0.4134	9.686	10.984	10.918	17.721	8.829
8	0.4144	10.333	11.075	8.807	21.933	23.221
8	0.4154	9.155	13.160	7.900	27.182	18.960
8	0.4164	21.054	14.967	16.663	13.053	24.200
8	0.4174	13.163	13.868	15.178	3.543	12.540
8	0.4184	7.212	7.388	7.117	14.876	4.689
8	0.4194	6.557	6.606	6.811	15.662	9.017
8	0.4204	21.166	13.073	15.085	11.200	10.811
8	0.4214	25.722	23.075	17.094	21.075	14.275
8	0.4224	14.426	14.323	9.489	14.158	11.723
8	0.4234	13.565	12.902	10.522	15.606	13.788
8	0.4244	11.402	11.263	10.818	17.661	9.183
8	0.4254	14.567	19.800	16.963	24.198	22.497
8	0.4264	6.980	14.176	13.387	28.288	17.217
8	0.4274	21.358	18.390	16.346	23.403	19.384
8	0.4284	18.979	12.110	10.695	24.524	18.773
8	0.4294	10.484	15.483	9.322	22.498	11.753
8	0.4304	23.388	22.689	19.165	23.238	12.511
8	0.4314	6.479	4.752	2.753	13.928	8.578
8	0.4324	15.307	22.137	16.059	20.314	14.699
8	0.4334	12.969	15.762	13.333	26.269	24.632
8	0.4344	14.700	15.756	15.655	29.541	19.221
8	0.4354	24.494	22.294	24.090	23.623	14.382
8	0.4364	7.735	16.001	8.087	22.317	12.627
8	0.4374	17.597	17.985	17.880	31.343	16.436
8	0.4384	21.244	22.139	22.544	32.022	15.878
8	0.4394	18.435	19.293	17.111	17.682	9.167
8	0.4404	20.539	18.674	22.655	7.707	17.652
8	0.4414	25.859	34.192	26.217	19.537	9.217
8	0.4424	18.540	23.132	18.544	21.704	24.575
8	0.4434	38.250	35.653	40.780	23.726	18.287
8	0.4444	30.015	16.638	25.691	21.678	22.391

8	0.4454	14.264	16.835	15.118	18.791	14.174
8	0.4464	12.213	15.707	10.209	12.546	20.272
8	0.4474	11.019	15.744	9.610	24.088	28.643
8	0.4484	10.936	15.222	11.228	9.540	13.705
8	0.4494	11.922	17.078	13.093	19.918	21.756
8	0.4504	16.270	8.844	14.264	23.725	24.606
8	0.4514	19.395	12.481	16.430	20.370	23.312
8	0.4524	21.977	20.234	20.628	20.194	16.782
8	0.4534	25.044	25.979	21.690	16.793	15.016
8	0.4544	15.131	18.165	12.898	10.653	16.474
8	0.4554	12.896	9.946	13.984	4.341	18.760
8	0.4564	10.178	8.290	12.256	9.814	5.532
8	0.4574	23.085	14.821	17.792	9.159	10.320
8	0.4584	21.153	14.506	16.583	15.082	8.826
8	0.4594	19.782	16.307	12.613	17.618	19.292
8	0.4604	25.082	15.520	17.932	11.136	16.560
8	0.4614	21.944	18.913	23.718	10.884	8.917
8	0.4624	16.546	15.630	15.734	7.560	7.519
8	0.4634	18.859	10.753	14.654	10.437	11.087
8	0.4644	19.952	19.073	18.453	14.908	20.517
8	0.4654	29.848	25.590	23.903	17.587	19.505
8	0.4664	39.571	32.329	31.186	18.421	24.468
8	0.4674	15.913	13.912	13.679	3.334	3.442
8	0.4684	10.573	12.000	12.204	3.306	3.705
8	0.4694	25.819	25.399	25.005	7.750	13.184
8	0.4704	13.472	6.623	9.699	7.405	4.448
8	0.4714	24.443	15.245	18.800	8.096	7.913
8	0.4724	28.074	14.308	18.768	10.728	11.985
8	0.4734	16.327	20.632	19.629	16.498	10.312
8	0.4744	16.718	14.174	19.417	8.415	8.148
8	0.4754	14.178	15.025	17.096	8.855	4.282
8	0.4764	29.849	19.795	24.292	16.473	15.228
8	0.4774	29.821	19.799	24.246	16.463	15.168
8	0.4784	15.022	11.489	14.492	12.157	18.457
8	0.4794	10.668	9.408	14.096	9.988	15.290
8	0.4804	10.456	6.377	9.227	9.945	7.553
8	0.4814	13.600	9.864	8.349	8.171	6.931
8	0.4824	33.862	21.518	22.920	10.943	9.039
8	0.4834	27.843	16.077	18.793	15.816	13.809
8	0.4844	15.756	11.350	10.901	14.100	13.764
8	0.4854	18.902	17.194	16.308	20.024	10.950
8	0.4864	19.371	7.184	10.108	17.444	17.132
8	0.4874	15.612	9.424	10.870	6.038	8.678
8	0.4884	18.858	15.027	17.801	8.196	8.315
8	0.4894	32.840	13.968	18.359	13.074	20.804
8	0.4904	30.123	13.941	21.849	13.458	17.825
8	0.4914	44.373	23.794	25.180	18.042	22.447
8	0.4924	29.266	25.920	27.502	13.277	15.033
8	0.4934	19.742	12.036	13.575	9.840	10.915
8	0.4944	18.667	13.726	12.391	10.418	19.069

8	0.4954	10.932	10.251	11.246	11.412	7.262
8	0.4964	18.232	14.099	18.275	7.929	5.713
8	0.4974	36.948	19.127	23.573	22.847	16.767
8	0.4984	21.564	14.165	14.879	21.062	20.378
8	0.4994	11.662	10.158	7.703	13.783	13.554
8	0.5004	14.951	7.770	10.888	8.575	13.849
8	0.5014	12.524	7.673	12.029	12.571	11.171
8	0.5024	36.267	17.836	22.053	17.290	15.563
8	0.5034	27.036	21.627	20.747	8.722	10.901
8	0.5044	11.114	8.883	8.438	7.294	6.437
8	0.5054	16.878	13.870	20.073	12.254	4.552
8	0.5064	34.972	22.659	30.682	11.395	8.932
8	0.5074	25.547	14.073	19.828	17.105	14.096
8	0.5084	11.348	8.278	8.694	16.580	21.045
8	0.5094	23.581	20.249	20.988	16.359	10.025
8	0.5104	15.477	12.999	10.097	5.986	8.324
8	0.5114	13.345	12.763	7.726	7.316	8.628
8	0.5124	20.919	16.913	13.160	13.802	18.864
8	0.5134	16.510	11.983	16.307	9.922	17.129
8	0.5144	33.732	18.732	19.046	12.284	22.552
8	0.5154	21.521	22.664	18.292	6.861	10.606
8	0.5164	31.435	20.592	24.640	9.233	13.851
8	0.5174	19.866	11.110	16.524	7.239	18.496
8	0.5184	9.235	13.235	9.930	20.372	23.126
8	0.5194	9.190	13.265	9.900	20.457	23.088
8	0.5204	7.221	12.047	11.441	18.600	13.535
8	0.5214	7.207	12.043	11.431	18.605	13.526
8	0.5224	13.611	5.559	15.339	27.741	21.470
8	0.5234	2.754	6.511	3.363	10.928	9.984
8	0.5244	11.122	13.792	11.826	10.324	10.651
8	0.5254	11.093	13.770	11.855	10.352	10.759
8	0.5264	26.737	16.446	24.752	16.029	14.111
8	0.5274	17.252	10.402	13.035	30.660	14.267
8	0.5284	13.778	12.921	13.809	14.084	12.947
8	0.5294	21.089	9.415	20.773	14.330	23.628
8	0.5304	13.424	6.180	8.706	15.127	15.189
8	0.5314	20.061	8.804	14.096	10.699	11.479
8	0.5324	18.669	10.353	15.565	12.416	17.201
8	0.5334	13.620	7.777	16.490	15.618	9.338
8	0.5344	8.041	4.188	8.896	13.353	8.687
8	0.5354	12.847	13.502	7.536	23.403	14.482
8	0.5364	9.677	5.762	9.210	7.608	4.793
8	0.5374	26.154	22.933	28.576	18.810	13.587
8	0.5384	29.568	18.110	26.665	19.596	22.208
8	0.5394	26.202	16.908	25.707	23.787	21.281
8	0.5404	23.752	15.918	23.884	13.971	8.819
8	0.5414	12.199	9.045	10.414	25.037	19.299
8	0.5424	9.834	13.269	13.150	12.932	4.953
8	0.5434	29.241	22.933	29.769	12.725	20.957
8	0.5444	23.931	20.619	24.203	20.713	23.095

8	0.5454	11.232	8.501	14.912	5.777	16.078
8	0.5464	19.837	16.446	15.005	13.763	17.910
8	0.5474	25.338	16.871	19.059	12.245	23.265
8	0.5484	20.210	14.112	23.523	6.626	10.002
8	0.5494	17.138	8.796	15.490	10.287	14.931
8	0.5504	23.409	24.557	24.595	16.727	22.748
8	0.5514	15.952	11.462	13.723	8.411	13.748
8	0.5524	15.967	11.474	13.732	8.425	13.773
8	0.5534	20.562	12.963	21.072	8.150	16.449
8	0.5544	20.194	15.128	20.188	19.122	15.446
8	0.5554	22.810	15.079	25.150	10.879	15.834
8	0.5564	21.192	17.988	23.155	15.966	14.517
8	0.5574	20.373	15.322	24.597	10.044	8.336
8	0.5584	20.320	11.451	20.029	11.602	10.315
8	0.5594	30.179	22.132	26.457	11.350	12.798
8	0.5604	23.264	20.106	19.083	11.838	7.585
8	0.5614	13.661	11.880	8.821	14.326	11.043
8	0.5624	15.347	15.218	12.285	10.927	14.745
8	0.5634	12.427	10.169	12.170	10.893	15.872
8	0.5644	15.442	14.125	16.105	10.078	16.174
8	0.5654	14.595	12.292	11.044	9.485	21.658
8	0.5664	12.687	11.770	19.977	6.622	14.093
8	0.5674	12.282	12.971	11.322	8.167	14.247
8	0.5684	5.208	7.952	5.501	4.449	2.876
8	0.5694	27.891	19.853	19.910	8.432	11.328
8	0.5704	14.589	15.515	13.701	13.486	14.107
8	0.5714	15.715	10.486	13.346	11.419	11.825
8	0.5724	25.754	17.923	22.126	12.717	23.706
8	0.5734	20.758	9.889	13.617	8.162	16.967
8	0.5744	10.581	12.262	9.178	9.417	7.663
8	0.5754	5.615	8.937	8.457	11.744	8.604
8	0.5764	20.331	15.606	20.221	13.582	19.504
8	0.5774	32.712	22.066	20.869	10.668	14.106
8	0.5784	16.532	15.003	18.020	6.303	9.472
8	0.5794	15.701	8.453	10.787	8.573	15.806
8	0.5804	18.533	8.537	13.575	11.216	13.473
8	0.5814	11.900	8.708	12.997	7.186	10.427
8	0.5824	10.050	7.055	11.094	9.849	8.901
8	0.5834	18.686	19.103	16.268	5.654	8.081
8	0.5844	20.970	15.721	15.711	7.290	6.253
8	0.5854	13.015	11.739	12.654	6.244	8.952
8	0.5864	16.290	14.357	14.241	9.951	15.331
8	0.5874	7.097	11.830	10.664	6.952	5.694
8	0.5884	7.594	11.514	5.717	5.468	7.330
8	0.5894	2.250	4.920	2.499	5.453	3.442
8	0.5904	8.386	12.221	10.823	11.532	6.331
8	0.5914	14.242	16.978	14.138	19.990	12.956
8	0.5924	16.120	16.291	17.873	12.043	13.174
8	0.5934	17.971	13.928	10.774	12.379	15.880
8	0.5944	9.410	9.162	10.809	13.232	26.334

8	0.5954	30.361	19.535	24.358	8.440	11.945
8	0.5964	9.581	8.772	10.876	8.630	9.562
8	0.5974	6.237	7.484	5.787	4.699	10.319
8	0.5984	16.635	11.572	11.564	15.990	13.894
8	0.5994	23.856	24.866	16.964	18.082	16.596
8	0.6004	15.817	14.568	9.839	18.194	16.657
8	0.6014	15.888	13.695	10.862	12.991	10.402
8	0.6024	16.774	8.992	16.424	4.501	6.390
8	0.6034	3.983	8.507	6.984	12.017	18.890
8	0.6044	4.226	12.045	12.110	14.087	24.957
8	0.6054	14.370	14.772	11.719	12.012	8.562
8	0.6064	12.893	10.400	11.556	4.548	15.496
8	0.6074	16.456	13.394	13.674	8.140	12.498
8	0.6084	17.682	15.393	17.783	9.608	13.821
8	0.6094	10.628	19.406	14.875	6.143	4.586
8	0.6104	9.683	11.894	10.580	8.749	6.839
8	0.6114	10.009	6.946	7.277	15.460	9.428
8	0.6124	11.943	12.238	10.960	11.704	8.464
8	0.6134	18.171	9.064	10.915	16.220	14.883
8	0.6144	20.915	15.712	20.426	16.852	10.513
8	0.6154	10.198	12.831	10.388	13.269	10.279
8	0.6164	9.673	6.226	7.263	9.420	21.449
8	0.6174	3.310	4.966	1.389	6.161	12.484
8	0.6184	10.451	10.372	10.147	10.894	7.949
8	0.6194	18.276	14.795	14.589	19.663	10.795
8	0.6204	14.012	14.174	11.897	13.097	6.697
8	0.6214	14.085	12.500	14.080	6.655	13.565
8	0.6224	6.167	5.356	4.394	9.776	16.933
8	0.6234	20.573	15.970	14.965	9.088	13.594
8	0.6244	5.791	6.256	5.676	10.108	10.024
8	0.6254	4.637	3.652	4.146	4.923	8.487
8	0.6264	7.298	3.849	6.705	5.935	9.846
8	0.6274	11.180	13.958	9.063	8.601	9.294
8	0.6284	8.291	15.138	7.558	14.775	11.781
8	0.6294	18.833	18.312	16.687	8.734	3.976
8	0.6304	6.422	4.524	5.610	9.109	4.140
8	0.6314	9.178	9.906	10.061	8.038	4.242
8	0.6324	18.329	16.545	12.913	13.234	7.101
8	0.6334	3.355	9.319	6.103	12.830	14.947
8	0.6344	4.561	11.311	6.991	19.470	6.449
8	0.6354	6.221	9.328	8.337	8.990	6.236
8	0.6364	9.463	14.215	14.057	10.509	5.593
8	0.6374	9.462	14.207	14.054	10.502	5.604
8	0.6384	5.221	6.642	7.248	5.853	2.823
8	0.6394	7.982	8.620	9.629	9.183	7.998
8	0.6404	12.352	11.480	12.938	13.792	10.713
8	0.6414	12.637	11.615	13.102	13.606	10.727
8	0.6424	16.072	18.703	16.798	13.073	18.301
8	0.6434	5.316	5.029	5.813	4.004	14.907
8	0.6444	4.064	5.603	8.360	10.730	22.762

8	0.6454	12.968	14.216	16.193	12.428	29.724
8	0.6464	12.174	14.787	13.762	19.007	21.092
8	0.6474	9.131	10.999	6.968	9.331	18.403
8	0.6484	14.866	16.152	16.199	8.239	15.934
8	0.6494	9.659	15.156	15.009	8.872	11.613
8	0.6504	30.224	21.457	29.075	10.633	10.964
8	0.6514	26.817	18.874	23.011	11.686	17.657
8	0.6524	6.851	4.940	6.918	16.951	23.372
8	0.6534	16.894	9.965	13.903	10.895	26.567
8	0.6544	29.219	18.528	24.020	8.782	9.941
8	0.6554	32.716	20.081	24.238	9.052	10.555
8	0.6564	20.459	11.425	13.662	11.980	14.133
8	0.6574	40.692	29.147	33.906	13.113	7.380
8	0.6584	39.196	28.299	31.732	12.409	9.113
8	0.6594	23.216	16.659	22.752	11.195	11.218
8	0.6604	15.833	16.054	7.928	14.802	10.270
8	0.6614	19.152	22.382	15.072	18.532	15.527
8	0.6624	8.111	13.637	8.172	14.652	13.220
8	0.6634	15.697	20.232	14.187	12.816	16.671
8	0.6644	19.285	19.328	17.746	13.695	12.309
8	0.6654	15.042	16.493	13.189	19.675	16.827
8	0.6664	13.297	13.674	10.827	15.020	13.182
8	0.6674	24.971	12.574	14.133	16.317	12.861
8	0.6684	19.208	13.031	11.226	14.616	18.224
8	0.6694	19.975	19.021	16.525	14.606	12.512
8	0.6704	19.224	21.573	16.704	18.143	8.197
8	0.6714	18.955	13.983	10.214	23.691	17.887
8	0.6724	24.960	16.903	18.001	16.607	19.488
8	0.6734	23.544	18.001	15.966	20.354	16.156
8	0.6744	24.179	19.809	17.426	15.609	15.520
8	0.6754	19.423	15.162	17.924	11.001	9.366
8	0.6764	29.480	18.306	20.073	16.072	15.231
8	0.6774	33.920	13.309	16.511	13.537	17.935
8	0.6784	24.627	13.154	18.600	19.012	16.824
8	0.6794	24.511	13.157	18.538	18.905	16.797
8	0.6804	4.225	5.358	5.912	7.413	7.468
8	0.6814	15.753	8.789	16.028	5.752	2.409
8	0.6824	15.579	13.741	16.141	10.165	6.755
8	0.6834	9.629	2.553	6.973	12.799	11.855
8	0.6844	11.876	14.940	10.744	10.128	6.599
8	0.6854	14.822	4.939	5.712	17.535	13.487
8	0.6864	10.228	9.103	5.716	13.071	16.003
8	0.6874	13.952	12.611	14.770	12.873	4.503
8	0.6884	19.067	11.708	12.683	23.509	8.534
8	0.6894	14.205	11.390	10.326	22.880	10.252
8	0.6904	18.754	9.298	13.224	9.262	7.148
8	0.6914	23.137	18.610	21.345	11.514	7.626
8	0.6924	20.546	15.243	15.830	13.096	7.720
8	0.6934	24.182	16.979	15.718	29.015	18.364
8	0.6944	27.045	23.067	23.042	17.032	13.210

8	0.6954	23.995	17.312	20.632	21.995	10.641
8	0.6964	28.858	14.257	19.533	20.643	13.306
8	0.6974	23.774	10.481	14.331	19.022	15.540
8	0.6984	26.292	14.693	19.305	12.128	12.200
8	0.6994	22.829	10.837	18.186	6.359	8.349
8	0.7004	26.303	15.253	18.837	10.869	12.725
8	0.7014	11.207	5.011	9.677	9.423	3.928
8	0.7024	26.798	9.174	21.388	21.126	15.713
8	0.7034	22.462	9.005	18.710	19.884	13.155
8	0.7044	16.809	6.998	10.357	13.036	10.535
8	0.7054	22.311	14.935	15.765	21.663	21.717
8	0.7064	18.380	13.351	14.007	28.511	12.496
8	0.7074	19.351	16.126	15.663	21.904	20.540
8	0.7084	18.154	12.729	18.360	22.891	14.124
8	0.7094	16.208	5.328	8.843	19.065	10.326
8	0.7104	18.144	11.513	13.398	20.071	15.127
8	0.7114	17.974	11.509	13.341	20.255	15.071
8	0.7124	14.020	7.405	8.379	23.013	7.869
8	0.7134	15.816	13.386	19.552	8.759	2.493
8	0.7144	17.868	17.331	17.310	17.205	10.758
8	0.7154	9.569	15.520	10.570	28.576	8.066
8	0.7164	15.412	21.678	13.282	20.465	7.103
8	0.7174	7.885	6.636	8.634	3.220	2.594
8	0.7184	8.670	6.058	10.079	11.310	4.855
8	0.7194	11.936	16.163	11.990	24.627	9.291
8	0.7204	13.637	14.002	10.041	21.412	18.329
8	0.7214	14.952	8.497	12.655	18.695	10.653
8	0.7224	10.636	8.262	6.532	20.800	15.877
8	0.7234	15.957	10.558	10.736	16.745	17.462
8	0.7244	6.879	11.107	13.559	9.386	6.596
8	0.7254	12.311	3.867	9.378	15.972	14.838
8	0.7264	12.545	8.414	10.768	27.545	11.972
8	0.7274	9.841	9.532	6.737	18.288	15.247
8	0.7284	18.891	17.716	17.622	24.542	19.426
8	0.7294	23.401	17.463	20.409	12.219	13.296
8	0.7304	14.663	12.704	12.650	13.163	8.185
8	0.7314	7.235	12.688	8.277	12.508	5.312
8	0.7324	15.979	9.984	12.281	17.914	13.081
8	0.7334	12.319	10.375	11.692	15.192	12.437
8	0.7344	9.233	9.609	9.374	3.394	6.375
8	0.7354	3.131	3.625	2.616	5.870	8.076
8	0.7364	5.627	10.433	9.370	4.444	6.989
8	0.7374	10.277	7.003	8.676	9.775	12.150
8	0.7384	12.649	11.206	12.220	16.097	17.640
8	0.7394	9.663	9.614	8.034	9.510	14.485
8	0.7404	7.888	13.704	9.377	20.063	12.607
8	0.7414	9.822	12.549	10.862	27.847	15.179
8	0.7424	12.435	15.501	17.845	13.695	25.695
8	0.7434	21.598	10.561	21.542	25.515	21.696
8	0.7444	9.391	7.889	7.587	14.408	10.071

8	0.7454	9.201	4.195	7.568	10.096	11.658
8	0.7464	14.404	11.957	14.634	12.383	18.262
8	0.7474	11.633	11.687	11.893	11.829	7.827
8	0.7484	16.091	20.915	19.615	16.568	13.352
8	0.7494	14.772	16.321	12.001	14.003	14.083
8	0.7504	16.102	19.768	13.073	20.320	21.057
8	0.7514	10.327	9.672	10.828	18.678	13.951
8	0.7524	10.327	9.680	10.830	18.713	13.950
8	0.7534	18.312	18.753	19.268	16.278	14.199
8	0.7544	24.833	21.258	23.782	11.702	20.475
8	0.7554	17.127	13.663	16.885	16.605	12.092
8	0.7564	23.010	17.030	16.007	14.671	19.492
8	0.7574	23.824	15.469	21.400	19.316	12.672
8	0.7584	20.390	10.339	15.017	19.345	22.718
8	0.7594	25.213	11.944	15.329	15.089	12.345
8	0.7604	14.596	16.352	9.817	11.419	13.062
8	0.7614	12.785	14.950	10.926	14.159	14.987
8	0.7624	13.058	15.610	14.150	25.817	26.941
8	0.7634	19.351	14.654	18.681	21.083	10.294
8	0.7644	15.449	9.930	15.787	17.726	14.216
8	0.7654	10.191	6.864	9.882	7.942	5.430
8	0.7664	10.540	10.183	10.638	6.735	4.330
8	0.7674	9.645	6.672	9.608	8.825	7.796
8	0.7684	16.705	11.969	13.650	10.219	9.847
8	0.7694	15.742	14.597	13.429	3.389	14.691
8	0.7704	14.158	15.544	11.871	23.688	12.027
8	0.7714	16.903	10.075	15.305	14.453	11.327
8	0.7724	10.203	7.968	9.412	20.079	7.765
8	0.7734	16.513	11.462	13.442	16.153	3.002
8	0.7744	30.167	15.505	26.863	22.631	9.259
8	0.7754	20.675	8.681	14.467	25.372	20.353
8	0.7764	14.975	8.299	12.791	11.980	5.577
8	0.7774	24.223	21.352	24.076	15.516	13.443
8	0.7784	33.276	12.974	28.288	14.314	9.357
8	0.7794	23.917	20.078	22.315	18.954	16.739
8	0.7804	5.801	6.316	6.163	11.958	17.821
8	0.7814	23.853	17.129	24.902	37.080	15.654
8	0.7824	16.571	15.347	17.599	26.228	16.048
8	0.7834	13.544	11.680	14.690	16.451	20.277
8	0.7844	14.863	18.282	16.391	21.723	6.093
8	0.7854	18.286	18.111	24.882	14.013	10.782
8	0.7864	16.512	12.939	16.498	13.390	8.750
8	0.7874	24.513	14.617	23.924	28.380	15.396
8	0.7884	13.909	13.479	15.055	10.002	19.025
8	0.7894	23.930	18.827	19.182	14.003	26.154
8	0.7904	14.164	12.679	13.755	11.253	22.056
8	0.7914	21.501	15.849	17.155	35.754	23.460
8	0.7924	33.649	11.118	24.466	24.787	22.232
8	0.7934	10.471	5.622	10.454	17.672	13.949
8	0.7944	11.474	7.689	15.807	17.355	10.776

8	0.7954	15.602	11.584	14.415	16.879	23.386
8	0.7964	17.554	15.805	15.848	16.182	12.731
8	0.7974	26.177	22.946	25.582	24.270	17.616
8	0.7984	23.391	14.255	13.246	13.878	18.690
8	0.7994	7.212	5.708	6.283	12.856	10.969
8	0.8004	11.114	6.091	9.821	8.019	16.416
8	0.8014	11.119	6.097	9.824	8.016	16.502
8	0.8024	8.688	5.051	15.249	11.375	4.669
8	0.8034	11.404	12.488	15.807	9.090	14.904
8	0.8044	16.091	11.250	20.145	11.631	20.733
8	0.8054	5.355	4.577	4.659	6.838	4.859
8	0.8064	11.256	10.560	17.599	10.828	14.382
8	0.8074	7.941	7.156	10.099	7.267	10.694
8	0.8084	15.683	8.084	9.042	21.245	17.229
8	0.8094	15.583	8.047	8.979	21.415	17.229
8	0.8104	24.124	22.267	24.416	33.122	34.652
8	0.8114	15.620	16.417	18.767	11.200	16.725
8	0.8124	11.730	9.964	11.005	4.374	16.490
8	0.8134	8.765	6.765	6.061	13.629	20.341
8	0.8144	10.639	7.192	7.037	14.967	22.793
8	0.8154	18.257	9.721	10.293	7.306	17.121
8	0.8164	9.738	6.958	7.760	19.960	19.077
8	0.8174	15.043	13.970	12.998	18.604	20.405
8	0.8184	11.735	14.331	12.373	15.795	31.331
8	0.8194	9.070	12.429	7.542	19.225	13.089
8	0.8204	5.016	7.262	1.951	20.189	9.067
8	0.8214	13.939	8.068	11.774	5.910	10.283
8	0.8224	8.887	8.540	8.967	5.504	8.458
8	0.8234	15.414	15.456	16.847	8.243	7.211
8	0.8244	8.124	9.642	9.921	27.039	21.846
8	0.8254	18.214	17.528	16.888	25.791	25.577
8	0.8264	16.621	16.839	13.086	29.662	19.590
8	0.8274	17.415	9.155	13.347	23.021	8.997
8	0.8284	10.805	6.827	8.786	10.624	13.370
8	0.8294	6.457	7.307	8.346	9.606	11.969
8	0.8304	5.635	1.927	6.158	8.150	13.081
8	0.8314	16.198	6.878	13.901	16.513	14.271
8	0.8324	16.900	6.335	13.888	20.574	13.855
8	0.8334	16.525	13.020	15.787	12.297	10.321
8	0.8344	16.012	9.178	16.323	11.553	13.757
8	0.8354	8.450	4.552	8.558	16.500	21.594
8	0.8364	4.879	6.737	4.984	7.625	18.060
8	0.8374	10.791	10.344	5.898	18.672	17.986
8	0.8384	14.152	6.811	10.297	25.643	13.689
8	0.8394	15.261	10.268	14.218	25.849	13.858
8	0.8404	26.639	17.463	28.181	28.222	19.426
8	0.8414	19.635	13.630	16.161	21.251	8.797
8	0.8424	15.690	7.874	10.850	19.983	15.638
8	0.8434	14.671	7.154	11.332	15.582	21.915
8	0.8444	11.919	12.774	11.302	23.476	22.018

8	0.8454	15.771	11.586	23.688	16.555	20.993
8	0.8464	11.849	14.604	14.072	10.085	15.838
8	0.8474	10.898	10.675	13.440	16.483	16.625
8	0.8484	11.057	14.734	10.711	20.013	8.611
8	0.8494	16.979	14.637	12.682	22.716	12.230
8	0.8504	22.661	13.432	21.367	25.621	17.261
8	0.8514	15.817	8.569	18.000	18.163	5.176
8	0.8524	7.453	6.118	5.363	15.918	12.802
8	0.8534	16.016	14.853	13.506	18.785	19.233
8	0.8544	22.692	20.315	17.156	17.953	7.655
8	0.8554	8.865	15.353	10.397	12.368	3.569
8	0.8564	11.230	4.809	8.162	16.759	18.459
8	0.8574	10.723	12.339	12.774	16.975	20.615
8	0.8584	13.558	14.413	12.529	20.942	24.931
8	0.8594	9.382	12.930	9.800	9.776	11.571
8	0.8604	12.961	10.393	9.905	14.768	25.487
8	0.8614	15.953	15.115	13.586	13.521	21.017
8	0.8624	28.422	12.597	21.000	21.917	14.681
8	0.8634	15.037	18.008	15.779	17.456	12.558
8	0.8644	5.604	5.456	4.087	15.055	20.587
8	0.8654	10.032	12.336	11.051	18.952	22.544
8	0.8664	22.810	14.245	18.610	14.773	13.413
8	0.8674	16.073	17.296	13.871	16.350	9.342
8	0.8684	6.031	5.255	3.187	9.701	6.583
8	0.8694	7.154	4.095	4.781	10.065	6.252
8	0.8704	15.441	9.236	8.636	24.426	15.677
8	0.8714	20.546	17.862	15.237	29.765	16.115
8	0.8724	15.709	17.978	14.964	17.482	14.733
8	0.8734	18.393	16.584	17.596	13.799	19.718
8	0.8744	17.349	14.661	16.916	18.993	18.310
8	0.8754	6.793	10.395	7.615	17.994	15.426
8	0.8764	5.201	4.491	5.776	20.018	13.332
8	0.8774	10.508	5.920	7.737	20.662	10.415
8	0.8784	11.971	9.554	11.995	15.725	12.518
8	0.8794	8.315	13.566	11.438	16.164	12.718
8	0.8804	14.500	14.005	14.245	16.603	13.686
8	0.8814	18.931	14.650	16.499	20.776	14.214
8	0.8824	17.155	20.062	15.838	17.722	16.436
8	0.8834	7.888	9.258	8.048	21.767	20.285
8	0.8844	10.779	15.688	9.680	9.600	6.519
8	0.8854	18.371	15.302	14.046	15.599	6.373
8	0.8864	13.757	11.806	12.152	9.111	9.003
8	0.8874	13.299	10.526	17.478	13.146	11.966
8	0.8884	16.617	11.163	16.123	19.845	12.622
8	0.8894	13.183	11.080	12.514	19.008	10.369
8	0.8904	7.852	11.620	10.892	19.014	7.431
8	0.8914	6.907	7.829	8.827	16.665	8.300
8	0.8924	11.397	8.539	11.108	4.164	7.772
8	0.8934	15.128	10.313	16.673	18.698	14.146
8	0.8944	15.138	10.327	16.746	18.685	13.939

8	0.8954	13.826	8.797	13.162	24.782	17.398
8	0.8964	9.808	8.815	8.468	10.965	13.616
8	0.8974	8.898	8.270	11.619	14.523	9.731
8	0.8984	7.055	8.298	11.738	11.021	6.838
8	0.8994	11.018	9.338	9.761	13.290	12.818
8	0.9004	9.711	4.594	5.113	24.411	18.293
8	0.9014	12.186	10.854	13.866	12.267	17.329
8	0.9024	11.697	12.033	15.739	9.538	17.952
8	0.9034	10.989	11.843	16.088	12.090	8.311
8	0.9044	11.041	11.809	14.535	10.863	6.873
8	0.9054	9.542	12.576	12.055	23.272	8.269
8	0.9064	17.938	18.509	18.288	17.484	10.340
8	0.9074	18.274	21.308	16.010	16.647	11.253
8	0.9084	12.954	21.390	13.530	12.109	10.574
8	0.9094	16.892	17.635	16.445	15.069	9.637
8	0.9104	11.055	19.044	13.330	17.203	7.515
8	0.9114	11.063	19.048	13.326	17.281	7.502
8	0.9124	11.070	19.053	13.321	17.362	7.490
8	0.9134	13.691	18.180	16.940	8.232	9.348
8	0.9144	17.574	21.931	20.802	18.325	10.382
8	0.9154	15.844	21.315	18.838	20.202	12.242
8	0.9164	13.614	20.722	16.700	16.124	15.255
8	0.9174	16.065	17.663	20.712	22.075	7.311
8	0.9184	16.176	12.998	12.052	12.927	12.174
8	0.9194	14.896	12.180	12.923	13.688	12.787
8	0.9204	11.317	14.192	16.013	15.780	6.355
8	0.9214	12.286	14.235	15.823	11.725	12.098
8	0.9224	8.988	8.951	10.642	15.694	7.782
8	0.9234	12.564	6.873	12.763	9.945	10.165
8	0.9244	12.654	9.693	14.677	4.808	7.357
8	0.9254	8.080	10.137	13.517	8.874	12.461
8	0.9264	8.444	10.760	13.962	9.887	7.778
8	0.9274	8.413	10.778	13.911	9.955	7.948
8	0.9284	6.287	7.718	6.075	5.462	17.856
8	0.9294	4.423	6.670	7.155	6.170	7.312
8	0.9304	6.197	8.470	12.582	7.022	3.988
8	0.9314	8.390	8.255	14.752	23.520	4.574
8	0.9324	10.095	14.115	17.239	32.723	9.652
8	0.9334	7.603	9.610	13.714	18.903	11.537
8	0.9344	6.109	13.301	7.607	18.386	9.338
8	0.9354	9.553	9.668	9.787	12.345	3.603
8	0.9364	11.654	9.163	15.333	9.265	8.188
8	0.9374	16.715	22.216	25.687	21.944	14.680
8	0.9384	17.131	24.414	20.665	31.509	20.785
8	0.9394	20.005	28.134	21.053	30.953	15.870
8	0.9404	17.635	23.090	21.826	21.528	11.614
8	0.9414	13.180	18.546	13.572	12.286	20.311
8	0.9424	15.177	20.579	23.338	13.235	10.044
8	0.9434	12.083	13.077	18.917	20.192	19.064
8	0.9444	11.045	10.186	14.955	31.594	12.214

8	0.9454	16.541	13.308	18.079	9.502	13.575
8	0.9464	24.598	25.981	33.611	22.924	10.469
8	0.9474	23.536	21.330	23.180	13.666	6.147
8	0.9484	12.445	13.869	10.903	18.173	7.951
8	0.9494	6.450	6.748	10.051	19.308	8.542
8	0.9504	15.148	14.628	25.117	15.580	13.638
8	0.9514	12.922	13.766	15.913	22.210	12.505
8	0.9524	12.885	13.753	15.927	22.054	12.617
8	0.9534	18.477	23.994	22.726	31.953	15.844
8	0.9544	14.429	19.740	14.764	25.889	14.667
8	0.9554	9.407	12.101	11.047	15.594	8.846
8	0.9564	12.048	17.060	15.261	17.444	13.034
8	0.9574	15.705	19.354	21.053	15.509	14.668
8	0.9584	12.571	13.839	15.533	15.806	11.908
8	0.9594	11.220	10.432	13.776	9.916	13.451
8	0.9604	15.499	15.107	16.975	9.552	18.488
8	0.9614	18.361	20.317	22.446	7.705	18.414
8	0.9624	13.617	15.342	15.619	12.162	15.139
8	0.9634	9.306	12.322	12.593	9.648	12.092
8	0.9644	8.856	8.098	11.312	10.176	10.957
8	0.9654	13.389	9.418	15.830	14.170	8.354
8	0.9664	21.050	22.167	21.014	19.469	16.627
8	0.9674	12.037	14.133	14.763	16.535	18.237
8	0.9684	20.533	16.345	29.784	21.011	9.915
8	0.9694	13.953	11.944	15.164	19.534	12.879
8	0.9704	10.481	16.848	12.271	12.580	17.015
8	0.9714	10.499	16.735	12.172	12.470	17.165
8	0.9724	10.348	11.837	7.807	19.884	17.991
8	0.9734	7.250	10.334	9.132	11.873	17.735
8	0.9744	7.321	10.258	9.022	11.707	18.155
8	0.9754	11.920	9.269	9.887	9.699	21.219
8	0.9764	12.941	8.873	14.036	10.980	6.654
8	0.9774	23.939	21.275	27.904	18.580	9.075
8	0.9784	14.260	12.503	13.290	18.467	17.207
8	0.9794	14.667	11.919	14.301	15.009	15.979
8	0.9804	14.644	14.127	14.133	21.560	19.657
8	0.9814	13.728	16.744	13.542	12.359	22.307
8	0.9824	16.830	11.947	10.136	10.415	15.827
8	0.9834	12.673	14.057	10.635	23.446	16.291
8	0.9844	15.280	14.398	18.285	20.747	20.307
8	0.9854	20.783	15.427	20.326	29.087	23.528
8	0.9864	19.519	8.606	14.597	27.336	24.484
8	0.9874	14.639	7.821	6.486	14.751	24.096
8	0.9884	18.618	18.194	15.410	14.272	23.664
8	0.9894	18.652	18.229	15.391	14.271	23.637
8	0.9904	11.046	9.369	9.507	16.000	13.964
8	0.9914	13.311	9.171	9.252	19.590	20.660
8	0.9924	10.877	8.753	4.819	20.981	12.654
8	0.9934	10.818	9.760	5.487	22.640	19.061
8	0.9944	12.793	10.574	10.660	25.628	23.153

8	0.9954	26.882	18.901	18.138	15.795	21.772
8	0.9964	17.524	16.642	13.370	16.268	12.750
8	0.9974	5.969	9.881	4.594	19.081	10.809
8	0.9984	13.437	9.175	5.553	20.060	20.419
8	0.9994	9.722	6.994	4.951	8.330	13.189
8	1.0004	10.380	7.979	6.965	10.930	14.007
8	1.0014	19.507	9.765	12.902	20.383	19.975
8	1.0024	7.714	11.653	6.579	12.168	10.978
8	1.0034	8.344	16.026	5.190	23.417	28.030
8	1.0044	13.430	18.919	10.035	23.430	20.500
8	1.0054	10.554	8.228	3.883	28.908	17.326
8	1.0064	15.198	16.570	9.556	27.011	26.054
8	1.0074	19.603	13.760	15.703	17.532	19.216
8	1.0084	14.393	18.664	12.331	32.304	27.764
8	1.0094	11.314	9.068	11.849	17.298	17.150
8	1.0104	13.439	18.091	9.487	22.614	15.790
8	1.0114	16.076	16.573	13.765	27.542	25.047
8	1.0124	18.026	13.160	11.719	10.169	11.430
8	1.0134	12.329	12.399	9.930	15.777	19.924
8	1.0144	13.238	6.184	5.333	17.880	34.127
8	1.0154	8.112	9.551	3.739	15.442	12.098
8	1.0164	13.493	11.073	6.809	25.235	23.825
8	1.0174	14.952	10.235	7.946	20.061	19.879
8	1.0184	11.631	13.321	9.649	20.877	16.815
8	1.0194	3.146	4.517	4.010	14.143	10.379
8	1.0204	16.428	9.310	9.665	17.082	19.611
8	1.0214	21.035	14.452	17.593	18.818	17.543
8	1.0224	22.126	10.753	13.142	26.628	24.970
8	1.0234	10.434	9.371	9.914	9.765	13.968
8	1.0244	8.574	8.197	8.563	10.690	13.527
8	1.0254	28.811	18.449	16.909	12.955	26.415
8	1.0264	24.834	24.352	18.434	15.982	14.803
8	1.0274	14.209	19.870	14.338	12.897	15.178
8	1.0284	26.576	18.356	19.010	26.031	13.740
8	1.0294	21.955	15.927	12.518	17.746	31.749
8	1.0304	31.352	14.575	15.593	17.251	34.364
8	1.0314	14.715	8.742	6.558	11.346	18.291
8	1.0324	12.104	5.849	5.048	18.008	31.000
8	1.0334	20.266	22.228	19.701	13.768	21.180
8	1.0344	26.719	32.830	23.756	12.522	23.618
8	1.0354	17.604	20.235	11.999	15.274	12.711
8	1.0364	17.560	14.671	12.058	6.683	14.276
8	1.0374	14.972	16.759	8.999	17.965	19.047
8	1.0384	8.301	8.741	3.528	9.139	12.449
8	1.0394	4.550	9.104	6.702	13.108	20.923
8	1.0404	10.287	9.569	6.773	8.080	13.385
8	1.0414	6.777	11.936	8.940	11.183	6.066
8	1.0424	9.394	12.013	8.357	13.902	12.537
8	1.0434	12.058	11.854	8.281	24.719	12.554
8	1.0444	22.711	17.808	19.724	10.323	23.632

8	1.0454	12.205	10.415	12.390	13.677	14.077
8	1.0464	17.488	9.110	11.229	16.512	19.274
8	1.0474	21.162	9.554	14.543	20.714	20.552
8	1.0484	8.714	5.000	4.885	8.940	15.309
8	1.0494	16.149	17.299	14.149	14.618	9.485
8	1.0504	10.650	10.365	7.960	11.230	16.939
8	1.0514	17.627	11.661	15.850	18.088	14.712
8	1.0524	15.515	16.565	14.114	12.790	18.038
8	1.0534	12.081	8.479	9.877	11.288	13.963
8	1.0544	8.718	9.114	7.647	8.545	3.781
8	1.0554	13.287	8.228	9.409	15.140	12.510
8	1.0564	14.692	12.958	8.730	12.144	12.926
8	1.0574	20.735	9.174	12.256	17.384	30.710
8	1.0584	10.894	11.888	10.472	7.602	20.455
8	1.0594	12.925	9.312	8.272	14.936	26.525
8	1.0604	19.805	9.234	12.586	20.284	23.206
8	1.0614	24.535	13.610	15.547	23.665	34.530
8	1.0624	22.660	14.311	17.243	20.672	19.367
8	1.0634	17.575	12.843	14.160	18.198	13.091
8	1.0644	12.292	12.242	13.530	12.268	18.254
8	1.0654	16.509	11.421	16.753	21.538	19.282
8	1.0664	26.907	18.160	19.815	13.660	25.093
8	1.0674	21.934	14.182	19.865	23.569	24.267
8	1.0684	11.582	15.725	16.644	21.845	25.485
8	1.0694	17.972	16.058	20.216	25.191	26.270
8	1.0704	13.289	9.333	12.502	28.552	31.094
8	1.0714	20.403	19.102	18.674	15.641	31.666
8	1.0724	37.053	24.581	30.949	12.441	34.070
8	1.0734	25.133	14.744	23.021	12.931	22.345
8	1.0744	23.871	19.637	21.584	18.144	13.845
8	1.0754	27.291	19.679	19.505	12.823	20.506
8	1.0764	25.722	21.449	14.652	20.954	22.432
8	1.0774	14.834	15.558	17.243	29.062	13.724
8	1.0784	13.750	12.768	12.604	31.380	23.396
8	1.0794	15.665	14.158	11.940	16.821	25.281
8	1.0804	15.713	7.340	12.104	12.355	20.005
8	1.0814	16.251	5.867	10.337	12.218	26.081
8	1.0824	9.782	12.768	8.413	12.436	19.381
8	1.0834	9.486	6.075	4.014	18.056	20.504
8	1.0844	16.430	14.667	17.759	9.657	18.244
8	1.0854	8.420	13.832	9.928	12.006	15.812
8	1.0864	18.152	12.720	11.201	23.774	23.672
8	1.0874	13.135	9.912	6.300	18.372	17.530
8	1.0884	12.784	11.147	10.059	22.967	23.980
8	1.0894	16.779	10.305	9.243	7.469	22.531
8	1.0904	13.685	15.513	9.661	29.135	29.473
8	1.0914	13.085	10.861	7.844	7.446	18.471
8	1.0924	11.509	10.151	5.562	11.698	19.630
8	1.0934	18.288	26.580	17.402	18.472	16.057
8	1.0944	18.535	26.544	17.676	18.496	16.001

8	1.0954	10.113	15.888	9.256	18.451	12.304
8	1.0964	10.550	11.735	8.936	11.168	21.223
8	1.0974	14.766	12.695	5.960	19.003	28.026
8	1.0984	17.147	20.182	12.982	22.237	22.350
8	1.0994	18.321	14.313	18.995	17.531	18.495
8	1.1004	15.711	14.836	16.105	20.632	23.358
8	1.1014	15.801	14.881	16.192	20.617	23.156
8	1.1024	17.794	16.648	18.213	16.158	9.954
8	1.1034	15.742	16.206	14.649	23.479	5.402
8	1.1044	9.529	13.595	10.252	16.788	9.966
8	1.1054	30.090	25.418	23.672	27.008	13.604
8	1.1064	20.586	14.491	18.090	10.354	2.033
8	1.1074	4.698	7.923	6.725	6.818	3.302
8	1.1084	8.707	5.839	8.306	7.511	0.807
8	1.1094	0.000	0.000	0.000	0.000	0.000
9	0.0005	0.000	0.000	0.000	0.000	0.000
9	0.0015	0.000	0.000	0.000	0.000	0.000
9	0.0025	14.736	11.593	12.195	21.288	23.003
9	0.0035	19.380	15.285	16.570	18.426	17.328
9	0.0045	9.806	10.674	8.082	27.227	22.310
9	0.0055	9.917	13.114	7.995	29.641	14.600
9	0.0065	11.500	5.150	11.443	11.834	6.382
9	0.0075	14.616	17.396	11.300	34.032	18.174
9	0.0085	12.166	8.452	8.925	9.277	9.786
9	0.0095	7.136	6.431	5.499	20.117	7.051
9	0.0105	7.128	6.520	5.585	19.932	7.083
9	0.0115	10.948	8.946	11.255	23.624	8.418
9	0.0125	14.128	11.498	13.200	12.014	19.525
9	0.0135	10.201	7.227	6.925	7.448	12.140
9	0.0145	8.810	8.528	10.325	12.404	11.887
9	0.0155	9.904	10.593	9.511	26.621	25.344
9	0.0165	11.831	8.302	9.594	14.675	13.347
9	0.0175	8.846	8.271	8.842	22.356	21.096
9	0.0185	12.923	15.017	9.583	26.427	20.235
9	0.0195	14.489	12.114	14.552	25.157	24.950
9	0.0205	19.161	11.367	11.075	19.367	11.137
9	0.0215	15.565	19.006	11.595	20.684	10.194
9	0.0225	16.647	17.649	18.312	19.527	10.970
9	0.0235	22.453	18.833	22.173	22.099	29.197
9	0.0245	13.780	14.091	16.414	22.634	13.373
9	0.0255	13.058	12.209	13.532	10.022	7.105
9	0.0265	16.063	13.718	15.116	8.492	12.377
9	0.0275	18.702	11.771	16.240	17.743	16.895
9	0.0285	10.565	12.990	11.348	14.101	18.058
9	0.0295	12.380	24.004	15.248	21.963	25.508
9	0.0305	14.274	16.261	16.937	15.951	18.338
9	0.0315	15.932	21.421	19.777	10.638	11.860
9	0.0325	11.369	10.871	11.699	7.826	12.913
9	0.0335	22.793	37.760	23.196	21.951	14.969
9	0.0345	29.919	43.292	26.725	19.894	18.343

9	0.0355	16.323	23.394	13.215	18.559	16.700
9	0.0365	15.719	21.864	16.234	22.691	28.356
9	0.0375	20.023	22.923	22.447	8.702	19.502
9	0.0385	17.925	17.916	17.120	13.688	23.597
9	0.0395	20.787	20.019	17.632	18.290	14.006
9	0.0405	28.921	18.334	18.323	24.930	16.088
9	0.0415	17.414	16.548	16.497	17.637	7.939
9	0.0425	26.587	16.815	18.731	11.938	19.314
9	0.0435	24.282	18.771	21.408	16.411	20.581
9	0.0445	19.060	16.097	20.348	19.134	15.840
9	0.0455	15.703	11.966	13.641	3.092	17.125
9	0.0465	13.466	15.800	17.860	3.578	8.682
9	0.0475	15.390	13.950	20.537	10.790	24.564
9	0.0485	23.724	15.359	21.972	10.370	18.357
9	0.0495	23.305	12.598	26.035	18.181	23.760
9	0.0505	28.214	21.605	21.732	13.316	12.184
9	0.0515	26.302	21.861	25.988	10.962	23.097
9	0.0525	22.945	28.667	18.725	7.970	18.236
9	0.0535	17.847	18.479	21.834	12.824	19.335
9	0.0545	12.833	10.806	11.937	21.477	23.535
9	0.0555	19.605	15.290	19.511	11.300	17.345
9	0.0565	13.521	7.820	11.681	4.370	3.600
9	0.0575	10.154	6.240	9.293	8.070	7.188
9	0.0585	21.033	17.755	17.103	17.140	12.313
9	0.0595	29.807	16.507	20.936	16.209	25.686
9	0.0605	13.536	8.017	10.465	12.777	14.095
9	0.0615	17.229	14.117	11.937	13.682	26.432
9	0.0625	20.921	13.376	15.199	13.830	23.881
9	0.0635	18.025	8.225	11.603	12.508	10.712
9	0.0645	10.630	11.115	8.499	17.491	12.397
9	0.0655	19.283	17.049	18.375	6.887	20.625
9	0.0665	13.977	23.248	17.079	18.026	11.044
9	0.0675	14.241	23.299	20.092	17.735	14.500
9	0.0685	11.507	19.097	12.934	12.080	11.970
9	0.0695	14.264	11.840	10.451	9.221	13.224
9	0.0705	24.266	20.384	15.712	6.303	6.640
9	0.0715	20.703	10.556	12.555	7.116	11.957
9	0.0725	20.673	10.502	12.534	7.116	11.975
9	0.0735	33.742	27.667	22.539	19.402	22.223
9	0.0745	18.978	15.525	14.199	16.568	18.745
9	0.0755	24.484	11.402	17.296	20.806	21.083
9	0.0765	18.327	11.819	14.924	15.550	15.560
9	0.0775	29.419	26.099	26.555	11.051	12.795
9	0.0785	31.210	23.191	22.692	11.541	19.360
9	0.0795	21.312	18.301	17.432	6.694	20.194
9	0.0805	22.178	12.640	13.886	9.777	14.122
9	0.0815	26.699	18.325	20.924	10.814	13.090
9	0.0825	25.207	19.957	18.905	7.969	9.961
9	0.0835	24.125	23.229	26.256	30.875	17.091
9	0.0845	26.508	24.962	28.271	16.413	15.716

9	0.0855	36.504	26.008	29.211	15.099	24.869
9	0.0865	29.062	16.939	18.583	21.434	22.648
9	0.0875	31.997	17.594	20.049	16.077	21.254
9	0.0885	8.656	7.706	11.424	12.910	12.497
9	0.0895	13.622	7.712	17.167	9.224	6.694
9	0.0905	18.527	16.750	14.309	6.296	17.125
9	0.0915	18.658	14.095	16.673	5.494	14.377
9	0.0925	27.199	15.007	23.634	13.702	5.974
9	0.0935	10.764	10.496	12.806	20.263	9.495
9	0.0945	26.493	21.241	24.281	18.048	18.072
9	0.0955	14.634	18.830	12.855	25.275	7.300
9	0.0965	26.361	25.758	14.935	16.083	10.239
9	0.0975	12.344	6.065	11.193	12.602	10.369
9	0.0985	11.979	12.059	10.111	15.855	12.192
9	0.0995	9.930	9.424	7.774	9.055	8.394
9	0.1005	21.416	15.261	22.283	20.025	14.264
9	0.1015	17.834	10.266	16.747	16.620	9.170
9	0.1025	20.028	8.262	17.211	28.720	10.995
9	0.1035	12.583	16.933	11.153	14.599	6.114
9	0.1045	29.304	25.789	25.267	12.403	13.321
9	0.1055	24.764	16.451	18.374	23.937	21.359
9	0.1065	19.336	6.880	12.049	23.163	18.006
9	0.1075	12.471	12.217	6.380	16.907	23.719
9	0.1085	15.770	18.934	14.522	23.676	17.909
9	0.1095	19.250	14.236	23.980	14.272	8.236
9	0.1105	11.816	11.862	14.567	15.180	6.681
9	0.1115	15.846	10.854	13.760	15.847	14.220
9	0.1125	19.905	7.238	10.947	29.119	21.559
9	0.1135	13.689	10.664	13.293	15.023	23.504
9	0.1145	5.577	3.408	8.734	3.426	12.195
9	0.1155	17.204	15.213	17.886	9.193	15.972
9	0.1165	11.892	7.638	16.659	7.674	6.163
9	0.1175	17.197	19.524	20.413	15.765	21.914
9	0.1185	12.275	10.883	15.070	16.398	17.075
9	0.1195	11.629	14.210	10.095	15.081	10.335
9	0.1205	10.294	14.302	12.171	12.099	12.423
9	0.1215	15.287	11.548	16.492	14.695	9.758
9	0.1225	10.476	12.548	14.259	6.750	19.637
9	0.1235	16.357	20.954	19.184	17.000	17.107
9	0.1245	16.458	11.992	12.070	19.970	26.528
9	0.1255	18.513	15.735	14.425	14.782	16.153
9	0.1265	8.002	11.895	12.186	12.038	10.265
9	0.1275	6.448	8.303	5.704	18.086	11.752
9	0.1285	18.506	12.412	17.634	10.653	16.441
9	0.1295	22.580	17.589	17.120	12.397	12.839
9	0.1305	36.777	23.086	26.302	6.286	12.422
9	0.1315	17.171	15.275	16.622	13.616	18.732
9	0.1325	11.074	11.278	16.493	13.092	25.843
9	0.1335	9.774	8.700	13.124	10.434	5.755
9	0.1345	7.191	10.797	9.116	9.158	7.661

9	0.1355	22.594	12.297	12.825	13.735	14.049
9	0.1365	23.779	11.943	21.935	9.857	19.768
9	0.1375	30.585	18.299	30.003	10.038	11.011
9	0.1385	40.400	19.460	23.848	13.860	19.937
9	0.1395	20.675	19.215	20.002	18.158	35.418
9	0.1405	15.201	11.018	9.913	11.473	25.363
9	0.1415	9.245	11.110	7.435	13.579	26.141
9	0.1425	35.950	22.042	26.809	16.416	24.175
9	0.1435	15.482	10.944	13.858	10.905	18.907
9	0.1445	23.033	16.768	19.885	18.818	15.618
9	0.1455	33.064	18.246	27.706	21.653	19.359
9	0.1465	18.982	20.742	25.087	24.838	14.762
9	0.1475	14.642	10.845	9.474	21.281	17.386
9	0.1485	19.496	11.074	10.456	25.376	22.616
9	0.1495	19.619	8.176	12.357	23.333	16.568
9	0.1505	17.051	7.735	15.274	22.112	14.710
9	0.1515	13.492	5.724	8.228	10.669	10.396
9	0.1525	22.248	16.572	13.428	14.044	10.327
9	0.1535	16.989	12.735	20.431	11.469	7.423
9	0.1545	15.078	11.773	23.582	14.755	14.024
9	0.1555	17.423	11.809	17.979	12.585	17.869
9	0.1565	16.349	13.794	19.837	14.824	11.654
9	0.1575	16.325	6.671	12.143	15.730	11.120
9	0.1585	12.449	7.825	17.408	7.458	7.764
9	0.1595	5.433	4.955	11.269	12.586	12.756
9	0.1605	19.833	14.447	20.813	8.582	8.689
9	0.1615	14.609	14.065	13.914	12.943	9.979
9	0.1625	13.277	7.584	9.423	12.303	10.323
9	0.1635	13.654	6.625	10.030	10.792	13.103
9	0.1645	20.737	8.252	15.528	12.791	11.460
9	0.1655	21.566	10.483	17.003	11.517	8.795
9	0.1665	15.201	14.203	19.725	12.348	15.590
9	0.1675	11.512	6.979	8.732	16.151	16.075
9	0.1685	7.181	6.539	5.062	13.870	19.100
9	0.1695	13.071	8.992	12.579	8.605	5.694
9	0.1705	17.189	11.911	15.666	12.505	7.268
9	0.1715	20.008	12.020	17.705	6.896	5.120
9	0.1725	18.972	16.188	24.499	6.823	10.994
9	0.1735	16.238	15.381	20.307	7.927	10.813
9	0.1745	13.268	6.558	13.189	7.091	8.728
9	0.1755	19.766	7.533	12.853	8.921	4.062
9	0.1765	7.145	18.617	9.041	19.022	15.200
9	0.1775	22.510	21.579	18.770	12.725	9.969
9	0.1785	10.903	12.051	12.748	17.886	12.200
9	0.1795	16.033	17.667	15.810	9.792	7.693
9	0.1805	15.774	16.441	19.125	15.379	12.691
9	0.1815	10.951	9.074	12.236	9.329	13.698
9	0.1825	12.603	14.103	20.048	14.134	21.181
9	0.1835	9.580	15.820	9.783	12.367	11.501
9	0.1845	13.610	14.879	16.106	7.733	6.780

9	0.1855	19.001	12.433	20.279	18.789	12.934
9	0.1865	29.716	27.699	24.219	15.813	7.143
9	0.1875	15.349	15.959	14.072	8.538	9.222
9	0.1885	35.675	20.409	27.877	11.619	8.446
9	0.1895	6.713	9.054	7.953	6.097	6.206
9	0.1905	31.070	28.117	26.729	19.940	10.950
9	0.1915	12.454	7.068	8.514	12.996	10.318
9	0.1925	15.602	6.589	11.300	6.189	3.757
9	0.1935	16.785	18.535	18.221	12.156	7.911
9	0.1945	15.041	22.482	21.649	16.281	10.399
9	0.1955	5.997	8.724	7.568	10.248	7.940
9	0.1965	5.557	6.726	8.741	8.232	10.643
9	0.1975	17.582	12.746	13.727	16.680	13.943
9	0.1985	26.363	14.594	25.156	19.451	17.781
9	0.1995	39.993	21.604	34.727	12.943	16.028
9	0.2005	17.545	17.960	17.239	10.900	11.755
9	0.2015	12.846	15.094	11.574	10.899	13.817
9	0.2025	9.855	8.629	6.635	10.743	7.843
9	0.2035	9.864	8.899	6.697	10.859	7.818
9	0.2045	6.799	5.398	4.448	4.062	4.917
9	0.2055	6.151	2.507	3.172	9.429	12.198
9	0.2065	9.461	5.022	5.705	5.951	17.879
9	0.2075	10.575	6.528	10.016	5.708	7.393
9	0.2085	14.031	11.515	15.331	8.394	5.410
9	0.2095	9.895	9.458	11.103	7.884	7.809
9	0.2105	15.445	9.894	16.145	17.243	13.180
9	0.2115	18.329	11.141	13.895	8.002	16.868
9	0.2125	9.562	5.171	10.516	6.579	8.422
9	0.2135	22.028	14.459	14.289	11.292	12.812
9	0.2145	15.898	17.965	16.840	11.738	12.308
9	0.2155	16.221	12.629	18.408	10.046	12.158
9	0.2165	13.226	5.807	8.686	19.229	9.203
9	0.2175	7.161	7.356	6.497	7.716	10.560
9	0.2185	9.316	11.737	11.858	14.018	9.576
9	0.2195	21.127	13.460	21.834	13.986	17.576
9	0.2205	16.205	8.015	12.445	6.310	14.074
9	0.2215	18.376	15.351	26.003	10.516	5.983
9	0.2225	11.935	11.816	12.752	14.374	16.681
9	0.2235	17.554	16.415	22.377	12.431	11.091
9	0.2245	14.638	10.743	14.297	8.865	5.405
9	0.2255	20.531	9.917	20.347	8.178	12.115
9	0.2265	25.950	16.668	21.454	11.147	14.343
9	0.2275	20.169	16.929	26.426	15.095	9.364
9	0.2285	19.169	9.655	14.482	12.833	9.647
9	0.2295	14.425	8.052	14.975	4.224	6.982
9	0.2305	7.261	3.607	5.830	6.194	7.301
9	0.2315	12.416	7.132	9.434	8.071	8.894
9	0.2325	35.460	20.015	27.850	6.253	16.234
9	0.2335	25.050	9.866	11.163	13.402	17.205
9	0.2345	16.564	13.181	17.608	10.730	18.907

9	0.2355	13.589	10.514	14.364	12.764	9.528
9	0.2365	21.506	15.505	19.770	15.077	10.955
9	0.2375	16.144	17.331	12.373	10.876	11.027
9	0.2385	19.523	14.569	12.257	15.246	26.309
9	0.2395	16.199	6.764	6.978	24.068	26.476
9	0.2405	32.061	18.168	21.178	17.053	21.298
9	0.2415	13.160	14.725	9.080	17.469	22.515
9	0.2425	17.062	13.033	14.736	13.116	10.613
9	0.2435	21.545	16.189	21.557	6.169	14.318
9	0.2445	10.899	11.200	9.919	14.750	15.006
9	0.2455	21.650	24.811	20.805	16.862	20.115
9	0.2465	14.115	6.216	8.788	18.616	26.649
9	0.2475	19.076	10.350	10.750	10.829	30.793
9	0.2485	16.113	11.410	9.158	11.843	24.830
9	0.2495	25.254	18.719	23.356	5.980	14.536
9	0.2505	27.080	13.409	19.568	9.018	22.848
9	0.2515	24.269	10.440	18.667	18.104	29.949
9	0.2525	22.499	13.737	17.843	18.951	22.529
9	0.2535	17.526	11.301	15.252	12.564	20.659
9	0.2545	11.799	5.983	6.757	10.896	8.886
9	0.2555	11.271	9.699	7.724	8.407	7.045
9	0.2565	16.774	15.830	24.761	14.433	16.772
9	0.2575	18.949	16.504	28.691	11.803	17.546
9	0.2585	17.439	18.125	19.386	13.665	17.932
9	0.2595	9.144	10.707	3.528	15.765	23.925
9	0.2605	30.736	14.920	18.487	7.138	9.301
9	0.2615	31.031	17.700	26.727	14.543	17.713
9	0.2625	22.440	12.112	25.345	14.660	8.686
9	0.2635	10.277	6.868	10.196	5.674	9.935
9	0.2645	15.335	16.462	16.829	18.832	10.665
9	0.2655	13.935	11.849	11.685	11.736	10.093
9	0.2665	19.218	10.607	8.680	11.938	18.941
9	0.2675	17.297	11.664	10.136	13.483	18.142
9	0.2685	23.904	13.381	22.078	9.072	9.719
9	0.2695	12.619	11.829	18.093	9.618	23.710
9	0.2705	5.265	3.821	6.782	13.355	14.947
9	0.2715	6.768	7.401	4.190	18.863	15.704
9	0.2725	2.752	7.894	4.596	12.636	14.237
9	0.2735	3.510	5.403	8.218	4.714	19.059
9	0.2745	7.670	7.416	6.136	4.984	19.625
9	0.2755	11.729	13.823	14.896	8.438	14.831
9	0.2765	6.085	10.333	8.965	4.237	6.243
9	0.2775	38.205	20.948	27.076	5.213	6.445
9	0.2785	10.990	5.951	8.113	5.360	4.443
9	0.2795	7.218	4.392	4.169	8.785	7.294
9	0.2805	5.957	3.693	3.124	9.528	6.547
9	0.2815	2.989	1.747	2.126	6.943	8.409
9	0.2825	9.703	8.461	9.780	5.889	9.875
9	0.2835	8.636	8.183	10.456	6.014	10.511
9	0.2845	21.228	12.741	15.832	9.613	14.680

9	0.2855	8.905	6.289	7.995	1.065	4.456
9	0.2865	8.563	5.317	6.164	1.543	3.129
9	0.2875	5.034	2.837	4.800	2.833	7.690
9	0.2885	12.300	13.613	18.061	6.143	11.057
9	0.2895	12.866	4.802	11.337	6.795	11.258
9	0.2905	10.064	9.778	9.213	6.689	9.889
9	0.2915	10.066	9.780	9.215	6.690	9.891
9	0.2925	12.132	4.511	8.270	3.100	12.572
9	0.2935	16.766	11.221	15.937	5.035	11.971
9	0.2945	22.286	12.761	18.137	9.369	13.543
9	0.2955	12.917	10.750	13.084	15.717	11.881
9	0.2965	34.191	26.137	24.985	18.273	11.476
9	0.2975	22.620	16.368	15.541	13.923	9.307
9	0.2985	15.122	11.340	9.409	5.782	15.094
9	0.2995	24.507	16.833	19.591	11.942	21.463
9	0.3005	7.629	6.506	8.375	3.380	14.593
9	0.3015	24.198	18.069	20.597	6.857	18.398
9	0.3025	40.621	18.569	26.478	7.471	8.739
9	0.3035	29.610	11.623	20.395	7.545	12.189
9	0.3045	20.106	6.944	9.083	4.829	22.618
9	0.3055	28.638	18.343	21.454	7.509	12.550
9	0.3065	25.892	20.934	30.252	11.723	19.316
9	0.3075	37.300	20.746	27.406	13.861	28.563
9	0.3085	38.047	18.920	27.263	10.421	20.882
9	0.3095	11.783	13.177	14.635	15.006	17.068
9	0.3105	14.216	10.900	11.289	10.615	6.948
9	0.3115	15.563	11.022	11.244	7.097	15.544
9	0.3125	12.943	6.275	7.518	8.861	11.737
9	0.3135	26.655	18.255	17.950	5.960	12.739
9	0.3145	19.315	9.398	12.409	4.069	9.260
9	0.3155	7.847	8.812	6.676	5.327	7.715
9	0.3165	8.792	6.618	5.452	11.883	21.877
9	0.3175	8.737	8.089	6.746	12.042	12.326
9	0.3185	11.373	6.121	4.702	3.062	11.583
9	0.3195	20.954	13.655	11.320	12.276	21.155
9	0.3205	20.823	11.606	9.439	7.536	11.135
9	0.3215	24.934	12.373	13.712	7.146	13.508
9	0.3225	11.765	8.114	10.123	7.578	12.536
9	0.3235	11.387	7.120	8.370	5.857	11.993
9	0.3245	16.822	12.267	12.760	14.182	21.636
9	0.3255	15.030	9.294	10.331	7.376	10.294
9	0.3265	10.016	11.786	10.181	18.548	11.128
9	0.3275	9.786	11.734	10.081	18.528	11.099
9	0.3285	12.181	11.526	11.950	11.293	10.688
9	0.3295	14.330	16.224	16.353	12.773	12.596
9	0.3305	23.508	24.967	22.382	24.051	16.841
9	0.3315	17.716	19.674	18.539	14.744	12.391
9	0.3325	23.222	18.151	18.517	12.544	5.534
9	0.3335	27.338	16.875	24.335	7.179	16.232
9	0.3345	20.218	16.491	19.120	10.333	23.330

9	0.3355	7.577	7.490	5.459	8.787	28.254
9	0.3365	14.691	10.573	9.268	11.573	17.893
9	0.3375	20.816	13.029	17.171	9.970	15.734
9	0.3385	18.012	10.444	14.909	17.565	15.433
9	0.3395	15.193	9.293	12.659	14.491	11.555
9	0.3405	21.761	13.889	12.612	10.399	13.326
9	0.3415	14.812	9.836	11.894	6.548	21.890
9	0.3425	16.804	13.304	12.842	23.073	16.437
9	0.3435	26.443	16.930	13.251	5.929	21.368
9	0.3445	32.058	25.016	27.184	18.840	25.957
9	0.3455	9.045	5.625	8.322	7.707	14.408
9	0.3465	9.010	5.625	8.298	7.716	14.439
9	0.3475	10.033	12.255	11.859	7.421	21.860
9	0.3485	19.561	18.279	17.436	8.186	10.096
9	0.3495	13.210	16.956	11.311	15.118	9.697
9	0.3505	10.366	10.092	12.208	9.553	18.061
9	0.3515	17.172	13.432	12.401	15.976	24.305
9	0.3525	18.362	22.327	24.180	13.549	17.800
9	0.3535	14.222	12.591	13.571	13.680	10.103
9	0.3545	13.329	9.757	13.324	7.663	9.421
9	0.3555	11.915	11.878	8.005	14.628	16.128
9	0.3565	23.112	14.861	13.242	13.815	12.843
9	0.3575	15.638	16.005	12.790	10.626	9.912
9	0.3585	12.436	13.607	13.220	6.379	4.674
9	0.3595	7.233	4.993	5.370	9.598	9.021
9	0.3605	24.365	19.687	19.833	9.061	4.650
9	0.3615	15.379	13.937	19.543	17.837	7.314
9	0.3625	9.849	16.007	18.174	23.720	11.366
9	0.3635	15.989	15.240	20.194	26.402	11.773
9	0.3645	16.339	12.094	20.260	19.358	5.155
9	0.3655	16.542	14.987	12.490	11.385	6.092
9	0.3665	26.001	12.895	19.996	16.760	22.698
9	0.3675	24.573	13.978	24.270	6.027	11.951
9	0.3685	24.305	7.467	22.069	16.232	10.238
9	0.3695	9.629	7.646	8.586	13.477	12.095
9	0.3705	10.922	3.567	4.405	8.724	16.677
9	0.3715	28.541	14.867	18.370	8.422	12.682
9	0.3725	23.537	20.294	19.850	14.790	15.297
9	0.3735	18.337	12.493	16.545	7.987	10.246
9	0.3745	18.857	20.508	14.248	19.339	10.362
9	0.3755	21.082	14.661	16.031	15.441	10.085
9	0.3765	12.296	7.465	5.613	17.336	11.656
9	0.3775	27.156	19.166	20.521	15.734	20.043
9	0.3785	30.371	23.645	24.253	10.118	18.818
9	0.3795	20.817	18.316	14.768	11.382	15.953
9	0.3805	14.643	13.706	11.652	6.101	16.652
9	0.3815	19.791	18.214	17.041	10.450	10.021
9	0.3825	14.668	10.247	12.512	7.890	19.933
9	0.3835	11.635	11.062	11.925	16.787	14.947
9	0.3845	4.522	7.592	4.484	8.397	6.765

9	0.3855	19.886	17.143	18.194	12.499	10.547
9	0.3865	6.723	4.256	10.026	9.847	8.332
9	0.3875	6.545	13.273	10.620	11.046	6.358
9	0.3885	8.992	11.934	7.071	12.681	10.950
9	0.3895	9.804	6.002	9.262	4.128	6.747
9	0.3905	24.389	16.584	25.012	5.313	14.012
9	0.3915	13.944	9.668	13.843	7.936	5.990
9	0.3925	7.452	5.689	5.498	7.831	2.727
9	0.3935	14.454	18.639	12.784	10.040	4.851
9	0.3945	7.727	3.726	7.820	8.652	3.144
9	0.3955	13.921	11.801	9.309	15.244	6.632
9	0.3965	10.239	6.457	3.895	12.334	10.533
9	0.3975	14.437	10.170	9.896	7.709	12.601
9	0.3985	15.241	10.521	14.601	6.802	10.003
9	0.3995	13.545	9.625	10.806	7.876	7.075
9	0.4005	15.823	10.822	17.390	6.153	6.516
9	0.4015	10.084	6.782	10.087	9.192	15.875
9	0.4025	12.847	6.161	6.098	7.213	10.036
9	0.4035	13.835	12.261	15.461	6.023	13.736
9	0.4045	6.486	6.996	7.936	6.446	12.163
9	0.4055	20.012	19.004	21.124	6.862	5.213
9	0.4065	14.337	18.628	16.234	6.921	11.062
9	0.4075	11.389	7.003	7.838	4.641	11.754
9	0.4085	16.609	7.815	12.491	7.426	10.364
9	0.4095	19.428	17.392	15.011	10.835	7.959
9	0.4105	19.417	20.166	16.568	11.293	12.253
9	0.4115	7.460	6.654	9.892	7.257	9.527
9	0.4125	8.747	8.046	6.644	6.139	6.575
9	0.4135	14.798	12.535	12.403	4.887	6.583
9	0.4145	18.770	18.949	14.786	9.516	8.234
9	0.4155	21.097	16.109	14.654	6.023	11.093
9	0.4165	13.628	14.481	9.778	14.593	8.001
9	0.4175	14.010	12.945	15.002	5.477	13.958
9	0.4185	12.002	8.291	8.837	4.746	8.064
9	0.4195	23.834	18.874	18.005	11.267	9.288
9	0.4205	14.536	16.076	15.921	5.135	11.555
9	0.4215	17.949	25.394	20.260	10.597	7.971
9	0.4225	17.304	15.538	14.123	4.543	8.024
9	0.4235	16.206	26.597	16.669	10.894	10.780
9	0.4245	12.742	13.692	11.396	3.444	2.373
9	0.4255	12.886	13.071	11.569	4.420	11.159
9	0.4265	5.283	5.333	4.597	6.384	10.741
9	0.4275	10.256	12.261	10.470	9.986	4.136
9	0.4285	10.858	7.500	9.762	4.332	6.081
9	0.4295	9.915	7.243	12.421	2.904	6.380
9	0.4305	15.104	15.363	14.649	3.447	8.685
9	0.4315	22.305	21.979	22.127	6.302	8.238
9	0.4325	21.585	20.568	22.537	5.157	5.882
9	0.4335	15.161	17.113	16.715	6.041	7.958
9	0.4345	18.502	18.355	18.773	13.127	5.247

9	0.4355	16.877	20.697	15.992	14.598	7.432
9	0.4365	17.844	20.849	17.058	13.137	7.571
9	0.4375	16.230	18.116	17.217	12.563	10.454
9	0.4385	18.833	20.278	14.689	15.591	5.845
9	0.4395	16.042	14.021	17.455	10.040	6.881
9	0.4405	18.177	16.639	19.721	7.251	12.613
9	0.4415	14.064	15.471	12.410	18.628	11.333
9	0.4425	17.369	18.009	17.695	16.191	13.600
9	0.4435	25.265	22.350	24.072	8.264	6.612
9	0.4445	16.541	16.912	17.516	10.870	14.901
9	0.4455	16.962	22.795	21.482	12.725	7.650
9	0.4465	19.653	17.711	23.100	12.359	7.990
9	0.4475	19.647	17.701	23.077	12.366	8.003
9	0.4485	12.035	13.222	9.528	5.998	8.578
9	0.4495	11.344	12.728	12.696	4.358	11.250
9	0.4505	10.606	11.352	11.189	11.234	10.161
9	0.4515	15.690	22.479	15.680	10.820	10.511
9	0.4525	14.750	17.780	11.890	14.088	13.060
9	0.4535	12.185	14.131	8.866	9.003	13.813
9	0.4545	18.378	23.351	19.188	16.715	9.580
9	0.4555	8.447	10.649	12.864	9.989	11.339
9	0.4565	5.542	5.860	8.291	8.510	10.052
9	0.4575	11.939	14.101	8.802	13.315	10.031
9	0.4585	10.301	10.983	7.510	8.526	19.502
9	0.4595	11.938	15.247	9.241	10.289	18.213
9	0.4605	21.956	19.655	20.225	12.934	15.881
9	0.4615	20.015	21.947	15.278	13.586	11.553
9	0.4625	17.446	22.626	19.186	21.530	8.785
9	0.4635	21.041	27.286	19.995	24.141	16.178
9	0.4645	13.964	16.744	15.625	16.566	7.836
9	0.4655	19.349	17.833	19.757	11.221	17.069
9	0.4665	8.938	17.444	13.030	14.183	14.013
9	0.4675	14.128	13.684	11.304	20.845	8.038
9	0.4685	12.333	10.075	7.612	16.999	17.178
9	0.4695	21.569	24.410	18.499	17.422	12.167
9	0.4705	12.411	20.527	14.072	18.562	14.490
9	0.4715	8.506	14.581	13.144	22.309	11.149
9	0.4725	3.426	6.207	10.192	14.291	5.122
9	0.4735	6.308	11.911	12.446	9.181	14.277
9	0.4745	17.324	15.771	21.976	10.162	14.983
9	0.4755	18.792	14.765	19.316	9.016	6.515
9	0.4765	13.017	16.619	21.380	8.873	13.724
9	0.4775	6.746	10.240	10.248	11.343	14.468
9	0.4785	10.609	8.548	13.705	10.701	11.954
9	0.4795	6.005	2.713	2.122	9.337	14.200
9	0.4805	3.746	1.674	1.513	8.169	10.845
9	0.4815	5.748	5.041	5.467	7.865	15.761
9	0.4825	9.893	17.563	13.636	24.751	7.682
9	0.4835	10.675	13.434	11.328	13.970	13.088
9	0.4845	13.068	10.831	10.983	13.618	11.332

9	0.4855	13.487	10.847	15.280	11.919	8.008
9	0.4865	22.343	20.067	28.183	11.910	12.472
9	0.4875	16.669	19.323	17.558	15.380	15.474
9	0.4885	7.504	7.787	8.975	11.972	12.218
9	0.4895	4.991	10.218	5.721	7.591	8.185
9	0.4905	5.001	10.223	5.732	7.594	8.196
9	0.4915	5.540	10.622	5.316	16.139	5.498
9	0.4925	14.159	16.591	19.872	16.690	22.409
9	0.4935	18.517	20.818	25.306	22.048	18.690
9	0.4945	18.505	20.811	25.311	22.049	18.701
9	0.4955	20.051	14.478	16.569	25.951	17.321
9	0.4965	19.089	20.934	16.484	28.927	17.245
9	0.4975	10.209	13.198	16.701	17.481	11.358
9	0.4985	18.542	20.212	24.590	17.831	15.333
9	0.4995	22.672	16.389	24.843	20.283	13.836
9	0.5005	16.520	11.069	17.327	6.175	22.974
9	0.5015	10.343	11.243	13.058	15.978	19.623
9	0.5025	16.278	15.415	18.938	11.122	20.110
9	0.5035	16.253	15.423	19.076	11.165	20.052
9	0.5045	8.814	8.510	14.550	11.421	19.678
9	0.5055	10.944	5.140	11.467	8.982	19.680
9	0.5065	2.353	5.338	3.351	7.771	5.965
9	0.5075	10.273	16.049	11.764	13.422	8.606
9	0.5085	18.073	24.442	22.463	16.706	15.674
9	0.5095	16.535	17.934	19.109	6.613	13.232
9	0.5105	15.019	14.177	19.364	7.428	14.628
9	0.5115	12.052	12.445	13.275	9.943	19.532
9	0.5125	15.579	20.103	18.913	16.458	27.501
9	0.5135	20.172	14.151	13.298	6.544	24.622
9	0.5145	8.317	13.692	8.749	9.459	18.302
9	0.5155	9.395	10.523	10.378	10.330	8.395
9	0.5165	10.003	16.993	16.401	9.302	13.447
9	0.5175	8.912	5.291	6.633	14.291	14.531
9	0.5185	19.117	14.922	12.128	15.382	14.247
9	0.5195	14.112	8.805	15.389	7.810	10.198
9	0.5205	13.530	13.045	14.532	13.243	7.775
9	0.5215	10.309	10.917	12.819	12.987	12.343
9	0.5225	8.631	13.410	16.592	10.024	13.774
9	0.5235	4.604	14.327	11.245	15.110	17.243
9	0.5245	4.602	7.937	4.343	10.136	16.232
9	0.5255	7.338	6.878	5.590	9.738	15.391
9	0.5265	16.002	18.164	13.648	11.849	20.456
9	0.5275	28.760	28.805	24.996	6.014	18.415
9	0.5285	7.023	7.027	4.682	4.781	16.605
9	0.5295	6.702	10.368	14.445	10.747	16.643
9	0.5305	7.903	10.443	11.546	7.125	11.768
9	0.5315	14.893	9.767	13.011	2.384	8.111
9	0.5325	13.021	11.702	13.894	6.456	22.270
9	0.5335	11.852	11.737	12.582	6.464	11.404
9	0.5345	18.867	26.359	24.214	12.940	19.897

9	0.5355	17.991	16.996	22.530	8.006	13.494
9	0.5365	14.069	14.345	13.220	9.309	17.537
9	0.5375	18.346	14.351	13.124	5.374	12.567
9	0.5385	9.857	10.123	8.360	9.778	11.711
9	0.5395	13.803	15.770	16.424	10.218	9.234
9	0.5405	5.797	9.283	14.812	10.884	15.487
9	0.5415	18.135	18.554	14.674	5.564	16.719
9	0.5425	11.073	12.090	12.306	6.891	17.630
9	0.5435	15.623	20.124	20.116	7.189	14.603
9	0.5445	8.114	11.526	4.882	9.711	13.156
9	0.5455	16.117	11.320	15.320	13.258	20.628
9	0.5465	9.390	12.408	10.137	6.590	22.088
9	0.5475	12.453	10.859	13.184	4.385	11.841
9	0.5485	14.826	10.592	12.649	7.613	16.042
9	0.5495	13.578	12.790	12.675	5.065	11.257
9	0.5505	10.150	10.893	11.255	5.767	10.857
9	0.5515	17.801	24.626	13.480	13.524	14.017
9	0.5525	7.505	12.158	12.558	7.512	11.744
9	0.5535	11.253	19.927	16.751	12.204	13.436
9	0.5545	12.776	16.836	13.732	7.192	13.358
9	0.5555	7.774	12.915	9.654	22.902	19.782
9	0.5565	13.849	17.881	17.104	18.618	30.886
9	0.5575	9.342	15.333	13.680	14.440	21.713
9	0.5585	16.535	22.380	22.956	11.791	17.968
9	0.5595	5.780	8.507	11.775	16.611	20.183
9	0.5605	10.509	14.811	15.010	10.855	18.369
9	0.5615	13.893	12.282	10.758	16.144	18.137
9	0.5625	20.303	10.284	9.519	18.212	20.990
9	0.5635	11.746	19.862	15.130	13.780	25.205
9	0.5645	12.929	14.149	17.473	25.264	18.427
9	0.5655	7.342	9.801	6.716	9.731	18.649
9	0.5665	9.350	10.834	5.573	16.547	4.680
9	0.5675	18.546	26.256	20.116	22.291	18.596
9	0.5685	15.014	19.648	20.313	9.959	10.499
9	0.5695	14.963	19.585	20.183	10.213	10.481
9	0.5705	10.209	11.802	12.071	17.117	11.355
9	0.5715	13.470	21.701	16.319	20.358	16.001
9	0.5725	11.879	18.008	15.819	7.206	22.208
9	0.5735	15.394	14.696	17.808	7.839	19.471
9	0.5745	7.249	15.606	9.159	14.960	19.299
9	0.5755	7.247	12.295	9.371	14.713	15.218
9	0.5765	5.717	7.338	11.798	9.795	17.157
9	0.5775	12.396	18.429	15.698	20.068	18.418
9	0.5785	6.050	5.980	6.212	12.405	14.099
9	0.5795	10.253	9.950	10.029	10.417	9.433
9	0.5805	14.766	17.000	12.450	10.243	21.054
9	0.5815	5.875	5.368	2.582	3.960	19.391
9	0.5825	7.387	11.130	9.340	12.628	18.382
9	0.5835	9.529	13.660	14.426	11.443	16.153
9	0.5845	9.515	13.670	14.417	11.578	16.138

9	0.5855	7.318	8.004	8.966	15.049	11.653
9	0.5865	10.997	16.102	14.245	19.595	21.518
9	0.5875	9.528	15.831	10.001	39.353	21.189
9	0.5885	5.278	16.628	9.356	19.270	15.461
9	0.5895	9.162	17.384	11.104	16.824	11.651
9	0.5905	4.624	8.902	5.830	17.813	21.239
9	0.5915	11.801	7.268	6.897	13.910	29.526
9	0.5925	9.951	13.108	15.717	6.879	17.277
9	0.5935	13.079	17.308	9.185	17.924	12.888
9	0.5945	10.615	16.493	7.870	21.016	18.815
9	0.5955	2.616	9.311	6.928	17.823	23.256
9	0.5965	3.830	9.085	2.870	16.054	8.897
9	0.5975	8.429	13.774	7.221	14.456	9.621
9	0.5985	16.444	23.910	21.955	20.789	14.125
9	0.5995	13.251	14.457	17.327	7.892	13.269
9	0.6005	6.129	6.584	10.016	8.097	6.807
9	0.6015	9.035	12.773	12.340	17.351	19.078
9	0.6025	2.541	5.376	4.623	16.386	10.105
9	0.6035	7.638	11.636	5.893	9.533	9.761
9	0.6045	3.903	3.499	4.314	17.218	16.226
9	0.6055	2.623	3.484	2.094	13.528	13.607
9	0.6065	13.734	10.886	9.999	9.510	25.398
9	0.6075	9.638	10.278	8.652	8.970	25.631
9	0.6085	7.787	15.240	12.447	16.787	7.903
9	0.6095	4.573	10.999	5.767	17.407	9.837
9	0.6105	13.275	16.158	11.844	18.427	22.603
9	0.6115	5.928	6.736	4.696	13.093	13.955
9	0.6125	6.897	11.435	10.970	13.093	17.058
9	0.6135	11.524	18.740	15.396	9.751	10.092
9	0.6145	9.460	12.044	8.359	13.534	13.430
9	0.6155	6.789	13.165	11.951	14.754	10.593
9	0.6165	7.131	11.771	10.547	14.690	6.844
9	0.6175	4.654	4.946	9.483	9.622	10.930
9	0.6185	6.006	9.459	6.146	7.724	9.097
9	0.6195	10.473	15.723	9.180	15.652	17.304
9	0.6205	6.550	6.127	6.219	10.157	22.501
9	0.6215	3.899	4.573	5.001	9.303	14.939
9	0.6225	6.555	10.723	7.206	9.413	8.988
9	0.6235	9.283	23.013	13.505	16.005	12.784
9	0.6245	14.896	17.975	13.627	12.491	9.352
9	0.6255	9.149	11.911	10.543	16.592	4.609
9	0.6265	9.336	10.221	8.874	20.664	17.068
9	0.6275	8.445	9.818	9.112	11.439	10.336
9	0.6285	6.529	13.758	6.878	14.695	14.958
9	0.6295	8.351	14.433	6.944	13.758	21.004
9	0.6305	2.388	6.497	3.279	19.052	14.316
9	0.6315	3.283	6.166	2.029	8.378	13.870
9	0.6325	6.049	12.102	11.131	7.889	9.305
9	0.6335	11.796	19.931	18.338	22.308	12.642
9	0.6345	11.763	19.924	18.348	22.322	12.714

9	0.6355	5.193	8.814	9.133	6.423	6.592
9	0.6365	2.422	6.966	4.530	11.401	7.977
9	0.6375	9.564	13.412	9.541	18.840	13.721
9	0.6385	5.026	7.058	4.682	8.012	6.533
9	0.6395	5.731	9.580	7.689	10.528	18.122
9	0.6405	14.646	20.270	22.865	11.514	7.506
9	0.6415	11.857	21.403	15.910	26.411	14.279
9	0.6425	8.789	13.145	6.513	17.449	14.384
9	0.6435	17.096	17.511	12.050	16.242	16.201
9	0.6445	4.862	1.209	6.388	16.278	13.552
9	0.6455	9.786	20.806	15.012	13.785	10.784
9	0.6465	9.345	17.070	11.705	13.737	16.634
9	0.6475	14.197	14.885	12.451	17.290	24.342
9	0.6485	10.866	7.202	7.967	16.323	15.476
9	0.6495	12.787	5.861	7.045	5.729	15.694
9	0.6505	12.788	5.873	7.048	5.764	15.663
9	0.6515	6.894	4.129	9.290	8.624	13.176
9	0.6525	10.408	11.377	8.155	20.130	12.201
9	0.6535	16.426	26.466	17.187	18.391	21.986
9	0.6545	14.876	19.644	16.530	12.783	15.594
9	0.6555	11.521	9.863	7.999	20.970	15.678
9	0.6565	12.748	10.271	11.721	11.186	17.784
9	0.6575	8.300	3.578	5.570	12.001	15.268
9	0.6585	11.710	8.079	10.427	8.836	15.240
9	0.6595	13.432	11.486	9.330	10.042	12.449
9	0.6605	7.347	10.521	7.835	11.370	9.388
9	0.6615	4.819	5.000	6.374	13.430	8.512
9	0.6625	14.195	15.312	10.114	19.373	25.512
9	0.6635	6.503	4.517	6.186	20.528	19.586
9	0.6645	11.073	8.761	9.088	24.929	16.467
9	0.6655	6.922	20.594	7.806	29.117	13.041
9	0.6665	12.003	16.807	12.181	30.261	23.279
9	0.6675	10.956	17.067	15.921	21.907	6.310
9	0.6685	6.923	8.416	8.702	23.426	9.864
9	0.6695	3.725	3.754	3.704	12.281	7.089
9	0.6705	13.336	14.284	14.677	8.986	19.197
9	0.6715	16.517	15.795	10.855	21.551	26.133
9	0.6725	9.761	15.260	12.305	28.230	23.146
9	0.6735	11.013	11.894	12.471	16.721	27.640
9	0.6745	11.585	9.659	11.752	18.626	34.717
9	0.6755	11.382	9.338	6.803	21.368	18.440
9	0.6765	14.607	15.100	8.083	17.264	26.775
9	0.6775	10.967	7.860	8.312	21.559	27.017
9	0.6785	7.932	9.899	6.521	21.278	18.160
9	0.6795	10.158	15.586	6.597	25.851	19.564
9	0.6805	8.194	13.628	5.086	32.141	19.608
9	0.6815	18.208	15.295	11.321	8.411	21.111
9	0.6825	14.280	28.140	15.262	20.079	24.666
9	0.6835	17.647	23.019	20.146	11.732	25.909
9	0.6845	14.078	16.598	11.141	16.192	28.219

9	0.6855	26.715	25.147	22.713	16.511	36.002
9	0.6865	10.784	9.850	10.242	16.196	12.446
9	0.6875	14.065	12.574	11.479	21.883	24.946
9	0.6885	16.292	19.079	15.164	12.835	29.819
9	0.6895	16.126	19.415	15.211	8.618	20.885
9	0.6905	19.413	18.862	18.001	19.121	44.850
9	0.6915	14.096	18.082	14.515	6.859	15.545
9	0.6925	12.101	11.986	11.809	13.332	23.678
9	0.6935	16.108	17.030	17.564	9.505	25.147
9	0.6945	12.488	14.851	7.519	15.405	24.907
9	0.6955	4.972	7.055	5.130	12.966	20.627
9	0.6965	27.262	29.545	22.883	18.925	36.152
9	0.6975	23.757	20.810	20.798	13.954	26.038
9	0.6985	22.581	19.616	21.261	23.980	36.610
9	0.6995	8.292	14.607	10.953	23.006	20.577
9	0.7005	11.511	13.069	10.059	18.295	16.833
9	0.7015	15.528	21.534	14.815	23.811	35.367
9	0.7025	16.522	17.984	14.095	14.613	22.806
9	0.7035	16.503	21.168	9.802	20.001	32.913
9	0.7045	16.426	27.145	20.662	17.243	17.862
9	0.7055	11.255	13.795	8.585	12.927	32.172
9	0.7065	13.866	13.565	9.634	20.050	31.231
9	0.7075	8.673	7.731	8.966	13.998	26.958
9	0.7085	8.854	13.722	12.534	15.014	22.103
9	0.7095	14.343	15.148	11.429	12.992	22.750
9	0.7105	11.575	14.518	7.607	14.076	22.154
9	0.7115	8.082	9.199	7.463	11.818	20.215
9	0.7125	13.178	14.343	11.425	13.415	24.746
9	0.7135	10.239	13.281	11.541	9.999	17.856
9	0.7145	10.348	8.852	9.123	7.495	14.111
9	0.7155	10.049	7.657	8.910	9.567	12.594
9	0.7165	15.265	16.226	15.593	15.929	25.855
9	0.7175	13.158	11.832	8.706	20.860	22.474
9	0.7185	11.170	13.642	13.472	15.844	20.366
9	0.7195	14.930	14.693	12.175	21.095	18.422
9	0.7205	13.166	16.473	13.638	16.879	22.376
9	0.7215	13.450	9.629	14.322	21.351	30.689
9	0.7225	18.739	17.800	18.789	17.056	20.563
9	0.7235	11.974	18.575	13.073	14.414	12.976
9	0.7245	11.944	18.607	13.061	14.385	12.986
9	0.7255	13.549	16.447	16.286	17.310	23.337
9	0.7265	13.389	16.084	15.855	17.282	23.333
9	0.7275	18.860	20.528	13.016	17.249	20.813
9	0.7285	16.140	20.422	12.933	13.578	23.251
9	0.7295	11.590	20.250	15.117	16.230	22.496
9	0.7305	11.589	20.251	15.118	16.234	22.500
9	0.7315	8.671	16.719	10.960	9.551	12.847
9	0.7325	9.320	12.889	8.930	10.628	17.660
9	0.7335	15.370	25.285	10.025	15.864	15.173
9	0.7345	27.300	44.526	22.112	31.342	24.160

9	0.7355	10.294	18.706	11.458	24.794	26.821
9	0.7365	6.756	20.321	9.293	22.573	18.744
9	0.7375	7.157	14.370	6.869	19.597	21.021
9	0.7385	24.911	27.390	19.686	15.949	19.842
9	0.7395	16.280	28.162	15.359	14.863	16.311
9	0.7405	18.219	19.650	15.305	11.875	15.528
9	0.7415	7.434	11.516	7.913	17.781	9.101
9	0.7425	22.837	24.354	19.519	7.068	21.003
9	0.7435	11.998	12.620	9.773	18.811	36.355
9	0.7445	5.951	7.832	5.955	10.858	32.266
9	0.7455	8.558	25.728	12.114	24.816	16.276
9	0.7465	9.414	15.162	12.156	9.090	11.375
9	0.7475	10.360	12.546	11.651	5.101	12.497
9	0.7485	10.986	14.487	12.176	23.911	34.269
9	0.7495	13.160	20.509	19.604	13.188	21.697
9	0.7505	12.764	20.487	19.781	19.200	23.974
9	0.7515	10.665	17.467	16.458	15.036	13.954
9	0.7525	10.042	18.591	15.652	15.921	23.303
9	0.7535	8.025	15.469	14.736	16.289	26.241
9	0.7545	10.088	15.626	15.806	21.066	19.910
9	0.7555	16.501	29.556	17.023	27.141	23.495
9	0.7565	13.745	21.161	10.882	17.901	20.888
9	0.7575	16.403	24.662	10.644	28.623	27.110
9	0.7585	12.083	14.078	7.691	20.160	11.790
9	0.7595	29.240	40.828	35.880	22.949	13.884
9	0.7605	30.018	28.857	33.373	15.982	18.485
9	0.7615	2.206	4.351	6.985	18.676	12.501
9	0.7625	8.410	16.874	11.988	17.402	13.367
9	0.7635	13.097	22.589	17.071	19.420	7.491
9	0.7645	15.835	25.068	15.939	15.154	10.458
9	0.7655	14.428	19.200	11.900	12.079	27.321
9	0.7665	6.633	20.493	7.713	24.086	14.699
9	0.7675	4.726	19.143	4.942	36.586	20.221
9	0.7685	10.199	19.110	11.944	13.470	29.245
9	0.7695	12.208	16.799	13.321	16.032	33.086
9	0.7705	11.589	11.088	10.331	14.278	25.836
9	0.7715	11.562	11.103	10.327	14.268	25.825
9	0.7725	10.210	17.827	11.672	16.000	7.905
9	0.7735	23.483	37.022	27.876	11.353	15.753
9	0.7745	16.357	21.345	18.806	10.425	34.058
9	0.7755	17.852	29.441	17.387	27.650	21.781
9	0.7765	22.763	21.437	14.820	17.452	11.950
9	0.7775	12.514	15.925	7.229	17.917	16.509
9	0.7785	10.541	14.901	4.368	17.504	26.775
9	0.7795	7.410	7.217	5.605	16.089	12.638
9	0.7805	11.237	15.750	11.792	19.628	21.999
9	0.7815	4.044	16.794	6.040	19.912	20.997
9	0.7825	7.531	17.305	7.120	15.280	15.048
9	0.7835	15.024	25.286	14.433	18.932	17.767
9	0.7845	13.555	8.863	9.044	14.672	12.118

9	0.7855	15.185	23.386	16.676	17.864	12.477
9	0.7865	10.904	16.593	9.304	17.428	13.202
9	0.7875	18.127	27.691	18.843	18.475	14.972
9	0.7885	7.884	7.677	6.630	11.478	23.978
9	0.7895	14.810	16.597	19.506	13.128	19.103
9	0.7905	16.938	17.548	14.685	18.288	17.504
9	0.7915	11.348	11.491	8.987	9.419	13.999
9	0.7925	8.403	10.202	4.249	13.473	14.522
9	0.7935	11.113	14.224	9.900	23.763	13.576
9	0.7945	12.478	20.413	18.782	27.505	15.006
9	0.7955	10.242	8.933	11.331	13.671	11.172
9	0.7965	11.963	14.095	14.355	13.075	17.927
9	0.7975	3.283	8.410	3.848	19.571	11.615
9	0.7985	7.752	9.383	8.403	16.002	13.709
9	0.7995	6.159	9.307	5.908	20.493	7.550
9	0.8005	10.013	19.520	15.148	15.346	13.662
9	0.8015	16.409	20.948	18.283	21.570	19.553
9	0.8025	12.793	13.867	12.805	25.793	21.023
9	0.8035	19.461	23.080	21.384	19.049	10.532
9	0.8045	14.528	21.982	19.092	24.602	17.465
9	0.8055	10.485	21.738	13.189	20.550	18.570
9	0.8065	10.281	13.021	9.780	11.599	14.358
9	0.8075	17.331	10.040	13.794	7.524	20.105
9	0.8085	11.450	7.311	10.602	15.802	18.949
9	0.8095	10.927	9.923	12.231	6.873	10.570
9	0.8105	14.920	17.698	12.252	8.215	8.027
9	0.8115	9.760	16.889	11.634	8.167	12.817
9	0.8125	11.726	10.389	15.787	7.828	14.569
9	0.8135	13.910	14.724	14.176	7.756	17.051
9	0.8145	4.036	8.592	4.664	7.766	9.442
9	0.8155	8.570	20.384	16.365	16.973	18.039
9	0.8165	16.559	25.260	19.911	18.616	18.255
9	0.8175	11.617	13.170	12.749	7.511	11.308
9	0.8185	21.078	18.243	24.065	11.675	19.489
9	0.8195	10.980	17.573	12.004	8.159	12.467
9	0.8205	12.721	17.012	16.133	11.590	11.909
9	0.8215	12.292	14.387	13.684	7.549	10.047
9	0.8225	16.107	21.594	19.519	9.005	13.141
9	0.8235	15.507	23.660	17.737	11.793	10.733
9	0.8245	10.969	22.103	16.515	8.721	15.724
9	0.8255	9.094	12.549	17.383	12.967	11.753
9	0.8265	15.662	17.270	23.348	4.962	12.664
9	0.8275	14.863	17.861	17.042	26.409	15.144
9	0.8285	16.005	14.143	14.195	14.208	8.272
9	0.8295	12.036	8.511	10.810	11.516	10.269
9	0.8305	17.191	10.021	8.940	12.483	9.308
9	0.8315	25.282	22.278	26.245	13.059	14.935
9	0.8325	8.548	9.612	12.439	15.704	8.741
9	0.8335	11.645	9.185	12.194	13.988	5.436
9	0.8345	10.431	9.723	14.677	14.857	13.341

9	0.8355	10.595	12.039	13.091	11.397	6.575
9	0.8365	26.508	15.641	18.859	27.399	13.497
9	0.8375	25.824	19.395	27.174	17.834	8.310
9	0.8385	13.160	14.551	12.424	12.820	6.844
9	0.8395	12.116	12.610	14.716	9.196	9.891
9	0.8405	16.018	17.157	16.865	14.189	18.423
9	0.8415	13.657	15.708	16.945	15.232	19.191
9	0.8425	11.106	20.808	17.795	17.420	10.421
9	0.8435	10.040	9.725	9.867	13.561	9.144
9	0.8445	10.843	11.523	11.665	13.882	3.412
9	0.8455	5.947	11.514	6.206	12.696	7.014
9	0.8465	6.041	11.608	6.308	12.733	7.039
9	0.8475	15.395	13.031	12.848	10.384	11.676
9	0.8485	10.857	11.501	15.109	7.896	7.471
9	0.8495	18.424	24.201	24.014	17.865	11.639
9	0.8505	18.288	24.231	24.000	17.907	11.689
9	0.8515	17.051	19.757	22.609	10.610	8.194
9	0.8525	13.689	21.870	20.349	15.368	8.313
9	0.8535	21.423	25.755	22.641	11.713	5.299
9	0.8545	18.442	27.866	21.176	17.355	6.497
9	0.8555	12.125	20.313	9.756	14.485	8.316
9	0.8565	14.507	15.479	20.008	12.837	17.901
9	0.8575	7.888	11.780	10.680	17.063	13.530
9	0.8585	1.835	5.311	1.373	15.722	9.231
9	0.8595	1.836	5.311	1.373	15.722	9.225
9	0.8605	5.056	8.137	7.195	15.565	9.124
9	0.8615	10.490	17.222	14.200	11.566	8.720
9	0.8625	12.247	13.367	16.314	10.580	12.673
9	0.8635	18.133	15.652	17.824	10.029	9.938
9	0.8645	7.376	10.606	8.374	10.749	7.973
9	0.8655	5.734	6.494	10.656	11.279	9.683
9	0.8665	8.939	3.231	5.682	10.704	6.032
9	0.8675	12.871	7.511	10.083	9.887	3.987
9	0.8685	10.782	11.867	13.803	18.058	11.123
9	0.8695	14.321	10.980	6.216	17.604	18.382
9	0.8705	16.022	12.230	8.246	16.925	14.586
9	0.8715	5.740	5.223	3.970	10.270	4.867
9	0.8725	14.814	12.166	14.021	18.636	12.345
9	0.8735	18.954	14.612	17.019	9.161	8.323
9	0.8745	5.723	9.061	8.301	7.989	6.163
9	0.8755	8.195	11.147	11.619	16.778	10.134
9	0.8765	11.320	14.027	15.782	18.648	12.230
9	0.8775	10.624	7.083	12.336	13.537	11.821
9	0.8785	23.510	19.272	25.863	10.304	11.473
9	0.8795	4.114	4.745	3.920	9.772	4.700
9	0.8805	17.492	23.455	23.131	10.454	11.571
9	0.8815	13.502	24.235	21.663	23.575	27.841
9	0.8825	13.172	14.583	19.445	18.371	14.713
9	0.8835	21.174	24.779	19.359	10.315	19.439
9	0.8845	9.946	6.028	9.971	5.453	14.763

9	0.8855	11.963	7.812	10.738	15.833	12.732
9	0.8865	10.485	12.311	12.491	6.115	12.187
9	0.8875	11.863	11.817	9.774	7.471	18.058
9	0.8885	13.233	14.147	22.591	5.856	13.463
9	0.8895	14.783	13.309	13.116	7.765	15.164
9	0.8905	12.722	14.840	12.022	17.270	8.941
9	0.8915	29.899	31.226	30.999	17.737	11.481
9	0.8925	24.193	27.600	24.978	21.441	11.555
9	0.8935	13.176	13.157	14.062	13.210	17.091
9	0.8945	7.977	9.207	8.046	12.783	8.252
9	0.8955	19.333	17.888	21.300	15.430	7.050
9	0.8965	17.745	22.927	21.616	11.304	9.435
9	0.8975	10.025	11.529	17.600	10.640	8.714
9	0.8985	8.362	11.442	9.941	8.367	7.407
9	0.8995	13.924	19.340	16.352	8.002	7.149
9	0.9005	11.238	16.246	13.434	6.794	6.401
9	0.9015	6.848	12.633	9.439	7.318	2.704
9	0.9025	0.000	0.000	0.000	0.000	0.000
10	0.0027	0.000	0.000	0.000	0.000	0.000
10	0.0037	14.549	7.773	14.047	13.966	23.960
10	0.0047	8.058	12.374	10.995	14.067	21.692
10	0.0057	10.745	7.540	13.047	7.732	16.898
10	0.0067	16.589	9.566	14.969	14.876	22.922
10	0.0077	13.683	10.435	12.464	13.586	25.102
10	0.0087	11.574	9.809	12.179	9.825	24.589
10	0.0097	13.307	8.446	15.443	10.291	19.676
10	0.0107	20.887	9.733	14.862	12.296	29.134
10	0.0117	11.274	13.859	13.533	14.778	18.547
10	0.0127	10.887	3.860	8.643	14.084	11.598
10	0.0137	7.980	3.970	11.102	12.776	19.038
10	0.0147	9.775	11.995	9.631	6.890	18.666
10	0.0157	7.356	8.503	9.529	12.114	14.238
10	0.0167	7.645	10.727	8.588	7.185	8.188
10	0.0177	17.817	12.772	16.332	12.606	16.803
10	0.0187	14.365	14.468	11.276	9.255	10.819
10	0.0197	18.318	15.390	18.952	13.463	16.728
10	0.0207	12.188	13.056	19.221	10.379	25.335
10	0.0217	4.845	8.219	6.775	6.395	6.780
10	0.0227	3.054	5.557	8.306	11.855	10.123
10	0.0237	9.045	7.379	16.663	14.801	13.794
10	0.0247	27.005	17.801	30.076	20.472	12.066
10	0.0257	5.919	6.648	11.225	18.630	15.227
10	0.0267	5.832	6.396	6.803	19.073	8.694
10	0.0277	13.595	12.421	12.450	14.875	6.451
10	0.0287	14.332	7.016	12.848	15.056	14.520
10	0.0297	10.677	11.996	13.140	15.633	12.870
10	0.0307	20.356	14.796	16.839	12.331	16.114
10	0.0317	19.661	13.528	25.465	13.615	14.507
10	0.0327	14.871	4.551	10.617	12.813	25.023
10	0.0337	18.139	7.603	17.565	18.725	24.162

10	0.0347	11.739	7.916	9.392	15.860	17.939
10	0.0357	13.112	10.998	12.094	14.344	16.743
10	0.0367	13.093	14.602	14.586	21.426	25.269
10	0.0377	4.327	6.726	5.484	8.830	9.474
10	0.0387	21.306	22.731	24.250	12.676	7.470
10	0.0397	16.802	14.845	17.520	11.530	12.790
10	0.0407	13.226	11.840	16.485	19.181	10.058
10	0.0417	19.469	16.864	18.348	27.040	16.692
10	0.0427	20.660	9.172	19.097	18.899	18.739
10	0.0437	28.378	19.778	23.803	11.396	8.377
10	0.0447	18.376	20.948	26.694	18.462	12.044
10	0.0457	16.734	9.945	18.822	15.055	6.712
10	0.0467	21.449	20.930	24.168	13.537	18.241
10	0.0477	22.595	17.824	15.828	24.838	26.436
10	0.0487	31.463	32.656	41.513	20.228	14.097
10	0.0497	25.652	24.944	29.632	21.767	10.822
10	0.0507	21.371	20.537	24.743	17.091	13.137
10	0.0517	17.170	8.623	15.067	18.808	24.572
10	0.0527	15.286	9.167	14.993	24.741	24.353
10	0.0537	21.743	24.584	30.955	24.739	14.511
10	0.0547	15.246	19.822	18.413	24.205	18.287
10	0.0557	20.321	13.638	20.093	16.053	12.844
10	0.0567	21.085	13.238	11.382	19.320	12.096
10	0.0577	12.682	15.815	12.060	21.637	19.152
10	0.0587	14.554	15.306	18.300	20.073	14.914
10	0.0597	13.524	9.476	17.071	16.869	9.890
10	0.0607	15.680	21.763	26.078	14.619	7.935
10	0.0617	15.760	16.302	25.403	12.176	8.783
10	0.0627	25.726	25.167	32.151	18.821	16.948
10	0.0637	22.315	23.574	28.952	15.832	10.864
10	0.0647	20.712	21.512	26.035	8.670	9.007
10	0.0657	13.927	17.858	20.510	4.790	6.940
10	0.0667	9.870	11.680	12.098	10.569	13.678
10	0.0677	6.612	7.037	8.131	9.886	4.072
10	0.0687	12.173	12.304	12.630	14.344	15.347
10	0.0697	12.078	9.977	15.244	17.590	14.528
10	0.0707	12.695	9.472	12.414	9.657	14.983
10	0.0717	15.656	19.860	17.659	22.094	14.428
10	0.0727	17.313	19.836	21.604	12.176	14.355
10	0.0737	16.141	14.761	15.972	7.441	15.540
10	0.0747	14.764	14.735	10.946	17.463	23.065
10	0.0757	20.869	16.036	15.639	18.454	23.202
10	0.0767	10.489	14.188	11.833	11.821	22.293
10	0.0777	16.655	22.450	22.148	19.945	16.896
10	0.0787	10.072	4.079	8.769	13.060	11.536
10	0.0797	6.580	5.870	8.660	13.962	16.892
10	0.0807	8.809	9.624	12.454	11.647	15.253
10	0.0817	8.273	13.207	19.370	8.911	15.892
10	0.0827	10.368	9.222	10.683	19.404	21.303
10	0.0837	16.262	15.852	19.318	19.602	20.243

10	0.0847	7.966	13.786	14.772	19.780	14.240
10	0.0857	13.390	15.606	22.980	22.327	17.922
10	0.0867	5.315	10.403	8.860	19.158	18.099
10	0.0877	5.417	5.709	5.794	11.544	20.591
10	0.0887	9.066	9.856	10.907	17.137	20.370
10	0.0897	3.601	3.525	5.661	10.747	18.115
10	0.0907	7.459	5.406	4.901	3.714	22.552
10	0.0917	19.017	15.445	17.724	9.154	10.052
10	0.0927	14.908	12.796	14.511	14.062	18.607
10	0.0937	15.514	4.755	12.150	17.480	17.981
10	0.0947	15.764	11.896	17.456	27.616	11.974
10	0.0957	20.145	13.182	21.659	15.132	10.926
10	0.0967	17.681	17.375	21.654	11.554	13.756
10	0.0977	18.609	15.629	18.757	16.604	17.505
10	0.0987	10.683	8.590	10.592	11.128	7.618
10	0.0997	10.189	8.510	12.493	23.603	22.271
10	0.1007	7.714	10.731	16.187	20.495	19.762
10	0.1017	13.411	9.732	10.045	14.837	12.227
10	0.1027	11.967	5.525	7.718	12.708	10.833
10	0.1037	13.400	7.840	13.178	7.232	14.276
10	0.1047	19.647	9.995	14.260	19.358	17.035
10	0.1057	14.319	11.110	15.797	17.914	22.394
10	0.1067	14.496	11.427	15.766	16.544	9.343
10	0.1077	22.163	10.618	18.767	13.607	13.147
10	0.1087	9.781	8.025	4.446	12.668	10.687
10	0.1097	14.643	12.448	14.679	13.889	17.907
10	0.1107	14.874	10.749	15.675	9.490	11.354
10	0.1117	31.846	21.117	30.517	6.754	11.117
10	0.1127	13.831	10.084	14.755	6.218	7.598
10	0.1137	20.047	16.662	21.167	10.025	9.519
10	0.1147	12.918	9.809	14.273	6.713	4.788
10	0.1157	12.048	14.108	17.282	10.945	6.156
10	0.1167	18.814	14.156	25.666	14.267	9.964
10	0.1177	5.583	4.424	4.704	3.127	2.130
10	0.1187	11.705	6.530	10.268	9.350	5.920
10	0.1197	17.075	14.942	15.878	10.745	13.695
10	0.1207	9.560	9.684	8.333	6.966	11.687
10	0.1217	12.431	9.371	12.282	4.877	4.479
10	0.1227	12.208	7.750	10.349	7.302	5.212
10	0.1237	20.718	7.868	18.339	17.315	5.352
10	0.1247	12.419	3.357	7.490	9.228	6.018
10	0.1257	13.214	7.948	8.387	11.086	10.379
10	0.1267	7.362	5.683	9.085	10.106	16.626
10	0.1277	11.582	10.702	15.828	10.422	17.672
10	0.1287	9.453	11.598	14.344	13.551	9.549
10	0.1297	20.396	8.565	21.825	13.988	4.864
10	0.1307	18.148	6.737	18.360	17.406	1.835
10	0.1317	22.715	13.635	23.747	5.255	4.303
10	0.1327	18.618	11.483	17.723	9.760	4.160
10	0.1337	12.304	9.400	9.735	11.995	4.962

10	0.1347	22.755	24.429	27.108	8.685	5.944
10	0.1357	26.250	14.432	24.937	9.719	2.353
10	0.1367	10.432	10.270	14.404	12.209	13.112
10	0.1377	18.512	20.837	18.674	11.249	7.235
10	0.1387	15.299	13.595	15.137	10.138	13.287
10	0.1397	15.292	13.584	15.100	10.086	13.355
10	0.1407	11.814	7.668	14.482	9.511	13.384
10	0.1417	9.152	8.288	10.029	5.250	13.971
10	0.1427	29.296	19.975	25.233	6.368	8.725
10	0.1437	22.525	19.429	21.431	12.244	9.193
10	0.1447	19.756	9.989	15.284	11.008	17.639
10	0.1457	17.032	5.975	13.094	12.952	19.273
10	0.1467	7.879	8.585	11.617	14.407	10.815
10	0.1477	14.092	14.123	18.578	6.813	12.637
10	0.1487	13.862	16.206	19.908	15.334	15.970
10	0.1497	8.290	13.867	12.246	22.302	10.753
10	0.1507	24.710	24.165	27.478	11.463	21.948
10	0.1517	21.967	21.425	23.022	14.133	15.822
10	0.1527	21.854	19.117	26.213	19.418	18.910
10	0.1537	22.014	19.263	26.340	19.340	19.046
10	0.1547	14.574	12.740	10.529	21.734	21.058
10	0.1557	14.456	12.699	10.438	21.794	21.085
10	0.1567	16.242	11.188	15.717	13.409	13.605
10	0.1577	7.831	12.305	7.226	14.959	8.360
10	0.1587	19.254	21.060	15.199	20.914	11.633
10	0.1597	16.955	11.502	11.050	11.513	15.241
10	0.1607	10.089	13.240	11.317	10.513	15.190
10	0.1617	18.396	17.857	12.611	25.705	11.136
10	0.1627	19.227	24.020	17.475	26.434	13.319
10	0.1637	11.374	14.461	16.649	12.405	22.845
10	0.1647	14.032	11.828	12.963	20.270	25.369
10	0.1657	8.384	9.147	8.287	19.122	24.292
10	0.1667	7.499	11.518	7.506	21.127	24.369
10	0.1677	14.185	25.464	21.898	21.885	12.428
10	0.1687	9.921	20.488	19.209	23.498	13.052
10	0.1697	20.687	26.866	19.266	15.836	11.144
10	0.1707	26.144	27.025	23.150	24.017	26.153
10	0.1717	11.344	15.230	13.839	12.800	25.251
10	0.1727	8.934	7.864	6.491	13.464	8.364
10	0.1737	6.143	12.835	7.907	17.519	8.792
10	0.1747	6.440	12.019	10.597	18.447	7.326
10	0.1757	8.774	18.779	12.923	24.554	9.908
10	0.1767	8.218	9.301	13.165	7.574	11.804
10	0.1777	6.054	10.977	8.231	8.572	12.504
10	0.1787	8.938	14.263	11.094	13.003	17.599
10	0.1797	6.293	6.949	7.896	11.410	19.289
10	0.1807	4.994	6.841	2.927	10.646	21.047
10	0.1817	7.546	7.825	9.317	13.862	11.716
10	0.1827	7.540	9.842	9.978	15.173	11.843
10	0.1837	10.762	9.704	7.435	5.370	11.521

10	0.1847	10.157	9.211	7.436	7.368	10.764
10	0.1857	3.987	4.338	4.561	6.816	11.674
10	0.1867	2.689	7.756	2.410	9.226	15.774
10	0.1877	9.676	14.194	13.649	14.689	18.918
10	0.1887	10.466	13.689	9.160	15.219	18.528
10	0.1897	6.224	5.071	8.078	4.821	7.854
10	0.1907	10.506	12.345	13.560	6.310	7.172
10	0.1917	12.956	11.587	14.353	15.073	14.434
10	0.1927	18.864	10.666	13.494	11.416	9.710
10	0.1937	3.600	6.566	11.878	5.326	13.559
10	0.1947	8.533	7.776	9.576	8.732	10.157
10	0.1957	20.330	16.194	19.042	11.482	13.237
10	0.1967	18.034	14.871	18.665	11.391	20.433
10	0.1977	15.134	11.307	13.648	14.396	12.680
10	0.1987	7.392	5.284	6.497	11.082	11.490
10	0.1997	7.066	10.260	8.425	15.086	15.410
10	0.2007	15.542	9.463	10.046	10.993	17.613
10	0.2017	6.240	4.936	11.464	8.612	20.877
10	0.2027	6.599	6.291	9.681	3.328	12.786
10	0.2037	8.875	6.524	8.257	7.490	18.131
10	0.2047	4.794	6.554	7.973	7.795	11.103
10	0.2057	13.802	10.755	12.802	11.420	23.429
10	0.2067	16.657	14.902	17.777	13.089	13.854
10	0.2077	9.347	8.741	13.418	4.478	9.149
10	0.2087	9.343	8.744	13.411	4.456	9.158
10	0.2097	13.984	11.931	17.148	4.913	6.837
10	0.2107	7.885	12.025	12.072	9.172	18.103
10	0.2117	14.603	18.686	26.291	6.548	14.285
10	0.2127	5.306	7.960	10.398	5.709	2.462
10	0.2137	5.639	5.744	7.915	7.367	5.218
10	0.2147	7.311	3.882	6.240	5.233	15.809
10	0.2157	3.323	5.884	8.787	7.823	27.484
10	0.2167	6.627	13.235	10.677	9.709	14.673
10	0.2177	6.713	11.649	7.955	13.993	19.903
10	0.2187	1.319	5.785	3.838	12.474	9.502
10	0.2197	10.306	9.961	13.529	16.424	12.133
10	0.2207	7.513	14.959	15.530	13.819	20.671
10	0.2217	9.687	17.986	17.553	20.423	10.019
10	0.2227	6.982	10.286	10.346	4.641	7.528
10	0.2237	9.380	13.641	15.627	7.040	10.703
10	0.2247	10.350	9.723	13.885	8.722	9.855
10	0.2257	9.056	11.723	10.801	13.722	10.683
10	0.2267	10.529	17.940	13.819	23.239	22.829
10	0.2277	18.002	9.602	20.436	9.271	20.427
10	0.2287	13.950	9.029	14.497	11.484	21.012
10	0.2297	6.598	14.228	8.039	14.744	13.152
10	0.2307	25.205	14.209	19.354	19.570	20.298
10	0.2317	25.212	14.224	19.357	19.563	20.306
10	0.2327	20.909	19.046	23.486	15.813	16.037
10	0.2337	26.342	17.472	24.240	13.625	14.659

10	0.2347	16.185	10.367	15.318	6.906	9.685
10	0.2357	18.135	22.419	19.550	9.874	10.022
10	0.2367	18.107	22.388	19.533	9.878	9.999
10	0.2377	26.879	14.425	24.484	25.043	12.048
10	0.2387	26.674	14.051	25.138	21.888	13.103
10	0.2397	23.803	9.748	21.283	19.568	14.514
10	0.2407	8.761	6.937	18.151	5.353	11.355
10	0.2417	19.124	15.667	20.066	9.327	15.914
10	0.2427	5.219	6.448	6.068	8.132	11.754
10	0.2437	13.016	13.499	15.725	8.664	11.924
10	0.2447	28.883	24.406	33.313	16.956	6.630
10	0.2457	13.939	12.010	14.228	14.882	16.182
10	0.2467	14.999	16.773	17.045	7.506	6.747
10	0.2477	6.612	11.728	12.872	5.061	10.445
10	0.2487	7.030	7.124	9.378	4.210	11.410
10	0.2497	16.860	19.253	21.145	7.033	11.039
10	0.2507	7.988	5.764	6.889	11.506	17.267
10	0.2517	14.617	21.453	19.492	12.985	19.766
10	0.2527	20.849	20.302	22.957	12.962	19.875
10	0.2537	13.209	18.092	17.465	15.805	21.969
10	0.2547	14.381	10.471	14.151	10.123	21.443
10	0.2557	20.019	11.421	13.560	21.508	23.747
10	0.2567	17.773	12.383	14.827	25.557	21.834
10	0.2577	15.068	17.322	21.350	16.501	27.532
10	0.2587	11.685	16.002	20.871	8.217	17.210
10	0.2597	20.833	15.752	18.268	17.032	13.388
10	0.2607	18.428	15.852	15.435	29.363	19.563
10	0.2617	8.864	9.744	14.718	9.454	17.233
10	0.2627	14.166	16.543	19.814	13.059	21.393
10	0.2637	27.381	35.989	24.475	17.490	28.208
10	0.2647	13.115	10.335	13.048	6.996	8.768
10	0.2657	3.917	8.751	11.083	4.631	9.037
10	0.2667	9.583	8.171	10.277	7.486	11.022
10	0.2677	10.994	10.992	7.805	6.417	11.457
10	0.2687	3.017	5.149	5.642	10.307	10.527
10	0.2697	5.618	8.375	6.775	5.637	7.123
10	0.2707	10.182	7.411	5.551	9.310	12.469
10	0.2717	2.420	3.950	4.140	11.260	18.997
10	0.2727	6.209	9.551	10.808	9.859	18.102
10	0.2737	6.032	6.607	7.855	8.343	12.749
10	0.2747	1.622	4.344	3.168	8.226	7.502
10	0.2757	15.994	6.209	15.477	10.563	19.885
10	0.2767	9.023	6.142	12.626	8.637	18.334
10	0.2777	9.481	10.438	10.073	11.237	3.796
10	0.2787	9.762	7.901	7.263	11.994	6.879
10	0.2797	13.647	15.708	16.754	12.183	8.799
10	0.2807	20.140	19.905	19.779	20.835	12.389
10	0.2817	9.903	5.634	10.133	12.389	19.577
10	0.2827	12.173	9.996	11.507	5.657	16.661
10	0.2837	14.631	16.047	15.533	11.947	7.356

10	0.2847	13.101	12.065	11.097	15.030	19.530
10	0.2857	10.389	19.921	9.265	21.097	16.637
10	0.2867	12.466	24.491	20.729	23.474	21.266
10	0.2877	10.733	23.164	19.619	15.766	18.276
10	0.2887	7.091	12.698	16.381	12.623	14.285
10	0.2897	11.256	11.550	11.728	20.234	22.508
10	0.2907	6.040	7.462	6.570	12.005	7.704
10	0.2917	19.522	12.612	19.523	9.600	12.692
10	0.2927	12.312	15.715	17.379	6.486	12.273
10	0.2937	14.907	18.840	16.467	19.877	10.167
10	0.2947	11.310	13.655	13.951	24.544	22.189
10	0.2957	14.689	12.692	17.036	17.903	22.119
10	0.2967	15.952	13.220	15.109	11.913	11.286
10	0.2977	16.340	9.022	11.776	11.969	15.151
10	0.2987	17.021	10.766	10.994	13.860	14.309
10	0.2997	16.769	17.464	14.268	18.924	16.361
10	0.3007	17.761	19.384	19.360	19.612	8.021
10	0.3017	15.488	13.965	17.782	7.096	14.623
10	0.3027	7.567	6.188	6.010	3.647	6.675
10	0.3037	15.190	14.533	16.233	9.152	8.656
10	0.3047	16.917	11.267	13.082	11.671	5.944
10	0.3057	18.307	10.113	12.503	14.880	10.384
10	0.3067	7.578	11.389	9.147	17.593	13.911
10	0.3077	14.808	10.936	14.575	15.723	16.243
10	0.3087	8.667	12.925	16.925	13.106	16.193
10	0.3097	7.760	12.883	11.335	14.161	7.913
10	0.3107	12.198	16.724	22.516	4.681	9.989
10	0.3117	16.880	14.306	19.125	11.667	12.221
10	0.3127	21.036	23.409	27.236	9.234	9.178
10	0.3137	22.286	31.412	32.602	11.302	9.922
10	0.3147	14.023	16.591	15.863	4.599	5.525
10	0.3157	19.231	17.538	19.638	15.412	14.515
10	0.3167	12.904	16.457	18.008	10.356	17.169
10	0.3177	5.560	8.018	9.152	15.363	11.529
10	0.3187	11.974	15.191	18.236	14.269	16.570
10	0.3197	9.735	17.455	19.054	13.366	15.719
10	0.3207	9.227	11.412	13.172	6.456	16.135
10	0.3217	4.246	2.017	3.632	1.908	3.894
10	0.3227	11.458	8.608	13.310	10.286	10.716
10	0.3237	14.475	16.884	16.380	9.481	3.797
10	0.3247	12.727	5.605	11.397	8.674	17.452
10	0.3257	11.094	3.278	6.409	7.911	4.520
10	0.3267	16.955	12.278	20.500	9.549	6.197
10	0.3277	24.208	21.425	23.002	15.731	17.744
10	0.3287	11.383	9.534	9.451	0.752	12.351
10	0.3297	5.746	2.007	5.443	3.433	4.440
10	0.3307	9.157	6.722	7.511	10.407	17.836
10	0.3317	7.631	6.870	7.013	10.557	17.487
10	0.3327	10.251	13.058	14.191	13.550	16.900
10	0.3337	9.643	15.314	14.854	12.772	16.776

10	0.3347	7.597	4.819	12.045	4.676	6.744
10	0.3357	6.018	3.598	9.027	8.024	2.439
10	0.3367	12.263	13.245	18.667	12.085	6.270
10	0.3377	25.598	24.746	36.620	15.577	4.191
10	0.3387	25.556	24.753	36.623	15.559	4.192
10	0.3397	19.152	14.188	22.467	6.277	4.868
10	0.3407	16.360	15.912	14.579	7.524	5.008
10	0.3417	22.491	25.852	26.528	7.969	11.758
10	0.3427	9.396	8.853	11.618	16.166	17.486
10	0.3437	10.216	13.077	16.768	11.162	19.761
10	0.3447	7.490	13.863	13.223	19.399	12.248
10	0.3457	7.529	13.926	13.247	19.447	12.236
10	0.3467	14.382	9.796	16.303	16.073	11.681
10	0.3477	16.890	21.730	22.000	16.938	6.272
10	0.3487	14.198	16.055	12.722	19.122	20.131
10	0.3497	6.320	6.249	8.600	7.315	12.505
10	0.3507	4.612	6.374	5.170	11.503	15.851
10	0.3517	13.777	13.256	16.028	9.675	9.224
10	0.3527	15.387	11.268	14.717	7.643	11.357
10	0.3537	25.054	27.189	29.139	13.528	11.499
10	0.3547	28.902	26.267	29.607	12.644	13.250
10	0.3557	20.651	29.988	31.996	6.623	15.575
10	0.3567	14.005	19.425	26.902	7.159	23.408
10	0.3577	20.099	18.190	24.818	16.113	21.543
10	0.3587	16.823	14.867	21.569	16.799	19.861
10	0.3597	7.407	5.585	11.392	9.447	17.274
10	0.3607	17.637	24.426	26.920	11.150	13.277
10	0.3617	18.391	18.591	26.611	5.113	24.092
10	0.3627	12.063	14.030	14.385	5.452	11.766
10	0.3637	6.834	10.026	10.978	5.951	5.285
10	0.3647	8.849	11.162	9.767	10.223	8.562
10	0.3657	9.708	15.476	13.200	10.561	5.537
10	0.3667	23.751	25.095	22.937	15.828	17.318
10	0.3677	16.814	17.499	19.690	16.871	17.943
10	0.3687	14.105	13.575	15.500	11.986	15.643
10	0.3697	18.453	16.065	20.400	12.595	13.588
10	0.3707	27.943	26.432	22.713	12.982	19.345
10	0.3717	22.766	20.683	27.759	10.506	19.396
10	0.3727	22.817	16.093	25.497	10.251	19.510
10	0.3737	12.795	18.099	18.461	9.847	16.539
10	0.3747	12.305	17.209	15.662	10.698	16.003
10	0.3757	8.776	11.095	8.982	7.197	7.711
10	0.3767	4.427	2.571	5.489	9.186	6.783
10	0.3777	7.513	5.627	12.425	10.860	13.859
10	0.3787	14.971	14.203	22.558	9.490	11.088
10	0.3797	7.675	11.353	13.164	5.893	3.610
10	0.3807	4.630	4.533	4.831	8.509	3.375
10	0.3817	7.363	9.997	8.785	9.197	9.888
10	0.3827	8.375	9.638	9.209	8.693	13.744
10	0.3837	12.945	16.340	19.920	14.232	12.676

10	0.3847	14.880	22.298	19.323	10.012	5.234
10	0.3857	10.289	14.026	13.369	10.758	12.360
10	0.3867	16.309	11.996	18.399	7.594	13.054
10	0.3877	14.243	21.724	16.683	16.362	16.124
10	0.3887	18.605	24.970	22.433	14.058	25.630
10	0.3897	6.218	9.072	7.576	15.516	21.135
10	0.3907	6.268	5.977	6.149	7.018	4.882
10	0.3917	7.791	7.796	11.839	5.308	9.272
10	0.3927	10.710	14.606	18.568	15.557	24.298
10	0.3937	10.054	5.982	8.605	12.941	8.525
10	0.3947	5.792	3.370	4.706	8.307	12.139
10	0.3957	11.324	16.058	10.990	9.562	17.925
10	0.3967	12.627	14.853	14.550	7.625	11.739
10	0.3977	8.350	8.578	9.196	15.700	20.674
10	0.3987	3.178	7.649	9.159	12.152	16.651
10	0.3997	9.236	9.277	11.203	6.950	10.338
10	0.4007	18.219	17.284	19.845	9.799	22.446
10	0.4017	9.435	16.086	11.287	15.850	21.170
10	0.4027	14.458	18.176	19.111	19.452	18.509
10	0.4037	11.182	9.057	14.494	13.084	19.644
10	0.4047	12.525	17.988	23.131	8.458	19.431
10	0.4057	10.457	8.468	14.350	15.755	17.273
10	0.4067	11.716	11.145	18.093	13.974	18.723
10	0.4077	13.964	11.446	14.556	8.645	9.285
10	0.4087	21.839	19.430	23.087	19.722	19.641
10	0.4097	26.111	25.485	24.176	7.123	16.755
10	0.4107	21.241	10.395	15.537	9.412	17.090
10	0.4117	16.796	12.468	13.735	15.642	23.311
10	0.4127	4.104	5.646	5.201	5.173	13.885
10	0.4137	2.643	3.773	3.692	5.358	16.170
10	0.4147	1.982	2.776	3.077	3.376	11.597
10	0.4157	6.081	3.302	5.701	9.499	9.659
10	0.4167	4.884	4.487	6.707	10.884	12.958
10	0.4177	2.171	1.998	2.258	4.918	11.493
10	0.4187	7.839	5.691	7.959	12.215	17.809
10	0.4197	10.775	7.837	9.550	11.181	15.946
10	0.4207	11.588	3.191	10.808	6.926	6.007
10	0.4217	15.421	12.953	16.476	8.800	7.160
10	0.4227	17.656	22.025	20.166	13.628	15.189
10	0.4237	11.077	18.523	12.854	11.875	10.333
10	0.4247	3.687	3.961	3.444	5.317	4.896
10	0.4257	1.319	0.872	3.494	1.782	1.981
10	0.4267	6.600	4.732	10.525	4.929	8.348
10	0.4277	17.335	12.723	14.278	9.836	14.803
10	0.4287	16.164	16.492	21.076	8.081	16.384
10	0.4297	14.139	13.707	13.099	8.403	12.060
10	0.4307	7.946	11.094	14.616	7.073	16.974
10	0.4317	10.528	5.934	7.597	4.230	21.616
10	0.4327	10.263	10.441	10.711	3.389	16.755
10	0.4337	5.662	6.907	9.663	6.459	21.840

10	0.4347	7.517	16.855	17.354	12.686	28.830
10	0.4357	7.641	10.959	12.008	10.554	32.315
10	0.4367	8.251	15.968	15.076	7.547	22.488
10	0.4377	5.828	6.129	4.950	7.914	24.826
10	0.4387	8.076	11.135	5.659	18.114	25.192
10	0.4397	11.354	11.597	12.721	14.456	35.852
10	0.4407	6.608	7.512	8.924	15.026	28.408
10	0.4417	10.067	10.556	11.430	14.662	35.802
10	0.4427	5.317	3.387	6.358	6.551	9.046
10	0.4437	5.963	3.373	7.009	6.603	8.153
10	0.4447	7.326	8.779	6.557	9.380	4.251
10	0.4457	12.544	12.246	8.914	11.393	10.498
10	0.4467	11.068	21.732	20.936	10.480	13.778
10	0.4477	16.092	14.448	14.223	11.440	16.364
10	0.4487	21.464	16.651	14.606	9.512	24.642
10	0.4497	18.405	15.857	16.212	12.822	22.162
10	0.4507	11.898	18.675	14.684	13.272	32.329
10	0.4517	10.114	7.125	12.813	16.635	30.595
10	0.4527	13.629	12.602	11.022	10.111	24.094
10	0.4537	5.997	5.412	5.413	11.008	21.669
10	0.4547	5.997	4.720	5.947	9.784	10.495
10	0.4557	9.582	9.702	6.973	5.567	5.627
10	0.4567	34.825	31.919	26.273	7.900	10.243
10	0.4577	6.619	16.702	9.783	14.308	28.214
10	0.4587	14.927	32.242	18.671	20.010	19.107
10	0.4597	13.533	16.734	12.889	21.824	18.228
10	0.4607	7.807	6.375	6.970	12.886	13.915
10	0.4617	14.089	6.297	11.548	18.873	18.555
10	0.4627	13.623	11.941	9.895	15.655	29.702
10	0.4637	4.316	8.089	4.966	16.054	26.257
10	0.4647	8.361	8.541	6.834	13.800	24.285
10	0.4657	19.747	27.720	13.073	21.954	14.691
10	0.4667	15.484	11.066	10.044	18.201	26.732
10	0.4677	19.168	16.218	17.448	22.796	21.119
10	0.4687	5.486	4.705	6.511	27.188	27.222
10	0.4697	9.597	9.496	9.728	12.536	25.839
10	0.4707	11.578	7.136	11.583	7.138	19.847
10	0.4717	6.669	6.904	7.081	11.445	20.119
10	0.4727	18.956	23.310	16.406	6.661	20.264
10	0.4737	6.593	14.964	10.224	14.428	24.871
10	0.4747	11.227	11.765	10.450	3.909	8.400
10	0.4757	7.583	5.348	5.372	4.748	9.589
10	0.4767	6.474	5.452	5.077	3.332	4.455
10	0.4777	4.403	10.050	4.222	3.367	10.412
10	0.4787	10.702	20.105	10.890	11.453	23.003
10	0.4797	5.868	7.082	4.618	7.509	24.793
10	0.4807	7.701	7.035	5.933	6.756	24.328
10	0.4817	7.361	9.355	4.030	18.260	24.184
10	0.4827	10.922	9.356	7.931	21.857	30.019
10	0.4837	16.731	9.753	12.163	10.041	12.857

10	0.4847	14.402	9.161	12.924	4.650	17.428
10	0.4857	8.554	10.222	8.486	12.254	17.882
10	0.4867	9.554	7.375	6.636	14.084	32.349
10	0.4877	4.443	7.175	4.882	12.066	19.177
10	0.4887	12.756	20.891	15.351	12.411	20.020
10	0.4897	13.245	20.356	18.337	9.752	13.473
10	0.4907	23.721	14.776	13.646	3.531	16.388
10	0.4917	23.835	14.829	13.704	3.524	16.451
10	0.4927	12.134	4.941	7.808	6.766	13.091
10	0.4937	9.106	3.288	7.348	8.617	11.344
10	0.4947	3.572	1.785	3.069	3.316	4.835
10	0.4957	13.652	9.787	11.547	8.230	21.080
10	0.4967	9.846	11.224	8.859	16.517	27.937
10	0.4977	9.471	7.922	6.690	14.640	15.600
10	0.4987	3.878	7.935	3.929	9.815	10.774
10	0.4997	8.712	9.661	11.734	4.254	15.078
10	0.5007	3.306	5.242	2.640	15.561	25.536
10	0.5017	7.365	6.664	6.450	7.923	14.014
10	0.5027	6.646	5.460	5.988	6.551	7.893
10	0.5037	3.046	3.213	3.057	4.343	4.019
10	0.5047	5.857	9.086	10.574	9.795	21.818
10	0.5057	5.863	9.092	10.560	9.793	21.705
10	0.5067	4.717	9.620	4.571	14.036	18.725
10	0.5077	9.738	13.840	5.426	31.681	17.099
10	0.5087	5.751	7.956	3.908	18.354	21.619
10	0.5097	28.753	24.333	24.854	15.275	12.404
10	0.5107	13.886	8.994	12.631	7.305	11.253
10	0.5117	7.956	6.818	5.110	9.309	8.707
10	0.5127	11.150	8.059	6.160	9.779	12.748
10	0.5137	14.342	15.893	14.222	5.940	13.655
10	0.5147	5.982	10.339	9.564	16.430	19.967
10	0.5157	9.945	10.545	10.492	21.357	28.056
10	0.5167	9.409	9.259	6.254	10.410	20.401
10	0.5177	12.390	13.551	8.145	7.879	15.248
10	0.5187	23.767	20.305	18.495	13.139	14.753
10	0.5197	16.690	12.313	8.566	9.315	19.228
10	0.5207	19.117	19.479	12.890	6.146	9.871
10	0.5217	14.543	16.899	11.701	11.799	7.703
10	0.5227	13.532	22.047	10.332	19.100	19.811
10	0.5237	15.228	25.609	14.026	16.673	7.493
10	0.5247	25.365	26.241	15.957	13.390	16.356
10	0.5257	25.401	26.212	15.929	13.394	16.392
10	0.5267	17.805	21.808	16.422	9.160	17.786
10	0.5277	8.858	11.472	13.659	15.905	13.281
10	0.5287	8.870	11.365	13.499	15.818	12.908
10	0.5297	6.194	8.953	11.576	14.528	15.023
10	0.5307	6.265	14.237	14.136	9.347	10.792
10	0.5317	6.265	14.229	14.133	9.351	10.790
10	0.5327	14.123	15.681	17.137	13.944	19.128
10	0.5337	15.309	13.970	15.758	10.228	7.613

10	0.5347	15.317	13.941	15.817	10.209	7.575
10	0.5357	15.324	13.912	15.876	10.189	7.537
10	0.5367	12.182	19.209	12.822	10.806	7.304
10	0.5377	12.385	20.915	12.258	14.637	19.254
10	0.5387	11.949	9.560	9.401	2.674	3.126
10	0.5397	17.030	22.283	14.344	4.690	14.151
10	0.5407	24.848	21.919	19.727	12.260	26.780
10	0.5417	21.721	27.845	22.246	11.361	27.958
10	0.5427	18.315	29.913	18.950	22.431	36.718
10	0.5437	17.228	34.787	19.032	23.084	34.918
10	0.5447	13.980	21.578	16.081	10.276	10.847
10	0.5457	14.987	16.076	21.055	7.060	14.151
10	0.5467	14.987	16.074	21.052	7.061	14.167
10	0.5477	13.408	19.827	11.007	4.860	17.737
10	0.5487	12.945	15.929	6.045	16.864	17.995
10	0.5497	13.773	12.661	9.601	5.353	8.042
10	0.5507	13.938	21.405	10.965	19.421	19.449
10	0.5517	4.869	18.334	13.223	18.504	14.083
10	0.5527	11.982	18.577	13.278	9.984	21.684
10	0.5537	15.886	17.850	16.206	10.882	14.698
10	0.5547	5.058	5.679	10.678	13.324	11.691
10	0.5557	6.349	13.029	8.134	15.040	13.442
10	0.5567	9.592	19.892	13.213	11.488	13.305
10	0.5577	9.762	15.635	8.346	6.747	20.673
10	0.5587	6.496	3.170	2.813	7.529	14.822
10	0.5597	7.478	4.433	3.614	8.644	16.379
10	0.5607	9.705	14.008	8.319	5.935	12.213
10	0.5617	9.197	8.693	6.337	5.779	13.604
10	0.5627	3.775	4.964	8.360	3.579	13.936
10	0.5637	9.003	12.022	11.165	4.255	7.119
10	0.5647	6.233	5.941	3.977	4.056	5.365
10	0.5657	5.938	3.883	4.046	2.914	4.350
10	0.5667	9.654	6.845	8.380	9.371	11.113
10	0.5677	19.666	14.120	17.213	5.499	19.950
10	0.5687	21.617	22.432	27.532	10.721	23.664
10	0.5697	21.627	22.399	27.418	10.652	23.612
10	0.5707	7.639	13.263	11.059	11.538	33.101
10	0.5717	7.662	13.140	10.956	11.491	32.961
10	0.5727	7.419	16.520	7.125	8.003	26.148
10	0.5737	13.778	11.308	12.328	5.411	7.299
10	0.5747	15.639	22.359	13.189	11.397	28.768
10	0.5757	12.928	19.437	12.002	18.947	30.057
10	0.5767	11.677	7.159	9.437	12.648	20.237
10	0.5777	14.300	16.285	12.528	13.053	13.823
10	0.5787	13.359	17.049	16.383	11.023	10.141
10	0.5797	16.593	23.130	23.414	10.042	19.855
10	0.5807	12.862	7.095	10.613	10.612	17.696
10	0.5817	12.998	7.180	10.740	10.604	17.556
10	0.5827	8.636	9.274	8.475	17.180	10.179
10	0.5837	8.613	9.231	8.436	17.176	10.147

10	0.5847	10.978	8.670	11.030	11.343	22.661
10	0.5857	12.536	7.908	11.835	15.870	21.887
10	0.5867	17.842	16.814	16.146	10.636	25.771
10	0.5877	14.393	8.771	17.953	14.855	16.872
10	0.5887	20.666	24.871	24.276	12.565	32.362
10	0.5897	10.153	9.696	8.361	10.621	11.444
10	0.5907	8.514	9.447	9.317	24.540	21.977
10	0.5917	8.879	12.525	9.560	15.636	28.899
10	0.5927	14.765	17.390	15.408	19.881	24.028
10	0.5937	14.983	15.358	16.881	18.721	20.603
10	0.5947	4.321	5.686	8.958	8.389	8.666
10	0.5957	6.167	11.532	5.535	8.134	16.792
10	0.5967	16.458	13.967	11.494	8.425	18.664
10	0.5977	7.775	9.459	10.591	13.868	10.817
10	0.5987	7.787	6.368	11.138	15.979	4.133
10	0.5997	8.358	7.589	6.691	20.534	15.075
10	0.6007	16.444	14.609	17.717	20.078	10.261
10	0.6017	6.592	15.859	10.588	12.261	12.574
10	0.6027	12.759	15.286	14.601	23.498	17.795
10	0.6037	14.187	11.962	14.702	6.923	14.486
10	0.6047	16.500	11.272	17.035	5.580	12.999
10	0.6057	18.736	17.900	24.927	5.749	17.002
10	0.6067	5.280	9.115	6.292	15.132	5.813
10	0.6077	9.204	13.934	12.302	22.653	9.125
10	0.6087	9.155	10.295	11.741	15.585	21.789
10	0.6097	16.333	20.428	17.964	11.188	13.741
10	0.6107	10.197	8.303	15.180	12.313	9.619
10	0.6117	13.150	9.645	12.897	5.347	13.742
10	0.6127	20.029	18.378	25.626	7.604	18.246
10	0.6137	18.250	14.949	22.425	8.329	17.125
10	0.6147	14.931	11.702	14.431	9.199	21.576
10	0.6157	11.694	12.923	20.258	15.891	18.350
10	0.6167	8.129	10.031	17.591	11.025	26.003
10	0.6177	8.186	10.135	17.717	11.019	25.976
10	0.6187	9.064	11.262	14.097	6.075	9.911
10	0.6197	14.907	12.268	12.013	9.901	10.952
10	0.6207	24.738	16.012	25.404	9.740	12.095
10	0.6217	22.602	14.718	18.768	12.812	18.036
10	0.6227	13.741	12.186	20.260	13.692	34.933
10	0.6237	9.277	14.896	18.635	6.614	9.066
10	0.6247	12.979	12.556	14.896	13.795	11.161
10	0.6257	9.847	9.736	13.546	8.892	11.769
10	0.6267	13.190	9.986	15.405	9.842	14.357
10	0.6277	11.957	8.144	16.628	18.691	7.721
10	0.6287	13.430	6.122	16.928	14.233	15.866
10	0.6297	9.357	8.649	11.505	13.800	18.748
10	0.6307	7.393	7.077	8.479	4.777	12.552
10	0.6317	18.449	22.649	18.171	12.183	17.617
10	0.6327	8.691	6.495	7.831	10.381	8.346
10	0.6337	12.550	11.092	12.448	2.742	6.647

10	0.6347	7.995	6.249	5.614	10.619	8.734
10	0.6357	9.822	9.786	18.750	10.346	13.294
10	0.6367	12.487	9.009	17.623	13.386	13.584
10	0.6377	9.509	14.776	10.601	17.382	12.480
10	0.6387	18.399	21.133	23.045	11.377	12.803
10	0.6397	8.437	10.311	15.877	11.175	12.782
10	0.6407	11.572	18.661	14.753	16.970	21.032
10	0.6417	6.531	12.230	6.462	10.405	18.922
10	0.6427	12.252	21.268	14.107	10.324	24.685
10	0.6437	9.070	11.607	13.415	16.044	18.397
10	0.6447	9.242	13.024	12.624	13.139	9.035
10	0.6457	17.126	12.695	21.524	5.966	14.129
10	0.6467	13.367	13.797	21.826	10.491	7.427
10	0.6477	14.516	18.804	17.697	7.180	8.069
10	0.6487	9.551	16.852	13.123	26.638	18.197
10	0.6497	11.698	12.536	12.084	17.166	6.761
10	0.6507	7.067	9.166	5.411	13.534	10.141
10	0.6517	16.774	15.087	15.980	7.469	7.680
10	0.6527	12.338	12.601	16.848	4.053	22.562
10	0.6537	9.382	10.072	16.040	10.072	28.961
10	0.6547	5.365	10.191	8.787	13.751	24.717
10	0.6557	8.336	11.760	12.899	10.994	9.548
10	0.6567	9.577	4.646	12.279	7.422	2.147
10	0.6577	11.728	10.545	13.221	17.085	14.650
10	0.6587	14.271	16.394	17.350	14.208	9.269
10	0.6597	10.414	14.089	11.866	25.233	9.623
10	0.6607	8.329	13.128	9.034	10.126	11.115
10	0.6617	18.890	18.105	17.117	11.813	33.114
10	0.6627	15.945	11.892	11.852	8.351	17.362
10	0.6637	9.282	8.109	7.310	10.424	14.811
10	0.6647	9.296	8.135	7.349	10.478	14.770
10	0.6657	21.428	11.916	20.595	12.610	19.702
10	0.6667	20.525	12.407	20.922	9.149	6.422
10	0.6677	19.062	8.306	16.335	11.782	8.278
10	0.6687	10.149	12.096	9.107	20.513	9.856
10	0.6697	12.775	18.519	22.248	17.391	17.771
10	0.6707	12.773	18.528	22.262	17.264	17.681
10	0.6717	13.118	13.092	10.568	16.031	26.538
10	0.6727	15.567	18.676	15.203	21.427	26.116
10	0.6737	14.025	10.467	12.818	13.291	19.201
10	0.6747	20.494	6.665	13.977	12.452	24.114
10	0.6757	10.670	10.726	10.700	16.098	21.731
10	0.6767	11.087	18.791	14.174	23.440	12.227
10	0.6777	12.387	13.982	10.916	7.220	13.060
10	0.6787	12.564	11.655	13.304	7.150	6.389
10	0.6797	13.221	6.992	10.642	9.410	10.368
10	0.6807	14.551	11.064	6.505	16.151	24.329
10	0.6817	9.610	11.580	8.498	23.291	29.366
10	0.6827	12.653	13.226	10.723	24.994	26.858
10	0.6837	17.557	13.459	16.046	11.876	11.762

10	0.6847	15.770	14.819	17.229	8.896	4.998
10	0.6857	10.984	12.470	10.728	11.334	23.511
10	0.6867	7.559	5.815	4.910	5.129	25.904
10	0.6877	14.754	13.439	8.306	12.034	23.695
10	0.6887	16.450	15.100	13.343	18.597	15.177
10	0.6897	10.729	12.904	7.673	15.125	10.503
10	0.6907	6.625	9.067	10.249	5.118	6.228
10	0.6917	3.588	3.987	4.603	4.701	1.789
10	0.6927	14.837	13.639	10.365	7.586	8.603
10	0.6937	10.980	7.839	6.971	14.356	17.321
10	0.6947	11.932	13.137	12.718	14.599	17.589
10	0.6957	16.943	11.956	12.530	11.503	18.916
10	0.6967	10.609	7.434	9.460	12.510	11.489
10	0.6977	12.356	8.601	6.505	13.138	11.093
10	0.6987	12.190	7.985	20.544	10.540	16.123
10	0.6997	17.628	17.440	20.680	16.216	19.364
10	0.7007	18.224	15.104	20.329	15.216	20.922
10	0.7017	28.542	11.413	12.830	19.475	23.152
10	0.7027	5.281	6.232	6.315	19.993	13.025
10	0.7037	5.240	12.240	6.279	17.238	8.518
10	0.7047	5.824	8.660	2.428	24.559	12.371
10	0.7057	11.716	12.556	7.113	9.668	15.803
10	0.7067	9.238	9.450	7.399	16.027	12.476
10	0.7077	6.359	11.465	8.386	17.529	15.962
10	0.7087	19.989	10.262	9.211	7.832	12.078
10	0.7097	29.586	11.780	17.975	18.035	16.086
10	0.7107	12.854	7.779	6.691	19.670	18.636
10	0.7117	9.130	9.949	10.869	4.872	15.571
10	0.7127	5.932	7.063	6.869	5.257	8.862
10	0.7137	22.544	15.738	23.609	8.826	21.246
10	0.7147	20.221	16.312	21.168	7.753	6.454
10	0.7157	14.316	23.662	18.813	11.955	19.159
10	0.7167	6.911	4.616	7.220	14.157	22.093
10	0.7177	16.076	19.729	18.770	7.241	14.893
10	0.7187	16.150	19.739	18.806	7.276	14.915
10	0.7197	16.491	16.414	19.318	10.433	17.510
10	0.7207	16.572	13.402	11.780	6.040	7.450
10	0.7217	9.556	7.904	8.848	6.910	13.857
10	0.7227	21.568	7.305	17.886	16.247	21.035
10	0.7237	10.986	8.063	10.469	17.087	8.313
10	0.7247	8.939	13.837	11.678	6.893	16.250
10	0.7257	7.764	8.223	7.705	10.288	30.418
10	0.7267	9.932	5.963	7.020	10.171	17.015
10	0.7277	9.243	5.045	4.636	11.011	12.532
10	0.7287	8.982	14.386	12.569	14.740	18.547
10	0.7297	21.425	14.750	14.900	14.207	19.808
10	0.7307	12.187	9.282	14.373	5.459	30.087
10	0.7317	11.390	12.653	13.311	6.951	13.345
10	0.7327	17.131	10.562	13.010	10.555	17.431
10	0.7337	17.122	20.568	24.080	10.845	26.494

10	0.7347	16.704	9.537	18.683	4.666	22.006
10	0.7357	9.636	2.040	3.681	5.581	23.382
10	0.7367	25.336	12.520	13.009	11.260	21.548
10	0.7377	12.310	3.936	4.698	9.125	13.281
10	0.7387	16.314	10.786	12.307	7.411	10.780
10	0.7397	14.044	9.446	8.192	5.795	14.092
10	0.7407	21.125	12.520	13.486	11.508	26.630
10	0.7417	16.585	8.073	16.414	9.095	24.279
10	0.7427	14.882	9.167	9.719	18.776	34.983
10	0.7437	15.131	12.443	13.120	14.354	21.153
10	0.7447	11.593	9.439	15.269	9.593	25.375
10	0.7457	12.260	5.369	6.357	7.399	11.647
10	0.7467	16.275	5.774	7.336	13.276	15.896
10	0.7477	9.872	3.109	5.771	12.822	35.716
10	0.7487	5.945	1.855	3.138	4.591	13.316
10	0.7497	14.615	7.799	12.177	15.964	24.189
10	0.7507	7.467	4.003	4.721	11.694	18.102
10	0.7517	13.235	17.733	10.228	18.355	23.681
10	0.7527	10.794	10.911	9.616	14.728	19.841
10	0.7537	13.811	11.948	13.454	6.249	11.251
10	0.7547	5.784	8.787	8.261	6.804	8.437
10	0.7557	9.653	15.067	9.359	11.935	13.163
10	0.7567	9.374	9.658	10.548	10.420	12.182
10	0.7577	24.817	17.341	20.313	12.000	16.127
10	0.7587	22.563	16.880	15.682	13.071	20.412
10	0.7597	9.232	8.292	9.697	9.745	12.090
10	0.7607	15.954	13.163	10.204	21.806	18.071
10	0.7617	22.834	18.426	11.555	8.092	8.345
10	0.7627	16.201	18.221	20.635	22.516	12.459
10	0.7637	15.191	7.446	9.793	6.580	12.937
10	0.7647	6.337	5.363	4.467	6.677	18.596
10	0.7657	5.273	11.326	5.099	24.670	22.934
10	0.7667	18.449	5.778	11.106	19.245	18.097
10	0.7677	22.990	23.090	19.320	14.629	24.215
10	0.7687	28.589	21.361	23.126	17.646	16.029
10	0.7697	7.378	9.932	5.897	21.457	17.926
10	0.7707	21.609	25.697	15.641	21.805	19.453
10	0.7717	11.034	13.527	9.269	16.863	18.994
10	0.7727	7.181	6.091	5.838	8.060	7.949
10	0.7737	17.502	13.129	19.164	16.661	13.841
10	0.7747	38.455	32.031	37.788	12.071	15.907
10	0.7757	29.318	29.574	29.225	11.242	15.579
10	0.7767	16.533	17.170	16.798	11.097	17.568
10	0.7777	17.074	16.510	16.541	10.340	24.123
10	0.7787	20.877	21.757	20.173	9.214	18.656
10	0.7797	22.180	22.162	19.884	13.569	10.886
10	0.7807	27.902	22.189	17.930	22.764	20.352
10	0.7817	21.651	16.750	19.836	23.649	9.795
10	0.7827	21.584	16.741	19.789	23.521	9.739
10	0.7837	17.770	15.232	14.089	8.485	15.664

10	0.7847	5.086	5.206	3.945	13.783	10.746
10	0.7857	5.157	6.204	4.382	8.524	12.075
10	0.7867	9.784	9.465	5.841	8.648	21.581
10	0.7877	17.944	11.657	11.595	16.217	30.599
10	0.7887	25.377	15.964	16.696	16.050	24.911
10	0.7897	25.375	15.970	16.704	15.991	24.913
10	0.7907	21.191	23.944	19.779	14.866	16.245
10	0.7917	7.856	15.202	10.200	17.912	15.999
10	0.7927	11.607	9.581	11.859	7.565	10.777
10	0.7937	26.236	24.122	16.137	24.825	20.701
10	0.7947	18.176	17.944	14.728	13.681	12.860
10	0.7957	7.608	9.713	7.726	16.170	14.470
10	0.7967	2.573	4.469	3.391	13.887	19.142
10	0.7977	9.784	7.206	10.636	7.469	25.169
10	0.7987	17.116	17.071	20.256	14.384	10.047
10	0.7997	23.380	14.975	16.125	12.628	14.092
10	0.8007	10.090	13.272	12.951	13.963	8.435
10	0.8017	14.122	16.348	13.692	11.491	17.757
10	0.8027	16.725	13.769	15.239	7.696	12.516
10	0.8037	16.290	17.012	9.493	12.897	17.845
10	0.8047	14.950	15.442	13.184	7.021	15.324
10	0.8057	13.794	18.727	12.647	13.689	20.002
10	0.8067	5.012	6.575	4.454	6.732	6.107
10	0.8077	8.576	7.510	13.005	15.949	11.905
10	0.8087	4.311	6.212	5.513	13.424	4.409
10	0.8097	6.638	6.088	5.246	5.348	5.783
10	0.8107	12.279	12.092	10.958	10.129	11.522
10	0.8117	16.840	16.646	14.323	14.920	15.815
10	0.8127	16.922	19.293	17.170	14.663	16.001
10	0.8137	11.906	10.070	11.945	8.953	15.969
10	0.8147	4.524	12.562	8.166	13.067	9.762
10	0.8157	7.949	8.710	9.949	5.650	8.037
10	0.8167	13.521	9.494	11.360	14.015	31.760
10	0.8177	11.988	8.164	13.515	6.534	12.406
10	0.8187	17.756	14.183	17.100	10.865	14.156
10	0.8197	10.673	8.175	7.723	11.088	15.557
10	0.8207	6.718	5.760	8.645	13.856	12.087
10	0.8217	6.698	5.727	8.656	13.855	12.027
10	0.8227	7.622	9.070	7.669	10.363	20.491
10	0.8237	8.163	8.893	12.630	19.641	23.813
10	0.8247	8.148	8.889	12.611	19.606	23.796
10	0.8257	4.808	2.239	6.414	9.169	9.919
10	0.8267	12.895	8.401	12.898	3.199	9.380
10	0.8277	5.091	9.147	9.174	13.463	8.510
10	0.8287	11.899	15.804	13.504	21.973	12.285
10	0.8297	8.785	6.622	6.559	14.425	18.312
10	0.8307	6.201	4.914	6.057	13.166	12.968
10	0.8317	8.601	10.192	11.164	20.462	19.360
10	0.8327	12.700	8.518	12.686	13.449	19.384
10	0.8337	5.497	7.503	5.010	12.416	9.416

10	0.8347	10.053	16.075	12.008	10.714	19.192
10	0.8357	10.413	12.075	12.802	12.891	16.009
10	0.8367	8.110	16.913	11.331	22.318	9.960
10	0.8377	12.132	18.350	21.678	17.115	9.029
10	0.8387	11.437	12.378	10.306	16.785	11.992
10	0.8397	18.920	15.085	15.212	13.990	16.711
10	0.8407	8.605	9.987	11.157	14.520	27.020
10	0.8417	10.991	16.984	14.219	19.218	25.572
10	0.8427	14.684	11.881	12.466	19.507	19.194
10	0.8437	13.318	9.948	11.815	13.936	18.230
10	0.8447	14.808	11.550	11.674	8.236	23.177
10	0.8457	18.025	9.943	9.026	11.017	16.954
10	0.8467	11.087	6.630	5.686	10.828	17.076
10	0.8477	14.544	13.403	9.345	15.743	15.956
10	0.8487	14.507	13.365	11.053	21.502	12.747
10	0.8497	8.149	9.635	7.147	16.681	13.099
10	0.8507	21.328	14.155	19.094	23.227	12.308
10	0.8517	23.419	12.993	19.162	20.368	14.043
10	0.8527	24.072	14.035	22.667	18.999	13.018
10	0.8537	18.054	9.162	17.256	16.795	16.396
10	0.8547	17.060	14.046	11.715	19.484	12.668
10	0.8557	24.756	26.122	20.010	21.167	22.875
10	0.8567	18.420	13.127	11.587	24.973	29.373
10	0.8577	10.002	10.539	5.419	14.322	32.358
10	0.8587	15.469	8.770	11.286	14.675	28.529
10	0.8597	21.558	13.042	12.201	21.870	22.805
10	0.8607	22.343	14.369	14.253	17.264	20.631
10	0.8617	26.404	19.810	23.645	9.677	11.030
10	0.8627	16.629	22.714	13.736	13.214	21.287
10	0.8637	9.856	13.276	10.330	10.128	13.029
10	0.8647	26.231	22.729	19.932	19.605	20.304
10	0.8657	19.417	18.607	14.257	26.353	21.255
10	0.8667	19.402	13.721	15.154	27.183	29.622
10	0.8677	20.848	16.711	16.145	24.898	33.670
10	0.8687	16.759	20.617	11.280	36.014	24.873
10	0.8697	22.600	22.597	16.860	23.742	22.763
10	0.8707	30.763	28.183	23.214	22.556	17.335
10	0.8717	17.120	19.160	21.848	25.537	18.688
10	0.8727	12.756	26.761	15.397	31.851	34.161
10	0.8737	21.748	23.579	12.950	36.661	32.266
10	0.8747	27.237	30.200	19.194	33.864	29.627
10	0.8757	16.738	12.107	11.396	27.903	43.932
10	0.8767	24.717	14.968	17.262	16.032	13.253
10	0.8777	39.310	20.166	28.703	25.349	20.450
10	0.8787	10.029	11.092	9.243	21.889	26.355
10	0.8797	16.317	13.668	12.715	17.688	12.430
10	0.8807	7.166	5.290	11.022	4.906	4.266
10	0.8817	10.490	8.397	10.147	17.049	14.526
10	0.8827	7.058	5.179	7.152	8.620	11.403
10	0.8837	12.437	10.017	12.160	12.455	25.828

10	0.8847	10.932	9.097	10.816	10.964	26.623
10	0.8857	8.637	6.888	8.919	3.594	6.489
10	0.8867	5.905	9.465	6.496	6.182	10.000
10	0.8877	6.933	14.586	8.832	21.493	24.947
10	0.8887	12.336	15.588	12.635	23.721	28.595
10	0.8897	21.562	6.697	14.338	22.801	10.743
10	0.8907	19.087	5.673	11.575	24.520	13.576
10	0.8917	21.157	12.189	17.581	13.132	23.902
10	0.8927	23.640	15.141	18.189	12.968	23.595
10	0.8937	20.136	11.648	12.991	14.479	23.714
10	0.8947	16.395	15.915	18.322	11.111	19.088
10	0.8957	21.467	12.907	16.470	16.452	13.548
10	0.8967	17.251	10.292	9.168	12.726	13.746
10	0.8977	23.749	11.860	16.487	23.509	16.271
10	0.8987	13.050	17.608	16.237	21.114	21.968
10	0.8997	14.841	14.052	15.834	17.713	14.116
10	0.9007	14.844	14.043	15.817	17.712	14.108
10	0.9017	13.507	13.734	10.840	12.122	13.288
10	0.9027	9.504	6.746	4.201	26.866	13.946
10	0.9037	24.157	15.662	18.863	20.646	27.021
10	0.9047	24.457	19.557	19.156	16.026	18.441
10	0.9057	15.548	11.142	14.901	21.006	16.109
10	0.9067	15.153	9.706	16.285	8.882	12.215
10	0.9077	13.979	7.295	13.512	11.670	7.655
10	0.9087	13.947	7.282	13.538	11.724	7.606
10	0.9097	5.440	9.890	7.149	16.314	9.880
10	0.9107	11.633	11.550	10.563	14.982	17.055
10	0.9117	21.604	20.598	20.220	16.599	10.666
10	0.9127	7.227	8.817	10.985	11.837	13.719
10	0.9137	13.951	14.737	14.605	15.689	17.992
10	0.9147	17.962	16.384	17.813	15.531	15.186
10	0.9157	18.549	9.062	17.244	13.521	11.563
10	0.9167	10.462	11.893	10.433	10.934	11.843
10	0.9177	11.354	13.460	12.563	14.221	8.243
10	0.9187	8.577	11.960	6.939	15.847	6.511
10	0.9197	7.246	17.080	11.187	14.956	6.681
10	0.9207	16.271	26.968	22.292	14.531	15.311
10	0.9217	13.033	16.517	19.658	4.731	13.380
10	0.9227	5.451	6.639	6.831	8.566	8.204
10	0.9237	11.443	12.768	14.702	13.585	17.838
10	0.9247	18.618	12.071	9.081	21.862	15.508
10	0.9257	34.179	26.364	35.814	14.203	11.131
10	0.9267	34.186	26.365	35.811	14.164	11.139
10	0.9277	18.138	18.214	16.202	5.633	9.433
10	0.9287	13.670	20.399	15.101	16.243	12.033
10	0.9297	13.816	23.860	18.355	19.079	9.909
10	0.9307	12.147	22.844	15.481	19.921	8.214
10	0.9317	14.235	16.166	16.958	16.748	8.589
10	0.9327	5.466	9.291	8.132	3.915	12.170
10	0.9337	6.995	11.563	12.002	13.362	7.497

10	0.9347	9.019	14.235	10.908	12.828	11.524
10	0.9357	5.287	6.835	7.951	14.367	7.409
10	0.9367	9.530	11.638	10.391	18.561	21.404
10	0.9377	11.459	11.684	10.061	12.772	12.252
10	0.9387	12.024	9.426	9.071	16.174	11.767
10	0.9397	14.649	15.763	15.036	17.074	24.169
10	0.9407	15.106	9.621	9.904	17.788	14.743
10	0.9417	20.025	19.461	16.470	14.913	13.629
10	0.9427	10.907	12.901	9.406	13.654	12.740
10	0.9437	17.562	12.508	9.116	14.880	15.384
10	0.9447	21.445	31.843	21.212	26.484	16.925
10	0.9457	20.036	21.732	14.215	21.770	9.198
10	0.9467	9.306	12.335	8.675	15.870	18.930
10	0.9477	17.605	15.878	17.140	8.896	23.666
10	0.9487	11.856	17.770	18.402	14.149	14.090
10	0.9497	9.466	23.917	13.753	19.818	6.201
10	0.9507	41.472	35.529	23.587	26.382	22.265
10	0.9517	17.507	26.418	22.088	26.084	15.222
10	0.9527	7.958	12.839	12.057	23.335	11.991
10	0.9537	10.629	11.976	9.090	16.958	11.798
10	0.9547	5.964	6.211	5.897	17.122	17.445
10	0.9557	16.174	32.056	20.598	24.555	16.512
10	0.9567	8.269	10.814	6.039	32.602	21.138
10	0.9577	11.359	15.735	10.223	16.317	18.578
10	0.9587	12.419	10.569	13.734	12.392	15.919
10	0.9597	21.350	20.479	20.057	21.938	10.194
10	0.9607	28.734	21.630	18.487	18.892	17.234
10	0.9617	18.843	18.129	14.860	20.225	12.124
10	0.9627	19.452	14.575	10.775	21.191	11.373
10	0.9637	12.238	8.966	4.037	17.130	11.481
10	0.9647	12.783	12.223	9.076	24.801	21.163
10	0.9657	12.500	19.514	14.061	17.834	22.752
10	0.9667	19.612	28.096	10.392	22.130	16.561
10	0.9677	11.128	17.800	15.954	20.295	16.596
10	0.9687	8.051	10.767	5.008	19.298	19.084
10	0.9697	34.869	20.974	19.410	13.028	18.506
10	0.9707	18.603	17.472	14.721	13.487	19.232
10	0.9717	15.740	23.964	12.504	24.412	13.573
10	0.9727	9.511	17.582	7.220	16.262	14.910
10	0.9737	15.782	22.733	15.910	23.722	21.035
10	0.9747	16.518	18.947	22.280	16.551	20.814
10	0.9757	8.609	16.286	7.405	21.498	28.263
10	0.9767	9.186	14.602	12.773	14.811	12.805
10	0.9777	13.592	19.657	10.860	15.606	13.129
10	0.9787	9.638	17.892	11.767	14.593	7.402
10	0.9797	11.182	21.310	11.958	19.869	12.394
10	0.9807	9.770	19.675	12.963	26.980	11.397
10	0.9817	11.665	14.777	15.353	15.093	21.919
10	0.9827	9.606	15.767	13.177	21.026	14.535
10	0.9837	12.460	17.649	10.345	21.343	25.728

10	0.9847	6.286	15.918	11.005	14.797	12.898
10	0.9857	5.393	12.605	10.089	17.891	15.304
10	0.9867	2.814	6.161	5.682	13.622	19.610
10	0.9877	10.123	9.862	7.154	11.363	12.691
10	0.9887	6.892	17.859	9.939	16.736	8.925
10	0.9897	0.000	0.000	0.000	0.000	0.000
11	0.0020	0.000	0.000	0.000	0.000	0.000
11	0.0030	6.164	5.229	7.360	4.997	9.739
11	0.0040	13.844	7.676	11.278	13.218	19.586
11	0.0050	10.230	8.648	9.066	5.601	13.438
11	0.0060	12.425	8.286	12.347	7.377	10.684
11	0.0070	19.110	10.845	15.420	8.281	15.528
11	0.0080	9.451	4.367	4.142	7.694	12.903
11	0.0090	9.473	5.772	9.354	9.263	13.495
11	0.0100	11.850	9.677	19.125	13.412	10.278
11	0.0110	7.240	9.763	12.536	13.790	34.930
11	0.0120	9.375	12.322	15.840	12.576	17.308
11	0.0130	12.489	13.779	10.774	10.466	5.540
11	0.0140	14.454	9.059	11.786	6.785	5.495
11	0.0150	14.450	9.039	11.788	6.736	5.453
11	0.0160	11.239	6.756	8.482	11.919	8.120
11	0.0170	9.429	11.363	10.226	11.574	12.395
11	0.0180	15.297	21.267	17.303	22.816	20.182
11	0.0190	12.827	9.848	10.305	10.928	11.229
11	0.0200	9.043	10.196	11.144	16.547	16.712
11	0.0210	11.866	15.791	13.219	20.915	13.266
11	0.0220	11.419	9.735	8.514	16.901	11.749
11	0.0230	8.468	15.670	17.624	6.059	6.396
11	0.0240	12.314	9.913	17.534	6.695	10.152
11	0.0250	13.250	10.372	18.401	11.857	8.923
11	0.0260	17.597	18.959	20.305	11.691	19.428
11	0.0270	9.539	12.543	11.652	17.973	15.980
11	0.0280	11.811	7.153	9.017	5.134	7.574
11	0.0290	13.927	10.045	11.952	5.725	7.849
11	0.0300	31.396	14.257	23.151	12.843	7.953
11	0.0310	34.220	17.275	30.964	14.363	15.963
11	0.0320	17.495	11.249	13.234	8.359	13.519
11	0.0330	13.266	5.355	3.730	18.122	17.055
11	0.0340	11.860	7.696	6.023	14.386	16.483
11	0.0350	3.270	7.380	3.551	8.054	5.875
11	0.0360	3.280	7.410	3.552	7.965	5.847
11	0.0370	2.765	2.585	1.815	5.737	3.227
11	0.0380	6.230	9.704	12.850	13.385	13.607
11	0.0390	11.140	17.132	18.795	13.773	9.000
11	0.0400	12.743	12.193	19.825	8.262	16.159
11	0.0410	10.292	14.751	18.658	12.384	15.124
11	0.0420	9.696	9.611	9.396	12.866	9.911
11	0.0430	13.969	17.194	19.608	16.838	16.606
11	0.0440	11.002	20.537	22.008	7.956	11.229
11	0.0450	8.788	13.397	11.939	16.639	13.187

11	0.0460	4.889	8.441	8.915	8.121	8.583
11	0.0470	8.552	9.343	13.498	10.904	15.235
11	0.0480	9.261	7.133	7.198	7.165	13.693
11	0.0490	10.430	11.341	11.079	7.709	4.964
11	0.0500	4.167	10.915	10.909	19.575	16.273
11	0.0510	4.459	8.337	10.017	13.115	17.307
11	0.0520	10.674	11.854	11.705	9.794	11.529
11	0.0530	8.592	10.027	9.994	14.078	18.644
11	0.0540	11.496	7.358	10.471	12.818	17.869
11	0.0550	8.528	5.049	6.693	10.861	27.334
11	0.0560	9.483	11.120	8.944	13.548	11.309
11	0.0570	9.406	6.834	8.298	12.589	12.858
11	0.0580	8.228	4.665	7.456	12.791	21.693
11	0.0590	2.759	3.781	4.780	12.277	17.171
11	0.0600	5.808	6.437	7.338	9.593	16.732
11	0.0610	10.526	14.581	13.015	7.196	9.921
11	0.0620	4.257	6.409	5.942	7.656	11.075
11	0.0630	2.970	5.078	6.961	10.124	20.321
11	0.0640	4.542	5.140	5.033	10.339	8.963
11	0.0650	7.372	5.429	7.678	7.402	14.780
11	0.0660	10.492	11.832	12.067	11.053	11.234
11	0.0670	9.171	6.251	7.034	11.533	10.814
11	0.0680	5.163	8.227	4.569	9.683	13.596
11	0.0690	3.161	3.651	3.071	1.067	6.964
11	0.0700	3.614	13.398	8.614	11.203	14.411
11	0.0710	4.148	10.856	5.779	13.405	20.490
11	0.0720	5.441	11.631	7.611	11.503	16.499
11	0.0730	13.207	10.904	13.671	5.827	11.933
11	0.0740	4.258	4.909	5.260	9.679	9.102
11	0.0750	7.940	14.452	10.193	16.353	10.700
11	0.0760	4.264	10.062	4.947	8.038	9.321
11	0.0770	10.506	17.338	9.048	13.248	24.476
11	0.0780	8.588	7.375	7.303	5.956	12.019
11	0.0790	4.084	4.620	3.194	4.793	9.579
11	0.0800	8.707	13.152	12.016	15.137	16.079
11	0.0810	12.974	15.679	14.330	15.446	9.593
11	0.0820	9.811	11.450	12.632	5.930	17.535
11	0.0830	8.893	15.049	11.325	12.851	17.949
11	0.0840	12.824	21.115	12.013	14.540	4.471
11	0.0850	7.837	12.989	10.217	11.100	5.304
11	0.0860	8.137	12.525	9.235	11.765	4.177
11	0.0870	11.230	26.036	19.466	26.992	14.006
11	0.0880	10.547	17.205	15.976	28.526	14.518
11	0.0890	12.098	13.174	21.077	23.128	14.943
11	0.0900	11.116	11.098	13.046	11.728	19.105
11	0.0910	11.576	13.499	16.079	10.079	19.313
11	0.0920	13.406	12.406	21.964	13.941	19.995
11	0.0930	13.197	11.950	16.495	18.521	11.671
11	0.0940	10.745	23.030	18.226	20.528	13.198
11	0.0950	14.803	18.418	18.392	20.103	7.942

11	0.0960	10.074	13.863	14.241	19.466	11.239
11	0.0970	9.439	17.794	11.092	14.153	11.679
11	0.0980	11.572	16.805	12.172	15.814	13.636
11	0.0990	3.136	5.113	5.543	10.649	12.787
11	0.1000	5.178	4.755	6.586	3.339	13.488
11	0.1010	5.844	7.501	6.927	4.339	5.778
11	0.1020	9.069	8.828	10.577	5.852	13.678
11	0.1030	12.108	16.283	12.369	12.138	24.935
11	0.1040	10.969	21.656	12.283	19.940	21.272
11	0.1050	6.397	14.481	10.449	13.953	19.116
11	0.1060	8.999	9.808	12.401	7.611	12.146
11	0.1070	11.012	10.655	10.572	8.916	19.019
11	0.1080	8.328	11.741	7.065	4.657	3.140
11	0.1090	3.579	8.687	7.497	6.261	7.347
11	0.1100	9.247	13.906	11.391	12.722	9.688
11	0.1110	5.200	8.063	4.791	12.353	12.169
11	0.1120	14.186	12.733	14.423	9.622	15.157
11	0.1130	11.603	11.776	7.710	30.537	18.746
11	0.1140	8.029	8.793	8.722	25.853	19.600
11	0.1150	0.960	4.985	5.278	9.477	6.017
11	0.1160	0.955	4.041	4.512	8.640	9.551
11	0.1170	2.858	3.666	3.294	9.602	5.765
11	0.1180	7.857	8.395	8.302	6.889	9.251
11	0.1190	14.717	12.136	14.969	9.309	14.015
11	0.1200	15.652	16.607	11.300	8.338	21.038
11	0.1210	6.438	14.723	8.156	10.380	6.303
11	0.1220	7.602	13.087	8.583	11.096	8.740
11	0.1230	7.705	9.652	9.545	8.148	4.222
11	0.1240	8.789	11.394	9.482	9.823	8.137
11	0.1250	9.148	10.174	11.424	11.007	14.647
11	0.1260	8.627	16.197	13.606	15.815	8.457
11	0.1270	6.329	9.865	12.214	11.602	19.506
11	0.1280	13.789	13.155	13.893	5.603	16.668
11	0.1290	3.505	2.469	6.007	11.307	16.582
11	0.1300	8.171	5.351	6.099	13.553	9.807
11	0.1310	12.249	12.371	16.151	12.307	12.000
11	0.1320	9.385	11.931	15.014	7.465	27.082
11	0.1330	9.522	11.985	15.172	7.410	27.090
11	0.1340	10.074	11.081	11.938	19.463	12.974
11	0.1350	9.632	12.920	11.100	18.249	15.181
11	0.1360	10.129	8.205	14.303	11.163	17.452
11	0.1370	6.183	11.926	11.574	10.063	15.102
11	0.1380	4.978	12.540	10.783	13.352	15.909
11	0.1390	8.749	8.983	12.626	9.577	11.202
11	0.1400	8.918	9.094	12.864	9.444	11.178
11	0.1410	11.601	8.751	13.854	8.134	6.310
11	0.1420	18.288	12.785	17.025	7.247	14.868
11	0.1430	18.063	13.326	15.437	17.706	10.082
11	0.1440	7.961	6.036	5.598	4.887	3.795
11	0.1450	10.164	11.290	10.217	13.695	14.623

11	0.1460	9.628	11.407	12.764	9.582	8.514
11	0.1470	5.745	11.218	6.552	7.171	5.686
11	0.1480	6.444	7.932	6.821	4.728	5.808
11	0.1490	6.783	7.824	8.868	4.702	9.190
11	0.1500	7.634	7.456	7.473	6.578	9.375
11	0.1510	2.328	1.376	3.438	2.770	3.096
11	0.1520	10.450	13.764	20.632	9.583	10.669
11	0.1530	6.944	8.805	8.490	14.356	8.524
11	0.1540	19.367	21.551	28.042	7.842	8.582
11	0.1550	13.921	22.896	16.279	12.871	11.508
11	0.1560	19.264	13.502	19.218	19.321	8.109
11	0.1570	5.319	9.961	7.909	5.152	4.570
11	0.1580	5.430	8.758	10.470	4.369	8.956
11	0.1590	5.216	9.644	9.078	8.571	9.309
11	0.1600	7.703	9.553	11.179	15.706	11.496
11	0.1610	5.054	6.088	11.193	13.697	6.404
11	0.1620	6.042	11.577	10.136	15.611	6.707
11	0.1630	9.331	10.289	13.835	9.631	11.449
11	0.1640	8.795	8.966	8.663	10.892	16.374
11	0.1650	7.999	6.031	7.393	5.083	12.504
11	0.1660	4.057	4.826	10.184	14.467	7.079
11	0.1670	9.038	13.579	14.653	12.376	15.345
11	0.1680	11.398	20.248	20.724	17.576	20.001
11	0.1690	20.542	18.953	23.305	12.881	6.592
11	0.1700	27.859	21.678	20.311	10.710	16.815
11	0.1710	11.070	11.053	12.655	14.646	10.748
11	0.1720	13.792	12.119	16.118	16.989	9.192
11	0.1730	6.484	8.395	13.690	12.373	7.203
11	0.1740	6.633	11.503	12.999	13.645	8.158
11	0.1750	6.669	7.560	10.064	5.363	7.674
11	0.1760	28.779	18.316	23.180	10.924	5.906
11	0.1770	8.133	6.057	7.191	8.819	7.437
11	0.1780	4.344	5.631	8.010	6.952	9.849
11	0.1790	6.012	4.855	9.869	8.015	14.833
11	0.1800	6.094	5.390	10.096	11.246	13.128
11	0.1810	4.720	3.972	8.188	10.076	21.140
11	0.1820	2.452	2.284	5.191	3.514	5.245
11	0.1830	24.635	10.600	21.696	7.690	9.744
11	0.1840	10.118	14.404	14.462	12.225	17.134
11	0.1850	7.867	9.088	7.298	11.347	17.944
11	0.1860	6.084	7.046	7.113	14.060	7.155
11	0.1870	7.104	9.714	8.289	10.886	6.878
11	0.1880	12.032	10.597	10.636	10.277	13.408
11	0.1890	15.495	11.497	12.868	18.161	18.111
11	0.1900	6.556	6.744	9.907	18.849	16.119
11	0.1910	6.567	8.883	10.435	7.408	21.737
11	0.1920	7.289	6.172	5.785	7.920	19.645
11	0.1930	8.907	6.807	7.477	7.380	8.239
11	0.1940	8.565	9.648	5.305	15.011	13.367
11	0.1950	4.001	7.398	6.177	15.192	9.750

11	0.1960	15.434	10.995	11.889	8.088	26.441
11	0.1970	6.726	6.199	5.902	21.127	21.348
11	0.1980	9.125	14.244	12.593	26.738	21.647
11	0.1990	7.007	11.787	6.920	25.420	25.953
11	0.2000	7.325	17.829	9.233	26.491	18.376
11	0.2010	20.291	7.806	14.883	18.448	19.793
11	0.2020	8.573	9.932	7.675	14.201	19.910
11	0.2030	11.372	16.229	12.287	21.407	14.444
11	0.2040	7.293	11.974	8.334	22.750	25.497
11	0.2050	3.059	3.301	2.881	16.592	11.628
11	0.2060	7.309	7.689	4.220	20.557	21.932
11	0.2070	5.359	5.904	3.752	13.090	21.862
11	0.2080	11.667	7.579	8.151	11.510	12.837
11	0.2090	11.586	7.160	7.558	11.316	14.048
11	0.2100	7.621	7.912	3.866	13.812	19.013
11	0.2110	13.991	14.131	6.674	10.071	20.958
11	0.2120	9.334	9.983	4.860	17.020	30.715
11	0.2130	13.071	17.014	12.706	19.547	22.884
11	0.2140	11.484	14.759	14.407	17.647	17.974
11	0.2150	10.455	11.308	7.891	17.482	12.996
11	0.2160	15.084	9.692	8.480	21.222	16.056
11	0.2170	9.332	9.568	8.231	13.853	15.093
11	0.2180	7.668	7.151	9.060	7.741	12.785
11	0.2190	9.785	9.325	8.351	14.042	22.449
11	0.2200	14.727	8.640	11.327	7.397	21.819
11	0.2210	14.024	9.758	6.988	18.084	17.368
11	0.2220	7.208	7.979	6.355	17.570	18.744
11	0.2230	19.091	10.115	12.413	19.219	24.864
11	0.2240	18.848	9.442	14.194	16.124	26.166
11	0.2250	17.606	17.936	17.812	11.399	15.411
11	0.2260	17.278	13.110	18.267	15.404	23.857
11	0.2270	13.919	10.907	11.377	5.948	21.725
11	0.2280	15.020	10.355	12.789	15.833	21.521
11	0.2290	20.307	18.074	22.908	8.897	20.780
11	0.2300	8.296	4.874	9.142	12.162	15.337
11	0.2310	16.094	5.087	7.315	15.373	24.142
11	0.2320	9.895	13.109	7.615	15.441	15.623
11	0.2330	19.884	9.355	16.607	11.719	13.552
11	0.2340	20.050	11.707	16.328	13.135	9.854
11	0.2350	9.518	15.149	13.975	17.242	10.340
11	0.2360	14.361	9.493	15.210	8.568	9.474
11	0.2370	11.949	10.022	10.310	7.459	14.897
11	0.2380	14.195	11.930	9.448	15.124	15.656
11	0.2390	18.747	15.321	14.352	11.579	23.389
11	0.2400	22.870	17.844	19.233	9.978	25.951
11	0.2410	11.641	9.330	12.657	6.291	18.536
11	0.2420	20.027	14.715	10.756	11.320	16.118
11	0.2430	6.659	7.106	12.439	2.409	5.450
11	0.2440	8.069	12.329	12.654	14.067	14.018
11	0.2450	7.354	8.179	8.268	9.982	12.336

11	0.2460	8.796	9.587	10.999	9.328	9.154
11	0.2470	13.615	8.494	10.922	5.380	11.666
11	0.2480	15.866	10.377	12.667	6.226	16.742
11	0.2490	18.500	13.540	16.908	14.026	11.288
11	0.2500	12.134	9.906	13.044	12.167	18.079
11	0.2510	14.035	8.435	10.162	10.632	18.289
11	0.2520	20.024	19.865	14.608	11.067	28.217
11	0.2530	7.716	6.795	8.581	4.446	4.505
11	0.2540	11.341	7.490	12.395	5.488	4.447
11	0.2550	11.663	8.726	6.189	6.028	10.948
11	0.2560	16.565	15.745	16.262	9.714	20.334
11	0.2570	8.995	14.647	11.455	12.516	15.208
11	0.2580	4.991	9.788	7.980	12.764	12.479
11	0.2590	14.962	20.638	13.728	8.721	23.995
11	0.2600	10.698	14.333	14.881	9.304	16.598
11	0.2610	21.107	14.333	13.887	16.990	20.131
11	0.2620	15.745	12.941	11.189	10.393	19.686
11	0.2630	18.413	20.190	19.038	12.554	15.707
11	0.2640	9.626	13.504	12.340	4.731	2.893
11	0.2650	12.153	13.970	14.662	12.543	6.025
11	0.2660	9.956	5.905	8.549	17.309	7.498
11	0.2670	3.657	10.962	4.123	17.001	10.052
11	0.2680	5.321	9.161	4.956	11.423	10.177
11	0.2690	14.974	14.197	14.276	10.515	21.637
11	0.2700	14.397	19.608	19.470	6.001	23.203
11	0.2710	15.051	13.328	9.554	7.889	17.394
11	0.2720	4.815	9.090	11.231	9.901	16.063
11	0.2730	8.007	15.319	12.896	17.643	14.717
11	0.2740	7.299	10.852	9.255	15.260	18.356
11	0.2750	12.190	20.652	14.338	20.702	31.470
11	0.2760	10.322	14.239	9.690	17.593	17.140
11	0.2770	8.168	10.834	9.643	10.104	12.869
11	0.2780	9.575	10.209	9.087	18.744	18.780
11	0.2790	9.726	14.040	11.850	14.741	9.027
11	0.2800	13.199	20.227	15.169	12.312	11.150
11	0.2810	15.074	15.249	14.393	6.562	20.172
11	0.2820	14.166	10.736	16.515	19.266	12.324
11	0.2830	12.186	13.717	14.487	9.386	16.013
11	0.2840	9.331	7.998	13.536	8.120	10.876
11	0.2850	12.894	12.848	17.876	14.969	26.411
11	0.2860	5.948	6.334	7.035	5.694	14.975
11	0.2870	8.674	13.162	10.214	5.210	13.151
11	0.2880	4.601	6.804	5.004	5.797	5.763
11	0.2890	13.135	13.344	10.694	11.883	13.858
11	0.2900	20.814	23.425	20.798	20.791	23.053
11	0.2910	18.945	14.329	20.384	8.669	18.090
11	0.2920	17.010	19.033	17.643	10.037	19.895
11	0.2930	10.116	14.845	12.612	25.237	31.163
11	0.2940	12.968	12.544	16.097	19.385	28.710
11	0.2950	13.578	11.565	13.039	9.333	23.960

11	0.2960	3.313	3.983	3.916	5.065	16.676
11	0.2970	7.550	6.169	5.889	11.308	23.891
11	0.2980	15.240	14.788	16.507	15.115	29.581
11	0.2990	14.603	14.397	14.514	11.409	12.968
11	0.3000	6.803	9.596	10.382	10.936	20.994
11	0.3010	14.648	15.437	16.861	12.299	19.411
11	0.3020	14.922	13.190	12.408	11.190	14.709
11	0.3030	17.508	18.141	20.169	6.375	17.282
11	0.3040	16.001	15.434	17.654	8.141	14.088
11	0.3050	18.725	24.051	23.451	13.589	12.477
11	0.3060	19.176	15.169	17.692	8.827	7.262
11	0.3070	8.958	9.019	8.433	5.802	11.187
11	0.3080	10.756	7.475	10.075	15.352	10.207
11	0.3090	14.894	17.119	18.287	10.234	10.436
11	0.3100	19.477	26.058	21.739	13.299	14.685
11	0.3110	21.862	17.691	19.510	7.653	6.749
11	0.3120	17.519	15.805	16.986	19.654	7.833
11	0.3130	16.838	19.344	20.599	9.798	7.377
11	0.3140	15.121	21.472	20.907	8.354	7.162
11	0.3150	6.282	9.953	4.498	12.164	10.548
11	0.3160	4.599	8.856	7.367	8.232	6.244
11	0.3170	13.087	9.744	13.602	3.544	2.294
11	0.3180	18.683	12.782	13.767	7.458	10.897
11	0.3190	10.762	8.460	7.692	19.983	9.720
11	0.3200	10.129	8.596	10.645	9.779	15.488
11	0.3210	12.753	18.280	13.543	9.224	22.713
11	0.3220	15.834	16.565	14.958	8.787	19.613
11	0.3230	6.123	8.054	9.836	9.038	8.936
11	0.3240	12.925	11.651	10.714	10.017	11.872
11	0.3250	16.499	15.030	13.321	7.725	20.065
11	0.3260	18.288	9.503	12.297	16.076	13.435
11	0.3270	11.843	10.052	10.917	7.035	8.545
11	0.3280	4.620	3.844	5.461	6.652	10.375
11	0.3290	12.349	11.096	12.881	6.798	12.219
11	0.3300	12.439	16.722	14.410	15.380	14.837
11	0.3310	14.634	20.668	11.517	9.676	10.149
11	0.3320	7.449	11.605	11.207	11.071	12.681
11	0.3330	11.320	16.334	14.000	13.840	12.974
11	0.3340	14.911	14.869	12.826	11.277	14.458
11	0.3350	16.047	14.960	13.936	12.941	13.983
11	0.3360	9.089	7.383	11.484	13.494	12.240
11	0.3370	10.398	10.572	10.850	4.822	7.285
11	0.3380	11.638	14.371	13.569	11.662	17.467
11	0.3390	9.128	14.745	11.576	11.326	19.167
11	0.3400	21.660	26.176	24.044	8.456	13.368
11	0.3410	10.110	16.582	15.522	10.060	4.833
11	0.3420	9.484	11.701	9.382	3.810	2.110
11	0.3430	7.831	10.858	7.222	9.634	14.372
11	0.3440	13.238	12.937	12.523	9.594	14.571
11	0.3450	13.883	12.593	15.454	15.285	13.604

11	0.3460	29.523	25.717	27.147	12.579	17.135
11	0.3470	17.164	9.831	12.907	12.788	5.654
11	0.3480	35.076	20.256	26.066	7.652	12.673
11	0.3490	8.834	12.894	14.254	6.815	7.118
11	0.3500	15.762	11.668	21.657	13.537	7.051
11	0.3510	13.415	7.412	16.127	9.880	10.709
11	0.3520	26.223	21.109	30.838	10.786	4.880
11	0.3530	18.817	12.032	14.967	10.185	9.079
11	0.3540	29.901	20.294	18.348	11.071	18.520
11	0.3550	24.868	14.843	27.319	10.610	13.386
11	0.3560	9.282	14.132	17.556	10.153	11.707
11	0.3570	21.530	13.994	18.503	10.103	28.622
11	0.3580	10.796	15.536	17.161	14.530	15.609
11	0.3590	8.923	14.308	12.432	13.552	5.116
11	0.3600	21.044	25.630	25.765	22.924	10.777
11	0.3610	16.412	16.969	22.510	11.586	19.618
11	0.3620	20.789	9.994	21.122	18.637	18.647
11	0.3630	15.948	12.191	20.580	13.448	6.998
11	0.3640	28.163	24.243	31.075	12.825	19.778
11	0.3650	24.321	20.488	15.502	15.094	22.483
11	0.3660	27.876	19.319	22.505	16.982	18.231
11	0.3670	18.913	15.632	20.959	13.527	12.850
11	0.3680	9.990	8.221	13.433	7.727	9.651
11	0.3690	14.919	11.920	15.698	16.674	18.532
11	0.3700	26.946	15.047	22.760	19.027	27.748
11	0.3710	19.201	13.679	20.145	20.251	17.103
11	0.3720	12.523	15.320	20.069	10.878	13.138
11	0.3730	21.458	19.923	18.714	15.187	17.371
11	0.3740	11.824	7.520	7.555	15.564	13.694
11	0.3750	10.850	12.801	12.434	5.183	2.372
11	0.3760	13.636	10.990	14.299	9.440	4.502
11	0.3770	17.402	8.275	16.768	9.842	8.623
11	0.3780	7.400	6.974	13.776	5.510	4.576
11	0.3790	5.596	6.865	12.300	0.261	3.781
11	0.3800	8.865	9.307	17.408	1.259	11.180
11	0.3810	8.982	15.434	17.387	4.206	12.874
11	0.3820	6.262	9.460	7.062	4.335	8.555
11	0.3830	6.258	9.453	7.059	4.334	8.571
11	0.3840	11.043	14.532	18.969	5.841	10.783
11	0.3850	13.874	20.746	20.610	17.452	12.579
11	0.3860	24.357	26.550	30.280	11.532	15.055
11	0.3870	11.260	10.107	15.068	8.563	13.169
11	0.3880	12.960	10.762	16.949	11.850	4.325
11	0.3890	17.068	18.322	16.107	27.539	8.783
11	0.3900	12.339	17.598	14.262	15.331	6.424
11	0.3910	5.291	10.572	9.726	12.195	10.403
11	0.3920	17.328	17.616	20.125	16.315	14.916
11	0.3930	19.157	22.182	27.000	11.267	18.184
11	0.3940	9.005	7.374	7.994	12.703	5.272
11	0.3950	8.983	12.777	8.235	13.951	7.026

11	0.3960	7.410	11.132	9.781	13.263	15.846
11	0.3970	14.695	10.938	13.885	15.081	9.823
11	0.3980	10.976	8.383	13.531	14.106	9.329
11	0.3990	31.639	24.858	30.408	13.395	15.524
11	0.4000	14.902	14.956	17.815	8.385	14.456
11	0.4010	8.248	12.841	7.563	12.789	15.250
11	0.4020	6.850	8.788	5.773	17.767	7.900
11	0.4030	5.793	2.859	1.798	11.160	11.027
11	0.4040	9.730	9.676	6.735	24.613	16.266
11	0.4050	9.713	9.679	6.713	24.601	16.298
11	0.4060	28.412	21.995	25.499	10.267	8.463
11	0.4070	11.124	14.158	13.124	6.890	15.926
11	0.4080	13.987	18.722	15.953	10.399	20.380
11	0.4090	20.047	24.667	26.083	10.111	17.012
11	0.4100	15.144	15.475	16.121	9.646	11.930
11	0.4110	24.803	22.910	27.398	19.231	13.963
11	0.4120	13.231	10.141	15.660	15.475	9.106
11	0.4130	13.101	12.406	15.611	14.813	7.879
11	0.4140	18.890	15.317	15.301	10.643	9.573
11	0.4150	27.939	23.200	26.321	3.411	8.478
11	0.4160	22.199	16.869	23.293	10.061	12.019
11	0.4170	11.506	19.634	16.423	10.341	17.551
11	0.4180	16.969	18.834	19.361	11.264	14.991
11	0.4190	19.481	14.808	17.469	30.369	14.215
11	0.4200	25.444	16.018	23.694	16.058	15.609
11	0.4210	21.098	12.069	17.515	9.520	18.482
11	0.4220	18.822	28.139	21.246	21.391	17.301
11	0.4230	30.471	23.534	20.434	7.346	15.091
11	0.4240	21.590	21.843	21.919	9.009	20.887
11	0.4250	19.785	19.323	20.587	10.726	16.305
11	0.4260	21.650	21.003	28.827	12.640	15.823
11	0.4270	20.754	26.509	25.934	15.646	24.663
11	0.4280	20.800	17.533	17.654	19.383	25.534
11	0.4290	20.742	18.011	14.277	11.316	11.341
11	0.4300	27.814	18.594	19.796	12.484	17.265
11	0.4310	7.759	12.680	9.192	11.504	7.998
11	0.4320	10.745	13.908	14.577	12.533	4.062
11	0.4330	20.127	20.669	22.799	20.218	12.315
11	0.4340	25.861	22.346	21.716	26.772	17.174
11	0.4350	24.803	25.131	25.961	20.436	19.868
11	0.4360	20.368	21.726	21.013	19.442	16.713
11	0.4370	13.994	16.722	19.909	2.393	15.542
11	0.4380	23.216	21.804	28.199	6.192	15.705
11	0.4390	19.252	19.325	22.469	15.388	11.966
11	0.4400	14.476	17.861	15.715	14.255	16.372
11	0.4410	13.002	19.520	16.429	12.126	19.920
11	0.4420	20.787	20.283	19.838	16.932	23.743
11	0.4430	12.675	11.662	14.733	4.972	8.876
11	0.4440	16.888	16.343	15.654	15.387	11.049
11	0.4450	12.522	22.509	11.603	30.361	14.433

11	0.4460	16.789	29.998	20.415	24.057	18.913
11	0.4470	22.625	24.285	28.494	13.214	8.907
11	0.4480	15.413	16.726	18.269	7.180	10.112
11	0.4490	17.705	18.533	13.869	11.143	13.119
11	0.4500	15.409	17.294	18.543	12.491	18.044
11	0.4510	8.924	9.410	10.424	22.056	24.070
11	0.4520	17.417	14.523	11.401	11.035	16.482
11	0.4530	19.464	16.190	16.146	17.376	18.509
11	0.4540	20.378	28.906	23.988	14.140	15.049
11	0.4550	15.910	12.982	9.730	9.751	22.450
11	0.4560	15.311	5.517	12.479	12.594	9.168
11	0.4570	21.331	26.213	22.748	11.115	10.617
11	0.4580	17.248	15.395	18.259	11.129	4.244
11	0.4590	26.939	19.297	31.647	13.673	12.621
11	0.4600	18.079	21.965	21.105	6.723	9.669
11	0.4610	25.963	14.903	22.895	10.314	7.749
11	0.4620	6.882	6.773	9.861	7.256	7.130
11	0.4630	8.920	6.847	14.420	15.373	8.393
11	0.4640	18.437	8.496	17.144	19.093	20.046
11	0.4650	10.037	9.495	17.698	3.250	8.242
11	0.4660	11.967	14.204	18.345	10.365	10.841
11	0.4670	6.158	7.350	11.806	6.314	5.438
11	0.4680	6.819	5.442	9.233	4.172	11.469
11	0.4690	16.937	9.991	11.962	10.784	9.941
11	0.4700	13.850	19.446	19.702	13.401	11.886
11	0.4710	7.811	6.158	6.871	12.334	12.237
11	0.4720	10.394	14.117	14.315	27.284	24.496
11	0.4730	10.409	14.086	14.310	27.220	24.534
11	0.4740	10.425	14.060	14.304	27.179	24.568
11	0.4750	11.255	9.470	13.330	21.246	9.127
11	0.4760	9.312	8.515	12.741	9.655	10.541
11	0.4770	9.192	6.213	5.737	8.393	16.004
11	0.4780	4.855	4.748	3.269	4.198	3.893
11	0.4790	8.017	7.223	8.181	5.774	7.289
11	0.4800	11.401	7.547	12.867	19.617	7.448
11	0.4810	8.247	12.410	12.572	20.121	19.597
11	0.4820	3.316	12.765	8.858	14.563	14.331
11	0.4830	6.720	11.217	11.859	14.569	22.417
11	0.4840	8.165	13.225	16.119	6.037	15.329
11	0.4850	7.297	8.733	8.584	14.184	20.997
11	0.4860	2.071	3.333	1.103	10.716	7.918
11	0.4870	7.978	8.510	8.775	6.978	18.849
11	0.4880	4.926	3.570	6.865	7.147	10.580
11	0.4890	4.520	3.607	9.297	4.681	6.297
11	0.4900	7.212	4.102	5.989	4.639	7.032
11	0.4910	15.039	14.843	18.711	15.485	24.785
11	0.4920	6.495	5.074	7.386	12.074	18.547
11	0.4930	2.940	2.223	1.916	3.318	15.612
11	0.4940	2.667	2.371	3.390	8.495	7.663
11	0.4950	9.882	12.404	10.626	19.752	21.962

11	0.4960	10.699	13.266	14.080	11.015	21.319
11	0.4970	13.153	11.457	14.841	13.287	16.678
11	0.4980	9.992	14.526	4.873	12.316	20.855
11	0.4990	13.251	14.166	14.078	11.568	20.519
11	0.5000	15.940	14.695	15.682	5.237	16.281
11	0.5010	14.922	15.546	14.808	15.443	10.152
11	0.5020	10.287	13.881	12.570	11.027	13.532
11	0.5030	8.842	9.256	9.078	9.232	15.718
11	0.5040	12.090	7.615	18.799	10.701	7.467
11	0.5050	12.450	6.382	9.691	9.133	8.840
11	0.5060	18.131	12.006	12.295	12.260	12.831
11	0.5070	8.924	6.647	11.243	12.019	10.463
11	0.5080	6.288	8.793	12.054	11.030	8.344
11	0.5090	2.208	7.774	8.054	7.571	11.017
11	0.5100	6.321	6.640	10.673	8.862	13.621
11	0.5110	6.520	3.518	15.301	9.105	14.743
11	0.5120	9.275	11.785	15.629	8.887	17.680
11	0.5130	5.116	8.210	7.074	2.954	11.101
11	0.5140	3.292	13.751	3.573	8.822	9.871
11	0.5150	3.056	8.518	8.489	8.711	22.219
11	0.5160	7.900	12.240	12.088	8.823	15.277
11	0.5170	9.913	8.145	15.037	4.220	16.152
11	0.5180	4.740	4.531	9.328	2.936	11.104
11	0.5190	10.341	14.438	11.403	14.577	19.610
11	0.5200	3.357	4.828	2.557	5.486	6.384
11	0.5210	6.287	7.709	10.493	5.754	9.162
11	0.5220	7.272	7.151	7.354	10.406	15.250
11	0.5230	7.272	7.150	7.353	10.416	15.250
11	0.5240	3.898	5.690	6.689	9.092	24.128
11	0.5250	9.143	15.554	13.667	10.873	21.880
11	0.5260	7.092	6.917	14.248	17.415	17.680
11	0.5270	15.391	17.284	16.774	21.971	13.330
11	0.5280	18.620	17.308	20.828	9.739	20.479
11	0.5290	14.721	15.937	19.822	6.083	18.413
11	0.5300	12.759	15.624	12.554	13.613	19.161
11	0.5310	11.862	8.071	11.250	16.356	15.469
11	0.5320	10.527	6.462	10.628	6.343	14.945
11	0.5330	8.941	7.761	12.103	4.444	18.239
11	0.5340	16.280	13.021	16.013	12.596	24.646
11	0.5350	7.852	12.424	6.253	26.905	23.383
11	0.5360	8.350	7.936	10.847	11.268	17.093
11	0.5370	11.877	10.091	12.694	17.521	14.845
11	0.5380	8.252	11.138	9.231	15.755	19.144
11	0.5390	23.025	17.338	12.724	19.479	21.621
11	0.5400	11.876	20.143	14.991	16.235	13.691
11	0.5410	22.947	24.701	22.083	9.689	8.987
11	0.5420	6.331	5.539	15.535	9.729	7.269
11	0.5430	22.235	15.306	22.922	9.903	10.737
11	0.5440	17.650	17.524	14.667	22.979	13.967
11	0.5450	18.278	12.658	11.651	4.411	19.986

11	0.5460	11.850	18.482	15.899	17.333	20.036
11	0.5470	8.684	10.226	7.913	12.460	18.186
11	0.5480	10.005	12.712	13.474	9.739	16.959
11	0.5490	11.932	15.886	14.952	7.321	19.661
11	0.5500	10.601	12.097	10.963	11.838	19.122
11	0.5510	10.848	13.569	18.542	10.002	14.179
11	0.5520	12.133	11.874	13.958	12.692	12.967
11	0.5530	20.644	15.078	27.599	18.342	20.415
11	0.5540	31.699	21.458	34.760	8.322	16.116
11	0.5550	16.400	14.297	17.772	12.973	8.516
11	0.5560	18.289	17.824	20.058	8.686	14.984
11	0.5570	13.381	17.863	15.397	19.608	16.092
11	0.5580	25.277	11.343	24.543	19.257	13.658
11	0.5590	21.159	10.784	25.823	23.495	12.218
11	0.5600	13.380	7.173	13.100	23.835	6.301
11	0.5610	5.455	8.050	6.944	8.924	12.807
11	0.5620	17.863	13.059	13.198	7.952	22.173
11	0.5630	11.579	10.168	9.996	14.101	19.341
11	0.5640	10.242	6.771	12.552	12.260	10.672
11	0.5650	9.966	5.296	11.000	9.139	7.877
11	0.5660	10.086	8.533	11.204	9.715	15.154
11	0.5670	18.162	15.897	21.080	16.453	17.568
11	0.5680	10.802	11.382	12.384	12.574	19.732
11	0.5690	15.980	13.605	13.169	12.959	11.638
11	0.5700	9.737	12.313	13.067	6.948	7.428
11	0.5710	14.001	12.805	16.509	11.077	14.820
11	0.5720	32.540	27.723	28.842	17.969	24.822
11	0.5730	26.037	24.655	28.415	13.639	14.505
11	0.5740	15.621	15.778	15.836	12.676	15.927
11	0.5750	16.012	14.383	14.354	6.131	18.466
11	0.5760	7.654	9.543	11.867	7.358	16.051
11	0.5770	11.631	14.362	13.194	13.455	11.692
11	0.5780	21.723	21.018	19.835	18.182	14.596
11	0.5790	10.955	13.109	11.770	10.155	6.330
11	0.5800	4.521	3.963	5.358	13.815	14.043
11	0.5810	12.513	13.611	8.541	14.586	13.909
11	0.5820	14.376	14.387	11.108	14.537	15.221
11	0.5830	17.400	20.840	12.085	12.569	16.536
11	0.5840	14.948	14.493	10.020	10.415	15.384
11	0.5850	5.648	9.705	12.114	20.057	21.429
11	0.5860	6.628	5.885	10.881	19.801	20.623
11	0.5870	5.346	5.735	8.255	10.589	14.834
11	0.5880	11.863	13.538	21.750	9.101	7.740
11	0.5890	7.897	7.726	9.495	7.736	5.749
11	0.5900	10.774	8.247	12.198	9.656	10.517
11	0.5910	12.526	7.660	15.192	7.070	13.161
11	0.5920	13.834	10.254	14.477	13.486	10.011
11	0.5930	2.808	5.848	5.453	17.364	15.925
11	0.5940	11.992	5.346	8.036	26.540	16.016
11	0.5950	3.961	6.523	5.234	6.699	8.977

11	0.5960	12.463	12.559	15.018	11.478	4.672
11	0.5970	8.053	6.456	7.803	12.415	12.858
11	0.5980	7.627	7.122	9.641	23.171	18.428
11	0.5990	18.740	25.673	24.544	19.408	19.310
11	0.6000	11.819	14.155	17.061	10.773	13.297
11	0.6010	17.322	19.091	18.469	7.659	13.517
11	0.6020	22.909	22.726	25.300	7.786	14.593
11	0.6030	17.228	19.453	16.725	15.528	19.929
11	0.6040	15.006	16.309	15.425	9.839	15.623
11	0.6050	3.305	2.339	5.050	1.815	4.424
11	0.6060	3.285	2.196	5.007	2.281	4.829
11	0.6070	5.721	5.639	8.091	9.881	6.916
11	0.6080	3.970	8.538	4.242	15.969	6.379
11	0.6090	7.867	7.382	8.500	21.756	18.869
11	0.6100	7.629	6.331	9.245	11.944	12.060
11	0.6110	1.450	4.543	2.042	12.514	13.023
11	0.6120	6.047	5.317	10.843	16.909	11.971
11	0.6130	6.662	6.447	8.163	6.072	13.680
11	0.6140	9.106	8.749	9.526	13.176	8.566
11	0.6150	11.980	8.473	16.050	11.440	12.381
11	0.6160	8.868	10.627	8.293	8.523	12.268
11	0.6170	6.540	11.228	8.249	13.931	9.854
11	0.6180	6.538	11.228	8.246	13.882	9.845
11	0.6190	4.661	6.632	11.600	12.158	12.050
11	0.6200	9.289	10.134	13.653	12.762	12.792
11	0.6210	9.089	8.809	15.788	12.048	16.301
11	0.6220	11.953	8.434	13.524	17.851	18.407
11	0.6230	18.217	10.876	13.554	15.135	14.088
11	0.6240	12.779	11.260	7.727	9.673	14.863
11	0.6250	18.590	9.411	12.308	11.879	21.971
11	0.6260	18.648	15.982	13.895	13.012	17.479
11	0.6270	19.235	17.197	15.455	11.074	19.061
11	0.6280	14.409	10.755	13.517	22.391	17.770
11	0.6290	12.469	10.318	10.075	9.881	15.590
11	0.6300	10.952	12.696	12.651	11.408	19.190
11	0.6310	9.225	8.496	9.164	10.944	11.630
11	0.6320	4.160	5.979	5.731	7.340	12.118
11	0.6330	4.888	10.162	12.627	14.265	20.901
11	0.6340	4.569	3.511	7.418	16.432	21.113
11	0.6350	1.795	1.817	3.567	9.554	13.744
11	0.6360	5.109	4.242	4.199	15.774	16.066
11	0.6370	8.405	7.411	7.214	11.408	25.999
11	0.6380	9.721	8.550	8.151	9.286	16.726
11	0.6390	5.952	7.081	8.666	6.818	11.050
11	0.6400	4.026	4.288	4.481	3.794	11.167
11	0.6410	6.770	8.249	15.306	7.922	14.954
11	0.6420	7.944	5.289	9.122	10.389	15.922
11	0.6430	8.998	11.252	13.027	7.560	14.072
11	0.6440	8.081	4.886	10.279	10.662	8.059
11	0.6450	10.370	6.659	9.582	12.139	19.140

11	0.6460	9.484	8.924	8.870	15.484	21.437
11	0.6470	13.623	15.721	16.932	23.098	11.734
11	0.6480	12.203	13.480	9.564	22.410	20.072
11	0.6490	10.651	6.326	14.787	12.313	10.831
11	0.6500	17.121	10.624	19.339	20.545	8.319
11	0.6510	9.408	7.414	12.178	5.675	20.115
11	0.6520	10.212	5.472	12.929	16.491	18.296
11	0.6530	7.022	10.912	16.936	7.496	16.825
11	0.6540	6.942	10.721	16.747	7.411	16.766
11	0.6550	8.730	15.923	18.264	7.799	20.138
11	0.6560	13.337	15.782	15.056	11.494	23.259
11	0.6570	16.576	11.538	9.530	18.942	21.375
11	0.6580	10.095	8.549	7.498	12.019	10.604
11	0.6590	16.476	15.053	17.502	18.818	25.229
11	0.6600	15.091	10.915	16.116	12.955	21.314
11	0.6610	8.665	8.203	12.296	8.657	21.351
11	0.6620	11.895	15.809	10.852	12.118	15.302
11	0.6630	12.915	10.844	12.925	10.750	8.806
11	0.6640	9.090	8.510	9.101	8.629	9.170
11	0.6650	5.988	2.691	3.846	5.776	10.344
11	0.6660	11.522	8.289	9.961	12.370	10.831
11	0.6670	7.503	4.285	8.117	5.373	6.466
11	0.6680	23.752	19.222	20.495	13.150	9.724
11	0.6690	17.403	15.922	17.817	7.603	11.819
11	0.6700	15.119	9.588	15.393	9.217	11.380
11	0.6710	13.289	9.474	21.558	11.786	14.210
11	0.6720	8.539	5.937	11.446	11.720	10.115
11	0.6730	17.332	13.869	22.613	17.917	19.197
11	0.6740	14.489	13.259	14.756	8.625	11.094
11	0.6750	18.827	9.799	22.405	10.181	12.219
11	0.6760	8.495	4.296	11.534	7.825	9.974
11	0.6770	10.999	12.071	13.259	11.383	10.116
11	0.6780	10.961	16.157	15.558	13.517	12.926
11	0.6790	9.480	6.627	9.235	10.919	10.574
11	0.6800	18.646	15.279	17.802	13.995	10.424
11	0.6810	7.743	10.310	7.774	9.873	19.541
11	0.6820	4.861	7.840	5.192	10.634	12.855
11	0.6830	11.510	11.904	13.217	9.761	15.524
11	0.6840	7.693	9.460	7.694	9.862	12.746
11	0.6850	14.337	13.172	13.529	13.871	17.357
11	0.6860	13.320	10.763	16.494	16.705	19.977
11	0.6870	12.386	7.783	19.105	4.805	10.509
11	0.6880	6.555	5.967	7.628	5.794	14.769
11	0.6890	2.737	2.355	1.500	7.926	7.193
11	0.6900	19.087	20.020	17.011	17.464	13.709
11	0.6910	13.546	6.141	9.499	17.613	14.357
11	0.6920	13.976	9.862	13.517	14.962	14.359
11	0.6930	14.569	16.056	17.776	5.336	12.655
11	0.6940	16.909	17.930	18.524	6.863	15.432
11	0.6950	1.999	1.709	1.353	5.227	6.423

11	0.6960	5.462	4.792	4.896	5.317	10.206
11	0.6970	5.885	5.411	5.789	12.399	9.737
11	0.6980	10.176	7.520	10.867	16.951	10.629
11	0.6990	12.975	14.477	11.880	14.495	10.822
11	0.7000	15.468	11.483	17.377	11.370	18.938
11	0.7010	18.512	19.146	22.922	4.781	7.614
11	0.7020	14.864	14.886	16.623	13.668	18.042
11	0.7030	6.780	9.892	7.410	12.881	12.580
11	0.7040	5.064	4.678	5.560	8.505	6.403
11	0.7050	4.765	5.749	7.428	4.049	4.715
11	0.7060	7.359	8.776	10.393	4.850	6.207
11	0.7070	12.450	13.743	18.256	12.725	13.707
11	0.7080	9.172	8.865	14.594	8.810	11.127
11	0.7090	0.690	1.382	0.852	2.844	1.319
11	0.7100	8.352	7.568	9.636	5.252	2.419
11	0.7110	9.624	6.532	8.932	3.509	3.252
11	0.7120	12.990	9.723	7.357	8.134	6.931
11	0.7130	22.524	19.674	26.284	14.654	9.457
11	0.7140	9.735	6.972	11.771	9.221	8.711
11	0.7150	19.549	16.326	13.001	10.472	8.246
11	0.7160	9.056	11.696	15.689	6.439	2.534
11	0.7170	8.821	7.978	10.573	6.909	5.728
11	0.7180	6.797	4.067	8.958	4.730	11.810
11	0.7190	16.468	14.099	20.994	16.009	11.907
11	0.7200	13.674	9.523	18.892	8.802	8.576
11	0.7210	18.154	11.917	14.871	16.394	19.038
11	0.7220	8.660	3.259	6.459	7.031	5.456
11	0.7230	3.889	4.528	4.676	3.807	9.330
11	0.7240	15.660	12.675	13.778	11.840	8.373
11	0.7250	17.678	21.245	21.648	16.590	9.150
11	0.7260	5.101	4.109	5.283	5.399	7.004
11	0.7270	18.101	17.221	21.072	8.179	10.725
11	0.7280	19.258	17.291	11.513	24.875	14.779
11	0.7290	25.560	20.422	23.984	17.634	11.557
11	0.7300	13.808	8.729	11.971	20.797	9.757
11	0.7310	13.795	8.746	11.968	20.862	9.762
11	0.7320	5.018	4.236	7.997	11.920	9.513
11	0.7330	8.308	2.880	12.953	7.854	7.833
11	0.7340	5.687	7.504	11.558	7.430	9.054
11	0.7350	11.462	12.683	13.550	20.127	11.630
11	0.7360	7.698	8.974	8.213	18.574	11.101
11	0.7370	7.096	8.619	5.986	16.617	8.693
11	0.7380	6.125	13.831	11.518	12.353	2.927
11	0.7390	5.107	6.753	4.635	11.479	5.450
11	0.7400	12.488	6.350	11.084	12.826	6.473
11	0.7410	10.651	7.342	7.938	6.409	10.411
11	0.7420	13.293	9.962	13.239	10.340	6.184
11	0.7430	12.468	12.835	13.724	27.424	6.900
11	0.7440	13.787	8.634	12.023	17.642	9.518
11	0.7450	20.244	12.364	14.384	15.340	14.851

11	0.7460	8.474	7.369	10.716	19.044	12.844
11	0.7470	13.032	11.450	11.643	12.793	10.654
11	0.7480	16.267	6.286	11.008	10.901	7.838
11	0.7490	14.857	10.852	10.954	21.007	9.263
11	0.7500	7.316	8.266	10.083	16.868	6.386
11	0.7510	13.518	5.712	17.011	16.751	13.147
11	0.7520	19.488	15.492	23.645	7.032	5.861
11	0.7530	23.582	15.434	19.988	13.178	6.273
11	0.7540	7.990	6.843	7.358	18.907	5.824
11	0.7550	9.773	5.694	6.077	8.225	7.790
11	0.7560	13.183	10.668	13.052	14.783	10.804
11	0.7570	8.935	10.469	14.694	12.638	11.335
11	0.7580	12.236	8.842	14.959	11.866	15.102
11	0.7590	12.419	9.185	14.033	10.552	11.516
11	0.7600	15.049	11.246	16.053	7.248	14.191
11	0.7610	16.878	12.955	15.235	11.249	8.929
11	0.7620	17.984	16.841	21.517	19.614	9.445
11	0.7630	17.796	13.279	17.635	5.099	4.527
11	0.7640	9.636	15.188	14.277	15.086	8.775
11	0.7650	21.660	25.371	30.414	8.100	5.713
11	0.7660	14.502	19.996	18.583	17.915	12.517
11	0.7670	14.067	19.959	18.290	17.981	12.149
11	0.7680	17.138	19.955	18.175	19.728	10.181
11	0.7690	25.869	24.751	28.860	17.111	6.852
11	0.7700	15.492	15.031	23.295	14.574	9.034
11	0.7710	24.538	9.706	21.904	23.410	4.841
11	0.7720	12.283	10.597	12.555	9.701	17.209
11	0.7730	9.498	10.175	10.556	18.680	11.903
11	0.7740	4.063	4.604	4.379	10.515	8.330
11	0.7750	13.796	9.740	18.381	14.546	5.643
11	0.7760	19.130	17.267	28.373	21.676	12.765
11	0.7770	9.200	5.277	12.078	17.640	12.137
11	0.7780	11.006	4.446	11.387	15.200	6.457
11	0.7790	12.976	10.070	13.000	15.433	11.104
11	0.7800	26.820	13.206	19.818	11.603	17.910
11	0.7810	14.427	13.483	15.218	7.374	19.999
11	0.7820	15.506	11.904	17.918	15.837	6.648
11	0.7830	13.394	13.468	13.783	23.667	11.571
11	0.7840	11.485	14.617	14.705	15.260	9.270
11	0.7850	7.460	5.199	5.236	13.716	9.189
11	0.7860	14.451	14.010	18.625	13.963	8.384
11	0.7870	14.298	15.754	18.496	20.847	22.278
11	0.7880	11.012	14.395	16.108	9.552	9.846
11	0.7890	20.309	27.000	25.696	11.214	11.896
11	0.7900	17.449	27.935	27.010	6.608	8.164
11	0.7910	19.959	32.543	27.829	21.451	16.963
11	0.7920	14.863	23.389	19.836	24.947	24.379
11	0.7930	13.225	7.948	12.847	13.221	19.853
11	0.7940	17.389	9.705	17.479	21.778	26.715
11	0.7950	7.654	10.657	9.913	17.828	17.969

11	0.7960	9.860	14.336	10.261	18.779	14.840
11	0.7970	15.356	22.200	23.880	12.649	11.221
11	0.7980	15.027	18.170	15.597	14.671	9.858
11	0.7990	17.208	16.110	15.756	15.982	22.370
11	0.8000	14.861	16.192	16.437	14.064	16.650
11	0.8010	14.833	16.157	16.506	14.058	16.567
11	0.8020	17.572	19.357	18.903	15.134	17.007
11	0.8030	12.253	15.474	16.606	14.932	10.387
11	0.8040	13.832	15.136	14.433	13.640	11.885
11	0.8050	13.811	10.675	14.500	19.409	12.337
11	0.8060	7.604	7.064	6.974	8.029	4.003
11	0.8070	9.966	7.811	7.741	6.883	1.331
11	0.8080	25.329	26.644	27.988	17.701	13.959
11	0.8090	21.078	19.847	22.635	16.677	14.374
11	0.8100	26.426	33.458	31.354	10.876	11.185
11	0.8110	11.872	14.666	15.826	13.291	10.804
11	0.8120	11.872	14.639	15.808	13.244	10.777
11	0.8130	17.044	20.481	14.686	18.107	11.393
11	0.8140	14.729	9.619	17.437	14.939	13.718
11	0.8150	13.017	8.168	11.012	8.990	12.321
11	0.8160	12.116	13.235	14.710	15.700	10.792
11	0.8170	12.166	12.352	14.914	6.646	6.840
11	0.8180	10.271	6.553	9.509	9.150	8.443
11	0.8190	13.159	9.041	15.972	4.610	5.138
11	0.8200	9.285	10.946	8.885	9.367	12.958
11	0.8210	11.267	9.242	8.957	13.363	14.831
11	0.8220	14.447	14.940	13.114	16.864	15.860
11	0.8230	16.269	25.944	18.803	17.255	17.240
11	0.8240	20.688	17.746	16.275	22.161	16.655
11	0.8250	21.626	15.955	22.848	12.339	5.676
11	0.8260	21.724	16.017	22.871	12.414	5.596
11	0.8270	18.023	17.404	22.762	13.721	12.659
11	0.8280	12.981	14.477	16.130	15.030	8.725
11	0.8290	18.927	14.145	19.716	9.484	9.107
11	0.8300	17.207	12.492	15.833	11.078	2.079
11	0.8310	22.681	16.420	18.836	20.505	10.623
11	0.8320	12.247	12.201	10.489	15.652	7.406
11	0.8330	13.890	11.364	9.369	8.376	9.672
11	0.8340	14.922	6.189	14.379	9.196	6.591
11	0.8350	12.504	7.238	10.186	4.191	7.027
11	0.8360	7.664	5.170	8.152	10.653	4.897
11	0.8370	8.280	10.507	10.051	11.963	8.609
11	0.8380	16.115	15.382	13.949	28.801	14.390
11	0.8390	10.791	9.617	7.359	19.561	23.881
11	0.8400	11.529	11.864	13.277	15.124	12.586
11	0.8410	6.055	7.498	6.125	10.730	19.498
11	0.8420	10.420	6.830	8.137	13.970	17.216
11	0.8430	14.044	12.104	15.303	24.546	13.876
11	0.8440	17.562	9.551	15.321	17.119	5.437
11	0.8450	19.326	20.378	17.787	23.911	12.839

11	0.8460	11.506	8.555	12.714	5.985	6.329
11	0.8470	28.323	14.639	23.894	5.859	4.361
11	0.8480	25.123	12.518	23.002	5.291	7.573
11	0.8490	18.218	12.204	18.128	6.109	6.123
11	0.8500	16.381	8.246	14.340	19.711	9.431
11	0.8510	22.598	14.871	20.512	12.826	8.189
11	0.8520	13.130	10.922	16.146	15.069	20.879
11	0.8530	5.561	4.951	4.366	6.078	4.791
11	0.8540	20.623	14.474	15.368	11.772	8.510
11	0.8550	14.187	16.450	15.923	14.403	14.489
11	0.8560	19.285	11.477	14.424	10.605	18.125
11	0.8570	12.668	11.186	16.526	4.094	17.415
11	0.8580	21.543	13.719	20.674	10.598	20.267
11	0.8590	14.899	17.715	19.722	16.938	20.754
11	0.8600	8.662	6.139	7.195	11.366	18.594
11	0.8610	15.987	5.351	10.474	11.044	19.613
11	0.8620	16.736	7.042	9.535	14.192	13.618
11	0.8630	7.076	5.193	3.754	16.260	10.643
11	0.8640	8.600	6.354	9.687	10.723	11.720
11	0.8650	9.646	9.795	14.756	8.700	14.188
11	0.8660	5.549	3.418	4.218	9.676	5.521
11	0.8670	3.562	2.563	5.193	10.885	2.693
11	0.8680	4.739	6.381	8.007	6.244	6.358
11	0.8690	8.918	10.518	6.307	8.058	5.894
11	0.8700	7.827	7.539	4.484	17.617	9.216
11	0.8710	7.790	7.538	4.472	17.628	9.098
11	0.8720	13.567	8.880	14.882	18.421	16.790
11	0.8730	5.763	6.592	7.807	8.721	9.036
11	0.8740	5.389	6.258	5.017	14.368	5.954
11	0.8750	5.825	12.885	7.688	25.182	10.315
11	0.8760	5.790	12.766	7.545	25.285	10.514
11	0.8770	13.463	5.122	6.889	5.487	10.759
11	0.8780	4.912	3.807	3.438	15.578	16.106
11	0.8790	21.863	17.865	25.067	17.584	8.845
11	0.8800	21.693	17.799	25.053	17.454	8.889
11	0.8810	16.591	12.878	11.458	31.596	21.395
11	0.8820	5.490	9.828	7.971	22.188	11.177
11	0.8830	8.049	8.118	10.024	20.556	11.095
11	0.8840	16.542	7.871	11.798	14.916	7.644
11	0.8850	11.873	7.804	10.021	12.769	7.949
11	0.8860	9.786	7.072	10.537	18.431	9.188
11	0.8870	21.311	11.768	16.996	12.259	17.686
11	0.8880	12.197	13.450	10.826	8.706	14.240
11	0.8890	17.510	13.647	13.217	9.450	8.738
11	0.8900	15.283	15.573	14.183	16.375	9.776
11	0.8910	13.585	13.410	13.829	16.113	12.928
11	0.8920	10.883	8.200	6.074	13.714	14.514
11	0.8930	6.099	10.738	5.856	24.940	11.759
11	0.8940	6.311	13.008	3.961	22.636	18.678
11	0.8950	10.422	16.278	10.508	19.433	10.147

11	0.8960	12.636	13.551	12.441	15.778	12.610
11	0.8970	16.312	16.269	18.102	19.581	16.885
11	0.8980	17.326	21.283	18.809	17.955	19.930
11	0.8990	10.672	10.699	10.356	11.683	14.605
11	0.9000	7.470	13.521	10.558	13.735	15.776
11	0.9010	11.292	14.971	13.886	14.391	10.031
11	0.9020	10.549	17.451	12.482	21.743	11.031
11	0.9030	7.354	13.045	8.685	24.217	10.790
11	0.9040	9.329	12.345	12.257	22.478	13.257
11	0.9050	18.426	20.371	22.266	25.950	8.629
11	0.9060	14.415	12.636	9.997	18.920	12.700
11	0.9070	8.652	11.196	11.693	14.322	15.937
11	0.9080	20.633	16.586	19.510	17.871	23.699
11	0.9090	21.349	15.842	21.470	17.787	15.212
11	0.9100	6.679	7.648	9.351	15.908	6.941
11	0.9110	14.499	12.308	13.210	20.563	5.384
11	0.9120	20.521	17.291	15.778	15.862	26.018
11	0.9130	15.914	15.334	13.388	28.230	22.109
11	0.9140	14.545	15.125	10.764	21.559	22.529
11	0.9150	18.625	17.503	18.854	24.995	23.959
11	0.9160	17.214	10.641	10.663	20.823	10.413
11	0.9170	13.606	13.349	14.772	21.019	7.949
11	0.9180	10.780	8.901	11.080	18.192	4.428
11	0.9190	13.445	14.017	14.192	18.159	23.044
11	0.9200	16.193	17.872	17.106	20.975	15.467
11	0.9210	15.492	19.031	18.375	22.525	16.100
11	0.9220	10.526	13.821	13.691	23.829	18.467
11	0.9230	7.322	14.639	7.113	21.659	15.767
11	0.9240	21.299	21.820	21.680	19.670	15.191
11	0.9250	7.572	9.258	4.624	8.639	9.138
11	0.9260	5.356	11.497	9.195	14.578	5.368
11	0.9270	2.104	5.578	1.125	4.753	7.403
11	0.9280	3.489	5.226	2.665	7.161	7.680
11	0.9290	9.658	16.242	9.424	15.082	10.577
11	0.9300	9.822	10.538	5.515	18.758	17.534
11	0.9310	11.939	11.037	13.947	23.664	15.978
11	0.9320	3.441	7.335	5.125	10.954	3.607
11	0.9330	6.822	7.885	6.644	8.446	8.359
11	0.9340	4.234	4.871	5.747	10.243	9.120
11	0.9350	8.999	9.290	13.165	16.814	13.988
11	0.9360	8.443	6.848	4.915	9.017	6.122
11	0.9370	14.117	11.627	8.741	24.630	7.363
11	0.9380	12.988	11.057	5.550	23.162	18.729
11	0.9390	10.701	5.693	5.786	18.058	22.050
11	0.9400	14.372	7.443	7.581	6.835	10.112
11	0.9410	6.700	12.548	7.888	5.954	0.879
11	0.9420	7.996	8.684	6.712	12.645	15.009
11	0.9430	14.027	15.473	10.955	14.966	11.604
11	0.9440	13.124	18.724	15.739	12.321	13.327
11	0.9450	7.299	4.466	2.959	11.981	10.733

11	0.9460	8.975	11.042	8.867	14.615	11.070
11	0.9470	12.687	13.834	11.248	18.912	18.942
11	0.9480	12.507	15.616	10.471	31.491	9.361
11	0.9490	14.338	9.861	12.778	16.509	10.481
11	0.9500	13.542	17.349	11.004	15.250	16.133
11	0.9510	8.097	14.968	8.820	13.641	6.003
11	0.9520	7.398	13.453	4.957	18.086	8.135
11	0.9530	16.427	20.180	14.002	22.448	6.340
11	0.9540	15.582	15.309	13.623	14.799	18.445
11	0.9550	14.351	9.374	18.033	19.813	17.802
11	0.9560	26.163	20.977	27.725	17.764	28.237
11	0.9570	32.168	18.715	34.589	20.285	8.495
11	0.9580	32.123	18.714	34.510	20.178	8.405
11	0.9590	23.209	15.672	21.547	26.396	29.084
11	0.9600	27.791	23.355	17.320	16.165	23.903
11	0.9610	14.949	15.675	13.037	14.150	16.445
11	0.9620	13.805	15.336	16.445	14.171	18.646
11	0.9630	12.792	13.733	7.638	9.811	15.089
11	0.9640	23.752	16.805	24.480	23.745	8.099
11	0.9650	3.247	12.832	6.189	20.531	9.185
11	0.9660	11.040	19.322	13.007	14.981	9.208
11	0.9670	13.921	25.362	16.664	15.206	10.720
11	0.9680	10.830	14.425	9.662	16.653	19.968
11	0.9690	10.839	14.418	9.602	16.673	19.703
11	0.9700	6.593	7.946	6.242	7.203	13.878
11	0.9710	9.769	13.716	11.700	22.198	25.580
11	0.9720	17.167	19.797	16.768	21.416	14.402
11	0.9730	16.873	20.017	20.483	30.537	15.686
11	0.9740	4.593	9.321	4.502	17.512	22.949
11	0.9750	4.436	8.096	2.738	20.009	22.968
11	0.9760	4.534	5.206	2.525	12.007	10.194
11	0.9770	3.996	9.273	5.414	16.384	21.183
11	0.9780	9.601	13.459	14.842	14.787	18.499
11	0.9790	12.401	21.135	19.048	21.866	14.547
11	0.9800	16.723	19.003	19.016	33.890	19.325
11	0.9810	15.541	22.439	16.658	23.510	21.251
11	0.9820	20.428	23.942	20.440	13.407	27.448
11	0.9830	27.699	29.025	23.171	17.253	23.434
11	0.9840	26.722	31.650	27.387	21.102	13.608
11	0.9850	13.986	12.633	11.352	19.939	22.422
11	0.9860	13.059	9.794	9.291	15.408	7.782
11	0.9870	26.429	21.195	26.982	11.351	16.914
11	0.9880	18.815	21.605	18.140	14.104	16.163
11	0.9890	20.872	30.609	27.049	16.095	10.334
11	0.9900	20.586	26.772	23.951	16.031	7.975
11	0.9910	13.505	19.902	13.441	21.086	17.488
11	0.9920	11.502	15.074	13.626	13.838	14.730
11	0.9930	9.348	14.390	16.913	21.804	27.701
11	0.9940	14.167	14.290	14.265	21.153	17.765
11	0.9950	16.206	22.522	13.932	16.370	25.752

11	0.9960	15.545	21.813	19.979	17.064	17.888
11	0.9970	21.305	21.560	20.820	18.927	24.804
11	0.9980	28.765	31.998	25.229	22.000	24.772
11	0.9990	10.210	14.986	15.861	16.116	36.621
11	1.0000	15.301	17.169	15.694	13.692	15.332
11	1.0010	13.404	22.596	12.123	18.589	13.478
11	1.0020	13.379	15.954	9.957	11.543	20.270
11	1.0030	21.353	16.268	14.980	15.009	25.023
11	1.0040	10.392	14.028	7.733	16.411	16.613
11	1.0050	9.415	16.732	9.369	15.124	12.863
11	1.0060	9.523	19.564	9.689	26.250	16.565
11	1.0070	17.915	17.754	15.125	16.071	15.792
11	1.0080	8.001	12.724	12.147	9.543	9.691
11	1.0090	19.718	16.719	18.221	21.811	8.694
11	1.0100	11.149	12.281	10.053	12.863	8.435
11	1.0110	6.500	13.723	8.032	16.792	11.838
11	1.0120	5.323	9.283	7.232	10.408	13.643
11	1.0130	7.404	12.014	10.200	22.879	12.366
11	1.0140	10.527	14.343	13.326	18.586	8.969
11	1.0150	9.102	12.930	13.500	17.931	14.502
11	1.0160	10.044	13.053	17.249	12.418	16.649
11	1.0170	11.993	9.249	8.434	16.738	22.773
11	1.0180	23.382	18.132	19.626	19.811	19.204
11	1.0190	14.131	11.601	12.164	20.098	22.629
11	1.0200	14.410	13.778	16.922	13.331	16.456
11	1.0210	15.947	19.885	21.427	12.671	12.979
11	1.0220	12.987	13.313	12.342	17.076	18.109
11	1.0230	12.933	13.223	12.315	16.996	18.171
11	1.0240	12.880	13.134	12.293	16.913	18.229
11	1.0250	3.907	12.472	5.491	22.576	20.455
11	1.0260	13.561	18.878	9.059	21.648	14.834
11	1.0270	16.438	25.365	14.976	20.943	13.772
11	1.0280	10.154	12.382	7.094	15.801	19.490
11	1.0290	13.392	22.433	13.598	15.677	16.726
11	1.0300	21.633	24.185	19.220	15.695	21.940
11	1.0310	18.465	14.344	17.351	16.901	11.349
11	1.0320	12.490	13.171	7.128	12.859	5.618
11	1.0330	12.388	14.656	9.453	17.862	7.488
11	1.0340	10.586	15.168	11.688	13.911	13.333
11	1.0350	12.333	10.139	17.194	10.343	18.444
11	1.0360	11.585	12.675	13.028	18.510	18.909
11	1.0370	16.769	11.779	14.585	9.984	13.824
11	1.0380	16.796	11.524	14.608	10.007	13.955
11	1.0390	8.914	8.826	6.794	16.740	18.368
11	1.0400	21.268	19.822	17.481	16.969	14.005
11	1.0410	18.112	23.152	21.425	19.462	16.559
11	1.0420	18.480	24.938	18.649	12.128	11.340
11	1.0430	19.539	26.797	16.473	8.023	14.203
11	1.0440	8.535	10.319	6.404	24.992	14.396
11	1.0450	8.124	4.872	5.138	16.320	13.039

11	1.0460	8.761	10.657	12.497	21.997	10.425
11	1.0470	8.683	8.705	7.328	9.662	8.129
11	1.0480	8.047	9.188	7.391	10.694	8.383
11	1.0490	3.923	10.395	6.262	13.853	13.204
11	1.0500	12.723	22.472	18.278	19.473	16.576
11	1.0510	17.175	16.486	11.100	12.397	15.291
11	1.0520	23.898	21.781	20.776	12.658	25.186
11	1.0530	0.000	0.000	0.000	0.000	0.000
12	0.0001	0.000	0.000	0.000	0.000	0.000
12	0.0011	11.601	11.778	10.832	23.181	25.505
12	0.0021	9.025	7.242	6.798	20.293	16.772
12	0.0031	9.824	13.628	13.667	26.090	13.966
12	0.0041	8.790	5.811	11.721	20.734	23.538
12	0.0051	10.563	7.376	7.450	9.383	10.884
12	0.0061	20.918	14.813	19.079	14.997	16.117
12	0.0071	21.433	8.633	16.053	14.674	7.882
12	0.0081	14.481	8.331	12.874	17.483	11.200
12	0.0091	19.307	17.911	14.710	20.338	14.118
12	0.0101	21.579	16.103	15.279	18.083	9.718
12	0.0111	11.501	12.297	10.925	13.843	3.110
12	0.0121	3.600	2.326	3.281	4.375	2.039
12	0.0131	10.538	6.340	10.013	18.082	10.747
12	0.0141	21.947	10.502	16.418	20.397	16.336
12	0.0151	19.714	19.950	9.223	20.884	18.844
12	0.0161	19.840	24.584	15.836	26.871	18.211
12	0.0171	13.244	15.651	9.366	27.183	12.827
12	0.0181	15.782	20.869	13.426	35.785	17.662
12	0.0191	13.333	14.525	12.410	23.502	20.860
12	0.0201	17.909	17.335	14.460	25.231	15.123
12	0.0211	19.555	17.691	10.702	20.529	16.488
12	0.0221	15.808	17.882	11.340	19.019	15.278
12	0.0231	9.856	20.448	9.710	25.813	15.467
12	0.0241	14.572	17.282	13.164	12.564	18.373
12	0.0251	15.416	14.957	13.882	17.395	8.250
12	0.0261	4.852	8.547	9.411	15.645	5.510
12	0.0271	9.267	15.236	13.201	9.336	4.344
12	0.0281	13.601	15.867	12.961	15.977	14.954
12	0.0291	9.416	21.651	12.421	26.667	15.223
12	0.0301	7.910	10.042	13.492	16.962	16.064
12	0.0311	6.216	5.115	8.144	13.078	11.590
12	0.0321	18.574	15.914	17.978	15.728	24.506
12	0.0331	10.064	14.127	14.337	22.458	8.641
12	0.0341	11.953	7.467	10.773	14.241	15.336
12	0.0351	12.376	11.197	10.434	20.054	10.228
12	0.0361	12.873	10.822	12.141	11.411	18.183
12	0.0371	27.417	16.194	14.327	27.395	18.203
12	0.0381	19.965	12.583	15.201	21.410	18.223
12	0.0391	9.545	7.237	6.986	13.858	15.147
12	0.0401	10.965	10.753	6.513	22.370	9.542
12	0.0411	8.563	16.784	10.710	28.509	9.084

12	0.0421	31.210	24.987	25.665	13.775	17.023
12	0.0431	10.342	13.398	9.966	23.045	15.771
12	0.0441	14.639	29.331	16.779	23.049	15.596
12	0.0451	16.360	18.333	14.727	21.614	16.432
12	0.0461	21.459	18.320	15.723	13.464	11.537
12	0.0471	19.843	10.416	15.043	12.533	17.406
12	0.0481	12.669	10.290	10.426	21.086	17.118
12	0.0491	18.003	22.250	13.419	19.829	15.038
12	0.0501	13.187	17.349	11.503	16.680	13.397
12	0.0511	7.012	8.022	4.817	9.109	13.157
12	0.0521	13.661	10.993	9.033	22.735	11.027
12	0.0531	11.708	14.074	10.336	17.786	14.074
12	0.0541	15.396	8.699	13.762	14.003	13.854
12	0.0551	12.555	11.037	14.540	12.975	17.637
12	0.0561	10.271	12.475	13.240	14.524	12.505
12	0.0571	10.215	12.443	13.178	14.492	12.555
12	0.0581	10.166	12.417	13.116	14.460	12.604
12	0.0591	8.175	8.410	8.184	10.882	10.946
12	0.0601	12.670	15.098	10.794	19.250	14.134
12	0.0611	13.818	19.679	13.970	19.418	12.924
12	0.0621	13.896	19.797	14.085	19.468	12.881
12	0.0631	15.512	17.287	21.556	35.235	12.598
12	0.0641	18.372	18.058	24.752	29.610	13.037
12	0.0651	13.360	10.927	7.918	21.852	13.563
12	0.0661	9.964	10.585	10.238	24.153	19.694
12	0.0671	11.152	14.618	10.322	27.713	15.483
12	0.0681	20.835	17.287	16.309	26.502	22.255
12	0.0691	8.272	14.674	8.274	23.099	22.617
12	0.0701	13.602	11.099	14.974	21.117	29.687
12	0.0711	6.066	8.139	13.247	15.913	22.124
12	0.0721	14.543	17.233	19.659	22.528	27.200
12	0.0731	21.086	21.768	24.753	17.953	19.495
12	0.0741	12.554	13.259	14.337	20.982	13.301
12	0.0751	9.586	9.127	8.159	24.683	7.926
12	0.0761	15.776	16.177	9.914	8.947	15.270
12	0.0771	13.718	8.534	10.261	29.742	14.970
12	0.0781	13.558	7.671	19.195	16.267	17.087
12	0.0791	18.094	14.839	29.925	14.317	18.940
12	0.0801	13.472	8.346	11.804	21.631	13.667
12	0.0811	8.044	7.009	8.449	23.207	14.331
12	0.0821	16.315	18.134	18.248	25.534	5.302
12	0.0831	19.837	17.270	17.542	24.112	23.170
12	0.0841	19.003	23.274	18.797	23.256	18.007
12	0.0851	17.175	29.987	22.705	22.585	19.208
12	0.0861	25.482	31.192	24.226	24.252	7.760
12	0.0871	20.673	24.308	20.645	39.979	21.145
12	0.0881	11.219	12.227	13.192	34.509	12.299
12	0.0891	14.036	14.567	16.388	31.666	17.723
12	0.0901	4.602	8.137	5.217	15.903	6.946
12	0.0911	12.553	19.061	15.164	22.229	13.483

12	0.0921	21.180	27.114	26.767	22.382	15.042
12	0.0931	21.561	20.345	20.996	26.791	7.643
12	0.0941	17.696	26.479	22.606	22.586	24.222
12	0.0951	18.575	18.481	19.446	8.253	16.583
12	0.0961	15.334	19.442	16.516	19.683	16.909
12	0.0971	7.470	18.555	10.438	29.938	9.704
12	0.0981	7.139	13.203	5.948	16.281	6.309
12	0.0991	25.780	16.412	16.814	24.187	18.391
12	0.1001	11.410	15.803	12.139	31.844	10.947
12	0.1011	10.386	13.143	12.289	27.686	22.001
12	0.1021	10.316	10.559	15.986	16.539	16.541
12	0.1031	20.332	10.682	17.576	25.181	12.142
12	0.1041	17.487	16.226	13.745	11.056	10.817
12	0.1051	22.681	11.667	27.452	23.953	17.806
12	0.1061	9.709	13.565	14.055	31.179	14.190
12	0.1071	12.293	21.258	18.782	12.782	16.176
12	0.1081	18.106	24.064	20.081	20.972	20.024
12	0.1091	10.343	16.515	10.495	29.127	15.820
12	0.1101	10.147	15.946	8.423	30.310	18.380
12	0.1111	17.409	17.292	13.622	30.290	13.066
12	0.1121	16.239	13.937	13.226	30.773	21.650
12	0.1131	13.532	18.356	11.754	29.018	16.852
12	0.1141	16.690	12.363	14.813	20.127	21.693
12	0.1151	14.811	8.998	9.833	22.496	21.304
12	0.1161	9.620	13.113	12.610	9.255	16.026
12	0.1171	21.736	24.336	25.086	17.875	11.688
12	0.1181	14.245	21.639	12.075	16.683	19.638
12	0.1191	15.368	22.458	14.890	19.352	19.265
12	0.1201	21.624	27.158	22.355	21.580	14.729
12	0.1211	17.055	25.812	18.767	32.012	8.500
12	0.1221	19.458	15.350	19.149	26.228	15.238
12	0.1231	14.075	18.570	16.769	21.714	9.376
12	0.1241	12.977	21.895	15.931	22.306	10.285
12	0.1251	12.553	17.882	12.211	20.067	8.390
12	0.1261	16.929	10.410	13.487	25.180	10.791
12	0.1271	16.563	16.029	13.328	28.978	22.082
12	0.1281	12.434	14.318	10.977	20.671	15.447
12	0.1291	17.895	20.923	16.887	16.078	10.211
12	0.1301	30.535	26.042	20.129	22.417	15.224
12	0.1311	16.194	22.287	18.076	20.248	11.125
12	0.1321	28.310	33.955	32.919	22.328	16.744
12	0.1331	33.133	26.367	28.542	22.218	11.258
12	0.1341	40.616	44.679	41.871	24.929	17.169
12	0.1351	26.335	26.088	23.951	26.679	18.874
12	0.1361	25.425	16.255	21.041	22.780	17.152
12	0.1371	19.146	16.317	16.836	16.803	10.691
12	0.1381	11.624	9.030	5.728	18.871	13.692
12	0.1391	20.466	28.570	19.294	12.628	4.372
12	0.1401	23.908	33.870	22.947	8.699	13.741
12	0.1411	27.344	41.633	32.816	10.352	15.897

12	0.1421	26.500	30.948	27.565	28.814	15.846
12	0.1431	26.861	29.610	25.570	26.675	22.049
12	0.1441	20.829	21.281	17.381	21.476	36.453
12	0.1451	17.923	11.048	13.046	17.025	21.922
12	0.1461	11.391	17.149	13.919	19.603	16.935
12	0.1471	23.569	24.549	22.744	15.214	21.446
12	0.1481	23.951	26.602	19.793	21.628	18.060
12	0.1491	28.624	34.410	32.922	27.445	13.972
12	0.1501	37.502	48.984	33.023	23.544	29.971
12	0.1511	36.257	32.555	28.009	16.573	26.011
12	0.1521	18.685	13.981	9.794	20.574	10.223
12	0.1531	20.841	19.522	21.407	17.903	7.932
12	0.1541	34.977	28.824	24.244	29.238	16.242
12	0.1551	9.639	21.269	10.260	24.667	14.584
12	0.1561	17.643	23.358	15.569	16.661	9.598
12	0.1571	20.105	15.899	20.433	22.119	16.897
12	0.1581	15.063	18.746	18.989	24.556	9.783
12	0.1591	14.240	23.343	17.927	16.702	11.489
12	0.1601	12.692	15.368	12.681	10.950	5.474
12	0.1611	14.871	22.122	18.583	14.428	11.983
12	0.1621	22.579	29.394	25.223	19.604	16.446
12	0.1631	18.435	24.833	21.574	20.489	14.046
12	0.1641	18.664	21.892	19.319	15.738	11.463
12	0.1651	26.377	33.257	27.393	19.109	21.614
12	0.1661	21.941	20.121	21.955	18.986	15.218
12	0.1671	22.544	18.927	20.894	18.956	16.078
12	0.1681	18.621	20.090	18.309	6.232	17.108
12	0.1691	19.055	18.829	21.566	9.798	7.587
12	0.1701	17.218	31.568	20.012	20.215	13.308
12	0.1711	29.405	34.816	35.601	18.447	22.127
12	0.1721	16.965	18.399	19.631	12.750	13.500
12	0.1731	16.726	15.795	16.188	17.428	12.011
12	0.1741	11.985	12.711	9.973	15.089	17.438
12	0.1751	13.079	19.304	14.212	16.807	16.498
12	0.1761	14.964	24.631	18.718	13.034	12.730
12	0.1771	12.891	21.338	18.974	13.032	13.090
12	0.1781	13.513	17.560	20.519	8.450	5.200
12	0.1791	7.980	13.795	10.433	14.969	7.350
12	0.1801	7.420	10.596	8.057	12.545	6.313
12	0.1811	13.577	15.742	12.183	17.962	15.843
12	0.1821	6.870	15.783	12.097	12.851	3.348
12	0.1831	9.788	16.937	15.608	9.594	5.205
12	0.1841	10.313	17.113	13.832	14.667	14.003
12	0.1851	15.073	24.016	18.780	17.692	14.439
12	0.1861	22.539	22.653	16.041	10.029	12.823
12	0.1871	11.976	13.558	9.856	22.025	18.164
12	0.1881	11.109	14.058	10.021	22.034	18.777
12	0.1891	30.253	43.879	29.760	23.571	21.936
12	0.1901	10.537	12.346	13.973	9.174	7.864
12	0.1911	15.905	16.163	16.491	10.785	8.823

12	0.1921	27.036	31.641	30.692	18.294	18.879
12	0.1931	22.808	20.510	24.831	16.646	22.868
12	0.1941	14.000	29.188	16.427	24.140	14.812
12	0.1951	12.056	19.878	13.651	24.148	12.645
12	0.1961	9.572	8.513	7.807	7.980	11.056
12	0.1971	5.408	17.989	5.989	16.002	9.528
12	0.1981	20.288	17.555	16.428	17.627	18.576
12	0.1991	9.186	16.790	12.256	34.159	19.121
12	0.2001	11.796	19.638	15.679	31.689	17.699
12	0.2011	13.851	16.276	11.957	37.091	11.684
12	0.2021	13.754	18.306	14.777	34.468	7.306
12	0.2031	7.988	6.175	7.122	13.709	14.560
12	0.2041	8.656	6.710	9.279	12.505	18.823
12	0.2051	10.936	14.093	14.705	7.693	10.022
12	0.2061	9.375	18.765	14.249	14.284	11.464
12	0.2071	6.993	17.159	14.326	20.261	17.512
12	0.2081	16.494	19.421	15.984	19.302	31.352
12	0.2091	9.332	15.005	10.012	33.662	21.283
12	0.2101	17.453	15.544	15.730	15.093	7.401
12	0.2111	16.583	18.611	18.683	8.158	12.335
12	0.2121	12.029	17.388	11.156	17.919	11.516
12	0.2131	9.019	14.618	11.049	20.021	11.777
12	0.2141	10.484	17.124	8.366	9.734	12.392
12	0.2151	9.958	15.943	7.463	13.095	15.351
12	0.2161	14.675	15.052	11.225	18.822	22.748
12	0.2171	25.588	19.837	22.004	17.110	20.584
12	0.2181	9.480	15.365	10.774	22.061	22.186
12	0.2191	12.946	17.070	15.001	16.606	18.838
12	0.2201	11.243	17.194	12.994	17.746	19.767
12	0.2211	13.029	9.990	10.602	9.754	12.864
12	0.2221	16.441	12.894	11.368	16.231	21.706
12	0.2231	7.400	6.999	6.107	12.179	18.312
12	0.2241	9.334	9.567	9.322	6.057	14.553
12	0.2251	12.365	12.845	11.573	12.308	12.302
12	0.2261	8.299	15.658	8.800	12.632	11.082
12	0.2271	16.502	19.025	13.260	18.733	15.201
12	0.2281	13.397	23.898	15.174	29.546	18.452
12	0.2291	6.438	14.645	9.695	27.615	19.140
12	0.2301	6.644	9.271	6.912	13.957	14.410
12	0.2311	12.416	15.186	13.161	18.926	9.352
12	0.2321	12.068	16.280	13.201	12.894	8.445
12	0.2331	13.449	20.265	14.865	18.359	17.019
12	0.2341	10.140	18.370	11.949	15.270	20.974
12	0.2351	5.990	10.393	6.095	11.902	17.998
12	0.2361	3.522	7.014	5.442	6.322	7.287
12	0.2371	1.895	4.848	1.016	7.038	2.581
12	0.2381	5.976	10.287	5.937	12.818	17.145
12	0.2391	3.083	6.037	2.267	4.898	1.589
12	0.2401	3.500	9.617	3.058	6.871	2.379
12	0.2411	2.345	8.698	3.352	10.382	2.472

12	0.2421	13.266	11.403	13.253	10.157	7.485
12	0.2431	6.623	7.414	8.561	9.090	12.550
12	0.2441	22.709	34.290	19.367	13.517	20.579
12	0.2451	12.090	8.534	9.224	13.875	11.831
12	0.2461	20.734	26.934	21.004	23.748	9.435
12	0.2471	14.793	19.336	21.348	15.776	9.955
12	0.2481	18.429	17.686	20.768	14.182	16.714
12	0.2491	7.327	9.854	5.737	14.934	13.121
12	0.2501	14.403	12.466	18.100	11.920	27.095
12	0.2511	14.464	20.911	20.832	22.206	10.497
12	0.2521	9.885	17.144	15.319	15.607	12.243
12	0.2531	16.068	10.810	13.520	7.141	12.858
12	0.2541	2.944	5.320	2.891	1.662	3.083
12	0.2551	7.602	7.669	8.877	2.938	8.430
12	0.2561	15.948	16.532	17.126	12.364	7.538
12	0.2571	12.914	8.436	15.016	13.142	11.956
12	0.2581	23.164	17.883	18.498	9.896	10.086
12	0.2591	14.350	8.162	7.354	10.730	10.179
12	0.2601	12.062	17.453	13.915	14.176	14.863
12	0.2611	10.921	11.828	12.665	16.135	25.826
12	0.2621	12.769	15.242	14.289	7.705	9.337
12	0.2631	8.532	8.961	9.233	12.496	9.350
12	0.2641	9.171	10.028	8.678	11.868	15.460
12	0.2651	5.457	7.463	3.080	16.068	10.510
12	0.2661	14.459	19.402	12.888	11.396	7.886
12	0.2671	13.435	13.931	16.263	11.102	9.475
12	0.2681	9.808	16.017	13.204	17.942	18.040
12	0.2691	12.357	16.655	12.154	22.062	15.564
12	0.2701	12.911	20.266	11.210	23.832	19.560
12	0.2711	14.745	7.608	11.257	12.903	16.995
12	0.2721	13.818	6.500	9.904	13.028	16.730
12	0.2731	20.883	16.185	15.136	10.342	14.389
12	0.2741	9.812	12.310	9.169	10.169	10.277
12	0.2751	12.925	7.800	12.367	14.765	10.183
12	0.2761	14.902	25.309	20.454	26.722	16.079
12	0.2771	28.847	17.152	21.440	22.559	7.802
12	0.2781	17.234	16.156	12.614	16.439	15.966
12	0.2791	11.955	14.047	13.100	15.556	15.589
12	0.2801	8.808	5.889	6.580	14.085	8.211
12	0.2811	36.027	15.083	20.848	6.992	9.281
12	0.2821	5.725	9.211	10.271	6.035	15.147
12	0.2831	6.590	5.986	6.956	11.298	6.897
12	0.2841	6.655	5.962	6.850	11.310	6.874
12	0.2851	17.225	9.423	12.645	14.859	11.913
12	0.2861	23.268	14.517	14.450	20.538	9.744
12	0.2871	19.119	10.699	12.576	19.731	12.493
12	0.2881	23.276	14.673	16.451	15.767	15.412
12	0.2891	21.258	11.321	11.411	14.090	16.896
12	0.2901	4.823	1.854	4.479	12.426	12.577
12	0.2911	15.701	15.369	16.538	16.364	23.637

12	0.2921	12.142	12.544	7.249	23.973	15.771
12	0.2931	12.038	13.539	11.472	20.658	17.328
12	0.2941	27.737	20.147	12.828	16.267	20.687
12	0.2951	10.865	5.982	10.182	4.767	12.083
12	0.2961	10.460	9.830	11.235	5.605	7.878
12	0.2971	11.650	11.681	11.855	19.002	12.564
12	0.2981	9.434	9.492	6.147	17.053	21.311
12	0.2991	6.686	16.903	8.172	26.284	16.531
12	0.3001	17.542	14.462	11.183	13.088	12.703
12	0.3011	18.673	11.063	12.427	14.731	19.622
12	0.3021	17.677	21.878	14.950	14.832	7.260
12	0.3031	10.019	7.006	8.012	16.891	11.063
12	0.3041	8.916	13.364	10.431	24.111	16.675
12	0.3051	12.666	7.982	11.396	26.257	17.013
12	0.3061	20.663	14.530	13.072	25.476	12.679
12	0.3071	22.513	8.558	11.056	20.270	21.967
12	0.3081	22.596	17.139	13.167	23.838	25.045
12	0.3091	31.580	25.703	18.054	19.169	19.570
12	0.3101	18.414	15.457	12.642	20.737	14.055
12	0.3111	9.530	15.882	8.979	21.837	13.135
12	0.3121	13.675	9.253	12.667	21.048	16.368
12	0.3131	15.414	14.559	16.550	12.563	14.952
12	0.3141	12.759	12.726	11.323	20.953	15.644
12	0.3151	16.192	14.889	11.456	10.048	13.560
12	0.3161	23.611	13.882	15.312	6.124	14.467
12	0.3171	17.357	15.216	9.950	15.789	10.698
12	0.3181	18.095	17.026	12.188	19.712	10.505
12	0.3191	14.631	11.653	8.191	15.965	18.690
12	0.3201	11.055	5.181	6.824	16.385	21.225
12	0.3211	16.633	16.010	16.023	14.876	16.188
12	0.3221	19.437	20.975	16.596	18.764	35.552
12	0.3231	19.047	18.334	15.897	10.710	13.770
12	0.3241	12.052	12.341	11.729	10.461	9.863
12	0.3251	3.600	5.244	4.904	12.058	7.623
12	0.3261	13.798	18.332	9.753	16.552	12.839
12	0.3271	10.649	11.563	13.123	17.075	14.117
12	0.3281	12.801	12.141	14.177	22.087	18.810
12	0.3291	13.497	12.107	10.967	16.345	15.509
12	0.3301	32.806	22.445	24.378	20.489	19.469
12	0.3311	24.294	20.146	16.835	16.656	17.829
12	0.3321	8.721	7.561	5.735	13.674	10.421
12	0.3331	5.092	4.234	4.216	7.454	7.970
12	0.3341	13.421	15.932	12.304	19.369	18.982
12	0.3351	19.342	20.190	20.778	8.964	13.544
12	0.3361	8.219	4.007	6.664	3.902	7.501
12	0.3371	8.622	10.267	6.606	8.377	6.015
12	0.3381	15.493	19.293	10.391	15.492	16.241
12	0.3391	7.499	5.976	6.447	9.682	10.047
12	0.3401	11.180	11.845	6.276	14.473	8.499
12	0.3411	7.803	8.291	5.300	14.840	5.719

12	0.3421	11.825	12.481	9.985	12.724	9.058
12	0.3431	3.398	0.620	1.962	6.542	4.828
12	0.3441	6.109	6.227	4.262	8.075	7.078
12	0.3451	15.160	9.902	13.547	14.832	21.410
12	0.3461	16.168	10.539	16.200	12.499	13.299
12	0.3471	9.757	10.712	12.229	2.452	4.349
12	0.3481	23.345	15.290	18.130	13.120	21.164
12	0.3491	16.805	15.209	9.606	14.499	18.123
12	0.3501	19.634	24.904	16.319	13.117	17.063
12	0.3511	12.306	13.060	12.890	7.180	3.479
12	0.3521	9.102	7.287	8.083	14.045	10.368
12	0.3531	7.737	4.868	7.166	15.059	14.089
12	0.3541	7.570	5.756	7.248	20.148	9.126
12	0.3551	9.716	5.124	9.532	24.450	8.855
12	0.3561	5.306	10.947	7.842	16.460	8.940
12	0.3571	11.884	8.384	12.547	20.217	9.126
12	0.3581	11.703	7.773	12.048	15.357	8.808
12	0.3591	13.076	12.811	12.928	18.055	7.048
12	0.3601	16.097	10.009	9.853	28.229	25.123
12	0.3611	16.730	10.715	10.024	10.476	8.068
12	0.3621	13.357	4.493	7.391	12.823	9.472
12	0.3631	7.142	5.571	4.314	13.669	10.953
12	0.3641	8.989	8.785	10.359	4.951	5.359
12	0.3651	13.223	9.756	11.439	12.961	14.572
12	0.3661	8.796	7.090	8.215	8.034	12.411
12	0.3671	10.844	16.638	10.129	11.697	6.988
12	0.3681	12.754	12.552	13.219	15.473	5.885
12	0.3691	14.734	8.350	17.356	18.713	11.238
12	0.3701	6.040	5.180	8.122	3.861	4.117
12	0.3711	14.134	8.054	11.704	22.137	17.468
12	0.3721	18.247	8.550	10.198	19.555	19.941
12	0.3731	6.799	4.760	3.295	12.669	8.243
12	0.3741	2.589	2.225	2.768	14.054	6.244
12	0.3751	9.513	9.204	11.421	23.027	17.401
12	0.3761	17.046	12.049	12.372	14.432	16.773
12	0.3771	13.157	19.981	13.640	24.378	13.119
12	0.3781	12.475	13.589	13.378	18.235	21.009
12	0.3791	18.621	14.547	13.537	16.820	9.522
12	0.3801	11.474	7.396	13.015	16.382	11.338
12	0.3811	17.468	16.506	15.150	18.865	18.856
12	0.3821	18.067	16.574	15.437	31.209	20.935
12	0.3831	18.578	20.308	16.263	15.836	17.997
12	0.3841	12.782	12.288	8.951	19.883	22.569
12	0.3851	12.421	15.949	10.181	27.786	22.334
12	0.3861	11.891	13.827	11.232	21.867	15.782
12	0.3871	3.600	6.280	6.040	4.360	13.017
12	0.3881	6.160	3.157	5.829	6.547	9.993
12	0.3891	14.141	7.533	12.556	21.162	21.297
12	0.3901	9.441	5.886	9.447	14.204	7.588
12	0.3911	14.346	17.810	15.949	14.405	8.655

12	0.3921	10.574	8.019	6.849	18.784	15.730
12	0.3931	4.533	7.192	5.279	13.692	11.905
12	0.3941	6.186	6.162	6.931	14.812	8.737
12	0.3951	6.069	6.297	7.852	15.571	7.911
12	0.3961	7.067	7.095	6.549	18.977	11.866
12	0.3971	4.499	7.164	4.652	23.975	8.881
12	0.3981	8.973	7.265	5.896	26.698	12.734
12	0.3991	7.287	8.388	4.769	10.584	6.722
12	0.4001	10.294	14.862	10.327	15.395	15.303
12	0.4011	6.093	9.520	4.364	11.714	9.375
12	0.4021	5.806	7.623	5.120	7.116	10.896
12	0.4031	6.396	7.263	4.014	6.550	13.698
12	0.4041	3.178	5.738	4.436	6.490	6.877
12	0.4051	2.885	4.514	3.923	6.347	4.641
12	0.4061	11.069	7.808	7.947	8.503	9.215
12	0.4071	9.612	9.161	10.579	12.354	12.222
12	0.4081	20.854	14.476	20.029	12.324	12.130
12	0.4091	21.843	13.254	19.223	18.287	19.938
12	0.4101	22.350	13.345	19.448	18.052	19.584
12	0.4111	21.974	13.281	19.126	17.395	19.045
12	0.4121	18.587	14.286	12.258	10.766	18.655
12	0.4131	17.947	14.286	12.286	10.779	18.637
12	0.4141	13.855	17.965	16.264	14.424	15.533
12	0.4151	10.763	18.397	12.232	15.722	18.429
12	0.4161	8.641	9.308	13.219	14.795	10.778
12	0.4171	15.898	10.615	19.661	12.063	24.539
12	0.4181	10.525	10.133	7.874	10.500	21.721
12	0.4191	6.370	8.277	7.956	8.134	9.928
12	0.4201	12.267	15.257	17.196	7.293	11.174
12	0.4211	11.136	12.819	13.822	14.244	7.251
12	0.4221	16.759	16.616	14.196	11.087	9.211
12	0.4231	10.315	12.854	6.808	16.992	14.449
12	0.4241	8.574	11.692	5.806	10.059	13.625
12	0.4251	12.954	16.965	16.988	16.677	17.876
12	0.4261	14.016	16.684	13.292	11.712	13.301
12	0.4271	9.993	8.784	7.429	14.593	16.893
12	0.4281	10.111	11.485	10.285	17.662	23.599
12	0.4291	10.145	11.107	11.922	14.582	23.378
12	0.4301	7.026	10.649	10.882	25.329	25.730
12	0.4311	16.638	16.110	13.048	23.508	13.703
12	0.4321	18.534	19.593	19.232	15.628	10.676
12	0.4331	14.116	13.818	17.106	13.539	6.342
12	0.4341	11.918	19.761	12.345	22.957	16.391
12	0.4351	9.813	14.417	9.108	11.010	4.691
12	0.4361	10.834	8.763	7.928	12.461	13.813
12	0.4371	15.410	13.143	13.987	14.214	15.339
12	0.4381	16.908	12.640	9.246	16.010	18.139
12	0.4391	5.838	7.067	10.563	10.879	13.695
12	0.4401	11.000	4.944	6.996	23.335	16.101
12	0.4411	13.909	8.918	10.660	14.526	10.608

12	0.4421	13.639	11.551	13.096	12.058	9.256
12	0.4431	13.882	16.126	14.046	17.873	16.563
12	0.4441	16.389	11.344	10.287	8.526	18.212
12	0.4451	11.232	4.535	3.975	12.018	18.654
12	0.4461	8.021	6.030	6.223	11.970	14.040
12	0.4471	20.041	22.852	18.535	10.876	20.031
12	0.4481	11.437	10.815	12.244	5.756	9.187
12	0.4491	10.100	13.218	14.992	13.183	26.090
12	0.4501	12.307	14.056	14.612	9.136	11.727
12	0.4511	24.524	27.966	28.124	14.991	21.107
12	0.4521	15.265	20.123	16.094	10.905	10.204
12	0.4531	21.354	22.994	13.750	10.476	6.671
12	0.4541	7.554	12.410	5.739	12.546	9.459
12	0.4551	8.302	9.218	6.415	10.543	12.537
12	0.4561	13.928	12.484	10.544	8.052	6.908
12	0.4571	10.072	12.402	10.789	16.033	22.252
12	0.4581	9.464	15.502	13.107	15.316	22.366
12	0.4591	15.143	20.493	11.826	11.771	12.363
12	0.4601	17.135	16.396	12.180	12.600	13.958
12	0.4611	17.187	22.570	16.106	6.401	16.831
12	0.4621	21.954	20.259	21.761	14.183	21.526
12	0.4631	9.886	18.280	12.163	17.758	13.439
12	0.4641	14.614	8.385	12.782	16.114	24.463
12	0.4651	12.769	10.902	8.644	8.370	22.072
12	0.4661	20.428	23.844	21.666	15.078	16.185
12	0.4671	10.298	17.102	10.015	20.963	20.401
12	0.4681	26.534	22.271	23.664	9.043	15.806
12	0.4691	26.723	27.285	25.884	11.583	14.620
12	0.4701	16.841	17.066	22.090	8.851	8.959
12	0.4711	18.757	19.999	17.034	6.560	11.696
12	0.4721	9.761	21.358	12.920	14.658	17.977
12	0.4731	10.112	21.694	16.983	10.852	13.448
12	0.4741	9.947	11.473	12.546	10.543	11.557
12	0.4751	7.424	9.229	8.114	10.276	12.140
12	0.4761	8.767	8.118	8.024	12.834	8.169
12	0.4771	8.079	5.202	7.479	10.699	7.760
12	0.4781	11.773	6.371	9.320	6.571	10.631
12	0.4791	19.517	15.814	12.587	7.063	9.326
12	0.4801	2.310	3.286	2.304	4.030	8.669
12	0.4811	5.724	10.355	8.444	18.506	24.488
12	0.4821	18.767	24.179	20.225	23.162	13.014
12	0.4831	10.688	15.119	11.950	10.726	18.579
12	0.4841	16.822	10.501	12.533	9.910	16.154
12	0.4851	22.275	24.055	18.461	14.804	19.514
12	0.4861	13.173	10.007	9.974	13.696	15.542
12	0.4871	8.901	6.939	8.484	9.151	8.185
12	0.4881	19.077	8.047	12.673	14.402	22.090
12	0.4891	12.679	13.185	8.715	15.023	20.065
12	0.4901	17.267	16.447	14.920	10.838	9.167
12	0.4911	21.940	20.201	17.097	7.845	13.231

12	0.4921	14.320	16.538	16.874	7.862	13.464
12	0.4931	17.299	22.335	19.314	10.282	19.207
12	0.4941	23.024	27.187	20.211	18.448	18.456
12	0.4951	28.263	22.616	27.326	13.667	9.657
12	0.4961	14.932	13.860	17.421	3.601	10.818
12	0.4971	20.496	20.924	14.012	16.106	10.563
12	0.4981	25.040	19.465	17.821	6.048	23.675
12	0.4991	13.028	10.582	9.718	10.554	17.092
12	0.5001	15.268	12.369	11.128	9.234	14.712
12	0.5011	22.555	23.131	15.096	12.446	13.548
12	0.5021	29.415	21.745	16.930	11.525	20.978
12	0.5031	15.324	12.124	11.039	13.617	4.487
12	0.5041	10.504	10.848	9.599	18.807	15.106
12	0.5051	19.699	12.102	7.623	12.291	17.146
12	0.5061	19.368	12.078	7.656	12.527	16.607
12	0.5071	15.314	6.950	7.708	8.876	14.815
12	0.5081	20.198	10.739	13.838	16.804	21.434
12	0.5091	8.072	3.337	9.198	10.978	14.454
12	0.5101	16.095	10.165	12.269	16.616	12.004
12	0.5111	18.346	15.185	14.473	16.428	14.623
12	0.5121	23.802	12.928	17.257	10.126	8.555
12	0.5131	12.328	8.567	7.780	9.211	16.268
12	0.5141	13.618	7.046	10.898	8.981	22.009
12	0.5151	14.390	11.545	11.717	18.851	17.843
12	0.5161	17.977	10.349	11.721	13.049	16.200
12	0.5171	12.823	10.918	10.195	7.908	3.668
12	0.5181	6.897	5.392	5.905	6.333	8.218
12	0.5191	12.515	9.252	10.271	17.086	9.066
12	0.5201	15.863	9.057	11.105	12.499	12.539
12	0.5211	11.867	10.146	13.395	8.812	12.884
12	0.5221	12.028	11.505	14.641	8.770	13.145
12	0.5231	11.433	10.774	10.315	7.809	14.688
12	0.5241	7.774	10.379	5.512	19.266	9.955
12	0.5251	9.283	5.433	10.602	14.321	19.300
12	0.5261	9.405	5.873	7.934	11.637	8.032
12	0.5271	8.686	7.675	8.408	13.930	6.058
12	0.5281	8.980	11.621	8.832	13.421	10.621
12	0.5291	9.967	12.401	5.966	6.256	8.548
12	0.5301	18.180	16.602	15.090	8.226	9.683
12	0.5311	7.695	11.051	8.894	8.202	10.559
12	0.5321	7.696	9.503	7.087	10.770	9.370
12	0.5331	8.567	11.019	7.041	12.878	7.083
12	0.5341	8.633	13.688	6.453	8.608	5.330
12	0.5351	7.381	10.573	7.129	10.683	13.749
12	0.5361	6.585	5.858	4.635	14.353	10.078
12	0.5371	9.061	9.827	6.241	18.026	6.368
12	0.5381	10.254	9.392	11.114	6.195	5.307
12	0.5391	9.949	7.229	4.820	21.470	23.711
12	0.5401	11.519	13.107	9.590	12.702	8.990
12	0.5411	30.674	20.014	22.945	9.913	10.372

12	0.5421	19.900	13.723	17.918	12.391	6.279
12	0.5431	28.281	18.291	20.203	9.576	18.614
12	0.5441	18.072	12.071	12.686	13.333	13.081
12	0.5451	13.959	11.978	12.163	8.591	9.915
12	0.5461	14.222	19.835	14.487	24.884	16.489
12	0.5471	16.575	12.692	9.603	15.925	15.148
12	0.5481	22.595	21.616	21.669	16.194	15.497
12	0.5491	17.515	12.444	12.601	13.685	19.637
12	0.5501	17.483	10.952	10.311	16.745	14.849
12	0.5511	11.616	12.615	13.249	15.252	8.094
12	0.5521	13.753	14.509	10.390	8.282	5.602
12	0.5531	18.815	18.318	11.914	23.267	20.149
12	0.5541	12.308	10.361	10.536	20.263	13.350
12	0.5551	18.835	14.256	15.942	31.590	22.139
12	0.5561	21.363	29.168	25.521	16.974	9.187
12	0.5571	16.475	18.764	13.790	7.021	15.491
12	0.5581	11.309	11.883	10.419	8.592	9.727
12	0.5591	10.928	19.184	12.603	19.941	12.824
12	0.5601	26.980	30.169	19.087	19.832	16.472
12	0.5611	18.199	13.280	13.367	16.139	14.918
12	0.5621	8.094	4.803	3.478	9.245	13.473
12	0.5631	6.196	1.054	1.485	5.745	17.276
12	0.5641	13.578	13.859	14.254	14.869	19.518
12	0.5651	17.776	14.960	18.068	12.912	11.447
12	0.5661	15.965	13.270	13.719	8.852	8.838
12	0.5671	18.417	18.157	14.151	8.942	12.285
12	0.5681	12.836	12.051	11.720	8.550	8.511
12	0.5691	11.338	9.112	10.727	18.581	11.067
12	0.5701	14.358	13.818	15.083	8.785	16.564
12	0.5711	8.193	8.986	8.929	7.185	3.418
12	0.5721	12.944	16.755	13.376	10.586	10.458
12	0.5731	15.657	10.727	13.534	13.280	13.677
12	0.5741	11.852	12.022	9.669	10.696	10.459
12	0.5751	18.051	20.471	10.492	10.868	12.926
12	0.5761	17.398	12.140	10.463	7.155	17.235
12	0.5771	11.957	6.768	4.122	6.804	16.346
12	0.5781	7.085	2.352	2.190	12.781	11.008
12	0.5791	13.532	19.734	14.899	19.354	7.078
12	0.5801	14.832	18.217	12.604	14.359	14.046
12	0.5811	16.831	20.250	15.350	12.379	11.389
12	0.5821	10.532	11.689	8.366	11.776	11.958
12	0.5831	11.889	12.553	10.576	15.903	14.954
12	0.5841	28.702	25.396	21.404	13.841	11.511
12	0.5851	18.550	12.538	18.656	9.352	7.730
12	0.5861	14.347	13.383	14.620	17.335	16.106
12	0.5871	16.028	14.623	16.529	17.297	16.094
12	0.5881	11.313	10.649	10.544	4.402	16.972
12	0.5891	12.980	14.350	11.065	8.610	11.029
12	0.5901	13.236	9.951	14.701	10.183	13.018
12	0.5911	20.194	20.118	23.384	10.589	18.794

12	0.5921	22.329	19.840	26.906	7.863	10.816
12	0.5931	11.910	13.981	19.986	7.663	14.918
12	0.5941	17.909	17.304	24.520	7.797	12.769
12	0.5951	13.206	11.304	14.746	6.790	7.432
12	0.5961	17.378	17.113	15.785	12.473	5.637
12	0.5971	21.344	17.446	16.579	14.253	14.515
12	0.5981	21.419	16.920	16.202	14.561	14.937
12	0.5991	9.291	16.519	16.369	14.577	8.797
12	0.6001	12.348	18.175	17.712	14.282	10.163
12	0.6011	12.241	16.834	17.886	13.489	6.485
12	0.6021	14.719	17.848	17.289	14.818	7.822
12	0.6031	21.057	20.606	23.116	14.863	4.742
12	0.6041	24.863	17.737	21.646	14.270	4.719
12	0.6051	24.914	17.915	21.700	14.238	4.746
12	0.6061	15.419	10.585	12.038	13.176	8.065
12	0.6071	22.765	21.319	19.618	11.951	3.537
12	0.6081	14.934	18.256	18.489	15.000	12.952
12	0.6091	14.296	22.633	18.037	20.146	17.308
12	0.6101	30.468	33.539	36.246	15.155	8.283
12	0.6111	19.193	24.151	30.999	12.794	2.870
12	0.6121	22.214	19.841	29.179	8.659	4.614
12	0.6131	17.611	17.162	24.646	15.059	6.958
12	0.6141	26.290	25.469	30.294	9.605	14.636
12	0.6151	22.006	19.672	25.302	5.445	7.020
12	0.6161	13.492	11.494	19.717	12.745	21.801
12	0.6171	22.772	27.298	25.910	9.114	11.722
12	0.6181	23.426	28.997	26.460	8.565	12.733
12	0.6191	17.271	14.097	17.214	5.713	10.723
12	0.6201	22.818	15.461	21.037	14.234	6.322
12	0.6211	20.476	14.412	21.637	12.287	13.483
12	0.6221	18.065	19.186	23.436	10.674	17.838
12	0.6231	13.577	17.464	21.012	7.151	8.517
12	0.6241	12.263	11.145	15.096	6.171	12.334
12	0.6251	22.879	21.900	32.783	17.457	16.234
12	0.6261	24.098	20.330	27.105	22.356	13.189
12	0.6271	26.764	18.114	24.627	9.433	10.352
12	0.6281	15.771	20.228	16.503	10.832	9.794
12	0.6291	10.746	17.269	11.060	15.999	10.802
12	0.6301	12.496	11.262	13.065	16.156	15.026
12	0.6311	14.983	17.908	22.641	13.765	20.731
12	0.6321	14.994	14.637	22.348	15.417	17.512
12	0.6331	14.593	14.015	20.219	10.916	10.063
12	0.6341	8.773	11.911	11.749	14.416	14.140
12	0.6351	7.458	8.714	12.442	4.852	11.471
12	0.6361	19.502	26.433	19.745	7.566	28.730
12	0.6371	15.640	21.947	18.640	11.844	22.196
12	0.6381	9.791	15.407	15.393	7.690	19.056
12	0.6391	7.988	8.953	14.403	4.151	10.111
12	0.6401	7.178	11.845	13.180	7.905	17.079
12	0.6411	16.922	14.435	20.378	5.505	12.092

12	0.6421	13.869	16.893	15.775	5.972	14.493
12	0.6431	9.659	11.326	11.336	8.716	16.360
12	0.6441	3.503	5.287	6.357	12.992	6.180
12	0.6451	13.553	14.068	15.138	12.803	5.151
12	0.6461	12.520	11.241	16.359	10.146	22.305
12	0.6471	11.504	17.613	14.501	5.832	16.544
12	0.6481	16.608	20.742	21.510	13.037	17.991
12	0.6491	13.226	10.950	18.311	8.410	11.110
12	0.6501	15.290	15.482	16.757	14.863	25.290
12	0.6511	12.224	13.926	15.262	8.855	8.599
12	0.6521	7.136	8.213	9.362	6.869	8.224
12	0.6531	9.948	8.588	12.914	8.770	4.555
12	0.6541	15.881	10.168	16.323	17.475	17.915
12	0.6551	9.782	16.360	15.580	14.710	10.782
12	0.6561	16.595	13.315	20.869	15.899	12.533
12	0.6571	12.993	12.384	14.338	8.141	19.507
12	0.6581	8.538	10.581	14.502	8.316	16.986
12	0.6591	8.615	10.954	8.031	4.135	8.018
12	0.6601	7.511	9.102	9.004	8.462	15.683
12	0.6611	6.024	8.006	4.205	11.460	20.692
12	0.6621	21.689	10.953	16.141	14.389	19.743
12	0.6631	26.562	17.821	26.012	20.619	12.855
12	0.6641	29.548	23.867	28.595	10.161	14.613
12	0.6651	18.391	15.545	16.563	8.141	17.866
12	0.6661	20.961	15.877	22.716	8.359	16.187
12	0.6671	7.814	11.293	13.075	7.849	13.857
12	0.6681	10.689	17.520	13.514	14.473	17.082
12	0.6691	19.593	15.021	14.478	21.154	19.613
12	0.6701	15.845	13.894	14.405	7.380	12.260
12	0.6711	13.787	16.311	15.961	8.907	18.422
12	0.6721	12.875	18.478	15.241	16.636	23.739
12	0.6731	20.186	13.701	15.186	7.291	20.758
12	0.6741	11.675	12.978	11.279	7.594	12.763
12	0.6751	10.001	10.796	12.868	8.586	17.275
12	0.6761	9.351	14.565	13.269	15.723	22.560
12	0.6771	9.120	14.060	12.770	15.463	22.331
12	0.6781	8.918	13.559	12.284	15.214	22.094
12	0.6791	8.747	13.071	11.819	14.981	21.854
12	0.6801	8.611	12.603	11.384	14.768	21.616
12	0.6811	8.511	12.161	10.984	14.577	21.382
12	0.6821	8.446	11.750	10.624	14.412	21.156
12	0.6831	26.875	25.823	28.478	12.113	17.320
12	0.6841	12.220	12.502	14.510	11.328	7.249
12	0.6851	0.576	1.363	0.168	1.863	0.339
12	0.6861	8.679	10.157	7.787	9.489	14.334
12	0.6871	13.663	14.933	11.411	11.819	12.443
12	0.6881	15.654	10.298	15.242	11.595	11.383
12	0.6891	12.412	10.805	16.466	12.438	29.847
12	0.6901	8.833	11.676	14.430	9.224	12.485
12	0.6911	10.152	13.568	15.898	12.959	9.804

12	0.6921	17.619	23.706	26.181	20.945	22.199
12	0.6931	12.153	17.221	19.020	13.399	13.548
12	0.6941	4.223	11.898	12.075	4.189	18.021
12	0.6951	10.217	18.839	20.738	7.866	11.614
12	0.6961	11.531	12.470	15.222	15.567	14.099
12	0.6971	14.476	18.101	12.994	12.919	8.884
12	0.6981	11.874	12.321	11.332	11.035	15.659
12	0.6991	15.164	19.451	16.937	11.940	15.716
12	0.7001	10.461	18.356	16.135	18.313	8.125
12	0.7011	5.501	8.717	7.402	15.461	10.610
12	0.7021	13.788	20.338	22.814	16.670	12.790
12	0.7031	13.800	19.175	20.867	21.133	12.914
12	0.7041	8.109	8.541	9.602	13.699	12.675
12	0.7051	16.878	26.588	16.771	16.444	9.529
12	0.7061	12.918	22.047	18.846	16.285	16.609
12	0.7071	19.735	20.539	22.842	15.376	16.762
12	0.7081	30.592	25.773	28.700	14.871	15.380
12	0.7091	21.433	31.826	29.613	14.935	18.942
12	0.7101	18.700	20.713	20.457	23.215	20.220
12	0.7111	16.048	27.816	26.287	22.042	17.445
12	0.7121	17.125	16.662	15.997	20.248	15.039
12	0.7131	19.112	16.303	20.715	7.548	17.972
12	0.7141	22.688	23.549	26.527	11.892	11.567
12	0.7151	22.865	20.243	20.184	19.018	26.023
12	0.7161	17.900	18.418	16.184	11.415	14.892
12	0.7171	11.066	12.633	15.607	15.402	9.593
12	0.7181	19.040	29.409	26.180	22.075	17.471
12	0.7191	21.766	23.172	24.464	9.964	13.309
12	0.7201	24.353	18.980	17.973	13.568	10.259
12	0.7211	17.201	13.357	16.967	8.095	13.854
12	0.7221	10.902	5.634	11.753	11.807	22.859
12	0.7231	8.249	7.494	8.123	7.929	6.240
12	0.7241	19.799	7.780	13.612	6.983	14.048
12	0.7251	29.319	19.854	20.100	6.576	10.561
12	0.7261	14.840	17.738	13.633	6.624	13.944
12	0.7271	21.657	29.276	17.985	11.141	15.630
12	0.7281	24.450	31.889	25.983	10.773	22.131
12	0.7291	26.553	25.920	22.668	9.694	13.274
12	0.7301	29.280	30.188	31.315	11.498	17.928
12	0.7311	32.589	25.119	33.729	15.001	12.130
12	0.7321	15.010	13.879	17.040	17.235	11.726
12	0.7331	18.286	11.852	10.397	4.113	9.784
12	0.7341	13.606	26.064	18.923	19.072	9.099
12	0.7351	14.751	21.030	12.605	17.396	12.766
12	0.7361	15.099	17.531	13.981	10.948	10.982
12	0.7371	32.408	19.155	15.447	8.364	14.696
12	0.7381	11.902	14.653	8.909	18.903	7.930
12	0.7391	4.066	13.693	9.545	9.800	15.385
12	0.7401	12.788	13.705	9.024	8.452	19.091
12	0.7411	10.479	9.613	7.278	4.943	22.129

12	0.7421	16.007	13.852	13.312	11.238	16.073
12	0.7431	9.612	7.854	10.218	7.108	15.357
12	0.7441	12.041	17.725	15.885	2.300	12.074
12	0.7451	12.801	15.735	12.201	4.828	14.120
12	0.7461	21.749	20.361	17.222	7.446	20.617
12	0.7471	18.294	15.370	12.784	7.934	19.569
12	0.7481	18.097	15.037	12.691	8.010	19.549
12	0.7491	3.336	4.420	5.318	7.499	9.020
12	0.7501	6.473	7.205	8.896	7.236	10.448
12	0.7511	18.572	25.336	15.295	15.825	23.417
12	0.7521	9.272	11.589	9.489	11.823	22.298
12	0.7531	21.420	16.404	15.074	15.614	20.864
12	0.7541	26.051	22.553	31.806	10.877	27.893
12	0.7551	22.272	16.460	20.908	12.077	16.043
12	0.7561	13.172	12.650	17.227	13.828	25.565
12	0.7571	11.582	6.511	10.130	17.058	19.881
12	0.7581	9.835	5.078	10.525	7.046	18.715
12	0.7591	7.357	10.729	6.397	13.533	15.048
12	0.7601	25.312	16.176	16.535	5.795	18.525
12	0.7611	18.218	19.979	24.852	9.014	24.167
12	0.7621	8.124	9.303	15.582	6.415	14.809
12	0.7631	17.199	9.708	11.379	15.783	25.077
12	0.7641	15.654	20.595	21.279	16.052	7.899
12	0.7651	16.357	19.022	19.423	23.924	14.632
12	0.7661	8.971	11.379	13.196	6.968	10.919
12	0.7671	13.907	10.771	12.420	22.162	15.402
12	0.7681	19.680	8.246	9.119	28.852	25.949
12	0.7691	5.936	9.786	6.489	15.936	15.536
12	0.7701	9.788	13.816	9.335	10.840	14.176
12	0.7711	7.811	13.635	7.967	16.470	13.158
12	0.7721	9.675	13.193	11.090	15.474	16.931
12	0.7731	6.663	7.044	8.531	7.126	15.630
12	0.7741	12.763	16.814	11.924	15.473	16.232
12	0.7751	23.779	17.871	20.185	16.042	14.874
12	0.7761	12.131	10.139	10.681	9.251	23.351
12	0.7771	9.854	14.019	5.511	12.206	21.492
12	0.7781	11.997	21.669	14.908	17.087	5.714
12	0.7791	8.818	10.799	10.956	17.410	13.214
12	0.7801	10.423	11.885	10.613	13.471	9.506
12	0.7811	10.834	18.693	17.450	17.512	13.196
12	0.7821	11.026	15.522	9.231	11.155	13.968
12	0.7831	13.009	15.618	12.411	10.225	23.904
12	0.7841	26.491	24.867	26.003	16.432	21.117
12	0.7851	18.007	17.911	17.712	24.215	22.245
12	0.7861	12.606	14.523	9.085	13.369	13.573
12	0.7871	19.216	20.264	15.753	18.356	25.142
12	0.7881	15.028	19.063	18.322	12.423	11.143
12	0.7891	13.341	12.592	14.712	13.164	14.488
12	0.7901	12.728	21.474	18.698	10.265	18.889
12	0.7911	12.565	21.313	15.385	22.497	19.997

12	0.7921	9.632	8.565	9.000	10.162	15.685
12	0.7931	18.094	15.729	19.553	9.855	16.992
12	0.7941	13.860	27.608	16.721	17.301	24.511
12	0.7951	17.420	23.148	17.767	24.919	22.462
12	0.7961	14.058	30.443	20.655	23.385	30.119
12	0.7971	17.048	23.865	18.449	15.341	17.754
12	0.7981	17.157	17.559	16.171	6.736	19.723
12	0.7991	8.659	16.935	11.670	15.054	13.276
12	0.8001	8.372	11.824	6.724	21.797	16.837
12	0.8011	13.644	23.508	13.881	23.813	21.899
12	0.8021	10.316	15.615	11.466	19.228	22.481
12	0.8031	8.455	15.521	7.469	17.793	18.701
12	0.8041	10.441	17.165	9.947	24.290	14.418
12	0.8051	15.332	22.191	16.517	26.327	23.473
12	0.8061	11.754	20.248	14.564	22.395	14.572
12	0.8071	8.088	26.352	22.022	24.096	20.360
12	0.8081	16.061	12.407	13.333	14.136	17.959
12	0.8091	4.804	5.858	5.609	12.774	5.241
12	0.8101	11.266	22.862	10.197	24.683	15.848
12	0.8111	10.724	21.482	8.812	23.471	14.614
12	0.8121	16.378	11.445	21.486	16.942	8.726
12	0.8131	9.191	15.025	16.259	16.077	15.034
12	0.8141	11.905	17.839	11.216	24.481	12.045
12	0.8151	13.075	13.090	14.266	23.290	12.346
12	0.8161	13.374	8.595	16.858	20.425	11.982
12	0.8171	12.086	7.564	11.510	14.422	19.003
12	0.8181	11.743	20.131	11.067	14.121	10.412
12	0.8191	3.018	4.785	4.776	20.692	5.807
12	0.8201	8.341	7.964	9.292	16.045	7.465
12	0.8211	6.709	6.371	6.022	16.374	4.641
12	0.8221	11.419	21.163	13.202	27.783	7.263
12	0.8231	12.943	14.005	16.145	15.785	8.824
12	0.8241	2.514	5.755	8.253	11.163	3.936
12	0.8251	0.000	0.000	0.000	0.000	0.000
13	0.0014	0.000	0.000	0.000	0.000	0.000
13	0.0024	9.824	8.985	8.546	7.372	10.548
13	0.0034	12.348	7.686	6.001	19.472	10.842
13	0.0044	18.218	22.031	12.058	19.209	13.737
13	0.0054	14.476	12.169	9.047	11.115	11.601
13	0.0064	16.833	16.446	17.432	17.826	16.012
13	0.0074	15.500	19.416	13.108	18.347	16.593
13	0.0084	18.100	20.371	17.570	15.667	11.535
13	0.0094	18.472	19.592	15.052	18.849	10.432
13	0.0104	18.053	21.693	15.005	13.507	19.392
13	0.0114	22.011	30.156	24.710	22.406	17.072
13	0.0124	16.704	28.253	19.592	25.413	12.056
13	0.0134	16.725	20.087	15.762	19.664	10.130
13	0.0144	16.218	16.814	11.570	21.672	8.643
13	0.0154	12.435	17.110	9.404	18.806	16.744
13	0.0164	11.180	15.167	10.307	9.273	3.089

13	0.0174	7.883	12.347	9.502	8.853	10.263
13	0.0184	12.301	19.096	11.797	18.330	11.003
13	0.0194	10.821	13.791	9.058	16.590	11.504
13	0.0204	11.654	11.592	6.092	14.817	8.347
13	0.0214	11.551	11.460	6.080	14.765	8.198
13	0.0224	12.105	11.961	6.009	17.322	9.922
13	0.0234	13.710	11.891	6.627	11.994	24.587
13	0.0244	15.887	16.223	13.612	12.334	10.144
13	0.0254	22.495	30.521	18.015	22.287	6.035
13	0.0264	15.952	17.947	18.753	14.657	14.818
13	0.0274	9.904	13.249	7.346	17.741	13.399
13	0.0284	13.612	9.786	11.425	11.296	8.922
13	0.0294	11.186	14.059	10.977	4.825	10.092
13	0.0304	13.526	20.306	12.226	8.817	7.408
13	0.0314	15.727	20.710	12.822	14.642	10.751
13	0.0324	15.826	22.224	11.968	15.417	18.314
13	0.0334	19.840	22.428	14.566	16.423	15.630
13	0.0344	14.790	11.886	9.777	9.281	12.304
13	0.0354	11.804	14.832	10.826	13.375	16.295
13	0.0364	16.610	13.930	14.325	26.342	29.994
13	0.0374	24.362	24.026	22.407	14.135	14.623
13	0.0384	19.355	22.215	18.506	12.979	20.514
13	0.0394	16.414	13.233	10.242	8.838	8.360
13	0.0404	9.990	8.672	12.130	10.077	11.462
13	0.0414	14.204	11.670	14.329	12.031	7.195
13	0.0424	22.095	21.122	20.975	18.382	14.485
13	0.0434	9.069	9.821	8.516	12.715	17.795
13	0.0444	10.633	11.399	8.686	11.100	9.562
13	0.0454	10.169	10.640	8.249	11.619	9.794
13	0.0464	18.957	22.565	17.409	11.642	10.411
13	0.0474	12.011	13.002	8.528	13.746	14.814
13	0.0484	12.185	16.821	16.072	7.448	8.647
13	0.0494	14.745	16.704	15.173	10.652	16.865
13	0.0504	4.306	7.655	6.454	14.099	16.255
13	0.0514	3.580	5.700	10.945	17.194	16.589
13	0.0524	5.353	4.891	9.807	9.479	13.690
13	0.0534	17.180	15.480	11.817	12.980	20.763
13	0.0544	16.748	12.452	11.461	16.363	24.571
13	0.0554	9.452	10.792	13.072	6.352	12.880
13	0.0564	17.737	25.184	19.323	16.406	14.078
13	0.0574	14.143	10.942	14.948	15.925	10.242
13	0.0584	12.782	5.895	13.650	11.804	15.612
13	0.0594	14.566	11.561	13.132	6.370	17.119
13	0.0604	14.589	11.566	13.099	6.333	17.160
13	0.0614	14.016	23.575	23.896	20.832	20.243
13	0.0624	30.623	13.347	23.304	10.160	20.746
13	0.0634	20.877	17.058	22.616	13.165	11.561
13	0.0644	12.888	9.811	14.560	10.206	8.397
13	0.0654	4.111	10.017	7.789	10.893	10.492
13	0.0664	7.832	5.649	8.975	12.305	10.495

13	0.0674	13.284	18.655	16.350	15.091	9.774
13	0.0684	15.440	14.480	15.079	11.800	6.206
13	0.0694	10.625	10.086	7.900	11.406	16.204
13	0.0704	8.059	9.019	14.007	8.265	12.292
13	0.0714	10.292	8.584	10.950	8.075	2.885
13	0.0724	10.280	17.329	14.555	8.323	6.975
13	0.0734	12.138	18.276	15.355	6.854	4.811
13	0.0744	23.148	26.325	31.395	14.381	10.591
13	0.0754	23.089	26.233	31.339	14.325	10.535
13	0.0764	10.433	15.158	12.944	13.676	10.245
13	0.0774	8.920	15.149	11.244	12.237	12.400
13	0.0784	14.556	17.822	21.859	10.711	10.909
13	0.0794	8.366	9.875	10.080	10.388	9.236
13	0.0804	11.219	6.785	10.454	7.298	4.149
13	0.0814	9.647	13.123	9.989	15.321	8.740
13	0.0824	10.302	11.518	9.185	10.424	11.354
13	0.0834	9.715	14.639	11.432	11.680	8.322
13	0.0844	15.111	8.815	16.885	15.642	16.694
13	0.0854	7.739	9.608	12.552	12.541	9.130
13	0.0864	11.133	7.119	11.538	11.417	9.686
13	0.0874	16.318	25.911	21.715	16.698	9.066
13	0.0884	7.787	5.343	7.889	10.789	7.371
13	0.0894	15.850	10.956	15.474	8.876	6.534
13	0.0904	12.641	9.154	13.904	20.020	15.053
13	0.0914	7.443	7.944	11.174	18.289	12.197
13	0.0924	9.883	9.626	8.817	15.997	13.257
13	0.0934	12.261	23.229	14.301	13.990	11.612
13	0.0944	8.638	20.376	11.308	19.401	10.245
13	0.0954	7.476	7.762	7.086	12.137	4.671
13	0.0964	7.552	7.066	9.496	11.159	14.725
13	0.0974	11.412	12.469	11.693	13.611	12.483
13	0.0984	19.928	11.711	16.651	19.777	14.821
13	0.0994	19.904	12.265	20.831	14.808	8.136
13	0.1004	12.514	4.373	11.591	8.931	6.746
13	0.1014	15.261	5.778	10.198	12.487	14.681
13	0.1024	13.897	10.171	15.612	7.162	6.259
13	0.1034	15.130	13.472	14.854	9.513	11.211
13	0.1044	12.819	14.585	14.937	12.921	7.038
13	0.1054	14.034	13.496	12.874	5.896	4.287
13	0.1064	8.194	3.815	6.373	7.659	5.898
13	0.1074	12.212	14.969	13.414	6.331	5.112
13	0.1084	18.091	19.410	18.523	18.804	11.473
13	0.1094	14.302	21.111	19.131	15.780	11.496
13	0.1104	12.738	8.882	9.776	10.011	11.393
13	0.1114	12.324	9.174	7.197	21.193	19.127
13	0.1124	14.377	13.850	13.351	9.634	9.116
13	0.1134	15.958	19.845	12.663	9.755	17.354
13	0.1144	27.704	24.802	35.532	14.173	12.158
13	0.1154	25.552	20.586	27.435	13.525	10.967
13	0.1164	11.793	13.723	9.639	17.159	3.643

13	0.1174	20.220	20.039	27.332	7.922	12.210
13	0.1184	17.601	26.394	22.923	16.110	8.738
13	0.1194	15.708	11.555	16.990	14.952	6.090
13	0.1204	20.465	21.197	23.956	7.588	7.963
13	0.1214	18.696	21.778	21.699	7.440	17.026
13	0.1224	28.563	26.486	24.016	7.440	15.217
13	0.1234	18.042	17.576	11.834	8.612	13.731
13	0.1244	17.389	15.617	11.304	5.539	21.232
13	0.1254	19.932	20.622	22.332	10.796	18.987
13	0.1264	23.504	19.297	32.592	9.190	18.005
13	0.1274	8.183	2.673	8.356	5.447	6.172
13	0.1284	5.265	7.321	8.078	5.344	17.998
13	0.1294	9.814	4.827	7.001	2.470	11.541
13	0.1304	31.695	20.875	25.703	4.609	15.766
13	0.1314	36.526	26.932	39.574	8.061	9.685
13	0.1324	25.378	28.145	35.238	8.116	9.298
13	0.1334	15.110	14.571	15.172	9.263	14.225
13	0.1344	16.988	12.825	12.764	8.098	9.951
13	0.1354	4.724	4.975	1.782	8.821	7.473
13	0.1364	8.161	6.697	7.023	16.778	10.824
13	0.1374	11.587	6.101	15.149	12.061	11.357
13	0.1384	8.647	4.950	9.857	17.715	11.678
13	0.1394	8.714	4.967	9.861	17.737	11.743
13	0.1404	9.324	10.275	9.259	18.393	12.955
13	0.1414	9.389	8.240	7.907	9.117	11.007
13	0.1424	9.432	8.224	7.873	9.082	11.141
13	0.1434	8.373	9.432	10.893	14.829	16.018
13	0.1444	8.423	9.470	10.951	14.918	16.022
13	0.1454	7.719	8.632	5.864	21.888	11.508
13	0.1464	7.742	8.631	5.895	21.952	11.488
13	0.1474	11.885	7.354	8.993	13.986	13.998
13	0.1484	18.863	14.129	19.221	8.085	16.231
13	0.1494	17.213	21.454	25.697	16.555	17.494
13	0.1504	11.910	14.594	8.887	19.272	10.502
13	0.1514	15.979	9.374	11.036	10.985	16.378
13	0.1524	13.183	17.582	15.157	11.370	9.022
13	0.1534	16.665	19.408	18.147	10.151	7.059
13	0.1544	27.924	28.035	29.189	12.655	8.691
13	0.1554	10.889	12.432	10.413	9.591	5.439
13	0.1564	4.410	5.659	4.984	3.436	5.068
13	0.1574	7.664	7.158	6.749	5.671	6.068
13	0.1584	16.866	18.934	17.004	11.084	15.143
13	0.1594	15.267	8.685	12.618	11.323	11.252
13	0.1604	12.919	15.266	13.827	18.699	9.596
13	0.1614	24.971	22.810	23.750	11.733	24.168
13	0.1624	8.916	10.983	7.506	6.780	9.340
13	0.1634	13.644	19.007	14.188	8.606	5.188
13	0.1644	13.574	18.943	14.090	8.643	5.183
13	0.1654	13.521	18.893	14.009	8.681	5.178
13	0.1664	17.121	9.395	15.283	14.418	15.900

13	0.1674	21.553	13.504	14.377	14.492	35.459
13	0.1684	20.362	5.464	10.001	13.187	26.853
13	0.1694	7.860	8.954	7.680	6.475	18.675
13	0.1704	14.208	9.831	11.008	20.661	14.295
13	0.1714	15.003	9.533	13.870	9.937	13.276
13	0.1724	16.097	12.719	15.181	20.553	10.991
13	0.1734	18.736	13.293	17.779	10.292	21.861
13	0.1744	19.344	16.941	14.183	11.320	27.166
13	0.1754	9.051	15.060	12.108	20.580	7.262
13	0.1764	16.343	13.560	18.567	14.820	13.955
13	0.1774	9.942	10.618	7.220	14.841	17.891
13	0.1784	13.638	9.605	8.602	13.291	27.097
13	0.1794	9.582	12.468	8.284	32.734	20.557
13	0.1804	16.398	16.190	16.981	14.445	20.354
13	0.1814	12.689	10.988	14.603	19.209	21.508
13	0.1824	15.942	18.731	16.418	27.117	21.891
13	0.1834	14.897	9.182	13.170	11.616	15.050
13	0.1844	8.952	15.294	9.663	14.115	5.822
13	0.1854	25.555	17.443	25.691	11.846	10.807
13	0.1864	20.865	17.046	16.363	15.241	18.476
13	0.1874	19.611	17.019	16.369	19.104	18.438
13	0.1884	23.687	17.514	17.337	31.711	16.481
13	0.1894	14.035	12.049	13.982	22.200	20.056
13	0.1904	12.883	8.812	12.551	18.475	16.247
13	0.1914	12.475	19.739	11.832	31.462	18.403
13	0.1924	10.221	20.460	13.999	32.004	14.864
13	0.1934	15.854	11.525	16.615	40.060	26.461
13	0.1944	15.541	9.587	18.494	23.822	30.675
13	0.1954	6.100	10.687	10.398	19.018	29.463
13	0.1964	17.140	13.859	19.370	14.708	19.574
13	0.1974	14.446	16.015	21.558	20.001	20.112
13	0.1984	17.626	22.485	19.955	29.063	31.963
13	0.1994	9.824	21.281	11.795	31.881	23.394
13	0.2004	11.299	8.236	15.854	21.313	17.651
13	0.2014	8.296	11.889	11.388	18.401	26.097
13	0.2024	14.896	18.962	19.034	28.825	14.254
13	0.2034	10.818	16.011	11.263	23.626	12.200
13	0.2044	11.747	13.633	11.818	11.374	14.837
13	0.2054	2.233	6.297	2.640	22.934	14.649
13	0.2064	2.787	6.900	4.277	26.434	22.194
13	0.2074	10.583	6.055	4.788	26.568	28.708
13	0.2084	10.447	11.162	10.948	18.205	12.058
13	0.2094	7.937	13.976	10.401	17.394	5.721
13	0.2104	15.049	15.474	13.522	23.453	13.773
13	0.2114	20.007	18.089	16.022	25.689	18.451
13	0.2124	21.949	20.144	26.628	12.709	14.961
13	0.2134	25.517	19.685	26.432	15.374	15.769
13	0.2144	15.512	18.902	19.805	22.685	22.679
13	0.2154	8.947	6.879	6.625	17.134	16.372
13	0.2164	5.719	4.922	5.082	11.672	12.909

13	0.2174	7.080	10.568	9.554	3.018	9.491
13	0.2184	11.620	12.929	11.167	9.329	12.463
13	0.2194	22.577	20.745	17.995	15.882	14.314
13	0.2204	21.042	20.516	18.100	32.673	25.265
13	0.2214	21.107	20.510	18.119	32.544	25.134
13	0.2224	12.779	11.630	9.964	25.157	11.982
13	0.2234	17.767	15.202	20.064	26.279	16.822
13	0.2244	19.005	12.812	19.644	16.947	10.877
13	0.2254	12.728	14.462	16.781	23.596	19.507
13	0.2264	13.513	16.730	18.477	14.580	14.753
13	0.2274	13.554	14.304	16.833	10.660	9.756
13	0.2284	14.870	18.119	21.720	11.187	13.588
13	0.2294	8.705	11.218	14.084	14.925	13.324
13	0.2304	21.352	16.533	22.343	16.628	14.469
13	0.2314	27.025	16.793	22.608	16.453	26.685
13	0.2324	14.977	14.129	13.552	26.208	22.561
13	0.2334	10.030	16.644	19.102	16.247	16.709
13	0.2344	17.613	10.167	15.726	3.946	8.331
13	0.2354	13.622	10.995	11.558	16.644	24.234
13	0.2364	12.843	10.088	10.123	18.352	16.608
13	0.2374	25.469	21.027	23.899	24.310	14.364
13	0.2384	11.663	8.165	11.397	17.720	20.458
13	0.2394	14.646	13.514	13.522	17.722	10.547
13	0.2404	14.680	15.898	14.659	24.131	11.088
13	0.2414	15.836	11.731	15.792	25.402	19.096
13	0.2424	14.118	8.499	14.564	25.451	18.707
13	0.2434	9.289	3.902	11.567	10.771	10.869
13	0.2444	13.289	7.269	12.739	8.820	12.745
13	0.2454	14.143	12.684	11.310	14.138	7.254
13	0.2464	10.837	9.929	11.130	10.268	8.666
13	0.2474	11.878	10.933	13.228	11.259	8.359
13	0.2484	8.303	9.157	3.892	20.505	7.279
13	0.2494	15.122	15.861	12.222	30.461	24.002
13	0.2504	12.111	12.622	12.485	19.970	11.625
13	0.2514	2.296	2.843	4.080	15.587	8.537
13	0.2524	4.917	6.554	6.575	19.368	14.284
13	0.2534	10.166	8.506	10.485	15.231	17.884
13	0.2544	8.826	10.274	7.140	16.429	14.567
13	0.2554	5.782	7.839	7.398	24.919	8.922
13	0.2564	4.611	6.439	6.591	10.338	8.928
13	0.2574	12.399	11.544	12.399	22.085	15.954
13	0.2584	19.594	16.692	21.107	21.450	13.316
13	0.2594	19.569	14.354	13.380	19.280	16.660
13	0.2604	11.521	6.602	12.188	23.126	15.908
13	0.2614	8.974	8.493	9.223	18.393	23.911
13	0.2624	12.271	3.884	10.449	12.392	13.409
13	0.2634	16.041	9.948	14.483	11.918	12.943
13	0.2644	7.460	4.117	7.389	13.068	9.432
13	0.2654	13.368	10.470	12.187	22.326	20.506
13	0.2664	11.108	12.589	11.714	26.330	10.048

13	0.2674	9.025	8.302	9.790	22.520	11.623
13	0.2684	4.841	12.824	6.708	21.179	11.239
13	0.2694	5.098	11.831	5.044	23.299	15.764
13	0.2704	13.122	15.434	12.635	24.057	17.499
13	0.2714	9.471	15.318	11.923	28.994	21.360
13	0.2724	13.132	11.293	10.114	32.389	13.490
13	0.2734	8.593	9.190	5.910	25.072	13.712
13	0.2744	6.886	7.501	6.056	21.165	15.088
13	0.2754	7.760	5.886	7.183	11.024	16.347
13	0.2764	9.543	11.364	9.832	16.112	5.393
13	0.2774	11.574	13.688	14.595	12.996	11.676
13	0.2784	5.013	8.039	5.399	14.901	11.864
13	0.2794	6.142	8.722	6.051	16.373	10.543
13	0.2804	11.445	11.423	13.286	18.229	24.027
13	0.2814	1.691	6.030	4.205	14.006	9.440
13	0.2824	1.255	8.454	4.542	13.243	5.216
13	0.2834	11.061	21.734	16.172	20.356	6.737
13	0.2844	13.619	26.648	16.326	23.503	10.085
13	0.2854	10.112	14.393	9.749	18.730	6.566
13	0.2864	17.823	18.205	25.424	26.122	7.224
13	0.2874	18.342	14.233	23.092	17.308	9.576
13	0.2884	7.320	9.083	9.562	19.682	4.670
13	0.2894	7.717	11.747	11.928	29.959	3.553
13	0.2904	4.688	10.939	4.737	19.771	6.946
13	0.2914	6.819	4.730	8.159	20.131	8.336
13	0.2924	8.307	5.197	9.062	18.474	7.159
13	0.2934	7.758	6.001	7.110	14.954	11.594
13	0.2944	7.436	3.337	5.014	8.464	9.754
13	0.2954	9.277	9.835	11.339	13.109	10.833
13	0.2964	9.770	15.952	15.743	18.881	7.868
13	0.2974	19.039	16.620	22.491	11.856	12.345
13	0.2984	17.340	19.818	13.848	25.138	13.940
13	0.2994	11.774	13.654	11.951	24.580	11.763
13	0.3004	9.249	14.272	10.957	25.374	11.011
13	0.3014	10.515	18.087	13.745	33.208	12.038
13	0.3024	7.227	13.333	7.865	27.626	15.630
13	0.3034	5.345	7.223	4.227	18.708	5.266
13	0.3044	7.258	11.129	8.782	12.329	7.452
13	0.3054	2.979	6.693	3.266	12.861	2.871
13	0.3064	3.503	3.120	3.415	6.817	2.491
13	0.3074	1.845	1.282	3.580	1.957	2.230
13	0.3084	9.951	10.251	6.235	21.631	13.232
13	0.3094	8.015	7.247	5.928	12.457	2.205
13	0.3104	10.811	3.466	6.503	17.162	16.530
13	0.3114	6.958	14.607	10.565	37.626	18.325
13	0.3124	12.153	14.633	13.456	37.151	17.547
13	0.3134	9.863	9.521	10.616	20.272	9.989
13	0.3144	6.535	7.486	6.799	17.291	11.186
13	0.3154	10.304	8.966	8.545	30.715	11.114
13	0.3164	8.702	8.327	10.308	18.537	6.148

13	0.3174	6.965	6.229	10.102	16.497	10.490
13	0.3184	17.834	16.435	19.475	12.908	13.028
13	0.3194	12.780	12.846	10.964	17.611	9.331
13	0.3204	21.693	13.653	19.708	12.870	14.820
13	0.3214	23.705	20.592	24.115	23.643	14.188
13	0.3224	18.462	17.763	12.397	27.710	11.045
13	0.3234	14.122	11.569	11.600	24.666	9.348
13	0.3244	20.159	13.118	18.063	20.180	15.432
13	0.3254	15.400	11.813	9.077	7.582	6.550
13	0.3264	9.824	7.094	9.154	9.093	4.474
13	0.3274	11.541	16.212	15.268	8.458	9.855
13	0.3284	18.185	20.761	21.962	14.332	8.928
13	0.3294	18.190	20.793	22.051	14.345	8.938
13	0.3304	17.062	16.988	15.828	21.968	16.480
13	0.3314	15.453	14.775	16.895	25.248	9.079
13	0.3324	13.182	11.538	14.368	14.436	11.028
13	0.3334	14.945	14.047	17.493	11.447	6.456
13	0.3344	16.849	12.356	23.927	13.746	11.269
13	0.3354	14.007	12.756	14.443	12.905	14.384
13	0.3364	7.424	7.080	12.556	13.374	13.052
13	0.3374	10.212	13.478	12.365	22.815	11.463
13	0.3384	12.636	10.991	13.547	19.488	12.575
13	0.3394	19.606	19.476	20.241	12.333	10.782
13	0.3404	17.091	14.600	29.108	17.447	11.974
13	0.3414	17.104	14.608	29.118	17.462	11.972
13	0.3424	14.310	7.477	17.480	16.045	11.224
13	0.3434	8.936	13.493	12.845	19.900	9.802
13	0.3444	8.885	13.634	12.667	20.124	9.701
13	0.3454	11.546	12.929	12.225	17.817	12.725
13	0.3464	8.817	6.185	11.270	14.617	9.666
13	0.3474	13.408	7.588	15.370	21.406	9.765
13	0.3484	31.481	13.274	27.477	21.125	11.712
13	0.3494	22.144	17.546	26.102	11.653	10.421
13	0.3504	16.818	13.073	26.033	12.799	12.631
13	0.3514	16.664	12.915	25.858	12.758	12.697
13	0.3524	12.246	17.598	11.027	11.562	16.597
13	0.3534	12.709	7.610	13.399	14.988	27.108
13	0.3544	12.713	7.614	13.401	14.968	27.083
13	0.3554	16.999	9.312	22.196	31.140	9.691
13	0.3564	21.770	10.029	22.174	21.236	19.013
13	0.3574	11.289	8.168	10.950	9.530	11.646
13	0.3584	8.758	8.256	10.952	19.957	13.856
13	0.3594	8.740	8.428	11.049	20.098	13.859
13	0.3604	9.271	5.766	6.026	16.803	6.821
13	0.3614	13.417	9.378	10.691	15.268	22.271
13	0.3624	13.072	8.371	9.569	19.243	32.762
13	0.3634	17.300	13.731	18.913	33.617	20.513
13	0.3644	19.048	11.936	16.150	22.328	16.826
13	0.3654	20.066	10.202	20.474	31.928	15.704
13	0.3664	20.992	11.541	18.272	20.077	10.362

13	0.3674	13.973	4.287	13.094	19.154	11.090
13	0.3684	13.810	8.382	12.006	28.233	20.008
13	0.3694	23.306	8.853	19.988	31.372	42.935
13	0.3704	12.402	7.833	14.565	22.459	32.925
13	0.3714	10.561	8.464	16.344	17.361	10.327
13	0.3724	13.207	8.457	17.974	22.753	15.273
13	0.3734	10.527	11.793	14.482	14.627	24.911
13	0.3744	21.630	18.575	21.877	14.043	6.363
13	0.3754	22.570	9.883	15.369	26.331	31.798
13	0.3764	16.890	17.462	13.660	23.854	14.644
13	0.3774	15.030	10.551	15.774	15.774	22.145
13	0.3784	21.097	10.051	16.371	34.527	31.303
13	0.3794	24.416	13.100	23.444	16.382	15.586
13	0.3804	22.851	18.430	25.304	19.038	14.658
13	0.3814	12.915	11.604	18.007	16.680	12.710
13	0.3824	17.426	12.009	18.652	22.612	17.538
13	0.3834	17.216	15.936	19.452	19.558	12.638
13	0.3844	13.769	13.625	15.940	20.270	16.248
13	0.3854	13.444	12.368	16.280	25.173	16.482
13	0.3864	15.561	6.361	13.968	14.995	14.513
13	0.3874	7.508	10.282	11.558	18.332	18.585
13	0.3884	10.577	11.056	18.015	19.547	20.761
13	0.3894	23.362	10.943	27.981	18.431	8.677
13	0.3904	18.914	14.557	15.811	9.477	10.884
13	0.3914	6.515	3.355	6.141	6.063	4.902
13	0.3924	24.142	14.577	20.459	22.293	4.407
13	0.3934	23.392	14.733	11.408	34.579	14.247
13	0.3944	7.488	8.068	6.066	28.922	16.948
13	0.3954	11.361	6.732	8.400	27.487	29.169
13	0.3964	11.319	8.662	11.252	16.252	19.072
13	0.3974	7.932	6.138	5.224	19.741	18.165
13	0.3984	16.496	10.161	14.859	14.937	22.867
13	0.3994	17.391	7.833	12.148	22.779	30.678
13	0.4004	15.402	6.944	15.220	15.009	24.345
13	0.4014	14.091	9.900	13.842	26.295	22.818
13	0.4024	13.519	8.899	16.320	8.559	23.807
13	0.4034	16.129	8.204	9.853	26.763	17.240
13	0.4044	12.164	7.157	6.569	19.908	15.219
13	0.4054	2.609	7.429	3.905	17.927	12.461
13	0.4064	3.096	10.560	3.027	21.704	7.701
13	0.4074	12.394	9.852	8.552	5.096	16.900
13	0.4084	10.503	6.306	6.662	24.772	21.872
13	0.4094	11.191	6.222	12.141	24.046	20.950
13	0.4104	17.222	3.443	12.203	18.544	23.497
13	0.4114	6.256	1.369	5.148	7.470	11.964
13	0.4124	10.847	7.339	9.783	22.602	16.503
13	0.4134	9.892	11.549	10.301	13.707	15.090
13	0.4144	2.677	6.617	5.624	8.437	11.588
13	0.4154	14.419	8.144	10.607	24.109	17.018
13	0.4164	28.584	18.821	22.906	29.814	18.139

13	0.4174	12.030	9.216	12.842	11.325	14.504
13	0.4184	16.498	13.312	17.214	14.159	15.508
13	0.4194	9.188	10.746	9.259	7.331	6.856
13	0.4204	7.750	8.111	7.094	22.964	20.082
13	0.4214	21.996	6.628	13.461	15.759	19.960
13	0.4224	8.656	12.013	9.412	12.758	23.465
13	0.4234	23.783	16.725	16.386	25.302	34.277
13	0.4244	10.052	7.244	8.784	17.712	23.092
13	0.4254	7.509	4.347	7.975	16.585	21.014
13	0.4264	11.985	10.089	12.109	9.904	25.477
13	0.4274	4.318	1.678	4.782	6.640	19.520
13	0.4284	6.666	6.239	7.870	6.892	15.607
13	0.4294	15.028	12.582	10.071	18.845	24.519
13	0.4304	30.577	13.714	16.459	15.558	17.684
13	0.4314	23.923	11.733	13.716	13.904	17.816
13	0.4324	8.243	7.079	8.255	7.127	16.603
13	0.4334	12.820	9.308	13.746	13.411	19.829
13	0.4344	19.992	12.757	14.088	10.265	21.350
13	0.4354	10.495	9.485	8.411	19.422	29.144
13	0.4364	14.411	6.612	9.228	28.850	22.618
13	0.4374	23.419	8.805	12.240	22.752	30.044
13	0.4384	23.463	16.660	19.989	15.222	15.314
13	0.4394	19.269	20.112	20.083	11.367	6.361
13	0.4404	19.937	13.584	14.185	11.801	6.430
13	0.4414	12.206	13.985	11.393	13.854	1.957
13	0.4424	16.063	7.932	10.070	6.817	11.701
13	0.4434	13.455	5.124	10.584	10.921	14.024
13	0.4444	18.166	9.045	15.454	9.435	8.333
13	0.4454	11.847	9.493	13.817	6.888	6.427
13	0.4464	7.119	2.001	5.067	4.843	3.708
13	0.4474	3.734	2.807	0.838	11.068	5.568
13	0.4484	9.847	6.486	3.804	7.673	5.889
13	0.4494	7.834	4.468	3.313	4.514	5.669
13	0.4504	3.774	4.820	3.311	9.552	11.214
13	0.4514	11.176	5.702	7.926	15.061	19.781
13	0.4524	3.158	3.356	3.005	1.019	1.530
13	0.4534	13.298	10.094	9.991	10.088	4.574
13	0.4544	10.412	10.905	8.441	10.469	4.473
13	0.4554	5.798	3.328	5.578	9.193	7.021
13	0.4564	11.838	7.418	10.142	10.692	14.020
13	0.4574	6.556	8.337	11.177	4.511	9.904
13	0.4584	7.315	4.580	6.885	6.681	5.104
13	0.4594	12.085	10.535	15.035	11.358	12.073
13	0.4604	5.058	8.341	6.233	6.103	4.839
13	0.4614	8.749	9.738	8.206	12.418	15.347
13	0.4624	8.277	11.103	8.458	12.363	11.151
13	0.4634	13.298	11.874	9.569	19.659	16.292
13	0.4644	11.818	7.312	9.323	11.328	8.461
13	0.4654	9.030	10.289	11.512	2.562	7.593
13	0.4664	21.645	20.391	20.628	11.748	9.857

13	0.4674	13.520	14.211	15.517	11.518	16.435
13	0.4684	19.913	8.694	15.799	16.402	12.517
13	0.4694	12.067	4.730	7.605	12.928	24.022
13	0.4704	12.071	4.746	7.613	12.918	24.028
13	0.4714	7.353	2.811	7.336	5.346	15.820
13	0.4724	7.912	4.808	5.188	9.964	10.990
13	0.4734	9.114	3.579	10.387	10.417	10.701
13	0.4744	2.486	1.399	1.526	1.896	7.703
13	0.4754	7.548	3.237	7.064	8.456	13.031
13	0.4764	13.920	6.149	8.836	13.769	18.644
13	0.4774	4.003	3.774	5.118	7.692	5.272
13	0.4784	6.010	10.133	5.360	18.075	6.652
13	0.4794	9.362	12.121	6.763	20.590	13.138
13	0.4804	6.832	5.054	6.710	12.538	13.980
13	0.4814	4.287	3.977	4.611	12.926	10.388
13	0.4824	7.048	4.024	4.949	10.565	16.937
13	0.4834	7.556	8.644	9.662	13.571	13.430
13	0.4844	9.999	7.675	9.578	12.850	5.766
13	0.4854	12.837	5.328	9.365	6.361	13.028
13	0.4864	13.666	12.854	8.823	17.958	16.428
13	0.4874	6.573	11.323	6.787	15.547	14.319
13	0.4884	14.119	11.872	11.316	19.251	21.565
13	0.4894	6.462	9.468	5.647	10.497	6.142
13	0.4904	5.691	7.344	6.227	9.584	15.494
13	0.4914	8.662	9.943	8.099	10.422	15.987
13	0.4924	11.682	9.451	11.418	15.317	12.171
13	0.4934	6.134	6.845	6.271	11.081	6.684
13	0.4944	4.225	8.239	5.633	6.821	9.067
13	0.4954	7.084	7.628	5.043	5.195	3.912
13	0.4964	15.395	8.109	8.467	13.224	9.867
13	0.4974	13.100	7.575	7.786	8.157	4.323
13	0.4984	6.776	9.257	9.965	7.874	5.789
13	0.4994	5.970	9.299	9.192	5.902	9.365
13	0.5004	9.280	11.725	11.116	9.425	10.019
13	0.5014	4.640	5.144	6.551	3.436	6.062
13	0.5024	4.069	5.010	4.900	5.284	2.962
13	0.5034	7.367	10.209	7.615	6.981	8.847
13	0.5044	2.624	4.377	2.541	5.320	8.124
13	0.5054	2.160	6.957	3.650	6.131	4.680
13	0.5064	1.627	5.795	3.611	11.313	7.509
13	0.5074	6.567	15.190	7.065	15.805	8.865
13	0.5084	11.036	9.712	8.245	13.361	18.189
13	0.5094	11.979	9.784	8.292	9.765	13.249
13	0.5104	17.109	15.192	14.096	6.407	15.702
13	0.5114	11.004	19.391	16.047	6.829	10.222
13	0.5124	12.016	6.930	10.000	14.452	15.751
13	0.5134	8.793	5.724	12.240	8.940	10.455
13	0.5144	3.630	2.184	5.181	1.537	10.236
13	0.5154	9.771	10.588	11.656	6.900	11.663
13	0.5164	6.253	8.098	6.094	12.481	11.029

13	0.5174	8.580	6.865	10.118	10.918	6.489
13	0.5184	6.310	5.696	11.086	4.645	14.429
13	0.5194	26.653	22.287	32.275	5.545	19.949
13	0.5204	26.664	22.315	32.278	5.423	20.011
13	0.5214	15.251	16.403	26.222	9.878	24.323
13	0.5224	15.217	16.379	26.163	9.885	24.301
13	0.5234	10.530	8.158	13.827	10.607	18.310
13	0.5244	10.343	8.827	10.728	12.831	13.981
13	0.5254	6.064	7.117	10.702	10.125	11.215
13	0.5264	8.112	5.609	9.456	7.221	19.149
13	0.5274	12.501	7.981	4.914	8.118	19.333
13	0.5284	9.118	8.097	5.100	5.039	12.106
13	0.5294	10.369	3.686	10.691	11.690	20.638
13	0.5304	6.447	4.279	4.726	23.618	15.431
13	0.5314	11.414	5.128	14.572	12.810	21.983
13	0.5324	9.660	11.938	13.767	11.541	10.725
13	0.5334	9.091	9.414	13.592	6.064	9.368
13	0.5344	10.595	6.714	5.027	15.096	24.381
13	0.5354	11.801	13.017	8.940	8.488	18.208
13	0.5364	16.237	12.332	18.860	11.017	25.594
13	0.5374	16.282	12.360	18.938	10.894	25.492
13	0.5384	13.532	9.544	13.030	4.342	6.796
13	0.5394	16.320	9.393	10.404	7.125	11.763
13	0.5404	23.010	13.320	17.429	15.182	12.587
13	0.5414	8.633	3.308	5.713	5.753	11.568
13	0.5424	10.059	7.100	13.226	11.878	17.725
13	0.5434	9.989	5.009	8.566	10.773	28.824
13	0.5444	10.550	5.181	7.561	11.962	25.482
13	0.5454	4.627	3.948	6.015	8.345	19.238
13	0.5464	6.315	7.767	7.284	16.223	25.272
13	0.5474	7.526	8.070	6.292	28.397	21.326
13	0.5484	14.894	17.897	13.111	23.500	26.983
13	0.5494	9.825	6.852	9.127	17.352	28.002
13	0.5504	4.467	4.749	4.662	7.299	2.954
13	0.5514	1.446	1.812	1.179	10.626	1.239
13	0.5524	1.372	2.090	2.838	8.595	3.048
13	0.5534	7.773	14.094	12.545	7.232	15.140
13	0.5544	13.353	11.471	10.940	18.351	18.997
13	0.5554	8.149	6.907	7.621	15.511	15.676
13	0.5564	8.206	6.932	7.639	15.475	15.582
13	0.5574	12.687	10.087	6.765	20.744	24.520
13	0.5584	9.968	13.262	10.519	19.280	23.133
13	0.5594	21.097	18.672	20.306	13.971	17.182
13	0.5604	21.205	11.190	10.400	17.641	31.894
13	0.5614	10.826	14.209	12.092	10.093	12.471
13	0.5624	8.123	15.131	10.940	8.366	13.139
13	0.5634	8.099	15.296	8.483	17.036	11.309
13	0.5644	6.012	12.785	6.118	23.368	5.877
13	0.5654	12.603	11.226	11.468	14.204	17.612
13	0.5664	25.101	15.933	12.265	26.269	32.049

13	0.5674	12.811	14.567	6.784	4.635	13.909
13	0.5684	10.149	7.181	6.579	13.192	19.338
13	0.5694	15.052	10.853	10.704	8.536	14.275
13	0.5704	16.352	14.475	13.719	11.492	13.755
13	0.5714	4.124	10.128	4.012	22.929	24.550
13	0.5724	7.862	11.428	6.120	21.870	17.621
13	0.5734	5.722	12.569	7.810	11.686	10.255
13	0.5744	4.746	10.676	6.668	11.811	10.644
13	0.5754	6.871	15.088	8.101	13.634	9.885
13	0.5764	3.401	6.414	3.323	6.888	10.360
13	0.5774	6.224	16.805	7.819	10.313	15.586
13	0.5784	12.093	10.059	11.078	5.317	18.677
13	0.5794	4.392	8.090	5.812	13.089	15.630
13	0.5804	11.377	12.884	14.553	21.247	19.660
13	0.5814	13.186	13.224	15.668	19.831	21.857
13	0.5824	12.707	14.251	17.738	3.316	9.754
13	0.5834	7.023	8.052	5.854	5.810	19.345
13	0.5844	11.078	5.805	6.796	14.195	16.854
13	0.5854	7.316	9.652	4.368	21.128	19.011
13	0.5864	11.434	13.060	8.128	19.806	20.444
13	0.5874	17.219	13.405	11.592	23.547	13.863
13	0.5884	17.176	13.428	11.574	23.488	13.850
13	0.5894	22.171	8.487	13.875	30.081	17.662
13	0.5904	19.643	5.760	13.082	28.208	18.473
13	0.5914	18.094	8.162	11.294	10.869	16.225
13	0.5924	3.506	6.602	5.067	12.601	15.170
13	0.5934	7.151	3.931	4.682	13.840	18.171
13	0.5944	5.747	9.591	5.996	13.562	17.667
13	0.5954	7.846	21.612	11.112	24.451	15.277
13	0.5964	8.445	14.016	9.719	18.703	11.279
13	0.5974	1.877	7.000	2.884	17.379	14.000
13	0.5984	4.173	8.672	3.920	13.881	14.482
13	0.5994	4.962	6.129	5.699	13.619	6.561
13	0.6004	2.303	6.880	4.686	7.692	9.759
13	0.6014	3.710	10.313	4.850	14.265	22.241
13	0.6024	5.990	10.030	10.080	10.930	14.197
13	0.6034	13.883	18.937	14.064	8.872	13.602
13	0.6044	10.382	14.193	13.948	7.647	17.525
13	0.6054	10.371	14.193	14.000	7.626	17.783
13	0.6064	9.250	9.870	12.425	6.206	11.809
13	0.6074	8.019	8.500	9.205	14.534	11.821
13	0.6084	8.000	8.525	9.212	14.570	11.823
13	0.6094	7.349	6.897	9.731	8.555	8.804
13	0.6104	1.373	3.062	1.814	6.844	6.597
13	0.6114	3.018	6.175	4.578	10.585	13.537
13	0.6124	2.737	5.216	1.354	11.628	14.214
13	0.6134	5.956	8.350	5.430	11.282	12.170
13	0.6144	5.721	8.910	5.552	17.432	13.756
13	0.6154	4.474	2.547	2.575	1.756	8.709
13	0.6164	4.956	2.971	3.955	2.771	11.422

13	0.6174	4.967	2.979	3.976	2.779	11.393
13	0.6184	4.979	2.986	3.996	2.788	11.364
13	0.6194	3.069	3.515	2.984	4.396	8.490
13	0.6204	10.549	9.926	12.024	19.919	16.038
13	0.6214	7.510	11.933	9.609	13.729	13.925
13	0.6224	5.405	12.624	9.232	22.554	23.976
13	0.6234	3.740	4.455	4.064	13.499	16.268
13	0.6244	4.074	12.549	7.794	9.108	9.464
13	0.6254	12.003	22.698	15.140	30.745	25.673
13	0.6264	8.765	22.340	11.987	25.934	17.747
13	0.6274	8.565	22.229	12.018	26.034	17.810
13	0.6284	1.997	6.628	5.127	16.876	11.065
13	0.6294	2.776	10.488	6.374	7.153	10.842
13	0.6304	5.469	11.775	8.039	12.191	14.692
13	0.6314	8.618	10.710	7.009	15.346	20.213
13	0.6324	13.663	23.727	14.629	31.113	23.477
13	0.6334	15.458	22.902	23.213	18.709	22.219
13	0.6344	8.250	12.199	12.610	16.128	25.627
13	0.6354	9.715	10.855	7.915	9.835	16.399
13	0.6364	16.760	17.623	17.308	10.240	14.733
13	0.6374	16.812	17.664	17.349	10.215	14.736
13	0.6384	11.156	11.498	7.374	9.854	11.187
13	0.6394	10.953	14.564	9.281	14.791	17.177
13	0.6404	10.970	14.607	9.301	14.813	17.175
13	0.6414	8.226	12.323	7.258	11.594	5.460
13	0.6424	13.286	20.560	12.954	16.925	16.270
13	0.6434	13.235	20.523	12.937	16.860	16.269
13	0.6444	11.226	14.833	13.158	11.938	13.597
13	0.6454	10.030	16.119	8.645	8.179	11.665
13	0.6464	10.393	13.470	9.693	5.953	9.647
13	0.6474	14.717	12.606	13.087	11.032	9.210
13	0.6484	16.566	17.669	13.105	9.388	8.835
13	0.6494	14.941	20.218	15.040	8.654	12.123
13	0.6504	16.096	23.927	16.780	9.827	10.689
13	0.6514	17.922	23.237	24.673	9.129	8.221
13	0.6524	13.954	17.624	17.214	8.535	5.175
13	0.6534	13.714	11.888	14.296	6.833	4.369
13	0.6544	13.719	11.901	14.298	6.853	4.385
13	0.6554	8.234	9.152	8.090	5.501	5.708
13	0.6564	7.952	13.848	9.961	10.121	6.162
13	0.6574	7.980	9.311	10.181	6.545	7.433
13	0.6584	8.115	9.822	9.849	9.729	9.833
13	0.6594	11.883	15.702	14.646	16.992	6.472
13	0.6604	20.643	19.599	23.468	11.284	7.688
13	0.6614	20.597	19.481	21.564	9.607	11.323
13	0.6624	16.538	14.710	17.986	19.795	14.322
13	0.6634	22.215	11.663	19.434	13.839	23.888
13	0.6644	13.052	10.404	11.757	16.375	20.251
13	0.6654	10.242	5.471	8.418	17.786	19.237
13	0.6664	13.417	8.670	11.380	14.847	12.977

13	0.6674	11.316	8.266	8.060	19.706	16.226
13	0.6684	11.809	6.142	9.307	13.576	7.581
13	0.6694	14.609	14.161	14.584	27.581	23.889
13	0.6704	11.051	12.650	10.472	14.801	21.319
13	0.6714	11.049	13.555	14.512	19.240	6.721
13	0.6724	14.638	18.535	20.562	18.659	20.392
13	0.6734	9.605	21.949	16.152	25.103	23.488
13	0.6744	9.746	19.164	11.329	27.670	22.302
13	0.6754	13.328	14.918	18.078	13.930	15.787
13	0.6764	10.555	19.082	16.152	22.220	11.103
13	0.6774	10.174	19.217	14.736	18.475	23.029
13	0.6784	5.028	2.161	4.261	5.384	17.679
13	0.6794	7.879	4.373	7.786	10.398	8.311
13	0.6804	4.424	6.938	10.319	16.580	9.249
13	0.6814	8.010	8.861	15.899	13.001	11.061
13	0.6824	17.438	17.833	19.623	14.449	19.335
13	0.6834	15.543	15.860	16.258	9.111	13.094
13	0.6844	6.635	20.305	14.330	20.942	15.287
13	0.6854	8.668	15.487	14.344	14.245	9.585
13	0.6864	12.205	13.804	20.751	13.479	14.876
13	0.6874	16.064	16.695	25.780	17.132	6.071
13	0.6884	16.283	11.405	10.920	23.203	10.781
13	0.6894	15.760	15.986	13.076	21.251	21.285
13	0.6904	12.791	21.874	19.106	23.219	17.134
13	0.6914	11.942	10.610	9.026	11.553	13.792
13	0.6924	4.155	6.322	4.270	14.515	11.034
13	0.6934	11.780	15.349	18.260	17.614	9.811
13	0.6944	15.565	13.652	17.805	23.708	20.172
13	0.6954	9.546	5.510	7.813	22.912	11.416
13	0.6964	8.786	7.539	10.516	20.309	8.178
13	0.6974	12.243	13.626	8.482	20.573	8.897
13	0.6984	18.022	13.204	8.906	12.847	12.512
13	0.6994	14.529	15.777	18.509	8.557	17.229
13	0.7004	14.877	18.423	21.563	21.075	20.073
13	0.7014	11.168	15.845	14.023	22.241	8.487
13	0.7024	15.793	8.554	13.678	29.020	15.154
13	0.7034	22.430	14.001	19.676	10.387	16.343
13	0.7044	24.500	19.909	20.696	18.156	20.747
13	0.7054	25.263	14.320	26.338	19.195	15.202
13	0.7064	29.322	15.664	29.642	28.670	14.064
13	0.7074	19.780	16.193	20.762	10.168	10.745
13	0.7084	17.747	11.006	18.690	10.878	13.101
13	0.7094	4.962	6.933	9.283	18.395	9.971
13	0.7104	4.440	5.621	7.604	12.198	8.010
13	0.7114	9.034	10.398	11.045	16.145	10.288
13	0.7124	12.851	11.558	16.213	18.287	19.566
13	0.7134	8.390	7.042	10.882	15.325	18.707
13	0.7144	10.909	6.904	9.643	7.884	11.923
13	0.7154	12.118	11.486	10.769	8.226	13.693
13	0.7164	7.391	14.235	10.800	15.579	21.444

13	0.7174	7.388	11.282	8.695	17.551	22.868
13	0.7184	16.367	18.497	21.173	18.953	16.813
13	0.7194	10.012	20.591	14.389	14.088	9.275
13	0.7204	15.068	20.853	16.610	8.982	6.650
13	0.7214	6.595	9.138	6.907	5.580	7.545
13	0.7224	10.998	12.020	8.335	7.008	6.017
13	0.7234	8.576	14.232	8.965	15.208	12.239
13	0.7244	10.918	22.429	14.229	13.773	12.672
13	0.7254	11.952	17.415	16.506	8.634	21.797
13	0.7264	12.018	14.714	14.787	11.780	19.145
13	0.7274	19.451	24.293	15.489	11.006	21.404
13	0.7284	12.513	16.413	10.717	12.807	27.884
13	0.7294	7.171	6.098	5.883	9.972	10.354
13	0.7304	8.072	4.369	11.755	9.304	18.629
13	0.7314	10.021	6.658	13.262	14.855	22.380
13	0.7324	15.390	18.008	15.768	17.400	16.745
13	0.7334	9.603	8.603	9.003	8.556	6.534
13	0.7344	3.817	6.664	6.800	7.619	4.992
13	0.7354	19.506	17.356	19.437	9.493	3.757
13	0.7364	10.518	7.942	9.577	9.568	17.028
13	0.7374	17.765	13.866	17.657	8.273	10.783
13	0.7384	8.370	8.560	11.579	14.859	19.709
13	0.7394	8.377	10.578	8.842	20.486	16.535
13	0.7404	12.826	11.451	9.164	19.966	14.395
13	0.7414	8.654	9.257	10.094	5.196	5.859
13	0.7424	8.571	10.296	14.369	28.261	11.876
13	0.7434	26.065	14.105	22.043	18.541	12.607
13	0.7444	11.666	26.859	14.218	12.710	8.093
13	0.7454	11.297	12.526	10.152	13.945	24.221
13	0.7464	11.227	17.347	18.794	9.638	15.793
13	0.7474	14.213	13.066	15.638	17.336	11.386
13	0.7484	18.850	16.128	21.155	23.528	23.117
13	0.7494	16.110	25.779	25.088	18.294	13.832
13	0.7504	11.730	20.153	17.602	20.889	13.292
13	0.7514	10.299	26.798	15.979	14.427	8.409
13	0.7524	6.138	17.963	10.606	15.445	7.374
13	0.7534	8.804	25.806	12.740	35.722	10.530
13	0.7544	7.695	15.245	14.353	18.009	7.804
13	0.7554	16.279	23.921	20.567	13.965	8.423
13	0.7564	17.327	20.271	18.514	32.852	13.034
13	0.7574	22.040	21.558	18.463	15.530	21.316
13	0.7584	21.941	22.900	21.638	27.076	16.051
13	0.7594	18.048	12.543	12.802	20.627	19.056
13	0.7604	22.833	14.741	19.899	13.503	10.249
13	0.7614	31.018	22.913	25.878	11.609	24.763
13	0.7624	16.756	29.117	22.282	15.578	21.812
13	0.7634	12.968	23.924	17.419	8.076	6.144
13	0.7644	11.078	18.383	9.271	11.098	13.656
13	0.7654	23.071	19.240	14.876	12.766	21.362
13	0.7664	5.751	5.102	7.355	9.788	7.552

13	0.7674	17.223	26.618	22.582	17.777	13.741
13	0.7684	29.926	19.517	29.825	19.520	12.067
13	0.7694	22.248	20.648	20.013	12.510	13.556
13	0.7704	16.800	19.106	24.905	17.898	18.732
13	0.7714	14.307	19.811	19.305	17.366	16.280
13	0.7724	22.306	25.388	21.024	6.589	19.713
13	0.7734	7.906	14.120	12.100	18.152	22.920
13	0.7744	14.057	20.912	12.409	23.539	12.624
13	0.7754	23.614	29.467	25.951	21.236	13.208
13	0.7764	10.600	8.179	10.614	12.654	12.815
13	0.7774	17.482	24.427	12.300	27.528	21.253
13	0.7784	9.872	10.872	11.243	18.220	12.259
13	0.7794	20.625	23.857	18.628	12.268	16.896
13	0.7804	39.640	31.209	35.433	15.685	19.416
13	0.7814	22.409	32.411	20.445	15.200	19.249
13	0.7824	31.241	26.137	19.600	10.994	19.796
13	0.7834	28.697	34.750	31.151	11.158	13.848
13	0.7844	37.149	50.104	37.223	18.213	13.938
13	0.7854	19.504	20.351	13.669	13.587	21.448
13	0.7864	24.230	26.376	20.655	17.437	19.710
13	0.7874	19.249	21.218	19.016	10.948	12.509
13	0.7884	13.025	25.613	19.453	28.483	19.523
13	0.7894	17.141	25.386	15.772	17.929	29.730
13	0.7904	8.097	14.251	10.720	20.232	20.418
13	0.7914	11.937	15.703	14.539	13.993	14.341
13	0.7924	8.346	11.167	8.397	16.189	18.403
13	0.7934	9.434	15.813	12.124	10.631	12.722
13	0.7944	11.224	20.758	14.599	16.132	13.258
13	0.7954	15.177	22.158	18.290	15.213	24.093
13	0.7964	11.529	15.467	12.173	10.078	13.124
13	0.7974	7.993	8.809	9.020	13.895	10.136
13	0.7984	15.351	18.083	11.166	15.138	20.979
13	0.7994	16.589	12.743	11.791	13.199	20.544
13	0.8004	9.737	10.030	13.391	15.350	13.746
13	0.8014	17.481	26.346	23.002	10.203	17.001
13	0.8024	12.382	12.989	11.337	3.045	6.750
13	0.8034	8.174	14.785	10.401	8.662	13.673
13	0.8044	8.307	8.475	11.559	8.445	30.913
13	0.8054	11.827	14.579	14.070	6.134	8.550
13	0.8064	0.000	0.000	0.000	0.000	0.000
14	0.0010	0.000	0.000	0.000	0.000	0.000
14	0.0020	0.000	0.000	0.000	0.000	0.000
14	0.0030	15.201	4.514	12.064	23.444	15.772
14	0.0040	20.099	10.276	13.047	37.218	21.423
14	0.0050	16.914	10.363	7.741	15.146	12.731
14	0.0060	22.429	15.841	8.909	23.199	22.886
14	0.0070	18.491	15.651	13.182	26.707	17.774
14	0.0080	34.657	25.179	28.736	17.832	27.083
14	0.0090	12.819	10.379	8.539	8.930	16.871
14	0.0100	24.618	12.746	20.235	42.698	21.683

14	0.0110	16.221	16.051	14.699	31.997	12.568
14	0.0120	20.841	16.735	11.961	24.860	16.709
14	0.0130	21.907	17.946	13.427	21.440	22.125
14	0.0140	17.930	13.755	11.743	29.424	6.388
14	0.0150	19.181	12.282	13.463	31.616	16.314
14	0.0160	12.721	14.109	5.733	27.397	8.326
14	0.0170	31.615	15.610	20.231	21.159	18.501
14	0.0180	16.859	17.919	8.814	31.233	12.848
14	0.0190	32.214	16.063	15.846	44.702	33.457
14	0.0200	26.438	12.371	10.969	35.766	17.069
14	0.0210	22.276	19.758	6.929	37.490	21.701
14	0.0220	18.471	17.561	9.515	31.909	19.286
14	0.0230	26.438	15.081	14.712	26.499	15.200
14	0.0240	25.505	13.093	13.155	30.843	18.704
14	0.0250	18.949	13.661	7.724	39.013	20.740
14	0.0260	21.077	11.652	7.657	24.735	20.269
14	0.0270	13.783	11.243	9.317	20.884	16.281
14	0.0280	19.238	18.161	15.893	33.714	17.595
14	0.0290	20.215	14.300	9.472	19.892	12.394
14	0.0300	22.051	15.386	12.480	34.515	15.240
14	0.0310	14.326	9.548	5.071	14.535	9.434
14	0.0320	20.450	14.211	13.848	14.279	12.922
14	0.0330	32.498	17.618	16.946	26.998	15.634
14	0.0340	15.864	16.144	13.341	25.293	10.633
14	0.0350	12.169	14.269	9.563	24.317	6.660
14	0.0360	11.312	17.238	16.759	15.726	13.528
14	0.0370	13.149	21.031	14.007	35.167	27.261
14	0.0380	5.458	9.202	4.210	26.560	13.037
14	0.0390	12.269	12.462	14.411	29.225	22.349
14	0.0400	17.566	13.762	15.571	31.549	20.248
14	0.0410	5.921	12.171	8.751	29.503	11.641
14	0.0420	15.168	18.960	13.377	35.104	14.239
14	0.0430	11.583	15.629	9.190	19.965	17.890
14	0.0440	10.821	11.536	9.272	23.614	9.278
14	0.0450	15.148	11.855	10.030	28.042	15.180
14	0.0460	10.971	7.277	10.403	12.736	18.102
14	0.0470	9.267	10.200	15.199	24.050	10.952
14	0.0480	8.069	5.917	6.906	15.319	14.221
14	0.0490	21.906	14.095	17.532	12.667	11.839
14	0.0500	13.990	6.081	13.714	35.889	15.393
14	0.0510	8.756	12.412	8.625	38.519	26.643
14	0.0520	22.376	12.123	8.934	55.976	35.246
14	0.0530	11.315	14.014	16.842	42.518	24.443
14	0.0540	13.395	15.554	14.608	24.714	16.097
14	0.0550	27.523	13.328	25.922	38.590	24.296
14	0.0560	15.190	5.498	10.769	26.826	18.574
14	0.0570	19.627	21.689	16.919	21.132	14.528
14	0.0580	23.469	21.171	19.338	41.540	17.052
14	0.0590	13.776	12.983	9.588	53.082	14.916
14	0.0600	21.055	14.865	16.307	49.504	31.770

14	0.0610	39.837	20.203	26.228	46.726	37.893
14	0.0620	34.528	9.615	18.271	45.743	27.704
14	0.0630	16.259	14.599	12.743	26.362	16.804
14	0.0640	3.579	4.657	4.214	6.060	5.880
14	0.0650	15.880	11.111	12.071	35.539	10.978
14	0.0660	15.742	10.489	12.109	23.423	8.320
14	0.0670	13.354	10.910	15.136	23.943	12.323
14	0.0680	13.725	10.233	7.428	38.249	16.815
14	0.0690	7.615	5.328	5.943	10.290	13.298
14	0.0700	10.187	6.729	12.723	16.273	8.922
14	0.0710	7.690	7.314	10.334	7.922	9.228
14	0.0720	10.838	5.689	7.420	23.144	12.340
14	0.0730	23.330	10.170	18.142	41.825	17.348
14	0.0740	31.435	18.991	22.738	40.647	22.501
14	0.0750	26.938	25.653	24.175	57.612	34.977
14	0.0760	20.257	12.846	18.672	33.118	30.440
14	0.0770	18.609	17.206	13.986	44.779	17.237
14	0.0780	21.499	7.023	17.967	43.621	18.644
14	0.0790	12.655	7.426	10.900	14.639	5.896
14	0.0800	14.381	8.878	12.642	21.992	14.297
14	0.0810	15.143	7.562	14.555	38.919	9.974
14	0.0820	21.579	5.532	16.429	46.283	9.619
14	0.0830	26.384	12.590	21.515	47.575	29.559
14	0.0840	23.009	16.561	22.368	47.561	29.117
14	0.0850	7.406	7.464	8.759	38.291	24.278
14	0.0860	8.431	11.748	8.788	27.651	6.790
14	0.0870	16.544	19.897	15.349	67.463	31.112
14	0.0880	7.576	12.598	6.589	32.766	14.862
14	0.0890	19.329	9.894	14.336	9.163	20.011
14	0.0900	10.658	9.873	14.298	47.940	25.211
14	0.0910	17.200	20.789	16.641	55.734	11.676
14	0.0920	17.238	16.560	10.314	53.484	27.251
14	0.0930	16.236	21.028	16.388	26.160	21.235
14	0.0940	11.225	13.126	14.773	18.854	15.173
14	0.0950	10.170	17.550	10.112	39.271	13.183
14	0.0960	10.607	9.233	10.460	41.608	24.546
14	0.0970	13.801	13.749	15.473	26.626	19.533
14	0.0980	21.323	19.176	22.899	41.429	16.463
14	0.0990	9.307	12.449	9.150	50.092	43.037
14	0.1000	17.729	16.483	19.959	80.997	39.101
14	0.1010	14.981	13.729	19.659	68.543	16.398
14	0.1020	19.600	25.002	17.826	83.775	23.620
14	0.1030	12.420	14.990	10.953	25.537	31.765
14	0.1040	9.152	10.779	8.748	25.222	15.689
14	0.1050	13.758	8.758	13.018	37.204	17.160
14	0.1060	5.451	16.513	6.402	84.887	17.496
14	0.1070	7.562	17.471	10.286	104.211	23.440
14	0.1080	7.581	17.815	10.211	105.327	23.477
14	0.1090	7.578	18.058	10.106	105.465	23.507
14	0.1100	15.275	13.848	14.271	73.570	21.435

14	0.1110	18.426	19.967	17.817	74.102	17.081
14	0.1120	20.660	25.703	23.610	79.377	21.655
14	0.1130	11.079	8.649	18.061	32.399	12.593
14	0.1140	16.809	23.539	21.089	27.357	32.153
14	0.1150	19.205	30.761	17.911	112.050	31.061
14	0.1160	11.003	26.447	13.765	78.296	19.282
14	0.1170	16.490	27.922	21.180	60.805	17.535
14	0.1180	34.971	29.626	32.705	62.478	31.059
14	0.1190	17.747	16.872	17.563	61.921	32.852
14	0.1200	24.747	17.807	27.493	83.564	19.937
14	0.1210	22.043	22.101	21.255	100.040	27.042
14	0.1220	31.330	22.632	27.701	79.324	29.608
14	0.1230	31.412	22.813	27.901	79.552	29.602
14	0.1240	26.581	27.703	26.712	72.329	26.285
14	0.1250	9.507	23.054	10.439	38.812	25.321
14	0.1260	13.849	22.424	12.336	48.968	31.837
14	0.1270	13.827	22.437	12.345	49.112	31.795
14	0.1280	9.372	16.672	12.520	36.616	20.196
14	0.1290	10.329	19.499	12.121	31.050	16.837
14	0.1300	13.525	19.095	18.068	47.509	37.791
14	0.1310	17.125	27.838	19.116	48.402	34.718
14	0.1320	14.716	16.180	17.879	19.982	21.122
14	0.1330	12.317	18.790	18.715	35.433	24.205
14	0.1340	21.381	18.989	16.672	67.182	38.504
14	0.1350	0.931	17.587	1.607	51.917	11.042
14	0.1360	23.654	18.410	20.000	69.598	23.199
14	0.1370	21.750	20.646	20.872	35.019	31.516
14	0.1380	42.120	30.530	32.567	74.817	35.073
14	0.1390	27.336	24.813	33.410	43.861	28.513
14	0.1400	16.754	35.180	21.931	64.065	22.582
14	0.1410	15.527	22.102	18.881	49.268	2.771
14	0.1420	14.866	22.402	18.535	77.877	17.080
14	0.1430	16.318	17.800	12.225	62.507	30.289
14	0.1440	13.463	17.020	12.402	56.673	29.506
14	0.1450	41.313	29.085	28.197	86.702	38.675
14	0.1460	34.245	24.855	22.564	55.750	40.930
14	0.1470	38.631	44.720	32.159	38.947	27.184
14	0.1480	16.646	22.647	12.361	44.811	36.760
14	0.1490	24.133	34.055	16.792	63.418	41.888
14	0.1500	24.732	22.462	19.974	73.380	30.068
14	0.1510	14.224	18.637	20.999	52.846	36.790
14	0.1520	15.715	20.944	15.337	40.787	11.096
14	0.1530	24.911	32.427	21.900	71.388	23.015
14	0.1540	7.265	13.962	4.935	40.521	14.361
14	0.1550	13.535	18.537	16.050	65.051	25.274
14	0.1560	16.445	23.153	16.861	77.987	17.896
14	0.1570	19.521	23.486	18.504	30.553	25.264
14	0.1580	6.606	8.677	9.408	12.383	10.599
14	0.1590	20.889	16.053	20.911	29.055	16.755
14	0.1600	15.778	18.271	20.211	25.898	14.466

14	0.1610	14.144	21.790	19.809	23.747	13.910
14	0.1620	16.226	20.607	24.360	60.120	22.582
14	0.1630	12.215	23.944	27.926	50.520	26.527
14	0.1640	12.356	16.837	13.990	47.073	32.634
14	0.1650	25.417	27.286	22.895	34.908	17.218
14	0.1660	19.907	29.451	15.954	35.068	16.824
14	0.1670	19.355	31.269	15.112	45.544	20.409
14	0.1680	20.117	30.785	17.182	51.358	22.095
14	0.1690	25.929	18.650	30.405	16.574	15.388
14	0.1700	19.442	19.629	17.474	53.244	19.978
14	0.1710	25.315	15.741	24.926	18.967	18.539
14	0.1720	22.575	15.210	23.780	37.885	41.492
14	0.1730	22.546	15.191	23.882	37.677	41.231
14	0.1740	23.534	23.458	23.745	36.859	30.325
14	0.1750	16.695	16.656	13.427	42.377	28.879
14	0.1760	21.486	18.561	15.803	38.438	27.753
14	0.1770	18.140	24.088	28.628	25.741	19.297
14	0.1780	18.514	19.940	21.462	9.895	17.104
14	0.1790	9.319	12.251	12.518	30.852	13.149
14	0.1800	14.928	6.973	16.032	33.262	9.797
14	0.1810	21.571	13.079	20.892	31.107	12.674
14	0.1820	15.416	9.712	19.665	23.800	27.481
14	0.1830	7.739	17.113	8.454	47.269	21.906
14	0.1840	20.363	19.250	23.254	28.817	18.033
14	0.1850	15.047	12.465	13.304	36.043	13.568
14	0.1860	20.127	21.599	17.664	25.225	10.370
14	0.1870	12.390	18.536	11.113	27.014	24.137
14	0.1880	11.828	21.310	19.183	22.874	30.310
14	0.1890	14.717	13.091	13.266	22.470	19.246
14	0.1900	14.120	16.406	11.327	37.363	41.469
14	0.1910	18.917	18.734	13.549	42.615	37.101
14	0.1920	19.620	8.497	12.488	15.275	11.153
14	0.1930	19.269	21.590	20.046	46.057	30.144
14	0.1940	19.543	19.862	20.057	42.031	28.469
14	0.1950	9.418	12.630	12.430	22.147	17.437
14	0.1960	20.140	12.717	16.237	19.684	18.155
14	0.1970	9.416	4.726	11.339	19.199	10.931
14	0.1980	24.726	18.882	21.286	17.797	22.315
14	0.1990	16.659	19.927	13.462	39.039	28.274
14	0.2000	9.718	21.892	14.166	44.356	19.383
14	0.2010	8.157	18.404	12.162	45.532	15.688
14	0.2020	32.959	25.329	28.613	25.106	20.872
14	0.2030	13.020	19.962	11.270	29.611	19.085
14	0.2040	16.893	19.951	16.259	31.919	33.965
14	0.2050	17.585	17.362	14.956	34.809	19.242
14	0.2060	5.883	15.914	7.775	31.365	16.559
14	0.2070	19.686	13.716	14.001	16.030	9.655
14	0.2080	19.085	15.775	15.926	24.670	10.487
14	0.2090	6.553	17.516	11.116	33.916	8.871
14	0.2100	8.235	8.677	5.545	22.916	17.603

14	0.2110	14.470	16.239	19.885	22.075	9.885
14	0.2120	14.446	13.291	16.863	20.286	13.330
14	0.2130	9.222	11.355	14.256	29.451	20.295
14	0.2140	5.124	7.241	6.798	21.844	9.319
14	0.2150	17.253	16.499	14.052	35.457	9.333
14	0.2160	15.440	17.635	14.806	23.603	8.424
14	0.2170	8.601	8.914	9.631	19.605	19.301
14	0.2180	7.237	8.459	10.127	16.883	8.819
14	0.2190	10.599	9.154	10.717	22.832	10.234
14	0.2200	6.170	20.426	10.323	28.105	13.119
14	0.2210	8.862	14.968	9.462	18.115	15.796
14	0.2220	11.366	17.014	15.592	14.963	9.831
14	0.2230	9.481	15.567	12.604	16.871	15.052
14	0.2240	11.662	15.466	11.914	14.024	9.502
14	0.2250	9.513	13.886	15.364	21.153	11.002
14	0.2260	10.729	12.121	15.860	16.160	9.584
14	0.2270	8.530	14.890	10.247	28.987	10.732
14	0.2280	9.481	10.517	6.680	22.056	16.180
14	0.2290	11.386	16.772	16.210	24.694	7.840
14	0.2300	24.395	19.002	22.856	14.318	18.741
14	0.2310	10.221	11.744	9.047	18.482	7.886
14	0.2320	11.573	14.442	14.553	20.522	20.772
14	0.2330	13.135	22.568	19.363	23.194	17.107
14	0.2340	13.843	28.417	23.136	27.184	23.166
14	0.2350	13.116	15.963	19.352	17.059	19.016
14	0.2360	6.862	13.460	10.314	22.650	10.257
14	0.2370	9.076	17.661	16.556	29.388	12.554
14	0.2380	16.072	24.573	16.618	33.517	12.154
14	0.2390	17.782	19.327	19.268	30.826	7.912
14	0.2400	11.213	10.375	12.869	9.006	18.880
14	0.2410	11.394	20.054	18.523	22.992	38.637
14	0.2420	14.207	18.899	14.897	26.389	12.965
14	0.2430	14.222	18.935	14.913	26.379	12.976
14	0.2440	16.180	19.224	10.917	17.010	13.770
14	0.2450	16.879	28.904	17.537	25.100	10.291
14	0.2460	23.635	24.323	26.430	24.518	21.449
14	0.2470	16.394	21.400	16.105	27.145	19.021
14	0.2480	11.342	17.680	12.932	33.761	17.013
14	0.2490	18.195	25.464	22.909	19.489	20.761
14	0.2500	11.089	16.398	10.963	27.472	16.337
14	0.2510	21.784	28.015	23.061	22.192	18.039
14	0.2520	22.039	24.905	24.540	11.081	9.387
14	0.2530	15.911	15.599	12.504	15.183	19.733
14	0.2540	12.197	18.001	18.339	13.112	12.472
14	0.2550	9.731	8.657	10.009	9.839	11.302
14	0.2560	12.050	8.606	8.558	17.008	8.214
14	0.2570	21.594	14.175	17.537	18.529	14.765
14	0.2580	21.592	14.212	17.560	18.499	14.715
14	0.2590	15.837	11.821	10.952	23.199	17.630
14	0.2600	8.494	16.506	9.891	21.736	7.504

14	0.2610	7.366	11.092	10.418	12.172	6.291
14	0.2620	16.584	9.097	11.644	12.797	5.634
14	0.2630	16.167	21.592	11.139	27.267	15.490
14	0.2640	21.329	12.843	15.709	17.398	18.153
14	0.2650	12.335	8.232	6.811	12.691	10.878
14	0.2660	7.166	18.188	12.319	14.932	14.955
14	0.2670	29.551	20.026	22.021	22.743	22.054
14	0.2680	12.697	17.328	15.527	7.909	10.558
14	0.2690	24.275	25.645	18.911	32.116	13.865
14	0.2700	23.089	16.210	19.410	20.028	11.968
14	0.2710	16.403	7.117	11.193	14.302	7.617
14	0.2720	13.692	16.327	18.835	19.959	4.607
14	0.2730	15.751	21.501	16.582	21.335	9.166
14	0.2740	22.667	36.271	26.023	18.274	14.754
14	0.2750	19.300	16.144	18.282	19.779	12.923
14	0.2760	21.650	26.964	27.481	25.569	14.822
14	0.2770	22.952	14.907	15.800	17.259	18.052
14	0.2780	19.809	20.341	11.861	24.799	14.994
14	0.2790	12.497	12.142	16.658	14.618	15.337
14	0.2800	18.433	12.506	20.011	9.532	13.910
14	0.2810	7.912	8.332	11.195	13.787	11.847
14	0.2820	12.051	8.344	12.676	7.153	17.588
14	0.2830	13.974	14.001	13.951	15.894	13.316
14	0.2840	12.295	5.578	8.481	11.539	19.392
14	0.2850	19.767	13.079	15.511	8.376	10.786
14	0.2860	11.442	12.937	11.935	14.697	14.126
14	0.2870	11.892	14.194	6.858	28.516	22.742
14	0.2880	7.192	16.065	15.120	19.861	14.419
14	0.2890	8.597	11.102	7.209	15.748	14.924
14	0.2900	12.790	14.113	11.229	12.343	25.044
14	0.2910	11.562	17.325	12.971	23.267	23.359
14	0.2920	26.070	27.085	17.980	21.316	17.623
14	0.2930	18.003	14.031	16.514	10.368	17.097
14	0.2940	20.456	13.056	25.211	14.620	11.402
14	0.2950	14.023	13.737	15.949	18.225	14.767
14	0.2960	12.568	12.298	17.137	9.912	7.561
14	0.2970	7.849	9.981	11.849	11.225	15.771
14	0.2980	15.015	7.878	6.902	12.427	12.199
14	0.2990	14.887	7.826	6.837	12.475	12.023
14	0.3000	9.424	6.344	7.263	16.619	8.590
14	0.3010	11.909	11.402	16.964	15.573	3.209
14	0.3020	24.628	15.985	22.613	18.783	12.462
14	0.3030	16.945	9.001	11.734	14.951	19.047
14	0.3040	19.497	14.153	18.616	24.629	15.747
14	0.3050	5.308	8.431	5.794	14.774	20.097
14	0.3060	9.524	7.705	8.571	15.926	12.499
14	0.3070	13.434	10.648	13.500	9.944	6.755
14	0.3080	20.959	19.366	19.574	14.434	13.703
14	0.3090	14.776	16.233	17.986	10.292	14.458
14	0.3100	15.108	15.880	14.251	10.764	22.668

14	0.3110	19.663	27.462	16.563	11.605	22.364
14	0.3120	27.432	34.263	25.224	18.303	12.817
14	0.3130	11.711	24.003	22.302	21.360	13.661
14	0.3140	15.739	17.564	16.467	25.977	15.274
14	0.3150	18.460	19.537	11.854	19.976	11.277
14	0.3160	7.385	8.659	6.426	5.578	13.789
14	0.3170	3.794	7.052	6.937	8.220	6.568
14	0.3180	23.546	19.042	22.316	28.349	16.288
14	0.3190	21.355	31.545	31.159	28.648	21.867
14	0.3200	21.356	31.569	31.110	28.677	21.860
14	0.3210	17.673	25.155	16.725	29.831	26.064
14	0.3220	16.496	28.194	19.923	32.241	15.443
14	0.3230	14.276	21.474	13.773	29.369	16.584
14	0.3240	15.726	15.201	14.666	22.992	27.041
14	0.3250	16.561	11.590	13.446	18.403	10.495
14	0.3260	3.691	3.773	1.097	10.702	4.646
14	0.3270	10.297	18.626	14.415	11.338	19.617
14	0.3280	17.262	19.587	17.447	18.334	22.094
14	0.3290	24.043	26.137	14.197	23.944	19.886
14	0.3300	9.415	16.896	10.001	20.204	12.271
14	0.3310	5.187	13.042	7.500	30.658	12.566
14	0.3320	8.306	8.514	10.523	9.500	7.857
14	0.3330	6.559	7.347	5.496	6.680	7.495
14	0.3340	11.390	10.706	8.670	9.892	14.675
14	0.3350	20.439	18.746	11.360	11.112	28.363
14	0.3360	12.780	27.566	14.452	34.892	14.963
14	0.3370	13.320	15.733	14.801	10.606	27.462
14	0.3380	3.903	3.382	3.031	11.402	25.476
14	0.3390	18.259	17.365	14.296	4.101	20.790
14	0.3400	23.506	31.187	19.276	16.518	12.731
14	0.3410	18.479	17.992	12.955	15.588	22.711
14	0.3420	5.215	6.875	3.406	12.851	8.633
14	0.3430	12.327	14.191	4.366	16.318	12.682
14	0.3440	12.330	14.274	4.321	16.515	12.542
14	0.3450	12.922	14.524	8.352	15.237	11.616
14	0.3460	13.903	17.541	14.177	18.529	25.695
14	0.3470	14.168	23.496	17.414	24.103	16.707
14	0.3480	14.256	23.437	17.523	24.092	16.615
14	0.3490	11.863	10.183	12.054	13.572	23.190
14	0.3500	26.303	22.579	22.022	25.891	16.321
14	0.3510	14.118	12.932	16.281	14.677	7.836
14	0.3520	16.946	15.983	15.833	12.335	14.523
14	0.3530	17.234	14.621	18.129	9.107	29.086
14	0.3540	19.309	21.501	20.220	18.467	12.108
14	0.3550	23.655	20.430	25.614	18.670	12.574
14	0.3560	29.845	23.837	29.761	14.515	26.551
14	0.3570	20.154	13.215	17.835	7.587	6.607
14	0.3580	7.773	9.138	10.108	11.113	9.268
14	0.3590	18.544	27.640	16.838	29.201	23.998
14	0.3600	18.397	20.058	14.073	25.200	5.363

14	0.3610	24.327	17.514	17.885	12.787	32.540
14	0.3620	20.272	14.958	14.783	15.595	29.457
14	0.3630	15.394	21.294	13.641	22.173	18.342
14	0.3640	9.618	11.449	12.584	22.289	15.444
14	0.3650	11.014	12.296	11.218	11.792	17.928
14	0.3660	8.186	11.033	12.940	11.889	14.505
14	0.3670	25.405	36.528	26.413	20.519	22.277
14	0.3680	9.170	22.844	14.191	17.456	8.406
14	0.3690	14.136	21.087	21.908	16.055	6.918
14	0.3700	14.319	21.209	19.668	18.103	6.459
14	0.3710	19.883	15.053	17.123	11.319	9.158
14	0.3720	22.582	22.326	21.143	14.948	9.583
14	0.3730	16.923	15.038	17.899	15.889	18.417
14	0.3740	17.514	15.935	15.877	13.726	24.307
14	0.3750	7.979	7.376	6.929	13.633	22.063
14	0.3760	13.180	20.940	19.225	23.208	6.986
14	0.3770	20.769	28.282	24.872	19.979	15.432
14	0.3780	22.088	22.739	22.058	18.055	15.076
14	0.3790	15.868	25.576	18.975	22.577	6.764
14	0.3800	20.024	13.813	15.147	13.090	12.116
14	0.3810	12.130	8.431	8.185	10.635	17.912
14	0.3820	16.086	23.612	11.191	20.319	10.955
14	0.3830	28.644	16.667	23.961	11.010	16.273
14	0.3840	19.453	23.916	17.960	19.437	10.872
14	0.3850	17.332	18.475	14.041	15.557	11.041
14	0.3860	17.357	18.559	14.080	15.605	11.034
14	0.3870	18.685	17.598	10.867	17.045	21.731
14	0.3880	27.223	18.057	17.631	14.136	17.714
14	0.3890	12.027	12.327	12.000	23.569	12.519
14	0.3900	16.997	18.622	18.375	24.121	18.374
14	0.3910	22.821	27.588	23.086	14.527	11.183
14	0.3920	16.549	14.104	13.122	12.390	6.096
14	0.3930	25.495	17.566	25.696	8.179	16.085
14	0.3940	15.299	6.417	12.441	12.904	14.083
14	0.3950	6.269	8.211	8.678	7.383	16.841
14	0.3960	8.257	7.760	5.541	21.385	30.689
14	0.3970	18.510	13.801	13.616	19.523	33.077
14	0.3980	21.804	17.670	21.032	16.398	13.137
14	0.3990	11.996	11.057	10.687	11.087	12.579
14	0.4000	8.797	9.158	10.107	6.459	14.518
14	0.4010	13.535	16.183	14.150	15.464	12.894
14	0.4020	6.098	5.139	7.833	6.274	4.613
14	0.4030	18.622	10.207	13.448	7.959	18.625
14	0.4040	14.815	13.714	13.316	7.865	21.298
14	0.4050	9.659	16.316	18.404	12.106	14.022
14	0.4060	12.619	14.285	18.399	5.305	10.339
14	0.4070	12.094	15.588	17.198	8.078	11.723
14	0.4080	8.104	13.053	15.181	12.232	31.602
14	0.4090	12.206	14.751	15.422	13.565	16.812
14	0.4100	12.581	11.005	18.249	16.969	19.369

14	0.4110	11.024	8.303	10.707	18.341	16.077
14	0.4120	13.694	8.873	12.707	11.182	13.232
14	0.4130	19.428	13.344	17.936	21.609	15.743
14	0.4140	7.444	7.047	9.484	3.475	9.345
14	0.4150	14.568	16.654	18.202	21.447	18.386
14	0.4160	9.303	5.570	7.183	10.033	17.588
14	0.4170	22.605	11.453	14.730	16.046	15.933
14	0.4180	16.996	10.867	9.615	8.730	14.728
14	0.4190	16.924	10.997	9.700	8.742	14.790
14	0.4200	7.570	8.009	6.478	7.149	4.210
14	0.4210	15.513	10.889	12.963	19.086	24.120
14	0.4220	13.064	11.720	10.677	12.864	10.681
14	0.4230	6.830	9.361	8.134	8.848	9.533
14	0.4240	13.591	8.772	10.646	15.244	6.576
14	0.4250	13.507	10.149	9.039	20.930	16.514
14	0.4260	14.767	10.903	11.053	19.494	17.039
14	0.4270	5.945	11.755	7.143	15.542	14.332
14	0.4280	18.189	15.675	13.913	19.946	19.070
14	0.4290	15.257	19.019	10.580	17.715	22.229
14	0.4300	7.585	12.351	7.803	11.386	11.651
14	0.4310	17.450	8.423	10.006	14.935	21.541
14	0.4320	15.283	16.242	15.190	13.264	12.724
14	0.4330	7.785	9.112	6.382	11.653	11.384
14	0.4340	13.855	15.541	11.501	7.258	16.008
14	0.4350	19.910	13.010	10.953	10.167	31.578
14	0.4360	22.425	13.775	15.744	23.395	19.001
14	0.4370	18.186	11.111	14.074	13.703	17.943
14	0.4380	16.986	11.419	12.229	17.091	23.949
14	0.4390	11.186	19.567	11.314	11.567	9.070
14	0.4400	5.878	16.822	9.662	14.979	18.098
14	0.4410	11.218	10.072	11.145	13.781	19.310
14	0.4420	12.559	12.354	11.727	16.115	7.225
14	0.4430	21.434	24.774	18.191	26.189	9.860
14	0.4440	13.288	13.524	11.135	9.801	10.185
14	0.4450	20.302	13.519	13.706	12.333	6.557
14	0.4460	26.620	22.434	20.191	9.888	8.260
14	0.4470	26.659	22.364	20.216	9.759	8.144
14	0.4480	8.041	14.561	7.748	22.759	20.436
14	0.4490	5.853	10.941	10.236	17.245	10.675
14	0.4500	5.931	6.812	4.966	17.359	11.365
14	0.4510	8.583	8.432	7.604	16.090	21.092
14	0.4520	10.985	15.013	7.152	17.957	23.252
14	0.4530	16.168	19.025	15.890	17.721	13.390
14	0.4540	15.907	21.549	18.956	20.875	13.498
14	0.4550	19.599	15.531	20.458	9.853	6.208
14	0.4560	22.803	19.470	20.553	22.864	13.699
14	0.4570	16.713	20.867	11.123	9.076	11.097
14	0.4580	17.776	22.022	11.851	9.015	11.837
14	0.4590	19.628	19.407	12.838	15.876	20.580
14	0.4600	14.616	16.953	15.427	14.947	15.489

14	0.4610	18.437	17.507	19.659	15.704	12.312
14	0.4620	25.607	19.841	23.833	7.745	11.875
14	0.4630	19.480	28.632	24.148	22.621	20.950
14	0.4640	22.422	27.001	27.133	12.445	14.757
14	0.4650	22.123	16.820	16.110	17.619	15.901
14	0.4660	17.429	19.651	14.182	18.172	15.446
14	0.4670	9.965	11.717	9.361	15.345	16.349
14	0.4680	25.006	23.924	16.603	12.006	12.925
14	0.4690	20.964	25.868	17.739	10.752	16.245
14	0.4700	29.186	31.793	21.468	19.114	20.172
14	0.4710	18.505	26.911	20.729	18.451	13.163
14	0.4720	16.947	27.467	15.687	19.075	13.792
14	0.4730	5.631	9.693	5.687	22.331	16.889
14	0.4740	12.307	10.981	8.700	13.849	7.967
14	0.4750	11.981	5.332	7.482	9.632	8.226
14	0.4760	14.406	8.878	10.454	5.750	13.677
14	0.4770	5.266	9.701	5.148	7.591	10.374
14	0.4780	7.270	8.928	6.552	5.150	11.353
14	0.4790	21.809	23.070	23.978	12.971	10.904
14	0.4800	19.942	25.942	23.512	14.528	9.294
14	0.4810	19.189	25.192	23.435	16.295	18.958
14	0.4820	15.208	19.460	18.116	11.419	19.181
14	0.4830	14.717	22.617	13.731	10.813	16.966
14	0.4840	20.054	15.310	18.153	9.955	13.841
14	0.4850	6.500	4.209	5.710	6.060	10.053
14	0.4860	13.048	16.433	12.965	14.269	7.192
14	0.4870	21.380	25.295	21.956	16.346	12.820
14	0.4880	19.506	22.071	14.480	23.770	25.172
14	0.4890	13.477	11.926	11.142	15.292	17.358
14	0.4900	17.176	20.019	23.318	8.945	11.567
14	0.4910	21.196	18.790	22.755	6.639	16.323
14	0.4920	21.540	19.583	23.270	13.412	20.132
14	0.4930	17.113	11.696	19.178	19.223	19.182
14	0.4940	7.745	13.332	13.545	12.509	9.935
14	0.4950	19.208	14.975	22.663	5.958	9.765
14	0.4960	17.192	14.288	18.611	7.895	14.485
14	0.4970	18.853	18.567	18.982	17.862	9.626
14	0.4980	15.529	19.919	24.147	16.219	18.077
14	0.4990	13.135	13.419	12.473	19.536	14.879
14	0.5000	15.814	17.009	16.741	12.612	21.990
14	0.5010	9.050	8.766	11.137	15.223	7.776
14	0.5020	11.096	11.894	14.130	12.483	13.724
14	0.5030	15.125	16.573	10.010	14.044	12.950
14	0.5040	14.774	11.020	14.223	17.004	9.216
14	0.5050	13.807	11.007	11.874	16.095	21.720
14	0.5060	16.959	22.660	15.461	6.787	16.075
14	0.5070	19.378	21.198	16.647	11.739	15.631
14	0.5080	16.920	25.056	19.693	19.469	15.665
14	0.5090	6.518	10.520	6.710	17.635	23.322
14	0.5100	18.950	14.666	16.646	18.540	17.740

14	0.5110	20.852	19.103	15.295	12.118	14.466
14	0.5120	17.745	13.107	14.084	14.193	24.552
14	0.5130	19.285	19.797	13.711	17.262	22.276
14	0.5140	13.944	13.970	19.528	16.702	18.775
14	0.5150	15.833	12.058	16.933	16.603	19.253
14	0.5160	17.939	20.624	22.620	26.710	16.227
14	0.5170	16.039	17.604	18.158	30.109	14.250
14	0.5180	9.389	20.523	11.642	23.309	6.531
14	0.5190	23.064	29.788	19.796	17.437	7.685
14	0.5200	21.265	23.093	18.817	18.179	12.001
14	0.5210	16.520	21.390	15.910	23.246	16.162
14	0.5220	19.796	25.722	18.919	13.299	13.682
14	0.5230	12.011	13.600	14.510	20.522	12.982
14	0.5240	15.871	11.216	15.032	17.449	11.645
14	0.5250	30.279	15.901	31.865	23.153	9.211
14	0.5260	24.432	16.822	21.001	23.059	7.037
14	0.5270	21.609	14.508	17.490	10.041	19.830
14	0.5280	14.357	12.374	13.352	13.007	19.291
14	0.5290	11.249	14.350	11.692	17.262	10.849
14	0.5300	11.203	6.123	11.454	16.308	7.243
14	0.5310	24.035	23.829	19.360	18.949	20.819
14	0.5320	21.074	20.925	17.376	18.638	22.822
14	0.5330	6.317	10.166	6.118	18.355	18.779
14	0.5340	7.283	7.970	8.278	14.944	14.932
14	0.5350	24.764	22.756	17.233	19.894	16.543
14	0.5360	7.342	9.522	12.594	10.137	14.178
14	0.5370	11.906	16.166	17.671	9.578	8.254
14	0.5380	26.013	23.767	22.188	13.392	15.136
14	0.5390	6.906	14.609	14.095	11.486	12.340
14	0.5400	11.532	13.586	14.164	13.570	21.134
14	0.5410	13.222	12.961	15.371	18.592	18.674
14	0.5420	12.627	11.935	14.856	17.969	18.728
14	0.5430	10.526	16.025	14.388	19.471	12.075
14	0.5440	17.968	13.655	11.014	12.465	16.376
14	0.5450	5.668	10.367	8.449	16.849	16.520
14	0.5460	13.364	18.693	9.832	14.852	17.340
14	0.5470	25.466	27.265	22.661	16.199	19.897
14	0.5480	8.028	12.728	8.879	17.123	11.161
14	0.5490	8.367	16.260	10.275	14.611	8.052
14	0.5500	8.913	16.335	11.824	20.460	18.976
14	0.5510	16.613	22.643	17.789	20.303	21.589
14	0.5520	18.935	16.757	17.206	12.476	12.365
14	0.5530	11.382	15.529	10.354	13.561	14.625
14	0.5540	16.715	20.048	12.583	12.832	17.953
14	0.5550	20.449	29.365	23.854	21.506	17.432
14	0.5560	21.466	30.565	24.693	22.171	16.721
14	0.5570	16.445	26.468	19.021	16.448	15.785
14	0.5580	7.544	13.313	9.123	6.546	7.354
14	0.5590	11.674	14.702	14.371	15.284	14.136
14	0.5600	22.544	19.444	19.379	19.076	16.275

14	0.5610	10.412	11.446	11.479	17.995	21.697
14	0.5620	11.236	16.578	15.236	8.651	9.596
14	0.5630	9.634	18.309	12.783	20.438	7.239
14	0.5640	12.849	20.113	12.807	21.110	20.276
14	0.5650	19.522	28.216	19.214	24.374	22.911
14	0.5660	15.631	19.647	14.826	19.332	15.991
14	0.5670	9.815	14.317	13.110	6.001	7.490
14	0.5680	22.658	31.310	15.683	16.684	15.111
14	0.5690	17.718	26.736	21.677	20.537	22.465
14	0.5700	8.480	16.919	14.156	21.003	20.688
14	0.5710	6.323	10.435	10.061	21.126	14.238
14	0.5720	9.196	11.928	9.689	26.415	16.975
14	0.5730	9.186	11.077	9.869	15.823	21.656
14	0.5740	10.773	11.551	9.172	13.872	17.044
14	0.5750	25.642	38.008	28.383	20.627	12.072
14	0.5760	14.660	21.502	18.902	13.455	7.740
14	0.5770	12.355	21.791	20.873	16.647	6.649
14	0.5780	20.258	26.261	23.694	11.909	8.031
14	0.5790	14.950	24.457	20.591	11.167	12.624
14	0.5800	8.233	16.025	14.719	15.464	16.508
14	0.5810	8.083	15.003	14.560	20.624	17.549
14	0.5820	29.290	31.245	25.231	19.498	12.921
14	0.5830	20.295	9.315	11.149	16.826	9.281
14	0.5840	17.194	22.772	13.448	16.363	11.979
14	0.5850	10.149	17.987	13.829	8.694	12.140
14	0.5860	5.858	11.783	6.610	9.167	11.723
14	0.5870	8.822	13.255	7.319	10.197	11.345
14	0.5880	14.904	15.454	12.487	6.749	12.307
14	0.5890	17.881	17.989	14.178	5.593	9.744
14	0.5900	19.477	23.038	16.527	3.539	6.263
14	0.5910	23.014	27.029	20.967	12.778	12.724
14	0.5920	17.565	15.897	12.329	13.366	14.753
14	0.5930	14.241	6.706	10.925	14.242	12.622
14	0.5940	10.353	14.381	9.424	25.900	8.644
14	0.5950	25.828	22.291	19.995	12.805	11.504
14	0.5960	14.863	21.615	16.239	20.359	4.680
14	0.5970	21.007	24.718	19.387	23.675	11.007
14	0.5980	16.922	11.020	12.663	12.336	19.173
14	0.5990	15.486	12.564	11.567	17.256	16.606
14	0.6000	20.965	18.074	12.527	13.809	17.743
14	0.6010	14.842	16.409	14.701	11.668	9.944
14	0.6020	12.355	15.403	10.901	18.478	15.986
14	0.6030	19.240	17.982	15.532	12.724	10.378
14	0.6040	17.660	10.504	15.046	18.290	15.298
14	0.6050	10.591	8.348	6.259	14.987	15.678
14	0.6060	9.714	11.917	6.906	13.833	14.711
14	0.6070	11.570	13.288	10.213	19.722	14.610
14	0.6080	8.350	19.161	14.483	19.275	4.346
14	0.6090	8.220	16.969	13.940	14.732	8.959
14	0.6100	6.497	9.263	8.784	15.410	6.869

14	0.6110	8.650	10.171	8.834	14.557	10.909
14	0.6120	6.920	7.623	6.425	23.580	14.329
14	0.6130	16.590	18.427	14.211	20.413	16.558
14	0.6140	18.825	7.001	12.165	17.058	16.092
14	0.6150	11.614	8.818	8.675	10.388	16.378
14	0.6160	14.230	13.289	12.231	7.852	8.119
14	0.6170	9.261	7.067	7.414	4.265	6.429
14	0.6180	6.929	7.957	6.556	6.043	11.098
14	0.6190	6.930	7.952	6.555	6.043	11.098
14	0.6200	15.458	14.329	10.411	4.304	10.046
14	0.6210	13.302	7.930	9.516	6.988	9.095
14	0.6220	10.737	5.745	6.742	3.180	8.107
14	0.6230	7.815	3.841	5.124	7.134	10.824
14	0.6240	11.414	6.667	5.826	9.494	8.591
14	0.6250	11.468	11.284	9.123	8.691	5.573
14	0.6260	7.104	9.191	7.656	7.291	4.003
14	0.6270	12.253	15.544	14.256	27.760	20.926
14	0.6280	20.844	13.959	16.354	20.387	16.176
14	0.6290	7.428	5.969	6.163	17.081	13.910
14	0.6300	16.159	16.409	12.882	12.284	10.169
14	0.6310	15.810	19.343	13.720	10.950	11.343
14	0.6320	11.752	8.648	9.918	8.234	4.076
14	0.6330	6.927	13.143	8.026	13.770	13.725
14	0.6340	10.825	12.218	10.139	9.032	8.575
14	0.6350	12.247	13.633	12.731	15.644	22.556
14	0.6360	16.307	10.849	12.071	21.008	15.848
14	0.6370	9.821	12.480	11.772	12.593	7.021
14	0.6380	5.749	7.774	8.968	5.418	14.085
14	0.6390	11.762	12.482	12.528	9.735	14.342
14	0.6400	11.504	9.025	11.214	8.333	19.106
14	0.6410	17.018	19.931	17.346	16.187	21.682
14	0.6420	17.628	19.438	12.705	16.420	20.657
14	0.6430	12.292	11.651	9.129	11.117	18.396
14	0.6440	4.882	5.449	4.483	13.282	14.715
14	0.6450	12.015	8.462	10.808	11.754	19.565
14	0.6460	15.329	8.137	9.295	11.089	24.792
14	0.6470	15.467	8.386	9.370	11.173	26.155
14	0.6480	26.028	14.428	19.958	11.559	26.476
14	0.6490	15.778	19.151	14.053	25.989	24.977
14	0.6500	14.742	22.936	16.401	24.476	20.970
14	0.6510	13.496	14.197	14.306	16.138	15.569
14	0.6520	6.120	6.599	4.916	9.468	9.727
14	0.6530	19.135	14.771	14.848	11.264	13.425
14	0.6540	10.539	10.112	9.093	4.015	10.027
14	0.6550	10.080	10.099	8.094	13.670	13.319
14	0.6560	13.374	10.078	12.154	15.637	18.728
14	0.6570	8.850	13.508	5.280	22.106	17.370
14	0.6580	8.324	9.399	5.741	4.518	6.791
14	0.6590	24.419	30.574	26.621	29.674	23.252
14	0.6600	7.605	6.879	6.465	11.403	13.771

14	0.6610	14.359	8.522	9.092	12.190	24.257
14	0.6620	4.462	2.778	5.597	7.058	15.989
14	0.6630	4.639	3.675	6.603	10.561	13.127
14	0.6640	15.524	12.152	7.313	13.800	15.961
14	0.6650	9.543	7.027	7.085	9.101	14.943
14	0.6660	7.895	8.157	6.443	10.797	10.966
14	0.6670	11.952	4.393	6.102	8.667	15.270
14	0.6680	7.907	11.097	6.184	23.272	19.484
14	0.6690	15.357	12.025	8.934	14.437	19.502
14	0.6700	5.276	5.826	4.429	3.340	14.116
14	0.6710	3.041	3.826	1.538	5.065	9.895
14	0.6720	18.584	7.580	9.290	7.861	7.991
14	0.6730	2.296	4.219	3.415	0.888	7.009
14	0.6740	1.069	2.412	2.074	0.462	1.847
14	0.6750	5.735	5.116	4.943	3.232	3.738
14	0.6760	7.548	4.731	4.098	6.245	12.485
14	0.6770	12.124	7.471	6.477	7.291	20.686
14	0.6780	10.821	8.457	5.034	7.135	20.360
14	0.6790	7.920	7.256	4.323	13.444	5.040
14	0.6800	8.506	10.192	12.298	19.145	10.542
14	0.6810	7.859	11.374	13.275	8.239	11.246
14	0.6820	12.866	15.521	15.081	8.433	5.085
14	0.6830	21.674	17.476	17.294	16.733	6.212
14	0.6840	18.962	7.821	12.643	11.641	6.583
14	0.6850	21.491	17.540	18.153	14.410	11.146
14	0.6860	14.939	17.027	15.131	16.184	15.195
14	0.6870	14.902	16.995	15.085	16.182	15.141
14	0.6880	18.284	13.800	12.561	17.342	18.777
14	0.6890	6.893	12.005	7.342	17.963	13.741
14	0.6900	10.507	12.577	12.264	19.418	16.065
14	0.6910	12.299	16.585	12.580	18.597	15.999
14	0.6920	17.204	15.502	13.777	13.822	21.681
14	0.6930	11.973	10.045	11.232	10.366	23.383
14	0.6940	18.891	16.121	18.382	15.382	29.501
14	0.6950	7.829	8.315	5.429	18.548	14.782
14	0.6960	7.014	8.190	7.634	14.512	8.210
14	0.6970	2.209	5.192	3.863	13.245	6.003
14	0.6980	6.242	10.423	6.600	14.315	6.044
14	0.6990	7.631	5.873	6.465	3.302	6.772
14	0.7000	13.222	15.093	15.025	8.529	15.463
14	0.7010	11.779	14.854	13.268	15.846	14.586
14	0.7020	11.666	13.334	11.130	19.419	19.686
14	0.7030	16.367	10.223	13.152	12.464	18.817
14	0.7040	24.504	23.553	18.785	20.836	29.600
14	0.7050	4.422	3.761	4.620	6.087	6.297
14	0.7060	6.587	9.127	7.399	8.661	12.391
14	0.7070	15.046	10.305	13.788	6.938	12.994
14	0.7080	7.822	5.500	10.952	14.147	15.320
14	0.7090	9.609	4.889	10.089	23.286	14.939
14	0.7100	6.929	6.422	11.110	9.909	18.504

14	0.7110	4.906	6.209	5.374	12.664	15.222
14	0.7120	7.089	13.538	11.645	12.362	6.353
14	0.7130	9.105	8.674	11.641	7.686	10.898
14	0.7140	12.559	12.761	12.090	17.669	29.098
14	0.7150	13.977	12.577	14.451	12.597	23.302
14	0.7160	11.424	12.858	18.807	14.848	10.886
14	0.7170	12.765	12.692	13.728	11.363	17.390
14	0.7180	16.722	16.261	14.524	7.222	23.327
14	0.7190	12.367	20.578	18.599	9.338	13.992
14	0.7200	5.388	9.215	9.119	8.997	10.928
14	0.7210	14.359	15.576	13.248	10.521	13.748
14	0.7220	11.495	10.092	8.550	9.782	20.357
14	0.7230	10.916	9.128	12.263	15.305	18.584
14	0.7240	15.248	12.162	11.353	14.844	10.589
14	0.7250	25.386	14.766	15.267	8.151	21.770
14	0.7260	18.822	17.586	12.654	10.175	17.578
14	0.7270	27.829	14.625	14.369	11.128	25.007
14	0.7280	35.329	35.002	29.139	13.573	11.361
14	0.7290	24.936	27.224	21.849	10.761	20.604
14	0.7300	15.766	15.501	18.172	6.462	11.523
14	0.7310	12.038	12.246	15.038	6.981	17.520
14	0.7320	13.834	12.403	15.339	12.127	17.732
14	0.7330	18.032	13.286	17.230	16.177	24.554
14	0.7340	9.470	8.661	11.168	12.086	14.919
14	0.7350	9.716	11.490	9.843	10.377	14.190
14	0.7360	15.196	13.889	12.410	12.829	22.768
14	0.7370	15.211	12.428	12.824	13.136	20.171
14	0.7380	12.552	12.803	11.138	6.770	7.461
14	0.7390	10.636	10.393	11.170	16.492	10.347
14	0.7400	17.135	12.500	16.357	20.517	17.435
14	0.7410	18.708	15.867	20.806	15.624	12.224
14	0.7420	4.427	10.157	4.986	10.208	12.575
14	0.7430	15.208	11.427	13.592	18.501	13.795
14	0.7440	18.854	11.699	19.587	13.494	12.653
14	0.7450	5.710	4.767	5.143	3.488	12.440
14	0.7460	15.390	15.589	11.726	15.310	29.315
14	0.7470	9.341	11.900	8.044	14.653	21.759
14	0.7480	9.586	10.342	8.158	11.803	24.002
14	0.7490	9.490	9.050	13.972	8.305	11.638
14	0.7500	2.555	5.145	7.138	7.532	20.007
14	0.7510	15.413	11.901	13.542	4.503	15.573
14	0.7520	6.624	3.404	3.856	3.505	11.929
14	0.7530	2.343	3.431	4.624	10.859	7.859
14	0.7540	1.451	1.318	3.444	3.236	4.415
14	0.7550	5.894	3.763	8.129	3.933	4.148
14	0.7560	14.260	13.636	12.782	11.153	11.745
14	0.7570	21.571	21.944	24.172	9.635	13.158
14	0.7580	7.195	5.443	5.252	8.526	16.065
14	0.7590	21.679	15.367	14.372	13.270	16.762
14	0.7600	28.036	30.043	15.188	15.440	19.142

14	0.7610	23.025	22.092	13.257	8.904	17.067
14	0.7620	20.628	17.548	12.367	19.240	26.117
14	0.7630	14.206	11.924	9.292	21.365	27.301
14	0.7640	6.206	7.646	4.659	13.767	19.547
14	0.7650	15.143	13.177	9.771	13.045	23.133
14	0.7660	17.488	13.255	15.221	14.520	12.253
14	0.7670	23.174	18.547	19.316	21.168	28.703
14	0.7680	16.617	14.475	11.697	28.497	32.296
14	0.7690	14.168	16.232	18.040	18.028	25.790
14	0.7700	15.080	13.227	15.373	11.128	22.293
14	0.7710	16.930	12.203	12.432	15.180	30.607
14	0.7720	10.012	3.906	3.166	12.622	24.136
14	0.7730	12.073	8.627	9.895	19.047	22.134
14	0.7740	16.069	10.293	12.390	13.773	39.760
14	0.7750	11.515	17.994	10.868	22.424	33.557
14	0.7760	12.428	11.945	10.653	10.085	13.425
14	0.7770	10.570	5.216	9.693	7.518	14.965
14	0.7780	10.707	12.107	15.855	13.735	28.069
14	0.7790	8.940	12.144	12.425	11.040	27.072
14	0.7800	8.969	11.946	12.221	11.113	27.192
14	0.7810	12.794	12.815	17.437	14.680	25.320
14	0.7820	13.220	9.393	19.345	11.708	14.308
14	0.7830	17.191	11.769	18.165	9.492	26.417
14	0.7840	10.087	5.949	13.994	19.182	12.940
14	0.7850	7.349	6.843	11.281	14.024	19.409
14	0.7860	7.492	5.280	8.668	10.172	14.688
14	0.7870	9.312	11.610	12.664	17.835	21.733
14	0.7880	15.737	12.565	15.534	21.725	22.913
14	0.7890	15.234	14.929	12.548	17.002	35.495
14	0.7900	9.549	8.450	7.649	15.523	22.517
14	0.7910	6.594	7.108	7.451	26.771	25.416
14	0.7920	12.053	8.606	9.673	23.041	16.543
14	0.7930	28.223	20.596	19.460	17.105	23.711
14	0.7940	24.739	16.993	23.452	8.726	14.171
14	0.7950	15.480	13.596	15.418	3.452	13.392
14	0.7960	20.101	10.085	13.944	12.721	21.280
14	0.7970	18.757	13.024	18.216	24.894	32.992
14	0.7980	16.363	11.610	16.629	11.422	8.984
14	0.7990	8.924	9.972	8.689	22.038	16.676
14	0.8000	18.748	14.107	15.260	21.507	23.661
14	0.8010	13.069	10.005	12.296	12.679	19.787
14	0.8020	25.733	17.570	17.762	7.042	17.819
14	0.8030	26.373	16.081	18.287	7.024	17.250
14	0.8040	20.085	6.490	19.505	14.432	28.847
14	0.8050	10.227	8.918	11.689	14.330	34.774
14	0.8060	7.644	5.955	5.801	12.769	15.791
14	0.8070	9.389	6.462	8.552	9.914	14.308
14	0.8080	9.553	8.881	14.401	9.917	10.630
14	0.8090	11.580	7.965	9.053	11.269	7.110
14	0.8100	17.026	18.610	17.819	12.738	9.968

14	0.8110	21.908	19.304	17.955	15.281	11.184
14	0.8120	11.614	10.541	12.093	11.700	9.675
14	0.8130	13.645	5.978	11.622	12.198	12.136
14	0.8140	9.477	8.863	10.166	21.121	30.003
14	0.8150	10.366	7.605	11.074	11.848	14.734
14	0.8160	6.807	5.553	7.067	6.872	12.793
14	0.8170	14.908	14.437	15.125	6.938	14.759
14	0.8180	10.683	8.432	8.481	9.977	24.579
14	0.8190	10.661	8.883	9.509	12.953	21.471
14	0.8200	9.638	8.183	16.478	10.453	22.206
14	0.8210	9.566	11.528	12.261	22.515	17.097
14	0.8220	15.422	10.141	16.090	17.749	17.681
14	0.8230	17.400	20.053	20.133	15.916	15.947
14	0.8240	10.134	13.458	15.391	20.761	15.224
14	0.8250	8.863	11.359	11.679	16.862	16.573
14	0.8260	13.654	12.406	18.626	8.229	17.989
14	0.8270	10.943	13.418	12.091	22.808	20.497
14	0.8280	11.202	13.370	13.210	21.774	22.967
14	0.8290	22.122	19.855	18.810	26.029	31.198
14	0.8300	2.471	2.111	2.706	5.614	18.169
14	0.8310	12.013	10.750	7.624	9.143	12.012
14	0.8320	12.242	10.142	16.159	6.757	16.251
14	0.8330	8.711	9.001	13.895	7.726	4.301
14	0.8340	18.353	10.557	21.463	24.802	7.521
14	0.8350	19.966	12.623	20.065	16.433	16.626
14	0.8360	9.545	10.514	10.567	15.370	15.917
14	0.8370	6.362	8.857	7.651	15.361	18.202
14	0.8380	7.556	14.382	9.564	17.538	22.256
14	0.8390	16.720	22.153	22.074	7.554	14.509
14	0.8400	17.157	20.540	21.449	9.579	10.058
14	0.8410	12.526	11.774	22.235	21.387	16.610
14	0.8420	12.242	8.658	15.904	9.018	23.165
14	0.8430	8.814	7.305	8.349	20.162	17.004
14	0.8440	7.482	6.964	7.870	20.240	20.802
14	0.8450	14.781	12.402	18.128	17.166	34.230
14	0.8460	10.620	8.488	10.274	15.930	16.794
14	0.8470	12.164	12.812	10.556	21.072	34.022
14	0.8480	7.682	7.085	11.205	9.502	7.475
14	0.8490	10.747	7.944	9.375	7.286	9.443
14	0.8500	10.175	11.673	12.158	8.719	15.202
14	0.8510	15.031	14.050	16.935	16.772	22.738
14	0.8520	11.908	12.034	7.238	23.620	29.199
14	0.8530	10.861	11.793	11.686	26.673	35.195
14	0.8540	17.941	20.437	16.413	11.644	17.938
14	0.8550	8.582	8.983	11.617	17.627	21.314
14	0.8560	3.916	5.832	5.856	11.034	21.241
14	0.8570	11.811	15.874	14.608	12.924	20.918
14	0.8580	6.924	5.200	6.330	10.404	22.749
14	0.8590	15.546	14.886	14.145	15.059	24.323
14	0.8600	14.822	15.897	11.663	14.021	16.432

14	0.8610	10.540	7.921	7.151	12.758	15.820
14	0.8620	17.942	13.506	11.485	16.770	17.647
14	0.8630	11.170	8.527	12.056	5.711	19.093
14	0.8640	18.232	13.253	18.342	12.851	21.487
14	0.8650	4.166	4.081	5.085	8.872	6.110
14	0.8660	11.621	9.037	13.789	4.688	5.082
14	0.8670	16.263	15.460	17.063	12.479	18.044
14	0.8680	13.401	14.200	12.732	14.598	12.978
14	0.8690	9.599	13.076	13.666	18.609	30.938
14	0.8700	9.571	12.551	12.684	15.573	15.827
14	0.8710	17.013	13.532	16.940	16.239	8.020
14	0.8720	8.260	9.200	13.115	10.192	14.033
14	0.8730	4.803	11.066	11.798	18.811	15.169
14	0.8740	6.367	13.171	8.197	16.610	12.400
14	0.8750	4.464	10.847	6.815	13.072	15.568
14	0.8760	4.531	9.014	3.592	8.494	3.874
14	0.8770	12.082	14.096	14.705	16.291	12.407
14	0.8780	11.293	11.771	13.032	9.507	13.520
14	0.8790	11.892	15.131	16.401	19.672	15.580
14	0.8800	10.097	14.551	8.999	16.323	18.209
14	0.8810	16.451	26.079	17.605	16.954	14.401
14	0.8820	11.721	26.542	17.912	15.437	11.181
14	0.8830	7.645	11.932	14.345	10.661	9.672
14	0.8840	17.584	7.855	12.566	13.388	9.240
14	0.8850	10.830	10.291	13.188	18.041	17.830
14	0.8860	9.600	13.804	15.593	15.165	10.354
14	0.8870	9.941	10.596	14.958	9.106	15.458
14	0.8880	9.024	12.100	12.698	23.344	8.892
14	0.8890	8.951	12.171	12.782	23.394	8.912
14	0.8900	12.627	22.499	24.261	25.317	16.911
14	0.8910	14.398	14.779	21.141	13.802	21.240
14	0.8920	12.312	15.425	23.365	14.021	23.614
14	0.8930	13.747	10.623	18.207	17.526	16.112
14	0.8940	14.161	11.202	18.950	17.588	16.111
14	0.8950	20.691	16.807	23.502	9.734	24.140
14	0.8960	14.430	18.457	20.010	10.891	14.532
14	0.8970	14.225	19.927	18.231	8.728	13.050
14	0.8980	12.922	14.401	15.977	12.848	13.210
14	0.8990	14.129	17.750	17.067	14.600	5.241
14	0.9000	14.063	17.591	17.058	14.716	5.198
14	0.9010	15.770	13.216	22.849	7.636	12.705
14	0.9020	25.261	30.152	35.458	16.198	19.281
14	0.9030	13.421	15.983	14.156	16.611	18.778
14	0.9040	8.312	12.073	10.683	11.458	6.822
14	0.9050	5.548	5.632	4.145	7.386	14.366
14	0.9060	8.170	7.575	9.342	6.136	8.183
14	0.9070	15.848	10.455	11.777	8.992	17.614
14	0.9080	19.953	11.051	20.198	10.813	8.432
14	0.9090	14.049	13.660	10.649	10.949	20.090
14	0.9100	17.001	16.048	13.535	10.063	20.137

14	0.9110	12.601	11.720	11.763	6.776	22.637
14	0.9120	10.367	11.013	7.462	18.515	20.227
14	0.9130	14.703	12.576	9.716	13.631	9.092
14	0.9140	15.830	11.999	11.513	10.646	13.832
14	0.9150	19.384	18.525	16.929	4.999	14.559
14	0.9160	12.948	10.825	15.334	14.827	12.412
14	0.9170	10.899	15.830	16.792	15.165	15.695
14	0.9180	9.275	13.314	6.934	10.044	8.711
14	0.9190	17.212	17.828	12.110	8.237	13.606
14	0.9200	10.899	16.494	13.767	21.645	9.670
14	0.9210	5.029	8.517	5.765	11.243	5.637
14	0.9220	6.814	7.449	8.880	9.067	15.870
14	0.9230	0.000	0.000	0.000	0.000	0.000
15	0.0000	0.000	0.000	0.000	0.000	0.000
15	0.0010	0.000	0.000	0.000	0.000	0.000
15	0.0020	12.678	7.204	9.779	18.661	25.662
15	0.0030	12.664	7.155	9.771	18.608	25.523
15	0.0040	12.652	7.114	9.765	18.557	25.376
15	0.0050	3.132	3.207	7.376	9.500	13.366
15	0.0060	3.184	3.165	7.442	9.392	13.289
15	0.0070	3.237	3.130	7.510	9.284	13.215
15	0.0080	5.550	9.587	9.839	13.152	14.111
15	0.0090	5.552	9.587	9.846	13.146	14.085
15	0.0100	6.711	5.058	6.940	16.414	16.403
15	0.0110	6.622	5.046	6.902	16.375	16.446
15	0.0120	9.318	8.215	5.416	19.321	19.227
15	0.0130	7.884	7.418	12.982	10.802	15.483
15	0.0140	7.756	6.618	8.228	10.451	17.937
15	0.0150	7.763	6.677	8.188	10.570	17.853
15	0.0160	10.716	9.740	10.304	14.518	15.007
15	0.0170	13.062	12.812	11.224	19.770	26.696
15	0.0180	23.388	16.330	14.002	18.097	28.186
15	0.0190	15.229	15.387	12.726	19.855	19.656
15	0.0200	14.267	19.869	16.861	26.310	14.790
15	0.0210	14.280	19.846	17.122	26.158	14.961
15	0.0220	11.807	12.306	14.904	11.726	22.993
15	0.0230	7.525	9.514	13.558	7.691	17.716
15	0.0240	7.466	5.296	7.947	10.485	10.257
15	0.0250	16.309	6.983	13.108	16.583	12.870
15	0.0260	3.876	6.623	6.312	16.102	11.255
15	0.0270	16.924	13.050	13.818	20.889	20.625
15	0.0280	6.563	10.416	11.480	16.113	15.867
15	0.0290	12.152	13.304	12.258	12.462	20.067
15	0.0300	11.135	8.239	5.444	21.996	28.121
15	0.0310	5.765	9.285	7.550	10.082	16.473
15	0.0320	7.850	12.283	14.971	15.962	18.718
15	0.0330	6.069	9.892	10.816	12.662	26.000
15	0.0340	25.994	15.618	23.426	26.747	18.387
15	0.0350	13.602	12.760	15.430	20.624	13.713
15	0.0360	8.357	10.218	10.368	15.573	11.993

15	0.0370	5.057	7.051	6.854	13.158	11.326
15	0.0380	4.985	6.396	5.096	18.222	17.279
15	0.0390	5.508	6.150	5.730	10.196	15.843
15	0.0400	13.272	12.279	13.726	17.532	13.672
15	0.0410	10.349	14.791	18.448	16.353	18.618
15	0.0420	7.993	13.982	14.833	13.043	16.841
15	0.0430	6.234	7.430	12.156	12.480	20.885
15	0.0440	9.736	13.218	14.495	15.432	20.075
15	0.0450	9.510	12.990	13.943	15.476	19.759
15	0.0460	9.246	11.957	10.847	16.098	27.737
15	0.0470	11.649	11.350	13.465	16.979	28.338
15	0.0480	18.535	22.824	16.304	24.836	25.915
15	0.0490	16.147	14.897	17.738	30.023	27.729
15	0.0500	11.354	9.654	10.388	13.142	19.591
15	0.0510	11.397	7.439	13.449	24.362	19.329
15	0.0520	19.190	9.631	20.495	21.055	15.801
15	0.0530	12.256	6.049	11.091	18.625	22.679
15	0.0540	11.491	7.391	10.298	22.437	15.649
15	0.0550	11.918	8.095	14.159	25.998	11.651
15	0.0560	11.935	8.104	14.136	26.173	11.714
15	0.0570	9.783	14.934	8.131	26.931	13.428
15	0.0580	10.564	11.709	13.316	18.458	16.427
15	0.0590	4.154	5.519	5.220	15.261	8.951
15	0.0600	5.868	6.452	5.442	12.944	13.065
15	0.0610	12.127	14.049	15.503	11.734	14.686
15	0.0620	8.498	6.364	9.053	8.027	14.589
15	0.0630	10.555	8.736	12.985	13.336	16.822
15	0.0640	8.748	12.002	10.564	17.969	18.209
15	0.0650	18.156	16.168	16.297	15.088	30.377
15	0.0660	23.760	21.627	19.590	29.094	29.877
15	0.0670	17.203	13.167	19.518	24.411	16.724
15	0.0680	15.299	8.798	12.478	16.401	18.140
15	0.0690	14.548	9.756	10.771	12.297	22.710
15	0.0700	33.179	28.043	34.249	21.082	24.121
15	0.0710	18.227	22.644	22.767	22.318	31.086
15	0.0720	20.233	21.086	24.769	28.983	27.461
15	0.0730	11.384	13.235	19.394	20.248	31.198
15	0.0740	11.264	13.196	19.407	19.574	31.028
15	0.0750	13.788	16.818	14.649	21.639	17.574
15	0.0760	11.344	18.821	16.789	20.288	26.823
15	0.0770	22.655	17.340	20.519	21.022	26.509
15	0.0780	23.980	24.786	23.991	20.591	32.757
15	0.0790	21.732	24.509	23.471	29.289	31.129
15	0.0800	15.207	13.747	16.769	19.655	20.991
15	0.0810	15.542	19.129	19.415	17.110	23.836
15	0.0820	25.948	15.697	19.511	17.031	30.907
15	0.0830	20.518	8.511	15.914	16.054	35.761
15	0.0840	15.976	8.071	14.158	13.789	20.390
15	0.0850	10.517	10.196	10.222	19.074	20.573
15	0.0860	15.460	6.292	15.124	5.820	12.159

15	0.0870	18.687	7.024	16.505	8.029	12.574
15	0.0880	8.071	9.922	10.240	11.808	18.425
15	0.0890	27.803	16.254	24.538	6.416	12.663
15	0.0900	23.913	12.380	20.564	15.478	13.710
15	0.0910	17.420	9.414	19.230	6.142	10.699
15	0.0920	19.664	12.306	15.604	5.819	5.250
15	0.0930	6.267	6.955	9.018	6.426	4.982
15	0.0940	13.471	7.723	16.636	4.594	3.648
15	0.0950	17.891	11.394	18.756	14.185	14.936
15	0.0960	16.578	6.106	12.505	9.676	23.521
15	0.0970	19.024	15.741	15.050	14.968	16.457
15	0.0980	16.400	19.427	13.312	27.786	16.629
15	0.0990	15.473	11.572	14.107	19.559	17.599
15	0.1000	14.863	13.038	12.323	29.779	22.919
15	0.1010	18.374	8.936	12.240	8.922	9.386
15	0.1020	16.587	8.427	11.105	20.318	15.355
15	0.1030	9.564	8.689	9.423	11.917	16.917
15	0.1040	20.457	14.937	14.255	15.271	12.416
15	0.1050	10.234	9.373	13.646	9.443	14.610
15	0.1060	14.005	14.427	17.250	18.585	10.486
15	0.1070	15.653	13.975	23.121	16.750	15.300
15	0.1080	21.808	14.854	24.699	9.847	16.872
15	0.1090	20.367	15.693	23.819	11.673	25.139
15	0.1100	17.127	9.932	11.656	15.021	10.833
15	0.1110	9.199	8.787	5.789	16.114	17.393
15	0.1120	8.350	5.735	6.591	26.672	19.874
15	0.1130	4.475	6.680	9.275	18.734	20.612
15	0.1140	6.307	8.886	13.152	17.392	21.940
15	0.1150	5.490	6.279	6.496	18.021	26.730
15	0.1160	16.149	12.364	17.021	22.265	18.679
15	0.1170	16.217	12.399	17.026	22.177	18.686
15	0.1180	16.275	12.428	17.017	22.087	18.693
15	0.1190	10.009	7.395	11.137	19.673	15.618
15	0.1200	14.960	16.462	11.640	25.089	29.486
15	0.1210	17.577	18.229	15.009	17.565	28.529
15	0.1220	11.832	13.065	14.341	16.089	34.092
15	0.1230	8.926	8.706	10.607	15.978	22.655
15	0.1240	10.812	13.483	16.659	22.924	30.042
15	0.1250	19.651	15.345	20.007	19.769	24.889
15	0.1260	11.247	9.585	11.898	14.448	32.479
15	0.1270	13.897	9.298	15.415	14.851	28.753
15	0.1280	13.392	5.628	9.380	13.777	34.199
15	0.1290	13.404	9.133	18.123	11.597	24.492
15	0.1300	17.241	15.010	19.719	14.572	25.472
15	0.1310	19.109	15.073	21.694	11.606	19.277
15	0.1320	16.061	21.572	18.796	23.293	28.086
15	0.1330	16.103	19.318	13.592	13.676	23.847
15	0.1340	15.098	16.238	8.956	18.017	19.770
15	0.1350	9.299	7.141	7.600	12.105	17.510
15	0.1360	22.049	20.578	25.879	9.415	20.418

15	0.1370	15.844	14.438	18.793	16.575	13.258
15	0.1380	18.971	11.060	14.576	18.317	22.714
15	0.1390	13.901	13.149	11.989	20.744	27.320
15	0.1400	13.030	15.645	14.815	11.921	10.769
15	0.1410	19.981	8.704	16.204	22.605	13.173
15	0.1420	15.993	13.110	13.512	20.918	25.977
15	0.1430	13.115	12.297	14.481	19.332	24.536
15	0.1440	12.809	10.324	11.807	12.634	6.139
15	0.1450	23.245	11.889	15.886	17.614	12.818
15	0.1460	23.244	11.890	15.886	17.610	12.810
15	0.1470	8.766	13.783	7.764	9.713	10.734
15	0.1480	7.391	11.391	6.472	9.988	6.124
15	0.1490	7.575	6.017	5.332	9.942	6.903
15	0.1500	13.055	6.869	8.237	11.460	9.542
15	0.1510	13.096	6.901	8.281	11.421	9.503
15	0.1520	13.117	10.660	11.388	12.799	26.068
15	0.1530	13.089	10.658	11.388	12.822	26.077
15	0.1540	6.680	6.419	7.531	20.563	20.280
15	0.1550	3.047	3.675	7.035	15.733	11.680
15	0.1560	7.099	4.327	8.173	12.165	16.131
15	0.1570	29.248	25.946	28.422	10.027	13.790
15	0.1580	19.373	16.895	23.872	16.038	17.511
15	0.1590	10.358	6.374	15.570	13.029	18.531
15	0.1600	17.347	16.825	15.587	17.342	23.276
15	0.1610	12.692	7.772	6.534	16.379	25.194
15	0.1620	13.466	13.412	6.834	15.705	17.672
15	0.1630	1.195	2.699	4.841	4.261	9.609
15	0.1640	21.227	26.139	23.307	14.886	14.549
15	0.1650	14.649	16.576	12.908	17.749	21.746
15	0.1660	10.010	13.027	10.911	13.373	21.963
15	0.1670	9.861	10.114	13.450	8.406	11.241
15	0.1680	13.551	12.683	11.681	13.683	20.817
15	0.1690	6.413	9.009	8.170	18.945	11.289
15	0.1700	18.818	18.703	18.945	18.342	21.537
15	0.1710	17.188	13.997	15.348	10.033	12.187
15	0.1720	17.141	14.026	15.170	10.139	12.093
15	0.1730	11.217	12.206	12.298	12.582	20.404
15	0.1740	11.118	6.252	8.150	16.784	19.101
15	0.1750	17.757	14.262	15.856	22.333	16.740
15	0.1760	9.593	11.284	7.664	17.386	13.481
15	0.1770	7.748	8.323	8.968	19.020	13.682
15	0.1780	13.334	15.725	12.923	21.398	17.646
15	0.1790	22.755	15.454	20.954	17.795	20.165
15	0.1800	22.473	21.218	22.020	20.538	27.790
15	0.1810	10.147	11.767	14.539	21.641	25.552
15	0.1820	18.996	17.722	20.266	15.846	24.416
15	0.1830	6.368	6.656	6.834	8.602	7.451
15	0.1840	10.615	12.627	8.819	14.660	14.901
15	0.1850	11.605	9.510	10.462	12.826	13.076
15	0.1860	10.743	9.909	10.354	22.489	13.126

15	0.1870	10.711	9.761	10.401	18.571	18.085
15	0.1880	13.858	11.884	13.734	16.106	13.277
15	0.1890	11.028	11.971	11.535	13.038	21.587
15	0.1900	9.012	7.441	6.185	12.727	14.588
15	0.1910	14.836	8.658	7.187	12.790	16.937
15	0.1920	15.705	18.588	10.425	15.467	8.692
15	0.1930	14.496	11.071	14.409	18.752	12.913
15	0.1940	8.531	8.678	8.841	18.523	12.439
15	0.1950	5.989	6.542	6.326	19.051	10.619
15	0.1960	9.232	11.840	8.949	22.809	19.967
15	0.1970	9.441	14.417	11.238	16.188	20.081
15	0.1980	14.432	15.860	13.560	13.841	11.216
15	0.1990	10.513	10.633	10.342	12.414	18.876
15	0.2000	6.509	9.715	7.847	10.056	11.783
15	0.2010	7.881	12.757	18.156	12.976	16.080
15	0.2020	8.359	8.432	7.413	9.716	9.452
15	0.2030	8.378	8.471	7.415	9.729	9.481
15	0.2040	7.003	5.875	5.621	8.947	20.528
15	0.2050	5.975	6.676	5.072	12.360	16.391
15	0.2060	6.904	10.468	8.613	15.203	15.090
15	0.2070	8.809	11.846	10.104	14.629	10.649
15	0.2080	21.935	16.614	13.814	20.550	18.825
15	0.2090	9.359	9.029	8.220	14.413	17.603
15	0.2100	8.776	13.986	11.894	14.246	9.103
15	0.2110	6.060	8.202	9.670	20.314	16.468
15	0.2120	14.840	7.092	8.352	7.898	10.821
15	0.2130	16.739	12.287	11.173	8.586	8.579
15	0.2140	15.290	18.408	9.860	24.194	11.879
15	0.2150	13.586	9.362	9.585	15.976	16.868
15	0.2160	9.271	15.382	12.210	14.301	16.316
15	0.2170	14.414	13.333	10.366	24.147	21.861
15	0.2180	16.283	13.170	12.298	16.970	16.709
15	0.2190	14.717	20.460	12.963	29.440	9.350
15	0.2200	17.741	12.845	10.985	15.339	12.906
15	0.2210	12.790	7.970	8.688	21.439	16.634
15	0.2220	16.636	18.409	15.238	23.071	20.288
15	0.2230	12.486	18.765	11.490	17.880	9.687
15	0.2240	19.464	23.676	16.925	15.183	11.193
15	0.2250	13.427	22.203	13.156	24.097	10.128
15	0.2260	21.296	28.086	18.247	21.410	11.288
15	0.2270	14.925	14.628	11.025	21.952	9.292
15	0.2280	14.812	9.505	8.212	23.373	9.627
15	0.2290	17.202	20.444	15.680	24.322	13.114
15	0.2300	13.972	21.263	16.641	33.384	12.035
15	0.2310	17.524	18.151	18.837	18.691	12.923
15	0.2320	13.429	17.631	14.480	22.404	14.388
15	0.2330	10.829	12.953	10.124	15.752	18.457
15	0.2340	7.652	6.323	3.082	12.478	15.036
15	0.2350	12.447	16.807	11.680	31.236	10.703
15	0.2360	14.554	22.182	12.404	30.756	13.128

15	0.2370	11.402	18.188	13.202	26.541	8.623
15	0.2380	18.995	16.804	15.539	17.265	5.904
15	0.2390	18.461	11.784	10.725	22.767	14.783
15	0.2400	9.230	11.454	3.422	28.913	18.627
15	0.2410	7.537	12.248	7.572	17.444	18.335
15	0.2420	22.157	17.000	15.384	25.580	20.875
15	0.2430	19.958	19.325	16.764	31.790	13.500
15	0.2440	13.999	12.366	11.126	29.072	19.008
15	0.2450	18.119	19.302	14.487	15.008	10.012
15	0.2460	14.474	10.813	10.194	19.127	27.110
15	0.2470	14.493	10.793	10.185	18.905	26.937
15	0.2480	5.920	11.545	7.910	13.249	19.437
15	0.2490	21.603	16.556	15.174	25.072	24.071
15	0.2500	18.294	11.714	12.665	19.764	16.424
15	0.2510	23.732	14.922	16.071	13.721	15.847
15	0.2520	3.762	3.473	3.080	8.493	12.993
15	0.2530	10.098	14.334	8.349	17.477	15.014
15	0.2540	14.301	15.966	10.572	22.845	19.144
15	0.2550	14.121	9.581	8.580	16.959	13.451
15	0.2560	15.366	13.285	12.947	17.959	20.821
15	0.2570	22.281	14.305	13.419	17.606	11.182
15	0.2580	25.067	16.103	10.745	17.383	25.008
15	0.2590	12.464	16.279	10.553	25.162	23.154
15	0.2600	9.131	10.615	5.403	23.263	17.891
15	0.2610	18.775	17.682	14.326	24.136	25.145
15	0.2620	10.924	18.518	12.192	27.317	14.637
15	0.2630	13.804	13.828	8.734	19.257	13.625
15	0.2640	22.034	19.626	13.773	17.800	12.072
15	0.2650	14.947	15.970	10.345	19.180	12.639
15	0.2660	12.490	24.772	10.336	24.070	14.691
15	0.2670	19.990	18.261	9.300	21.051	15.667
15	0.2680	15.670	17.922	11.539	18.700	13.171
15	0.2690	12.741	13.517	10.681	31.464	28.838
15	0.2700	10.989	15.370	13.910	11.585	26.197
15	0.2710	7.968	9.490	7.114	6.452	8.171
15	0.2720	18.580	10.031	10.929	20.378	28.441
15	0.2730	17.234	10.001	8.870	24.307	22.201
15	0.2740	17.232	10.001	8.874	24.308	22.202
15	0.2750	17.231	10.002	8.877	24.310	22.203
15	0.2760	14.462	9.363	9.611	12.951	16.106
15	0.2770	27.961	21.361	14.907	19.513	14.473
15	0.2780	16.810	10.092	10.173	20.056	13.195
15	0.2790	13.962	17.429	15.606	32.781	21.052
15	0.2800	18.355	10.092	13.482	19.059	27.094
15	0.2810	19.527	13.497	12.723	27.069	20.491
15	0.2820	26.588	23.798	17.735	34.484	23.763
15	0.2830	21.414	15.302	16.312	21.135	25.858
15	0.2840	11.534	6.964	5.265	12.104	18.989
15	0.2850	19.818	24.323	14.672	18.285	17.062
15	0.2860	11.493	18.014	9.762	22.121	15.428

15	0.2870	15.574	20.406	12.009	21.746	15.365
15	0.2880	11.397	16.293	13.027	16.330	14.177
15	0.2890	23.733	23.676	21.825	10.808	16.697
15	0.2900	27.868	22.245	25.288	9.157	11.343
15	0.2910	17.117	18.610	15.296	18.093	10.333
15	0.2920	33.337	28.061	27.333	13.453	26.815
15	0.2930	11.383	11.869	8.025	6.747	12.415
15	0.2940	11.751	19.990	12.697	21.003	12.227
15	0.2950	11.857	22.987	13.486	21.625	14.674
15	0.2960	35.361	34.077	26.704	18.201	15.997
15	0.2970	19.028	23.682	16.095	9.767	5.434
15	0.2980	37.420	45.078	32.617	17.363	9.071
15	0.2990	18.029	28.130	20.243	24.014	11.887
15	0.3000	20.253	22.805	17.842	19.734	14.133
15	0.3010	20.596	26.388	26.313	19.081	9.070
15	0.3020	12.423	12.637	16.888	18.432	14.896
15	0.3030	10.564	19.089	13.574	18.455	11.704
15	0.3040	8.692	13.501	11.278	11.140	9.060
15	0.3050	9.613	15.646	13.019	11.197	8.230
15	0.3060	23.422	24.175	14.578	26.851	20.666
15	0.3070	23.696	16.048	13.817	6.692	8.612
15	0.3080	8.253	9.903	10.068	12.776	8.674
15	0.3090	10.403	7.510	7.554	13.295	13.552
15	0.3100	14.501	12.907	14.185	10.895	7.294
15	0.3110	18.751	18.717	20.578	7.640	6.394
15	0.3120	14.589	15.180	14.026	15.137	9.412
15	0.3130	12.644	15.079	9.493	24.112	12.496
15	0.3140	17.213	17.651	20.824	19.408	9.480
15	0.3150	13.763	16.480	17.116	23.441	16.050
15	0.3160	4.272	10.244	6.315	12.928	6.294
15	0.3170	11.490	12.844	13.604	14.437	9.995
15	0.3180	12.697	21.047	14.303	29.158	11.377
15	0.3190	3.196	4.988	3.976	2.796	3.888
15	0.3200	2.878	4.035	5.033	6.454	12.654
15	0.3210	14.416	15.033	10.455	13.644	17.750
15	0.3220	15.511	18.170	15.897	20.228	17.639
15	0.3230	9.906	19.490	10.802	16.780	11.973
15	0.3240	12.015	9.845	13.869	9.477	16.550
15	0.3250	16.102	26.737	19.403	34.802	17.285
15	0.3260	18.775	25.628	17.320	18.348	19.452
15	0.3270	15.518	21.965	18.795	16.292	17.238
15	0.3280	9.649	18.024	13.667	13.646	10.628
15	0.3290	10.898	19.949	14.818	21.857	14.157
15	0.3300	9.771	16.782	9.804	17.955	10.632
15	0.3310	9.742	8.717	8.521	13.673	8.017
15	0.3320	8.656	6.748	9.658	14.273	7.462
15	0.3330	15.878	7.810	11.172	16.081	5.085
15	0.3340	11.999	13.359	16.015	17.402	12.259
15	0.3350	10.965	9.880	13.897	21.107	13.991
15	0.3360	13.757	4.595	11.345	15.559	10.253

15	0.3370	7.107	11.729	13.676	14.612	3.646
15	0.3380	18.611	20.444	23.631	13.623	11.832
15	0.3390	19.281	13.963	18.168	23.584	21.788
15	0.3400	11.525	7.109	11.705	21.145	19.712
15	0.3410	6.249	5.101	7.185	13.168	7.474
15	0.3420	7.125	11.430	8.582	16.407	9.499
15	0.3430	8.246	13.591	7.924	14.561	10.731
15	0.3440	9.979	12.165	9.214	15.186	14.802
15	0.3450	15.146	16.501	12.794	20.403	8.834
15	0.3460	16.281	23.882	15.189	13.714	7.190
15	0.3470	12.070	17.089	13.336	15.841	19.259
15	0.3480	9.615	15.963	13.354	15.872	10.711
15	0.3490	21.720	22.508	25.584	10.081	8.596
15	0.3500	10.885	14.769	13.233	4.679	10.654
15	0.3510	14.000	14.191	17.583	22.227	15.028
15	0.3520	14.922	21.362	12.926	27.473	17.568
15	0.3530	17.416	19.730	22.771	21.017	15.818
15	0.3540	13.194	18.960	16.118	22.163	20.596
15	0.3550	23.806	30.824	21.890	21.081	15.343
15	0.3560	23.789	30.784	21.840	21.035	15.360
15	0.3570	18.473	28.264	19.942	16.769	16.804
15	0.3580	14.868	12.972	19.292	12.899	14.393
15	0.3590	25.616	26.509	31.754	14.815	23.941
15	0.3600	20.348	29.769	23.987	28.251	25.798
15	0.3610	5.105	6.131	4.124	18.913	6.777
15	0.3620	11.916	9.008	10.516	13.869	9.065
15	0.3630	7.097	5.224	4.935	8.662	5.459
15	0.3640	9.935	9.353	10.491	15.615	13.054
15	0.3650	14.764	28.092	22.526	26.620	9.046
15	0.3660	8.854	22.919	14.293	14.098	8.964
15	0.3670	13.214	14.397	17.492	16.607	14.094
15	0.3680	12.057	19.459	16.904	20.744	15.310
15	0.3690	3.767	3.361	3.936	7.240	7.309
15	0.3700	8.779	4.218	5.304	10.508	7.692
15	0.3710	5.693	9.562	6.655	10.296	6.270
15	0.3720	8.663	8.980	9.356	16.026	4.856
15	0.3730	3.871	9.507	5.276	14.340	5.767
15	0.3740	6.360	12.591	7.187	22.532	10.557
15	0.3750	9.397	13.631	8.849	18.269	9.879
15	0.3760	18.417	12.838	15.041	18.470	9.737
15	0.3770	17.903	22.367	17.001	29.328	15.779
15	0.3780	14.630	18.784	11.062	28.607	9.324
15	0.3790	24.505	26.505	27.381	21.244	6.894
15	0.3800	17.424	24.150	20.488	18.389	15.669
15	0.3810	18.213	23.474	21.452	26.899	23.185
15	0.3820	18.734	21.267	21.586	18.870	13.588
15	0.3830	13.459	20.564	15.667	10.136	6.137
15	0.3840	11.716	24.571	17.051	25.368	10.808
15	0.3850	10.990	16.511	12.549	12.494	10.199
15	0.3860	10.894	16.381	12.419	12.512	10.262

15	0.3870	10.616	11.359	11.296	20.635	13.700
15	0.3880	13.681	9.150	10.811	9.054	4.446
15	0.3890	10.861	7.041	8.101	9.140	12.463
15	0.3900	9.640	11.490	7.750	10.282	13.276
15	0.3910	9.328	18.619	13.448	8.743	2.690
15	0.3920	6.686	13.920	8.384	18.022	12.774
15	0.3930	8.592	15.447	7.283	19.912	20.504
15	0.3940	10.804	18.821	11.936	15.074	19.401
15	0.3950	17.875	19.970	16.555	16.334	11.522
15	0.3960	22.472	24.522	30.382	31.851	16.732
15	0.3970	12.665	27.314	22.167	19.460	11.921
15	0.3980	13.234	25.886	17.958	25.920	15.299
15	0.3990	17.176	28.606	21.278	23.370	10.268
15	0.4000	10.091	10.468	9.632	11.863	12.672
15	0.4010	6.936	11.630	8.749	13.254	8.900
15	0.4020	10.691	24.483	20.158	25.202	13.502
15	0.4030	8.188	9.240	7.286	20.700	14.457
15	0.4040	5.661	8.077	9.726	10.310	13.104
15	0.4050	5.632	6.923	8.496	9.377	8.528
15	0.4060	5.251	12.479	13.337	23.696	13.127
15	0.4070	5.171	14.269	11.883	21.018	9.292
15	0.4080	2.635	8.523	4.688	10.197	4.172
15	0.4090	4.578	2.880	5.044	13.453	5.971
15	0.4100	17.488	20.499	17.222	13.021	11.055
15	0.4110	11.666	21.733	19.960	15.544	14.143
15	0.4120	8.282	10.943	10.303	18.090	19.088
15	0.4130	10.812	7.722	4.930	15.290	16.649
15	0.4140	8.884	4.530	3.377	4.531	15.646
15	0.4150	11.029	15.281	11.696	3.507	14.344
15	0.4160	8.613	14.077	11.699	3.530	7.372
15	0.4170	8.615	14.079	11.705	3.530	7.373
15	0.4180	12.098	15.818	14.892	19.441	17.091
15	0.4190	16.381	19.874	16.516	19.194	14.679
15	0.4200	12.380	14.300	15.247	9.186	8.590
15	0.4210	13.823	17.592	12.125	12.656	9.273
15	0.4220	13.705	18.747	14.711	8.079	10.298
15	0.4230	20.377	20.909	19.490	14.274	17.578
15	0.4240	16.579	21.575	17.127	19.396	19.751
15	0.4250	14.168	15.289	8.072	14.173	19.657
15	0.4260	10.785	16.763	10.530	13.567	16.076
15	0.4270	12.111	19.002	12.883	12.313	16.041
15	0.4280	21.456	20.284	15.966	7.004	17.316
15	0.4290	17.775	20.612	12.754	13.258	15.828
15	0.4300	14.117	17.578	11.336	14.325	11.259
15	0.4310	14.221	17.581	11.342	14.353	11.220
15	0.4320	11.845	11.120	9.787	14.387	14.979
15	0.4330	6.320	5.761	3.831	12.520	8.862
15	0.4340	9.641	13.438	5.241	22.301	11.682
15	0.4350	8.892	8.555	4.279	19.235	13.491
15	0.4360	11.217	14.379	6.740	17.643	18.628

15	0.4370	18.741	14.778	19.224	20.706	15.792
15	0.4380	15.962	12.572	11.082	19.124	17.219
15	0.4390	13.014	10.655	6.771	17.647	19.014
15	0.4400	16.915	15.402	11.229	11.039	19.837
15	0.4410	16.910	15.386	11.217	11.045	19.838
15	0.4420	16.904	15.368	11.204	11.049	19.839
15	0.4430	16.898	15.349	11.191	11.053	19.840
15	0.4440	16.891	15.328	11.177	11.055	19.841
15	0.4450	10.405	12.049	7.343	9.169	15.975
15	0.4460	16.858	22.356	14.702	17.179	16.173
15	0.4470	29.935	28.530	24.911	16.674	12.767
15	0.4480	8.066	13.809	8.535	19.584	9.708
15	0.4490	13.992	19.807	12.695	15.118	11.480
15	0.4500	29.032	17.418	19.930	18.342	8.913
15	0.4510	14.202	12.403	7.445	9.462	12.532
15	0.4520	18.263	19.497	9.315	15.375	14.107
15	0.4530	7.323	8.459	5.592	15.339	25.805
15	0.4540	11.545	16.340	7.314	23.545	24.028
15	0.4550	13.737	24.343	14.618	20.374	17.792
15	0.4560	11.062	16.401	12.445	10.384	18.326
15	0.4570	16.672	20.695	17.952	19.222	16.862
15	0.4580	22.090	23.816	25.245	18.935	19.321
15	0.4590	12.075	19.100	20.331	9.796	15.582
15	0.4600	12.124	19.100	20.371	9.765	15.608
15	0.4610	4.600	15.064	6.524	14.632	11.855
15	0.4620	13.439	8.787	10.698	3.991	11.731
15	0.4630	13.528	8.960	10.803	4.073	11.620
15	0.4640	17.121	18.111	14.633	13.908	9.112
15	0.4650	11.703	14.409	12.921	9.320	10.964
15	0.4660	22.086	26.959	18.586	15.196	10.587
15	0.4670	19.015	27.807	19.353	12.764	11.787
15	0.4680	7.019	13.914	10.569	20.867	18.418
15	0.4690	7.255	11.692	7.281	23.646	20.433
15	0.4700	12.938	9.976	8.977	13.830	12.259
15	0.4710	18.885	15.662	15.073	9.741	9.337
15	0.4720	14.305	17.256	13.394	21.474	16.579
15	0.4730	16.152	10.833	10.679	18.707	11.138
15	0.4740	15.236	17.212	14.645	11.475	8.697
15	0.4750	10.381	10.361	9.964	18.940	14.926
15	0.4760	16.975	12.524	12.440	26.531	20.372
15	0.4770	14.820	16.395	14.707	30.040	18.423
15	0.4780	14.862	16.385	14.734	29.961	18.574
15	0.4790	10.294	17.247	12.247	21.505	11.999
15	0.4800	10.322	17.266	12.280	21.505	11.990
15	0.4810	3.962	2.933	8.877	10.196	20.730
15	0.4820	23.138	24.728	22.276	14.767	9.771
15	0.4830	10.671	15.948	7.611	14.258	23.364
15	0.4840	14.805	9.354	9.788	11.694	11.346
15	0.4850	18.746	8.529	14.050	11.715	8.996
15	0.4860	16.427	16.139	20.748	9.614	11.197

15	0.4870	8.232	11.172	12.343	21.738	14.831
15	0.4880	10.340	15.930	17.600	16.841	4.875
15	0.4890	25.453	18.325	23.565	16.476	10.561
15	0.4900	25.454	18.326	23.569	16.479	10.562
15	0.4910	6.320	6.020	6.053	0.601	5.256
15	0.4920	18.397	15.311	16.038	5.548	8.140
15	0.4930	39.406	21.252	20.218	15.130	10.755
15	0.4940	25.027	19.604	18.284	11.075	9.817
15	0.4950	26.887	11.033	8.752	9.372	8.128
15	0.4960	6.015	6.907	3.548	5.993	2.109
15	0.4970	23.429	18.402	20.851	16.357	4.942
15	0.4980	20.811	21.128	19.880	21.839	8.260
15	0.4990	21.710	25.541	27.868	8.134	6.592
15	0.5000	5.479	6.413	5.379	8.184	5.452
15	0.5010	43.096	34.359	34.418	5.752	5.053
15	0.5020	13.728	16.674	18.263	7.827	7.843
15	0.5030	37.730	37.140	28.497	15.886	9.564
15	0.5040	19.504	17.341	13.142	12.422	9.579
15	0.5050	13.446	23.391	10.179	15.051	11.774
15	0.5060	14.373	24.823	16.637	10.328	13.670
15	0.5070	9.464	15.674	13.979	6.187	10.893
15	0.5080	14.438	17.231	18.022	7.756	13.987
15	0.5090	12.792	16.311	14.723	11.260	14.426
15	0.5100	16.370	15.090	15.037	14.366	18.879
15	0.5110	21.775	24.828	19.119	18.052	8.749
15	0.5120	12.713	24.790	21.768	13.930	18.076
15	0.5130	6.244	16.190	14.856	10.836	16.723
15	0.5140	15.777	16.387	8.809	6.957	8.884
15	0.5150	6.581	12.996	7.034	9.759	5.216
15	0.5160	19.028	13.823	17.624	7.182	10.002
15	0.5170	12.709	16.645	16.128	13.506	17.009
15	0.5180	11.876	16.372	12.464	11.406	7.478
15	0.5190	14.057	7.932	10.717	7.035	5.869
15	0.5200	14.793	15.523	16.092	16.359	14.306
15	0.5210	12.055	17.933	14.834	15.918	10.968
15	0.5220	28.415	27.018	22.049	7.992	6.010
15	0.5230	22.886	21.509	20.835	13.811	12.534
15	0.5240	7.973	9.133	12.064	12.835	14.162
15	0.5250	12.131	11.785	7.654	7.045	5.701
15	0.5260	14.410	18.052	16.469	8.640	6.888
15	0.5270	14.240	12.986	14.944	11.854	13.914
15	0.5280	17.356	16.307	14.711	21.055	8.571
15	0.5290	14.557	6.202	12.898	8.648	13.423
15	0.5300	15.842	11.326	12.209	10.624	11.702
15	0.5310	13.334	9.636	10.663	15.801	15.122
15	0.5320	14.963	11.705	10.919	20.827	18.565
15	0.5330	10.866	9.732	11.432	16.911	13.909
15	0.5340	24.801	25.897	18.879	15.413	15.174
15	0.5350	26.314	21.631	20.483	14.141	9.391
15	0.5360	11.236	14.026	12.388	14.103	7.670

15	0.5370	15.726	18.944	13.159	14.188	9.873
15	0.5380	35.311	27.550	29.385	18.769	19.888
15	0.5390	16.531	17.726	19.071	16.880	19.518
15	0.5400	18.412	12.463	12.649	12.086	15.173
15	0.5410	22.055	19.617	22.024	14.397	24.491
15	0.5420	30.969	21.267	21.726	17.218	25.008
15	0.5430	28.255	23.191	28.862	19.818	18.837
15	0.5440	53.194	40.958	46.485	18.795	17.816
15	0.5450	25.972	20.488	19.754	11.204	10.635
15	0.5460	11.538	5.654	12.132	8.283	17.936
15	0.5470	19.606	9.103	17.873	9.219	17.239
15	0.5480	43.772	30.622	35.642	28.229	21.640
15	0.5490	32.766	28.556	31.701	25.482	13.045
15	0.5500	24.032	12.593	21.484	16.694	12.507
15	0.5510	24.193	23.574	28.345	18.082	26.698
15	0.5520	29.402	21.624	31.068	20.275	13.884
15	0.5530	22.228	14.039	23.962	13.309	7.138
15	0.5540	9.020	7.237	10.104	13.816	9.471
15	0.5550	13.243	9.670	13.628	15.809	7.714
15	0.5560	16.560	11.377	15.157	18.639	15.278
15	0.5570	33.401	24.760	26.933	30.025	22.505
15	0.5580	24.391	15.791	21.267	22.680	23.029
15	0.5590	8.759	9.656	11.390	19.493	16.738
15	0.5600	15.108	17.643	19.118	19.225	24.520
15	0.5610	35.140	25.917	31.746	19.541	30.545
15	0.5620	24.533	17.515	23.914	27.641	29.728
15	0.5630	23.767	16.237	14.592	6.486	26.138
15	0.5640	23.446	17.434	15.641	19.205	30.776
15	0.5650	35.918	26.702	32.136	22.015	22.248
15	0.5660	25.843	21.817	30.721	12.968	16.341
15	0.5670	23.660	16.829	23.575	12.473	16.466
15	0.5680	33.551	22.973	33.685	8.950	14.987
15	0.5690	34.483	21.113	25.081	5.936	24.748
15	0.5700	20.007	21.365	18.097	15.165	13.756
15	0.5710	20.163	16.259	16.707	16.352	20.277
15	0.5720	28.787	17.978	28.308	19.138	26.218
15	0.5730	20.135	16.795	20.159	10.107	14.630
15	0.5740	30.519	29.721	34.387	28.758	24.330
15	0.5750	30.108	28.677	32.355	33.430	20.474
15	0.5760	31.820	23.075	20.959	24.277	15.288
15	0.5770	31.944	25.097	22.147	10.159	14.867
15	0.5780	48.303	23.138	33.188	23.421	18.463
15	0.5790	39.823	27.346	30.624	22.148	19.159
15	0.5800	34.783	21.347	29.263	24.181	27.263
15	0.5810	39.839	26.786	38.060	10.504	19.574
15	0.5820	34.421	15.238	24.967	14.648	13.636
15	0.5830	38.704	19.028	27.483	8.672	19.142
15	0.5840	13.742	12.120	10.503	21.138	20.085
15	0.5850	23.623	15.351	15.465	20.901	9.449
15	0.5860	22.870	15.777	16.525	26.330	17.116

15	0.5870	12.829	15.643	13.948	18.901	21.903
15	0.5880	19.036	21.758	21.521	5.747	5.427
15	0.5890	32.290	20.448	26.391	1.786	6.241
15	0.5900	17.198	12.035	15.157	14.589	19.271
15	0.5910	15.950	17.179	14.366	18.691	16.698
15	0.5920	15.147	16.734	14.789	12.027	8.618
15	0.5930	7.109	15.898	11.113	15.555	9.438
15	0.5940	7.584	9.615	9.203	14.559	12.637
15	0.5950	4.233	8.075	7.670	8.039	17.335
15	0.5960	14.250	16.180	17.288	24.501	9.744
15	0.5970	13.800	13.633	9.633	11.670	11.060
15	0.5980	17.889	16.888	16.079	10.394	16.034
15	0.5990	23.012	23.475	20.832	29.621	17.756
15	0.6000	12.144	8.345	9.817	12.469	10.880
15	0.6010	14.308	14.191	14.130	15.441	12.721
15	0.6020	21.298	22.079	19.985	19.320	14.436
15	0.6030	26.104	27.861	25.721	19.605	13.209
15	0.6040	13.147	9.844	11.687	4.677	10.306
15	0.6050	5.838	8.251	4.503	15.756	13.343
15	0.6060	16.286	18.801	18.141	16.928	10.773
15	0.6070	10.594	11.355	6.988	25.371	13.271
15	0.6080	24.932	31.901	25.682	27.099	11.526
15	0.6090	23.005	23.262	18.119	18.889	20.016
15	0.6100	26.979	31.811	24.247	22.069	18.320
15	0.6110	21.806	26.606	25.000	15.348	17.514
15	0.6120	17.149	11.901	18.011	16.483	6.751
15	0.6130	16.153	7.100	8.526	10.639	7.379
15	0.6140	22.717	7.998	13.039	12.052	16.829
15	0.6150	9.815	3.947	8.760	9.388	14.566
15	0.6160	9.759	4.221	9.698	14.554	14.871
15	0.6170	28.878	17.345	25.138	28.347	16.276
15	0.6180	41.568	24.439	38.322	24.494	7.368
15	0.6190	16.361	15.333	17.096	14.776	13.266
15	0.6200	28.844	11.692	18.263	21.407	20.265
15	0.6210	12.955	20.771	14.918	25.157	16.750
15	0.6220	33.662	22.292	28.082	18.458	8.805
15	0.6230	11.296	15.151	7.950	25.380	9.227
15	0.6240	14.205	12.583	13.507	16.686	7.101
15	0.6250	28.318	16.787	23.248	21.866	18.396
15	0.6260	26.513	19.523	20.185	12.405	18.208
15	0.6270	24.019	23.717	16.838	19.757	14.077
15	0.6280	14.741	17.134	10.200	10.678	8.789
15	0.6290	23.366	17.488	16.557	11.609	19.460
15	0.6300	8.995	14.547	11.023	9.970	13.266
15	0.6310	11.829	17.523	11.635	10.090	8.413
15	0.6320	22.760	22.318	19.622	15.001	19.211
15	0.6330	20.935	15.116	15.709	2.290	3.779
15	0.6340	13.128	15.041	14.504	8.658	8.490
15	0.6350	15.129	16.770	18.999	12.939	4.333
15	0.6360	15.322	23.183	15.205	12.046	3.871

15	0.6370	18.989	18.529	14.346	22.439	14.581
15	0.6380	21.861	15.405	20.370	4.104	6.950
15	0.6390	17.278	12.598	15.786	7.921	13.530
15	0.6400	16.099	15.072	12.908	16.719	21.531
15	0.6410	11.245	18.566	17.718	18.927	9.309
15	0.6420	13.442	15.116	10.462	20.705	19.617
15	0.6430	14.963	12.190	10.100	16.622	16.584
15	0.6440	23.970	29.879	25.326	15.505	13.428
15	0.6450	26.492	19.114	24.911	13.286	11.266
15	0.6460	11.919	23.160	23.860	15.600	11.732
15	0.6470	9.288	16.769	8.574	12.224	17.188
15	0.6480	10.189	13.701	12.256	10.594	10.863
15	0.6490	9.499	7.882	9.568	10.321	13.671
15	0.6500	12.775	7.229	10.165	10.697	15.081
15	0.6510	22.042	15.038	13.614	14.419	7.794
15	0.6520	6.501	4.311	5.332	11.538	6.833
15	0.6530	5.951	8.718	7.214	13.276	8.591
15	0.6540	15.945	10.679	14.369	12.250	5.494
15	0.6550	15.932	10.583	14.288	12.234	5.495
15	0.6560	7.578	7.329	7.902	6.866	4.729
15	0.6570	6.122	5.431	4.744	8.648	8.416
15	0.6580	17.429	18.204	13.963	8.206	9.582
15	0.6590	4.303	7.696	6.674	11.124	5.445
15	0.6600	3.716	7.206	5.212	15.717	17.939
15	0.6610	10.535	9.570	9.074	12.470	18.186
15	0.6620	22.077	10.156	16.125	8.899	14.042
15	0.6630	18.659	14.166	17.040	23.793	18.581
15	0.6640	13.763	5.215	7.538	10.555	16.235
15	0.6650	9.631	5.739	9.116	6.553	11.008
15	0.6660	16.802	13.535	15.733	7.648	15.186
15	0.6670	21.930	13.266	22.100	11.666	12.174
15	0.6680	12.851	13.285	13.554	9.568	11.851
15	0.6690	16.971	13.243	21.015	17.353	10.723
15	0.6700	23.685	19.823	26.139	11.022	13.098
15	0.6710	7.734	4.655	6.427	3.271	8.624
15	0.6720	8.191	4.778	6.914	7.990	9.127
15	0.6730	40.594	36.930	36.486	16.126	18.363
15	0.6740	25.492	24.356	20.780	12.263	16.859
15	0.6750	20.368	29.019	25.367	15.088	10.451
15	0.6760	10.274	15.458	14.595	17.272	9.136
15	0.6770	7.344	12.409	10.531	6.518	17.633
15	0.6780	6.710	8.644	4.309	12.082	9.938
15	0.6790	18.932	19.577	19.028	11.297	8.291
15	0.6800	17.104	24.156	16.296	27.168	20.501
15	0.6810	14.359	18.232	20.112	19.029	25.122
15	0.6820	21.309	26.539	25.926	15.894	21.999
15	0.6830	23.918	28.036	21.980	13.766	4.771
15	0.6840	16.362	14.906	19.080	7.721	11.614
15	0.6850	15.143	16.833	18.156	15.579	13.389
15	0.6860	26.869	26.364	28.409	13.143	15.109

15	0.6870	23.043	27.081	23.097	11.128	13.242
15	0.6880	13.563	14.366	12.842	13.813	23.571
15	0.6890	12.014	11.613	10.682	7.302	6.797
15	0.6900	26.264	24.517	25.823	12.159	18.835
15	0.6910	20.142	14.638	20.446	11.476	5.960
15	0.6920	22.098	14.951	21.051	12.228	16.587
15	0.6930	16.130	16.622	18.865	5.748	11.631
15	0.6940	13.630	8.695	9.321	15.589	18.829
15	0.6950	34.264	35.753	31.349	13.234	8.729
15	0.6960	16.428	21.596	13.350	18.954	14.674
15	0.6970	21.643	21.142	18.854	12.966	16.566
15	0.6980	26.855	18.132	15.188	19.635	21.067
15	0.6990	17.159	12.589	14.730	9.151	29.917
15	0.7000	18.467	12.119	11.196	16.307	27.285
15	0.7010	10.337	8.521	12.002	26.414	15.884
15	0.7020	11.144	8.888	11.066	23.013	11.829
15	0.7030	11.294	10.174	12.137	10.682	5.214
15	0.7040	21.996	17.455	14.918	11.054	7.439
15	0.7050	18.273	14.522	19.744	20.435	8.509
15	0.7060	17.584	13.128	19.472	18.304	11.481
15	0.7070	16.824	16.867	23.929	19.264	24.873
15	0.7080	15.076	12.460	15.278	17.656	22.289
15	0.7090	13.999	10.211	10.752	20.663	14.303
15	0.7100	18.123	12.799	16.111	18.611	10.345
15	0.7110	7.095	6.076	5.089	9.909	9.752
15	0.7120	16.424	18.469	14.832	20.936	12.180
15	0.7130	13.969	17.113	16.931	7.103	8.306
15	0.7140	28.401	21.166	25.270	5.937	11.328
15	0.7150	14.855	16.594	13.254	17.389	13.338
15	0.7160	18.352	19.433	20.575	21.895	17.202
15	0.7170	27.743	18.188	26.844	18.381	13.403
15	0.7180	12.445	20.201	16.148	13.780	10.577
15	0.7190	15.884	16.653	16.773	21.620	16.982
15	0.7200	13.788	20.244	20.408	17.594	11.033
15	0.7210	18.833	17.040	22.184	14.825	7.002
15	0.7220	20.970	20.641	24.402	15.654	8.562
15	0.7230	12.831	13.040	12.042	13.152	8.797
15	0.7240	10.729	9.458	6.673	14.319	11.632
15	0.7250	11.115	11.724	11.719	23.504	19.869
15	0.7260	17.571	16.688	19.459	26.585	20.455
15	0.7270	14.621	19.835	19.370	19.102	17.654
15	0.7280	21.295	14.742	20.873	4.761	4.215
15	0.7290	7.493	11.686	5.760	20.799	12.369
15	0.7300	7.743	16.312	13.569	14.220	12.105
15	0.7310	12.780	18.004	18.100	11.234	10.147
15	0.7320	15.439	24.571	19.105	11.177	11.946
15	0.7330	8.505	14.533	8.867	19.494	9.085
15	0.7340	13.745	17.973	13.477	18.781	16.426
15	0.7350	10.386	8.567	6.777	10.970	11.523
15	0.7360	10.093	10.058	11.713	13.308	16.039

15	0.7370	16.911	17.009	17.243	7.942	13.264
15	0.7380	10.544	16.174	14.820	14.544	18.182
15	0.7390	21.478	21.333	20.958	21.617	14.636
15	0.7400	26.518	28.254	28.224	21.686	11.739
15	0.7410	21.449	27.370	18.441	27.396	14.398
15	0.7420	29.229	31.499	27.072	27.354	26.180
15	0.7430	19.741	29.371	20.919	15.815	14.719
15	0.7440	21.600	37.177	20.598	38.147	22.868
15	0.7450	23.187	31.868	26.506	27.077	10.637
15	0.7460	28.534	39.880	32.181	21.616	7.625
15	0.7470	20.704	24.641	28.652	29.990	20.881
15	0.7480	20.457	24.284	28.606	29.717	20.861
15	0.7490	32.311	38.273	35.375	27.378	19.016
15	0.7500	25.624	20.163	21.167	32.990	32.597
15	0.7510	25.755	19.897	21.438	32.677	32.737
15	0.7520	13.088	12.603	11.191	43.342	20.619
15	0.7530	18.894	14.393	10.754	29.923	12.488
15	0.7540	18.851	14.373	10.688	29.605	12.569
15	0.7550	24.100	14.115	14.062	32.389	19.711
15	0.7560	20.371	18.148	20.525	20.721	14.373
15	0.7570	16.523	15.442	16.059	22.650	9.731
15	0.7580	15.235	13.285	15.601	16.496	16.857
15	0.7590	12.742	20.445	13.423	28.398	15.563
15	0.7600	5.279	15.706	9.255	31.161	23.099
15	0.7610	13.014	18.542	10.303	29.971	28.671
15	0.7620	8.390	7.794	10.006	15.981	12.044
15	0.7630	7.796	9.640	7.298	21.494	10.083
15	0.7640	7.732	9.554	7.191	21.063	9.922
15	0.7650	8.026	4.099	7.006	10.921	13.768
15	0.7660	8.454	6.957	6.064	7.317	13.109
15	0.7670	11.727	18.155	12.478	21.710	15.672
15	0.7680	15.617	16.827	19.090	16.665	18.749
15	0.7690	12.388	10.554	10.880	13.784	10.623
15	0.7700	36.788	32.382	31.634	14.033	17.333
15	0.7710	17.781	15.009	18.867	12.820	11.985
15	0.7720	18.282	13.620	11.935	16.499	14.258
15	0.7730	19.007	11.775	10.409	16.033	27.834
15	0.7740	25.899	19.564	13.802	15.952	24.728
15	0.7750	19.033	24.020	16.521	19.716	12.309
15	0.7760	10.191	13.505	17.933	10.257	13.210
15	0.7770	12.861	14.697	11.326	10.163	22.095
15	0.7780	13.273	20.056	14.357	11.046	22.087
15	0.7790	24.018	26.266	27.699	12.647	24.693
15	0.7800	12.914	14.708	17.805	12.988	17.739
15	0.7810	16.833	23.963	21.876	12.742	13.773
15	0.7820	21.171	33.165	27.234	13.297	9.025
15	0.7830	21.459	18.206	20.071	15.118	11.133
15	0.7840	9.330	11.142	12.121	9.796	7.526
15	0.7850	8.645	9.144	11.843	9.965	19.330
15	0.7860	17.997	11.657	15.374	3.338	11.179

15	0.7870	11.187	12.128	9.403	6.740	12.899
15	0.7880	14.564	16.036	12.743	7.870	11.002
15	0.7890	12.541	15.804	13.932	18.733	37.777
15	0.7900	0.000	0.000	0.000	0.000	0.000
16	0.0009	0.000	0.000	0.000	0.000	0.000
16	0.0019	19.504	16.946	20.450	9.001	20.320
16	0.0029	17.884	19.729	22.523	14.893	15.311
16	0.0039	21.608	29.626	29.680	14.942	20.522
16	0.0049	12.421	15.977	11.022	16.333	30.607
16	0.0059	25.159	24.481	23.591	6.104	29.021
16	0.0069	27.377	26.105	20.258	20.119	23.667
16	0.0079	30.240	29.873	31.015	24.209	21.030
16	0.0089	38.247	20.190	33.921	21.306	33.223
16	0.0099	21.306	16.692	21.688	13.910	25.227
16	0.0109	31.009	24.857	33.603	5.599	9.792
16	0.0119	33.878	27.143	26.592	17.047	32.033
16	0.0129	27.693	30.005	32.688	20.289	9.494
16	0.0139	30.846	32.705	33.796	8.542	12.658
16	0.0149	15.729	18.872	15.017	13.009	34.224
16	0.0159	15.882	20.137	20.662	22.641	38.727
16	0.0169	17.840	9.773	10.448	15.584	34.520
16	0.0179	15.808	10.183	18.144	20.038	31.613
16	0.0189	14.462	14.623	16.173	7.620	20.359
16	0.0199	16.194	18.522	17.829	17.274	24.201
16	0.0209	24.333	28.446	29.627	14.572	30.580
16	0.0219	27.009	38.852	33.020	24.676	15.851
16	0.0229	4.722	8.700	11.697	12.693	13.764
16	0.0239	8.332	18.577	13.517	14.429	13.885
16	0.0249	9.374	12.203	8.443	14.131	11.940
16	0.0259	13.484	14.604	11.178	11.330	18.778
16	0.0269	22.545	23.982	32.301	7.039	14.439
16	0.0279	24.166	29.588	31.596	11.133	12.814
16	0.0289	18.916	24.371	20.519	10.462	24.628
16	0.0299	19.151	20.137	24.843	24.180	23.648
16	0.0309	19.918	19.229	27.213	18.294	20.176
16	0.0319	17.464	14.339	15.921	14.127	16.777
16	0.0329	24.421	16.512	12.278	12.405	12.060
16	0.0339	17.004	19.963	15.139	11.231	10.587
16	0.0349	16.830	12.399	18.427	11.705	22.057
16	0.0359	12.199	16.916	14.925	17.849	13.741
16	0.0369	9.390	17.794	12.180	24.888	20.839
16	0.0379	16.274	19.464	20.625	19.547	24.407
16	0.0389	13.982	19.988	18.608	20.079	21.848
16	0.0399	12.077	9.875	12.436	21.070	23.466
16	0.0409	17.447	13.387	18.894	27.796	27.434
16	0.0419	9.341	19.572	10.973	24.914	17.901
16	0.0429	19.244	11.170	15.782	14.752	19.305
16	0.0439	19.192	11.094	15.853	14.668	19.466
16	0.0449	24.849	21.179	17.718	21.747	21.651
16	0.0459	8.818	13.704	9.847	21.670	16.229

16	0.0469	14.242	13.653	16.533	13.634	12.924
16	0.0479	11.986	14.219	10.911	17.061	14.136
16	0.0489	10.944	18.334	15.558	18.226	20.501
16	0.0499	14.422	20.816	17.689	20.798	15.917
16	0.0509	14.929	20.484	17.665	20.637	15.745
16	0.0519	15.807	15.157	17.013	24.757	22.128
16	0.0529	10.698	11.991	11.746	12.869	10.275
16	0.0539	8.562	11.070	15.044	5.906	5.499
16	0.0549	14.428	15.935	13.573	23.498	18.337
16	0.0559	15.019	12.679	14.985	19.403	13.631
16	0.0569	11.123	11.899	10.215	23.717	9.849
16	0.0579	17.016	14.179	16.508	23.115	11.969
16	0.0589	11.790	9.368	8.632	18.868	14.767
16	0.0599	11.284	13.186	11.906	13.507	13.300
16	0.0609	22.282	22.633	17.378	15.219	16.446
16	0.0619	23.168	23.259	19.972	12.857	15.946
16	0.0629	16.969	17.757	15.689	12.603	12.822
16	0.0639	11.431	11.970	12.464	21.286	21.796
16	0.0649	16.640	17.349	17.136	18.072	11.145
16	0.0659	21.897	27.030	21.324	10.880	24.050
16	0.0669	11.482	10.369	11.862	9.962	10.478
16	0.0679	19.715	10.712	17.472	12.698	11.664
16	0.0689	9.895	8.434	12.087	10.057	11.275
16	0.0699	16.861	13.567	10.155	10.769	17.812
16	0.0709	10.073	13.901	6.791	15.734	17.984
16	0.0719	9.554	11.928	6.674	26.170	13.323
16	0.0729	19.570	20.763	18.269	26.550	17.797
16	0.0739	20.868	21.434	18.537	21.348	12.745
16	0.0749	31.163	17.020	26.223	17.485	9.507
16	0.0759	21.858	16.115	15.844	13.015	9.848
16	0.0769	20.858	25.246	14.496	15.015	7.207
16	0.0779	17.918	16.644	15.660	23.089	14.255
16	0.0789	17.538	17.635	16.394	22.965	17.103
16	0.0799	13.282	9.335	16.293	19.809	5.402
16	0.0809	10.679	8.373	11.643	9.929	16.539
16	0.0819	8.958	8.233	11.716	9.086	17.220
16	0.0829	12.537	12.272	13.555	28.566	22.991
16	0.0839	18.440	18.905	15.413	20.313	17.007
16	0.0849	29.382	21.009	20.904	21.852	19.481
16	0.0859	20.273	21.733	19.612	23.329	15.581
16	0.0869	17.381	20.962	16.777	18.515	15.927
16	0.0879	10.455	13.502	20.071	11.096	18.471
16	0.0889	18.941	21.341	17.267	17.294	33.937
16	0.0899	10.294	9.367	13.613	17.016	22.001
16	0.0909	13.529	11.865	17.078	17.762	15.381
16	0.0919	24.238	22.167	29.663	18.100	27.053
16	0.0929	18.234	9.663	13.898	19.945	18.142
16	0.0939	24.089	25.456	26.039	24.341	29.053
16	0.0949	15.639	19.831	16.844	14.776	17.153
16	0.0959	16.164	15.361	12.009	15.057	17.775

16	0.0969	14.753	17.069	16.030	18.571	11.348
16	0.0979	20.888	17.697	25.904	12.621	15.591
16	0.0989	9.531	9.203	14.050	8.044	9.637
16	0.0999	6.687	8.064	7.580	12.104	10.525
16	0.1009	12.190	11.646	16.551	8.030	18.111
16	0.1019	14.814	17.039	21.975	25.403	13.630
16	0.1029	19.918	13.270	20.324	21.136	13.962
16	0.1039	14.991	18.726	20.055	16.236	16.703
16	0.1049	15.433	26.992	25.974	20.196	9.771
16	0.1059	13.239	18.504	12.409	15.316	12.840
16	0.1069	13.619	18.704	12.686	15.749	12.668
16	0.1079	18.231	16.497	18.790	8.603	15.191
16	0.1089	15.986	18.538	16.530	20.421	25.172
16	0.1099	13.781	21.637	13.987	24.235	14.062
16	0.1109	21.754	29.507	24.915	30.482	18.022
16	0.1119	22.284	29.676	28.497	13.944	14.893
16	0.1129	20.632	23.277	18.653	31.255	13.601
16	0.1139	15.807	31.627	15.362	31.112	9.229
16	0.1149	31.917	33.285	27.371	28.716	23.954
16	0.1159	22.652	31.521	18.369	17.097	13.375
16	0.1169	19.314	24.646	19.429	23.001	11.483
16	0.1179	23.936	24.500	26.467	18.776	9.991
16	0.1189	8.738	17.935	11.100	28.966	4.797
16	0.1199	25.518	26.246	16.516	29.325	7.138
16	0.1209	16.759	20.955	20.806	10.535	10.542
16	0.1219	20.838	25.105	16.897	8.857	9.648
16	0.1229	19.475	12.988	14.224	14.233	5.957
16	0.1239	5.068	6.073	4.980	22.334	7.257
16	0.1249	33.516	31.196	28.279	16.181	11.358
16	0.1259	13.880	11.873	11.881	8.680	9.697
16	0.1269	11.898	15.023	11.356	10.854	13.986
16	0.1279	12.131	14.687	9.398	14.058	13.247
16	0.1289	18.204	22.724	19.212	14.910	22.737
16	0.1299	32.304	24.406	30.189	16.023	11.512
16	0.1309	20.374	20.208	24.147	18.424	10.487
16	0.1319	19.374	13.885	17.651	9.867	14.152
16	0.1329	12.156	21.845	15.085	12.740	20.136
16	0.1339	14.627	17.325	17.234	16.905	6.916
16	0.1349	14.685	17.498	17.322	17.046	7.003
16	0.1359	17.313	22.303	18.193	22.270	13.076
16	0.1369	10.959	10.999	11.288	14.932	9.463
16	0.1379	7.037	12.523	6.742	25.998	13.910
16	0.1389	12.178	16.984	14.929	20.080	16.236
16	0.1399	12.738	6.919	8.994	17.020	6.229
16	0.1409	11.613	6.652	8.631	19.708	7.062
16	0.1419	18.069	16.498	16.312	17.585	13.122
16	0.1429	33.924	22.061	27.231	24.160	13.246
16	0.1439	19.798	13.443	13.773	16.251	17.851
16	0.1449	17.332	16.260	17.125	14.322	16.340
16	0.1459	12.723	18.118	14.463	17.696	11.194

16	0.1469	10.459	20.436	13.213	17.832	13.497
16	0.1479	21.185	23.678	21.411	18.065	19.080
16	0.1489	9.586	19.307	12.591	14.744	11.101
16	0.1499	30.295	20.331	14.459	18.492	16.440
16	0.1509	33.002	18.261	14.923	10.588	14.671
16	0.1519	14.378	15.080	12.195	12.242	22.203
16	0.1529	14.977	9.790	12.685	10.960	17.419
16	0.1539	11.436	17.028	14.008	14.552	20.480
16	0.1549	17.559	26.746	19.808	29.240	21.283
16	0.1559	23.355	27.680	24.615	10.555	8.537
16	0.1569	14.151	13.868	14.164	21.131	14.880
16	0.1579	26.349	28.625	30.328	13.841	6.913
16	0.1589	27.510	29.823	29.211	12.062	7.831
16	0.1599	13.727	16.770	13.754	17.281	10.693
16	0.1609	23.528	23.055	19.027	24.831	12.374
16	0.1619	12.013	12.090	8.946	13.244	13.861
16	0.1629	23.642	12.329	20.330	14.527	15.641
16	0.1639	26.010	23.160	23.275	17.789	14.896
16	0.1649	17.095	28.528	17.791	15.980	17.765
16	0.1659	20.143	9.708	14.272	16.368	19.982
16	0.1669	11.143	6.863	10.154	11.396	13.686
16	0.1679	21.855	17.313	18.631	16.446	10.289
16	0.1689	23.029	16.443	21.676	12.837	8.959
16	0.1699	26.855	14.967	14.273	19.021	11.060
16	0.1709	24.931	17.763	19.001	21.342	22.374
16	0.1719	25.758	28.483	20.613	12.365	11.078
16	0.1729	21.991	16.042	16.162	23.477	20.497
16	0.1739	25.058	19.398	17.012	14.474	15.572
16	0.1749	28.070	17.497	21.238	9.257	11.221
16	0.1759	24.767	21.214	23.089	13.403	18.542
16	0.1769	12.083	15.485	15.333	16.269	13.294
16	0.1779	15.469	13.488	13.975	12.623	10.163
16	0.1789	7.495	12.796	8.338	16.807	7.198
16	0.1799	12.693	13.872	10.251	16.393	8.123
16	0.1809	43.158	30.843	31.268	16.800	10.756
16	0.1819	17.329	17.395	20.329	17.274	11.076
16	0.1829	29.000	28.869	21.940	22.454	11.779
16	0.1839	15.264	18.627	12.334	13.924	11.165
16	0.1849	28.880	25.716	20.682	13.038	7.415
16	0.1859	38.171	30.939	22.318	21.275	20.697
16	0.1869	14.514	17.459	12.671	16.404	10.673
16	0.1879	14.483	17.383	12.496	16.234	10.859
16	0.1889	14.590	15.069	14.848	12.488	9.517
16	0.1899	9.294	20.483	12.938	12.521	6.954
16	0.1909	17.853	15.831	14.521	7.067	10.960
16	0.1919	15.297	11.941	10.787	11.760	14.092
16	0.1929	16.604	7.367	12.171	21.953	20.782
16	0.1939	19.162	18.293	19.238	18.213	10.128
16	0.1949	20.119	27.832	18.947	16.822	5.046
16	0.1959	13.061	21.497	15.632	18.379	10.833

16	0.1969	17.073	15.698	16.244	16.548	9.959
16	0.1979	14.963	10.708	10.937	14.312	10.037
16	0.1989	20.347	10.446	14.179	19.201	8.500
16	0.1999	22.622	17.057	16.134	10.855	8.650
16	0.2009	13.782	18.115	16.141	13.515	5.451
16	0.2019	16.660	17.028	15.248	15.449	8.094
16	0.2029	15.514	20.967	15.323	25.555	13.357
16	0.2039	8.744	16.042	11.983	26.199	10.291
16	0.2049	15.633	13.293	15.938	15.272	10.581
16	0.2059	11.347	21.129	13.956	21.812	10.315
16	0.2069	11.780	19.156	13.071	21.989	9.885
16	0.2079	15.047	21.392	13.611	23.610	12.387
16	0.2089	16.233	11.421	15.135	14.273	14.615
16	0.2099	13.676	16.848	10.090	15.001	12.574
16	0.2109	5.536	10.184	6.756	25.419	16.885
16	0.2119	10.248	18.998	17.854	15.964	11.309
16	0.2129	6.003	10.780	7.019	12.582	13.006
16	0.2139	11.238	17.484	13.112	11.279	17.869
16	0.2149	10.551	17.501	9.841	18.200	16.086
16	0.2159	29.716	24.544	26.251	13.052	23.589
16	0.2169	17.479	13.018	11.715	14.786	10.593
16	0.2179	15.611	16.809	12.343	17.131	3.003
16	0.2189	5.753	10.109	7.273	7.906	3.035
16	0.2199	11.401	19.293	14.088	15.894	5.851
16	0.2209	13.656	20.509	12.295	14.251	9.974
16	0.2219	11.686	18.941	10.992	10.848	10.683
16	0.2229	4.838	10.638	4.752	9.176	6.477
16	0.2239	7.190	9.527	6.408	13.426	5.441
16	0.2249	7.111	9.756	6.458	13.873	5.205
16	0.2259	10.533	17.408	15.343	17.652	4.084
16	0.2269	13.433	18.119	14.778	17.549	8.226
16	0.2279	14.373	12.251	13.952	10.630	9.951
16	0.2289	4.960	5.078	3.983	5.807	4.339
16	0.2299	11.344	15.719	12.195	16.480	6.075
16	0.2309	12.446	18.781	15.393	14.536	9.729
16	0.2319	13.760	22.645	14.478	27.219	10.098
16	0.2329	18.656	26.194	23.978	16.870	6.195
16	0.2339	20.263	28.174	27.001	17.349	6.297
16	0.2349	14.403	28.069	19.736	20.948	6.193
16	0.2359	11.551	16.092	13.572	12.637	14.270
16	0.2369	8.589	14.216	9.809	17.578	5.316
16	0.2379	16.799	13.326	16.690	15.367	15.958
16	0.2389	21.460	28.423	23.769	12.971	10.479
16	0.2399	16.498	10.506	14.807	13.032	13.513
16	0.2409	8.361	11.018	12.079	5.967	8.382
16	0.2419	11.271	16.317	17.147	13.227	18.765
16	0.2429	20.131	19.763	17.173	14.724	10.311
16	0.2439	13.627	17.045	13.950	13.844	9.467
16	0.2449	10.651	16.469	12.095	12.867	7.803
16	0.2459	11.890	18.021	12.117	14.987	5.911

16	0.2469	19.427	23.498	23.705	11.704	8.701
16	0.2479	17.103	16.934	17.902	9.529	7.761
16	0.2489	9.841	9.102	12.522	8.400	5.421
16	0.2499	11.407	11.253	8.173	16.408	14.961
16	0.2509	16.594	16.723	16.582	24.492	5.940
16	0.2519	12.364	21.463	16.589	21.474	3.708
16	0.2529	2.036	13.570	7.832	12.236	9.317
16	0.2539	21.130	27.303	22.607	16.935	8.298
16	0.2549	20.863	30.408	22.256	24.619	6.905
16	0.2559	13.952	10.807	10.793	9.707	13.605
16	0.2569	12.255	19.169	11.992	18.110	21.392
16	0.2579	20.034	22.879	21.977	9.601	12.651
16	0.2589	12.999	18.776	14.255	17.010	15.224
16	0.2599	19.913	26.293	22.467	12.076	6.470
16	0.2609	13.227	17.683	11.340	9.869	13.047
16	0.2619	11.030	20.398	15.027	18.548	12.741
16	0.2629	12.309	24.916	16.005	14.790	18.022
16	0.2639	8.631	12.525	6.768	11.670	4.611
16	0.2649	6.445	14.462	10.655	17.173	7.371
16	0.2659	10.013	7.982	7.353	6.622	12.745
16	0.2669	10.880	18.955	9.639	13.639	9.250
16	0.2679	18.862	13.044	16.678	10.377	13.102
16	0.2689	6.523	6.007	9.645	7.033	7.656
16	0.2699	10.398	14.842	13.337	15.719	2.697
16	0.2709	13.020	13.183	12.038	9.780	10.187
16	0.2719	16.740	9.571	10.606	9.539	8.916
16	0.2729	19.008	22.932	14.916	13.910	5.036
16	0.2739	14.152	15.317	13.220	22.145	11.313
16	0.2749	15.956	15.763	19.565	8.990	1.766
16	0.2759	15.516	24.018	18.193	10.031	14.311
16	0.2769	14.105	10.491	16.923	9.753	9.294
16	0.2779	7.786	24.995	14.481	20.968	12.346
16	0.2789	13.124	26.507	18.418	19.776	7.615
16	0.2799	18.700	29.379	21.726	10.504	12.030
16	0.2809	28.055	25.186	19.537	7.422	12.800
16	0.2819	13.122	16.593	16.196	13.087	5.184
16	0.2829	13.062	9.925	11.275	12.488	12.519
16	0.2839	8.552	21.739	13.369	18.038	10.732
16	0.2849	18.806	28.970	19.708	24.657	14.421
16	0.2859	24.254	26.260	19.093	9.944	8.968
16	0.2869	12.495	21.738	15.398	12.093	8.691
16	0.2879	9.550	19.165	12.276	14.117	9.282
16	0.2889	8.740	19.147	10.230	20.212	15.505
16	0.2899	14.596	24.766	13.469	25.087	9.717
16	0.2909	18.325	21.929	19.785	14.573	3.437
16	0.2919	19.903	16.479	17.482	17.494	7.981
16	0.2929	31.575	24.535	27.160	22.639	10.184
16	0.2939	10.192	26.782	13.812	13.762	6.665
16	0.2949	13.110	14.808	12.548	4.320	12.813
16	0.2959	12.761	26.068	18.935	16.430	10.495

16	0.2969	25.289	36.595	32.470	9.423	13.989
16	0.2979	14.645	21.276	11.189	16.388	11.875
16	0.2989	15.535	25.374	11.519	14.460	9.201
16	0.2999	9.622	19.945	10.455	12.829	8.368
16	0.3009	11.466	23.089	12.885	9.179	8.770
16	0.3019	7.502	14.356	12.282	11.970	4.547
16	0.3029	12.752	21.078	15.271	10.608	6.553
16	0.3039	14.945	15.124	17.317	14.337	10.115
16	0.3049	11.170	14.574	12.711	12.312	8.704
16	0.3059	10.943	17.604	7.665	14.433	8.022
16	0.3069	12.090	13.815	7.598	9.252	6.986
16	0.3079	14.597	19.904	15.934	12.471	8.907
16	0.3089	14.513	19.854	13.962	11.164	8.792
16	0.3099	13.682	19.542	13.174	11.286	8.899
16	0.3109	11.120	17.420	11.561	7.059	7.068
16	0.3119	8.056	8.112	4.385	7.491	6.229
16	0.3129	10.251	11.192	6.219	13.690	11.202
16	0.3139	18.286	18.690	13.792	10.450	8.735
16	0.3149	14.373	15.382	13.856	12.606	10.518
16	0.3159	19.215	18.579	17.939	11.513	13.443
16	0.3169	10.601	15.071	10.173	12.022	6.017
16	0.3179	14.255	28.298	19.166	11.633	6.689
16	0.3189	13.160	20.524	13.670	16.577	10.242
16	0.3199	12.083	14.688	14.249	10.373	12.678
16	0.3209	14.916	16.756	17.516	10.114	12.929
16	0.3219	14.085	15.417	7.743	20.533	13.036
16	0.3229	19.070	22.945	14.263	17.534	9.318
16	0.3239	18.988	22.904	14.274	17.532	9.283
16	0.3249	16.130	12.265	8.981	17.045	9.052
16	0.3259	16.410	15.933	10.680	13.545	11.344
16	0.3269	14.506	16.995	10.672	13.874	14.804
16	0.3279	5.983	7.490	3.542	11.023	9.450
16	0.3289	3.853	8.378	7.645	16.665	14.483
16	0.3299	15.506	12.051	14.758	6.316	5.058
16	0.3309	22.117	28.314	23.133	6.290	6.746
16	0.3319	27.779	22.134	18.897	14.348	14.047
16	0.3329	17.188	14.085	16.235	23.289	11.565
16	0.3339	12.774	25.091	14.633	22.857	4.008
16	0.3349	19.773	26.120	20.976	13.825	14.479
16	0.3359	9.575	6.683	8.803	10.742	12.016
16	0.3369	16.796	24.323	20.803	7.290	16.092
16	0.3379	18.687	29.977	22.018	10.138	18.961
16	0.3389	23.183	25.403	24.937	16.440	17.859
16	0.3399	18.260	26.136	23.896	14.494	20.850
16	0.3409	18.673	22.619	20.378	12.624	13.199
16	0.3419	11.109	11.320	10.986	11.309	12.670
16	0.3429	14.466	15.457	18.909	15.428	12.694
16	0.3439	16.762	19.780	20.581	17.241	14.067
16	0.3449	15.795	18.426	19.822	11.961	12.346
16	0.3459	12.010	10.049	14.006	9.462	17.492

16	0.3469	11.010	15.667	17.336	7.840	11.916
16	0.3479	17.501	19.601	20.014	9.125	25.113
16	0.3489	10.325	12.411	11.279	13.792	13.495
16	0.3499	15.862	19.499	18.018	13.125	13.767
16	0.3509	12.677	12.628	15.018	5.070	16.699
16	0.3519	12.176	14.096	13.598	11.896	16.215
16	0.3529	16.832	15.677	19.008	5.445	12.632
16	0.3539	13.616	15.276	17.899	14.566	11.145
16	0.3549	16.566	17.101	24.694	7.459	15.710
16	0.3559	15.415	15.311	20.740	7.838	21.376
16	0.3569	14.351	10.464	13.893	17.582	8.325
16	0.3579	11.485	24.482	14.325	25.389	19.817
16	0.3589	15.969	20.991	15.240	16.148	12.936
16	0.3599	11.745	18.620	17.362	12.590	15.657
16	0.3609	9.840	10.332	13.038	9.441	5.846
16	0.3619	9.714	17.336	15.229	10.164	5.397
16	0.3629	13.606	18.984	21.724	7.971	8.329
16	0.3639	13.343	20.133	22.296	8.977	9.917
16	0.3649	7.272	21.872	15.673	14.135	7.888
16	0.3659	12.456	26.197	20.283	17.793	11.053
16	0.3669	14.509	25.953	22.240	16.235	8.226
16	0.3679	16.679	21.716	22.532	9.835	15.432
16	0.3689	17.734	24.511	27.533	10.908	12.879
16	0.3699	11.504	14.715	10.851	8.866	8.688
16	0.3709	4.688	16.396	11.977	7.515	8.419
16	0.3719	16.750	14.933	13.377	13.994	4.363
16	0.3729	20.098	21.498	25.980	11.988	13.719
16	0.3739	10.218	18.990	14.272	12.836	12.679
16	0.3749	7.835	15.410	13.930	23.897	11.182
16	0.3759	16.754	15.088	17.589	6.651	11.788
16	0.3769	11.850	7.639	10.294	11.234	17.134
16	0.3779	7.763	5.801	10.426	10.610	18.950
16	0.3789	7.260	10.284	10.126	12.800	15.400
16	0.3799	8.039	15.475	12.239	20.452	14.798
16	0.3809	8.562	15.964	13.241	9.585	10.932
16	0.3819	13.123	20.114	13.794	12.800	13.969
16	0.3829	11.263	21.111	14.734	14.725	14.328
16	0.3839	8.832	20.982	14.763	22.378	28.270
16	0.3849	15.157	20.666	14.523	37.529	16.051
16	0.3859	14.057	15.626	14.777	15.728	7.589
16	0.3869	14.897	11.983	17.845	7.972	13.229
16	0.3879	8.777	9.200	15.997	6.345	14.020
16	0.3889	14.918	17.515	18.111	20.945	11.549
16	0.3899	20.688	20.000	21.145	14.453	5.503
16	0.3909	10.676	22.299	23.392	11.856	19.062
16	0.3919	8.031	11.761	16.800	9.363	15.241
16	0.3929	15.888	17.904	27.044	6.860	23.673
16	0.3939	9.997	13.277	18.391	11.684	18.403
16	0.3949	7.034	15.433	15.170	15.621	12.222
16	0.3959	5.455	9.154	9.133	19.438	27.690

16	0.3969	5.990	14.075	13.218	13.785	17.098
16	0.3979	8.035	8.554	7.060	17.477	18.905
16	0.3989	4.970	15.388	7.612	9.221	3.320
16	0.3999	4.993	15.403	7.603	9.231	3.336
16	0.4009	4.204	15.825	5.979	13.126	2.172
16	0.4019	10.515	22.859	12.310	14.812	7.409
16	0.4029	11.778	22.274	14.452	12.539	8.979
16	0.4039	10.011	12.113	12.103	9.351	13.435
16	0.4049	10.210	12.392	12.132	9.149	13.408
16	0.4059	9.844	10.328	12.015	12.468	18.674
16	0.4069	5.765	2.693	5.222	3.007	17.515
16	0.4079	2.094	6.833	5.770	9.831	11.520
16	0.4089	18.392	8.880	19.425	22.525	14.502
16	0.4099	13.108	10.781	17.704	18.498	18.059
16	0.4109	6.157	10.593	12.579	7.956	22.406
16	0.4119	4.295	9.071	9.045	5.799	13.966
16	0.4129	6.635	9.971	11.193	12.664	17.039
16	0.4139	9.973	11.070	11.643	8.785	15.677
16	0.4149	10.622	14.875	16.584	10.092	11.736
16	0.4159	7.832	10.387	12.173	9.612	8.493
16	0.4169	10.523	15.762	14.678	13.783	5.551
16	0.4179	8.931	14.765	11.670	10.276	16.386
16	0.4189	6.185	8.645	7.420	7.669	18.703
16	0.4199	7.961	8.816	9.667	4.746	7.678
16	0.4209	9.117	13.088	18.236	14.976	11.203
16	0.4219	6.448	9.247	12.445	11.922	5.777
16	0.4229	0.857	3.629	5.047	5.467	2.593
16	0.4239	0.141	3.604	2.697	3.865	3.291
16	0.4249	9.175	14.177	12.706	8.626	7.274
16	0.4259	4.234	6.444	4.177	14.312	13.069
16	0.4269	5.246	13.884	11.261	13.584	21.510
16	0.4279	21.281	18.545	25.090	14.843	11.952
16	0.4289	7.224	18.860	16.360	10.479	5.811
16	0.4299	16.138	8.347	12.981	11.852	9.459
16	0.4309	17.741	8.198	16.452	13.156	20.240
16	0.4319	19.679	9.033	15.536	12.473	13.517
16	0.4329	20.433	10.621	16.330	21.926	15.847
16	0.4339	10.719	12.284	10.115	16.881	14.780
16	0.4349	3.780	10.599	7.621	17.228	12.918
16	0.4359	5.227	8.928	10.915	9.371	10.547
16	0.4369	20.748	21.202	23.577	16.927	19.697
16	0.4379	7.719	8.056	9.165	22.130	28.600
16	0.4389	8.591	7.573	12.598	16.269	13.757
16	0.4399	8.813	9.655	14.612	13.479	12.589
16	0.4409	4.939	7.682	6.577	6.907	7.367
16	0.4419	7.192	10.040	9.270	6.380	12.364
16	0.4429	7.140	9.925	8.525	5.618	4.739
16	0.4439	8.525	9.965	7.912	10.185	7.057
16	0.4449	8.904	10.231	8.118	10.087	7.251
16	0.4459	3.452	6.193	3.236	5.981	5.939

16	0.4469	1.735	2.900	0.314	2.698	8.428
16	0.4479	6.080	6.860	5.473	6.237	11.481
16	0.4489	6.255	10.946	4.569	14.068	19.478
16	0.4499	8.279	10.110	11.992	16.064	23.932
16	0.4509	3.534	6.612	6.886	10.648	20.955
16	0.4519	10.836	15.587	13.588	20.336	14.433
16	0.4529	7.203	14.168	11.765	10.733	14.122
16	0.4539	8.505	16.080	11.348	7.806	8.754
16	0.4549	7.041	14.420	10.888	8.948	7.589
16	0.4559	10.991	13.027	10.579	16.691	12.716
16	0.4569	13.278	10.741	13.729	11.162	18.249
16	0.4579	7.213	9.509	8.679	8.378	22.121
16	0.4589	12.589	22.290	16.829	10.590	9.954
16	0.4599	10.818	16.287	15.194	14.544	12.535
16	0.4609	14.182	19.650	19.836	17.600	14.137
16	0.4619	8.124	12.640	9.876	12.351	11.104
16	0.4629	10.785	16.709	13.272	16.750	19.572
16	0.4639	10.667	17.073	12.971	13.452	19.306
16	0.4649	20.411	22.616	20.823	21.093	20.596
16	0.4659	8.956	17.981	10.392	12.187	6.628
16	0.4669	12.169	17.972	10.037	12.236	12.411
16	0.4679	7.053	15.552	8.909	19.553	6.644
16	0.4689	11.417	12.543	10.102	18.352	3.317
16	0.4699	11.318	12.149	9.905	18.124	3.321
16	0.4709	6.432	10.767	7.322	8.691	3.141
16	0.4719	6.379	10.531	5.812	9.969	6.483
16	0.4729	16.510	14.429	14.157	24.016	17.520
16	0.4739	18.642	14.561	15.004	11.687	16.361
16	0.4749	9.614	14.602	8.038	10.143	14.332
16	0.4759	9.552	14.012	7.574	10.002	14.160
16	0.4769	10.149	19.576	11.411	20.090	12.270
16	0.4779	11.557	11.822	8.292	17.218	20.895
16	0.4789	5.975	11.353	4.871	8.272	9.157
16	0.4799	5.747	7.476	4.063	10.156	14.125
16	0.4809	5.408	4.376	2.107	2.861	9.427
16	0.4819	12.953	8.653	14.903	12.603	6.622
16	0.4829	4.754	9.726	6.424	15.035	7.513
16	0.4839	4.782	9.707	4.611	13.394	11.247
16	0.4849	3.321	9.635	4.150	12.864	5.728
16	0.4859	5.300	11.167	5.995	18.925	5.132
16	0.4869	7.268	12.009	7.551	22.133	11.397
16	0.4879	11.310	14.010	12.118	24.137	20.291
16	0.4889	6.609	7.873	4.470	18.405	18.332
16	0.4899	10.920	9.146	8.692	24.326	13.041
16	0.4909	6.021	6.819	5.803	16.648	9.291
16	0.4919	6.185	6.802	5.973	16.694	9.057
16	0.4929	4.101	10.836	6.468	11.941	2.103
16	0.4939	9.985	9.834	10.834	18.915	2.676
16	0.4949	10.514	14.049	8.157	16.832	3.227
16	0.4959	10.636	14.046	8.190	16.798	3.133

16	0.4969	8.292	10.693	7.000	12.983	8.653
16	0.4979	6.213	8.306	8.742	10.763	3.994
16	0.4989	5.966	6.485	3.040	15.191	12.095
16	0.4999	18.208	14.465	14.383	19.987	16.293
16	0.5009	12.573	10.689	12.548	21.156	8.490
16	0.5019	15.141	19.235	10.946	26.489	19.072
16	0.5029	17.705	18.933	9.806	28.069	19.396
16	0.5039	6.404	14.695	12.730	22.261	13.234
16	0.5049	9.421	9.801	11.353	18.100	11.294
16	0.5059	9.967	15.400	12.258	18.976	10.998
16	0.5069	7.431	10.628	8.102	18.372	10.526
16	0.5079	7.971	6.418	7.634	4.888	9.965
16	0.5089	4.712	5.953	6.261	17.859	9.181
16	0.5099	11.448	13.449	9.109	16.887	16.580
16	0.5109	16.610	14.823	9.624	25.770	20.374
16	0.5119	8.767	5.954	8.105	17.241	21.724
16	0.5129	12.364	13.577	9.476	15.482	7.597
16	0.5139	10.687	4.368	8.154	7.515	10.505
16	0.5149	8.266	6.992	6.550	15.819	16.866
16	0.5159	17.351	16.046	9.840	25.366	23.760
16	0.5169	21.458	16.360	15.886	23.965	14.539
16	0.5179	10.149	6.981	4.516	13.504	5.340
16	0.5189	9.557	11.278	7.472	12.558	7.890
16	0.5199	18.325	19.255	17.255	16.567	12.325
16	0.5209	13.780	14.014	10.361	22.716	13.613
16	0.5219	5.126	9.972	5.673	21.096	11.977
16	0.5229	15.872	13.765	15.906	15.569	10.677
16	0.5239	8.616	6.923	8.663	7.111	8.509
16	0.5249	10.413	6.290	9.374	15.431	12.569
16	0.5259	10.814	9.989	10.427	11.222	10.772
16	0.5269	9.543	7.948	12.395	14.395	7.307
16	0.5279	26.351	14.163	17.575	23.989	8.894
16	0.5289	8.067	7.438	8.364	16.718	12.953
16	0.5299	5.811	6.156	6.172	12.668	10.513
16	0.5309	7.691	9.376	11.900	19.194	9.206
16	0.5319	4.984	5.783	5.157	12.553	2.229
16	0.5329	13.444	10.781	7.603	7.762	3.521
16	0.5339	10.743	6.314	8.220	7.935	6.243
16	0.5349	5.326	5.188	2.280	10.020	6.228
16	0.5359	15.194	14.426	14.010	15.837	23.621
16	0.5369	17.879	15.495	11.284	11.647	7.654
16	0.5379	22.599	21.531	14.807	16.499	9.972
16	0.5389	19.166	16.674	9.774	18.576	7.628
16	0.5399	7.638	12.553	6.950	21.059	12.631
16	0.5409	20.318	19.943	9.703	10.138	11.450
16	0.5419	12.717	10.581	10.734	31.206	18.806
16	0.5429	17.166	11.245	11.416	27.940	21.650
16	0.5439	13.054	8.727	8.426	18.310	23.715
16	0.5449	23.064	15.449	20.047	7.129	12.024
16	0.5459	17.161	17.256	14.598	16.702	8.127

16	0.5469	27.323	20.047	28.349	13.016	11.908
16	0.5479	14.448	14.547	12.258	15.454	26.210
16	0.5489	10.257	15.347	8.419	13.668	21.896
16	0.5499	14.287	13.879	15.352	15.842	16.231
16	0.5509	9.139	9.083	6.615	3.248	8.292
16	0.5519	8.689	7.754	7.767	6.029	8.181
16	0.5529	8.063	7.896	10.786	12.298	6.384
16	0.5539	13.001	14.063	11.865	12.571	3.286
16	0.5549	15.216	5.868	10.299	13.850	7.635
16	0.5559	14.127	15.318	8.635	9.482	13.896
16	0.5569	16.239	14.029	14.931	10.485	7.625
16	0.5579	25.934	21.755	25.664	9.162	9.087
16	0.5589	21.530	19.790	17.414	19.638	21.212
16	0.5599	10.193	11.784	8.120	11.982	10.863
16	0.5609	13.346	14.406	12.122	13.687	3.929
16	0.5619	12.594	15.104	11.383	15.080	12.443
16	0.5629	14.079	9.706	9.281	14.675	9.791
16	0.5639	14.503	12.784	14.745	16.108	14.357
16	0.5649	8.240	12.089	9.640	16.129	24.650
16	0.5659	7.980	11.625	9.243	15.643	24.631
16	0.5669	19.397	15.509	17.466	20.216	15.037
16	0.5679	10.364	12.086	8.505	10.220	11.572
16	0.5689	13.804	11.793	7.682	15.288	21.436
16	0.5699	15.632	7.256	14.253	12.406	20.137
16	0.5709	16.441	14.564	15.542	16.277	13.183
16	0.5719	21.682	12.701	13.004	12.492	9.969
16	0.5729	18.163	14.795	13.193	10.079	10.777
16	0.5739	16.920	10.976	10.660	14.951	15.038
16	0.5749	21.617	12.245	15.753	11.922	14.985
16	0.5759	11.586	5.460	7.025	6.911	6.684
16	0.5769	11.034	8.914	11.789	3.652	6.377
16	0.5779	10.477	8.200	7.033	5.868	4.493
16	0.5789	13.016	5.177	7.711	5.387	12.927
16	0.5799	17.986	14.059	15.677	17.874	15.189
16	0.5809	17.988	14.091	15.691	17.828	15.114
16	0.5819	16.981	12.268	13.088	11.566	12.342
16	0.5829	20.608	11.307	11.415	11.743	10.451
16	0.5839	19.688	12.680	11.110	12.954	11.564
16	0.5849	12.094	8.205	7.368	3.010	5.615
16	0.5859	19.176	16.812	12.311	17.350	8.984
16	0.5869	8.242	19.623	7.465	22.149	12.679
16	0.5879	5.956	14.202	6.060	9.964	5.768
16	0.5889	5.100	5.071	4.141	11.010	8.695
16	0.5899	3.278	4.889	7.077	7.254	10.081
16	0.5909	9.378	8.204	12.102	4.420	5.403
16	0.5919	12.497	11.402	12.716	5.084	5.384
16	0.5929	23.961	18.143	19.082	10.720	10.100
16	0.5939	22.395	15.217	16.367	13.226	16.377
16	0.5949	8.414	6.011	4.449	11.008	11.337
16	0.5959	8.740	7.902	6.052	9.156	5.909

16	0.5969	13.366	13.177	13.573	6.920	3.533
16	0.5979	10.570	10.032	8.500	10.394	4.298
16	0.5989	7.967	5.851	5.719	3.807	11.336
16	0.5999	12.165	9.099	7.345	7.126	5.719
16	0.6009	13.111	13.174	10.263	11.768	10.792
16	0.6019	8.656	8.905	10.779	14.928	15.297
16	0.6029	6.579	6.276	6.446	14.265	20.079
16	0.6039	5.015	2.958	3.277	9.216	6.373
16	0.6049	14.405	11.038	7.646	15.543	11.868
16	0.6059	21.267	23.881	19.826	13.919	9.974
16	0.6069	12.040	9.596	11.128	14.709	8.228
16	0.6079	13.280	15.726	13.771	18.547	16.232
16	0.6089	12.005	6.907	6.800	10.259	9.061
16	0.6099	13.748	5.567	14.199	10.741	8.900
16	0.6109	15.897	6.623	11.805	10.173	5.805
16	0.6119	13.010	8.653	10.050	13.446	5.981
16	0.6129	17.294	17.439	15.680	17.566	12.327
16	0.6139	12.199	6.370	8.773	16.303	13.668
16	0.6149	19.381	15.989	11.670	18.201	23.338
16	0.6159	26.623	14.783	14.002	18.975	18.735
16	0.6169	25.650	11.848	17.316	15.693	7.210
16	0.6179	20.504	7.388	20.009	10.769	12.735
16	0.6189	19.557	12.383	19.998	3.467	8.961
16	0.6199	21.396	16.892	16.909	10.052	10.039
16	0.6209	7.640	5.837	10.184	3.225	8.047
16	0.6219	9.970	5.239	8.151	8.071	10.387
16	0.6229	12.713	8.936	10.282	11.051	11.533
16	0.6239	19.150	10.346	12.764	15.693	10.148
16	0.6249	16.655	11.655	15.458	12.329	15.895
16	0.6259	14.171	10.246	15.941	13.125	26.191
16	0.6269	13.473	18.871	18.741	13.631	15.998
16	0.6279	18.534	15.619	20.514	12.289	10.541
16	0.6289	13.390	12.882	14.004	6.414	14.546
16	0.6299	12.749	6.131	10.446	11.302	19.078
16	0.6309	23.635	20.858	27.619	8.953	14.010
16	0.6319	14.728	13.627	18.290	11.413	13.462
16	0.6329	18.174	14.139	17.019	10.713	7.385
16	0.6339	7.158	7.142	6.673	5.527	15.417
16	0.6349	10.046	8.647	10.980	12.329	18.469
16	0.6359	20.926	9.781	17.929	7.102	10.861
16	0.6369	17.693	13.303	15.585	12.433	6.740
16	0.6379	14.029	12.666	11.040	5.073	2.291
16	0.6389	13.313	8.372	9.200	6.306	9.347
16	0.6399	8.571	2.979	5.351	3.069	11.951
16	0.6409	9.334	7.298	5.972	5.218	9.309
16	0.6419	13.099	7.304	8.755	11.263	17.339
16	0.6429	18.006	13.392	19.682	18.082	16.645
16	0.6439	15.897	13.310	15.006	20.542	26.470
16	0.6449	29.203	15.008	19.767	16.914	19.309
16	0.6459	18.909	13.822	13.191	11.213	8.735

16	0.6469	26.206	16.479	16.558	6.681	13.409
16	0.6479	16.589	8.917	12.511	13.089	15.779
16	0.6489	23.512	15.421	23.890	20.443	26.849
16	0.6499	27.837	20.393	28.715	21.289	19.215
16	0.6509	16.313	9.901	15.765	18.272	32.782
16	0.6519	20.687	14.250	16.756	13.469	22.310
16	0.6529	30.706	20.456	31.609	19.411	23.181
16	0.6539	35.943	26.638	32.246	12.601	20.808
16	0.6549	17.095	14.102	24.970	15.721	13.626
16	0.6559	25.117	21.132	32.522	19.505	18.044
16	0.6569	15.133	9.672	13.559	10.112	6.949
16	0.6579	23.106	20.095	22.614	27.938	35.589
16	0.6589	15.406	19.751	19.234	12.859	13.592
16	0.6599	20.650	17.190	23.860	20.386	27.283
16	0.6609	13.367	10.368	12.178	18.509	9.189
16	0.6619	12.677	11.721	11.821	11.703	14.724
16	0.6629	12.712	13.877	12.267	17.216	15.457
16	0.6639	8.157	9.304	9.493	8.373	10.823
16	0.6649	16.028	8.841	11.636	15.210	16.666
16	0.6659	18.246	10.330	14.856	8.827	8.694
16	0.6669	14.029	14.718	14.743	12.357	24.717
16	0.6679	10.835	9.859	15.207	13.952	12.237
16	0.6689	6.335	3.733	9.469	5.190	8.176
16	0.6699	10.610	7.480	9.617	3.461	17.196
16	0.6709	17.819	15.796	18.206	6.391	22.116
16	0.6719	16.182	10.325	11.218	10.781	17.438
16	0.6729	13.150	8.442	12.166	10.699	10.559
16	0.6739	26.459	19.320	27.810	9.486	11.371
16	0.6749	34.643	25.172	41.061	3.769	23.284
16	0.6759	16.481	17.562	16.975	13.901	9.964
16	0.6769	11.227	11.969	9.601	5.927	11.653
16	0.6779	8.904	6.440	14.500	10.781	9.583
16	0.6789	14.475	11.191	15.607	6.810	12.490
16	0.6799	13.459	13.805	16.372	10.515	13.215
16	0.6809	17.868	16.824	16.603	13.188	10.182
16	0.6819	20.961	20.066	16.436	8.052	13.993
16	0.6829	22.951	20.411	16.352	6.209	10.842
16	0.6839	29.075	27.663	22.817	5.342	14.099
16	0.6849	18.864	17.985	12.882	9.863	12.097
16	0.6859	37.009	29.522	22.966	14.212	18.515
16	0.6869	23.911	15.431	22.109	8.842	7.222
16	0.6879	19.690	15.335	16.054	9.234	9.382
16	0.6889	20.638	15.936	17.060	9.170	9.379
16	0.6899	24.817	19.741	27.540	11.309	13.433
16	0.6909	11.520	10.356	9.923	13.949	12.331
16	0.6919	8.242	14.634	14.861	12.536	24.036
16	0.6929	16.260	11.320	21.745	10.538	10.388
16	0.6939	19.014	15.096	20.462	10.467	12.314
16	0.6949	10.116	7.536	11.846	10.627	4.296
16	0.6959	9.912	7.170	11.665	10.102	11.689

16	0.6969	10.863	6.680	12.648	6.934	17.913
16	0.6979	15.046	12.613	18.358	10.681	13.255
16	0.6989	16.318	17.054	22.839	15.399	13.836
16	0.6999	15.965	14.592	17.895	14.447	7.897
16	0.7009	11.401	10.032	11.305	11.236	19.065
16	0.7019	7.179	5.271	9.808	9.863	8.962
16	0.7029	13.378	9.441	14.782	11.526	10.958
16	0.7039	20.841	16.594	21.181	9.301	9.484
16	0.7049	16.696	11.938	16.382	11.271	7.775
16	0.7059	18.589	10.632	10.836	15.559	7.576
16	0.7069	16.211	17.484	14.617	16.011	10.868
16	0.7079	9.384	11.356	10.502	15.381	8.671
16	0.7089	14.694	18.585	21.749	6.844	9.872
16	0.7099	18.250	15.286	11.557	10.182	11.381
16	0.7109	6.176	9.558	3.908	16.299	9.568
16	0.7119	24.607	17.747	18.425	10.610	12.324
16	0.7129	22.384	16.131	17.425	10.498	13.589
16	0.7139	13.509	14.913	19.215	22.778	18.377
16	0.7149	20.453	22.674	17.553	27.175	34.218
16	0.7159	9.298	15.306	13.237	15.520	11.769
16	0.7169	10.758	12.587	7.245	8.602	9.723
16	0.7179	9.754	11.160	4.065	12.114	7.907
16	0.7189	15.517	10.889	8.279	4.896	11.870
16	0.7199	10.024	11.498	8.632	7.318	7.709
16	0.7209	9.715	12.481	8.066	12.001	17.823
16	0.7219	2.513	8.272	7.947	15.310	10.281
16	0.7229	9.833	7.631	11.797	10.538	7.208
16	0.7239	15.870	13.216	11.968	11.424	21.304
16	0.7249	12.791	8.552	11.411	14.234	5.986
16	0.7259	19.516	15.060	11.855	16.280	18.345
16	0.7269	19.480	15.062	11.821	16.396	18.351
16	0.7279	16.382	12.481	11.066	13.556	20.603
16	0.7289	20.589	22.435	23.530	13.070	8.066
16	0.7299	10.153	14.734	17.182	13.130	7.719
16	0.7309	15.315	18.582	13.866	16.055	16.984
16	0.7319	12.595	9.992	11.601	15.886	18.699
16	0.7329	15.439	15.565	19.939	13.757	14.018
16	0.7339	18.177	17.127	15.148	14.120	23.341
16	0.7349	19.683	20.259	25.646	7.418	10.400
16	0.7359	12.709	11.625	17.235	7.915	19.629
16	0.7369	4.530	8.928	10.692	6.953	6.934
16	0.7379	13.914	10.340	10.189	8.144	15.134
16	0.7389	14.818	6.602	7.091	4.384	9.536
16	0.7399	9.852	9.108	4.524	9.287	18.407
16	0.7409	24.205	16.714	13.488	7.235	18.063
16	0.7419	24.887	20.405	18.099	7.068	9.167
16	0.7429	8.401	10.113	16.992	9.409	18.897
16	0.7439	11.247	8.288	14.296	6.851	17.073
16	0.7449	22.001	12.701	15.451	11.246	22.777
16	0.7459	10.255	7.894	13.198	10.282	25.702

16	0.7469	9.104	7.320	9.317	7.298	16.546
16	0.7479	14.126	18.283	18.616	7.636	17.584
16	0.7489	8.440	9.373	8.380	7.619	18.833
16	0.7499	9.039	6.278	6.973	6.584	19.991
16	0.7509	5.398	4.790	9.302	8.136	14.543
16	0.7519	12.267	10.772	14.044	3.866	6.145
16	0.7529	14.987	11.725	13.819	9.608	12.393
16	0.7539	11.592	7.929	10.044	13.611	14.734
16	0.7549	11.494	11.496	10.706	12.458	16.415
16	0.7559	9.996	11.233	11.299	7.842	12.912
16	0.7569	16.408	10.586	13.777	16.345	12.398
16	0.7579	31.191	31.418	23.481	11.650	17.531
16	0.7589	23.340	21.161	21.072	10.150	16.994
16	0.7599	17.025	16.651	20.510	10.070	20.892
16	0.7609	22.381	24.993	28.738	15.445	30.609
16	0.7619	22.188	25.456	24.585	16.142	19.332
16	0.7629	8.499	13.206	16.957	15.817	21.763
16	0.7639	10.181	8.925	12.207	7.189	15.760
16	0.7649	9.229	9.657	13.383	11.272	13.338
16	0.7659	17.748	15.537	18.072	22.637	25.619
16	0.7669	27.117	17.113	25.529	21.706	20.367
16	0.7679	20.147	12.866	11.802	18.877	19.039
16	0.7689	13.163	7.265	7.016	17.251	15.740
16	0.7699	11.281	7.344	12.300	12.242	19.901
16	0.7709	13.485	10.265	14.388	15.820	28.260
16	0.7719	11.424	10.042	11.234	14.399	17.218
16	0.7729	14.302	16.135	20.730	10.688	17.916
16	0.7739	21.236	19.013	17.006	12.863	27.249
16	0.7749	13.970	10.359	10.441	20.928	25.754
16	0.7759	0.000	0.000	0.000	0.000	0.000
17	0.0017	0.000	0.000	0.000	0.000	0.000
17	0.0027	14.920	15.041	17.413	24.499	21.972
17	0.0037	8.996	7.868	11.442	12.816	8.420
17	0.0047	20.696	18.577	20.282	16.377	9.984
17	0.0057	19.468	20.977	14.316	18.470	27.100
17	0.0067	16.556	11.066	13.847	13.561	15.161
17	0.0077	16.916	14.989	16.435	12.579	11.007
17	0.0087	13.448	13.885	15.142	14.536	5.951
17	0.0097	19.673	17.049	22.174	15.719	7.429
17	0.0107	11.004	7.187	10.156	6.666	13.730
17	0.0117	10.008	10.644	13.727	22.842	15.533
17	0.0127	8.390	7.631	10.089	22.748	22.440
17	0.0137	11.470	18.055	18.470	27.612	20.069
17	0.0147	7.045	12.591	12.498	18.959	11.375
17	0.0157	14.098	16.405	20.295	16.444	5.009
17	0.0167	12.560	10.739	15.476	13.051	8.655
17	0.0177	8.414	5.341	8.642	7.213	13.115
17	0.0187	11.333	9.373	12.474	14.942	17.091
17	0.0197	12.867	11.409	18.898	15.053	13.824
17	0.0207	9.741	11.453	8.870	19.135	17.560

17	0.0217	9.328	7.904	9.613	14.517	19.509
17	0.0227	11.660	15.065	9.123	18.786	16.740
17	0.0237	7.966	7.277	8.121	8.934	12.734
17	0.0247	16.738	16.534	17.085	11.054	20.021
17	0.0257	11.572	5.533	9.355	17.833	19.473
17	0.0267	16.925	9.316	11.274	10.166	21.373
17	0.0277	15.125	7.494	7.118	8.476	11.201
17	0.0287	7.530	4.384	7.725	9.872	9.570
17	0.0297	13.870	10.057	12.336	21.089	20.495
17	0.0307	9.572	7.915	9.456	12.506	26.492
17	0.0317	11.374	9.814	11.745	11.876	12.796
17	0.0327	9.395	4.615	7.398	12.857	11.044
17	0.0337	12.782	14.895	13.407	13.767	14.152
17	0.0347	14.247	21.235	16.567	23.104	5.751
17	0.0357	10.139	11.253	14.143	19.760	6.833
17	0.0367	6.212	6.127	10.223	15.985	7.043
17	0.0377	9.091	11.155	12.586	18.994	10.507
17	0.0387	8.929	14.977	14.626	16.417	8.191
17	0.0397	9.159	10.732	6.473	10.542	20.785
17	0.0407	9.677	7.511	5.822	15.475	15.462
17	0.0417	5.944	7.405	2.615	11.984	7.538
17	0.0427	17.879	12.816	14.619	10.364	21.625
17	0.0437	11.696	14.545	18.690	11.457	15.110
17	0.0447	12.297	16.975	15.350	23.807	17.384
17	0.0457	13.851	16.793	14.399	19.196	12.643
17	0.0467	9.478	10.711	10.607	18.476	17.187
17	0.0477	12.619	17.045	11.446	11.971	9.569
17	0.0487	10.473	16.068	8.217	21.381	13.702
17	0.0497	8.616	20.423	11.175	22.424	13.592
17	0.0507	9.754	9.458	8.720	24.237	15.002
17	0.0517	12.795	8.271	14.050	12.903	14.946
17	0.0527	12.624	15.902	13.138	12.961	14.873
17	0.0537	9.312	12.907	9.115	10.814	5.956
17	0.0547	16.347	21.589	18.135	13.465	7.889
17	0.0557	13.467	22.620	16.989	20.487	7.090
17	0.0567	10.521	12.496	11.640	21.342	15.871
17	0.0577	15.481	13.828	12.861	21.639	7.098
17	0.0587	19.396	14.314	17.144	35.245	22.874
17	0.0597	20.961	12.430	14.620	11.601	16.362
17	0.0607	19.696	20.148	9.660	16.342	12.012
17	0.0617	18.303	25.595	12.243	20.781	14.707
17	0.0627	18.615	17.382	18.333	12.507	7.958
17	0.0637	12.956	18.279	15.528	23.815	5.089
17	0.0647	13.879	5.125	11.903	18.267	8.291
17	0.0657	9.952	10.474	12.968	21.015	11.857
17	0.0667	16.134	24.170	19.154	21.747	20.495
17	0.0677	16.515	24.571	15.801	20.325	14.826
17	0.0687	18.117	14.898	18.661	10.680	11.636
17	0.0697	13.230	14.315	13.275	14.687	3.656
17	0.0707	13.537	9.127	9.987	21.176	18.172

17	0.0717	15.016	16.170	6.394	24.533	7.476
17	0.0727	16.830	11.894	9.433	19.197	14.862
17	0.0737	15.382	10.173	10.067	13.316	9.954
17	0.0747	9.156	8.170	9.989	18.317	12.241
17	0.0757	14.131	8.185	11.923	11.003	9.767
17	0.0767	8.067	6.179	8.367	8.346	6.315
17	0.0777	9.547	8.579	8.954	8.051	12.566
17	0.0787	8.308	6.872	6.371	10.112	11.996
17	0.0797	19.607	17.261	13.081	18.204	14.984
17	0.0807	22.788	17.895	18.085	11.783	8.335
17	0.0817	26.326	15.919	20.092	10.547	10.401
17	0.0827	12.798	11.032	14.807	21.329	30.001
17	0.0837	7.500	13.076	10.071	15.832	14.485
17	0.0847	11.270	12.569	10.523	22.967	21.591
17	0.0857	9.708	15.177	11.383	11.191	10.108
17	0.0867	9.244	15.622	16.584	13.131	12.285
17	0.0877	10.766	12.578	11.134	4.233	5.686
17	0.0887	9.403	11.899	7.118	16.135	13.699
17	0.0897	19.827	20.358	11.195	22.736	17.643
17	0.0907	11.964	22.637	11.087	17.294	20.933
17	0.0917	8.039	3.818	7.493	7.721	10.110
17	0.0927	11.651	14.963	10.793	26.143	22.310
17	0.0937	10.484	21.562	12.108	22.741	19.339
17	0.0947	11.696	16.226	8.236	17.039	6.468
17	0.0957	12.261	13.618	9.042	7.403	10.313
17	0.0967	15.655	18.999	14.693	14.598	16.210
17	0.0977	18.712	21.454	17.408	14.741	9.400
17	0.0987	25.611	21.470	18.251	2.921	12.614
17	0.0997	16.600	16.202	13.651	5.390	11.029
17	0.1007	16.894	15.965	16.762	4.914	6.799
17	0.1017	16.903	15.958	16.636	4.965	6.821
17	0.1027	16.890	15.933	16.488	5.015	6.840
17	0.1037	13.924	8.309	12.262	13.231	7.725
17	0.1047	15.716	9.011	12.009	16.110	12.955
17	0.1057	21.851	15.391	15.994	22.930	12.371
17	0.1067	20.524	19.173	15.781	18.539	15.045
17	0.1077	20.541	19.166	15.767	18.528	14.986
17	0.1087	21.838	16.349	15.306	10.825	14.498
17	0.1097	21.960	16.366	15.280	10.783	14.457
17	0.1107	22.079	16.379	15.250	10.742	14.428
17	0.1117	22.196	16.388	15.218	10.701	14.411
17	0.1127	22.309	16.392	15.183	10.662	14.407
17	0.1137	20.274	10.277	14.673	8.964	14.399
17	0.1147	20.273	10.247	14.627	8.909	14.297
17	0.1157	28.929	22.715	21.864	9.624	22.492
17	0.1167	11.109	9.948	7.365	13.749	22.487
17	0.1177	26.058	11.256	19.474	9.812	15.160
17	0.1187	21.618	10.494	17.219	7.826	16.935
17	0.1197	9.760	4.898	9.636	6.171	5.930
17	0.1207	13.522	14.613	8.372	16.357	19.597

17	0.1217	18.059	15.905	17.464	9.031	20.052
17	0.1227	21.740	20.386	20.815	9.428	21.510
17	0.1237	24.777	15.081	22.892	13.618	17.164
17	0.1247	22.275	15.329	21.068	10.159	21.817
17	0.1257	25.710	18.990	22.084	5.217	18.096
17	0.1267	22.669	15.485	17.499	17.749	17.841
17	0.1277	26.918	18.924	18.106	19.394	28.204
17	0.1287	19.807	21.109	18.760	19.054	14.565
17	0.1297	15.320	15.305	11.354	10.718	11.793
17	0.1307	19.846	16.329	10.989	8.884	15.093
17	0.1317	26.064	20.472	17.016	15.628	15.867
17	0.1327	15.069	10.640	9.625	18.797	9.229
17	0.1337	16.259	11.216	8.141	10.562	27.581
17	0.1347	15.948	10.898	9.960	15.636	9.848
17	0.1357	20.272	15.421	11.414	16.318	13.616
17	0.1367	14.102	9.016	8.791	16.589	24.437
17	0.1377	16.537	17.423	10.010	15.848	31.425
17	0.1387	15.747	10.577	10.195	8.152	22.252
17	0.1397	14.580	13.526	7.555	16.983	22.621
17	0.1407	10.019	12.504	6.640	17.602	12.586
17	0.1417	11.367	13.039	8.044	12.256	14.188
17	0.1427	13.857	12.410	9.450	13.625	15.993
17	0.1437	12.346	9.831	5.370	20.234	16.349
17	0.1447	7.891	6.083	3.485	15.120	20.649
17	0.1457	17.433	13.732	11.703	18.578	20.296
17	0.1467	22.252	23.581	11.917	17.030	28.748
17	0.1477	16.993	20.102	8.203	13.496	31.225
17	0.1487	19.936	17.705	7.799	12.644	21.825
17	0.1497	8.951	9.539	5.512	22.015	15.296
17	0.1507	9.846	8.140	7.269	19.958	21.033
17	0.1517	12.872	15.234	13.201	18.997	23.125
17	0.1527	24.487	16.975	19.693	11.644	24.288
17	0.1537	18.296	13.267	7.203	7.932	19.819
17	0.1547	16.053	12.493	13.716	18.974	17.895
17	0.1557	11.847	9.182	7.228	6.212	13.450
17	0.1567	16.631	15.649	10.138	15.762	17.370
17	0.1577	12.508	14.906	13.859	15.289	22.856
17	0.1587	16.641	14.721	10.308	12.689	18.237
17	0.1597	13.001	9.905	6.779	22.038	28.967
17	0.1607	12.497	13.196	7.888	16.248	16.737
17	0.1617	6.202	9.748	7.188	14.607	18.061
17	0.1627	14.848	16.082	10.023	14.502	13.496
17	0.1637	10.657	9.769	7.699	12.259	21.989
17	0.1647	11.620	10.214	11.294	14.467	21.340
17	0.1657	15.244	16.329	20.594	18.643	19.679
17	0.1667	10.649	12.924	15.469	6.986	16.087
17	0.1677	16.119	17.566	20.762	5.217	11.072
17	0.1687	17.236	19.382	16.772	9.659	12.575
17	0.1697	9.149	8.022	14.787	9.842	15.550
17	0.1707	15.937	13.155	19.485	9.819	17.093

17	0.1717	19.904	14.313	17.467	11.882	18.909
17	0.1727	19.416	17.194	16.706	14.922	23.032
17	0.1737	15.840	12.015	15.028	11.564	12.793
17	0.1747	8.475	9.790	9.702	15.826	15.524
17	0.1757	12.511	19.369	19.767	16.747	15.626
17	0.1767	6.441	9.216	6.750	17.576	23.947
17	0.1777	7.559	5.403	8.883	4.783	24.548
17	0.1787	13.175	15.530	20.143	14.561	30.086
17	0.1797	11.144	12.199	21.189	11.660	18.494
17	0.1807	17.162	12.139	19.210	18.103	22.599
17	0.1817	10.521	6.892	15.004	9.877	14.718
17	0.1827	5.043	6.012	9.538	11.003	12.839
17	0.1837	17.994	13.540	14.903	24.021	23.119
17	0.1847	15.614	16.208	18.348	21.486	21.102
17	0.1857	14.853	18.723	22.153	27.131	17.846
17	0.1867	8.924	9.279	4.626	14.732	18.536
17	0.1877	14.499	6.496	10.467	7.239	18.325
17	0.1887	12.981	9.650	12.952	9.125	14.198
17	0.1897	15.326	9.063	6.588	14.290	21.719
17	0.1907	25.749	25.479	27.643	14.157	10.272
17	0.1917	13.149	15.632	14.982	13.476	10.077
17	0.1927	10.349	12.208	11.415	20.847	19.305
17	0.1937	19.783	17.532	14.144	24.326	18.136
17	0.1947	22.880	20.196	27.180	19.375	21.124
17	0.1957	13.656	11.315	14.215	14.315	14.634
17	0.1967	11.797	10.636	13.669	10.415	12.507
17	0.1977	3.830	8.786	3.594	17.568	12.980
17	0.1987	9.303	16.017	10.698	13.523	13.157
17	0.1997	10.761	14.598	8.765	32.622	24.787
17	0.2007	10.186	14.447	11.042	36.940	25.958
17	0.2017	10.673	19.763	11.315	26.042	8.193
17	0.2027	12.845	20.233	15.603	18.594	9.466
17	0.2037	10.356	10.584	15.317	12.599	11.621
17	0.2047	4.797	5.708	7.454	6.998	9.745
17	0.2057	14.823	17.521	13.650	24.289	21.649
17	0.2067	17.796	13.498	12.022	23.045	18.631
17	0.2077	12.923	9.980	10.630	19.534	12.799
17	0.2087	19.113	8.257	12.682	22.169	18.576
17	0.2097	18.341	13.612	17.669	20.257	13.577
17	0.2107	8.434	10.821	8.163	10.030	10.923
17	0.2117	11.089	9.809	11.244	12.717	7.403
17	0.2127	7.867	11.451	5.043	16.033	18.782
17	0.2137	21.093	15.575	13.312	17.726	14.644
17	0.2147	22.682	23.283	18.184	22.877	25.032
17	0.2157	18.710	12.864	16.564	25.118	14.446
17	0.2167	14.211	9.497	14.914	15.978	25.326
17	0.2177	5.040	9.794	9.809	15.464	20.227
17	0.2187	16.710	18.072	17.949	18.442	15.688
17	0.2197	27.000	26.328	27.696	10.598	23.193
17	0.2207	13.553	13.939	12.637	19.498	8.736

17	0.2217	11.693	8.375	10.841	10.286	12.338
17	0.2227	20.502	14.117	13.091	19.855	6.412
17	0.2237	26.474	20.523	22.086	12.677	12.297
17	0.2247	17.469	8.065	8.400	11.207	23.939
17	0.2257	15.951	7.546	6.469	10.234	17.882
17	0.2267	18.939	15.218	15.477	14.965	9.531
17	0.2277	16.021	7.918	15.118	17.507	8.734
17	0.2287	14.889	14.198	8.732	17.630	8.941
17	0.2297	12.596	9.162	9.934	7.498	8.253
17	0.2307	10.820	6.471	11.128	6.552	11.594
17	0.2317	17.318	11.374	6.696	21.593	26.095
17	0.2327	15.242	14.725	12.348	19.459	10.472
17	0.2337	10.745	8.175	12.598	14.709	9.966
17	0.2347	20.949	16.519	20.614	10.810	17.608
17	0.2357	20.037	13.932	16.053	10.425	16.896
17	0.2367	22.220	20.257	10.565	10.291	9.435
17	0.2377	12.108	11.180	11.611	15.234	8.231
17	0.2387	8.617	5.457	4.720	9.941	19.031
17	0.2397	5.594	6.300	5.299	7.918	4.722
17	0.2407	17.679	10.228	18.834	11.757	4.130
17	0.2417	19.373	12.635	13.670	10.814	13.866
17	0.2427	20.796	15.242	14.686	26.653	10.496
17	0.2437	10.843	8.702	11.230	15.959	4.419
17	0.2447	2.792	9.145	6.260	8.311	10.816
17	0.2457	20.177	14.645	11.203	10.761	9.965
17	0.2467	18.923	13.021	13.972	23.032	8.806
17	0.2477	14.288	15.195	8.217	26.144	12.881
17	0.2487	9.003	11.106	5.218	13.037	6.746
17	0.2497	12.168	11.564	11.649	8.253	12.873
17	0.2507	16.442	9.136	14.666	14.714	12.828
17	0.2517	12.393	7.237	5.990	15.435	28.747
17	0.2527	18.244	12.781	14.926	13.253	12.334
17	0.2537	12.399	4.928	8.109	4.390	16.782
17	0.2547	11.822	13.127	14.250	10.275	16.402
17	0.2557	13.707	11.318	15.191	9.054	10.239
17	0.2567	19.276	18.071	17.390	10.903	10.341
17	0.2577	25.794	20.645	27.083	11.961	5.286
17	0.2587	24.377	18.717	22.807	13.690	13.622
17	0.2597	8.627	8.546	7.821	18.642	21.665
17	0.2607	17.887	17.899	16.833	13.156	16.516
17	0.2617	17.532	17.142	16.353	14.941	19.116
17	0.2627	11.256	8.644	8.468	8.855	9.144
17	0.2637	17.278	10.656	9.622	10.962	9.835
17	0.2647	13.743	11.042	12.321	14.108	14.827
17	0.2657	12.676	16.605	6.975	11.823	12.281
17	0.2667	17.886	21.078	19.496	13.354	21.603
17	0.2677	18.424	15.590	16.778	19.294	21.457
17	0.2687	14.883	14.549	15.219	12.161	20.297
17	0.2697	17.972	18.325	15.593	8.833	13.465
17	0.2707	23.980	14.553	12.724	11.224	11.846

17	0.2717	5.738	4.751	4.712	7.691	14.909
17	0.2727	19.478	11.543	15.426	12.747	18.744
17	0.2737	6.242	7.110	6.522	8.600	7.290
17	0.2747	17.837	11.607	15.141	8.229	16.244
17	0.2757	14.986	9.545	11.032	8.874	15.940
17	0.2767	14.255	13.188	8.805	17.674	21.599
17	0.2777	8.279	10.184	8.932	11.800	18.052
17	0.2787	16.751	12.886	17.926	8.979	24.926
17	0.2797	10.586	16.261	14.266	13.459	12.179
17	0.2807	16.959	22.092	22.494	13.530	13.620
17	0.2817	17.158	22.022	22.155	15.711	20.490
17	0.2827	15.106	8.515	11.205	14.604	21.016
17	0.2837	16.029	17.889	11.619	24.134	18.963
17	0.2847	17.220	18.542	17.439	7.223	7.861
17	0.2857	8.811	9.751	7.759	7.219	4.703
17	0.2867	12.165	12.799	11.353	15.066	17.791
17	0.2877	12.201	12.793	11.340	15.026	17.852
17	0.2887	15.594	16.348	14.737	15.236	23.262
17	0.2897	10.335	10.809	12.770	9.714	18.509
17	0.2907	14.807	13.267	7.688	8.589	15.061
17	0.2917	10.604	10.016	10.469	5.315	4.609
17	0.2927	14.013	15.606	13.634	8.117	5.994
17	0.2937	13.558	18.901	12.263	15.591	7.154
17	0.2947	9.803	16.839	12.944	12.453	5.022
17	0.2957	9.216	7.321	9.892	8.813	18.480
17	0.2967	9.396	7.589	9.982	8.940	18.531
17	0.2977	9.150	6.718	5.352	7.831	12.498
17	0.2987	10.495	11.404	9.004	7.598	10.877
17	0.2997	19.166	13.811	13.838	12.377	13.127
17	0.3007	17.628	14.808	14.178	30.670	26.578
17	0.3017	15.631	16.601	11.546	14.314	10.690
17	0.3027	10.860	13.108	10.080	15.340	7.588
17	0.3037	16.769	17.585	17.727	8.578	9.749
17	0.3047	19.194	25.277	24.066	20.797	12.080
17	0.3057	21.102	24.742	19.383	9.633	14.822
17	0.3067	17.257	15.169	12.712	3.535	8.179
17	0.3077	12.440	12.296	9.717	3.807	20.653
17	0.3087	10.059	9.740	7.065	16.767	18.024
17	0.3097	10.478	12.502	8.910	21.003	20.760
17	0.3107	13.693	18.724	10.822	13.210	25.802
17	0.3117	11.383	17.488	12.746	16.235	27.909
17	0.3127	19.328	20.736	12.556	6.529	23.480
17	0.3137	13.634	7.814	11.236	11.084	19.469
17	0.3147	22.560	21.749	13.389	12.998	31.657
17	0.3157	25.106	25.271	19.101	8.606	11.924
17	0.3167	17.588	17.453	15.209	7.333	18.133
17	0.3177	15.827	20.946	10.984	10.534	15.018
17	0.3187	15.440	21.855	15.903	12.650	20.063
17	0.3197	12.908	17.732	17.319	14.062	16.516
17	0.3207	13.588	25.022	10.983	18.921	19.329

17	0.3217	17.683	15.409	19.291	11.914	12.672
17	0.3227	20.937	12.733	10.065	12.950	19.214
17	0.3237	10.670	13.622	4.332	19.768	16.641
17	0.3247	20.787	27.922	15.318	19.102	6.291
17	0.3257	17.255	26.566	14.085	14.895	6.069
17	0.3267	23.771	25.659	15.076	14.206	8.573
17	0.3277	22.678	25.229	16.699	21.870	12.192
17	0.3287	25.729	26.222	23.062	19.156	18.777
17	0.3297	16.478	19.380	14.190	21.569	17.340
17	0.3307	19.752	23.040	15.526	13.506	8.919
17	0.3317	22.472	23.502	14.230	9.925	15.010
17	0.3327	13.618	14.283	16.032	7.315	14.369
17	0.3337	8.338	4.493	7.587	4.813	8.977
17	0.3347	11.937	3.680	10.986	3.352	12.167
17	0.3357	18.144	12.479	12.680	9.666	14.999
17	0.3367	17.398	11.356	12.156	15.040	15.890
17	0.3377	18.077	13.169	13.417	13.116	16.179
17	0.3387	15.787	19.236	17.661	9.743	14.284
17	0.3397	15.662	18.997	21.270	11.640	16.748
17	0.3407	8.173	12.263	8.231	14.503	13.414
17	0.3417	18.319	15.640	13.441	10.531	7.985
17	0.3427	8.666	7.513	3.469	5.387	4.938
17	0.3437	10.611	11.779	9.550	11.481	3.032
17	0.3447	13.265	13.285	6.834	7.909	1.948
17	0.3457	14.470	13.833	7.141	10.399	5.960
17	0.3467	10.905	11.310	11.167	3.005	9.006
17	0.3477	20.408	14.867	9.931	12.023	27.979
17	0.3487	13.341	13.272	6.310	16.201	18.271
17	0.3497	4.842	3.432	3.615	1.871	8.086
17	0.3507	4.837	3.441	3.631	1.884	8.094
17	0.3517	9.489	3.864	5.269	4.456	6.319
17	0.3527	12.527	7.258	9.606	5.249	1.755
17	0.3537	18.232	16.423	14.749	22.798	13.171
17	0.3547	18.165	19.133	13.878	23.888	14.152
17	0.3557	20.569	18.673	16.154	21.208	23.643
17	0.3567	20.808	18.363	20.669	16.960	21.013
17	0.3577	18.004	15.235	13.930	15.293	6.290
17	0.3587	28.815	27.465	26.287	11.210	14.257
17	0.3597	6.664	8.926	9.425	4.576	10.149
17	0.3607	8.250	5.043	5.728	14.043	3.719
17	0.3617	23.937	29.046	24.871	20.065	11.361
17	0.3627	15.971	19.941	24.102	14.310	10.137
17	0.3637	20.948	17.433	12.407	13.971	7.948
17	0.3647	7.098	8.332	5.860	6.006	12.096
17	0.3657	18.018	16.146	16.647	19.326	14.442
17	0.3667	17.547	14.181	18.814	17.935	9.007
17	0.3677	16.268	15.679	11.481	16.843	16.761
17	0.3687	20.097	16.401	14.130	23.587	15.701
17	0.3697	17.272	12.100	11.258	7.811	7.379
17	0.3707	13.145	13.296	5.958	13.596	8.988

17	0.3717	14.488	15.484	12.245	12.236	6.069
17	0.3727	15.854	14.671	9.707	13.306	12.296
17	0.3737	8.113	6.829	2.268	12.878	9.062
17	0.3747	10.564	12.199	7.627	9.744	9.115
17	0.3757	17.839	17.877	16.816	8.307	16.602
17	0.3767	14.726	13.678	12.495	15.456	10.989
17	0.3777	16.085	11.959	11.843	19.982	8.453
17	0.3787	22.918	16.983	16.158	17.078	11.948
17	0.3797	13.323	11.873	13.005	13.131	9.744
17	0.3807	16.620	13.698	12.605	8.357	7.627
17	0.3817	14.125	11.819	8.566	14.872	7.142
17	0.3827	22.872	21.264	24.385	15.890	13.423
17	0.3837	28.443	25.311	28.272	13.926	14.817
17	0.3847	16.637	15.958	17.576	15.901	10.170
17	0.3857	19.890	14.067	17.158	11.413	16.681
17	0.3867	25.995	15.790	16.218	24.680	11.204
17	0.3877	11.597	9.844	9.625	14.153	17.241
17	0.3887	23.279	11.350	16.875	10.584	14.886
17	0.3897	19.047	12.687	12.504	12.514	19.214
17	0.3907	20.966	15.548	12.122	13.099	11.858
17	0.3917	19.497	18.400	10.823	17.677	13.657
17	0.3927	19.627	18.434	10.886	17.518	13.568
17	0.3937	19.757	18.460	10.945	17.362	13.483
17	0.3947	24.128	23.026	20.328	16.669	11.336
17	0.3957	20.667	12.924	16.068	9.956	7.516
17	0.3967	16.605	14.867	16.478	12.730	7.069
17	0.3977	15.918	7.923	16.513	12.707	13.214
17	0.3987	15.679	6.159	13.287	18.186	12.285
17	0.3997	13.985	10.706	8.767	11.956	14.487
17	0.4007	12.540	10.675	12.715	2.846	16.962
17	0.4017	23.620	21.691	27.759	6.783	8.722
17	0.4027	24.974	20.108	25.685	3.500	14.788
17	0.4037	24.985	19.866	25.537	3.449	14.799
17	0.4047	22.123	16.732	21.763	9.988	9.778
17	0.4057	18.824	15.763	10.921	13.954	8.817
17	0.4067	10.858	8.747	12.523	9.853	5.894
17	0.4077	16.127	14.016	11.631	9.810	5.834
17	0.4087	18.625	20.850	14.812	10.860	17.089
17	0.4097	16.149	11.897	7.152	7.734	19.076
17	0.4107	32.619	25.442	19.899	13.648	16.768
17	0.4117	30.504	20.699	20.933	14.462	12.348
17	0.4127	17.764	6.544	10.481	8.620	10.024
17	0.4137	13.092	3.489	4.963	9.644	12.265
17	0.4147	23.058	12.914	14.247	9.041	13.196
17	0.4157	24.459	18.463	21.003	12.549	10.388
17	0.4167	11.196	13.551	9.025	12.589	5.384
17	0.4177	18.526	14.126	14.176	15.881	8.207
17	0.4187	19.870	15.324	16.133	18.734	9.901
17	0.4197	16.898	11.424	11.141	14.053	19.829
17	0.4207	14.442	14.550	9.839	19.984	12.621

17	0.4217	21.106	21.031	19.929	17.805	12.831
17	0.4227	19.716	17.708	15.629	17.116	8.483
17	0.4237	15.691	17.100	17.940	12.375	10.048
17	0.4247	9.778	7.533	9.144	6.706	5.462
17	0.4257	13.110	12.362	19.728	6.603	10.394
17	0.4267	3.923	1.837	5.127	13.479	10.364
17	0.4277	14.434	9.888	12.121	10.102	9.805
17	0.4287	16.171	11.326	11.941	9.436	7.958
17	0.4297	23.275	20.618	18.714	16.270	14.863
17	0.4307	23.408	14.258	14.731	12.472	14.868
17	0.4317	18.560	10.606	18.037	16.768	16.706
17	0.4327	18.213	10.697	12.621	12.519	9.745
17	0.4337	15.184	10.066	10.856	4.518	9.779
17	0.4347	17.297	18.100	11.292	11.746	6.893
17	0.4357	17.407	11.712	12.300	9.925	15.438
17	0.4367	16.615	8.824	10.171	6.919	9.272
17	0.4377	18.944	9.617	12.365	9.990	8.373
17	0.4387	12.214	6.431	5.215	8.191	11.869
17	0.4397	9.795	6.138	9.632	5.353	9.070
17	0.4407	11.818	8.766	12.687	4.010	4.394
17	0.4417	12.141	15.630	7.016	16.396	7.737
17	0.4427	10.650	12.867	6.226	20.269	7.678
17	0.4437	20.419	12.194	11.153	12.431	11.774
17	0.4447	20.108	12.453	10.993	18.297	18.087
17	0.4457	11.085	9.634	6.205	8.790	8.816
17	0.4467	10.943	7.659	9.858	9.656	10.387
17	0.4477	22.872	11.102	20.553	8.038	10.774
17	0.4487	15.963	7.235	11.430	11.003	6.884
17	0.4497	20.248	8.427	12.765	9.878	8.562
17	0.4507	15.911	10.558	11.942	18.583	14.795
17	0.4517	13.526	7.048	10.265	10.264	10.216
17	0.4527	10.474	5.812	3.818	8.770	8.907
17	0.4537	27.473	23.116	26.668	16.811	9.909
17	0.4547	16.016	11.956	13.632	12.824	11.996
17	0.4557	14.906	9.266	9.690	11.229	20.101
17	0.4567	14.112	13.347	12.820	11.387	6.854
17	0.4577	19.208	12.229	11.621	6.175	11.618
17	0.4587	23.660	18.612	15.905	10.960	15.011
17	0.4597	16.576	9.991	6.954	10.260	11.185
17	0.4607	12.380	12.632	15.484	9.208	6.505
17	0.4617	13.030	7.502	10.067	7.149	8.670
17	0.4627	18.975	16.215	13.643	19.616	4.369
17	0.4637	21.964	19.305	20.043	18.606	6.462
17	0.4647	15.076	9.075	10.299	10.628	8.377
17	0.4657	14.708	12.192	11.588	6.750	7.513
17	0.4667	19.183	13.988	13.275	7.436	7.485
17	0.4677	25.049	23.615	20.962	16.327	14.064
17	0.4687	18.959	17.296	15.960	13.334	6.270
17	0.4697	22.793	16.206	15.395	13.205	10.816
17	0.4707	19.730	11.066	18.082	17.960	14.613

17	0.4717	18.688	11.655	13.552	10.312	20.963
17	0.4727	12.150	11.505	11.436	6.375	7.931
17	0.4737	11.523	18.846	18.734	17.604	12.300
17	0.4747	10.077	6.248	6.566	13.709	7.700
17	0.4757	12.935	7.928	10.449	11.081	11.388
17	0.4767	12.296	7.507	10.071	11.156	11.449
17	0.4777	12.546	7.577	9.746	7.633	9.230
17	0.4787	12.395	6.803	6.548	5.509	10.606
17	0.4797	5.356	4.156	5.186	11.455	7.361
17	0.4807	6.809	4.495	5.392	8.051	5.311
17	0.4817	5.716	5.116	5.845	7.977	4.082
17	0.4827	30.009	9.469	11.553	17.386	9.541
17	0.4837	7.095	5.742	5.063	10.491	9.132
17	0.4847	14.226	9.205	7.823	10.088	7.511
17	0.4857	22.130	14.083	13.230	14.931	15.863
17	0.4867	17.599	10.926	11.848	20.695	9.343
17	0.4877	19.871	11.799	14.435	14.176	14.646
17	0.4887	16.823	9.472	11.975	15.241	7.388
17	0.4897	14.019	6.496	14.663	18.141	7.057
17	0.4907	23.191	6.199	18.764	25.522	9.304
17	0.4917	23.106	6.211	18.632	25.453	9.244
17	0.4927	21.966	5.802	17.976	15.102	12.725
17	0.4937	22.846	7.313	14.559	17.166	7.980
17	0.4947	10.665	7.530	5.472	7.568	7.707
17	0.4957	16.614	12.852	11.062	12.610	8.067
17	0.4967	20.913	10.937	14.637	15.529	23.931
17	0.4977	15.163	7.732	7.418	19.357	12.750
17	0.4987	14.940	8.403	10.031	10.478	16.091
17	0.4997	22.938	16.259	18.792	10.289	13.278
17	0.5007	24.863	13.502	21.446	13.169	17.598
17	0.5017	16.547	12.427	11.759	10.544	18.641
17	0.5027	16.143	20.185	13.325	20.783	12.913
17	0.5037	20.610	10.569	13.018	5.785	12.500
17	0.5047	22.795	17.408	17.365	18.055	16.823
17	0.5057	22.803	17.351	17.367	18.033	16.801
17	0.5067	23.458	12.266	16.704	10.991	13.804
17	0.5077	20.479	14.958	14.168	11.039	8.041
17	0.5087	20.891	14.280	14.546	14.952	14.475
17	0.5097	21.807	11.534	8.385	11.924	6.968
17	0.5107	25.454	14.585	16.095	20.642	27.264
17	0.5117	14.191	5.512	8.389	10.875	12.770
17	0.5127	9.124	6.897	9.530	8.527	8.882
17	0.5137	7.178	3.694	5.467	6.071	16.931
17	0.5147	19.325	12.357	16.005	13.938	15.831
17	0.5157	24.161	9.065	10.464	13.973	28.287
17	0.5167	10.632	3.126	3.845	16.111	17.681
17	0.5177	21.798	8.931	15.192	14.989	11.325
17	0.5187	14.703	6.332	8.883	8.572	13.755
17	0.5197	13.222	5.545	8.467	9.446	12.977
17	0.5207	13.618	13.300	11.845	12.773	10.376

17	0.5217	9.502	9.500	10.514	12.223	13.388
17	0.5227	10.096	9.252	8.903	14.533	17.219
17	0.5237	10.578	8.817	8.061	12.929	15.689
17	0.5247	13.977	8.772	8.047	11.271	16.163
17	0.5257	15.060	4.725	7.279	9.870	16.725
17	0.5267	17.115	9.573	6.975	5.538	11.523
17	0.5277	18.266	9.394	6.378	7.348	7.707
17	0.5287	17.780	13.605	12.518	17.871	16.295
17	0.5297	21.843	21.005	15.477	16.540	18.715
17	0.5307	21.864	21.013	15.510	16.526	18.710
17	0.5317	17.255	19.034	9.681	24.527	14.091
17	0.5327	12.979	11.569	10.770	17.247	16.098
17	0.5337	25.015	16.354	16.244	20.849	13.082
17	0.5347	25.989	16.090	13.945	17.659	11.734
17	0.5357	24.193	18.162	14.553	8.735	10.610
17	0.5367	23.932	18.023	14.478	8.707	10.540
17	0.5377	12.997	9.733	8.623	6.528	11.941
17	0.5387	13.049	9.809	8.664	6.425	11.930
17	0.5397	9.702	7.844	8.178	8.740	12.682
17	0.5407	23.893	14.578	18.826	6.297	18.422
17	0.5417	20.657	13.289	13.299	12.626	19.194
17	0.5427	15.326	9.276	8.493	11.503	17.204
17	0.5437	7.294	7.304	6.889	14.087	23.928
17	0.5447	37.547	15.156	13.680	18.659	22.440
17	0.5457	21.259	11.604	14.668	7.339	18.881
17	0.5467	22.111	10.498	11.869	11.325	14.337
17	0.5477	6.251	3.516	2.429	9.054	6.359
17	0.5487	9.159	3.067	2.965	8.615	9.085
17	0.5497	8.311	3.318	2.523	10.413	8.674
17	0.5507	15.738	11.426	9.421	6.012	9.597
17	0.5517	15.236	11.521	13.048	6.957	5.633
17	0.5527	26.591	18.349	23.734	6.281	8.565
17	0.5537	36.212	20.810	28.885	19.086	11.633
17	0.5547	21.522	14.885	19.276	13.715	17.725
17	0.5557	21.379	14.758	17.668	16.666	15.631
17	0.5567	11.806	9.238	8.882	15.249	9.511
17	0.5577	18.752	9.379	13.608	10.501	15.544
17	0.5587	21.439	14.385	16.823	9.323	13.575
17	0.5597	24.270	13.282	14.993	15.939	19.619
17	0.5607	22.209	12.730	13.283	18.281	12.568
17	0.5617	16.847	17.843	17.612	23.783	12.286
17	0.5627	16.848	17.816	17.604	23.680	12.225
17	0.5637	21.029	25.276	13.436	18.272	13.852
17	0.5647	5.019	4.621	2.107	4.506	0.782
17	0.5657	14.724	6.196	6.772	11.336	9.938
17	0.5667	14.711	6.151	6.777	11.252	9.871
17	0.5677	14.699	6.107	6.783	11.170	9.810
17	0.5687	9.152	5.595	5.405	19.867	11.588
17	0.5697	17.329	8.317	11.151	17.187	11.213
17	0.5707	21.410	6.589	14.155	14.022	10.534

17	0.5717	19.666	15.866	14.604	12.831	12.038
17	0.5727	19.922	19.242	20.323	14.016	2.971
17	0.5737	32.315	12.446	18.372	10.650	12.627
17	0.5747	20.249	13.384	10.422	12.461	13.881
17	0.5757	12.914	12.137	6.977	18.299	9.710
17	0.5767	16.596	13.999	8.728	16.181	14.040
17	0.5777	8.453	2.988	4.316	11.626	9.751
17	0.5787	9.319	4.719	6.530	16.009	10.728
17	0.5797	14.638	10.388	12.116	10.851	14.363
17	0.5807	21.623	11.189	15.030	9.943	25.937
17	0.5817	18.530	17.606	18.275	11.921	13.175
17	0.5827	17.558	16.988	13.339	10.352	10.158
17	0.5837	20.538	16.146	11.869	5.795	9.217
17	0.5847	15.087	11.707	14.108	12.038	12.008
17	0.5857	12.027	15.840	12.379	18.350	13.910
17	0.5867	12.822	4.255	5.035	8.293	16.539
17	0.5877	16.723	8.738	10.443	15.256	12.148
17	0.5887	19.887	11.038	11.155	11.001	10.379
17	0.5897	12.847	11.126	9.356	11.037	12.288
17	0.5907	16.660	5.947	7.273	14.998	20.307
17	0.5917	22.078	16.785	14.230	8.964	16.682
17	0.5927	12.620	11.998	9.378	11.474	12.017
17	0.5937	22.147	20.364	18.655	13.414	12.472
17	0.5947	19.265	8.690	5.611	14.226	13.962
17	0.5957	14.097	9.059	5.546	23.339	17.328
17	0.5967	13.088	19.008	10.028	23.888	13.008
17	0.5977	12.399	15.356	15.636	14.563	12.909
17	0.5987	6.567	13.362	14.848	6.459	7.498
17	0.5997	4.028	7.359	5.962	14.809	7.366
17	0.6007	8.454	9.977	8.659	13.148	11.337
17	0.6017	7.219	8.483	7.837	10.154	3.750
17	0.6027	14.465	18.237	22.458	11.528	15.598
17	0.6037	8.625	5.155	9.675	13.088	12.739
17	0.6047	10.879	11.560	10.315	18.926	9.953
17	0.6057	8.921	8.328	8.304	16.287	11.206
17	0.6067	8.374	12.283	10.115	15.063	13.894
17	0.6077	13.061	17.679	16.413	14.583	15.371
17	0.6087	18.436	23.972	24.762	11.922	23.549
17	0.6097	5.577	7.067	10.096	5.506	17.081
17	0.6107	6.978	2.569	7.619	10.343	15.670
17	0.6117	8.019	6.967	10.940	14.009	14.778
17	0.6127	5.310	4.229	7.087	12.939	15.286
17	0.6137	7.664	21.694	12.631	13.787	16.945
17	0.6147	6.789	9.427	5.425	4.356	8.899
17	0.6157	12.140	14.791	9.403	5.379	6.532
17	0.6167	3.648	4.047	6.796	3.656	5.585
17	0.6177	3.543	9.771	8.694	9.044	15.547
17	0.6187	3.287	6.942	10.422	4.308	16.101
17	0.6197	6.797	7.458	10.016	12.656	20.868
17	0.6207	8.081	16.859	12.606	15.789	10.142

17	0.6217	13.885	21.225	14.990	16.164	12.624
17	0.6227	13.238	20.318	19.131	18.473	17.184
17	0.6237	19.374	24.696	24.631	9.858	13.755
17	0.6247	25.027	31.845	32.907	14.804	26.184
17	0.6257	10.474	12.679	19.840	13.397	15.155
17	0.6267	9.787	10.408	11.889	9.549	12.585
17	0.6277	7.955	10.130	6.695	10.746	8.311
17	0.6287	4.687	5.258	7.033	15.769	18.899
17	0.6297	16.515	14.275	12.837	12.178	9.595
17	0.6307	7.538	8.148	9.355	8.803	4.763
17	0.6317	4.806	6.187	6.108	11.422	7.298
17	0.6327	1.349	5.900	6.000	9.425	6.990
17	0.6337	7.287	7.852	14.210	15.701	12.762
17	0.6347	11.457	11.052	13.127	10.417	6.981
17	0.6357	6.838	11.921	10.363	15.479	14.660
17	0.6367	10.747	6.804	8.056	19.403	11.544
17	0.6377	12.399	13.384	7.045	22.994	24.132
17	0.6387	8.682	5.694	6.404	27.550	19.831
17	0.6397	9.684	11.987	17.082	11.480	21.555
17	0.6407	11.539	14.222	18.794	19.894	19.059
17	0.6417	5.904	11.599	8.072	19.227	9.783
17	0.6427	16.189	19.559	18.242	20.406	19.799
17	0.6437	8.872	13.806	16.856	14.770	23.122
17	0.6447	8.555	5.823	4.385	15.178	12.782
17	0.6457	5.954	7.428	3.292	14.571	8.142
17	0.6467	21.232	9.735	12.944	14.418	25.560
17	0.6477	15.510	15.633	16.415	14.301	19.358
17	0.6487	16.305	15.774	16.602	14.374	19.319
17	0.6497	17.768	9.963	13.421	8.795	4.639
17	0.6507	13.111	10.463	12.037	9.782	18.198
17	0.6517	15.314	12.976	11.029	7.346	20.319
17	0.6527	12.550	10.503	8.614	7.422	16.190
17	0.6537	6.319	3.474	4.301	5.213	5.926
17	0.6547	16.081	16.994	12.691	12.484	8.366
17	0.6557	11.039	8.924	9.102	8.949	23.715
17	0.6567	10.973	13.712	10.940	14.851	14.550
17	0.6577	15.777	19.561	22.428	16.006	18.631
17	0.6587	13.270	15.573	17.584	17.151	19.387
17	0.6597	17.828	14.231	17.210	13.814	13.570
17	0.6607	8.863	13.914	8.623	19.809	22.827
17	0.6617	15.531	16.449	20.495	34.602	27.058
17	0.6627	14.905	15.393	21.076	14.850	21.316
17	0.6637	10.581	9.937	10.716	12.237	11.036
17	0.6647	9.705	9.172	10.561	7.237	9.108
17	0.6657	7.992	9.296	8.560	15.466	14.491
17	0.6667	15.636	15.125	11.049	20.216	16.113
17	0.6677	15.848	18.061	13.680	20.186	13.972
17	0.6687	8.408	6.533	9.260	18.294	16.086
17	0.6697	16.286	17.877	16.642	17.237	12.740
17	0.6707	5.863	4.296	6.127	6.833	9.749

17	0.6717	11.394	7.004	9.088	14.075	10.086
17	0.6727	14.302	8.342	9.718	12.657	10.664
17	0.6737	22.557	16.404	21.631	10.437	8.841
17	0.6747	16.029	13.876	14.417	7.900	6.544
17	0.6757	21.129	18.297	26.071	2.483	5.282
17	0.6767	3.481	2.145	1.283	5.370	16.045
17	0.6777	12.760	9.357	13.389	14.530	16.964
17	0.6787	11.272	11.399	16.084	14.902	12.473
17	0.6797	13.050	10.678	19.557	7.708	7.894
17	0.6807	10.644	7.180	12.455	6.492	4.851
17	0.6817	6.952	5.708	8.167	3.079	10.086
17	0.6827	10.482	8.258	8.896	7.279	10.518
17	0.6837	11.811	10.930	9.410	16.330	19.640
17	0.6847	15.665	13.094	12.571	14.231	14.109
17	0.6857	8.913	10.364	9.215	10.796	11.579
17	0.6867	21.010	18.818	23.910	10.309	12.378
17	0.6877	9.660	15.579	13.374	11.478	13.511
17	0.6887	7.942	8.288	10.861	5.493	7.422
17	0.6897	15.909	11.890	19.773	7.885	9.397
17	0.6907	15.049	10.804	15.504	11.202	7.918
17	0.6917	10.525	11.276	10.088	17.799	20.448
17	0.6927	12.265	9.908	10.598	10.095	14.916
17	0.6937	18.012	12.359	21.535	6.579	10.060
17	0.6947	18.203	12.404	21.695	6.576	10.050
17	0.6957	8.962	7.585	8.779	8.319	14.865
17	0.6967	12.882	13.033	19.351	5.635	9.805
17	0.6977	18.092	10.093	15.796	10.884	7.645
17	0.6987	22.196	18.106	29.349	7.985	7.821
17	0.6997	21.598	18.715	25.467	6.468	7.623
17	0.7007	22.150	20.468	30.572	4.348	10.643
17	0.7017	21.883	20.418	30.368	4.361	10.514
17	0.7027	13.723	14.862	19.060	8.235	15.601
17	0.7037	7.113	6.755	10.648	15.112	19.550
17	0.7047	6.334	7.020	8.772	9.718	9.775
17	0.7057	6.282	6.848	8.674	9.581	9.745
17	0.7067	27.202	22.210	21.753	11.652	20.972
17	0.7077	13.178	10.132	8.876	11.339	13.912
17	0.7087	8.179	8.592	7.205	10.667	12.290
17	0.7097	10.502	9.709	9.828	14.230	11.503
17	0.7107	9.383	8.293	8.419	2.662	9.216
17	0.7117	6.277	4.537	6.748	2.239	4.047
17	0.7127	12.052	12.190	12.072	13.631	9.759
17	0.7137	20.277	11.546	16.669	13.225	11.856
17	0.7147	8.665	7.746	7.712	16.239	12.884
17	0.7157	9.388	10.298	10.964	8.379	8.932
17	0.7167	6.710	6.681	8.537	11.913	6.900
17	0.7177	0.151	0.682	0.307	0.760	0.119
17	0.7187	3.893	3.070	3.237	0.210	1.237
18	0.0014	0.000	0.000	0.000	0.000	0.000
18	0.0024	12.256	12.629	16.951	11.541	18.625

18	0.0034	9.889	11.227	7.948	8.429	11.977
18	0.0044	12.548	14.125	21.877	12.331	19.678
18	0.0054	14.314	25.212	22.052	14.753	26.375
18	0.0064	5.018	9.759	7.906	17.587	17.122
18	0.0074	8.113	4.750	6.487	19.299	16.069
18	0.0084	22.966	29.678	23.308	24.174	9.480
18	0.0094	20.132	27.955	24.569	19.139	15.853
18	0.0104	11.813	18.279	18.471	15.502	17.499
18	0.0114	9.771	14.722	14.207	11.309	11.921
18	0.0124	12.217	12.426	12.426	17.890	12.649
18	0.0134	9.133	17.800	12.312	26.019	18.517
18	0.0144	6.365	7.328	6.060	8.696	12.243
18	0.0154	12.365	16.908	17.559	25.606	28.502
18	0.0164	9.724	13.272	18.385	19.259	19.686
18	0.0174	10.665	15.076	17.175	22.160	25.928
18	0.0184	15.487	13.877	19.457	15.263	18.459
18	0.0194	7.899	14.170	15.371	9.340	12.271
18	0.0204	10.747	11.267	9.650	13.802	13.784
18	0.0214	11.593	16.020	12.937	18.559	13.632
18	0.0224	9.356	11.622	10.634	11.278	7.246
18	0.0234	6.641	3.337	6.919	2.923	6.902
18	0.0244	3.538	2.170	6.885	6.419	6.675
18	0.0254	4.974	9.316	13.505	8.737	5.452
18	0.0264	8.931	13.813	16.345	9.423	9.704
18	0.0274	7.221	4.740	9.517	19.399	12.897
18	0.0284	13.926	28.567	30.288	12.812	16.275
18	0.0294	9.814	18.769	19.055	17.746	22.621
18	0.0304	12.818	18.516	18.286	14.323	23.067
18	0.0314	4.530	10.736	7.986	13.675	13.436
18	0.0324	9.376	13.230	14.533	18.609	12.395
18	0.0334	11.747	18.004	18.469	19.942	17.520
18	0.0344	11.583	16.523	16.510	17.971	11.635
18	0.0354	6.162	13.740	11.552	12.246	9.469
18	0.0364	6.213	13.752	11.652	12.266	9.474
18	0.0374	6.840	15.802	13.258	20.849	16.126
18	0.0384	8.971	18.687	13.373	17.000	11.657
18	0.0394	13.828	24.461	23.504	19.480	20.852
18	0.0404	18.477	25.741	23.394	24.655	14.932
18	0.0414	16.128	17.843	20.437	15.920	24.382
18	0.0424	17.127	17.383	25.596	16.620	20.211
18	0.0434	7.704	11.482	19.542	7.709	10.097
18	0.0444	13.198	27.020	18.377	24.387	22.101
18	0.0454	15.028	24.101	23.068	26.677	11.084
18	0.0464	15.160	26.449	25.273	33.566	11.409
18	0.0474	7.870	8.083	11.147	37.892	11.272
18	0.0484	8.956	6.246	11.235	35.064	12.688
18	0.0494	11.031	17.981	17.065	19.501	20.652
18	0.0504	8.750	20.058	18.430	15.634	13.001
18	0.0514	3.749	11.957	7.680	16.988	6.507
18	0.0524	7.202	11.784	5.974	19.601	9.298

18	0.0534	6.984	11.635	5.822	19.491	9.200
18	0.0544	15.665	17.346	18.878	19.827	9.216
18	0.0554	10.527	17.156	19.546	16.712	18.366
18	0.0564	6.248	9.912	13.924	8.307	9.606
18	0.0574	9.191	12.732	13.178	6.474	13.425
18	0.0584	6.210	16.812	11.608	12.373	16.296
18	0.0594	6.503	10.156	13.073	12.353	12.447
18	0.0604	20.179	31.262	27.330	18.542	10.685
18	0.0614	14.656	17.552	19.379	12.562	19.222
18	0.0624	14.675	12.315	17.875	8.703	10.995
18	0.0634	18.245	20.543	18.604	10.529	10.197
18	0.0644	9.930	13.485	9.401	16.773	11.084
18	0.0654	7.446	14.066	11.478	10.848	4.064
18	0.0664	7.666	9.377	9.631	10.911	5.749
18	0.0674	12.671	21.569	13.680	10.590	9.993
18	0.0684	10.095	18.048	13.684	13.315	6.634
18	0.0694	15.229	20.754	18.540	9.877	12.062
18	0.0704	8.313	7.675	8.352	6.259	10.077
18	0.0714	15.126	19.604	17.434	15.975	13.850
18	0.0724	22.764	22.002	18.783	13.307	18.599
18	0.0734	19.230	22.556	16.594	21.413	30.154
18	0.0744	9.535	11.793	10.803	8.044	3.801
18	0.0754	11.240	15.541	12.977	13.386	7.562
18	0.0764	15.547	22.486	21.358	9.729	7.962
18	0.0774	14.309	19.332	21.697	10.897	13.064
18	0.0784	3.428	3.774	2.541	5.691	9.081
18	0.0794	6.851	5.752	6.377	2.717	4.081
18	0.0804	12.929	9.504	19.179	7.562	12.321
18	0.0814	17.738	21.192	21.770	10.685	21.193
18	0.0824	15.674	20.290	17.277	13.333	30.573
18	0.0834	9.929	19.894	25.204	18.986	20.099
18	0.0844	16.567	19.876	24.563	14.627	11.957
18	0.0854	13.646	22.789	20.452	20.009	5.616
18	0.0864	13.934	12.495	9.020	8.171	5.643
18	0.0874	11.928	19.533	18.099	11.844	14.924
18	0.0884	11.461	16.885	17.525	11.542	14.099
18	0.0894	11.889	16.598	17.766	11.454	15.371
18	0.0904	8.967	10.587	14.726	12.657	13.704
18	0.0914	11.276	8.931	10.407	18.123	19.542
18	0.0924	11.246	15.002	15.288	21.853	19.231
18	0.0934	11.425	15.942	17.690	24.760	18.620
18	0.0944	7.799	7.580	12.937	19.242	17.522
18	0.0954	6.410	5.367	6.850	6.376	19.566
18	0.0964	12.625	21.515	19.718	10.428	13.778
18	0.0974	11.641	16.727	14.390	12.778	13.680
18	0.0984	11.751	4.079	11.517	8.235	11.229
18	0.0994	13.082	17.723	15.876	15.528	23.390
18	0.1004	9.343	5.669	7.637	20.140	28.622
18	0.1014	9.677	9.497	8.669	11.746	26.364
18	0.1024	12.132	20.199	18.329	27.832	34.382

18	0.1034	4.051	11.872	7.624	21.659	27.751
18	0.1044	5.010	5.147	2.549	8.746	20.576
18	0.1054	7.654	6.540	8.122	13.398	26.724
18	0.1064	6.684	7.451	5.870	5.360	18.889
18	0.1074	6.544	4.536	4.111	8.055	15.615
18	0.1084	11.468	7.070	8.342	16.235	22.486
18	0.1094	8.159	11.281	10.935	20.438	23.634
18	0.1104	13.637	19.241	16.274	23.999	21.023
18	0.1114	15.708	14.324	17.158	25.203	12.181
18	0.1124	23.669	12.661	25.981	18.057	16.962
18	0.1134	8.753	11.837	16.077	22.719	20.482
18	0.1144	12.148	8.928	14.900	9.225	14.440
18	0.1154	23.487	26.112	24.966	18.240	13.729
18	0.1164	23.562	26.126	24.988	18.260	13.731
18	0.1174	18.514	29.071	20.939	13.288	11.652
18	0.1184	18.579	29.278	20.942	13.370	11.652
18	0.1194	13.663	14.196	16.330	21.865	8.694
18	0.1204	11.104	8.444	8.090	15.225	8.796
18	0.1214	13.058	11.328	19.770	10.613	9.518
18	0.1224	8.901	8.128	9.475	12.079	13.126
18	0.1234	25.648	9.919	21.904	15.725	13.422
18	0.1244	21.044	10.672	18.634	15.986	18.579
18	0.1254	13.340	11.239	15.331	9.074	10.544
18	0.1264	15.704	18.520	16.828	12.473	15.058
18	0.1274	20.447	16.019	17.384	10.944	7.352
18	0.1284	7.526	7.370	10.122	14.013	22.813
18	0.1294	7.598	3.939	5.831	11.192	11.354
18	0.1304	18.155	15.649	15.758	15.001	17.375
18	0.1314	9.472	13.826	10.861	9.637	13.418
18	0.1324	17.106	18.070	19.136	10.683	18.119
18	0.1334	18.891	19.087	23.692	5.020	8.785
18	0.1344	12.910	7.425	13.826	8.402	11.143
18	0.1354	12.837	6.697	16.197	16.980	13.928
18	0.1364	13.550	11.856	17.164	14.945	13.667
18	0.1374	7.668	3.694	9.326	5.978	8.749
18	0.1384	20.148	4.784	17.422	5.977	0.750
18	0.1394	16.390	4.229	15.132	8.988	10.913
18	0.1404	11.031	17.739	14.896	8.804	13.211
18	0.1414	13.427	16.711	18.156	14.576	7.614
18	0.1424	6.824	6.508	8.946	4.891	4.013
18	0.1434	11.395	8.701	9.625	4.868	5.452
18	0.1444	11.402	8.704	9.631	4.851	5.441
18	0.1454	21.841	17.583	17.904	12.612	17.310
18	0.1464	21.829	17.559	17.885	12.601	17.315
18	0.1474	11.088	11.300	14.484	12.333	18.537
18	0.1484	10.404	12.651	15.093	12.819	16.825
18	0.1494	13.548	21.133	17.393	12.858	16.415
18	0.1504	13.735	17.822	18.072	18.653	11.317
18	0.1514	10.149	11.962	14.050	8.159	7.921
18	0.1524	34.825	16.203	29.372	24.062	17.893

18	0.1534	22.524	18.704	27.531	14.583	24.235
18	0.1544	16.094	16.837	13.817	9.204	16.713
18	0.1554	14.767	16.939	16.553	22.750	24.344
18	0.1564	12.507	8.925	11.540	15.846	20.314
18	0.1574	11.372	10.711	14.153	16.624	21.626
18	0.1584	16.233	18.669	21.879	13.346	22.360
18	0.1594	14.610	12.009	21.592	9.305	13.827
18	0.1604	17.311	16.961	19.286	20.862	26.896
18	0.1614	11.885	8.230	12.192	13.021	14.115
18	0.1624	11.085	12.558	11.906	15.232	17.794
18	0.1634	15.173	10.207	15.131	13.055	15.052
18	0.1644	10.923	9.931	13.468	14.658	13.967
18	0.1654	7.798	12.133	13.009	18.083	11.884
18	0.1664	16.120	14.137	16.559	14.207	28.434
18	0.1674	13.857	12.091	10.582	9.395	11.603
18	0.1684	14.110	9.029	10.643	8.963	23.582
18	0.1694	8.773	11.688	11.390	14.123	18.733
18	0.1704	11.938	10.935	9.902	12.796	18.633
18	0.1714	19.996	14.969	21.180	14.205	16.890
18	0.1724	14.842	15.975	18.344	13.967	17.219
18	0.1734	23.081	23.694	25.768	23.589	25.962
18	0.1744	15.101	15.696	17.509	13.209	13.483
18	0.1754	16.791	13.914	14.640	19.600	20.935
18	0.1764	30.926	19.047	24.771	25.825	21.984
18	0.1774	18.593	16.053	13.462	23.579	19.034
18	0.1784	16.334	15.236	16.448	32.895	23.314
18	0.1794	12.822	9.849	11.579	28.896	21.060
18	0.1804	8.592	8.770	4.839	14.204	14.129
18	0.1814	14.638	20.048	11.897	29.914	15.758
18	0.1824	22.362	19.903	18.575	11.737	16.376
18	0.1834	32.107	19.567	28.367	18.956	16.537
18	0.1844	22.042	20.076	19.065	22.349	24.456
18	0.1854	18.397	11.146	15.857	27.865	20.002
18	0.1864	22.766	17.582	18.742	22.163	12.169
18	0.1874	16.419	9.528	12.483	14.519	14.735
18	0.1884	22.713	18.567	18.301	20.194	19.730
18	0.1894	22.834	18.529	18.298	20.175	19.712
18	0.1904	13.900	10.699	12.777	14.443	21.216
18	0.1914	11.213	14.058	9.936	14.322	13.281
18	0.1924	14.767	13.972	11.972	29.079	21.792
18	0.1934	19.142	10.291	11.766	18.964	14.248
18	0.1944	20.449	14.328	19.324	13.642	20.583
18	0.1954	8.560	20.199	10.994	19.969	15.998
18	0.1964	4.487	14.563	8.362	12.842	21.645
18	0.1974	12.703	13.113	9.750	27.918	14.126
18	0.1984	17.130	18.312	12.731	26.308	25.329
18	0.1994	16.353	15.183	11.478	23.930	6.461
18	0.2004	41.391	16.379	25.633	23.539	24.911
18	0.2014	21.761	10.289	9.999	29.109	38.760
18	0.2024	13.801	11.230	9.007	16.757	15.805

18	0.2034	12.426	12.263	8.113	19.060	12.185
18	0.2044	12.141	9.023	10.065	27.874	22.438
18	0.2054	6.645	12.036	9.045	22.369	14.497
18	0.2064	22.477	11.755	13.383	17.507	24.914
18	0.2074	15.339	9.012	10.482	19.010	27.810
18	0.2084	15.871	13.627	15.321	16.833	26.829
18	0.2094	14.744	17.326	16.233	13.095	11.494
18	0.2104	3.420	7.397	3.460	11.286	9.964
18	0.2114	3.280	4.468	3.083	8.278	7.100
18	0.2124	10.488	7.487	9.690	21.859	13.602
18	0.2134	8.920	9.866	12.818	13.628	19.532
18	0.2144	8.257	7.771	11.258	18.091	31.589
18	0.2154	20.642	20.816	22.505	16.313	10.928
18	0.2164	38.111	17.137	28.215	23.806	21.015
18	0.2174	43.674	21.014	32.095	24.559	8.592
18	0.2184	11.965	7.669	11.178	14.807	5.522
18	0.2194	13.836	9.548	9.938	17.063	11.855
18	0.2204	7.688	9.489	9.597	6.232	13.142
18	0.2214	12.553	6.581	10.284	11.428	18.842
18	0.2224	10.036	11.010	7.775	22.453	19.089
18	0.2234	13.175	12.126	10.491	13.000	11.753
18	0.2244	16.152	11.245	16.551	13.644	11.223
18	0.2254	14.684	8.301	14.245	28.491	17.535
18	0.2264	22.900	13.472	22.059	11.894	14.059
18	0.2274	21.614	12.962	19.534	13.355	8.955
18	0.2284	22.388	7.673	16.909	30.058	23.274
18	0.2294	18.049	10.776	17.165	18.664	12.399
18	0.2304	10.545	6.643	12.306	15.647	3.923
18	0.2314	11.202	3.712	7.149	10.465	3.396
18	0.2324	6.362	4.759	4.209	15.880	5.266
18	0.2334	9.674	8.397	8.994	20.081	8.727
18	0.2344	18.701	9.818	9.238	29.040	25.949
18	0.2354	6.848	5.902	3.384	10.322	7.523
18	0.2364	8.490	8.181	6.621	5.002	5.257
18	0.2374	8.575	4.718	3.987	4.089	6.854
18	0.2384	12.859	3.801	4.712	4.471	4.695
18	0.2394	15.176	6.543	5.613	5.078	6.587
18	0.2404	15.416	5.894	6.732	11.582	10.661
18	0.2414	12.925	5.588	6.797	17.809	8.916
18	0.2424	15.837	6.250	9.587	16.657	12.529
18	0.2434	19.825	7.813	10.739	10.221	6.514
18	0.2444	13.949	8.080	7.090	15.195	8.063
18	0.2454	12.652	5.980	6.244	5.982	7.311
18	0.2464	22.159	5.493	14.152	14.049	7.274
18	0.2474	12.927	8.200	9.947	11.472	8.621
18	0.2484	34.784	16.752	27.802	25.360	11.470
18	0.2494	20.651	13.720	11.858	13.232	9.366
18	0.2504	19.267	9.823	12.631	17.849	11.145
18	0.2514	19.044	9.666	12.479	17.848	11.109
18	0.2524	7.843	5.253	3.146	14.419	11.689

18	0.2534	7.381	5.785	5.438	9.806	8.333
18	0.2544	14.814	9.405	7.785	9.986	8.221
18	0.2554	19.807	10.376	11.660	16.118	7.495
18	0.2564	13.744	10.550	12.797	17.940	14.564
18	0.2574	4.027	7.300	3.466	12.245	9.764
18	0.2584	9.836	8.030	5.115	17.066	13.070
18	0.2594	27.046	9.544	17.467	23.984	27.281
18	0.2604	19.967	11.499	16.720	17.044	13.711
18	0.2614	19.267	14.120	8.044	21.969	19.164
18	0.2624	19.413	10.107	12.697	16.184	8.955
18	0.2634	11.651	4.727	3.439	15.002	14.043
18	0.2644	15.875	8.043	6.062	22.790	11.458
18	0.2654	6.650	6.470	5.705	23.209	10.976
18	0.2664	8.391	6.847	7.800	14.977	8.776
18	0.2674	13.781	6.871	10.228	10.766	11.329
18	0.2684	20.212	17.833	12.465	37.583	15.016
18	0.2694	11.179	3.652	3.063	17.335	16.721
18	0.2704	14.750	5.112	16.967	17.563	14.015
18	0.2714	21.758	5.765	18.784	19.321	13.745
18	0.2724	15.078	11.136	11.853	16.325	12.673
18	0.2734	8.845	6.962	8.115	21.902	17.138
18	0.2744	7.297	14.281	8.800	17.548	6.671
18	0.2754	13.077	9.201	12.147	24.118	13.736
18	0.2764	1.571	4.198	3.654	3.553	10.885
18	0.2774	9.770	7.258	8.935	9.527	8.608
18	0.2784	12.261	5.438	10.821	8.914	12.465
18	0.2794	12.330	10.623	14.361	15.565	15.803
18	0.2804	19.062	13.957	17.086	23.416	7.205
18	0.2814	14.667	16.699	20.125	22.403	19.859
18	0.2824	12.145	9.179	18.840	25.433	17.182
18	0.2834	10.133	9.993	13.546	16.302	11.492
18	0.2844	10.661	9.118	8.761	25.437	12.625
18	0.2854	12.943	10.715	11.085	20.594	15.686
18	0.2864	15.670	9.070	11.202	17.479	13.098
18	0.2874	10.062	8.681	9.095	18.819	16.818
18	0.2884	6.807	8.613	7.868	18.032	14.378
18	0.2894	12.996	10.957	12.301	17.156	11.991
18	0.2904	7.526	6.662	7.413	19.519	10.897
18	0.2914	9.116	4.893	4.626	7.612	8.780
18	0.2924	10.744	9.806	8.983	14.974	11.051
18	0.2934	10.356	6.772	6.114	12.219	17.456
18	0.2944	6.949	5.118	8.116	18.584	29.757
18	0.2954	17.661	9.011	13.268	16.972	21.058
18	0.2964	8.098	7.958	7.496	14.420	17.949
18	0.2974	19.330	18.283	26.901	17.227	17.645
18	0.2984	13.914	15.099	21.820	19.988	7.892
18	0.2994	15.363	11.607	15.030	9.166	23.470
18	0.3004	15.736	10.656	19.181	15.906	22.832
18	0.3014	15.033	12.716	17.339	9.172	14.321
18	0.3024	16.411	14.430	22.911	16.360	12.364

18	0.3034	20.594	18.182	26.159	21.525	22.277
18	0.3044	11.431	7.677	14.333	5.267	9.824
18	0.3054	6.306	7.371	12.304	3.076	6.918
18	0.3064	10.593	13.088	15.611	7.904	8.711
18	0.3074	13.338	10.054	12.364	20.578	17.654
18	0.3084	11.818	11.759	11.216	13.152	18.060
18	0.3094	11.408	11.394	10.796	12.676	19.536
18	0.3104	4.476	7.863	8.985	12.439	4.713
18	0.3114	16.197	15.141	19.834	12.591	9.028
18	0.3124	16.510	9.544	9.972	14.163	18.335
18	0.3134	10.009	16.422	21.766	7.331	11.175
18	0.3144	18.805	17.251	24.011	23.300	15.066
18	0.3154	14.070	13.700	21.318	7.066	4.403
18	0.3164	14.700	16.081	19.670	13.309	9.248
18	0.3174	12.753	17.649	17.473	18.317	9.208
18	0.3184	8.491	12.408	11.332	17.210	13.838
18	0.3194	12.114	10.987	16.061	13.709	20.437
18	0.3204	7.782	9.709	11.039	17.786	13.163
18	0.3214	6.546	11.886	8.651	9.700	7.126
18	0.3224	12.909	11.593	11.530	16.174	18.550
18	0.3234	14.860	11.421	21.253	13.220	27.159
18	0.3244	12.196	14.895	18.580	6.954	22.714
18	0.3254	10.937	7.647	7.629	8.804	10.637
18	0.3264	11.784	13.037	17.888	19.314	10.077
18	0.3274	13.159	17.383	19.479	30.862	29.196
18	0.3284	9.728	20.024	27.389	19.872	22.093
18	0.3294	4.799	11.107	12.417	8.912	5.677
18	0.3304	18.752	18.862	19.694	10.369	15.987
18	0.3314	10.510	8.506	13.106	11.227	22.622
18	0.3324	19.882	21.070	29.209	8.954	23.817
18	0.3334	12.796	13.711	16.191	16.846	10.369
18	0.3344	21.812	10.431	19.743	15.661	17.546
18	0.3354	14.976	5.260	14.662	18.989	11.525
18	0.3364	17.232	9.608	15.209	22.378	16.245
18	0.3374	8.025	15.549	12.989	19.444	15.979
18	0.3384	4.954	13.541	8.953	17.774	22.151
18	0.3394	7.507	10.374	12.057	13.145	10.607
18	0.3404	10.940	8.802	7.854	12.052	12.093
18	0.3414	8.510	5.878	7.942	5.508	2.067
18	0.3424	9.305	7.874	14.559	3.145	1.118
18	0.3434	7.161	7.911	9.475	3.870	9.899
18	0.3444	7.881	8.273	10.311	6.887	12.323
18	0.3454	2.999	7.952	6.546	5.454	6.839
18	0.3464	2.979	7.918	6.525	5.440	6.830
18	0.3474	4.495	7.886	6.889	6.038	9.255
18	0.3484	6.894	5.210	11.289	10.028	1.684
18	0.3494	7.641	5.044	11.538	11.251	1.576
18	0.3504	6.160	9.069	10.979	4.127	6.547
18	0.3514	6.800	8.314	9.264	4.522	2.977
18	0.3524	3.418	8.403	8.254	6.721	11.497

18	0.3534	4.828	6.344	5.973	5.245	12.067
18	0.3544	2.920	8.792	4.675	8.973	14.358
18	0.3554	3.858	3.934	7.868	6.034	6.331
18	0.3564	2.805	1.984	5.450	5.396	4.151
18	0.3574	7.237	8.519	17.392	9.063	21.392
18	0.3584	7.162	6.241	10.968	5.716	11.340
18	0.3594	2.084	2.631	2.448	2.090	16.834
18	0.3604	0.923	0.559	1.193	2.338	9.447
18	0.3614	4.736	6.118	4.366	14.825	14.117
18	0.3624	7.431	9.964	12.601	7.723	12.428
18	0.3634	11.142	11.179	13.253	11.689	11.495
18	0.3644	14.885	12.658	19.053	4.641	6.093
18	0.3654	19.211	16.258	29.678	3.442	7.805
18	0.3664	15.546	14.326	19.027	11.816	11.117
18	0.3674	6.818	6.634	6.992	17.138	19.041
18	0.3684	16.566	19.130	20.922	16.741	10.124
18	0.3694	14.555	16.860	17.259	15.646	13.215
18	0.3704	14.552	16.879	17.298	15.743	13.199
18	0.3714	6.881	10.737	5.588	14.491	17.168
18	0.3724	22.328	16.013	19.601	7.368	15.963
18	0.3734	6.895	7.069	9.195	9.427	7.756
18	0.3744	4.658	4.177	4.610	4.086	13.109
18	0.3754	11.699	11.691	12.164	7.545	8.846
18	0.3764	6.186	8.426	8.031	7.075	7.687
18	0.3774	8.185	9.613	7.785	2.650	2.663
18	0.3784	2.641	2.357	3.775	2.064	1.255
18	0.3794	4.777	4.880	7.035	7.661	5.902
18	0.3804	3.615	2.314	7.532	5.801	12.658
18	0.3814	9.825	8.821	14.764	6.870	8.668
18	0.3824	18.668	13.127	20.096	17.015	11.836
18	0.3834	7.702	9.956	10.567	16.667	12.019
18	0.3844	4.160	6.137	6.400	7.841	9.368
18	0.3854	2.549	0.618	1.374	4.886	4.500
18	0.3864	2.656	4.369	4.060	12.483	6.119
18	0.3874	8.952	7.300	13.625	16.150	6.566
18	0.3884	13.540	11.256	18.102	17.159	9.608
18	0.3894	10.429	14.106	13.100	14.300	9.128
18	0.3904	8.754	12.261	11.335	9.284	11.389
18	0.3914	7.147	10.875	11.315	10.304	12.118
18	0.3924	9.223	12.945	14.490	11.508	18.144
18	0.3934	11.327	9.619	10.812	12.262	17.625
18	0.3944	10.043	8.279	11.845	20.469	22.457
18	0.3954	10.025	8.306	11.742	20.399	22.423
18	0.3964	9.217	12.698	10.780	20.784	20.262
18	0.3974	8.259	8.029	10.104	19.491	22.993
18	0.3984	6.386	6.593	6.403	4.716	11.682
18	0.3994	13.691	5.252	6.726	12.375	11.416
18	0.4004	13.008	9.611	12.979	28.129	21.765
18	0.4014	13.361	10.029	12.588	12.649	25.527
18	0.4024	15.464	13.605	8.964	18.519	31.296

18	0.4034	16.024	10.722	10.662	21.542	23.758
18	0.4044	12.169	11.042	12.697	13.416	19.397
18	0.4054	10.527	11.159	10.710	14.930	22.645
18	0.4064	8.301	8.881	10.005	13.533	17.711
18	0.4074	5.981	7.725	6.708	6.758	4.033
18	0.4084	2.937	2.465	3.038	6.504	9.192
18	0.4094	5.534	3.578	4.361	8.300	18.364
18	0.4104	5.801	7.825	7.136	15.812	14.487
18	0.4114	7.777	6.389	8.495	10.349	10.660
18	0.4124	12.014	9.531	18.382	4.494	13.436
18	0.4134	9.767	14.781	15.779	15.939	14.725
18	0.4144	6.900	8.858	7.609	24.967	13.372
18	0.4154	13.150	15.369	16.829	18.215	21.591
18	0.4164	22.384	17.489	19.935	12.554	17.034
18	0.4174	24.611	17.520	20.645	13.299	13.515
18	0.4184	9.766	7.649	11.779	14.500	16.524
18	0.4194	15.087	17.519	14.446	14.559	9.247
18	0.4204	20.753	22.341	25.213	18.368	12.687
18	0.4214	12.837	11.585	13.387	11.919	13.381
18	0.4224	14.811	6.998	15.837	13.708	14.436
18	0.4234	12.444	8.328	14.123	7.684	9.110
18	0.4244	13.953	14.526	19.269	5.699	3.785
18	0.4254	20.942	20.538	23.051	8.260	7.620
18	0.4264	17.308	17.754	26.158	8.594	3.215
18	0.4274	8.872	5.695	8.962	8.037	16.140
18	0.4284	12.529	5.201	13.904	4.695	14.098
18	0.4294	10.631	8.664	15.019	13.882	14.120
18	0.4304	13.505	12.178	16.332	9.785	12.496
18	0.4314	20.526	16.683	28.051	17.654	8.664
18	0.4324	18.337	20.740	18.188	8.006	10.188
18	0.4334	10.129	9.543	12.826	5.961	9.970
18	0.4344	14.810	15.667	18.200	6.128	10.421
18	0.4354	22.128	18.808	25.592	16.892	14.512
18	0.4364	15.141	16.334	17.926	13.739	4.938
18	0.4374	10.230	12.004	14.978	15.281	7.037
18	0.4384	7.806	7.066	11.026	9.958	6.910
18	0.4394	13.902	11.885	15.585	7.515	10.784
18	0.4404	13.991	11.903	15.643	7.623	10.739
18	0.4414	14.080	11.926	15.701	7.732	10.695
18	0.4424	14.168	11.953	15.760	7.840	10.655
18	0.4434	10.432	8.726	15.013	13.706	28.345
18	0.4444	10.831	10.401	15.175	8.286	23.440
18	0.4454	19.732	11.734	16.160	10.874	12.642
18	0.4464	12.689	7.752	13.737	10.514	10.455
18	0.4474	20.013	16.685	19.736	16.789	17.128
18	0.4484	17.726	15.462	24.690	9.002	18.181
18	0.4494	25.708	27.391	30.979	9.468	13.179
18	0.4504	15.382	5.612	19.946	15.088	11.696
18	0.4514	16.648	13.781	22.335	15.445	12.246
18	0.4524	6.267	5.345	6.407	13.748	15.635

18	0.4534	14.320	11.593	16.734	11.629	20.316
18	0.4544	4.931	7.735	6.034	10.855	12.578
18	0.4554	10.565	20.902	14.770	17.564	7.454
18	0.4564	8.008	11.893	15.419	12.649	15.008
18	0.4574	13.252	19.957	22.246	13.029	17.714
18	0.4584	11.669	6.793	9.040	4.344	10.683
18	0.4594	9.118	10.281	12.314	5.460	9.679
18	0.4604	18.556	11.210	21.684	5.563	8.700
18	0.4614	19.922	8.387	20.119	15.754	16.939
18	0.4624	17.856	10.573	19.995	13.109	16.168
18	0.4634	12.910	8.100	9.552	12.655	14.130
18	0.4644	14.244	7.177	14.819	14.050	16.725
18	0.4654	12.756	8.759	14.620	8.044	13.024
18	0.4664	6.943	7.623	9.109	7.887	17.142
18	0.4674	14.225	16.734	18.052	11.721	18.455
18	0.4684	14.868	10.599	17.292	12.736	20.256
18	0.4694	14.863	10.601	17.310	12.712	20.279
18	0.4704	12.180	9.139	18.695	13.363	9.448
18	0.4714	20.456	12.203	21.395	16.869	11.379
18	0.4724	14.055	6.403	19.014	8.527	8.997
18	0.4734	17.116	14.286	20.585	23.042	19.966
18	0.4744	9.680	4.236	11.659	17.441	12.473
18	0.4754	25.939	14.058	23.529	26.185	15.116
18	0.4764	22.825	12.574	21.161	21.741	15.462
18	0.4774	11.033	5.757	12.096	9.706	15.575
18	0.4784	22.387	7.269	13.862	19.239	22.349
18	0.4794	20.687	9.110	15.705	15.775	15.027
18	0.4804	12.744	9.303	9.601	7.489	23.979
18	0.4814	12.448	8.941	9.121	7.460	23.770
18	0.4824	13.476	7.337	14.583	12.621	7.904
18	0.4834	23.355	14.890	23.232	30.915	14.720
18	0.4844	16.459	9.542	16.119	11.319	6.905
18	0.4854	16.010	11.456	20.548	13.673	6.985
18	0.4864	16.098	11.382	20.617	13.679	7.008
18	0.4874	15.360	13.340	13.201	9.000	6.812
18	0.4884	20.932	23.523	16.502	16.526	15.400
18	0.4894	11.385	6.688	8.045	21.297	14.006
18	0.4904	9.594	4.185	9.827	12.791	8.014
18	0.4914	10.274	4.853	10.779	13.679	8.735
18	0.4924	12.710	7.934	10.450	4.379	14.427
18	0.4934	15.007	12.814	13.071	13.857	22.148
18	0.4944	5.951	9.546	5.481	6.942	18.511
18	0.4954	12.903	12.951	13.333	28.398	20.106
18	0.4964	9.015	6.300	12.955	27.272	9.248
18	0.4974	22.582	17.202	25.772	24.892	13.126
18	0.4984	17.693	10.377	15.276	12.543	7.306
18	0.4994	9.863	13.562	12.636	20.563	9.525
18	0.5004	15.985	11.097	18.398	23.058	25.834
18	0.5014	16.032	11.037	18.341	23.069	25.961
18	0.5024	16.078	10.978	18.296	23.045	26.078

18	0.5034	11.481	6.865	16.019	17.964	16.015
18	0.5044	19.895	11.248	20.809	17.057	16.469
18	0.5054	22.482	10.568	22.621	16.231	14.082
18	0.5064	31.004	18.893	26.691	23.379	23.202
18	0.5074	16.877	20.646	18.664	16.684	11.320
18	0.5084	10.565	8.958	7.864	18.521	17.898
18	0.5094	9.305	4.899	7.301	19.274	19.639
18	0.5104	12.196	5.550	9.470	30.252	32.799
18	0.5114	17.736	9.798	16.846	25.255	21.432
18	0.5124	22.098	10.087	13.695	33.373	26.205
18	0.5134	18.210	20.637	17.047	18.450	19.092
18	0.5144	22.957	9.236	20.076	18.444	15.963
18	0.5154	21.826	14.683	23.506	20.269	4.613
18	0.5164	21.764	14.695	23.341	20.072	4.579
18	0.5174	22.204	14.195	20.421	12.540	6.074
18	0.5184	29.822	11.592	24.441	16.281	9.966
18	0.5194	17.075	7.966	9.054	16.198	12.747
18	0.5204	9.012	8.529	7.620	10.639	25.904
18	0.5214	10.383	9.230	12.570	8.183	9.904
18	0.5224	17.271	6.253	11.031	20.091	18.235
18	0.5234	15.405	7.697	12.275	22.996	20.321
18	0.5244	13.663	4.826	10.091	11.644	9.803
18	0.5254	15.821	4.992	11.768	14.526	7.750
18	0.5264	11.232	5.625	11.082	12.579	9.445
18	0.5274	5.828	4.976	3.791	17.224	12.653
18	0.5284	8.741	4.577	7.574	5.639	2.812
18	0.5294	9.241	6.214	7.181	7.752	13.473
18	0.5304	17.829	3.767	10.498	19.798	30.738
18	0.5314	20.519	8.296	14.444	20.956	30.966
18	0.5324	20.882	8.480	14.460	20.762	30.913
18	0.5334	19.146	12.721	19.411	14.496	18.323
18	0.5344	26.422	6.492	17.893	19.641	9.942
18	0.5354	14.442	10.726	11.591	8.355	10.886
18	0.5364	15.706	13.936	19.080	23.975	18.401
18	0.5374	15.090	11.384	10.759	18.913	18.494
18	0.5384	32.484	14.796	13.309	22.330	12.386
18	0.5394	18.408	21.485	16.549	16.062	10.433
18	0.5404	15.634	14.766	13.747	10.134	14.129
18	0.5414	12.085	10.528	8.787	9.900	11.048
18	0.5424	17.713	9.345	8.303	9.496	17.385
18	0.5434	12.028	7.878	14.029	10.650	24.161
18	0.5444	7.888	5.364	5.272	7.635	16.024
18	0.5454	10.765	4.228	10.717	4.702	15.915
18	0.5464	5.605	4.061	8.880	5.212	24.507
18	0.5474	12.028	6.560	8.856	8.191	24.487
18	0.5484	20.205	11.200	17.723	8.928	17.999
18	0.5494	14.251	8.373	13.227	12.979	7.460
18	0.5504	23.355	11.601	17.650	13.553	17.986
18	0.5514	17.846	10.889	13.511	14.854	14.127
18	0.5524	10.776	6.979	9.866	6.409	10.890

18	0.5534	15.676	19.707	21.728	15.784	15.292
18	0.5544	8.543	11.849	7.583	7.891	10.948
18	0.5554	10.012	8.630	9.529	6.210	18.847
18	0.5564	9.826	8.340	7.596	8.383	18.141
18	0.5574	21.123	19.666	14.975	20.071	20.922
18	0.5584	11.289	7.506	12.122	7.343	12.465
18	0.5594	4.448	3.510	6.502	10.885	9.497
18	0.5604	4.719	3.694	6.194	13.313	9.100
18	0.5614	12.824	10.318	14.935	16.697	14.335
18	0.5624	17.108	16.118	19.015	14.194	13.409
18	0.5634	17.082	16.198	19.060	14.225	13.354
18	0.5644	12.661	14.119	14.115	17.315	20.520
18	0.5654	8.908	13.349	14.673	14.288	21.745
18	0.5664	8.916	13.357	14.907	14.371	21.904
18	0.5674	8.931	13.364	15.141	14.468	22.055
18	0.5684	15.518	8.037	11.857	11.862	16.449
18	0.5694	15.368	17.759	11.052	13.246	13.058
18	0.5704	20.843	19.282	17.074	14.537	9.683
18	0.5714	18.428	15.490	12.496	13.259	12.168
18	0.5724	14.533	17.055	13.767	17.438	7.917
18	0.5734	14.423	16.946	13.655	17.425	7.805
18	0.5744	10.160	7.310	8.806	14.101	16.921
18	0.5754	10.128	7.197	8.727	14.061	16.804
18	0.5764	12.675	15.029	11.313	17.591	24.338
18	0.5774	17.543	19.831	22.659	9.959	21.289
18	0.5784	10.714	10.808	15.834	12.021	21.776
18	0.5794	13.001	7.309	11.881	5.658	9.444
18	0.5804	12.890	7.296	11.841	5.702	9.201
18	0.5814	12.781	7.285	11.802	5.753	8.962
18	0.5824	15.736	14.206	19.725	13.082	17.505
18	0.5834	12.449	7.735	12.978	15.544	8.461
18	0.5844	11.174	8.488	11.329	5.692	8.597
18	0.5854	5.936	4.243	2.829	9.814	9.850
18	0.5864	15.290	14.137	12.886	4.048	20.601
18	0.5874	17.431	18.385	13.731	4.882	12.517
18	0.5884	8.401	10.650	9.896	15.048	14.870
18	0.5894	8.321	6.000	8.166	10.735	6.214
18	0.5904	9.517	17.146	12.132	11.446	12.538
18	0.5914	15.697	8.296	13.167	12.151	18.629
18	0.5924	16.646	9.132	15.556	13.902	9.260
18	0.5934	15.028	15.210	16.608	15.496	17.722
18	0.5944	12.003	12.659	15.497	10.389	10.586
18	0.5954	11.224	10.945	12.466	11.430	12.409
18	0.5964	7.192	8.164	7.761	8.525	12.524
18	0.5974	15.251	13.277	14.511	16.172	28.405
18	0.5984	9.459	8.898	8.022	7.189	18.543
18	0.5994	9.616	8.896	8.077	7.191	18.539
18	0.6004	7.329	10.521	12.191	9.367	13.780
18	0.6014	17.262	20.841	21.911	12.810	18.468
18	0.6024	5.691	10.669	10.182	14.402	18.891

18	0.6034	4.283	6.055	4.432	19.716	33.834
18	0.6044	7.835	5.053	8.252	21.570	26.420
18	0.6054	7.813	6.366	2.900	19.532	26.007
18	0.6064	20.854	12.934	20.777	8.463	13.761
18	0.6074	14.459	12.422	16.400	15.396	16.683
18	0.6084	8.188	6.355	10.965	6.944	9.321
18	0.6094	0.898	1.083	1.142	1.180	1.328
19	0.0023	0.000	0.000	0.000	0.000	0.000
19	0.0033	13.030	7.676	11.572	9.576	8.357
19	0.0043	6.408	10.462	5.499	14.700	8.844
19	0.0053	13.688	17.488	16.702	16.634	21.694
19	0.0063	16.443	17.194	17.649	16.865	25.128
19	0.0073	17.629	20.599	17.924	23.014	22.699
19	0.0083	11.591	12.638	9.499	10.571	11.540
19	0.0093	17.407	20.190	12.116	15.620	16.801
19	0.0103	18.374	21.028	13.205	18.718	19.722
19	0.0113	4.676	9.172	6.401	9.535	14.514
19	0.0123	6.413	8.294	5.003	6.688	21.152
19	0.0133	19.740	17.016	14.980	12.759	23.782
19	0.0143	14.779	15.992	14.995	15.829	18.556
19	0.0153	18.036	16.204	14.083	12.624	16.390
19	0.0163	11.810	8.374	11.224	10.873	10.303
19	0.0173	7.223	10.671	8.985	13.381	11.167
19	0.0183	4.785	5.949	8.195	8.204	16.459
19	0.0193	21.871	25.617	22.791	8.060	12.418
19	0.0203	26.633	21.626	22.165	19.139	23.635
19	0.0213	23.355	18.073	17.435	28.281	32.081
19	0.0223	15.128	15.458	12.931	24.103	28.602
19	0.0233	10.441	15.344	9.565	13.922	31.270
19	0.0243	9.358	6.519	5.841	13.474	12.689
19	0.0253	8.823	9.605	13.307	13.553	18.917
19	0.0263	14.714	16.212	16.823	15.237	24.963
19	0.0273	17.628	16.171	17.732	7.928	14.290
19	0.0283	15.047	11.744	10.264	11.627	11.873
19	0.0293	16.722	13.387	18.905	15.146	18.547
19	0.0303	29.724	18.183	30.372	13.465	30.039
19	0.0313	12.401	14.242	10.661	19.402	14.850
19	0.0323	11.106	11.746	6.689	10.777	13.365
19	0.0333	7.153	10.361	10.808	10.786	15.132
19	0.0343	15.691	18.161	11.901	17.094	17.298
19	0.0353	12.816	14.421	14.082	19.286	13.249
19	0.0363	17.578	16.568	17.317	20.363	22.751
19	0.0373	10.890	12.443	8.479	19.497	25.583
19	0.0383	9.345	12.516	12.171	20.289	31.128
19	0.0393	20.746	11.240	15.381	23.405	24.441
19	0.0403	13.404	7.514	10.682	13.423	33.747
19	0.0413	9.447	11.518	13.046	14.063	26.621
19	0.0423	8.909	15.063	10.730	12.952	17.650
19	0.0433	10.703	14.293	12.055	12.803	27.420
19	0.0443	20.524	16.273	20.084	10.485	31.607

19	0.0453	11.394	10.393	10.890	4.333	7.606
19	0.0463	19.936	14.569	22.048	14.174	24.554
19	0.0473	17.616	13.997	12.500	7.237	21.505
19	0.0483	10.278	11.831	9.892	21.745	33.910
19	0.0493	14.098	12.633	9.270	12.052	30.402
19	0.0503	11.572	8.918	9.169	12.907	28.107
19	0.0513	13.414	9.012	11.813	11.184	25.641
19	0.0523	14.932	10.337	11.132	18.998	24.932
19	0.0533	20.963	14.077	13.456	18.145	32.642
19	0.0543	19.731	8.784	14.580	19.996	23.883
19	0.0553	11.099	7.978	9.643	20.335	37.866
19	0.0563	12.595	12.546	17.274	17.257	10.847
19	0.0573	12.476	12.526	17.028	17.266	10.880
19	0.0583	10.528	7.611	15.308	8.013	21.600
19	0.0593	8.184	7.594	9.914	12.131	35.835
19	0.0603	8.156	11.695	9.182	16.158	29.019
19	0.0613	11.371	11.059	16.544	24.235	30.037
19	0.0623	15.215	19.995	18.397	21.619	25.498
19	0.0633	13.151	17.357	13.036	12.683	15.361
19	0.0643	16.759	9.140	8.446	15.978	27.269
19	0.0653	18.679	8.698	12.996	14.556	24.697
19	0.0663	20.913	18.289	17.628	13.878	26.634
19	0.0673	12.483	9.059	6.926	10.795	17.673
19	0.0683	11.060	6.343	9.103	8.671	21.041
19	0.0693	7.644	9.738	8.520	9.820	17.702
19	0.0703	13.988	13.829	10.578	12.182	18.447
19	0.0713	12.247	20.198	17.854	15.524	13.100
19	0.0723	19.462	18.148	13.126	13.023	15.034
19	0.0733	20.604	17.064	15.873	25.626	10.345
19	0.0743	11.285	8.129	7.936	17.145	16.688
19	0.0753	11.331	7.945	8.041	16.694	16.733
19	0.0763	15.954	16.622	15.379	19.095	27.048
19	0.0773	18.978	19.234	21.462	20.683	25.024
19	0.0783	24.386	21.456	21.413	10.531	12.448
19	0.0793	14.918	13.378	17.077	11.407	8.876
19	0.0803	15.310	10.352	14.970	13.269	14.120
19	0.0813	20.426	15.020	20.798	12.012	15.904
19	0.0823	20.595	15.008	20.696	11.973	16.380
19	0.0833	24.410	13.182	17.717	12.000	10.169
19	0.0843	24.490	13.150	17.454	11.319	10.302
19	0.0853	24.439	13.114	17.217	10.644	10.243
19	0.0863	25.222	14.853	17.751	9.417	9.338
19	0.0873	17.013	13.240	15.964	14.667	17.907
19	0.0883	24.092	16.473	18.031	14.877	28.284
19	0.0893	18.060	10.402	16.687	14.035	20.630
19	0.0903	14.021	14.172	15.918	8.133	12.082
19	0.0913	15.328	15.642	17.942	6.129	11.288
19	0.0923	15.323	15.647	17.887	6.083	11.323
19	0.0933	13.056	14.414	20.317	7.958	2.494
19	0.0943	13.986	13.284	20.815	10.992	6.178

19	0.0953	13.923	13.262	20.844	11.102	6.024
19	0.0963	23.996	20.915	20.291	18.688	32.956
19	0.0973	13.513	10.818	13.018	10.656	24.746
19	0.0983	13.452	10.826	12.957	10.619	24.721
19	0.0993	8.976	8.950	12.115	13.674	20.038
19	0.1003	16.180	19.176	18.267	7.287	25.846
19	0.1013	14.241	13.103	18.616	10.095	16.143
19	0.1023	16.737	15.826	12.668	6.521	36.213
19	0.1033	14.083	11.505	14.699	7.218	26.084
19	0.1043	14.052	11.533	14.876	7.273	26.078
19	0.1053	18.303	17.780	20.465	6.743	8.584
19	0.1063	12.542	12.092	16.835	7.887	16.035
19	0.1073	13.204	11.392	15.405	12.615	17.429
19	0.1083	14.660	9.299	15.994	12.549	12.688
19	0.1093	14.732	12.463	17.866	15.118	25.981
19	0.1103	20.637	25.556	26.721	7.577	28.948
19	0.1113	11.772	10.581	15.409	6.849	13.084
19	0.1123	20.257	14.110	26.830	7.420	11.930
19	0.1133	16.204	13.629	16.237	12.797	14.975
19	0.1143	19.059	12.671	16.393	20.235	15.417
19	0.1153	11.758	6.878	7.571	11.859	8.976
19	0.1163	12.772	10.388	16.516	15.734	7.611
19	0.1173	13.242	10.323	13.441	12.479	8.210
19	0.1183	8.010	5.516	9.054	16.123	7.055
19	0.1193	15.824	7.543	14.735	25.801	7.025
19	0.1203	6.750	6.239	6.595	16.689	11.929
19	0.1213	6.132	2.734	3.305	7.763	10.579
19	0.1223	13.228	6.034	14.892	15.895	19.088
19	0.1233	10.788	12.118	16.700	12.379	8.681
19	0.1243	11.343	15.678	17.383	8.419	14.595
19	0.1253	10.041	14.767	16.825	10.013	12.263
19	0.1263	14.181	8.279	7.796	7.058	7.713
19	0.1273	6.642	12.253	5.914	12.413	10.248
19	0.1283	12.028	11.029	12.128	13.644	8.972
19	0.1293	16.332	16.632	19.478	11.185	4.765
19	0.1303	10.885	8.388	8.279	22.356	11.223
19	0.1313	11.764	7.496	12.403	16.732	10.031
19	0.1323	17.117	18.050	17.253	12.450	9.366
19	0.1333	9.255	9.854	7.390	19.621	17.597
19	0.1343	17.921	10.887	11.714	17.962	7.005
19	0.1353	11.937	7.970	13.669	12.337	5.690
19	0.1363	5.359	8.246	5.051	15.067	10.253
19	0.1373	5.304	8.078	4.943	14.968	10.240
19	0.1383	21.578	12.788	20.272	22.908	21.070
19	0.1393	24.576	13.552	16.510	8.255	17.526
19	0.1403	16.710	13.078	11.509	8.664	7.634
19	0.1413	8.681	15.591	12.700	13.611	4.190
19	0.1423	12.568	20.423	15.007	28.162	12.573
19	0.1433	5.234	12.591	7.439	16.958	5.542
19	0.1443	17.491	17.316	15.388	27.057	12.995

19	0.1453	17.852	8.728	18.983	18.116	10.903
19	0.1463	19.150	11.010	12.568	24.144	20.805
19	0.1473	24.488	23.153	24.246	21.111	11.875
19	0.1483	21.788	26.350	25.083	15.070	8.615
19	0.1493	16.850	22.088	17.737	24.844	13.354
19	0.1503	14.879	18.065	13.940	30.697	8.394
19	0.1513	10.805	12.663	9.269	15.948	8.172
19	0.1523	27.317	19.340	23.775	19.536	20.859
19	0.1533	18.532	11.842	16.615	9.466	7.427
19	0.1543	19.260	17.468	24.733	15.578	10.621
19	0.1553	22.269	20.274	20.235	26.217	19.502
19	0.1563	26.222	16.981	22.655	19.906	11.400
19	0.1573	14.078	10.588	13.755	18.317	7.372
19	0.1583	21.733	13.584	18.029	18.745	15.548
19	0.1593	23.115	17.959	23.211	23.473	14.094
19	0.1603	13.408	18.856	19.747	6.073	7.062
19	0.1613	32.189	28.637	23.045	19.373	22.471
19	0.1623	30.968	20.931	27.964	10.371	20.061
19	0.1633	26.437	17.204	20.010	19.182	15.777
19	0.1643	30.377	22.556	19.096	13.579	13.087
19	0.1653	13.177	10.419	11.287	17.648	13.017
19	0.1663	20.075	17.989	21.229	13.739	12.434
19	0.1673	24.755	14.545	22.147	16.131	24.671
19	0.1683	26.263	18.993	25.127	18.802	11.493
19	0.1693	28.566	24.437	27.170	16.864	12.436
19	0.1703	22.069	12.544	16.731	25.924	13.635
19	0.1713	18.633	16.128	16.603	18.191	10.442
19	0.1723	17.159	17.595	15.549	14.852	8.734
19	0.1733	19.864	14.314	14.371	9.605	16.138
19	0.1743	19.992	14.112	15.350	6.139	14.782
19	0.1753	17.046	16.592	14.222	8.302	18.349
19	0.1763	18.136	12.934	15.563	7.585	13.412
19	0.1773	13.635	7.795	10.097	13.989	11.271
19	0.1783	18.482	13.666	17.607	16.496	17.543
19	0.1793	11.725	9.427	9.394	12.707	14.800
19	0.1803	14.021	14.320	12.519	15.505	13.288
19	0.1813	7.390	3.284	8.432	6.960	9.141
19	0.1823	7.404	3.283	8.414	6.945	9.070
19	0.1833	6.555	7.905	7.464	11.218	18.811
19	0.1843	22.183	15.645	21.419	9.052	11.711
19	0.1853	32.975	25.124	34.799	10.249	11.049
19	0.1863	18.933	15.205	19.020	7.803	12.734
19	0.1873	19.737	16.424	19.137	6.089	6.705
19	0.1883	25.238	19.352	32.333	10.511	20.128
19	0.1893	31.607	21.834	35.164	23.370	12.222
19	0.1903	20.576	9.574	23.815	14.527	19.061
19	0.1913	23.351	21.896	31.055	8.788	13.450
19	0.1923	23.524	24.317	36.810	10.204	14.900
19	0.1933	22.091	18.547	27.640	5.280	12.631
19	0.1943	19.153	15.600	28.127	13.201	9.790

19	0.1953	13.896	8.909	23.325	15.712	16.017
19	0.1963	12.507	9.802	23.757	13.368	15.173
19	0.1973	12.594	12.722	22.757	17.007	28.422
19	0.1983	31.561	22.004	41.242	15.143	22.747
19	0.1993	30.718	24.595	44.845	13.794	17.440
19	0.2003	30.468	23.005	39.774	21.430	11.007
19	0.2013	28.431	20.642	28.524	25.021	20.934
19	0.2023	28.457	20.625	28.541	25.025	21.013
19	0.2033	18.810	15.573	17.892	14.626	17.557
19	0.2043	22.542	18.533	20.379	11.671	24.806
19	0.2053	19.202	12.102	20.704	7.168	15.604
19	0.2063	22.533	16.875	31.102	13.702	7.458
19	0.2073	18.905	12.966	18.122	13.198	20.463
19	0.2083	22.660	18.760	26.770	7.971	16.069
19	0.2093	26.764	21.483	35.493	12.901	17.868
19	0.2103	26.311	18.267	37.908	15.043	12.233
19	0.2113	11.254	9.372	14.354	20.024	19.082
19	0.2123	31.352	26.510	33.607	12.706	9.979
19	0.2133	25.132	13.144	35.031	15.746	14.911
19	0.2143	26.549	15.444	42.967	19.667	21.927
19	0.2153	17.326	15.769	18.711	14.501	14.209
19	0.2163	20.964	22.343	27.627	20.830	18.045
19	0.2173	31.479	23.591	33.330	12.608	23.821
19	0.2183	25.635	23.044	35.328	11.486	15.731
19	0.2193	31.514	29.850	44.139	11.368	18.631
19	0.2203	24.013	36.182	33.601	13.379	9.622
19	0.2213	35.548	46.680	48.734	25.573	18.919
19	0.2223	35.931	38.221	40.722	12.915	6.694
19	0.2233	29.630	31.743	38.048	15.146	17.267
19	0.2243	29.357	27.015	42.500	18.255	20.364
19	0.2253	39.799	40.006	40.431	15.843	23.039
19	0.2263	26.963	19.884	33.128	18.233	8.495
19	0.2273	26.936	15.891	33.187	12.358	12.948
19	0.2283	26.396	16.123	29.979	11.409	10.675
19	0.2293	38.178	24.852	42.222	12.967	11.187
19	0.2303	32.340	21.931	40.126	5.564	1.962
19	0.2313	34.049	24.913	41.220	6.975	3.090
19	0.2323	31.433	30.260	37.733	7.670	20.917
19	0.2333	44.992	35.575	48.021	10.908	13.945
19	0.2343	35.970	29.313	45.072	17.546	20.871
19	0.2353	13.388	14.727	14.720	6.573	20.978
19	0.2363	24.916	24.226	25.993	11.747	14.825
19	0.2373	21.003	17.594	34.635	19.460	18.794
19	0.2383	20.841	17.441	34.886	19.470	18.729
19	0.2393	27.822	14.238	33.018	27.714	25.478
19	0.2403	26.692	17.817	31.805	17.058	9.150
19	0.2413	22.857	12.114	22.710	7.037	5.063
19	0.2423	28.952	14.274	25.223	19.739	20.790
19	0.2433	33.394	24.355	35.117	14.159	15.602
19	0.2443	41.600	25.964	41.940	16.860	20.254

19	0.2453	40.092	28.417	51.239	23.048	20.447
19	0.2463	46.417	34.344	52.153	24.434	26.892
19	0.2473	43.128	25.653	50.965	14.860	22.502
19	0.2483	41.185	32.215	43.054	18.700	22.912
19	0.2493	35.527	31.856	44.442	23.872	18.032
19	0.2503	44.207	32.270	52.871	21.309	17.133
19	0.2513	33.755	28.658	38.961	18.378	22.416
19	0.2523	38.167	36.485	45.371	16.274	23.003
19	0.2533	24.505	31.156	32.399	6.729	13.230
19	0.2543	20.364	19.879	28.320	8.381	12.716
19	0.2553	39.817	31.042	48.286	18.417	15.760
19	0.2563	41.145	30.738	51.468	18.053	18.409
19	0.2573	41.763	34.784	54.311	17.501	20.969
19	0.2583	45.409	40.329	57.343	14.862	19.921
19	0.2593	41.229	33.631	52.998	13.349	17.493
19	0.2603	31.888	23.782	33.679	6.369	10.995
19	0.2613	40.566	31.936	50.455	26.629	21.130
19	0.2623	32.040	22.368	38.143	12.794	7.293
19	0.2633	36.794	27.080	44.691	14.814	6.215
19	0.2643	36.258	23.563	44.371	24.033	14.719
19	0.2653	36.264	23.572	44.390	24.045	14.732
19	0.2663	27.473	22.767	37.320	17.905	16.067
19	0.2673	22.320	27.761	41.659	13.081	11.811
19	0.2683	40.726	33.595	51.658	9.852	15.304
19	0.2693	36.288	23.495	40.113	20.154	38.378
19	0.2703	23.466	16.064	29.338	18.858	16.311
19	0.2713	26.964	20.390	31.503	18.667	26.317
19	0.2723	19.729	19.384	15.292	22.046	30.644
19	0.2733	15.913	17.761	9.516	22.967	15.017
19	0.2743	22.280	20.427	21.665	15.888	21.942
19	0.2753	17.545	20.293	23.452	19.140	18.181
19	0.2763	44.342	35.029	52.215	20.032	18.624
19	0.2773	39.388	24.004	47.282	20.340	15.838
19	0.2783	41.460	28.244	54.812	20.505	22.075
19	0.2793	24.910	23.950	31.089	10.466	14.801
19	0.2803	24.302	23.098	29.158	9.727	6.204
19	0.2813	27.985	29.592	34.509	22.478	13.940
19	0.2823	34.802	29.072	35.615	21.574	22.155
19	0.2833	41.180	34.636	43.345	20.849	24.361
19	0.2843	33.960	30.372	40.533	15.729	18.530
19	0.2853	36.404	29.215	37.464	23.256	28.688
19	0.2863	26.212	25.569	30.742	12.357	14.777
19	0.2873	30.305	24.034	34.438	14.837	17.566
19	0.2883	30.826	25.673	40.332	12.904	15.912
19	0.2893	21.087	20.516	21.787	22.800	21.761
19	0.2903	29.323	16.584	22.067	30.002	19.174
19	0.2913	15.951	7.500	13.010	15.980	17.698
19	0.2923	25.388	15.462	20.941	18.839	16.925
19	0.2933	27.474	18.170	24.248	29.473	25.980
19	0.2943	13.202	18.273	16.529	26.637	15.086

19	0.2953	16.752	19.138	22.637	17.509	12.453
19	0.2963	23.071	18.756	26.950	15.201	18.638
19	0.2973	29.017	25.729	27.774	19.843	18.608
19	0.2983	21.932	24.034	25.511	20.896	5.951
19	0.2993	20.548	16.502	13.350	22.473	8.908
19	0.3003	27.055	19.231	25.231	17.046	5.858
19	0.3013	21.260	17.568	23.218	10.514	14.149
19	0.3023	20.078	25.295	26.511	11.156	22.164
19	0.3033	12.100	9.912	8.099	21.635	17.977
19	0.3043	9.318	11.941	10.530	18.621	20.050
19	0.3053	10.773	10.723	5.259	24.604	20.396
19	0.3063	24.387	16.336	21.886	18.329	18.924
19	0.3073	30.851	21.389	27.845	30.296	16.437
19	0.3083	26.038	16.735	23.677	25.660	20.346
19	0.3093	30.577	20.782	28.328	28.381	18.351
19	0.3103	18.865	20.150	24.976	14.266	3.700
19	0.3113	22.300	18.365	18.224	19.425	8.925
19	0.3123	22.575	14.591	22.245	22.756	22.697
19	0.3133	16.743	11.585	22.967	20.634	11.076
19	0.3143	20.470	19.234	27.273	22.758	4.485
19	0.3153	11.965	14.223	14.488	17.767	3.172
19	0.3163	3.612	9.200	6.985	20.338	16.462
19	0.3173	5.362	10.470	5.318	25.868	8.148
19	0.3183	21.672	12.679	18.198	31.308	29.649
19	0.3193	17.663	11.463	14.809	26.377	17.859
19	0.3203	17.045	21.738	21.129	35.015	15.949
19	0.3213	32.268	27.730	30.154	22.805	14.973
19	0.3223	24.574	20.830	26.454	18.009	13.092
19	0.3233	21.535	23.683	23.675	16.491	8.754
19	0.3243	21.106	19.032	20.671	16.438	6.103
19	0.3253	6.561	4.002	5.687	4.970	4.874
19	0.3263	10.840	12.706	11.309	9.139	7.951
19	0.3273	15.727	12.141	12.646	17.245	10.141
19	0.3283	11.476	8.432	11.809	21.374	16.062
19	0.3293	22.459	14.545	24.097	20.078	10.472
19	0.3303	23.792	13.936	24.485	26.876	13.461
19	0.3313	22.623	24.828	33.632	36.097	20.233
19	0.3323	27.628	25.022	35.385	31.061	19.353
19	0.3333	18.724	15.444	16.062	26.299	8.051
19	0.3343	21.430	18.328	28.784	20.585	12.603
19	0.3353	18.006	15.419	24.880	15.084	5.957
19	0.3363	17.303	13.950	23.451	20.092	5.340
19	0.3373	8.161	7.787	9.223	23.230	10.131
19	0.3383	21.392	15.736	26.332	12.050	9.920
19	0.3393	18.308	17.628	18.062	19.972	6.671
19	0.3403	23.931	19.034	23.707	15.154	7.408
19	0.3413	16.933	13.355	15.640	13.398	6.488
19	0.3423	22.172	19.824	27.011	14.472	9.232
19	0.3433	14.570	9.994	19.042	10.948	8.272
19	0.3443	12.745	11.359	16.781	13.362	8.890

19	0.3453	22.442	20.196	24.697	21.667	13.487
19	0.3463	25.774	19.279	28.715	23.110	17.792
19	0.3473	12.780	14.323	20.349	16.222	8.022
19	0.3483	16.106	15.253	20.138	19.050	16.136
19	0.3493	18.209	12.715	22.804	22.790	19.194
19	0.3503	12.402	9.869	14.106	19.071	8.246
19	0.3513	7.295	9.378	10.430	20.477	12.934
19	0.3523	13.010	17.887	20.480	20.588	7.786
19	0.3533	31.108	24.422	26.633	25.305	9.671
19	0.3543	20.463	5.020	12.947	25.797	18.061
19	0.3553	6.816	8.281	11.295	19.821	9.732
19	0.3563	13.507	12.131	10.968	20.287	16.883
19	0.3573	6.388	11.719	7.597	18.970	15.619
19	0.3583	6.396	11.730	7.613	18.964	15.622
19	0.3593	8.267	13.510	11.741	19.769	10.934
19	0.3603	22.439	19.376	27.119	14.565	6.193
19	0.3613	18.548	14.243	18.936	26.524	11.745
19	0.3623	26.953	31.704	28.050	36.486	13.115
19	0.3633	14.513	18.230	15.887	30.405	18.189
19	0.3643	19.360	19.190	21.310	18.126	14.550
19	0.3653	17.929	14.384	20.317	16.436	18.813
19	0.3663	13.227	19.964	16.709	21.951	14.235
19	0.3673	13.548	19.362	19.114	32.576	11.196
19	0.3683	14.948	19.036	15.732	25.905	14.272
19	0.3693	21.506	21.016	18.340	27.157	11.896
19	0.3703	21.440	21.002	18.190	27.415	11.929
19	0.3713	14.487	21.016	17.084	24.338	16.360
19	0.3723	8.790	18.424	13.076	28.519	12.445
19	0.3733	11.218	22.052	12.694	13.392	16.384
19	0.3743	22.810	32.694	26.676	30.720	24.747
19	0.3753	4.802	10.881	14.174	25.412	23.077
19	0.3763	11.787	10.848	21.340	9.660	22.556
19	0.3773	10.003	12.348	15.492	18.244	22.085
19	0.3783	11.892	13.927	14.446	37.282	17.532
19	0.3793	13.052	16.826	21.837	9.482	6.351
19	0.3803	9.469	12.462	12.374	6.778	11.829
19	0.3813	12.540	12.088	12.131	4.348	8.164
19	0.3823	13.730	15.253	17.815	16.073	12.003
19	0.3833	8.481	13.111	17.384	20.972	19.186
19	0.3843	8.299	10.167	11.724	8.574	15.575
19	0.3853	13.919	23.164	18.588	17.771	7.744
19	0.3863	26.919	24.129	32.290	31.992	10.443
19	0.3873	15.747	19.755	24.113	31.622	10.325
19	0.3883	16.139	17.342	19.856	27.840	15.783
19	0.3893	25.553	12.314	22.260	38.172	26.550
19	0.3903	10.261	7.637	10.856	10.615	6.462
19	0.3913	14.116	8.958	21.595	16.409	5.003
19	0.3923	8.559	18.831	15.469	44.006	17.364
19	0.3933	5.740	12.734	5.400	27.512	11.466
19	0.3943	11.056	12.115	14.578	10.886	5.388

19	0.3953	14.924	15.086	20.595	31.950	12.094
19	0.3963	11.357	7.424	11.239	27.776	14.634
19	0.3973	18.650	14.249	20.177	26.592	16.268
19	0.3983	9.747	15.580	15.859	13.689	11.541
19	0.3993	14.966	18.414	21.071	24.091	14.121
19	0.4003	18.613	15.158	21.562	27.504	20.116
19	0.4013	29.617	17.002	24.942	38.202	20.042
19	0.4023	22.217	17.384	22.025	25.981	29.582
19	0.4033	17.887	12.206	17.960	24.168	22.184
19	0.4043	21.703	21.898	18.533	16.747	23.141
19	0.4053	8.545	10.917	8.662	24.646	8.744
19	0.4063	13.301	26.644	18.257	37.051	24.682
19	0.4073	14.177	27.340	21.573	32.692	19.812
19	0.4083	10.706	24.862	21.875	38.466	12.439
19	0.4093	10.794	22.560	14.146	34.083	19.321
19	0.4103	14.533	30.725	23.224	24.064	15.289
19	0.4113	14.662	20.024	17.429	28.823	36.232
19	0.4123	16.002	14.587	21.913	19.024	18.234
19	0.4133	13.531	17.003	18.961	23.767	18.676
19	0.4143	15.385	13.372	13.897	15.915	24.990
19	0.4153	11.838	29.061	25.953	34.636	19.644
19	0.4163	9.793	17.516	13.933	15.320	18.090
19	0.4173	2.532	5.172	6.909	17.263	10.701
19	0.4183	2.527	5.159	6.823	17.274	10.779
19	0.4193	2.112	5.117	6.158	19.684	17.117
19	0.4203	5.996	7.430	9.653	25.980	20.356
19	0.4213	5.981	7.424	9.688	26.009	20.336
19	0.4223	9.656	10.541	8.147	16.938	12.954
19	0.4233	6.561	5.939	9.539	18.575	10.467
19	0.4243	8.283	11.467	13.478	18.728	6.874
19	0.4253	10.252	9.588	9.686	8.505	11.857
19	0.4263	6.584	16.030	9.970	19.794	8.189
19	0.4273	26.555	16.906	27.413	32.542	12.189
19	0.4283	14.100	11.074	18.527	22.954	8.524
19	0.4293	10.139	7.657	15.152	17.401	7.965
19	0.4303	6.673	8.812	7.714	18.050	9.203
19	0.4313	4.438	6.988	5.470	13.048	11.574
19	0.4323	10.607	10.510	12.588	18.038	13.111
19	0.4333	13.651	11.817	19.226	23.644	11.109
19	0.4343	15.085	17.782	20.068	34.673	6.657
19	0.4353	17.178	13.158	17.145	22.919	8.521
19	0.4363	14.237	16.198	15.804	17.650	10.997
19	0.4373	20.406	21.086	25.251	16.963	13.553
19	0.4383	25.669	19.162	24.768	17.476	9.390
19	0.4393	16.303	15.954	16.698	39.934	12.772
19	0.4403	31.870	23.727	30.856	35.244	18.535
19	0.4413	28.309	28.624	29.966	25.222	12.182
19	0.4423	7.307	4.681	7.521	11.806	8.503
19	0.4433	20.070	11.578	18.160	19.109	6.397
19	0.4443	29.038	14.546	21.123	22.567	10.813

19	0.4453	24.281	19.692	21.131	24.202	14.465
19	0.4463	23.616	23.842	19.716	17.226	20.377
19	0.4473	28.468	18.188	28.866	19.520	14.609
19	0.4483	10.413	7.677	9.400	15.082	10.899
19	0.4493	9.991	12.571	13.074	31.481	7.975
19	0.4503	13.567	13.883	14.015	16.747	18.156
19	0.4513	15.186	17.081	18.811	18.530	16.145
19	0.4523	13.140	18.060	15.043	21.939	6.548
19	0.4533	10.938	10.583	9.510	14.569	5.330
19	0.4543	11.054	13.535	11.474	27.522	3.168
19	0.4553	3.833	5.719	2.337	6.756	6.351
19	0.4563	8.321	14.102	7.714	17.706	7.202
19	0.4573	10.451	8.415	10.619	21.077	4.652
19	0.4583	10.155	22.239	12.764	15.877	5.395
19	0.4593	29.677	19.575	30.217	36.400	11.624
19	0.4603	13.525	14.523	10.490	25.061	12.392
19	0.4613	27.464	28.177	29.838	22.541	6.991
19	0.4623	14.059	14.724	17.412	27.817	10.762
19	0.4633	20.435	17.175	24.392	8.512	11.510
19	0.4643	16.771	15.872	18.608	30.180	21.941
19	0.4653	13.762	17.322	15.155	30.441	20.949
19	0.4663	24.011	25.203	24.716	27.885	22.987
19	0.4673	24.115	19.491	25.520	27.345	20.121
19	0.4683	18.689	9.332	21.016	30.528	15.952
19	0.4693	20.601	6.777	13.480	18.513	14.363
19	0.4703	17.577	5.107	14.150	16.826	15.356
19	0.4713	16.020	16.257	16.423	18.495	4.041
19	0.4723	17.493	19.375	24.406	19.070	13.396
19	0.4733	20.417	17.337	23.135	21.902	19.321
19	0.4743	20.802	18.950	18.822	19.795	19.179
19	0.4753	14.959	18.384	19.351	31.472	13.643
19	0.4763	9.588	6.461	10.867	24.334	15.190
19	0.4773	18.491	12.509	22.417	32.534	26.656
19	0.4783	10.779	8.912	14.509	10.678	4.133
19	0.4793	11.587	9.684	7.352	16.075	9.103
19	0.4803	19.836	20.334	22.406	34.745	18.438
19	0.4813	11.050	8.628	12.421	20.969	11.634
19	0.4823	17.579	19.763	19.975	34.426	21.918
19	0.4833	17.547	20.050	19.987	34.640	22.017
19	0.4843	27.206	17.992	25.568	18.361	28.179
19	0.4853	37.028	28.159	28.045	17.589	33.620
19	0.4863	16.601	18.751	16.700	13.827	12.878
19	0.4873	13.516	8.667	15.831	14.573	19.342
19	0.4883	25.373	15.921	25.907	20.019	10.754
19	0.4893	22.676	10.350	26.512	19.393	11.995
19	0.4903	16.672	11.378	19.884	34.620	11.942
19	0.4913	10.313	9.813	15.228	27.439	8.124
19	0.4923	14.334	13.089	9.696	17.248	14.159
19	0.4933	30.367	26.034	29.287	16.696	14.608
19	0.4943	30.529	26.048	29.451	16.645	14.565

19	0.4953	29.147	12.715	27.685	15.432	11.133
19	0.4963	23.876	27.801	33.530	11.583	12.382
19	0.4973	15.259	9.799	17.405	16.648	17.808
19	0.4983	29.487	16.899	25.249	19.589	23.041
19	0.4993	17.774	16.527	19.160	24.907	9.660
19	0.5003	11.098	9.739	11.761	21.026	14.894
19	0.5013	28.223	15.559	23.270	32.551	26.781
19	0.5023	23.791	12.898	24.807	26.832	25.799
19	0.5033	19.866	18.539	23.723	27.096	17.254
19	0.5043	20.811	16.152	18.807	25.515	19.745
19	0.5053	16.963	19.900	16.963	31.163	17.896
19	0.5063	16.308	14.803	12.609	14.143	12.073
19	0.5073	15.325	20.167	14.425	12.209	16.334
19	0.5083	23.621	23.684	20.870	9.343	20.617
19	0.5093	13.719	14.835	18.833	14.640	24.061
19	0.5103	18.820	13.475	22.077	15.593	27.898
19	0.5113	21.130	18.546	22.229	8.773	15.580
19	0.5123	25.988	21.201	23.737	30.805	36.025
19	0.5133	24.057	13.982	26.311	26.561	11.667
19	0.5143	16.688	15.505	15.333	18.287	10.322
19	0.5153	17.463	12.723	18.241	16.711	18.321
19	0.5163	15.930	12.883	17.576	25.797	27.282
19	0.5173	31.382	17.829	24.779	18.251	21.043
19	0.5183	21.223	10.984	9.824	8.319	14.280
19	0.5193	16.074	15.922	13.962	14.336	19.744
19	0.5203	31.703	24.534	19.976	20.102	17.830
19	0.5213	20.243	26.046	16.871	27.915	11.980
19	0.5223	12.442	11.776	9.075	25.920	15.913
19	0.5233	11.868	12.506	10.564	19.908	10.589
19	0.5243	14.485	13.143	12.808	14.393	12.004
19	0.5253	12.844	19.991	14.982	21.186	18.148
19	0.5263	27.156	31.299	26.371	19.461	27.392
19	0.5273	27.176	31.551	26.549	19.596	27.749
19	0.5283	14.495	21.845	19.319	12.074	23.409
19	0.5293	14.399	20.399	18.722	13.051	22.030
19	0.5303	16.469	15.936	16.153	11.748	17.689
19	0.5313	10.111	12.771	6.604	9.038	19.110
19	0.5323	10.585	10.499	9.788	10.597	11.372
19	0.5333	14.231	19.359	16.699	14.380	4.567
19	0.5343	17.334	28.775	19.882	30.938	14.684
19	0.5353	22.236	26.934	25.689	27.398	15.803
19	0.5363	19.120	20.374	16.851	17.955	18.014
19	0.5373	10.621	18.576	15.859	17.177	17.561
19	0.5383	22.549	27.935	23.062	15.879	19.280
19	0.5393	14.949	19.965	12.143	13.932	17.416
19	0.5403	12.975	17.742	17.459	18.921	15.705
19	0.5413	17.604	26.843	20.731	20.731	14.551
19	0.5423	19.646	22.288	23.708	10.764	10.562
19	0.5433	22.133	30.057	24.769	10.607	12.919
19	0.5443	17.613	18.591	19.501	8.256	3.781

19	0.5453	10.774	10.636	9.917	10.766	6.116
19	0.5463	7.300	15.573	10.750	8.102	8.770
19	0.5473	9.956	12.752	10.784	5.405	5.008
19	0.5483	13.705	12.580	13.146	5.567	9.887
19	0.5493	21.214	19.788	19.708	13.439	7.620
19	0.5503	14.834	29.926	18.580	9.466	9.854
19	0.5513	17.753	23.542	19.141	8.815	7.612
19	0.5523	18.680	25.833	20.401	12.316	11.204
19	0.5533	10.625	15.932	10.149	15.140	12.889
19	0.5543	10.088	11.327	11.341	13.592	7.869
19	0.5553	18.359	20.727	25.622	10.730	19.378
19	0.5563	19.397	21.327	22.158	17.000	13.469
19	0.5573	19.212	19.750	20.617	11.819	15.293
19	0.5583	21.945	22.960	26.433	10.590	9.647
19	0.5593	26.417	35.749	33.490	8.386	7.615
19	0.5603	23.085	31.649	31.406	5.525	10.244
19	0.5613	11.032	16.610	15.236	4.750	10.566
19	0.5623	16.559	20.870	20.226	3.924	11.374
19	0.5633	12.475	14.669	11.413	12.364	12.061
19	0.5643	21.743	24.112	26.825	11.408	24.205
19	0.5653	17.342	22.348	21.249	8.839	13.838
19	0.5663	18.033	15.323	20.217	16.795	11.292
19	0.5673	15.914	16.063	21.919	16.259	22.550
19	0.5683	8.053	9.475	8.961	3.338	24.644
19	0.5693	8.220	5.781	4.199	9.325	18.371
19	0.5703	11.991	11.518	13.268	19.997	23.437
19	0.5713	12.067	6.203	12.476	13.302	14.541
19	0.5723	15.238	10.771	14.668	17.970	13.579
19	0.5733	13.023	15.841	21.563	20.589	23.330
19	0.5743	21.071	13.853	23.809	21.728	14.488
19	0.5753	21.036	13.842	23.770	21.733	14.340
19	0.5763	20.454	21.232	25.849	15.664	11.492
19	0.5773	22.314	12.227	23.302	10.226	8.420
19	0.5783	21.300	13.541	19.913	9.650	7.993
19	0.5793	8.104	16.177	18.789	15.998	19.383
19	0.5803	5.843	12.997	14.912	17.904	17.059
19	0.5813	13.770	17.502	13.862	21.816	23.551
19	0.5823	11.099	8.566	11.460	4.605	11.748
19	0.5833	6.784	5.968	15.155	12.310	9.094
19	0.5843	8.802	13.749	20.819	23.308	13.955
19	0.5853	14.767	7.357	17.163	26.140	7.907
19	0.5863	12.732	20.775	15.570	26.056	10.366
19	0.5873	8.920	15.714	10.005	29.206	11.121
19	0.5883	10.668	18.728	13.857	11.352	10.373
19	0.5893	10.991	19.403	23.226	23.307	21.746
19	0.5903	13.599	12.698	17.597	17.318	13.746
19	0.5913	12.266	13.362	19.359	7.681	15.686
19	0.5923	19.302	18.903	34.373	15.977	10.431
19	0.5933	16.773	15.483	16.177	12.761	11.221
19	0.5943	7.657	7.990	15.418	6.369	16.293

19	0.5953	15.793	19.579	34.905	13.689	8.942
19	0.5963	2.771	6.406	12.202	6.939	13.697
19	0.5973	5.517	8.050	5.651	12.935	10.075
19	0.5983	15.125	17.697	16.557	11.829	13.672
19	0.5993	19.525	15.010	23.197	15.525	17.506
19	0.6003	25.977	26.663	42.890	14.391	8.560
19	0.6013	16.881	15.899	18.053	16.214	20.550
19	0.6023	15.484	20.217	23.934	15.971	27.735
19	0.6033	6.704	2.667	6.121	13.224	15.795
19	0.6043	26.997	14.173	23.973	22.259	28.218
19	0.6053	26.241	22.248	30.114	16.426	27.117
19	0.6063	13.204	8.994	21.661	11.539	24.495
19	0.6073	13.173	10.942	13.806	12.796	11.231
19	0.6083	21.518	18.869	27.036	12.546	14.109
19	0.6093	15.433	9.934	24.027	19.422	14.648
19	0.6103	17.811	11.477	21.121	20.024	9.253
19	0.6113	19.107	10.943	12.392	17.358	13.338
19	0.6123	27.435	16.128	29.500	19.493	18.682
19	0.6133	28.197	15.967	26.571	19.678	18.333
19	0.6143	16.316	12.245	24.627	25.157	21.556
19	0.6153	21.259	14.369	23.356	23.189	15.975
19	0.6163	22.564	12.064	19.344	19.458	14.462
19	0.6173	13.694	14.394	7.551	17.175	19.252
19	0.6183	14.516	10.892	13.225	9.472	15.822
19	0.6193	13.108	6.122	12.162	10.137	18.019
19	0.6203	0.000	0.943	0.292	0.535	0.001
19	0.6213	0.338	0.158	0.551	0.339	0.081
20	0.0017	0.000	0.000	0.000	0.000	0.000
20	0.0027	8.415	10.463	13.861	11.101	16.443
20	0.0037	14.178	12.396	14.878	4.632	13.642
20	0.0047	7.677	8.854	9.123	6.923	10.782
20	0.0057	15.852	10.001	12.713	5.099	6.719
20	0.0067	9.532	5.283	6.038	8.508	7.812
20	0.0077	15.529	9.885	9.448	18.831	10.514
20	0.0087	23.176	14.929	24.170	4.423	16.889
20	0.0097	11.744	17.145	15.651	15.526	14.963
20	0.0107	16.725	10.099	17.627	10.208	18.324
20	0.0117	13.583	7.558	12.173	13.573	15.019
20	0.0127	18.418	17.919	22.607	13.033	7.728
20	0.0137	7.515	6.314	5.813	11.590	13.741
20	0.0147	9.075	6.882	5.192	12.887	9.166
20	0.0157	13.452	14.525	16.096	13.204	21.190
20	0.0167	18.780	13.151	15.420	10.722	20.250
20	0.0177	14.508	6.838	13.929	12.846	15.000
20	0.0187	8.991	16.724	19.933	10.130	14.919
20	0.0197	13.363	12.272	13.501	5.641	18.137
20	0.0207	11.620	13.749	15.052	9.532	14.854
20	0.0217	22.251	21.196	21.318	11.746	12.536
20	0.0227	17.839	15.276	15.536	7.185	14.320
20	0.0237	14.637	12.671	16.461	10.447	14.858

20	0.0247	15.055	16.448	20.447	7.669	14.070
20	0.0257	8.506	10.074	16.248	5.189	13.262
20	0.0267	8.603	6.362	13.577	12.805	14.783
20	0.0277	10.658	8.048	15.073	9.264	12.703
20	0.0287	9.994	6.945	9.766	9.095	6.362
20	0.0297	9.647	7.245	10.977	11.577	14.156
20	0.0307	17.954	13.528	19.162	7.390	18.874
20	0.0317	13.700	11.480	16.290	12.456	25.908
20	0.0327	20.540	14.239	20.742	7.340	13.584
20	0.0337	15.305	15.079	17.202	7.031	15.508
20	0.0347	16.392	16.857	16.628	6.030	14.122
20	0.0357	16.395	16.872	16.639	6.127	14.098
20	0.0367	6.158	7.982	5.470	6.526	20.001
20	0.0377	3.300	4.334	3.277	6.512	3.697
20	0.0387	6.538	7.414	8.930	5.830	5.226
20	0.0397	6.638	7.048	7.334	4.608	9.271
20	0.0407	7.458	4.117	7.351	11.195	5.850
20	0.0417	12.634	13.699	18.649	12.529	12.327
20	0.0427	17.923	12.830	16.196	7.477	11.000
20	0.0437	17.248	17.712	19.327	7.103	11.569
20	0.0447	17.206	17.556	19.182	7.126	11.623
20	0.0457	15.392	14.618	19.368	13.781	16.004
20	0.0467	20.567	16.411	14.796	7.978	16.730
20	0.0477	15.972	15.665	16.319	16.877	11.370
20	0.0487	24.768	30.774	24.473	22.849	15.471
20	0.0497	27.799	26.963	22.959	27.822	14.766
20	0.0507	24.686	20.968	17.657	22.672	16.371
20	0.0517	12.321	10.793	11.060	15.339	22.241
20	0.0527	9.658	6.515	9.787	11.576	13.996
20	0.0537	12.390	11.193	12.761	12.599	13.539
20	0.0547	10.859	12.222	9.555	19.427	6.708
20	0.0557	19.466	13.814	12.616	16.758	15.807
20	0.0567	17.092	12.644	11.872	12.924	16.553
20	0.0577	21.950	19.071	20.292	14.896	24.525
20	0.0587	17.095	22.319	24.182	15.850	19.165
20	0.0597	16.366	18.781	17.725	22.461	18.511
20	0.0607	18.420	19.442	12.593	19.102	12.347
20	0.0617	19.302	15.886	8.571	18.781	20.227
20	0.0627	16.635	8.291	11.414	26.445	22.299
20	0.0637	8.844	14.975	10.841	14.755	4.113
20	0.0647	11.239	17.978	10.703	14.784	9.327
20	0.0657	11.508	23.784	11.996	14.643	11.365
20	0.0667	14.467	9.465	10.632	11.821	10.291
20	0.0677	11.466	12.957	11.669	11.070	9.667
20	0.0687	6.936	23.528	15.974	21.858	7.576
20	0.0697	6.162	11.667	9.839	23.603	11.250
20	0.0707	12.738	25.272	19.655	31.618	15.687
20	0.0717	17.525	13.081	13.513	14.794	8.921
20	0.0727	10.413	9.101	10.835	9.179	8.912
20	0.0737	12.477	10.433	12.034	15.870	6.444

20	0.0747	10.923	14.520	16.208	18.636	20.455
20	0.0757	10.817	9.415	8.181	10.456	9.406
20	0.0767	11.746	13.976	11.130	10.266	5.232
20	0.0777	10.782	9.434	9.510	10.954	7.496
20	0.0787	18.892	12.860	12.999	11.517	16.780
20	0.0797	14.922	15.030	9.483	9.283	12.432
20	0.0807	13.575	15.653	9.482	15.839	15.315
20	0.0817	16.928	11.214	13.118	15.162	12.717
20	0.0827	12.183	8.272	8.136	9.836	9.191
20	0.0837	16.449	7.895	14.063	11.937	16.912
20	0.0847	9.403	3.826	8.744	14.211	8.983
20	0.0857	2.633	4.318	3.266	18.833	9.511
20	0.0867	2.693	1.295	1.701	3.794	3.191
20	0.0877	4.448	6.801	8.996	14.841	11.618
20	0.0887	8.783	5.215	9.407	10.257	13.536
20	0.0897	10.844	7.415	13.803	11.137	10.707
20	0.0907	6.829	5.325	9.711	6.530	7.612
20	0.0917	13.028	5.448	9.901	9.783	13.092
20	0.0927	7.425	2.951	5.631	8.546	11.274
20	0.0937	8.624	6.226	13.825	6.428	11.864
20	0.0947	13.054	11.790	14.478	8.693	16.740
20	0.0957	6.828	5.548	12.018	6.793	9.776
20	0.0967	9.675	8.759	16.238	12.471	17.856
20	0.0977	7.357	5.465	12.824	13.548	13.204
20	0.0987	9.796	8.509	7.746	8.299	10.567
20	0.0997	11.620	9.888	12.396	13.067	19.523
20	0.1007	9.962	11.204	8.595	11.904	15.685
20	0.1017	7.281	2.556	9.596	4.463	5.658
20	0.1027	13.995	11.157	16.807	10.831	8.376
20	0.1037	13.988	11.230	16.854	11.008	8.704
20	0.1047	6.928	8.246	8.570	8.034	9.245
20	0.1057	8.784	12.055	7.924	14.241	8.778
20	0.1067	10.487	6.129	10.402	12.750	7.566
20	0.1077	13.571	13.212	13.429	13.536	10.197
20	0.1087	8.150	10.638	11.188	19.357	6.422
20	0.1097	4.003	5.983	5.926	10.161	7.525
20	0.1107	7.973	8.976	16.483	7.526	8.412
20	0.1117	13.085	10.509	14.002	13.957	4.850
20	0.1127	14.363	11.685	14.606	18.199	9.865
20	0.1137	11.470	12.507	14.032	7.308	13.233
20	0.1147	8.525	10.771	13.304	10.626	10.200
20	0.1157	14.347	10.127	18.302	10.635	11.099
20	0.1167	17.636	8.843	12.377	13.764	21.744
20	0.1177	7.993	6.013	8.289	7.998	4.577
20	0.1187	14.081	6.145	9.815	14.572	11.042
20	0.1197	14.380	10.487	11.606	10.881	8.266
20	0.1207	15.135	16.368	17.222	5.405	5.152
20	0.1217	10.995	20.623	13.851	18.892	7.312
20	0.1227	27.591	10.721	20.780	20.140	27.674
20	0.1237	27.615	10.695	20.788	20.199	27.749

20	0.1247	16.572	17.127	19.055	11.188	20.157
20	0.1257	12.321	13.549	9.977	15.784	26.345
20	0.1267	15.189	13.293	15.759	22.420	15.677
20	0.1277	16.696	22.109	18.358	20.932	19.181
20	0.1287	24.168	14.246	15.896	12.849	20.061
20	0.1297	21.952	13.858	14.989	11.545	11.557
20	0.1307	11.909	11.025	11.477	14.116	22.347
20	0.1317	19.024	12.540	19.097	14.016	12.565
20	0.1327	19.558	17.387	18.648	18.492	21.693
20	0.1337	14.590	14.686	14.932	15.392	13.506
20	0.1347	15.127	17.704	16.362	11.482	5.604
20	0.1357	20.952	10.804	17.152	12.062	6.192
20	0.1367	18.378	16.094	18.774	17.761	13.757
20	0.1377	22.559	21.755	16.472	18.180	19.779
20	0.1387	8.998	5.660	10.157	14.085	7.766
20	0.1397	2.589	0.751	2.508	4.165	2.519
20	0.1407	5.818	3.019	7.649	2.382	1.799
20	0.1417	9.123	8.716	10.536	6.350	8.188
20	0.1427	13.890	12.502	11.694	9.547	12.381
20	0.1437	17.390	13.030	13.552	15.455	13.060
20	0.1447	12.488	10.790	13.089	15.158	11.068
20	0.1457	10.659	2.676	10.303	16.249	5.355
20	0.1467	20.634	9.183	13.447	12.477	9.790
20	0.1477	22.150	17.787	18.496	7.999	17.126
20	0.1487	18.425	26.843	21.818	12.174	9.266
20	0.1497	10.793	12.049	9.709	11.050	16.256
20	0.1507	6.091	6.540	7.057	9.012	12.918
20	0.1517	7.873	5.376	7.342	12.627	10.687
20	0.1527	13.093	8.811	10.552	9.485	4.964
20	0.1537	17.396	17.549	19.993	11.567	24.158
20	0.1547	18.786	17.934	13.861	14.928	25.127
20	0.1557	13.608	14.053	10.563	19.783	15.164
20	0.1567	12.133	4.594	8.794	12.010	18.614
20	0.1577	13.151	14.237	16.898	15.165	12.948
20	0.1587	10.580	13.851	15.507	22.132	29.737
20	0.1597	13.274	11.530	13.189	20.347	18.029
20	0.1607	12.919	13.027	11.428	12.097	12.547
20	0.1617	6.926	7.411	11.188	12.739	11.309
20	0.1627	13.507	10.776	15.541	11.867	10.018
20	0.1637	9.762	7.736	7.969	9.608	5.325
20	0.1647	9.288	9.014	6.355	10.924	11.907
20	0.1657	9.809	12.400	15.568	9.592	9.320
20	0.1667	18.199	17.079	21.365	11.797	8.647
20	0.1677	4.180	4.275	5.907	7.045	11.149
20	0.1687	7.268	13.163	8.128	16.327	9.833
20	0.1697	16.083	18.493	13.370	21.724	17.672
20	0.1707	14.178	13.586	16.988	17.597	20.223
20	0.1717	12.128	7.498	12.187	18.318	13.991
20	0.1727	9.920	10.052	12.169	12.788	13.609
20	0.1737	15.754	19.872	20.138	16.091	25.467

20	0.1747	16.232	12.912	17.343	7.563	28.492
20	0.1757	14.150	14.900	17.610	10.723	27.870
20	0.1767	19.973	7.368	16.305	15.004	16.665
20	0.1777	12.494	14.222	16.060	14.457	9.332
20	0.1787	8.012	7.979	11.494	18.640	21.046
20	0.1797	5.839	10.781	10.154	14.282	23.891
20	0.1807	6.416	4.965	7.106	16.909	21.194
20	0.1817	8.770	6.584	5.504	11.583	15.485
20	0.1827	7.519	1.592	4.559	11.471	8.923
20	0.1837	7.749	9.388	9.199	9.367	5.808
20	0.1847	11.789	11.201	14.303	8.186	8.094
20	0.1857	6.117	6.634	7.981	4.499	14.684
20	0.1867	15.671	13.020	12.263	8.950	17.431
20	0.1877	6.961	5.804	7.286	14.496	15.416
20	0.1887	10.015	13.538	13.747	20.510	9.333
20	0.1897	8.030	4.008	11.679	15.208	21.199
20	0.1907	18.509	12.616	22.077	20.074	10.611
20	0.1917	35.061	17.053	22.934	11.157	11.814
20	0.1927	18.601	12.779	13.024	18.630	18.074
20	0.1937	28.220	9.056	15.637	22.524	26.028
20	0.1947	27.242	21.401	29.314	15.498	17.246
20	0.1957	15.051	9.993	16.221	10.378	12.456
20	0.1967	18.723	14.145	14.634	10.377	19.384
20	0.1977	17.192	13.806	14.029	17.504	23.658
20	0.1987	16.942	22.738	22.059	10.377	16.769
20	0.1997	14.145	13.023	8.398	4.736	18.118
20	0.2007	16.342	14.305	16.747	20.418	13.575
20	0.2017	14.978	9.036	14.294	22.424	13.962
20	0.2027	7.290	9.613	5.989	26.765	11.753
20	0.2037	9.676	7.418	12.765	12.148	15.123
20	0.2047	13.283	16.828	16.802	27.656	24.245
20	0.2057	13.350	9.378	7.835	25.809	16.672
20	0.2067	10.277	7.823	10.215	17.922	22.741
20	0.2077	23.236	28.180	27.424	26.923	20.965
20	0.2087	16.069	21.777	20.252	20.009	14.957
20	0.2097	8.377	9.408	9.545	12.674	24.730
20	0.2107	13.244	16.418	12.950	16.004	25.161
20	0.2117	9.209	8.907	12.260	12.388	28.889
20	0.2127	11.168	15.599	9.759	15.747	26.274
20	0.2137	14.492	10.609	11.408	7.325	13.757
20	0.2147	9.056	14.203	18.510	13.836	23.054
20	0.2157	8.401	5.578	11.628	16.168	22.197
20	0.2167	7.661	7.461	10.240	13.829	20.994
20	0.2177	8.435	10.150	10.914	23.042	27.172
20	0.2187	8.440	10.161	10.886	23.031	27.074
20	0.2197	9.579	11.536	14.044	4.186	6.240
20	0.2207	10.657	16.574	18.308	11.581	15.273
20	0.2217	9.575	5.028	7.708	16.514	31.314
20	0.2227	11.152	9.533	10.711	17.211	14.772
20	0.2237	9.970	9.790	13.418	13.509	12.479

20	0.2247	8.996	10.338	12.489	15.023	16.166
20	0.2257	23.474	21.926	17.024	23.141	13.088
20	0.2267	14.739	11.968	8.992	19.600	19.174
20	0.2277	16.004	17.486	17.556	11.948	21.910
20	0.2287	27.434	20.790	14.686	15.304	8.799
20	0.2297	20.098	19.707	24.775	20.254	23.078
20	0.2307	13.988	14.901	17.138	12.744	18.524
20	0.2317	24.449	20.145	18.267	17.854	23.709
20	0.2327	10.160	8.639	10.810	12.242	11.019
20	0.2337	7.865	7.078	10.577	7.747	11.611
20	0.2347	8.206	8.587	11.432	8.800	12.810
20	0.2357	9.879	10.319	14.510	6.618	6.214
20	0.2367	6.097	6.028	7.552	4.290	5.686
20	0.2377	1.876	3.695	2.058	6.233	6.899
20	0.2387	8.465	12.025	11.172	12.575	19.700
20	0.2397	10.200	6.638	12.618	21.227	26.828
20	0.2407	21.254	22.680	20.834	17.834	30.789
20	0.2417	21.605	17.365	17.555	12.735	21.931
20	0.2427	16.872	10.557	16.797	18.587	27.421
20	0.2437	9.050	6.915	9.944	12.589	18.233
20	0.2447	15.700	12.242	13.511	8.376	17.731
20	0.2457	7.454	5.440	8.474	7.581	25.379
20	0.2467	11.393	9.757	10.740	7.995	19.990
20	0.2477	10.309	8.682	9.895	18.437	13.567
20	0.2487	8.074	5.826	5.102	6.986	30.449
20	0.2497	16.154	10.879	14.805	11.410	30.946
20	0.2507	18.215	11.753	12.827	14.602	29.529
20	0.2517	8.225	7.024	4.810	10.685	14.242
20	0.2527	10.456	9.426	13.824	13.882	14.649
20	0.2537	7.906	13.544	7.166	26.824	42.487
20	0.2547	7.912	13.451	7.108	26.919	42.624
20	0.2557	6.331	8.619	6.752	11.316	26.815
20	0.2567	8.210	8.742	9.439	12.442	20.916
20	0.2577	4.804	6.867	3.367	7.900	7.143
20	0.2587	8.204	13.980	9.985	6.959	7.803
20	0.2597	23.049	16.499	12.384	3.974	11.538
20	0.2607	27.524	17.455	12.403	7.247	8.178
20	0.2617	17.545	14.346	14.347	18.597	24.243
20	0.2627	17.927	11.835	10.638	8.857	23.506
20	0.2637	24.457	12.826	11.442	9.692	19.722
20	0.2647	11.724	6.708	4.867	13.570	13.219
20	0.2657	14.041	9.840	10.067	21.037	12.902
20	0.2667	29.260	13.054	13.596	17.349	17.561
20	0.2677	13.870	13.416	15.314	15.294	14.991
20	0.2687	9.358	12.323	15.071	25.185	15.883
20	0.2697	12.046	18.579	12.673	12.972	16.705
20	0.2707	12.256	13.705	14.308	20.218	9.639
20	0.2717	11.635	10.539	12.404	25.955	19.843
20	0.2727	4.770	11.416	7.699	19.333	17.970
20	0.2737	21.563	12.376	13.411	20.330	20.395

20	0.2747	16.652	13.445	19.331	15.665	15.611
20	0.2757	15.581	12.171	22.505	9.332	12.830
20	0.2767	15.077	13.406	12.633	16.278	10.974
20	0.2777	7.071	10.886	8.270	25.097	13.422
20	0.2787	20.373	12.583	20.115	12.282	9.955
20	0.2797	20.650	13.210	14.196	15.703	10.852
20	0.2807	11.696	11.428	12.714	14.923	9.595
20	0.2817	16.546	10.065	15.575	21.865	12.479
20	0.2827	6.841	6.120	12.197	17.102	9.663
20	0.2837	15.629	7.484	12.344	9.670	15.065
20	0.2847	8.481	5.598	5.656	13.950	15.800
20	0.2857	24.506	10.289	11.109	16.083	15.404
20	0.2867	13.966	7.316	14.172	24.146	19.393
20	0.2877	6.084	5.591	9.208	9.186	10.695
20	0.2887	7.109	2.683	6.833	19.425	8.473
20	0.2897	12.138	11.019	12.675	10.981	11.118
20	0.2907	7.167	8.176	11.504	15.964	13.321
20	0.2917	5.464	7.420	5.018	12.455	12.328
20	0.2927	9.007	6.487	10.837	15.100	15.804
20	0.2937	7.441	4.170	9.356	15.781	15.233
20	0.2947	5.972	0.957	4.510	10.458	8.266
20	0.2957	7.307	6.851	8.363	14.809	12.077
20	0.2967	4.867	12.015	10.789	15.291	16.185
20	0.2977	5.395	4.420	7.529	13.590	16.432
20	0.2987	1.946	5.813	4.664	13.477	13.762
20	0.2997	12.232	2.604	9.142	11.792	4.297
20	0.3007	17.183	7.828	7.870	17.042	20.749
20	0.3017	17.187	7.871	7.864	17.155	20.768
20	0.3027	8.613	7.749	14.899	8.899	10.679
20	0.3037	10.896	8.682	15.905	17.288	9.547
20	0.3047	5.389	6.200	9.864	18.465	19.263
20	0.3057	6.571	3.261	4.782	7.945	8.176
20	0.3067	6.297	8.453	8.565	14.482	11.379
20	0.3077	9.805	10.113	10.454	21.893	27.679
20	0.3087	8.122	4.000	9.404	7.871	9.486
20	0.3097	5.450	9.374	6.726	9.369	9.117
20	0.3107	16.560	13.055	9.582	19.177	9.738
20	0.3117	16.615	11.859	10.528	17.037	11.657
20	0.3127	4.199	9.134	8.186	8.662	9.626
20	0.3137	19.032	12.561	17.806	8.255	7.244
20	0.3147	12.364	7.306	6.352	11.949	7.746
20	0.3157	9.606	3.819	4.649	16.714	9.981
20	0.3167	9.528	12.274	8.830	14.927	16.593
20	0.3177	11.876	12.251	13.590	19.895	18.674
20	0.3187	10.983	5.547	6.955	12.009	11.659
20	0.3197	6.863	11.322	6.564	26.744	19.292
20	0.3207	3.972	6.465	6.648	12.166	15.203
20	0.3217	8.870	10.405	10.331	12.370	15.492
20	0.3227	5.677	8.666	8.309	5.199	10.476
20	0.3237	6.603	4.827	7.300	8.196	11.846

20	0.3247	9.227	10.141	12.953	13.586	21.490
20	0.3257	9.370	7.161	9.306	15.637	9.682
20	0.3267	10.965	5.840	7.476	19.276	16.249
20	0.3277	11.520	6.125	12.169	19.079	10.614
20	0.3287	7.640	3.128	6.428	16.473	7.858
20	0.3297	7.423	2.484	7.715	27.513	8.135
20	0.3307	8.852	9.583	9.274	18.914	6.820
20	0.3317	7.663	8.331	7.545	14.438	15.444
20	0.3327	16.382	12.685	19.958	12.208	9.512
20	0.3337	9.694	7.951	15.550	11.955	8.390
20	0.3347	8.210	7.610	8.808	14.154	9.421
20	0.3357	11.267	15.727	9.814	20.154	10.482
20	0.3367	11.077	17.591	18.210	20.572	12.763
20	0.3377	16.227	14.530	22.713	16.526	11.361
20	0.3387	13.980	13.263	17.706	21.273	15.011
20	0.3397	10.967	7.703	12.041	18.900	14.518
20	0.3407	5.052	14.107	7.254	16.325	8.333
20	0.3417	15.056	13.337	17.173	15.184	8.349
20	0.3427	10.159	8.243	12.754	9.637	3.246
20	0.3437	9.287	8.187	6.254	9.712	5.163
20	0.3447	14.183	10.184	8.950	12.159	6.635
20	0.3457	17.763	9.154	10.036	15.903	18.631
20	0.3467	14.790	12.618	9.381	11.959	10.204
20	0.3477	13.361	12.815	9.313	11.070	7.524
20	0.3487	19.674	11.068	15.883	13.666	8.582
20	0.3497	14.572	17.736	15.192	20.665	14.936
20	0.3507	16.503	17.307	15.523	25.048	13.992
20	0.3517	8.405	6.872	6.259	20.189	14.657
20	0.3527	7.104	5.715	5.961	12.375	13.024
20	0.3537	13.256	14.905	16.755	16.343	14.565
20	0.3547	9.153	8.902	9.793	21.457	25.955
20	0.3557	6.201	5.585	5.544	15.478	16.618
20	0.3567	16.404	9.626	13.804	14.216	10.964
20	0.3577	20.080	8.033	17.022	26.735	11.651
20	0.3587	17.981	9.479	16.161	26.616	23.343
20	0.3597	23.544	16.094	26.093	27.112	14.411
20	0.3607	10.231	9.836	8.383	24.956	15.075
20	0.3617	1.891	4.081	4.029	5.720	2.522
20	0.3627	7.582	3.978	9.322	19.970	6.783
20	0.3637	11.896	8.513	13.710	21.694	12.165
20	0.3647	10.317	4.610	10.730	22.241	19.035
20	0.3657	16.792	4.509	8.145	17.480	23.906
20	0.3667	8.625	1.410	3.090	16.215	10.371
20	0.3677	7.400	5.119	4.361	12.315	6.306
20	0.3687	5.298	5.217	4.360	8.450	6.746
20	0.3697	10.865	5.386	6.721	13.030	12.768
20	0.3707	11.195	4.971	7.048	13.844	13.153
20	0.3717	9.524	8.228	9.080	31.570	16.648
20	0.3727	7.136	12.646	12.340	22.201	7.073
20	0.3737	4.364	10.969	6.688	22.262	4.110

20	0.3747	13.101	14.295	15.163	8.790	3.401
20	0.3757	8.382	5.481	7.030	8.562	5.367
20	0.3767	6.702	3.167	8.068	21.317	14.901
20	0.3777	7.132	6.038	9.714	22.830	7.617
20	0.3787	6.387	5.388	9.196	19.721	5.696
20	0.3797	21.663	10.312	24.685	21.917	20.957
20	0.3807	9.068	12.606	9.008	19.023	12.708
20	0.3817	7.438	9.304	9.259	25.294	11.060
20	0.3827	11.740	10.616	10.062	18.382	4.247
20	0.3837	9.259	9.841	13.613	13.611	13.930
20	0.3847	16.248	7.505	19.041	12.012	4.799
20	0.3857	9.967	7.384	7.552	16.040	10.960
20	0.3867	10.169	16.221	11.356	19.914	12.306
20	0.3877	6.707	14.773	8.428	24.036	12.571
20	0.3887	7.896	12.151	8.659	20.629	9.075
20	0.3897	10.820	10.048	10.369	24.711	11.107
20	0.3907	11.176	8.299	9.358	29.167	17.946
20	0.3917	9.996	7.182	12.418	25.137	24.432
20	0.3927	12.667	11.202	19.487	23.869	14.169
20	0.3937	9.551	4.930	13.997	15.513	6.773
20	0.3947	9.545	4.893	13.951	15.482	6.756
20	0.3957	16.788	11.593	17.715	15.556	15.482
20	0.3967	13.684	10.482	14.733	18.560	13.278
20	0.3977	11.341	7.157	11.083	23.223	10.270
20	0.3987	6.040	8.216	7.519	26.518	13.830
20	0.3997	4.793	7.723	8.137	16.366	13.595
20	0.4007	4.943	11.698	9.905	22.634	27.114
20	0.4017	11.151	10.235	15.321	17.237	11.368
20	0.4027	23.425	16.341	21.664	17.137	14.493
20	0.4037	10.183	11.079	15.763	24.305	15.029
20	0.4047	11.254	12.448	9.952	27.972	18.728
20	0.4057	12.811	15.255	13.456	20.147	18.296
20	0.4067	12.546	9.789	9.582	20.803	13.895
20	0.4077	14.409	10.308	14.794	9.174	9.659
20	0.4087	9.703	8.153	9.810	8.326	11.378
20	0.4097	8.661	11.851	13.559	19.418	8.418
20	0.4107	9.504	7.602	10.661	15.120	8.690
20	0.4117	12.189	11.776	17.535	7.175	11.299
20	0.4127	16.848	12.471	18.781	24.504	11.218
20	0.4137	15.398	11.468	20.552	20.431	10.089
20	0.4147	14.805	12.147	17.713	14.674	10.718
20	0.4157	16.854	16.986	19.247	23.417	14.815
20	0.4167	15.267	8.710	18.302	31.539	14.023
20	0.4177	14.583	6.727	13.206	27.817	24.638
20	0.4187	9.606	7.257	7.855	22.630	17.900
20	0.4197	11.711	7.311	17.085	15.206	7.007
20	0.4207	7.928	1.324	10.840	7.713	5.466
20	0.4217	19.270	26.067	27.981	23.828	10.187
20	0.4227	13.712	21.972	20.083	24.543	17.897
20	0.4237	9.224	21.512	16.258	24.455	20.788

20	0.4247	12.273	17.315	19.107	26.033	8.104
20	0.4257	7.552	6.340	9.540	25.932	10.230
20	0.4267	14.359	14.229	18.480	22.264	7.252
20	0.4277	16.264	15.272	20.954	19.368	3.280
20	0.4287	13.005	13.777	15.089	16.807	7.423
20	0.4297	11.854	15.725	20.634	31.077	18.786
20	0.4307	13.444	15.977	22.475	16.348	8.398
20	0.4317	13.438	15.939	22.493	16.329	8.450
20	0.4327	14.734	18.284	21.005	28.616	21.915
20	0.4337	23.526	19.706	14.406	25.797	15.675
20	0.4347	15.973	19.356	17.698	26.113	21.004
20	0.4357	18.258	17.283	17.433	15.856	16.303
20	0.4367	18.310	17.355	17.289	15.701	16.654
20	0.4377	14.822	10.177	15.671	20.970	17.163
20	0.4387	17.138	22.472	17.974	19.444	13.792
20	0.4397	11.580	18.143	15.232	26.861	15.669
20	0.4407	7.678	14.621	10.493	17.467	17.131
20	0.4417	9.163	16.265	13.896	25.271	14.508
20	0.4427	21.187	21.804	26.156	21.272	9.237
20	0.4437	10.679	15.509	13.874	23.350	12.344
20	0.4447	5.663	10.202	9.825	18.051	14.409
20	0.4457	5.657	10.200	9.787	18.051	14.358
20	0.4467	10.214	14.135	13.582	21.732	20.420
20	0.4477	10.211	14.127	13.551	21.689	20.353
20	0.4487	12.395	11.374	12.958	11.243	12.839
20	0.4497	10.982	12.730	11.126	16.026	8.877
20	0.4507	22.444	28.747	28.786	19.531	12.174
20	0.4517	22.687	27.840	28.682	24.116	14.090
20	0.4527	13.837	18.684	16.047	23.546	16.373
20	0.4537	8.642	14.255	8.759	21.536	8.079
20	0.4547	7.063	16.057	11.902	18.627	6.970
20	0.4557	23.317	26.644	22.508	28.757	7.503
20	0.4567	19.018	24.044	20.532	23.926	10.056
20	0.4577	18.489	17.762	19.428	16.267	15.583
20	0.4587	8.810	16.755	10.773	10.854	9.813
20	0.4597	10.272	8.435	11.739	18.692	5.073
20	0.4607	12.035	14.372	13.268	30.453	11.122
20	0.4617	12.612	10.758	14.029	8.179	10.129
20	0.4627	7.730	12.387	7.946	26.238	10.466
20	0.4637	8.303	9.827	8.985	19.678	12.134
20	0.4647	8.085	11.612	10.802	15.900	10.338
20	0.4657	17.512	21.540	19.361	15.233	10.373
20	0.4667	15.954	20.664	21.711	9.909	9.041
20	0.4677	25.056	25.426	27.065	18.994	6.404
20	0.4687	14.596	14.059	16.877	22.223	9.707
20	0.4697	17.141	13.645	15.922	10.808	12.803
20	0.4707	6.160	8.741	10.898	8.426	12.899
20	0.4717	8.343	12.056	13.249	17.242	9.296
20	0.4727	12.775	14.730	17.667	19.227	9.880
20	0.4737	8.172	10.343	7.807	18.051	23.868

20	0.4747	7.607	11.655	9.861	21.495	17.379
20	0.4757	6.043	11.351	10.482	23.141	5.484
20	0.4767	1.433	10.877	3.299	19.284	2.048
20	0.4777	6.168	13.724	10.922	15.095	3.536
20	0.4787	4.593	11.970	8.761	8.886	7.592
20	0.4797	8.128	11.349	13.894	20.082	8.361
20	0.4807	4.379	9.277	6.093	14.553	10.326
20	0.4817	12.525	10.021	16.302	6.846	7.594
20	0.4827	7.569	4.571	9.896	8.585	11.502
20	0.4837	8.002	7.408	11.407	11.465	13.420
20	0.4847	6.536	10.001	8.591	15.436	9.315
20	0.4857	11.913	11.751	15.196	21.260	9.108
20	0.4867	8.839	7.537	7.782	23.950	18.584
20	0.4877	8.825	9.816	8.173	17.909	16.605
20	0.4887	6.965	10.847	6.664	13.443	15.021
20	0.4897	6.959	10.871	6.614	13.646	14.956
20	0.4907	4.869	3.991	4.276	15.938	13.984
20	0.4917	4.865	3.991	4.274	15.937	14.003
20	0.4927	6.578	11.492	8.279	15.686	13.285
20	0.4937	5.242	15.446	5.354	29.588	8.420
20	0.4947	9.111	17.247	9.587	29.839	10.777
20	0.4957	10.086	18.542	13.497	19.317	11.774
20	0.4967	9.378	11.064	11.635	35.599	9.454
20	0.4977	19.130	23.608	14.162	22.655	16.070
20	0.4987	13.017	17.648	10.878	22.666	13.156
20	0.4997	11.195	15.467	13.573	20.261	28.848
20	0.5007	9.160	9.432	13.384	16.605	7.320
20	0.5017	8.848	16.652	12.329	22.763	9.528
20	0.5027	9.548	22.554	12.979	27.139	7.053
20	0.5037	12.217	11.064	11.722	24.935	6.595
20	0.5047	13.647	13.837	12.341	25.587	7.158
20	0.5057	17.492	16.133	17.393	26.304	8.678
20	0.5067	9.479	15.667	12.327	23.730	9.509
20	0.5077	15.837	15.578	15.296	21.717	20.284
20	0.5087	9.305	13.741	7.566	12.843	14.433
20	0.5097	11.755	13.554	14.200	11.815	20.698
20	0.5107	26.406	20.207	20.955	19.323	22.480
20	0.5117	9.912	15.129	11.165	20.559	19.057
20	0.5127	12.200	17.381	13.584	27.050	24.399
20	0.5137	16.776	20.736	16.592	27.618	23.994
20	0.5147	9.675	17.193	11.117	11.668	17.172
20	0.5157	18.366	16.978	16.306	15.913	10.625
20	0.5167	5.518	8.741	6.583	23.029	13.186
20	0.5177	8.506	13.570	9.574	17.328	13.808
20	0.5187	11.806	17.005	15.782	26.674	15.931
20	0.5197	7.704	7.649	9.186	10.863	7.376
20	0.5207	9.438	7.316	5.732	21.784	13.118
20	0.5217	12.862	24.347	14.836	24.879	15.534
20	0.5227	16.621	13.774	13.566	26.278	8.767
20	0.5237	11.814	11.880	17.331	12.560	9.653

20	0.5247	16.922	15.015	8.678	26.646	15.759
20	0.5257	15.920	14.555	15.802	12.686	7.101
20	0.5267	7.158	10.059	7.511	16.384	8.814
20	0.5277	6.952	7.851	5.640	20.322	11.973
20	0.5287	13.176	10.289	13.905	15.789	5.913
20	0.5297	9.257	12.261	11.117	18.449	6.815
20	0.5307	10.238	10.002	8.502	19.752	21.322
20	0.5317	16.572	18.957	15.206	14.989	25.056
20	0.5327	14.138	22.010	13.467	20.777	21.319
20	0.5337	11.752	18.837	13.165	11.730	11.144
20	0.5347	14.595	18.412	21.092	15.331	18.775
20	0.5357	16.751	21.422	22.682	15.945	19.156
20	0.5367	9.004	11.508	13.357	17.571	16.919
20	0.5377	7.865	10.588	13.619	12.151	11.103
20	0.5387	13.826	16.503	14.368	15.001	17.779
20	0.5397	7.942	5.643	11.320	9.769	21.030
20	0.5407	8.987	9.549	14.474	21.171	19.907
20	0.5417	18.772	17.464	15.021	14.263	22.066
20	0.5427	15.359	12.491	16.483	9.478	14.983
20	0.5437	27.801	25.485	30.994	15.496	20.110
20	0.5447	19.628	9.037	16.256	11.941	11.318
20	0.5457	5.162	5.487	5.382	20.970	12.165
20	0.5467	5.821	9.646	8.858	19.839	19.314
20	0.5477	8.377	13.793	14.007	22.289	17.873
20	0.5487	12.441	19.225	15.444	24.064	18.279
20	0.5497	9.665	13.794	14.974	14.139	22.403
20	0.5507	13.770	9.078	14.356	14.343	26.880
20	0.5517	18.175	8.927	11.883	10.499	9.662
20	0.5527	21.617	11.080	15.481	9.626	12.402
20	0.5537	13.913	9.235	11.886	15.611	13.416
20	0.5547	11.037	8.518	12.973	11.577	4.591
20	0.5557	22.578	19.476	22.960	12.448	18.444
20	0.5567	17.006	11.862	21.626	18.679	16.457
20	0.5577	12.593	13.388	14.436	20.984	32.454
20	0.5587	14.667	12.470	19.002	28.611	25.768
20	0.5597	13.617	14.776	18.098	22.357	19.558
20	0.5607	19.420	17.969	24.104	30.187	22.995
20	0.5617	17.469	15.413	22.075	14.231	15.847
20	0.5627	11.264	12.032	15.856	11.582	16.113
20	0.5637	18.079	11.998	21.714	24.868	15.267
20	0.5647	16.226	10.468	14.961	20.773	18.448
20	0.5657	19.127	11.418	16.464	21.629	19.573
20	0.5667	16.544	6.419	14.186	19.570	12.156
20	0.5677	11.122	7.120	11.636	15.859	17.309
20	0.5687	19.097	20.053	25.026	25.790	23.072
20	0.5697	8.852	4.945	9.059	8.791	9.798
20	0.5707	15.736	20.466	21.355	18.095	21.108
20	0.5717	6.374	6.615	5.613	5.613	7.112
20	0.5727	5.414	5.409	9.377	11.286	10.578
20	0.5737	23.028	20.033	28.583	19.015	31.141

20	0.5747	14.955	10.167	19.686	15.701	13.224
20	0.5757	13.354	11.081	13.674	11.954	16.040
20	0.5767	10.435	10.778	10.507	13.680	26.676
20	0.5777	21.419	22.851	21.444	21.288	25.047
20	0.5787	18.886	18.601	14.747	18.990	35.716
20	0.5797	33.580	27.653	43.317	18.228	25.568
20	0.5807	17.754	14.771	23.379	16.619	24.630
20	0.5817	17.542	12.649	16.378	24.186	15.567
20	0.5827	19.381	15.940	16.228	20.809	21.253
20	0.5837	10.175	14.136	10.039	15.998	20.232
20	0.5847	18.183	15.415	13.217	35.026	34.236
20	0.5857	17.426	14.392	17.960	20.958	16.456
20	0.5867	12.230	14.309	16.838	17.648	25.972
20	0.5877	9.651	7.755	8.858	19.050	15.455
20	0.5887	7.978	5.435	7.031	24.450	14.756
20	0.5897	7.062	8.327	9.504	36.513	24.036
20	0.5907	14.665	8.689	13.654	24.080	18.621
20	0.5917	18.202	9.788	17.534	36.122	36.337
20	0.5927	7.353	4.297	6.008	10.633	8.383
20	0.5937	17.473	11.237	20.084	13.362	10.738
20	0.5947	11.157	8.164	12.904	10.895	10.196
20	0.5957	11.432	12.113	12.319	26.829	24.923
20	0.5967	11.777	16.549	15.197	27.703	28.094
20	0.5977	4.910	11.591	5.593	35.610	27.919
20	0.5987	7.812	12.362	8.672	31.635	23.059
20	0.5997	8.876	10.999	10.719	31.203	29.284
20	0.6007	4.654	14.001	3.328	33.838	16.020
20	0.6017	8.879	14.534	9.264	23.130	22.279
20	0.6027	14.944	19.040	10.058	20.694	17.606
20	0.6037	15.023	20.034	10.604	20.839	19.668
20	0.6047	14.340	12.907	11.907	25.434	22.328
20	0.6057	7.037	11.224	14.932	20.860	17.875
20	0.6067	8.131	9.721	16.824	15.798	18.648
20	0.6077	10.307	14.504	16.087	20.273	23.186
20	0.6087	22.067	17.365	22.416	14.999	12.621
20	0.6097	12.254	9.305	18.300	19.872	16.245
20	0.6107	20.398	11.508	11.372	24.518	21.161
20	0.6117	11.531	9.335	13.866	23.433	15.359
20	0.6127	11.402	11.517	13.835	12.870	18.791
20	0.6137	12.525	11.122	11.006	24.878	22.918
20	0.6147	13.102	12.255	15.148	22.487	18.935
20	0.6157	12.296	14.984	19.637	9.837	24.296
20	0.6167	14.712	17.739	18.050	24.487	16.933
20	0.6177	13.190	16.388	15.012	25.134	17.581
20	0.6187	11.294	18.050	10.716	14.882	22.222
20	0.6197	18.233	17.905	15.058	20.560	13.889
20	0.6207	9.635	17.170	14.430	33.781	12.955
20	0.6217	16.865	18.138	15.895	24.266	12.156
20	0.6227	12.460	14.668	12.622	28.888	12.554
20	0.6237	10.925	9.483	12.978	11.356	9.517

20	0.6247	11.612	7.315	12.818	16.019	11.163
20	0.6257	9.514	13.878	12.972	26.250	32.820
20	0.6267	6.647	13.886	9.230	27.851	27.398
20	0.6277	8.081	10.545	15.515	14.134	20.694
20	0.6287	13.011	16.841	16.879	31.483	19.225
20	0.6297	10.232	14.534	15.211	21.112	19.129
20	0.6307	9.197	17.377	16.260	19.180	14.116
20	0.6317	11.206	16.602	15.559	22.505	15.128
20	0.6327	11.641	18.454	11.304	11.885	25.284
20	0.6337	14.316	17.802	12.893	11.350	18.051
20	0.6347	12.454	12.348	9.424	26.967	21.740
20	0.6357	18.013	13.133	14.640	10.763	25.701
20	0.6367	12.363	11.324	7.432	25.791	17.600
20	0.6377	20.259	19.537	23.424	13.487	11.678
20	0.6387	22.798	24.752	21.770	12.614	20.722
20	0.6397	18.070	16.380	17.542	8.494	17.648
20	0.6407	31.051	23.330	27.586	8.190	10.798
20	0.6417	30.443	24.802	24.996	14.479	18.432
20	0.6427	15.124	14.248	8.502	17.076	12.342
20	0.6437	22.086	19.958	15.689	21.563	14.789
20	0.6447	13.993	23.441	20.655	14.744	15.267
20	0.6457	9.214	14.708	8.908	6.805	9.129
20	0.6467	9.770	15.844	15.035	7.361	7.158
20	0.6477	11.164	14.191	13.848	12.444	22.543
20	0.6487	13.375	13.472	17.513	13.438	27.270
20	0.6497	13.371	13.485	17.481	13.420	27.352
20	0.6507	12.074	16.576	11.416	13.962	14.739
20	0.6517	13.858	22.544	13.947	12.424	28.467
20	0.6527	12.130	15.848	13.733	19.477	16.615
20	0.6537	8.568	12.271	4.227	3.347	6.814
20	0.6547	18.105	18.009	14.945	8.183	13.654
20	0.6557	11.429	22.424	13.094	14.490	12.644
20	0.6567	12.389	15.232	12.067	16.038	23.517
20	0.6577	17.064	18.673	21.368	13.852	7.884
20	0.6587	10.809	13.560	10.443	16.585	7.155
20	0.6597	15.530	13.964	9.136	9.361	6.888
20	0.6607	15.605	18.644	14.535	19.298	18.444
20	0.6617	16.647	22.472	21.446	14.507	18.821
20	0.6627	22.688	17.132	22.123	14.278	6.923
20	0.6637	15.177	10.070	11.838	18.382	10.587
20	0.6647	15.146	19.853	17.801	17.403	15.527
20	0.6657	19.394	24.433	24.414	9.675	17.354
20	0.6667	29.268	24.421	24.922	8.848	25.042
20	0.6677	31.217	28.241	25.300	9.399	20.652
20	0.6687	24.562	21.048	20.680	4.664	18.104
20	0.6697	39.827	26.943	29.302	7.208	13.119
20	0.6707	29.381	19.588	26.037	10.817	14.461
20	0.6717	33.677	30.563	40.684	8.962	19.551
20	0.6727	27.757	24.959	24.628	11.073	20.053
20	0.6737	15.712	18.766	18.601	10.817	18.920

20	0.6747	23.887	16.806	22.429	11.604	19.060
20	0.6757	14.225	8.713	11.005	6.334	11.693
20	0.6767	28.273	35.972	31.634	14.984	19.165
20	0.6777	22.293	27.827	23.339	13.655	12.702
20	0.6787	23.697	23.455	21.405	8.282	16.891
20	0.6797	24.575	22.172	21.400	14.531	21.897
20	0.6807	30.319	33.825	30.638	14.404	21.661
20	0.6817	24.265	29.172	25.238	18.010	13.664
20	0.6827	26.101	31.187	27.392	15.148	14.848
20	0.6837	25.477	24.648	17.617	9.666	18.100
20	0.6847	20.871	23.662	17.278	12.406	12.316
20	0.6857	20.307	18.547	16.119	12.390	10.521
20	0.6867	20.952	22.034	17.823	9.025	20.866
20	0.6877	23.693	18.129	18.104	5.793	19.071
20	0.6887	15.043	17.719	15.884	6.559	21.177
20	0.6897	14.929	21.221	20.279	9.160	10.924
20	0.6907	13.008	22.322	11.261	17.256	12.873
20	0.6917	22.099	21.830	22.449	11.216	20.379
20	0.6927	20.778	23.605	20.491	12.628	26.705
20	0.6937	15.211	19.174	13.289	14.916	23.376
20	0.6947	26.846	36.461	30.061	17.420	26.334
20	0.6957	21.105	27.074	23.064	8.845	11.363
20	0.6967	11.008	22.371	13.167	17.654	18.084
20	0.6977	21.821	27.025	24.425	11.723	12.641
20	0.6987	15.015	20.493	18.969	12.693	8.373
20	0.6997	19.106	25.030	21.822	12.860	11.271
20	0.7007	34.462	32.642	31.402	9.172	14.166
20	0.7017	22.921	30.082	21.120	11.725	12.672
20	0.7027	7.589	21.055	6.854	14.176	8.967
20	0.7037	7.161	13.291	6.641	13.336	12.668
20	0.7047	5.537	5.730	5.809	3.820	10.975
20	0.7057	3.735	4.355	6.712	2.384	10.038
20	0.7067	19.380	21.355	24.142	6.544	15.408
20	0.7077	19.416	21.197	24.100	6.424	14.951
20	0.7087	22.995	24.163	16.075	12.065	12.161
20	0.7097	20.790	19.907	19.564	9.557	13.492
20	0.7107	15.684	29.799	25.109	7.056	8.062
20	0.7117	0.000	0.000	0.000	0.000	0.000
21	0.0005	0.000	0.000	0.000	0.000	0.000
21	0.0015	18.491	13.919	15.248	11.801	13.071
21	0.0025	9.689	19.507	10.870	34.882	23.140
21	0.0035	13.070	9.434	8.244	29.768	11.302
21	0.0045	15.016	12.755	21.848	16.584	17.992
21	0.0055	9.884	5.951	14.648	14.031	15.918
21	0.0065	10.391	7.806	17.568	25.244	25.926
21	0.0075	22.855	22.728	33.239	19.065	20.708
21	0.0085	19.093	15.270	15.720	13.386	21.410
21	0.0095	22.169	15.963	24.439	17.435	14.828
21	0.0105	16.952	23.213	24.039	16.230	29.787
21	0.0115	23.107	19.612	15.212	25.910	28.329

21	0.0125	16.020	11.619	12.107	29.383	15.359
21	0.0135	15.017	7.060	8.671	25.714	29.249
21	0.0145	15.432	12.354	8.460	37.193	19.679
21	0.0155	7.972	13.452	7.366	26.396	35.693
21	0.0165	7.892	12.420	8.130	22.284	25.615
21	0.0175	10.364	13.086	11.879	18.225	35.508
21	0.0185	13.324	16.967	21.103	9.868	30.920
21	0.0195	13.476	11.992	13.918	14.088	36.219
21	0.0205	10.713	9.905	8.192	10.171	19.832
21	0.0215	16.022	21.020	19.439	21.722	16.679
21	0.0225	8.516	15.682	11.069	14.840	11.119
21	0.0235	5.173	17.083	9.397	13.873	14.364
21	0.0245	4.029	11.639	7.512	18.206	22.644
21	0.0255	7.902	8.981	6.280	19.546	8.751
21	0.0265	11.820	11.523	12.659	21.005	5.952
21	0.0275	14.827	6.896	12.016	10.673	12.324
21	0.0285	18.357	13.793	16.592	9.307	17.804
21	0.0295	14.817	13.019	15.817	19.020	27.731
21	0.0305	8.815	14.389	9.095	15.403	6.256
21	0.0315	17.993	14.143	11.278	20.028	12.790
21	0.0325	24.144	15.250	18.420	16.741	17.834
21	0.0335	10.111	10.863	12.105	16.697	23.656
21	0.0345	29.385	23.969	29.178	25.135	13.193
21	0.0355	17.077	14.256	16.735	14.505	17.430
21	0.0365	12.260	9.444	11.911	16.194	20.258
21	0.0375	18.361	19.391	20.843	13.066	23.817
21	0.0385	14.803	21.283	17.361	19.469	7.250
21	0.0395	25.244	24.985	22.691	7.872	9.889
21	0.0405	20.603	22.839	22.360	20.433	9.087
21	0.0415	23.908	27.151	26.666	9.325	8.144
21	0.0425	19.648	21.113	20.051	16.484	10.636
21	0.0435	18.280	23.222	21.886	20.011	15.624
21	0.0445	10.844	10.222	12.026	19.565	8.986
21	0.0455	24.277	23.382	18.554	15.566	9.722
21	0.0465	35.286	33.997	29.712	11.821	23.184
21	0.0475	21.497	13.670	15.137	13.291	19.174
21	0.0485	22.074	12.456	13.556	8.383	12.892
21	0.0495	22.186	12.628	13.759	8.490	12.935
21	0.0505	8.988	3.642	7.239	5.052	11.326
21	0.0515	9.588	7.135	10.261	13.541	7.193
21	0.0525	12.779	8.765	12.067	5.273	14.201
21	0.0535	10.392	9.897	10.358	9.680	6.770
21	0.0545	25.853	21.197	17.571	9.502	11.087
21	0.0555	25.719	21.116	17.538	9.547	10.783
21	0.0565	39.145	32.139	38.233	13.995	11.323
21	0.0575	28.688	34.742	28.530	12.701	7.238
21	0.0585	21.481	19.940	20.544	12.520	10.664
21	0.0595	23.576	13.138	18.941	13.214	11.585
21	0.0605	15.632	11.568	14.703	16.252	10.070
21	0.0615	22.401	20.500	25.235	17.852	7.087

21	0.0625	24.415	20.261	17.549	20.577	20.243
21	0.0635	28.406	20.830	26.315	21.791	14.628
21	0.0645	28.428	25.672	21.988	11.228	14.053
21	0.0655	22.211	21.234	19.405	18.020	19.331
21	0.0665	24.561	17.154	18.798	15.516	16.508
21	0.0675	9.455	10.400	8.632	5.753	8.070
21	0.0685	33.952	28.468	29.883	26.114	9.695
21	0.0695	13.521	13.279	14.447	7.996	5.920
21	0.0705	16.104	18.849	19.456	17.663	11.563
21	0.0715	15.242	14.061	12.230	15.898	6.265
21	0.0725	18.706	19.891	19.074	19.442	15.014
21	0.0735	28.526	23.885	25.294	15.945	6.477
21	0.0745	33.537	29.951	33.724	25.710	6.621
21	0.0755	29.919	21.034	36.803	22.192	6.039
21	0.0765	17.512	12.579	10.831	28.182	13.832
21	0.0775	11.485	13.579	13.312	15.174	3.314
21	0.0785	26.565	19.106	14.748	19.721	10.529
21	0.0795	45.185	31.492	29.977	34.357	11.642
21	0.0805	9.112	9.782	8.641	12.750	4.303
21	0.0815	10.216	6.403	6.565	15.700	6.149
21	0.0825	15.825	7.312	9.267	23.254	9.498
21	0.0835	25.805	12.352	19.643	7.521	12.790
21	0.0845	34.296	19.289	24.819	13.122	6.991
21	0.0855	34.396	19.345	24.917	13.161	6.936
21	0.0865	43.387	24.994	33.163	14.045	6.553
21	0.0875	24.892	12.545	19.093	27.990	11.561
21	0.0885	22.078	13.010	16.062	18.104	12.780
21	0.0895	13.335	9.670	9.962	21.758	16.344
21	0.0905	15.711	13.789	12.976	10.689	9.112
21	0.0915	12.672	13.850	13.861	15.583	13.999
21	0.0925	11.438	12.829	11.199	17.234	10.387
21	0.0935	18.746	14.794	13.431	11.867	17.096
21	0.0945	14.743	15.570	15.510	13.035	6.573
21	0.0955	15.203	18.954	14.696	18.645	7.207
21	0.0965	17.461	13.063	16.174	14.343	13.320
21	0.0975	9.049	7.984	9.708	7.291	6.433
21	0.0985	7.830	8.039	6.954	14.348	7.251
21	0.0995	28.732	16.612	18.657	10.065	8.562
21	0.1005	15.290	15.654	14.885	5.950	3.864
21	0.1015	20.340	16.670	18.896	22.530	11.960
21	0.1025	19.018	22.566	23.067	18.809	9.615
21	0.1035	22.590	18.480	23.593	15.385	27.081
21	0.1045	19.760	10.950	19.698	14.480	15.820
21	0.1055	11.437	7.729	14.975	6.927	10.181
21	0.1065	27.563	8.804	20.922	13.358	6.086
21	0.1075	24.468	23.635	24.802	9.451	13.711
21	0.1085	26.714	23.952	19.981	10.556	9.755
21	0.1095	14.015	9.555	12.737	10.469	15.800
21	0.1105	31.231	23.332	17.616	14.578	17.044
21	0.1115	27.864	16.207	24.129	13.016	9.108

21	0.1125	20.926	19.131	18.109	9.946	8.601
21	0.1135	21.446	20.698	19.143	17.530	18.487
21	0.1145	27.333	20.340	21.045	13.575	16.613
21	0.1155	16.587	7.762	8.502	9.458	26.901
21	0.1165	20.493	16.436	19.340	16.268	7.811
21	0.1175	14.189	11.129	14.130	14.751	7.945
21	0.1185	15.529	18.111	17.369	4.318	12.470
21	0.1195	14.395	12.191	16.473	9.342	10.080
21	0.1205	29.359	22.986	19.596	10.804	28.339
21	0.1215	10.208	7.957	9.774	11.820	18.279
21	0.1225	25.801	23.508	18.825	17.359	22.005
21	0.1235	18.092	14.442	14.524	14.481	20.398
21	0.1245	20.308	13.505	16.888	9.623	31.668
21	0.1255	23.211	23.456	21.888	13.098	10.820
21	0.1265	14.360	15.644	10.230	10.908	12.347
21	0.1275	16.642	18.221	13.056	22.022	15.103
21	0.1285	21.597	17.804	18.558	8.972	9.381
21	0.1295	23.596	19.771	19.033	12.190	12.693
21	0.1305	20.782	25.900	18.206	14.759	10.372
21	0.1315	20.429	29.445	13.309	16.291	21.118
21	0.1325	23.577	25.260	18.716	17.842	3.447
21	0.1335	27.649	23.788	18.077	15.372	11.905
21	0.1345	14.761	12.206	16.220	4.299	6.344
21	0.1355	27.149	23.989	22.153	3.005	6.742
21	0.1365	39.167	28.470	24.493	15.033	26.026
21	0.1375	24.712	21.844	19.474	9.925	4.446
21	0.1385	40.688	21.295	37.125	12.593	4.241
21	0.1395	29.718	13.431	27.298	9.533	3.479
21	0.1405	21.468	19.135	20.942	12.490	10.500
21	0.1415	20.200	19.391	15.916	15.649	12.431
21	0.1425	11.647	16.805	13.714	12.525	14.977
21	0.1435	17.378	15.453	16.380	9.337	13.565
21	0.1445	21.157	22.675	23.040	17.969	10.386
21	0.1455	29.415	26.565	29.799	21.267	16.872
21	0.1465	22.756	20.527	26.235	21.563	17.885
21	0.1475	22.758	20.524	26.214	21.572	17.867
21	0.1485	16.265	21.717	15.098	14.849	13.672
21	0.1495	11.856	16.611	19.957	22.041	12.379
21	0.1505	33.075	25.237	32.272	14.800	12.246
21	0.1515	23.126	24.581	24.223	15.073	8.949
21	0.1525	26.892	15.710	23.853	13.628	10.265
21	0.1535	24.896	20.643	23.281	16.839	10.858
21	0.1545	25.589	15.272	28.898	12.668	15.186
21	0.1555	27.753	16.651	32.602	17.479	16.585
21	0.1565	26.103	23.437	27.058	5.209	9.821
21	0.1575	26.515	22.237	22.385	9.191	13.554
21	0.1585	21.516	22.683	21.944	10.129	6.086
21	0.1595	16.472	15.910	16.115	10.927	10.195
21	0.1605	16.173	14.731	15.299	5.463	18.211
21	0.1615	24.172	16.691	19.931	13.579	22.288

21	0.1625	22.912	22.216	15.685	18.318	18.570
21	0.1635	27.462	23.288	23.638	13.622	15.353
21	0.1645	17.487	22.483	18.885	11.180	18.851
21	0.1655	19.530	16.825	18.403	7.454	11.532
21	0.1665	20.294	17.523	17.459	9.082	14.497
21	0.1675	19.100	16.519	22.431	8.367	12.840
21	0.1685	13.528	17.699	19.173	14.569	7.761
21	0.1695	11.894	16.624	15.471	16.182	9.092
21	0.1705	23.433	19.648	18.788	8.241	12.875
21	0.1715	24.799	19.056	18.290	11.358	14.622
21	0.1725	13.426	16.823	16.156	16.200	24.367
21	0.1735	19.756	22.315	20.986	17.639	28.173
21	0.1745	12.415	17.918	17.804	15.764	14.726
21	0.1755	23.043	18.301	22.805	9.811	17.276
21	0.1765	15.365	16.793	14.559	11.288	15.117
21	0.1775	15.599	13.900	15.776	17.597	6.360
21	0.1785	11.976	11.787	12.749	12.514	6.237
21	0.1795	19.522	15.237	14.909	16.192	20.889
21	0.1805	20.746	19.694	21.396	15.953	19.148
21	0.1815	12.672	18.359	16.964	10.572	9.648
21	0.1825	23.653	27.875	22.427	18.676	8.370
21	0.1835	16.285	16.464	17.297	12.033	4.803
21	0.1845	15.284	20.526	17.757	5.992	18.671
21	0.1855	22.635	19.903	23.068	9.370	11.505
21	0.1865	12.542	15.237	11.649	17.900	12.072
21	0.1875	8.095	17.073	7.540	19.136	6.607
21	0.1885	11.806	10.755	9.719	18.687	19.566
21	0.1895	9.961	7.967	6.944	9.619	9.345
21	0.1905	9.313	15.314	13.249	15.735	11.928
21	0.1915	22.330	22.053	23.945	12.859	7.511
21	0.1925	24.605	22.476	23.278	14.279	8.384
21	0.1935	18.182	17.299	23.580	12.749	6.310
21	0.1945	18.172	17.241	23.581	12.757	6.294
21	0.1955	16.551	18.314	17.839	16.412	6.715
21	0.1965	17.027	20.659	20.008	16.170	12.497
21	0.1975	16.848	13.463	15.218	10.922	16.282
21	0.1985	20.454	18.689	22.071	7.699	16.841
21	0.1995	18.857	16.371	19.027	13.493	20.974
21	0.2005	17.198	17.328	19.813	17.519	15.899
21	0.2015	17.639	21.852	26.031	10.667	14.404
21	0.2025	19.073	23.293	26.628	7.107	12.453
21	0.2035	14.030	11.392	18.812	9.806	13.198
21	0.2045	4.498	9.172	8.361	13.238	11.333
21	0.2055	7.716	14.959	9.910	17.969	10.024
21	0.2065	17.211	13.658	11.199	9.959	9.392
21	0.2075	9.743	4.635	5.708	13.590	11.760
21	0.2085	13.601	9.491	13.793	8.937	9.884
21	0.2095	18.652	15.709	18.878	9.312	4.639
21	0.2105	12.948	15.006	16.381	8.115	13.411
21	0.2115	14.159	16.753	15.292	11.645	17.116

21	0.2125	24.028	16.569	17.131	15.586	16.620
21	0.2135	19.193	15.759	12.577	14.554	9.541
21	0.2145	10.895	5.292	9.271	11.108	10.941
21	0.2155	15.441	8.371	10.583	10.016	14.747
21	0.2165	14.412	10.633	12.547	9.289	11.819
21	0.2175	8.749	11.898	8.571	9.740	17.679
21	0.2185	6.215	5.526	5.886	9.065	9.809
21	0.2195	10.490	9.524	11.312	11.983	10.462
21	0.2205	12.886	10.464	12.216	13.712	14.509
21	0.2215	12.420	9.438	9.622	12.713	13.425
21	0.2225	20.713	13.424	19.219	15.712	16.622
21	0.2235	21.604	18.693	19.946	13.035	13.798
21	0.2245	14.884	12.613	11.325	12.154	16.359
21	0.2255	30.501	16.330	18.907	9.973	14.022
21	0.2265	21.821	8.897	13.401	5.374	6.217
21	0.2275	5.132	1.270	3.921	3.545	1.764
21	0.2285	7.546	5.400	5.771	6.605	2.590
21	0.2295	13.767	4.606	9.129	11.933	15.056
21	0.2305	6.737	7.427	7.671	2.879	9.091
21	0.2315	11.614	12.936	13.976	8.529	15.041
21	0.2325	6.884	9.411	9.738	11.060	8.167
21	0.2335	15.787	15.598	18.934	15.360	13.878
21	0.2345	23.249	17.941	16.963	19.128	14.945
21	0.2355	11.950	10.896	6.568	20.943	18.314
21	0.2365	11.945	10.974	6.609	21.085	18.049
21	0.2375	11.941	11.055	6.650	21.225	17.780
21	0.2385	11.348	13.308	8.979	23.077	23.314
21	0.2395	8.523	11.455	8.945	16.346	5.425
21	0.2405	19.711	13.654	12.158	18.444	21.532
21	0.2415	16.196	18.405	18.596	12.746	11.278
21	0.2425	11.780	7.800	10.792	3.579	15.689
21	0.2435	8.037	14.897	9.985	13.676	15.645
21	0.2445	8.879	6.122	7.242	13.445	14.766
21	0.2455	11.357	7.029	8.107	14.199	20.468
21	0.2465	8.699	6.484	8.500	10.355	20.993
21	0.2475	3.116	1.856	2.694	3.798	2.262
21	0.2485	11.268	14.977	13.270	4.846	14.407
21	0.2495	7.028	6.614	5.827	15.766	14.093
21	0.2505	9.397	7.093	7.189	20.605	14.015
21	0.2515	7.958	4.627	6.109	19.784	18.435
21	0.2525	7.556	5.327	7.095	10.062	7.965
21	0.2535	7.582	4.431	4.868	7.162	5.763
21	0.2545	4.928	6.536	3.328	18.633	13.430
21	0.2555	14.941	19.086	13.851	12.762	15.718
21	0.2565	13.666	17.995	11.848	8.755	18.348
21	0.2575	16.312	20.016	11.816	22.831	18.590
21	0.2585	16.979	25.487	18.367	18.097	7.876
21	0.2595	21.934	20.390	17.391	10.105	11.444
21	0.2605	10.783	11.843	11.977	15.403	8.731
21	0.2615	9.901	11.211	10.403	14.249	5.582

21	0.2625	9.436	12.671	10.339	10.561	4.214
21	0.2635	8.629	16.278	8.778	8.188	1.234
21	0.2645	13.666	13.916	9.653	18.713	18.543
21	0.2655	11.715	12.291	6.974	20.509	13.846
21	0.2665	10.451	6.329	7.627	22.601	20.673
21	0.2675	12.723	17.635	17.675	14.166	16.412
21	0.2685	10.471	13.405	10.958	11.326	9.822
21	0.2695	15.748	18.947	12.496	18.735	19.901
21	0.2705	12.092	12.493	9.316	16.396	17.648
21	0.2715	12.225	13.370	7.959	8.549	15.030
21	0.2725	8.856	10.805	9.957	9.824	16.243
21	0.2735	17.032	10.382	11.148	11.495	26.892
21	0.2745	16.635	14.364	19.424	16.156	20.000
21	0.2755	16.534	18.652	15.359	6.837	12.001
21	0.2765	11.329	15.264	14.796	9.877	11.861
21	0.2775	4.737	10.964	4.380	8.838	10.419
21	0.2785	10.357	7.545	7.548	5.690	18.804
21	0.2795	2.921	4.085	5.161	8.180	9.587
21	0.2805	11.255	13.950	13.659	11.074	15.858
21	0.2815	19.751	13.031	9.994	18.382	30.158
21	0.2825	7.496	5.242	2.556	12.021	27.348
21	0.2835	34.132	32.139	29.769	14.025	30.938
21	0.2845	23.731	22.752	21.040	14.153	17.763
21	0.2855	15.584	6.736	12.920	9.630	10.047
21	0.2865	4.562	4.198	2.310	9.571	17.342
21	0.2875	3.679	5.221	6.505	11.611	14.573
21	0.2885	10.957	14.124	12.344	14.495	7.871
21	0.2895	9.706	6.523	4.615	15.637	10.594
21	0.2905	9.420	13.002	11.654	8.896	11.587
21	0.2915	9.420	13.025	11.656	8.975	11.592
21	0.2925	12.761	5.505	7.726	11.665	24.629
21	0.2935	6.629	7.562	6.438	5.873	11.608
21	0.2945	9.912	7.663	9.485	3.188	5.732
21	0.2955	7.794	4.324	6.645	6.619	6.759
21	0.2965	8.645	4.640	6.561	6.559	5.912
21	0.2975	5.663	2.378	2.506	5.300	7.289
21	0.2985	10.336	7.092	10.489	11.453	13.236
21	0.2995	11.603	11.855	12.599	12.413	5.987
21	0.3005	14.009	12.769	16.223	14.369	16.930
21	0.3015	13.773	10.746	10.960	8.937	11.046
21	0.3025	7.175	4.576	9.440	5.757	19.743
21	0.3035	5.259	2.971	5.446	4.533	19.680
21	0.3045	3.041	1.943	2.721	3.432	12.468
21	0.3055	9.903	5.902	7.005	6.530	5.315
21	0.3065	14.446	6.739	11.907	5.537	5.709
21	0.3075	26.080	12.291	22.107	13.437	12.960
21	0.3085	5.533	4.366	6.215	20.350	24.532
21	0.3095	2.152	10.298	3.222	18.431	12.340
21	0.3105	8.713	6.537	4.629	9.007	10.846
21	0.3115	15.429	16.028	15.030	23.673	19.898

21	0.3125	15.627	21.530	19.372	23.998	22.716
21	0.3135	8.025	9.394	12.588	10.139	15.353
21	0.3145	16.402	18.227	19.631	18.657	23.113
21	0.3155	10.428	13.859	14.017	15.448	21.555
21	0.3165	17.153	17.265	18.198	5.279	10.762
21	0.3175	15.649	16.645	17.459	6.709	19.279
21	0.3185	31.195	26.218	28.330	12.122	19.083
21	0.3195	17.263	23.280	22.290	7.806	12.558
21	0.3205	12.481	17.231	13.476	7.201	21.679
21	0.3215	12.733	18.017	12.652	10.861	26.726
21	0.3225	13.510	13.929	13.640	12.488	15.301
21	0.3235	15.967	16.638	14.799	11.526	15.953
21	0.3245	21.071	20.536	20.956	18.319	24.791
21	0.3255	29.996	18.864	25.828	15.707	22.543
21	0.3265	20.018	12.345	17.917	5.601	9.739
21	0.3275	29.587	21.339	26.836	4.500	8.465
21	0.3285	9.539	15.684	10.156	11.669	11.301
21	0.3295	9.801	13.282	8.769	19.407	18.154
21	0.3305	14.618	13.806	11.226	20.068	28.382
21	0.3315	12.011	15.050	14.005	14.952	29.729
21	0.3325	18.072	13.427	12.957	16.868	37.972
21	0.3335	11.272	21.737	14.652	13.064	18.979
21	0.3345	19.546	18.147	14.794	13.124	28.074
21	0.3355	14.082	13.329	10.951	7.564	11.565
21	0.3365	10.941	11.273	11.836	8.244	21.439
21	0.3375	21.089	17.198	14.273	12.985	24.738
21	0.3385	10.663	12.172	10.700	11.775	21.305
21	0.3395	19.347	14.829	19.856	13.315	17.168
21	0.3405	8.820	8.160	9.741	14.829	12.665
21	0.3415	14.720	15.085	12.000	14.854	21.124
21	0.3425	11.069	12.413	11.969	11.614	4.337
21	0.3435	5.839	2.860	4.811	11.806	33.432
21	0.3445	11.613	10.451	14.720	16.591	30.681
21	0.3455	19.376	15.675	27.167	16.595	33.715
21	0.3465	10.995	12.023	15.920	19.018	27.275
21	0.3475	14.493	11.977	17.463	16.876	18.952
21	0.3485	10.680	10.419	12.349	14.071	14.858
21	0.3495	12.308	8.749	12.867	7.259	11.864
21	0.3505	11.632	7.057	10.728	12.007	11.350
21	0.3515	12.562	7.305	8.203	15.265	7.436
21	0.3525	10.052	13.819	6.482	21.889	20.921
21	0.3535	7.793	8.924	7.536	9.346	10.223
21	0.3545	14.829	11.233	12.325	12.516	12.686
21	0.3555	12.638	7.462	7.747	22.477	24.360
21	0.3565	16.166	9.308	15.010	14.976	21.690
21	0.3575	12.573	9.056	8.554	9.207	13.421
21	0.3585	20.679	15.768	16.784	11.826	11.228
21	0.3595	15.021	8.010	12.677	28.988	27.248
21	0.3605	5.324	10.446	11.397	16.191	21.509
21	0.3615	13.967	17.935	14.842	31.250	39.833

21	0.3625	15.877	18.098	18.754	36.603	34.173
21	0.3635	11.420	5.710	8.587	18.575	31.031
21	0.3645	20.884	10.373	10.710	8.410	34.518
21	0.3655	21.707	17.027	11.891	22.238	34.891
21	0.3665	12.551	9.326	5.796	23.548	12.909
21	0.3675	12.592	16.076	14.603	28.897	31.036
21	0.3685	16.769	18.216	15.605	19.050	21.339
21	0.3695	13.478	13.881	13.400	24.442	33.154
21	0.3705	16.675	15.569	17.484	31.704	32.156
21	0.3715	12.348	14.632	13.571	13.486	31.029
21	0.3725	9.351	3.717	7.799	10.967	24.042
21	0.3735	9.236	3.610	7.733	10.883	23.859
21	0.3745	13.033	6.028	8.759	14.880	15.628
21	0.3755	15.530	13.221	13.573	22.246	21.600
21	0.3765	14.548	13.714	13.798	27.592	23.742
21	0.3775	16.697	13.449	17.054	29.505	18.002
21	0.3785	16.563	18.527	19.090	29.942	14.813
21	0.3795	15.706	16.129	20.375	28.867	25.308
21	0.3805	12.981	15.589	16.728	22.270	24.248
21	0.3815	14.358	13.959	14.083	28.801	26.908
21	0.3825	10.016	8.033	9.926	20.941	27.331
21	0.3835	13.074	9.573	14.888	13.196	13.369
21	0.3845	11.714	16.638	17.308	15.427	12.649
21	0.3855	36.124	24.273	23.329	13.117	29.154
21	0.3865	23.402	18.853	20.647	12.835	29.109
21	0.3875	23.254	16.303	17.549	21.022	18.410
21	0.3885	7.403	9.422	6.859	30.596	24.276
21	0.3895	7.597	10.412	9.398	7.747	9.832
21	0.3905	13.178	22.518	18.033	23.084	19.873
21	0.3915	10.069	17.716	15.427	15.688	23.737
21	0.3925	8.913	9.424	7.057	15.659	18.080
21	0.3935	19.018	19.477	20.550	22.228	21.422
21	0.3945	7.175	16.635	13.653	20.830	30.245
21	0.3955	13.870	16.394	17.979	32.357	28.674
21	0.3965	7.928	6.379	11.002	17.412	23.413
21	0.3975	10.746	7.520	7.452	25.964	29.839
21	0.3985	10.933	9.876	12.644	7.768	13.479
21	0.3995	29.208	22.813	24.073	16.983	20.958
21	0.4005	16.110	15.530	14.350	23.942	15.725
21	0.4015	13.200	14.464	10.589	27.609	31.051
21	0.4025	9.829	9.837	15.466	19.420	26.321
21	0.4035	5.863	9.332	5.284	28.782	19.920
21	0.4045	14.619	12.975	13.787	29.166	29.131
21	0.4055	21.114	24.727	19.758	16.707	11.494
21	0.4065	6.987	4.154	6.212	2.706	2.789
21	0.4075	12.251	9.688	10.751	18.554	23.489
21	0.4085	12.392	7.373	11.691	12.978	19.725
21	0.4095	12.774	15.557	13.452	19.471	18.210
21	0.4105	17.416	13.905	20.514	17.963	16.865
21	0.4115	17.969	19.828	22.499	9.582	12.595

21	0.4125	12.282	11.190	17.259	14.622	11.199
21	0.4135	16.166	10.701	17.019	20.084	21.195
21	0.4145	15.992	16.684	18.763	16.815	19.286
21	0.4155	23.171	18.390	17.612	18.269	22.920
21	0.4165	28.910	15.529	21.338	21.991	25.259
21	0.4175	30.860	25.209	27.109	14.073	32.192
21	0.4185	14.059	18.535	16.523	15.768	25.724
21	0.4195	25.995	24.042	20.184	23.567	24.779
21	0.4205	20.432	20.043	21.041	25.112	22.594
21	0.4215	18.920	10.689	16.209	16.882	17.317
21	0.4225	10.743	12.660	13.247	15.546	14.415
21	0.4235	10.692	12.462	13.064	15.381	14.297
21	0.4245	14.056	15.705	7.948	15.645	19.546
21	0.4255	15.062	10.887	12.273	5.732	12.681
21	0.4265	10.277	10.103	9.901	5.837	13.242
21	0.4275	17.183	19.068	17.302	13.576	17.703
21	0.4285	16.699	14.672	18.399	10.151	19.176
21	0.4295	9.943	6.101	7.184	4.211	8.619
21	0.4305	10.639	8.205	13.249	10.069	14.262
21	0.4315	18.103	18.636	19.085	15.593	29.262
21	0.4325	8.735	10.873	9.954	23.779	28.415
21	0.4335	14.476	15.744	14.400	19.212	17.891
21	0.4345	16.387	16.446	17.328	16.384	21.498
21	0.4355	3.959	8.647	4.643	21.113	13.424
21	0.4365	11.520	12.641	12.176	14.168	14.462
21	0.4375	13.978	14.715	14.165	17.539	17.873
21	0.4385	11.811	14.819	7.355	15.425	19.074
21	0.4395	23.599	16.399	18.280	16.010	28.119
21	0.4405	23.816	17.822	20.489	12.758	25.632
21	0.4415	9.010	11.046	9.286	18.476	18.176
21	0.4425	10.467	13.398	14.537	20.490	25.548
21	0.4435	4.313	5.804	4.434	12.827	22.830
21	0.4445	18.188	11.210	12.990	12.353	18.560
21	0.4455	17.423	12.378	17.707	15.516	20.857
21	0.4465	14.934	15.060	12.637	6.395	15.880
21	0.4475	8.785	11.333	9.468	4.054	3.952
21	0.4485	6.384	8.767	8.076	3.864	9.921
21	0.4495	7.362	10.562	9.142	8.871	9.326
21	0.4505	10.025	4.545	9.026	9.619	15.051
21	0.4515	8.309	6.939	7.818	9.153	13.287
21	0.4525	10.543	7.980	8.800	14.170	14.966
21	0.4535	12.772	8.416	10.620	13.136	15.336
21	0.4545	6.138	12.054	8.119	16.038	20.614
21	0.4555	4.875	6.866	4.491	12.405	22.339
21	0.4565	6.757	9.450	6.730	15.376	23.530
21	0.4575	6.835	11.150	10.427	15.018	25.385
21	0.4585	12.286	13.883	17.394	16.454	23.871
21	0.4595	12.549	13.309	15.260	13.369	15.915
21	0.4605	11.833	14.627	15.424	11.309	14.836
21	0.4615	11.140	15.194	12.927	12.377	18.894

21	0.4625	8.156	13.943	10.274	16.377	19.140
21	0.4635	10.297	10.300	11.046	13.933	9.136
21	0.4645	15.854	11.846	15.938	11.905	12.154
21	0.4655	18.151	17.378	17.816	16.572	15.251
21	0.4665	6.541	9.078	9.921	7.645	14.681
21	0.4675	5.024	6.952	6.727	7.469	7.960
21	0.4685	13.379	13.456	14.849	13.218	8.716
21	0.4695	9.613	15.775	17.259	17.141	19.870
21	0.4705	11.187	6.694	8.203	16.956	24.287
21	0.4715	12.313	7.987	12.254	15.119	20.606
21	0.4725	11.902	9.945	11.812	18.429	21.610
21	0.4735	14.175	16.661	17.544	18.517	14.960
21	0.4745	15.264	15.455	15.198	8.690	11.227
21	0.4755	16.434	16.543	16.809	8.878	12.557
21	0.4765	15.200	14.704	15.067	8.200	4.992
21	0.4775	9.911	11.469	13.843	7.422	6.862
21	0.4785	12.036	13.758	12.636	15.013	19.286
21	0.4795	13.758	14.833	16.797	13.068	12.304
21	0.4805	12.304	18.033	19.165	9.062	8.753
21	0.4815	6.844	9.977	13.577	14.380	11.003
21	0.4825	7.975	8.386	11.958	11.777	7.869
21	0.4835	8.174	10.987	10.318	14.681	11.560
21	0.4845	20.494	19.755	23.665	11.758	13.001
21	0.4855	15.580	14.325	25.373	10.905	8.747
21	0.4865	15.553	14.284	25.350	11.019	9.044
21	0.4875	14.944	12.471	11.927	12.929	15.078
21	0.4885	13.507	8.171	15.748	18.040	38.820
21	0.4895	10.336	13.592	12.760	20.671	9.212
21	0.4905	20.637	15.200	18.433	18.169	28.526
21	0.4915	15.044	13.062	18.133	17.791	23.445
21	0.4925	15.373	12.203	17.894	9.007	14.288
21	0.4935	7.557	5.443	7.595	10.207	13.566
21	0.4945	14.930	8.155	17.691	11.858	11.640
21	0.4955	6.194	2.823	9.838	16.144	16.414
21	0.4965	13.552	12.494	12.682	7.329	18.298
21	0.4975	9.030	8.042	10.028	20.762	18.629
21	0.4985	7.861	6.331	12.406	18.375	16.844
21	0.4995	7.243	8.844	9.529	9.973	5.213
21	0.5005	14.355	7.107	15.257	8.674	16.672
21	0.5015	11.856	8.782	13.233	11.611	18.500
21	0.5025	16.831	9.371	18.742	11.977	19.150
21	0.5035	18.150	8.811	20.750	13.666	9.118
21	0.5045	16.554	7.356	15.443	20.363	24.213
21	0.5055	6.616	3.936	10.977	8.659	16.002
21	0.5065	10.500	4.080	12.873	24.047	36.298
21	0.5075	15.456	10.188	17.198	9.806	8.925
21	0.5085	16.701	8.119	13.946	20.016	23.699
21	0.5095	14.808	7.710	15.388	19.319	31.433
21	0.5105	16.479	13.854	11.735	16.232	30.331
21	0.5115	12.545	5.767	11.458	12.738	11.477

21	0.5125	17.576	7.402	18.441	20.539	6.909
21	0.5135	18.321	12.335	18.417	14.098	5.769
21	0.5145	11.403	10.087	8.938	8.213	9.337
21	0.5155	10.618	5.761	10.898	12.149	11.988
21	0.5165	16.492	9.664	14.305	22.629	23.459
21	0.5175	11.504	9.328	12.004	17.914	9.643
21	0.5185	9.143	11.329	6.253	12.155	7.469
21	0.5195	15.767	21.714	17.468	10.492	17.529
21	0.5205	13.567	8.498	19.082	10.385	9.127
21	0.5215	6.141	4.396	7.509	6.490	6.210
21	0.5225	7.189	4.075	6.008	12.154	9.840
21	0.5235	16.969	10.912	13.835	13.483	8.208
21	0.5245	8.660	11.152	14.151	16.618	12.709
21	0.5255	3.863	8.657	8.634	10.715	7.028
21	0.5265	13.411	8.226	15.564	23.319	11.208
21	0.5275	15.280	13.922	13.571	13.893	10.914
21	0.5285	10.278	10.651	9.839	8.051	11.453
21	0.5295	9.139	12.660	12.818	11.680	8.043
21	0.5305	22.542	13.644	21.617	20.364	22.794
21	0.5315	11.504	6.813	12.527	18.990	9.785
21	0.5325	7.556	8.786	8.877	18.355	9.477
21	0.5335	11.063	8.941	11.435	4.256	10.090
21	0.5345	9.714	6.144	12.481	10.587	5.446
21	0.5355	16.095	10.997	14.005	25.239	13.056
21	0.5365	6.253	5.661	4.904	6.692	13.526
21	0.5375	10.330	11.076	7.343	8.175	6.589
21	0.5385	12.089	11.153	6.975	14.204	11.636
21	0.5395	9.575	6.413	6.944	17.192	9.995
21	0.5405	10.604	4.612	5.605	13.259	8.998
21	0.5415	17.644	8.581	17.388	10.344	9.918
21	0.5425	15.945	9.763	14.817	15.873	19.003
21	0.5435	21.241	12.035	21.793	18.939	14.510
21	0.5445	13.378	9.497	10.344	9.873	15.296
21	0.5455	21.055	10.871	21.621	16.213	16.531
21	0.5465	14.810	8.369	9.731	19.140	13.014
21	0.5475	16.816	13.879	13.388	19.325	16.317
21	0.5485	4.792	5.681	7.078	8.852	6.617
21	0.5495	4.724	8.063	7.371	6.464	3.429
21	0.5505	11.115	6.120	7.231	6.368	6.229
21	0.5515	11.896	7.773	11.586	9.613	9.161
21	0.5525	10.731	4.477	11.130	8.325	11.179
21	0.5535	13.104	7.509	11.772	24.079	12.111
21	0.5545	11.458	5.135	12.794	18.081	6.838
21	0.5555	9.327	5.444	13.129	16.791	6.466
21	0.5565	10.039	17.234	13.725	19.619	8.493
21	0.5575	7.109	11.018	7.674	11.969	8.393
21	0.5585	16.444	15.713	18.929	20.920	19.529
21	0.5595	16.773	10.449	14.191	13.458	15.624
21	0.5605	10.700	7.082	10.634	8.191	4.590
21	0.5615	15.327	15.678	14.897	16.085	16.768

21	0.5625	11.528	10.513	10.397	9.653	15.143
21	0.5635	8.930	5.896	7.141	12.918	13.561
21	0.5645	12.845	9.089	9.741	16.149	25.179
21	0.5655	10.732	6.310	8.641	16.652	22.034
21	0.5665	7.579	9.802	11.122	15.264	10.552
21	0.5675	13.014	5.531	14.191	13.770	15.917
21	0.5685	14.388	7.216	12.472	12.340	17.659
21	0.5695	13.399	8.632	12.430	17.816	26.135
21	0.5705	18.261	11.268	12.835	17.168	23.112
21	0.5715	13.047	11.456	11.901	15.923	18.674
21	0.5725	16.709	23.448	15.467	12.230	16.826
21	0.5735	15.830	22.037	17.685	12.868	14.661
21	0.5745	15.729	22.128	19.259	10.195	12.607
21	0.5755	14.706	10.881	9.672	7.677	16.675
21	0.5765	14.619	12.822	8.739	5.853	11.690
21	0.5775	10.359	12.873	6.407	7.412	10.735
21	0.5785	13.696	12.775	9.727	12.334	8.557
21	0.5795	25.466	16.814	14.954	12.126	22.948
21	0.5805	13.231	10.671	10.500	10.378	16.561
21	0.5815	6.839	8.748	3.808	7.615	14.826
21	0.5825	14.767	17.211	11.695	8.790	21.913
21	0.5835	11.509	15.897	12.291	8.171	16.065
21	0.5845	15.940	16.683	12.356	19.196	20.005
21	0.5855	28.731	30.998	29.990	11.141	12.818
21	0.5865	15.344	17.961	13.771	12.366	18.821
21	0.5875	16.242	16.523	13.357	15.030	18.221
21	0.5885	13.447	13.655	22.886	14.783	30.971
21	0.5895	12.786	8.968	11.273	19.742	17.354
21	0.5905	15.242	17.041	16.084	13.113	22.792
21	0.5915	2.780	5.520	4.897	23.274	21.521
21	0.5925	20.064	21.420	16.540	10.069	11.501
21	0.5935	12.320	15.574	9.884	16.842	15.418
21	0.5945	13.852	17.772	9.750	11.828	18.099
21	0.5955	9.024	12.719	8.127	11.857	5.520
21	0.5965	19.228	24.832	21.245	10.301	16.613
21	0.5975	11.099	15.650	12.977	18.448	21.320
21	0.5985	21.816	25.316	17.767	13.399	14.470
21	0.5995	19.424	13.937	16.105	21.086	17.955
21	0.6005	9.756	13.211	10.543	15.978	11.807
21	0.6015	7.927	13.345	9.076	13.703	11.512
21	0.6025	15.622	22.110	20.903	27.959	12.902
21	0.6035	10.933	13.125	10.742	18.363	9.435
21	0.6045	11.234	12.280	12.401	18.049	17.706
21	0.6055	10.010	20.574	18.061	13.929	18.330
21	0.6065	12.509	22.141	16.092	24.324	21.715
21	0.6075	12.756	21.794	11.907	22.598	17.418
21	0.6085	6.879	18.097	9.085	22.859	15.065
21	0.6095	8.138	21.817	12.390	17.223	23.531
21	0.6105	14.557	12.986	13.291	12.720	15.840
21	0.6115	13.194	24.140	9.598	24.476	12.869

21	0.6125	11.323	16.256	14.287	18.952	13.444
21	0.6135	9.142	10.813	11.533	10.625	17.828
21	0.6145	5.001	5.944	5.504	6.138	13.337
21	0.6155	9.904	19.779	15.057	16.933	12.782
21	0.6165	11.240	22.342	12.901	17.141	10.931
21	0.6175	9.353	13.699	10.582	17.365	24.105
21	0.6185	9.135	10.359	12.073	17.230	22.770
21	0.6195	0.977	4.622	1.954	5.268	3.206
21	0.6205	7.806	9.289	8.425	14.889	12.766
21	0.6215	10.765	13.095	10.447	14.246	13.579
21	0.6225	11.232	10.377	5.381	23.230	13.798
21	0.6235	9.672	14.712	12.590	23.755	9.440
21	0.6245	18.341	23.581	21.856	22.686	16.654
21	0.6255	11.073	8.464	9.504	15.905	16.324
21	0.6265	9.809	13.938	9.920	20.982	5.221
21	0.6275	11.531	14.925	13.368	12.204	11.160
21	0.6285	4.883	11.580	6.299	11.010	6.716
21	0.6295	12.795	13.110	10.023	14.311	12.661
21	0.6305	19.327	15.725	14.474	11.625	11.227
21	0.6315	14.911	11.441	14.467	10.568	13.392
21	0.6325	17.134	14.304	10.649	6.734	16.903
21	0.6335	11.793	17.011	10.781	12.984	12.556
21	0.6345	10.400	11.778	10.515	14.468	11.242
21	0.6355	19.920	16.773	15.440	12.994	15.349
21	0.6365	12.847	12.434	14.329	9.066	5.922
21	0.6375	23.524	11.892	9.917	10.868	25.705
21	0.6385	21.138	13.153	14.339	12.267	19.342
21	0.6395	25.956	16.831	16.963	14.361	17.276
21	0.6405	7.366	6.948	5.365	9.281	14.630
21	0.6415	7.117	7.771	5.553	12.628	15.571
21	0.6425	10.794	13.773	9.469	5.338	6.152
21	0.6435	20.765	18.613	14.470	8.602	13.634
21	0.6445	19.926	18.462	14.078	11.581	19.144
21	0.6455	6.665	8.703	4.825	9.709	14.228
21	0.6465	20.822	15.512	13.114	15.655	32.056
21	0.6475	20.705	22.169	15.562	12.806	13.045
21	0.6485	4.850	4.405	3.436	9.180	13.065
21	0.6495	16.116	7.313	9.745	5.888	6.315
21	0.6505	16.353	6.104	9.418	5.444	9.403
21	0.6515	16.076	6.043	9.441	5.259	8.811
21	0.6525	3.133	3.293	5.550	0.834	1.235
21	0.6535	11.578	10.436	10.932	6.333	4.988
21	0.6545	15.587	14.050	10.887	12.579	13.308
21	0.6555	15.649	14.118	10.969	12.590	13.308
21	0.6565	14.437	14.751	13.511	11.714	10.979
21	0.6575	19.733	25.257	20.555	11.650	17.751
21	0.6585	22.151	24.694	18.008	17.943	22.890
21	0.6595	16.066	16.268	12.812	12.293	19.304
21	0.6605	11.645	6.259	8.746	10.024	21.751
21	0.6615	9.661	5.962	7.166	13.065	23.392

21	0.6625	15.679	16.949	20.084	14.373	12.080
21	0.6635	15.721	14.165	14.148	11.420	19.949
21	0.6645	12.413	14.502	13.973	6.793	10.213
21	0.6655	16.483	11.630	13.217	6.669	7.943
21	0.6665	10.827	13.147	7.810	11.134	19.616
21	0.6675	0.740	0.008	0.692	2.853	0.653
22	0.0007	0.000	0.000	0.000	0.000	0.000
22	0.0017	3.543	4.634	4.600	9.375	25.810
22	0.0027	14.855	12.371	14.230	10.606	19.669
22	0.0037	17.059	15.732	16.995	13.472	12.619
22	0.0047	10.320	10.500	7.683	10.873	10.616
22	0.0057	10.597	10.575	7.808	10.956	10.765
22	0.0067	11.195	15.140	8.598	13.298	13.925
22	0.0077	11.648	17.554	15.854	14.437	16.488
22	0.0087	12.117	11.768	13.024	7.973	10.697
22	0.0097	11.763	11.746	10.086	7.715	17.180
22	0.0107	18.322	27.923	19.982	12.535	13.452
22	0.0117	12.746	21.468	17.679	7.374	16.797
22	0.0127	11.308	9.357	9.462	15.573	20.667
22	0.0137	19.329	20.368	17.682	7.300	17.579
22	0.0147	11.441	13.384	13.883	10.242	10.499
22	0.0157	4.622	8.323	5.699	17.786	13.823
22	0.0167	4.072	3.151	2.305	4.858	13.663
22	0.0177	5.857	5.339	3.873	10.628	16.575
22	0.0187	7.462	6.516	7.320	7.124	12.866
22	0.0197	9.851	19.339	10.347	14.971	15.987
22	0.0207	8.585	6.234	3.286	10.579	20.012
22	0.0217	4.864	11.295	6.646	10.614	18.430
22	0.0227	17.407	16.270	14.879	12.732	19.018
22	0.0237	9.473	15.971	13.427	13.506	19.514
22	0.0247	7.791	4.842	6.593	9.067	16.599
22	0.0257	16.974	10.023	14.231	13.171	24.094
22	0.0267	14.778	6.844	13.433	5.986	14.221
22	0.0277	4.288	4.319	8.122	12.959	15.549
22	0.0287	16.307	17.531	16.231	14.078	19.109
22	0.0297	11.597	7.180	12.164	10.642	16.185
22	0.0307	9.793	8.088	12.190	3.451	13.412
22	0.0317	12.088	8.443	10.248	13.787	18.074
22	0.0327	21.942	13.687	18.510	18.917	31.138
22	0.0337	11.202	8.084	11.038	15.609	21.928
22	0.0347	11.021	10.516	10.611	9.236	16.108
22	0.0357	10.871	13.205	15.489	11.698	25.312
22	0.0367	5.435	7.573	3.090	11.737	18.977
22	0.0377	4.100	6.588	2.821	12.827	20.780
22	0.0387	6.596	7.528	6.208	10.593	20.252
22	0.0397	10.849	11.873	7.099	16.812	20.693
22	0.0407	17.600	7.804	15.085	11.617	22.481
22	0.0417	11.799	6.749	10.515	13.292	24.313
22	0.0427	11.059	10.040	11.900	8.474	25.644
22	0.0437	12.553	11.125	11.703	9.522	19.458

22	0.0447	11.193	11.972	15.074	6.924	5.471
22	0.0457	12.201	13.712	13.666	15.649	17.733
22	0.0467	15.199	12.912	20.608	13.056	11.928
22	0.0477	24.944	13.299	19.341	9.469	24.936
22	0.0487	14.429	9.405	13.885	14.089	15.733
22	0.0497	7.957	8.661	8.735	16.201	21.981
22	0.0507	15.108	7.680	11.238	8.448	7.319
22	0.0517	16.897	12.847	13.669	25.302	13.015
22	0.0527	16.628	11.725	13.052	15.350	24.131
22	0.0537	15.766	12.461	11.671	15.685	17.615
22	0.0547	27.030	19.323	22.876	16.018	20.375
22	0.0557	26.808	19.271	22.691	15.401	20.220
22	0.0567	12.966	8.466	13.245	8.785	24.307
22	0.0577	14.979	14.161	11.512	17.814	21.907
22	0.0587	18.774	19.438	23.967	19.847	24.344
22	0.0597	7.883	6.953	8.733	11.183	14.956
22	0.0607	11.657	14.489	14.061	12.636	9.400
22	0.0617	16.980	16.113	19.365	16.072	21.212
22	0.0627	17.536	14.820	15.288	7.801	11.645
22	0.0637	12.137	13.054	11.873	6.928	11.169
22	0.0647	8.397	6.720	8.845	12.201	10.775
22	0.0657	17.061	14.881	17.253	16.799	18.184
22	0.0667	4.674	9.204	9.976	13.806	15.010
22	0.0677	9.051	15.203	15.267	6.669	10.197
22	0.0687	10.518	9.336	11.239	7.487	9.783
22	0.0697	10.648	6.795	8.790	20.505	18.941
22	0.0707	10.183	10.786	6.599	30.477	15.696
22	0.0717	6.663	11.080	9.977	10.949	19.376
22	0.0727	9.546	11.403	12.896	16.157	24.622
22	0.0737	15.195	9.519	10.429	7.340	20.167
22	0.0747	8.997	9.467	8.466	18.664	17.304
22	0.0757	8.721	13.749	9.651	13.160	15.864
22	0.0767	5.021	6.359	5.605	7.952	6.724
22	0.0777	7.568	5.669	8.802	9.737	11.132
22	0.0787	8.420	7.320	10.824	10.963	19.720
22	0.0797	9.313	5.833	8.454	15.469	14.825
22	0.0807	4.333	6.729	4.854	14.503	10.335
22	0.0817	11.879	7.468	10.116	12.482	19.911
22	0.0827	11.859	7.551	10.125	12.444	19.802
22	0.0837	12.229	19.736	12.142	20.743	17.000
22	0.0847	10.495	22.740	11.985	25.592	18.990
22	0.0857	10.509	9.179	7.034	18.997	14.003
22	0.0867	5.483	5.889	5.107	15.016	11.149
22	0.0877	8.226	6.332	6.697	18.475	22.798
22	0.0887	5.801	6.624	6.157	24.305	12.921
22	0.0897	8.873	11.107	12.560	20.180	16.236
22	0.0907	5.657	9.318	5.420	15.675	8.547
22	0.0917	6.540	7.663	7.864	7.421	6.355
22	0.0927	6.757	6.411	8.633	16.385	14.088
22	0.0937	11.225	5.882	6.634	11.478	9.730

22	0.0947	15.222	5.330	13.656	12.156	4.917
22	0.0957	9.380	13.566	9.625	21.791	10.584
22	0.0967	6.016	8.137	6.242	16.721	15.156
22	0.0977	12.642	9.226	5.986	14.599	17.127
22	0.0987	4.862	10.539	4.552	20.670	10.773
22	0.0997	8.150	10.311	9.903	18.902	8.486
22	0.1007	8.938	8.586	11.745	20.597	18.102
22	0.1017	6.463	10.024	10.465	12.366	14.071
22	0.1027	6.190	9.841	7.355	11.551	14.803
22	0.1037	16.321	15.838	18.356	28.912	9.055
22	0.1047	9.790	12.332	11.123	26.848	8.308
22	0.1057	11.673	11.810	13.123	13.041	10.016
22	0.1067	8.924	3.776	7.722	10.600	11.035
22	0.1077	8.550	5.187	5.973	22.516	15.177
22	0.1087	8.042	10.140	11.728	17.417	26.653
22	0.1097	5.537	7.517	7.695	11.588	16.725
22	0.1107	6.476	6.225	6.832	10.061	4.592
22	0.1117	6.485	4.399	4.958	11.732	6.325
22	0.1127	8.599	5.228	6.936	3.765	17.133
22	0.1137	11.151	9.379	10.764	8.927	16.827
22	0.1147	8.667	5.738	10.001	6.289	5.650
22	0.1157	9.624	8.094	12.913	12.467	21.328
22	0.1167	11.020	8.366	12.497	10.722	13.772
22	0.1177	5.036	4.102	6.073	5.997	8.341
22	0.1187	11.180	7.545	6.888	16.508	11.038
22	0.1197	13.821	14.240	12.462	20.498	20.086
22	0.1207	12.770	12.897	10.538	24.356	12.942
22	0.1217	11.666	7.993	11.684	18.765	11.950
22	0.1227	8.868	8.446	4.780	22.385	6.800
22	0.1237	5.163	11.904	4.995	31.983	13.625
22	0.1247	9.857	10.600	9.981	27.632	9.082
22	0.1257	14.746	13.186	15.322	23.591	14.170
22	0.1267	12.824	12.632	10.983	25.690	10.051
22	0.1277	15.310	17.560	12.609	30.128	8.365
22	0.1287	13.974	6.040	6.790	23.359	12.391
22	0.1297	15.794	10.582	15.999	11.894	23.293
22	0.1307	12.549	12.964	10.427	16.352	25.855
22	0.1317	8.111	9.830	13.674	7.079	4.377
22	0.1327	15.491	10.829	16.610	18.971	3.105
22	0.1337	12.401	12.059	8.580	24.341	21.466
22	0.1347	18.827	12.958	14.681	15.823	18.680
22	0.1357	17.526	12.224	18.792	11.049	14.776
22	0.1367	13.619	7.918	7.057	22.639	13.902
22	0.1377	22.537	28.256	19.553	39.823	20.463
22	0.1387	14.200	18.270	14.472	32.178	18.904
22	0.1397	13.324	15.293	13.653	22.988	12.015
22	0.1407	7.459	6.799	5.091	19.484	3.329
22	0.1417	9.157	12.362	11.421	16.492	8.743
22	0.1427	12.893	10.906	10.917	23.715	24.761
22	0.1437	12.767	11.006	10.883	23.882	24.642

22	0.1447	12.645	11.120	10.860	24.046	24.520
22	0.1457	17.257	18.683	13.660	31.288	22.143
22	0.1467	12.759	13.946	10.454	23.909	11.167
22	0.1477	9.332	9.750	8.801	21.833	20.004
22	0.1487	9.048	5.787	5.472	10.087	23.620
22	0.1497	18.729	8.278	10.131	16.979	16.825
22	0.1507	11.674	5.365	5.753	17.501	12.911
22	0.1517	8.912	6.123	9.087	7.191	8.874
22	0.1527	13.498	18.085	12.983	18.777	5.455
22	0.1537	11.110	8.134	9.732	15.364	19.726
22	0.1547	14.518	13.409	16.476	15.628	18.328
22	0.1557	18.029	15.456	16.427	23.965	8.436
22	0.1567	11.669	12.073	14.847	17.849	9.702
22	0.1577	15.385	12.319	14.161	17.743	14.387
22	0.1587	11.010	6.601	9.635	24.192	19.393
22	0.1597	7.359	6.032	5.531	21.954	8.632
22	0.1607	11.522	12.788	7.270	19.319	12.310
22	0.1617	11.905	5.324	4.835	13.725	12.720
22	0.1627	17.310	13.579	14.097	14.492	14.205
22	0.1637	23.831	16.720	18.608	16.949	14.565
22	0.1647	24.410	16.868	18.876	16.651	15.729
22	0.1657	15.814	14.528	15.573	16.011	15.740
22	0.1667	4.727	8.685	8.156	9.139	2.700
22	0.1677	15.883	5.724	11.681	22.224	11.657
22	0.1687	14.313	6.151	8.739	17.769	10.753
22	0.1697	10.458	10.196	8.534	19.957	15.015
22	0.1707	10.469	10.232	8.566	19.871	14.956
22	0.1717	12.886	10.996	9.732	20.383	12.547
22	0.1727	10.089	8.425	7.458	18.758	14.746
22	0.1737	9.645	5.333	6.838	19.937	13.177
22	0.1747	15.620	14.005	14.801	13.866	15.299
22	0.1757	9.892	4.752	9.445	17.571	16.907
22	0.1767	20.043	5.489	16.578	12.515	18.696
22	0.1777	7.303	7.960	6.911	12.288	3.132
22	0.1787	3.033	4.481	1.775	10.842	5.538
22	0.1797	9.899	8.570	8.998	26.901	17.331
22	0.1807	12.110	9.788	10.105	21.825	19.678
22	0.1817	15.336	7.672	12.475	23.303	14.435
22	0.1827	19.441	11.214	16.556	18.437	12.569
22	0.1837	12.599	8.778	11.592	20.562	21.674
22	0.1847	13.954	7.207	13.620	18.253	10.505
22	0.1857	5.738	8.091	4.867	20.354	11.068
22	0.1867	16.448	8.437	12.123	12.795	12.780
22	0.1877	4.871	5.338	3.116	10.292	9.962
22	0.1887	16.688	14.151	16.928	14.069	10.238
22	0.1897	10.069	13.856	10.638	19.950	11.579
22	0.1907	8.543	9.851	8.252	11.571	8.603
22	0.1917	10.714	13.232	9.901	17.211	19.882
22	0.1927	18.625	19.348	15.702	23.951	7.460
22	0.1937	9.403	12.931	10.024	19.820	13.439

22	0.1947	10.692	11.718	9.653	18.539	13.312
22	0.1957	18.239	15.232	19.390	14.303	23.642
22	0.1967	18.706	16.956	21.649	12.302	24.317
22	0.1977	16.660	14.202	14.900	14.081	12.401
22	0.1987	17.364	17.245	20.281	15.406	13.431
22	0.1997	15.456	10.576	13.279	12.174	7.014
22	0.2007	13.160	11.647	13.560	14.319	15.160
22	0.2017	22.078	17.540	20.269	14.797	8.315
22	0.2027	12.726	15.705	14.869	18.195	11.240
22	0.2037	12.429	8.863	12.645	10.540	6.504
22	0.2047	6.061	7.470	5.352	12.101	9.428
22	0.2057	4.307	8.570	5.587	16.239	9.279
22	0.2067	15.163	12.454	12.341	15.186	5.788
22	0.2077	14.690	17.747	21.163	13.292	8.578
22	0.2087	24.319	21.432	24.352	17.473	9.759
22	0.2097	2.176	2.829	4.679	8.477	6.686
22	0.2107	7.319	9.781	6.917	11.581	3.334
22	0.2117	9.544	18.251	15.630	20.866	9.118
22	0.2127	27.563	25.587	25.097	10.544	11.554
22	0.2137	9.114	12.470	9.520	19.675	21.479
22	0.2147	13.561	22.073	19.592	21.528	19.080
22	0.2157	13.879	13.528	16.768	12.931	9.595
22	0.2167	14.543	8.333	12.385	7.313	7.591
22	0.2177	10.390	6.766	9.657	10.271	5.353
22	0.2187	6.396	8.820	11.971	8.728	5.294
22	0.2197	5.746	7.950	11.784	9.577	3.086
22	0.2207	6.018	7.402	12.304	8.421	9.719
22	0.2217	10.132	16.450	15.206	13.708	7.651
22	0.2227	8.924	16.065	15.314	20.898	9.550
22	0.2237	8.929	16.075	15.347	20.884	9.551
22	0.2247	14.410	13.789	21.747	14.379	9.695
22	0.2257	9.295	6.596	11.626	5.255	8.369
22	0.2267	5.835	5.505	7.025	7.375	8.093
22	0.2277	11.650	13.537	14.840	7.256	10.472
22	0.2287	16.323	19.740	18.339	17.594	14.258
22	0.2297	13.727	19.884	21.888	5.896	9.347
22	0.2307	15.469	14.519	14.125	3.081	6.775
22	0.2317	12.903	10.702	7.749	17.018	6.809
22	0.2327	12.851	12.573	13.075	14.522	11.984
22	0.2337	16.426	24.773	12.726	22.512	15.441
22	0.2347	13.375	8.795	11.131	9.338	6.621
22	0.2357	15.658	17.055	13.936	8.333	6.848
22	0.2367	9.780	14.187	14.163	8.922	5.514
22	0.2377	11.967	15.106	10.081	9.516	8.240
22	0.2387	11.969	15.109	10.075	9.515	8.249
22	0.2397	5.587	5.887	5.659	8.481	11.537
22	0.2407	6.607	16.437	15.130	9.104	11.033
22	0.2417	6.319	13.952	12.000	24.703	17.297
22	0.2427	6.180	9.502	12.608	21.420	19.196
22	0.2437	6.207	9.580	12.662	21.452	19.267

22	0.2447	8.209	8.607	14.451	25.377	18.389
22	0.2457	10.737	11.869	18.824	15.739	12.877
22	0.2467	10.799	11.908	18.836	15.852	12.942
22	0.2477	7.131	9.637	14.625	16.648	12.417
22	0.2487	5.068	7.277	3.898	16.918	5.308
22	0.2497	7.862	12.991	10.592	15.365	7.593
22	0.2507	7.885	13.063	10.607	15.409	7.614
22	0.2517	6.802	13.231	8.068	13.228	18.343
22	0.2527	9.419	12.012	15.987	13.065	12.253
22	0.2537	8.124	10.879	12.368	17.403	13.242
22	0.2547	10.147	11.981	13.484	19.413	16.558
22	0.2557	16.580	12.093	18.642	13.348	7.495
22	0.2567	11.895	14.978	19.189	16.988	17.232
22	0.2577	20.539	21.089	23.882	22.648	16.853
22	0.2587	18.245	16.050	20.757	15.641	14.117
22	0.2597	17.625	26.243	20.911	16.748	11.807
22	0.2607	14.399	20.266	18.342	18.388	6.630
22	0.2617	14.835	8.802	12.116	14.238	9.827
22	0.2627	16.126	17.097	19.104	18.875	12.930
22	0.2637	17.043	24.033	25.376	20.854	8.712
22	0.2647	24.145	24.336	27.403	19.360	10.599
22	0.2657	18.522	26.326	23.036	15.859	10.999
22	0.2667	17.899	10.440	13.911	17.860	17.935
22	0.2677	5.126	2.742	3.458	19.865	24.124
22	0.2687	9.799	16.875	13.648	18.619	15.344
22	0.2697	14.887	16.039	19.690	15.592	19.230
22	0.2707	29.079	16.400	25.700	15.474	12.188
22	0.2717	13.915	16.739	14.278	14.775	8.520
22	0.2727	10.512	15.573	14.303	17.258	9.941
22	0.2737	11.566	15.032	13.056	15.244	9.958
22	0.2747	23.507	15.699	24.669	14.031	11.864
22	0.2757	24.919	18.751	23.075	17.969	18.345
22	0.2767	10.037	7.843	5.733	15.101	14.256
22	0.2777	29.964	18.082	24.020	27.410	16.611
22	0.2787	26.642	20.458	22.851	28.851	11.590
22	0.2797	33.797	23.477	29.272	25.730	17.153
22	0.2807	27.679	25.817	27.903	18.422	11.977
22	0.2817	18.476	18.117	15.503	23.490	11.907
22	0.2827	17.193	17.313	18.984	11.066	5.074
22	0.2837	7.994	16.473	14.447	13.683	7.559
22	0.2847	18.398	13.636	17.618	20.299	16.728
22	0.2857	29.107	19.744	22.294	14.823	16.774
22	0.2867	5.717	12.226	8.816	16.330	6.012
22	0.2877	6.123	9.488	6.510	13.757	8.462
22	0.2887	3.722	2.318	5.457	3.020	0.770
22	0.2897	7.924	5.399	5.589	5.905	2.529
22	0.2907	12.577	19.043	10.407	14.596	5.849
22	0.2917	7.511	15.257	8.682	19.912	11.248
22	0.2927	10.294	12.541	9.897	14.793	22.027
22	0.2937	16.851	11.307	8.547	23.217	22.214

22	0.2947	19.478	17.647	15.003	13.475	13.585
22	0.2957	16.440	18.132	11.353	15.394	8.181
22	0.2967	11.553	21.429	12.173	17.459	4.574
22	0.2977	11.867	18.651	9.224	22.355	21.013
22	0.2987	7.999	17.119	11.412	22.401	7.182
22	0.2997	9.942	3.729	4.742	17.453	6.173
22	0.3007	15.009	14.601	14.666	17.063	13.182
22	0.3017	16.589	30.170	18.230	33.845	20.810
22	0.3027	17.297	24.677	15.511	24.978	19.475
22	0.3037	14.377	16.521	13.764	16.671	12.073
22	0.3047	13.278	11.270	10.267	18.887	14.225
22	0.3057	9.999	10.936	8.570	14.258	12.776
22	0.3067	11.898	14.639	9.342	18.694	9.754
22	0.3077	16.076	18.350	14.252	10.654	8.895
22	0.3087	12.605	8.848	11.617	11.812	12.939
22	0.3097	12.762	10.285	15.266	9.635	10.386
22	0.3107	13.303	9.282	12.160	7.901	8.861
22	0.3117	21.576	11.281	18.249	15.908	14.628
22	0.3127	9.763	10.363	10.618	6.018	13.413
22	0.3137	13.269	16.121	16.952	14.296	8.369
22	0.3147	8.771	7.508	7.336	9.567	7.938
22	0.3157	7.873	13.791	8.920	12.107	9.594
22	0.3167	9.043	10.124	9.085	15.742	14.941
22	0.3177	14.184	23.723	13.568	22.015	16.038
22	0.3187	12.566	9.921	13.788	15.187	16.339
22	0.3197	5.330	8.577	10.888	11.776	6.409
22	0.3207	10.475	8.685	8.341	11.465	10.683
22	0.3217	11.668	11.222	13.777	9.646	6.621
22	0.3227	12.984	5.383	8.935	6.273	18.233
22	0.3237	11.959	17.287	13.229	16.159	22.118
22	0.3247	9.606	17.792	15.362	18.724	25.591
22	0.3257	7.742	14.084	9.434	18.597	24.678
22	0.3267	12.703	13.055	17.006	15.384	11.034
22	0.3277	23.647	19.904	21.865	17.577	19.824
22	0.3287	6.696	11.362	10.598	16.987	14.969
22	0.3297	15.240	19.384	15.476	21.136	14.065
22	0.3307	11.666	11.012	11.137	13.319	12.164
22	0.3317	7.685	7.536	6.046	18.427	13.159
22	0.3327	10.339	13.628	8.780	16.083	15.230
22	0.3337	16.734	23.710	15.311	20.233	13.264
22	0.3347	5.473	7.085	5.441	11.161	14.001
22	0.3357	3.023	5.771	3.822	12.553	8.920
22	0.3367	3.930	9.852	4.922	13.336	7.833
22	0.3377	2.774	8.760	3.108	5.793	2.903
22	0.3387	4.027	8.287	4.349	4.430	3.277
22	0.3397	3.642	9.459	5.199	5.072	3.739
22	0.3407	17.737	20.375	18.059	13.606	6.526
22	0.3417	8.314	17.112	10.950	16.650	12.123
22	0.3427	18.952	26.510	19.911	15.260	11.472
22	0.3437	13.095	14.131	14.196	4.842	10.421

22	0.3447	9.422	13.750	8.187	10.615	13.213
22	0.3457	9.135	11.979	9.464	9.068	3.555
22	0.3467	7.172	11.519	10.264	5.500	12.887
22	0.3477	11.030	19.336	11.917	11.758	13.923
22	0.3487	15.013	17.654	15.897	9.798	11.235
22	0.3497	19.242	16.489	18.839	12.571	13.824
22	0.3507	16.120	15.883	11.267	16.245	12.767
22	0.3517	15.763	26.854	16.108	21.408	20.282
22	0.3527	14.681	14.483	19.143	11.917	13.238
22	0.3537	6.063	15.932	7.662	21.585	12.462
22	0.3547	7.789	8.107	7.963	7.427	18.247
22	0.3557	10.941	17.203	12.125	10.363	14.742
22	0.3567	12.876	27.359	18.993	35.721	14.199
22	0.3577	12.117	20.290	13.936	15.887	13.521
22	0.3587	20.601	17.516	18.727	8.696	11.420
22	0.3597	29.773	37.592	40.412	26.857	26.353
22	0.3607	23.466	26.852	25.622	12.849	25.162
22	0.3617	25.409	31.976	34.765	13.209	13.964
22	0.3627	12.374	19.084	13.059	16.388	11.745
22	0.3637	7.454	14.547	7.874	12.810	5.365
22	0.3647	13.976	19.342	12.309	17.601	5.994
22	0.3657	14.815	18.386	12.784	16.895	30.991
22	0.3667	10.025	12.402	17.366	15.036	17.245
22	0.3677	9.121	10.854	16.824	20.232	24.838
22	0.3687	7.636	4.324	7.435	9.755	18.500
22	0.3697	9.610	4.828	8.039	9.541	8.804
22	0.3707	14.739	9.417	17.767	10.089	12.482
22	0.3717	13.097	10.868	15.209	14.055	9.586
22	0.3727	12.621	12.052	15.885	12.627	10.403
22	0.3737	12.390	11.817	12.631	15.337	10.484
22	0.3747	17.196	19.281	20.765	15.736	12.845
22	0.3757	15.642	13.483	19.070	10.933	11.880
22	0.3767	15.386	14.405	16.997	18.822	11.401
22	0.3777	12.930	11.769	14.550	17.876	15.951
22	0.3787	12.232	14.822	17.325	9.753	24.376
22	0.3797	8.258	15.872	13.381	12.396	9.059
22	0.3807	7.315	16.883	13.287	19.031	19.359
22	0.3817	5.476	17.542	19.862	19.866	19.605
22	0.3827	16.908	23.656	27.305	22.655	18.743
22	0.3837	17.564	21.711	24.849	17.346	14.730
22	0.3847	11.656	15.030	15.043	20.375	21.364
22	0.3857	16.403	19.109	24.944	9.772	17.044
22	0.3867	18.612	17.289	21.797	20.389	19.523
22	0.3877	16.824	21.905	22.875	15.391	22.631
22	0.3887	13.365	16.049	19.433	22.416	15.170
22	0.3897	20.835	24.292	29.552	18.819	13.285
22	0.3907	11.819	15.298	13.697	14.840	15.966
22	0.3917	10.263	17.445	15.939	12.288	13.811
22	0.3927	7.352	9.743	9.529	15.917	14.900
22	0.3937	19.818	23.250	25.744	17.779	13.919

22	0.3947	11.301	12.789	19.302	21.984	11.028
22	0.3957	14.552	10.891	18.376	6.966	10.859
22	0.3967	13.288	14.633	18.791	7.385	8.452
22	0.3977	12.127	15.396	16.486	5.712	9.399
22	0.3987	9.294	8.902	9.731	6.661	7.374
22	0.3997	12.438	13.160	14.612	10.189	7.845
22	0.4007	13.541	16.717	17.498	12.134	12.581
22	0.4017	9.471	9.648	10.756	16.449	7.252
22	0.4027	13.274	18.416	18.928	15.175	9.313
22	0.4037	13.389	9.122	11.861	14.437	16.441
22	0.4047	15.340	13.818	18.404	15.667	12.510
22	0.4057	13.778	9.724	14.938	12.938	10.950
22	0.4067	13.754	14.879	16.897	9.312	13.826
22	0.4077	12.011	11.576	12.036	12.418	12.463
22	0.4087	10.051	13.419	14.980	7.846	11.421
22	0.4097	8.528	13.238	14.186	15.080	13.937
22	0.4107	9.009	16.546	13.569	16.014	11.408
22	0.4117	10.386	9.707	10.949	14.524	11.594
22	0.4127	11.302	13.382	14.020	12.435	19.647
22	0.4137	13.396	12.009	14.089	11.024	13.784
22	0.4147	10.949	12.097	13.618	12.772	10.020
22	0.4157	8.985	12.178	6.723	17.383	12.053
22	0.4167	22.045	14.770	18.074	21.519	9.946
22	0.4177	7.516	10.932	7.075	20.671	15.554
22	0.4187	16.577	13.577	13.710	14.175	14.427
22	0.4197	12.003	9.501	11.481	8.347	11.552
22	0.4207	17.029	20.774	12.867	9.847	15.030
22	0.4217	11.022	15.076	11.178	6.544	7.997
22	0.4227	6.457	12.637	11.270	5.209	3.636
22	0.4237	9.316	8.832	9.912	11.978	9.371
22	0.4247	11.558	7.024	9.847	9.982	8.799
22	0.4257	25.029	13.539	22.439	19.491	13.523
22	0.4267	8.259	8.935	9.577	8.363	7.446
22	0.4277	6.362	6.171	6.294	13.429	11.438
22	0.4287	8.391	11.872	9.697	26.147	15.690
22	0.4297	13.451	12.616	15.144	17.912	17.075
22	0.4307	10.340	22.243	12.185	28.449	15.067
22	0.4317	10.643	22.578	12.934	29.576	17.305
22	0.4327	16.111	24.558	27.385	21.872	17.291
22	0.4337	14.341	6.530	16.055	10.386	10.697
22	0.4347	14.989	6.716	16.458	10.401	10.436
22	0.4357	16.488	12.023	15.996	11.849	11.518
22	0.4367	9.075	10.279	8.221	13.201	10.579
22	0.4377	7.510	14.043	11.357	22.304	14.335
22	0.4387	13.117	15.447	11.878	23.028	22.455
22	0.4397	13.290	15.489	11.885	22.935	22.370
22	0.4407	5.195	8.443	5.315	5.055	13.347
22	0.4417	7.418	10.662	8.683	11.990	21.726
22	0.4427	9.606	8.685	9.642	9.960	21.014
22	0.4437	13.654	15.293	16.301	23.158	28.159

22	0.4447	6.910	7.272	9.073	15.271	18.572
22	0.4457	5.417	11.764	7.628	11.997	12.019
22	0.4467	4.218	12.412	6.137	21.042	13.824
22	0.4477	25.650	19.728	27.898	22.563	21.447
22	0.4487	21.373	15.718	23.205	14.707	17.793
22	0.4497	2.235	5.530	7.334	14.141	15.085
22	0.4507	1.921	4.771	6.027	8.781	12.957
22	0.4517	9.427	5.229	7.404	9.126	7.411
22	0.4527	4.734	13.294	9.984	17.116	4.438
22	0.4537	9.360	11.936	12.201	9.376	7.704
22	0.4547	22.467	32.394	36.324	11.831	25.975
22	0.4557	17.466	24.980	29.043	14.613	19.529
22	0.4567	10.772	19.148	19.369	15.999	22.543
22	0.4577	12.232	11.879	13.125	25.619	17.985
22	0.4587	15.923	17.128	15.338	13.240	10.718
22	0.4597	15.330	14.932	17.778	19.300	21.642
22	0.4607	17.691	14.598	19.824	13.794	8.567
22	0.4617	19.369	18.772	20.092	18.205	16.433
22	0.4627	18.567	18.908	23.856	15.164	22.883
22	0.4637	13.049	24.575	27.028	14.440	17.088
22	0.4647	12.920	24.724	27.178	14.615	17.087
22	0.4657	30.313	18.549	24.791	15.879	17.629
22	0.4667	30.539	20.274	22.718	12.303	26.265
22	0.4677	25.683	18.117	18.430	12.185	20.383
22	0.4687	14.346	11.471	14.668	5.887	21.058
22	0.4697	35.257	30.681	37.062	7.025	20.566
22	0.4707	20.971	25.831	20.989	23.537	16.663
22	0.4717	22.775	23.663	18.022	9.114	17.067
22	0.4727	22.631	23.102	19.949	7.804	31.446
22	0.4737	21.595	19.638	19.999	23.010	12.853
22	0.4747	16.261	22.821	16.579	12.927	10.789
22	0.4757	16.470	14.360	16.981	11.511	10.468
22	0.4767	9.177	9.596	10.996	14.347	9.624
22	0.4777	4.100	4.876	7.549	9.593	9.754
22	0.4787	16.214	21.828	19.675	11.449	6.832
22	0.4797	20.793	18.112	25.350	14.976	10.535
22	0.4807	15.434	19.114	17.924	9.261	17.440
22	0.4817	23.044	23.648	26.769	18.436	20.270
22	0.4827	23.114	23.693	26.826	18.433	20.329
22	0.4837	12.459	12.512	15.813	15.311	14.155
22	0.4847	19.408	18.784	27.279	6.037	10.243
22	0.4857	10.309	13.299	19.463	2.573	9.401
22	0.4867	16.023	9.089	15.445	12.053	28.267
22	0.4877	33.553	31.270	35.127	13.140	20.072
22	0.4887	32.119	30.451	31.039	5.031	17.408
22	0.4897	33.581	32.470	33.496	4.194	15.160
22	0.4907	10.421	8.924	18.880	7.973	13.205
22	0.4917	16.710	9.573	18.089	6.922	7.852
22	0.4927	4.009	2.589	3.138	4.515	14.994
22	0.4937	3.979	3.957	2.623	8.904	8.524

22	0.4947	10.277	20.063	14.815	12.240	11.644
22	0.4957	26.937	29.471	26.331	17.540	16.925
22	0.4967	30.577	23.648	29.788	8.603	6.037
22	0.4977	30.587	23.652	29.798	8.601	6.037
22	0.4987	27.685	19.541	30.093	11.011	30.504
22	0.4997	20.278	13.406	24.384	6.850	9.975
22	0.5007	14.116	7.720	10.246	5.604	12.153
22	0.5017	16.291	15.245	15.709	10.860	19.421
22	0.5027	17.507	20.393	22.756	15.361	7.363
22	0.5037	19.023	23.813	23.882	12.607	6.231
22	0.5047	12.506	12.578	11.727	12.375	11.819
22	0.5057	9.332	10.950	13.749	5.008	10.460
22	0.5067	15.477	14.000	15.901	10.510	38.321
22	0.5077	15.400	13.948	15.447	9.886	21.558
22	0.5087	18.203	17.613	15.959	13.448	21.204
22	0.5097	17.246	17.614	17.694	21.960	16.707
22	0.5107	14.787	24.589	16.166	16.617	16.142
22	0.5117	20.101	16.188	18.280	18.064	20.450
22	0.5127	16.321	15.809	15.719	17.118	15.217
22	0.5137	16.320	15.797	15.716	17.119	15.213
22	0.5147	23.997	25.996	31.117	12.812	11.394
22	0.5157	24.047	26.022	31.160	12.828	11.413
22	0.5167	8.241	14.860	18.248	11.938	9.894
22	0.5177	17.049	24.233	27.260	6.989	10.717
22	0.5187	19.644	22.554	30.858	6.407	16.524
22	0.5197	14.110	15.603	19.502	15.559	10.115
22	0.5207	27.485	19.500	29.449	7.532	12.047
22	0.5217	11.913	10.351	19.343	5.269	8.141
22	0.5227	10.665	6.989	15.049	5.902	6.083
22	0.5237	11.503	10.131	13.239	5.109	7.046
22	0.5247	24.793	23.780	28.966	17.853	19.409
22	0.5257	34.938	28.622	35.949	25.408	21.370
22	0.5267	37.511	32.501	47.294	23.612	22.907
22	0.5277	27.078	26.012	28.132	16.178	14.824
22	0.5287	36.706	24.966	33.099	29.005	14.146
22	0.5297	25.609	29.767	32.401	25.299	19.596
22	0.5307	18.963	20.143	31.215	12.040	19.142
22	0.5317	28.771	30.004	29.671	14.632	8.536
22	0.5327	27.086	32.689	35.419	15.331	10.412
22	0.5337	27.351	34.564	37.673	16.291	20.615
22	0.5347	10.717	17.545	15.082	11.884	19.472
22	0.5357	17.703	28.225	25.727	12.753	16.857
22	0.5367	17.714	25.259	19.147	16.691	23.615
22	0.5377	30.366	36.354	44.192	16.725	18.135
22	0.5387	14.391	23.876	24.443	19.747	18.541
22	0.5397	16.528	24.284	19.183	24.716	15.239
22	0.5407	9.279	14.049	11.705	20.519	11.252
22	0.5417	24.309	29.117	23.317	27.002	17.852
22	0.5427	19.289	21.113	24.750	19.126	22.661
22	0.5437	27.208	24.665	28.125	11.515	19.789

22	0.5447	16.494	18.380	17.330	16.179	15.619
22	0.5457	18.193	18.219	17.505	15.321	20.068
22	0.5467	14.870	23.835	21.152	10.107	17.392
22	0.5477	11.700	13.538	13.051	14.441	21.267
22	0.5487	14.312	14.542	17.759	11.553	17.350
22	0.5497	17.445	13.555	16.890	25.206	8.416
22	0.5507	22.181	22.569	25.243	4.110	8.488
22	0.5517	12.638	16.531	16.964	20.858	15.147
22	0.5527	13.120	17.561	13.548	17.180	22.489
22	0.5537	13.347	18.176	13.757	16.800	23.022
22	0.5547	13.524	18.689	13.926	16.261	23.370
22	0.5557	24.285	20.969	27.380	6.895	14.374
22	0.5567	28.353	19.857	26.365	7.481	17.362
22	0.5577	11.045	17.224	14.536	13.611	13.520
22	0.5587	11.010	6.107	9.990	8.937	18.979
22	0.5597	25.489	13.104	17.263	9.000	26.045
22	0.5607	20.986	15.526	20.107	12.536	16.876
22	0.5617	14.573	10.829	14.938	17.845	12.710
22	0.5627	10.264	8.737	11.329	16.694	23.169
22	0.5637	13.737	12.763	15.738	24.160	22.481
22	0.5647	15.421	8.133	16.522	17.687	20.625
22	0.5657	12.152	9.765	15.128	20.201	7.982
22	0.5667	20.035	8.991	11.376	23.492	39.235
22	0.5677	10.869	5.082	9.520	17.987	14.993
22	0.5687	6.938	7.538	9.222	13.793	15.457
22	0.5697	6.257	6.817	8.538	13.696	15.347
22	0.5707	5.581	4.355	8.998	9.441	5.505
22	0.5717	15.455	11.225	20.500	10.371	14.539
22	0.5727	10.972	13.100	12.565	33.256	21.547
22	0.5737	10.461	13.110	12.260	33.858	20.693
22	0.5747	8.208	12.095	9.508	22.005	17.505
22	0.5757	6.211	12.432	8.312	18.160	15.438
22	0.5767	6.026	12.389	8.213	18.168	15.176
22	0.5777	3.733	2.949	2.433	6.042	8.938
22	0.5787	0.000	0.000	0.000	0.000	0.000
23	0.0007	0.000	0.000	0.000	0.000	0.000
23	0.0017	6.859	8.962	5.871	13.955	22.153
23	0.0027	11.096	10.222	4.248	13.150	16.968
23	0.0037	9.956	7.980	5.275	4.619	23.304
23	0.0047	9.633	11.670	5.550	8.189	15.475
23	0.0057	5.366	4.487	2.475	9.343	17.735
23	0.0067	5.318	8.900	6.626	7.186	4.551
23	0.0077	2.889	6.863	4.745	4.296	6.297
23	0.0087	13.747	14.969	9.345	6.580	2.618
23	0.0097	6.798	10.429	5.304	2.098	5.496
23	0.0107	9.459	13.258	5.990	8.073	11.520
23	0.0117	15.606	14.189	8.147	16.222	25.531
23	0.0127	14.615	11.170	8.869	13.871	26.705
23	0.0137	10.178	8.260	5.650	11.649	10.227
23	0.0147	4.687	5.056	4.216	5.021	5.614

23	0.0157	19.888	16.662	14.100	6.190	17.099
23	0.0167	7.684	8.407	5.175	6.629	10.392
23	0.0177	8.222	9.583	10.733	11.459	7.936
23	0.0187	8.213	8.835	10.043	10.329	17.304
23	0.0197	11.088	5.277	6.722	3.404	10.973
23	0.0207	10.030	8.505	8.754	18.214	11.203
23	0.0217	14.071	12.477	10.419	18.606	7.797
23	0.0227	15.367	13.724	11.604	18.679	11.167
23	0.0237	4.105	5.905	3.525	15.546	5.208
23	0.0247	4.255	6.019	2.911	20.828	6.301
23	0.0257	3.692	4.471	4.370	19.712	11.496
23	0.0267	11.661	10.432	8.369	13.158	24.471
23	0.0277	11.952	10.541	8.054	18.940	26.497
23	0.0287	9.342	7.230	5.457	17.960	26.037
23	0.0297	7.825	9.148	2.932	18.441	11.527
23	0.0307	14.066	11.510	7.610	14.763	31.064
23	0.0317	8.912	9.321	7.802	16.983	21.581
23	0.0327	10.399	9.704	2.028	16.747	9.543
23	0.0337	8.347	8.540	3.103	18.555	17.930
23	0.0347	17.432	16.891	11.519	24.254	24.183
23	0.0357	13.579	13.847	6.204	20.409	23.282
23	0.0367	13.501	15.045	5.677	29.060	23.170
23	0.0377	18.715	19.910	17.957	31.153	20.711
23	0.0387	25.219	23.288	18.734	24.469	18.307
23	0.0397	13.231	21.346	10.825	31.798	20.026
23	0.0407	17.165	10.349	7.320	14.679	15.172
23	0.0417	14.769	19.560	10.091	20.895	12.874
23	0.0427	15.484	19.229	16.246	20.283	18.589
23	0.0437	8.317	7.478	6.002	19.362	12.047
23	0.0447	15.268	12.746	13.662	22.582	16.222
23	0.0457	10.323	10.633	7.327	20.691	17.245
23	0.0467	11.989	15.450	11.253	25.857	15.902
23	0.0477	21.624	13.448	14.378	25.292	17.977
23	0.0487	25.507	12.756	17.005	20.783	17.358
23	0.0497	16.062	11.971	9.635	13.488	17.482
23	0.0507	23.684	16.611	16.847	11.651	18.954
23	0.0517	19.071	8.942	10.738	16.992	11.811
23	0.0527	9.188	5.215	5.372	7.964	19.887
23	0.0537	10.562	10.629	6.286	14.998	23.388
23	0.0547	19.504	19.999	14.772	27.365	17.732
23	0.0557	19.947	5.051	6.643	9.042	14.281
23	0.0567	10.059	8.148	4.271	10.791	19.125
23	0.0577	9.763	9.214	6.889	22.632	16.328
23	0.0587	14.991	8.027	9.199	14.660	17.332
23	0.0597	9.562	3.568	2.925	16.790	14.701
23	0.0607	18.872	8.374	13.735	16.755	15.546
23	0.0617	13.476	11.924	8.965	17.910	15.744
23	0.0627	17.143	13.073	14.166	12.607	21.962
23	0.0637	17.647	16.078	16.097	20.453	36.153
23	0.0647	14.077	11.909	14.311	23.203	26.408

23	0.0657	12.417	7.433	6.047	12.065	19.068
23	0.0667	19.652	8.444	13.419	19.533	15.612
23	0.0677	21.176	10.397	12.422	17.157	26.463
23	0.0687	23.586	14.930	18.594	20.658	32.958
23	0.0697	22.013	22.157	19.081	17.653	17.085
23	0.0707	13.316	9.433	12.306	15.237	19.393
23	0.0717	13.211	7.590	8.303	17.175	12.953
23	0.0727	14.482	7.656	8.480	18.331	11.280
23	0.0737	19.274	10.597	9.496	29.184	19.154
23	0.0747	8.680	4.019	7.726	10.329	12.003
23	0.0757	18.864	7.557	8.335	9.540	28.116
23	0.0767	19.417	13.785	10.262	30.053	27.046
23	0.0777	11.505	6.422	6.480	16.685	15.072
23	0.0787	20.413	6.504	7.340	17.770	30.271
23	0.0797	20.367	6.406	7.311	17.787	30.172
23	0.0807	13.151	5.671	3.982	8.211	10.893
23	0.0817	19.934	4.110	8.866	9.437	17.729
23	0.0827	17.727	7.422	6.306	8.927	14.167
23	0.0837	15.119	6.146	8.043	7.027	8.562
23	0.0847	10.323	5.519	3.307	5.406	16.916
23	0.0857	10.846	5.901	4.286	14.112	15.476
23	0.0867	7.648	9.109	4.003	23.105	10.441
23	0.0877	8.574	4.589	4.784	10.227	8.089
23	0.0887	8.355	7.215	7.243	12.440	9.572
23	0.0897	9.676	9.985	9.740	15.178	8.813
23	0.0907	10.907	9.610	8.880	11.348	13.022
23	0.0917	20.582	12.788	11.352	9.502	19.878
23	0.0927	22.788	15.627	14.033	12.788	20.499
23	0.0937	19.279	12.724	11.599	11.160	26.744
23	0.0947	7.772	2.842	8.259	12.439	22.315
23	0.0957	12.617	5.046	10.658	11.664	15.463
23	0.0967	9.656	5.974	9.197	3.931	13.516
23	0.0977	4.353	2.769	5.211	9.878	4.643
23	0.0987	11.802	10.763	3.269	7.612	12.053
23	0.0997	23.512	19.243	13.978	15.682	10.638
23	0.1007	13.167	6.276	7.330	16.446	14.513
23	0.1017	12.525	8.985	7.635	13.344	13.963
23	0.1027	19.824	13.944	10.073	15.354	16.067
23	0.1037	10.259	9.635	6.378	22.966	29.303
23	0.1047	12.545	8.393	5.910	17.091	19.499
23	0.1057	12.986	11.567	8.144	20.689	24.324
23	0.1067	20.976	12.574	12.419	25.502	18.789
23	0.1077	18.281	16.576	13.127	21.818	15.221
23	0.1087	9.297	7.227	7.323	5.292	10.555
23	0.1097	10.749	5.811	4.782	8.219	13.136
23	0.1107	13.435	10.736	6.885	13.163	9.132
23	0.1117	7.481	10.221	7.936	17.683	16.136
23	0.1127	6.324	5.892	6.664	13.119	23.910
23	0.1137	8.958	8.855	8.602	12.657	14.781
23	0.1147	2.075	0.896	0.673	4.670	5.637

23	0.1157	5.429	4.725	2.752	4.079	10.742
23	0.1167	9.684	7.762	4.705	5.385	7.634
23	0.1177	12.121	14.857	10.404	13.083	9.666
23	0.1187	4.451	2.891	6.123	8.293	3.994
23	0.1197	11.750	6.151	7.510	12.467	14.302
23	0.1207	13.431	4.373	6.979	14.963	18.352
23	0.1217	12.318	6.855	7.905	7.884	17.490
23	0.1227	16.505	12.862	15.817	14.943	21.134
23	0.1237	11.133	12.377	5.876	19.618	16.287
23	0.1247	5.262	14.098	6.009	12.781	13.641
23	0.1257	11.418	15.180	11.367	9.849	19.445
23	0.1267	6.289	9.196	8.804	15.719	18.251
23	0.1277	13.375	9.731	11.130	13.512	19.647
23	0.1287	15.470	7.522	8.898	9.528	30.533
23	0.1297	15.923	9.981	8.538	15.903	27.333
23	0.1307	16.163	16.025	12.393	24.471	20.560
23	0.1317	15.604	8.055	6.158	12.977	16.697
23	0.1327	8.261	15.445	5.626	10.842	19.724
23	0.1337	21.842	21.658	17.340	14.274	30.019
23	0.1347	13.163	9.536	6.949	17.723	26.842
23	0.1357	11.303	13.367	8.829	19.726	28.252
23	0.1367	9.692	11.213	6.796	17.550	18.135
23	0.1377	7.960	9.630	9.206	15.426	15.104
23	0.1387	11.862	14.446	12.494	15.022	21.251
23	0.1397	12.603	9.351	9.606	5.900	20.684
23	0.1407	13.781	12.072	12.274	11.783	16.062
23	0.1417	17.383	9.366	11.297	19.211	22.435
23	0.1427	17.214	11.443	10.760	17.140	17.698
23	0.1437	15.157	15.925	10.862	14.599	6.928
23	0.1447	14.115	15.188	6.457	21.227	24.337
23	0.1457	13.710	14.620	8.675	20.757	16.400
23	0.1467	2.893	9.107	4.114	20.606	16.159
23	0.1477	17.894	7.668	7.870	20.161	21.245
23	0.1487	8.967	10.278	6.865	19.496	21.561
23	0.1497	15.653	15.334	9.945	21.127	16.660
23	0.1507	8.828	10.009	5.557	20.517	16.864
23	0.1517	4.467	8.666	4.752	21.359	25.450
23	0.1527	14.106	19.305	13.084	22.798	19.395
23	0.1537	7.934	8.678	4.283	17.631	14.970
23	0.1547	13.449	13.469	8.970	16.946	20.191
23	0.1557	13.383	13.450	8.939	16.934	20.295
23	0.1567	30.596	34.293	20.841	16.372	27.251
23	0.1577	10.595	18.310	10.541	18.611	23.486
23	0.1587	18.815	22.709	11.316	23.110	21.087
23	0.1597	8.442	14.187	7.980	18.435	17.316
23	0.1607	11.358	15.642	9.630	31.233	19.293
23	0.1617	13.229	11.275	8.400	17.699	12.350
23	0.1627	12.246	25.665	16.277	22.445	17.128
23	0.1637	8.986	16.445	15.212	25.532	32.601
23	0.1647	6.258	7.119	6.572	20.792	23.843

23	0.1657	8.025	7.543	6.364	22.918	33.924
23	0.1667	4.609	1.892	2.401	10.419	17.590
23	0.1677	2.950	7.730	4.625	7.971	5.234
23	0.1687	5.689	6.715	4.066	8.905	10.591
23	0.1697	4.734	7.101	2.906	13.978	7.182
23	0.1707	5.850	9.843	7.261	15.401	3.367
23	0.1717	9.093	12.944	11.412	9.344	15.368
23	0.1727	8.464	16.638	16.317	25.197	22.858
23	0.1737	3.817	8.506	10.719	9.245	12.557
23	0.1747	7.707	13.169	10.353	14.293	18.950
23	0.1757	9.367	7.645	7.634	11.291	20.024
23	0.1767	16.769	22.016	19.537	21.313	24.435
23	0.1777	23.531	24.406	19.546	15.991	20.018
23	0.1787	11.129	15.215	17.343	12.842	10.744
23	0.1797	15.477	16.956	19.360	17.005	14.659
23	0.1807	11.350	11.683	10.636	15.191	24.430
23	0.1817	11.958	13.530	10.697	6.306	11.254
23	0.1827	10.628	11.255	9.572	9.734	12.293
23	0.1837	17.891	21.042	14.342	18.397	19.633
23	0.1847	15.012	16.915	10.967	14.376	19.182
23	0.1857	15.774	14.119	10.701	9.249	20.087
23	0.1867	12.979	8.141	10.594	7.801	12.614
23	0.1877	4.230	5.600	5.355	5.629	5.924
23	0.1887	8.597	9.772	12.975	9.308	10.753
23	0.1897	23.308	31.542	27.289	23.591	16.035
23	0.1907	17.265	13.163	12.632	25.167	9.427
23	0.1917	14.406	16.419	17.847	26.689	15.990
23	0.1927	11.201	22.366	16.186	28.514	16.368
23	0.1937	13.752	16.651	19.371	20.422	9.914
23	0.1947	11.363	11.818	10.687	15.933	16.781
23	0.1957	20.724	13.984	12.688	16.137	25.120
23	0.1967	13.154	13.639	9.615	17.434	22.608
23	0.1977	11.488	15.755	9.744	22.239	21.366
23	0.1987	10.812	6.719	7.383	12.830	20.568
23	0.1997	9.182	14.362	9.105	11.483	18.540
23	0.2007	24.507	31.217	29.867	13.476	13.896
23	0.2017	22.114	23.948	24.029	21.391	13.406
23	0.2027	19.320	17.622	21.514	14.685	8.437
23	0.2037	9.727	14.268	10.106	11.307	18.549
23	0.2047	13.082	16.000	10.847	20.449	13.400
23	0.2057	8.781	10.982	8.186	18.044	15.575
23	0.2067	1.376	4.627	3.300	9.977	8.828
23	0.2077	1.380	4.665	3.301	10.062	8.785
23	0.2087	4.882	8.027	7.619	10.861	11.103
23	0.2097	6.258	6.013	5.136	6.478	6.976
23	0.2107	8.649	11.568	7.069	11.955	13.298
23	0.2117	12.621	9.978	11.250	24.148	14.433
23	0.2127	16.539	16.470	17.061	20.834	12.252
23	0.2137	7.223	12.705	8.243	14.849	7.404
23	0.2147	6.657	7.007	8.812	25.300	6.770

23	0.2157	12.473	19.474	14.307	27.975	10.231
23	0.2167	24.977	33.972	32.872	18.665	12.005
23	0.2177	24.699	22.611	20.819	18.602	9.795
23	0.2187	16.001	14.770	17.190	19.274	16.233
23	0.2197	9.757	11.840	10.163	3.114	12.133
23	0.2207	10.932	8.063	2.913	6.336	7.366
23	0.2217	18.410	15.870	10.858	10.101	13.822
23	0.2227	18.014	19.163	15.037	14.451	15.025
23	0.2237	12.176	20.963	14.222	15.597	12.555
23	0.2247	14.590	14.488	9.645	18.428	23.125
23	0.2257	14.831	16.299	10.338	9.826	24.596
23	0.2267	7.965	14.875	10.017	23.139	8.957
23	0.2277	13.015	18.450	16.045	20.249	18.331
23	0.2287	10.282	12.457	8.618	9.414	21.394
23	0.2297	15.843	14.057	15.030	27.387	17.420
23	0.2307	11.955	19.376	15.948	26.547	22.794
23	0.2317	10.094	12.084	7.237	13.099	17.965
23	0.2327	13.588	11.647	7.160	12.060	4.908
23	0.2337	5.868	10.339	6.093	16.842	10.774
23	0.2347	8.369	8.045	5.885	8.567	12.990
23	0.2357	15.858	22.716	13.360	15.799	18.245
23	0.2367	11.263	17.000	12.564	12.805	15.591
23	0.2377	10.771	12.365	12.763	8.772	19.554
23	0.2387	8.528	8.353	8.602	3.840	10.411
23	0.2397	16.962	19.260	15.748	19.377	12.962
23	0.2407	15.200	16.559	14.737	17.159	29.336
23	0.2417	5.538	13.381	11.482	15.728	19.742
23	0.2427	11.226	13.697	9.885	16.282	9.089
23	0.2437	25.296	23.732	19.841	11.149	21.081
23	0.2447	11.012	11.231	8.383	9.347	9.664
23	0.2457	6.642	12.258	9.274	13.426	14.958
23	0.2467	11.088	17.190	11.007	18.934	13.647
23	0.2477	9.347	17.399	9.554	28.646	13.818
23	0.2487	9.793	11.285	5.869	14.988	14.578
23	0.2497	11.977	19.327	10.447	22.353	21.710
23	0.2507	11.965	19.365	10.466	22.311	21.676
23	0.2517	11.953	19.405	10.484	22.271	21.643
23	0.2527	7.470	6.523	5.633	6.824	9.391
23	0.2537	4.937	8.005	7.543	10.137	4.499
23	0.2547	4.910	8.090	7.550	10.170	4.543
23	0.2557	10.405	8.981	6.711	15.948	16.638
23	0.2567	10.597	14.017	9.425	14.859	12.316
23	0.2577	21.633	23.210	11.960	20.798	19.215
23	0.2587	22.205	16.952	12.613	17.433	12.034
23	0.2597	22.059	16.373	14.138	21.792	13.827
23	0.2607	14.497	9.661	13.253	19.423	15.751
23	0.2617	13.453	7.982	10.973	21.302	17.038
23	0.2627	8.202	9.056	10.035	14.616	13.492
23	0.2637	7.636	11.726	7.997	21.059	11.846
23	0.2647	8.066	9.434	6.125	14.807	12.375

23	0.2657	11.848	12.042	8.897	10.137	10.959
23	0.2667	12.144	14.879	10.411	19.568	23.351
23	0.2677	12.150	14.881	10.418	19.540	23.325
23	0.2687	19.409	17.815	16.659	19.052	18.041
23	0.2697	8.290	8.215	7.818	20.774	13.428
23	0.2707	9.817	7.129	7.999	19.189	9.777
23	0.2717	9.792	7.145	8.037	19.063	9.719
23	0.2727	9.456	9.692	8.105	17.640	10.487
23	0.2737	10.489	10.178	13.355	12.418	11.454
23	0.2747	21.749	18.997	21.600	17.784	16.801
23	0.2757	19.412	13.168	12.605	20.071	21.113
23	0.2767	14.238	10.693	13.918	22.507	12.876
23	0.2777	18.994	9.604	11.413	12.402	13.900
23	0.2787	9.263	15.249	10.393	9.431	7.309
23	0.2797	7.724	8.672	8.395	6.075	5.929
23	0.2807	12.605	13.171	9.837	14.278	6.107
23	0.2817	18.724	16.509	11.887	28.774	14.409
23	0.2827	19.424	16.676	12.401	25.050	17.853
23	0.2837	20.149	13.262	17.258	13.370	9.766
23	0.2847	23.852	14.219	23.329	19.023	7.915
23	0.2857	12.715	10.979	13.002	23.231	5.928
23	0.2867	19.101	19.065	16.921	7.028	9.092
23	0.2877	23.334	25.705	24.344	7.521	9.708
23	0.2887	11.778	11.187	7.308	14.707	8.743
23	0.2897	16.777	14.880	11.211	10.598	12.624
23	0.2907	17.061	14.252	14.862	22.325	22.946
23	0.2917	14.805	10.208	9.795	27.364	17.108
23	0.2927	23.238	19.164	17.604	16.894	23.990
23	0.2937	17.090	20.460	15.340	17.632	23.990
23	0.2947	20.266	16.172	14.664	17.422	10.991
23	0.2957	7.760	9.312	8.328	10.936	15.754
23	0.2967	6.696	11.042	7.065	19.412	17.954
23	0.2977	6.696	11.042	7.065	19.411	17.955
23	0.2987	9.638	10.493	7.219	17.257	7.026
23	0.2997	5.892	7.001	6.543	9.939	11.516
23	0.3007	13.310	9.405	12.246	12.725	3.177
23	0.3017	9.136	9.094	13.936	15.654	11.698
23	0.3027	9.401	14.986	14.428	17.481	25.501
23	0.3037	9.504	15.056	14.477	17.370	25.462
23	0.3047	17.068	18.426	12.708	26.515	14.455
23	0.3057	15.957	15.293	9.473	38.313	19.240
23	0.3067	20.933	18.811	13.331	39.017	22.306
23	0.3077	14.809	16.795	15.182	29.400	16.572
23	0.3087	16.200	10.137	9.027	28.172	12.036
23	0.3097	12.537	22.617	14.402	39.041	18.909
23	0.3107	21.005	19.689	20.748	19.892	15.218
23	0.3117	7.263	15.743	11.714	14.468	6.771
23	0.3127	13.455	19.882	17.020	14.269	6.776
23	0.3137	8.928	8.619	11.188	19.444	9.627
23	0.3147	23.521	18.422	18.925	12.049	9.284

23	0.3157	20.853	21.285	18.396	15.365	10.946
23	0.3167	20.107	18.171	16.480	10.417	10.197
23	0.3177	18.710	11.057	9.351	14.828	18.199
23	0.3187	13.870	19.836	12.776	25.731	14.590
23	0.3197	14.129	15.631	13.954	27.747	11.010
23	0.3207	14.123	15.620	13.947	27.728	11.016
23	0.3217	22.983	20.758	22.601	14.566	9.003
23	0.3227	20.481	20.023	15.315	14.563	12.621
23	0.3237	20.474	20.087	15.313	14.637	12.665
23	0.3247	25.133	19.579	16.700	13.729	15.441
23	0.3257	24.686	18.065	10.878	12.356	12.011
23	0.3267	11.279	19.200	11.200	23.364	9.332
23	0.3277	11.455	9.343	9.436	16.658	12.874
23	0.3287	12.099	7.424	12.466	15.107	17.701
23	0.3297	13.136	10.045	13.927	8.489	20.056
23	0.3307	5.429	8.540	3.421	15.142	14.551
23	0.3317	15.112	10.554	7.472	18.848	11.680
23	0.3327	12.621	12.262	8.590	15.961	12.937
23	0.3337	11.032	13.302	10.027	13.466	14.122
23	0.3347	19.320	12.193	12.736	22.949	6.199
23	0.3357	9.178	11.949	9.013	24.862	6.957
23	0.3367	28.727	28.222	28.785	18.806	20.551
23	0.3377	7.911	11.631	9.617	7.437	3.666
23	0.3387	15.931	10.241	10.468	10.678	14.679
23	0.3397	6.869	9.438	10.265	10.335	15.236
23	0.3407	7.308	12.011	13.193	18.334	28.356
23	0.3417	14.473	18.946	23.270	20.033	30.263
23	0.3427	20.665	18.826	20.371	14.661	18.434
23	0.3437	14.188	9.030	10.032	18.970	19.748
23	0.3447	8.772	5.964	5.663	15.374	10.771
23	0.3457	5.973	9.132	9.536	16.178	13.876
23	0.3467	11.767	10.933	14.086	14.218	10.490
23	0.3477	10.559	12.562	8.243	14.798	8.845
23	0.3487	12.045	8.740	15.061	11.246	20.785
23	0.3497	15.107	13.291	12.250	7.231	17.434
23	0.3507	12.667	11.587	11.634	16.445	25.969
23	0.3517	11.301	12.757	13.650	12.683	17.035
23	0.3527	13.759	14.569	14.796	13.524	19.730
23	0.3537	12.506	15.293	13.398	11.502	12.684
23	0.3547	9.148	12.432	9.741	16.371	10.080
23	0.3557	5.046	6.580	4.856	4.423	2.274
23	0.3567	3.481	6.947	4.055	13.050	6.096
23	0.3577	7.791	13.547	8.127	27.289	10.370
23	0.3587	17.031	17.364	14.926	16.440	22.946
23	0.3597	10.885	13.988	7.321	25.545	13.648
23	0.3607	10.562	12.748	8.695	9.802	10.383
23	0.3617	10.609	12.864	8.791	9.772	10.468
23	0.3627	6.884	15.077	9.552	21.720	14.039
23	0.3637	11.163	15.866	21.018	14.606	8.170
23	0.3647	15.245	14.333	21.370	8.757	12.370

23	0.3657	11.278	6.318	8.525	11.849	6.701
23	0.3667	16.870	12.343	16.747	10.776	13.135
23	0.3677	10.376	9.087	8.585	14.304	17.846
23	0.3687	8.498	7.994	9.979	10.415	6.554
23	0.3697	3.907	2.203	3.987	13.990	4.829
23	0.3707	8.823	11.269	11.407	9.981	6.212
23	0.3717	7.361	9.905	7.679	15.600	6.848
23	0.3727	9.958	12.107	12.482	17.414	17.402
23	0.3737	9.449	9.442	14.039	11.339	12.182
23	0.3747	6.850	8.319	7.607	14.351	15.971
23	0.3757	8.665	8.172	9.062	16.787	8.876
23	0.3767	10.576	9.333	8.971	13.226	4.674
23	0.3777	13.341	9.259	18.576	13.847	11.727
23	0.3787	8.397	10.523	10.166	9.460	14.909
23	0.3797	6.937	7.787	9.204	5.201	13.466
23	0.3807	12.043	16.585	13.440	16.159	13.762
23	0.3817	11.119	18.927	21.279	12.241	15.355
23	0.3827	5.840	12.817	9.802	15.631	7.614
23	0.3837	14.204	19.902	21.106	14.252	12.713
23	0.3847	15.181	13.027	15.608	8.871	14.568
23	0.3857	5.329	4.370	6.649	12.570	12.744
23	0.3867	5.553	6.394	6.577	6.566	6.929
23	0.3877	16.809	14.245	23.112	11.094	16.550
23	0.3887	13.027	11.000	7.004	13.697	25.889
23	0.3897	14.451	11.679	12.054	16.625	14.609
23	0.3907	17.237	16.324	11.671	14.044	20.310
23	0.3917	16.595	11.251	13.732	17.994	18.351
23	0.3927	11.960	13.008	12.900	17.716	28.072
23	0.3937	11.607	11.945	8.497	5.982	12.076
23	0.3947	8.793	10.624	7.336	7.127	12.638
23	0.3957	8.542	9.007	7.202	4.245	17.095
23	0.3967	9.407	9.056	9.207	14.358	15.777
23	0.3977	11.666	10.218	10.229	24.673	21.299
23	0.3987	15.777	16.445	15.575	20.738	19.170
23	0.3997	9.125	9.555	8.949	22.544	10.159
23	0.4007	7.283	11.524	8.058	30.087	9.895
23	0.4017	4.488	3.833	2.984	23.718	10.437
23	0.4027	11.266	10.440	14.405	14.429	25.161
23	0.4037	6.064	8.375	10.553	8.378	25.625
23	0.4047	12.167	14.176	15.528	12.731	30.500
23	0.4057	10.888	14.335	15.188	12.483	14.563
23	0.4067	8.237	13.537	14.726	10.995	6.998
23	0.4077	7.434	16.991	7.457	19.222	17.577
23	0.4087	19.479	9.014	15.181	15.372	19.151
23	0.4097	9.355	13.226	12.195	15.121	8.612
23	0.4107	7.659	14.380	12.844	22.961	12.929
23	0.4117	14.585	18.087	16.999	15.706	18.588
23	0.4127	10.871	16.078	10.671	8.525	10.869
23	0.4137	9.274	10.070	10.805	10.434	14.831
23	0.4147	13.999	12.998	15.139	9.261	19.781

23	0.4157	20.442	15.041	19.654	8.598	16.382
23	0.4167	18.493	22.829	23.104	13.014	14.815
23	0.4177	18.462	19.117	25.368	13.632	17.252
23	0.4187	14.297	10.498	16.610	14.070	13.154
23	0.4197	9.900	9.548	12.898	11.331	19.521
23	0.4207	6.566	7.524	10.558	7.883	21.918
23	0.4217	8.469	14.990	12.894	9.803	12.594
23	0.4227	12.642	14.934	18.760	8.386	10.960
23	0.4237	7.000	12.665	13.212	14.628	13.450
23	0.4247	14.309	16.836	23.801	6.236	7.279
23	0.4257	13.379	12.169	19.083	9.117	12.770
23	0.4267	16.277	11.639	18.188	8.540	12.019
23	0.4277	13.894	17.951	21.422	8.565	13.249
23	0.4287	10.015	9.783	14.351	10.895	11.852
23	0.4297	10.383	8.101	11.723	9.404	11.693
23	0.4307	10.427	9.938	10.066	13.783	20.768
23	0.4317	17.777	17.109	14.713	4.795	16.349
23	0.4327	14.278	17.002	16.613	12.083	23.476
23	0.4337	17.145	20.930	23.615	12.618	19.203
23	0.4347	10.090	19.289	10.096	14.490	13.812
23	0.4357	18.010	18.969	15.495	8.300	27.383
23	0.4367	20.338	17.607	18.357	11.920	19.049
23	0.4377	21.002	17.753	17.624	10.729	31.326
23	0.4387	21.316	19.870	20.777	22.154	26.721
23	0.4397	22.866	25.370	28.844	15.648	22.694
23	0.4407	14.363	13.765	14.069	18.441	16.784
23	0.4417	12.596	13.385	11.966	12.615	9.286
23	0.4427	11.337	16.352	19.053	13.136	10.246
23	0.4437	13.336	16.731	18.304	11.954	12.693
23	0.4447	10.110	15.268	15.917	7.066	7.504
23	0.4457	17.773	14.342	16.386	6.029	9.716
23	0.4467	14.462	8.911	8.475	10.790	15.735
23	0.4477	9.411	8.789	9.309	16.541	23.039
23	0.4487	11.388	7.773	12.103	13.220	13.333
23	0.4497	15.608	19.592	16.918	13.760	13.883
23	0.4507	19.520	18.935	21.537	12.676	14.571
23	0.4517	19.524	17.262	18.593	11.262	22.895
23	0.4527	16.606	15.056	12.243	8.169	19.865
23	0.4537	11.018	15.029	16.445	24.459	20.715
23	0.4547	9.445	12.090	10.122	10.999	11.149
23	0.4557	15.123	12.629	13.262	11.294	5.520
23	0.4567	19.746	21.434	21.158	19.124	10.441
23	0.4577	23.360	23.260	22.434	14.444	15.348
23	0.4587	19.829	15.877	15.227	8.653	13.922
23	0.4597	15.039	13.253	17.686	9.940	9.507
23	0.4607	12.189	15.907	20.214	11.045	10.329
23	0.4617	16.356	13.449	14.902	12.919	15.363
23	0.4627	10.884	15.397	11.355	10.739	20.154
23	0.4637	13.681	17.032	14.709	12.854	16.170
23	0.4647	14.595	12.519	16.710	12.761	13.660

23	0.4657	6.887	9.097	8.952	11.032	9.584
23	0.4667	17.252	15.972	17.796	6.776	6.979
23	0.4677	13.528	11.522	11.852	9.528	13.883
23	0.4687	25.396	18.724	18.684	7.895	27.518
23	0.4697	16.067	14.465	24.742	10.932	18.071
23	0.4707	17.044	13.178	25.044	12.374	18.164
23	0.4717	14.216	9.073	14.640	3.679	10.429
23	0.4727	13.650	15.223	17.817	8.826	8.933
23	0.4737	23.678	28.665	25.186	15.047	14.681
23	0.4747	11.222	10.618	13.321	9.450	16.547
23	0.4757	20.188	14.059	20.109	15.794	11.420
23	0.4767	23.417	23.705	26.338	16.701	17.474
23	0.4777	18.301	15.819	19.773	10.126	14.298
23	0.4787	25.475	22.785	11.463	11.999	13.191
23	0.4797	22.072	17.110	25.071	9.176	7.893
23	0.4807	8.140	4.481	6.135	5.779	7.939
23	0.4817	14.233	12.349	16.763	6.548	7.907
23	0.4827	11.769	18.791	18.248	12.115	12.002
23	0.4837	12.053	7.128	13.004	7.180	11.937
23	0.4847	10.600	5.590	7.612	8.467	5.058
23	0.4857	9.562	12.980	19.847	10.603	6.796
23	0.4867	4.879	6.804	11.152	8.577	12.955
23	0.4877	6.230	12.802	11.430	12.722	9.332
23	0.4887	8.760	8.915	15.202	9.610	3.304
23	0.4897	10.090	18.623	16.534	16.911	11.929
23	0.4907	10.092	18.611	16.529	16.982	12.026
23	0.4917	6.565	8.870	10.270	14.791	18.655
23	0.4927	11.131	17.216	20.843	9.761	6.429
23	0.4937	0.000	0.000	0.000	0.000	0.000
24	0.0020	0.000	0.000	0.000	0.000	0.000
24	0.0030	15.568	6.074	13.730	8.531	19.882
24	0.0040	25.640	20.560	22.515	17.256	17.440
24	0.0050	8.238	8.796	12.169	12.957	12.533
24	0.0060	17.143	20.571	19.933	17.569	11.343
24	0.0070	15.850	12.236	13.911	13.020	10.240
24	0.0080	16.691	12.320	16.056	18.273	4.493
24	0.0090	15.890	11.467	13.893	15.066	20.168
24	0.0100	13.035	10.551	17.672	9.450	18.417
24	0.0110	12.834	10.553	17.653	9.408	17.452
24	0.0120	11.391	7.311	11.411	14.820	6.136
24	0.0130	9.117	13.540	12.067	14.574	23.507
24	0.0140	8.414	12.750	12.467	13.423	13.165
24	0.0150	23.941	17.346	23.581	5.781	13.966
24	0.0160	20.844	20.801	20.506	7.761	13.589
24	0.0170	11.138	12.174	14.438	10.390	15.047
24	0.0180	17.899	13.684	13.466	13.354	18.372
24	0.0190	24.212	17.836	21.902	13.684	15.953
24	0.0200	22.014	18.653	25.725	12.449	10.218
24	0.0210	21.722	16.989	15.366	13.478	10.405
24	0.0220	12.445	11.107	6.592	7.773	9.047

24	0.0230	13.324	17.045	8.073	17.202	14.505
24	0.0240	19.832	26.443	16.714	19.445	13.800
24	0.0250	11.733	12.922	13.022	9.484	7.114
24	0.0260	19.162	10.385	15.814	13.120	8.091
24	0.0270	14.508	14.292	12.852	6.479	6.761
24	0.0280	18.881	18.996	17.222	6.975	11.013
24	0.0290	19.556	20.293	16.470	12.181	10.297
24	0.0300	19.810	15.183	16.726	19.701	15.382
24	0.0310	23.407	13.422	19.964	8.612	14.688
24	0.0320	25.024	18.495	20.126	5.355	14.546
24	0.0330	24.586	23.413	19.331	7.505	16.334
24	0.0340	15.719	9.409	9.056	8.700	5.953
24	0.0350	27.806	24.444	20.173	4.893	12.187
24	0.0360	45.262	32.863	31.125	11.728	30.325
24	0.0370	25.585	16.652	19.737	5.317	12.104
24	0.0380	27.761	22.137	25.914	8.498	28.687
24	0.0390	20.934	13.764	15.046	10.917	21.206
24	0.0400	15.038	13.897	15.591	13.767	18.149
24	0.0410	13.313	10.041	11.945	23.266	9.838
24	0.0420	15.122	16.107	19.240	6.474	20.592
24	0.0430	8.992	9.719	6.588	9.226	9.989
24	0.0440	14.398	12.724	17.216	7.925	12.426
24	0.0450	24.604	20.905	20.613	14.784	15.603
24	0.0460	37.728	28.324	27.192	16.709	15.152
24	0.0470	16.148	14.067	14.733	18.410	10.882
24	0.0480	15.537	14.325	14.655	21.417	18.719
24	0.0490	28.482	22.371	23.777	22.399	19.613
24	0.0500	39.639	21.766	32.617	19.471	28.411
24	0.0510	17.394	16.709	23.167	13.436	18.533
24	0.0520	15.468	18.712	21.027	9.262	8.938
24	0.0530	17.662	12.346	15.402	13.408	8.131
24	0.0540	16.642	15.656	17.621	19.198	10.185
24	0.0550	14.275	13.578	13.028	24.569	3.006
24	0.0560	25.243	17.599	25.079	16.106	12.382
24	0.0570	16.727	13.682	15.953	13.575	12.042
24	0.0580	16.730	15.168	14.439	11.902	10.421
24	0.0590	4.954	8.825	8.801	15.478	5.601
24	0.0600	14.549	12.528	15.018	9.170	14.652
24	0.0610	15.336	17.888	16.856	4.831	18.451
24	0.0620	18.997	11.386	12.741	18.313	10.067
24	0.0630	17.400	14.401	15.422	13.383	11.902
24	0.0640	13.042	5.039	9.057	12.897	10.460
24	0.0650	13.563	15.667	13.632	21.701	5.098
24	0.0660	21.454	22.909	22.213	18.750	17.718
24	0.0670	25.653	15.946	16.041	15.265	12.359
24	0.0680	23.667	17.838	17.488	19.869	10.547
24	0.0690	23.941	15.536	17.571	19.780	12.862
24	0.0700	14.014	9.102	10.649	17.878	17.027
24	0.0710	6.392	4.756	4.898	15.631	4.685
24	0.0720	22.598	19.661	19.572	23.506	14.566

24	0.0730	10.195	10.111	11.951	7.368	10.922
24	0.0740	23.989	15.255	20.824	8.092	9.229
24	0.0750	24.592	15.727	23.496	21.410	20.501
24	0.0760	16.938	10.859	14.772	16.438	8.016
24	0.0770	25.265	20.402	23.008	19.594	8.501
24	0.0780	14.044	10.822	10.786	16.521	9.536
24	0.0790	27.721	20.773	21.822	18.917	13.252
24	0.0800	22.820	13.835	18.036	12.351	14.056
24	0.0810	9.310	11.374	11.451	8.535	8.389
24	0.0820	15.925	18.176	15.114	15.896	13.694
24	0.0830	23.966	23.613	18.314	18.539	17.404
24	0.0840	35.646	17.241	17.753	15.166	19.773
24	0.0850	21.190	9.114	13.063	18.379	12.785
24	0.0860	18.237	4.522	11.104	18.447	14.792
24	0.0870	10.387	7.390	8.584	17.896	8.596
24	0.0880	34.730	12.997	25.640	13.467	17.144
24	0.0890	28.460	13.456	20.008	16.132	11.647
24	0.0900	20.487	18.053	18.840	20.141	27.448
24	0.0910	18.339	14.590	14.483	17.972	19.594
24	0.0920	13.921	11.376	11.177	13.910	13.043
24	0.0930	22.593	18.030	16.393	17.277	17.241
24	0.0940	11.974	17.968	16.272	26.210	17.517
24	0.0950	15.767	18.852	14.851	19.774	23.000
24	0.0960	13.104	12.617	10.779	12.741	22.476
24	0.0970	18.137	7.779	13.174	15.799	23.162
24	0.0980	16.618	3.917	11.611	12.835	9.383
24	0.0990	8.952	14.720	12.532	18.843	15.063
24	0.1000	22.023	20.842	24.737	25.187	14.162
24	0.1010	15.394	19.080	21.741	21.763	13.848
24	0.1020	10.189	13.034	7.376	21.676	16.779
24	0.1030	21.317	5.956	10.019	17.718	9.634
24	0.1040	16.556	9.879	11.123	16.564	29.925
24	0.1050	13.433	14.520	12.843	10.836	15.492
24	0.1060	6.663	2.962	4.647	13.189	21.221
24	0.1070	20.247	10.074	16.122	11.937	20.748
24	0.1080	21.372	20.281	19.863	19.413	15.087
24	0.1090	19.690	13.496	23.455	12.647	11.773
24	0.1100	12.680	12.700	14.809	17.182	3.422
24	0.1110	10.763	5.356	5.945	23.602	13.738
24	0.1120	12.694	6.043	6.803	13.639	12.966
24	0.1130	15.352	5.713	9.751	18.754	16.286
24	0.1140	18.056	10.066	13.616	16.202	17.034
24	0.1150	14.553	9.579	17.656	13.929	18.228
24	0.1160	9.623	4.442	7.589	9.717	14.478
24	0.1170	11.214	5.450	8.603	10.930	7.002
24	0.1180	23.138	9.866	12.700	15.191	27.255
24	0.1190	15.007	9.125	16.274	18.952	26.221
24	0.1200	33.242	22.177	26.950	15.599	23.806
24	0.1210	17.029	12.369	22.408	15.795	14.525
24	0.1220	9.603	4.583	12.872	13.944	20.085

24	0.1230	23.892	15.764	16.857	21.095	16.252
24	0.1240	25.617	18.311	18.086	20.386	33.922
24	0.1250	18.099	10.627	11.808	23.097	20.293
24	0.1260	12.646	6.106	7.526	28.901	23.970
24	0.1270	7.259	5.989	7.032	10.887	15.821
24	0.1280	6.144	12.045	7.208	17.950	18.180
24	0.1290	10.542	7.657	11.681	12.970	22.234
24	0.1300	14.934	3.737	7.414	23.300	23.640
24	0.1310	8.103	1.637	3.884	20.734	15.679
24	0.1320	18.381	5.196	9.780	17.979	16.153
24	0.1330	6.859	3.184	4.449	13.365	7.565
24	0.1340	12.464	7.037	7.326	18.801	21.579
24	0.1350	12.731	4.974	13.004	20.467	20.796
24	0.1360	9.881	8.202	8.180	16.399	9.192
24	0.1370	12.338	16.581	16.930	13.767	13.007
24	0.1380	13.417	13.247	13.360	9.321	11.291
24	0.1390	10.036	2.704	8.562	10.558	8.509
24	0.1400	11.299	3.886	10.095	11.061	5.493
24	0.1410	13.243	3.784	8.542	9.900	6.688
24	0.1420	16.855	14.030	16.033	14.634	13.975
24	0.1430	17.058	16.381	16.861	8.663	17.214
24	0.1440	16.610	9.038	15.903	15.662	13.871
24	0.1450	16.140	12.445	13.990	23.748	13.764
24	0.1460	22.218	19.964	19.080	19.355	16.548
24	0.1470	15.669	10.555	6.856	9.458	19.221
24	0.1480	9.982	4.766	8.927	6.525	12.341
24	0.1490	18.746	7.946	14.128	22.625	15.423
24	0.1500	35.590	16.781	21.815	17.311	16.431
24	0.1510	17.180	14.875	17.490	19.451	19.550
24	0.1520	27.312	16.105	23.876	15.829	9.735
24	0.1530	21.520	12.969	17.267	15.106	13.631
24	0.1540	23.646	27.035	18.495	9.116	21.234
24	0.1550	8.291	7.780	6.678	15.503	21.672
24	0.1560	22.128	6.180	10.835	11.087	6.737
24	0.1570	33.647	9.502	21.002	6.497	4.470
24	0.1580	12.778	7.353	8.994	7.288	13.741
24	0.1590	12.531	6.049	10.657	11.529	12.677
24	0.1600	13.568	6.658	8.393	11.201	11.003
24	0.1610	13.869	7.550	11.560	11.115	6.831
24	0.1620	12.441	11.077	16.497	17.172	18.495
24	0.1630	23.434	20.428	24.203	25.458	13.868
24	0.1640	27.704	22.338	22.984	23.237	20.499
24	0.1650	27.734	22.771	23.653	22.667	20.648
24	0.1660	17.199	15.336	17.737	12.000	10.869
24	0.1670	7.510	3.061	5.809	22.059	17.230
24	0.1680	8.678	7.894	7.715	23.173	17.279
24	0.1690	7.285	8.932	7.038	20.257	9.711
24	0.1700	6.702	12.446	7.153	13.863	9.767
24	0.1710	8.693	15.432	11.186	22.935	15.946
24	0.1720	10.228	8.984	8.329	17.283	13.038

24	0.1730	15.341	14.580	17.046	18.100	18.739
24	0.1740	13.965	8.768	15.711	16.229	18.136
24	0.1750	10.226	13.241	13.031	10.501	12.564
24	0.1760	16.488	17.978	13.002	20.466	8.778
24	0.1770	13.776	12.974	10.165	11.812	9.293
24	0.1780	18.532	19.092	16.190	11.704	22.450
24	0.1790	8.880	9.247	9.239	10.891	6.646
24	0.1800	15.630	12.333	12.076	11.271	9.543
24	0.1810	13.566	12.380	8.277	13.503	22.910
24	0.1820	19.673	20.416	17.805	14.540	28.366
24	0.1830	20.937	17.189	16.616	15.974	11.301
24	0.1840	21.347	13.048	15.045	13.530	17.098
24	0.1850	15.985	13.100	14.882	19.885	17.780
24	0.1860	14.142	12.154	15.140	14.329	11.264
24	0.1870	23.685	15.668	23.183	14.014	19.203
24	0.1880	10.408	6.913	4.473	19.387	21.392
24	0.1890	16.499	8.138	10.416	22.354	15.530
24	0.1900	5.855	6.991	3.597	18.433	13.553
24	0.1910	12.356	13.556	11.286	14.934	11.316
24	0.1920	13.399	14.245	11.680	14.231	11.862
24	0.1930	8.614	8.650	6.367	15.673	10.671
24	0.1940	10.216	7.517	9.477	19.729	12.479
24	0.1950	31.073	18.953	25.606	15.580	17.001
24	0.1960	5.441	4.950	9.618	15.317	9.493
24	0.1970	10.110	4.614	7.002	10.977	12.975
24	0.1980	21.641	12.617	13.206	14.915	19.814
24	0.1990	18.514	11.447	11.873	18.265	13.088
24	0.2000	2.858	6.367	2.039	11.794	13.094
24	0.2010	4.130	5.956	4.178	12.469	12.788
24	0.2020	5.602	5.700	5.587	11.303	14.122
24	0.2030	27.614	20.587	21.921	10.795	16.073
24	0.2040	15.809	14.567	13.787	5.429	5.020
24	0.2050	11.901	11.195	8.767	15.015	19.802
24	0.2060	8.663	12.303	9.575	13.265	19.588
24	0.2070	10.786	17.451	8.436	21.408	28.995
24	0.2080	17.808	16.030	12.469	15.867	15.339
24	0.2090	16.193	18.023	18.341	13.171	10.506
24	0.2100	12.724	18.328	13.794	17.484	16.908
24	0.2110	8.696	7.363	4.371	12.473	9.626
24	0.2120	13.208	13.338	7.949	21.843	12.034
24	0.2130	5.309	12.816	5.254	24.473	8.718
24	0.2140	18.397	27.818	14.488	21.045	14.475
24	0.2150	9.351	12.727	6.993	10.189	13.210
24	0.2160	22.510	22.078	23.153	13.957	18.109
24	0.2170	15.513	24.418	19.746	24.856	23.624
24	0.2180	9.294	16.058	12.158	18.283	13.963
24	0.2190	9.927	10.614	9.825	9.569	15.282
24	0.2200	12.467	9.605	8.506	19.645	11.646
24	0.2210	12.695	12.378	12.671	18.117	12.767
24	0.2220	13.040	13.977	11.111	11.463	9.607

24	0.2230	18.764	23.953	17.685	15.691	17.228
24	0.2240	14.972	15.293	22.445	10.624	19.864
24	0.2250	14.738	15.173	22.267	10.577	19.671
24	0.2260	15.496	13.305	13.172	12.461	23.030
24	0.2270	14.745	13.029	8.231	13.850	9.643
24	0.2280	8.192	17.659	9.069	20.375	9.402
24	0.2290	11.486	9.655	9.009	10.557	11.176
24	0.2300	15.294	11.057	9.828	10.621	19.771
24	0.2310	8.905	12.658	7.349	18.341	14.369
24	0.2320	15.467	18.736	9.584	17.419	17.400
24	0.2330	14.364	11.164	4.986	10.298	25.367
24	0.2340	9.529	11.397	8.672	9.969	17.555
24	0.2350	11.778	14.841	11.821	12.781	20.608
24	0.2360	9.531	12.461	7.041	10.710	15.518
24	0.2370	24.034	10.455	16.732	7.179	16.441
24	0.2380	25.084	15.535	18.709	15.252	19.582
24	0.2390	13.150	9.716	8.802	10.257	22.050
24	0.2400	15.194	12.703	5.699	14.102	17.704
24	0.2410	14.093	15.772	11.763	8.702	16.732
24	0.2420	15.104	13.846	12.814	18.824	19.986
24	0.2430	15.679	14.110	13.072	18.783	19.979
24	0.2440	4.868	10.268	3.944	12.466	8.930
24	0.2450	12.449	10.655	10.217	13.425	21.241
24	0.2460	11.791	18.436	8.693	11.700	20.470
24	0.2470	6.204	5.562	5.443	9.106	21.262
24	0.2480	6.983	10.629	8.914	14.982	28.272
24	0.2490	9.821	9.525	10.170	16.189	19.531
24	0.2500	15.380	15.960	12.280	11.410	16.825
24	0.2510	12.262	6.774	7.661	6.513	26.469
24	0.2520	7.564	6.382	4.392	6.970	8.694
24	0.2530	5.410	6.420	5.238	13.655	3.750
24	0.2540	6.598	5.795	5.932	8.072	11.288
24	0.2550	11.900	13.738	10.231	11.070	18.443
24	0.2560	13.801	20.329	19.471	9.096	21.109
24	0.2570	6.349	15.489	13.305	13.177	21.073
24	0.2580	10.406	10.712	14.621	9.745	19.885
24	0.2590	16.536	11.383	18.119	17.052	14.934
24	0.2600	8.083	5.055	6.380	22.130	26.181
24	0.2610	5.791	1.487	3.202	9.649	14.385
24	0.2620	9.168	12.841	7.892	16.739	25.936
24	0.2630	10.853	8.329	5.480	15.894	17.881
24	0.2640	11.118	8.900	5.351	16.773	17.746
24	0.2650	15.183	13.627	10.924	18.724	30.401
24	0.2660	16.333	19.108	16.910	18.562	16.018
24	0.2670	14.532	17.552	15.532	18.662	14.661
24	0.2680	16.369	9.007	7.321	16.775	19.567
24	0.2690	16.308	8.324	7.330	13.299	19.118
24	0.2700	16.518	21.074	11.039	20.400	22.420
24	0.2710	12.825	20.236	6.970	17.832	25.270
24	0.2720	22.239	25.994	21.210	21.384	35.791

24	0.2730	15.526	11.649	11.560	24.209	14.554
24	0.2740	23.367	14.985	21.681	19.018	16.842
24	0.2750	11.534	9.683	12.314	19.174	19.547
24	0.2760	11.179	8.748	12.330	18.756	19.173
24	0.2770	11.266	10.579	14.910	16.525	15.848
24	0.2780	21.498	16.263	12.941	20.232	18.968
24	0.2790	18.104	18.239	11.827	8.311	19.978
24	0.2800	24.822	25.331	19.983	18.407	21.197
24	0.2810	16.078	16.239	21.517	7.195	21.421
24	0.2820	17.172	17.226	19.273	17.985	23.273
24	0.2830	17.416	14.291	13.307	15.913	28.202
24	0.2840	15.762	17.122	10.086	17.238	20.639
24	0.2850	17.288	24.527	16.158	19.274	15.027
24	0.2860	17.324	24.687	16.098	20.037	15.065
24	0.2870	10.322	10.483	10.881	18.308	19.108
24	0.2880	7.977	10.228	5.848	16.816	18.149
24	0.2890	28.185	13.595	20.106	14.946	14.953
24	0.2900	16.893	17.895	11.655	15.795	19.318
24	0.2910	12.343	13.233	11.700	15.243	24.438
24	0.2920	19.101	8.136	12.598	13.840	18.155
24	0.2930	15.775	18.678	11.432	20.209	15.984
24	0.2940	11.421	8.304	7.293	14.405	8.466
24	0.2950	7.722	10.228	6.892	9.873	10.856
24	0.2960	19.028	17.735	10.385	12.682	24.921
24	0.2970	14.542	7.586	7.021	10.960	22.916
24	0.2980	13.637	9.849	3.528	15.979	20.619
24	0.2990	9.852	6.224	3.313	13.805	27.473
24	0.3000	18.400	6.362	10.030	16.053	25.033
24	0.3010	13.138	10.595	10.826	18.016	27.860
24	0.3020	23.289	22.658	15.408	21.051	24.679
24	0.3030	15.025	12.371	9.047	13.773	30.653
24	0.3040	10.787	11.357	7.552	8.880	23.847
24	0.3050	12.077	15.905	11.038	16.090	26.785
24	0.3060	13.425	8.885	9.838	11.592	26.896
24	0.3070	8.331	11.364	5.660	9.151	11.897
24	0.3080	15.345	9.745	13.553	12.249	13.072
24	0.3090	24.579	15.136	18.184	19.133	16.617
24	0.3100	21.354	11.420	10.620	21.682	30.437
24	0.3110	22.157	11.540	11.956	19.435	27.539
24	0.3120	23.825	16.443	15.995	13.639	28.590
24	0.3130	26.228	13.711	20.408	12.973	23.141
24	0.3140	26.650	15.616	17.380	12.526	21.820
24	0.3150	16.489	8.329	6.414	12.200	30.451
24	0.3160	18.740	10.062	5.461	19.053	29.554
24	0.3170	24.675	10.946	7.922	13.038	31.899
24	0.3180	22.116	9.086	14.152	18.413	35.251
24	0.3190	10.461	14.233	11.808	18.506	33.026
24	0.3200	7.096	4.679	7.285	9.665	13.805
24	0.3210	13.328	12.862	14.305	10.446	13.611
24	0.3220	19.764	8.968	9.123	15.717	31.407

24	0.3230	22.098	24.694	23.069	13.301	13.808
24	0.3240	11.753	12.328	10.472	14.434	31.171
24	0.3250	16.792	16.445	16.384	12.445	26.751
24	0.3260	18.762	17.514	19.225	11.772	35.195
24	0.3270	11.081	15.062	11.864	15.778	39.146
24	0.3280	14.786	16.558	14.166	14.731	30.355
24	0.3290	13.883	13.503	14.933	20.765	17.655
24	0.3300	13.026	11.128	13.061	19.130	18.058
24	0.3310	21.757	13.625	10.600	21.813	31.427
24	0.3320	23.118	16.611	17.985	13.505	30.101
24	0.3330	17.202	13.284	16.898	31.156	25.088
24	0.3340	15.113	8.480	12.700	23.674	29.582
24	0.3350	18.546	13.345	11.749	21.176	24.814
24	0.3360	26.210	13.757	14.494	3.630	22.633
24	0.3370	2.548	2.913	0.561	8.684	10.513
24	0.3380	18.165	10.547	8.202	19.987	34.854
24	0.3390	18.453	9.834	13.820	12.625	22.972
24	0.3400	11.844	4.998	7.770	15.279	17.161
24	0.3410	15.241	11.610	6.604	15.147	28.974
24	0.3420	22.206	16.364	13.293	20.264	28.633
24	0.3430	35.233	20.607	22.296	18.724	24.634
24	0.3440	31.294	11.640	20.650	17.807	19.582
24	0.3450	23.010	21.883	19.540	14.192	24.076
24	0.3460	19.804	18.848	13.897	15.698	19.066
24	0.3470	6.892	6.575	7.579	7.506	10.145
24	0.3480	6.798	10.275	8.037	11.774	18.457
24	0.3490	9.646	9.289	10.465	14.757	22.238
24	0.3500	15.169	10.171	10.753	15.382	34.144
24	0.3510	7.701	9.627	5.995	15.295	10.697
24	0.3520	16.366	12.310	6.818	14.835	32.741
24	0.3530	10.854	12.273	4.256	20.977	20.053
24	0.3540	17.499	15.627	17.633	26.444	21.324
24	0.3550	18.013	11.265	13.703	28.461	23.314
24	0.3560	23.972	11.506	14.683	22.263	28.539
24	0.3570	25.238	12.028	15.293	22.346	29.057
24	0.3580	20.793	14.291	17.210	17.001	26.170
24	0.3590	21.433	12.625	19.457	14.321	36.384
24	0.3600	8.466	7.290	6.978	24.447	40.517
24	0.3610	12.939	10.606	10.196	16.464	25.496
24	0.3620	13.908	13.744	10.913	17.723	25.775
24	0.3630	10.503	9.004	9.322	17.430	28.119
24	0.3640	14.670	8.710	10.112	23.782	21.280
24	0.3650	15.976	4.751	9.610	19.725	21.513
24	0.3660	15.927	5.185	10.687	20.565	26.673
24	0.3670	15.801	13.281	10.560	22.052	35.771
24	0.3680	15.567	15.192	13.245	20.120	38.390
24	0.3690	13.957	12.533	14.912	15.110	18.068
24	0.3700	16.748	9.878	14.051	22.009	19.215
24	0.3710	14.053	9.111	16.897	24.558	21.953
24	0.3720	8.991	6.782	11.487	25.907	15.533

24	0.3730	14.452	9.967	13.501	27.289	20.313
24	0.3740	36.069	11.159	16.452	30.266	22.942
24	0.3750	13.434	9.888	11.581	21.944	22.181
24	0.3760	20.767	12.340	18.531	27.696	23.882
24	0.3770	8.056	7.133	8.930	22.321	37.252
24	0.3780	13.723	9.122	9.863	12.153	33.227
24	0.3790	18.866	12.666	13.662	23.831	20.335
24	0.3800	29.012	16.824	20.781	32.073	22.160
24	0.3810	25.151	26.681	21.379	35.664	24.958
24	0.3820	25.113	24.532	20.682	35.817	37.306
24	0.3830	19.121	12.260	9.552	28.674	28.141
24	0.3840	21.121	12.632	11.741	28.415	27.580
24	0.3850	29.114	23.730	16.335	26.207	23.185
24	0.3860	17.290	11.332	11.298	25.069	15.466
24	0.3870	19.724	16.017	19.424	31.882	32.119
24	0.3880	21.035	18.388	13.452	28.639	27.526
24	0.3890	21.528	16.371	12.267	29.333	32.882
24	0.3900	18.830	20.130	12.930	24.547	29.933
24	0.3910	23.275	20.711	18.809	25.086	18.438
24	0.3920	14.483	13.803	12.204	27.975	19.121
24	0.3930	14.516	17.787	10.359	30.931	28.047
24	0.3940	33.010	37.874	33.616	33.735	24.804
24	0.3950	14.790	16.065	9.929	28.751	42.217
24	0.3960	36.802	27.336	30.916	19.842	35.780
24	0.3970	34.583	21.015	19.643	21.967	25.800
24	0.3980	30.250	23.199	23.370	18.272	9.788
24	0.3990	19.641	16.628	17.952	16.640	18.357
24	0.4000	20.990	15.630	15.937	23.111	21.303
24	0.4010	22.810	9.070	14.694	18.924	19.608
24	0.4020	19.594	16.503	20.140	15.465	23.187
24	0.4030	24.431	19.466	21.487	17.676	22.143
24	0.4040	18.537	18.874	18.072	29.323	17.926
24	0.4050	10.782	12.743	9.956	24.144	14.140
24	0.4060	18.522	15.928	15.491	27.461	21.804
24	0.4070	7.620	9.650	9.938	16.937	12.984
24	0.4080	14.672	6.308	9.310	15.868	19.621
24	0.4090	11.664	5.845	6.256	15.976	16.179
24	0.4100	13.081	11.404	8.592	21.409	18.674
24	0.4110	12.698	7.969	11.050	12.308	21.112
24	0.4120	28.319	31.624	28.650	21.530	18.755
24	0.4130	20.974	21.047	18.594	28.564	14.175
24	0.4140	10.666	5.935	5.566	23.861	10.266
24	0.4150	13.964	9.782	6.345	17.216	19.947
24	0.4160	7.220	2.641	5.524	17.708	8.781
24	0.4170	7.781	2.661	5.759	31.092	10.488
24	0.4180	5.564	2.965	3.594	7.283	5.694
24	0.4190	12.055	14.400	15.678	27.352	4.475
24	0.4200	20.908	21.029	18.814	17.580	5.509
24	0.4210	11.552	12.889	15.383	14.018	6.738
24	0.4220	20.070	12.686	16.674	13.077	8.662

24	0.4230	13.462	13.536	17.699	27.594	5.673
24	0.4240	29.720	14.873	27.085	28.388	14.540
24	0.4250	29.190	14.806	26.700	28.175	14.499
24	0.4260	21.051	9.703	22.786	28.809	14.292
24	0.4270	11.601	5.411	11.864	17.401	8.473
24	0.4280	5.716	3.127	7.515	18.753	2.901
24	0.4290	13.824	8.452	11.413	11.008	7.343
24	0.4300	4.335	4.206	2.455	5.599	4.691
24	0.4310	0.973	2.408	3.148	2.889	3.117
24	0.4320	1.412	0.554	0.259	2.296	4.101
24	0.4330	8.975	6.872	8.609	4.312	7.126
24	0.4340	6.766	5.913	6.360	11.180	5.756
24	0.4350	5.892	4.822	6.131	6.809	2.251
24	0.4360	9.794	10.501	8.449	12.837	12.893
24	0.4370	6.275	23.307	12.050	36.865	14.034
24	0.4380	12.424	16.307	14.675	31.175	15.702
24	0.4390	8.469	23.594	12.015	35.322	10.261
24	0.4400	13.652	23.646	18.304	38.735	11.712
24	0.4410	13.036	21.632	22.866	30.794	12.242
24	0.4420	12.079	17.209	20.517	36.026	12.378
24	0.4430	13.582	27.001	19.594	41.275	12.533
24	0.4440	27.129	13.071	15.637	21.157	13.428
24	0.4450	16.654	8.691	12.067	22.095	10.007
24	0.4460	9.817	10.621	7.167	31.376	12.293
24	0.4470	9.982	12.405	11.008	28.981	10.732
24	0.4480	10.266	8.490	12.159	16.887	11.301
24	0.4490	8.994	10.422	6.766	13.591	9.790
24	0.4500	16.485	18.408	20.204	14.725	10.971
24	0.4510	23.137	30.496	27.523	28.320	10.665
24	0.4520	17.991	20.098	15.290	35.670	7.304
24	0.4530	7.682	9.450	12.664	27.157	10.619
24	0.4540	11.038	19.766	13.329	34.541	11.573
24	0.4550	10.778	17.799	15.356	27.402	12.030
24	0.4560	11.607	13.912	9.758	18.536	9.927
24	0.4570	17.137	13.868	14.783	17.939	8.644
24	0.4580	18.382	13.420	21.097	12.104	10.825
24	0.4590	22.056	19.550	24.962	18.854	12.703
24	0.4600	9.148	11.807	8.683	18.814	10.424
24	0.4610	15.656	25.652	17.067	27.986	16.960
24	0.4620	16.550	29.573	17.337	19.713	19.165
24	0.4630	14.556	18.039	16.708	33.916	18.488
24	0.4640	9.702	11.460	9.859	13.062	7.902
24	0.4650	10.908	10.102	10.423	12.619	9.445
24	0.4660	20.442	21.707	25.189	15.697	12.581
24	0.4670	8.476	12.473	5.989	23.836	13.285
24	0.4680	16.621	19.572	14.370	24.045	19.964
24	0.4690	22.445	22.506	22.975	21.974	6.551
24	0.4700	9.183	14.036	12.442	13.128	10.950
24	0.4710	7.929	9.482	5.119	22.335	18.748
24	0.4720	12.088	9.507	13.413	7.450	10.874

24	0.4730	20.785	19.082	18.533	17.610	10.858
24	0.4740	17.280	26.442	19.896	23.103	5.087
24	0.4750	16.246	12.570	15.612	8.983	3.971
24	0.4760	20.734	16.633	15.226	12.922	9.251
24	0.4770	21.336	18.955	16.519	19.978	13.413
24	0.4780	22.550	30.313	24.520	21.252	10.856
24	0.4790	15.793	24.191	19.618	23.173	9.772
24	0.4800	20.587	18.833	18.731	22.265	8.989
24	0.4810	20.613	21.508	20.239	19.193	11.193
24	0.4820	26.000	27.977	24.250	18.692	7.640
24	0.4830	14.665	18.609	19.305	16.090	7.588
24	0.4840	25.074	17.643	29.450	22.906	12.163
24	0.4850	16.459	22.831	15.922	37.659	7.090
24	0.4860	19.537	22.271	16.184	19.147	4.757
24	0.4870	21.341	28.279	23.247	26.690	11.303
24	0.4880	10.261	15.457	9.772	18.753	7.780
24	0.4890	4.126	8.593	5.477	15.772	5.624
24	0.4900	11.374	14.026	14.366	17.384	5.501
24	0.4910	16.907	21.389	19.434	20.445	5.604
24	0.4920	11.624	20.883	14.028	30.863	6.975
24	0.4930	15.491	21.550	18.035	26.428	12.498
24	0.4940	15.895	18.482	14.089	33.099	5.453
24	0.4950	13.242	18.547	12.253	27.700	8.030
24	0.4960	22.040	22.900	15.141	19.189	14.262
24	0.4970	13.250	13.752	11.817	11.596	4.891
24	0.4980	13.283	20.000	16.677	19.835	6.283
24	0.4990	24.259	29.540	24.734	20.516	11.188
24	0.5000	23.799	18.920	18.309	19.252	15.172
24	0.5010	16.188	16.600	14.106	22.665	17.311
24	0.5020	14.902	21.560	12.901	25.607	16.268
24	0.5030	17.281	25.199	18.978	29.809	18.041
24	0.5040	22.029	20.171	21.492	28.361	15.212
24	0.5050	19.275	18.534	16.493	25.591	8.596
24	0.5060	7.572	11.034	11.996	21.824	10.133
24	0.5070	11.510	15.442	10.022	21.279	15.999
24	0.5080	25.044	24.239	16.635	14.709	17.490
24	0.5090	23.606	22.360	17.356	20.488	12.219
24	0.5100	33.560	32.509	34.796	27.773	19.481
24	0.5110	34.066	22.695	30.938	18.725	26.175
24	0.5120	13.465	11.382	14.527	14.511	21.656
24	0.5130	27.521	15.723	16.429	27.744	28.239
24	0.5140	21.582	22.565	20.977	28.440	9.868
24	0.5150	14.568	14.568	12.485	12.994	8.256
24	0.5160	14.613	8.949	8.855	7.571	10.850
24	0.5170	10.639	14.369	7.411	17.996	16.778
24	0.5180	21.446	15.387	19.572	15.555	18.521
24	0.5190	15.794	21.802	15.192	18.641	17.930
24	0.5200	18.172	21.233	19.930	11.847	14.377
24	0.5210	26.394	25.895	27.658	14.068	14.346
24	0.5220	10.694	15.282	15.456	14.504	8.339

24	0.5230	16.515	18.210	21.629	13.700	9.086
24	0.5240	26.686	29.532	31.956	13.632	17.926
24	0.5250	21.257	26.004	21.341	11.462	16.610
24	0.5260	11.890	16.217	16.866	16.691	8.465
24	0.5270	16.246	18.416	19.813	21.925	13.454
24	0.5280	20.309	21.321	25.166	13.177	16.905
24	0.5290	12.868	13.246	18.329	25.956	18.695
24	0.5300	19.114	21.464	29.203	23.651	19.186
24	0.5310	17.395	16.208	16.364	10.536	22.369
24	0.5320	15.157	11.346	16.295	9.313	13.282
24	0.5330	24.429	17.077	22.032	19.785	18.324
24	0.5340	15.289	20.218	17.870	14.703	10.460
24	0.5350	15.582	20.387	18.382	13.680	10.559
24	0.5360	20.874	18.305	21.776	8.871	28.977
24	0.5370	24.993	15.092	16.756	9.008	23.462
24	0.5380	25.016	15.112	16.806	8.971	23.499
24	0.5390	27.791	17.468	22.330	8.743	12.370
24	0.5400	13.214	11.723	15.781	14.600	14.364
24	0.5410	23.424	16.052	29.341	14.194	18.752
24	0.5420	13.836	12.194	18.078	10.346	10.335
24	0.5430	28.697	20.407	29.201	13.075	20.310
24	0.5440	22.837	12.917	24.444	14.435	26.328
24	0.5450	10.670	14.046	17.612	7.463	21.057
24	0.5460	18.051	8.974	18.931	12.409	12.056
24	0.5470	18.637	11.744	21.416	11.081	7.051
24	0.5480	20.994	10.854	23.577	13.825	12.653
24	0.5490	14.267	10.009	18.076	7.987	10.270
24	0.5500	29.887	19.183	32.239	12.596	10.216
24	0.5510	28.508	20.662	38.443	9.874	16.077
24	0.5520	17.706	14.439	29.202	10.912	15.854
24	0.5530	23.565	15.120	24.715	15.707	19.408
24	0.5540	17.950	19.408	19.855	8.950	9.832
24	0.5550	23.751	13.709	18.988	9.085	17.999
24	0.5560	13.413	14.645	11.948	15.982	21.430
24	0.5570	19.475	7.648	14.215	21.551	11.187
24	0.5580	17.958	16.091	19.516	12.876	18.893
24	0.5590	32.261	22.753	28.519	8.275	16.629
24	0.5600	18.829	8.694	19.703	10.360	9.958
24	0.5610	17.533	6.663	12.728	11.123	9.704
24	0.5620	14.325	16.329	17.177	13.408	17.537
24	0.5630	11.928	19.727	18.724	22.111	14.189
24	0.5640	19.509	12.355	15.312	5.342	19.809
24	0.5650	29.667	18.797	19.918	22.437	20.383
24	0.5660	20.596	22.591	16.782	23.765	21.095
24	0.5670	18.629	11.674	15.798	19.397	27.401
24	0.5680	18.819	18.797	16.757	15.980	20.300
24	0.5690	11.057	11.982	20.990	15.198	17.248
24	0.5700	17.045	14.356	20.691	24.247	18.076
24	0.5710	28.264	22.650	25.543	14.939	28.149
24	0.5720	7.576	13.955	13.861	14.826	23.856

24	0.5730	6.250	4.520	7.757	14.699	14.204
24	0.5740	12.847	18.561	16.851	14.954	17.016
24	0.5750	12.261	15.917	21.584	13.853	13.849
24	0.5760	10.856	15.640	21.291	13.758	11.291
24	0.5770	19.998	11.040	25.094	16.832	19.116
24	0.5780	15.260	11.075	18.305	17.991	18.050
24	0.5790	16.022	23.342	23.421	13.549	18.967
24	0.5800	25.594	19.085	24.917	20.134	20.526
24	0.5810	27.243	25.310	27.406	24.984	28.370
24	0.5820	35.600	30.846	29.640	12.831	27.426
24	0.5830	20.678	23.485	17.424	14.361	13.259
24	0.5840	31.519	29.457	28.795	21.654	21.215
24	0.5850	26.464	18.447	25.259	13.490	18.663
24	0.5860	16.045	12.130	16.294	20.243	19.807
24	0.5870	12.593	11.244	19.526	16.068	24.310
24	0.5880	24.082	21.674	24.435	17.324	35.088
24	0.5890	15.043	14.236	16.720	14.758	24.591
24	0.5900	13.026	10.358	13.787	12.910	23.970
24	0.5910	32.252	21.796	35.406	13.228	26.442
24	0.5920	21.222	8.514	21.259	10.194	17.522
24	0.5930	25.336	9.578	18.804	18.066	13.396
24	0.5940	18.431	19.686	19.165	24.619	21.617
24	0.5950	15.992	25.288	19.877	23.060	6.607
24	0.5960	19.537	22.588	24.817	21.599	17.719
24	0.5970	16.225	20.276	24.620	15.305	15.909
24	0.5980	19.227	11.164	19.985	11.659	15.999
24	0.5990	23.972	21.043	35.614	15.932	14.253
24	0.6000	21.297	24.877	29.887	21.764	16.011
24	0.6010	14.906	21.108	17.903	14.691	16.107
24	0.6020	23.554	13.267	24.368	21.171	14.868
24	0.6030	30.246	21.439	27.553	23.969	20.495
24	0.6040	26.783	16.021	23.162	14.566	11.620
24	0.6050	21.941	17.669	25.227	13.232	23.243
24	0.6060	12.996	9.080	9.433	4.878	7.770
24	0.6070	10.384	6.152	5.754	6.572	3.893
24	0.6080	9.870	4.768	7.070	5.835	4.273
24	0.6090	11.764	15.788	14.291	16.857	17.060
24	0.6100	14.718	10.797	17.413	15.570	14.466
24	0.6110	16.019	12.505	14.978	11.337	14.199
24	0.6120	11.236	14.683	17.654	19.294	14.371
24	0.6130	11.980	17.463	19.896	16.377	11.174
24	0.6140	12.237	5.988	11.108	15.888	21.849
24	0.6150	7.406	13.778	18.305	13.598	21.779
24	0.6160	0.883	0.993	1.043	1.168	1.314
24	0.6170	0.000	0.000	0.000	0.000	0.000
25	0.0008	0.000	0.000	0.000	0.000	0.000
25	0.0018	12.861	7.505	12.348	15.620	16.776
25	0.0028	7.121	8.740	11.970	4.878	20.293
25	0.0038	7.623	14.465	11.880	12.244	29.155
25	0.0048	8.968	14.034	15.156	20.245	23.101

25	0.0058	18.029	19.554	21.667	10.121	20.808
25	0.0068	11.904	15.486	15.273	13.613	14.617
25	0.0078	19.368	19.018	25.290	9.756	18.856
25	0.0088	18.772	20.364	18.140	11.516	11.166
25	0.0098	17.129	11.596	18.629	12.564	13.764
25	0.0108	16.828	13.127	18.620	22.154	25.903
25	0.0118	10.586	11.572	20.763	16.929	22.240
25	0.0128	10.949	14.520	15.212	9.642	22.695
25	0.0138	16.306	19.927	12.516	17.819	38.852
25	0.0148	6.046	6.473	9.961	9.041	17.059
25	0.0158	10.769	18.232	17.194	9.005	7.049
25	0.0168	10.933	16.702	21.136	8.956	14.776
25	0.0178	19.100	22.223	28.010	12.516	22.445
25	0.0188	27.544	27.435	33.244	11.083	33.173
25	0.0198	19.229	19.777	21.602	6.994	16.525
25	0.0208	8.666	11.137	16.491	11.070	16.889
25	0.0218	14.516	9.208	18.467	17.853	43.327
25	0.0228	14.754	14.063	17.842	14.458	20.474
25	0.0238	14.026	21.530	23.297	15.647	21.675
25	0.0248	12.614	17.534	15.395	12.697	28.042
25	0.0258	12.620	17.547	15.379	12.701	28.169
25	0.0268	10.576	14.103	20.781	7.041	30.539
25	0.0278	16.550	21.925	18.348	5.995	25.370
25	0.0288	15.509	21.707	16.525	23.133	17.139
25	0.0298	9.377	13.275	16.604	18.700	21.700
25	0.0308	8.106	8.732	8.374	10.628	12.624
25	0.0318	6.238	5.919	6.252	7.254	10.270
25	0.0328	5.146	2.263	2.562	12.830	12.584
25	0.0338	10.610	16.221	14.189	14.248	19.064
25	0.0348	12.745	11.526	9.029	11.544	34.203
25	0.0358	15.127	7.677	8.664	10.944	30.001
25	0.0368	5.850	3.976	5.860	15.806	23.778
25	0.0378	14.201	9.580	19.590	14.566	23.384
25	0.0388	10.913	12.477	19.527	13.023	32.110
25	0.0398	9.455	6.912	10.122	14.936	27.698
25	0.0408	8.720	2.698	10.892	18.370	15.523
25	0.0418	8.477	8.839	17.019	10.708	17.047
25	0.0428	14.371	9.930	19.784	12.344	22.910
25	0.0438	10.263	10.963	4.875	22.693	21.171
25	0.0448	10.193	10.818	4.861	22.792	21.140
25	0.0458	9.590	8.750	8.130	22.468	21.321
25	0.0468	13.953	12.261	16.705	19.878	16.324
25	0.0478	15.018	8.845	11.021	22.719	21.281
25	0.0488	14.773	8.932	8.296	19.458	16.235
25	0.0498	12.561	16.373	12.044	21.318	14.685
25	0.0508	23.128	15.247	18.252	21.889	20.549
25	0.0518	21.020	21.342	24.234	22.961	22.061
25	0.0528	12.189	12.553	13.167	16.526	27.308
25	0.0538	18.001	12.591	15.894	14.564	15.246
25	0.0548	9.889	13.417	17.792	23.146	25.121

25	0.0558	11.116	15.289	15.948	24.808	11.054
25	0.0568	9.208	19.615	9.852	33.251	19.393
25	0.0578	9.278	15.002	10.040	15.291	12.417
25	0.0588	7.086	10.452	9.204	14.264	6.301
25	0.0598	9.832	11.668	15.321	15.943	9.944
25	0.0608	12.848	15.071	15.507	19.799	21.323
25	0.0618	15.437	22.079	20.306	27.781	12.913
25	0.0628	13.291	18.685	18.394	22.062	17.406
25	0.0638	15.432	10.447	15.915	24.147	20.785
25	0.0648	18.604	16.889	15.442	19.189	14.126
25	0.0658	13.821	7.364	11.152	15.297	16.040
25	0.0668	12.176	20.612	18.173	26.925	27.523
25	0.0678	13.643	14.230	18.466	5.680	13.646
25	0.0688	22.390	21.069	22.427	22.500	22.297
25	0.0698	13.857	19.728	19.782	24.570	24.248
25	0.0708	12.342	8.783	15.909	25.459	16.218
25	0.0718	8.357	9.571	9.696	26.219	14.528
25	0.0728	11.373	5.301	10.091	14.692	15.949
25	0.0738	9.662	10.033	11.822	18.096	15.014
25	0.0748	14.659	18.161	13.126	17.788	20.937
25	0.0758	22.827	18.333	19.802	13.011	21.095
25	0.0768	13.739	13.464	15.075	19.800	29.224
25	0.0778	16.559	23.101	16.494	15.753	24.679
25	0.0788	9.970	12.188	5.738	14.240	8.662
25	0.0798	15.979	17.081	11.448	15.013	12.755
25	0.0808	17.442	10.999	12.954	19.433	27.093
25	0.0818	8.461	14.049	16.136	18.772	16.611
25	0.0828	16.472	13.901	18.970	22.710	24.653
25	0.0838	17.216	13.288	14.853	17.441	24.949
25	0.0848	16.798	13.193	14.550	17.604	24.884
25	0.0858	15.908	17.716	15.237	15.866	17.605
25	0.0868	11.615	10.017	11.668	13.380	18.087
25	0.0878	8.762	11.891	11.493	21.971	17.013
25	0.0888	11.554	7.367	8.728	25.890	14.955
25	0.0898	13.080	6.788	6.008	19.041	27.172
25	0.0908	9.020	9.141	7.526	18.481	14.996
25	0.0918	15.783	21.520	24.566	17.326	18.965
25	0.0928	18.600	27.836	30.595	15.185	20.922
25	0.0938	17.644	22.059	21.152	26.357	17.098
25	0.0948	13.431	17.689	12.171	14.604	13.266
25	0.0958	5.758	4.475	7.010	21.440	25.178
25	0.0968	17.376	14.545	22.577	18.903	29.983
25	0.0978	16.628	28.150	30.053	18.399	25.941
25	0.0988	27.946	21.696	29.337	12.273	18.536
25	0.0998	9.479	12.241	10.421	24.033	19.926
25	0.1008	5.392	10.288	6.971	28.910	26.524
25	0.1018	13.710	14.754	18.692	28.472	33.709
25	0.1028	15.165	17.116	19.978	9.535	29.295
25	0.1038	19.041	22.459	22.895	17.655	24.167
25	0.1048	15.693	12.795	14.464	21.451	12.388

25	0.1058	13.468	8.990	10.520	23.743	25.862
25	0.1068	8.100	6.130	3.214	19.518	16.691
25	0.1078	8.044	5.951	3.139	19.391	16.670
25	0.1088	14.564	9.917	10.231	11.651	17.040
25	0.1098	23.002	18.942	24.162	13.930	27.086
25	0.1108	26.355	19.880	22.009	14.398	22.520
25	0.1118	8.482	7.433	5.583	21.643	18.658
25	0.1128	20.432	15.230	17.713	9.784	16.218
25	0.1138	19.348	13.114	16.516	13.907	16.356
25	0.1148	18.319	15.924	19.731	9.792	18.612
25	0.1158	35.036	38.851	48.482	12.183	24.864
25	0.1168	25.663	25.582	26.546	16.897	20.140
25	0.1178	8.997	13.076	10.325	15.401	14.387
25	0.1188	20.990	24.787	22.057	15.292	8.952
25	0.1198	16.105	11.184	12.649	9.911	14.310
25	0.1208	11.136	9.545	8.730	11.049	18.574
25	0.1218	10.811	13.614	10.329	13.120	31.527
25	0.1228	10.416	10.905	6.817	18.644	27.776
25	0.1238	13.199	12.799	17.648	22.670	25.346
25	0.1248	13.297	13.609	13.742	28.272	36.891
25	0.1258	12.572	12.644	10.841	18.044	16.611
25	0.1268	18.671	18.574	16.177	19.284	17.379
25	0.1278	17.018	17.880	16.165	24.074	23.985
25	0.1288	11.758	15.258	9.015	14.729	21.142
25	0.1298	13.022	9.421	9.461	8.745	18.217
25	0.1308	7.991	6.793	6.689	4.825	10.514
25	0.1318	3.878	2.036	2.651	6.194	11.121
25	0.1328	6.776	5.773	4.032	16.357	6.929
25	0.1338	26.172	18.834	25.327	27.635	25.357
25	0.1348	21.805	24.120	18.424	16.293	18.002
25	0.1358	7.488	12.655	5.901	17.716	15.410
25	0.1368	7.789	9.833	3.320	22.870	16.077
25	0.1378	14.108	11.703	6.488	14.299	11.582
25	0.1388	12.700	11.469	10.906	18.747	18.226
25	0.1398	22.061	26.800	21.608	29.552	18.882
25	0.1408	12.977	14.477	12.507	14.198	21.427
25	0.1418	14.746	14.086	14.455	25.643	21.116
25	0.1428	7.395	12.815	9.761	22.960	23.260
25	0.1438	7.304	12.762	9.729	22.994	23.253
25	0.1448	9.853	12.034	11.587	24.783	26.915
25	0.1458	15.964	11.254	13.021	24.283	11.121
25	0.1468	11.701	14.570	11.492	21.630	17.428
25	0.1478	23.011	18.661	16.007	33.180	27.402
25	0.1488	22.972	18.596	15.992	33.389	27.251
25	0.1498	25.827	24.088	17.729	30.386	18.924
25	0.1508	23.245	17.107	17.064	19.508	17.912
25	0.1518	23.557	23.962	18.407	29.989	22.242
25	0.1528	21.528	17.838	20.245	30.256	14.876
25	0.1538	17.299	20.453	13.922	50.355	17.990
25	0.1548	11.902	11.316	8.389	12.501	10.467

25	0.1558	10.711	8.763	9.427	21.888	8.680
25	0.1568	8.266	5.803	7.560	31.543	17.985
25	0.1578	13.627	3.548	10.623	31.538	12.317
25	0.1588	6.986	4.022	6.529	25.148	13.227
25	0.1598	19.277	13.706	15.301	35.632	20.151
25	0.1608	17.334	20.103	15.622	31.713	12.522
25	0.1618	15.048	18.443	12.962	21.013	20.109
25	0.1628	14.479	11.400	13.344	17.003	6.899
25	0.1638	22.695	21.073	17.773	27.505	15.681
25	0.1648	12.771	8.775	7.594	19.884	10.346
25	0.1658	12.098	10.166	8.334	23.628	7.416
25	0.1668	23.066	14.803	23.409	20.682	7.917
25	0.1678	25.049	18.851	12.840	22.567	15.494
25	0.1688	28.097	21.477	14.226	26.538	14.885
25	0.1698	15.637	14.034	15.971	16.731	13.955
25	0.1708	20.912	14.708	13.885	29.656	13.505
25	0.1718	9.728	6.627	5.260	20.269	12.835
25	0.1728	8.285	7.292	3.128	18.832	21.360
25	0.1738	7.207	9.180	6.179	20.276	6.433
25	0.1748	6.452	10.183	4.517	25.769	18.587
25	0.1758	2.251	9.434	1.739	45.347	17.411
25	0.1768	13.652	11.730	10.820	29.844	8.997
25	0.1778	15.500	12.779	15.157	22.073	6.119
25	0.1788	21.529	24.293	15.976	16.769	16.229
25	0.1798	16.936	10.270	11.321	15.410	12.304
25	0.1808	13.173	5.016	9.511	17.099	9.120
25	0.1818	9.507	3.633	5.672	22.567	3.920
25	0.1828	14.551	10.559	12.777	24.133	7.095
25	0.1838	10.329	9.660	12.180	23.535	10.911
25	0.1848	20.935	25.105	19.526	36.944	16.836
25	0.1858	5.028	7.628	4.321	26.567	9.834
25	0.1868	10.084	8.166	4.495	12.296	6.472
25	0.1878	4.893	9.399	5.333	19.361	18.751
25	0.1888	4.698	7.316	9.096	11.239	14.848
25	0.1898	4.703	7.306	9.114	11.230	14.858
25	0.1908	4.680	8.005	5.107	26.053	18.805
25	0.1918	4.661	7.958	5.125	26.014	18.817
25	0.1928	3.148	5.430	1.307	22.677	11.290
25	0.1938	8.540	9.056	8.581	20.559	14.085
25	0.1948	12.697	8.335	6.527	10.680	7.711
25	0.1958	16.557	3.635	10.121	18.704	15.084
25	0.1968	12.721	7.318	8.840	24.329	22.189
25	0.1978	12.742	7.277	8.834	24.322	22.339
25	0.1988	15.312	13.371	12.757	20.077	16.843
25	0.1998	9.313	6.252	8.242	11.223	18.094
25	0.2008	9.650	12.546	9.494	23.257	14.638
25	0.2018	6.442	8.819	10.455	15.084	8.984
25	0.2028	13.784	12.053	11.530	16.481	7.877
25	0.2038	13.409	9.907	10.282	17.957	5.985
25	0.2048	22.024	12.077	14.394	29.101	17.045

25	0.2058	9.914	10.165	8.154	34.282	12.781
25	0.2068	5.208	9.625	7.322	37.422	13.830
25	0.2078	5.454	14.110	6.668	22.267	11.211
25	0.2088	5.958	11.094	7.238	14.954	14.458
25	0.2098	19.930	18.721	14.633	14.722	25.172
25	0.2108	20.017	18.768	14.845	14.644	25.239
25	0.2118	16.335	9.642	6.403	28.242	16.797
25	0.2128	13.492	12.762	9.047	15.711	12.728
25	0.2138	12.085	11.921	5.311	23.713	17.538
25	0.2148	9.544	16.592	7.854	30.338	16.097
25	0.2158	12.868	16.792	10.433	20.839	15.151
25	0.2168	6.832	11.680	8.394	21.530	10.418
25	0.2178	15.142	16.872	12.966	38.018	18.535
25	0.2188	17.583	23.748	16.883	28.323	17.139
25	0.2198	13.459	21.262	14.265	27.586	12.189
25	0.2208	12.824	19.841	9.421	25.305	13.486
25	0.2218	24.116	18.572	15.327	18.172	16.798
25	0.2228	13.898	10.140	5.470	7.866	6.936
25	0.2238	22.338	16.145	10.128	22.341	13.439
25	0.2248	10.478	17.244	7.129	19.274	14.099
25	0.2258	8.094	16.830	8.471	24.890	13.000
25	0.2268	19.703	23.242	19.296	26.286	30.524
25	0.2278	16.331	24.068	18.087	21.043	21.033
25	0.2288	8.763	15.356	8.845	23.403	9.873
25	0.2298	29.198	29.769	26.296	15.271	4.856
25	0.2308	21.942	25.256	20.250	15.895	21.938
25	0.2318	17.887	17.824	18.707	24.006	10.875
25	0.2328	15.400	14.964	10.229	28.186	15.682
25	0.2338	11.604	8.618	8.978	21.522	13.994
25	0.2348	13.667	18.815	19.151	17.923	22.963
25	0.2358	22.725	23.893	27.825	23.543	14.114
25	0.2368	16.724	22.414	21.660	26.106	15.403
25	0.2378	18.608	18.081	17.081	24.601	12.014
25	0.2388	9.231	10.949	7.537	20.204	8.939
25	0.2398	11.714	12.214	11.313	26.752	27.102
25	0.2408	12.841	21.307	17.578	24.366	23.236
25	0.2418	13.761	32.461	22.516	27.159	15.354
25	0.2428	6.452	14.894	7.803	16.868	17.661
25	0.2438	2.853	9.401	6.010	12.119	3.616
25	0.2448	10.081	4.335	5.439	13.043	13.827
25	0.2458	22.114	14.761	13.728	7.251	14.244
25	0.2468	9.063	9.124	9.172	13.195	15.571
25	0.2478	10.557	7.705	6.335	7.907	14.726
25	0.2488	8.594	8.400	4.491	15.806	12.425
25	0.2498	11.361	9.578	10.804	20.718	21.479
25	0.2508	15.219	7.395	6.605	15.567	18.944
25	0.2518	9.341	8.256	8.309	18.186	16.063
25	0.2528	10.515	16.131	9.753	18.177	19.884
25	0.2538	25.810	20.749	22.143	16.054	25.692
25	0.2548	16.586	19.923	16.453	27.906	29.002

25	0.2558	12.987	11.606	6.386	25.723	32.492
25	0.2568	13.361	11.495	11.658	10.372	15.450
25	0.2578	16.422	17.792	18.209	16.106	21.755
25	0.2588	9.247	12.446	10.676	15.558	20.734
25	0.2598	16.600	17.359	10.550	13.529	13.402
25	0.2608	10.026	12.620	8.071	10.274	6.802
25	0.2618	10.091	11.450	10.965	14.139	12.482
25	0.2628	12.608	8.556	8.576	10.864	19.094
25	0.2638	14.969	23.135	14.725	17.037	16.021
25	0.2648	18.545	21.826	13.632	21.491	10.302
25	0.2658	7.691	17.284	7.838	16.324	15.458
25	0.2668	11.478	19.698	11.742	20.433	25.029
25	0.2678	12.808	18.153	13.542	10.420	16.798
25	0.2688	7.315	6.349	4.768	9.527	19.569
25	0.2698	4.354	6.044	3.251	4.108	2.998
25	0.2708	12.104	18.646	6.655	16.085	6.720
25	0.2718	6.719	12.527	5.572	12.144	5.760
25	0.2728	7.772	11.937	5.329	20.572	17.474
25	0.2738	3.379	10.329	4.667	16.231	16.987
25	0.2748	11.674	14.058	13.103	12.081	13.037
25	0.2758	9.443	5.293	6.248	6.867	17.331
25	0.2768	6.528	8.306	3.414	8.522	16.361
25	0.2778	8.434	13.687	5.393	15.933	20.526
25	0.2788	13.154	10.078	13.004	11.051	14.787
25	0.2798	6.804	10.805	4.591	11.907	9.308
25	0.2808	7.327	21.047	11.176	24.455	7.935
25	0.2818	10.972	19.908	8.339	11.126	4.550
25	0.2828	11.910	15.760	9.935	12.636	4.846
25	0.2838	13.739	23.029	13.419	16.529	7.873
25	0.2848	15.483	22.293	15.826	19.928	15.051
25	0.2858	6.895	10.568	6.821	12.213	16.978
25	0.2868	11.517	14.055	9.409	8.641	24.743
25	0.2878	8.048	12.178	8.797	10.419	20.810
25	0.2888	16.312	12.394	9.781	16.207	17.542
25	0.2898	8.536	9.693	7.907	14.898	24.115
25	0.2908	12.164	17.824	19.029	6.159	9.201
25	0.2918	14.588	22.165	16.448	14.430	10.529
25	0.2928	14.043	19.321	15.619	19.058	15.781
25	0.2938	7.993	14.496	9.824	14.016	15.615
25	0.2948	6.953	5.785	5.760	3.673	11.104
25	0.2958	7.809	18.227	9.894	10.754	7.816
25	0.2968	7.862	11.320	8.843	7.525	8.906
25	0.2978	7.320	10.507	10.969	7.606	24.875
25	0.2988	7.285	10.563	10.915	7.595	24.872
25	0.2998	11.687	14.802	9.098	8.653	7.974
25	0.3008	9.580	15.546	12.115	13.069	19.564
25	0.3018	5.079	2.901	6.031	10.205	8.931
25	0.3028	11.192	19.814	17.292	9.700	4.938
25	0.3038	7.258	11.288	10.380	6.613	6.483
25	0.3048	4.335	4.279	3.997	6.478	13.482

25	0.3058	7.851	5.538	6.488	3.813	4.387
25	0.3068	8.634	4.790	6.497	5.293	6.238
25	0.3078	6.503	5.211	3.598	5.248	4.344
25	0.3088	6.829	8.404	9.159	2.114	6.788
25	0.3098	11.173	6.806	7.436	12.032	16.695
25	0.3108	10.591	6.954	4.919	11.540	9.270
25	0.3118	7.656	12.821	7.207	7.863	10.693
25	0.3128	13.751	13.056	12.238	9.931	5.763
25	0.3138	9.474	9.582	8.698	14.381	12.868
25	0.3148	9.564	9.618	8.754	14.283	12.902
25	0.3158	9.832	3.720	7.329	12.605	12.217
25	0.3168	12.720	5.274	9.875	19.542	12.840
25	0.3178	8.074	7.945	8.272	11.552	10.014
25	0.3188	10.972	16.244	14.114	13.545	13.063
25	0.3198	12.382	8.268	7.547	8.804	15.900
25	0.3208	8.045	9.768	8.906	11.048	4.986
25	0.3218	18.202	18.390	16.948	13.693	11.544
25	0.3228	8.646	10.397	7.687	5.873	15.448
25	0.3238	6.392	9.695	4.566	13.318	10.044
25	0.3248	10.342	9.790	12.156	5.549	14.130
25	0.3258	14.129	19.718	9.587	17.374	11.449
25	0.3268	5.566	5.957	5.644	11.968	12.574
25	0.3278	5.619	5.949	5.627	11.795	12.316
25	0.3288	8.505	7.843	10.429	8.002	10.731
25	0.3298	13.620	9.858	10.631	10.865	9.369
25	0.3308	11.607	13.469	9.330	15.681	15.107
25	0.3318	11.112	8.820	9.144	23.148	17.413
25	0.3328	25.001	21.028	20.394	11.161	14.272
25	0.3338	13.035	19.253	14.621	19.886	14.765
25	0.3348	8.436	13.271	8.948	24.060	22.899
25	0.3358	11.324	11.778	10.768	9.736	26.351
25	0.3368	7.826	11.739	8.803	11.374	26.032
25	0.3378	6.992	1.841	2.701	8.256	23.482
25	0.3388	8.027	9.504	7.042	9.685	9.456
25	0.3398	7.911	10.821	7.093	10.877	12.198
25	0.3408	6.365	15.818	9.715	16.600	21.839
25	0.3418	8.596	17.888	11.886	32.202	17.480
25	0.3428	8.607	17.734	11.796	32.126	17.339
25	0.3438	9.708	8.447	9.651	12.564	11.352
25	0.3448	11.341	18.759	12.665	15.878	18.211
25	0.3458	8.855	13.838	10.522	21.864	21.894
25	0.3468	14.415	15.361	10.590	13.951	19.068
25	0.3478	8.251	7.107	9.641	8.920	10.767
25	0.3488	8.253	7.110	9.643	8.922	10.771
25	0.3498	5.433	6.768	4.989	16.222	18.471
25	0.3508	5.581	4.428	4.168	15.491	18.170
25	0.3518	9.619	9.913	7.783	13.780	17.471
25	0.3528	8.237	7.912	8.401	17.710	27.923
25	0.3538	10.221	11.158	14.578	11.754	18.448
25	0.3548	11.554	21.565	18.160	31.885	27.943

25	0.3558	12.247	15.180	12.238	19.671	21.915
25	0.3568	16.444	12.564	14.618	6.312	17.390
25	0.3578	11.774	7.606	8.000	6.668	13.365
25	0.3588	10.143	5.445	9.146	5.975	13.309
25	0.3598	8.084	10.713	11.633	9.757	8.532
25	0.3608	7.889	9.658	9.217	7.299	9.760
25	0.3618	12.716	11.447	11.439	9.574	12.173
25	0.3628	5.796	6.741	3.910	8.507	15.967
25	0.3638	14.247	11.020	9.449	17.770	21.191
25	0.3648	5.386	11.149	8.346	14.695	17.500
25	0.3658	5.407	11.206	8.338	14.785	17.460
25	0.3668	10.746	12.798	13.934	12.136	15.503
25	0.3678	5.940	9.360	11.402	10.606	16.528
25	0.3688	18.660	23.325	21.501	8.193	22.485
25	0.3698	13.203	18.095	14.775	14.873	19.726
25	0.3708	8.728	10.111	12.722	20.610	14.594
25	0.3718	11.202	12.628	9.453	7.561	13.051
25	0.3728	8.690	5.614	7.715	14.249	11.796
25	0.3738	8.789	5.758	7.839	14.258	11.764
25	0.3748	8.682	9.422	10.617	13.680	13.334
25	0.3758	8.014	18.061	14.785	10.698	12.871
25	0.3768	13.979	10.927	9.890	9.028	10.321
25	0.3778	18.709	18.661	13.869	14.406	9.659
25	0.3788	12.208	12.515	8.835	17.544	15.286
25	0.3798	9.650	10.354	12.032	9.257	17.214
25	0.3808	5.200	6.287	5.478	14.427	21.725
25	0.3818	9.686	10.507	8.087	18.733	21.033
25	0.3828	13.366	11.568	10.372	12.656	9.762
25	0.3838	9.241	13.330	7.265	18.027	13.557
25	0.3848	8.903	15.265	8.281	11.917	14.688
25	0.3858	10.048	12.651	12.186	12.240	19.696
25	0.3868	4.738	9.250	6.628	16.341	19.735
25	0.3878	4.746	9.274	6.649	16.359	19.796
25	0.3888	5.442	9.866	5.493	18.786	22.271
25	0.3898	10.997	11.182	14.885	12.009	6.542
25	0.3908	8.859	8.798	10.474	2.766	12.904
25	0.3918	11.419	7.525	10.516	10.133	29.265
25	0.3928	13.112	14.115	13.938	13.255	24.348
25	0.3938	3.179	2.715	4.347	6.103	22.238
25	0.3948	5.120	7.302	6.738	13.988	23.729
25	0.3958	7.358	7.448	10.583	8.816	21.752
25	0.3968	9.038	9.255	11.917	15.414	27.605
25	0.3978	9.539	11.137	8.818	12.307	26.811
25	0.3988	14.026	11.537	13.866	15.705	20.650
25	0.3998	13.511	11.388	12.460	13.686	17.872
25	0.4008	7.208	7.975	7.870	9.656	22.817
25	0.4018	9.496	6.934	8.941	9.089	19.323
25	0.4028	3.846	7.115	9.709	12.809	23.678
25	0.4038	7.816	7.187	10.094	13.756	18.642
25	0.4048	11.105	13.467	9.029	17.793	23.969

25	0.4058	5.517	7.292	7.614	16.876	18.330
25	0.4068	12.094	14.476	12.888	10.456	11.703
25	0.4078	6.743	5.763	4.427	3.331	11.518
25	0.4088	6.742	5.756	4.410	3.312	11.473
25	0.4098	3.957	3.600	3.037	10.350	22.556
25	0.4108	5.942	4.973	7.479	6.659	13.450
25	0.4118	7.478	11.296	11.960	10.762	24.744
25	0.4128	18.891	17.265	14.937	17.411	24.310
25	0.4138	11.961	7.787	7.289	12.195	24.424
25	0.4148	0.000	0.000	0.000	0.000	0.000
26	0.0016	0.000	0.000	0.000	0.000	0.000
26	0.0026	7.024	4.498	6.390	3.039	7.888
26	0.0036	14.562	6.033	8.887	21.205	20.678
26	0.0046	14.915	7.067	9.145	12.534	8.939
26	0.0056	18.519	8.770	8.859	22.277	17.539
26	0.0066	18.068	11.457	12.534	15.351	15.228
26	0.0076	22.044	13.014	13.554	8.190	7.834
26	0.0086	12.093	6.667	6.989	9.212	9.971
26	0.0096	15.368	12.321	15.591	7.034	16.116
26	0.0106	13.546	10.168	8.142	4.126	16.782
26	0.0116	9.309	3.392	5.821	10.834	18.062
26	0.0126	6.621	4.407	5.569	11.904	11.810
26	0.0136	8.423	4.782	4.537	4.361	14.070
26	0.0146	11.189	6.313	7.741	5.346	7.016
26	0.0156	12.462	7.245	12.318	6.267	9.373
26	0.0166	6.504	7.004	5.906	11.057	6.819
26	0.0176	17.267	7.399	5.959	10.879	16.283
26	0.0186	8.513	15.115	9.260	8.704	14.625
26	0.0196	7.764	7.332	7.151	14.047	6.345
26	0.0206	8.828	14.752	5.147	20.531	7.972
26	0.0216	10.574	13.818	9.661	9.471	7.213
26	0.0226	11.434	13.580	11.346	10.123	12.926
26	0.0236	11.013	4.442	8.921	7.637	5.948
26	0.0246	4.386	9.102	3.655	13.320	11.206
26	0.0256	13.606	15.494	10.212	16.625	15.343
26	0.0266	17.722	13.062	13.201	12.989	9.068
26	0.0276	19.591	11.506	11.895	18.881	9.332
26	0.0286	11.002	13.410	11.483	11.608	6.218
26	0.0296	10.183	10.547	9.471	11.368	12.380
26	0.0306	2.008	5.166	3.095	7.324	11.471
26	0.0316	2.172	3.557	1.482	8.061	7.677
26	0.0326	6.314	7.045	5.780	5.980	7.706
26	0.0336	7.223	10.609	7.171	13.075	11.790
26	0.0346	11.831	10.652	10.152	23.115	16.845
26	0.0356	11.187	8.657	7.883	16.216	10.147
26	0.0366	10.941	16.776	15.955	11.005	13.556
26	0.0376	17.742	15.122	17.075	12.590	15.914
26	0.0386	10.353	9.721	11.824	12.834	12.643
26	0.0396	12.515	15.845	14.681	13.123	7.440
26	0.0406	15.619	16.899	14.602	26.239	13.652

26	0.0416	11.685	11.463	14.187	13.362	15.849
26	0.0426	12.439	11.482	11.418	13.343	12.101
26	0.0436	13.773	18.603	18.571	20.222	11.775
26	0.0446	13.387	15.463	13.820	24.436	9.089
26	0.0456	15.038	23.602	23.766	16.620	10.455
26	0.0466	26.192	29.498	29.846	27.778	14.446
26	0.0476	17.061	26.490	17.759	15.465	8.871
26	0.0486	23.364	22.625	18.160	14.071	11.962
26	0.0496	14.997	14.033	16.475	16.601	26.158
26	0.0506	12.805	25.049	18.020	23.544	13.178
26	0.0516	9.760	10.089	9.078	14.851	16.780
26	0.0526	9.305	10.810	8.195	26.125	7.640
26	0.0536	11.000	13.458	13.595	14.144	15.024
26	0.0546	20.637	19.877	21.133	18.559	14.859
26	0.0556	16.428	14.380	18.908	13.036	16.514
26	0.0566	11.435	11.833	12.399	9.294	16.219
26	0.0576	11.182	3.835	6.396	16.391	19.052
26	0.0586	10.742	7.966	10.046	6.945	15.931
26	0.0596	12.017	12.096	13.140	22.652	22.489
26	0.0606	10.328	17.883	14.508	3.644	8.934
26	0.0616	6.268	8.639	7.382	10.193	12.226
26	0.0626	7.471	7.167	7.325	11.446	13.768
26	0.0636	19.194	14.492	19.780	19.482	11.860
26	0.0646	11.553	8.617	15.552	7.799	9.235
26	0.0656	15.103	20.342	13.893	12.351	8.334
26	0.0666	18.415	16.439	21.891	7.749	5.460
26	0.0676	13.361	16.811	16.016	26.894	10.468
26	0.0686	18.452	17.635	19.998	15.011	13.533
26	0.0696	26.728	17.563	28.945	9.920	8.478
26	0.0706	8.713	10.451	8.357	18.305	13.441
26	0.0716	12.477	13.482	15.804	24.022	18.730
26	0.0726	5.657	8.969	8.768	17.854	15.864
26	0.0736	12.682	11.779	17.276	9.342	12.059
26	0.0746	9.524	6.016	12.999	15.767	10.479
26	0.0756	16.383	11.952	13.845	8.836	16.630
26	0.0766	14.793	8.846	11.048	15.496	16.592
26	0.0776	22.617	11.409	17.844	16.769	13.723
26	0.0786	16.338	11.009	17.160	17.917	15.404
26	0.0796	10.144	6.685	10.047	17.372	8.539
26	0.0806	11.324	8.960	7.536	19.683	26.892
26	0.0816	7.959	8.870	10.487	7.780	11.612
26	0.0826	5.987	2.861	3.590	8.765	9.688
26	0.0836	16.252	10.307	17.193	28.455	19.718
26	0.0846	9.162	5.230	10.140	6.469	13.137
26	0.0856	14.079	5.544	11.035	15.220	11.769
26	0.0866	18.851	6.894	18.750	19.562	14.298
26	0.0876	29.943	19.348	24.366	25.277	13.103
26	0.0886	15.400	10.509	11.259	36.066	15.337
26	0.0896	20.057	14.869	18.530	13.440	24.644
26	0.0906	5.506	9.944	9.216	10.184	15.166

26	0.0916	13.479	16.074	15.513	15.895	19.242
26	0.0926	15.374	14.810	15.329	10.267	5.376
26	0.0936	13.570	12.697	17.388	11.492	20.803
26	0.0946	11.658	5.443	10.906	20.895	14.296
26	0.0956	7.978	3.801	8.347	22.525	22.354
26	0.0966	25.356	14.010	27.785	17.985	23.231
26	0.0976	15.372	10.940	13.840	18.561	27.110
26	0.0986	17.428	15.160	16.345	22.990	32.662
26	0.0996	8.695	7.449	6.685	12.163	30.896
26	0.1006	14.650	10.831	12.971	13.680	19.146
26	0.1016	12.242	3.526	7.037	8.229	15.827
26	0.1026	10.687	8.081	7.596	19.631	16.416
26	0.1036	8.013	9.185	11.001	19.478	16.245
26	0.1046	6.822	6.439	9.933	12.910	11.662
26	0.1056	9.749	9.470	10.283	17.771	14.755
26	0.1066	8.771	14.571	11.025	24.650	14.445
26	0.1076	9.607	10.268	5.351	26.981	16.378
26	0.1086	14.673	13.345	10.784	33.994	29.178
26	0.1096	19.535	24.671	13.104	26.036	24.697
26	0.1106	11.782	8.458	12.664	21.974	19.891
26	0.1116	18.101	13.329	9.755	21.353	19.843
26	0.1126	9.827	15.063	8.941	25.827	20.822
26	0.1136	25.459	22.125	15.410	9.835	16.740
26	0.1146	20.549	14.905	15.242	13.761	25.365
26	0.1156	24.772	14.080	12.440	26.910	20.799
26	0.1166	16.111	23.307	18.382	13.193	23.789
26	0.1176	15.961	12.822	12.397	7.554	9.698
26	0.1186	26.643	21.530	18.859	10.971	14.472
26	0.1196	18.694	16.835	18.804	11.824	8.714
26	0.1206	15.723	9.553	15.065	21.545	6.328
26	0.1216	17.295	21.063	14.017	21.896	22.959
26	0.1226	20.773	21.292	24.367	19.126	20.130
26	0.1236	18.015	7.873	18.273	13.918	25.042
26	0.1246	8.237	6.046	10.528	19.639	7.982
26	0.1256	9.336	4.955	10.999	10.222	4.133
26	0.1266	16.647	11.417	11.183	14.166	25.289
26	0.1276	20.177	15.349	18.371	25.061	15.588
26	0.1286	18.919	10.971	15.296	9.963	13.546
26	0.1296	21.345	11.885	11.013	14.255	28.679
26	0.1306	21.801	10.819	13.089	11.179	22.523
26	0.1316	15.863	16.156	14.834	9.915	19.571
26	0.1326	15.049	12.180	9.420	12.343	24.924
26	0.1336	15.492	13.336	11.941	16.648	26.533
26	0.1346	25.022	16.941	23.348	21.850	22.656
26	0.1356	17.825	17.473	20.193	14.146	20.520
26	0.1366	31.111	20.887	28.639	10.798	13.902
26	0.1376	25.803	28.762	25.343	9.995	12.973
26	0.1386	24.177	29.265	22.248	12.064	16.589
26	0.1396	18.931	18.503	15.458	9.119	11.268
26	0.1406	13.161	15.190	7.188	10.908	18.541

26	0.1416	25.278	26.997	21.110	14.043	19.988
26	0.1426	23.537	23.370	18.387	6.621	25.732
26	0.1436	24.111	15.370	16.733	10.575	29.508
26	0.1446	15.486	16.761	19.073	6.995	22.684
26	0.1456	18.147	18.870	14.214	13.421	18.327
26	0.1466	18.796	17.712	16.971	6.453	23.109
26	0.1476	6.191	4.416	4.730	2.094	4.239
26	0.1486	12.975	11.920	9.688	5.744	19.942
26	0.1496	35.125	25.390	30.144	17.335	25.844
26	0.1506	25.710	18.727	23.821	16.833	22.793
26	0.1516	20.085	18.590	20.571	13.686	17.369
26	0.1526	26.948	19.865	20.911	19.531	25.181
26	0.1536	15.046	12.767	10.810	14.193	19.251
26	0.1546	8.778	12.393	8.763	9.035	9.180
26	0.1556	28.707	27.755	22.625	8.933	9.490
26	0.1566	9.872	9.449	4.562	6.950	17.667
26	0.1576	9.808	10.876	6.052	7.067	12.388
26	0.1586	11.502	7.874	8.032	5.843	16.175
26	0.1596	9.224	8.034	10.521	5.346	14.635
26	0.1606	18.216	15.827	15.824	11.789	17.163
26	0.1616	15.420	10.882	13.859	11.539	32.885
26	0.1626	7.362	8.871	7.634	10.821	23.060
26	0.1636	14.007	16.162	12.180	9.739	10.169
26	0.1646	6.271	2.987	4.125	4.324	19.435
26	0.1656	2.270	2.620	0.971	3.869	3.504
26	0.1666	7.558	5.444	4.400	6.980	12.278
26	0.1676	18.652	14.521	10.284	18.681	24.942
26	0.1686	15.645	20.383	8.957	12.917	19.412
26	0.1696	24.070	15.934	17.458	8.714	21.967
26	0.1706	14.512	13.914	9.898	12.755	13.045
26	0.1716	30.634	25.560	27.597	11.390	18.924
26	0.1726	28.430	33.551	26.411	8.338	13.094
26	0.1736	20.552	23.717	16.413	12.809	17.692
26	0.1746	13.315	10.068	9.762	13.148	13.230
26	0.1756	20.034	16.976	15.920	14.149	20.728
26	0.1766	20.031	16.975	15.926	14.156	20.761
26	0.1776	10.835	7.990	7.232	5.876	15.955
26	0.1786	15.921	12.121	11.421	15.672	21.786
26	0.1796	18.762	20.339	14.699	17.001	18.225
26	0.1806	11.221	11.367	8.811	13.248	14.616
26	0.1816	16.023	12.589	10.466	11.546	29.019
26	0.1826	12.366	14.749	10.898	17.891	22.246
26	0.1836	24.339	19.757	15.371	9.666	25.339
26	0.1846	17.966	16.799	16.014	14.842	24.012
26	0.1856	6.288	6.254	4.447	5.716	10.021
26	0.1866	7.116	6.429	3.939	7.346	9.968
26	0.1876	18.071	24.295	15.495	15.756	16.879
26	0.1886	12.689	16.557	13.598	12.577	20.635
26	0.1896	10.637	12.327	10.338	11.593	13.430
26	0.1906	4.119	5.913	3.235	9.607	8.562

26	0.1916	8.393	9.831	5.090	17.469	21.846
26	0.1926	18.772	18.204	11.621	22.526	31.931
26	0.1936	11.691	10.982	6.715	5.686	18.288
26	0.1946	7.267	10.691	10.209	7.586	9.580
26	0.1956	8.082	14.174	8.045	9.843	15.212
26	0.1966	4.853	9.733	3.918	7.943	10.497
26	0.1976	12.905	14.299	9.765	12.278	4.653
26	0.1986	8.671	16.992	8.412	17.237	19.859
26	0.1996	20.412	11.763	10.749	10.434	17.059
26	0.2006	22.437	11.175	16.083	16.413	9.632
26	0.2016	26.881	18.847	19.762	13.221	16.120
26	0.2026	23.934	17.407	15.524	13.749	31.413
26	0.2036	5.500	8.098	4.221	11.657	11.326
26	0.2046	8.296	10.144	6.563	7.368	3.522
26	0.2056	10.155	11.335	8.054	7.106	5.719
26	0.2066	8.318	10.252	5.390	6.865	15.486
26	0.2076	5.721	3.727	4.982	3.164	4.863
26	0.2086	12.541	18.902	12.367	9.123	12.472
26	0.2096	10.235	11.899	7.092	10.844	4.022
26	0.2106	11.408	3.190	4.539	5.473	21.082
26	0.2116	22.203	15.251	17.352	16.729	38.724
26	0.2126	13.189	6.998	9.495	17.494	27.099
26	0.2136	7.331	5.909	1.568	10.337	9.248
26	0.2146	13.352	12.819	9.074	19.645	25.952
26	0.2156	18.374	19.514	13.366	19.678	19.131
26	0.2166	2.470	4.321	2.561	15.986	15.258
26	0.2176	10.480	9.067	6.626	11.702	19.815
26	0.2186	26.037	17.094	19.122	14.656	27.069
26	0.2196	16.748	22.210	13.959	13.643	20.336
26	0.2206	12.930	18.738	14.216	7.461	3.751
26	0.2216	16.201	13.984	13.282	12.963	8.717
26	0.2226	21.955	16.912	16.899	8.177	17.401
26	0.2236	11.791	14.147	9.325	15.786	17.621
26	0.2246	9.655	7.001	9.257	5.505	8.050
26	0.2256	8.474	12.874	9.392	6.100	15.393
26	0.2266	9.933	12.353	10.247	7.462	14.296
26	0.2276	17.900	23.359	22.221	10.240	11.839
26	0.2286	31.333	26.008	25.645	9.116	21.711
26	0.2296	15.120	12.941	13.591	15.658	26.052
26	0.2306	14.162	6.581	12.828	19.338	27.695
26	0.2316	21.277	15.595	16.928	15.660	33.929
26	0.2326	18.414	12.976	16.513	13.267	8.832
26	0.2336	10.978	10.224	13.796	11.778	10.636
26	0.2346	20.537	11.646	21.222	9.357	15.732
26	0.2356	19.283	11.540	12.330	15.160	15.354
26	0.2366	25.254	12.763	19.771	10.312	32.309
26	0.2376	25.465	12.938	19.874	10.191	32.230
26	0.2386	30.491	19.657	24.122	15.707	40.099
26	0.2396	33.298	19.493	19.918	14.184	28.780
26	0.2406	32.672	23.461	20.040	12.265	34.162

26	0.2416	24.539	21.872	14.892	10.754	36.205
26	0.2426	19.188	10.711	8.162	15.986	45.111
26	0.2436	14.736	12.167	6.970	12.052	33.280
26	0.2446	23.553	18.097	17.843	15.996	30.952
26	0.2456	19.830	16.192	12.174	13.433	28.255
26	0.2466	26.209	18.816	17.795	16.082	13.941
26	0.2476	12.078	15.468	12.959	19.823	13.077
26	0.2486	31.811	26.720	21.738	12.882	23.698
26	0.2496	27.603	17.608	18.586	13.211	24.246
26	0.2506	27.182	17.588	18.324	13.112	24.093
26	0.2516	18.199	13.519	14.476	12.203	9.130
26	0.2526	18.571	13.310	14.734	12.193	9.009
26	0.2536	32.163	36.874	33.170	13.333	13.786
26	0.2546	15.973	12.807	11.692	18.433	10.117
26	0.2556	15.617	12.792	11.859	18.162	10.146
26	0.2566	16.638	11.092	11.327	12.331	14.080
26	0.2576	14.336	9.609	14.269	11.991	12.191
26	0.2586	7.248	9.084	10.558	22.468	21.244
26	0.2596	24.737	17.491	18.900	8.984	19.659
26	0.2606	13.613	11.230	11.385	11.030	9.717
26	0.2616	28.341	16.657	18.891	22.874	17.077
26	0.2626	23.917	19.573	19.950	18.399	10.832
26	0.2636	20.705	12.577	14.451	20.328	14.498
26	0.2646	19.750	19.449	15.208	9.598	22.419
26	0.2656	22.137	13.253	11.781	12.437	17.774
26	0.2666	13.571	22.743	9.728	12.657	18.501
26	0.2676	11.960	20.694	10.932	9.475	7.534
26	0.2686	34.538	16.064	17.526	20.739	20.942
26	0.2696	25.254	15.991	13.826	19.032	27.160
26	0.2706	12.285	8.768	5.157	10.835	14.806
26	0.2716	20.571	13.196	11.384	11.582	23.119
26	0.2726	22.412	13.546	14.991	6.036	8.903
26	0.2736	22.960	21.410	14.776	11.796	22.869
26	0.2746	15.445	10.918	9.976	7.927	21.984
26	0.2756	10.288	8.470	10.196	3.222	12.586
26	0.2766	8.624	10.547	10.175	10.745	12.448
26	0.2776	7.732	14.648	7.853	13.433	10.305
26	0.2786	11.007	7.682	6.544	7.072	11.428
26	0.2796	22.946	18.019	21.574	11.999	20.821
26	0.2806	28.170	27.571	20.692	16.551	15.923
26	0.2816	28.089	12.873	15.289	15.148	18.283
26	0.2826	15.391	14.119	8.849	10.314	13.164
26	0.2836	11.918	16.538	12.441	10.428	15.249
26	0.2846	8.443	9.800	10.043	2.784	12.307
26	0.2856	17.705	20.787	19.389	16.921	29.469
26	0.2866	25.374	20.235	14.204	18.279	29.589
26	0.2876	9.118	10.564	10.095	15.796	7.591
26	0.2886	9.479	10.431	9.307	11.258	13.550
26	0.2896	7.843	10.073	8.747	12.698	19.589
26	0.2906	20.668	16.802	16.186	6.507	25.152

26	0.2916	20.062	15.704	15.330	8.908	27.741
26	0.2926	12.124	15.835	12.697	8.430	18.544
26	0.2936	10.705	15.762	12.029	10.777	21.097
26	0.2946	10.578	14.278	12.395	14.701	20.834
26	0.2956	5.108	6.912	5.972	7.347	15.111
26	0.2966	6.583	8.739	7.252	12.552	14.531
26	0.2976	17.900	14.500	13.825	10.055	9.640
26	0.2986	10.581	16.066	13.075	8.618	11.107
26	0.2996	8.687	13.835	10.015	11.827	13.738
26	0.3006	12.261	14.781	10.837	10.168	17.207
26	0.3016	13.934	9.145	10.917	12.998	20.027
26	0.3026	3.732	9.865	6.209	6.963	9.549
26	0.3036	6.506	14.515	7.122	9.667	10.446
26	0.3046	13.255	18.482	13.679	16.679	24.530
26	0.3056	21.932	8.562	11.190	7.630	25.597
26	0.3066	26.897	11.954	14.842	10.973	21.312
26	0.3076	14.185	16.238	10.773	7.649	16.570
26	0.3086	19.833	18.266	18.692	11.679	22.990
26	0.3096	21.822	11.484	17.863	12.638	22.961
26	0.3106	10.311	12.448	11.385	9.608	21.345
26	0.3116	25.244	25.248	24.108	10.193	23.770
26	0.3126	27.572	28.920	28.169	14.873	26.671
26	0.3136	27.690	27.365	24.945	12.738	22.225
26	0.3146	21.968	21.912	17.496	13.705	24.273
26	0.3156	14.738	14.158	10.136	7.573	21.544
26	0.3166	18.710	17.776	16.843	5.405	26.189
26	0.3176	16.401	21.692	19.646	13.316	11.155
26	0.3186	9.747	11.095	12.616	14.785	21.565
26	0.3196	11.392	13.742	6.636	8.653	10.798
26	0.3206	29.328	20.848	23.752	14.693	18.309
26	0.3216	15.744	12.943	11.767	8.122	16.320
26	0.3226	15.054	7.452	9.943	15.030	18.610
26	0.3236	21.071	11.544	11.201	8.250	34.325
26	0.3246	19.621	13.833	14.182	5.385	21.798
26	0.3256	29.100	20.308	19.340	12.102	29.360
26	0.3266	30.810	27.937	24.489	13.199	14.556
26	0.3276	12.667	9.602	10.981	11.867	24.384
26	0.3286	14.845	9.017	10.500	10.925	11.089
26	0.3296	30.423	19.124	20.909	11.371	19.320
26	0.3306	25.971	14.091	17.614	10.152	26.926
26	0.3316	16.145	11.454	17.181	8.156	9.766
26	0.3326	15.638	10.300	7.155	11.640	22.542
26	0.3336	12.800	7.423	6.900	15.902	21.445
26	0.3346	16.727	10.601	11.285	19.537	24.783
26	0.3356	15.372	9.259	8.845	10.105	20.421
26	0.3366	13.186	11.741	16.504	18.838	28.511
26	0.3376	20.841	20.756	19.117	9.426	25.328
26	0.3386	22.268	12.908	15.067	9.699	21.068
26	0.3396	16.852	14.012	11.761	16.397	19.231
26	0.3406	20.300	8.666	13.791	11.078	17.227

26	0.3416	25.320	19.820	26.530	21.092	20.792
26	0.3426	22.253	12.652	14.093	7.082	9.687
26	0.3436	20.637	12.070	13.859	14.589	23.461
26	0.3446	14.309	8.159	15.593	13.541	20.930
26	0.3456	25.393	12.962	19.238	6.192	29.946
26	0.3466	24.222	11.066	14.978	10.850	32.600
26	0.3476	25.483	17.867	18.651	9.984	34.445
26	0.3486	27.445	17.684	14.417	14.473	19.801
26	0.3496	23.137	19.773	22.647	17.526	19.436
26	0.3506	18.515	13.910	16.254	9.652	28.150
26	0.3516	10.172	9.513	12.183	13.511	16.378
26	0.3526	24.294	13.558	17.106	10.982	18.421
26	0.3536	20.861	15.173	8.658	16.923	26.384
26	0.3546	14.929	7.727	9.462	12.757	25.417
26	0.3556	34.252	29.071	27.182	12.913	29.437
26	0.3566	25.370	17.897	20.109	16.183	24.774
26	0.3576	20.220	5.102	12.865	13.607	20.585
26	0.3586	20.211	14.283	19.325	15.247	19.094
26	0.3596	18.998	12.421	14.844	16.453	21.993
26	0.3606	6.063	6.823	6.980	6.740	9.930
26	0.3616	12.013	8.098	8.532	16.838	10.311
26	0.3626	13.315	9.059	11.805	18.660	11.382
26	0.3636	11.636	7.034	5.804	17.065	14.927
26	0.3646	10.041	10.658	13.153	13.059	13.527
26	0.3656	12.074	12.517	19.716	12.103	15.027
26	0.3666	12.580	9.902	15.972	12.387	19.223
26	0.3676	24.561	22.330	28.614	14.490	18.689
26	0.3686	15.893	7.161	14.823	15.125	13.324
26	0.3696	15.742	15.154	17.755	15.560	19.924
26	0.3706	16.098	14.611	12.774	14.566	25.103
26	0.3716	15.260	7.320	12.845	11.291	15.048
26	0.3726	22.292	13.471	18.227	15.955	20.551
26	0.3736	15.810	4.288	3.570	8.913	10.064
26	0.3746	25.188	19.902	20.889	10.489	7.564
26	0.3756	20.464	17.279	19.120	22.429	29.797
26	0.3766	12.390	15.328	11.932	30.498	28.597
26	0.3776	15.149	21.236	14.774	20.535	15.511
26	0.3786	18.265	17.741	14.235	26.788	17.539
26	0.3796	21.162	11.782	13.818	29.175	19.591
26	0.3806	32.146	16.323	15.689	20.725	13.649
26	0.3816	20.012	14.564	17.971	25.631	16.342
26	0.3826	12.879	7.936	14.476	9.639	18.600
26	0.3836	42.084	19.570	24.194	24.562	11.760
26	0.3846	18.345	15.228	20.810	17.257	18.051
26	0.3856	14.248	15.269	13.827	18.456	11.244
26	0.3866	19.275	14.617	16.726	25.166	26.804
26	0.3876	16.925	8.892	9.272	16.572	20.687
26	0.3886	20.460	4.834	10.082	16.211	16.520
26	0.3896	18.418	9.395	11.243	9.597	19.552
26	0.3906	17.598	13.240	13.083	8.164	13.207

26	0.3916	10.677	8.264	6.520	10.924	9.052
26	0.3926	8.148	9.080	8.931	12.022	10.558
26	0.3936	7.141	6.187	7.153	12.236	13.824
26	0.3946	25.570	10.205	19.690	8.958	19.253
26	0.3956	24.272	15.451	19.385	12.691	21.942
26	0.3966	25.266	16.016	24.849	11.879	28.525
26	0.3976	17.534	9.623	12.359	17.464	26.582
26	0.3986	22.512	18.012	16.656	10.941	29.345
26	0.3996	18.098	17.276	17.852	6.677	27.054
26	0.4006	17.985	18.855	17.232	8.669	19.800
26	0.4016	25.802	22.339	21.342	8.937	13.381
26	0.4026	14.179	7.802	7.507	7.360	18.424
26	0.4036	8.061	7.775	7.182	10.093	12.706
26	0.4046	13.676	10.500	14.209	14.229	18.750
26	0.4056	21.408	12.854	16.227	20.170	25.973
26	0.4066	19.793	23.154	18.880	18.329	18.775
26	0.4076	18.720	12.876	9.623	14.932	19.770
26	0.4086	12.557	6.112	8.190	10.695	16.871
26	0.4096	20.417	13.990	13.769	14.156	22.122
26	0.4106	20.034	15.436	16.877	15.380	28.726
26	0.4116	20.557	11.430	20.791	13.252	11.185
26	0.4126	20.651	11.055	21.879	11.942	15.147
26	0.4136	27.298	25.813	25.724	7.982	12.164
26	0.4146	29.275	27.947	26.247	9.638	16.475
26	0.4156	25.458	11.442	16.042	6.145	14.248
26	0.4166	26.845	14.368	23.651	10.135	18.918
26	0.4176	25.879	15.184	23.480	15.254	25.820
26	0.4186	24.975	14.523	22.533	24.709	24.970
26	0.4196	24.964	14.522	22.549	24.704	24.976
26	0.4206	24.951	14.520	22.565	24.705	24.983
26	0.4216	29.304	14.063	14.791	11.045	22.692
26	0.4226	21.022	7.082	9.980	16.813	24.743
26	0.4236	22.313	9.839	12.794	14.996	25.991
26	0.4246	19.472	14.338	14.232	9.772	23.217
26	0.4256	4.884	5.984	5.409	15.388	15.794
26	0.4266	16.722	16.532	23.036	16.838	16.265
26	0.4276	23.379	11.971	16.037	18.050	21.610
26	0.4286	39.975	19.584	27.707	18.070	20.255
26	0.4296	39.985	19.599	27.718	17.915	20.212
26	0.4306	25.863	23.577	23.334	19.080	37.094
26	0.4316	26.805	22.778	26.803	14.414	24.036
26	0.4326	17.000	10.485	9.247	16.073	21.110
26	0.4336	23.440	12.267	18.875	19.492	23.193
26	0.4346	28.946	15.190	20.279	17.723	23.371
26	0.4356	39.946	14.672	34.498	22.776	28.000
26	0.4366	26.488	12.617	9.649	15.554	24.076
26	0.4376	22.073	15.580	13.668	17.062	23.740
26	0.4386	23.078	15.249	20.815	14.892	28.899
26	0.4396	28.072	18.677	29.130	23.810	20.076
26	0.4406	27.356	16.222	17.837	23.285	30.965

26	0.4416	28.664	9.031	16.614	21.148	27.650
26	0.4426	27.956	15.145	18.301	8.429	20.776
26	0.4436	20.768	12.607	15.531	13.939	35.211
26	0.4446	23.803	25.598	20.865	18.336	16.421
26	0.4456	25.694	26.582	20.726	6.241	18.937
26	0.4466	27.918	25.691	25.712	6.188	16.742
26	0.4476	21.450	20.232	17.796	14.871	17.344
26	0.4486	7.896	8.943	9.009	5.523	14.189
26	0.4496	6.454	7.024	4.987	8.783	7.222
26	0.4506	8.681	6.764	8.005	6.498	14.101
26	0.4516	29.432	16.889	25.701	10.040	17.816
26	0.4526	24.226	16.062	21.040	4.931	13.175
26	0.4536	8.799	4.034	6.781	7.996	35.072
26	0.4546	12.565	14.787	15.171	15.777	9.272
26	0.4556	40.840	27.811	36.371	10.450	11.815
26	0.4566	25.410	15.856	22.818	9.881	18.297
26	0.4576	18.203	19.138	22.402	9.182	21.940
26	0.4586	6.153	9.013	11.551	8.641	28.965
26	0.4596	33.111	24.490	28.509	13.056	25.116
26	0.4606	19.516	22.687	17.339	11.960	16.982
26	0.4616	29.129	22.083	28.700	16.920	30.674
26	0.4626	20.702	10.793	11.354	8.741	18.248
26	0.4636	16.640	18.492	12.967	8.367	7.674
26	0.4646	16.660	17.855	12.466	11.271	21.188
26	0.4656	15.736	8.332	11.552	4.566	20.541
26	0.4666	24.316	15.105	19.951	7.597	20.107
26	0.4676	40.879	28.553	33.279	21.376	24.965
26	0.4686	41.085	28.128	33.880	21.828	24.890
26	0.4696	27.825	20.404	23.502	27.117	24.273
26	0.4706	23.959	16.598	18.560	14.765	25.722
26	0.4716	21.361	19.854	16.854	26.024	26.465
26	0.4726	24.901	9.750	16.564	13.186	17.794
26	0.4736	21.457	9.286	17.576	11.636	20.014
26	0.4746	21.133	14.453	22.418	20.861	23.589
26	0.4756	34.450	27.068	31.267	34.805	21.826
26	0.4766	18.735	2.811	17.626	23.027	19.360
26	0.4776	26.792	14.696	27.654	20.683	17.673
26	0.4786	35.793	19.724	26.298	17.141	20.773
26	0.4796	23.161	22.023	15.859	12.239	14.071
26	0.4806	31.651	33.886	31.571	26.971	21.074
26	0.4816	26.236	19.264	22.247	16.345	20.209
26	0.4826	14.728	9.733	12.985	14.311	21.113
26	0.4836	18.742	10.153	19.138	14.954	21.786
26	0.4846	11.351	8.022	13.685	8.716	20.284
26	0.4856	17.490	10.882	16.131	10.915	19.166
26	0.4866	18.815	6.397	13.882	19.634	30.129
26	0.4876	14.804	9.828	12.608	20.636	36.068
26	0.4886	5.498	9.541	8.212	19.997	14.968
26	0.4896	15.119	14.121	13.411	26.375	16.898
26	0.4906	35.066	23.631	27.690	27.050	24.859

26	0.4916	23.090	17.371	18.122	20.232	17.076
26	0.4926	22.303	12.383	23.896	10.006	7.912
26	0.4936	28.466	21.933	27.489	11.516	16.070
26	0.4946	23.057	17.570	19.378	15.777	18.980
26	0.4956	27.011	13.789	20.382	20.019	18.284
26	0.4966	24.392	7.881	15.669	25.675	20.736
26	0.4976	18.789	7.235	15.038	15.792	12.553
26	0.4986	16.687	16.745	19.057	22.164	8.377
26	0.4996	13.697	10.109	16.178	14.515	11.412
26	0.5006	13.693	10.106	16.175	14.517	11.413
26	0.5016	0.000	0.000	0.000	0.000	0.000
27	0.0020	0.000	0.000	0.000	0.000	0.000
27	0.0030	13.017	6.656	11.348	12.621	18.271
27	0.0040	9.662	12.244	8.978	24.501	23.572
27	0.0050	3.566	7.368	3.462	4.689	7.351
27	0.0060	7.850	5.122	6.217	5.384	13.528
27	0.0070	14.158	7.521	11.114	15.128	16.324
27	0.0080	11.664	10.525	8.591	10.938	20.434
27	0.0090	25.022	18.580	21.880	11.094	12.579
27	0.0100	12.551	11.665	10.444	19.981	20.258
27	0.0110	15.370	13.742	15.110	13.738	12.367
27	0.0120	14.516	12.153	13.556	9.077	12.533
27	0.0130	24.290	16.533	13.331	12.409	26.074
27	0.0140	16.356	14.591	12.646	7.327	12.442
27	0.0150	11.032	10.360	10.145	14.948	12.527
27	0.0160	15.669	16.305	17.651	9.969	15.054
27	0.0170	24.751	10.701	23.617	15.954	16.571
27	0.0180	13.000	13.160	11.200	7.815	6.439
27	0.0190	13.933	14.420	12.718	12.676	16.322
27	0.0200	13.758	15.772	12.330	9.406	11.809
27	0.0210	9.563	8.213	9.693	7.277	25.240
27	0.0220	7.324	10.097	10.586	8.702	13.536
27	0.0230	6.719	9.943	10.091	10.720	19.464
27	0.0240	14.303	12.452	13.110	10.636	15.573
27	0.0250	17.842	17.984	17.847	9.815	14.665
27	0.0260	18.496	15.323	15.613	17.250	12.409
27	0.0270	13.265	14.128	12.665	14.340	9.880
27	0.0280	17.187	21.140	18.841	11.199	14.848
27	0.0290	12.341	19.685	15.452	15.008	19.321
27	0.0300	10.588	19.702	10.953	19.046	7.932
27	0.0310	8.453	12.170	9.666	13.965	13.534
27	0.0320	8.393	13.990	7.203	8.716	24.870
27	0.0330	10.448	7.936	5.996	17.726	34.180
27	0.0340	7.844	11.003	5.820	18.067	28.391
27	0.0350	7.735	5.654	3.389	13.480	23.830
27	0.0360	5.013	3.652	4.197	17.120	29.537
27	0.0370	12.343	11.355	9.024	23.054	26.506
27	0.0380	7.570	7.586	5.946	17.371	6.562
27	0.0390	4.062	10.966	4.265	20.064	5.168
27	0.0400	17.401	8.462	11.337	18.270	4.731

27	0.0410	27.113	34.577	25.204	22.064	15.022
27	0.0420	13.633	27.769	14.584	22.952	14.153
27	0.0430	12.645	18.610	11.692	19.734	17.105
27	0.0440	10.897	10.085	10.214	20.070	9.812
27	0.0450	15.388	15.716	14.425	15.929	15.589
27	0.0460	9.271	19.505	7.803	17.220	15.529
27	0.0470	9.130	8.451	7.717	10.394	14.625
27	0.0480	12.152	11.942	9.936	10.757	8.409
27	0.0490	9.934	10.660	7.450	18.448	20.427
27	0.0500	12.186	15.684	9.325	14.986	19.420
27	0.0510	20.504	14.731	16.282	13.248	10.546
27	0.0520	23.402	15.420	20.242	18.404	12.957
27	0.0530	28.897	19.625	18.619	7.869	17.215
27	0.0540	16.407	14.674	12.077	10.474	16.990
27	0.0550	20.773	17.725	12.340	18.372	13.026
27	0.0560	6.505	10.527	4.876	12.913	10.191
27	0.0570	12.989	14.803	15.723	13.372	16.435
27	0.0580	9.677	12.069	8.605	18.077	17.118
27	0.0590	14.403	11.817	12.354	10.786	9.523
27	0.0600	15.561	7.457	9.870	19.804	17.099
27	0.0610	11.971	19.381	15.221	18.283	8.278
27	0.0620	13.885	24.592	19.585	16.207	7.880
27	0.0630	10.594	18.847	15.749	23.594	13.005
27	0.0640	22.856	22.467	19.102	16.603	9.455
27	0.0650	11.893	16.829	10.958	13.988	18.846
27	0.0660	24.471	20.129	16.973	23.685	12.955
27	0.0670	18.332	10.509	14.254	30.064	19.058
27	0.0680	22.788	25.999	23.791	21.398	22.867
27	0.0690	15.058	27.150	19.197	7.784	8.831
27	0.0700	8.831	18.068	11.795	9.197	7.100
27	0.0710	6.440	11.376	9.391	11.357	10.503
27	0.0720	8.757	11.490	8.525	7.102	13.981
27	0.0730	6.427	12.676	8.124	1.306	4.804
27	0.0740	26.822	19.910	22.533	18.627	15.831
27	0.0750	15.456	15.274	17.607	14.344	9.911
27	0.0760	11.776	20.864	16.253	20.557	17.493
27	0.0770	11.173	13.223	11.425	19.728	7.874
27	0.0780	14.855	9.348	10.731	7.510	9.866
27	0.0790	11.157	11.606	9.521	8.504	11.451
27	0.0800	20.261	23.638	17.483	19.073	16.808
27	0.0810	14.820	11.117	13.530	6.377	12.842
27	0.0820	13.173	17.493	12.202	12.125	8.687
27	0.0830	15.173	17.056	11.528	15.781	14.344
27	0.0840	14.625	15.555	12.987	12.042	10.671
27	0.0850	6.557	5.644	5.834	7.287	3.542
27	0.0860	16.423	15.741	12.761	13.973	6.696
27	0.0870	11.817	12.421	8.136	16.602	10.524
27	0.0880	4.729	13.505	10.027	20.562	13.906
27	0.0890	2.647	11.453	7.136	19.923	8.730
27	0.0900	6.926	17.427	8.032	18.232	7.960

27	0.0910	10.447	13.561	7.527	10.413	15.048
27	0.0920	5.279	9.786	4.376	10.567	8.177
27	0.0930	15.183	12.734	9.614	13.626	7.687
27	0.0940	7.594	11.394	6.813	15.800	13.153
27	0.0950	7.544	17.682	11.720	28.892	6.679
27	0.0960	7.161	10.599	7.445	30.044	17.292
27	0.0970	6.892	9.153	5.990	18.145	14.673
27	0.0980	22.091	12.860	20.643	18.457	12.048
27	0.0990	12.237	10.138	12.382	15.635	10.379
27	0.1000	13.728	9.061	8.108	14.781	18.618
27	0.1010	10.939	15.371	11.614	13.255	6.235
27	0.1020	6.939	7.279	6.402	11.576	6.232
27	0.1030	16.769	12.381	11.350	8.495	19.442
27	0.1040	5.630	5.688	3.670	10.517	17.176
27	0.1050	4.847	4.977	3.464	11.124	8.461
27	0.1060	4.502	3.713	2.649	10.028	13.464
27	0.1070	8.946	6.929	6.732	14.755	11.966
27	0.1080	11.361	7.936	9.984	13.251	13.027
27	0.1090	12.865	15.418	13.844	9.528	15.317
27	0.1100	12.794	15.184	14.530	12.637	14.155
27	0.1110	10.861	13.072	12.248	17.673	9.786
27	0.1120	16.964	17.811	12.236	26.683	9.547
27	0.1130	12.242	12.267	15.539	10.388	9.551
27	0.1140	11.854	18.928	13.100	12.978	12.874
27	0.1150	21.793	10.156	16.859	10.677	14.988
27	0.1160	19.404	10.838	13.464	18.195	15.275
27	0.1170	8.488	11.970	9.862	14.887	10.329
27	0.1180	6.433	9.607	9.891	13.048	7.034
27	0.1190	18.145	15.402	18.709	14.892	10.463
27	0.1200	14.642	14.915	17.879	21.525	9.036
27	0.1210	16.858	16.104	17.765	3.915	10.175
27	0.1220	14.091	11.390	9.553	7.187	12.946
27	0.1230	7.326	8.429	7.115	10.034	7.939
27	0.1240	4.765	4.162	4.510	3.510	9.434
27	0.1250	19.019	10.506	11.574	11.065	15.891
27	0.1260	13.553	10.083	11.822	16.286	8.806
27	0.1270	11.633	18.215	13.153	23.964	9.055
27	0.1280	13.566	15.120	12.065	26.849	13.075
27	0.1290	26.339	25.163	20.058	15.319	14.152
27	0.1300	15.257	18.593	6.216	22.571	11.767
27	0.1310	11.363	11.279	6.301	10.968	7.520
27	0.1320	14.065	13.273	8.755	11.451	5.628
27	0.1330	25.086	18.314	18.360	8.453	15.533
27	0.1340	16.898	16.079	18.829	6.699	9.942
27	0.1350	18.512	16.896	22.951	12.561	14.059
27	0.1360	17.289	16.075	18.258	12.264	8.639
27	0.1370	9.844	8.080	8.833	6.828	5.351
27	0.1380	16.925	14.341	15.464	8.924	8.634
27	0.1390	11.423	9.022	15.635	6.478	8.426
27	0.1400	9.971	13.756	9.585	3.207	2.062

27	0.1410	18.936	14.040	15.333	8.880	10.970
27	0.1420	18.807	19.407	20.829	19.828	6.507
27	0.1430	14.280	18.553	11.935	15.921	19.042
27	0.1440	24.597	17.846	19.932	15.662	5.660
27	0.1450	20.826	22.202	17.466	14.010	4.969
27	0.1460	16.111	15.817	16.474	13.340	16.798
27	0.1470	22.747	18.770	12.748	13.409	15.013
27	0.1480	18.902	20.519	17.934	12.779	10.138
27	0.1490	10.408	16.067	12.209	17.406	5.056
27	0.1500	9.677	11.717	11.055	15.766	3.973
27	0.1510	12.292	16.913	14.449	16.215	11.765
27	0.1520	12.175	15.580	12.389	17.372	12.264
27	0.1530	19.491	24.450	21.321	21.620	15.290
27	0.1540	12.923	16.491	18.108	25.650	9.740
27	0.1550	24.811	22.725	20.782	27.832	11.797
27	0.1560	24.384	15.159	21.458	16.230	9.189
27	0.1570	23.792	25.773	19.922	21.392	13.068
27	0.1580	12.487	11.340	13.034	9.574	9.526
27	0.1590	20.899	18.352	16.656	15.212	13.287
27	0.1600	24.303	18.811	16.290	7.980	8.427
27	0.1610	15.029	12.282	10.898	17.793	14.285
27	0.1620	22.671	23.146	19.035	9.231	16.844
27	0.1630	18.197	22.605	15.126	10.123	20.729
27	0.1640	14.144	23.014	11.331	16.175	19.840
27	0.1650	17.657	15.845	11.968	13.796	8.898
27	0.1660	6.866	8.354	7.318	15.562	8.190
27	0.1670	5.141	10.098	10.083	8.262	7.882
27	0.1680	11.346	9.525	15.132	6.991	8.674
27	0.1690	12.525	10.010	12.098	11.610	26.619
27	0.1700	14.591	13.630	12.454	16.542	27.672
27	0.1710	23.766	22.879	23.957	14.506	10.475
27	0.1720	17.471	19.241	18.427	16.637	14.062
27	0.1730	17.458	19.246	18.433	16.722	14.048
27	0.1740	19.738	19.456	17.594	16.379	16.770
27	0.1750	15.763	14.847	13.743	8.397	6.573
27	0.1760	14.936	15.675	13.035	15.944	8.587
27	0.1770	16.466	12.213	15.997	18.148	10.419
27	0.1780	18.101	16.748	13.826	27.526	17.283
27	0.1790	22.663	12.696	19.182	12.482	12.511
27	0.1800	16.070	11.123	16.745	16.774	14.007
27	0.1810	17.136	16.574	18.762	17.716	19.783
27	0.1820	18.229	10.967	19.925	21.039	10.770
27	0.1830	24.301	29.724	20.384	32.386	19.099
27	0.1840	10.318	9.075	14.256	21.594	34.208
27	0.1850	23.151	22.875	21.983	20.725	25.505
27	0.1860	17.119	15.199	17.669	17.516	20.089
27	0.1870	15.852	13.346	16.051	22.514	17.176
27	0.1880	13.072	17.366	17.659	23.193	17.564
27	0.1890	18.833	17.766	20.790	16.637	7.669
27	0.1900	13.050	8.179	12.893	5.791	5.462

27	0.1910	14.289	16.364	16.619	9.636	4.108
27	0.1920	11.463	13.399	12.408	11.399	17.529
27	0.1930	16.486	14.258	15.959	7.135	18.066
27	0.1940	15.596	17.166	15.626	15.248	10.446
27	0.1950	10.057	10.840	11.276	13.405	5.843
27	0.1960	15.110	17.302	18.318	14.380	7.681
27	0.1970	18.489	17.244	14.536	21.584	13.664
27	0.1980	10.733	11.506	10.545	11.779	24.967
27	0.1990	12.343	17.204	13.620	18.005	22.984
27	0.2000	35.592	27.817	31.346	19.746	10.215
27	0.2010	32.054	20.655	26.094	8.331	21.173
27	0.2020	17.545	13.357	15.789	12.225	6.458
27	0.2030	26.472	23.861	22.110	18.892	20.089
27	0.2040	10.748	9.504	7.876	15.010	8.552
27	0.2050	11.572	11.236	14.171	14.001	5.035
27	0.2060	14.559	19.578	17.771	13.263	11.412
27	0.2070	25.949	18.195	17.554	12.698	14.119
27	0.2080	8.704	13.034	11.876	17.359	10.530
27	0.2090	10.941	17.161	9.254	14.795	22.236
27	0.2100	13.220	11.610	10.993	23.264	14.210
27	0.2110	7.988	9.639	11.491	12.154	14.769
27	0.2120	13.645	12.730	13.392	16.247	16.155
27	0.2130	11.393	10.338	14.105	15.947	10.980
27	0.2140	18.885	15.459	17.152	16.798	8.750
27	0.2150	8.979	9.851	11.305	16.146	7.754
27	0.2160	9.744	7.247	14.957	25.393	14.257
27	0.2170	16.494	18.768	12.881	16.716	11.562
27	0.2180	8.261	11.243	13.594	16.538	13.903
27	0.2190	22.713	27.228	20.616	20.098	12.332
27	0.2200	16.131	16.257	14.893	17.592	9.914
27	0.2210	7.769	8.401	11.231	9.857	15.486
27	0.2220	14.227	17.528	10.765	16.187	10.154
27	0.2230	9.605	11.438	11.499	23.392	10.946
27	0.2240	18.487	23.792	19.824	22.995	24.762
27	0.2250	14.080	16.651	16.689	13.374	13.190
27	0.2260	15.679	17.264	16.398	7.594	8.077
27	0.2270	22.301	15.988	19.461	12.785	8.874
27	0.2280	27.252	11.037	15.887	13.004	9.519
27	0.2290	19.243	6.517	13.575	15.694	19.465
27	0.2300	21.732	16.246	18.954	13.454	17.855
27	0.2310	14.603	13.336	12.468	22.375	16.312
27	0.2320	21.439	19.102	21.719	20.308	13.016
27	0.2330	7.463	10.184	11.510	4.486	5.134
27	0.2340	11.684	12.788	16.532	13.136	9.733
27	0.2350	17.905	17.193	24.145	18.522	10.521
27	0.2360	16.211	17.140	14.333	15.963	4.665
27	0.2370	5.742	13.267	10.010	20.809	20.379
27	0.2380	10.369	11.930	10.138	20.532	13.885
27	0.2390	10.299	11.892	10.107	20.381	13.902
27	0.2400	24.258	24.117	26.300	18.431	15.800

27	0.2410	24.108	24.036	26.164	18.168	15.850
27	0.2420	23.957	23.954	26.027	17.908	15.900
27	0.2430	9.344	8.052	8.660	6.423	19.900
27	0.2440	10.533	6.882	11.868	7.993	8.035
27	0.2450	8.340	7.812	11.508	10.846	9.172
27	0.2460	8.850	8.647	16.280	14.870	16.441
27	0.2470	7.033	9.238	13.289	18.639	12.539
27	0.2480	8.699	9.564	6.111	15.871	10.040
27	0.2490	12.217	9.567	11.252	17.448	7.433
27	0.2500	12.225	9.516	11.215	17.408	7.449
27	0.2510	11.970	11.163	8.130	20.890	10.133
27	0.2520	8.104	11.085	9.411	19.521	20.942
27	0.2530	11.599	9.089	7.503	23.930	16.921
27	0.2540	8.824	12.515	11.070	17.517	15.790
27	0.2550	8.931	12.605	11.166	17.394	15.931
27	0.2560	5.149	15.298	11.469	18.830	13.160
27	0.2570	5.444	8.067	10.655	8.799	13.623
27	0.2580	14.736	12.184	12.196	21.152	10.375
27	0.2590	10.275	11.559	13.474	20.618	12.278
27	0.2600	11.441	10.952	13.970	13.742	14.310
27	0.2610	12.543	15.208	10.393	23.390	11.966
27	0.2620	19.297	13.927	12.752	22.412	14.019
27	0.2630	19.531	14.146	12.985	22.368	14.075
27	0.2640	19.743	14.351	13.192	22.323	14.135
27	0.2650	25.856	14.032	18.399	22.541	19.686
27	0.2660	8.317	8.561	7.799	16.269	17.991
27	0.2670	15.189	19.120	21.215	13.417	14.477
27	0.2680	15.873	16.106	18.639	12.418	13.952
27	0.2690	13.493	15.297	21.528	13.835	11.444
27	0.2700	8.621	12.820	8.602	15.059	13.825
27	0.2710	18.545	20.527	9.837	19.421	15.184
27	0.2720	15.195	7.926	16.066	24.913	18.172
27	0.2730	10.323	5.143	11.386	8.384	6.384
27	0.2740	5.994	11.326	10.544	11.699	12.692
27	0.2750	6.520	9.002	9.124	15.366	10.020
27	0.2760	11.652	9.538	13.610	28.566	18.934
27	0.2770	14.879	13.552	16.334	12.497	16.523
27	0.2780	9.465	10.138	9.723	10.972	20.880
27	0.2790	7.270	8.296	7.659	5.873	10.174
27	0.2800	4.643	8.556	10.887	6.624	11.927
27	0.2810	12.540	14.326	15.487	20.184	21.253
27	0.2820	13.188	12.799	7.763	7.918	26.074
27	0.2830	10.238	14.982	9.856	15.185	21.945
27	0.2840	13.609	6.301	15.671	18.721	15.325
27	0.2850	17.110	11.526	16.782	13.159	17.844
27	0.2860	17.136	11.526	16.801	13.115	17.718
27	0.2870	13.447	12.590	13.665	17.518	6.931
27	0.2880	7.493	8.159	8.704	28.347	14.143
27	0.2890	12.970	19.249	15.645	26.722	12.764
27	0.2900	11.641	15.091	11.635	27.853	9.487

27	0.2910	8.158	11.925	6.238	18.772	6.182
27	0.2920	13.132	15.036	12.512	15.251	5.636
27	0.2930	16.733	15.559	17.482	14.001	7.198
27	0.2940	8.378	8.971	4.982	12.452	7.634
27	0.2950	8.648	8.169	6.695	8.479	8.411
27	0.2960	8.191	9.553	7.622	8.104	15.626
27	0.2970	21.912	14.814	21.581	19.078	9.794
27	0.2980	21.932	14.789	21.595	19.192	9.843
27	0.2990	24.211	13.306	24.104	19.642	8.312
27	0.3000	15.546	16.913	11.544	12.972	16.932
27	0.3010	17.072	10.167	8.076	22.002	31.471
27	0.3020	7.490	10.145	5.556	17.623	21.354
27	0.3030	7.424	10.134	5.502	17.536	21.456
27	0.3040	18.292	19.494	15.275	17.137	21.166
27	0.3050	16.170	12.434	10.912	13.295	14.085
27	0.3060	16.181	12.416	10.918	13.220	14.144
27	0.3070	13.545	10.990	11.438	16.069	17.373
27	0.3080	11.713	5.653	10.110	12.662	19.635
27	0.3090	21.605	7.895	12.573	17.382	11.834
27	0.3100	12.240	14.356	9.630	11.911	16.370
27	0.3110	13.114	14.605	6.681	21.529	16.994
27	0.3120	6.716	5.813	7.765	6.718	10.483
27	0.3130	17.453	16.007	11.899	21.271	9.149
27	0.3140	21.867	17.830	13.578	18.081	31.157
27	0.3150	17.035	23.581	11.072	21.552	37.678
27	0.3160	13.523	13.414	14.569	17.462	22.562
27	0.3170	23.433	22.739	18.603	22.293	10.452
27	0.3180	15.812	21.788	15.893	15.322	20.378
27	0.3190	10.788	7.399	4.999	9.923	8.534
27	0.3200	15.923	11.019	13.442	20.024	19.906
27	0.3210	10.608	9.524	10.391	12.089	8.923
27	0.3220	9.506	7.651	8.656	21.452	14.020
27	0.3230	25.806	21.699	16.702	16.305	24.011
27	0.3240	26.498	24.206	17.212	14.976	21.398
27	0.3250	11.551	11.919	14.010	19.857	20.600
27	0.3260	20.630	19.954	18.369	13.649	21.183
27	0.3270	20.485	13.896	18.022	15.003	18.237
27	0.3280	14.957	11.905	15.756	10.959	14.898
27	0.3290	9.592	4.958	7.171	5.087	10.475
27	0.3300	13.281	16.580	10.031	9.644	8.064
27	0.3310	16.016	19.488	13.574	17.151	18.209
27	0.3320	29.284	24.793	19.412	13.623	22.510
27	0.3330	29.275	24.811	19.380	13.665	22.509
27	0.3340	23.980	22.792	22.229	15.814	12.807
27	0.3350	25.046	15.338	19.782	16.526	12.042
27	0.3360	26.691	12.777	16.413	16.003	17.368
27	0.3370	25.242	16.455	13.458	22.593	20.321
27	0.3380	17.520	17.017	11.627	23.567	23.190
27	0.3390	21.813	20.729	19.279	16.718	23.976
27	0.3400	16.043	14.733	13.177	12.741	11.813

27	0.3410	16.893	10.250	11.917	12.537	26.997
27	0.3420	21.768	12.215	13.852	7.167	16.789
27	0.3430	11.379	13.031	7.390	10.442	15.523
27	0.3440	8.684	12.374	5.206	17.417	9.899
27	0.3450	14.217	13.250	5.624	26.761	12.144
27	0.3460	5.033	6.500	1.358	19.564	8.496
27	0.3470	13.403	13.618	9.802	24.973	14.919
27	0.3480	12.661	15.410	10.053	16.973	12.514
27	0.3490	10.698	8.875	7.333	16.336	7.240
27	0.3500	17.771	20.203	13.926	21.195	13.755
27	0.3510	15.729	12.070	10.235	17.608	16.702
27	0.3520	13.713	26.624	15.001	35.654	16.761
27	0.3530	14.363	22.030	12.458	27.522	21.088
27	0.3540	14.013	16.364	11.486	8.867	5.650
27	0.3550	16.116	17.767	13.110	14.819	15.132
27	0.3560	8.369	9.830	9.595	21.769	16.945
27	0.3570	8.779	11.437	10.843	21.288	14.080
27	0.3580	6.546	12.073	5.710	13.220	15.184
27	0.3590	14.510	27.674	21.295	20.783	21.669
27	0.3600	20.407	33.340	23.835	26.258	17.919
27	0.3610	18.763	25.099	17.820	19.284	14.726
27	0.3620	8.782	17.342	9.496	24.903	17.868
27	0.3630	13.558	14.108	12.414	12.217	17.073
27	0.3640	29.520	26.184	22.412	16.397	13.701
27	0.3650	16.594	20.324	11.039	15.256	12.943
27	0.3660	8.863	10.176	7.032	12.917	11.615
27	0.3670	6.650	11.265	11.361	8.386	19.742
27	0.3680	21.449	24.204	18.667	26.787	27.098
27	0.3690	22.312	26.214	13.647	19.986	10.931
27	0.3700	4.554	17.459	7.276	19.133	14.885
27	0.3710	8.140	10.546	11.866	14.833	8.114
27	0.3720	6.142	12.341	6.062	16.340	15.273
27	0.3730	6.301	13.502	12.027	18.169	28.600
27	0.3740	7.512	18.180	14.283	26.034	22.737
27	0.3750	11.945	16.541	10.532	17.505	15.710
27	0.3760	9.666	12.729	7.260	14.988	23.093
27	0.3770	12.651	17.094	12.094	19.394	28.034
27	0.3780	8.615	18.516	11.184	11.111	22.388
27	0.3790	9.438	23.476	12.241	16.811	15.604
27	0.3800	8.150	20.120	6.012	15.921	15.377
27	0.3810	12.376	11.764	9.111	10.009	19.239
27	0.3820	15.375	24.149	12.238	14.646	34.004
27	0.3830	6.826	5.177	3.838	9.048	23.394
27	0.3840	13.404	10.763	5.903	6.781	17.314
27	0.3850	12.184	13.152	8.966	13.966	9.375
27	0.3860	5.520	9.156	9.189	8.189	19.897
27	0.3870	17.454	11.181	11.984	12.856	19.121
27	0.3880	9.452	16.205	11.103	16.631	23.675
27	0.3890	12.628	7.737	9.114	9.355	24.240
27	0.3900	14.474	9.702	14.926	5.106	14.553

27	0.3910	14.483	11.893	11.866	4.535	26.046
27	0.3920	18.666	10.535	10.893	6.436	26.738
27	0.3930	9.648	7.624	8.777	7.267	17.793
27	0.3940	11.129	7.340	6.775	5.493	5.084
27	0.3950	21.258	22.668	19.894	13.056	20.474
27	0.3960	14.640	19.747	16.425	15.653	20.948
27	0.3970	6.415	8.948	6.805	17.103	20.457
27	0.3980	14.757	26.245	15.829	34.844	23.906
27	0.3990	4.591	13.167	10.154	22.501	19.618
27	0.4000	10.400	18.552	15.022	21.416	17.572
27	0.4010	8.105	7.947	6.518	13.560	10.458
27	0.4020	14.706	15.703	12.217	15.227	13.280
27	0.4030	11.151	12.357	3.834	22.072	19.814
27	0.4040	10.987	12.157	3.711	22.117	19.909
27	0.4050	18.548	9.497	13.822	10.892	17.628
27	0.4060	17.257	22.770	12.596	13.007	30.944
27	0.4070	9.063	9.242	5.450	13.631	19.976
27	0.4080	10.306	11.238	9.580	16.210	13.108
27	0.4090	10.234	10.071	9.191	16.828	21.024
27	0.4100	21.232	15.266	12.116	14.306	25.611
27	0.4110	11.815	19.030	13.996	16.761	13.640
27	0.4120	7.466	11.974	7.198	20.760	12.191
27	0.4130	8.985	10.882	10.068	21.322	11.713
27	0.4140	16.284	9.754	11.745	9.955	27.035
27	0.4150	8.816	12.947	10.874	19.268	21.572
27	0.4160	17.823	17.240	20.752	10.347	17.733
27	0.4170	16.250	13.154	13.677	14.170	15.906
27	0.4180	6.598	19.953	7.697	9.259	4.306
27	0.4190	13.414	11.598	7.847	16.804	18.908
27	0.4200	12.193	9.358	11.108	9.371	17.326
27	0.4210	13.780	16.407	11.778	14.295	19.110
27	0.4220	14.963	18.676	13.300	20.751	16.905
27	0.4230	20.687	19.436	13.768	29.500	18.125
27	0.4240	17.453	8.336	15.910	24.014	19.965
27	0.4250	19.905	12.586	21.080	23.895	17.334
27	0.4260	19.269	23.086	20.295	27.130	28.606
27	0.4270	10.217	11.574	8.477	11.811	15.701
27	0.4280	18.401	15.440	11.912	11.528	22.653
27	0.4290	12.925	16.526	11.510	23.114	30.555
27	0.4300	13.795	13.636	9.412	18.710	17.772
27	0.4310	11.904	15.468	8.381	15.196	14.448
27	0.4320	3.599	5.169	3.196	20.374	17.541
27	0.4330	13.555	17.275	16.572	24.344	9.065
27	0.4340	13.693	16.587	12.437	14.721	17.905
27	0.4350	16.331	22.269	14.061	20.945	23.989
27	0.4360	9.568	15.201	12.890	16.521	9.937
27	0.4370	3.480	5.163	3.280	10.191	11.219
27	0.4380	7.997	8.225	8.092	5.652	17.961
27	0.4390	2.946	3.643	2.578	5.239	9.248
27	0.4400	2.195	2.992	3.520	5.203	12.163

27	0.4410	1.000	1.000	1.000	1.000	1.000
28	0.0003	0.000	0.000	0.000	0.000	0.000
28	0.0013	16.782	15.458	16.966	9.423	7.368
28	0.0023	24.496	21.788	26.660	9.213	13.008
28	0.0033	21.139	19.515	17.912	9.616	16.939
28	0.0043	15.518	13.114	17.309	9.822	12.188
28	0.0053	15.509	13.091	17.334	9.828	12.183
28	0.0063	10.991	13.781	16.594	9.224	10.211
28	0.0073	5.284	8.331	6.877	7.955	4.792
28	0.0083	25.438	17.196	24.035	6.446	8.876
28	0.0093	24.719	13.893	23.660	6.719	10.992
28	0.0103	16.508	16.950	17.582	19.153	19.039
28	0.0113	22.415	26.626	22.045	12.881	6.679
28	0.0123	22.059	23.217	18.050	13.742	6.685
28	0.0133	25.534	20.731	20.527	7.307	4.842
28	0.0143	20.247	19.177	15.492	7.680	5.646
28	0.0153	23.693	23.690	21.683	10.909	10.425
28	0.0163	28.258	29.692	24.118	13.172	13.997
28	0.0173	12.902	20.242	14.431	24.369	20.989
28	0.0183	15.209	21.619	16.361	16.905	15.374
28	0.0193	17.872	11.143	15.879	17.692	16.041
28	0.0203	13.086	12.716	16.390	13.614	14.757
28	0.0213	14.405	13.827	15.147	11.973	7.052
28	0.0223	10.341	11.595	13.050	8.381	3.905
28	0.0233	8.902	6.013	10.185	7.809	3.729
28	0.0243	10.668	9.575	11.310	8.429	8.878
28	0.0253	17.929	21.511	18.454	14.572	11.277
28	0.0263	16.113	17.079	13.515	19.665	14.248
28	0.0273	24.562	20.495	17.807	31.036	16.288
28	0.0283	14.545	9.158	11.002	10.486	11.522
28	0.0293	9.901	11.522	10.223	8.533	10.866
28	0.0303	15.972	13.454	16.960	10.641	5.618
28	0.0313	19.264	14.056	17.137	6.353	7.211
28	0.0323	20.069	10.287	15.963	8.713	7.928
28	0.0333	30.165	18.442	29.070	13.830	10.008
28	0.0343	21.158	17.297	26.171	15.004	10.050
28	0.0353	23.671	18.439	22.949	9.482	9.147
28	0.0363	13.875	18.835	22.174	18.904	11.534
28	0.0373	17.819	17.551	22.567	21.645	8.196
28	0.0383	8.265	16.113	11.353	19.337	7.235
28	0.0393	16.164	16.799	17.498	29.417	6.988
28	0.0403	25.735	19.952	25.842	23.096	19.287
28	0.0413	17.993	13.329	17.781	11.487	13.949
28	0.0423	8.593	14.284	13.624	26.965	17.189
28	0.0433	15.306	14.561	19.168	15.940	9.359
28	0.0443	17.097	12.096	16.877	22.038	20.561
28	0.0453	14.903	7.861	14.178	20.384	20.067
28	0.0463	17.302	10.662	15.733	12.253	9.328
28	0.0473	29.909	11.847	28.034	17.572	13.545
28	0.0483	26.968	10.134	21.514	15.826	8.919

28	0.0493	19.809	20.834	20.865	23.033	13.550
28	0.0503	15.716	11.032	16.314	17.919	15.169
28	0.0513	14.314	6.835	11.545	14.462	16.574
28	0.0523	15.134	8.992	17.103	16.074	23.853
28	0.0533	14.921	8.743	16.754	16.104	23.791
28	0.0543	25.092	17.505	18.277	21.533	19.312
28	0.0553	18.905	7.698	12.923	17.591	16.352
28	0.0563	5.598	6.490	7.400	9.179	9.838
28	0.0573	4.072	6.757	7.701	14.996	13.929
28	0.0583	10.925	6.319	12.608	17.249	16.087
28	0.0593	13.179	10.892	11.717	17.355	7.248
28	0.0603	6.499	6.117	10.666	20.618	13.908
28	0.0613	17.668	17.472	14.592	21.217	14.459
28	0.0623	17.643	17.403	14.549	21.176	14.470
28	0.0633	14.993	7.188	13.557	12.938	14.880
28	0.0643	14.283	7.767	13.696	13.392	7.287
28	0.0653	15.426	13.432	16.259	11.775	13.446
28	0.0663	14.449	12.905	10.770	9.590	17.210
28	0.0673	7.509	6.841	10.966	9.487	8.273
28	0.0683	18.219	19.594	19.324	13.667	12.461
28	0.0693	9.119	10.506	10.853	12.249	9.567
28	0.0703	20.490	20.489	17.271	15.824	12.008
28	0.0713	11.722	19.119	14.324	15.776	19.549
28	0.0723	12.028	8.438	9.220	13.008	9.397
28	0.0733	14.525	9.070	14.850	24.321	24.888
28	0.0743	9.435	6.607	15.376	12.120	6.006
28	0.0753	12.269	13.279	12.255	9.532	14.884
28	0.0763	17.230	7.323	11.239	14.894	10.600
28	0.0773	12.592	7.178	13.391	15.547	24.316
28	0.0783	15.669	9.224	14.878	20.731	31.583
28	0.0793	10.229	6.440	12.577	17.792	12.389
28	0.0803	7.967	4.414	8.974	16.494	23.536
28	0.0813	15.554	6.433	10.622	13.949	29.914
28	0.0823	13.447	11.227	12.024	9.622	18.655
28	0.0833	29.226	18.384	30.526	13.744	9.855
28	0.0843	8.856	2.052	6.757	16.407	11.960
28	0.0853	18.633	6.772	13.163	10.724	8.767
28	0.0863	12.744	6.549	8.960	17.193	17.497
28	0.0873	13.557	6.864	12.946	14.961	18.889
28	0.0883	14.620	11.269	13.131	18.644	16.289
28	0.0893	11.566	13.884	13.499	16.451	16.303
28	0.0903	11.053	4.941	9.208	11.591	22.218
28	0.0913	14.573	9.133	14.272	14.706	23.965
28	0.0923	15.974	17.350	17.183	11.407	13.870
28	0.0933	11.256	9.998	10.577	9.910	15.666
28	0.0943	10.645	11.104	10.606	19.725	12.536
28	0.0953	7.812	9.196	9.500	10.997	12.120
28	0.0963	17.386	13.687	11.750	8.475	18.745
28	0.0973	14.833	10.434	11.555	9.915	7.702
28	0.0983	14.267	14.639	17.698	15.220	9.904

28	0.0993	9.586	7.227	8.820	16.737	3.318
28	0.1003	6.366	6.005	7.047	7.270	5.560
28	0.1013	7.009	8.147	10.835	16.766	16.064
28	0.1023	8.741	10.270	12.326	20.903	25.691
28	0.1033	16.144	20.388	15.378	16.999	14.537
28	0.1043	11.309	20.562	17.483	18.802	12.384
28	0.1053	7.726	4.791	9.643	10.526	9.663
28	0.1063	4.692	6.831	4.611	12.848	9.120
28	0.1073	11.029	9.516	10.398	12.897	10.181
28	0.1083	16.077	4.329	14.863	5.033	5.719
28	0.1093	14.102	3.671	11.620	10.519	5.113
28	0.1103	4.399	5.319	6.250	7.228	2.499
28	0.1113	9.281	9.291	13.491	7.493	7.464
28	0.1123	15.975	13.361	23.113	2.139	11.043
28	0.1133	15.315	8.633	16.491	16.921	7.422
28	0.1143	21.100	13.193	21.845	7.504	9.137
28	0.1153	8.699	13.771	14.891	15.267	6.156
28	0.1163	4.224	6.486	7.860	11.416	10.429
28	0.1173	7.733	9.472	9.924	17.806	14.725
28	0.1183	9.646	10.479	10.290	14.551	12.586
28	0.1193	10.370	11.137	12.877	11.203	12.678
28	0.1203	7.712	7.094	7.182	21.782	14.569
28	0.1213	7.044	5.773	5.944	15.285	9.216
28	0.1223	16.822	14.993	19.264	9.754	12.732
28	0.1233	13.205	11.386	16.919	15.224	14.309
28	0.1243	13.004	11.173	17.732	20.916	7.889
28	0.1253	11.258	13.977	10.509	21.563	9.651
28	0.1263	10.013	10.340	12.705	11.533	6.148
28	0.1273	6.118	7.523	8.290	12.469	5.172
28	0.1283	14.788	7.055	14.377	15.922	15.165
28	0.1293	13.353	8.695	14.993	11.750	18.443
28	0.1303	19.982	9.325	18.555	18.809	7.306
28	0.1313	14.791	10.913	13.977	5.881	6.090
28	0.1323	12.029	17.510	18.295	6.049	7.298
28	0.1333	9.680	12.489	14.713	9.903	8.677
28	0.1343	19.554	22.631	31.777	13.356	7.116
28	0.1353	12.749	11.975	13.682	14.480	13.149
28	0.1363	13.015	14.756	18.327	11.559	10.948
28	0.1373	13.848	11.691	17.653	7.174	11.492
28	0.1383	19.392	18.971	29.688	18.498	11.227
28	0.1393	13.396	14.514	18.014	15.700	13.542
28	0.1403	23.634	20.195	27.949	12.814	4.778
28	0.1413	10.024	10.099	14.140	13.582	6.405
28	0.1423	7.016	5.419	8.631	5.294	4.865
28	0.1433	12.161	10.394	12.358	14.370	9.655
28	0.1443	9.190	7.756	8.153	14.197	13.068
28	0.1453	22.414	17.584	24.009	21.620	9.572
28	0.1463	14.143	5.402	11.439	15.765	7.990
28	0.1473	13.189	13.960	13.246	16.571	16.691
28	0.1483	18.714	18.397	20.273	13.939	8.344

28	0.1493	5.428	3.750	4.496	15.532	9.925
28	0.1503	9.008	9.144	12.871	6.800	5.955
28	0.1513	7.451	6.648	9.284	7.648	6.645
28	0.1523	3.775	3.369	5.493	7.464	2.506
28	0.1533	16.588	22.024	19.058	14.048	10.167
28	0.1543	10.149	13.799	8.898	10.114	4.774
28	0.1553	7.758	11.148	10.079	8.553	3.916
28	0.1563	15.024	9.965	13.843	12.260	3.869
28	0.1573	10.691	12.751	15.356	17.236	10.403
28	0.1583	17.268	23.340	22.737	18.196	3.895
28	0.1593	9.418	7.325	11.102	16.254	11.588
28	0.1603	12.916	17.214	19.405	22.269	8.708
28	0.1613	12.583	13.969	10.656	12.254	6.227
28	0.1623	7.900	10.815	8.498	5.870	8.636
28	0.1633	4.429	5.714	5.716	7.563	9.235
28	0.1643	10.971	8.281	13.739	16.009	14.832
28	0.1653	10.972	8.231	13.768	15.961	14.861
28	0.1663	4.164	6.127	5.524	9.636	10.736
28	0.1673	11.427	17.239	13.110	22.197	13.038
28	0.1683	11.345	17.226	13.045	22.158	12.991
28	0.1693	11.265	17.213	12.981	22.118	12.951
28	0.1703	9.302	16.449	11.832	14.667	6.708
28	0.1713	9.536	10.367	14.106	16.543	6.059
28	0.1723	13.807	15.846	18.946	12.131	13.098
28	0.1733	6.678	10.038	9.174	11.736	12.739
28	0.1743	7.453	12.163	11.709	11.486	10.980
28	0.1753	19.073	23.501	24.143	18.554	8.038
28	0.1763	19.047	23.430	24.102	18.539	8.023
28	0.1773	12.029	5.074	11.237	12.860	16.131
28	0.1783	10.790	7.933	7.860	9.946	6.730
28	0.1793	5.877	6.267	3.757	5.240	6.196
28	0.1803	4.037	8.529	3.114	8.793	2.745
28	0.1813	11.855	16.567	21.410	17.641	12.217
28	0.1823	20.944	10.754	22.038	14.796	20.658
28	0.1833	15.629	11.966	20.055	9.572	17.673
28	0.1843	10.353	9.225	12.704	9.327	6.205
28	0.1853	17.301	23.858	21.767	18.168	7.682
28	0.1863	11.806	13.013	9.601	15.695	13.756
28	0.1873	13.245	14.525	10.058	19.458	10.822
28	0.1883	13.075	14.509	9.947	19.733	10.838
28	0.1893	7.810	9.434	7.773	9.537	6.933
28	0.1903	3.287	3.661	1.890	8.287	5.391
28	0.1913	1.823	1.594	0.068	5.856	4.748
28	0.1923	7.709	5.435	7.061	13.228	12.142
28	0.1933	15.231	15.182	14.728	24.162	19.373
28	0.1943	14.819	13.741	15.932	15.637	14.843
28	0.1953	15.857	15.873	22.073	10.289	18.132
28	0.1963	6.008	9.126	12.035	6.228	11.438
28	0.1973	11.740	10.023	13.952	11.402	11.535
28	0.1983	8.593	10.107	9.671	5.920	5.246

28	0.1993	10.663	10.279	10.552	10.064	4.800
28	0.2003	12.034	12.117	9.611	7.046	10.213
28	0.2013	15.559	14.261	16.889	4.356	19.404
28	0.2023	15.125	17.220	14.408	11.904	12.866
28	0.2033	6.787	7.923	10.878	7.481	7.547
28	0.2043	11.990	10.377	10.516	6.891	8.203
28	0.2053	13.083	7.209	7.935	20.214	11.061
28	0.2063	12.908	5.932	10.139	8.601	9.320
28	0.2073	15.878	9.228	10.054	12.024	7.196
28	0.2083	13.082	10.931	9.550	11.437	5.366
28	0.2093	10.555	15.539	10.517	15.276	14.460
28	0.2103	13.168	9.939	7.680	15.960	7.898
28	0.2113	4.293	6.261	4.779	9.720	3.123
28	0.2123	5.149	3.466	3.776	5.513	4.362
28	0.2133	4.693	10.783	6.569	17.660	6.391
28	0.2143	11.621	16.740	13.001	15.933	15.669
28	0.2153	13.508	9.664	12.363	11.939	7.597
28	0.2163	9.059	4.099	8.206	13.300	12.355
28	0.2173	8.529	11.111	9.456	13.908	6.601
28	0.2183	8.730	13.194	11.806	14.650	7.049
28	0.2193	9.758	14.344	10.335	17.258	9.759
28	0.2203	7.819	16.925	13.219	20.622	13.420
28	0.2213	6.394	6.260	7.171	14.378	7.804
28	0.2223	4.768	6.724	5.745	12.237	6.355
28	0.2233	6.070	8.363	9.714	10.956	10.210
28	0.2243	9.534	15.109	12.095	16.382	14.934
28	0.2253	8.447	4.682	6.058	14.176	13.875
28	0.2263	10.158	20.683	16.338	12.086	10.993
28	0.2273	9.245	21.199	13.070	15.016	12.808
28	0.2283	12.647	22.762	18.205	17.893	16.852
28	0.2293	8.232	14.320	6.371	9.443	7.965
28	0.2303	4.420	8.128	7.251	8.464	6.978
28	0.2313	13.960	13.837	15.617	14.508	12.411
28	0.2323	8.907	12.704	11.611	12.005	12.342
28	0.2333	10.250	14.078	12.808	8.897	15.268
28	0.2343	6.895	18.154	8.980	16.757	8.784
28	0.2353	10.137	16.162	14.965	11.122	9.408
28	0.2363	6.561	12.875	9.648	10.607	12.148
28	0.2373	12.999	10.389	14.788	7.813	16.162
28	0.2383	16.268	24.914	19.493	13.447	12.612
28	0.2393	11.833	20.467	12.924	15.400	6.818
28	0.2403	15.845	7.889	13.595	8.382	22.170
28	0.2413	12.030	8.171	12.101	9.063	10.416
28	0.2423	17.349	27.193	21.389	14.214	10.452
28	0.2433	12.934	29.212	23.489	12.974	15.634
28	0.2443	23.021	17.831	20.989	22.507	7.691
28	0.2453	9.825	13.701	8.045	19.467	8.603
28	0.2463	15.731	25.603	17.243	15.424	11.185
28	0.2473	21.939	19.104	21.417	18.776	15.886
28	0.2483	26.414	18.687	22.283	12.876	13.549

28	0.2493	23.232	18.709	19.589	27.466	10.157
28	0.2503	4.803	7.966	8.990	18.404	18.755
28	0.2513	13.762	20.330	20.911	27.834	15.337
28	0.2523	14.167	26.754	18.458	24.805	19.978
28	0.2533	15.813	31.005	31.327	26.796	14.236
28	0.2543	30.883	39.097	41.689	28.397	10.869
28	0.2553	30.806	38.950	41.539	28.426	10.877
28	0.2563	17.979	32.589	29.297	27.236	27.886
28	0.2573	10.781	20.828	12.981	21.233	14.882
28	0.2583	9.109	12.923	11.908	16.162	6.698
28	0.2593	17.348	10.035	10.871	13.134	20.882
28	0.2603	18.542	12.668	14.449	9.793	20.069
28	0.2613	11.199	22.568	14.460	16.672	8.681
28	0.2623	10.557	17.930	9.139	15.969	8.995
28	0.2633	6.181	16.480	7.947	9.536	0.788
28	0.2643	8.042	21.348	12.168	14.365	6.462
28	0.2653	14.445	14.748	8.695	16.986	11.872
28	0.2663	11.941	13.788	10.550	8.685	10.490
28	0.2673	13.751	16.684	16.863	10.674	10.889
28	0.2683	9.836	11.421	10.316	9.778	10.468
28	0.2693	12.030	15.231	12.016	10.635	11.364
28	0.2703	14.081	15.852	19.135	18.870	29.491
28	0.2713	9.710	8.025	6.254	14.136	15.586
28	0.2723	12.581	25.599	12.907	23.384	16.462
28	0.2733	10.135	18.547	6.711	17.855	8.743
28	0.2743	11.575	14.200	12.494	30.360	19.140
28	0.2753	6.463	11.276	12.442	14.140	16.969
28	0.2763	10.087	14.623	9.940	25.075	12.900
28	0.2773	19.996	22.153	17.931	18.490	15.097
28	0.2783	15.508	24.227	16.861	24.077	20.244
28	0.2793	17.546	26.973	20.595	32.016	19.099
28	0.2803	13.021	27.558	17.197	23.479	15.205
28	0.2813	11.745	20.021	15.855	18.715	10.933
28	0.2823	9.427	19.959	16.616	17.971	11.517
28	0.2833	7.666	21.101	16.947	27.310	16.272
28	0.2843	9.525	21.543	17.015	13.944	14.618
28	0.2853	10.296	21.542	13.998	20.503	26.165
28	0.2863	12.857	14.605	12.231	19.029	10.683
28	0.2873	12.149	19.034	12.533	19.803	10.832
28	0.2883	13.434	22.052	17.138	26.758	19.795
28	0.2893	14.755	19.905	10.391	26.602	20.243
28	0.2903	16.743	8.405	12.780	12.654	13.766
28	0.2913	22.606	15.296	16.247	17.633	16.491
28	0.2923	10.727	8.438	5.698	15.470	19.737
28	0.2933	10.794	8.548	5.795	15.305	19.644
28	0.2943	19.113	10.060	9.977	14.279	21.991
28	0.2953	12.071	7.092	9.254	15.898	18.201
28	0.2963	22.146	8.478	14.392	14.151	16.891
28	0.2973	17.538	18.341	22.348	14.532	5.230
28	0.2983	12.372	9.661	17.514	20.018	15.305

28	0.2993	26.074	17.199	19.878	13.916	10.376
28	0.3003	18.642	20.683	15.029	22.696	8.172
28	0.3013	13.626	15.452	14.051	23.324	12.732
28	0.3023	24.040	22.857	20.745	17.088	20.485
28	0.3033	15.028	9.046	16.114	15.403	15.605
28	0.3043	26.527	23.938	22.666	21.424	15.526
28	0.3053	27.809	23.315	22.590	23.402	15.681
28	0.3063	19.580	17.333	19.867	14.228	6.334
28	0.3073	18.015	12.634	13.380	15.639	6.302
28	0.3083	8.616	9.275	7.954	18.564	8.974
28	0.3093	16.944	10.458	16.367	13.420	16.534
28	0.3103	17.107	17.728	15.798	11.336	8.160
28	0.3113	14.170	16.347	10.904	13.912	15.693
28	0.3123	8.028	9.769	5.879	27.668	13.086
28	0.3133	17.712	22.890	13.155	19.536	11.776
28	0.3143	10.439	17.936	15.191	25.922	16.187
28	0.3153	10.159	17.839	11.255	28.434	9.712
28	0.3163	13.744	10.401	11.837	16.974	18.685
28	0.3173	13.728	18.963	13.778	19.665	14.378
28	0.3183	11.042	10.392	12.678	17.131	9.324
28	0.3193	13.535	6.452	13.355	21.452	17.266
28	0.3203	11.013	9.296	10.696	4.117	9.688
28	0.3213	14.238	11.622	16.897	13.475	18.393
28	0.3223	10.890	12.348	9.437	15.961	20.639
28	0.3233	11.808	21.781	10.300	32.438	22.854
28	0.3243	19.924	19.815	21.309	17.327	11.677
28	0.3253	9.197	11.421	15.942	19.519	13.641
28	0.3263	9.382	8.328	13.360	8.618	16.466
28	0.3273	6.669	9.658	6.037	11.994	7.256
28	0.3283	15.141	13.386	10.938	18.399	13.199
28	0.3293	7.357	12.307	11.738	12.090	11.969
28	0.3303	7.829	15.782	13.266	18.712	15.503
28	0.3313	4.507	10.625	11.676	12.561	8.100
28	0.3323	10.768	14.227	8.199	16.819	21.880
28	0.3333	17.482	13.044	11.177	19.205	22.605
28	0.3343	18.231	18.085	16.724	17.352	14.137
28	0.3353	7.014	9.716	7.909	17.948	11.398
28	0.3363	10.394	10.417	9.678	11.397	5.493
28	0.3373	10.447	15.468	8.893	17.616	23.937
28	0.3383	9.814	18.204	9.465	17.726	21.034
28	0.3393	6.077	10.177	7.131	15.571	16.386
28	0.3403	6.050	12.926	8.623	20.196	12.405
28	0.3413	7.295	9.930	5.670	23.603	24.093
28	0.3423	12.953	17.756	17.857	24.153	11.996
28	0.3433	12.030	12.374	12.432	26.493	19.419
28	0.3443	15.998	16.679	20.756	16.841	11.373
28	0.3453	11.790	10.907	12.897	16.872	13.073
28	0.3463	4.549	2.717	6.713	9.608	10.455
28	0.3473	9.899	14.587	9.576	19.445	12.237
28	0.3483	10.869	17.775	11.076	23.648	11.072

28	0.3493	5.804	18.072	9.625	28.885	21.520
28	0.3503	15.872	23.074	18.583	19.107	12.639
28	0.3513	14.517	15.985	17.490	17.196	13.982
28	0.3523	11.691	12.715	14.472	12.961	10.081
28	0.3533	7.991	14.914	10.708	16.504	13.494
28	0.3543	22.692	25.516	18.441	16.841	13.962
28	0.3553	11.323	11.242	15.319	8.143	12.464
28	0.3563	8.105	16.763	10.769	18.047	20.039
28	0.3573	13.982	20.013	15.592	13.905	13.557
28	0.3583	14.097	18.258	13.409	12.974	10.016
28	0.3593	14.051	18.196	13.406	12.940	10.003
28	0.3603	22.245	23.557	21.922	16.965	13.082
28	0.3613	8.683	16.867	12.960	12.825	10.953
28	0.3623	8.042	15.375	10.657	14.668	8.926
28	0.3633	9.551	13.231	10.699	19.589	13.447
28	0.3643	12.463	9.246	8.497	15.286	14.309
28	0.3653	12.197	13.240	7.536	11.460	14.301
28	0.3663	11.411	11.665	7.145	18.443	11.798
28	0.3673	10.611	10.937	7.732	17.240	12.501
28	0.3683	12.541	11.917	8.262	18.019	14.880
28	0.3693	13.776	11.906	8.858	23.372	29.611
28	0.3703	21.359	23.633	21.379	12.302	17.757
28	0.3713	19.728	19.310	17.859	22.797	22.826
28	0.3723	4.629	8.891	6.665	15.063	20.756
28	0.3733	11.960	15.532	14.771	4.346	15.532
28	0.3743	9.092	9.384	10.557	9.239	16.537
28	0.3753	9.012	10.331	6.352	12.549	23.976
28	0.3763	5.566	8.707	4.849	16.723	19.243
28	0.3773	7.300	8.881	5.256	20.983	12.400
28	0.3783	12.228	12.947	9.073	12.155	13.178
28	0.3793	11.728	14.236	8.957	18.401	11.436
28	0.3803	18.682	13.286	10.320	10.562	8.426
28	0.3813	16.434	10.622	13.228	12.272	10.485
28	0.3823	13.507	10.917	11.611	21.846	22.730
28	0.3833	29.521	13.405	14.208	23.923	35.702
28	0.3843	10.894	10.150	9.949	14.028	9.729
28	0.3853	14.791	9.641	16.392	15.093	21.996
28	0.3863	11.245	5.049	7.705	4.820	12.901
28	0.3873	8.086	11.641	5.884	17.195	22.846
28	0.3883	9.931	7.919	5.247	17.569	20.841
28	0.3893	12.138	9.609	9.356	18.212	16.244
28	0.3903	13.085	11.202	9.500	10.984	9.240
28	0.3913	23.242	24.878	15.433	28.280	33.849
28	0.3923	8.871	11.888	8.279	20.414	12.626
28	0.3933	5.878	13.067	4.905	17.673	8.727
28	0.3943	5.987	13.561	6.905	21.260	13.966
28	0.3953	17.902	19.129	16.738	23.438	20.966
28	0.3963	8.189	11.647	10.667	16.268	19.499
28	0.3973	25.282	21.681	18.282	24.723	29.631
28	0.3983	21.538	11.515	17.351	26.642	29.509

28	0.3993	9.025	10.268	7.023	9.453	16.069
28	0.4003	12.018	9.199	7.616	14.201	12.403
28	0.4013	23.168	12.485	8.934	27.488	29.263
28	0.4023	17.189	16.260	17.187	22.269	16.303
28	0.4033	21.929	19.869	19.969	25.854	14.949
28	0.4043	17.889	20.699	16.431	19.991	19.239
28	0.4053	11.715	9.590	9.872	8.482	18.592
28	0.4063	24.110	11.289	16.770	20.941	33.385
28	0.4073	9.212	12.053	7.469	25.907	17.816
28	0.4083	11.852	6.063	7.313	20.278	12.258
28	0.4093	19.153	15.995	14.432	26.896	11.785
28	0.4103	26.675	25.793	22.059	24.583	22.431
28	0.4113	17.274	8.483	9.435	15.678	22.806
28	0.4123	10.030	7.742	10.496	13.306	18.093
28	0.4133	18.774	10.750	10.777	12.097	14.385
28	0.4143	9.317	14.323	8.521	23.080	9.458
28	0.4153	8.393	12.618	6.801	17.415	4.151
28	0.4163	13.823	8.686	9.329	12.247	8.513
28	0.4173	23.393	16.364	14.929	10.064	12.933
28	0.4183	17.853	16.035	12.776	20.170	10.314
28	0.4193	22.597	24.617	19.632	28.718	25.514
28	0.4203	15.857	14.129	13.545	15.667	18.188
28	0.4213	14.025	20.752	12.712	19.773	20.406
28	0.4223	14.814	17.665	15.198	12.609	15.857
28	0.4233	16.122	13.843	12.679	19.402	11.698
28	0.4243	11.513	9.167	9.070	23.048	23.862
28	0.4253	11.658	14.342	11.605	21.952	20.711
28	0.4263	11.167	8.715	7.025	11.124	17.605
28	0.4273	15.011	8.028	10.611	11.386	8.796
28	0.4283	17.538	21.076	18.566	10.783	11.234
28	0.4293	20.636	14.589	15.413	13.049	14.879
28	0.4303	12.043	9.625	9.880	10.189	19.791
28	0.4313	21.475	21.462	18.633	24.325	13.752
28	0.4323	1.716	2.003	2.075	2.185	2.751
29	0.0005	0.000	0.000	0.000	0.000	0.000
29	0.0015	9.637	13.145	11.898	18.090	7.769
29	0.0025	15.794	12.492	19.831	21.541	8.951
29	0.0035	18.243	18.683	17.309	17.020	17.573
29	0.0045	23.688	27.548	23.787	16.602	13.824
29	0.0055	12.940	10.385	11.592	10.664	9.600
29	0.0065	16.120	13.659	20.849	17.074	28.487
29	0.0075	11.943	13.532	16.273	13.270	14.151
29	0.0085	14.221	14.902	15.359	18.929	9.024
29	0.0095	13.764	19.105	22.598	13.100	11.882
29	0.0105	17.568	22.775	22.013	11.697	14.086
29	0.0115	13.715	11.358	13.466	8.275	12.654
29	0.0125	10.141	10.271	14.044	8.510	10.219
29	0.0135	11.085	13.690	14.963	10.484	12.284
29	0.0145	9.978	10.380	14.287	18.740	19.204
29	0.0155	10.527	11.109	12.258	13.208	18.874

29	0.0165	12.852	13.302	11.554	8.937	20.635
29	0.0175	6.616	12.165	11.159	12.743	14.039
29	0.0185	15.524	16.448	16.289	8.219	15.502
29	0.0195	15.727	25.518	17.165	33.286	21.484
29	0.0205	5.817	18.117	10.126	14.524	17.036
29	0.0215	15.790	22.711	13.420	17.087	26.605
29	0.0225	21.246	25.290	19.078	11.662	22.843
29	0.0235	14.053	13.607	16.300	8.811	19.982
29	0.0245	20.675	23.299	22.703	16.245	26.127
29	0.0255	20.683	23.484	22.802	16.217	26.088
29	0.0265	13.486	17.417	15.507	16.011	18.246
29	0.0275	25.616	23.160	26.222	12.822	16.863
29	0.0285	25.512	32.184	23.427	14.204	14.480
29	0.0295	11.474	16.689	12.961	13.602	15.834
29	0.0305	11.382	16.823	13.022	13.717	15.809
29	0.0315	11.001	25.132	14.393	25.223	15.433
29	0.0325	11.915	23.031	11.649	20.828	14.276
29	0.0335	15.745	25.060	15.206	16.556	16.671
29	0.0345	14.992	19.374	14.742	21.685	8.160
29	0.0355	20.095	24.144	19.624	12.335	18.650
29	0.0365	7.218	11.199	9.310	20.756	22.630
29	0.0375	7.987	8.381	3.465	12.371	14.514
29	0.0385	6.373	9.919	7.956	12.781	6.612
29	0.0395	17.358	15.065	15.481	10.579	22.450
29	0.0405	18.839	23.022	14.589	12.706	13.921
29	0.0415	21.252	23.451	19.385	12.734	12.676
29	0.0425	20.666	23.578	19.561	12.872	18.626
29	0.0435	13.623	15.508	14.343	6.935	15.405
29	0.0445	10.761	17.281	15.094	14.140	19.884
29	0.0455	4.765	16.236	10.363	14.514	9.392
29	0.0465	6.089	14.061	11.061	19.623	18.021
29	0.0475	13.198	16.883	22.254	18.158	24.898
29	0.0485	11.686	13.722	15.732	13.587	20.423
29	0.0495	13.630	13.947	15.925	9.249	5.722
29	0.0505	6.900	14.057	8.479	4.918	16.952
29	0.0515	10.477	12.951	13.771	10.186	19.272
29	0.0525	10.260	10.227	14.905	9.659	12.571
29	0.0535	10.284	14.209	14.392	20.024	9.069
29	0.0545	15.408	17.543	20.080	14.783	18.790
29	0.0555	7.466	13.108	13.118	8.776	10.757
29	0.0565	9.371	9.817	20.298	7.542	12.783
29	0.0575	22.341	27.260	30.619	7.542	6.289
29	0.0585	7.736	11.951	9.379	9.923	13.282
29	0.0595	7.482	8.737	13.512	9.819	13.249
29	0.0605	18.375	19.153	30.359	11.140	12.558
29	0.0615	9.738	11.458	12.244	12.057	9.679
29	0.0625	5.929	8.865	11.093	10.100	13.228
29	0.0635	11.614	23.343	15.619	22.379	14.313
29	0.0645	19.442	17.394	25.773	10.189	9.943
29	0.0655	10.946	19.651	20.157	14.739	17.112

29	0.0665	8.918	9.778	10.767	12.460	10.633
29	0.0675	9.197	12.093	10.092	16.972	14.033
29	0.0685	17.433	12.742	14.439	11.990	18.938
29	0.0695	10.867	7.531	7.014	6.417	11.067
29	0.0705	11.005	12.734	9.824	18.613	18.671
29	0.0715	10.682	12.774	9.156	18.605	17.908
29	0.0725	10.823	7.540	6.763	12.813	14.261
29	0.0735	11.609	12.742	12.779	6.506	14.480
29	0.0745	15.136	19.673	17.359	5.613	12.095
29	0.0755	18.007	25.024	19.291	7.260	10.728
29	0.0765	8.530	12.568	11.911	7.807	11.107
29	0.0775	10.752	10.829	15.269	10.435	7.712
29	0.0785	17.140	21.406	21.754	7.436	11.602
29	0.0795	16.357	16.564	17.939	8.184	9.638
29	0.0805	20.930	19.833	20.395	9.704	9.026
29	0.0815	16.507	20.805	18.377	14.024	7.138
29	0.0825	14.183	16.753	16.080	16.751	9.524
29	0.0835	14.259	14.266	12.824	15.173	11.272
29	0.0845	15.238	23.882	15.800	17.060	17.559
29	0.0855	13.052	18.467	16.160	14.567	13.437
29	0.0865	7.357	8.185	7.697	3.665	5.267
29	0.0875	15.434	17.989	18.942	6.541	6.670
29	0.0885	15.548	18.366	17.811	10.738	2.647
29	0.0895	22.505	22.925	19.673	10.715	2.565
29	0.0905	18.384	20.031	18.616	10.981	3.147
29	0.0915	10.577	6.940	8.293	4.161	4.474
29	0.0925	11.178	7.896	6.005	5.210	7.002
29	0.0935	6.299	6.124	5.207	8.672	6.016
29	0.0945	11.633	11.605	10.388	7.907	6.894
29	0.0955	15.479	18.815	22.967	7.942	4.336
29	0.0965	13.551	20.448	17.829	6.462	10.494
29	0.0975	11.017	8.608	15.343	12.595	9.948
29	0.0985	15.032	8.428	22.207	15.469	15.871
29	0.0995	8.298	13.727	14.451	13.909	8.764
29	0.1005	15.848	14.863	18.623	6.841	3.423
29	0.1015	13.893	17.788	16.788	22.088	11.529
29	0.1025	8.191	8.387	7.532	10.735	5.402
29	0.1035	16.617	20.944	17.065	20.194	13.183
29	0.1045	15.843	13.919	11.268	17.493	20.351
29	0.1055	6.493	11.918	10.939	11.146	15.580
29	0.1065	14.448	17.045	16.205	11.991	7.389
29	0.1075	11.968	18.262	15.047	18.973	9.983
29	0.1085	20.275	19.208	25.459	7.795	12.909
29	0.1095	9.486	14.190	15.793	6.498	9.589
29	0.1105	13.057	27.852	21.317	19.096	12.516
29	0.1115	5.241	17.155	9.461	7.050	12.989
29	0.1125	13.444	15.773	17.178	11.364	6.254
29	0.1135	17.084	7.753	16.229	12.603	18.328
29	0.1145	15.815	14.115	14.654	13.879	19.367
29	0.1155	14.467	23.723	22.553	10.580	11.470

29	0.1165	24.380	21.530	32.808	4.556	12.290
29	0.1175	21.647	19.522	28.465	6.377	11.509
29	0.1185	11.819	17.390	18.721	15.185	14.257
29	0.1195	8.324	15.536	12.176	11.751	7.748
29	0.1205	6.475	15.412	9.536	8.718	11.834
29	0.1215	11.894	11.046	14.593	11.440	14.632
29	0.1225	15.151	11.198	22.451	9.323	13.123
29	0.1235	7.508	11.151	13.129	2.616	7.381
29	0.1245	22.324	22.230	24.142	10.073	9.302
29	0.1255	19.216	28.649	25.034	19.030	19.742
29	0.1265	19.444	18.357	16.022	7.885	22.410
29	0.1275	18.976	23.685	19.426	35.583	15.077
29	0.1285	20.859	13.687	24.096	9.765	12.285
29	0.1295	21.788	21.517	29.515	8.173	11.052
29	0.1305	17.488	15.991	21.826	16.440	26.063
29	0.1315	12.288	14.923	18.640	12.001	19.579
29	0.1325	7.702	7.132	9.952	13.912	13.388
29	0.1335	7.213	9.331	12.072	6.009	10.778
29	0.1345	7.718	10.814	16.063	3.930	14.329
29	0.1355	4.995	8.756	10.046	7.994	9.184
29	0.1365	13.252	14.368	20.113	17.566	14.386
29	0.1375	14.519	14.957	19.734	15.037	15.630
29	0.1385	14.195	11.043	15.983	9.407	17.651
29	0.1395	3.331	11.929	10.539	15.170	22.625
29	0.1405	13.676	14.894	19.498	12.580	14.779
29	0.1415	6.541	4.921	7.562	7.269	10.643
29	0.1425	8.504	12.464	14.213	13.744	23.099
29	0.1435	10.597	15.732	16.627	17.051	27.699
29	0.1445	12.726	16.470	20.691	17.037	13.599
29	0.1455	9.436	10.705	12.564	9.773	20.262
29	0.1465	8.474	7.198	7.075	7.857	11.252
29	0.1475	8.482	7.296	7.064	8.071	11.211
29	0.1485	8.517	7.616	6.440	7.746	10.901
29	0.1495	7.360	5.877	8.683	10.157	25.706
29	0.1505	12.259	9.258	16.468	3.335	22.142
29	0.1515	25.338	21.275	22.464	13.850	19.884
29	0.1525	23.553	13.038	14.169	5.672	7.648
29	0.1535	17.255	17.806	11.970	11.431	15.776
29	0.1545	15.716	18.628	15.476	8.792	5.535
29	0.1555	15.478	18.990	16.571	9.706	19.700
29	0.1565	11.844	12.045	14.546	7.168	4.268
29	0.1575	11.961	12.172	14.655	7.614	4.337
29	0.1585	22.142	24.927	22.601	8.782	13.785
29	0.1595	15.783	18.759	16.682	7.865	15.274
29	0.1605	22.676	21.713	23.698	8.737	24.813
29	0.1615	23.146	24.559	23.350	18.637	20.311
29	0.1625	15.146	16.730	16.852	19.325	24.777
29	0.1635	15.208	16.717	16.915	19.247	24.768
29	0.1645	17.070	19.053	17.969	18.759	18.252
29	0.1655	15.084	12.314	13.720	16.568	15.433

29	0.1665	16.560	13.041	13.659	17.608	17.611
29	0.1675	23.265	23.053	22.570	16.214	15.026
29	0.1685	22.366	21.815	22.909	13.849	5.382
29	0.1695	18.403	16.080	25.419	9.395	23.902
29	0.1705	18.219	22.618	23.204	9.661	19.916
29	0.1715	25.556	17.431	22.713	12.107	13.021
29	0.1725	20.799	12.497	17.800	12.186	11.141
29	0.1735	31.660	21.949	25.889	6.956	14.612
29	0.1745	31.678	21.953	25.906	6.963	14.626
29	0.1755	18.835	14.606	17.061	4.913	16.923
29	0.1765	14.562	11.256	15.524	8.228	19.949
29	0.1775	14.649	11.249	15.628	8.172	19.953
29	0.1785	15.886	12.677	15.540	3.293	7.183
29	0.1795	18.917	14.780	18.217	10.850	10.693
29	0.1805	23.860	22.066	25.338	15.528	14.319
29	0.1815	17.510	20.883	20.568	12.578	10.875
29	0.1825	18.809	23.004	21.421	6.797	10.615
29	0.1835	17.151	15.203	22.090	4.730	21.717
29	0.1845	22.391	16.568	20.190	11.312	19.320
29	0.1855	29.464	19.105	24.551	11.865	15.201
29	0.1865	17.422	20.282	20.634	9.897	14.350
29	0.1875	26.719	18.696	19.475	5.286	17.459
29	0.1885	15.330	10.116	10.406	6.749	14.058
29	0.1895	9.622	22.206	18.651	13.372	25.362
29	0.1905	17.293	15.644	23.142	8.849	19.650
29	0.1915	17.486	17.578	20.102	8.622	14.655
29	0.1925	10.815	14.048	12.063	11.503	15.456
29	0.1935	9.286	6.267	6.719	13.027	19.082
29	0.1945	11.135	4.586	14.133	9.825	17.642
29	0.1955	22.719	13.117	25.426	10.979	17.385
29	0.1965	17.268	15.785	22.637	12.598	13.497
29	0.1975	13.249	7.866	14.637	18.154	33.203
29	0.1985	16.074	8.341	14.858	14.351	27.397
29	0.1995	8.436	10.569	13.884	9.101	14.506
29	0.2005	21.080	7.446	20.080	11.138	23.798
29	0.2015	13.778	13.472	17.073	5.497	28.383
29	0.2025	16.332	13.291	22.323	11.896	17.613
29	0.2035	9.408	7.238	13.419	20.161	25.653
29	0.2045	9.532	7.274	13.530	20.162	25.631
29	0.2055	24.258	28.382	32.837	27.441	19.238
29	0.2065	32.561	29.300	31.238	20.143	15.608
29	0.2075	23.506	31.366	30.863	20.541	17.821
29	0.2085	37.422	25.988	35.589	16.770	18.549
29	0.2095	17.776	17.102	25.400	15.590	20.335
29	0.2105	15.749	16.337	14.565	8.536	16.589
29	0.2115	15.724	14.647	25.041	21.916	25.605
29	0.2125	22.273	18.425	26.394	12.523	13.785
29	0.2135	14.406	25.466	20.220	23.375	15.919
29	0.2145	15.492	17.804	19.130	17.648	26.280
29	0.2155	11.302	6.514	10.745	4.259	13.223

29	0.2165	6.982	9.443	10.387	9.916	22.719
29	0.2175	11.432	6.531	13.171	21.206	24.204
29	0.2185	6.125	8.658	10.891	13.876	19.992
29	0.2195	5.668	11.284	9.012	14.036	23.974
29	0.2205	7.914	13.359	14.332	11.259	22.563
29	0.2215	13.723	21.426	23.345	22.697	28.663
29	0.2225	15.492	19.912	12.255	16.662	21.913
29	0.2235	8.890	15.331	10.829	7.521	14.479
29	0.2245	13.766	22.780	19.610	16.081	7.697
29	0.2255	26.678	28.725	31.515	23.120	10.530
29	0.2265	16.530	30.364	23.890	13.111	16.260
29	0.2275	14.456	22.644	16.262	14.537	12.113
29	0.2285	9.657	25.656	15.030	20.766	16.749
29	0.2295	7.564	16.289	15.504	15.669	22.471
29	0.2305	17.850	27.156	24.546	22.672	18.201
29	0.2315	15.256	28.862	20.672	20.390	20.538
29	0.2325	17.005	32.369	18.466	18.961	21.669
29	0.2335	16.509	25.632	19.289	19.587	23.980
29	0.2345	13.010	20.353	18.471	15.747	15.240
29	0.2355	23.489	23.550	27.798	15.245	9.741
29	0.2365	24.071	27.971	25.898	23.828	12.233
29	0.2375	13.178	17.386	10.365	16.465	11.191
29	0.2385	15.765	15.492	22.499	10.468	7.201
29	0.2395	7.923	13.106	14.076	13.475	8.266
29	0.2405	9.261	20.946	17.651	14.425	19.297
29	0.2415	9.175	14.139	15.312	23.837	23.295
29	0.2425	6.713	6.245	7.632	23.888	14.646
29	0.2435	9.855	6.943	9.022	12.724	9.538
29	0.2445	14.289	22.754	24.392	12.258	25.041
29	0.2455	14.036	22.502	24.172	12.234	24.763
29	0.2465	11.807	5.858	13.249	11.369	21.033
29	0.2475	10.648	16.538	11.898	14.400	13.475
29	0.2485	13.470	19.312	14.256	28.523	12.895
29	0.2495	15.041	19.052	19.708	15.199	23.500
29	0.2505	8.769	9.499	9.197	11.552	27.168
29	0.2515	8.875	8.614	10.701	8.107	20.651
29	0.2525	9.828	13.223	12.975	8.394	9.583
29	0.2535	12.944	18.120	21.919	32.450	22.531
29	0.2545	17.784	29.141	27.903	37.736	21.015
29	0.2555	21.170	16.546	21.175	16.958	22.785
29	0.2565	6.399	13.189	9.443	14.778	11.622
29	0.2575	27.980	30.265	32.656	30.689	26.539
29	0.2585	18.766	21.837	24.522	17.601	19.038
29	0.2595	15.005	18.011	17.289	23.472	11.553
29	0.2605	4.780	4.435	8.733	10.947	13.799
29	0.2615	5.175	11.798	14.098	17.145	22.590
29	0.2625	26.645	22.943	30.397	16.489	8.263
29	0.2635	19.594	12.046	17.538	19.213	17.482
29	0.2645	16.782	16.509	21.971	14.601	19.340
29	0.2655	10.817	12.975	17.908	5.242	13.714

29	0.2665	16.743	19.093	19.652	22.475	14.818
29	0.2675	22.534	15.909	20.915	14.394	27.756
29	0.2685	16.832	24.123	22.486	16.288	18.915
29	0.2695	10.175	23.401	14.023	16.638	20.123
29	0.2705	9.529	14.333	12.307	11.286	8.857
29	0.2715	8.504	17.118	11.697	16.840	17.493
29	0.2725	8.248	12.945	8.849	16.053	13.641
29	0.2735	8.143	13.060	8.713	16.642	13.718
29	0.2745	11.230	18.449	11.594	15.432	11.488
29	0.2755	17.023	20.873	14.760	24.761	23.880
29	0.2765	17.005	20.828	14.722	24.725	23.864
29	0.2775	8.703	8.503	10.410	19.992	9.872
29	0.2785	14.284	11.056	16.467	23.966	14.226
29	0.2795	14.137	11.035	16.367	23.662	14.273
29	0.2805	14.404	13.288	15.317	27.377	20.554
29	0.2815	14.850	9.740	14.396	21.250	20.141
29	0.2825	11.137	15.965	12.495	19.926	15.771
29	0.2835	6.068	14.939	10.828	21.770	13.504
29	0.2845	8.226	11.206	11.638	17.924	10.568
29	0.2855	5.229	7.971	12.182	17.354	17.648
29	0.2865	5.591	8.262	13.790	14.265	15.086
29	0.2875	10.348	8.633	12.784	11.168	8.923
29	0.2885	7.142	8.082	10.279	8.849	8.240
29	0.2895	9.420	4.843	11.205	7.591	9.046
29	0.2905	8.419	10.752	12.750	10.661	8.092
29	0.2915	8.406	10.745	12.752	10.662	8.100
29	0.2925	6.856	7.691	10.910	13.785	11.716
29	0.2935	4.523	11.847	8.533	16.023	17.458
29	0.2945	4.777	7.690	9.179	14.549	19.774
29	0.2955	10.586	14.273	9.393	27.490	19.060
29	0.2965	9.379	19.008	9.802	30.197	20.464
29	0.2975	6.310	15.146	8.205	28.316	15.087
29	0.2985	10.442	13.332	12.334	15.911	15.159
29	0.2995	9.479	8.109	14.245	12.564	14.431
29	0.3005	14.935	15.225	11.718	12.734	15.162
29	0.3015	15.898	16.287	15.980	17.542	16.842
29	0.3025	5.817	7.081	8.150	21.485	12.642
29	0.3035	8.640	12.795	7.729	10.256	8.543
29	0.3045	7.718	15.366	11.586	21.434	22.815
29	0.3055	4.956	9.874	6.046	25.758	27.124
29	0.3065	9.730	13.411	12.198	9.554	14.747
29	0.3075	13.718	12.521	12.309	6.199	22.459
29	0.3085	14.363	12.411	14.530	5.727	13.314
29	0.3095	9.487	8.655	8.604	11.101	16.649
29	0.3105	7.735	12.002	10.204	11.475	12.156
29	0.3115	7.412	11.601	11.746	10.340	6.186
29	0.3125	6.745	7.358	7.427	9.486	10.188
29	0.3135	11.354	11.466	10.986	15.442	16.431
29	0.3145	16.903	14.369	21.978	16.792	16.366
29	0.3155	10.162	9.148	13.235	4.816	9.885

29	0.3165	5.953	5.203	10.427	6.088	8.729
29	0.3175	4.492	5.570	6.676	9.051	9.945
29	0.3185	12.526	13.940	13.700	7.507	14.554
29	0.3195	14.960	14.807	16.084	11.219	12.428
29	0.3205	12.052	10.989	11.294	13.407	13.882
29	0.3215	8.691	10.906	12.431	13.997	14.053
29	0.3225	3.223	7.125	5.678	9.764	11.888
29	0.3235	7.667	12.883	11.503	16.608	9.548
29	0.3245	7.851	9.555	12.911	11.175	26.789
29	0.3255	5.301	6.621	11.393	14.953	33.169
29	0.3265	6.367	8.288	11.683	10.993	23.854
29	0.3275	4.361	8.431	8.711	6.568	8.757
29	0.3285	7.717	8.713	10.216	10.116	17.895
29	0.3295	5.272	7.717	8.136	12.884	27.906
29	0.3305	11.335	9.947	11.284	16.643	27.801
29	0.3315	13.126	12.157	11.903	11.290	19.747
29	0.3325	13.032	12.282	11.828	11.499	19.416
29	0.3335	7.170	3.147	7.736	9.857	19.434
29	0.3345	5.739	4.552	4.261	17.393	14.399
29	0.3355	4.203	8.642	6.286	12.234	7.242
29	0.3365	7.682	5.460	9.962	19.947	14.139
29	0.3375	5.331	6.332	3.465	8.957	9.240
29	0.3385	9.079	8.520	12.683	19.101	14.841
29	0.3395	16.426	7.423	8.561	18.515	23.374
29	0.3405	18.367	14.311	13.411	26.957	21.660
29	0.3415	9.713	11.344	5.231	14.089	14.906
29	0.3425	11.480	6.208	6.606	7.129	10.389
29	0.3435	20.358	9.917	19.336	18.630	17.332
29	0.3445	7.476	8.068	8.453	13.263	22.853
29	0.3455	18.292	13.009	16.398	18.356	26.392
29	0.3465	9.302	10.831	9.265	25.875	15.638
29	0.3475	14.652	9.204	7.663	15.498	19.306
29	0.3485	10.909	9.827	7.003	9.771	6.864
29	0.3495	10.799	9.716	6.815	9.717	6.876
29	0.3505	12.266	9.134	7.186	11.287	11.159
29	0.3515	11.768	17.381	12.269	19.868	25.205
29	0.3525	9.053	10.334	10.204	16.276	21.408
29	0.3535	6.979	9.750	7.092	10.984	23.551
29	0.3545	7.016	9.718	7.125	10.937	23.410
29	0.3555	11.545	12.150	10.355	22.074	23.599
29	0.3565	4.259	4.472	6.594	29.079	33.079
29	0.3575	2.479	6.208	6.109	5.916	16.812
29	0.3585	9.681	7.524	7.047	9.946	14.935
29	0.3595	14.503	18.756	17.485	19.696	12.367
29	0.3605	15.294	17.622	17.736	14.801	12.281
29	0.3615	13.479	15.165	15.801	21.160	30.647
29	0.3625	26.485	21.733	17.574	19.911	29.435
29	0.3635	26.414	21.631	17.496	19.839	29.414
29	0.3645	26.344	21.525	17.417	19.762	29.390
29	0.3655	26.273	21.414	17.334	19.681	29.362

29	0.3665	26.205	16.352	15.861	19.644	28.908
29	0.3675	7.151	3.993	5.409	9.704	16.119
29	0.3685	7.135	3.954	5.407	9.498	16.359
29	0.3695	7.119	3.917	5.408	9.289	16.598
29	0.3705	23.581	11.931	16.762	19.549	18.261
29	0.3715	9.743	6.193	7.234	12.196	22.109
29	0.3725	12.750	10.585	11.735	8.855	14.085
29	0.3735	14.122	9.862	12.380	14.734	21.539
29	0.3745	13.213	7.977	9.754	11.585	18.415
29	0.3755	13.529	9.153	11.911	17.428	16.953
29	0.3765	5.025	3.735	3.172	19.613	15.212
29	0.3775	7.049	5.419	6.676	20.804	26.173
29	0.3785	3.828	1.964	5.559	5.710	20.494
29	0.3795	19.037	17.992	18.919	12.702	24.543
29	0.3805	9.181	12.211	12.932	10.805	28.600
29	0.3815	10.943	21.415	17.792	9.717	21.568
29	0.3825	11.592	17.356	14.204	14.823	13.767
29	0.3835	9.242	13.539	10.158	18.754	15.829
29	0.3845	8.372	18.148	10.331	18.344	23.367
29	0.3855	9.778	16.320	13.466	15.071	16.929
29	0.3865	11.721	9.169	10.298	11.539	17.997
29	0.3875	7.095	11.075	7.726	11.489	24.602
29	0.3885	9.638	12.199	15.866	21.229	29.340
29	0.3895	10.375	8.344	15.056	18.168	21.200
29	0.3905	10.125	5.191	8.864	9.899	12.962
29	0.3915	16.325	8.816	14.384	5.343	17.470
29	0.3925	13.812	15.343	18.961	12.654	16.876
29	0.3935	24.674	22.547	20.908	18.824	36.798
29	0.3945	18.086	10.376	14.585	9.484	28.771
29	0.3955	8.615	14.021	12.427	13.715	11.896
29	0.3965	9.831	12.021	9.737	20.079	20.326
29	0.3975	13.002	6.054	8.810	11.990	22.332
29	0.3985	8.700	9.268	6.377	6.610	6.065
29	0.3995	23.082	20.612	15.906	15.611	12.949
29	0.4005	7.950	13.289	11.184	20.062	17.940
29	0.4015	21.940	15.758	19.145	15.273	15.596
29	0.4025	23.913	16.909	21.241	13.011	26.018
29	0.4035	13.249	8.949	8.990	10.722	24.187
29	0.4045	13.497	8.222	11.749	16.533	21.743
29	0.4055	11.536	11.372	13.203	15.260	23.042
29	0.4065	10.150	8.122	10.359	9.517	14.019
29	0.4075	9.990	8.017	10.313	9.435	13.933
29	0.4085	14.209	14.265	14.872	13.982	12.106
29	0.4095	15.243	19.007	15.370	11.958	23.581
29	0.4105	19.816	17.489	19.953	10.849	12.603
29	0.4115	11.907	11.159	12.535	15.388	9.767
29	0.4125	13.339	12.196	11.352	20.552	23.616
29	0.4135	15.632	9.812	14.975	13.315	12.278
29	0.4145	15.606	9.832	14.981	13.190	12.325
29	0.4155	17.202	10.929	15.896	16.575	5.974

29	0.4165	8.101	10.037	9.089	13.117	10.968
29	0.4175	9.570	7.476	9.786	11.833	14.276
29	0.4185	19.289	18.195	14.670	20.503	21.029
29	0.4195	19.435	18.499	14.834	20.571	20.951
29	0.4205	11.509	10.219	10.539	14.248	19.192
29	0.4215	11.752	8.866	11.977	8.657	10.259
29	0.4225	11.727	8.890	11.995	8.572	10.177
29	0.4235	11.252	10.112	13.100	12.243	8.651
29	0.4245	7.134	12.226	9.067	11.247	13.718
29	0.4255	10.861	7.203	9.601	11.120	15.869
29	0.4265	13.162	15.088	13.587	25.948	16.543
29	0.4275	13.151	15.065	13.582	25.975	16.535
29	0.4285	13.198	13.725	15.373	25.054	25.677
29	0.4295	10.044	10.586	9.365	6.482	10.635
29	0.4305	13.503	9.970	12.463	8.672	12.985
29	0.4315	5.413	5.337	4.682	15.573	23.361
29	0.4325	5.027	3.276	5.680	8.222	15.608
29	0.4335	5.349	4.850	7.287	4.564	9.688
29	0.4345	9.105	13.791	9.927	17.991	14.634
29	0.4355	8.117	15.358	8.041	19.206	18.916
29	0.4365	9.279	10.385	7.417	13.657	24.101
29	0.4375	6.337	7.992	4.843	13.417	17.434
29	0.4385	5.933	8.542	6.048	13.287	14.886
29	0.4395	7.923	9.872	8.490	10.821	11.937
29	0.4405	6.374	14.131	9.873	18.870	20.236
29	0.4415	15.355	14.007	9.354	18.584	21.758
29	0.4425	11.275	9.559	12.671	13.273	17.763
29	0.4435	4.699	8.910	5.915	8.944	22.006
29	0.4445	9.667	11.324	12.490	12.613	15.666
29	0.4455	14.025	6.143	12.000	10.513	13.498
29	0.4465	15.311	17.299	16.639	10.549	11.233
29	0.4475	9.919	15.417	9.940	8.612	12.921
29	0.4485	11.425	14.230	12.419	8.186	13.792
29	0.4495	12.245	16.750	18.198	18.886	17.696
29	0.4505	15.680	17.758	14.896	19.857	14.306
29	0.4515	14.251	10.035	12.671	13.563	11.586
29	0.4525	3.624	5.972	5.949	18.106	20.674
29	0.4535	6.854	12.342	9.721	21.005	18.375
29	0.4545	18.946	18.288	18.337	15.326	18.995
29	0.4555	19.524	26.264	24.085	14.061	14.645
29	0.4565	18.583	16.000	14.996	21.582	20.368
29	0.4575	18.990	9.961	12.279	11.026	28.654
29	0.4585	18.242	15.316	10.787	16.395	15.763
29	0.4595	13.835	12.491	11.308	9.760	14.401
29	0.4605	13.635	11.329	12.595	14.124	13.329
29	0.4615	17.026	16.494	11.261	13.949	15.841
29	0.4625	13.346	8.228	11.579	23.827	17.948
29	0.4635	8.509	10.951	8.338	20.466	24.982
29	0.4645	4.807	3.336	4.778	11.744	15.494
29	0.4655	10.629	10.958	11.968	25.451	22.549

29	0.4665	16.355	15.699	11.980	17.333	17.319
29	0.4675	5.995	7.736	5.880	20.041	18.327
29	0.4685	8.553	7.399	8.933	15.357	20.113
29	0.4695	14.914	10.380	9.747	20.297	27.791
29	0.4705	9.079	10.372	10.154	10.126	13.637
29	0.4715	8.629	6.553	6.067	7.104	15.725
29	0.4725	8.643	6.579	6.097	7.089	15.646
29	0.4735	6.138	4.592	7.313	10.957	14.802
29	0.4745	11.198	6.009	13.152	12.189	13.398
29	0.4755	11.279	6.085	13.298	12.250	13.326
29	0.4765	11.473	6.552	12.383	11.806	17.732
29	0.4775	10.733	8.363	9.595	16.524	16.523
29	0.4785	8.009	4.801	8.901	17.179	9.441
29	0.4795	12.405	12.620	14.971	19.415	7.674
29	0.4805	7.534	11.829	11.401	25.765	9.843
29	0.4815	6.049	8.782	9.617	18.805	8.435
29	0.4825	14.156	9.742	6.291	11.043	15.110
29	0.4835	0.000	0.000	0.000	0.000	0.000

S2 Fig

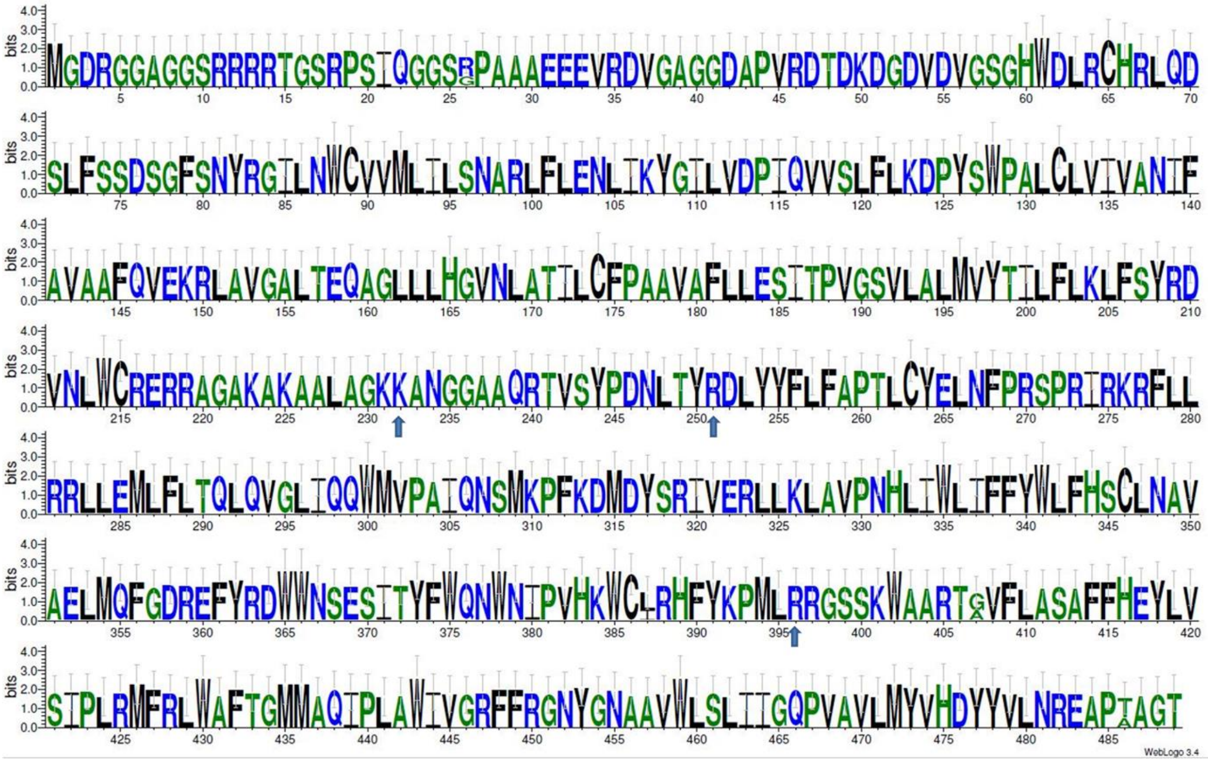

S3 Fig

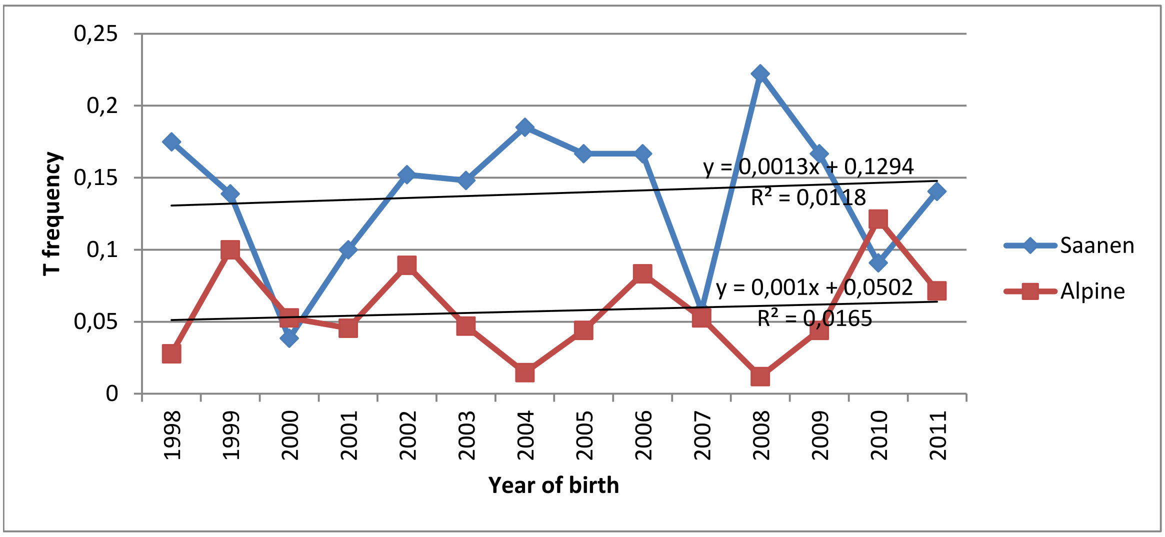

Year of birth	Alpine	Saanen
1998	0,0278	0,1750
1999	0,1000	0,1389
2000	0,0526	0,0385
2001	0,0455	0,1000
2002	0,0893	0,1522
2003	0,0469	0,1481
2004	0,0147	0,1852
2005	0,0441	0,1667
2006	0,0833	0,1667
2007	0,0526	0,0577
2008	0,0119	0,2222
2009	0,0441	0,1667
2010	0,1212	0,0909
2011	0,0714	0,1406

S3 Table: Percent identity matrix of the DGAT1 protein sequence between different species of ruminants

	Ceratotherium simun	Bubalus bubalis	Bos Taurus	Ovis aries	Capra hircus	Camelus dromedaries	Vicugna pacos
Bubalus bubalis	92.23						
Bos Taurus	93.25	98.98					
Ovis aries	93.25	98.16	99.18				
Capra hircus	93.25	98.16	99.18	100.00			
Camelus dromedaries	88.07	88.70	89.35	88.91	88.91		
Vicugna pacos	90.31	90.70	91.53	91.53	91.53	89.96	
Camelus bactrianus	91.94	91.93	92.75	92.75	92.75	94.31	95.84

S4 Table

Primer	Sequence (5'3')	Strand	Location on LT221856 (bp)	Location on CHIR_1.0, chromosome 14 (bp)	Amplified genome region	Protocol
DGAT1_00010_Up	AAGGCTGGCCTCTCCAATAG	plus	11-30	11241010-11241029	promotor	standard PCR
DGAT1_01489_Dn	TGGACCGAGGCCTAATTACC	minus	1470-1489	11242469-11242488		
DGAT1_00010_Up	AAGGCTGGCCTCTCCAATAG	plus	11-30	11241010-11241029	promotor	long range PCR
DGAT1_02730_Dn	CATAAGCTCCGGGAATGGAG	minus	2739-2758	11243710-11243729		
DGAT1_02505_Up	CTCAGCATTCGCTGCTTCTC	plus	2534-2553	11243505-11243524	promotor	long range PCR
DGAT1_04109_Dn	CAGCCTTGGCTCCGTTCT	minus	4101-4118	11245091-11245108		
DGAT1_03779_Up	GAATCATGCCCAGATCCTTC	plus	3799-3818	11244789-11244808	promotor	standard PCR
DGAT1_04607_Dn	TCCTGCAGGTCACACTTGTT	minus	4597-4616	ND		
DGAT1_04350_Up	CAGGGAGTAGCCATGTGGTC	plus	4361-4380	11245351-11245370	promotor	standard PCR
DGAT1_05308_Dn	CCAACAAGCCCTGGAGAAT	minus	5309-5327	11246299-11246317		
DGAT1_05090_Up	GTCACATTTCCTGGGGTTCA	plus	5100-5119	11246090-11246109	promotor	long range PCR
DGAT1_07698_Dn	GACCCGAGTGCAATCCTTT	minus	7689-7707	11248680-11248698		
DGAT1_07451_Up	CAAACCGCATGGACTTATCC	plus	7461-7480	11248452-11248471	promotor	long range PCR
DGAT1_09978_Dn	TTGCTCTATAGTCGGCTTCG	minus	8002-8021	11250959-11250978		
DGAT1_07451_Up	CAAACCGCATGGACTTATCC	plus	7461-7480	11248452-11248471	promotor	standard PCR
DGAT1_07868_Dn	CCGAGGCTTTGGCTTAGAC	minus	7859-7877	ND		
DGAT1_09801_Up	TTGCCAATTTACACCCGTCT	plus	7843-7862	ND	promotor	standard PCR
DGAT1_09978_Dn	TTGCTCTATAGTCGGCTTCG	minus	8002-8021	11250959-11250978		
DGAT1_07451_Up	CAAACCGCATGGACTTATCC	plus	7461-7480	11248452-11248471	promotor	standard PCR
DGAT1_07698_Dn	GACCCGAGTGCAATCCTTT	minus	7689-7707	11248680-11248698		
DGAT1_07451_Up	CAAACCGCATGGACTTATCC	plus	7461-7480	11248452-11248471	promotor	long range PCR
DGAT1_10825_Dn	GCATCTCGGGCCCTACTTAT	minus	9109-9128	11252066-11252085		
DGAT1_10763_Up	CTCCATCCCAGCTCAGTGTT	plus	8807-8826	11251764-11251783	promotor	standard PCR
DGAT1_12284_Dn	AACTGACAGTGCTCCCCAGA	minus	10325-10344	11253265-11253284		

DGAT1_12015_Up	GGCTCAGACAACCAAGGAAC	plus	10076-10095	11253016-11253035	promotor	long range PCR
DGAT1_14222_Dn	TAAGCAAAGCTGGGAAGACC	minus	12262-12281	11255203-11255222		
DGAT1_12713_Up	CTCCTAGAGGCCCACTCCTC	plus	10774-10793	11253714-11253733	promotor	long range PCR
DGAT1_15492_Dn	ACGTCTCCGTCCTTGTCTGT	minus	13532-13551	11256510-11256529		
DGAT1_12713_Up	CTCCTAGAGGCCCACTCCTC	plus	10774-10793	11253714-11253733	promotor	standard PCR
DGAT1_14222_Dn	TAAGCAAAGCTGGGAAGACC	minus	12262-12281	11255203-11255222		
DGAT1_15133_Up	GAGCTACGCTTCCCAGGACT	plus	13193-13212	11256134-11256153	exon 1 /intron1	standard PCR
DGAT1_15728_Dn	CCAACCTCCCGCTAAGTTTC	minus	13739-13758	11256709-11256728		
DGAT1_15669_Up	GCGTGACCCCTAACCTTTG	plus	13592-13610	11256570-11256588	intron 1	standard PCR
DGAT1_16894_dn	CATCCTCGCCACCAACTG	minus	14898-14915	11257877-11257894		
DGAT1_15669_Up	GCGTGACCCCTAACCTTTG	plus	13592-13610	11256570-11256588	intron 1	standard PCR
DGAT1_16434_Dn	GACTGACCTGTTGCCCATTT	minus	14436-14455	11257415-11257434		
DGAT1_16267_Up	CATCGGCTAAGTCCTGCTG	plus	14298-14316	11257268-11257286	intron 1	standard PCR
DGAT1_16894_dn	CATCCTCGCCACCAACTG	minus	14898-14915	11257877-11257894		
DGAT1_16803_up	AGGCCAGATGGGCAGAAG	plus	14824-14841	11257803-11257820	intron 1	standard PCR
DGAT1_17904_dn	AATCCCCACAGAGGTCCAG	minus	15767-15785	11258886-11258904		
DGAT1_17802_up	CAGAGAAGCTCTGGGTGAGG	plus	15683-15702	11258802-11258821	intron 1	standard PCR
DGAT1_18835_dn	GCTAAGCCTAGGGGAGATGG	minus	16697-16716	11259816-11259835		
DGAT1_18542_up	TCCTGGGTGCAGCCATCT	plus	16423-16440	ND	exon 2 to exon 5	long range PCR
DGAT1_22043_dn	GACCCCCTCCCTTCAGAG	minus	19706-19723	11263026-11263043		
DGAT1_18542_up	TCCTGGGTGCAGCCATCT	plus	16423-16440	ND	exon 2	standard PCR
DGAT1_19349_Dn	CAGCATCACCACACACCAAT	minus	17211-17230	11260330-11260349		
DGAT1_19239_up	GAGTGTCCATCTCCGCTCTC	plus	17120-17139	11260239-11260258	exon 2	standard PCR
DGAT1_20414_dn	GCATGGACTATTGGGTCTGG	minus	18089-18108	11261395-11261414		
DGAT1_20271_up	CCCCTCCAGCTGACGTGTCT	plus	17965-17984	11261271-11261290	exon 3 to exon 5	long range PCR
DGAT1_22043_dn	GACCCCCTCCCTTCAGAG	minus	19706-19723	11263026-11263043		

DGAT1_21866_Up	GTAAGCAGTGCCCTCACACC	plus	19540-19559	ND	intron 5 to intron 12	standard PCR
DGAT1_23269_Dn	GTAGAACTCGCGGTCTCCAA	minus	20929-20948	11264250-11264269		
ovDGAT1_6865_up	CCATCCTCTTCCTCAAGCTG	plus	19971-19990	ND	intron 7 to intron 12	standard PCR
DGAT1_23269_Dn	GTAGAACTCGCGGTCTCCAA	minus	20929-20948	11264250-11264269		
DGAT1_23175_up	ACCACCTCATCTGGCTCATC	plus	20854-20873	11264175-11264194	intron 13 to exon 17	standard PCR
DGAT1_24187_dn	GCTGTAGGGTTTCCAGAGCA	minus	21848-21867	11265168-11265187		
DGAT1_23459_Up	GATAGTGGGCCGCTTCTTC	plus	21501-21519	11264821-11264839	3'-end	long range PCR
DGAT1_25144_Dn	ATGGACGAACAGACCGATGA	minus	23185-23204	11266505-11266524		
DGAT1_25011_Up	CAGTCCCTTCCGAGACATTG	plus	23072-23091	11266392-11266411	3'-end	long range PCR
DGAT1_26711_Dn	GCAGTTTGGCTCGCACTC	minus	24615-24632	11268074-11268091		
DGAT1_26596_Up	CTCCCTCCCCAGTAGGACAG	plus	24518-24537	11267977-11267996	3'-end	long range PCR
DGAT1_28298_Dn	ATCCTCAGTCCAGGCAGAGA	minus	25909-25928	11269659-11269678		
DGAT1_29731_Up	CGATGTGGACCACCTTCC	plus	27362-27379	11271111-11271128	3'-end	long range PCR
DGAT1_31443_Dn	TGATCACTGAGGTGCCTACG	minus	29054-29073	11272822-11272803		
DGAT1_31239_Up	GAGGTCCAAGGCAGCTCTGT	plus	28870-28889	11272619-11272638	3'-end	standard PCR
DGAT1_32689_Dn	GTGGTTCACAGGATCTCCGTA	minus	30299-30319	11274068-11274048		
DGAT1_32663_Up	GCAGCTACGGAGATCCTGTG	plus	30294-30313	11274043-11274062	3'-end	long range PCR
DGAT1_34366_Dn	TAGAGCCTCTCCATCTTTGG	minus	31977-31996	ND		
DGAT1_34123_Up	TAGGGGATTGGAATGCAAAA	plus	31754-31773	ND	3'-end	standard PCR
DGAT1_35025_Dn	GGAATTCCATCACCTCCACT	minus	32636-32655	ND		
DGAT1_34772_Up	AGCGGTCAATGCAAAGAAAT	plus	32403-32422	ND	3'-end	standard PCR
DGAT1_35908_Dn	TCCAAGAAGCAAGGGTCTTT	minus	33519-33538	ND		
DGAT1_37001_Up	GAAAGCAAGGCTCCTGTGG	plus	34632-34650	11278381-11278399	3'-end	long range PCR
DGAT1_38726_Dn	AGACCAGCTGTGGTGGAACT	minus	36337-36356	11280086-11280105		
DGAT1_38516_Up	GGACTCCTCGGCAGGAAG	plus	36147-36164	11279896-11279913	3'-end	standard PCR
DGAT1_39611_Dn	TCACTAAGCCCTGCATTTCA	minus	37228-37247	11280977-11280996		

DGAT1_22102_Up	GGCAGGTAAGAAGGCCAAC	plus	20159-20177	71353600-71353615	exon 8	standard PCR-RFLP
DGAT1_22324_Dn	AGGAAGCGCTTTCGGATG	minus	20364-20381	ND		
DGAT1_23126_Up	GACACTTCTACAAGCCCATGC	plus	21184-21204	11264504-11264524	exon 15	standard PCR-RFLP
DGAT1_23310_Dn	GGGGATGCTCACCAGGTACT	minus	21348-21367	11264668-11264687		
DGAT1_00801_Dn	TGGGAAAGGACAGTTGGAGA	minus	792-811	11241791-11241810		sequencing
DGAT1_03104_Up	TAAAGCAAGGCTCCCAGAGA	plus	3115-3134	ND		sequencing
DGAT1_05090_Up	GTCACATTTCCTGGGGTTCA	plus	5100-5119	11246090-11246109		sequencing
DGAT1_05592_Up	GATGCGAGTCTTTCCCTCCT	plus	5593-5612	ND		sequencing
DGAT1_08448_Dn	CTCTCTGCACGTGTGACCAT	minus	8429-8448	11250263-11250282		sequencing
DGAT1_26923_Up	CAGCTCCCCTCGGTAAGTC	plus	24865-24883	ND		sequencing
DGAT1_27623_Dn	TCCTAGGGGTGAAGAGAAAGA	minus	25544-25564	ND		sequencing
DGAT1_1	GCGGGCTGAGGCCATG	plus	13378-13396	ND	complete cDNA	standard PCR
DGAT1_2	AGTGGTGAGGCAAAGCAGTC	minus	21675-21694	11264995-11265014		
DGAT1_3	CTGAGGGGATCCATGGGCGACCGCGGCGGCGCGGGC	plus	ND	ND	complete cDNA and TOPO vector	subcloning
DGAT1_4	ACCAGCCTCGAGGGGCTCAGGTGCCGGCTGTTG	minus	ND	ND		
DGAT1_R251L_up	CGACAACCTGACCTACCTCGATCTCTACTACTTCC	plus	20210-20229/20300-20315 (Partial Match)	ND	cDNA	Directed mutagenesis
DGAT1_R251L_dn	GGAAGTAGTAGAGATCGAGGTAGGTCAGGTTGTCG	minus				
DGAT1_W396R_up	AGCCCATGCTCCGGCGGGGCAGC	plus	21196-21218	11264516-11264538	cDNA	Directed mutagenesis
DGAT1_W396R_dn	GCTGCCCCGCCGGAGCATGGGCT	minus				
